# Supplementary material for: Quantifying the impact of taking medicines for primary prevention: a time-trade off study to elicit direct treatment disutility in the UK
Source: BMJ Open. 2023 Sep 21;13(9):e063800. doi: 10.1136/bmjopen-2022-063800 (PMC10514632; doi:10.1136/bmjopen-2022-063800)
Supplement: Supplementary data [file bmjopen-2022-063800supp003.pdf]

Quantifying the impact of taking medicines for long-term conditions

A survey to understand your views about taking medicines long term for the prevention of osteoporotic fractures

In this survey, we will be asking you about a drug treatment which may be offered to people at an increased risk of having a fracture from osteoporosis.

There are no right or wrong answers, we are just interested in hearing about your views.

Quantifying the impact of taking medicines for long-term conditions

Taking medicines long term for the prevention of osteoporotic fracture

Participant Consent Form

You are being invited to take part in a research study that aims to understand your views about taking medicines long-term. Before you decide, it is important for you to understand why the research is being done and what it will involve.

Please click on the sections below for information on the research.

What is the aim of the research?

Why have I been chosen?

What happens to the data collected?

How is confidentiality maintained?

How long will it take me?

Who will conduct the research?

What would I be asked to do if I took part?

Will the outcomes of the research be published?

What happens if I do not want to take part or if I change my mind?

Who has reviewed the research project?

I understand that my participation in the study is voluntary and that I am free to withdraw at any time without giving a reason by exiting the survey.

I confirm that I have read the information above.

- ◯ Yes, I would like to take part in the survey.
- ◯ No, I do not want to participate (exit survey).

0% 100%

What is your gender?

- ◯ Female
- ◯ Male

0% 100%

[QUOTA: QuotaCheck]

Note:

This page only contains quota information and only appears in preview mode.

0% 100%

By selecting one box in each group below, please indicate which statements best describe your own health state today.

Mobility

- ◯ I have no problems in walking about
- ◯ I have some problems in walking about
- ◯ I am confined to bed

Self-Care

- ◯ I have no problems with self-care
- ◯ I have some problems washing or dressing myself
- ◯ I am unable to wash or dress myself

Usual Activities (e.g. work, study, housework, family or leisure activities)

- ◯ I have no problems with performing my usual activities
- ◯ I have some problems with performing my usual activities
- ◯ I am unable to perform my usual activities

Pain/Discomfort

- ◯ I have no pain or discomfort
- ◯ I have moderate pain or discomfort
- ◯ I have extreme pain or discomfort

Anxiety/Depression

- ◯ I am not anxious or depressed
- ◯ I am moderately anxious or depressed
- ◯ I am extremely anxious or depressed

0% 100%

Part 1. Background

This section of the survey explains: osteoporosis (thinning of the bones), how medicine treatments may be used for people at risk of osteoporotic fracture, and what the treatment involves.

We will follow a story of Alex.

Click next to continue.

0% 100%

Part 1. Background

Alex has been to see a doctor who told her that she has weak bones.

The doctor has said Alex needs to start a medicine to prevent broken bones (fractures) because of her weak bones.

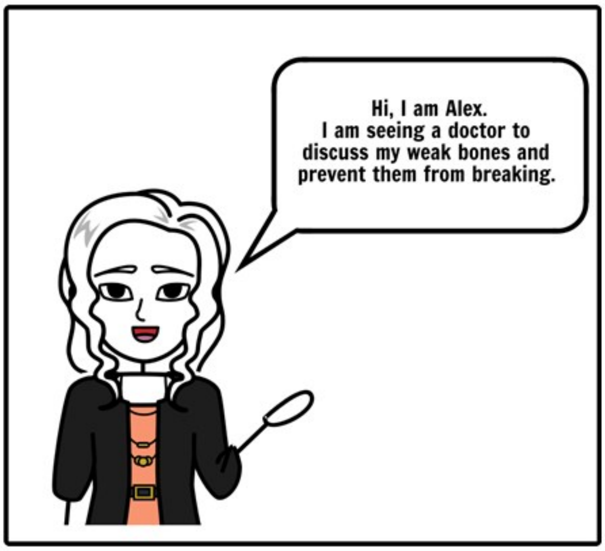

We will take you through Alex's story.

0% 100%

Part 1. Background

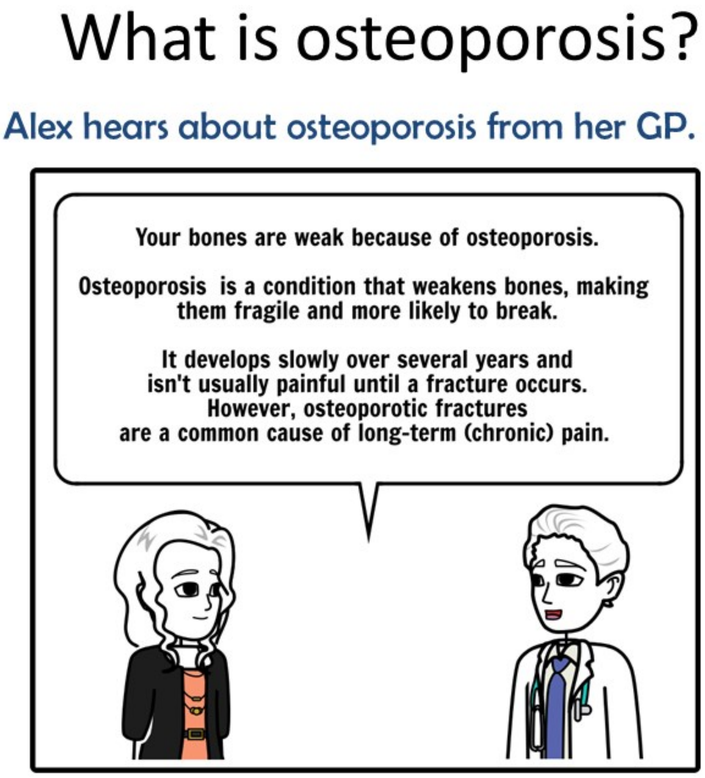

0% 100%

Part 1. Background

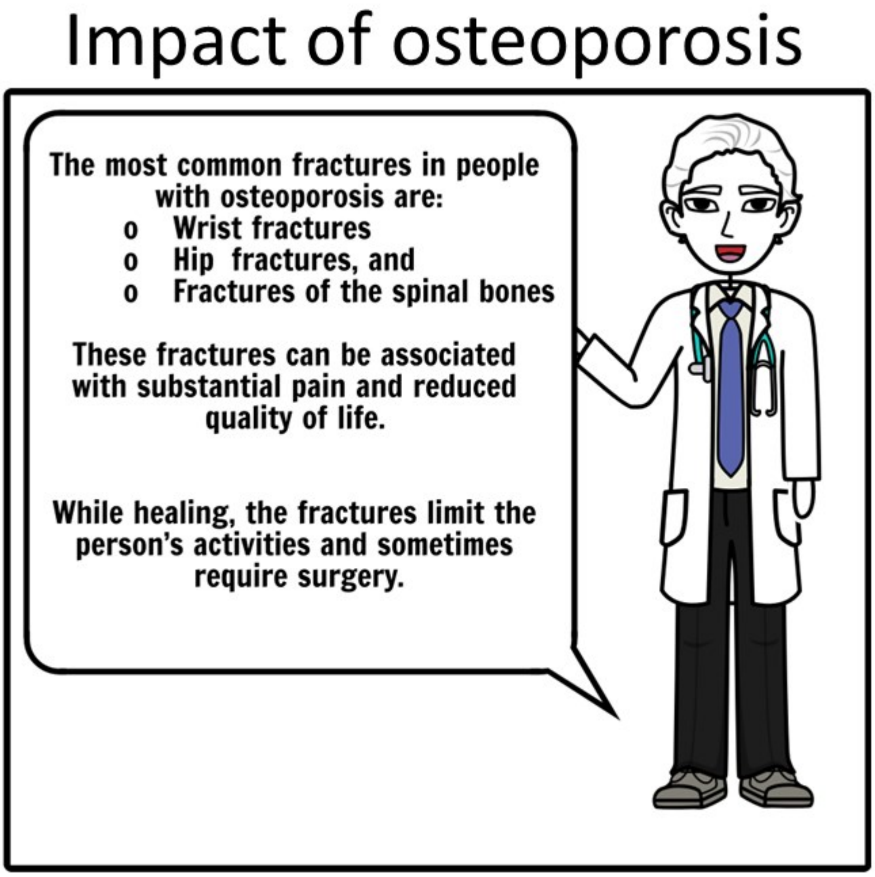

Part 1. Background

Risk of osteoporotic fractures

Her GP explains that Alex has some risk of fracture as her family has a history of hip fracture.

Also, Alex has a low body mass index (BMI). BMI is a measure of body fat.

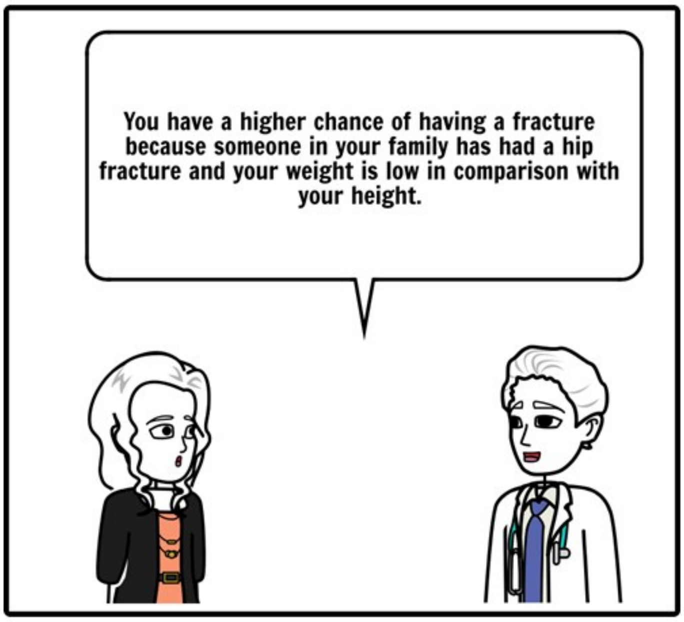

0% 100%

What is risk?

- **Risk** is: a term used to explain the chance that something bad might happen.

- **10% risk** means:

  Out of every **1,000** people, with osteoporosis,

  100 people would have a fracture, and **900** people would not.

0% 100%

What is a 10% risk?

- This diagram shows a 10% risk of having a fracture because of their osteoporosis.
- The people shaded blue are the ones who have a fracture.

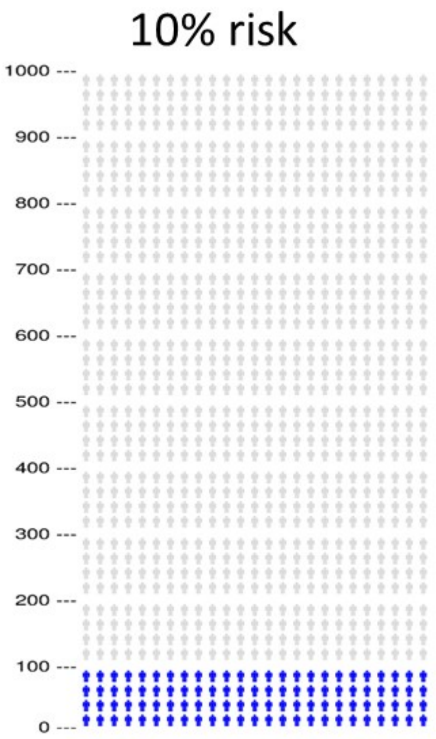

0% 100%

Part 1. Background

Prevention of fracture from osteoporosis

- Alex's doctor can estimate how likely it is that she will experience osteoporotic fracture over the next 5 years.
- The estimate is based on things such as Alex's age and sex, ethnicity, family fracture history, and body mass index.
- The doctor may prescribe certain medicines such as bisphosphonates to help reduce the chance of Alex having a fracture.
- The medicine will prevent some of the people who take it from having a fracture because of osteoporosis.
- Fractures may still happen in some of the people who take the medicine.

0% 100%

Prevention of fractures from osteoporosis

The doctor prescribes Alex a medicine to reduce the risk of her having a fracture in the future because of her osteoporosis.

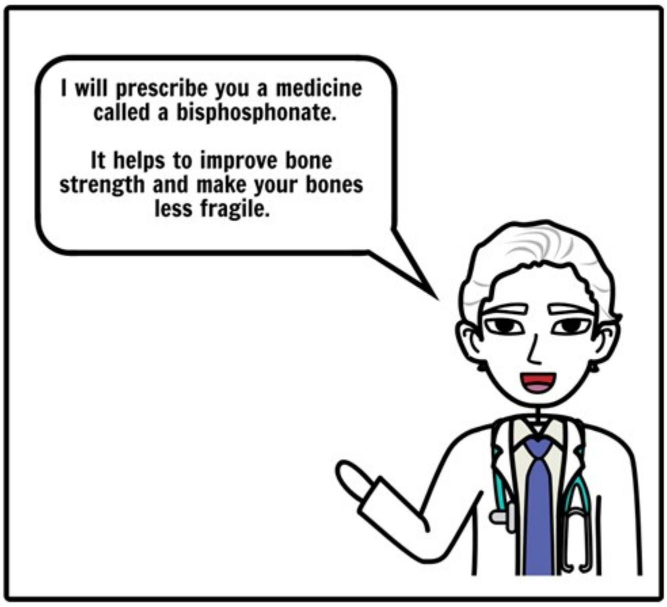

0% 100%

Collecting the medicine from the pharmacy

The pharmacist explains to Alex how to take the medicine.

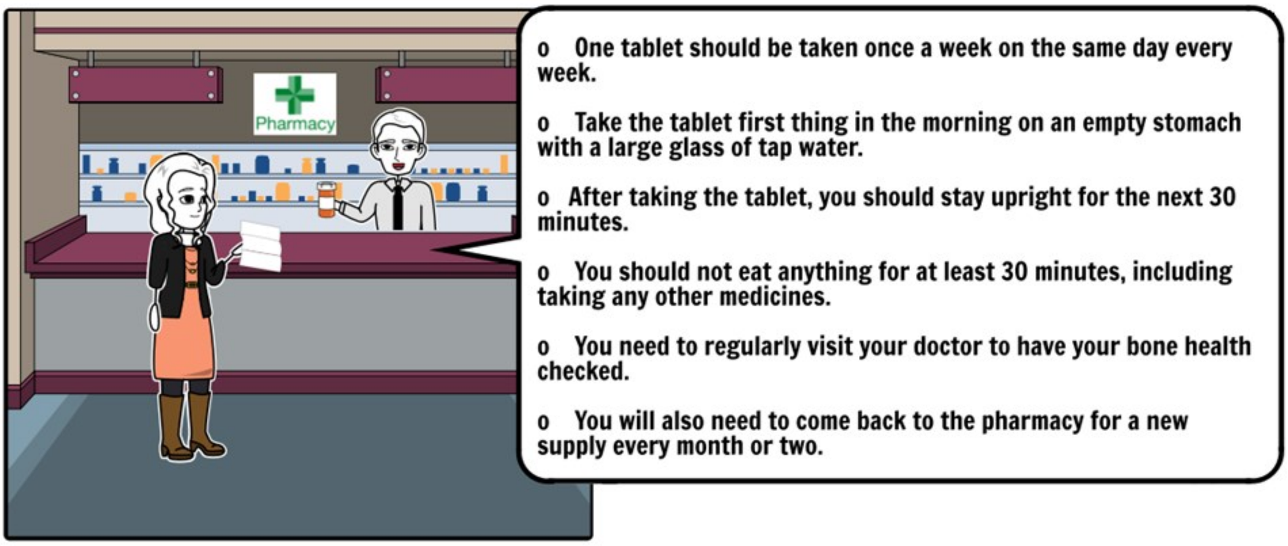

Taking the medicine for prevention

Alex goes home with her new tablets.

She then considers whether she wants to take them as the doctor and the pharmacist have advised.

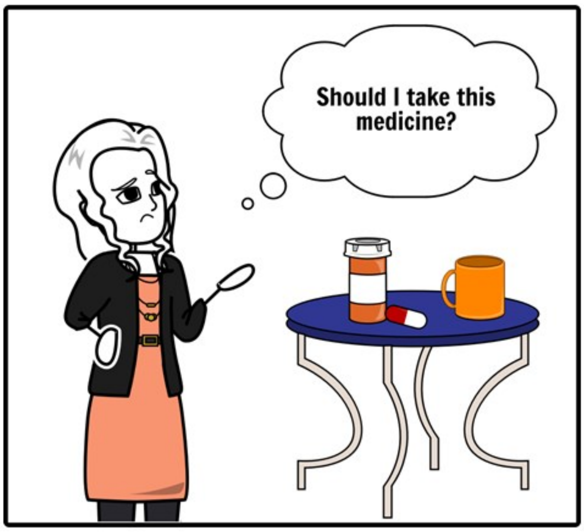

0% 100%

Part 1. Background

Alex may consider *three key things*,
when deciding whether to take the medicine.

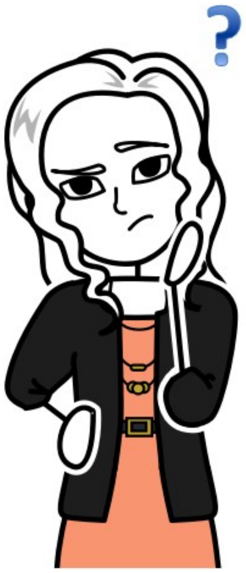

Effectiveness of the medicine

- How good the medicine is in terms of preventing a fracture from osteoporosis

Side effects of the medicine

- Whether there is any potential harm from the medicine

Inconvenience of the medicine

- Whether taking the medicine would fit in her lifestyle or cause her inconvenience

0% 100%

How effective is the medicine?

- Some people, but not everyone, who take the medicine will avoid a serious event, such as hip fracture, as a result of taking the tablet.
- The effectiveness of the medicine can be described in terms of a reduction in this risk.

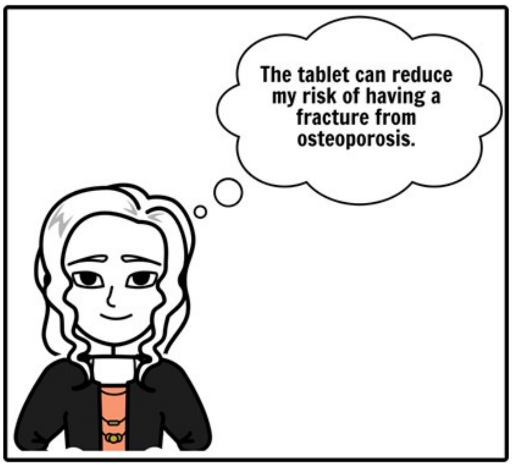

0% 100%

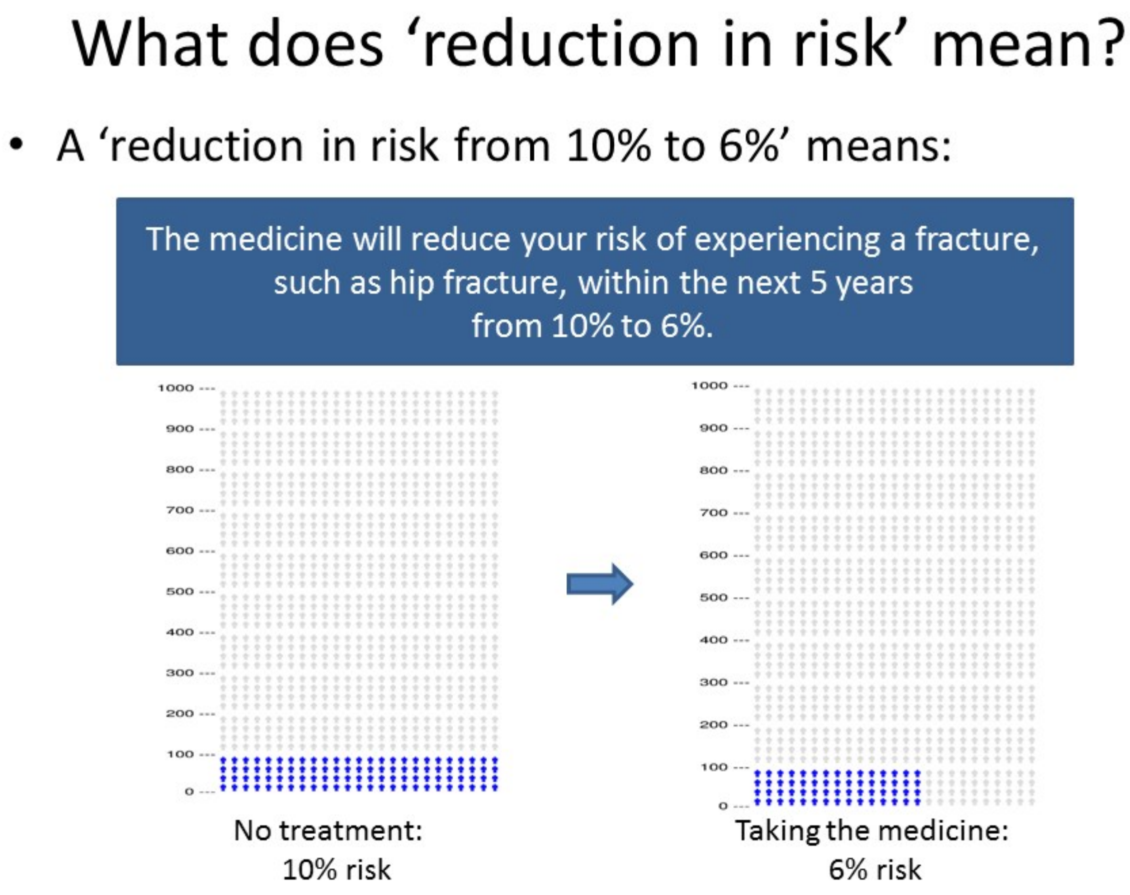
What does ‘reduction in risk’ mean?
• A ‘reduction in risk from 10% to 6%’ means:
The medicine will reduce your risk of experiencing a fracture, such as hip fracture, within the next 5 years from 10% to 6%.
1000
900
800
700
600
500
400
300
200
100
0
No treatment:
10% risk
Taking the medicine:
6% risk

0% 100%

Does the medicine have side effects?

The potential for side effects from the medicine can be described in terms of the **risk of a side effect**.

Alex hears about minor but more common side effects & severe but rarer side effects from her GP.

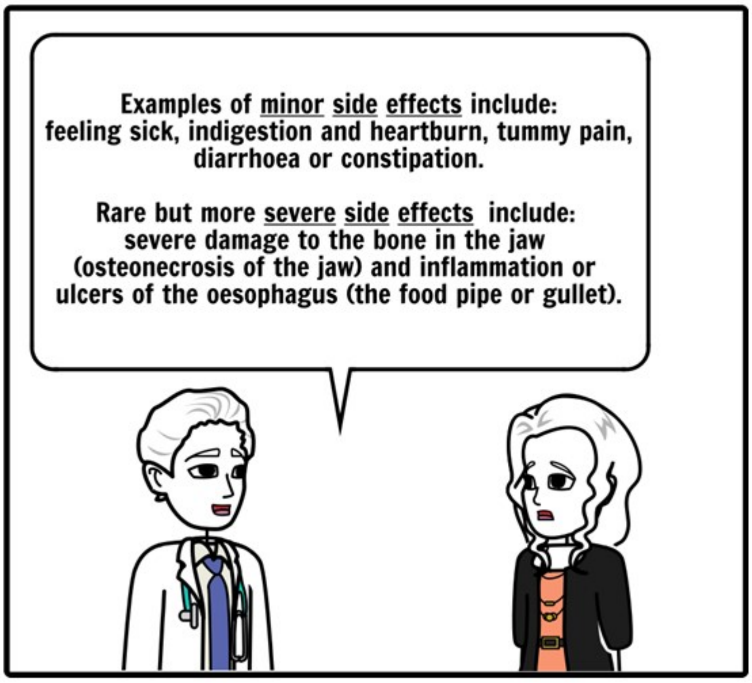

0% 100%

Part 1. Background

Risk of a minor side effect from the medicine

- A 5% risk of a minor side effect means:

  Among every **1,000** people taking the medicine,

  50 will experience a minor side effect such as either: feeling sick, indigestion, diarrhoea or constipation.

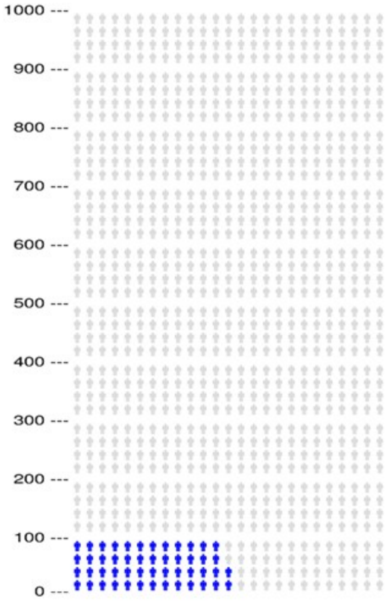

5% risk of a side effect

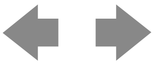

0% 100%

Risk of a severe side effect from the medicine

- A 0.3% risk of severe side effect means:

  Among every **1,000** people taking the medicine,

  3 will experience a severe side effect such as severe loss or destruction of the bone in the jaw.

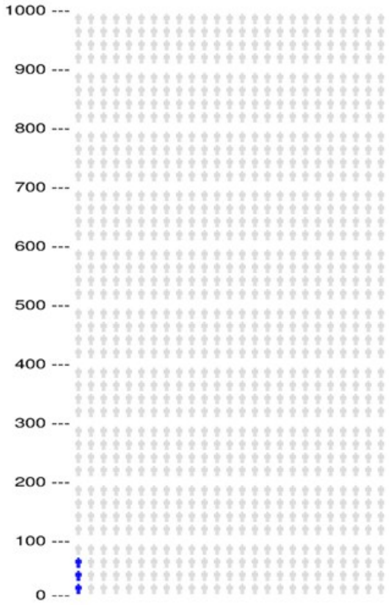

0.3% risk of a side effect

0% 100%

Part 1. Background

How inconvenient is it when taking the medicine?

Alex also thinks about what it means to have to take this medicine for the next 5 years.

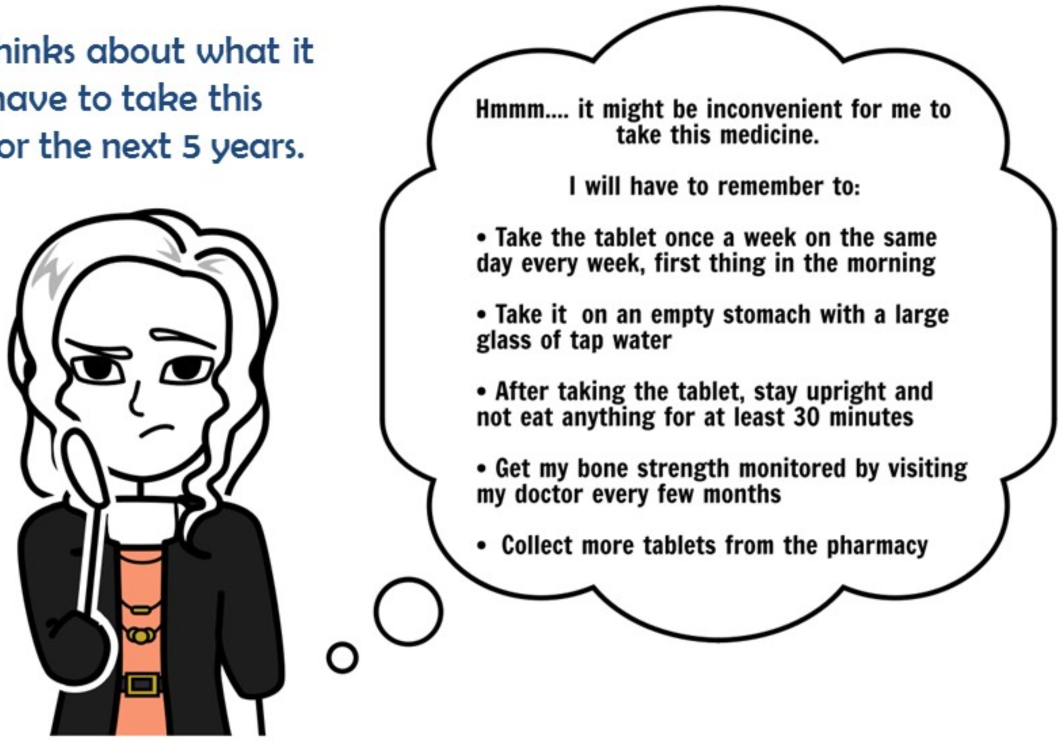

0% 100%

Part 1. Background

In the questions that follow in this survey,
we will ask you about how you think about taking a medicine to prevent osteoporotic fracture.

0% 100%

In general, how would you feel about taking a medicine for the first time?

- ◯ **Happy**
  I trust in my doctor and I am willing to try new things
- ◯ **Unsure**
  I'd like to think about it really carefully
- ◯ **Unhappy**
  I don't like the idea of trying a new medicine even if the doctor says it will help

0% 100%

Part 2: Your views on the inconvenience of taking a medicine to prevent fractures

Now, we will ask you about how you think about the inconvenience of taking a medicine to prevent osteoporotic fractures.

There are four questions.

Each question may look similar but differs in terms of the detail for each of the three features.

0% 100%

Warm up question

You have been told that you need to take a medicine to reduce the risk of fracture.

You can choose between two different medicines. We have called these Medicine A and Medicine B.

Medicine A is a pill that you only need to take once.

Medicine B is a pill that you need to take every week for 5 years.

Medicine A and Medicine B are equally effective in reducing your risk of having a fracture.
Medicine A and Medicine B will reduce your risk of having an osteoporotic fracture within the next 5 years from **10%** to **6%**.

In other words, in a crowd of **1,000** people in the UK, **100** are likely to have a fracture if they do not take the medicine. This is reduced to **60** people out of 1,000 who do take the medicine.
This information is also illustrated in this picture.

Risk of fracture

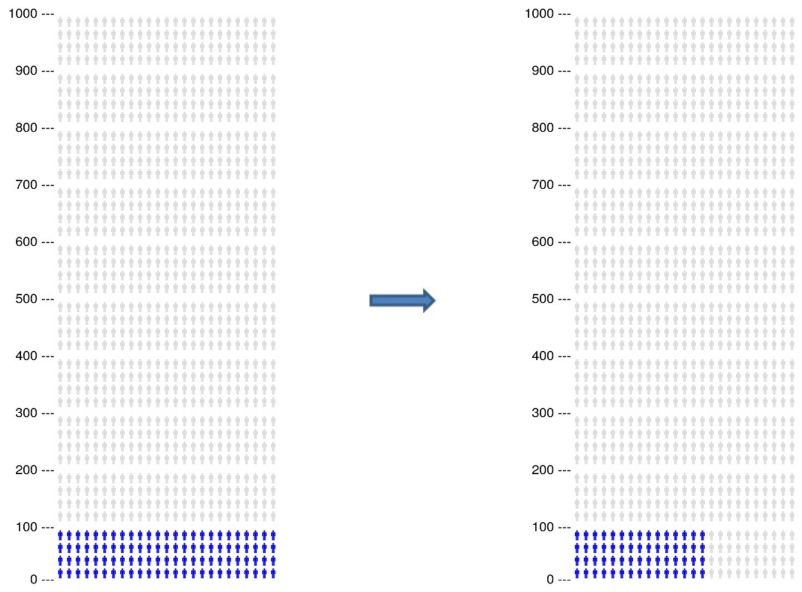

10%
No Treatment

6%
Taking the Medicine

(The figures shaded blue represent people who will have a fracture in the next 5 years)

In this scenario, you will not have any side effects from Medicine A or Medicine B.

Risk of a side effect

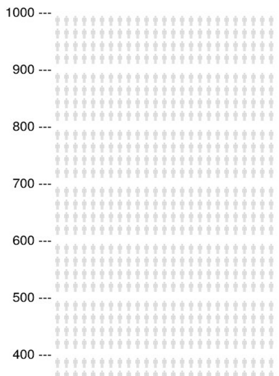

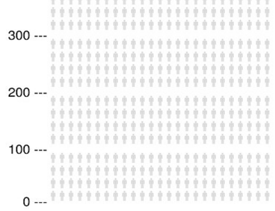

0%
No one will have a side effect.

0% 100%

Remember Medicine A and Medicine B are equally effective and have no risks of a side effect.

The only difference between Medicine A and Medicine B is how you take them.

Medicine A	Medicine B
You take a tablet only once in your life.<br>You will be given the tablet on the day you come in to see your GP.<br>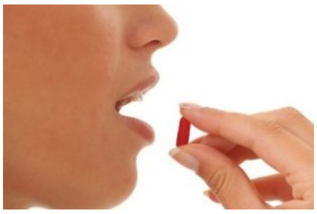	One tablet should be taken once a week on the same day every week.<br>You need to take this medicine for 5 years.<br><br>You need to take the tablet first thing in the morning on an empty stomach with a large glass of tap water.<br>After taking the tablet, you should stay upright for the next 30 minutes.<br>You should not eat anything for at least 30 minutes, including taking any other medicines.<br><br>You need to regularly visit your doctor to have your bone health checked.<br>You need to regularly visit a pharmacy for a new supply.<br>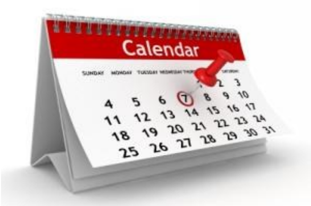 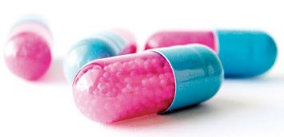

If you take Medicine A, you would live for **1 year** and then die.

If you take Medicine B, you would live for **5 years** and then die.

Would you prefer Medicine A or Medicine B, or are they the same?

Indicate your choice here. You can only choose one option:

◯ **Medicine A** — **Live for 1 year**

◯ **Medicine B** — Live for 5 years

◯ **Medicine A and Medicine B are the same.**

0% 100%

Now we are ready to start the survey questions.

0% 100%

Part 2

Question 1

You have been told that you need to take a medicine to reduce the risk of fracture.

You can choose between two different medicines. We have called these Medicine A and Medicine B.

Medicine A is a pill that you only need to take once.

Medicine B is a pill that you need to take every week for 5 years.

Medicine A and Medicine B are equally effective in reducing your risk of having a fracture.
Medicine A and Medicine B will reduce your risk of experiencing a fracture, such as hip fracture, within the next 5 years from **10%** to **6%**.

In other words, in a crowd of **1,000** people in the UK, **100** are likely to have a fracture if they do not take the medicine. This is reduced to **60** people out of 100 who do take the medicine.
This information is also illustrated in this picture.

Risk of fracture

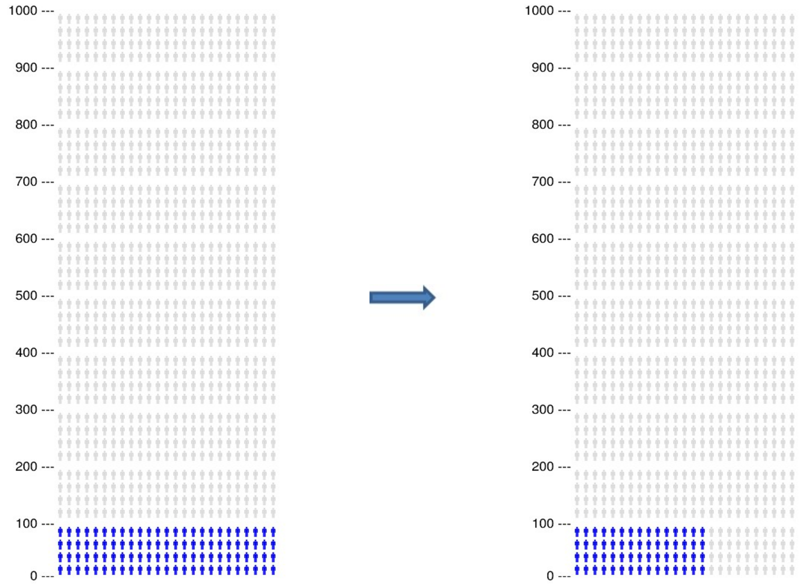

10%
No Treatment

6%
Taking the Medicine

(The figures shaded blue represent people who will have a fracture, such as hip fracture, within the next 5 years)

In this scenario, you will not have any side effects from Medicine A or Medicine B.

Risk of a side effect

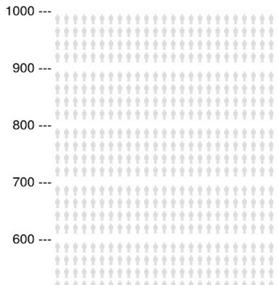

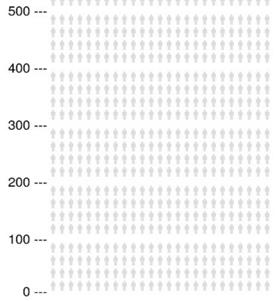

0 %
No one will have a side effect.

0% 100%

Repeated section

Remember Medicine A and Medicine B are equally effective and have no risks of a side effect.

The only difference between Medicine A and Medicine B is how you take them.

Medicine A	Medicine B
You take a tablet only once in your life. You will be given the tablet on the day you come in to see your GP. 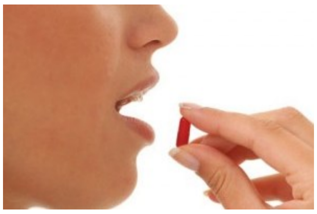	One tablet should be taken once a week on the same day every week. You need to take this medicine for 5 years. <br>You need to take the tablet first thing in the morning on an empty stomach with a large glass of tap water. After taking the tablet, you should stay upright for the next 30 minutes. You should not eat anything for at least 30 minutes, including taking any other medicines. <br>You need to regularly visit your doctor to have your bone health checked. You need to regularly visit a pharmacy for a new supply. 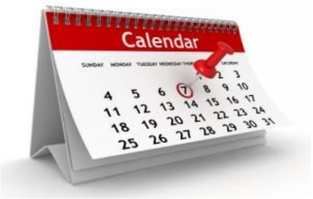 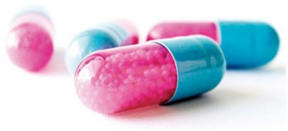

If you take Medicine A, you would live for **2.5 years (2 years and 6 months)** and then die.

If you take Medicine B, you would live for **5 years** and then die.

Would you prefer Medicine A or Medicine B, or are they the same?

Indicate your choice here. You can only choose one option:

◯ **Medicine A** Live for 2.5 years

◯ **Medicine B** Live for 5 years

◯ **The Same - Medicine A and Medicine B are the same.**

0% 100%

Repeated section

Remember Medicine A and Medicine B are equally effective and have no risks of a side effect.

The only difference between Medicine A and Medicine B is how you take them.

Medicine A	Medicine B
You take a tablet only once in your life.<br>You will be given the tablet on the day you come in to see your GP.<br>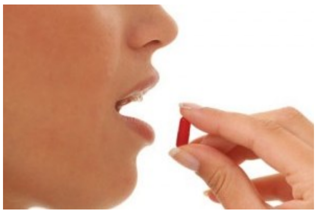	One tablet should be taken once a week on the same day every week.<br>You need to take this medicine for 5 years.<br><br>You need to take the tablet first thing in the morning on an empty stomach with a large glass of tap water.<br>After taking the tablet, you should stay upright for the next 30 minutes.<br>You should not eat anything for at least 30 minutes, including taking any other medicines.<br><br>You need to regularly visit your doctor to have your bone health checked.<br>You need to regularly visit a pharmacy for a new supply.<br>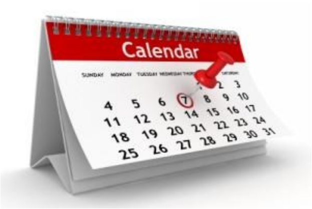 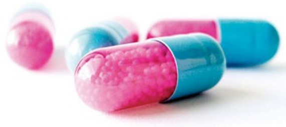

If you take Medicine A, you would live for **3 years** and then die.

If you take Medicine B, you would live for **5 years** and then die.

Would you prefer Medicine A or Medicine B, or are they the same?

Indicate your choice here. You can only choose one option:

◯ **Medicine A** Live for 3 years

◯ **Medicine B** Live for 5 years

◯ **The Same - Medicine A and Medicine B are the same.**

Repeated section

Remember Medicine A and Medicine B are equally effective and have no risks of a side effect.

The only difference between Medicine A and Medicine B is how you take them.

Medicine A	Medicine B
You take a tablet only once in your life. You will be given the tablet on the day you come in to see your GP.<br>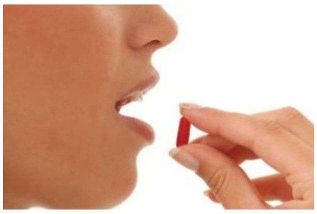	One tablet should be taken once a week on the same day every week.<br>You need to take this medicine for 5 years.<br><br>You need to take the tablet first thing in the morning on an empty stomach with a large glass of tap water.<br>After taking the tablet, you should stay upright for the next 30 minutes.<br>You should not eat anything for at least 30 minutes, including taking any other medicines.<br><br>You need to regularly visit your doctor to have your bone health checked.<br>You need to regularly visit a pharmacy for a new supply.<br>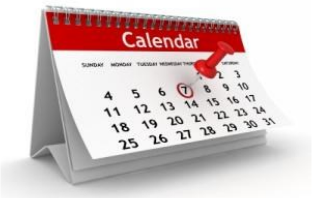 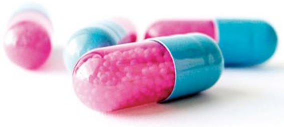

If you take Medicine A, you would live for **3.25 years** (3 years and 3 months) and then die.

If you take Medicine B, you would live for **5 years** and then die.

Would you prefer Medicine A or Medicine B, or are they the same?

Indicate your choice here. You can only choose one option:

- ◯ **Medicine A** — Live for 3 years 3 months
- ◯ **Medicine B** — Live for 5 years
- ◯ **The Same - Medicine A and Medicine B are the same.**

0% 100%

Repeated section

Remember Medicine A and Medicine B are equally effective and have no risks of a side effect.

The only difference between Medicine A and Medicine B is how you take them.

Medicine A	Medicine B
You take a tablet only once in your life. You will be given the tablet on the day you come in to see your GP. 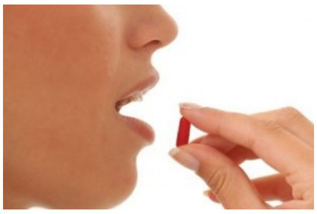	One tablet should be taken once a week on the same day every week.<br>You need to take this medicine for 5 years.<br><br>You need to take the tablet first thing in the morning on an empty stomach with a large glass of tap water.<br>After taking the tablet, you should stay upright for the next 30 minutes.<br>You should not eat anything for at least 30 minutes, including taking any other medicines.<br><br>You need to regularly visit your doctor to have your bone health checked.<br>You need to regularly visit a pharmacy for a new supply.<br>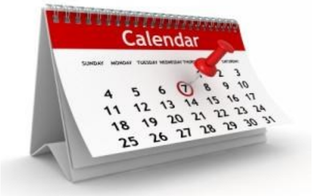 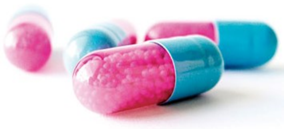

If you take Medicine A, you would live for **3.5 years** (3 years and 6 months) and then die.

If you take Medicine B, you would live for **5 years** and then die.

Would you prefer Medicine A or Medicine B, or are they the same?

Indicate your choice here. You can only choose one option:

○ **Medicine A** Live for 3 years 6 months

○ **Medicine B** Live for 5 years

○ **The Same - Medicine A and Medicine B are the same.**

0% 100%

Repeated section

Remember Medicine A and Medicine B are equally effective and have no risks of a side effect.

The only difference between Medicine A and Medicine B is how you take them.

Medicine A	Medicine B
You take a tablet only once in your life. You will be given the tablet on the day you come in to see your GP.<br>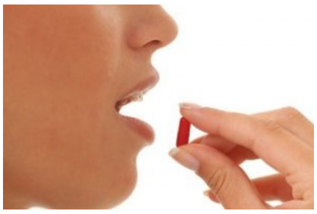	One tablet should be taken once a week on the same day every week.<br>You need to take this medicine for 5 years.<br><br>You need to take the tablet first thing in the morning on an empty stomach with a large glass of tap water.<br>After taking the tablet, you should stay upright for the next 30 minutes.<br>You should not eat anything for at least 30 minutes, including taking any other medicines.<br><br>You need to regularly visit your doctor to have your bone health checked.<br>You need to regularly visit a pharmacy for a new supply.<br>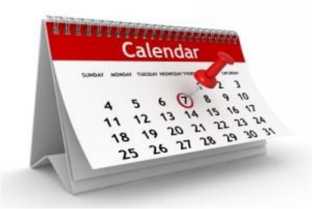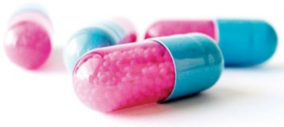

If you take Medicine A, you would live for **3.75 years** (3 years and 9 months) and then die.

If you take Medicine B, you would live for **5 years** and then die.

Would you prefer Medicine A or Medicine B, or are they the same?

Indicate your choice here. You can only choose one option:

○ **Medicine A** Live for 3 years 9 months

○ **Medicine B** Live for 5 years

○ **The Same - Medicine A and Medicine B are the same.**

0% 100%

Repeated section

Remember Medicine A and Medicine B are equally effective and have no risks of a side effect.

The only difference between Medicine A and Medicine B is how you take them.

Medicine A	Medicine B
You take a tablet only once in your life. You will be given the tablet on the day you come in to see your GP. 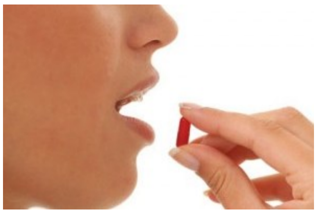	One tablet should be taken once a week on the same day every week. You need to take this medicine for 5 years. You need to take the tablet first thing in the morning on an empty stomach with a large glass of tap water. After taking the tablet, you should stay upright for the next 30 minutes. You should not eat anything for at least 30 minutes, including taking any other medicines. You need to regularly visit your doctor to have your bone health checked. You need to regularly visit a pharmacy for a new supply. 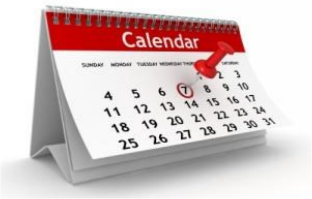 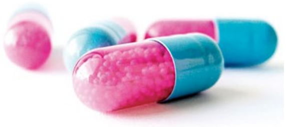

If you take Medicine A, you would live for **3 years and 10 months** and then die.

If you take Medicine B, you would live for **5 years** and then die.

Would you prefer Medicine A or Medicine B, or are they the same?

Indicate your choice here. You can only choose one option:

◯ **Medicine A** 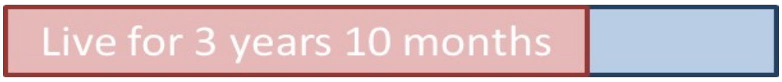

◯ **Medicine B** 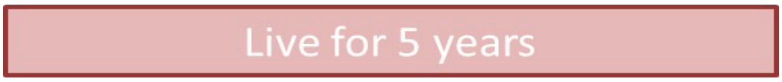

◯ **The Same - Medicine A and Medicine B are the same.**

0% 100%

Repeated section

Remember Medicine A and Medicine B are equally effective and have no risks of a side effect.

The only difference between Medicine A and Medicine B is how you take them.

Medicine A	Medicine B
You take a tablet only once in your life. You will be given the tablet on the day you come in to see your GP. 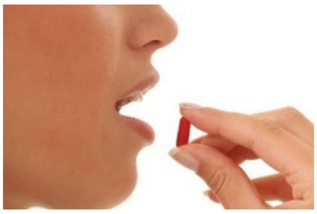	One tablet should be taken once a week on the same day every week. You need to take this medicine for 5 years. <br>You need to take the tablet first thing in the morning on an empty stomach with a large glass of tap water. After taking the tablet, you should stay upright for the next 30 minutes. You should not eat anything for at least 30 minutes, including taking any other medicines. <br>You need to regularly visit your doctor to have your bone health checked. You need to regularly visit a pharmacy for a new supply. 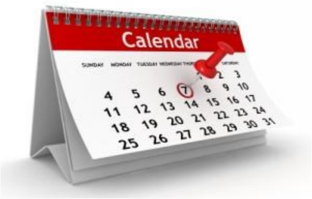 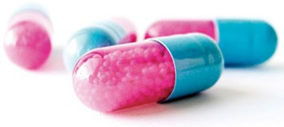

If you take Medicine A, you would live for **4 years** and then die.

If you take Medicine B, you would live for **5 years** and then die.

Would you prefer Medicine A or Medicine B, or are they the same?

Indicate your choice here. You can only choose one option:

◯ **Medicine A** Live for 4 years

◯ **Medicine B** Live for 5 years

◯ **The Same - Medicine A and Medicine B are the same.**

0% 100%

Repeated section

Remember Medicine A and Medicine B are equally effective and have no risks of a side effect.

The only difference between Medicine A and Medicine B is how you take them.

<table>
<tr><th>Medicine A</th><th>Medicine B</th></tr>
<tr><td>You take a tablet only once in your life. You will be given the tablet on the day you come in to see your GP.
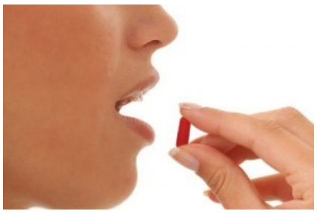</td><td>One tablet should be taken once a week on the same day every week.
You need to take this medicine for 5 years.

You need to take the tablet first thing in the morning on an empty stomach with a large glass of tap water.
After taking the tablet, you should stay upright for the next 30 minutes.
You should not eat anything for at least 30 minutes, including taking any other medicines.

You need to regularly visit your doctor to have your bone health checked.
You need to regularly visit a pharmacy for a new supply.
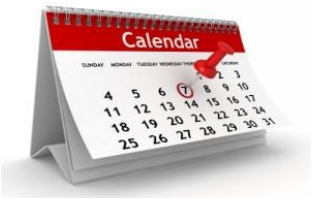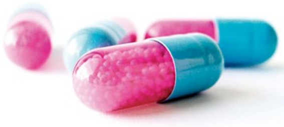</td></tr>
</table>

If you take Medicine A, you would live for **4 years and 2 months** and then die.

If you take Medicine B, you would live for **5 years** and then die.

Would you prefer Medicine A or Medicine B, or are they the same?

Indicate your choice here. You can only choose one option:

○ **Medicine A** Live for 4 years 2 months

○ **Medicine B** Live for 5 years

○ **The Same - Medicine A and Medicine B are the same.**

0% 100%

Repeated section

Remember Medicine A and Medicine B are equally effective and have no risks of a side effect.

The only difference between Medicine A and Medicine B is how you take them.

Medicine A	Medicine B
You take a tablet only once in your life. You will be given the tablet on the day you come in to see your GP. 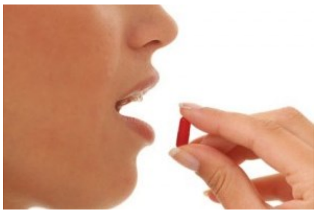	One tablet should be taken once a week on the same day every week. You need to take this medicine for 5 years.<br><br>You need to take the tablet first thing in the morning on an empty stomach with a large glass of tap water. After taking the tablet, you should stay upright for the next 30 minutes. You should not eat anything for at least 30 minutes, including taking any other medicines.<br><br>You need to regularly visit your doctor to have your bone health checked. You need to regularly visit a pharmacy for a new supply. 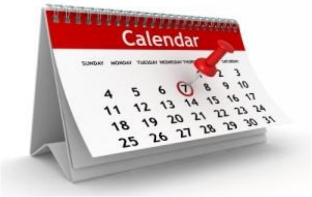 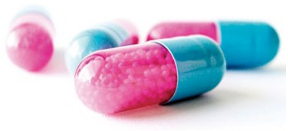

If you take Medicine A, you would live for **4.25 years** (4 years and 3 months) and then die.

If you take Medicine B, you would live for **5 years** and then die.

Would you prefer Medicine A or Medicine B, or are they the same?

Indicate your choice here. You can only choose one option:

- ◯ **Medicine A** — Live for 4 years 3 months
- ◯ **Medicine B** — Live for 5 years
- ◯ **The Same - Medicine A and Medicine B are the same.**

0% 100%

Repeated section

Remember Medicine A and Medicine B are equally effective and have no risks of a side effect.

The only difference between Medicine A and Medicine B is how you take them.

Medicine A	Medicine B
You take a tablet only once in your life. You will be given the tablet on the day you come in to see your GP.<br>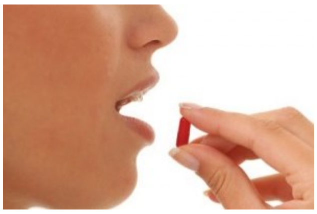	One tablet should be taken once a week on the same day every week.<br>You need to take this medicine for 5 years.<br><br>You need to take the tablet first thing in the morning on an empty stomach with a large glass of tap water.<br>After taking the tablet, you should stay upright for the next 30 minutes.<br>You should not eat anything for at least 30 minutes, including taking any other medicines.<br><br>You need to regularly visit your doctor to have your bone health checked.<br>You need to regularly visit a pharmacy for a new supply.<br>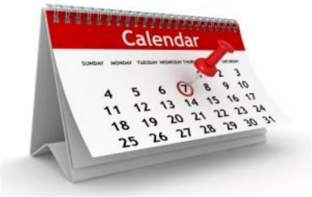<br>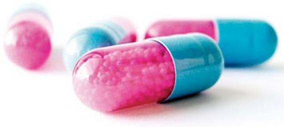

If you take Medicine A, you would live for **4 years 4 months** and then die.

If you take Medicine B, you would live for **5 years** and then die.

Would you prefer Medicine A or Medicine B, or are they the same?

Indicate your choice here. You can only choose one option:

◯ **Medicine A** Live for 4 years 4 months

◯ **Medicine B** Live for 5 years

◯ **The Same - Medicine A and Medicine B are the same.**

0% 100%

Repeated section

Remember Medicine A and Medicine B are equally effective and have no risks of a side effect.

The only difference between Medicine A and Medicine B is how you take them.

**Medicine A**	**Medicine B**
You take a tablet only once in your life. You will be given the tablet on the day you come in to see your GP.<br>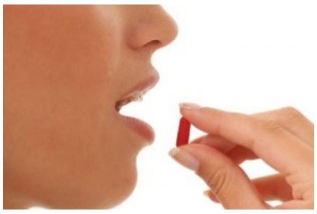	One tablet should be taken once a week on the same day every week.<br>You need to take this medicine for 5 years.<br><br>You need to take the tablet first thing in the morning on an empty stomach with a large glass of tap water.<br>After taking the tablet, you should stay upright for the next 30 minutes.<br>You should not eat anything for at least 30 minutes, including taking any other medicines.<br><br>You need to regularly visit your doctor to have your bone health checked.<br>You need to regularly visit a pharmacy for a new supply.<br>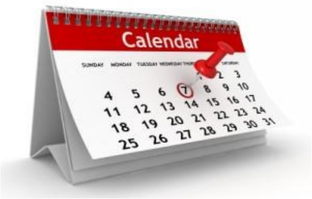 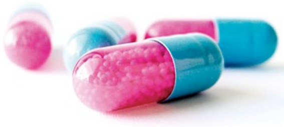

If you take Medicine A, you would live for **4.5 years** (4 years and 6 months) and then die.

If you take Medicine B, you would live for **5 years** and then die.

Would you prefer Medicine A or Medicine B, or are they the same?

Indicate your choice here. You can only choose one option:

○ **Medicine A** Live for 4 years 6 months

○ **Medicine B** Live for 5 years

○ **The Same - Medicine A and Medicine B are the same.**

0% 100%

Repeated section

Remember Medicine A and Medicine B are equally effective and have no risks of a side effect.

The only difference between Medicine A and Medicine B is how you take them.

Medicine A	Medicine B
You take a tablet only once in your life. You will be given the tablet on the day you come in to see your GP. 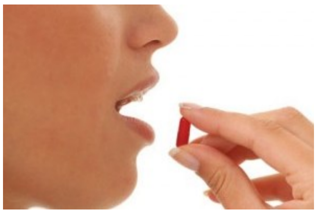	One tablet should be taken once a week on the same day every week. You need to take this medicine for 5 years.<br><br>You need to take the tablet first thing in the morning on an empty stomach with a large glass of tap water. After taking the tablet, you should stay upright for the next 30 minutes. You should not eat anything for at least 30 minutes, including taking any other medicines.<br><br>You need to regularly visit your doctor to have your bone health checked. You need to regularly visit a pharmacy for a new supply. 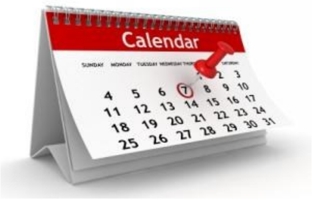 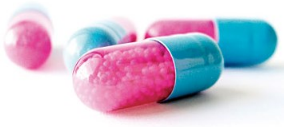

If you take Medicine A, you would live for **4 years and 7 months** and then die.

If you take Medicine B, you would live for **5 years** and then die.

Would you prefer Medicine A or Medicine B, or are they the same?

Indicate your choice here. You can only choose one option:

◯ **Medicine A** Live for 4 years 7 months

◯ **Medicine B** Live for 5 years

◯ **The Same - Medicine A and Medicine B are the same.**

0% 100%

Repeated section

Remember Medicine A and Medicine B are equally effective and have no risks of a side effect.

The only difference between Medicine A and Medicine B is how you take them.

Medicine A	Medicine B
You take a tablet only once in your life. You will be given the tablet on the day you come in to see your GP. 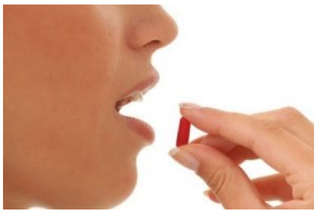	One tablet should be taken once a week on the same day every week.<br>You need to take this medicine for 5 years.<br><br>You need to take the tablet first thing in the morning on an empty stomach with a large glass of tap water.<br>After taking the tablet, you should stay upright for the next 30 minutes.<br>You should not eat anything for at least 30 minutes, including taking any other medicines.<br><br>You need to regularly visit your doctor to have your bone health checked.<br>You need to regularly visit a pharmacy for a new supply.<br>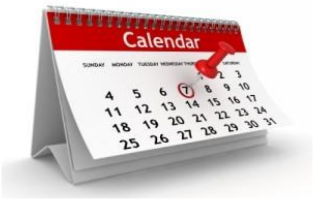 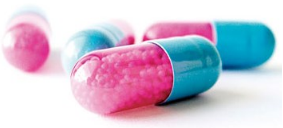

If you take Medicine A, you would live for **4 years and 9 months** and then die.

If you take Medicine B, you would live for **5 years** and then die.

Would you prefer Medicine A or Medicine B, or are they the same?

Indicate your choice here. You can only choose one option:

○ **Medicine A**

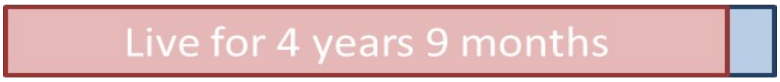

○ **Medicine B**

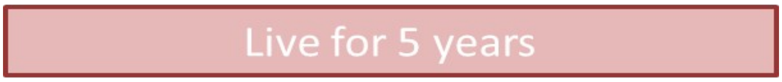

○ **The Same - Medicine A and Medicine B are the same.**

0% 100%

Repeated section

Remember Medicine A and Medicine B are equally effective and have no risks of a side effect.

The only difference between Medicine A and Medicine B is how you take them.

Medicine A	Medicine B
You take a tablet only once in your life. You will be given the tablet on the day you come in to see your GP.<br>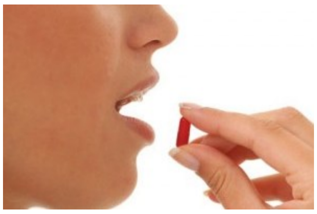	One tablet should be taken once a week on the same day every week.<br>You need to take this medicine for 5 years.<br><br>You need to take the tablet first thing in the morning on an empty stomach with a large glass of tap water.<br>After taking the tablet, you should stay upright for the next 30 minutes.<br>You should not eat anything for at least 30 minutes, including taking any other medicines.<br><br>You need to regularly visit your doctor to have your bone health checked.<br>You need to regularly visit a pharmacy for a new supply.<br>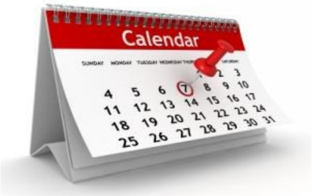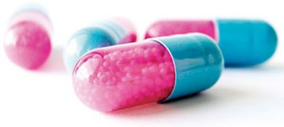

If you take Medicine A, you would live for **2 months less than 5 years** and then die.

If you take Medicine B, you would live for **5 years** and then die.

Would you prefer Medicine A or Medicine B, or are they the same?

Indicate your choice here. You can only choose one option:

◯ **Medicine A** Live for (5 years – 2 months)

◯ **Medicine B** Live for 5 years

◯ **The Same - Medicine A and Medicine B are the same.**

0% 100%

Repeated section

Remember Medicine A and Medicine B are equally effective and have no risks of a side effect.

The only difference between Medicine A and Medicine B is how you take them.

Medicine A	Medicine B
You take a tablet only once in your life. You will be given the tablet on the day you come in to see your GP.<br>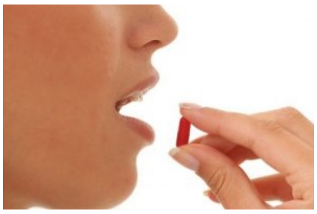	One tablet should be taken once a week on the same day every week.<br>You need to take this medicine for 5 years.<br><br>You need to take the tablet first thing in the morning on an empty stomach with a large glass of tap water.<br>After taking the tablet, you should stay upright for the next 30 minutes.<br>You should not eat anything for at least 30 minutes, including taking any other medicines.<br><br>You need to regularly visit your doctor to have your bone health checked.<br>You need to regularly visit a pharmacy for a new supply.<br>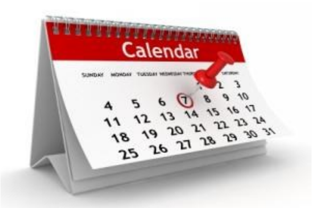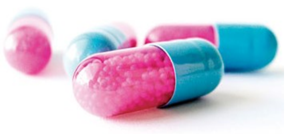

If you take Medicine A, you would live for **1 month less than 5 years** and then die.

If you take Medicine B, you would live for **5 years** and then die.

Would you prefer Medicine A or Medicine B, or are they the same?

Indicate your choice here. You can only choose one option:

◯ **Medicine A** Live for (5 years – 1 month)

◯ **Medicine B** Live for 5 years

◯ **The Same - Medicine A and Medicine B are the same.**

0% 100%

Repeated section

Remember Medicine A and Medicine B are equally effective and have no risks of a side effect.

The only difference between Medicine A and Medicine B is how you take them.

Medicine A	Medicine B
You take a tablet only once in your life. You will be given the tablet on the day you come in to see your GP.<br>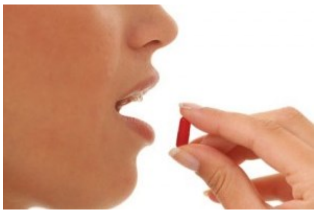	One tablet should be taken once a week on the same day every week.<br>You need to take this medicine for 5 years.<br><br>You need to take the tablet first thing in the morning on an empty stomach with a large glass of tap water.<br>After taking the tablet, you should stay upright for the next 30 minutes.<br>You should not eat anything for at least 30 minutes, including taking any other medicines.<br><br>You need to regularly visit your doctor to have your bone health checked.<br>You need to regularly visit a pharmacy for a new supply.<br>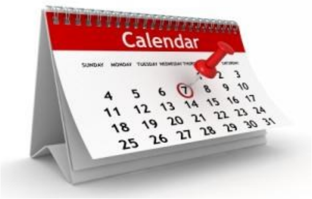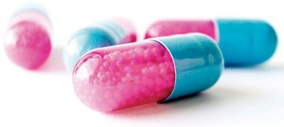

If you take Medicine A, you would live for **2 weeks less than 5 years** and then die.

If you take Medicine B, you would live for **5 years** and then die.

Would you prefer Medicine A or Medicine B, or are they the same?

Indicate your choice here. You can only choose one option:

◯ **Medicine A** Live for (5 years – 2 weeks)

◯ **Medicine B** Live for 5 years

◯ **The Same - Medicine A and Medicine B are the same.**

0% 100%

Repeated section

Remember Medicine A and Medicine B are equally effective and have no risks of a side effect.

The only difference between Medicine A and Medicine B is how you take them.

Medicine A	Medicine B
You take a tablet only once in your life.<br>You will be given the tablet on the day you come in to see your GP.<br>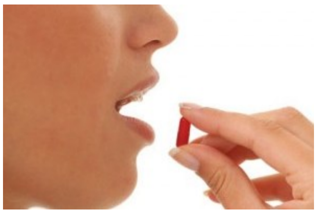	One tablet should be taken once a week on the same day every week.<br>You need to take this medicine for 5 years.<br><br>You need to take the tablet first thing in the morning on an empty stomach with a large glass of tap water.<br>After taking the tablet, you should stay upright for the next 30 minutes.<br>You should not eat anything for at least 30 minutes, including taking any other medicines.<br><br>You need to regularly visit your doctor to have your bone health checked.<br>You need to regularly visit a pharmacy for a new supply.<br>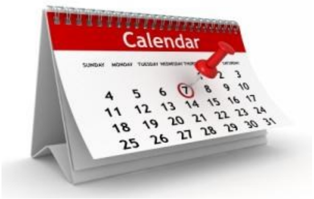 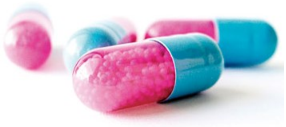

If you take Medicine A, you would live for **1 week less than 5 years** and then die.

If you take Medicine B, you would live for **5 years** and then die.

Would you prefer Medicine A or Medicine B, or are they the same?

Indicate your choice here. You can only choose one option:

◯ **Medicine A** Live for (5 years – 1 week)

◯ **Medicine B** Live for 5 years

◯ **The Same - Medicine A and Medicine B are the same.**

0% 100%

Repeated section

Remember Medicine A and Medicine B are equally effective and have no risks of a side effect.

The only difference between Medicine A and Medicine B is how you take them.

Medicine A	Medicine B
You take a tablet only once in your life. You will be given the tablet on the day you come in to see your GP.<br>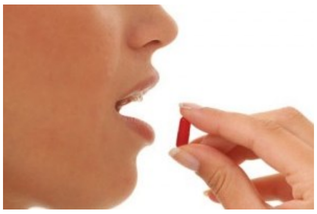	One tablet should be taken once a week on the same day every week.<br>You need to take this medicine for 5 years.<br><br>You need to take the tablet first thing in the morning on an empty stomach with a large glass of tap water.<br>After taking the tablet, you should stay upright for the next 30 minutes.<br>You should not eat anything for at least 30 minutes, including taking any other medicines.<br><br>You need to regularly visit your doctor to have your bone health checked.<br>You need to regularly visit a pharmacy for a new supply.<br>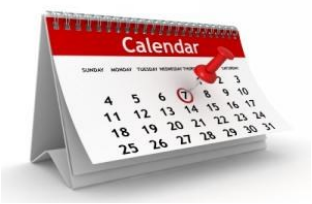 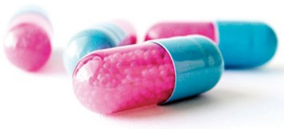

If you take Medicine A, you would live for **5 days less than 5 years** and then die.

If you take Medicine B, you would live for **5 years** and then die.

Would you prefer Medicine A or Medicine B, or are they the same?

Indicate your choice here. You can only choose one option:

◯ **Medicine A** Live for (5 years – 5 days)

◯ **Medicine B** Live for 5 years

◯ **The Same - Medicine A and Medicine B are the same.**

0% 100%

Repeated section

Remember Medicine A and Medicine B are equally effective and have no risks of a side effect.

The only difference between Medicine A and Medicine B is how you take them.

Medicine A	Medicine B
You take a tablet only once in your life.<br>You will be given the tablet on the day you come in to see your GP.<br>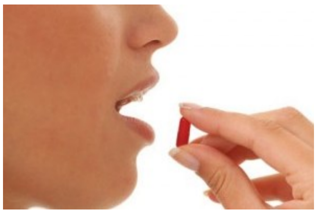	One tablet should be taken once a week on the same day every week.<br>You need to take this medicine for 5 years.<br><br>You need to take the tablet first thing in the morning on an empty stomach with a large glass of tap water.<br>After taking the tablet, you should stay upright for the next 30 minutes.<br>You should not eat anything for at least 30 minutes, including taking any other medicines.<br><br>You need to regularly visit your doctor to have your bone health checked.<br>You need to regularly visit a pharmacy for a new supply.<br>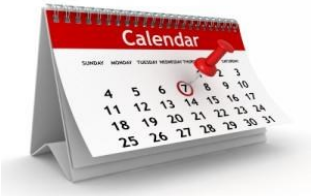 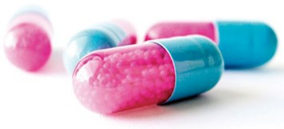

If you take Medicine A, you would live for **3 days less than 5 years** and then die.

If you take Medicine B, you would live for **5 years** and then die.

Would you prefer Medicine A or Medicine B, or are they the same?

Indicate your choice here. You can only choose one option:

○ **Medicine A** Live for (5 years – 3 days)

○ **Medicine B** Live for 5 years

○ **The Same - Medicine A and Medicine B are the same.**

0% 100%

Repeated section

Remember Medicine A and Medicine B are equally effective and have no risks of a side effect.

The only difference between Medicine A and Medicine B is how you take them.

Medicine A	Medicine B
You take a tablet only once in your life.<br>You will be given the tablet on the day you come in to see your GP.<br>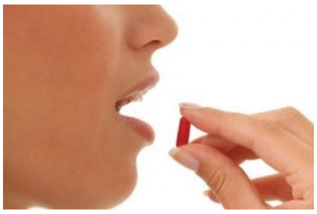	One tablet should be taken once a week on the same day every week.<br>You need to take this medicine for 5 years.<br><br>You need to take the tablet first thing in the morning on an empty stomach with a large glass of tap water.<br>After taking the tablet, you should stay upright for the next 30 minutes.<br>You should not eat anything for at least 30 minutes, including taking any other medicines.<br><br>You need to regularly visit your doctor to have your bone health checked.<br>You need to regularly visit a pharmacy for a new supply.<br>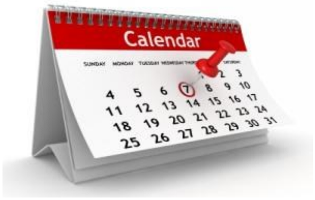 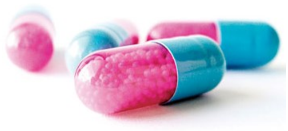

If you take Medicine A, you would live for **1 day less than 5 years** and then die.

If you take Medicine B, you would live for **5 years** and then die.

Would you prefer Medicine A or Medicine B, or are they the same?

Indicate your choice here. You can only choose one option:

◯ **Medicine A** Live for (5 years – 1 day)

◯ **Medicine B** Live for 5 years

◯ **The Same - Medicine A and Medicine B are the same.**

0% 100%

Question 1a.

You prefer living for 2.5 years (2 years and 6 months) on Medicine A, to living for 5 years on Medicine B.

This means **living fewer than 2.5 years on Medicine A** can be the same as living for 5 years on Medicine B.

How many years on Medicine A do you think would be the same as living for 5 years on Medicine B?

(Please give a number in years)

0% 100%

Part 2

Question 2

You have been told that you need to take a medicine to reduce the risk of fracture.

You can choose between two different medicines. We have called these Medicine A and Medicine B.

Medicine A is a pill that you only need to take once.

Medicine B is a pill that you need to take every week for 5 years.

Medicine A and Medicine B are equally effective in reducing your risk of having a fracture.
Medicine A and Medicine B will reduce your risk of experiencing a fracture, such as hip fracture, within the next 5 years from **10%** to **6%**.

In other words, in a crowd of **1,000** people in the UK, **100** are likely to have a fracture if they do not take the medicine. This is reduced to **60** people out of 1,000 who do take the medicine.
This information is also illustrated in this picture.

Risk of fracture

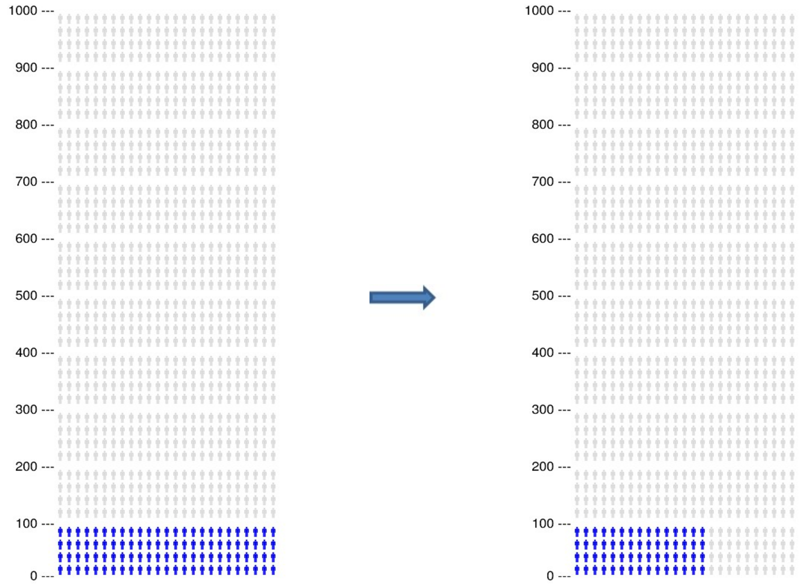

10%
No Treatment

6%
Taking the Medicine

(The figures shaded blue represent people who will have a fracture, such as hip fracture, within the next 5 years)

In this scenario, there is some risk of a minor side effect from Medicine A or Medicine B.

Among every **1,000** people taking Medicine A or Medicine B, **50** people are likely to have a <u>minor side effect</u> such as nausea (feeling sick) and indigestion.

Risk of a minor side effect

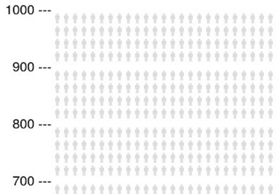

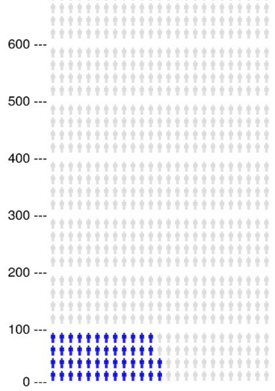

5% of people will have a minor side effect.

0% 100%

Repeated section

Remember Medicine A and Medicine B are equally effective and have some risk of a minor side effect.

The only difference between Medicine A and Medicine B is how you take them.

Medicine A	Medicine B
You take a tablet only once in your life.<br>You will be given the tablet on the day you come in to see your GP.<br>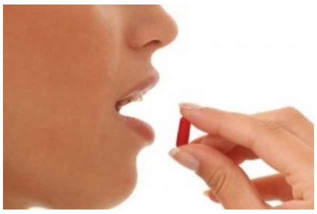	One tablet should be taken once a week on the same day every week.<br>You need to take this medicine for 5 years.<br><br>You need to take the tablet first thing in the morning on an empty stomach with a large glass of tap water.<br>After taking the tablet, you should stay upright for the next 30 minutes.<br>You should not eat anything for at least 30 minutes, including taking any other medicines.<br><br>You need to regularly visit your doctor to have your bone health checked.<br>You need to regularly visit a pharmacy for a new supply.<br>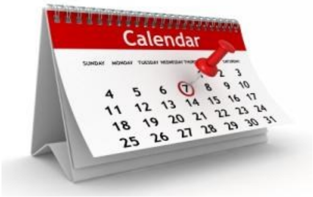 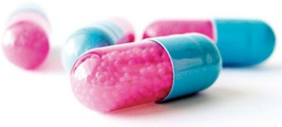

If you take Medicine A, you would live for **2.5 years (2 years and 6 months)** and then die.

If you take Medicine B, you would live for **5 years** and then die.

Would you prefer Medicine A or Medicine B, or are they the same?

Indicate your choice here. You can only choose one option:

○ **Medicine A**

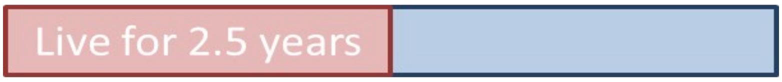

○ **Medicine B**

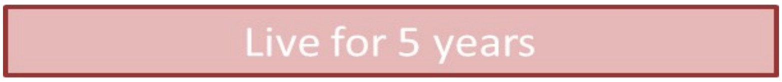

○ **The Same - Medicine A and Medicine B are the same.**

0% 100%

Repeated section

Remember Medicine A and Medicine B are equally effective and have some risk of a minor side effect.

The only difference between Medicine A and Medicine B is how you take them.

Medicine A	Medicine B
You take a tablet only once in your life.<br>You will be given the tablet on the day you come in to see your GP.<br>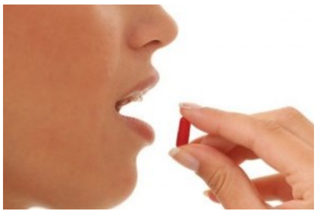	One tablet should be taken once a week on the same day every week.<br>You need to take this medicine for 5 years.<br><br>You need to take the tablet first thing in the morning on an empty stomach with a large glass of tap water.<br>After taking the tablet, you should stay upright for the next 30 minutes.<br>You should not eat anything for at least 30 minutes, including taking any other medicines.<br><br>You need to regularly visit your doctor to have your bone health checked.<br>You need to regularly visit a pharmacy for a new supply.<br>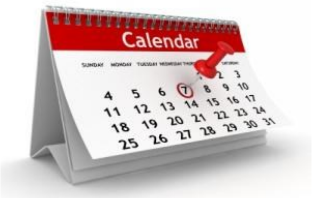 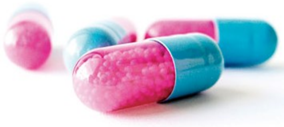

If you take Medicine A, you would live for **3 years** and then die.

If you take Medicine B, you would live for **5 years** and then die.

Would you prefer Medicine A or Medicine B, or are they the same?

Indicate your choice here. You can only choose one option:

◯ **Medicine A** Live for 3 years

◯ **Medicine B** Live for 5 years

◯ **The Same - Medicine A and Medicine B are the same.**

0% 100%

Repeated section

Remember Medicine A and Medicine B are equally effective and have some risk of a minor side effect.

The only difference between Medicine A and Medicine B is how you take them.

Medicine A	Medicine B
You take a tablet only once in your life. You will be given the tablet on the day you come in to see your GP. 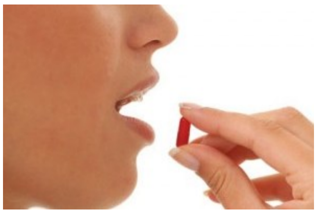	One tablet should be taken once a week on the same day every week. You need to take this medicine for 5 years.<br><br>You need to take the tablet first thing in the morning on an empty stomach with a large glass of tap water. After taking the tablet, you should stay upright for the next 30 minutes. You should not eat anything for at least 30 minutes, including taking any other medicines.<br><br>You need to regularly visit your doctor to have your bone health checked. You need to regularly visit a pharmacy for a new supply. 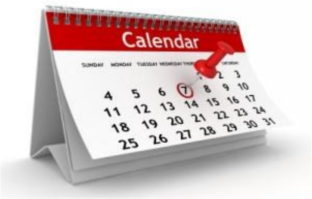 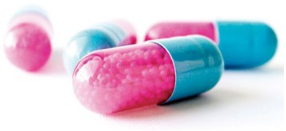

If you take Medicine A, you would live for **3.25 years** (3 years and 3 months) and then die.

If you take Medicine B, you would live for **5 years** and then die.

Would you prefer Medicine A or Medicine B, or are they the same?

Indicate your choice here. You can only choose one option:

○ **Medicine A** Live for 3 years 3 months

○ **Medicine B** Live for 5 years

○ **The Same - Medicine A and Medicine B are the same.**

0% 100%

Repeated section

Remember Medicine A and Medicine B are equally effective and have some risk of a minor side effect.

The only difference between Medicine A and Medicine B is how you take them.

Medicine A	Medicine B
You take a tablet only once in your life. You will be given the tablet on the day you come in to see your GP. 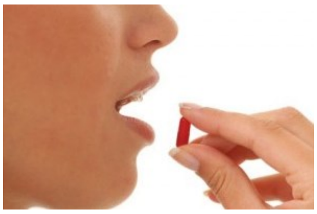	One tablet should be taken once a week on the same day every week. You need to take this medicine for 5 years.<br><br>You need to take the tablet first thing in the morning on an empty stomach with a large glass of tap water. After taking the tablet, you should stay upright for the next 30 minutes. You should not eat anything for at least 30 minutes, including taking any other medicines.<br><br>You need to regularly visit your doctor to have your bone health checked. You need to regularly visit a pharmacy for a new supply. 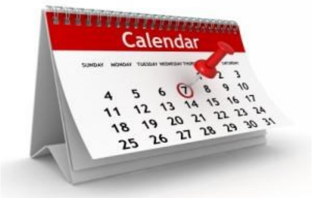 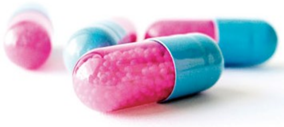

If you take Medicine A, you would live for **3.5 years** (3 years and 6 months) and then die.

If you take Medicine B, you would live for **5 years** and then die.

Would you prefer Medicine A or Medicine B, or are they the same?

Indicate your choice here. You can only choose one option:

○ **Medicine A** 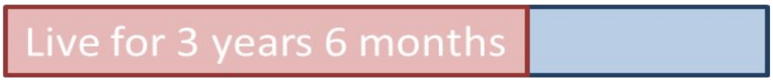

○ **Medicine B** 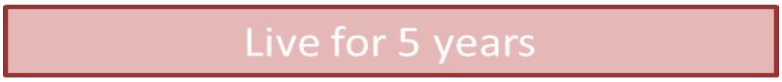

○ **The Same - Medicine A and Medicine B are the same.**

0% 100%

Repeated section

Remember Medicine A and Medicine B are equally effective and have some risk of a minor side effect.

The only difference between Medicine A and Medicine B is how you take them.

Medicine A	Medicine B
You take a tablet only once in your life.<br>You will be given the tablet on the day you come in to see your GP.<br>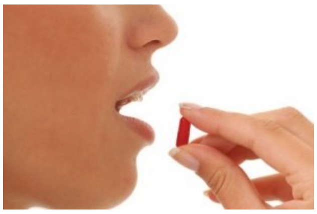	One tablet should be taken once a week on the same day every week.<br>You need to take this medicine for 5 years.<br><br>You need to take the tablet first thing in the morning on an empty stomach with a large glass of tap water.<br>After taking the tablet, you should stay upright for the next 30 minutes.<br>You should not eat anything for at least 30 minutes, including taking any other medicines.<br><br>You need to regularly visit your doctor to have your bone health checked.<br>You need to regularly visit a pharmacy for a new supply.<br>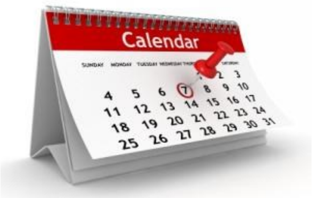 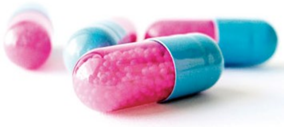

If you take Medicine A, you would live for **3.75 years** (3 years and 9 months) and then die.

If you take Medicine B, you would live for **5 years** and then die.

Would you prefer Medicine A or Medicine B, or are they the same?

Indicate your choice here. You can only choose one option:

◯ **Medicine A** 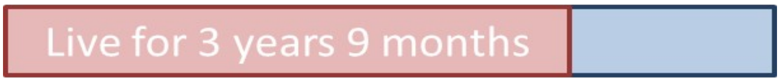

◯ **Medicine B** 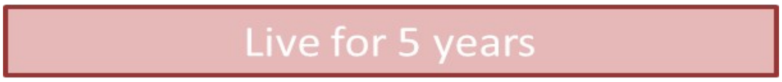

◯ **The Same - Medicine A and Medicine B are the same.**

0% 100%

Repeated section

Remember Medicine A and Medicine B are equally effective and have some risk of a minor side effect.

The only difference between Medicine A and Medicine B is how you take them.

Medicine A	Medicine B
You take a tablet only once in your life.<br>You will be given the tablet on the day you come in to see your GP.<br>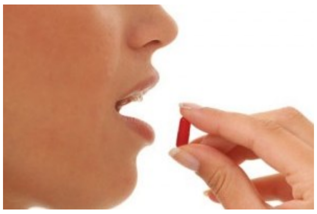	One tablet should be taken once a week on the same day every week.<br>You need to take this medicine for 5 years.<br><br>You need to take the tablet first thing in the morning on an empty stomach with a large glass of tap water.<br>After taking the tablet, you should stay upright for the next 30 minutes.<br>You should not eat anything for at least 30 minutes, including taking any other medicines.<br><br>You need to regularly visit your doctor to have your bone health checked.<br>You need to regularly visit a pharmacy for a new supply.<br>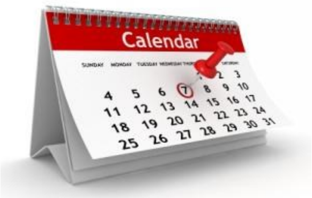 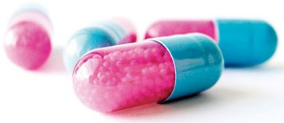

If you take Medicine A, you would live for **3 years and 10 months** and then die.

If you take Medicine B, you would live for **5 years** and then die.

Would you prefer Medicine A or Medicine B, or are they the same?

Indicate your choice here. You can only choose one option:

- ◯ **Medicine A** — Live for 3 years 10 months
- ◯ **Medicine B** — Live for 5 years
- ◯ **The Same - Medicine A and Medicine B are the same.**

0% 100%

Repeated section

Remember Medicine A and Medicine B are equally effective and have some risk of a minor side effect.

The only difference between Medicine A and Medicine B is how you take them.

Medicine A	Medicine B
You take a tablet only once in your life. You will be given the tablet on the day you come in to see your GP.<br>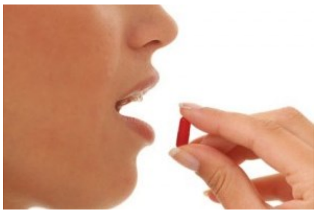	One tablet should be taken once a week on the same day every week.<br>You need to take this medicine for 5 years.<br><br>You need to take the tablet first thing in the morning on an empty stomach with a large glass of tap water.<br>After taking the tablet, you should stay upright for the next 30 minutes.<br>You should not eat anything for at least 30 minutes, including taking any other medicines.<br><br>You need to regularly visit your doctor to have your bone health checked.<br>You need to regularly visit a pharmacy for a new supply.<br>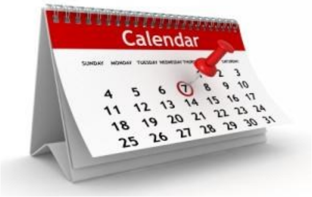 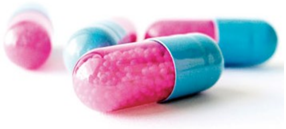

If you take Medicine A, you would live for **4 years** and then die.

If you take Medicine B, you would live for **5 years** and then die.

Would you prefer Medicine A or Medicine B, or are they the same?

Indicate your choice here. You can only choose one option:

◯ **Medicine A**

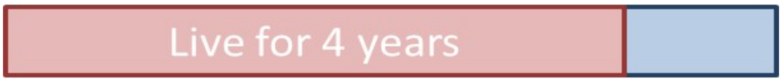

◯ **Medicine B**

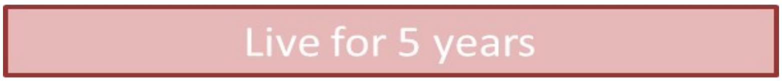

◯ **The Same - Medicine A and Medicine B are the same.**

0% 100%

Repeated section

Remember Medicine A and Medicine B are equally effective and have some risk of a minor side effect.

The only difference between Medicine A and Medicine B is how you take them.

Medicine A	Medicine B
You take a tablet only once in your life. You will be given the tablet on the day you come in to see your GP. 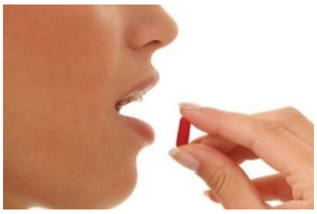	One tablet should be taken once a week on the same day every week.<br>You need to take this medicine for 5 years.<br><br>You need to take the tablet first thing in the morning on an empty stomach with a large glass of tap water.<br>After taking the tablet, you should stay upright for the next 30 minutes.<br>You should not eat anything for at least 30 minutes, including taking any other medicines.<br><br>You need to regularly visit your doctor to have your bone health checked.<br>You need to regularly visit a pharmacy for a new supply.<br>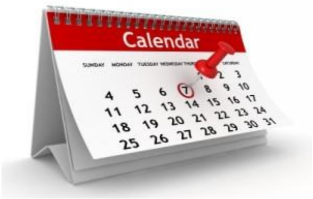 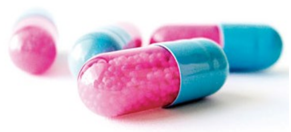

If you take Medicine A, you would live for **4 years and 2 months** and then die.

If you take Medicine B, you would live for **5 years** and then die.

Would you prefer Medicine A or Medicine B, or are they the same?

Indicate your choice here. You can only choose one option:

○ **Medicine A**

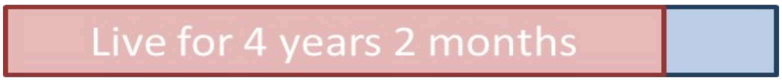

○ **Medicine B**

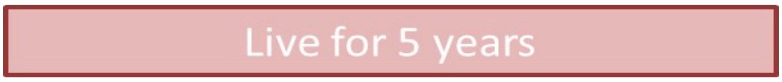

○ **The Same - Medicine A and Medicine B are the same.**

0% 100%

Repeated section

Remember Medicine A and Medicine B are equally effective and have some risk of a minor side effect.

The only difference between Medicine A and Medicine B is how you take them.

Medicine A	Medicine B
You take a tablet only once in your life. You will be given the tablet on the day you come in to see your GP.<br>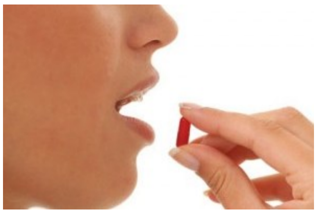	One tablet should be taken once a week on the same day every week.<br>You need to take this medicine for 5 years.<br><br>You need to take the tablet first thing in the morning on an empty stomach with a large glass of tap water.<br>After taking the tablet, you should stay upright for the next 30 minutes.<br>You should not eat anything for at least 30 minutes, including taking any other medicines.<br><br>You need to regularly visit your doctor to have your bone health checked.<br>You need to regularly visit a pharmacy for a new supply.<br>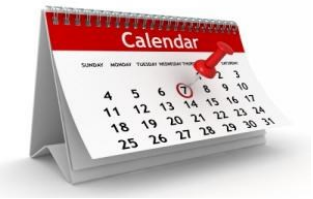 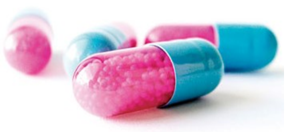

If you take Medicine A, you would live for **4.25 years** (4 years and 3 months) and then die.

If you take Medicine B, you would live for **5 years** and then die.

Would you prefer Medicine A or Medicine B, or are they the same?

Indicate your choice here. You can only choose one option:

○ **Medicine A** Live for 4 years 3 months

○ **Medicine B** Live for 5 years

○ **The Same - Medicine A and Medicine B are the same.**

0% 100%

Repeated section

Remember Medicine A and Medicine B are equally effective and have some risk of a minor side effect.

The only difference between Medicine A and Medicine B is how you take them.

Medicine A	Medicine B
You take a tablet only once in your life. You will be given the tablet on the day you come in to see your GP.<br>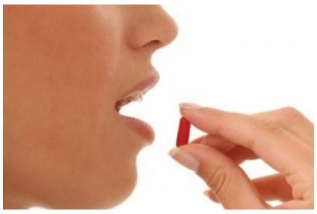	One tablet should be taken once a week on the same day every week.<br>You need to take this medicine for 5 years.<br><br>You need to take the tablet first thing in the morning on an empty stomach with a large glass of tap water.<br>After taking the tablet, you should stay upright for the next 30 minutes.<br>You should not eat anything for at least 30 minutes, including taking any other medicines.<br><br>You need to regularly visit your doctor to have your bone health checked.<br>You need to regularly visit a pharmacy for a new supply.<br>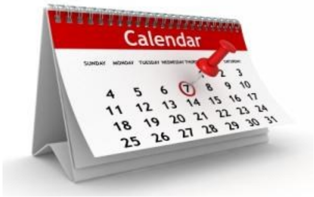 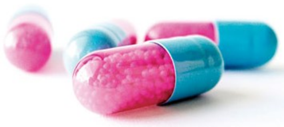

If you take Medicine A, you would live for **4 years 4 months** and then die.

If you take Medicine B, you would live for **5 years** and then die.

Would you prefer Medicine A or Medicine B, or are they the same?

Indicate your choice here. You can only choose one option:

○ **Medicine A** Live for 4 years 4 months

○ **Medicine B** Live for 5 years

○ **The Same - Medicine A and Medicine B are the same.**

0% 100%

Remember Medicine A and Medicine B are equally effective and have no risks of a minor side effect.

The only difference between Medicine A and Medicine B is how you take them.

Medicine A	Medicine B
You take a tablet only once in your life. You will be given the tablet on the day you come in to see your GP. 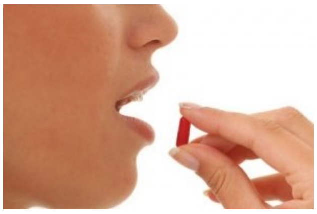	One tablet should be taken once a week on the same day every week. You need to take this medicine for 5 years. You need to take the tablet first thing in the morning on an empty stomach with a large glass of tap water. After taking the tablet, you should stay upright for the next 30 minutes. You should not eat anything for at least 30 minutes, including taking any other medicines. You need to regularly visit your doctor to have your bone health checked. You need to regularly visit a pharmacy for a new supply. 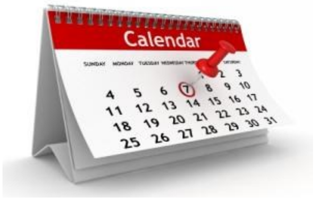 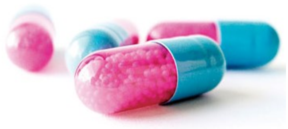

If you take Medicine A, you would live for **4.5 years** (4 years and 6 months) and then die.

If you take Medicine B, you would live for **5 years** and then die.

Would you prefer Medicine A or Medicine B, or are they the same?

Indicate your choice here. You can only choose one option:

○ **Medicine A** Live for 4 years 6 months

○ **Medicine B** Live for 5 years

○ **The Same - Medicine A and Medicine B are the same.**

Repeated section

Remember Medicine A and Medicine B are equally effective and have some risk of a minor side effect.

The only difference between Medicine A and Medicine B is how you take them.

Medicine A	Medicine B
You take a tablet only once in your life.<br>You will be given the tablet on the day you come in to see your GP.<br>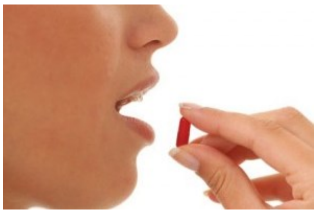	One tablet should be taken once a week on the same day every week.<br>You need to take this medicine for 5 years.<br><br>You need to take the tablet first thing in the morning on an empty stomach with a large glass of tap water.<br>After taking the tablet, you should stay upright for the next 30 minutes.<br>You should not eat anything for at least 30 minutes, including taking any other medicines.<br><br>You need to regularly visit your doctor to have your bone health checked.<br>You need to regularly visit a pharmacy for a new supply.<br>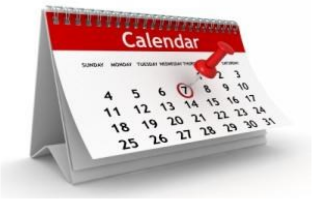 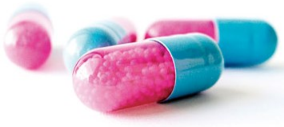

If you take Medicine A, you would live for **4 years and 7 months** and then die.

If you take Medicine B, you would live for **5 years** and then die.

Would you prefer Medicine A or Medicine B, or are they the same?

Indicate your choice here. You can only choose one option:

○ **Medicine A** Live for 4 years 7 months

○ **Medicine B** Live for 5 years

○ **The Same - Medicine A and Medicine B are the same.**

0% 100%

Repeated section

Remember Medicine A and Medicine B are equally effective and have some risk of a minor side effect.

The only difference between Medicine A and Medicine B is how you take them.

Medicine A	Medicine B
You take a tablet only once in your life. You will be given the tablet on the day you come in to see your GP.<br>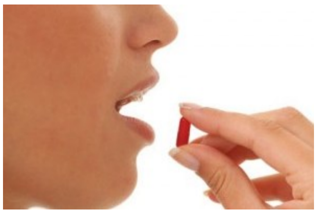	One tablet should be taken once a week on the same day every week.<br>You need to take this medicine for 5 years.<br><br>You need to take the tablet first thing in the morning on an empty stomach with a large glass of tap water.<br>After taking the tablet, you should stay upright for the next 30 minutes.<br>You should not eat anything for at least 30 minutes, including taking any other medicines.<br><br>You need to regularly visit your doctor to have your bone health checked.<br>You need to regularly visit a pharmacy for a new supply.<br>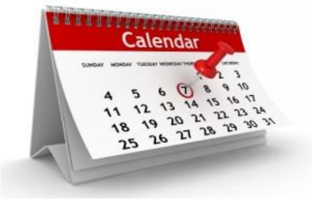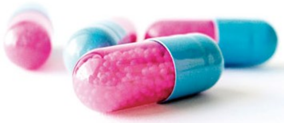

If you take Medicine A, you would live for **4 years and 9 months** and then die.

If you take Medicine B, you would live for **5 years** and then die.

Would you prefer Medicine A or Medicine B, or are they the same?

Indicate your choice here. You can only choose one option:

○ **Medicine A** Live for 4 years 9 months

○ **Medicine B** Live for 5 years

○ **The Same - Medicine A and Medicine B are the same.**

0% 100%

Repeated section

Remember Medicine A and Medicine B are equally effective and have some risk of a minor side effect.

The only difference between Medicine A and Medicine B is how you take them.

Medicine A	Medicine B
You take a tablet only once in your life.<br>You will be given the tablet on the day you come in to see your GP.<br>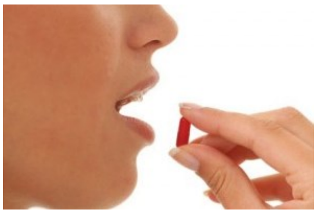	One tablet should be taken once a week on the same day every week.<br>You need to take this medicine for 5 years.<br><br>You need to take the tablet first thing in the morning on an empty stomach with a large glass of tap water.<br>After taking the tablet, you should stay upright for the next 30 minutes.<br>You should not eat anything for at least 30 minutes, including taking any other medicines.<br><br>You need to regularly visit your doctor to have your bone health checked.<br>You need to regularly visit a pharmacy for a new supply.<br>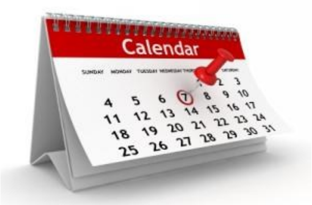 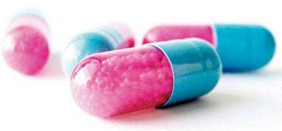

If you take Medicine A, you would live for **2 months less than 5 years** and then die.

If you take Medicine B, you would live for **5 years** and then die.

Would you prefer Medicine A or Medicine B, or are they the same?

Indicate your choice here. You can only choose one option:

◯ **Medicine A** 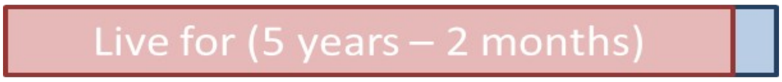

◯ **Medicine B** 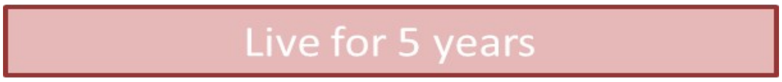

◯ **The Same - Medicine A and Medicine B are the same.**

0% 100%

Repeated section

Remember Medicine A and Medicine B are equally effective and have some risk of a minor side effect.

The only difference between Medicine A and Medicine B is how you take them.

Medicine A	Medicine B
You take a tablet only once in your life. You will be given the tablet on the day you come in to see your GP. 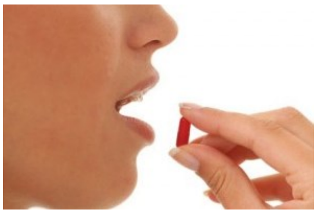	One tablet should be taken once a week on the same day every week.<br>You need to take this medicine for 5 years.<br><br>You need to take the tablet first thing in the morning on an empty stomach with a large glass of tap water.<br>After taking the tablet, you should stay upright for the next 30 minutes.<br>You should not eat anything for at least 30 minutes, including taking any other medicines.<br><br>You need to regularly visit your doctor to have your bone health checked.<br>You need to regularly visit a pharmacy for a new supply.<br>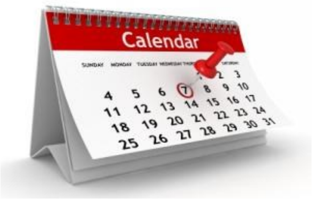 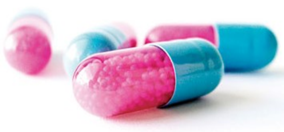

If you take Medicine A, you would live for **1 month less than 5 years** and then die.

If you take Medicine B, you would live for **5 years** and then die.

Would you prefer Medicine A or Medicine B, or are they the same?

Indicate your choice here. You can only choose one option:

○ **Medicine A** — Live for (5 years – 1 month)

○ **Medicine B** — Live for 5 years

○ **The Same - Medicine A and Medicine B are the same.**

0% 100%

Repeated section

Remember Medicine A and Medicine B are equally effective and have some risk of a minor side effect.

The only difference between Medicine A and Medicine B is how you take them.

Medicine A	Medicine B
You take a tablet only once in your life.<br>You will be given the tablet on the day you come in to see your GP.<br>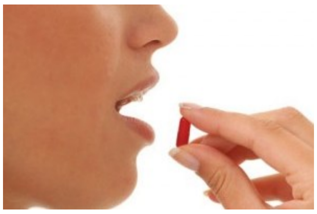	One tablet should be taken once a week on the same day every week.<br>You need to take this medicine for 5 years.<br><br>You need to take the tablet first thing in the morning on an empty stomach with a large glass of tap water.<br>After taking the tablet, you should stay upright for the next 30 minutes.<br>You should not eat anything for at least 30 minutes, including taking any other medicines.<br><br>You need to regularly visit your doctor to have your bone health checked.<br>You need to regularly visit a pharmacy for a new supply.<br>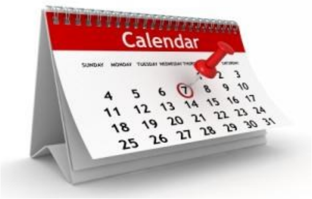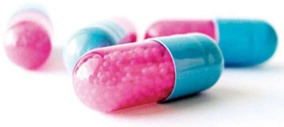

If you take Medicine A, you would live for **2 weeks less than 5 years** and then die.

If you take Medicine B, you would live for **5 years** and then die.

Would you prefer Medicine A or Medicine B, or are they the same?

Indicate your choice here. You can only choose one option:

○ **Medicine A** Live for (5 years – 2 weeks)

○ **Medicine B** Live for 5 years

○ **The Same - Medicine A and Medicine B are the same.**

0% 100%

Repeated section

Remember Medicine A and Medicine B are equally effective and have some risk of a minor side effect.

The only difference between Medicine A and Medicine B is how you take them.

Medicine A	Medicine B
You take a tablet only once in your life.<br>You will be given the tablet on the day you come in to see your GP.<br>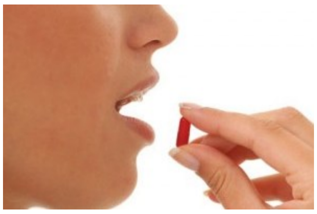	One tablet should be taken once a week on the same day every week.<br>You need to take this medicine for 5 years.<br><br>You need to take the tablet first thing in the morning on an empty stomach with a large glass of tap water.<br>After taking the tablet, you should stay upright for the next 30 minutes.<br>You should not eat anything for at least 30 minutes, including taking any other medicines.<br><br>You need to regularly visit your doctor to have your bone health checked.<br>You need to regularly visit a pharmacy for a new supply.<br>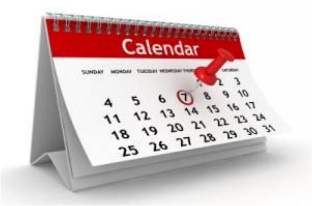 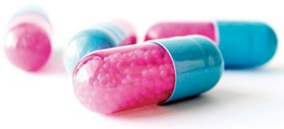

If you take Medicine A, you would live for **1 week less than 5 years** and then die.

If you take Medicine B, you would live for **5 years** and then die.

Would you prefer Medicine A or Medicine B, or are they the same?

Indicate your choice here. You can only choose one option:

○ **Medicine A** Live for (5 years – 1 week)

○ **Medicine B** Live for 5 years

○ **The Same - Medicine A and Medicine B are the same.**

0% 100%

Repeated section

Remember Medicine A and Medicine B are equally effective and have some risk of a minor side effect.

The only difference between Medicine A and Medicine B is how you take them.

Medicine A	Medicine B
You take a tablet only once in your life. You will be given the tablet on the day you come in to see your GP.<br>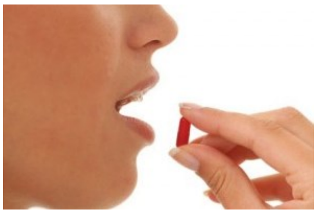	One tablet should be taken once a week on the same day every week.<br>You need to take this medicine for 5 years.<br><br>You need to take the tablet first thing in the morning on an empty stomach with a large glass of tap water.<br>After taking the tablet, you should stay upright for the next 30 minutes.<br>You should not eat anything for at least 30 minutes, including taking any other medicines.<br><br>You need to regularly visit your doctor to have your bone health checked.<br>You need to regularly visit a pharmacy for a new supply.<br>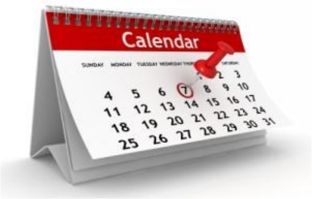 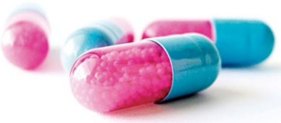

If you take Medicine A, you would live for **5 days less than 5 years** and then die.

If you take Medicine B, you would live for **5 years** and then die.

Would you prefer Medicine A or Medicine B, or are they the same?

Indicate your choice here. You can only choose one option:

◯ **Medicine A** Live for (5 years – 5 days)

◯ **Medicine B** Live for 5 years

◯ **The Same - Medicine A and Medicine B are the same.**

0% 100%

Repeated section

Remember Medicine A and Medicine B are equally effective and have some risk of a minor side effect.

The only difference between Medicine A and Medicine B is how you take them.

Medicine A	Medicine B
You take a tablet only once in your life.<br>You will be given the tablet on the day you come in to see your GP.<br>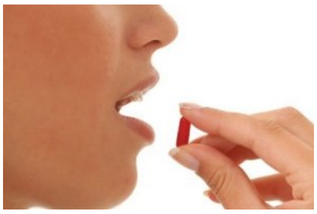	One tablet should be taken once a week on the same day every week.<br>You need to take this medicine for 5 years.<br><br>You need to take the tablet first thing in the morning on an empty stomach with a large glass of tap water.<br>After taking the tablet, you should stay upright for the next 30 minutes.<br>You should not eat anything for at least 30 minutes, including taking any other medicines.<br><br>You need to regularly visit your doctor to have your bone health checked.<br>You need to regularly visit a pharmacy for a new supply.<br>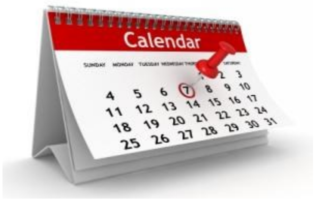 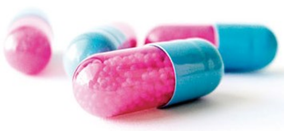

If you take Medicine A, you would live for **3 days less than 5 years** and then die.

If you take Medicine B, you would live for **5 years** and then die.

Would you prefer Medicine A or Medicine B, or are they the same?

Indicate your choice here. You can only choose one option:

○ **Medicine A** Live for (5 years – 3 days)

○ **Medicine B** Live for 5 years

○ **The Same - Medicine A and Medicine B are the same.**

0% 100%

Repeated section

Remember Medicine A and Medicine B are equally effective and have some risk of a minor side effect.

The only difference between Medicine A and Medicine B is how you take them.

Medicine A	Medicine B
You take a tablet only once in your life.<br>You will be given the tablet on the day you come in to see your GP.<br>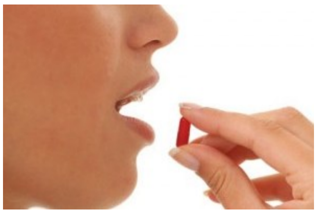	One tablet should be taken once a week on the same day every week.<br>You need to take this medicine for 5 years.<br><br>You need to take the tablet first thing in the morning on an empty stomach with a large glass of tap water.<br>After taking the tablet, you should stay upright for the next 30 minutes.<br>You should not eat anything for at least 30 minutes, including taking any other medicines.<br><br>You need to regularly visit your doctor to have your bone health checked.<br>You need to regularly visit a pharmacy for a new supply.<br>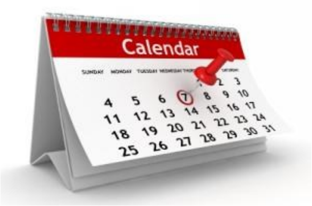 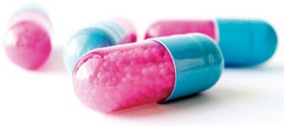

If you take Medicine A, you would live for **1 day less than 5 years** and then die.

If you take Medicine B, you would live for **5 years** and then die.

Would you prefer Medicine A or Medicine B, or are they the same?

Indicate your choice here. You can only choose one option:

○ **Medicine A** Live for (5 years – 1 day)

○ **Medicine B** Live for 5 years

○ **The Same - Medicine A and Medicine B are the same.**

0% 100%

Question 2a.

You prefer living for 2.5 years (2 years and 6 months) on Medicine A, to living for 5 years on Medicine B.

This means **living fewer than 2.5 years on Medicine A** can be the same as living for 5 years on Medicine B.

How many years on Medicine A do you think would be the same as living for 5 years on Medicine B?

(Please give a number in years) [ ]

0% 100%

Part 2

Question 3

You have been told that you need to take a medicine to reduce the risk of fracture.

You can choose between two different medicines. We have called these Medicine A and Medicine B.

Medicine A is a pill that you only need to take once.

Medicine B is a pill that you need to take every week for 5 years.

Medicine A and Medicine B are equally effective in reducing your risk of having a fracture.
Medicine A and Medicine B will reduce your risk of experiencing a fracture, such as hip fracture, within the next 5 years from **10%** to **6%**.

In other words, in a crowd of **1,000** people in the UK, **100** are likely to have a fracture if they do not take the medicine. This is reduced to **60** people out of 1,000 who do take the medicine.
This information is also illustrated in this picture.

Risk of fracture

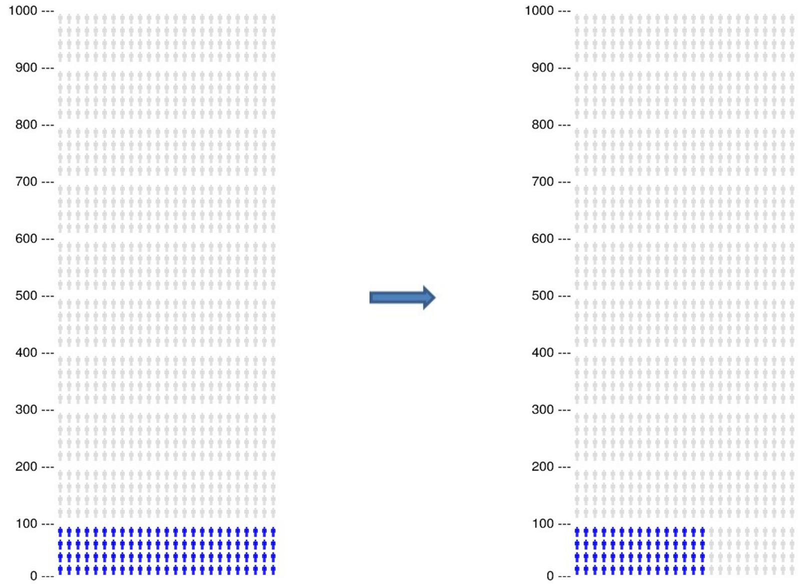

10%
No Treatment

6%
Taking the Medicine

(The figures shaded blue represent people who will have a fracture, such as hip fracture, within the next 5 years)

In this scenario, there is some risk of a severe side effect from Medicine A or Medicine B.

Among every 1,000 people taking Medicine A or Medicine B, **3** people are likely to have a severe side effect such as severe loss or destruction of the bone in the jaw.

Risk of a severe side effect

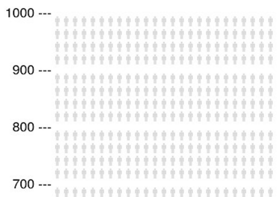

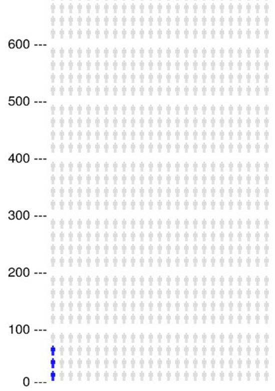

0.3% of people will have a severe side effect.

0% 100%

Repeated section

Remember Medicine A and Medicine B are equally effective and have some risk of a severe side effect.

The only difference between Medicine A and Medicine B is how you take them.

Medicine A	Medicine B
You take a tablet only once in your life. You will be given the tablet on the day you come in to see your GP. 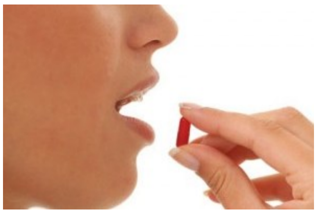	One tablet should be taken once a week on the same day every week. You need to take this medicine for 5 years.<br><br>You need to take the tablet first thing in the morning on an empty stomach with a large glass of tap water. After taking the tablet, you should stay upright for the next 30 minutes. You should not eat anything for at least 30 minutes, including taking any other medicines.<br><br>You need to regularly visit your doctor to have your bone health checked. You need to regularly visit a pharmacy for a new supply. 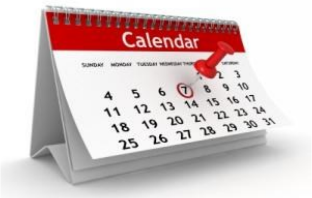 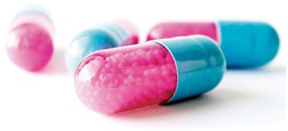

If you take Medicine A, you would live for **2.5 years (2 years and 6 months)** and then die.

If you take Medicine B, you would live for **5 years** and then die.

Would you prefer Medicine A or Medicine B, or are they the same?

Indicate your choice here. You can only choose one option:

○ **Medicine A** 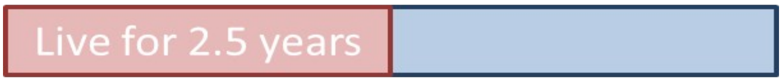

○ **Medicine B** 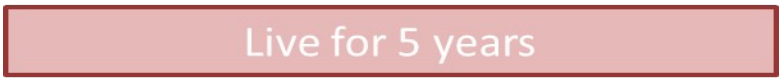

○ **The Same - Medicine A and Medicine B are the same.**

0% 100%

Repeated section

Remember Medicine A and Medicine B are equally effective and have some risk of a severe side effect.

The only difference between Medicine A and Medicine B is how you take them.

Medicine A	Medicine B
You take a tablet only once in your life. You will be given the tablet on the day you come in to see your GP.<br>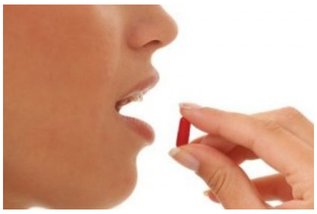	One tablet should be taken once a week on the same day every week.<br>You need to take this medicine for 5 years.<br><br>You need to take the tablet first thing in the morning on an empty stomach with a large glass of tap water.<br>After taking the tablet, you should stay upright for the next 30 minutes.<br>You should not eat anything for at least 30 minutes, including taking any other medicines.<br><br>You need to regularly visit your doctor to have your bone health checked.<br>You need to regularly visit a pharmacy for a new supply.<br>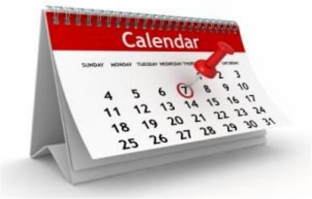 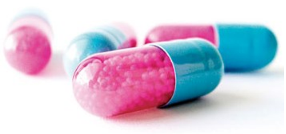

If you take Medicine A, you would live for **3 years** and then die.

If you take Medicine B, you would live for **5 years** and then die.

Would you prefer Medicine A or Medicine B, or are they the same?

Indicate your choice here. You can only choose one option:

◯ **Medicine A** Live for 3 years

◯ **Medicine B** Live for 5 years

◯ **The Same - Medicine A and Medicine B are the same.**

0% 100%

Repeated section

Remember Medicine A and Medicine B are equally effective and have some risk of a severe side effect.

The only difference between Medicine A and Medicine B is how you take them.

Medicine A	Medicine B
You take a tablet only once in your life.<br>You will be given the tablet on the day you come in to see your GP.<br>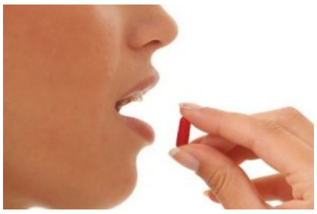	One tablet should be taken once a week on the same day every week.<br>You need to take this medicine for 5 years.<br><br>You need to take the tablet first thing in the morning on an empty stomach with a large glass of tap water.<br>After taking the tablet, you should stay upright for the next 30 minutes.<br>You should not eat anything for at least 30 minutes, including taking any other medicines.<br><br>You need to regularly visit your doctor to have your bone health checked.<br>You need to regularly visit a pharmacy for a new supply.<br>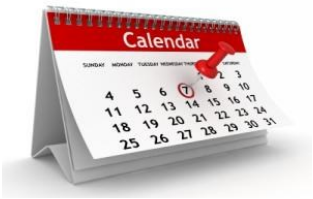 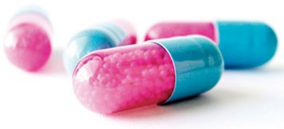

If you take Medicine A, you would live for **3.25 years** (3 years and 3 months) and then die.

If you take Medicine B, you would live for **5 years** and then die.

Would you prefer Medicine A or Medicine B, or are they the same?

Indicate your choice here. You can only choose one option:

◯ **Medicine A** Live for 3 years 3 months

◯ **Medicine B** Live for 5 years

◯ **The Same - Medicine A and Medicine B are the same.**

0% 100%

Repeated section

Remember Medicine A and Medicine B are equally effective and have some risk of a severe side effect.

The only difference between Medicine A and Medicine B is how you take them.

Medicine A	Medicine B
You take a tablet only once in your life.<br>You will be given the tablet on the day you come in to see your GP.<br>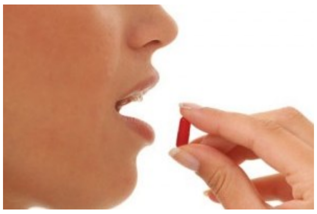	One tablet should be taken once a week on the same day every week.<br>You need to take this medicine for 5 years.<br><br>You need to take the tablet first thing in the morning on an empty stomach with a large glass of tap water.<br>After taking the tablet, you should stay upright for the next 30 minutes.<br>You should not eat anything for at least 30 minutes, including taking any other medicines.<br><br>You need to regularly visit your doctor to have your bone health checked.<br>You need to regularly visit a pharmacy for a new supply.<br>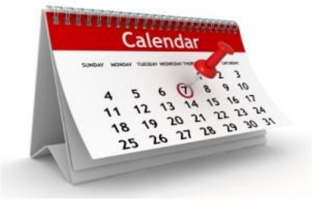 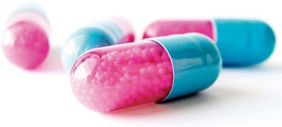

If you take Medicine A, you would live for **3.5 years** (3 years and 6 months) and then die.

If you take Medicine B, you would live for **5 years** and then die.

Would you prefer Medicine A or Medicine B, or are they the same?

Indicate your choice here. You can only choose one option:

◯ **Medicine A**

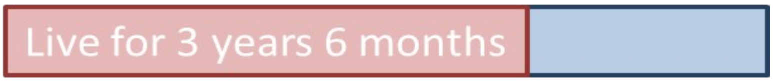

◯ **Medicine B**

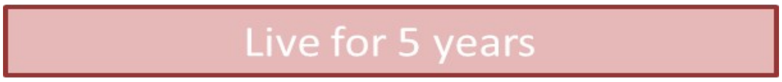

◯ **The Same - Medicine A and Medicine B are the same.**

0% 100%

Repeated section

Remember Medicine A and Medicine B are equally effective and have no risks of a side effect.

The only difference between Medicine A and Medicine B is how you take them.

Medicine A	Medicine B
You take a tablet only once in your life. You will be given the tablet on the day you come in to see your GP. 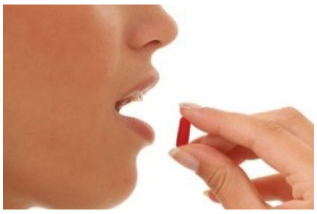	One tablet should be taken once a week on the same day every week. You need to take this medicine for 5 years.<br><br>You need to take the tablet first thing in the morning on an empty stomach with a large glass of tap water. After taking the tablet, you should stay upright for the next 30 minutes. You should not eat anything for at least 30 minutes, including taking any other medicines.<br><br>You need to regularly visit your doctor to have your bone health checked. You need to regularly visit a pharmacy for a new supply. 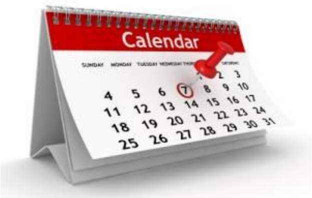 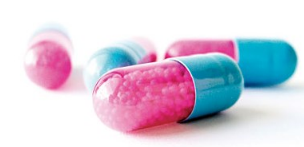

If you take Medicine A, you would live for **3.75 years** (3 years and 9 months) and then die.

If you take Medicine B, you would live for **5 years** and then die.

Would you prefer Medicine A or Medicine B, or are they the same?

Indicate your choice here. You can only choose one option:

◯ **Medicine A** 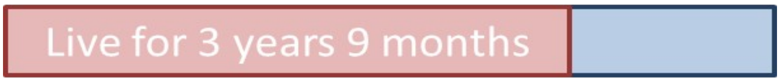

◯ **Medicine B** 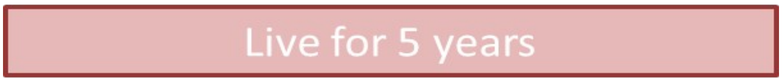

◯ **The Same - Medicine A and Medicine B are the same.**

0% 100%

Repeated section

Remember Medicine A and Medicine B are equally effective and have some risk of a severe side effect.

The only difference between Medicine A and Medicine B is how you take them.

Medicine A	Medicine B
You take a tablet only once in your life.<br>You will be given the tablet on the day you come in to see your GP.<br>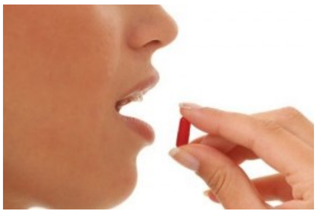	One tablet should be taken once a week on the same day every week.<br>You need to take this medicine for 5 years.<br><br>You need to take the tablet first thing in the morning on an empty stomach with a large glass of tap water.<br>After taking the tablet, you should stay upright for the next 30 minutes.<br>You should not eat anything for at least 30 minutes, including taking any other medicines.<br><br>You need to regularly visit your doctor to have your bone health checked.<br>You need to regularly visit a pharmacy for a new supply.<br>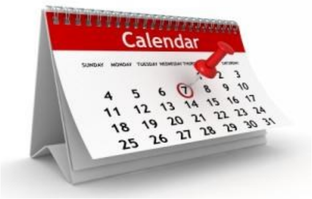 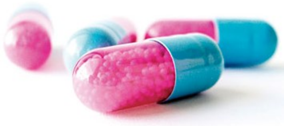

If you take Medicine A, you would live for **3 years and 10 months** and then die.

If you take Medicine B, you would live for **5 years** and then die.

Would you prefer Medicine A or Medicine B, or are they the same?

Indicate your choice here. You can only choose one option:

○ **Medicine A** 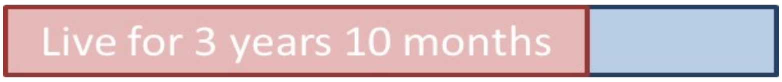

○ **Medicine B** 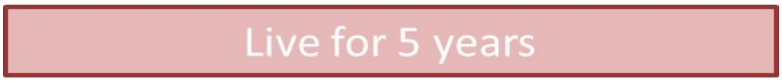

○ **The Same - Medicine A and Medicine B are the same.**

0% 100%

Repeated section

Remember Medicine A and Medicine B are equally effective and have some risk of a severe side effect.

The only difference between Medicine A and Medicine B is how you take them.

Medicine A	Medicine B
You take a tablet only once in your life. You will be given the tablet on the day you come in to see your GP.<br>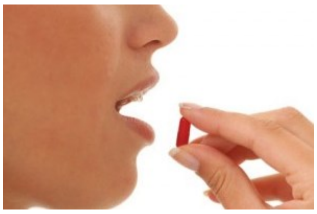	One tablet should be taken once a week on the same day every week.<br>You need to take this medicine for 5 years.<br><br>You need to take the tablet first thing in the morning on an empty stomach with a large glass of tap water.<br>After taking the tablet, you should stay upright for the next 30 minutes.<br>You should not eat anything for at least 30 minutes, including taking any other medicines.<br><br>You need to regularly visit your doctor to have your bone health checked.<br>You need to regularly visit a pharmacy for a new supply.<br>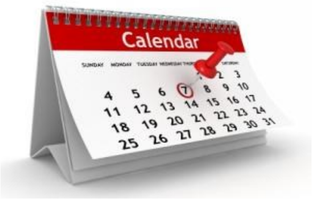 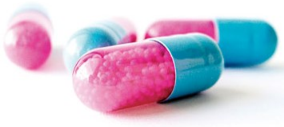

If you take Medicine A, you would live for **4 years** and then die.

If you take Medicine B, you would live for **5 years** and then die.

Would you prefer Medicine A or Medicine B, or are they the same?

Indicate your choice here. You can only choose one option:

○ **Medicine A**

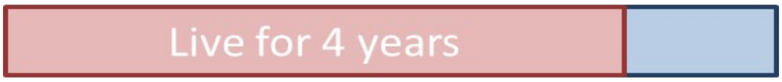

○ **Medicine B**

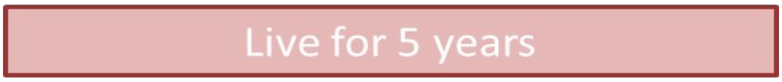

○ **The Same - Medicine A and Medicine B are the same.**

0% 100%

Repeated section

Remember Medicine A and Medicine B are equally effective and have some risk of a severe side effect.

The only difference between Medicine A and Medicine B is how you take them.

Medicine A	Medicine B
You take a tablet only once in your life. You will be given the tablet on the day you come in to see your GP.<br>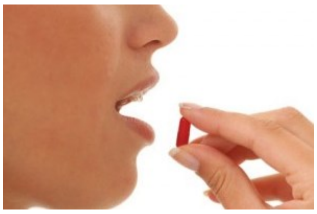	One tablet should be taken once a week on the same day every week.<br>You need to take this medicine for 5 years.<br><br>You need to take the tablet first thing in the morning on an empty stomach with a large glass of tap water.<br>After taking the tablet, you should stay upright for the next 30 minutes.<br>You should not eat anything for at least 30 minutes, including taking any other medicines.<br><br>You need to regularly visit your doctor to have your bone health checked.<br>You need to regularly visit a pharmacy for a new supply.<br>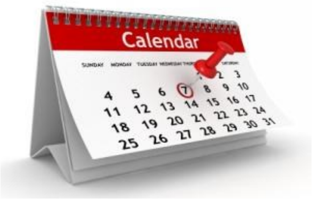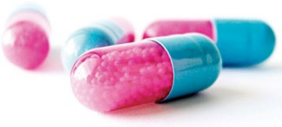

If you take Medicine A, you would live for **4 years and 2 months** and then die.

If you take Medicine B, you would live for **5 years** and then die.

Would you prefer Medicine A or Medicine B, or are they the same?

Indicate your choice here. You can only choose one option:

◯ **Medicine A** Live for 4 years 2 months

◯ **Medicine B** Live for 5 years

◯ **The Same - Medicine A and Medicine B are the same.**

0% 100%

Repeated section

Remember Medicine A and Medicine B are equally effective and have some risk of a severe side effect.

The only difference between Medicine A and Medicine B is how you take them.

Medicine A	Medicine B
You take a tablet only once in your life.<br>You will be given the tablet on the day you come in to see your GP.<br>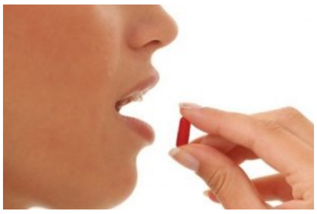	One tablet should be taken once a week on the same day every week.<br>You need to take this medicine for 5 years.<br><br>You need to take the tablet first thing in the morning on an empty stomach with a large glass of tap water.<br>After taking the tablet, you should stay upright for the next 30 minutes.<br>You should not eat anything for at least 30 minutes, including taking any other medicines.<br><br>You need to regularly visit your doctor to have your bone health checked.<br>You need to regularly visit a pharmacy for a new supply.<br>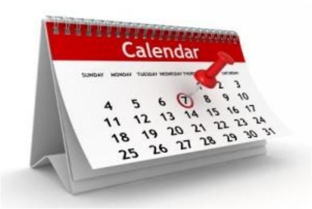 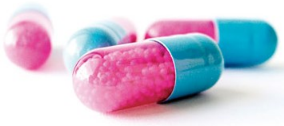

If you take Medicine A, you would live for **4.25 years** (4 years and 3 months) and then die.

If you take Medicine B, you would live for **5 years** and then die.

Would you prefer Medicine A or Medicine B, or are they the same?

Indicate your choice here. You can only choose one option:

○ **Medicine A**

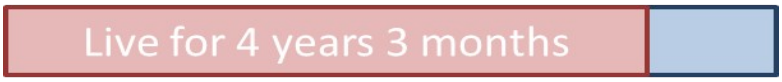

○ **Medicine B**

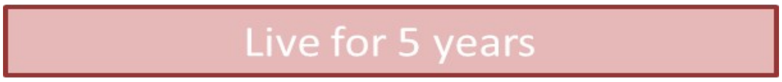

○ **The Same - Medicine A and Medicine B are the same.**

0% 100%

Repeated section

Remember Medicine A and Medicine B are equally effective and have some risk of a severe side effect.

The only difference between Medicine A and Medicine B is how you take them.

Medicine A	Medicine B
You take a tablet only once in your life. You will be given the tablet on the day you come in to see your GP.<br>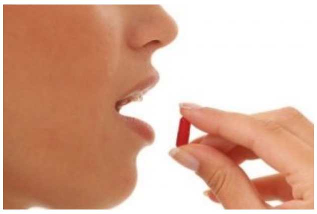	One tablet should be taken once a week on the same day every week.<br>You need to take this medicine for 5 years.<br><br>You need to take the tablet first thing in the morning on an empty stomach with a large glass of tap water.<br>After taking the tablet, you should stay upright for the next 30 minutes.<br>You should not eat anything for at least 30 minutes, including taking any other medicines.<br><br>You need to regularly visit your doctor to have your bone health checked.<br>You need to regularly visit a pharmacy for a new supply.<br>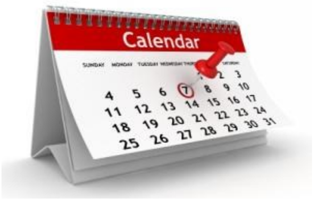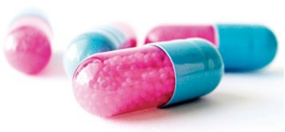

If you take Medicine A, you would live for **4 years 4 months** and then die.

If you take Medicine B, you would live for **5 years** and then die.

Would you prefer Medicine A or Medicine B, or are they the same?

Indicate your choice here. You can only choose one option:

○ **Medicine A** Live for 4 years 4 months

○ **Medicine B** Live for 5 years

○ **The Same - Medicine A and Medicine B are the same.**

Repeated section

Remember Medicine A and Medicine B are equally effective and have some risk of a severe side effect.

The only difference between Medicine A and Medicine B is how you take them.

Medicine A	Medicine B
You take a tablet only once in your life. You will be given the tablet on the day you come in to see your GP.<br>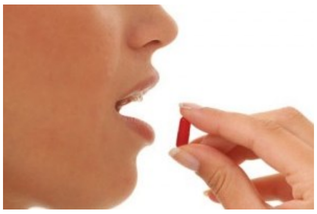	One tablet should be taken once a week on the same day every week.<br>You need to take this medicine for 5 years.<br><br>You need to take the tablet first thing in the morning on an empty stomach with a large glass of tap water.<br>After taking the tablet, you should stay upright for the next 30 minutes.<br>You should not eat anything for at least 30 minutes, including taking any other medicines.<br><br>You need to regularly visit your doctor to have your bone health checked.<br>You need to regularly visit a pharmacy for a new supply.<br>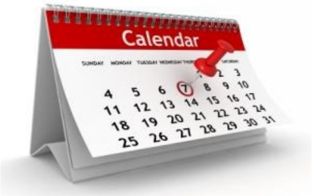 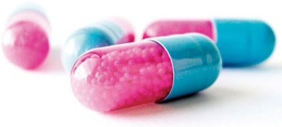

If you take Medicine A, you would live for **4.5 years** (4 years and 6 months) and then die.

If you take Medicine B, you would live for **5 years** and then die.

Would you prefer Medicine A or Medicine B, or are they the same?

Indicate your choice here. You can only choose one option:

◯ **Medicine A** Live for 4 years 6 months

◯ **Medicine B** Live for 5 years

◯ **The Same - Medicine A and Medicine B are the same.**

0% 100%

Repeated section

Remember Medicine A and Medicine B are equally effective and have some risk of a severe side effect.

The only difference between Medicine A and Medicine B is how you take them.

Medicine A	Medicine B
You take a tablet only once in your life. You will be given the tablet on the day you come in to see your GP. 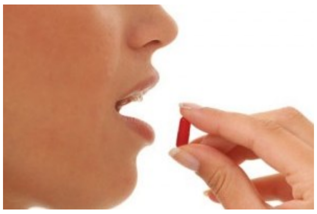	One tablet should be taken once a week on the same day every week.<br>You need to take this medicine for 5 years.<br><br>You need to take the tablet first thing in the morning on an empty stomach with a large glass of tap water.<br>After taking the tablet, you should stay upright for the next 30 minutes.<br>You should not eat anything for at least 30 minutes, including taking any other medicines.<br><br>You need to regularly visit your doctor to have your bone health checked.<br>You need to regularly visit a pharmacy for a new supply.<br>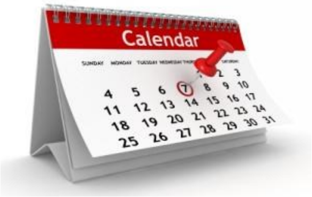 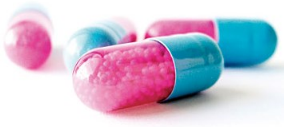

If you take Medicine A, you would live for **4 years and 7 months** and then die.

If you take Medicine B, you would live for **5 years** and then die.

Would you prefer Medicine A or Medicine B, or are they the same?

Indicate your choice here. You can only choose one option:

◯ **Medicine A** Live for 4 years 7 months

◯ **Medicine B** Live for 5 years

◯ **The Same - Medicine A and Medicine B are the same.**

0% 100%

Repeated section

Remember Medicine A and Medicine B are equally effective and have some risk of a severe side effect.

The only difference between Medicine A and Medicine B is how you take them.

Medicine A	Medicine B
You take a tablet only once in your life. You will be given the tablet on the day you come in to see your GP.<br>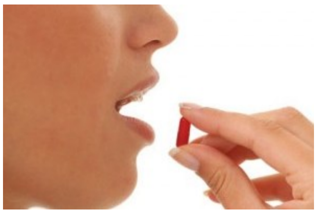	One tablet should be taken once a week on the same day every week.<br>You need to take this medicine for 5 years.<br><br>You need to take the tablet first thing in the morning on an empty stomach with a large glass of tap water.<br>After taking the tablet, you should stay upright for the next 30 minutes.<br>You should not eat anything for at least 30 minutes, including taking any other medicines.<br><br>You need to regularly visit your doctor to have your bone health checked.<br>You need to regularly visit a pharmacy for a new supply.<br>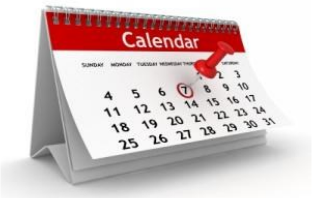 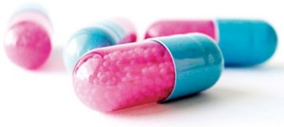

If you take Medicine A, you would live for **4 years and 9 months** and then die.

If you take Medicine B, you would live for **5 years** and then die.

Would you prefer Medicine A or Medicine B, or are they the same?

Indicate your choice here. You can only choose one option:

◯ **Medicine A** 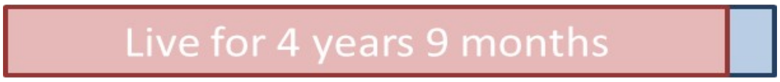

◯ **Medicine B** 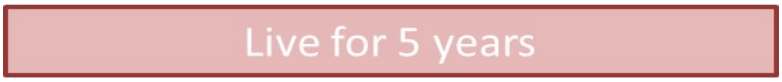

◯ **The Same - Medicine A and Medicine B are the same.**

0% 100%

Repeated section

Remember Medicine A and Medicine B are equally effective and have some risk of a severe side effect.

The only difference between Medicine A and Medicine B is how you take them.

Medicine A	Medicine B
You take a tablet only once in your life. You will be given the tablet on the day you come in to see your GP. 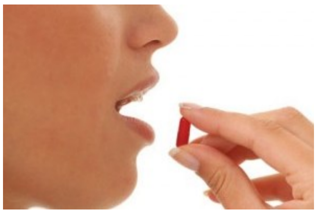	One tablet should be taken once a week on the same day every week. You need to take this medicine for 5 years.<br><br>You need to take the tablet first thing in the morning on an empty stomach with a large glass of tap water. After taking the tablet, you should stay upright for the next 30 minutes. You should not eat anything for at least 30 minutes, including taking any other medicines.<br><br>You need to regularly visit your doctor to have your bone health checked. You need to regularly visit a pharmacy for a new supply. 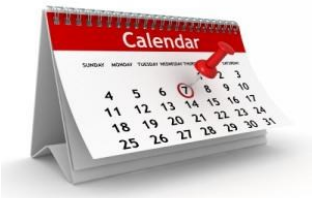 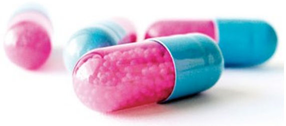

If you take Medicine A, you would live for **2 months less than 5 years** and then die.

If you take Medicine B, you would live for **5 years** and then die.

Would you prefer Medicine A or Medicine B, or are they the same?

Indicate your choice here. You can only choose one option:

◯ **Medicine A** Live for (5 years – 2 months)

◯ **Medicine B** Live for 5 years

◯ **The Same - Medicine A and Medicine B are the same.**

0% 100%

Repeated section

Remember Medicine A and Medicine B are equally effective and have some risk of a severe side effect.

The only difference between Medicine A and Medicine B is how you take them.

**Medicine A**	**Medicine B**
You take a tablet only once in your life.<br>You will be given the tablet on the day you come in to see your GP.<br>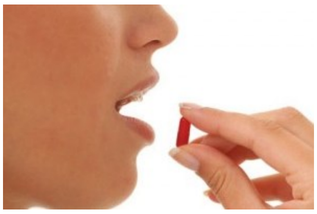	One tablet should be taken once a week on the same day every week.<br>You need to take this medicine for 5 years.<br><br>You need to take the tablet first thing in the morning on an empty stomach with a large glass of tap water.<br>After taking the tablet, you should stay upright for the next 30 minutes.<br>You should not eat anything for at least 30 minutes, including taking any other medicines.<br><br>You need to regularly visit your doctor to have your bone health checked.<br>You need to regularly visit a pharmacy for a new supply.<br>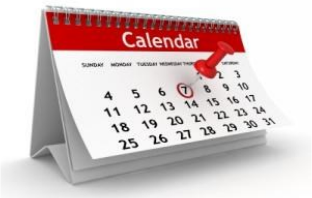 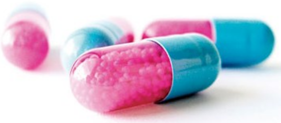

If you take Medicine A, you would live for **1 month less than 5 years** and then die.

If you take Medicine B, you would live for **5 years** and then die.

Would you prefer Medicine A or Medicine B, or are they the same?

Indicate your choice here. You can only choose one option:

○ **Medicine A** Live for (5 years – 1 month)

○ **Medicine B** Live for 5 years

○ **The Same - Medicine A and Medicine B are the same.**

0% 100%

Repeated section

Remember Medicine A and Medicine B are equally effective and have some risk of a severe side effect.

The only difference between Medicine A and Medicine B is how you take them.

Medicine A	Medicine B
You take a tablet only once in your life. You will be given the tablet on the day you come in to see your GP. 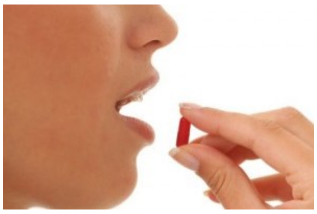	One tablet should be taken once a week on the same day every week.<br>You need to take this medicine for 5 years.<br><br>You need to take the tablet first thing in the morning on an empty stomach with a large glass of tap water.<br>After taking the tablet, you should stay upright for the next 30 minutes.<br>You should not eat anything for at least 30 minutes, including taking any other medicines.<br><br>You need to regularly visit your doctor to have your bone health checked.<br>You need to regularly visit a pharmacy for a new supply.<br>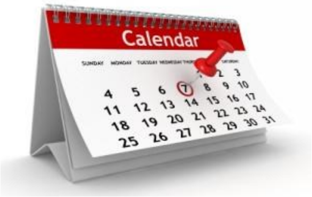 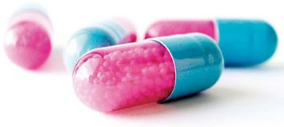

If you take Medicine A, you would live for **2 weeks less than 5 years** and then die.

If you take Medicine B, you would live for **5 years** and then die.

Would you prefer Medicine A or Medicine B, or are they the same?

Indicate your choice here. You can only choose one option:

○ **Medicine A** Live for (5 years – 2 weeks)

○ **Medicine B** Live for 5 years

○ **The Same - Medicine A and Medicine B are the same.**

0% 100%

Repeated section

Remember Medicine A and Medicine B are equally effective and have some risk of a severe side effect.

The only difference between Medicine A and Medicine B is how you take them.

Medicine A	Medicine B
You take a tablet only once in your life.<br>You will be given the tablet on the day you come in to see your GP.<br>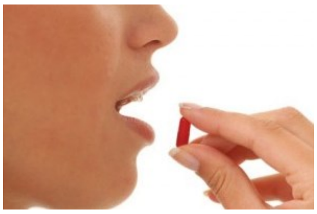	One tablet should be taken once a week on the same day every week.<br>You need to take this medicine for 5 years.<br><br>You need to take the tablet first thing in the morning on an empty stomach with a large glass of tap water.<br>After taking the tablet, you should stay upright for the next 30 minutes.<br>You should not eat anything for at least 30 minutes, including taking any other medicines.<br><br>You need to regularly visit your doctor to have your bone health checked.<br>You need to regularly visit a pharmacy for a new supply.<br>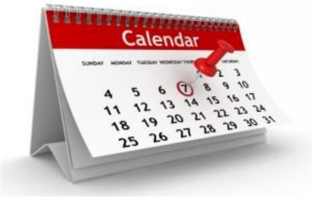 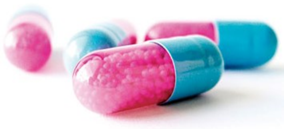

If you take Medicine A, you would live for **1 week less than 5 years** and then die.

If you take Medicine B, you would live for **5 years** and then die.

Would you prefer Medicine A or Medicine B, or are they the same?

Indicate your choice here. You can only choose one option:

◯ **Medicine A** Live for (5 years – 1 week)

◯ **Medicine B** Live for 5 years

◯ **The Same - Medicine A and Medicine B are the same.**

0% 100%

Repeated section

Remember Medicine A and Medicine B are equally effective and have some risk of a severe side effect.

The only difference between Medicine A and Medicine B is how you take them.

Medicine A	Medicine B
You take a tablet only once in your life. You will be given the tablet on the day you come in to see your GP. 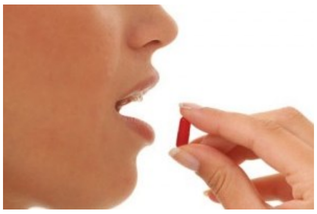	One tablet should be taken once a week on the same day every week.<br>You need to take this medicine for 5 years.<br><br>You need to take the tablet first thing in the morning on an empty stomach with a large glass of tap water.<br>After taking the tablet, you should stay upright for the next 30 minutes.<br>You should not eat anything for at least 30 minutes, including taking any other medicines.<br><br>You need to regularly visit your doctor to have your bone health checked.<br>You need to regularly visit a pharmacy for a new supply.<br>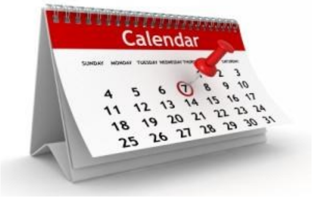 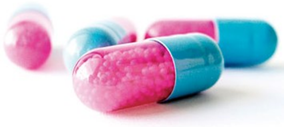

If you take Medicine A, you would live for **5 days less than 5 years** and then die.

If you take Medicine B, you would live for **5 years** and then die.

Would you prefer Medicine A or Medicine B, or are they the same?

Indicate your choice here. You can only choose one option:

○ **Medicine A** Live for (5 years – 5 days)

○ **Medicine B** Live for 5 years

○ **The Same - Medicine A and Medicine B are the same.**

0% 100%

Repeated section

Remember Medicine A and Medicine B are equally effective and have some risk of a severe side effect.

The only difference between Medicine A and Medicine B is how you take them.

Medicine A	Medicine B
You take a tablet only once in your life.<br>You will be given the tablet on the day you come in to see your GP.<br>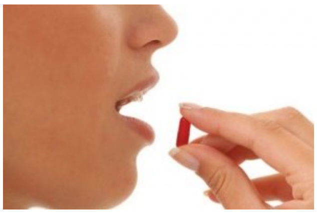	One tablet should be taken once a week on the same day every week.<br>You need to take this medicine for 5 years.<br><br>You need to take the tablet first thing in the morning on an empty stomach with a large glass of tap water.<br>After taking the tablet, you should stay upright for the next 30 minutes.<br>You should not eat anything for at least 30 minutes, including taking any other medicines.<br><br>You need to regularly visit your doctor to have your bone health checked.<br>You need to regularly visit a pharmacy for a new supply.<br>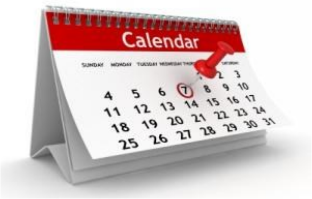 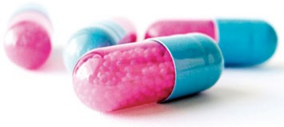

If you take Medicine A, you would live for **3 days less than 5 years** and then die.

If you take Medicine B, you would live for **5 years** and then die.

Would you prefer Medicine A or Medicine B, or are they the same?

Indicate your choice here. You can only choose one option:

◯ **Medicine A** Live for (5 years – 3 days)

◯ **Medicine B** Live for 5 years

◯ **The Same - Medicine A and Medicine B are the same.**

0% 100%

Repeated section

Remember Medicine A and Medicine B are equally effective and have some risk of a severe side effect.

The only difference between Medicine A and Medicine B is how you take them.

Medicine A	Medicine B
You take a tablet only once in your life. You will be given the tablet on the day you come in to see your GP.<br>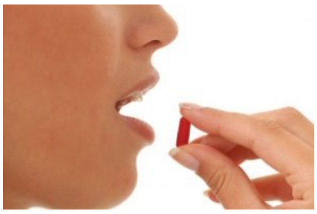	One tablet should be taken once a week on the same day every week.<br>You need to take this medicine for 5 years.<br><br>You need to take the tablet first thing in the morning on an empty stomach with a large glass of tap water.<br>After taking the tablet, you should stay upright for the next 30 minutes.<br>You should not eat anything for at least 30 minutes, including taking any other medicines.<br><br>You need to regularly visit your doctor to have your bone health checked.<br>You need to regularly visit a pharmacy for a new supply.<br>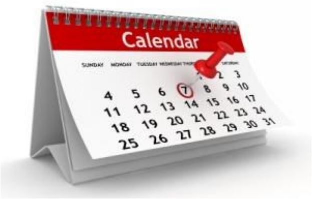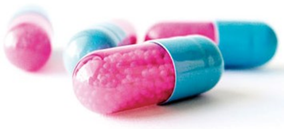

If you take Medicine A, you would live for **1 day less than 5 years** and then die.

If you take Medicine B, you would live for **5 years** and then die.

Would you prefer Medicine A or Medicine B, or are they the same?

Indicate your choice here. You can only choose one option:

○ **Medicine A** — Live for (5 years – 1 day)

○ **Medicine B** — Live for 5 years

○ **The Same - Medicine A and Medicine B are the same.**

0% 100%

Question 3a.

You prefer living for 2.5 years (2 years and 6 months) on Medicine A, to living for 5 years on Medicine B.

This means **living fewer than 2.5 years on Medicine A** can be the same as living for 5 years on Medicine B.

How many years on Medicine A do you think would be the same as living for 5 years on Medicine B?

(Please give a number in years)

0% 100%

Question 4

Now, Medicine A and Medicine B will give you less benefit than before because you belong to a group of people who may not respond to the treatment as well as other groups of people.

As before,

Medicine A is a pill that you only need to take once.

Medicine B is a pill that you need to take every week for 5 years.

Medicine A and Medicine B are equally effective in reducing your risk of having a fracture.

Medicine A and Medicine B will reduce your risk of experiencing a fracture, such as hip fracture, within the next 5 years from **10%** to **9%**.

In other words, in a crowd of **1,000** people in the UK, **100** are likely to have a fracture if they do not take the medicine. This is reduced to **90** people out of 1,000 who do take the medicine. This information is also illustrated in this picture.

Risk of fracture

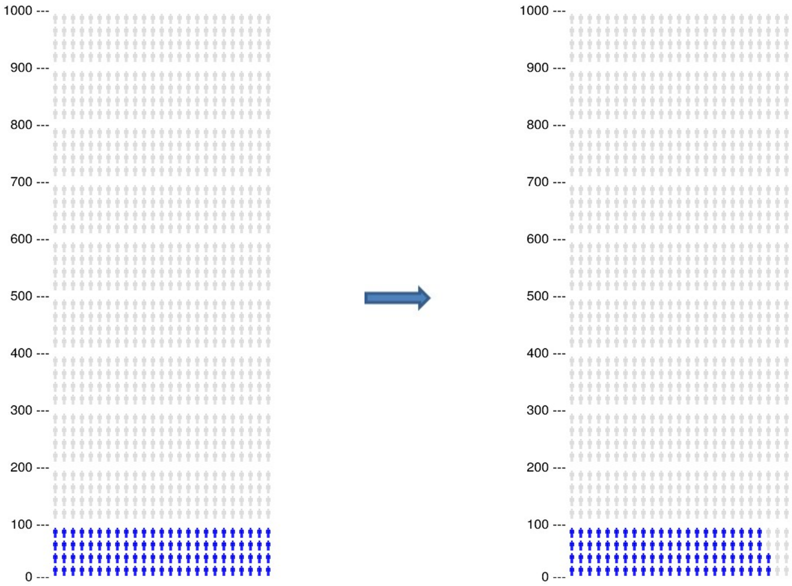

10%
No Treatment

9%
Taking the Medicine

(The figures shaded blue represent people who will have a fracture, such as hip fracture, within the next 5 years)

In this scenario, you will not have any side effects from Medicine A or Medicine B.

Risk of a side effect

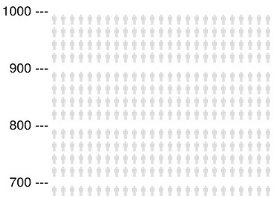

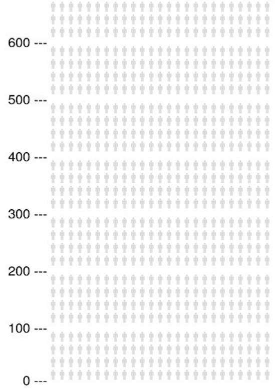

0%

No one will have a side effect.

0% 100%

Repeated section

Remember Medicine A and Medicine B are equally effective and have no risks of a side effect.

The only difference between Medicine A and Medicine B is how you take them.

Medicine A	Medicine B
You take a tablet only once in your life. You will be given the tablet on the day you come in to see your GP. 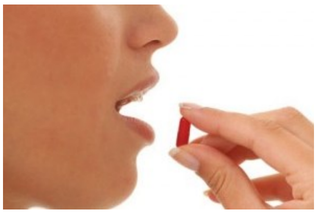	One tablet should be taken once a week on the same day every week.<br>You need to take this medicine for 5 years.<br><br>You need to take the tablet first thing in the morning on an empty stomach with a large glass of tap water.<br>After taking the tablet, you should stay upright for the next 30 minutes.<br>You should not eat anything for at least 30 minutes, including taking any other medicines.<br><br>You need to regularly visit your doctor to have your bone health checked.<br>You need to regularly visit a pharmacy for a new supply.<br>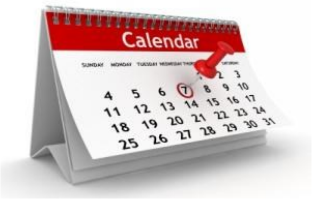 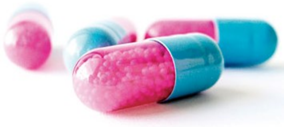

If you take Medicine A, you would live for **2.5 years (2 years and 6 months)** and then die.

If you take Medicine B, you would live for **5 years** and then die.

Would you prefer Medicine A or Medicine B, or are they the same?

Indicate your choice here. You can only choose one option:

◯ **Medicine A** Live for 2.5 years

◯ **Medicine B** Live for 5 years

◯ **The Same - Medicine A and Medicine B are the same.**

0% 100%

Repeated section

Remember Medicine A and Medicine B are equally effective and have no risks of a side effect.

The only difference between Medicine A and Medicine B is how you take them.

Medicine A	Medicine B
You take a tablet only once in your life. You will be given the tablet on the day you come in to see your GP.<br>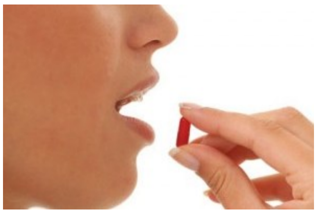	One tablet should be taken once a week on the same day every week.<br>You need to take this medicine for 5 years.<br><br>You need to take the tablet first thing in the morning on an empty stomach with a large glass of tap water.<br>After taking the tablet, you should stay upright for the next 30 minutes.<br>You should not eat anything for at least 30 minutes, including taking any other medicines.<br><br>You need to regularly visit your doctor to have your bone health checked.<br>You need to regularly visit a pharmacy for a new supply.<br>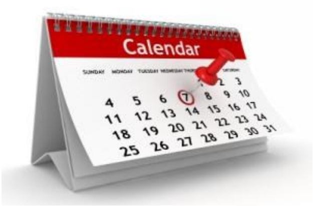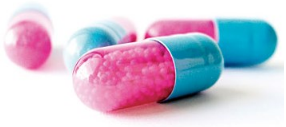

If you take Medicine A, you would live for **3 years** and then die.

If you take Medicine B, you would live for **5 years** and then die.

Would you prefer Medicine A or Medicine B, or are they the same?

Indicate your choice here. You can only choose one option:

◯ **Medicine A**

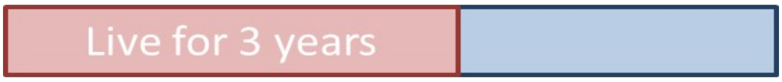

◯ **Medicine B**

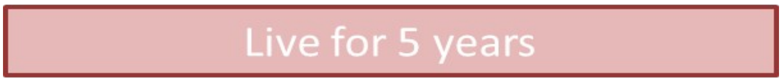

◯ **The Same - Medicine A and Medicine B are the same.**

0% 100%

Repeated section

Remember Medicine A and Medicine B are equally effective and have no risks of a side effect.

The only difference between Medicine A and Medicine B is how you take them.

Medicine A	Medicine B
You take a tablet only once in your life. You will be given the tablet on the day you come in to see your GP.<br>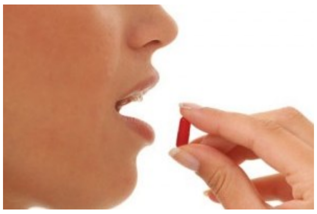	One tablet should be taken once a week on the same day every week.<br>You need to take this medicine for 5 years.<br><br>You need to take the tablet first thing in the morning on an empty stomach with a large glass of tap water.<br>After taking the tablet, you should stay upright for the next 30 minutes.<br>You should not eat anything for at least 30 minutes, including taking any other medicines.<br><br>You need to regularly visit your doctor to have your bone health checked.<br>You need to regularly visit a pharmacy for a new supply.<br>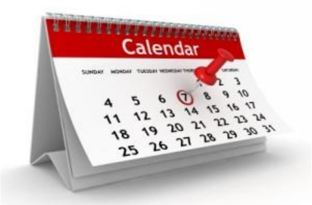 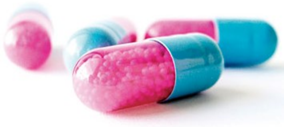

If you take Medicine A, you would live for **3.25 years** (3 years and 3 months) and then die.

If you take Medicine B, you would live for **5 years** and then die.

Would you prefer Medicine A or Medicine B, or are they the same?

Indicate your choice here. You can only choose one option:

◯ **Medicine A**

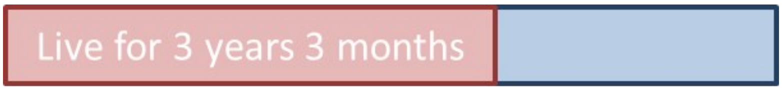

◯ **Medicine B**

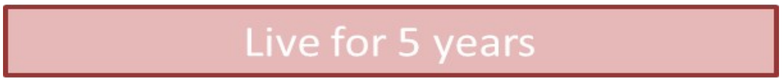

◯ **The Same - Medicine A and Medicine B are the same.**

0% 100%

Repeated section

Remember Medicine A and Medicine B are equally effective and have no risks of a side effect.

The only difference between Medicine A and Medicine B is how you take them.

Medicine A	Medicine B
You take a tablet only once in your life. You will be given the tablet on the day you come in to see your GP.<br>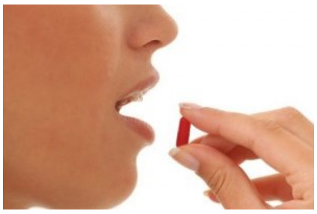	One tablet should be taken once a week on the same day every week.<br>You need to take this medicine for 5 years.<br><br>You need to take the tablet first thing in the morning on an empty stomach with a large glass of tap water.<br>After taking the tablet, you should stay upright for the next 30 minutes.<br>You should not eat anything for at least 30 minutes, including taking any other medicines.<br><br>You need to regularly visit your doctor to have your bone health checked.<br>You need to regularly visit a pharmacy for a new supply.<br>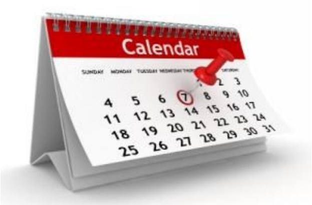 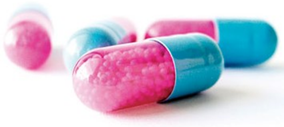

If you take Medicine A, you would live for **3.5 years** (3 years and 6 months) and then die.

If you take Medicine B, you would live for **5 years** and then die.

Would you prefer Medicine A or Medicine B, or are they the same?

Indicate your choice here. You can only choose one option:

◯ **Medicine A**

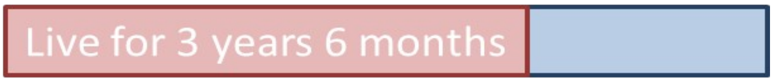

◯ **Medicine B**

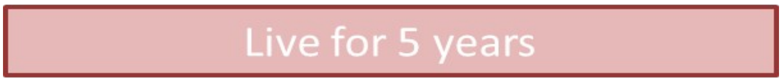

◯ **The Same - Medicine A and Medicine B are the same.**

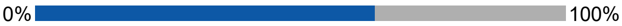

Repeated section

Remember Medicine A and Medicine B are equally effective and have no risks of a side effect.

The only difference between Medicine A and Medicine B is how you take them.

Medicine A	Medicine B
You take a tablet only once in your life.<br>You will be given the tablet on the day you come in to see your GP.<br>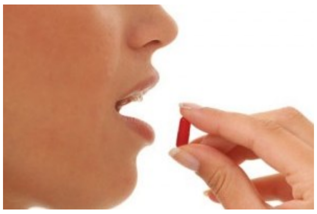	One tablet should be taken once a week on the same day every week.<br>You need to take this medicine for 5 years.<br><br>You need to take the tablet first thing in the morning on an empty stomach with a large glass of tap water.<br>After taking the tablet, you should stay upright for the next 30 minutes.<br>You should not eat anything for at least 30 minutes, including taking any other medicines.<br><br>You need to regularly visit your doctor to have your bone health checked.<br>You need to regularly visit a pharmacy for a new supply.<br>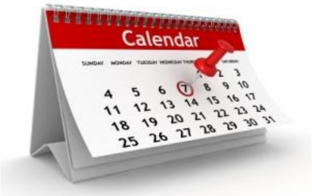 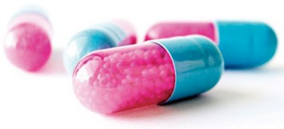

If you take Medicine A, you would live for **3.75 years** (3 years and 9 months) and then die.

If you take Medicine B, you would live for **5 years** and then die.

Would you prefer Medicine A or Medicine B, or are they the same?

Indicate your choice here. You can only choose one option:

◯ **Medicine A** Live for 3 years 9 months

◯ **Medicine B** Live for 5 years

◯ **The Same - Medicine A and Medicine B are the same.**

0% 100%

Repeated section

Remember Medicine A and Medicine B are equally effective and have no risks of a side effect.

The only difference between Medicine A and Medicine B is how you take them.

Medicine A	Medicine B
You take a tablet only once in your life.<br>You will be given the tablet on the day you come in to see your GP.<br>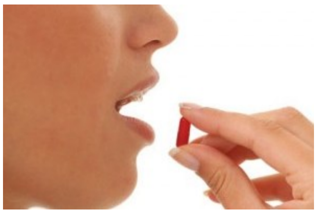	One tablet should be taken once a week on the same day every week.<br>You need to take this medicine for 5 years.<br><br>You need to take the tablet first thing in the morning on an empty stomach with a large glass of tap water.<br>After taking the tablet, you should stay upright for the next 30 minutes.<br>You should not eat anything for at least 30 minutes, including taking any other medicines.<br><br>You need to regularly visit your doctor to have your bone health checked.<br>You need to regularly visit a pharmacy for a new supply.<br>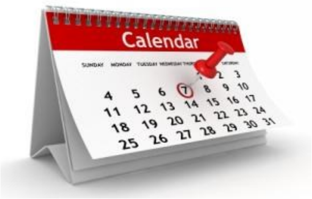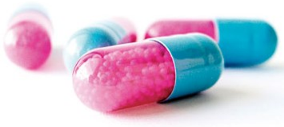

If you take Medicine A, you would live for **3 years and 10 months** and then die.

If you take Medicine B, you would live for **5 years** and then die.

Would you prefer Medicine A or Medicine B, or are they the same?

Indicate your choice here. You can only choose one option:

- ◯ **Medicine A** — Live for 3 years 10 months
- ◯ **Medicine B** — Live for 5 years
- ◯ **The Same - Medicine A and Medicine B are the same.**

0% 100%

Repeated section

Remember Medicine A and Medicine B are equally effective and have no risks of a side effect.

The only difference between Medicine A and Medicine B is how you take them.

Medicine A	Medicine B
You take a tablet only once in your life. You will be given the tablet on the day you come in to see your GP. 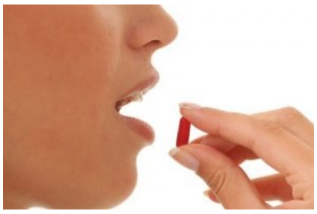	One tablet should be taken once a week on the same day every week.<br>You need to take this medicine for 5 years.<br><br>You need to take the tablet first thing in the morning on an empty stomach with a large glass of tap water.<br>After taking the tablet, you should stay upright for the next 30 minutes.<br>You should not eat anything for at least 30 minutes, including taking any other medicines.<br><br>You need to regularly visit your doctor to have your bone health checked.<br>You need to regularly visit a pharmacy for a new supply.<br>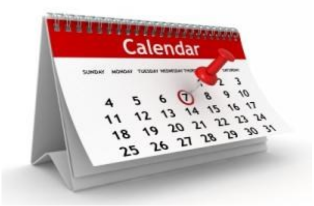 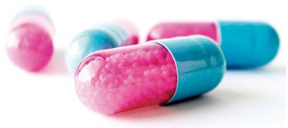

If you take Medicine A, you would live for **4 years** and then die.

If you take Medicine B, you would live for **5 years** and then die.

Would you prefer Medicine A or Medicine B, or are they the same?

Indicate your choice here. You can only choose one option:

◯ **Medicine A** Live for 4 years

◯ **Medicine B** Live for 5 years

◯ **The Same - Medicine A and Medicine B are the same.**

0% 100%

Repeated section

Remember Medicine A and Medicine B are equally effective and have no risks of a side effect.

The only difference between Medicine A and Medicine B is how you take them.

Medicine A	Medicine B
You take a tablet only once in your life.<br>You will be given the tablet on the day you come in to see your GP.<br>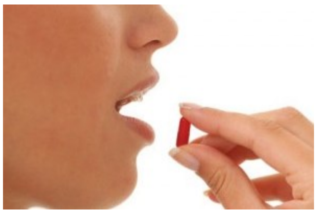	One tablet should be taken once a week on the same day every week.<br>You need to take this medicine for 5 years.<br><br>You need to take the tablet first thing in the morning on an empty stomach with a large glass of tap water.<br>After taking the tablet, you should stay upright for the next 30 minutes.<br>You should not eat anything for at least 30 minutes, including taking any other medicines.<br><br>You need to regularly visit your doctor to have your bone health checked.<br>You need to regularly visit a pharmacy for a new supply.<br>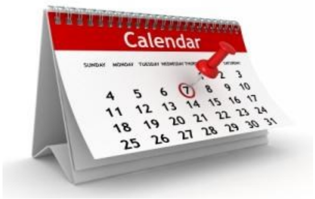 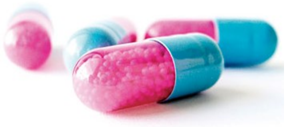

If you take Medicine A, you would live for **4 years and 2 months** and then die.

If you take Medicine B, you would live for **5 years** and then die.

Would you prefer Medicine A or Medicine B, or are they the same?

Indicate your choice here. You can only choose one option:

◯ **Medicine A** Live for 4 years 2 months

◯ **Medicine B** Live for 5 years

◯ **The Same - Medicine A and Medicine B are the same.**

Repeated section

Remember Medicine A and Medicine B are equally effective and have no risks of a side effect.

The only difference between Medicine A and Medicine B is how you take them.

Medicine A	Medicine B
You take a tablet only once in your life.<br>You will be given the tablet on the day you come in to see your GP.<br>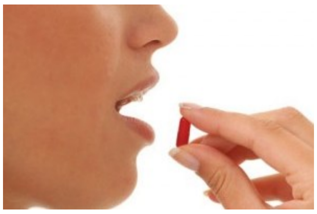	One tablet should be taken once a week on the same day every week.<br>You need to take this medicine for 5 years.<br><br>You need to take the tablet first thing in the morning on an empty stomach with a large glass of tap water.<br>After taking the tablet, you should stay upright for the next 30 minutes.<br>You should not eat anything for at least 30 minutes, including taking any other medicines.<br><br>You need to regularly visit your doctor to have your bone health checked.<br>You need to regularly visit a pharmacy for a new supply.<br>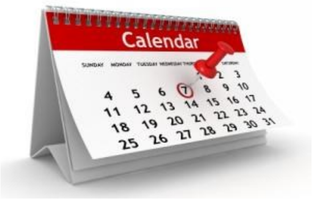 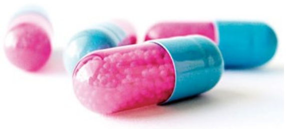

If you take Medicine A, you would live for **4.25 years** (4 years and 3 months) and then die.

If you take Medicine B, you would live for **5 years** and then die.

Would you prefer Medicine A or Medicine B, or are they the same?

Indicate your choice here. You can only choose one option:

○ **Medicine A**

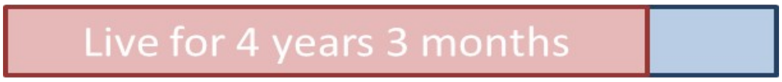

○ **Medicine B**

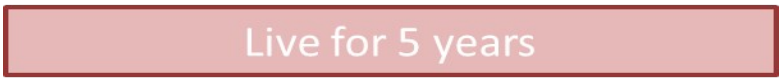

○ **The Same - Medicine A and Medicine B are the same.**

0% 100%

Repeated section

Remember Medicine A and Medicine B are equally effective and have no risks of a side effect.

The only difference between Medicine A and Medicine B is how you take them.

Medicine A	Medicine B
You take a tablet only once in your life.<br>You will be given the tablet on the day you come in to see your GP.<br>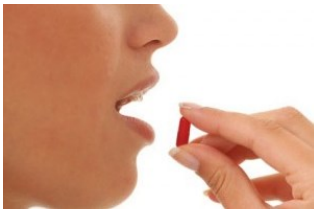	One tablet should be taken once a week on the same day every week.<br>You need to take this medicine for 5 years.<br><br>You need to take the tablet first thing in the morning on an empty stomach with a large glass of tap water.<br>After taking the tablet, you should stay upright for the next 30 minutes.<br>You should not eat anything for at least 30 minutes, including taking any other medicines.<br><br>You need to regularly visit your doctor to have your bone health checked.<br>You need to regularly visit a pharmacy for a new supply.<br>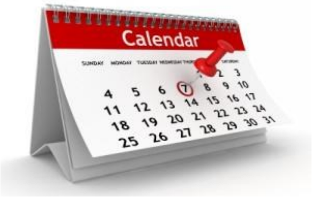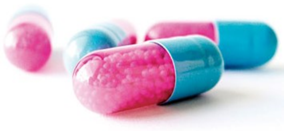

If you take Medicine A, you would live for **4 years 4 months** and then die.

If you take Medicine B, you would live for **5 years** and then die.

Would you prefer Medicine A or Medicine B, or are they the same?

Indicate your choice here. You can only choose one option:

◯ **Medicine A** Live for 4 years 4 months

◯ **Medicine B** Live for 5 years

◯ **The Same - Medicine A and Medicine B are the same.**

0% 100%

Repeated section

Remember Medicine A and Medicine B are equally effective and have no risks of a side effect.

The only difference between Medicine A and Medicine B is how you take them.

<table>
<tr><th>Medicine A</th><th>Medicine B</th></tr>
<tr><td>You take a tablet only once in your life. You will be given the tablet on the day you come in to see your GP.
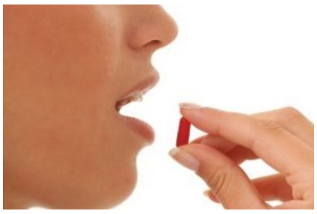</td><td>One tablet should be taken once a week on the same day every week.
You need to take this medicine for 5 years.

You need to take the tablet first thing in the morning on an empty stomach with a large glass of tap water.
After taking the tablet, you should stay upright for the next 30 minutes.
You should not eat anything for at least 30 minutes, including taking any other medicines.

You need to regularly visit your doctor to have your bone health checked.
You need to regularly visit a pharmacy for a new supply.
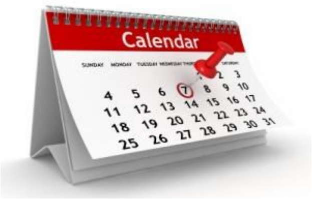

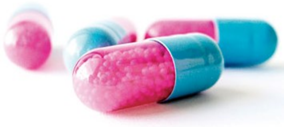</td></tr>
</table>

If you take Medicine A, you would live for **4.5 years** (4 years and 6 months) and then die.

If you take Medicine B, you would live for **5 years** and then die.

Would you prefer Medicine A or Medicine B, or are they the same?

Indicate your choice here. You can only choose one option:

- ◯ **Medicine A** — Live for 4 years 6 months
- ◯ **Medicine B** — Live for 5 years
- ◯ **The Same - Medicine A and Medicine B are the same.**

0% 100%

Repeated section

Remember Medicine A and Medicine B are equally effective and have no risks of a side effect.

The only difference between Medicine A and Medicine B is how you take them.

Medicine A	Medicine B
You take a tablet only once in your life. You will be given the tablet on the day you come in to see your GP. 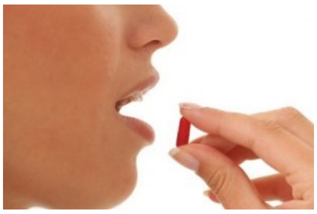	One tablet should be taken once a week on the same day every week. You need to take this medicine for 5 years. You need to take the tablet first thing in the morning on an empty stomach with a large glass of tap water. After taking the tablet, you should stay upright for the next 30 minutes. You should not eat anything for at least 30 minutes, including taking any other medicines. You need to regularly visit your doctor to have your bone health checked. You need to regularly visit a pharmacy for a new supply. 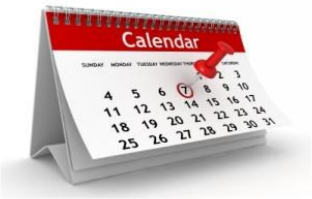 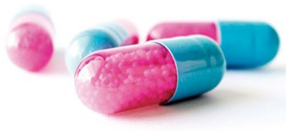

If you take Medicine A, you would live for **4 years and 7 months** and then die.

If you take Medicine B, you would live for **5 years** and then die.

Would you prefer Medicine A or Medicine B, or are they the same?

Indicate your choice here. You can only choose one option:

○ **Medicine A** 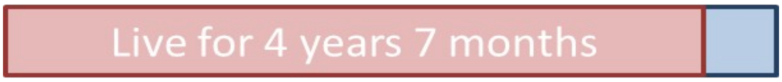

○ **Medicine B** 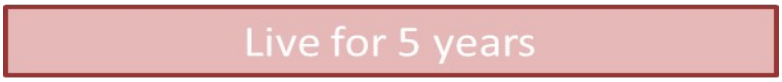

○ **The Same - Medicine A and Medicine B are the same.**

0% 100%

Repeated section

Remember Medicine A and Medicine B are equally effective and have no risks of a side effect.

The only difference between Medicine A and Medicine B is how you take them.

Medicine A	Medicine B
You take a tablet only once in your life. You will be given the tablet on the day you come in to see your GP.<br>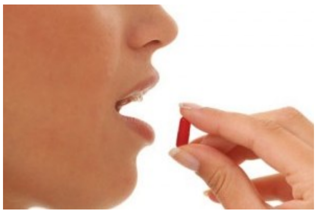	One tablet should be taken once a week on the same day every week.<br>You need to take this medicine for 5 years.<br><br>You need to take the tablet first thing in the morning on an empty stomach with a large glass of tap water.<br>After taking the tablet, you should stay upright for the next 30 minutes.<br>You should not eat anything for at least 30 minutes, including taking any other medicines.<br><br>You need to regularly visit your doctor to have your bone health checked.<br>You need to regularly visit a pharmacy for a new supply.<br>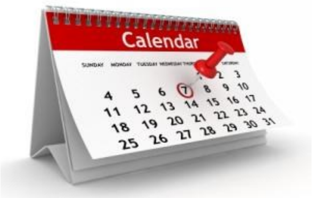 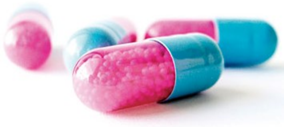

If you take Medicine A, you would live for **4 years and 9 months** and then die.

If you take Medicine B, you would live for **5 years** and then die.

Would you prefer Medicine A or Medicine B, or are they the same?

Indicate your choice here. You can only choose one option:

○ **Medicine A** — Live for 4 years 9 months

○ **Medicine B** — Live for 5 years

○ **The Same - Medicine A and Medicine B are the same.**

0% 100%

Repeated section

Remember Medicine A and Medicine B are equally effective and have no risks of a side effect.

The only difference between Medicine A and Medicine B is how you take them.

Medicine A	Medicine B
You take a tablet only once in your life.<br>You will be given the tablet on the day you come in to see your GP.<br>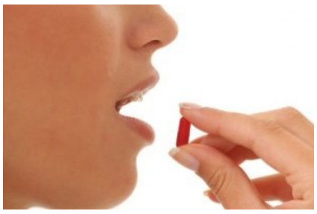	One tablet should be taken once a week on the same day every week.<br>You need to take this medicine for 5 years.<br><br>You need to take the tablet first thing in the morning on an empty stomach with a large glass of tap water.<br>After taking the tablet, you should stay upright for the next 30 minutes.<br>You should not eat anything for at least 30 minutes, including taking any other medicines.<br><br>You need to regularly visit your doctor to have your bone health checked.<br>You need to regularly visit a pharmacy for a new supply.<br>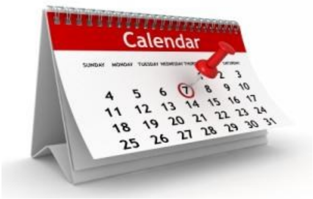 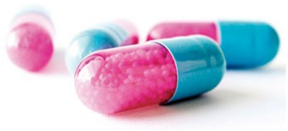

If you take Medicine A, you would live for **2 months less than 5 years** and then die.

If you take Medicine B, you would live for **5 years** and then die.

Would you prefer Medicine A or Medicine B, or are they the same?

Indicate your choice here. You can only choose one option:

◯ **Medicine A** Live for (5 years – 2 months)

◯ **Medicine B** Live for 5 years

◯ **The Same - Medicine A and Medicine B are the same.**

0% 100%

Repeated section

Remember Medicine A and Medicine B are equally effective and have no risks of a side effect.

The only difference between Medicine A and Medicine B is how you take them.

**Medicine A**	**Medicine B**
You take a tablet only once in your life. You will be given the tablet on the day you come in to see your GP. 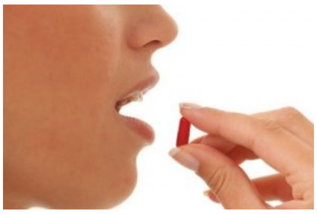	One tablet should be taken once a week on the same day every week. You need to take this medicine for 5 years.<br><br>You need to take the tablet first thing in the morning on an empty stomach with a large glass of tap water. After taking the tablet, you should stay upright for the next 30 minutes. You should not eat anything for at least 30 minutes, including taking any other medicines.<br><br>You need to regularly visit your doctor to have your bone health checked. You need to regularly visit a pharmacy for a new supply. 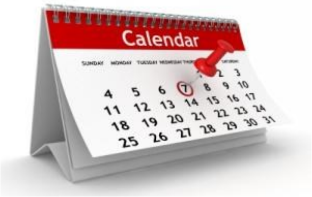 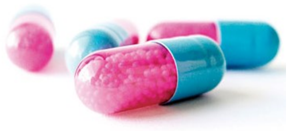

If you take Medicine A, you would live for **1 month less than 5 years** and then die.

If you take Medicine B, you would live for **5 years** and then die.

Would you prefer Medicine A or Medicine B, or are they the same?

Indicate your choice here. You can only choose one option:

◯ **Medicine A** Live for (5 years – 1 month)

◯ **Medicine B** Live for 5 years

◯ **The Same - Medicine A and Medicine B are the same.**

0% 100%

Repeated section

Remember Medicine A and Medicine B are equally effective and have no risks of a side effect.

The only difference between Medicine A and Medicine B is how you take them.

Medicine A	Medicine B
You take a tablet only once in your life. You will be given the tablet on the day you come in to see your GP.<br>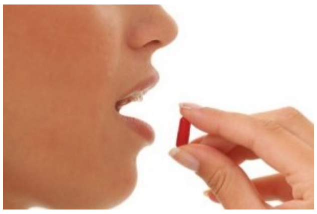	One tablet should be taken once a week on the same day every week.<br>You need to take this medicine for 5 years.<br><br>You need to take the tablet first thing in the morning on an empty stomach with a large glass of tap water.<br>After taking the tablet, you should stay upright for the next 30 minutes.<br>You should not eat anything for at least 30 minutes, including taking any other medicines.<br><br>You need to regularly visit your doctor to have your bone health checked.<br>You need to regularly visit a pharmacy for a new supply.<br>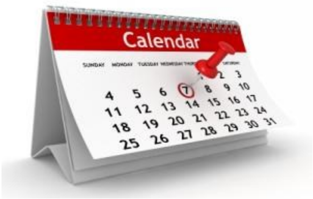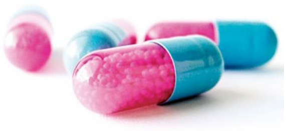

If you take Medicine A, you would live for **2 weeks less than 5 years** and then die.

If you take Medicine B, you would live for **5 years** and then die.

Would you prefer Medicine A or Medicine B, or are they the same?

Indicate your choice here. You can only choose one option:

◯ **Medicine A** Live for (5 years – 2 weeks)

◯ **Medicine B** Live for 5 years

◯ **The Same - Medicine A and Medicine B are the same.**

0% 100%

Repeated section

Remember Medicine A and Medicine B are equally effective and have no risks of a side effect.

The only difference between Medicine A and Medicine B is how you take them.

Medicine A	Medicine B
You take a tablet only once in your life.<br>You will be given the tablet on the day you come in to see your GP.<br>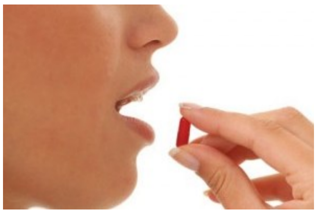	One tablet should be taken once a week on the same day every week.<br>You need to take this medicine for 5 years.<br><br>You need to take the tablet first thing in the morning on an empty stomach with a large glass of tap water.<br>After taking the tablet, you should stay upright for the next 30 minutes.<br>You should not eat anything for at least 30 minutes, including taking any other medicines.<br><br>You need to regularly visit your doctor to have your bone health checked.<br>You need to regularly visit a pharmacy for a new supply.<br>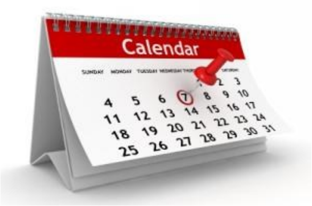 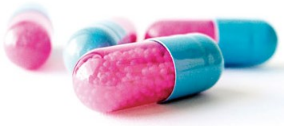

If you take Medicine A, you would live for **1 week less than 5 years** and then die.

If you take Medicine B, you would live for **5 years** and then die.

Would you prefer Medicine A or Medicine B, or are they the same?

Indicate your choice here. You can only choose one option:

◯ **Medicine A** Live for (5 years – 1 week)

◯ **Medicine B** Live for 5 years

◯ **The Same - Medicine A and Medicine B are the same.**

0% 100%

Repeated section

Remember Medicine A and Medicine B are equally effective and have no risks of a side effect.

The only difference between Medicine A and Medicine B is how you take them.

Medicine A	Medicine B
You take a tablet only once in your life. You will be given the tablet on the day you come in to see your GP. 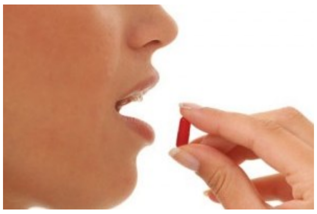	One tablet should be taken once a week on the same day every week.<br>You need to take this medicine for 5 years.<br><br>You need to take the tablet first thing in the morning on an empty stomach with a large glass of tap water.<br>After taking the tablet, you should stay upright for the next 30 minutes.<br>You should not eat anything for at least 30 minutes, including taking any other medicines.<br><br>You need to regularly visit your doctor to have your bone health checked.<br>You need to regularly visit a pharmacy for a new supply.<br>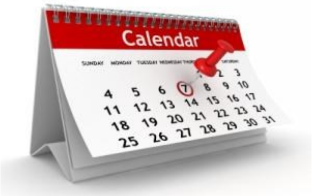 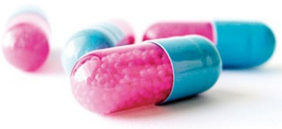

If you take Medicine A, you would live for **5 days less than 5 years** and then die.

If you take Medicine B, you would live for **5 years** and then die.

Would you prefer Medicine A or Medicine B, or are they the same?

Indicate your choice here. You can only choose one option:

◯ **Medicine A** Live for (5 years – 5 days)

◯ **Medicine B** Live for 5 years

◯ **The Same - Medicine A and Medicine B are the same.**

0% 100%

Repeated section

Remember Medicine A and Medicine B are equally effective and have no risks of a side effect.

The only difference between Medicine A and Medicine B is how you take them.

Medicine A	Medicine B
You take a tablet only once in your life. You will be given the tablet on the day you come in to see your GP.<br>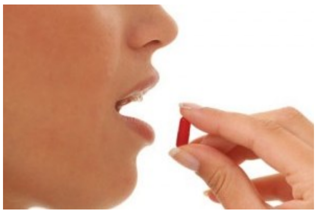	One tablet should be taken once a week on the same day every week.<br>You need to take this medicine for 5 years.<br><br>You need to take the tablet first thing in the morning on an empty stomach with a large glass of tap water.<br>After taking the tablet, you should stay upright for the next 30 minutes.<br>You should not eat anything for at least 30 minutes, including taking any other medicines.<br><br>You need to regularly visit your doctor to have your bone health checked.<br>You need to regularly visit a pharmacy for a new supply.<br>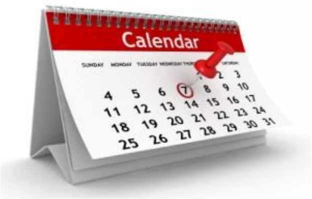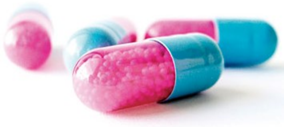

If you take Medicine A, you would live for **3 days less than 5 years** and then die.

If you take Medicine B, you would live for **5 years** and then die.

Would you prefer Medicine A or Medicine B, or are they the same?

Indicate your choice here. You can only choose one option:

○ **Medicine A** Live for (5 years – 3 days)

○ **Medicine B** Live for 5 years

○ **The Same - Medicine A and Medicine B are the same.**

0% 100%

Repeated section

Remember Medicine A and Medicine B are equally effective and have no risks of a side effect.

The only difference between Medicine A and Medicine B is how you take them.

Medicine A	Medicine B
You take a tablet only once in your life. You will be given the tablet on the day you come in to see your GP. 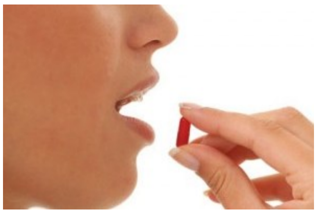	One tablet should be taken once a week on the same day every week.<br>You need to take this medicine for 5 years.<br><br>You need to take the tablet first thing in the morning on an empty stomach with a large glass of tap water.<br>After taking the tablet, you should stay upright for the next 30 minutes.<br>You should not eat anything for at least 30 minutes, including taking any other medicines.<br><br>You need to regularly visit your doctor to have your bone health checked.<br>You need to regularly visit a pharmacy for a new supply.<br>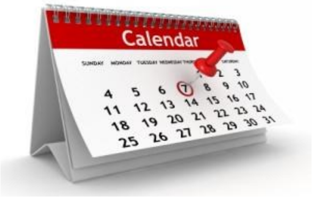 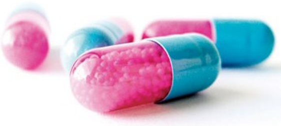

If you take Medicine A, you would live for **1 day less than 5 years** and then die.

If you take Medicine B, you would live for **5 years** and then die.

Would you prefer Medicine A or Medicine B, or are they the same?

Indicate your choice here. You can only choose one option:

◯ **Medicine A** Live for (5 years – 1 day)

◯ **Medicine B** Live for 5 years

◯ **The Same - Medicine A and Medicine B are the same.**

0% 100%

Question 4a.

You prefer living for 2.5 years (2 years and 6 months) on Medicine A, to living for 5 years on Medicine B.

This means **living fewer than 2.5 years on Medicine A** can be the same as living for 5 years on Medicine B.

How many years on Medicine A do you think would be the same as living for 5 years on Medicine B?

(Please give a number in years)

0% 100%

Was there anything so far which you think is unclear or that you didn't understand?

(Please provide details in the comment box below)

0% 100%

Question 5

You have been told that you need to take a medicine to reduce the risk of fracture.

You can choose between two different medicines. We have called these Medicine A and Medicine B.

Medicine A is a pill that you only need to take once.

Medicine B is a pill that you need to take every week for 5 years.

Medicine A and Medicine B are equally effective in reducing your risk of having a fracture.
Medicine A and Medicine B will reduce your risk of experiencing a fracture, such as hip fracture, within the next 5 years from **10%** to **6%**.

In other words, in a crowd of **1,000** people in the UK, **100** are likely to have a fracture if they do not take the medicine. This is reduced to **60** people out of 1,000 who do take the medicine.
This information is also illustrated in this picture.

Risk of fracture

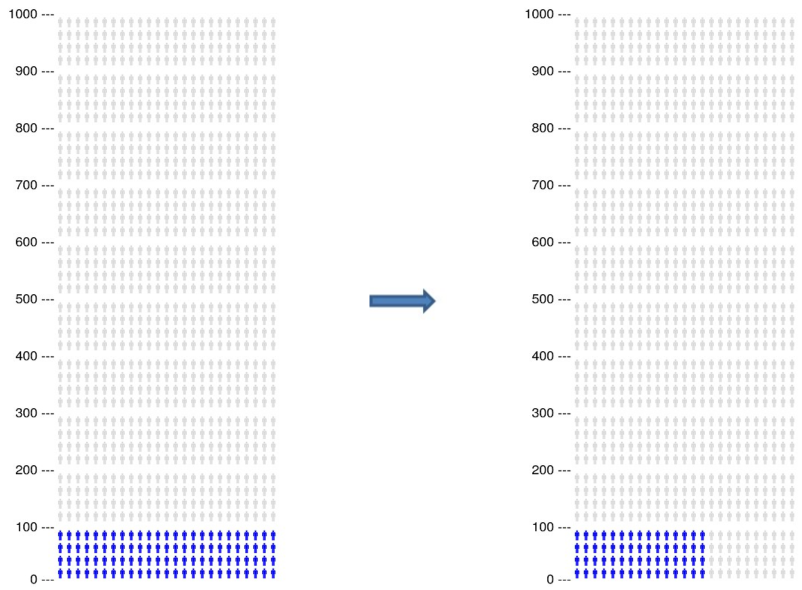

10%
No Treatment

6%
Taking the Medicine

(The figures shaded blue represent people who will have a fracture, such as hip fracture, within the next 5 years)

Please continue to the next page.

Repeated section

Remember Medicine A and Medicine B are equally effective.

However, there are some differences between Medicine A and Medicine B.

Medicine A	Medicine B
**No inconvenience**<br><br>You take a tablet only once in your life.<br>You will be given the tablet on the day you come in to see your GP.	**Inconvenience**<br><br>One tablet should be taken once a week on the same day every week.<br>You need to take this medicine for 5 years.<br><br>You need to take the tablet first thing in the morning on an empty stomach with a large glass of tap water.<br>After taking the tablet, you should stay upright for the next 30 minutes.<br><br>You should not eat anything for at least 30 minutes, including taking any other medicines.<br><br>You need to regularly visit your doctor to have your bone health checked.<br>You need to regularly visit a pharmacy for a new supply.
**Severe side effect - 0%**<br><br>No one taking Medicine A will have a severe side effect.	**Severe side effect - 0.7%**<br><br>Among every 1,000 people taking Medicine B, **7** will experience a severe side effect.
**Minor side effect - 0%**<br><br>No one taking Medicine A will have a minor side effect.	**Minor side effect - 13%**<br><br>Among every 1,000 people taking Medicine B, **130** will experience a minor side effect.

If you take Medicine A, you would live for **2.5 years (2 years and 6 months)** and then die.

If you take Medicine B, you would live for **5 years** and then die.

Would you prefer Medicine A or Medicine B, or are they the same?

Indicate your choice here. You can only choose one option:

◯ **Medicine A**

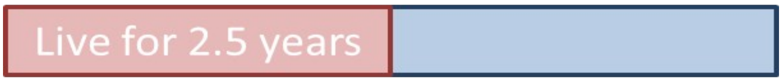

◯ **Medicine B**

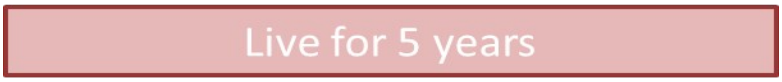

◯ **The Same - Medicine A and Medicine B are the same.**

0% 100%

Repeated section

Remember Medicine A and Medicine B are equally effective.

However, there are some differences between Medicine A and Medicine B.

Medicine A	Medicine B
**No inconvenience**<br><br>You take a tablet only once in your life.<br>You will be given the tablet on the day you come in to see your GP.	**Inconvenience**<br><br>One tablet should be taken once a week on the same day every week.<br>You need to take this medicine for 5 years.<br><br>You need to take the tablet first thing in the morning on an empty stomach with a large glass of tap water.<br>After taking the tablet, you should stay upright for the next 30 minutes.<br><br>You should not eat anything for at least 30 minutes, including taking any other medicines.<br><br>You need to regularly visit your doctor to have your bone health checked.<br>You need to regularly visit a pharmacy for a new supply.
**Severe side effect - 0%**<br><br>No one taking Medicine A will have a severe side effect.	**Severe side effect - 0.7%**<br><br>Among every 1,000 people taking Medicine B, **7** will experience a severe side effect.
**Minor side effect - 0%**<br><br>No one taking Medicine A will have a minor side effect.	**Minor side effect - 13%**<br><br>Among every 1,000 people taking Medicine B, **130** will experience a minor side effect.

If you take Medicine A, you would live for **3 years** and then die.

If you take Medicine B, you would live for **5 years** and then die.

Would you prefer Medicine A or Medicine B, or are they the same?

Indicate your choice here. You can only choose one option:

○ **Medicine A**

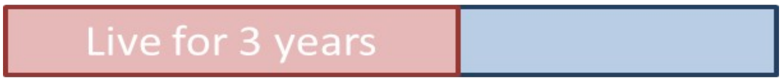

○ **Medicine B**

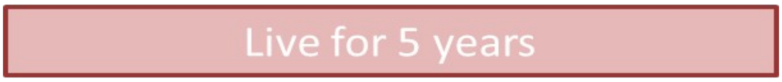

○ **The Same - Medicine A and Medicine B are the same.**

0% 100%

Repeated section

Remember Medicine A and Medicine B are equally effective.

However, there are some differences between Medicine A and Medicine B.

Medicine A	Medicine B
**No inconvenience**<br><br>You take a tablet only once in your life.<br>You will be given the tablet on the day you come in to see your GP.	**Inconvenience**<br><br>One tablet should be taken once a week on the same day every week.<br>You need to take this medicine for 5 years.<br><br>You need to take the tablet first thing in the morning on an empty stomach with a large glass of tap water.<br>After taking the tablet, you should stay upright for the next 30 minutes.<br><br>You should not eat anything for at least 30 minutes, including taking any other medicines.<br><br>You need to regularly visit your doctor to have your bone health checked.<br>You need to regularly visit a pharmacy for a new supply.
**Severe side effect - 0%**<br><br>No one taking Medicine A will have a severe side effect.	**Severe side effect - 0.7%**<br><br>Among every 1,000 people taking Medicine B, **7** will experience a severe side effect.
**Minor side effect - 0%**<br><br>No one taking Medicine A will have a minor side effect.	**Minor side effect - 13%**<br><br>Among every 1,000 people taking Medicine B, **130** will experience a minor side effect.

If you take Medicine A, you would live for **3.25 years** (3 years and 3 months) and then die.

If you take Medicine B, you would live for **5 years** and then die.

Would you prefer Medicine A or Medicine B, or are they the same?

Indicate your choice here. You can only choose one option:

◯ **Medicine A**

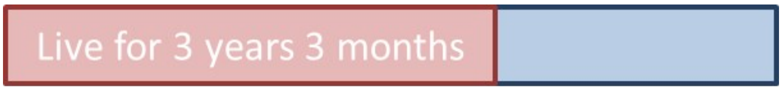

◯ **Medicine B**

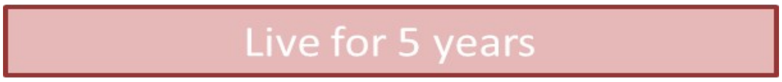

◯ **The Same - Medicine A and Medicine B are the same.**

0% 100%

Repeated section

Remember Medicine A and Medicine B are equally effective.

However, there are some differences between Medicine A and Medicine B.

Medicine A	Medicine B
**No inconvenience**<br><br>You take a tablet only once in your life.<br>You will be given the tablet on the day you come in to see your GP.	**Inconvenience**<br><br>One tablet should be taken once a week on the same day every week.<br>You need to take this medicine for 5 years.<br><br>You need to take the tablet first thing in the morning on an empty stomach with a large glass of tap water.<br>After taking the tablet, you should stay upright for the next 30 minutes.<br><br>You should not eat anything for at least 30 minutes, including taking any other medicines.<br><br>You need to regularly visit your doctor to have your bone health checked.<br>You need to regularly visit a pharmacy for a new supply.
**Severe side effect - 0%**<br><br>No one taking Medicine A will have a severe side effect.	**Severe side effect - 0.7%**<br><br>Among every 1,000 people taking Medicine B, **7** will experience a severe side effect.
**Minor side effect - 0%**<br><br>No one taking Medicine A will have a minor side effect.	**Minor side effect - 13%**<br><br>Among every 1,000 people taking Medicine B, **130** will experience a minor side effect.

If you take Medicine A, you would live for **3.5 years** (3 years and 6 months) and then die.

If you take Medicine B, you would live for **5 years** and then die.

Would you prefer Medicine A or Medicine B, or are they the same?

Indicate your choice here. You can only choose one option:

○ **Medicine A**

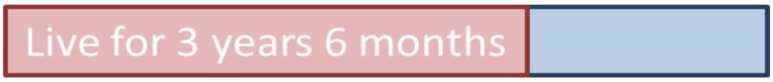

○ **Medicine B**

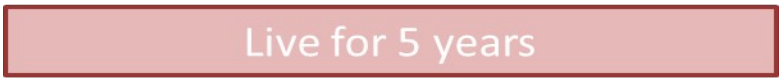

○ **The Same - Medicine A and Medicine B are the same.**

0% 100%

Repeated section

Remember Medicine A and Medicine B are equally effective.

However, there are some differences between Medicine A and Medicine B.

Medicine A	Medicine B
**No inconvenience**<br><br>You take a tablet only once in your life.<br>You will be given the tablet on the day you come in to see your GP.	**Inconvenience**<br><br>One tablet should be taken once a week on the same day every week.<br>You need to take this medicine for 5 years.<br><br>You need to take the tablet first thing in the morning on an empty stomach with a large glass of tap water.<br>After taking the tablet, you should stay upright for the next 30 minutes.<br><br>You should not eat anything for at least 30 minutes, including taking any other medicines.<br><br>You need to regularly visit your doctor to have your bone health checked.<br>You need to regularly visit a pharmacy for a new supply.
**Severe side effect - 0%**<br><br>No one taking Medicine A will have a severe side effect.	**Severe side effect - 0.7%**<br><br>Among every 1,000 people taking Medicine B, **7** will experience a severe side effect.
**Minor side effect - 0%**<br><br>No one taking Medicine A will have a minor side effect.	**Minor side effect - 13%**<br><br>Among every 1,000 people taking Medicine B, **130** will experience a minor side effect.

If you take Medicine A, you would live for **3.75 years** (3 years and 9 months) and then die.

If you take Medicine B, you would live for **5 years** and then die.

Would you prefer Medicine A or Medicine B, or are they the same?

Indicate your choice here. You can only choose one option:

○ **Medicine A**

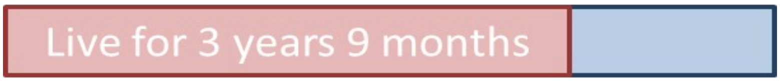

○ **Medicine B**

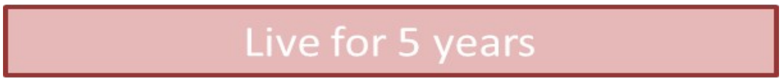

○ **The Same - Medicine A and Medicine B are the same.**

0% 100%

Repeated section

Remember Medicine A and Medicine B are equally effective.

However, there are some differences between Medicine A and Medicine B.

Medicine A	Medicine B
**No inconvenience**<br><br>You take a tablet only once in your life.<br>You will be given the tablet on the day you come in to see your GP.	**Inconvenience**<br><br>One tablet should be taken once a week on the same day every week.<br>You need to take this medicine for 5 years.<br><br>You need to take the tablet first thing in the morning on an empty stomach with a large glass of tap water.<br>After taking the tablet, you should stay upright for the next 30 minutes.<br><br>You should not eat anything for at least 30 minutes, including taking any other medicines.<br><br>You need to regularly visit your doctor to have your bone health checked.<br>You need to regularly visit a pharmacy for a new supply.
**Severe side effect - 0%**<br><br>No one taking Medicine A will have a severe side effect.	**Severe side effect - 0.7%**<br><br>Among every 1,000 people taking Medicine B, **7** will experience a severe side effect.
**Minor side effect - 0%**<br><br>No one taking Medicine A will have a minor side effect.	**Minor side effect - 13%**<br><br>Among every 1,000 people taking Medicine B, **130** will experience a minor side effect.

If you take Medicine A, you would live for **3 years and 10 months** and then die.

If you take Medicine B, you would live for **5 years** and then die.

Would you prefer Medicine A or Medicine B, or are they the same?

Indicate your choice here. You can only choose one option:

○ **Medicine A**

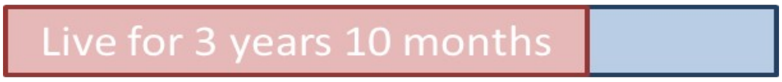

○ **Medicine B**

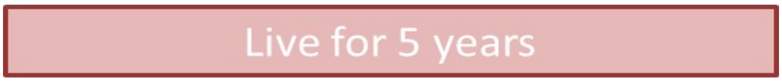

○ **The Same - Medicine A and Medicine B are the same.**

Repeated section

Remember Medicine A and Medicine B are equally effective.

However, there are some differences between Medicine A and Medicine B.

Medicine A	Medicine B
**No inconvenience**<br><br>You take a tablet only once in your life.<br>You will be given the tablet on the day you come in to see your GP.	**Inconvenience**<br><br>One tablet should be taken once a week on the same day every week.<br>You need to take this medicine for 5 years.<br><br>You need to take the tablet first thing in the morning on an empty stomach with a large glass of tap water.<br>After taking the tablet, you should stay upright for the next 30 minutes.<br><br>You should not eat anything for at least 30 minutes, including taking any other medicines.<br><br>You need to regularly visit your doctor to have your bone health checked.<br>You need to regularly visit a pharmacy for a new supply.
**Severe side effect - 0%**<br><br>No one taking Medicine A will have a severe side effect.	**Severe side effect - 0.7%**<br><br>Among every 1,000 people taking Medicine B, **7** will experience a severe side effect.
**Minor side effect - 0%**<br><br>No one taking Medicine A will have a minor side effect.	**Minor side effect - 13%**<br><br>Among every 1,000 people taking Medicine B, **130** will experience a minor side effect.

If you take Medicine A, you would live for **4 years** and then die.

If you take Medicine B, you would live for **5 years** and then die.

Would you prefer Medicine A or Medicine B, or are they the same?

Indicate your choice here. You can only choose one option:

○ **Medicine A**

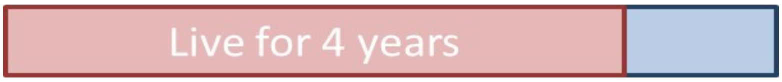

○ **Medicine B**

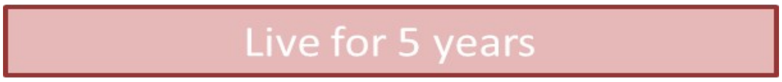

○ **The Same - Medicine A and Medicine B are the same.**

0% 100%

Repeated section

Remember Medicine A and Medicine B are equally effective.

However, there are some differences between Medicine A and Medicine B.

Medicine A	Medicine B
**No inconvenience**<br><br>You take a tablet only once in your life.<br>You will be given the tablet on the day you come in to see your GP.	**Inconvenience**<br><br>One tablet should be taken once a week on the same day every week.<br>You need to take this medicine for 5 years.<br><br>You need to take the tablet first thing in the morning on an empty stomach with a large glass of tap water.<br>After taking the tablet, you should stay upright for the next 30 minutes.<br><br>You should not eat anything for at least 30 minutes, including taking any other medicines.<br><br>You need to regularly visit your doctor to have your bone health checked.<br>You need to regularly visit a pharmacy for a new supply.
**Severe side effect - 0%**<br><br>No one taking Medicine A will have a severe side effect.	**Severe side effect - 0.7%**<br><br>Among every 1,000 people taking Medicine B, **7** will experience a severe side effect.
**Minor side effect - 0%**<br><br>No one taking Medicine A will have a minor side effect.	**Minor side effect - 13%**<br><br>Among every 1,000 people taking Medicine B, **130** will experience a minor side effect.

If you take Medicine A, you would live for **4 years and 2 months** and then die.

If you take Medicine B, you would live for **5 years** and then die.

Would you prefer Medicine A or Medicine B, or are they the same?

Indicate your choice here. You can only choose one option:

◯ **Medicine A**

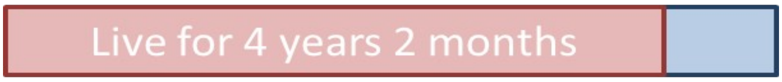

◯ **Medicine B**

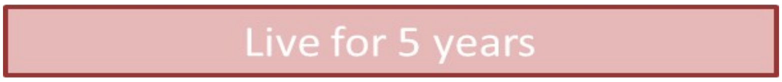

◯ **The Same - Medicine A and Medicine B are the same.**

Repeated section

Remember Medicine A and Medicine B are equally effective.

However, there are some differences between Medicine A and Medicine B.

Medicine A	Medicine B
**No inconvenience**<br><br>You take a tablet only once in your life.<br>You will be given the tablet on the day you come in to see your GP.	**Inconvenience**<br><br>One tablet should be taken once a week on the same day every week.<br>You need to take this medicine for 5 years.<br><br>You need to take the tablet first thing in the morning on an empty stomach with a large glass of tap water.<br>After taking the tablet, you should stay upright for the next 30 minutes.<br><br>You should not eat anything for at least 30 minutes, including taking any other medicines.<br><br>You need to regularly visit your doctor to have your bone health checked.<br>You need to regularly visit a pharmacy for a new supply.
**Severe side effect - 0%**<br><br>No one taking Medicine A will have a severe side effect.	**Severe side effect - 0.7%**<br><br>Among every 1,000 people taking Medicine B, **7** will experience a severe side effect.
**Minor side effect - 0%**<br><br>No one taking Medicine A will have a minor side effect.	**Minor side effect - 13%**<br><br>Among every 1,000 people taking Medicine B, **130** will experience a minor side effect.

If you take Medicine A, you would live for **4.25 years** (4 years and 3 months) and then die.

If you take Medicine B, you would live for **5 years** and then die.

Would you prefer Medicine A or Medicine B, or are they the same?

Indicate your choice here. You can only choose one option:

○ **Medicine A** Live for 4 years 3 months

○ **Medicine B** Live for 5 years

○ **The Same - Medicine A and Medicine B are the same.**

0% 100%

Repeated section

Remember Medicine A and Medicine B are equally effective.

However, there are some differences between Medicine A and Medicine B.

Medicine A	Medicine B
**No inconvenience**<br><br>You take a tablet only once in your life.<br>You will be given the tablet on the day you come in to see your GP.	**Inconvenience**<br><br>One tablet should be taken once a week on the same day every week.<br>You need to take this medicine for 5 years.<br><br>You need to take the tablet first thing in the morning on an empty stomach with a large glass of tap water.<br>After taking the tablet, you should stay upright for the next 30 minutes.<br><br>You should not eat anything for at least 30 minutes, including taking any other medicines.<br><br>You need to regularly visit your doctor to have your bone health checked.<br>You need to regularly visit a pharmacy for a new supply.
**Severe side effect - 0%**<br><br>No one taking Medicine A will have a severe side effect.	**Severe side effect - 0.7%**<br><br>Among every 1,000 people taking Medicine B, **7** will experience a severe side effect.
**Minor side effect - 0%**<br><br>No one taking Medicine A will have a minor side effect.	**Minor side effect - 13%**<br><br>Among every 1,000 people taking Medicine B, **130** will experience a minor side effect.

If you take Medicine A, you would live for **4 years 4 months** and then die.

If you take Medicine B, you would live for **5 years** and then die.

Would you prefer Medicine A or Medicine B, or are they the same?

Indicate your choice here. You can only choose one option:

◯ **Medicine A**

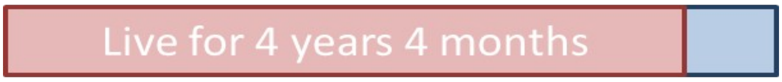

◯ **Medicine B**

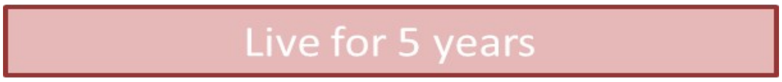

◯ **The Same - Medicine A and Medicine B are the same.**

0% 100%

Repeated section

Remember Medicine A and Medicine B are equally effective.

However, there are some differences between Medicine A and Medicine B.

Medicine A	Medicine B
**No inconvenience**<br><br>You take a tablet only once in your life.<br>You will be given the tablet on the day you come in to see your GP.	**Inconvenience**<br><br>One tablet should be taken once a week on the same day every week.<br>You need to take this medicine for 5 years.<br><br>You need to take the tablet first thing in the morning on an empty stomach with a large glass of tap water.<br>After taking the tablet, you should stay upright for the next 30 minutes.<br><br>You should not eat anything for at least 30 minutes, including taking any other medicines.<br><br>You need to regularly visit your doctor to have your bone health checked.<br>You need to regularly visit a pharmacy for a new supply.
**Severe side effect - 0%**<br><br>No one taking Medicine A will have a severe side effect.	**Severe side effect - 0.7%**<br><br>Among every 1,000 people taking Medicine B, **7** will experience a severe side effect.
**Minor side effect - 0%**<br><br>No one taking Medicine A will have a minor side effect.	**Minor side effect - 13%**<br><br>Among every 1,000 people taking Medicine B, **130** will experience a minor side effect.

If you take Medicine A, you would live for **4.5 years** (4 years and 6 months) and then die.

If you take Medicine B, you would live for **5 years** and then die.

Would you prefer Medicine A or Medicine B, or are they the same?

Indicate your choice here. You can only choose one option:

○ **Medicine A** 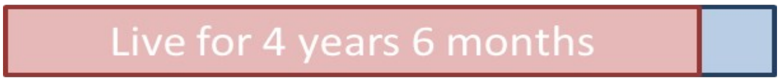

○ **Medicine B** 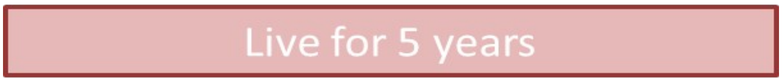

○ **The Same - Medicine A and Medicine B are the same.**

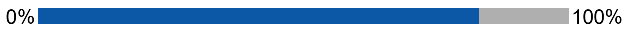
0%
100%

Repeated section

Remember Medicine A and Medicine B are equally effective.

However, there are some differences between Medicine A and Medicine B.

Medicine A	Medicine B
**No inconvenience**<br><br>You take a tablet only once in your life.<br>You will be given the tablet on the day you come in to see your GP.	**Inconvenience**<br><br>One tablet should be taken once a week on the same day every week.<br>You need to take this medicine for 5 years.<br><br>You need to take the tablet first thing in the morning on an empty stomach with a large glass of tap water.<br>After taking the tablet, you should stay upright for the next 30 minutes.<br><br>You should not eat anything for at least 30 minutes, including taking any other medicines.<br><br>You need to regularly visit your doctor to have your bone health checked.<br>You need to regularly visit a pharmacy for a new supply.
**Severe side effect - 0%**<br><br>No one taking Medicine A will have a severe side effect.	**Severe side effect - 0.7%**<br><br>Among every 1,000 people taking Medicine B, **7** will experience a severe side effect.
**Minor side effect - 0%**<br><br>No one taking Medicine A will have a minor side effect.	**Minor side effect - 13%**<br><br>Among every 1,000 people taking Medicine B, **130** will experience a minor side effect.

If you take Medicine A, you would live for **4 years and 7 months** and then die.

If you take Medicine B, you would live for **5 years** and then die.

Would you prefer Medicine A or Medicine B, or are they the same?

Indicate your choice here. You can only choose one option:

◯ **Medicine A**

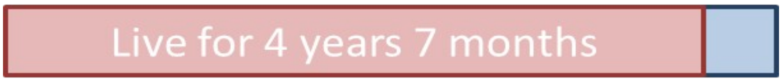

◯ **Medicine B**

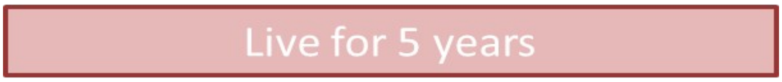

◯ **The Same - Medicine A and Medicine B are the same.**

0% 100%

Repeated section

Remember Medicine A and Medicine B are equally effective.

However, there are some differences between Medicine A and Medicine B.

Medicine A	Medicine B
**No inconvenience**<br><br>You take a tablet only once in your life.<br>You will be given the tablet on the day you come in to see your GP.	**Inconvenience**<br><br>One tablet should be taken once a week on the same day every week.<br>You need to take this medicine for 5 years.<br><br>You need to take the tablet first thing in the morning on an empty stomach with a large glass of tap water.<br>After taking the tablet, you should stay upright for the next 30 minutes.<br><br>You should not eat anything for at least 30 minutes, including taking any other medicines.<br><br>You need to regularly visit your doctor to have your bone health checked.<br>You need to regularly visit a pharmacy for a new supply.
**Severe side effect - 0%**<br><br>No one taking Medicine A will have a severe side effect.	**Severe side effect - 0.7%**<br><br>Among every 1,000 people taking Medicine B, **7** will experience a severe side effect.
**Minor side effect - 0%**<br><br>No one taking Medicine A will have a minor side effect.	**Minor side effect - 13%**<br><br>Among every 1,000 people taking Medicine B, **130** will experience a minor side effect.

If you take Medicine A, you would live for **4 years and 9 months** and then die.

If you take Medicine B, you would live for **5 years** and then die.

Would you prefer Medicine A or Medicine B, or are they the same?

Indicate your choice here. You can only choose one option:

○ **Medicine A**

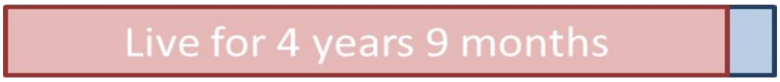

○ **Medicine B**

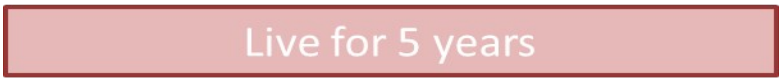

○ **The Same - Medicine A and Medicine B are the same.**

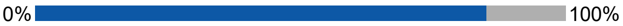
0%
100%

Repeated section

Remember Medicine A and Medicine B are equally effective.

However, there are some differences between Medicine A and Medicine B.

Medicine A	Medicine B
**No inconvenience**<br><br>You take a tablet only once in your life.<br>You will be given the tablet on the day you come in to see your GP.	**Inconvenience**<br><br>One tablet should be taken once a week on the same day every week.<br>You need to take this medicine for 5 years.<br><br>You need to take the tablet first thing in the morning on an empty stomach with a large glass of tap water.<br>After taking the tablet, you should stay upright for the next 30 minutes.<br><br>You should not eat anything for at least 30 minutes, including taking any other medicines.<br><br>You need to regularly visit your doctor to have your bone health checked.<br>You need to regularly visit a pharmacy for a new supply.
**Severe side effect - 0%**<br><br>No one taking Medicine A will have a severe side effect.	**Severe side effect - 0.7%**<br><br>Among every 1,000 people taking Medicine B, **7** will experience a severe side effect.
**Minor side effect - 0%**<br><br>No one taking Medicine A will have a minor side effect.	**Minor side effect - 13%**<br><br>Among every 1,000 people taking Medicine B, **130** will experience a minor side effect.

If you take Medicine A, you would live for **2 months less than 5 years** and then die.

If you take Medicine B, you would live for **5 years** and then die.

Would you prefer Medicine A or Medicine B, or are they the same?

Indicate your choice here. You can only choose one option:

◯ **Medicine A** — Live for (5 years – 2 months)

◯ **Medicine B** — Live for 5 years

◯ **The Same - Medicine A and Medicine B are the same.**

0% 100%

Repeated section

Remember Medicine A and Medicine B are equally effective.

However, there are some differences between Medicine A and Medicine B.

Medicine A	Medicine B
**No inconvenience**<br><br>You take a tablet only once in your life.<br>You will be given the tablet on the day you come in to see your GP.	**Inconvenience**<br><br>One tablet should be taken once a week on the same day every week.<br>You need to take this medicine for 5 years.<br><br>You need to take the tablet first thing in the morning on an empty stomach with a large glass of tap water.<br>After taking the tablet, you should stay upright for the next 30 minutes.<br><br>You should not eat anything for at least 30 minutes, including taking any other medicines.<br><br>You need to regularly visit your doctor to have your bone health checked.<br>You need to regularly visit a pharmacy for a new supply.
**Severe side effect - 0%**<br><br>No one taking Medicine A will have a severe side effect.	**Severe side effect - 0.7%**<br><br>Among every 1,000 people taking Medicine B, **7** will experience a severe side effect.
**Minor side effect - 0%**<br><br>No one taking Medicine A will have a minor side effect.	**Minor side effect - 13%**<br><br>Among every 1,000 people taking Medicine B, **130** will experience a minor side effect.

If you take Medicine A, you would live for **1 month less than 5 years** and then die.

If you take Medicine B, you would live for **5 years** and then die.

Would you prefer Medicine A or Medicine B, or are they the same?

Indicate your choice here. You can only choose one option:

◯ **Medicine A** Live for (5 years – 1 month)

◯ **Medicine B** Live for 5 years

◯ **The Same - Medicine A and Medicine B are the same.**

0% 100%

Repeated section

Remember Medicine A and Medicine B are equally effective.

However, there are some differences between Medicine A and Medicine B.

Medicine A	Medicine B
**No inconvenience**<br><br>You take a tablet only once in your life.<br>You will be given the tablet on the day you come in to see your GP.	**Inconvenience**<br><br>One tablet should be taken once a week on the same day every week.<br>You need to take this medicine for 5 years.<br><br>You need to take the tablet first thing in the morning on an empty stomach with a large glass of tap water.<br>After taking the tablet, you should stay upright for the next 30 minutes.<br><br>You should not eat anything for at least 30 minutes, including taking any other medicines.<br><br>You need to regularly visit your doctor to have your bone health checked.<br>You need to regularly visit a pharmacy for a new supply.
**Severe side effect - 0%**<br><br>No one taking Medicine A will have a severe side effect.	**Severe side effect - 0.7%**<br><br>Among every 1,000 people taking Medicine B, **7** will experience a severe side effect.
**Minor side effect - 0%**<br><br>No one taking Medicine A will have a minor side effect.	**Minor side effect - 13%**<br><br>Among every 1,000 people taking Medicine B, **130** will experience a minor side effect.

If you take Medicine A, you would live for **2 weeks less than 5 years** and then die.

If you take Medicine B, you would live for **5 years** and then die.

Would you prefer Medicine A or Medicine B, or are they the same?

Indicate your choice here. You can only choose one option:

◯ **Medicine A** Live for (5 years – 2 weeks)

◯ **Medicine B** Live for 5 years

◯ **The Same - Medicine A and Medicine B are the same.**

0% 100%

Repeated section

Remember Medicine A and Medicine B are equally effective.

However, there are some differences between Medicine A and Medicine B.

Medicine A	Medicine B
**No inconvenience**<br><br>You take a tablet only once in your life.<br>You will be given the tablet on the day you come in to see your GP.	**Inconvenience**<br><br>One tablet should be taken once a week on the same day every week.<br>You need to take this medicine for 5 years.<br><br>You need to take the tablet first thing in the morning on an empty stomach with a large glass of tap water.<br>After taking the tablet, you should stay upright for the next 30 minutes.<br><br>You should not eat anything for at least 30 minutes, including taking any other medicines.<br><br>You need to regularly visit your doctor to have your bone health checked.<br>You need to regularly visit a pharmacy for a new supply.
**Severe side effect - 0%**<br><br>No one taking Medicine A will have a severe side effect.	**Severe side effect - 0.7%**<br><br>Among every 1,000 people taking Medicine B, **7** will experience a severe side effect.
**Minor side effect - 0%**<br><br>No one taking Medicine A will have a minor side effect.	**Minor side effect - 13%**<br><br>Among every 1,000 people taking Medicine B, **130** will experience a minor side effect.

If you take Medicine A, you would live for **1 week less than 5 years** and then die.

If you take Medicine B, you would live for **5 years** and then die.

Would you prefer Medicine A or Medicine B, or are they the same?

Indicate your choice here. You can only choose one option:

◯ **Medicine A** Live for (5 years – 1 week)

◯ **Medicine B** Live for 5 years

◯ **The Same - Medicine A and Medicine B are the same.**

0% 100%

Repeated section

Remember Medicine A and Medicine B are equally effective.

However, there are some differences between Medicine A and Medicine B.

Medicine A	Medicine B
**No inconvenience**<br><br>You take a tablet only once in your life.<br>You will be given the tablet on the day you come in to see your GP.	**Inconvenience**<br><br>One tablet should be taken once a week on the same day every week.<br>You need to take this medicine for 5 years.<br><br>You need to take the tablet first thing in the morning on an empty stomach with a large glass of tap water.<br>After taking the tablet, you should stay upright for the next 30 minutes.<br><br>You should not eat anything for at least 30 minutes, including taking any other medicines.<br><br>You need to regularly visit your doctor to have your bone health checked.<br>You need to regularly visit a pharmacy for a new supply.
**Severe side effect - 0%**<br><br>No one taking Medicine A will have a severe side effect.	**Severe side effect - 0.7%**<br><br>Among every 1,000 people taking Medicine B, **7** will experience a severe side effect.
**Minor side effect - 0%**<br><br>No one taking Medicine A will have a minor side effect.	**Minor side effect - 13%**<br><br>Among every 1,000 people taking Medicine B, **130** will experience a minor side effect.

If you take Medicine A, you would live for **5 days less than 5 years** and then die.

If you take Medicine B, you would live for **5 years** and then die.

Would you prefer Medicine A or Medicine B, or are they the same?

Indicate your choice here. You can only choose one option:

○ **Medicine A** Live for (5 years – 5 days)

○ **Medicine B** Live for 5 years

○ **The Same - Medicine A and Medicine B are the same.**

0% 100%

Repeated section

Remember Medicine A and Medicine B are equally effective.

However, there are some differences between Medicine A and Medicine B.

Medicine A	Medicine B
**No inconvenience**<br><br>You take a tablet only once in your life.<br>You will be given the tablet on the day you come in to see your GP.	**Inconvenience**<br><br>One tablet should be taken once a week on the same day every week.<br>You need to take this medicine for 5 years.<br><br>You need to take the tablet first thing in the morning on an empty stomach with a large glass of tap water.<br>After taking the tablet, you should stay upright for the next 30 minutes.<br><br>You should not eat anything for at least 30 minutes, including taking any other medicines.<br><br>You need to regularly visit your doctor to have your bone health checked.<br>You need to regularly visit a pharmacy for a new supply.
**Severe side effect - 0%**<br><br>No one taking Medicine A will have a severe side effect.	**Severe side effect - 0.7%**<br><br>Among every 1,000 people taking Medicine B, **7** will experience a severe side effect.
**Minor side effect - 0%**<br><br>No one taking Medicine A will have a minor side effect.	**Minor side effect - 13%**<br><br>Among every 1,000 people taking Medicine B, **130** will experience a minor side effect.

If you take Medicine A, you would live for **3 days less than 5 years** and then die.

If you take Medicine B, you would live for **5 years** and then die.

Would you prefer Medicine A or Medicine B, or are they the same?

Indicate your choice here. You can only choose one option:

◯ **Medicine A** Live for (5 years – 3 days)

◯ **Medicine B** Live for 5 years

◯ **The Same - Medicine A and Medicine B are the same.**

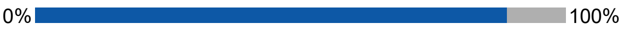
0%
100%

Repeated section

Remember Medicine A and Medicine B are equally effective.

However, there are some differences between Medicine A and Medicine B.

Medicine A	Medicine B
**No inconvenience**<br><br>You take a tablet only once in your life.<br>You will be given the tablet on the day you come in to see your GP.	**Inconvenience**<br><br>One tablet should be taken once a week on the same day every week.<br>You need to take this medicine for 5 years.<br><br>You need to take the tablet first thing in the morning on an empty stomach with a large glass of tap water.<br>After taking the tablet, you should stay upright for the next 30 minutes.<br><br>You should not eat anything for at least 30 minutes, including taking any other medicines.<br><br>You need to regularly visit your doctor to have your bone health checked.<br>You need to regularly visit a pharmacy for a new supply.
**Severe side effect - 0%**<br><br>No one taking Medicine A will have a severe side effect.	**Severe side effect - 0.7%**<br><br>Among every 1,000 people taking Medicine B, **7** will experience a severe side effect.
**Minor side effect - 0%**<br><br>No one taking Medicine A will have a minor side effect.	**Minor side effect - 13%**<br><br>Among every 1,000 people taking Medicine B, **130** will experience a minor side effect.

If you take Medicine A, you would live for **1 day less than 5 years** and then die.

If you take Medicine B, you would live for **5 years** and then die.

Would you prefer Medicine A or Medicine B, or are they the same?

Indicate your choice here. You can only choose one option:

○ **Medicine A** Live for (5 years – 1 day)

○ **Medicine B** Live for 5 years

○ **The Same - Medicine A and Medicine B are the same.**

Question 5a.

You prefer living for 2.5 years (2 years and 6 months) on Medicine A, to living for 5 years on Medicine B.

This means **living fewer than 2.5 years on Medicine A** can be the same as living for 5 years on Medicine B.

How many years on Medicine A do you think would be the same as living for 5 years on Medicine B?

(Please give a number in years) [ ]

0% 100%

Thank you for completing this section and indicating your choices.

Was there anything in this section which you think is unclear or that you didn't understand?

(Please provide details in the comment box below)

0% 100%

Part 3. Questions about you

We understand different people feel differently about different treatments.

In this part of the survey, we ask questions about you to understand if people's age, gender or experiences explain the choices made in Part 2.

0% 100%

We would like to learn about your experience.

Are you currently taking, or have you ever taken any of these medicines (bisphosphonates)?

Aclasta®
Actonel®
Alendronic acid
Binosto®
Bondronat®
Bonefos®
Bonviva®
Clasteon®
Fosamax®
Iasibon®
Ibandronic acid
Loron®
Pamidronate disodium
Quodixor®
Risedronate sodium
Sodium clodronate
Zerlinda®
Zoledronic acid
Zometa®

◯ Yes
◯ No

0% 100%

What do you think would be the **benefits** from taking this medicine (a bisphosphonate)?

What do you think would be the **harms** from taking this medicine (a bisphosphonate)?

Have you experienced any side effects from taking this medicine (a bisphosphonate)?

- ◯ Yes
- ◯ No

Do you find it inconvenient to take this medicine (a bisphosphonate)?
Please specify if you think it is inconvenient.

What is your opinion about taking this medicine (a bisphosphonate)?

- ◯ I don't mind taking them because I have to take them for a reason.
- ◯ I do not like taking them because they remind me that I am not perfectly healthy.
- ◯ I have no opinion.
- ◯ Other (please specify)

Do you take other medicines prescribed by your doctor?

- ◯ Yes
- ◯ No

In total, how many pills do you take on a regular basis?
(Please do not include pills you take only once in a while)

- ◯ None
- ◯ One
- ◯ Two to five
- ◯ Six to ten
- ◯ More than ten

How many different times a day do you regularly take pills?

- ◯ None
- ◯ Once per day
- ◯ 2 times a day
- ◯ 3 times a day
- ◯ More than 3 times a day

Do you find it inconvenient to take this medicine?
Please specify if you think it is inconvenient.

What is your opinion about taking this medicine?

- ◯ I don't mind taking them because I have to take them for a reason.
- ◯ I do not like taking them because they remind me that I am not perfectly healthy.
- ◯ I have no opinion.
- ◯ Other (please specify) ____

We would like to ask questions about you to better understand your choices.

What is your gender?

- ◯ Male
- ◯ Female
- ◯ Other (please specify) ______

Which age category do you fall into?

- ◯ 34 years or younger
- ◯ 35-44 years
- ◯ 45-54 years
- ◯ 55-64 years
- ◯ 65-74 years
- ◯ 75-84 years
- ◯ 85 years or over

Which best reflects your ethnic group?

- ◯ White British/Irish
- ◯ White other
- ◯ Mixed/Multiple ethnic origins
- ◯ Black/African/Caribbean/Black British
- ◯ Asian/Asian British
- ◯ Other (please specify) ______

0% 100%

What is your occupational status?

- ◯ Employed full-time
- ◯ Employed part-time
- ◯ Self-employed
- ◯ Unemployed
- ◯ Retired
- ◯ Looking after a home/family
- ◯ Student
- ◯ Freelance or temping
- ◯ Long-term sickness

Could you tell us what your occupation is?
(Previous occupation if retired.)

What is the highest level of education you have obtained?
(for example, GCSEs, Degrees, etc.)

What is your religion?

- ◯ No religion
- ◯ Christian
- ◯ Buddhist
- ◯ Jewish
- ◯ Hindu
- ◯ Muslim
- ◯ Sikh
- ◯ Other

How do you feel about numbers?

This section will ask you some questions to understand how familiar you are with numbers and probabilities.

If you are unsure of the answer, you can leave the response blank.

0% 100%

Question 1

Imagine I flip a coin 1,000 times.

What is your best guess about how many times the coin would fall heads up in 1,000 flips?

- ◯ Answer: [ ]
- ◯ Prefer not to answer
- ◯ Don't know.

Question 2

If there was a lottery where the chance of winning £10 is 1%, if 1,000 people bought a ticket, how many would you expect to win?

- ◯ Answer: [ ]
- ◯ Prefer not to answer
- ◯ Don't know.

Question 3

In another lottery, the chance of winning a car is 1 in 1,000.

What percentage of tickets in the lottery will win a car?

- ◯ Answer: [ ] %
- ◯ Prefer not to answer
- ◯ Don't know

0% 100%

How confident are you that you would answer the questions the same if faced with the situations in real-life?

	Very confident	Confident	Not sure	Not confident	Not confident at all	
Confident	◯	◯	◯	◯	◯	Not confident at all

How easy or difficult did you find making choices between alternatives?

	Very easy	Easy	Average	Somewhat difficult	Very difficult	
Easy	◯	◯	◯	◯	◯	Difficult

How easy or difficult did you find the survey to understand?

	Very easy	Easy	Average	Somewhat difficult	Very difficult	
Easy	◯	◯	◯	◯	◯	Difficult

0% 100%

Was there anything in the survey that was particularly unclear?

(Please write in the box below)

Is there anything else about the survey we could change to make it easier to uncerstand (for example, phrasing and explanation)?

(Optional) If you have any further comments or suggestions, please write them here.

0% 100%

Thank you for completing this survey

If you would like more information about osteoporosis please visit the NHS Choices website here.

MANCHESTER 1824
The University of Manchester

0% 100%

Note:

When respondents take the survey in regular mode this page will not be displayed. Respondents will be redirected to the link below:

https://dkr1.ssisurveys.com/projects/end?rst=1&psid=[Script]&basic=99423

0% 100%

Note:

When respondents take the survey in regular mode this page will not be displayed. Respondents will be redirected to the link below:

https://dkr1.ssisurveys.com/projects/end?rst=2&psid= [Script]

0% 100%

Note:

When respondents take the survey in regular mode this page will not be displayed. Respondents will be redirected to the link below:

https://dkr1.ssisurveys.com/projects/end?rst=3&psid= [Script]

0% 100%
