# Supplementary material for: Quantifying the impact of taking medicines for primary prevention: a time-trade off study to elicit direct treatment disutility in the UK
Source: BMJ Open. 2023 Sep 21;13(9):e063800. doi: 10.1136/bmjopen-2022-063800 (PMC10514632; doi:10.1136/bmjopen-2022-063800)
Supplement: Supplementary data [file bmjopen-2022-063800supp001.pdf]

Supplementary Appendix 1: The statin survey

See separate pdf file

Supplementary Appendix 2: The bisphosphonate survey

See separate pdf file

Supplementary Appendix 3: Training materials for statin survey

Statins for the prevention of CVD

Bobbie has been to see a doctor.

The doctor has said Bobbie needs to start a medicine to prevent serious events because of his heart disease.

We will take you through Bobbie's story.

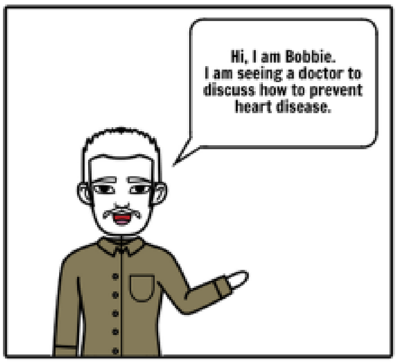

What is heart disease?

Bobbie hears about heart disease from his GP.

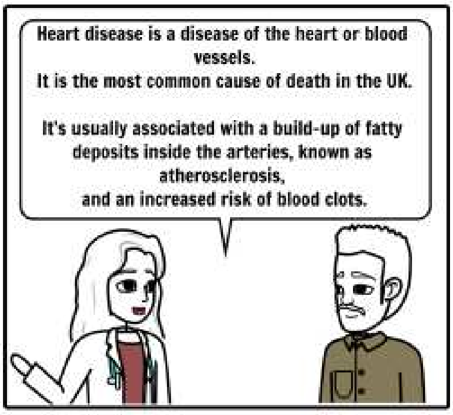

Impact of heart disease

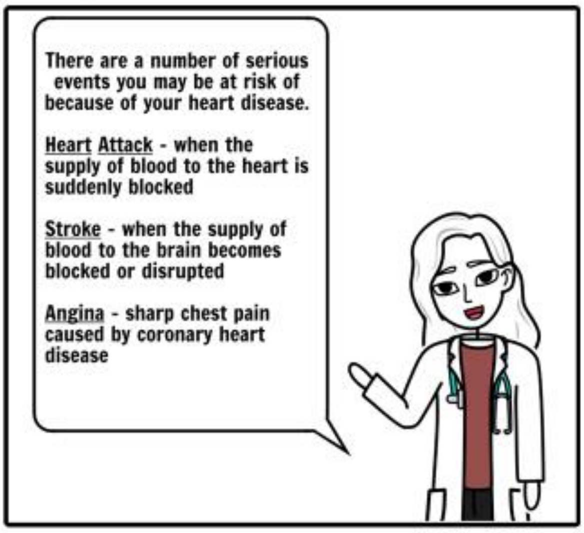

Risk of Heart disease

The GP explains that there is a chance that because of his cholesterol level Bobbie is at risk of developing one of these serious events in the future.

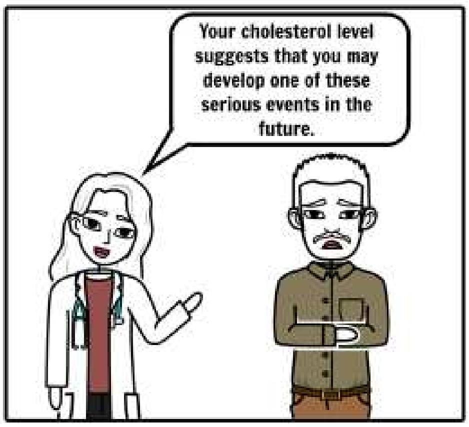

What is risk?

- **Risk** is: a term used to explain the chance that something bad might happen.
- **10% risk** means:
  Out of every **1,000** people, with heart disease,
  100 people would develop a heart attack, and **900** people would not.

What is a 10% risk?

- This diagram shows a 10% risk of having a heart attack because of their heart disease.
- The people shaded blue are the ones who have a heart attack.

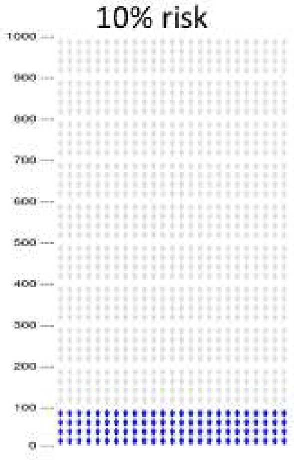

Prevention of serious events from heart disease

- Bobbie's doctor can estimate how likely it is that he will develop heart disease over the next 10 years.
- The estimate is based on things such as Bobbie's age and sex, family medical history, blood pressure and cholesterol level.
- The doctor may prescribe certain medicines such as statins to help reduce the chance of Bobbie having heart disease.
- The medicine will prevent some of the people who take it from having a serious event because of the heart disease.
- A serious event from the heart disease will still happen to some of the people who take the medicine.

Prevention of serious events from heart disease

The doctor prescribes Bobbie a medicine to reduce the chance of his heart disease causing a heart attack or other heart conditions in the future.

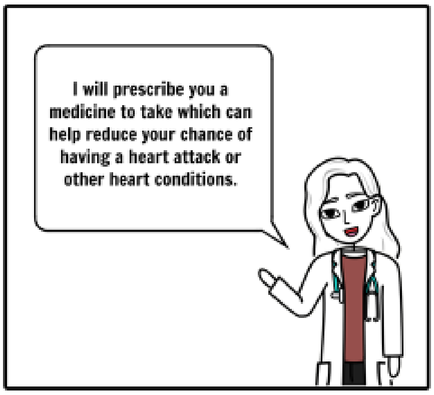

Collecting the medicine from the pharmacy

Bobbie goes to a pharmacy to collect his medicine.

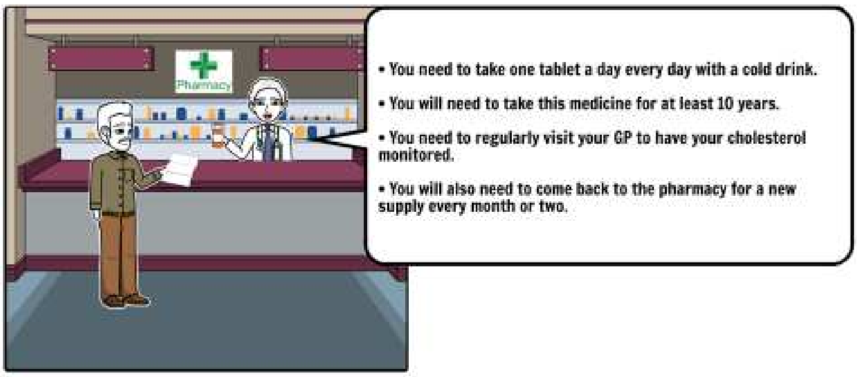

Taking the medicine for prevention

Bobbie goes home with his new tablets.

He then has to decide whether he wants to take them as the doctor and the pharmacist have advised.

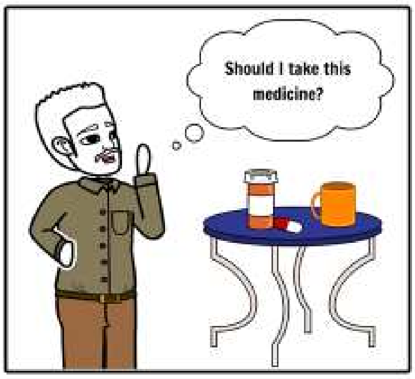

- Bobbie may consider three key things, when deciding whether to take the medicine.

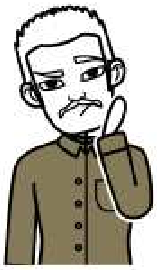

Effectiveness of the medicine

- How good the medicine is in terms of preventing a condition like a heart attack

Side effects of the medicine

- Whether there is any potential harm from the medicine

Inconvenience of the medicine

- Whether taking the medicine would fit in his lifestyle or cause him inconvenience

How effective is the medicine?

- Some people, but not everyone, who take the medicine will avoid a serious event, such as a heart attack or stroke as a result of taking the tablet.
- The effectiveness of the medicine can be described in terms of a reduction in this risk.

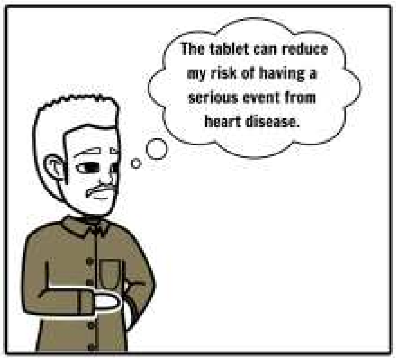

What does 'reduction in risk' mean?

- A 'reduction in risk from 10% to 6%' means:

The medicine will reduce your risk of experiencing an event, such as a heart attack, within the next 10 years from 10% to 6%.

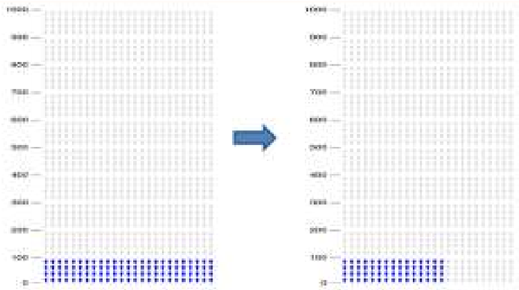

No treatment: 10% risk

Taking the medicine: 6% risk

Does the medicine have side effects?

The potential for side effects from the medicine can be described in terms of the risk of a side effect.

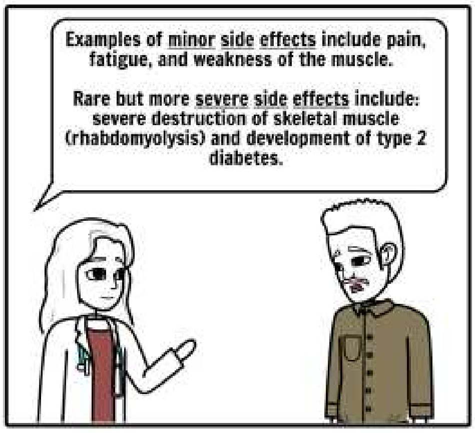

Risk of a minor side effect from the medicine

- A 5% risk of a **minor** side effect means:

Among every 1,000 people taking the medicine,

50 will experience a minor side effect such as either: pain, fatigue, and weakness of the muscle.

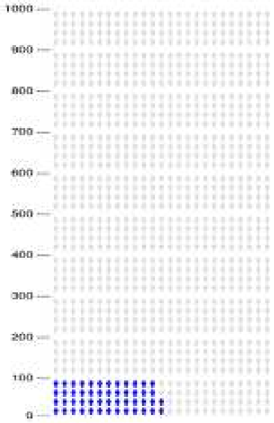

5% risk of a minor side effect

Risk of a severe side effect from the medicine

- A 0.1% risk of a **severe** side effect means:

Among every 1,000 people taking the medicine,

1 will experience a severe side effect such as either: severe destruction of skeletal muscle or development of type 2 diabetes.

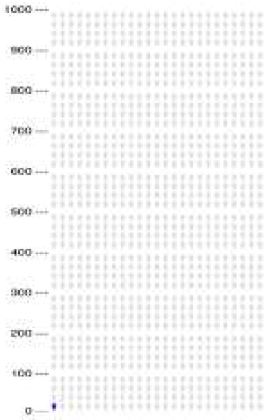

0.1% risk of a severe side effect

How inconvenient is it when taking the medicine?

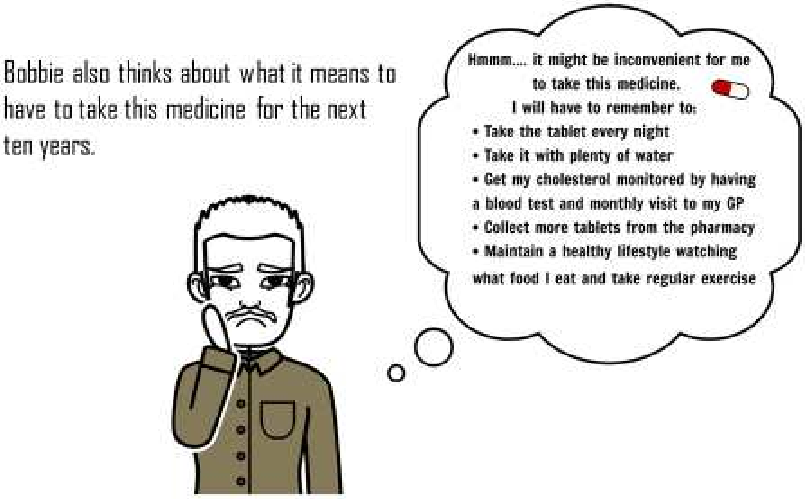

In the questions that follow in this survey, we will ask you about how you think about taking a medicine to prevent heart disease.

Supplementary Appendix 4: Training materials for bisphosphonates survey

Bisphosphonates for the prevention of osteoporotic fractures

Alex has been to see a doctor who told her that she has weak bones.

The doctor has said Alex needs to start a medicine to prevent broken bones (fractures) because of her weak bones.

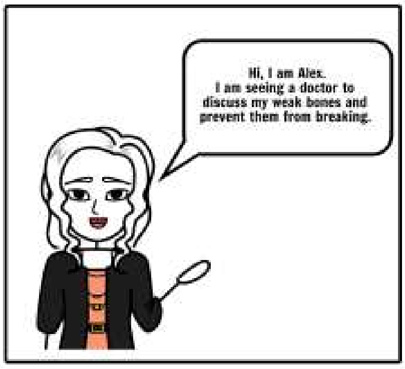

We will take you through Alex's story.

What is osteoporosis?

Alex hears about osteoporosis from her GP.

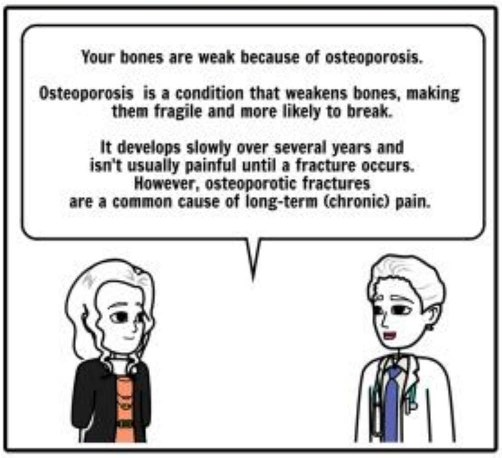

Impact of osteoporosis

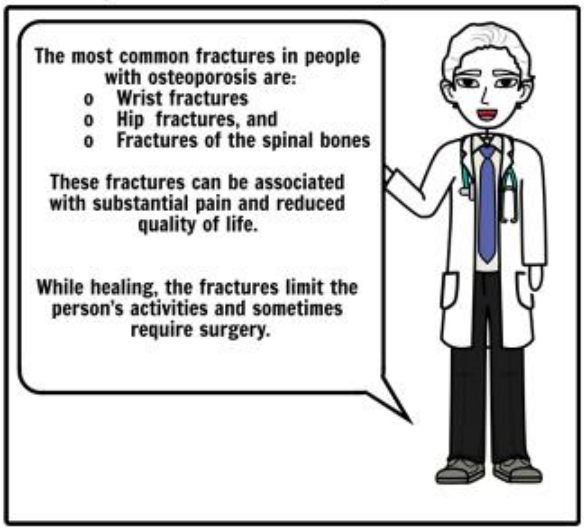

Risk of osteoporotic fractures

Her GP explains that Alex has some risk of fracture as her family has a history of hip fracture.

Also, Alex has a low body mass index (BMI). BMI is a measure of body fat.

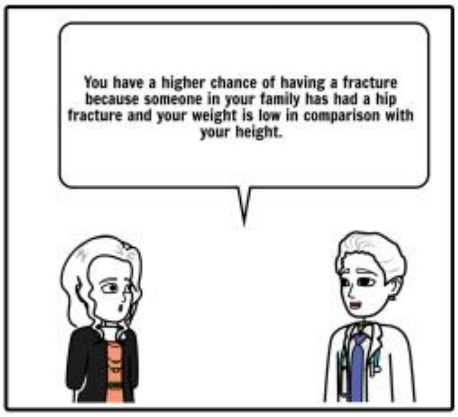

What is risk?

- **Risk** is: a term used to explain the chance that something bad might happen.

- **10% risk** means:

  Out of every **1,000** people, with osteoporosis,

  100 people would have a fracture, and **900** people would not.

What is a 10% risk?

- This diagram shows a 10% risk of having a fracture because of their osteoporosis.

- The people shaded blue are the ones who have a fracture.

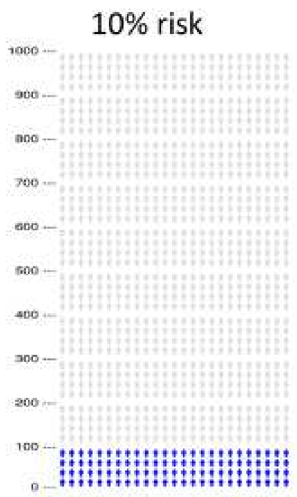

Prevention of fracture from osteoporosis

- Alex's doctor can estimate how likely it is that she will experience osteoporotic fracture over the next 5 years.
- The estimate is based on things such as Alex's age and sex, ethnicity, family fracture history, and body mass index.
- The doctor may prescribe certain medicines such as bisphosphonates to help reduce the chance of Alex having a fracture.
- The medicine will prevent some of the people who take it from having a fracture because of osteoporosis.
- Fractures may still happen in some of the people who take the medicine.

Prevention of fractures from osteoporosis

The doctor prescribes Alex a medicine to reduce the risk of her having a fracture in the future because of her osteoporosis.

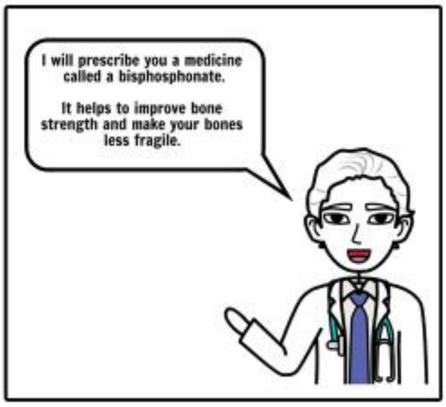

Collecting the medicine from the pharmacy

The pharmacist explains to Alex how to take the medicine.

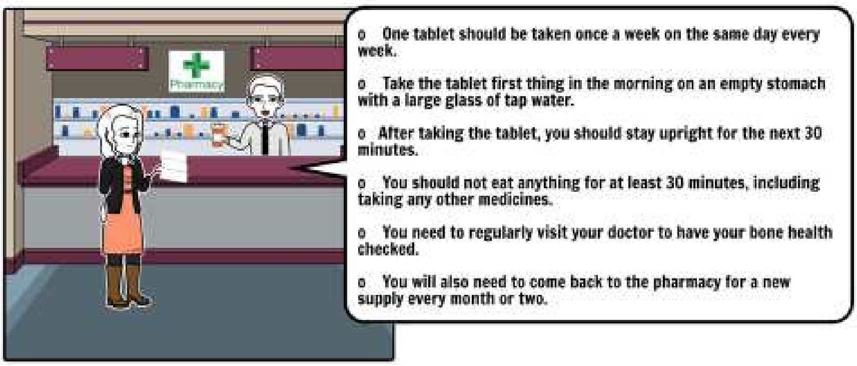

Taking the medicine for prevention

Alex goes home with her new tablets.

She then considers whether she wants to take them as the doctor and the pharmacist have advised.

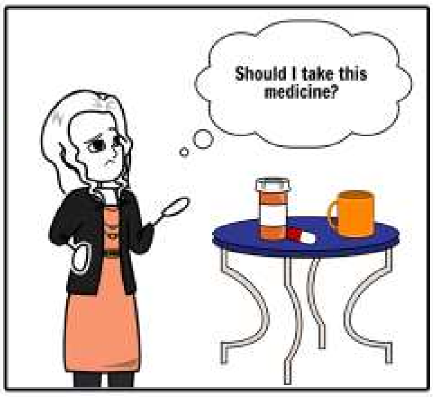

Alex may consider *three key things,*
when deciding whether to take the medicine.

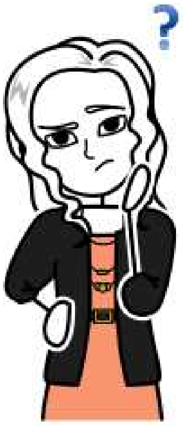

Effectiveness of the medicine

- How good the medicine is in terms of preventing a fracture from osteoporosis

Side effects of the medicine

- Whether there is any potential harm from the medicine

Inconvenience of the medicine

- Whether taking the medicine would fit in her lifestyle or cause her inconvenience

How effective is the medicine?

- Some people, but not everyone, who take the medicine will avoid a serious event, such as hip fracture, as a result of taking the tablet.

- The effectiveness of the medicine can be described in terms of a reduction in this risk.

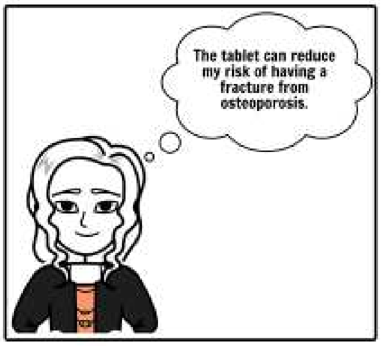

What does 'reduction in risk' mean?

- A 'reduction in risk from 10% to 6%' means:

The medicine will reduce your risk of experiencing a fracture, such as hip fracture, within the next 5 years from 10% to 6%.

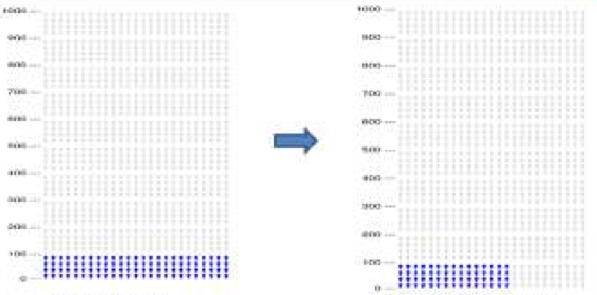

No treatment: 10% risk

Taking the medicine: 6% risk

Does the medicine have side effects?

The potential for side effects from the medicine can be described in terms of the **risk of a side effect**.

Alex hears about minor but more common side effects & severe but rarer side effects from her GP.

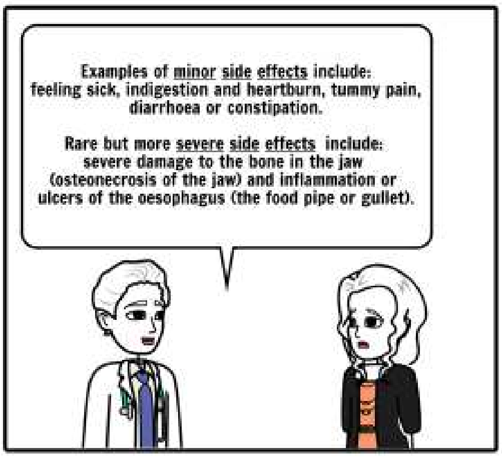

Risk of a minor side effect from the medicine

- A 5% risk of a minor side effect means:

Among every **1,000** people taking the medicine,

50 will experience a minor side effect such as either: feeling sick, indigestion, diarrhoea or constipation.

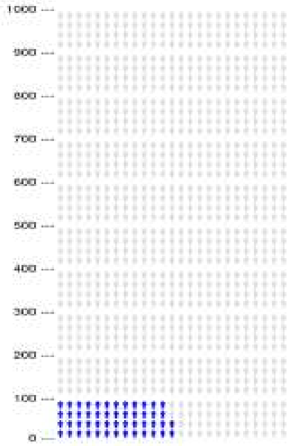

5% risk of a side effect

Risk of a severe side effect from the medicine

- A 0.3% risk of severe side effect means:

Among every **1,000** people taking the medicine,

3 will experience a severe side effect such as severe loss or destruction of the bone in the jaw.

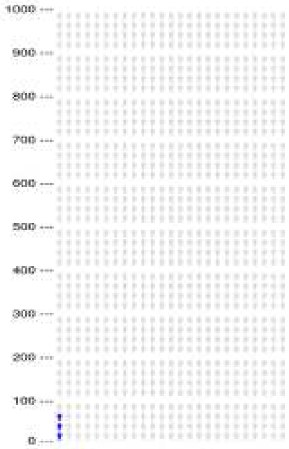

0.3% risk of a side effect

How inconvenient is it when taking the medicine?

Alex also thinks about what it means to have to take this medicine for the next 5 years.

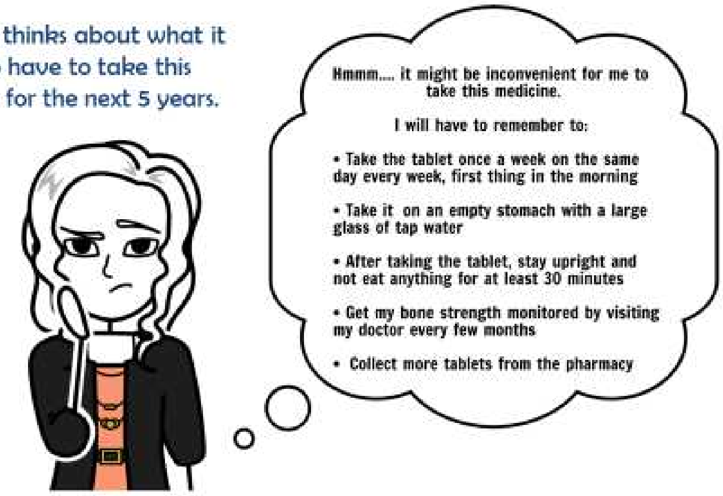

In the questions that follow in this survey,
we will ask you about how you think about taking a medicine to prevent osteoporotic fracture.

Supplementary Appendix 5: Stata ‘do’ file

```
****** ANALYSIS AND TABLES ******
/*
## ---------------------------
##
## Script name: script-01
##
## Purpose of script: Analyse data from DTD study
##
## Author: Dr. Alex Thompson
##
## Date Created: 07/08/2021
##
##
## Email: alexander.thompson@manchester.ac.uk
##
## ---------------------------
##
## Notes:
##
##
## ---------------------------
*/

clear all                                   // clears memory of everything
global location 1                           //
set cformat %5.3f                           // controls the output of tables so coefficients are rounded.

if ${location}==1 global cf `"xxxxxxxxxx"'

cd "${cf}"                                  // changes the working directory

** Location settings **
if ${location}==1 global sf `"xxxxxxx"'
if ${location}==1 global af `"xxxxxxx"'
if ${location}==1 global gf `"xxxxxxx"'
if ${location}==1 global cf `"xxxxxxx"'

global data           `"${sf}"'
global combined       `"${cf}"'
global save           `"${af}"'
global output         `"${gf}"'

cd "${combined}"

*** Prepare data ***

use statins.dta, clear

append using bisphos.dta, force
gen id = _n

gen patient = 1 if survey ==2 | survey==3
replace patient = 0 if patient==.

gen public = 1 if survey==1
replace public = 0 if public==.

drop if prescribed ==2 & patient==1         // People in 'patient population' not prescribed drugs by doctor

bysort id : gen howmany=_N

save combined.dta, replace
```

```
gen one = 1 if utility_q1==1

replace one = 0 if utility_q1!=1

gen zero = 1 if utility_q1==0.5

replace zero = 0 if utility_q1!=0.5

sum zero

save combined.dta, replace

***********************
*** TABLE 1 OUTPUTS ***
***********************


baselinetable	age sex ethnicity employment_status education1 religion ///
			prescribed other_medicines ///
			eq5d3l_u(cts) attitude  inconvenient opinion ///
			 other_pill_number other_pill_times if statins ==1  ///
			, by(public , totalcolumn) ///
			pcformat(%5.1f) meanformat(%5.3f) sdformat(%5.1f)  ///
			exportexcel("${output}/sumstats_statins.xls", replace) reportmissing

baselinetable	age sex ethnicity employment_status education1 religion ///
			prescribed other_medicines ///
			eq5d3l_u(cts) attitude  inconvenient opinion ///
			 other_pill_number other_pill_times if statins ==0  ///
			, by(public , totalcolumn) ///
			pcformat(%5.1f) meanformat(%5.3f) sdformat(%5.1f)  ///
			exportexcel("${output}/sumstats_bisphos.xls", replace) reportmissing

baselinetable	age sex ethnicity employment_status education1 religion ///
			prescribed other_medicines ///
			eq5d3l_u(cts) attitude  inconvenient opinion ///
			 other_pill_number other_pill_times if statins ==1  ///
			, by(public , totalcolumn) ///
			pcformat(%5.1f) meanformat(%5.3f) sdformat(%5.1f)  ///
			exportexcel("${output}/sumstats_statins_cat.xls", replace) reportmissing catvartab("#")

baselinetable	age sex ethnicity employment_status education1 religion ///
			prescribed other_medicines ///
			eq5d3l_u(cts) attitude  inconvenient opinion ///
			 other_pill_number other_pill_times  if statins ==0 ///
			, by(public , totalcolumn) ///
			pcformat(%5.1f) meanformat(%5.3f) sdformat(%5.1f)  ///
			exportexcel("${output}/sumstats_bisphos_cat.xls", replace) reportmissing  catvartab("#")


*****************************************************************************************************

***********************
*** Analysis 1 OUTPUTS ***
***********************


use combined.dta,clear

reshape long utility_q, i(id) j(question)

gen nontraders_half = 1 if utility_q==.5

drop  if utility_q==.5
```

```
ice  ${ice_set_cluster}  , m(5) ///
                        saving("${combined}/imputed_cluster.dta",replace) ///
                        genmiss(M)  ///
                                                                          cmd(  age:  ologit,  opinion:  ologit,  ethnicity:  ologit,
employment_status: ologit, education1: mlogit, attitude: ologit, ) match persist


qui use "${combined}/imputed_cluster.dta", clear

qui mi import ice, imputed(${ice_set_cluster}) clear

replace utility_q = 1-utility_q

***********************
*** Figure 1  ***
***********************
twoway (kdensity utility_q if statins==1 & _mi_m==0 & question==1 & patient==1 ) (kdensity utility_q if statins==1 & _mi_m==0
& question==2) (kdensity utility_q if statins==1 & _mi_m==0 & question==3) (kdensity utility_q if statins==1 & _mi_m==0 &
question==4),   ///
legend(label(1 "Q1 No side effect assumed") label(2 "Q2 Some minor side effect assumed") label(3 "Q3 Some severe side effect
assumed") label(4 "Q4 Reduced effectiveness assumed")  size(vsmall) just(center) ) ///
ytitle("Density" " ", size(small)) ///
xtitle(" " "Utility", size(small)) ///
name(kdensity_statins1,replace) ///
xlabel(0(0.1)0.5,  labsize(small)) ///
ylabel(0(5)30,  labsize(small) angle(horizontal) nogrid) ///
graphregion(fcolor(white) lwidth(large)) bgcolor(white) title("Statin Q1-Q4 utility values in patients",size(small) color(black)) ///
ysize(1) xsize(1) saving(kdensity_statins1,replace) scale(.9)

twoway (kdensity utility_q if statins==1 & _mi_m==0 & question==1 & patient==0 ) (kdensity utility_q if statins==1 & _mi_m==0
& question==2) (kdensity utility_q if statins==1 & _mi_m==0 & question==3) (kdensity utility_q if statins==1 & _mi_m==0 &
question==4),   ///
legend(label(1 "Q1 No side effect assumed") label(2 "Q2 Some minor side effect assumed") label(3 "Q3 Some severe side effect
assumed") label(4 "Q4 Reduced effectiveness assumed")  size(vsmall) just(center) ) ///
ytitle("Density" " ", size(small)) ///
xtitle(" " "Utility", size(small)) ///
name(kdensity_statins2,replace) ///
xlabel(0(0.1)0.5,  labsize(small)) ///
ylabel(0(5)30,  labsize(small) angle(horizontal) nogrid) ///
graphregion(fcolor(white) lwidth(large)) bgcolor(white) title("Statin Q1-Q4 utility values in the public",size(small) color(black)) ///
ysize(1) xsize(1) saving(kdensity_statins2,replace) scale(.9)

twoway (kdensity utility_q if statins==0 & _mi_m==0 & question==0 & patient==1 ) (kdensity utility_q if statins==0 & _mi_m==0
& question==2) (kdensity utility_q if statins==0 & _mi_m==0 & question==3) (kdensity utility_q if statins==0 & _mi_m==0 &
question==4),   ///
legend(legend(label(1 "Q1 No side effect assumed") label(2 "Q2 Some minor side effect assumed") label(3 "Q3 Some severe side
effect assumed") label(4 "Q4 Reduced effectiveness assumed")  size(vsmall) just(center) ) ///
label(1 "No side effect ") label(2 "Some minor side effect assumed") label(3 "Some severe side effect assumed") label(4 "Reduced
effectiveness assumed")  size(vsmall) just(center) ) ///
ytitle("Density" " ", size(small)) ///
xtitle(" " "Utility", size(small)) ///
name(kdensity_bisphos1,replace) ///
xlabel(0(0.1)0.5,  labsize(small)) ///
ylabel(0(2)10,  labsize(small) angle(horizontal) nogrid) ///
graphregion(fcolor(white)  lwidth(large))  bgcolor(white)  title("Bisphosphonates  Q1-Q4  utility  values  in  patients",size(small)
color(black)) ///
ysize(1) xsize(1) saving(kdensity_bisphos,replace) scale(.9)

twoway (kdensity utility_q if statins==0 & _mi_m==0 & question==0 & patient==0 ) (kdensity utility_q if statins==0 & _mi_m==0
& question==2) (kdensity utility_q if statins==0 & _mi_m==0 & question==3) (kdensity utility_q if statins==0 & _mi_m==0 &
question==4),   ///
legend(label(1 "Q1 No side effect assumed") label(2 "Q2 Some minor side effect assumed") label(3 "Q3 Some severe side effect
assumed") label(4 "Q4 Reduced effectiveness assumed")  size(vsmall) just(center) ) ///
ytitle("Density" " ", size(small)) ///
xtitle(" " "Utility", size(small)) ///
```

```
name(kdensity_bisphos2,replace) ///
xlabel(0(0.1)0.5,  labsize(small)) ///
ylabel(0(2)10,  labsize(small) angle(horizontal) nogrid) ///
graphregion(fcolor(white) lwidth(large)) bgcolor(white) title("Bisphosphonates Q1-Q4 utility values in the public",size(small) color(black)) ///
ysize(1) xsize(1) saving(kdensity_bisphos,replace) scale(.9)

grc1leg kdensity_statins1 kdensity_statins2 kdensity_bisphos1  kdensity_bisphos2 , scheme(s1manual) name(g1, replace) rows(2)
graph display g1, xsize(7) ysize(8)
graph export "${output}/kdensity1.tif",  replace width(2000)

***********************
*** Figure 1 alternative ***
***********************
replace utility_q = 1-utility_q

twoway (kdensity utility_q if statins==1 & _mi_m==0 & question==1 & patient==1 ) (kdensity utility_q if statins==1 & _mi_m==0 & question==2) (kdensity utility_q if statins==1 & _mi_m==0 & question==3) (kdensity utility_q if statins==1 & _mi_m==0 & question==4),   ///
legend(label(1 "Q1 No side effect") label(2 "Q2 Some minor side effect") label(3 "Q3 Some severe side effect") label(4 "Q4 Reduced effectiveness")  size(vsmall) just(center) ) ///
ytitle("Density" " ", size(small)) ///
xtitle(" " "Utility", size(small)) ///
name(kdensity_statins1a,replace) ///
xlabel(0.5(0.1)1,  labsize(small)) ///
ylabel(0(5)30,  labsize(small) angle(horizontal) nogrid) ///
graphregion(fcolor(white) lwidth(large)) bgcolor(white) title("Statin Q1-Q4 utility values in patients",size(small) color(black)) ///
ysize(1) xsize(1) saving(kdensity_statins1,replace) scale(.9)

twoway (kdensity utility_q if statins==1 & _mi_m==0 & question==1 & patient==0 ) (kdensity utility_q if statins==1 & _mi_m==0 & question==2) (kdensity utility_q if statins==1 & _mi_m==0 & question==3) (kdensity utility_q if statins==1 & _mi_m==0 & question==4),   ///
legend(label(1 "Q1 No side effect") label(2 "Q2 Some minor side effect") label(3 "Q3 Some severe side effect") label(4 "Q4 Reduced effectiveness")  size(vsmall) just(center) ) ///
ytitle("Density" " ", size(small)) ///
xtitle(" " "Utility", size(small)) ///
name(kdensity_statins2a,replace) ///
xlabel(0.5(0.1)1,  labsize(small)) ///
ylabel(0(5)30,  labsize(small) angle(horizontal) nogrid) ///
graphregion(fcolor(white) lwidth(large)) bgcolor(white) title("Statin Q1-Q4 utility values in the public",size(small) color(black)) ///
ysize(1) xsize(1) saving(kdensity_statins2,replace) scale(.9)

twoway (kdensity utility_q if statins==0 & _mi_m==0 & question==0 & patient==1 ) (kdensity utility_q if statins==0 & _mi_m==0 & question==2) (kdensity utility_q if statins==0 & _mi_m==0 & question==3) (kdensity utility_q if statins==0 & _mi_m==0 & question==4),   ///
legend(label(1 "Q1 No side effect") label(2 "Q2 Some minor side effect") label(3 "Q3 Some severe side effect") label(4 "Q4 Reduced effectiveness")  size(vsmall) just(center) ) ///
ytitle("Density" " ", size(small)) ///
xtitle(" " "Utility", size(small)) ///
name(kdensity_bisphos1a,replace) ///
xlabel(0.5(0.1)1,  labsize(small)) ///
ylabel(0(2)10,  labsize(small) angle(horizontal) nogrid) ///
graphregion(fcolor(white) lwidth(large)) bgcolor(white) title("Bisphosphonates Q1-Q4 utility values in patients",size(small) color(black)) ///
ysize(1) xsize(1) saving(kdensity_bisphos,replace) scale(.9)

twoway (kdensity utility_q if statins==0 & _mi_m==0 & question==0 & patient==0 ) (kdensity utility_q if statins==0 & _mi_m==0 & question==2) (kdensity utility_q if statins==0 & _mi_m==0 & question==3) (kdensity utility_q if statins==0 & _mi_m==0 & question==4),   ///
legend(label(1 "Q1 No side effect") label(2 "Q2 Some minor side effect") label(3 "Q3 Some severe side effect") label(4 "Q4 Reduced effectiveness")  size(vsmall) just(center) ) ///
ytitle("Density" " ", size(small)) ///
xtitle(" " "Utility", size(small)) ///
name(kdensity_bisphos2a,replace) ///
xlabel(0.5(0.1)1,  labsize(small)) ///
```

```
ylabel(0(2)10,  labsize(small) angle(horizontal) nogrid) ///
graphregion(fcolor(white)  lwidth(large))  bgcolor(white)  title("Bisphosphonates  Q1-Q4  utility  values  in  the  public",size(small) color(black)) ///
ysize(1) xsize(1) saving(kdensity_bisphos,replace) scale(.9)

grc1leg kdensity_statins1a kdensity_statins2a kdensity_bisphos1a  kdensity_bisphos2a                          ,            scheme(s1manual) name(g2, replace) rows(2)
graph display g2, xsize(7) ysize(8)
graph export "${output}/kdensity2.tif",  replace width(2000)


**********************
*** TABLE 2 OUTPUTS ***
**********************

gen str coding = ""
replace coding = "s1_p1_q1" if statins==1 & _mi_m==0 & question==1 & patient==1,
replace coding = "s1_p1_q2" if statins==1 & _mi_m==0 & question==2 & patient==1,
replace coding = "s1_p1_q3" if statins==1 & _mi_m==0 & question==3 & patient==1,
replace coding = "s1_p1_q4" if statins==1 & _mi_m==0 & question==4 & patient==1,
replace coding = "s1_p0_q1" if statins==1 & _mi_m==0 & question==1 & patient==0,
replace coding = "s1_p0_q2" if statins==1 & _mi_m==0 & question==2 & patient==0,
replace coding = "s1_p0_q3" if statins==1 & _mi_m==0 & question==3 & patient==0,
replace coding = "s1_p0_q4" if statins==1 & _mi_m==0 & question==4 & patient==0,
replace coding = "s0_p1_q1" if statins==0 & _mi_m==0 & question==1 & patient==1,
replace coding = "s0_p1_q2" if statins==0 & _mi_m==0 & question==2 & patient==1,
replace coding = "s0_p1_q3" if statins==0 & _mi_m==0 & question==3 & patient==1,
replace coding = "s0_p1_q4" if statins==0 & _mi_m==0 & question==4 & patient==1,
replace coding = "s0_p0_q1" if statins==0 & _mi_m==0 & question==1 & patient==0,
replace coding = "s0_p0_q2" if statins==0 & _mi_m==0 & question==2 & patient==0,
replace coding = "s0_p0_q3" if statins==0 & _mi_m==0 & question==3 & patient==0,
replace coding = "s0_p0_q4" if statins==0 & _mi_m==0 & question==4 & patient==0,

estpost tabstat utility_q , statistics(mean sd count skewness kurtosis p10 p25 p50 p75 p90) casewise by(coding)
esttab . using "${output}/table_tabstat.rtf", cells("mean sd count skewness kurtosis p10 p25 p50 p75 p90") noobs

gen str coding2 = ""

replace coding2 = "s1_p1" if statins==1 & _mi_m==0 & patient==1,
replace coding2 = "s1_p0" if statins==1 & _mi_m==0 & patient==0,
replace coding2 = "s0_p1" if statins==0 & _mi_m==0 & patient==1,
replace coding2 = "s0_p0" if statins==0 & _mi_m==0 & patient==0,

estpost tabstat utility_q , statistics(mean sd count skewness kurtosis p10 p25 p50 p75 p90) casewise by(coding2)

esttab . using "${output}/table_tabstat2.rtf", cells("mean sd count skewness kurtosis p10 p25 p50 p75 p90") noobs

gen str coding3 = ""
replace coding3 = "s1" if statins==1 & _mi_m==0
replace coding3 = "s0" if statins==0 & _mi_m==0

estpost tabstat utility_q , statistics(mean sd count skewness kurtosis p10 p25 p50 p75 p90) casewise by(coding3)

esttab . using "${output}/table_tabstat3.rtf", replace cells("mean sd count skewness kurtosis p10 p25 p50 p75 p90") noobs

gen trader = 1 if utility_q!=1 & utility_q!=0.5
replace trader = 0 if trader==.

estpost tabstat trader , statistics(mean sd count skewness kurtosis p10 p25 p50 p75 p90) casewise by(coding)
estpost tabstat trader , statistics(mean sd count skewness kurtosis p10 p25 p50 p75 p90) casewise by(coding2)
estpost tabstat trader , statistics(mean sd count skewness kurtosis p10 p25 p50 p75 p90) casewise by(coding3)

estpost tabstat trader if _mi_m==0 , statistics(mean sd count skewness kurtosis p10 p25 p50 p75 p90) casewise by(statins)

**********************
*** TABLE 3 OUTPUTS ***
```

```
**********************

mi estimate, post: reg utility_q i.sex i.ethnicity i.age i.other_pill_times i.other_pill_number eq5d_index question_2 question_3 question_4 statins public if statins == 1
estimates store m1

mi estimate, post: reg utility_q i.sex i.ethnicity i.age i.other_pill_times i.other_pill_number eq5d_index question_2 question_3 question_4 statins public if statins == 0
estimates store m2

gen yes = 1 if utility_q==1
replace yes = 0 if utility_q<1

mi estimate, saving(combined_logs,replace): logistic yes i.sex i.ethnicity i.age i.other_pill_times i.other_pill_number eq5d_index question_2 question_3 question_4 statins public
estimates store m3


esttab m1 m2 using "${output}/table_all_new.rtf" , ar2 label not replace ci
esttab m3 using "${output}/table_all_logist_new.rtf" , ar2 label not replace ci

mi test question_2 question_3 question_4
/*
. mi test question_2 question_3 question_4
note: assuming equal fractions of missing information

 ( 1)  question_2 = 0
 ( 2)  question_3 = 0
 ( 3)  question_4 = 0

       F(  3, 149.7) =    0.15
            Prob > F =    0.9310

.
*/


****************************************************
*** Appendix analysis 6 ***
****************************************************
**Propensity for dominant preferences**

gen yes = 1 if utility_q==1
replace yes = 0 if utility_q<1

gen no = 1 if utility_q==0.5
replace no = 0 if no==.

la var yes "Selected 1 for DTD"

mi estimate, post: logistic yes sex i.ethnicity i.education1 i.age public statins
estimates store m5


mi estimate, post: logistic no sex i.ethnicity i.education1 i.age public statins
estimates store m6

esttab m5 m6


qui use "${combined}/imputed_cluster.dta", clear

****************************************************
*** Appendix analysis 7 ***
```

```
****************************************************

************STATINS*****************

qui mi import ice, imputed(${ice_set_cluster}) clear
forvalues i =1/5 {
*ssc install zoib //install zero-one-inflated beta regression package
zoib utility_q   sex i.ethnicity i.education1 i.age i.question public i.other_pill_times i.other_pill_number  if _mi_m==1 & statins==1
predict beta_`i'

betamix utility_q      if  _mi_m==1  &  statins==1,  muvar(sex  i.ethnicity  i.education1  i.age  i.question  public  i.other_pill_times
i.other_pill_number ) lbound(0.5) ubound(1) pmass(1 0 1)  //zero and one inflated, including all values
predict mixedbeta_`i'

reg utility_q                              sex   i.ethnicity   i.education1   i.age   i.question     public   i.other_pill_times   i.other_pill_number     if
_mi_m==`i' & statins==1
predict yhat_`i'

}

mi estimate, saving(statin_ols,replace):                                   reg  utility_q   i.sex  i.ethnicity  i.education1  i.age  i.question    public
i.other_pill_times i.other_pill_number if statins==1

forvalues i =1/5 {
rmse utility_q yhat_`i'                              if _mi_m==`i'
rmse utility_q beta_`i'                              if _mi_m==`i'
rmse utility_q mixedbeta_`i'      if _mi_m==`i'

}

************BISPHOSPHONATES*****************
qui use "${combined}/imputed_cluster.dta", clear

qui mi import ice, imputed(${ice_set_cluster}) clear
forvalues i =1/5 {
*ssc install zoib //install zero-one-inflated beta regression package
zoib utility_q   sex i.ethnicity i.education1 i.age i.question public i.other_pill_times i.other_pill_number  if _mi_m==1 & statins==0
predict beta_`i'

betamix utility_q      if  _mi_m==1  &  statins==0,  muvar(sex  i.ethnicity  i.education1  i.age  i.question  public  i.other_pill_times
i.other_pill_number ) lbound(0.5) ubound(1) pmass(1 0 1)  //zero and one inflated, including all values
predict mixedbeta_`i'

reg utility_q                              sex   i.ethnicity   i.education1   i.age   i.question     public   i.other_pill_times   i.other_pill_number   if
_mi_m==`i' & statins==0
predict yhat_`i'

}

mi estimate, saving(statin_ols,replace):                                   reg  utility_q   i.sex  i.ethnicity  i.education1  i.age  i.question    public
i.other_pill_times i.other_pill_number if statins==0

forvalues i =1/5 {
rmse utility_q yhat_`i'                              if _mi_m==`i'
*gen ols_rmse_`i'  = r(yhat_`i')  if _mi_m==`i'
rmse utility_q beta_`i'                              if _mi_m==`i'
rmse utility_q mixedbeta_`i'      if _mi_m==`i'

}

forvalues i =1/5 {
mean yhat_`i' if _mi_m==`i'                    & statins==0
mean beta_`i' if _mi_m==`i'                    & statins==0
mean mixedbeta_`i' if _mi_m==`i'            & statins==0

}
```

*** Model fit check ***

```
twoway ( scatteri 0.5 0.5 1 1, recast(line) lcolor(gray) lwidth(medthin) lpattern(solid) ) ///
( scatter yhat_1 utility_q  if _mi_m==1 , mcolor(red)  msize(small) msymbol(circle) msymbol(circle_hollow)  ) ( scatter beta_1 utility_q if _mi_m==1 , mcolor(black)  msize(small) msymbol(circle) msymbol(circle_hollow)) ( scatter mixedbeta_1 utility_q  if _mi_m==1 , mcolor(green)  msize(small) msymbol(circle) msymbol(circle_hollow)), ///
 xlabel(0.5(0.25)1,nogrid  labsize(small))  ylabel(0.5(0.25)1,nogrid  labsize(small))  graphregion(  color(white)  )  legend(off)  yscale( nofextend ) xscale(nofextend) ///
ytitle("EQ-5D-3L Predicted JHC", size(small)) xtitle("EQ-5D-3L Observed JHC", size(small)) title(`"`vtext'"', size(small) color(black)) ///
xmtick( 0.5(0.25)1 , grid glcolor(gray) glpattern(dash) glwidth(vthin) nogextend ) ///
ymtick( 0.5(0.25)1 , grid glcolor(gray) glpattern(dash) glwidth(vthin) nogextend ) xsize(1) ysize(1)


twoway (kdensity x if statins==1) (kdensity x if statins==0) (kdensity y if statins==1) (kdensity y if statins==0)  (kdensity yhat if statins==1) (kdensity yhat if statins==0)
```

Supplementary Appendix 6: Description of the whole sample characteristics

	**Statin survey**			**Bisphosphonate survey**			
	Patient[a] N=260	Public[b] N=376	Total N=636	Patient[a] N=110	Public[b] N=359	Total N=469	Total N= 1105
**Age**							
Less than 35	2 (1.2%)	25 (7.9%)	27 (5.5%)	0 (0.0%)	26 (8.8%)	26 (7.5%)	53 (6%)
35-44	5 (2.9%)	74 (23.3%)	79 (16.2%)	1 (1.9%)	51 (17.3%)	52 (14.9%)	131 (16%)
45-54	10 (5.9%)	57 (17.9%)	67 (13.7%)	3 (5.7%)	41 (13.9%)	44 (12.6%)	111 (13%)
55-64	49 (28.8%)	84 (26.4%)	133 (27.3%)	14 (26.4%)	57 (19.3%)	71 (20.4%)	204 (24%)
65-74	77 (45.3%)	70 (22.0%)	147 (30.1%)	20 (37.7%)	107 (36.3%)	127 (36.5%)	274 (33%)
75+	27 (15.9%)	8 (2.5%)	35 (7.2%)	15 (28.3%)	13 (4.4%)	28 (8.0%)	63 (8%)
Missing	90	58	148	57	64	121	269
**Sex**							
Female	57 (33.5%)	159 (50.0%)	216 (44.3%)	46 (86.8%)	185 (62.9%)	231 (66.6%)	447 (54%)
Male	113 (66.5%)	159 (50.0%)	272 (55.7%)	7 (13.2%)	109 (37.1%)	116 (33.4%)	388 (46%)
Missing	90	58	148	57	65	122	270
**Ethnicity**							
White British/Irish	160 (94.1%)	288 (90.6%)	448 (91.8%)	51 (96.2%)	263 (89.2%)	314 (90.2%)	762 (91%)
White other	4 (2.4%)	16 (5.0%)	20 (4.1%)	1 (1.9%)	12 (4.1%)	13 (3.7%)	33 (4%)
Mixed/Multiple ethnic origins	0 (0.0%)	2 (0.6%)	2 (0.4%)	0 (0.0%)	5 (1.7%)	5 (1.4%)	7 (1%)
Black/African/Caribbean/Black British	0 (0.0%)	3 (0.9%)	3 (0.6%)	0 (0.0%)	4 (1.4%)	4 (1.1%)	7 (1%)
Asian/Asian British	0 (0.0%)	7 (2.2%)	7 (1.4%)	0 (0.0%)	9 (3.1%)	9 (2.6%)	16 (2%)
Chinese	0 (0.0%)	1 (0.3%)	1 (0.2%)	1 (1.9%)	2 (0.7%)	3 (0.9%)	4 (%)
Other ethnicity	6 (3.5%)	1 (0.3%)	7 (1.4%)	0 (0%)	0 (0%)	0 (0%)	(%)
Missing	90	58	148	57	64	121	269
**Number of pills taken daily**							
None	0 (0.0%)	133 (41.8%)	133 (27.3%)	0 (0.0%)	99 (33.6%)	99 (28.4%)	232 (28%)
One	4 (2.4%)	73 (23.0%)	77 (15.8%)	6 (11.3%)	51 (17.3%)	57 (16.4%)	134 (16%)
Two to five	123 (72.4%)	87 (27.4%)	210 (43.0%)	32 (60.4%)	109 (36.9%)	141 (40.5%)	351 (42%)
Six to ten	36 (21.2%)	18 (5.7%)	54 (11.1%)	9 (17.0%)	23 (7.8%)	32 (9.2%)	86 (10%)
More than ten	7 (4.1%)	7 (2.2%)	14 (2.9%)	6 (11.3%)	13 (4.4%)	19 (5.5%)	33 (4%)
Missing	90	58	148	57	64	121	269
**Number of different times pill taken per day**							
None	3 (1.8%)	134 (42.1%)	137 (28.1%)	0 (0.0%)	97 (32.9%)	97 (27.9%)	234 (28%)
Once per day	40 (23.5%)	104 (32.7%)	144 (29.5%)	22 (41.5%)	100 (33.9%)	122 (35.1%)	266 (32%)
2 times a day	100 (58.8%)	61 (19.2%)	161 (33.0%)	20 (37.7%)	68 (23.1%)	88 (25.3%)	249 (30%)
3 times a day	23 (13.5%)	13 (4.1%)	36 (7.4%)	7 (13.2%)	24 (8.1%)	31 (8.9%)	67 (8%)
More than 3 times a day	4 (2.4%)	6 (1.9%)	10 (2.0%)	4 (7.5%)	6 (2.0%)	10 (2.9%)	20 (2%)
Missing	90	58	148	57	64	121	269
**EQ-5D-3L utility[c]**	0.820 (0.2)	0.803 (0.3)	0.810 (0.3)	0.771 (0.2)	0.790 (0.2)	0.786 (0.2)	0.800 (0.3)
Missing	41	38	79	35	41	76	155

Footnote: [a] patient sample was recruited from general practitioners in the NHS Research Scotland Primary Care Network or the Scottish Health Research Register (SHARE); [b]public sample was recruited from Dynata; [c]Health status measured using the EQ-5D-3 level and transformed into a utility score

Supplementary Appendix 7: Propensity for dominant preference

Male	0.988
	[0.716, 1.364]
White other	1.263
	[0.594,2.684]
Mixed/Multiple ethnic	0.370
	[0.043,3.171]
Black/African/Caribbean/Bla	0.903
	[0.156,5.244]
Asian/Asian British	1.230
	[0.329, 4.604]
Chinese	0.748
	[0.077, 7.290]
Other ethnicity	0.568
	[0.066, 4.902]
Less than 35	0
	[.,.]
35-44	0.709
	[0.377, 1.333]
45-54	0.521
	[0.265, 1.023]
55-64	0.507
	[0.262,0.978]
65-74	0.480
	[0.2540, 0.907]
75+	0.962
	[0.399, 2.318]
Public	1.544*
	[1.047, 2.278]
None	0
	[.,.]
Once per day	1.092
	[0.600,1.986]
2 times a day	0.860
	[0.417,1.775]
3 times a day	1.077
	[0.427,2.715]
More than 3 times a day	2.797
	[0.819,9.554]
None	0
	[.,.]
One	1.0211
	[0.507,2.055]
Two to five	0.875
	[0.446,1.716]
Six to ten	1.012
	[0.386,2.654]
More than ten	1.263
	[0.451,3.536]
Statin sample	0.927
	[0.678,1.268]
Constant	0.367*
	[0.135,1.00]
Observations	1105

Supplementary Appendix 8: Root mean squared error (RMSE) for the competing models to fit TTO data

	Statins			Bisphosphonates		
Imputation number	OLS[1]	ZOIB[2]	MBR[3]	OLS[1]	ZOIB[2]	MBR[3]
Imputation 1	0.0692	0.0694	0.0699	0.0706	0.0719	0.0716
Imputation 2	0.0697	0.0703	0.0695	0.0717	0.0712	0.0718
Imputation 3	0.0693	0.0709	0.0715	0.0718	0.0721	0.0717
Imputation 4	0.0697	0.0705	0.0721	0.0706	0.0722	0.0733
Imputation 5	0.0698	0.0688	0.0717	0.0717	0.0693	0.0734
Mean	0.0695	0.0700	0.0709	0.0713	0.0713	0.0723

[1] Ordinary least squares; [2] Zero inflated beta regression; [3] Mixed beta regression
