# Supplementary material for: Quantifying the impact of taking medicines for primary prevention: a time-trade off study to elicit direct treatment disutility in the UK
Source: BMJ Open. 2023 Sep 21;13(9):e063800. doi: 10.1136/bmjopen-2022-063800 (PMC10514632; doi:10.1136/bmjopen-2022-063800)
Supplement: Author's manuscript [file bmjopen-2022-063800.draft_revisions.pdf]

BMJ Open

BMJ Open is committed to open peer review. As part of this commitment we make the peer review history of every article we publish publicly available.

When an article is published we post the peer reviewers' comments and the authors' responses online. We also post the versions of the paper that were used during peer review. These are the versions that the peer review comments apply to.

The versions of the paper that follow are the versions that were submitted during the peer review process. They are not the versions of record or the final published versions. They should not be cited or distributed as the published version of this manuscript.

BMJ Open is an open access journal and the full, final, typeset and author-corrected version of record of the manuscript is available on our site with no access controls, subscription charges or pay-per-view fees (http://bmjopen.bmj.com).

If you have any questions on BMJ Open's open peer review process please email info.bmjopen@bmj.com

BMJ Open

Quantifying the impact of taking medicines for primary prevention: a time-trade off study to elicit direct treatment disutility

Journal:	*BMJ Open*
Manuscript ID	bmjopen-2022-063800
Article Type:	Original research
Date Submitted by the Author:	28-Apr-2022
Complete List of Authors:	Thompson, Alexander; The University of Manchester<br>Youn, Ji-Hee; The University of Manchester<br>Guthrie, Bruce; University of Edinburgh College of Medicine and Veterinary Medicine,<br>Hainsworth, Robert; The University of Manchester<br>Donnan, Peter; University of Dundee, Dundee Epidemiology and Biostatistics Unit<br>Rogers, Gabriel; The University of Manchester<br>Morales, Daniel; University of Dundee, Division of Population Health Sciences<br>Payne, Katherine; The University of Manchester, Health Economics at Manchester
Keywords:	Health economics < HEALTH SERVICES ADMINISTRATION & MANAGEMENT, Coronary heart disease < CARDIOLOGY, PREVENTIVE MEDICINE

SCHOLARONE™
Manuscripts

I, the Submitting Author has the right to grant and does grant on behalf of all authors of the Work (as defined in the below author licence), an exclusive licence and/or a non-exclusive licence for contributions from authors who are: i) UK Crown employees; ii) where BMJ has agreed a CC-BY licence shall apply, and/or iii) in accordance with the terms applicable for US Federal Government officers or employees acting as part of their official duties; on a worldwide, perpetual, irrevocable, royalty-free basis to BMJ Publishing Group Ltd ("BMJ") its licensees and where the relevant Journal is co-owned by BMJ to the co-owners of the Journal, to publish the Work in this journal and any other BMJ products and to exploit all rights, as set out in our licence.

The Submitting Author accepts and understands that any supply made under these terms is made by BMJ to the Submitting Author unless you are acting as an employee on behalf of your employer or a postgraduate student of an affiliated institution which is paying any applicable article publishing charge ("APC") for Open Access articles. Where the Submitting Author wishes to make the Work available on an Open Access basis (and intends to pay the relevant APC), the terms of reuse of such Open Access shall be governed by a Creative Commons licence – details of these licences and which Creative Commons licence will apply to this Work are set out in our licence referred to above.

Other than as permitted in any relevant BMJ Author's Self Archiving Policies, I confirm this Work has not been accepted for publication elsewhere, is not being considered for publication elsewhere and does not duplicate material already published. I confirm all authors consent to publication of this Work and authorise the granting of this licence.

For peer review only

Title: Quantifying the impact of taking medicines for primary prevention: a time-trade off study to elicit direct treatment disutility

Running title: Estimating values for direct treatment disutility

Authors:

Alexander J Thompson, PhD[1a*] (alexander.thompson@manchester.ac.uk)

Ji-Hee Youn, PhD[1a]

Bruce Guthrie, MB BChir PhD[3]

Robert Hainsworth, MSc[1]

Peter Donnan, PhD[2]

Gabriel Rogers[1]

Daniel Morales, PhD[2]

Katherine Payne, PhD[1]

[a] joint first authors

[1] *Manchester Centre for Health Economics, Division of Population Health, Health Services Research and Primary Care, University of Manchester*

[2] *Division of Population Health and Genomics, University of Dundee*

[3] *Usher Institute, College of Medicine and Veterinary Medicine, University of Edinburgh*

**Corresponding author: Alexander Thompson, Floor 4, Jean Mcfarlane Building, Oxford Road, The University of Manchester, Manchester, M13 9PL, UK*

e-mail: alexander.thompson@manchester.ac.uk

ORCiD: 0000-0003-4930-5107

Keywords: harm, long-term conditions, utility, direct treatment disutility, time trade off, primary prevention, cardiovascular disease, statins, osteoporosis, bisphosphonates

Word Count: 3442

For peer review only

Abstract

Direct treatment disutility (DTD) represents an individual's disutility associated with the inconvenience of taking a medicine over a long period of time.

Objectives: The main aim of this study was to elicit DTD values for taking a statin or a bisphosphonate for primary prevention. A secondary aim was to understand factors which influence DTD values.

Methods

Design: In this cross-sectional study, participants were invited to complete a series of time-trade off (TTO) exercises embedded in online surveys. Respondents were asked to compare a one-off pill ('Medicine A') assumed to have no inconvenience and a daily pill ('Medicine B') over ten-years (statins) or five-years (bisphosphonates). The primary outcome was direct treatment disutility.

Participants and setting: Two samples of participants were recruited. First, a purposive sample of patients with experience of taking a statin (n=260) or bisphosphonate (n=100) were recruited from NHS primary care sampling frames. Patients needed to be aged over 30, have experience of taking the medicine of interest and have no diagnosis of dementia or of using dementia drugs. Second, a demographically balanced sample of members of the public were recruited for statins (n=376) and bisphosphonates (n=359) using an online panel company.

Analysis: Regression analysis explored whether self-reported characteristics factors and survey design influenced DTD values.

Results

A total of 879 respondents were included for analysis (514 for statins and 365 for bisphosphonates).The majority of respondents reported a disutility associated with medicine use. Mean DTD for statins was 0.034 and for bisphosphonates 0.067 respectively. Respondent characteristics including age and sex did not influence DTD but experience of taking a bisphosphonate reduced reported disutilities.

Conclusion

Statins and bisphosphonates have a quantifiable direct treatment disutility. The size of estimated DTDs suggest they are likely to be important in determining cost-effectiveness, particularly in individuals at low-risk when treated for primary prevention.

For peer review only - http://bmjopen.bmj.com/site/about/guidelines.xhtml

Strength and limitations

- We use a large representative sample of both public and patients for our estimated values.
- We used patients to inform and trial our survey and designed storyboards and visual arrays to contextualize the experiment and communicate absolute risk.
- The time trade-off methods employed were consistent with those first used to derive the EQ-5D-3L, a widely used measure of health-related quality of life.
- Survey respondents self-selected and this could be related to their ability to complete the survey or their self-reported health.

For peer review only

For peer review only - http://bmjopen.bmj.com/site/about/guidelines.xhtml

1 Introduction

Medicines for the primary prevention of disease are typically taken long-term, often for life, following identification that an individual is at risk of a harmful event, such as a stroke due to cardiovascular disease [1] or a hip fracture as a result of osteoporosis [2]. Taking a medicine is considered, in general, to have a minimal day-to-day treatment burden because, in the traditional clinical sense, the action of medicine-taking is perceived to be non-invasive. Yet there is qualitative evidence to suggest that taking a medicine long-term is associated with treatment burden [3]. Moreover, supporting this qualitative evidence is a quantitative literature seeking to estimate the utility of pill-taking, or alternatively, the negative impact of undergoing long-term treatment with medicines, called 'direct treatment disutility'.

Direct treatment disutility (DTD) represents an individual's strength of preference not to take a medicine. DTD could occur for a number of reasons including the inconvenience of obtaining prescriptions and medicines, needing to modify lifestyles to take medicines and attending healthcare visits for monitoring treatment [4]. DTD therefore can be considered in addition to the potential impact adverse drug reactions could have on an individual's utility and/or the out-of-pocket costs (financial burden) medicines may incur. To date, the quantification of DTD in the literature has focused primarily on long-term medicines for cardiovascular disease with values estimated using hypothetical thought experiments such as 'time trade-off' (TTO) exercises where respondents are often asked whether they would forego some length of life not to undergo treatment [5–7]. Estimates from this literature suggest that a substantial proportion of people would be willing to trade some length of life or risk of death not to take a long-term preventative treatment [5–7].

A small number of published cost-effectiveness analyses (CEA) have included DTD values and where incorporated in analysis, DTD has been an important factor for influencing cost-effectiveness results [8–14]. However, the DTD values used have often been assumed [9,12,13] rather than based on empirical estimates. Previous empirical DTD studies, likely for reasons of practicality, have adopted non-ideal study sizes [5] or sampling frames which are either not representative of the general population[6] or patients[7], potentially limiting use as part of CEA.

For peer review only - http://bmjopen.bmj.com/site/about/guidelines.xhtml

The aim of this study was to build upon the previous empirical literature and elicit values of DTD for long-term primary preventative medicines from a mixed sample of the general population and patients. We focus on two populations who might require long-term treatment with medicines: those taking statins for the primary prevention of cardiovascular disease (CVD) and bisphosphonates for the primary prevention of bone fractures. A secondary aim of this study was to explore if survey participant characteristics were associated with the elicited DTD values.

2 Methods

Ethical approval for this study was granted by an NHS Health Research Authority Research Ethics Committee (REC reference: 17/NW/0124; project number: 220492). Respondents gave informed consent after reading a participant information sheet online and indicating they agreed to participate in the survey online.

There is no agreement on the appropriate method to elicit DTD [5–7]. This study used the TTO method for the valuation exercise [15] [16]. This method is a widely recognised standard approach for eliciting utility values and has been used to generate the published tariff of utility values used by national decision-making bodies such as the National Institute for Health and Care Excellence (NICE) [17,18] who have used the EQ-5D-3L to quantify health-related quality of life, valued using a TTO exercise.

2.1 Selection of case studies

Statins for primary prevention of CVD was selected because it is an example of an orally administered medicine that is perceived to be benign, but which some people perceive as harmful. Bisphosphonates for the primary prevention of bone fractures was selected because it is an example of a medicine that has an obvious potential impact on day-to-day life. Patients undergoing treatment with bisphosphonates must take the medication on an empty stomach, drink a full glass of water, stand for 30 minutes after taking the medication and avoid food and drink for a further 2 hours.

For peer review only - http://bmjopen.bmj.com/site/about/guidelines.xhtml

2.2 Patient and public involvement

The genesis for the concept of direct treatment disutility (DTD) was informed by previous research conducted with patients exploring the impact of medications in those with multimorbidity [19]. To quantify DTD, two patient experts contributed experience of taking medicines long-term, alongside clinical input from the research team, to develop the description of the health (medicine-taking) states used in this study. Separate online surveys were designed for the two selected case studies (see Supplementary Appendix 1 for the statin survey and Supplementary Appendix 2 for the bisphosphonate survey). Pilot studies were conducted for each survey. First, a qualitative pilot study, using the think-aloud method [20,21] from a sample of patients from a GP practice in Greater Manchester was used to understand whether the surveys were sufficiently clear for respondents to complete as intended. Second, a quantitative pilot study with 30 respondents (for statin survey and bisphosphonate survey) identified by panel company Dynata was used to allow a preliminary analysis of data collected from the valuation exercise. No changes were made following the quantitative pilot study. The final surveys were formatted and administered online using Sawtooth software [22]. Respondents were sent a secure link to complete a survey.

2.3 Study sample

Two purposive samples of patients taking a statin or bisphosphonate were recruited from two sampling frames: from general practices via the NHS Research Scotland Primary Care Network[23] and the Scottish Health Research Register (SHARE – a register of people living in Scotland allowing recruitment after a search of their medical records)[24]. For both, the inclusion criteria for recruiting patients with experience of taking a statin (bisphosphonate) were: prescription of a statin (or a bisphosphonate) in the last year; aged 30 years and over; not been diagnosed with dementia (ICD10 code: F00, F01, F02, F03, G10, G20, G30, G31.0, F05.1, R54 and all child codes); not taking a dementia drug (all drugs in British National Formulary chapter 0411); not also taking a bisphosphonate (or statin as appropriate). Patients deemed unsuitable for any reason by their general practitioner were also excluded.

For peer review only - http://bmjopen.bmj.com/site/about/guidelines.xhtml

A purposive sample of members of the public were recruited using an online panel company (Dynata) [25]. This online panel company provided a sample of respondents with pre-defined (age 30 years and over; equal gender-balance) characteristics and a demographically-balanced sample from England and Scotland. There were no exclusion criteria for members of the general public who self-select if they were capable of completing an online survey.

Sample-size calculations for valuation exercises are not well established [26]. This study aimed for a pragmatic sample size of a minimum of 500 respondents (250 patients; 250 on-line panel) for each case study. This resulted in a target total sample size of 1000 respondents (500 for the statin survey and 500 for the bisphosphonate survey).

2.4 Valuation exercise

The TTO method employed was similar in approach to that taken by Hutchins et al [6,7] when valuing the utility of pill taking. See Supplementary Appendix 1 for the whole survey format. The duration within the experiment for daily pill taking was 10-years for statins and 5-years for bisphosphonates. Respondents were asked to imagine taking a pill every day for 10-years (5-years) and then dying. Respondents were then asked whether they would be willing to trade that health state for an alternative where only a single pill was taken at the start of the time-period. In this second health state, however, the respondent would live for a shorter duration and then die. A process of 'iteration' was then used to see whether respondents would trade between the health states of differing lengths until a point of indifference occurs between the two health states. The trade-off being quantified here was whether the participant would be willing to choose to live a shorter life but without daily pill taking or alternatively, to have a one-off pill but forego some length of life. The process of iteration to find the potential indifference point followed a standardised process called the Measurement and Valuation of Health (MVH) protocol [26] which has been recommended previously to encourage comparability between utility values elicited for the purposes of health technology assessment.

For peer review only - http://bmjopen.bmj.com/site/about/guidelines.xhtml

The experiments previously described were contextualised with four different background scenarios in order to understand whether DTD values differed depending on the framing of benefits and harms of the medications being used. We asked respondents to consider the experiment with the context that there was: no side-effects (scenario 1) associated with any of the pills; mild side-effects (scenario 2); severe side-effects (scenario 3); and reduced effectiveness (scenario 4). We developed training materials using a story-board approach [26] to enable the communication of the background concepts for respondents completing each survey (see Supplementary Appendix 3 & 4).

2.5 Data analysis

Summary statistics were calculated for a master sample combining data from SPCRN and SHARE ('patient respondents') and Dynata ('public respondents') for both statin and bisphosphonate questionnaires. For each of the four questions per respondent, the estimated utility was calculated as the ratio x/t, where x is the final selected time period for the Medicine A alternative (one pill taken once) and t is the full life years assumed for the Medicine B option (a pill taken every day for 10 years / 5 years) [16]. Respondents who indicated they would be unwilling to initiate preventative therapy, by selecting five years for Medicine A (or the lowest TTO score attainable of 0.5) in any one of the questions were removed from the dataset. This was because we inferred such respondents to have dominant preferences to not undergo preventative treatment [27,28].

Missing data for background characteristics as well as for the TTO scores were multiply imputed (m=5) using chained equations with predictive mean matching [29]. Differences in respondent TTO scores associated with medicine type (statins versus bisphosphonates), framing of the survey question (question 1, question 2, question 3, question 4), background characteristics (age, ethnicity, sex) and experience of taking medication (pills taken per day, number of times medication taken per day) were explored using simple regression modelling accounting for the multiply imputed datasets. Model specification was informed by summary statistics and kernel density plots of the DTD values. Sensitivity to alternative regression models, were explored with competing models compared using root mean squared error (RMSE). Propensity to trade was explored through a logistic regression for the whole sample with a

For peer review only - http://bmjopen.bmj.com/site/about/guidelines.xhtml

dummy variable coded 1 for those willing to trade (0.5 <TTO < 1) and 0 for those unwilling to trade (TTO = 1).

For the regression models, a $p < 0.05$ was considered statistically significant. Analysis was carried out in Microsoft Excel and Stata version 16.0.

3 Results

Characteristics for statin respondents (n=514) and bisphosphonate respondents (n=365) who were included in the analysis set are reported in Table 1. Characteristics for the whole sample, including those excluded for having dominant preferences are presented in Supplementary appendix 5. Demographic characteristics, and experience of taking medicines was not associated with having dominant preferences to avoid preventative treatment (n=226). However, respondents from the public were more likely to have dominant preferences than those from the public (Supplementary appendix 6).

In the analysis set, patients in the statin survey tended to be older versus those in the public cohort with a higher proportion of male respondents (66.5% versus 50.0%). Compared with their public counterparts, patient respondents also tended to take more pills, at more times of the day, yet surprisingly, they also reported comparatively better health (EQ-5D-3L utility: 0.827 versus 0.818). Patient respondents in the bisphosphonate sample also tended to be older than those from the public sample but with a higher proportion of females than in the public cohort (89.2% versus 62.4%). Bisphosphonate patient respondents tended to take more pills, at more times of the day versus those in the public. In contrast to the statin survey, bisphosphonate patient respondents reported comparatively lower health than public respondents (EQ-5D-3L utility: 0.779 versus 0.786 respectively).

Table 2 and Figure 1 show the pre-imputation distributional properties for TTO values reported by patients and public, stratified by the question-context, for both the statins and bisphosphonates surveys. Bisphosphonate patients reported much higher values than public respondents (difference: 0.024 (se: 0.006)) with this difference being statistically significant. For both statins and bisphosphonates, changing

For peer review only - http://bmjopen.bmj.com/site/about/guidelines.xhtml

the question context did not alter the mean TTO scores by more than 0.01. Irrespective of the type of question or respondent, there was a clear difference between statins and bisphosphonates survey results. Consequently, regression models were run separately on these two cohorts with ordinary least squares regression favoured as it typically produced the least amount of error (Supplementary appendix 7).

For peer review only

Table 1: Description of the sample characteristics

	Statin survey			Bisphosphonate survey			
	Patient[a]	Public[b]	Total	Patient[a]	Public[b]	Total	Total
	N=227	N=287	N=514	N=86	N=279	N=365	N= 879
**Age**							
Less than 35	1 (0.7%)	14 (6.1%)	15 (4.0%)	0 (0.0%)	18 (7.9%)	18 (6.8%)	33 (5.2%)
35-44	4 (2.8%)	44 (19.1%)	48 (12.9%)	1 (2.7%)	42 (18.5%)	43 (16.3%)	91 (14.3%)
45-54	6 (4.2%)	46 (20.0%)	52 (14.0%)	2 (5.4%)	32 (14.1%)	34 (12.9%)	86 (13.5%)
55-64	43 (30.3%)	63 (27.4%)	106 (28.5%)	10 (27.0%)	45 (19.8%)	55 (20.8%)	161 (25.3%)
65-74	66 (46.5%)	60 (26.1%)	126 (33.9%)	15 (40.5%)	80 (35.2%)	95 (36.0%)	221 (34.7%)
75+	22 (15.5%)	3 (1.3%)	25 (6.7%)	9 (24.3%)	10 (4.4%)	19 (7.2%)	44 (6.9%)
Missing	85	57	142	49	52	101	243
**Sex**							
Female	49 (34.5%)	115 (50.0%)	164 (44.1%)	33 (89.2%)	141 (62.4%)	174 (66.2%)	338 (53.2%)
Male	93 (65.5%)	115 (50.0%)	208 (55.9%)	4 (10.8%)	85 (37.6%)	89 (33.8%)	297 (46.8%)
Missing	85	57	142	49	53	102	244
**Ethnicity**							
White British/Irish	133 (93.7%)	211 (91.7%)	344 (92.5%)	36 (97.3%)	203 (89.4%)	239 (90.5%)	583 (91.7%)
White other	3 (2.1%)	10 (4.3%)	13 (3.5%)	1 (2.7%)	8 (3.5%)	9 (3.4%)	22 (3.5%)
Mixed/Multiple ethnic origins	0 (0.0%)	1 (0.4%)	1 (0.3%)	0 (0.0%)	5 (2.2%)	5 (1.9%)	6 (0.9%)
Black/African/Caribbean/Black	0 (0.0%)	3 (1.3%)	3 (0.8%)	0 (0.0%)	2 (0.9%)	2 (0.8%)	5 (0.8%)
Asian/Asian British	0 (0.0%)	4 (1.7%)	4 (1.1%)	0 (0.0%)	7 (3.1%)	7 (2.7%)	11 (1.7%)
Chinese	0 (0.0%)	1 (0.4%)	1 (0.3%)	0 (0.0%)	2 (0.9%)	2 (0.8%)	3 (0.5%)
Other ethnicity	6 (4.2%)	0 (0.0%)	6 (1.6%)	0 (0%)	0 (0%)	0 (0%)	6 (0.9%)
Missing	85	57	142	49	52	101	243
**Number of pills taken daily**							
None	0 (0.0%)	91 (39.6%)	91 (24.5%)	0 (0.0%)	77 (33.9%)	77 (29.2%)	168 (26.4%)
One	4 (2.8%)	52 (22.6%)	56 (15.1%)	4 (10.8%)	38 (16.7%)	42 (15.9%)	98 (15.4%)
Two to five	104 (73.2%)	69 (30.0%)	173 (46.5%)	23 (62.2%)	86 (37.9%)	109 (41.3%)	282 (44.3%)
Six to ten	31 (21.8%)	14 (6.1%)	45 (12.1%)	5 (13.5%)	16 (7.0%)	21 (8.0%)	66 (10.4%)
More than ten	3 (2.1%)	4 (1.7%)	7 (1.9%)	5 (13.5%)	10 (4.4%)	15 (5.7%)	22 (3.5%)
Missing	85	57	142	49	52	101	243
**Number of different times pill taken per day**							
None	3 (2.1%)	94 (40.9%)	97 (26.1%)	0 (0.0%)	75 (33.0%)	75 (28.4%)	172 (27.0%)
Once per day	33 (23.2%)	74 (32.2%)	107 (28.8%)	18 (48.6%)	75 (33.0%)	93 (35.2%)	200 (31.4%)
2 times a day	87 (61.3%)	48 (20.9%)	135 (36.3%)	12 (32.4%)	54 (23.8%)	66 (25.0%)	201 (31.6%)
3 times a day	17 (12.0%)	11 (4.8%)	28 (7.5%)	5 (13.5%)	19 (8.4%)	24 (9.1%)	52 (8.2%)
More than 3 times a day	2 (1.4%)	3 (1.3%)	5 (1.3%)	2 (5.4%)	4 (1.8%)	6 (2.3%)	11 (1.7%)
Missing	85	57	142	49	52	101	243
**EQ-5D-3L utility**	0.827 (0.2)	0.818 (0.2)	0.822 (0.2)	0.770 (0.2)	0.786 (0.2)	0.783 (0.2)	0.806 (0.2)
Missing	56	41	97	35	33	68	165

Footnote: [a] patient sample was recruited from general practitioners in the NHS Research Scotland Primary Care Network or the Scottish Health Research Register (SHARE); [b]public sample was

For peer review only - http://bmjopen.bmj.com/site/about/guidelines.xhtml

Table 2: Summary statistics of TTO values

Medicine	Respondent	Question context	Mean	Standard deviation	Count	p10*	p50*	p90*	Proportion reporting disutility
Statins	Public	No side effects	0.965	0.057	237	0.942	0.983	1	0.746
Statins	Public	Some minor side effects	0.964	0.063	233	0.942	0.995	1	0.721
Statins	Public	Some severe side effects	0.964	0.060	232	0.942	0.988	1	0.725
Statins	Public	Reduced effectiveness	0.964	0.063	232	0.942	0.983	1	0.735
**Statins in the public**			0.964	0.061	934	0.942	0.992	1	0.731
Statins	Patients	No side effects	0.974	0.054	161	0.942	0.996	1	0.718
Statins	Patients	Some minor side effects	0.972	0.056	156	0.942	0.996	1	0.718
Statins	Patients	Some severe side effects	0.968	0.060	150	0.942	0.997	1	0.718
Statins	Patients	Reduced effectiveness	0.970	0.055	148	0.942	0.997	1	0.714
**Statins in patients**			0.971	0.056	615	0.942	0.997	1	0.717
**Statins all respondents**			0.967	0.059	1549	0.942	0.995	1	0.725
Bisphosphonates	Public	No side effects	0.925	0.081	219	0.833	0.967	1	0.860
Bisphosphonates	Public	Some minor side effects	0.930	0.076	216	0.833	0.967	1	0.846
Bisphosphonates	Public	Some severe side effects	0.932	0.076	214	0.833	0.967	1	0.846
Bisphosphonates	Public	Reduced effectiveness	0.933	0.078	216	0.833	0.967	1	0.842
**Bisphosphonates in the public**			0.930	0.078	865	0.833	0.967	1	0.849
Bisphosphonates	Patients	No side effects	0.949	0.069	42	0.833	0.983	1	0.814
Bisphosphonates	Patients	Some minor side effects	0.955	0.055	40	0.867	0.967	1	0.826
Bisphosphonates	Patients	Some severe side effects	0.958	0.060	40	0.867	0.975	1	0.802
Bisphosphonates	Patients	Reduced effectiveness	0.956	0.065	39	0.850	0.967	1	0.802
**Bisphosphonates in patients**			0.954	0.062	161	0.850	0.967	1	0.811
**Bisphosphonates all respondents**			0.934	0.076	1026	0.833	0.967	1	.840
**All statins and bisphosphonates**			0.954	0.068	2575	0.833	0.975	1	0.773

For peer review only - http://bmjopen.bmj.com/site/about/guidelines.xhtml

The mean conditional TTO value associated with statin use was 0.966 which generates a DTD of 0.034 (calculated as 1, representing full health, minus the estimated utility score). The mean conditional TTO value associated with bisphosphonate use was 0.933 consequently giving a DTD of 0.067. In the statin cohort, none of the explanatory variables were significantly associated with TTO results, with the exception that respondents from the Asian/Asian British group reported higher DTD associated with statin-use (0.044 greater than white British / Irish respondents) although this finding is only based on a very small sample (n=7) (Table 3). In the bisphosphonate cohort, the public provided a DTD level that was 0.011 larger than patients undergoing treatment with bisphosphonates. Furthermore, those who had experience of taking medications more than three times a day provided a much lower DTD value than those who had no experience of daily medicine use. None of the other explanatory variables had a statistically significant impact on DTD size, including the number of pills taken per day. Results from the logistic regression model also reported in Table 3 suggest that respondents in the statin survey were less likely to trade than those in the bisphosphonate survey; this is consistent with the finding of higher mean TTO (lower mean DTD) values in the statin scenarios. No other variables in the logistic regression were statistically significant.

For peer review only

For peer review only - http://bmjopen.bmj.com/site/about/guidelines.xhtml

Table 3: Regression results

	Statins	Bisphosphonates	Statins & Bisphosphonates
	OLS	OLS	Logistic
	Utility score [95% CI]	Utility score [95% CI]	Traders [a] [95% CI]
Sex			
Female (Reference)	0	0	1
Male	-0.00282	0.00141	0.859
	[-0.0104,0.00478]	[-0.00984,0.0127]	[0.726,1.016]
Ethnicity			
White British/Irish (Reference)	0	0	1
White other	-0.00042	0.0223	1.629
	[-0.0157,0.0149]	[-0.00800,0.0525]	[0.873,3.04]
Mixed/Multiple ethnic origins	-0.0105	-0.00997	1.511
	[-0.0707,0.0497]	[-0.0458,0.0259]	[0.482,4.734]
Black/African/Caribbean/Black British	-0.0349	-0.0128	1.866
	[-0.0852,0.0154]	[-0.0650,0.0395]	[0.578,6.027]
Asian/Asian British	-0.0467**	-0.0127	2.516
	[-0.0790,-0.0144]	[-0.0483,0.0228]	[0.866,7.309]
Chinese	0.0198	-0.0364	3.813
	[-0.0359,0.0755]	[-0.0895,0.0168]	[0.448,32.477]
Other ethnicity	0.0145	0.00717	0.936
	[-0.0106,0.0395]	[-0.144,0.158]	[0.418,2.097]
Age			
Less than 35 (Reference)	0	0	1
35-44	-0.00906	-0.0198	1.028
	[-0.0299,0.0118]	[-0.0496,0.0100]	[0.658,1.607]
45-54	-0.00428	-0.000518	1.516
	[-0.0322,0.0237]	[-0.0349,0.0339]	[0.921,2.496]
55-64	0.00732	0.00321	0.943
	[-0.0145,0.0292]	[-0.0240,0.0305]	[0.615,1.446]
65-74	0.00472	-0.00585	1.129
	[-0.0176,0.0271]	[-0.0352,0.0235]	[0.711,1.792]
75+	0.00398	-0.0184	0.77
	[-0.0237,0.0317]	[-0.0545,0.0177]	[0.424,1.397]
Number of pills taken per day			
None (Reference)	0	0	1
One	0.00379	-0.00288	0.903
	[-0.0120,0.0196]	[-0.0216,0.0158]	[0.658,1.239]
Two to five	-0.00776	-0.00873	1.085
	[-0.0286,0.0131]	[-0.0272,0.00977]	[0.811,1.452]
Six to ten	-0.00794	-0.00263	0.867
	[-0.0346,0.0188]	[-0.0253,0.0201]	[0.583,1.289]
More than ten	0.00295	-0.0127	1.242
	[-0.0308,0.0367]	[-0.0411,0.0157]	[0.63,2.446]
Number of different times pill are taken per day			
None (Reference)	0	0	1
Once per day	0.00475	0.00892	0.788
	[-0.0149,0.0244]	[-0.00798,0.0258]	[0.596,1.042]
2 times a day	0.0176	0.00988	0.757

For peer review only - http://bmjopen.bmj.com/site/about/guidelines.xhtml

For peer review only

	[-0.00781,0.0430]	[-0.0111,0.0309]	[0.568,1.009]
3 times a day	0.0197	0.00321	0.856
	[-0.00677,0.0462]	[-0.0254,0.0318]	[0.562,1.303]
More than 3 times a day	0.0257	0.0426*	1.327
	[-0.0166,0.0680]	[0.00658,0.0785]	[0.549,3.205]
Respondent			
Patient (Reference)	0	0	1
Public	-0.00176	-0.0106*	1.004
	[-0.0118,0.00830]	[-0.0202,-0.000937]	[0.831,1.214]
Question context			
Question 1 (Reference)	0	0	1
Question 2	-0.00118	0.00795	0.938
	[-0.00978,0.00741]	[-0.00695,0.0229]	[0.746,1.178]
Question 3	-0.00266	0.0065	0.932
	[-0.0143,0.00893]	[-0.00796,0.0210]	[0.742,1.171]
Question 4	-0.00216	0.00709	0.941
	[-0.0117,0.00737]	[-0.00909,0.0233]	[0.749,1.182]
Sample			
Statins			0.531***
			[0.444,0.635]
Constant	0.969***	0.941***	6.11***
	[0.947,0.991]	[0.907,0.975]	[3.615,10.335]
Observations	2056	1460	3516
Individuals	514	365	879

* $p < 0.05$, ** $p < 0.01$, *** $p < 0.001$

[a] Those with TTO utility scores <0 are coded 1. Coefficients reflect odds ratios. Above 1 implies more likely to provide a DTD value or willing to trade, less than 1 implies less likely to provide a DTD.

4 Discussion

In this study we find that long-term statin use is associated with a direct treatment disutility (DTD) of 0.034 among people willing to take statins. We find that bisphosphonate use is associated with a DTD of 0.067 among people willing to take bisphosphonates. These values imply that, even if medicines have no adverse effects, the act of taking them has a nontrivial impact on people's health-related quality of life. For statins, our study suggests that respondents on average would trade approximately 17 weeks of full health over 10 years whilst for bisphosphonates it would be more than half a year of life over 10 years. The findings for statins are particularly striking given these treatments are often thought by medical professionals to have minimal impact on users' daily routines.

For peer review only - http://bmjopen.bmj.com/site/about/guidelines.xhtml

Existing empirical studies have estimated a range of values of DTD but the general order of the size of the disutility is around 0.01 on average, which is equivalent to a loss of 5 weeks of perfect health over 10 years. In line with previous empirical studies, we find evidence for three different groups or types of respondent: (1) some never trading, suggesting zero disutility associated with treatments; (2) some suggesting they would be unlikely to initiate treatment and (3) some willing to trade length of life for no ongoing treatment, suggesting a DTD. In our survey, the groups willing to trade and generate a DTD made up the majority of those surveyed with approximately 72% and 84% for statins and bisphosphonates respondents, respectively.

We find that estimated mean DTD do not differ depending on whether the treatments were framed as more or less effective or having more or fewer side-effects or based upon demographic characteristics such as age or sex. However, we do find evidence suggesting that those from an Asian/Asian British ethnic minority background might perceive a higher level of disutility associated with long-term statin use although this is based upon a small sample of participants. This may also be an important part of ethnic health disparities to medication adherence and intensification of treatment. For example, British South Asians have been shown to more slowly intensify diabetes treatment than white groups [30]. We found no difference between patient and public disutilities for statins but we did find that bisphosphonate patients generated smaller disutility values than the values coming from the general public. This finding could support theories rooted in experience utility [31,32] whereby those possessing the "lived" knowledge of disease, or treatment of disease, do not perceive the negative effects to be as severe as those in the general public, trying to imagine it. Our findings suggest there could be "hedonistic adaption" [33], with the additional disutility of bisphosphonates being less severe for those who are already taking medicines more than three times a day anyway.

The implications of our findings for future cost–utility analyses evaluating treatment pathways featuring statins or bisphosphonates (and potentially other oral medicines) are not straightforward. On the one hand, cost-effectiveness analyses should ideally capture the impact of all relevant costs and consequences associated with alternative forms of treatment [34], so it must be relevant that we have demonstrated that the average person anticipates the act of taking statins or bisphosphonates will have a nontrivial impact

For peer review only - http://bmjopen.bmj.com/site/about/guidelines.xhtml

on their health-related quality of life. Accounting for this disutility is likely to reduce the desirability of treatments that are currently considered very cost-effective: estimates of cost-effectiveness for long-term preventative interventions have been shown to be particularly sensitive to the inclusion of DTD[5–8,10,12–14][35]. Indeed, we have previously shown that, for some people for whom guidelines currently recommend statins (e.g. those at a 10% risk of a cardiovascular event over 10 years), a DTD that appears moderate in light of the current study (0.015) would result in treatment doing more harm than good [36]. Another reason routinely to account for DTD is that, without it, it is not possible to value innovations with positive process characteristics.

On the other hand, the apparent existence of distinct preference groups among our respondents requires careful consideration. A substantial minority of participants repeatedly indicated that they would be unwilling to trade any life expectancy to avoid taking these medicines, suggesting they consider any inconvenience with which they are associated negligible. It would be difficult to deny access to a treatment on the grounds that the average person would be bothered by its process characteristics, which is a danger if population-level cost-effectiveness estimates routinely incorporate average DTD. In view of these conflicting considerations, we recommend that decision-makers review scenarios with and without DTD. If evidence suggests that including DTD would materially alter the balance of benefits, harms and costs associated with treatment, this should be highlighted in population-level guidance, enabling prescribers at an individual level to engage in shared decision-making that gives appropriate weight to the person's preferences for avoiding the treatment's process characteristics. Such an approach fits well with the guideline development methods for NICE [37], which encourage the explicit identification of 'preference-sensitive decision-points', taking the practicalities of possible treatments into account.

Our study has several strengths. Firstly, the TTO methods we employ are consistent with the Measurement and Valuation of Health (MVH) protocol first used to derive the EQ-5D-3L. Moreover, unlike previous studies that have attempted to elicit these values, we use a large representative sample of both public and patients for our estimated values. Finally, due to the challenges associated with understanding and communicating risk, particularly in vulnerable older age-groups, we extensively trialed and developed the use of innovative approaches. For example, we used story-boards and visual

For peer review only - http://bmjopen.bmj.com/site/about/guidelines.xhtml

arrays to contextualize the TTO experiment and communicate absolute risk. There are some limitations to our study which need to be considered. Whilst we made best endeavours to communicate the experiment, the underlying absolute risks and the context for the research question, this had a set of clear trade-offs for participants. The length of the survey, the cognitive burden and the time required were noted as challenges. Some of our respondents reported that they had difficulty understanding the TTO questions whilst others reported inconsistent values across the survey questions. Secondly, those who took part in our survey ultimately self-selected and this could be related to their ability to complete the survey as well as their self-reported health. Applicability for a different patient or general population should be made based upon a careful judgement of the self-reported characteristics summarised for this study cohort.

5 Conclusion

Long-term preventative interventions, such as statins for cardiovascular disease or bisphosphonates for bone fractures, have a quantifiable direct treatment disutility associated with their use. The majority of respondents in our experiment, including public and patient-users, indicate at least some DTD. Bisphosphonates had larger disutilities than statins. Disutility was largely unaffected by respondents' self-reported characteristics but patient users of bisphosphonates did provide smaller DTDs than the public. Future model-based studies assessing the cost-effectiveness of long-term preventative interventions should incorporate DTD values within scenario analyses.

For peer review only - http://bmjopen.bmj.com/site/about/guidelines.xhtml

Declarations

Funding: This study/project is funded by the National Institute for Health Research (NIHR) Health Services and Delivery Research Programme (project reference 15/12/22). The views expressed are those of the authors and not necessarily those of the NIHR or the Department of Health and Social Care. The authors had full and sole access the data, and the funder had no role in the conduct of the research or the decision to publish.

Conflicts of interest. DM reports that he is supported by a Wellcome Trust Clinical Research Fellowship (214588/Z/18/Z) and is a member of the European Medicines Agency Pharmacovigilance Risk Assessment Committee. No other authors report any conflicts of interest directly relevant to the content of this article.

Consent to participate: Respondents gave informed consent after reading the participant information sheet online and indicating they agreed to participate in the survey online.

Consent for publication: The participant information sheet indicated that the results of the survey would be published and so we obtained informed consent

Data availability: It is not possible to share the original data as this was a criterion for consent of participants.

Code availability: Survey design and data analysis code can be made available on request.

Contribution: All authors meet International Committee of Medical Journal Editors (ICMJE) criteria for authorship.

AJT obtained funding for this study, was involved in formulating the research question, provided advice on the design for the overall study, analysed data and produced a first draft of the manuscript. AJT acts as guarantor for this work.

JY was involved in formulating the research question, completed the process for ethical approval, produced the online version of the survey and contributed to writing the manuscript.

BG obtained funding for this study, was involved in formulating the research question, provided advice on the design for the overall study, contributed to the design of the online survey and writing the manuscript.

RH helped clean and analyse the data and contributed to writing the manuscript.

PD obtained funding for this study, was involved in formulating the research question, provided advice on the design for the overall study, contributed to the design of the online survey and writing the manuscript.

GR contributed to data analysis and contributed to writing the manuscript.

DM obtained funding for this study, was involved in formulating the research question, provided advice on the design for the overall study and contributed to writing the manuscript

KP obtained funding for this study, formulated the research question, provided advice on the design for the overall study, contributed to the design of the online survey and oversaw data collection and analysis and contributed to writing the manuscript.

This manuscript has been read and approved by all the authors.

For peer review only - http://bmjopen.bmj.com/site/about/guidelines.xhtml

Estimating values for direct treatment disutility

For peer review only

For peer review only - http://bmjopen.bmj.com/site/about/guidelines.xhtml

Ethics approval

Ethical approval for this study was granted by an NHS Health Research Authority Research Ethics Committee (REC reference: 17/NW/0124; project number: 220492).

Acknowledgements: We dedicate the article to the memory of Graham Bell who helped us greatly when formulating the research question and study design. We also wish to thank our other patient and public representatives that were integral to the design of this study. Our sincere thanks goes to all the respondents who piloted the initial survey and completed the final surveys for this study. We acknowledge the insightful contributions of Dr Shona Livingstone, Research Fellow in Statistics, to the survey design.

For peer review only

References

1. Taylor F, Huffman MD, Macedo AF, Moore THM, Burke M, Davey Smith G, et al. Statins for the primary prevention of cardiovascular disease [Internet]. Vol. 2017, Cochrane Database of Systematic Reviews. 2013 [cited 2021 Mar 22]. Available from: www.cochranelibrary.com
2. Papapoulos SE. The role of bisphosphonates in the prevention and treatment of osteoporosis. Am J Med. 1993 Nov 30;95(5 SUPPL. 1):S48–52.
3. Sav A, Kendall E, Mcmillan SS, Kelly F, Whitty JA, King MA, et al. "You say treatment, I say hard work": Treatment burden among people with chronic illness and their carers in Australia. Heal Soc Care Community. 2013 Nov;21(6):665–74.
4. Thompson AJ, Payne K, Guthrie B. Do pills have no ills? Capturing the impact of direct treatment disutility. Pharmacoeconomics. 2015;
5. Fontana M, Asaria P, Moraldo M, Finegold J, Hassanally K, Manisty CH, et al. Patient-accessible tool for shared decision making in cardiovascular primary prevention: balancing longevity benefits against medication disutility. Circulation [Internet]. 2014 Jun 17 [cited 2015 Jan 23];129(24):2539–46. Available from: http://www.ncbi.nlm.nih.gov/pubmed/24744274
6. Hutchins R, Pignone MP, Sheridan SL, Viera a. J. Quantifying the utility of taking pills for preventing adverse health outcomes: a cross-sectional survey. BMJ Open [Internet]. 2015;5(5):e006505–e006505. Available from: http://bmjopen.bmj.com/cgi/doi/10.1136/bmjopen-2014-006505
7. Hutchins R, Viera a. J, Sheridan SL, Pignone MP. Quantifying the Utility of Taking Pills for Cardiovascular Prevention. Circ Cardiovasc Qual Outcomes [Internet]. 2015;1–10. Available from: http://circoutcomes.ahajournals.org/cgi/doi/10.1161/CIRCOUTCOMES.114.001240
8. Lazar LD, Pletcher MJ, Coxson PG, Bibbins-Domingo K, Goldman L. Cost-effectiveness of statin therapy for primary prevention in a low-cost statin era. Circulation [Internet]. 2011 Jul 12 [cited 2015 Jan 7];124(2):146–53. Available from: http://www.ncbi.nlm.nih.gov/pubmed/21709063
9. Timbie JW, Hayward RA, Vijan S. Variation in the net benefit of aggressive cardiovascular risk factor control across the US diabetes population. Arch Intern Med. 2010;170(12):1037–44.
10. Greving JP, Visseren FLJ, de Wit G a, Algra a. Statin treatment for primary prevention of vascular disease: whom to treat? Cost-effectiveness analysis. BMJ [Internet]. 2011 Jan [cited 2015 Jan 27];342:d1672. Available from: http://www.ncbi.nlm.nih.gov/pubmed/21450800
11. Augustovski FA, Cantor SB, Thach CT, Spann SJ. Aspirin for primary prevention of cardiovascular events. JGenInternMed. 1998;13:824–35.
12. Pletcher M, Lazar L. Comparing impact and cost-effectiveness of primary prevention strategies for lipid-lowering. Ann Intern … [Internet]. 2009 [cited 2015 Jan 27]; Available from: http://annals.org/article.aspx?articleid=744295
13. Pletcher MJ, Pignone M, Earnshaw S, McDade C, Phillips K a, Auer R, et al. Using the coronary artery calcium score to guide statin therapy: a cost-effectiveness analysis. Circ Cardiovasc Qual

For peer review only - http://bmjopen.bmj.com/site/about/guidelines.xhtml

Outcomes [Internet]. 2014 Mar [cited 2015 Jan 23];7(2):276–84. Available from: http://www.ncbi.nlm.nih.gov/pubmed/24619318

14. Pignone M, Earnshaw S. Aspirin, statins, or both drugs for the primary prevention of coronary heart disease events in men: a cost–utility analysis. Ann Intern … [Internet]. 2006 [cited 2015 Jan 27]; Available from: http://annals.org/article.aspx?articleid=720844

15. Torrance G. Utility approach to measuring health-related quality of life. J Chronic Dis. 1987;40:593–600.

16. Sackett DL, Torrance GW. The utility of different health states as perceived by the general public. J Chronic Dis. 1978;31(11):697–704.

17. NICE. Guide to the methods of technology appraisal 2013 [Internet]. 2013 [cited 2021 Apr 21]. Available from: https://www.nice.org.uk/process/pmg9/chapter/foreword

18. Dolan P, Gudex C, Kind P, Williams A. The time trade-off method: Results from a general population study. Health Econ. 1996;5(2):141–54.

19. Guthrie B, Thompson A, Dumbreck S, Flynn A, Alderson P, Nairn M, et al. Better guidelines for better care: accounting for multimorbidity in clinical guidelines – structured examination of exemplar guidelines and health economic modelling. Heal Serv Deliv Res [Internet]. 2017 Apr [cited 2017 Jul 12];5(16):1–150. Available from: https://www.journalslibrary.nihr.ac.uk/hsdr/hsdr05160

20. Ryan M, Watson V, Entwistle V. Rationalising the "irrational": A think aloud study of discrete choice experiment responses. Health Econ [Internet]. 2009 [cited 2021 Mar 22];18(3):321–36. Available from: https://pubmed.ncbi.nlm.nih.gov/18651601/

21. Cheraghi-Sohi S, Bower P, Mead N, McDonald R, Whalley D, Roland M. Making sense of patient priorities: Applying discrete choice methods in primary care using "think aloud" technique. Fam Pract [Internet]. 2007 Jun [cited 2021 Mar 22];24(3):276–82. Available from: https://pubmed.ncbi.nlm.nih.gov/17478438/

22. Sawtooth Software SSI Web 8.3.8. Sawtooth. Sawtooth Software SSI Web 8.3.8, (2012). 2012.

23. NHS Research Scotland. The NRS Primary Care Network [Internet]. Available from: https://www.nhsresearchscotland.org.uk/research-areas/primary-care

24. NHS Research Scotland. SHARE [Internet]. Available from: http://www.registerforshare.org/index.php:

25. Dynata. Dynata [Internet]. Available from: https://www.dynata.com/about-us/

26. Brazier J, Ratcliffe J, Saloman J, Tsuchiya A. Measuring and Valuing Health Benefits for Economic Evaluation - John Brazier; Julie Ratcliffe; Joshua Saloman; Aki Tsuchiya - Oxford University Press [Internet]. 2007 [cited 2021 Jul 19]. Available from: https://www.amazon.co.uk/Measuring-Valuing-Benefits-Economic-Evaluation/dp/0198569823

27. Scott A. Identifying and analysing dominant preferences in discrete choice experiments: An application in health care. J Econ Psychol. 2002 Jun 1;23(3):383–98.

28. Ryan M, Farrar S. Using conjoint analysis to elicit preferences for health care. BMJ [Internet]. 2000

For peer review only - http://bmjopen.bmj.com/site/about/guidelines.xhtml

Jun 3 [cited 2021 Jul 20];320(7248):1530–3. Available from: https://www-bmj-com.manchester.idm.oclc.org/content/320/7248/1530

29. White IR, Royston P, Wood AM. Tutorial in Biostatistics Multiple imputation using chained equations: Issues and guidance for practice. 2010 [cited 2018 Aug 30]; Available from: https://onlinelibrary.wiley.com/doi/pdf/10.1002/sim.4067

30. Mathur R, Farmer RE, Eastwood S V., Chaturvedi N, Douglas I, Smeeth L. Ethnic disparities in initiation and intensification of diabetes treatment in adults with type 2 diabetes in the UK, 1990–2017: A cohort study. PLOS Med [Internet]. 2020 May 1 [cited 2021 Aug 18];17(5):e1003106. Available from: https://journals.plos.org/plosmedicine/article?id=10.1371/journal.pmed.1003106

31. Dolan P, Kahneman D. Interpretations of utility and their implications for the valuation of health. Econ J. 2008;118(525):215–34.

32. Kahneman D, Wakker PP, Sarin R. Back to Bentham? Explorations of Experienced Utility. Source Q J Econ [Internet]. 1997 [cited 2021 Jul 30];112(2):375–405. Available from: https://about.jstor.org/terms

33. Carel H. Is Well-being Possible in Illness? Phenomenol Illn. 2016 Sep 22;130–49.

34. Drummond MF, Sculpher MJ, Claxton K, Stoddart GL, Torrance GW. Methods for the economic evaluation of healthcare programmes. Fourth Edi. Oxford; 2005.

35. Thompson A, Guthrie B, Payne K. Do Pills Have No Ills? Capturing the Impact of Direct Treatment Disutility. Pharmacoeconomics. 2016;34(4):333–6.

36. Thompson A, Guthrie B, Payne K. Using the Payoff Time in Decision-Analytic Models: A Case Study for Using Statins in Primary Prevention. Med Decis Mak [Internet]. 2017 [cited 2017 Apr 28];37(7):759–69. Available from: http://journals.sagepub.com/doi/pdf/10.1177/0272989X17700846

37. National Institute for Health and Care Excellence. Developing NICE guidelines: supporting-shared-decision-making [Internet]. 2020. Available from: https://www.nice.org.uk/process/pmg20/chapter/writing-the-guideline#supporting-shared-decision-making

For peer review only - http://bmjopen.bmj.com/site/about/guidelines.xhtml

Figure 1: Kernel density plots showing distribution of TTO responses stratified by medicine, question context and respondent type

For peer review only

For peer review only - http://bmjopen.bmj.com/site/about/guidelines.xhtml

Supplementary Appendices for:

Thompson AJ, Youn J, Payne K, Hainsworth R, Donnan P, Allen A, Bell G, Rogers G, Guthrie B. Quantifying the impact of taking medicines for primary prevention: a time-trade off study to elicit direct treatment disutility

Supplementary Appendix 1: The statin survey
Supplementary Appendix 2: The bisphosphonate survey
Supplementary Appendix 3: Training materials for statin survey
Supplementary Appendix 4: Training materials for bisphosphonates survey
Supplementary Appendix 5: Patient characteristics in those excluded
Supplementary Appendix 6: Propensity for dominant preferences
Supplementary Appendix 7: Performance of different models

For peer review only

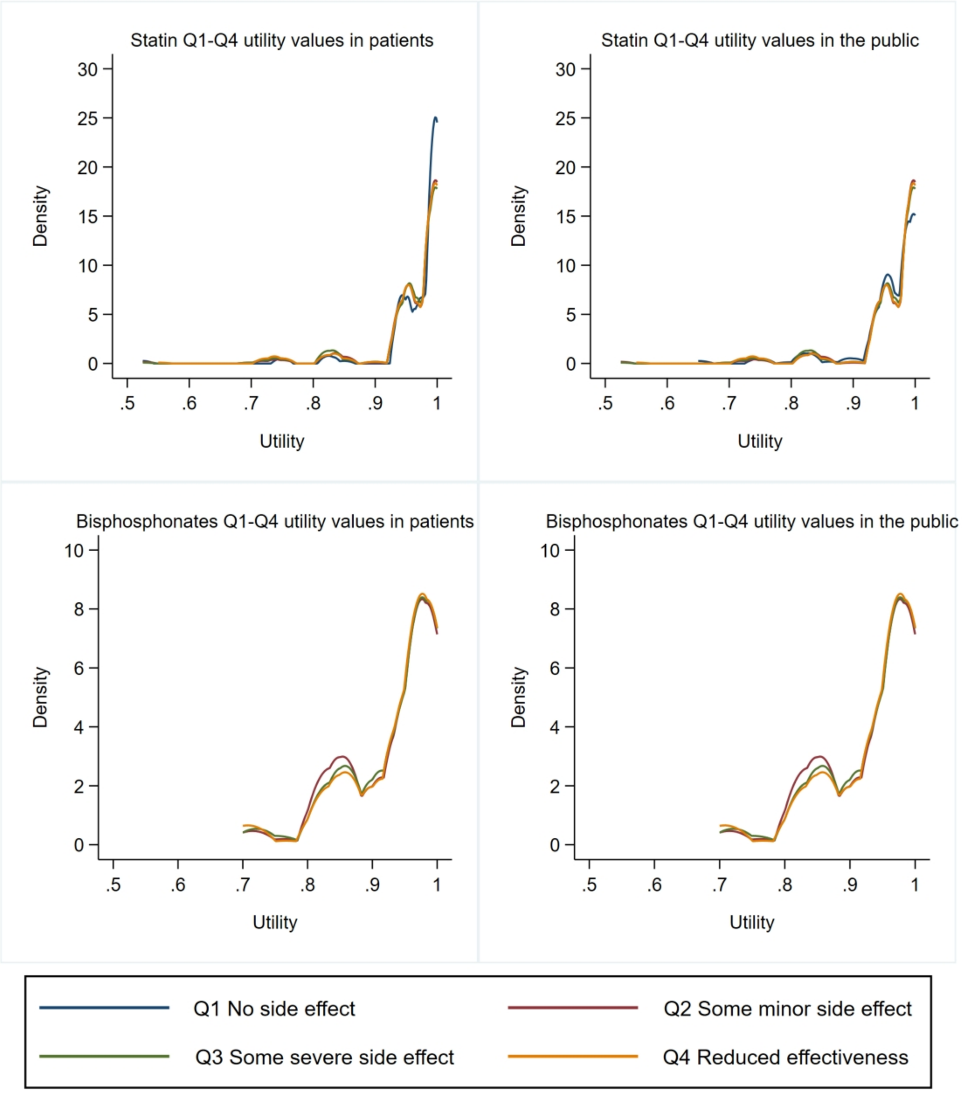

Kernel density plots showing distribution of TTO responses stratified by medicine, question context and respondent type

705x806mm (72 x 72 DPI)

Quantifying the impact of taking medicines for long-term conditions

A survey to understand your views about taking medicines long term for the prevention of heart disease

In this survey, we will be asking you about a drug treatment which may be offered to people at an increased risk of developing heart disease.

There are no right or wrong answers, we are just interested in hearing about your views.

For peer review only

Quantifying the impact of taking medicines for long-term conditions

Taking medicines long term for the prevention of heart disease

Participant Consent Form

You are being invited to take part in a research study that aims to understand your views about taking medicines long-term. We want to understand people's views on about what it is like to take a medicine every day for long periods of time. Before you decide, it is important for you to understand why the research is being done and what it will involve.

Please click on the sections below for information on the research.

What is the aim of the research?

Why have I been chosen?

What happens to the data collected?

How is confidentiality maintained?

How long will it take me?

Who will conduct the research?

What would I be asked to do if I took part?

Will the outcomes of the research be published?

What happens if I do not want to take part or if I change my mind?

Who has reviewed the research project?

I understand that my participation in the study is voluntary and that I am free to withdraw at any time without giving a reason by exiting the survey.

I confirm that I have read the information above.

- ◯ Yes, I would like to take part in the survey.
- ◯ No, I do not want to participate (exit survey).

0% 100%

What is your gender?

◯ Female
◯ Male

0% 100%

For peer review only

[QUOTA: QuotaCheck]

Note:

This page only contains quota information and only appears in preview mode.

0% 100%

For peer review only

By selecting one box in each group below, please indicate which statements best describe your own health state today.

Mobility

- ◯ I have no problems in walking about
- ◯ I have some problems in walking about
- ◯ I am confined to bed

Self-Care

- ◯ I have no problems with self-care
- ◯ I have some problems washing or dressing myself
- ◯ I am unable to wash or dress myself

Usual Activities (e.g. work, study, housework, family or leisure activities)

- ◯ I have no problems with performaing my usual activities
- ◯ I have some problems with performing my usual activities
- ◯ I am unable to perform my usual activities

Pain/Discomfort

- ◯ I have no pain or discomfort
- ◯ I have moderate pain or discomfort
- ◯ I have extreme pain or discomfort

Anxiety/Depression

- ◯ I am not anxious or depressed
- ◯ I am moderately anxious or depressed
- ◯ I am extremely anxious or depressed

0% 100%

Part 1. Study Background

This section of the survey explains: heart disease, how medicine treatments may be used for people at risk of heart disease, and what the treatment involves.

We will follow a story of Bobbie.

Click next to continue.

0% 100%

For peer review only

Part 1. Background

Bobbie has been to see a doctor.

The doctor has said Bobbie needs to start a medicine to prevent serious events because of his heart disease.

We will take you through Bobbie's story.

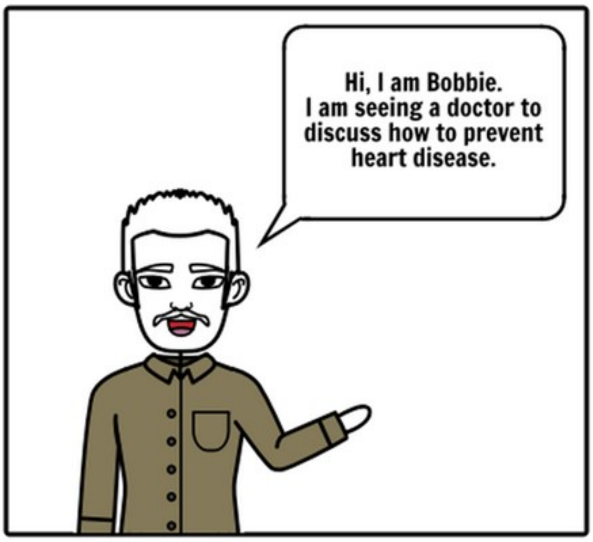

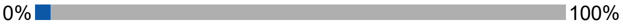

Part 1. Background

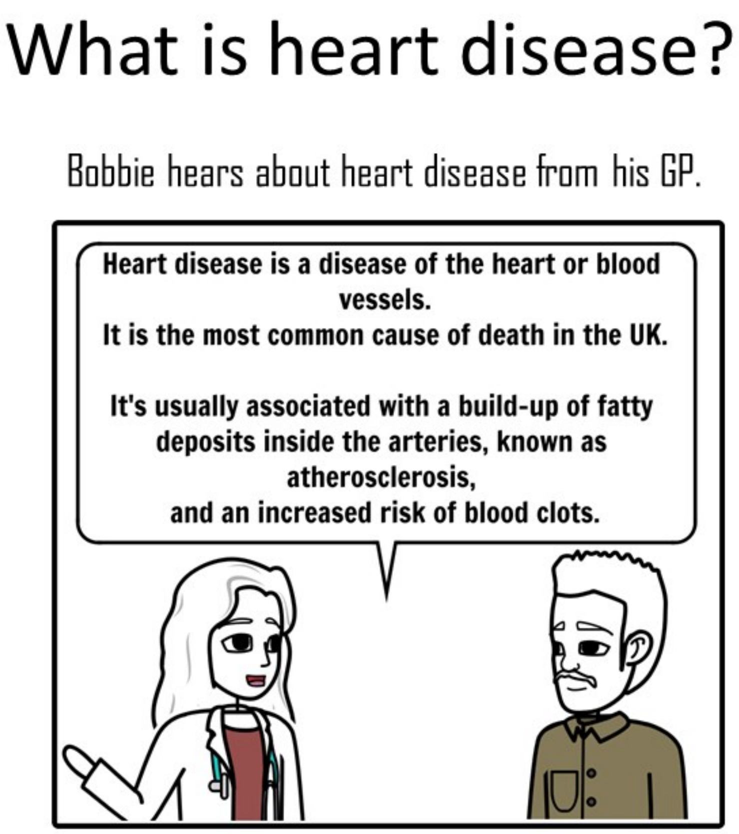

0% 100%

Part 1. Background

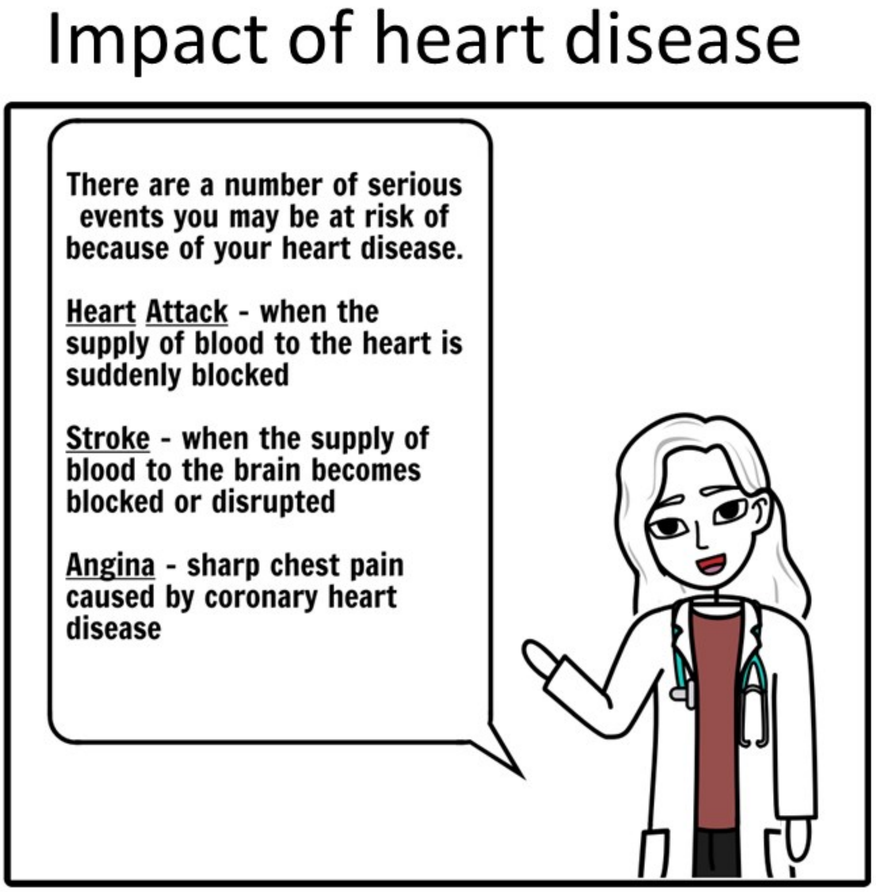

0% 100%

Part 1. Background

Risk of Heart disease

The GP explains that there is a chance that because of his cholesterol level Bobbie is at risk of developing one of these serious events in the future.

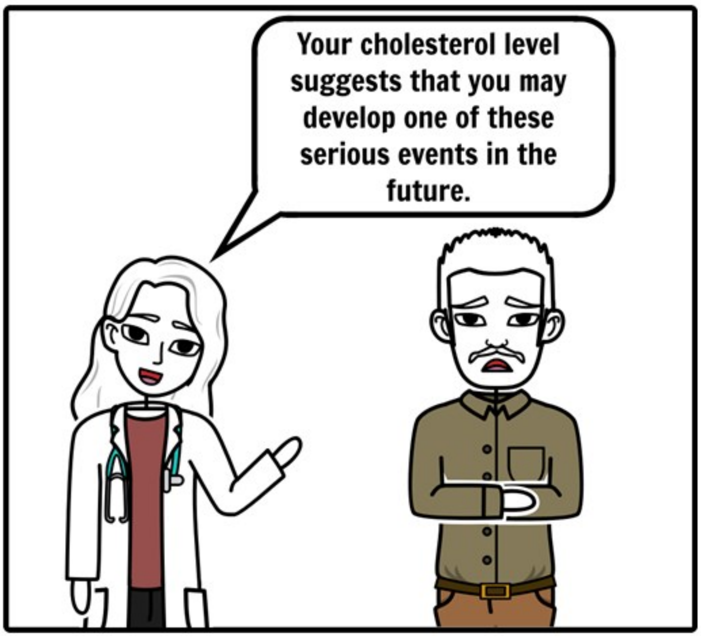

0% 100%

Part 1. Background

What is risk?

- **Risk** is: a term used to explain the chance that something bad might happen.

- **10% risk** means:

  Out of every **1,000** people, with heart disease,

  100 people would develop a heart attack, and **900** people would not.

0% 100%

Part 1. Background

What is a 10% risk?

- This diagram shows a 10% risk of having a heart attack because of their heart disease.
- The people shaded blue are the ones who have a heart attack.

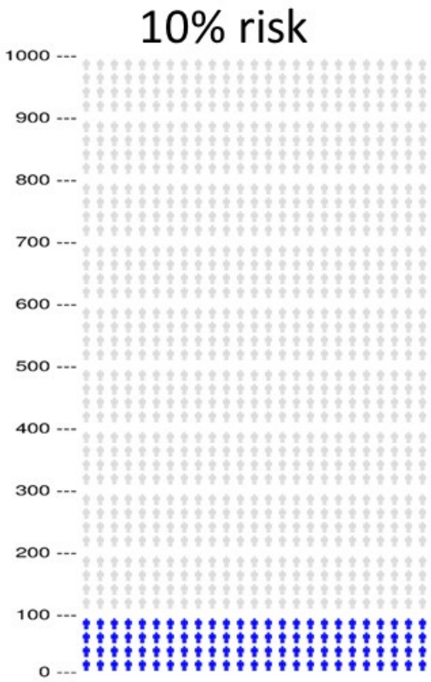

0% 100%

Part 1. Background

Prevention of serious events from heart disease

- Bobbie's doctor can estimate how likely it is that he will develop heart disease over the next 10 years.
- The estimate is based on things such as Bobbie's age and sex, family medical history, blood pressure and cholesterol level.
- The doctor may prescribe certain medicines such as statins to help reduce the chance of Bobbie having heart disease.
- The medicine will prevent some of the people who take it from having a serious event because of the heart disease.
- A serious event from the heart disease will still happen to some of the people who take the medicine.

0% 100%

Part 1. Background

Prevention of serious events from heart disease

The doctor prescribes Bobbie a medicine to reduce the chance of his heart disease causing a heart attack or other heart conditions in the future.

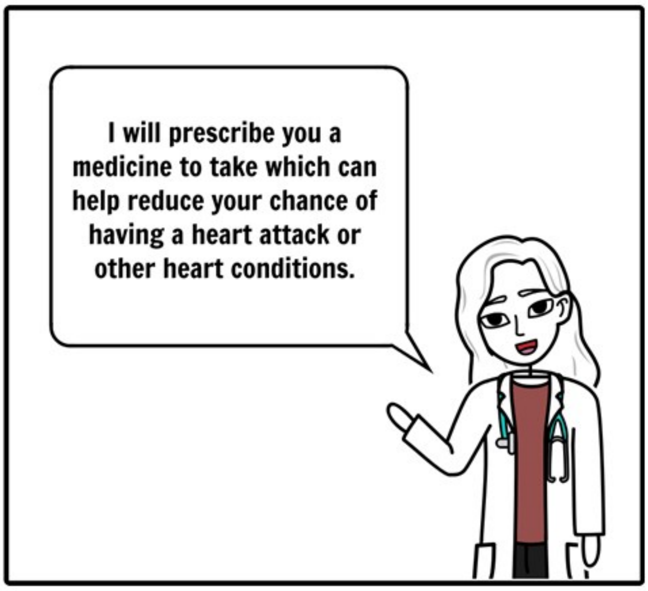

0% 100%

Part 1. Background

Collecting the medicine from the pharmacy

Bobbie goes to a pharmacy to collect his medicine.

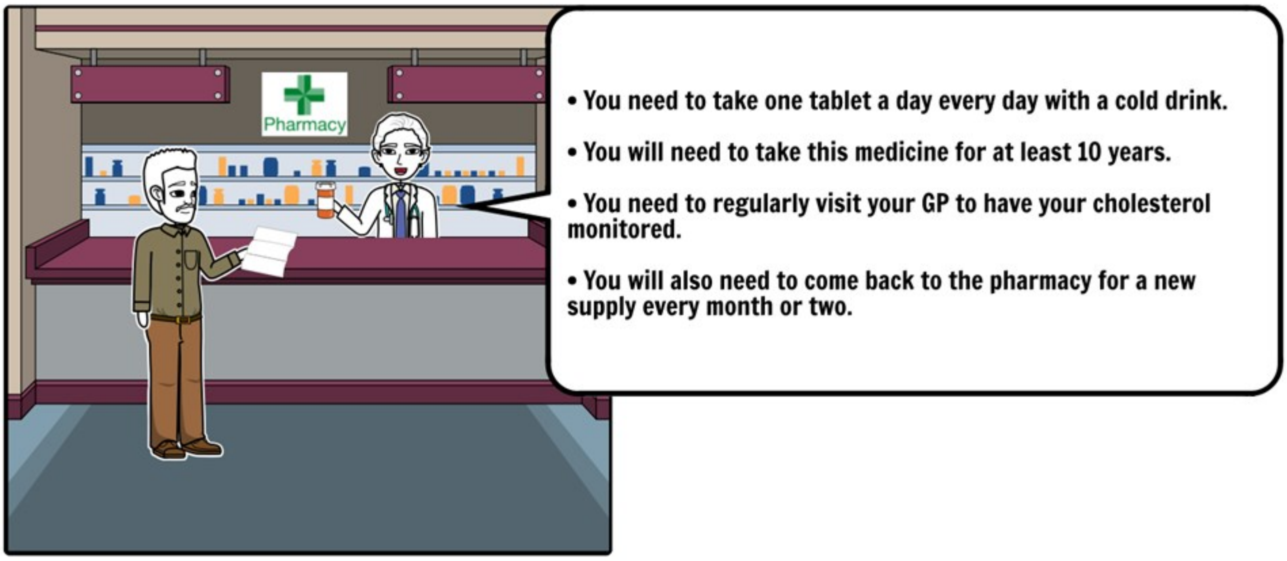

0% 100%

Part 1. Background

Taking the medicine for prevention

Bobbie goes home with his new tablets.

He then has to decide whether he wants to take them as the doctor and the pharmacist have advised.

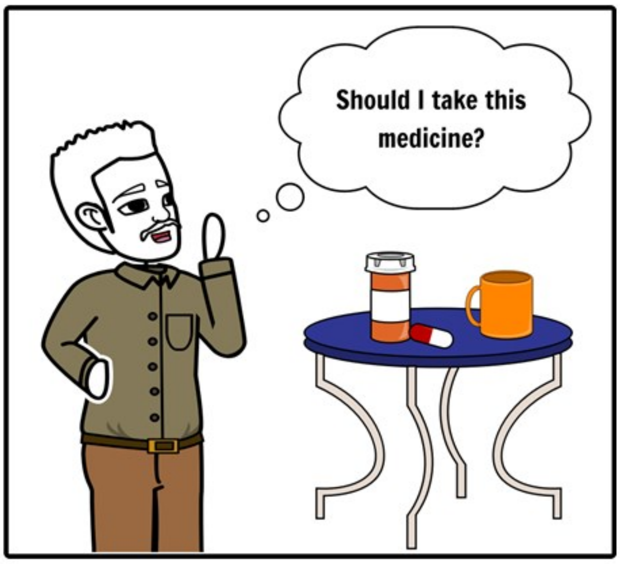

0% 100%

Part 1. Background

- Bobbie may consider three key things, when deciding whether to take the medicine.

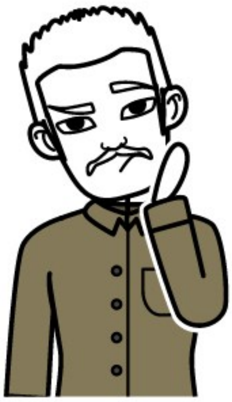

Effectiveness of the medicine

- How good the medicine is in terms of preventing a condition like a heart attack

Side effects of the medicine

- Whether there is any potential harm from the medicine

Inconvenience of the medicine

- Whether taking the medicine would fit in his lifestyle or cause him inconvenience

0% 100%

Part 1. Background

How effective is the medicine?

- Some people, but not everyone, who take the medicine will avoid a serious event, such as a heart attack or stroke as a result of taking the tablet.

- The effectiveness of the medicine can be described in terms of a reduction in this risk.

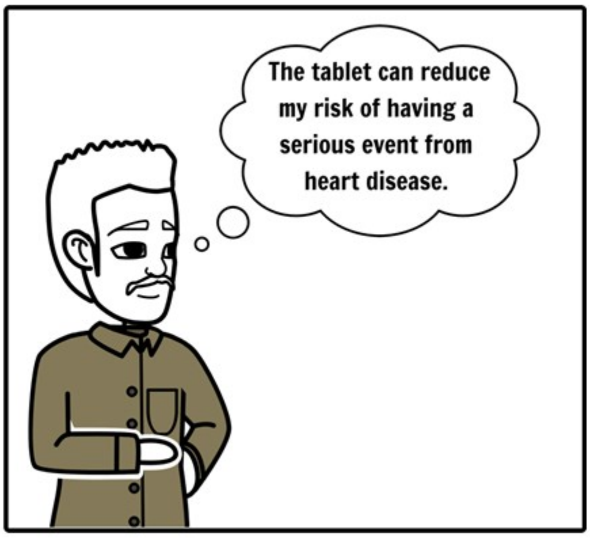

0% 100%

Part 1. Background

What does ‘reduction in risk’ mean?

- A ‘reduction in risk from 10% to 6%’ means:

The medicine will reduce your risk of experiencing an event, such as a heart attack, within the next 10 years from 10% to 6%.

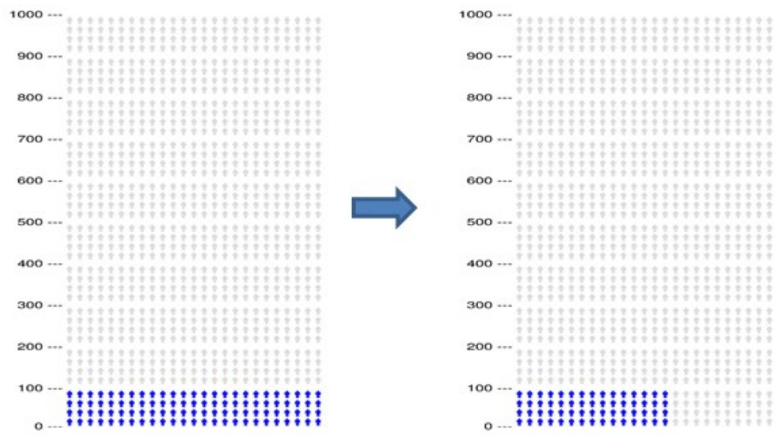

No treatment: 10% risk

Taking the medicine: 6% risk

0% 100%

Part 1. Background

Does the medicine have side effects?

The potential for side effects from the medicine can be described in terms of the risk of a side effect.

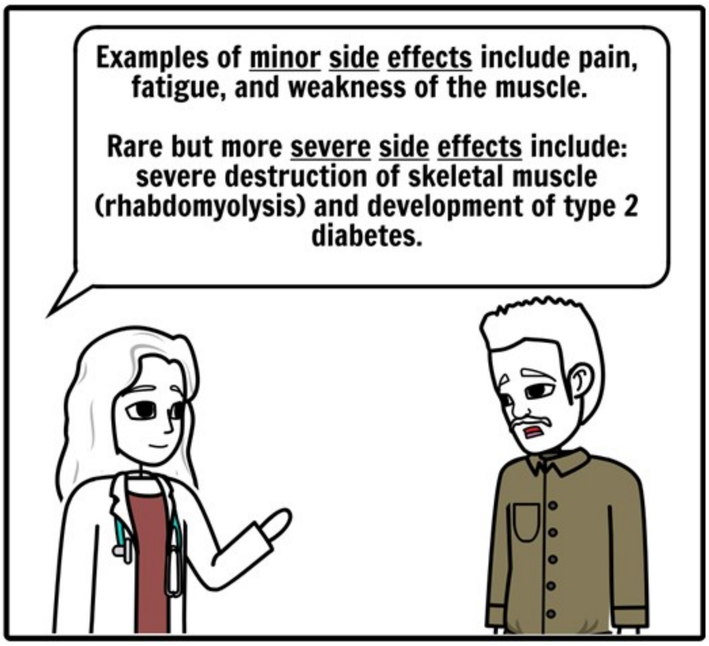

0% 100%

Part 1. Background

Risk of a minor side effect from the medicine

- A 5% risk of a **minor** side effect means:

Among every 1,000 people taking the medicine,

50 will experience a minor side effect such as either: pain, fatigue, and weakness of the muscle.

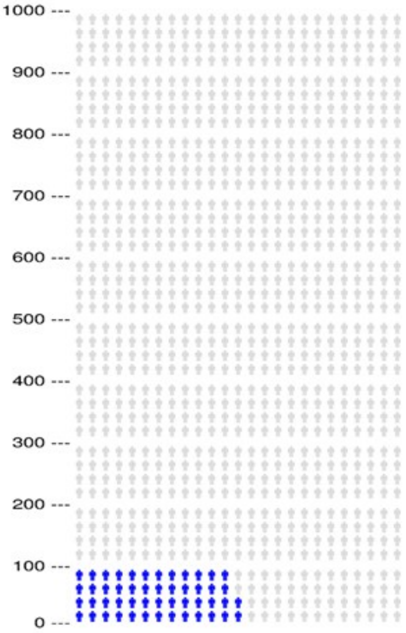

5% risk of a minor side effect

0% 100%

Part 1. Background

Risk of a severe side effect from the medicine

- A 0.1% risk of a **severe** side effect means:

Among every 1,000 people taking the medicine,

1 will experience a severe side effect such as either: severe destruction of skeletal muscle or development of type 2 diabetes.

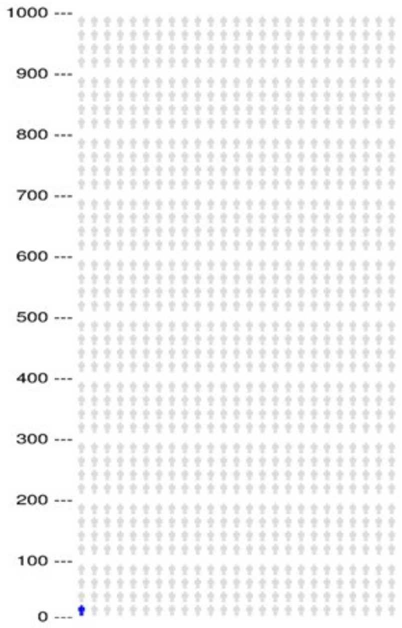

0.1% risk of a severe side effect

0% 100%

Part 1. Background

How inconvenient is it when taking the medicine?

Bobbie also thinks about what it means to have to take this medicine for the next ten years.

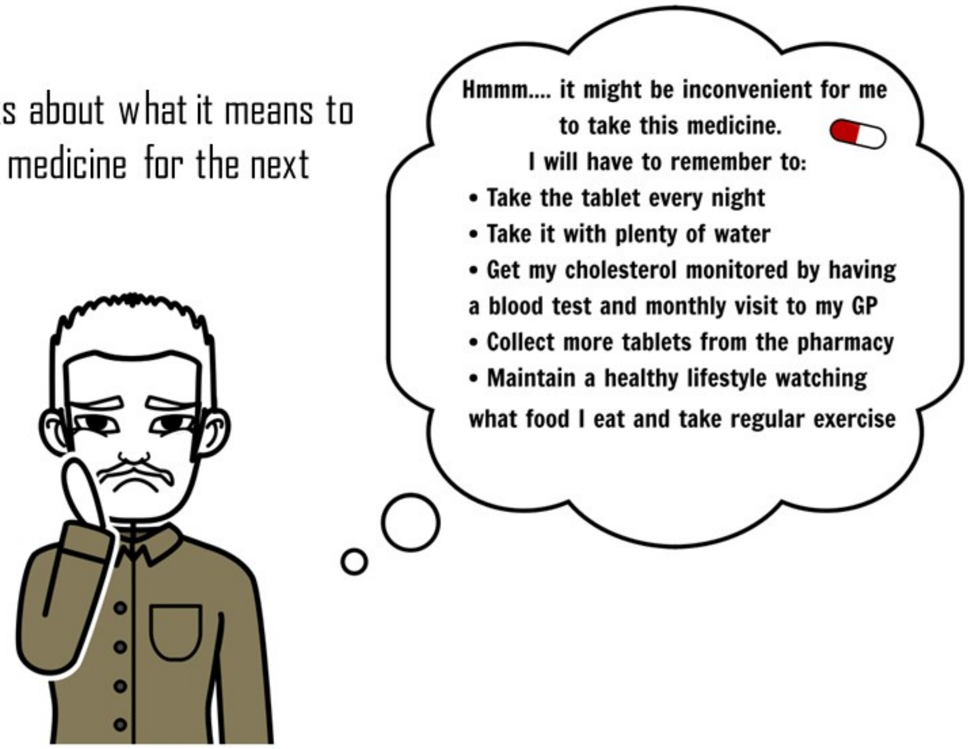

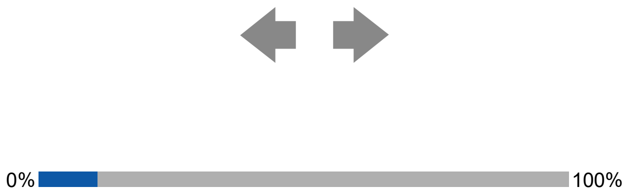

Part 1. Background

In the questions that follow in this survey, we will ask you about how you think about taking a medicine to prevent heart disease.

0% 100%

In general, how would you feel about taking a medicine for the first time?

- ◯ **Happy**
  I trust in my doctor and I am willing to try new things
- ◯ **Unsure**
  I'd like to think about it really carefully
- ◯ **Unhappy**
  I don't like the idea of trying a new medicine even if the doctor says it will help

0% 100%

For peer review only

Part 2: Your views on the inconvenience of taking a medicine to prevent heart disease

Now, we will ask you about how you think about the inconvenience of taking a medicine to prevent heart disease.

There are four questions.

Each question may look similar but differs in terms of the detail for each of the three features.

We will start with a practice question to show you an example of the type of question we will ask you.

0% 100%

For peer review only

Warm up question

You have been told that you need to take a medicine to reduce the risk of heart disease.

You can choose between two different medicines. We have called these Medicine A and Medicine B.

Medicine A is a pill that you only need to take once.

Medicine B is a pill that you need to take every day for ten years.

Medicine A and Medicine B are equally effective in reducing your risk of developing heart disease.
Medicine A and Medicine B will reduce your risk of having a heart attack or a stroke within the next 10 years from **10%** to **6%**.

In other words, in a crowd of **1,000** people in the UK, **100** are likely to have a heart attack or a stroke if they do not take the medicine. This is reduced to **60** people out of 1,000 who do take the medicine. This information is also illustrated in this picture.

Risk of heart disease

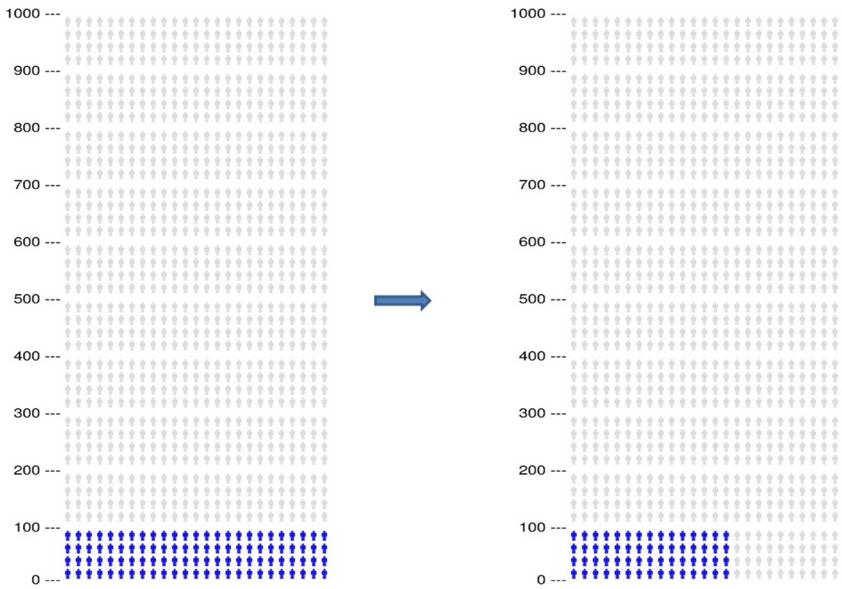

10%
No Treatment

6%
Taking the Medicine

(The figures shaded blue represent people who will have a heart attack or a stroke in the next 10 years)

In this scenario, you will not have any side effects from Medicine A or Medicine B.

Risk of a side effect

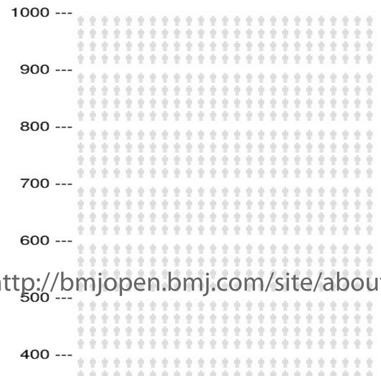

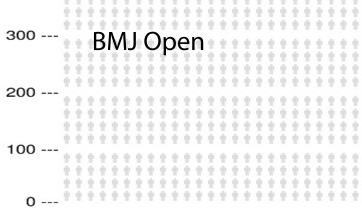

0%
No one will have a side effect.

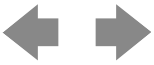

0% 100%

or peer review only

Remember Medicine A and Medicine B are equally effective and have no risks of a side effect.

The only difference between Medicine A and Medicine B is how you take them.

**Medicine A**	**Medicine B**
You take a tablet only once in your life.<br>You will be given the tablet on the day you come in to see your GP.<br>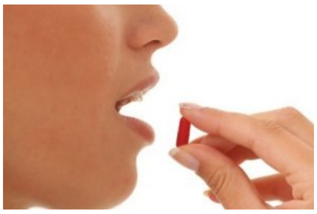	One tablet should be taken once every day, at night with cold water.<br>You need to take this medicine for 10 years.<br>You need to regularly visit a GP to receive a prescription.<br>You need to visit a pharmacy to fill a prescription.<br>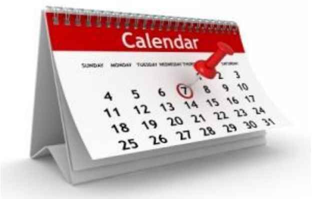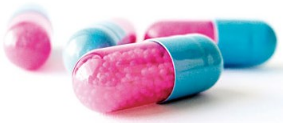

If you take Medicine A, you would live for **1 year** and then die.

If you take Medicine B, you would live for **10 years** and then die.

Would you prefer Medicine A or Medicine B, or are they the same?

Indicate your choice here. You can only choose one option:

Live for 1 year

◯ **Medicine A** 1

◯ **Medicine B** Live for 10 years

◯ **The Same - Medicine A and Medicine B are the same.**

0% 100%

Now we are ready to start the survey questions.

0% 100%

For peer review only

Part 2

Question 1

You have been told that you need to take a medicine to reduce the risk of heart disease.

You can choose between two different medicines. We have called these Medicine A and Medicine B.

Medicine A is a pill that you only need to take once.

Medicine B is a pill that you need to take every day for ten years.

Medicine A and Medicine B are equally effective in reducing your risk of developing heart disease.
Medicine A and Medicine B will reduce your risk of having a heart attack or a stroke within the next 10 years from **10%** to **6%**.

In other words, in a crowd of **1,000** people in the UK, **100** are likely to have a heart attack or a stroke if they do not take the medicine. This is reduced to **60** people out of 1,000 who do take the medicine. This information is also illustrated in this picture.

Risk of heart disease

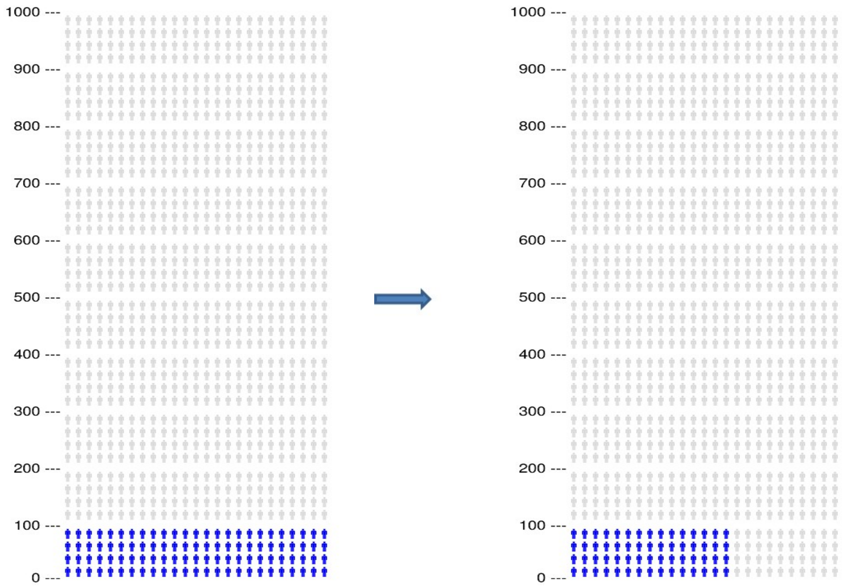

10%
No Treatment

6%
Taking the Medicine

(The figures shaded blue represent people who will have a heart attack or a stroke in the next 10 years)

In this scenario, you will not have any side effects from Medicine A or Medicine B.

Risk of a side effect

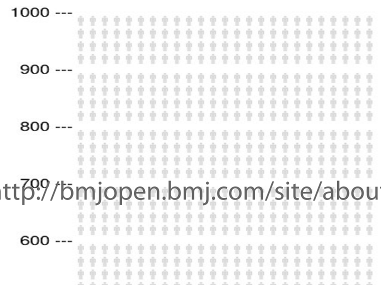

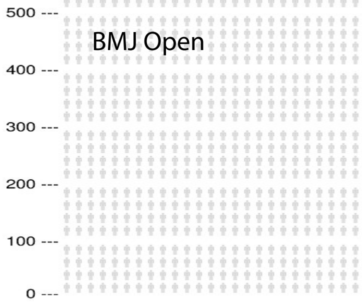

0 %
No one will have a side effect.

0% 100%

peer review only

Repeated section

Remember Medicine A and Medicine B are equally effective and have no risks of a side effect.

The only difference between Medicine A and Medicine B is how you take them.

Medicine A	Medicine B
You take a tablet only once in your life.<br>You will be given the tablet on the day you come in to see your GP.<br>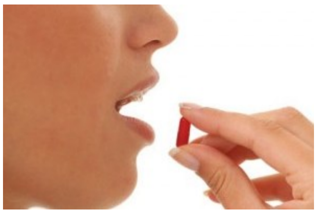	One tablet should be taken once every day, at night with cold water.<br>You need to take this medicine for 10 years.<br>You need to regularly visit a GP to receive a prescription.<br>You need to visit a pharmacy to fill a prescription.<br>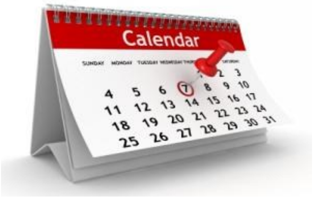<br>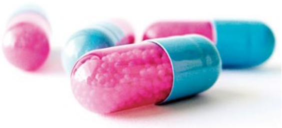

If you take Medicine A, you would live for **5 years** and then die.

If you take Medicine B, you would live for **10 years** and then die.

Would you prefer Medicine A or Medicine B, or are they the same?

Indicate your choice here. You can only choose one option:

- ◯ **Medicine A** — Live for 5 years
- ◯ **Medicine B** — Live for 10 years
- ◯ **Medicine A and Medicine B are the same.**

0% 100%

For peer review only

Repeated section

Remember Medicine A and Medicine B are equally effective and have no risks of a side effect.

The only difference between Medicine A and Medicine B is how you take them.

Medicine A	Medicine B
You take a tablet only once in your life.<br>You will be given the tablet on the day you come in to see your GP.<br>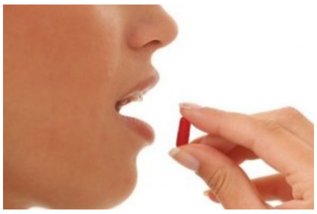	One tablet should be taken once every day, at night with cold water.<br>You need to take this medicine for 10 years.<br>You need to regularly visit a GP to receive a prescription.<br>You need to visit a pharmacy to fill a prescription.<br>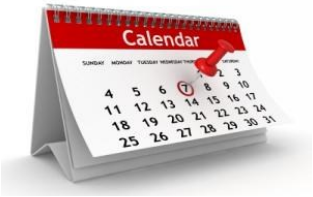<br>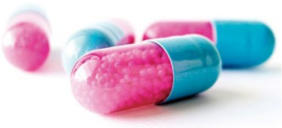

If you take Medicine A, you would live for **5.5 years** (5 years and 6 months) and then die.

If you take Medicine B, you would live for **10 years** and then die.

Would you prefer Medicine A or Medicine B, or are they the same?

Indicate your choice here. You can only choose one option:

◯ **Medicine A** Live for 5.5 years

◯ **Medicine B** Live for 10 years

◯ **Medicine A and Medicine B are the same.**

For peer review only

Repeated section

Remember Medicine A and Medicine B are equally effective and have no risks of a side effect.

The only difference between Medicine A and Medicine B is how you take them.

Medicine A	Medicine B
You take a tablet only once in your life.<br>You will be given the tablet on the day you come in to see your GP.<br>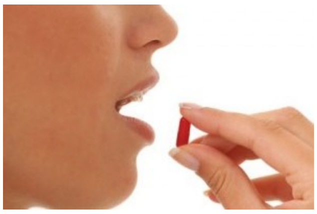	One tablet should be taken once every day, at night with cold water.<br>You need to take this medicine for 10 years.<br>You need to regularly visit a GP to receive a prescription.<br>You need to visit a pharmacy to fill a prescription.<br>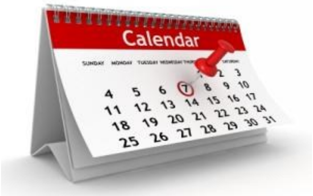<br>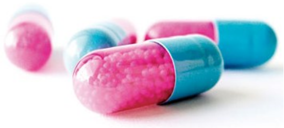

If you take Medicine A, you would live for **6 years** and then die.

If you take Medicine B, you would live for **10 years** and then die.

Would you prefer Medicine A or Medicine B, or are they the same?

Indicate your choice here. You can only choose one option:

◯ **Medicine A** Live for 6 years

◯ **Medicine B** Live for 10 years

◯ **Medicine A and Medicine B are the same.**

0% 100%

For peer review only

Repeated section

Remember Medicine A and Medicine B are equally effective and have no risks of a side effect.

The only difference between Medicine A and Medicine B is how you take them.

**Medicine A**	**Medicine B**
You take a tablet only once in your life. You will be given the tablet on the day you come in to see your GP. 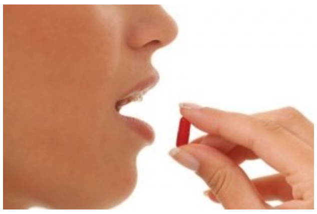	One tablet should be taken once every day, at night with cold water. You need to take this medicine for 10 years. You need to regularly visit a GP to receive a prescription. You need to visit a pharmacy to fill a prescription. 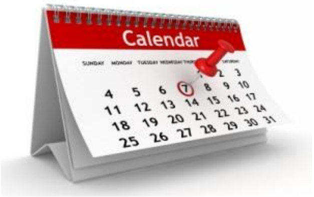 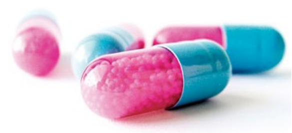

If you take Medicine A, you would live for **6.25 years (6 years and 3 months)** and then die.

If you take Medicine B, you would live for **10 years** and then die.

Would you prefer Medicine A or Medicine B, or are they the same?

Indicate your choice here. You can only choose one option:

◯ **Medicine A**

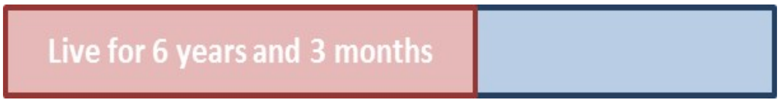

◯ **Medicine B**

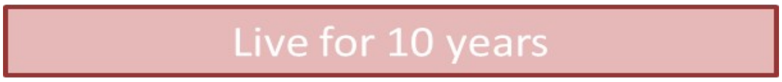

◯ **Medicine A and Medicine B are the same.**

For peer review only

Repeated section

Remember Medicine A and Medicine B are equally effective and have no risks of a side effect.

The only difference between Medicine A and Medicine B is how you take them.

Medicine A	Medicine B
You take a tablet only once in your life.<br>You will be given the tablet on the day you come in to see your GP.<br>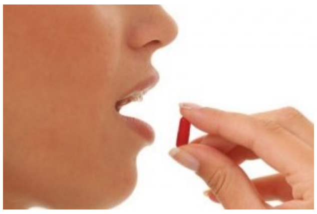	One tablet should be taken once every day, at night with cold water.<br>You need to take this medicine for 10 years.<br>You need to regularly visit a GP to receive a prescription.<br>You need to visit a pharmacy to fill a prescription.<br>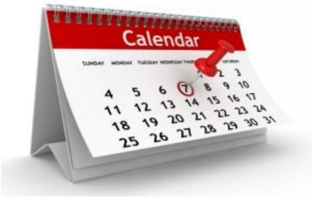<br><br>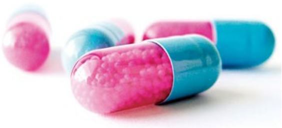

If you take Medicine A, you would live for **6.5 years** (6 years and 6 months) and then die.

If you take Medicine B, you would live for **10 years** and then die.

Would you prefer Medicine A or Medicine B, or are they the same?

Indicate your choice here. You can only choose one option:

◯ **Medicine A** Live for 6.5 years

◯ **Medicine B** Live for 10 years

◯ **Medicine A and Medicine B are the same.**

For peer review only

Repeated section

Remember Medicine A and Medicine B are equally effective and have no risks of a side effect.

The only difference between Medicine A and Medicine B is how you take them.

Medicine A	Medicine B
You take a tablet only once in your life.<br>You will be given the tablet on the day you come in to see your GP.<br>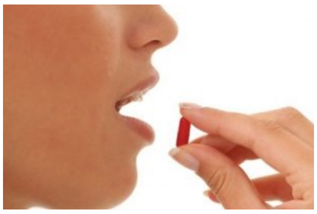	One tablet should be taken once every day, at night with cold water.<br>You need to take this medicine for 10 years.<br>You need to regularly visit a GP to receive a prescription.<br>You need to visit a pharmacy to fill a prescription.<br>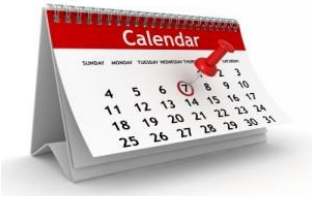<br>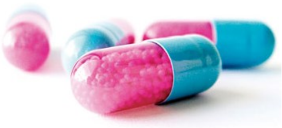

If you take Medicine A, you would live for **7 years** and then die.

If you take Medicine B, you would live for **10 years** and then die.

Would you prefer Medicine A or Medicine B, or are they the same?

Indicate your choice here. You can only choose one option:

○ **Medicine A** Live for 7 years

○ **Medicine B** Live for 10 years

○ **Medicine A and Medicine B are the same.**

For peer review only

Repeated section

Remember Medicine A and Medicine B are equally effective and have no risks of a side effect.

The only difference between Medicine A and Medicine B is how you take them.

**Medicine A**	**Medicine B**
You take a tablet only once in your life.<br>You will be given the tablet on the day you come in to see your GP.<br>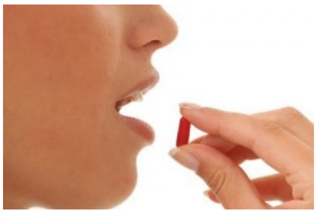	One tablet should be taken once every day, at night with cold water.<br>You need to take this medicine for 10 years.<br>You need to regularly visit a GP to receive a prescription.<br>You need to visit a pharmacy to fill a prescription.<br>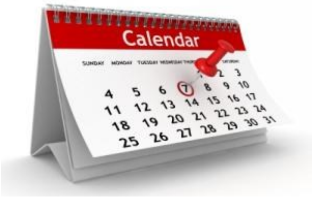<br>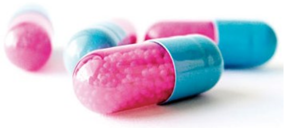

If you take Medicine A, you would live for **7.5 years** (7 years and 6 months) and then die.

If you take Medicine B, you would live for **10 years** and then die.

Would you prefer Medicine A or Medicine B, or are they the same?

Indicate your choice here. You can only choose one option:

○ **Medicine A** Live for 7.5 years

○ **Medicine B** Live for 10 years

○ **Medicine A and Medicine B are the same.**

0% 100%

For peer review only

Repeated section

Remember Medicine A and Medicine B are equally effective and have no risks of a side effect.

The only difference between Medicine A and Medicine B is how you take them.

Medicine A	Medicine B
You take a tablet only once in your life.<br>You will be given the tablet on the day you come in to see your GP.<br>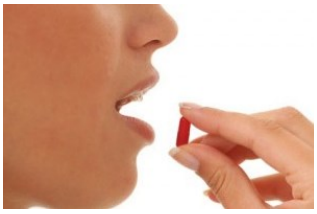	One tablet should be taken once every day, at night with cold water.<br>You need to take this medicine for 10 years.<br>You need to regularly visit a GP to receive a prescription.<br>You need to visit a pharmacy to fill a prescription.<br>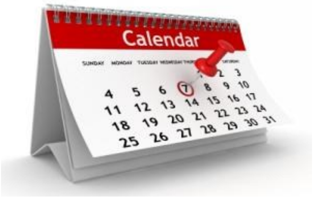<br>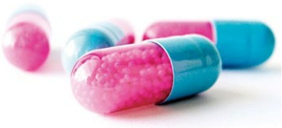

If you take Medicine A, you would live for **7.75 years (7 years and 9 months)** and then die.

If you take Medicine B, you would live for **10 years** and then die.

Would you prefer Medicine A or Medicine B, or are they the same?

Indicate your choice here. You can only choose one option:

◯ **Medicine A** Live for 7 years and 9 months

◯ **Medicine B** Live for 10 years

◯ **Medicine A and Medicine B are the same.**

0% 100%

For peer review only

Repeated section

Remember Medicine A and Medicine B are equally effective and have no risks of a side effect.

The only difference between Medicine A and Medicine B is how you take them.

Medicine A	Medicine B
You take a tablet only once in your life.<br>You will be given the tablet on the day you come in to see your GP.<br>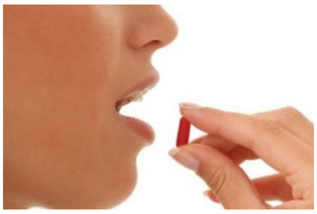	One tablet should be taken once every day, at night with cold water.<br>You need to take this medicine for 10 years.<br>You need to regularly visit a GP to receive a prescription.<br>You need to visit a pharmacy to fill a prescription.<br>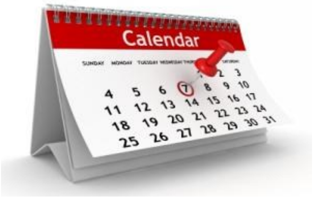<br>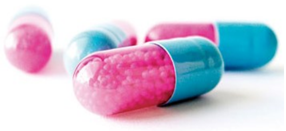

If you take Medicine A, you would live for **8 years** and then die.

If you take Medicine B, you would live for **10 years** and then die.

Would you prefer Medicine A or Medicine B, or are they the same?

Indicate your choice here. You can only choose one option:

◯ **Medicine A** Live for 8 years

◯ **Medicine B** Live for 10 years

◯ **Medicine A and Medicine B are the same.**

For peer review only

Repeated section

Remember Medicine A and Medicine B are equally effective and have no risks of a side effect.

The only difference between Medicine A and Medicine B is how you take them.

Medicine A	Medicine B
You take a tablet only once in your life.<br>You will be given the tablet on the day you come in to see your GP.<br>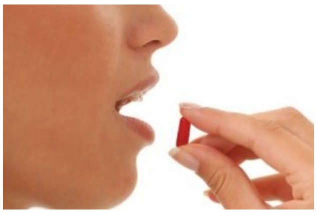	One tablet should be taken once every day, at night with cold water.<br>You need to take this medicine for 10 years.<br>You need to regularly visit a GP to receive a prescription.<br>You need to visit a pharmacy to fill a prescription.<br>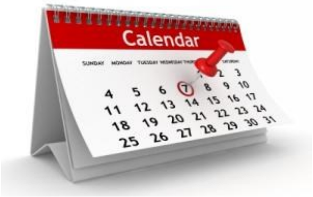<br>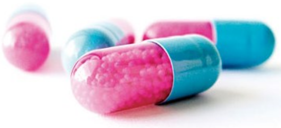

If you take Medicine A, you would live for **8.25 years (8 years and 3 months)** and then die.

If you take Medicine B, you would live for **10 years** and then die.

Would you prefer Medicine A or Medicine B, or are they the same?

Indicate your choice here. You can only choose one option:

○ **Medicine A** Live for 8 years and 3 months

○ **Medicine B** Live for 10 years

○ **Medicine A and Medicine B are the same.**

0% 100%

For peer review only

Repeated section

Remember Medicine A and Medicine B are equally effective and have no risks of a side effect.

The only difference between Medicine A and Medicine B is how you take them.

Medicine A	Medicine B
You take a tablet only once in your life.<br>You will be given the tablet on the day you come in to see your GP.<br>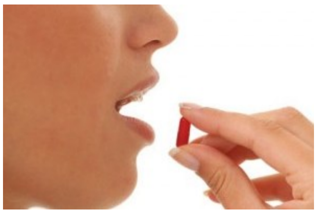	One tablet should be taken once every day, at night with cold water.<br>You need to take this medicine for 10 years.<br>You need to regularly visit a GP to receive a prescription.<br>You need to visit a pharmacy to fill a prescription.<br>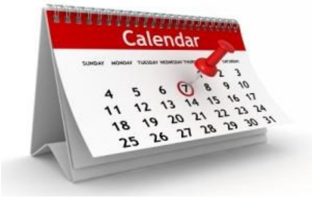<br><br>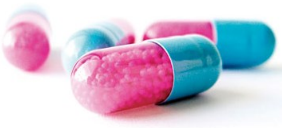

If you take Medicine A, you would live for **8.5 years** (8 years and 6 months) and then die.

If you take Medicine B, you would live for **10 years** and then die.

Would you prefer Medicine A or Medicine B, or are they the same?

Indicate your choice here. You can only choose one option:

◯ **Medicine A** Live for 8.5 years

◯ **Medicine B** Live for 10 years

◯ **Medicine A and Medicine B are the same.**

0% 100%

For peer review only

Repeated section

Remember Medicine A and Medicine B are equally effective and have no risks of a side effect.

The only difference between Medicine A and Medicine B is how you take them.

Medicine A	Medicine B
You take a tablet only once in your life.<br>You will be given the tablet on the day you come in to see your GP.<br>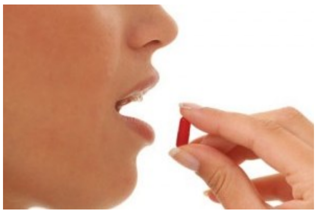	One tablet should be taken once every day, at night with cold water.<br>You need to take this medicine for 10 years.<br>You need to regularly visit a GP to receive a prescription.<br>You need to visit a pharmacy to fill a prescription.<br>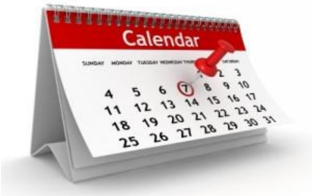<br>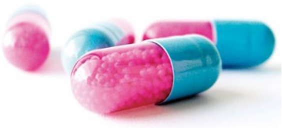

If you take Medicine A, you would live for **8.75 years (8 years and 9 months)** and then die.

If you take Medicine B, you would live for **10 years** and then die.

Would you prefer Medicine A or Medicine B, or are they the same?

Indicate your choice here. You can only choose one option:

○ **Medicine A**

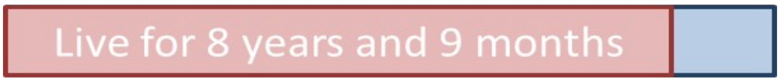

○ **Medicine B**

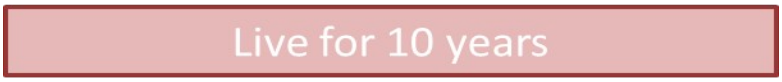

○ **Medicine A and Medicine B are the same.**

For peer review only

Repeated section

Remember Medicine A and Medicine B are equally effective and have no risks of a side effect.

The only difference between Medicine A and Medicine B is how you take them.

**Medicine A**	**Medicine B**
You take a tablet only once in your life.<br>You will be given the tablet on the day you come in to see your GP.<br>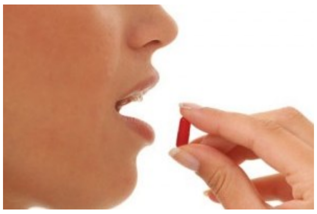	One tablet should be taken once every day, at night with cold water.<br>You need to take this medicine for 10 years.<br>You need to regularly visit a GP to receive a prescription.<br>You need to visit a pharmacy to fill a prescription.<br>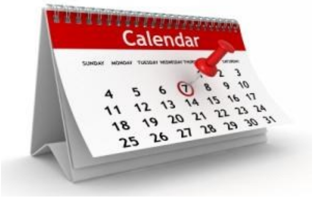<br>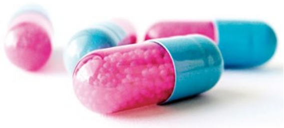

If you take Medicine A, you would live for **9 years** and then die.

If you take Medicine B, you would live for **10 years** and then die.

Would you prefer Medicine A or Medicine B, or are they the same?

Indicate your choice here. You can only choose one option:

◯ **Medicine A** Live for 9 years

◯ **Medicine B** Live for 10 years

◯ **Medicine A and Medicine B are the same.**

0% 100%

For peer review only

Repeated section

Remember Medicine A and Medicine B are equally effective and have no risks of a side effect.

The only difference between Medicine A and Medicine B is how you take them.

Medicine A	Medicine B
You take a tablet only once in your life.<br>You will be given the tablet on the day you come in to see your GP.<br>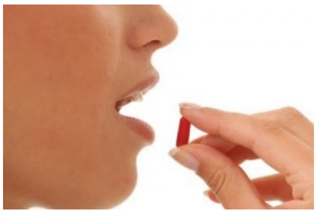	One tablet should be taken once every day, at night with cold water.<br>You need to take this medicine for 10 years.<br>You need to regularly visit a GP to receive a prescription.<br>You need to visit a pharmacy to fill a prescription.<br>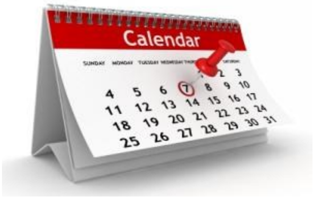<br>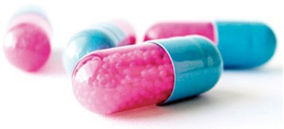

If you take Medicine A, you would live for **9 years and 1 month** and then die.

If you take Medicine B, you would live for **10 years** and then die.

Would you prefer Medicine A or Medicine B, or are they the same?

Indicate your choice here. You can only choose one option:

◯ **Medicine A** Live for 9 years 1 month

◯ **Medicine B** Live for 10 years

◯ **Medicine A and Medicine B are the same.**

0% 100%

For peer review only

Repeated section

Remember Medicine A and Medicine B are equally effective and have no risks of a side effect.

The only difference between Medicine A and Medicine B is how you take them.

**Medicine A**	**Medicine B**
You take a tablet only once in your life.<br>You will be given the tablet on the day you come in to see your GP.<br>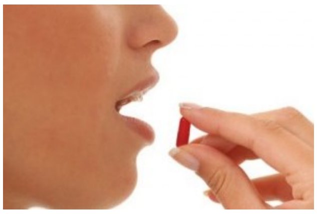	One tablet should be taken once every day, at night with cold water.<br>You need to take this medicine for 10 years.<br>You need to regularly visit a GP to receive a prescription.<br>You need to visit a pharmacy to fill a prescription.<br>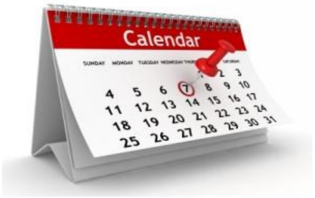 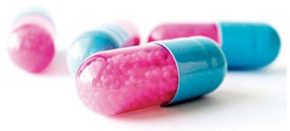

If you take Medicine A, you would live for **9.25 years (9 years and 3 months)** and then die.

If you take Medicine B, you would live for **10 years** and then die.

Would you prefer Medicine A or Medicine B, or are they the same?

Indicate your choice here. You can only choose one option:

◯ **Medicine A** Live for 9 years and 3 months

◯ **Medicine B** Live for 10 years

◯ **Medicine A and Medicine B are the same.**

0% 100%

For peer review only

Repeated section

Remember Medicine A and Medicine B are equally effective and have no risks of a side effect.

The only difference between Medicine A and Medicine B is how you take them.

**Medicine A**	**Medicine B**
You take a tablet only once in your life.<br>You will be given the tablet on the day you come in to see your GP.<br>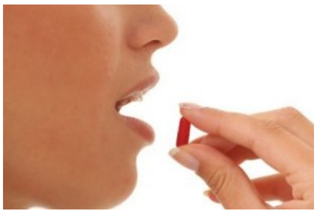	One tablet should be taken once every day, at night with cold water.<br>You need to take this medicine for 10 years.<br>You need to regularly visit a GP to receive a prescription.<br>You need to visit a pharmacy to fill a prescription.<br>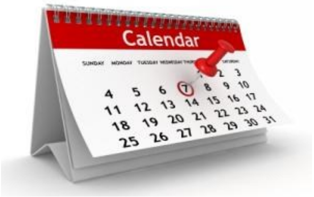<br>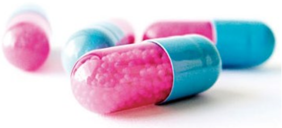

If you take Medicine A, you would live for **9 years and 4 months** and then die.

If you take Medicine B, you would live for **10 years** and then die.

Would you prefer Medicine A or Medicine B, or are they the same?

Indicate your choice here. You can only choose one option:

○ **Medicine A** Live for 9 years 4 months

○ **Medicine B** Live for 10 years

○ **Medicine A and Medicine B are the same.**

For peer review only

Repeated section

Remember Medicine A and Medicine B are equally effective and have no risks of a side effect.

The only difference between Medicine A and Medicine B is how you take them.

**Medicine A**	**Medicine B**
You take a tablet only once in your life.<br>You will be given the tablet on the day you come in to see your GP.<br>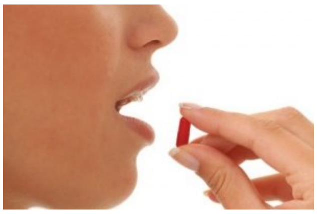	One tablet should be taken once every day, at night with cold water.<br>You need to take this medicine for 10 years.<br>You need to regularly visit a GP to receive a prescription.<br>You need to visit a pharmacy to fill a prescription.<br>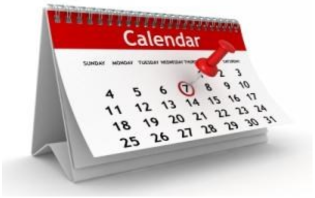<br>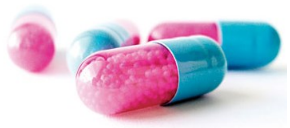

If you take Medicine A, you would live for **9 years and 5 months** and then die.

If you take Medicine B, you would live for **10 years** and then die.

Would you prefer Medicine A or Medicine B, or are they the same?

Indicate your choice here. You can only choose one option:

◯ **Medicine A** Live for 9 years 5 months

◯ **Medicine B** Live for 10 years

◯ **Medicine A and Medicine B are the same.**

For peer review only

Repeated section

Remember Medicine A and Medicine B are equally effective and have no risks of a side effect.

The only difference between Medicine A and Medicine B is how you take them.

Medicine A	Medicine B
You take a tablet only once in your life.<br>You will be given the tablet on the day you come in to see your GP.<br>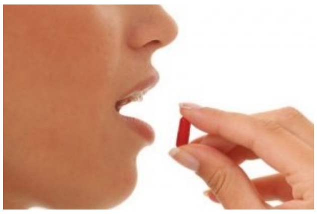	One tablet should be taken once every day, at night with cold water.<br>You need to take this medicine for 10 years.<br>You need to regularly visit a GP to receive a prescription.<br>You need to visit a pharmacy to fill a prescription.<br>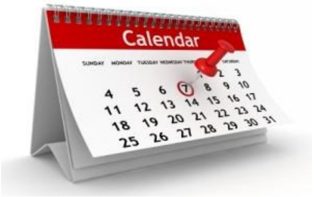<br>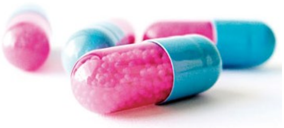

If you take Medicine A, you would live for **9.5 years** (9 years and 6 months) and then die.

If you take Medicine B, you would live for **10 years** and then die.

Would you prefer Medicine A or Medicine B, or are they the same?

Indicate your choice here. You can only choose one option:

○ **Medicine A** Live for 9.5 years

○ **Medicine B** Live for 10 years

○ **Medicine A and Medicine B are the same.**

0% 100%

For peer review only

Repeated section

Remember Medicine A and Medicine B are equally effective and have no risks of a side effect.

The only difference between Medicine A and Medicine B is how you take them.

Medicine A	Medicine B
You take a tablet only once in your life.<br>You will be given the tablet on the day you come in to see your GP.<br>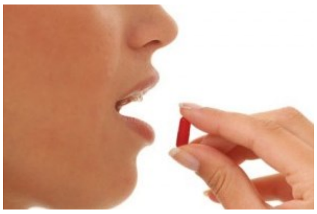	One tablet should be taken once every day, at night with cold water.<br>You need to take this medicine for 10 years.<br>You need to regularly visit a GP to receive a prescription.<br>You need to visit a pharmacy to fill a prescription.<br>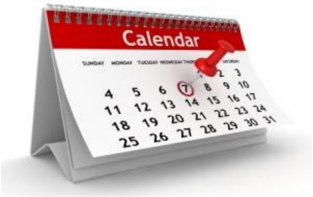<br>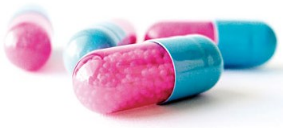

If you take Medicine A, you would live for **5 months less than 10 years** and then die.

If you take Medicine B, you would live for **10 years** and then die.

Would you prefer Medicine A or Medicine B, or are they the same?

Indicate your choice here. You can only choose one option:

◯ **Medicine A** Live for (10 years – 5 months)

◯ **Medicine B** Live for 10 years

◯ **Medicine A and Medicine B are the same.**

0% 100%

For peer review only

Repeated section

Remember Medicine A and Medicine B are equally effective and have no risks of a side effect.

The only difference between Medicine A and Medicine B is how you take them.

Medicine A	Medicine B
You take a tablet only once in your life.<br>You will be given the tablet on the day you come in to see your GP.<br>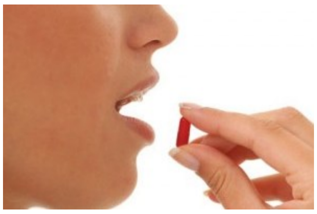	One tablet should be taken once every day, at night with cold water.<br>You need to take this medicine for 10 years.<br>You need to regularly visit a GP to receive a prescription.<br>You need to visit a pharmacy to fill a prescription.<br>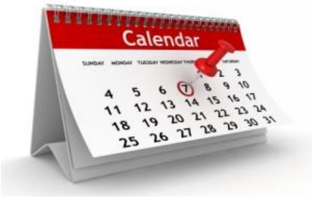<br>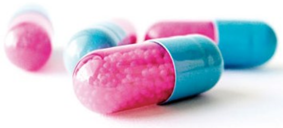

If you take Medicine A, you would live for **4 months less than 10 years** and then die.

If you take Medicine B, you would live for **10 years** and then die.

Would you prefer Medicine A or Medicine B, or are they the same?

Indicate your choice here. You can only choose one option:

◯ **Medicine A** Live for (10 years – 4 months)

◯ **Medicine B** Live for 10 years

◯ **Medicine A and Medicine B are the same.**

For peer review only

Repeated section

Remember Medicine A and Medicine B are equally effective and have no risks of a side effect.

The only difference between Medicine A and Medicine B is how you take them.

Medicine A	Medicine B
You take a tablet only once in your life.<br>You will be given the tablet on the day you come in to see your GP.<br>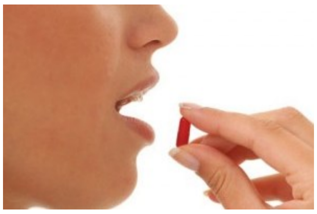	One tablet should be taken once every day, at night with cold water.<br>You need to take this medicine for 10 years.<br>You need to regularly visit a GP to receive a prescription.<br>You need to visit a pharmacy to fill a prescription.<br>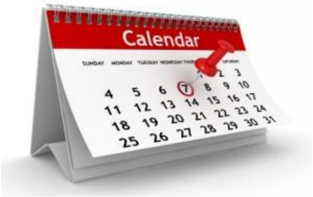<br>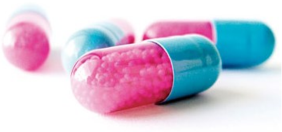

If you take Medicine A, you would live for **3 months less than 10 years** and then die.

If you take Medicine B, you would live for **10 years** and then die.

Would you prefer Medicine A or Medicine B, or are they the same?

Indicate your choice here. You can only choose one option:

◯ **Medicine A** Live for (10 years – 3 months)

◯ **Medicine B** Live for 10 years

◯ **Medicine A and Medicine B are the same.**

0% 100%

For peer review only

Repeated section

Remember Medicine A and Medicine B are equally effective and have no risks of a side effect.

The only difference between Medicine A and Medicine B is how you take them.

Medicine A	Medicine B
You take a tablet only once in your life.<br>You will be given the tablet on the day you come in to see your GP.<br>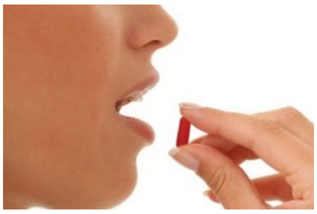	One tablet should be taken once every day, at night with cold water.<br>You need to take this medicine for 10 years.<br>You need to regularly visit a GP to receive a prescription.<br>You need to visit a pharmacy to fill a prescription.<br>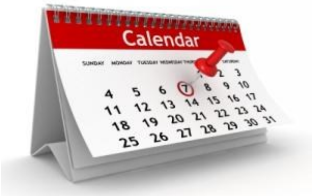<br>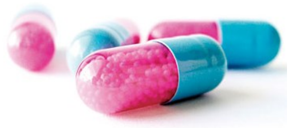

If you take Medicine A, you would live for **2 months less than 10 years** and then die.

If you take Medicine B, you would live for **10 years** and then die.

Would you prefer Medicine A or Medicine B, or are they the same?

Indicate your choice here. You can only choose one option:

◯ **Medicine A** Live for (10 years – 2 months)

◯ **Medicine B** Live for 10 years

◯ **Medicine A and Medicine B are the same.**

For peer review only

Repeated section

Remember Medicine A and Medicine B are equally effective and have no risks of a side effect.

The only difference between Medicine A and Medicine B is how you take them.

Medicine A	Medicine B
You take a tablet only once in your life.<br>You will be given the tablet on the day you come in to see your GP.<br>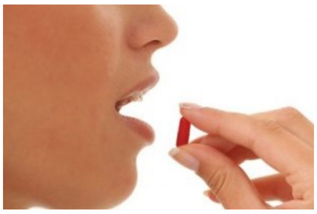	One tablet should be taken once every day, at night with cold water.<br>You need to take this medicine for 10 years.<br>You need to regularly visit a GP to receive a prescription.<br>You need to visit a pharmacy to fill a prescription.<br>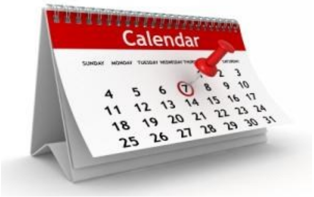<br>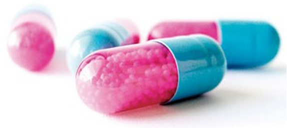

If you take Medicine A, you would live for **1 month less than 10 years** and then die.

If you take Medicine B, you would live for **10 years** and then die.

Would you prefer Medicine A or Medicine B, or are they the same?

Indicate your choice here. You can only choose one option:

◯ **Medicine A** Live for (10 years – 1 month)

◯ **Medicine B** Live for 10 years

◯ **Medicine A and Medicine B are the same.**

0% 100%

For peer review only

For peer review only - http://bmjopen.bmj.com/site/about/guidelines.xhtml

Repeated section

Remember Medicine A and Medicine B are equally effective and have no risks of a side effect.

The only difference between Medicine A and Medicine B is how you take them.

**Medicine A**	**Medicine B**
You take a tablet only once in your life.<br>You will be given the tablet on the day you come in to see your GP.<br>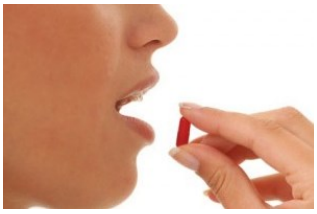	One tablet should be taken once every day, at night with cold water.<br>You need to take this medicine for 10 years.<br>You need to regularly visit a GP to receive a prescription.<br>You need to visit a pharmacy to fill a prescription.<br>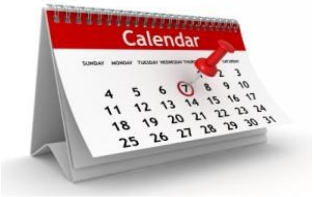<br>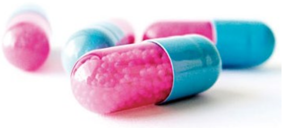

If you take Medicine A, you would live for **3 weeks less than 10 years** and then die.

If you take Medicine B, you would live for **10 years** and then die.

Would you prefer Medicine A or Medicine B, or are they the same?

Indicate your choice here. You can only choose one option:

◯ **Medicine A**

◯ **Medicine B** Live for 10 years

◯ **Medicine A and Medicine B are the same.**

0% 100%

For peer review only

Repeated section

Remember Medicine A and Medicine B are equally effective and have no risks of a side effect.

The only difference between Medicine A and Medicine B is how you take them.

**Medicine A**	**Medicine B**
You take a tablet only once in your life.<br>You will be given the tablet on the day you come in to see your GP.<br>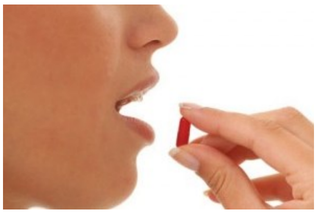	One tablet should be taken once every day, at night with cold water.<br>You need to take this medicine for 10 years.<br>You need to regularly visit a GP to receive a prescription.<br>You need to visit a pharmacy to fill a prescription.<br>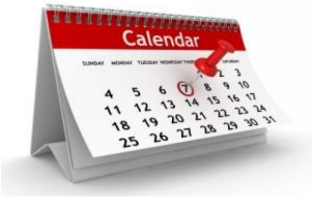<br>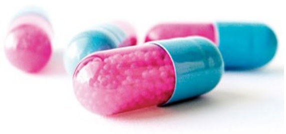

If you take Medicine A, you would live for **2 weeks less than 10 years** and then die.

If you take Medicine B, you would live for **10 years** and then die.

Would you prefer Medicine A or Medicine B, or are they the same?

Indicate your choice here. You can only choose one option:

◯ **Medicine A** Live for (10 years – 2 weeks)

◯ **Medicine B** Live for 10 years

◯ **Medicine A and Medicine B are the same.**

For peer review only

Repeated section

Remember Medicine A and Medicine B are equally effective and have no risks of a side effect.

The only difference between Medicine A and Medicine B is how you take them.

Medicine A	Medicine B
You take a tablet only once in your life.<br>You will be given the tablet on the day you come in to see your GP.<br>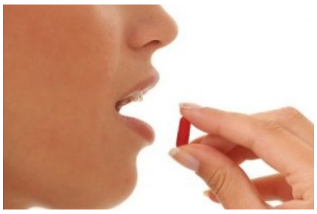	One tablet should be taken once every day, at night with cold water.<br>You need to take this medicine for 10 years.<br>You need to regularly visit a GP to receive a prescription.<br>You need to visit a pharmacy to fill a prescription.<br>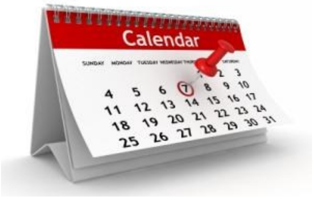 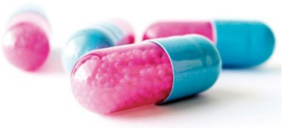

If you take Medicine A, you would live for **10 days less than 10 years** and then die.

If you take Medicine B, you would live for **10 years** and then die.

Would you prefer Medicine A or Medicine B, or are they the same?

Indicate your choice here. You can only choose one option:

○ **Medicine A** Live for (10 years – 10 days)

○ **Medicine B** Live for 10 years

○ **Medicine A and Medicine B are the same.**

For peer review only

Repeated section

Remember Medicine A and Medicine B are equally effective and have no risks of a side effect.

The only difference between Medicine A and Medicine B is how you take them.

Medicine A	Medicine B
You take a tablet only once in your life.<br>You will be given the tablet on the day you come in to see your GP.<br>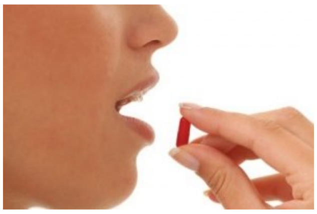	One tablet should be taken once every day, at night with cold water.<br>You need to take this medicine for 10 years.<br>You need to regularly visit a GP to receive a prescription.<br>You need to visit a pharmacy to fill a prescription.<br>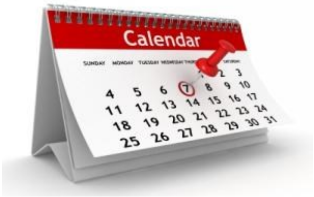<br><br>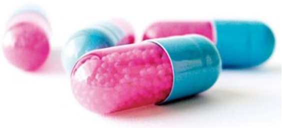

If you take Medicine A, you would live for **1 week less than 10 years** and then die.

If you take Medicine B, you would live for **10 years** and then die.

Would you prefer Medicine A or Medicine B, or are they the same?

Indicate your choice here. You can only choose one option:

◯ **Medicine A** Live for (10 years – 1 week)

◯ **Medicine B** Live for 10 years

◯ **Medicine A and Medicine B are the same.**

For peer review only

Repeated section

Remember Medicine A and Medicine B are equally effective and have no risks of a side effect.

The only difference between Medicine A and Medicine B is how you take them.

**Medicine A**	**Medicine B**
You take a tablet only once in your life.<br>You will be given the tablet on the day you come in to see your GP.<br>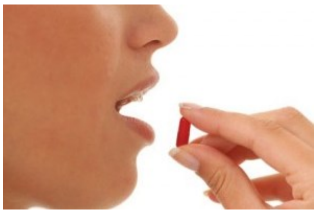	One tablet should be taken once every day, at night with cold water.<br>You need to take this medicine for 10 years.<br>You need to regularly visit a GP to receive a prescription.<br>You need to visit a pharmacy to fill a prescription.<br>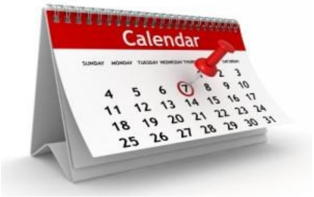<br><br>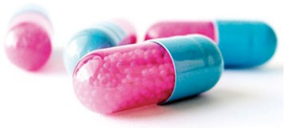

If you take Medicine A, you would live for **5 days less than 10 years** and then die.

If you take Medicine B, you would live for **10 years** and then die.

Would you prefer Medicine A or Medicine B, or are they the same?

Indicate your choice here. You can only choose one option:

○ **Medicine A** Live for (10 years – 5 days)

○ **Medicine B** Live for 10 years

○ **Medicine A and Medicine B are the same.**

0% 100%

For peer review only

Repeated section

Remember Medicine A and Medicine B are equally effective and have no risks of a side effect.

The only difference between Medicine A and Medicine B is how you take them.

Medicine A	Medicine B
You take a tablet only once in your life.<br>You will be given the tablet on the day you come in to see your GP.<br>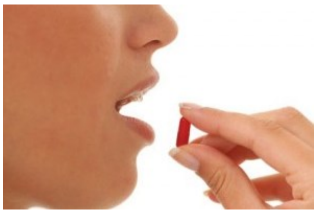	One tablet should be taken once every day, at night with cold water.<br>You need to take this medicine for 10 years.<br>You need to regularly visit a GP to receive a prescription.<br>You need to visit a pharmacy to fill a prescription.<br>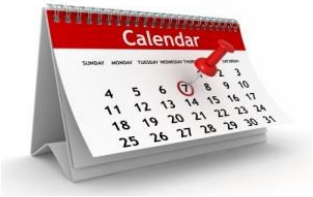<br>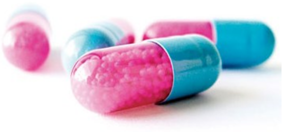

If you take Medicine A, you would live for **3 days less than 10 years** and then die.

If you take Medicine B, you would live for **10 years** and then die.

Would you prefer Medicine A or Medicine B, or are they the same?

Indicate your choice here. You can only choose one option:

◯ **Medicine A** Live for (10 years – 3 days)

◯ **Medicine B** Live for 10 years

◯ **Medicine A and Medicine B are the same.**

For peer review only

Repeated section

Remember Medicine A and Medicine B are equally effective and have no risks of a side effect.

The only difference between Medicine A and Medicine B is how you take them.

Medicine A	Medicine B
You take a tablet only once in your life.<br>You will be given the tablet on the day you come in to see your GP.<br>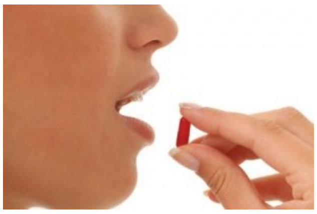	One tablet should be taken once every day, at night with cold water.<br>You need to take this medicine for 10 years.<br>You need to regularly visit a GP to receive a prescription.<br>You need to visit a pharmacy to fill a prescription.<br>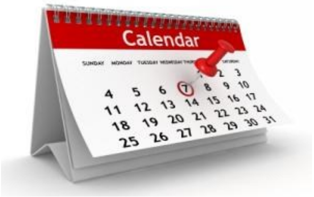 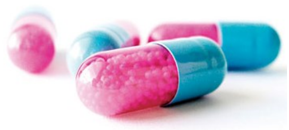

If you take Medicine A, you would live for **1 day less than 10 years** and then die.

If you take Medicine B, you would live for **10 years** and then die.

Would you prefer Medicine A or Medicine B, or are they the same?

Indicate your choice here. You can only choose one option:

○ **Medicine A** Live for (10 years – 1 day)

○ **Medicine B** Live for 10 years

○ **Medicine A and Medicine B are the same.**

0% 100%

For peer review only

Question 1a.

You prefer living for 5 years on Medicine A, to living for 10 years on Medicine B.

This means **living fewer than 5 years on Medicine A** can be the same as living for 10 years on Medicine B.

How many years on Medicine A do you think would be the same as living for 10 years on Medicine B?

(Please give a number in years)

0% 100%

peer review only

Part 2

Question 2

Now, we will assume that there is some risk of a minor side effect from either medicine.

As before,

Medicine A is a pill that you only need to take once.

Medicine B is a pill that you need to take every day for ten years.

Medicine A and Medicine B are equally effective in reducing your risk of developing heart disease.

Medicine A and Medicine B will reduce your risk of having a heart attack or a stroke within the next 10 years from **10%** to **6%**.

In other words, in a crowd of 1,000 people in the UK, **100** are likely to have a heart attack or a stroke if they do not take the medicine. This is reduced to **60** people out of 1,000 who do take the medicine. This information is also illustrated in this picture.

Risk of heart disease

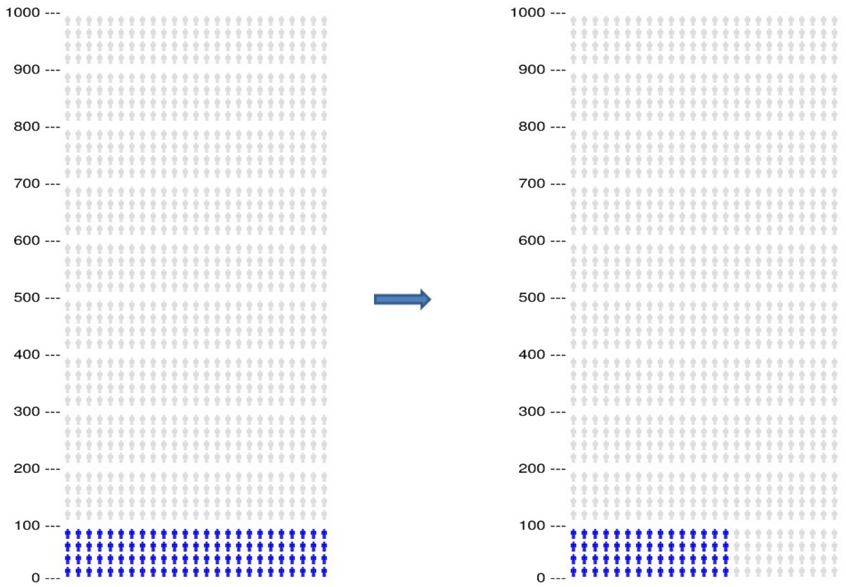

10%
No Treatment

6%
Taking the Medicine

(The figures shaded blue represent people who will have a heart attack or a stroke in the next 10 years)

In this scenario, there is some risk of a minor side effect from Medicine A or Medicine B.

Among every 1,000 people taking Medicine A or Medicine B, **30** people are likely to have a minor side effect such as muscle pain and fatigue.

Risk of a minor side effect

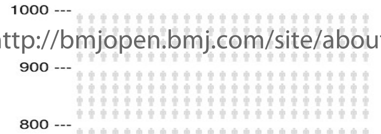

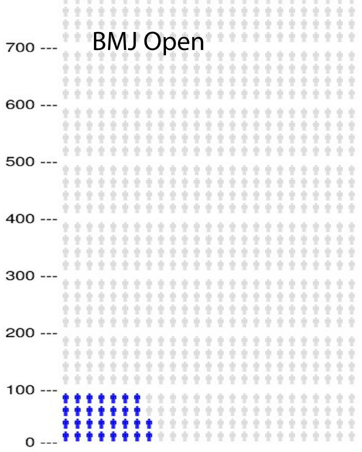

3% of people will have a minor side effect.

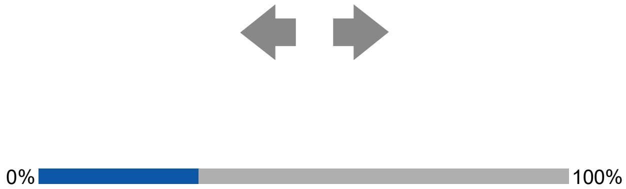

For peer review only

Repeated section

Remember Medicine A and Medicine B are equally effective and have **some risk of a side effect**.

The only difference between Medicine A and Medicine B is how you take them.

**Medicine A**	**Medicine B**
You take a tablet only once in your life.<br>You will be given the tablet on the day you come in to see your GP.<br>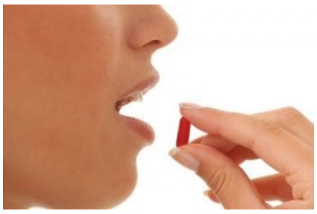	One tablet should be taken once every day, at night with cold water.<br>You need to take this medicine for 10 years.<br>You need to regularly visit a GP to receive a prescription.<br>You need to visit a pharmacy to fill a prescription.<br>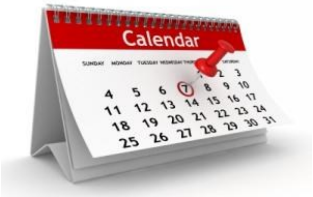<br>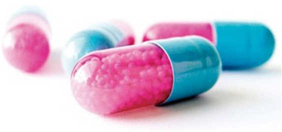

If you take Medicine A, you would live for **5 years** and then die.

If you take Medicine B, you would live for **10 years** and then die.

Would you prefer Medicine A or Medicine B, or are they the same?

Indicate your choice here. You can only choose one option:

◯ **Medicine A** Live for 5 years

◯ **Medicine B** Live for 10 years

◯ **Medicine A and Medicine B are the same.**

For peer review only

Repeated section

Remember Medicine A and Medicine B are equally effective and have **some risk of a side effect**.

The only difference between Medicine A and Medicine B is how you take them.

**Medicine A**	**Medicine B**
You take a tablet only once in your life.<br>You will be given the tablet on the day you come in to see your GP.<br>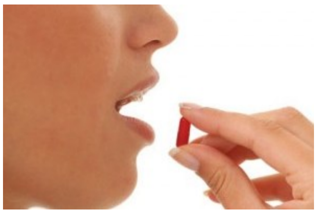	One tablet should be taken once every day, at night with cold water.<br>You need to take this medicine for 10 years.<br>You need to regularly visit a GP to receive a prescription.<br>You need to visit a pharmacy to fill a prescription.<br>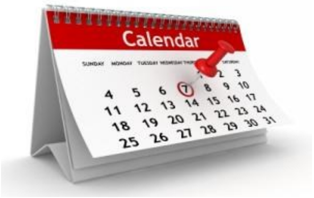<br>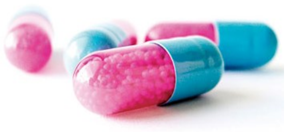

If you take Medicine A, you would live for **5.5 years** (5 years and 6 months) and then die.

If you take Medicine B, you would live for **10 years** and then die.

Would you prefer Medicine A or Medicine B, or are they the same?

Indicate your choice here. You can only choose one option:

○ **Medicine A** Live for 5.5 years

○ **Medicine B** Live for 10 years

○ **Medicine A and Medicine B are the same.**

For peer review only

Repeated section

Remember Medicine A and Medicine B are equally effective and have **some risk of a side effect**.

The only difference between Medicine A and Medicine B is how you take them.

**Medicine A**	**Medicine B**
You take a tablet only once in your life.<br>You will be given the tablet on the day you come in to see your GP.<br>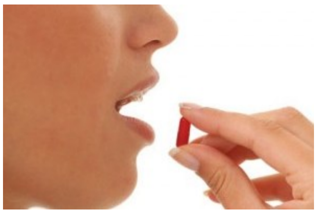	One tablet should be taken once every day, at night with cold water.<br>You need to take this medicine for 10 years.<br>You need to regularly visit a GP to receive a prescription.<br>You need to visit a pharmacy to fill a prescription.<br>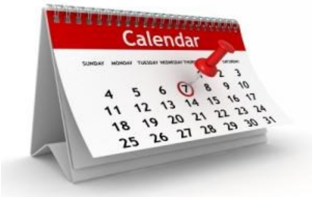<br>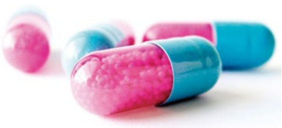

If you take Medicine A, you would live for **6 years** and then die.

If you take Medicine B, you would live for **10 years** and then die.

Would you prefer Medicine A or Medicine B, or are they the same?

Indicate your choice here. You can only choose one option:

◯ **Medicine A** Live for 6 years

◯ **Medicine B** Live for 10 years

◯ **Medicine A and Medicine B are the same.**

For peer review only

Repeated section

Remember Medicine A and Medicine B are equally effective and have **some risk of a side effect**.

The only difference between Medicine A and Medicine B is how you take them.

Medicine A	Medicine B
You take a tablet only once in your life.<br>You will be given the tablet on the day you come in to see your GP.<br>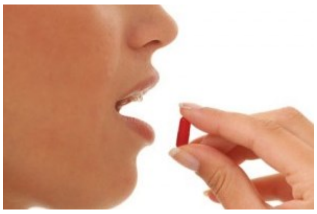	One tablet should be taken once every day, at night with cold water.<br>You need to take this medicine for 10 years.<br>You need to regularly visit a GP to receive a prescription.<br>You need to visit a pharmacy to fill a prescription.<br>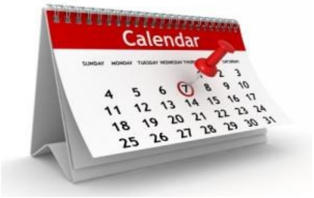<br>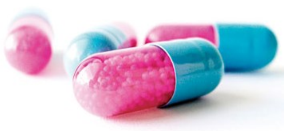

If you take Medicine A, you would live for **6.25 years (6 years and 3 months)** and then die.

If you take Medicine B, you would live for **10 years** and then die.

Would you prefer Medicine A or Medicine B, or are they the same?

Indicate your choice here. You can only choose one option:

○ **Medicine A** Live for 6 years and 3 months

○ **Medicine B** Live for 10 years

○ **Medicine A and Medicine B are the same.**

0% 100%

For peer review only

Repeated section

Remember Medicine A and Medicine B are equally effective and have **some risk of a side effect**.

The only difference between Medicine A and Medicine B is how you take them.

Medicine A	Medicine B
You take a tablet only once in your life.<br>You will be given the tablet on the day you come in to see your GP.<br>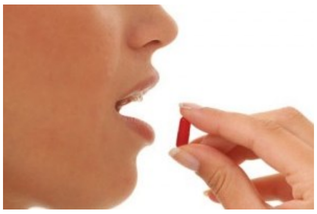	One tablet should be taken once every day, at night with cold water.<br>You need to take this medicine for 10 years.<br>You need to regularly visit a GP to receive a prescription.<br>You need to visit a pharmacy to fill a prescription.<br>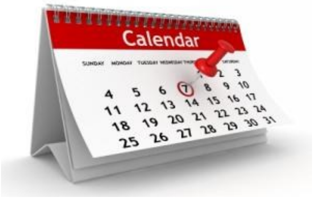<br><br>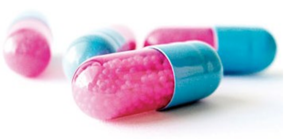

If you take Medicine A, you would live for **6.5 years** (6 years and 6 months) and then die.

If you take Medicine B, you would live for **10 years** and then die.

Would you prefer Medicine A or Medicine B, or are they the same?

Indicate your choice here. You can only choose one option:

◯ **Medicine A** Live for 6.5 years

◯ **Medicine B** Live for 10 years

◯ **Medicine A and Medicine B are the same.**

For peer review only

Repeated section

Remember Medicine A and Medicine B are equally effective and have **some risk of a side effect**.

The only difference between Medicine A and Medicine B is how you take them.

Medicine A	Medicine B
You take a tablet only once in your life.<br>You will be given the tablet on the day you come in to see your GP.<br>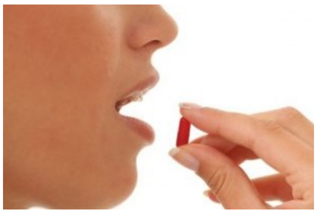	One tablet should be taken once every day, at night with cold water.<br>You need to take this medicine for 10 years.<br>You need to regularly visit a GP to receive a prescription.<br>You need to visit a pharmacy to fill a prescription.<br>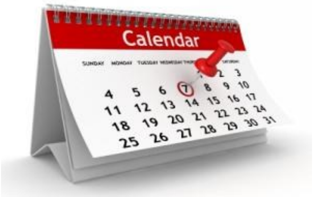 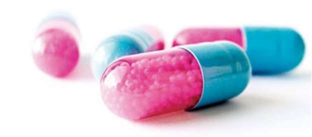

If you take Medicine A, you would live for **7 years** and then die.

If you take Medicine B, you would live for **10 years** and then die.

Would you prefer Medicine A or Medicine B, or are they the same?

Indicate your choice here. You can only choose one option:

◯ **Medicine A** Live for 7 years

◯ **Medicine B** Live for 10 years

◯ **Medicine A and Medicine B are the same.**

For peer review only

Repeated section

Remember Medicine A and Medicine B are equally effective and have **some risk of a side effect**.

The only difference between Medicine A and Medicine B is how you take them.

Medicine A	Medicine B
You take a tablet only once in your life.<br>You will be given the tablet on the day you come in to see your GP.<br>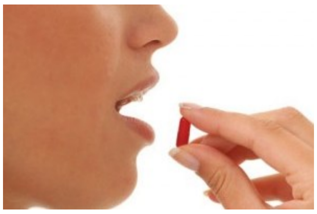	One tablet should be taken once every day, at night with cold water.<br>You need to take this medicine for 10 years.<br>You need to regularly visit a GP to receive a prescription.<br>You need to visit a pharmacy to fill a prescription.<br>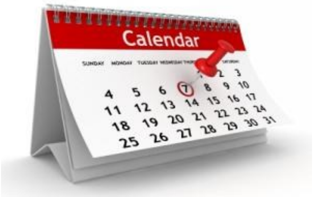<br>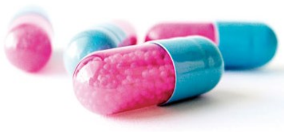

If you take Medicine A, you would live for **7.5 years** (7 years and 6 months) and then die.

If you take Medicine B, you would live for **10 years** and then die.

Would you prefer Medicine A or Medicine B, or are they the same?

Indicate your choice here. You can only choose one option:

○ **Medicine A** Live for 7.5 years

○ **Medicine B** Live for 10 years

○ **Medicine A and Medicine B are the same.**

For peer review only

Repeated section

Remember Medicine A and Medicine B are equally effective and have **some risk of a side effect**.

The only difference between Medicine A and Medicine B is how you take them.

Medicine A	Medicine B
You take a tablet only once in your life.<br>You will be given the tablet on the day you come in to see your GP.<br>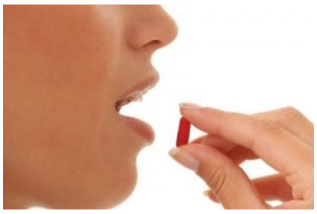	One tablet should be taken once every day, at night with cold water.<br>You need to take this medicine for 10 years.<br>You need to regularly visit a GP to receive a prescription.<br>You need to visit a pharmacy to fill a prescription.<br>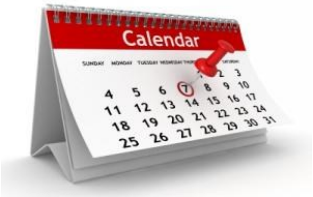<br>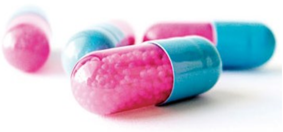

If you take Medicine A, you would live for **7.75 years (7 years and 9 months)** and then die.

If you take Medicine B, you would live for **10 years** and then die.

Would you prefer Medicine A or Medicine B, or are they the same?

Indicate your choice here. You can only choose one option:

○ **Medicine A** Live for 7 years and 9 months

○ **Medicine B** Live for 10 years

○ **Medicine A and Medicine B are the same.**

For peer review only

Repeated section

Remember Medicine A and Medicine B are equally effective and have **some risk of a side effect**.

The only difference between Medicine A and Medicine B is how you take them.

Medicine A	Medicine B
You take a tablet only once in your life.<br>You will be given the tablet on the day you come in to see your GP.<br>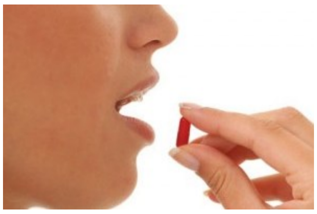	One tablet should be taken once every day, at night with cold water.<br>You need to take this medicine for 10 years.<br>You need to regularly visit a GP to receive a prescription.<br>You need to visit a pharmacy to fill a prescription.<br>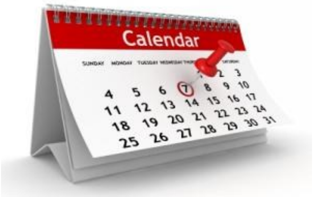<br>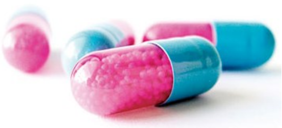

If you take Medicine A, you would live for **8 years** and then die.

If you take Medicine B, you would live for **10 years** and then die.

Would you prefer Medicine A or Medicine B, or are they the same?

Indicate your choice here. You can only choose one option:

◯ **Medicine A** 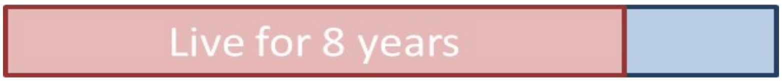

◯ **Medicine B** 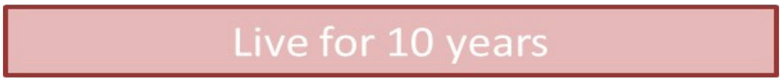

◯ **Medicine A and Medicine B are the same.**

For peer review only

Repeated section

Remember Medicine A and Medicine B are equally effective and have **some risk of a side effect**.

The only difference between Medicine A and Medicine B is how you take them.

Medicine A	Medicine B
You take a tablet only once in your life.<br>You will be given the tablet on the day you come in to see your GP.<br>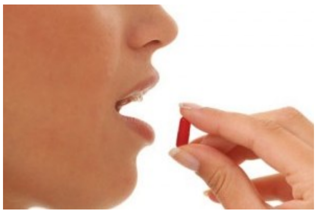	One tablet should be taken once every day, at night with cold water.<br>You need to take this medicine for 10 years.<br>You need to regularly visit a GP to receive a prescription.<br>You need to visit a pharmacy to fill a prescription.<br>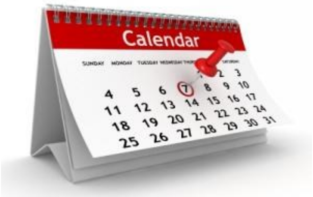<br>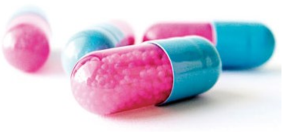

If you take Medicine A, you would live for **8.25 years (8 years and 3 months)** and then die.

If you take Medicine B, you would live for **10 years** and then die.

Would you prefer Medicine A or Medicine B, or are they the same?

Indicate your choice here. You can only choose one option:

◯ **Medicine A** Live for 8 years and 3 months

◯ **Medicine B** Live for 10 years

◯ **Medicine A and Medicine B are the same.**

0% 100%

For peer review only

Repeated section

Remember Medicine A and Medicine B are equally effective and have **some risk of a side effect**.

The only difference between Medicine A and Medicine B is how you take them.

Medicine A	Medicine B
You take a tablet only once in your life.<br>You will be given the tablet on the day you come in to see your GP.<br>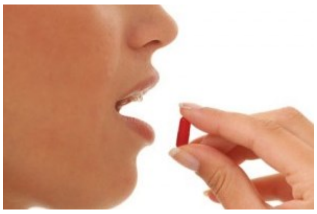	One tablet should be taken once every day, at night with cold water.<br>You need to take this medicine for 10 years.<br>You need to regularly visit a GP to receive a prescription.<br>You need to visit a pharmacy to fill a prescription.<br>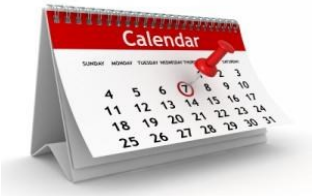<br>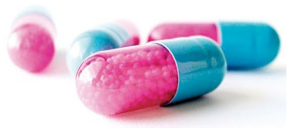

If you take Medicine A, you would live for **8.5 years** (8 years and 6 months) and then die.

If you take Medicine B, you would live for **10 years** and then die.

Would you prefer Medicine A or Medicine B, or are they the same?

Indicate your choice here. You can only choose one option:

◯ **Medicine A** Live for 8.5 years

◯ **Medicine B** Live for 10 years

◯ **Medicine A and Medicine B are the same.**

For peer review only

Repeated section

Remember Medicine A and Medicine B are equally effective and have **some risk of a side effect**.

The only difference between Medicine A and Medicine B is how you take them.

**Medicine A**	**Medicine B**
You take a tablet only once in your life.<br>You will be given the tablet on the day you come in to see your GP.<br>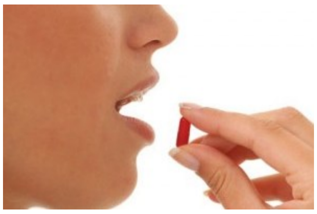	One tablet should be taken once every day, at night with cold water.<br>You need to take this medicine for 10 years.<br>You need to regularly visit a GP to receive a prescription.<br>You need to visit a pharmacy to fill a prescription.<br>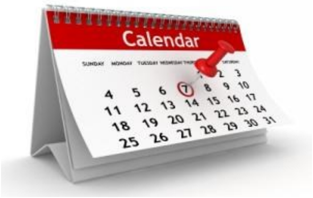<br>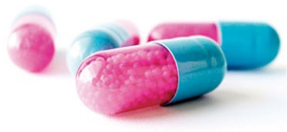

If you take Medicine A, you would live for **8.75 years (8 years and 9 months)** and then die.

If you take Medicine B, you would live for **10 years** and then die.

Would you prefer Medicine A or Medicine B, or are they the same?

Indicate your choice here. You can only choose one option:

○ **Medicine A** Live for 8 years and 9 months

○ **Medicine B** Live for 10 years

○ **Medicine A and Medicine B are the same.**

For peer review only

Repeated section

Remember Medicine A and Medicine B are equally effective and have **some risk of a side effect**.

The only difference between Medicine A and Medicine B is how you take them.

**Medicine A**	**Medicine B**
You take a tablet only once in your life.<br>You will be given the tablet on the day you come in to see your GP.<br>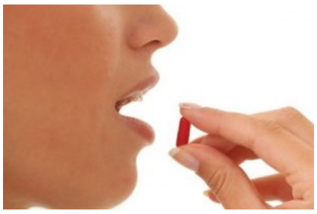	One tablet should be taken once every day, at night with cold water.<br>You need to take this medicine for 10 years.<br>You need to regularly visit a GP to receive a prescription.<br>You need to visit a pharmacy to fill a prescription.<br>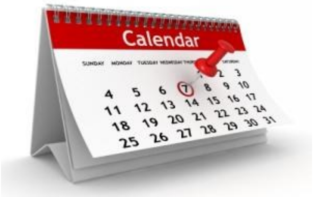<br>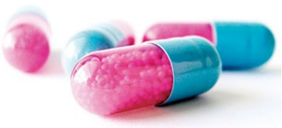

If you take Medicine A, you would live for **9 years** and then die.

If you take Medicine B, you would live for **10 years** and then die.

Would you prefer Medicine A or Medicine B, or are they the same?

Indicate your choice here. You can only choose one option:

○ **Medicine A** Live for 9 years

○ **Medicine B** Live for 10 years

○ **Medicine A and Medicine B are the same.**

0% 100%

For peer review only

Repeated section

Remember Medicine A and Medicine B are equally effective and have **some risk of a side effect**.

The only difference between Medicine A and Medicine B is how you take them.

Medicine A	Medicine B
You take a tablet only once in your life.<br>You will be given the tablet on the day you come in to see your GP.<br>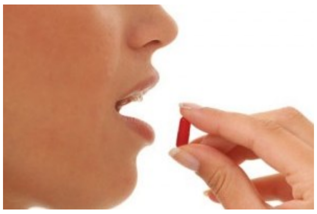	One tablet should be taken once every day, at night with cold water.<br>You need to take this medicine for 10 years.<br>You need to regularly visit a GP to receive a prescription.<br>You need to visit a pharmacy to fill a prescription.<br>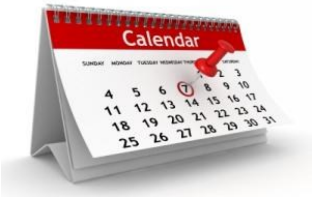<br>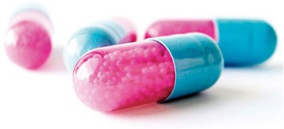

If you take Medicine A, you would live for **9 years and 1 month** and then die.

If you take Medicine B, you would live for **10 years** and then die.

Would you prefer Medicine A or Medicine B, or are they the same?

Indicate your choice here. You can only choose one option:

◯ **Medicine A** Live for 9 years 1 month

◯ **Medicine B** Live for 10 years

◯ **Medicine A and Medicine B are the same.**

For peer review only

Repeated section

Remember Medicine A and Medicine B are equally effective and have **some risk of a side effect**.

The only difference between Medicine A and Medicine B is how you take them.

Medicine A	Medicine B
You take a tablet only once in your life.<br>You will be given the tablet on the day you come in to see your GP.<br>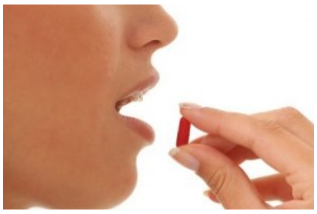	One tablet should be taken once every day, at night with cold water.<br>You need to take this medicine for 10 years.<br>You need to regularly visit a GP to receive a prescription.<br>You need to visit a pharmacy to fill a prescription.<br>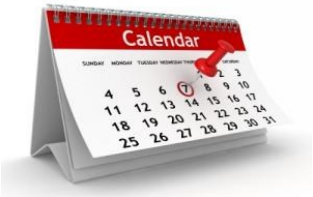<br>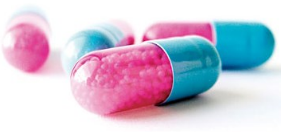

If you take Medicine A, you would live for **9.25 years (9 years and 3 months)** and then die.

If you take Medicine B, you would live for **10 years** and then die.

Would you prefer Medicine A or Medicine B, or are they the same?

Indicate your choice here. You can only choose one option:

◯ **Medicine A** Live for 9 years and 3 months

◯ **Medicine B** Live for 10 years

◯ **Medicine A and Medicine B are the same.**

0% 100%

For peer review only

Repeated section

Remember Medicine A and Medicine B are equally effective and have **some risk of a side effect**.

The only difference between Medicine A and Medicine B is how you take them.

**Medicine A**	**Medicine B**
You take a tablet only once in your life.<br>You will be given the tablet on the day you come in to see your GP.<br>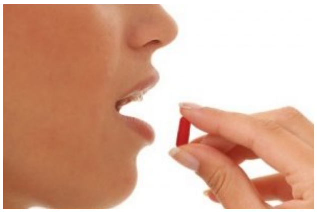	One tablet should be taken once every day, at night with cold water.<br>You need to take this medicine for 10 years.<br>You need to regularly visit a GP to receive a prescription.<br>You need to visit a pharmacy to fill a prescription.<br>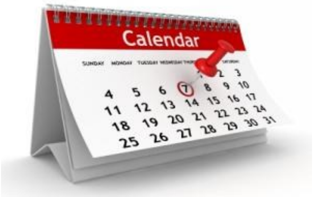<br>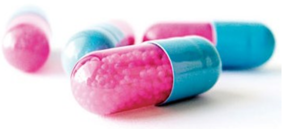

If you take Medicine A, you would live for **9 years and 4 months** and then die.

If you take Medicine B, you would live for **10 years** and then die.

Would you prefer Medicine A or Medicine B, or are they the same?

Indicate your choice here. You can only choose one option:

○ **Medicine A** Live for 9 years 4 months

○ **Medicine B** Live for 10 years

○ **Medicine A and Medicine B are the same.**

For peer review only

Repeated section

Remember Medicine A and Medicine B are equally effective and have **some risk of a side effect**.

The only difference between Medicine A and Medicine B is how you take them.

**Medicine A**	**Medicine B**
You take a tablet only once in your life.<br>You will be given the tablet on the day you come in to see your GP.<br>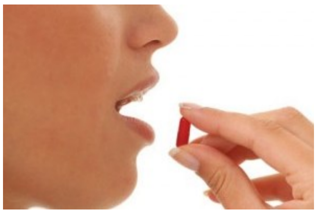	One tablet should be taken once every day, at night with cold water.<br>You need to take this medicine for 10 years.<br>You need to regularly visit a GP to receive a prescription.<br>You need to visit a pharmacy to fill a prescription.<br>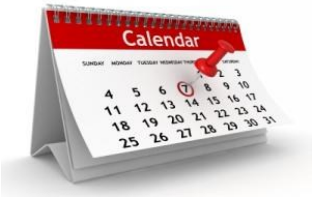<br><br>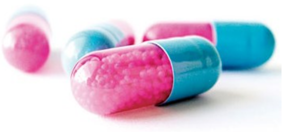

If you take Medicine A, you would live for **9 years and 5 months** and then die.

If you take Medicine B, you would live for **10 years** and then die.

Would you prefer Medicine A or Medicine B, or are they the same?

Indicate your choice here. You can only choose one option:

◯ **Medicine A** Live for 9 years 5 months

◯ **Medicine B** Live for 10 years

◯ **Medicine A and Medicine B are the same.**

For peer review only

Repeated section

Remember Medicine A and Medicine B are equally effective and have **some risk of a side effect**.

The only difference between Medicine A and Medicine B is how you take them.

**Medicine A**	**Medicine B**
You take a tablet only once in your life.<br>You will be given the tablet on the day you come in to see your GP.<br>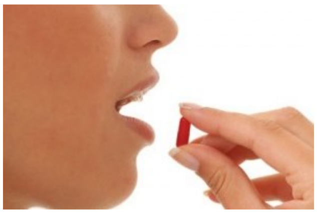	One tablet should be taken once every day, at night with cold water.<br>You need to take this medicine for 10 years.<br>You need to regularly visit a GP to receive a prescription.<br>You need to visit a pharmacy to fill a prescription.<br>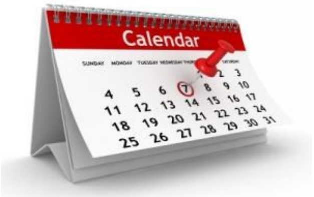<br>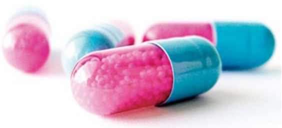

If you take Medicine A, you would live for **9.5 years** (9 years and 6 months) and then die.

If you take Medicine B, you would live for **10 years** and then die.

Would you prefer Medicine A or Medicine B, or are they the same?

Indicate your choice here. You can only choose one option:

◯ **Medicine A** Live for 9.5 years

◯ **Medicine B** Live for 10 years

◯ **Medicine A and Medicine B are the same.**

For peer review only

Repeated section

Remember Medicine A and Medicine B are equally effective and have **some risk of a side effect**.

The only difference between Medicine A and Medicine B is how you take them.

Medicine A	Medicine B
You take a tablet only once in your life.<br>You will be given the tablet on the day you come in to see your GP.<br>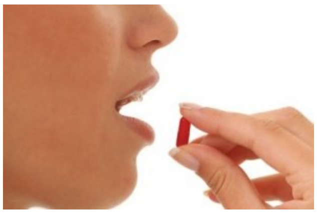	One tablet should be taken once every day, at night with cold water.<br>You need to take this medicine for 10 years.<br>You need to regularly visit a GP to receive a prescription.<br>You need to visit a pharmacy to fill a prescription.<br>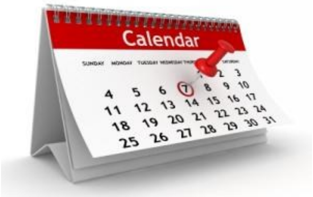<br>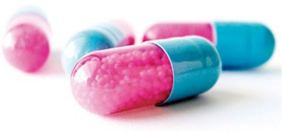

If you take Medicine A, you would live for **5 months less than 10 years** and then die.

If you take Medicine B, you would live for **10 years** and then die.

Would you prefer Medicine A or Medicine B, or are they the same?

Indicate your choice here. You can only choose one option:

○ **Medicine A** Live for (10 years – 5 months)

○ **Medicine B** Live for 10 years

○ **Medicine A and Medicine B are the same.**

For peer review only

Repeated section

Remember Medicine A and Medicine B are equally effective and have **some risk of a side effect**.

The only difference between Medicine A and Medicine B is how you take them.

**Medicine A**	**Medicine B**
You take a tablet only once in your life.<br>You will be given the tablet on the day you come in to see your GP.<br>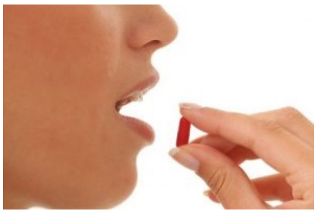	One tablet should be taken once every day, at night with cold water.<br>You need to take this medicine for 10 years.<br>You need to regularly visit a GP to receive a prescription.<br>You need to visit a pharmacy to fill a prescription.<br>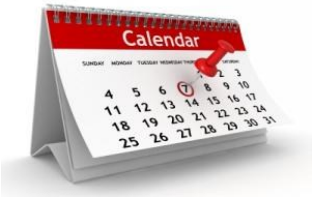<br>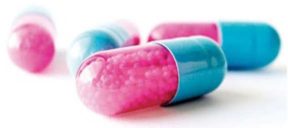

If you take Medicine A, you would live for **4 months less than 10 years** and then die.

If you take Medicine B, you would live for **10 years** and then die.

Would you prefer Medicine A or Medicine B, or are they the same?

Indicate your choice here. You can only choose one option:

◯ **Medicine A** Live for (10 years – 4 months)

◯ **Medicine B** Live for 10 years

◯ **Medicine A and Medicine B are the same.**

For peer review only

Repeated section

Remember Medicine A and Medicine B are equally effective and have **some risk of a side effect**.

The only difference between Medicine A and Medicine B is how you take them.

Medicine A	Medicine B
You take a tablet only once in your life.<br>You will be given the tablet on the day you come in to see your GP.<br>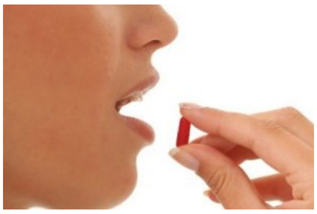	One tablet should be taken once every day, at night with cold water.<br>You need to take this medicine for 10 years.<br>You need to regularly visit a GP to receive a prescription.<br>You need to visit a pharmacy to fill a prescription.<br>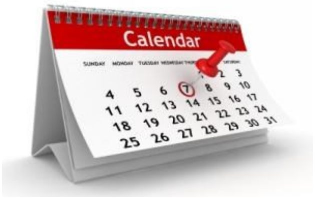<br>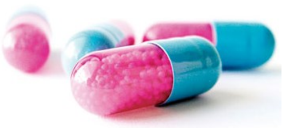

If you take Medicine A, you would live for **3 months less than 10 years** and then die.

If you take Medicine B, you would live for **10 years** and then die.

Would you prefer Medicine A or Medicine B, or are they the same?

Indicate your choice here. You can only choose one option:

○ **Medicine A** Live for (10 years – 3 months)

○ **Medicine B** Live for 10 years

○ **Medicine A and Medicine B are the same.**

For peer review only

Repeated section

Remember Medicine A and Medicine B are equally effective and have **some risk of a side effect**.

The only difference between Medicine A and Medicine B is how you take them.

Medicine A	Medicine B
You take a tablet only once in your life.<br>You will be given the tablet on the day you come in to see your GP.<br>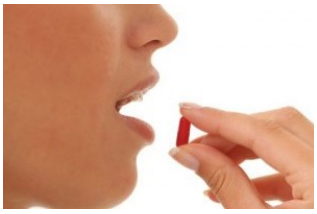	One tablet should be taken once every day, at night with cold water.<br>You need to take this medicine for 10 years.<br>You need to regularly visit a GP to receive a prescription.<br>You need to visit a pharmacy to fill a prescription.<br>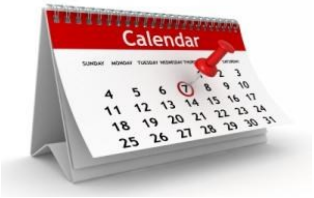<br><br>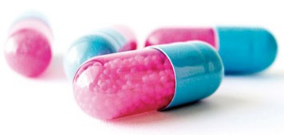

If you take Medicine A, you would live for **2 months less than 10 years** and then die.

If you take Medicine B, you would live for **10 years** and then die.

Would you prefer Medicine A or Medicine B, or are they the same?

Indicate your choice here. You can only choose one option:

◯ **Medicine A** Live for (10 years – 2 months)

◯ **Medicine B** Live for 10 years

◯ **Medicine A and Medicine B are the same.**

0% 100%

For peer review only

Repeated section

Remember Medicine A and Medicine B are equally effective and have **some risk of a side effect**.

The only difference between Medicine A and Medicine B is how you take them.

**Medicine A**	**Medicine B**
You take a tablet only once in your life.<br>You will be given the tablet on the day you come in to see your GP.<br>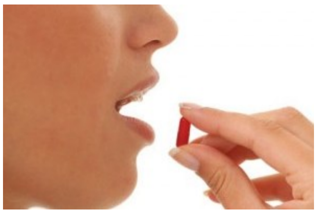	One tablet should be taken once every day, at night with cold water.<br>You need to take this medicine for 10 years.<br>You need to regularly visit a GP to receive a prescription.<br>You need to visit a pharmacy to fill a prescription.<br>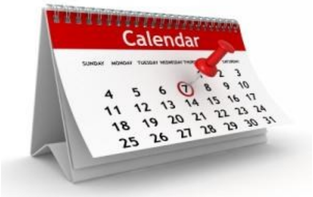<br>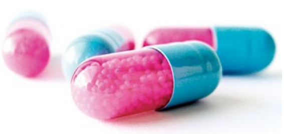

If you take Medicine A, you would live for **1 month less than 10 years** and then die.

If you take Medicine B, you would live for **10 years** and then die.

Would you prefer Medicine A or Medicine B, or are they the same?

Indicate your choice here. You can only choose one option:

○ **Medicine A** — Live for (10 years – 1 month)

○ **Medicine B** — Live for 10 years

○ **Medicine A and Medicine B are the same.**

For peer review only

Repeated section

Remember Medicine A and Medicine B are equally effective and have **some risk of a side effect**.

The only difference between Medicine A and Medicine B is how you take them.

**Medicine A**	**Medicine B**
You take a tablet only once in your life.<br>You will be given the tablet on the day you come in to see your GP.<br>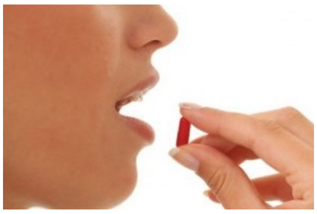	One tablet should be taken once every day, at night with cold water.<br>You need to take this medicine for 10 years.<br>You need to regularly visit a GP to receive a prescription.<br>You need to visit a pharmacy to fill a prescription.<br>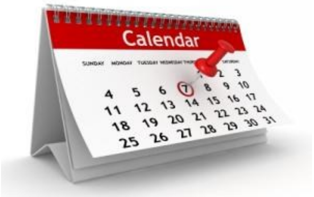<br>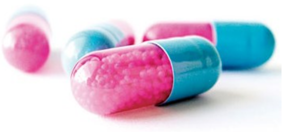

If you take Medicine A, you would live for **3 weeks less than 10 years** and then die.

If you take Medicine B, you would live for **10 years** and then die.

Would you prefer Medicine A or Medicine B, or are they the same?

Indicate your choice here. You can only choose one option:

◯ **Medicine A** Live for (10 years – 3 weeks)

◯ **Medicine B** Live for 10 years

◯ **Medicine A and Medicine B are the same.**

0% 100%

For peer review only

Repeated section

Remember Medicine A and Medicine B are equally effective and have **some risk of a side effect**.

The only difference between Medicine A and Medicine B is how you take them.

**Medicine A**	**Medicine B**
You take a tablet only once in your life.<br>You will be given the tablet on the day you come in to see your GP.<br>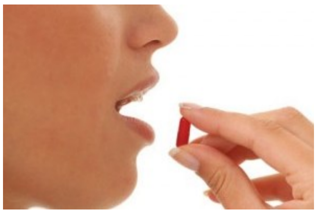	One tablet should be taken once every day, at night with cold water.<br>You need to take this medicine for 10 years.<br>You need to regularly visit a GP to receive a prescription.<br>You need to visit a pharmacy to fill a prescription.<br>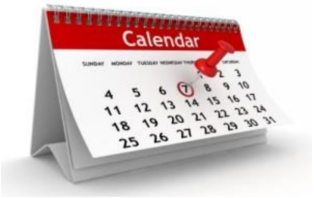<br>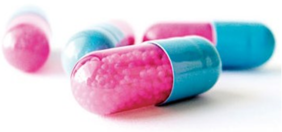

If you take Medicine A, you would live for **2 weeks less than 10 years** and then die.

If you take Medicine B, you would live for **10 years** and then die.

Would you prefer Medicine A or Medicine B, or are they the same?

Indicate your choice here. You can only choose one option:

○ **Medicine A** Live for (10 years – 2 weeks)

○ **Medicine B** Live for 10 years

○ **Medicine A and Medicine B are the same.**

0% 100%

For peer review only

Repeated section

Remember Medicine A and Medicine B are equally effective and have **some risk of a side effect**.

The only difference between Medicine A and Medicine B is how you take them.

**Medicine A**	**Medicine B**
You take a tablet only once in your life.<br>You will be given the tablet on the day you come in to see your GP.<br>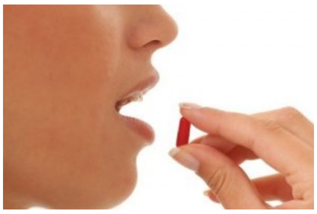	One tablet should be taken once every day, at night with cold water.<br>You need to take this medicine for 10 years.<br>You need to regularly visit a GP to receive a prescription.<br>You need to visit a pharmacy to fill a prescription.<br>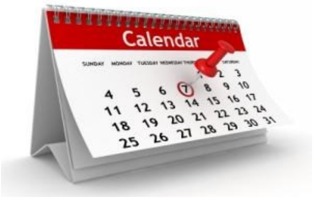<br>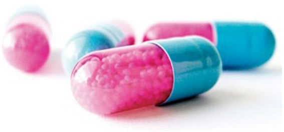

If you take Medicine A, you would live for **10 days less than 10 years** and then die.

If you take Medicine B, you would live for **10 years** and then die.

Would you prefer Medicine A or Medicine B, or are they the same?

Indicate your choice here. You can only choose one option:

◯ **Medicine A** Live for (10 years – 10 days)

◯ **Medicine B** Live for 10 years

◯ **Medicine A and Medicine B are the same.**

For peer review only

Repeated section

Remember Medicine A and Medicine B are equally effective and have **some risk of a side effect**.

The only difference between Medicine A and Medicine B is how you take them.

**Medicine A**	**Medicine B**
You take a tablet only once in your life.<br>You will be given the tablet on the day you come in to see your GP.<br>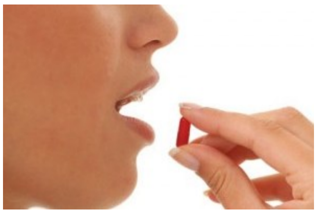	One tablet should be taken once every day, at night with cold water.<br>You need to take this medicine for 10 years.<br>You need to regularly visit a GP to receive a prescription.<br>You need to visit a pharmacy to fill a prescription.<br>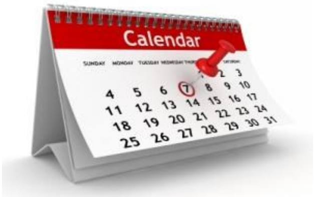<br>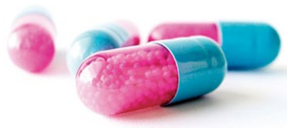

If you take Medicine A, you would live for **1 week less than 10 years** and then die.

If you take Medicine B, you would live for **10 years** and then die.

Would you prefer Medicine A or Medicine B, or are they the same?

Indicate your choice here. You can only choose one option:

◯ **Medicine A** Live for (10 years – 1 week)

◯ **Medicine B** Live for 10 years

◯ **Medicine A and Medicine B are the same.**

0% 100%

For peer review only

Repeated section

Remember Medicine A and Medicine B are equally effective and have **some risk of a side effect**.

The only difference between Medicine A and Medicine B is how you take them.

Medicine A	Medicine B
You take a tablet only once in your life.<br>You will be given the tablet on the day you come in to see your GP.<br>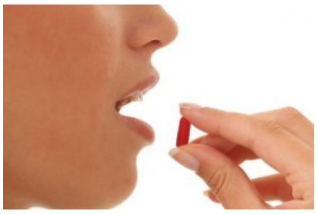	One tablet should be taken once every day, at night with cold water.<br>You need to take this medicine for 10 years.<br>You need to regularly visit a GP to receive a prescription.<br>You need to visit a pharmacy to fill a prescription.<br>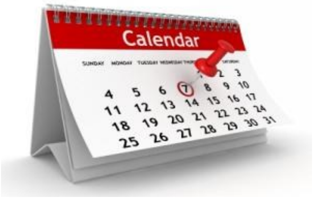<br>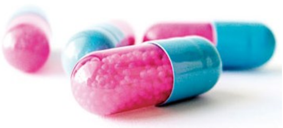

If you take Medicine A, you would live for **5 days less than 10 years** and then die.

If you take Medicine B, you would live for **10 years** and then die.

Would you prefer Medicine A or Medicine B, or are they the same?

Indicate your choice here. You can only choose one option:

○ **Medicine A** Live for (10 years – 5 days)

○ **Medicine B** Live for 10 years

○ **Medicine A and Medicine B are the same.**

For peer review only

Repeated section

Remember Medicine A and Medicine B are equally effective and have **some risk of a side effect**.

The only difference between Medicine A and Medicine B is how you take them.

**Medicine A**	**Medicine B**
You take a tablet only once in your life.<br>You will be given the tablet on the day you come in to see your GP.<br>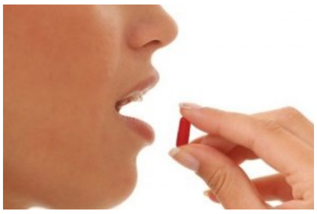	One tablet should be taken once every day, at night with cold water.<br>You need to take this medicine for 10 years.<br>You need to regularly visit a GP to receive a prescription.<br>You need to visit a pharmacy to fill a prescription.<br>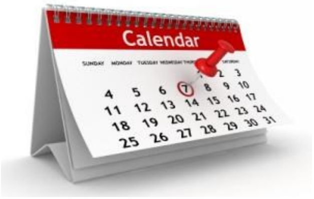 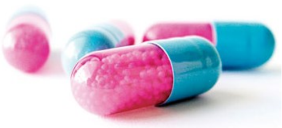

If you take Medicine A, you would live for **3 days less than 10 years** and then die.

If you take Medicine B, you would live for **10 years** and then die.

Would you prefer Medicine A or Medicine B, or are they the same?

Indicate your choice here. You can only choose one option:

○ **Medicine A** Live for (10 years – 3 days)

○ **Medicine B** Live for 10 years

○ **Medicine A and Medicine B are the same.**

For peer review only

Repeated section

Remember Medicine A and Medicine B are equally effective and have **some risk of a side effect**.

The only difference between Medicine A and Medicine B is how you take them.

Medicine A	Medicine B
You take a tablet only once in your life.<br>You will be given the tablet on the day you come in to see your GP.<br>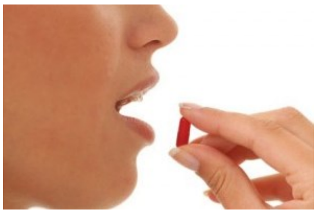	One tablet should be taken once every day, at night with cold water.<br>You need to take this medicine for 10 years.<br>You need to regularly visit a GP to receive a prescription.<br>You need to visit a pharmacy to fill a prescription.<br>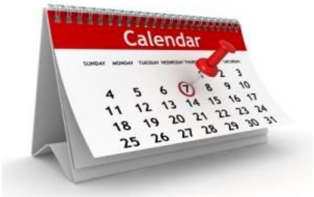<br>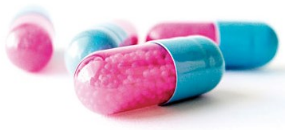

If you take Medicine A, you would live for **1 day less than 10 years** and then die.

If you take Medicine B, you would live for **10 years** and then die.

Would you prefer Medicine A or Medicine B, or are they the same?

Indicate your choice here. You can only choose one option:

○ **Medicine A** Live for (10 years – 1 day)

○ **Medicine B** Live for 10 years

○ **Medicine A and Medicine B are the same.**

0% 100%

For peer review only

Question 2a.

You prefer living for 5 years on Medicine A, to living for 10 years on Medicine B.

This means **living fewer than 5 years on Medicine A** can be the same as living for 10 years on Medicine B.

How many years on Medicine A do you think would be the same as living for 10 years on Medicine B?

(Please give a number in years)

0% 100%

peer review only

Part 2

Question 3

Now, we will assume that there is some risk of a severe side effect from either medicine.

As before,

Medicine A is a pill that you only need to take once.

Medicine B is a pill that you need to take every day for ten years.

Medicine A and Medicine B are equally effective in reducing your risk of developing heart disease.

Medicine A and Medicine B will reduce your risk of having a heart attack or a stroke within the next 10 years from **10%** to **6%**.

In other words, in a crowd of 1,000 people in the UK, **100** are likely to have a heart attack or a stroke if they do not take the medicine. This is reduced to **60** people out of 1,000 who do take the medicine. This information is also illustrated in this picture.

Risk of heart disease

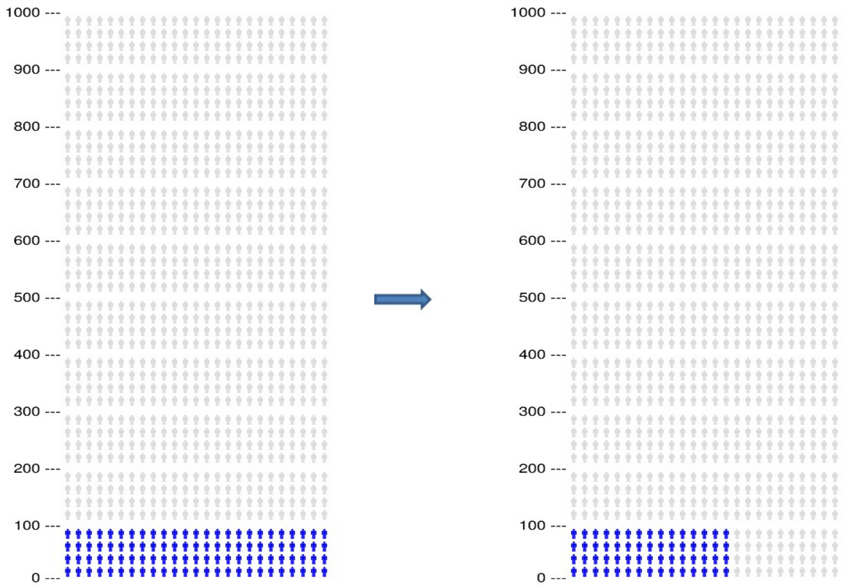

10%
No Treatment

6%
Taking the Medicine

(The figures shaded blue represent people who will have a heart attack or a stroke in the next 10 years)

In this scenario, there is some risk of a severe side effect from Medicine A or Medicine B.

Among every 1,000 people taking Medicine A or Medicine B, **3** people are likely to have a severe side effect such as severe destruction of skeletal muscle (rhabdomyolysis).

Risk of a severe side effect

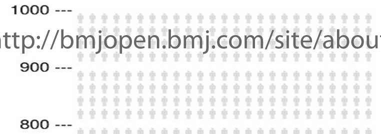

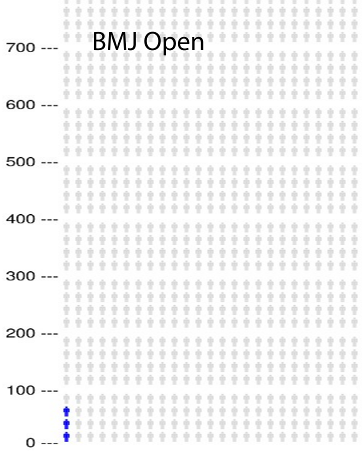

0.3% of people will have a severe side effect.

0% 100%

er review only

Repeated section

Remember Medicine A and Medicine B are equally effective and have **some risk of a side effect**.

The only difference between Medicine A and Medicine B is how you take them.

Medicine A	Medicine B
You take a tablet only once in your life.<br>You will be given the tablet on the day you come in to see your GP.<br>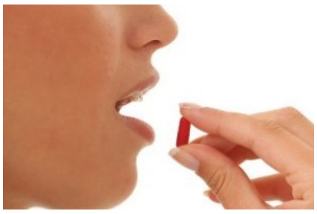	One tablet should be taken once every day, at night with cold water.<br>You need to take this medicine for 10 years.<br>You need to regularly visit a GP to receive a prescription.<br>You need to visit a pharmacy to fill a prescription.<br>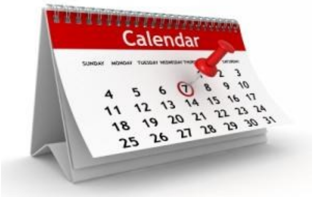<br>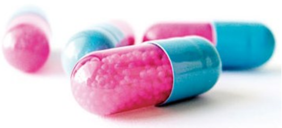

If you take Medicine A, you would live for **5 years** and then die.

If you take Medicine B, you would live for **10 years** and then die.

Would you prefer Medicine A or Medicine B, or are they the same?

Indicate your choice here. You can only choose one option:

○ **Medicine A** Live for 5 years

○ **Medicine B** Live for 10 years

○ **Medicine A and Medicine B are the same.**

For peer review only

Repeated section

Remember Medicine A and Medicine B are equally effective and have **some risk of a side effect**.

The only difference between Medicine A and Medicine B is how you take them.

**Medicine A**	**Medicine B**
You take a tablet only once in your life.<br>You will be given the tablet on the day you come in to see your GP.<br>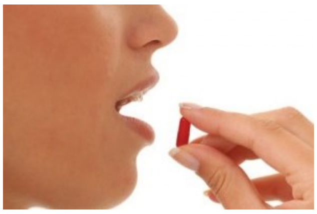	One tablet should be taken once every day, at night with cold water.<br>You need to take this medicine for 10 years.<br>You need to regularly visit a GP to receive a prescription.<br>You need to visit a pharmacy to fill a prescription.<br>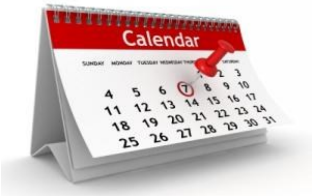<br>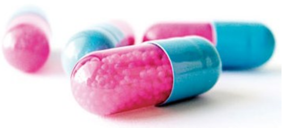

If you take Medicine A, you would live for **5.5 years** (5 years and 6 months) and then die.

If you take Medicine B, you would live for **10 years** and then die.

Would you prefer Medicine A or Medicine B, or are they the same?

Indicate your choice here. You can only choose one option:

◯ **Medicine A** 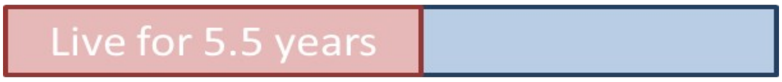

◯ **Medicine B** 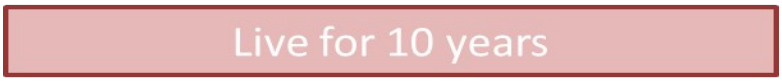

◯ **Medicine A and Medicine B are the same.**

0% 100%

For peer review only

Repeated section

Remember Medicine A and Medicine B are equally effective and have **some risk of a side effect**.

The only difference between Medicine A and Medicine B is how you take them.

**Medicine A**	**Medicine B**
You take a tablet only once in your life.<br>You will be given the tablet on the day you come in to see your GP.<br>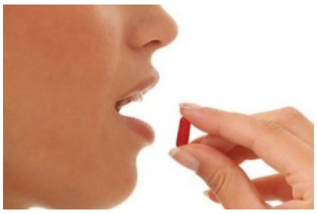	One tablet should be taken once every day, at night with cold water.<br>You need to take this medicine for 10 years.<br>You need to regularly visit a GP to receive a prescription.<br>You need to visit a pharmacy to fill a prescription.<br>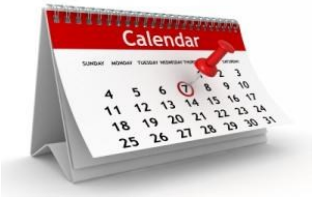<br>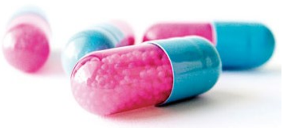

If you take Medicine A, you would live for **6 years** and then die.

If you take Medicine B, you would live for **10 years** and then die.

Would you prefer Medicine A or Medicine B, or are they the same?

Indicate your choice here. You can only choose one option:

◯ **Medicine A** 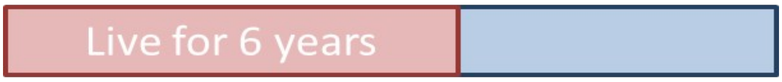

◯ **Medicine B** 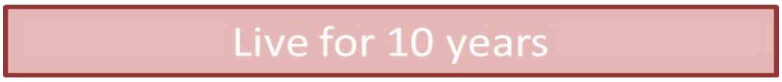

◯ **Medicine A and Medicine B are the same.**

For peer review only

Repeated section

Remember Medicine A and Medicine B are equally effective and have **some risk of a side effect**.

The only difference between Medicine A and Medicine B is how you take them.

Medicine A	Medicine B
You take a tablet only once in your life.<br>You will be given the tablet on the day you come in to see your GP.<br>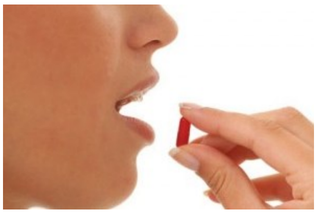	One tablet should be taken once every day, at night with cold water.<br>You need to take this medicine for 10 years.<br>You need to regularly visit a GP to receive a prescription.<br>You need to visit a pharmacy to fill a prescription.<br>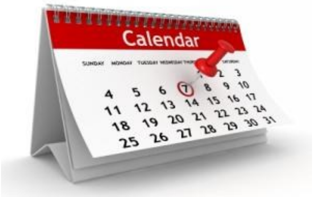<br><br>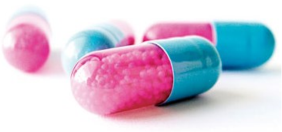

If you take Medicine A, you would live for **6.25 years (6 years and 3 months)** and then die.

If you take Medicine B, you would live for **10 years** and then die.

Would you prefer Medicine A or Medicine B, or are they the same?

Indicate your choice here. You can only choose one option:

◯ **Medicine A**

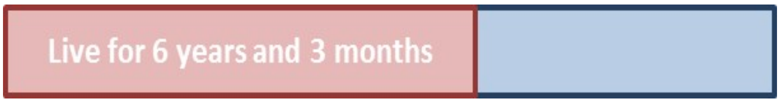

◯ **Medicine B**

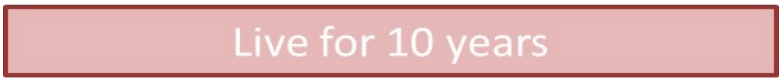

◯ **Medicine A and Medicine B are the same.**

0% 100%

For peer review only

Repeated section

Remember Medicine A and Medicine B are equally effective and have **some risk of a side effect**.

The only difference between Medicine A and Medicine B is how you take them.

Medicine A	Medicine B
You take a tablet only once in your life.<br>You will be given the tablet on the day you come in to see your GP.<br>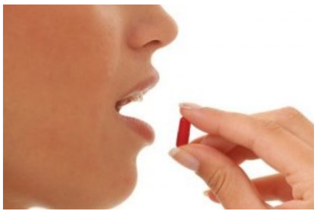	One tablet should be taken once every day, at night with cold water.<br>You need to take this medicine for 10 years.<br>You need to regularly visit a GP to receive a prescription.<br>You need to visit a pharmacy to fill a prescription.<br>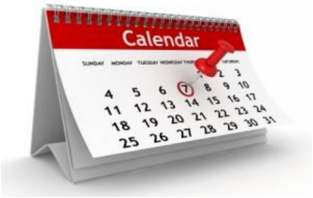<br><br>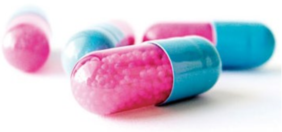

If you take Medicine A, you would live for **6.5 years** (6 years and 6 months) and then die.

If you take Medicine B, you would live for **10 years** and then die.

Would you prefer Medicine A or Medicine B, or are they the same?

Indicate your choice here. You can only choose one option:

- ◯ **Medicine A** — Live for 6.5 years
- ◯ **Medicine B** — Live for 10 years
- ◯ **Medicine A and Medicine B are the same.**

For peer review only

Repeated section

Remember Medicine A and Medicine B are equally effective and have **some risk of a side effect**.

The only difference between Medicine A and Medicine B is how you take them.

Medicine A	Medicine B
You take a tablet only once in your life.<br>You will be given the tablet on the day you come in to see your GP.<br>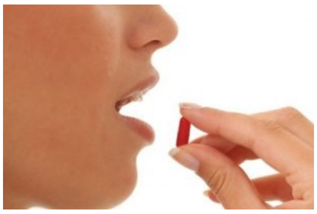	One tablet should be taken once every day, at night with cold water.<br>You need to take this medicine for 10 years.<br>You need to regularly visit a GP to receive a prescription.<br>You need to visit a pharmacy to fill a prescription.<br>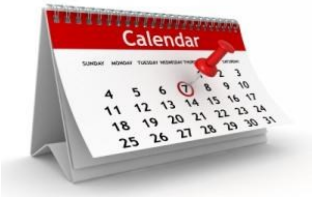<br><br>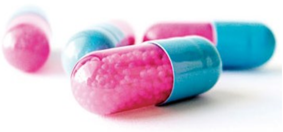

If you take Medicine A, you would live for **7 years** and then die.

If you take Medicine B, you would live for **10 years** and then die.

Would you prefer Medicine A or Medicine B, or are they the same?

Indicate your choice here. You can only choose one option:

◯ **Medicine A** Live for 7 years

◯ **Medicine B** Live for 10 years

◯ **Medicine A and Medicine B are the same.**

0% 100%

For peer review only

Repeated section

Remember Medicine A and Medicine B are equally effective and have **some risk of a side effect**.

The only difference between Medicine A and Medicine B is how you take them.

**Medicine A**	**Medicine B**
You take a tablet only once in your life.<br>You will be given the tablet on the day you come in to see your GP.<br>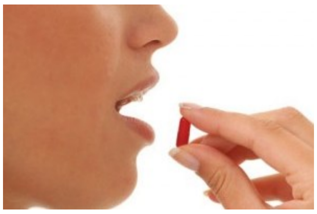	One tablet should be taken once every day, at night with cold water.<br>You need to take this medicine for 10 years.<br>You need to regularly visit a GP to receive a prescription.<br>You need to visit a pharmacy to fill a prescription.<br>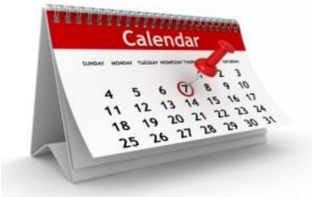<br>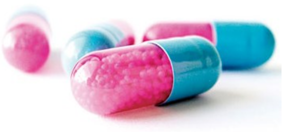

If you take Medicine A, you would live for **7.5 years** (7 years and 6 months) and then die.

If you take Medicine B, you would live for **10 years** and then die.

Would you prefer Medicine A or Medicine B, or are they the same?

Indicate your choice here. You can only choose one option:

- ◯ **Medicine A** — Live for 7.5 years
- ◯ **Medicine B** — Live for 10 years
- ◯ **Medicine A and Medicine B are the same.**

For peer review only

Repeated section

Remember Medicine A and Medicine B are equally effective and have **some risk of a side effect**.

The only difference between Medicine A and Medicine B is how you take them.

Medicine A	Medicine B
You take a tablet only once in your life.<br>You will be given the tablet on the day you come in to see your GP.<br>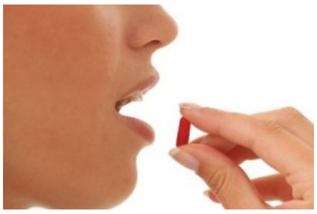	One tablet should be taken once every day, at night with cold water.<br>You need to take this medicine for 10 years.<br>You need to regularly visit a GP to receive a prescription.<br>You need to visit a pharmacy to fill a prescription.<br>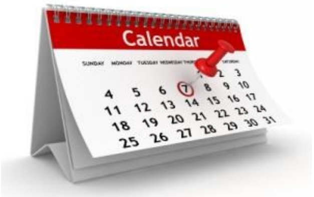<br>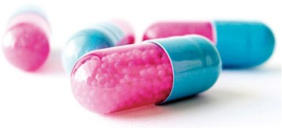

If you take Medicine A, you would live for **7.75 years (7 years and 9 months)** and then die.

If you take Medicine B, you would live for **10 years** and then die.

Would you prefer Medicine A or Medicine B, or are they the same?

Indicate your choice here. You can only choose one option:

○ **Medicine A** Live for 7 years and 9 months

○ **Medicine B** Live for 10 years

○ **Medicine A and Medicine B are the same.**

For peer review only

Repeated section

Remember Medicine A and Medicine B are equally effective and have **some risk of a side effect**.

The only difference between Medicine A and Medicine B is how you take them.

Medicine A	Medicine B
You take a tablet only once in your life.<br>You will be given the tablet on the day you come in to see your GP.<br>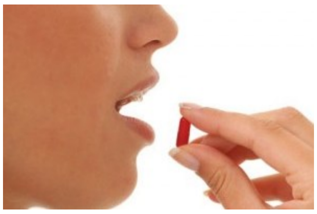	One tablet should be taken once every day, at night with cold water.<br>You need to take this medicine for 10 years.<br>You need to regularly visit a GP to receive a prescription.<br>You need to visit a pharmacy to fill a prescription.<br>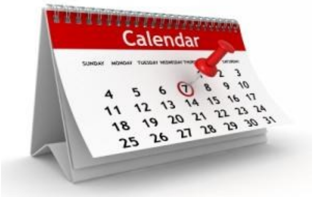<br>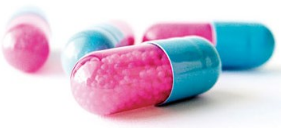

If you take Medicine A, you would live for **8 years** and then die.

If you take Medicine B, you would live for **10 years** and then die.

Would you prefer Medicine A or Medicine B, or are they the same?

Indicate your choice here. You can only choose one option:

◯ **Medicine A** 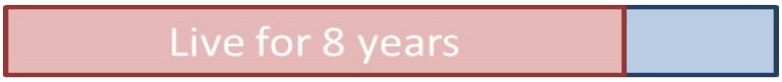

◯ **Medicine B** 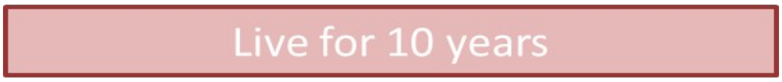

◯ **Medicine A and Medicine B are the same.**

For peer review only

Repeated section

Remember Medicine A and Medicine B are equally effective and have **some risk of a side effect**.

The only difference between Medicine A and Medicine B is how you take them.

Medicine A	Medicine B
You take a tablet only once in your life.<br>You will be given the tablet on the day you come in to see your GP.<br>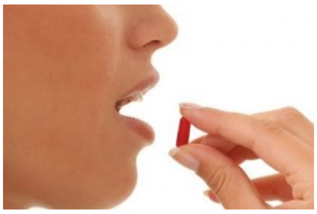	One tablet should be taken once every day, at night with cold water.<br>You need to take this medicine for 10 years.<br>You need to regularly visit a GP to receive a prescription.<br>You need to visit a pharmacy to fill a prescription.<br>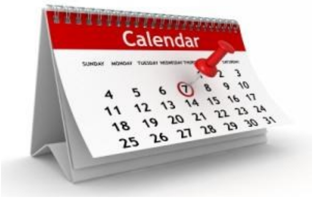<br>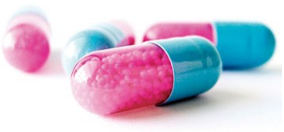

If you take Medicine A, you would live for **8.25 years (8 years and 3 months)** and then die.

If you take Medicine B, you would live for **10 years** and then die.

Would you prefer Medicine A or Medicine B, or are they the same?

Indicate your choice here. You can only choose one option:

◯ **Medicine A** Live for 8 years and 3 months

◯ **Medicine B** Live for 10 years

◯ **Medicine A and Medicine B are the same.**

0% 100%

For peer review only

Repeated section

Remember Medicine A and Medicine B are equally effective and have **some risk of a side effect**.

The only difference between Medicine A and Medicine B is how you take them.

Medicine A	Medicine B
You take a tablet only once in your life.<br>You will be given the tablet on the day you come in to see your GP.<br>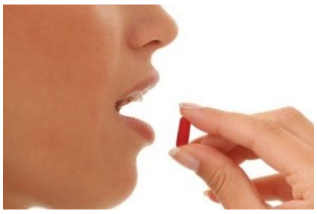	One tablet should be taken once every day, at night with cold water.<br>You need to take this medicine for 10 years.<br>You need to regularly visit a GP to receive a prescription.<br>You need to visit a pharmacy to fill a prescription.<br>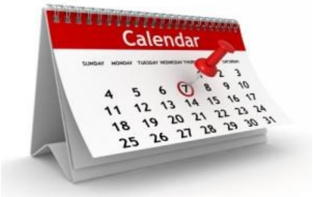<br>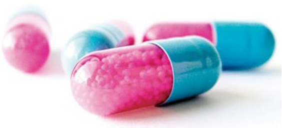

If you take Medicine A, you would live for **8.5 years** (8 years and 6 months) and then die.

If you take Medicine B, you would live for **10 years** and then die.

Would you prefer Medicine A or Medicine B, or are they the same?

Indicate your choice here. You can only choose one option:

◯ **Medicine A** Live for 8.5 years

◯ **Medicine B** Live for 10 years

◯ **Medicine A and Medicine B are the same.**

0% 100%

For peer review only

Repeated section

Remember Medicine A and Medicine B are equally effective and have **some risk of a side effect**.

The only difference between Medicine A and Medicine B is how you take them.

**Medicine A**	**Medicine B**
You take a tablet only once in your life.<br>You will be given the tablet on the day you come in to see your GP.<br>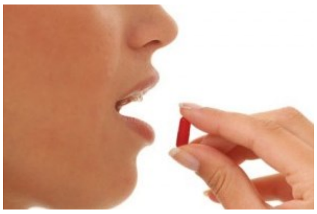	One tablet should be taken once every day, at night with cold water.<br>You need to take this medicine for 10 years.<br>You need to regularly visit a GP to receive a prescription.<br>You need to visit a pharmacy to fill a prescription.<br>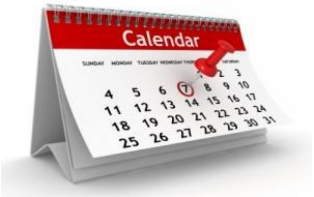<br>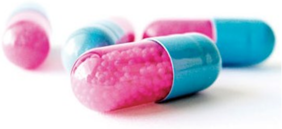

If you take Medicine A, you would live for **8.75 years (8 years and 9 months)** and then die.

If you take Medicine B, you would live for **10 years** and then die.

Would you prefer Medicine A or Medicine B, or are they the same?

Indicate your choice here. You can only choose one option:

○ **Medicine A** Live for 8 years and 9 months

○ **Medicine B** Live for 10 years

○ **Medicine A and Medicine B are the same.**

For peer review only

Repeated section

Remember Medicine A and Medicine B are equally effective and have **some risk of a side effect**.

The only difference between Medicine A and Medicine B is how you take them.

Medicine A	Medicine B
You take a tablet only once in your life.<br>You will be given the tablet on the day you come in to see your GP.<br>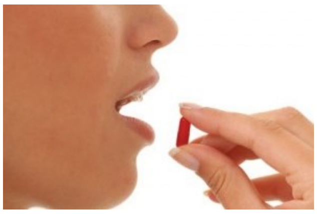	One tablet should be taken once every day, at night with cold water.<br>You need to take this medicine for 10 years.<br>You need to regularly visit a GP to receive a prescription.<br>You need to visit a pharmacy to fill a prescription.<br>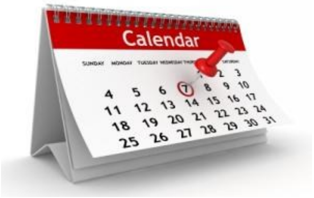 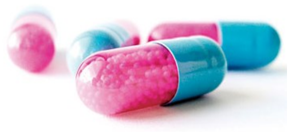

If you take Medicine A, you would live for **9 years** and then die.

If you take Medicine B, you would live for **10 years** and then die.

Would you prefer Medicine A or Medicine B, or are they the same?

Indicate your choice here. You can only choose one option:

○ **Medicine A** Live for 9 years

○ **Medicine B** Live for 10 years

○ **Medicine A and Medicine B are the same.**

For peer review only

Repeated section

Remember Medicine A and Medicine B are equally effective and have **some risk of a side effect**.

The only difference between Medicine A and Medicine B is how you take them.

**Medicine A**	**Medicine B**
You take a tablet only once in your life.<br>You will be given the tablet on the day you come in to see your GP.<br>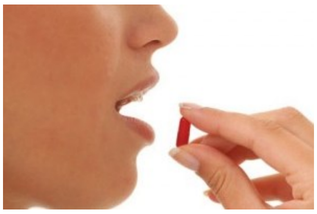	One tablet should be taken once every day, at night with cold water.<br>You need to take this medicine for 10 years.<br>You need to regularly visit a GP to receive a prescription.<br>You need to visit a pharmacy to fill a prescription.<br>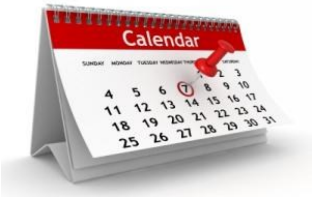<br>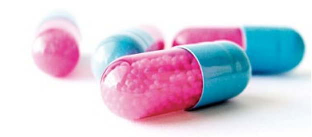

If you take Medicine A, you would live for **9 years and 1 month** and then die.

If you take Medicine B, you would live for **10 years** and then die.

Would you prefer Medicine A or Medicine B, or are they the same?

Indicate your choice here. You can only choose one option:

◯ **Medicine A** Live for 9 years 1 month

◯ **Medicine B** Live for 10 years

◯ **Medicine A and Medicine B are the same.**

For peer review only

Repeated section

Remember Medicine A and Medicine B are equally effective and have **some risk of a side effect**.

The only difference between Medicine A and Medicine B is how you take them.

**Medicine A**	**Medicine B**
You take a tablet only once in your life.<br>You will be given the tablet on the day you come in to see your GP.<br>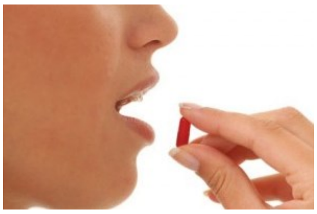	One tablet should be taken once every day, at night with cold water.<br>You need to take this medicine for 10 years.<br>You need to regularly visit a GP to receive a prescription.<br>You need to visit a pharmacy to fill a prescription.<br>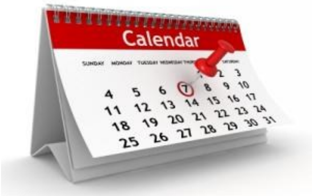<br>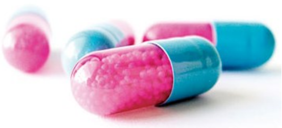

If you take Medicine A, you would live for **9.25 years (9 years and 3 months)** and then die.

If you take Medicine B, you would live for **10 years** and then die.

Would you prefer Medicine A or Medicine B, or are they the same?

Indicate your choice here. You can only choose one option:

○ **Medicine A** Live for 9 years and 3 months

○ **Medicine B** Live for 10 years

○ **Medicine A and Medicine B are the same.**

0% 100%

For peer review only

Repeated section

Remember Medicine A and Medicine B are equally effective and have **some risk of a side effect**.

The only difference between Medicine A and Medicine B is how you take them.

Medicine A	Medicine B
You take a tablet only once in your life.<br>You will be given the tablet on the day you come in to see your GP.<br>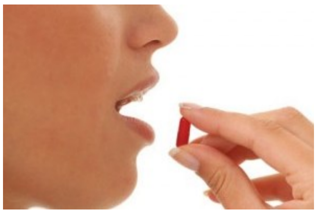	One tablet should be taken once every day, at night with cold water.<br>You need to take this medicine for 10 years.<br>You need to regularly visit a GP to receive a prescription.<br>You need to visit a pharmacy to fill a prescription.<br>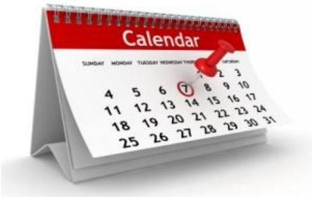<br><br>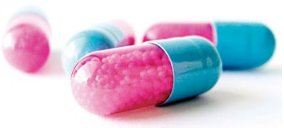

If you take Medicine A, you would live for **9 years and 4 months** and then die.

If you take Medicine B, you would live for **10 years** and then die.

Would you prefer Medicine A or Medicine B, or are they the same?

Indicate your choice here. You can only choose one option:

◯ **Medicine A** Live for 9 years 4 months

◯ **Medicine B** Live for 10 years

◯ **Medicine A and Medicine B are the same.**

0% 100%

For peer review only

Repeated section

Remember Medicine A and Medicine B are equally effective and have **some risk of a side effect**.

The only difference between Medicine A and Medicine B is how you take them.

**Medicine A**	**Medicine B**
You take a tablet only once in your life.<br>You will be given the tablet on the day you come in to see your GP.<br>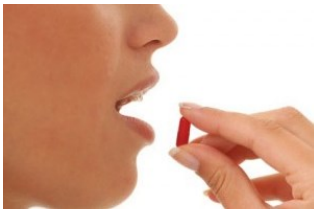	One tablet should be taken once every day, at night with cold water.<br>You need to take this medicine for 10 years.<br>You need to regularly visit a GP to receive a prescription.<br>You need to visit a pharmacy to fill a prescription.<br>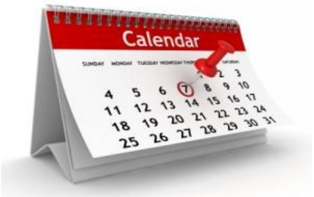 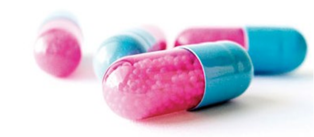

If you take Medicine A, you would live for **9 years and 5 months** and then die.

If you take Medicine B, you would live for **10 years** and then die.

Would you prefer Medicine A or Medicine B, or are they the same?

Indicate your choice here. You can only choose one option:

◯ **Medicine A** Live for 9 years 5 months

◯ **Medicine B** Live for 10 years

◯ **Medicine A and Medicine B are the same.**

For peer review only

Repeated section

Remember Medicine A and Medicine B are equally effective and have **some risk of a side effect**.

The only difference between Medicine A and Medicine B is how you take them.

Medicine A	Medicine B
You take a tablet only once in your life.<br>You will be given the tablet on the day you come in to see your GP.<br>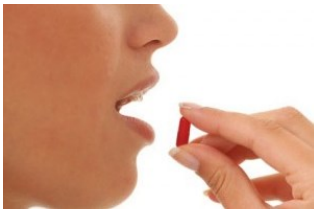	One tablet should be taken once every day, at night with cold water.<br>You need to take this medicine for 10 years.<br>You need to regularly visit a GP to receive a prescription.<br>You need to visit a pharmacy to fill a prescription.<br>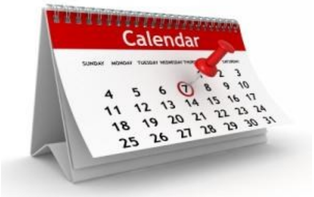<br><br>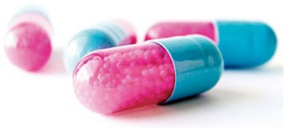

If you take Medicine A, you would live for **9.5 years** (9 years and 6 months) and then die.

If you take Medicine B, you would live for **10 years** and then die.

Would you prefer Medicine A or Medicine B, or are they the same?

Indicate your choice here. You can only choose one option:

○ **Medicine A** Live for 9.5 years

○ **Medicine B** Live for 10 years

○ **Medicine A and Medicine B are the same.**

0% 100%

For peer review only

Repeated section

Remember Medicine A and Medicine B are equally effective and have **some risk of a side effect**.

The only difference between Medicine A and Medicine B is how you take them.

**Medicine A**	**Medicine B**
You take a tablet only once in your life.<br>You will be given the tablet on the day you come in to see your GP.<br>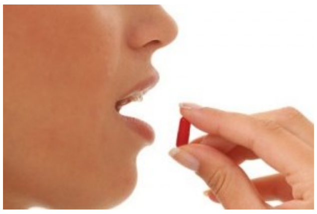	One tablet should be taken once every day, at night with cold water.<br>You need to take this medicine for 10 years.<br>You need to regularly visit a GP to receive a prescription.<br>You need to visit a pharmacy to fill a prescription.<br>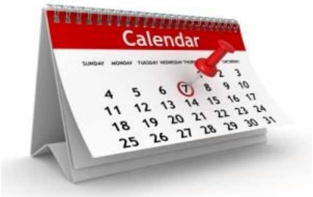<br>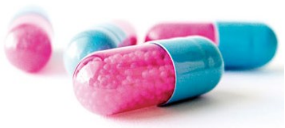

If you take Medicine A, you would live for **5 months less than 10 years** and then die.

If you take Medicine B, you would live for **10 years** and then die.

Would you prefer Medicine A or Medicine B, or are they the same?

Indicate your choice here. You can only choose one option:

○ **Medicine A** Live for (10 years – 5 months)

○ **Medicine B** Live for 10 years

○ **Medicine A and Medicine B are the same.**

For peer review only

Repeated section

Remember Medicine A and Medicine B are equally effective and have **some risk of a side effect**.

The only difference between Medicine A and Medicine B is how you take them.

**Medicine A**	**Medicine B**
You take a tablet only once in your life.<br>You will be given the tablet on the day you come in to see your GP.<br>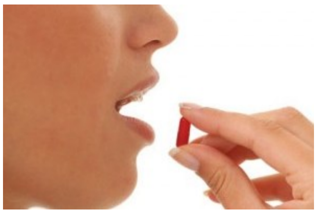	One tablet should be taken once every day, at night with cold water.<br>You need to take this medicine for 10 years.<br>You need to regularly visit a GP to receive a prescription.<br>You need to visit a pharmacy to fill a prescription.<br>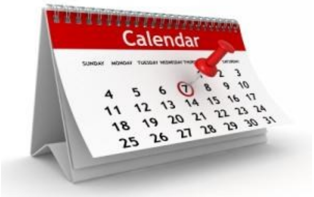<br><br>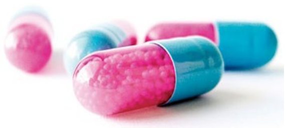

If you take Medicine A, you would live for **4 months less than 10 years** and then die.

If you take Medicine B, you would live for **10 years** and then die.

Would you prefer Medicine A or Medicine B, or are they the same?

Indicate your choice here. You can only choose one option:

◯ **Medicine A** Live for (10 years – 4 months)

◯ **Medicine B** Live for 10 years

◯ **Medicine A and Medicine B are the same.**

For peer review only

Repeated section

Remember Medicine A and Medicine B are equally effective and have **some risk of a side effect**.

The only difference between Medicine A and Medicine B is how you take them.

**Medicine A**	**Medicine B**
You take a tablet only once in your life.<br>You will be given the tablet on the day you come in to see your GP.<br>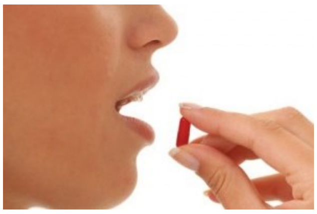	One tablet should be taken once every day, at night with cold water.<br>You need to take this medicine for 10 years.<br>You need to regularly visit a GP to receive a prescription.<br>You need to visit a pharmacy to fill a prescription.<br>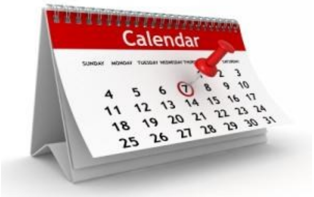<br>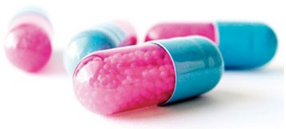

If you take Medicine A, you would live for **3 months less than 10 years** and then die.

If you take Medicine B, you would live for **10 years** and then die.

Would you prefer Medicine A or Medicine B, or are they the same?

Indicate your choice here. You can only choose one option:

○ **Medicine A** Live for (10 years – 3 months)

○ **Medicine B** Live for 10 years

○ **Medicine A and Medicine B are the same.**

For peer review only

Repeated section

Remember Medicine A and Medicine B are equally effective and have **some risk of a side effect**.

The only difference between Medicine A and Medicine B is how you take them.

Medicine A	Medicine B
You take a tablet only once in your life.<br>You will be given the tablet on the day you come in to see your GP.<br>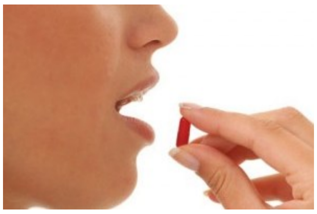	One tablet should be taken once every day, at night with cold water.<br>You need to take this medicine for 10 years.<br>You need to regularly visit a GP to receive a prescription.<br>You need to visit a pharmacy to fill a prescription.<br>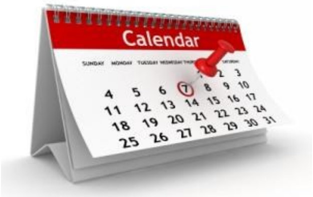<br>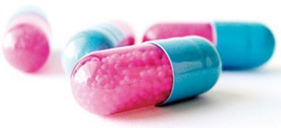

If you take Medicine A, you would live for **2 months less than 10 years** and then die.

If you take Medicine B, you would live for **10 years** and then die.

Would you prefer Medicine A or Medicine B, or are they the same?

Indicate your choice here. You can only choose one option:

○ **Medicine A** Live for (10 years – 2 months)

○ **Medicine B** Live for 10 years

○ **Medicine A and Medicine B are the same.**

For peer review only

Repeated section

Remember Medicine A and Medicine B are equally effective and have **some risk of a side effect**.

The only difference between Medicine A and Medicine B is how you take them.

**Medicine A**	**Medicine B**
You take a tablet only once in your life.<br>You will be given the tablet on the day you come in to see your GP.<br>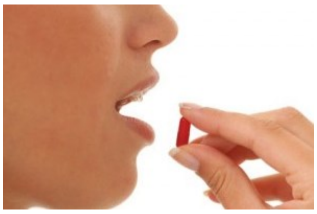	One tablet should be taken once every day, at night with cold water.<br>You need to take this medicine for 10 years.<br>You need to regularly visit a GP to receive a prescription.<br>You need to visit a pharmacy to fill a prescription.<br>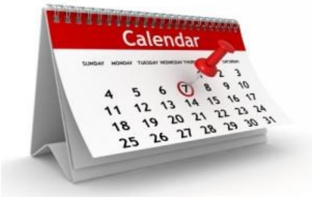<br><br>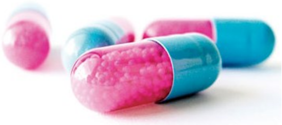

If you take Medicine A, you would live for **1 month less than 10 years** and then die.

If you take Medicine B, you would live for **10 years** and then die.

Would you prefer Medicine A or Medicine B, or are they the same?

Indicate your choice here. You can only choose one option:

○ **Medicine A** Live for (10 years – 1 month)

○ **Medicine B** Live for 10 years

○ **Medicine A and Medicine B are the same.**

0% 100%

For peer review only

Repeated section

Remember Medicine A and Medicine B are equally effective and have **some risk of a side effect**.

The only difference between Medicine A and Medicine B is how you take them.

**Medicine A**	**Medicine B**
You take a tablet only once in your life.<br>You will be given the tablet on the day you come in to see your GP.<br>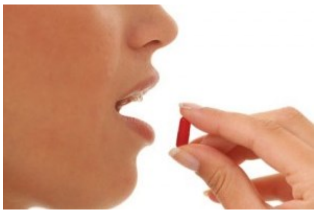	One tablet should be taken once every day, at night with cold water.<br>You need to take this medicine for 10 years.<br>You need to regularly visit a GP to receive a prescription.<br>You need to visit a pharmacy to fill a prescription.<br>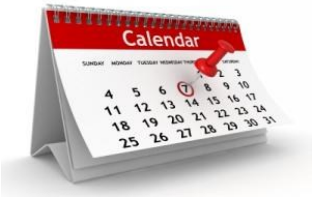<br>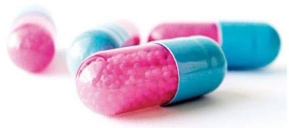

If you take Medicine A, you would live for **3 weeks less than 10 years** and then die.

If you take Medicine B, you would live for **10 years** and then die.

Would you prefer Medicine A or Medicine B, or are they the same?

Indicate your choice here. You can only choose one option:

◯ **Medicine A** Live for (10 years – 3 weeks)

◯ **Medicine B** Live for 10 years

◯ **Medicine A and Medicine B are the same.**

0% 100%

For peer review only

Repeated section

Remember Medicine A and Medicine B are equally effective and have **some risk of a side effect**.

The only difference between Medicine A and Medicine B is how you take them.

**Medicine A**	**Medicine B**
You take a tablet only once in your life.<br>You will be given the tablet on the day you come in to see your GP.<br>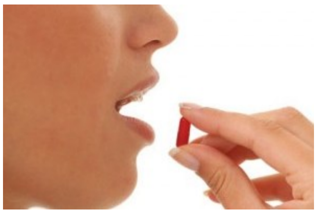	One tablet should be taken once every day, at night with cold water.<br>You need to take this medicine for 10 years.<br>You need to regularly visit a GP to receive a prescription.<br>You need to visit a pharmacy to fill a prescription.<br>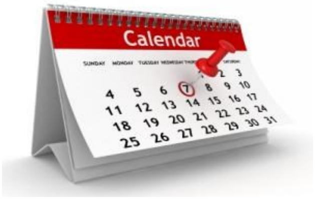<br>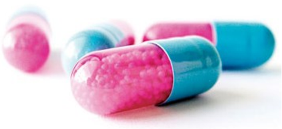

If you take Medicine A, you would live for **2 weeks less than 10 years** and then die.

If you take Medicine B, you would live for **10 years** and then die.

Would you prefer Medicine A or Medicine B, or are they the same?

Indicate your choice here. You can only choose one option:

◯ **Medicine A** Live for (10 years – 2 weeks)

◯ **Medicine B** Live for 10 years

◯ **Medicine A and Medicine B are the same.**

For peer review only

Repeated section

Remember Medicine A and Medicine B are equally effective and have **some risk of a side effect**.

The only difference between Medicine A and Medicine B is how you take them.

Medicine A	Medicine B
You take a tablet only once in your life.<br>You will be given the tablet on the day you come in to see your GP.<br>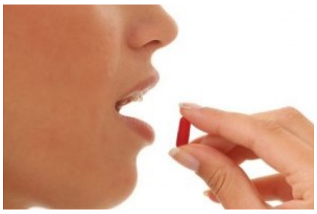	One tablet should be taken once every day, at night with cold water.<br>You need to take this medicine for 10 years.<br>You need to regularly visit a GP to receive a prescription.<br>You need to visit a pharmacy to fill a prescription.<br>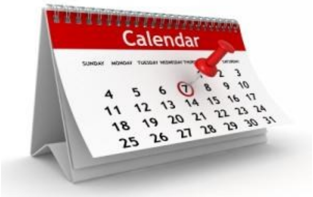<br>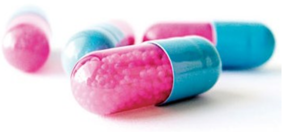

If you take Medicine A, you would live for **10 days less than 10 years** and then die.

If you take Medicine B, you would live for **10 years** and then die.

Would you prefer Medicine A or Medicine B, or are they the same?

Indicate your choice here. You can only choose one option:

◯ **Medicine A** Live for (10 years – 10 days)

◯ **Medicine B** Live for 10 years

◯ **Medicine A and Medicine B are the same.**

For peer review only

Repeated section

Remember Medicine A and Medicine B are equally effective and have **some risk of a side effect**.

The only difference between Medicine A and Medicine B is how you take them.

**Medicine A**	**Medicine B**
You take a tablet only once in your life.<br>You will be given the tablet on the day you come in to see your GP.<br>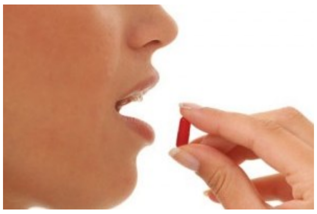	One tablet should be taken once every day, at night with cold water.<br>You need to take this medicine for 10 years.<br>You need to regularly visit a GP to receive a prescription.<br>You need to visit a pharmacy to fill a prescription.<br>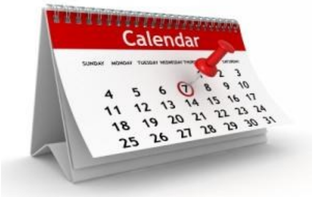<br>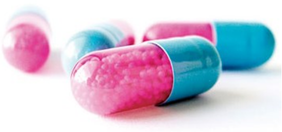

If you take Medicine A, you would live for **1 week less than 10 years** and then die.

If you take Medicine B, you would live for **10 years** and then die.

Would you prefer Medicine A or Medicine B, or are they the same?

Indicate your choice here. You can only choose one option:

◯ **Medicine A** Live for (10 years – 1 week)

◯ **Medicine B** Live for 10 years

◯ **Medicine A and Medicine B are the same.**

For peer review only

Repeated section

Remember Medicine A and Medicine B are equally effective and have **some risk of a side effect**.

The only difference between Medicine A and Medicine B is how you take them.

**Medicine A**	**Medicine B**
You take a tablet only once in your life.<br>You will be given the tablet on the day you come in to see your GP.<br>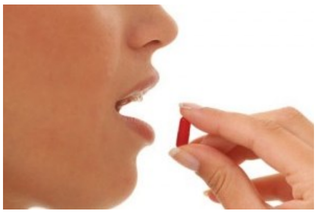	One tablet should be taken once every day, at night with cold water.<br>You need to take this medicine for 10 years.<br>You need to regularly visit a GP to receive a prescription.<br>You need to visit a pharmacy to fill a prescription.<br>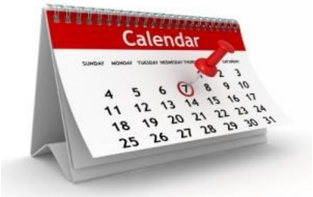<br>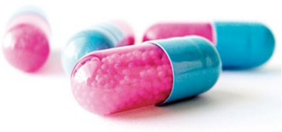

If you take Medicine A, you would live for **5 days less than 10 years** and then die.

If you take Medicine B, you would live for **10 years** and then die.

Would you prefer Medicine A or Medicine B, or are they the same?

Indicate your choice here. You can only choose one option:

◯ **Medicine A** Live for (10 years – 5 days)

◯ **Medicine B** Live for 10 years

◯ **Medicine A and Medicine B are the same.**

0% 100%

For peer review only

Repeated section

Remember Medicine A and Medicine B are equally effective and have **some risk of a side effect**.

The only difference between Medicine A and Medicine B is how you take them.

**Medicine A**	**Medicine B**
You take a tablet only once in your life.<br>You will be given the tablet on the day you come in to see your GP.<br>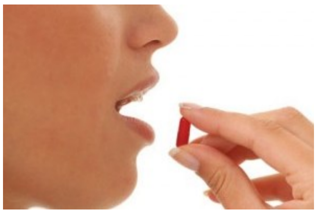	One tablet should be taken once every day, at night with cold water.<br>You need to take this medicine for 10 years.<br>You need to regularly visit a GP to receive a prescription.<br>You need to visit a pharmacy to fill a prescription.<br>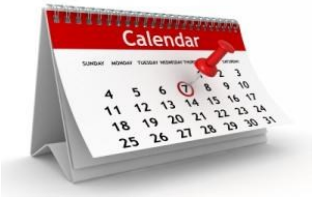<br>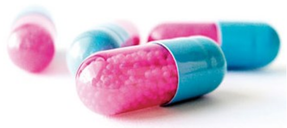

If you take Medicine A, you would live for **3 days less than 10 years** and then die.

If you take Medicine B, you would live for **10 years** and then die.

Would you prefer Medicine A or Medicine B, or are they the same?

Indicate your choice here. You can only choose one option:

◯ **Medicine A** Live for (10 years – 3 days)

◯ **Medicine B** Live for 10 years

◯ **Medicine A and Medicine B are the same.**

0% 100%

For peer review only

Repeated section

Remember Medicine A and Medicine B are equally effective and have **some risk of a side effect**.

The only difference between Medicine A and Medicine B is how you take them.

**Medicine A**	**Medicine B**
You take a tablet only once in your life.<br>You will be given the tablet on the day you come in to see your GP.<br>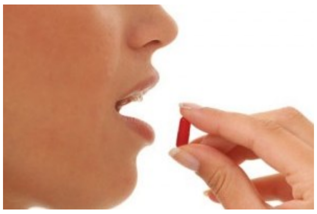	One tablet should be taken once every day, at night with cold water.<br>You need to take this medicine for 10 years.<br>You need to regularly visit a GP to receive a prescription.<br>You need to visit a pharmacy to fill a prescription.<br>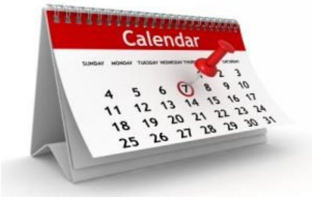<br>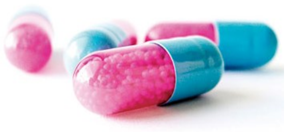

If you take Medicine A, you would live for **1 day less than 10 years** and then die.

If you take Medicine B, you would live for **10 years** and then die.

Would you prefer Medicine A or Medicine B, or are they the same?

Indicate your choice here. You can only choose one option:

○ **Medicine A** Live for (10 years – 1 day)

○ **Medicine B** Live for 10 years

○ **Medicine A and Medicine B are the same.**

For peer review only

Question 3a.

You prefer living for 5 years on Medicine A, to living for 10 years on Medicine B.

This means **living fewer than 5 years on Medicine A** can be the same as living for 10 years on Medicine B.

How many years on Medicine A do you think would be the same as living for 10 years on Medicine B?

(Please give a number in years)

0% 100%

peer review only

Part 2

Question 4

Now, Medicine A and Medicine B will give you less benefit than before because you belong to a group of people who may not respond to the treatment as well as other groups of people.

As before,

Medicine A is a pill that you only need to take once.

Medicine B is a pill that you need to take every day for ten years.

Medicine A and Medicine B are equally effective in reducing your risk of developing heart disease.

Medicine A and Medicine B will reduce your risk of having a heart attack or a stroke within the next 10 years from **10%** to **9%**.

In other words, in a crowd of **1,000** people in the UK, **100** are likely to have a heart attack or a stroke if they do not take the medicine. This is reduced to **90** people out of 1,000 who do take the medicine. This information is also illustrated in this picture.

Risk of heart disease

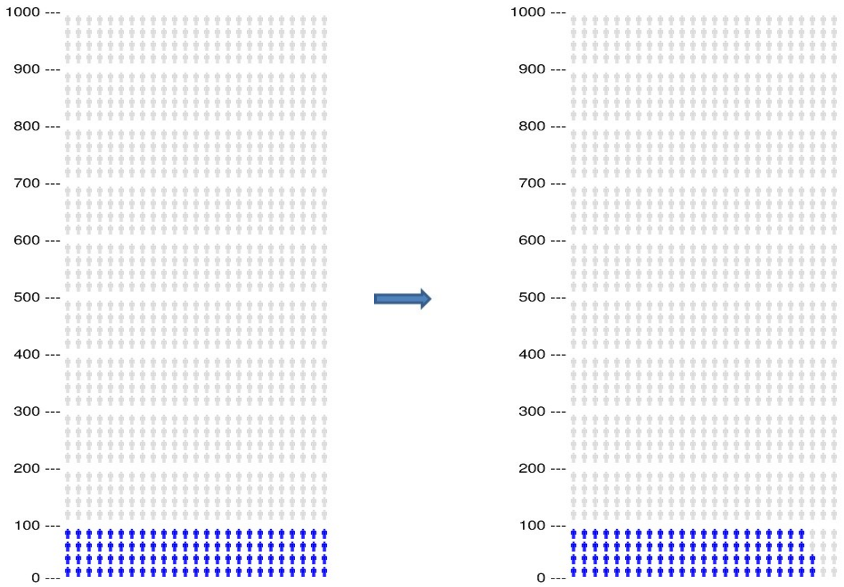

10%
No Treatment

9%
Taking the Medicine

(The figures shaded blue represent people who will have a heart attack or a stroke in the next 10 years)

In this scenario, you will not have any side effects from Medicine A or Medicine B.

Risk of a side effect

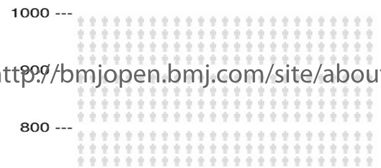

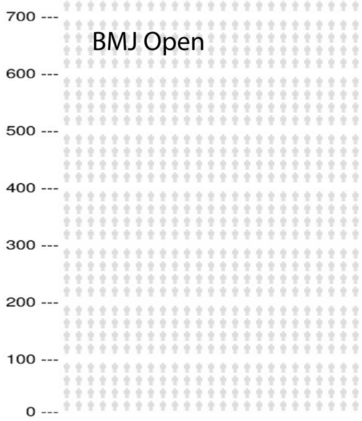

0%
No one will have a side effect.

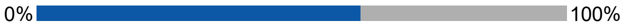

peer review only

Repeated section

Remember Medicine A and Medicine B are equally effective and have no risks of a side effect.

The only difference between Medicine A and Medicine B is how you take them.

Medicine A	Medicine B
You take a tablet only once in your life.<br>You will be given the tablet on the day you come in to see your GP.<br>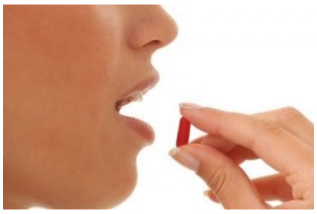	One tablet should be taken once every day, at night with cold water.<br>You need to take this medicine for 10 years.<br>You need to regularly visit a GP to receive a prescription.<br>You need to visit a pharmacy to fill a prescription.<br>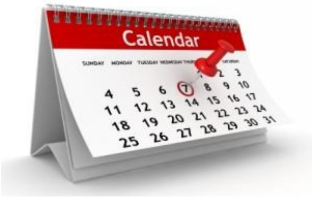<br><br>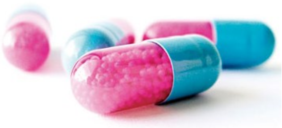

If you take Medicine A, you would live for **5 years** and then die.

If you take Medicine B, you would live for **10 years** and then die.

Would you prefer Medicine A or Medicine B, or are they the same?

Indicate your choice here. You can only choose one option:

◯ **Medicine A** 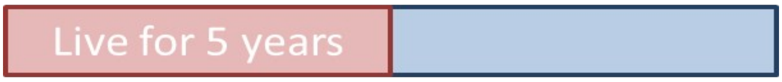

◯ **Medicine B** 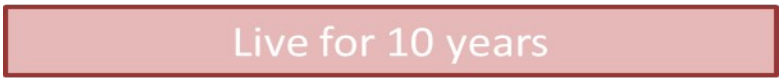

◯ **Medicine A and Medicine B are the same.**

For peer review only

Repeated section

Remember Medicine A and Medicine B are equally effective and have no risks of a side effect.

The only difference between Medicine A and Medicine B is how you take them.

**Medicine A**	**Medicine B**
You take a tablet only once in your life.<br>You will be given the tablet on the day you come in to see your GP.<br>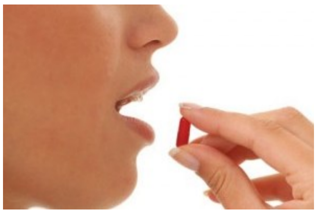	One tablet should be taken once every day, at night with cold water.<br>You need to take this medicine for 10 years.<br>You need to regularly visit a GP to receive a prescription.<br>You need to visit a pharmacy to fill a prescription.<br>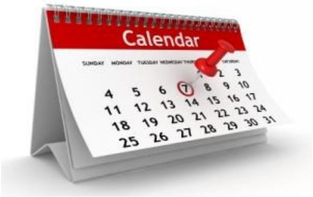<br>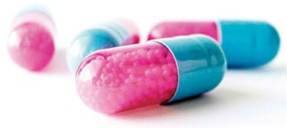

If you take Medicine A, you would live for **5.5 years (5 years and 6 months)** and then die.

If you take Medicine B, you would live for **10 years** and then die.

Would you prefer Medicine A or Medicine B, or are they the same?

Indicate your choice here. You can only choose one option:

◯ **Medicine A** Live for 5.5 years

◯ **Medicine B** Live for 10 years

◯ **Medicine A and Medicine B are the same.**

For peer review only

Repeated section

Remember Medicine A and Medicine B are equally effective and have no risks of a side effect.

The only difference between Medicine A and Medicine B is how you take them.

**Medicine A**	**Medicine B**
You take a tablet only once in your life.<br>You will be given the tablet on the day you come in to see your GP.<br>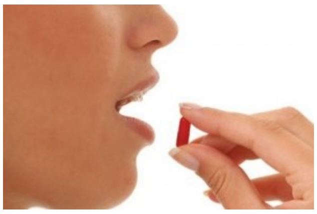	One tablet should be taken once every day, at night with cold water.<br>You need to take this medicine for 10 years.<br>You need to regularly visit a GP to receive a prescription.<br>You need to visit a pharmacy to fill a prescription.<br>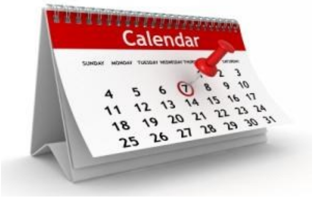<br>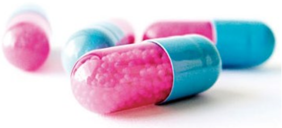

If you take Medicine A, you would live for **6 years** and then die.

If you take Medicine B, you would live for **10 years** and then die.

Would you prefer Medicine A or Medicine B, or are they the same?

Indicate your choice here. You can only choose one option:

◯ **Medicine A**

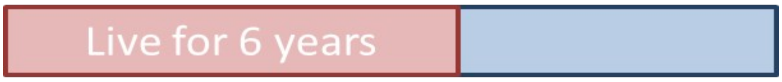

◯ **Medicine B**

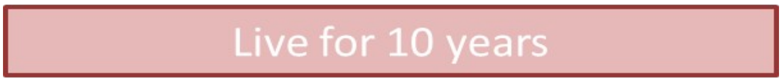

◯ **Medicine A and Medicine B are the same.**

0% 100%

For peer review only

Repeated section

Remember Medicine A and Medicine B are equally effective and have no risks of a side effect.

The only difference between Medicine A and Medicine B is how you take them.

Medicine A	Medicine B
You take a tablet only once in your life.<br>You will be given the tablet on the day you come in to see your GP.<br>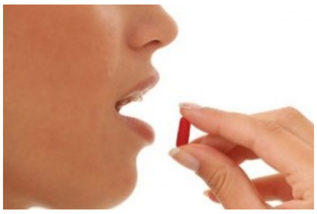	One tablet should be taken once every day, at night with cold water.<br>You need to take this medicine for 10 years.<br>You need to regularly visit a GP to receive a prescription.<br>You need to visit a pharmacy to fill a prescription.<br>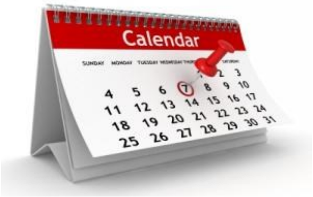 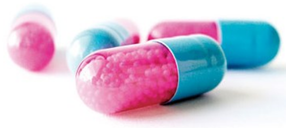

If you take Medicine A, you would live for **6.25 years (6 years and 3 months)** and then die.

If you take Medicine B, you would live for **10 years** and then die.

Would you prefer Medicine A or Medicine B, or are they the same?

Indicate your choice here. You can only choose one option:

◯ **Medicine A** Live for 6 years and 3 months

◯ **Medicine B** Live for 10 years

◯ **Medicine A and Medicine B are the same.**

For peer review only

Repeated section

Remember Medicine A and Medicine B are equally effective and have no risks of a side effect.

The only difference between Medicine A and Medicine B is how you take them.

**Medicine A**	**Medicine B**
You take a tablet only once in your life.<br>You will be given the tablet on the day you come in to see your GP.<br>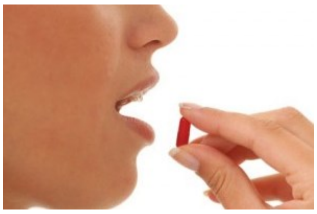	One tablet should be taken once every day, at night with cold water.<br>You need to take this medicine for 10 years.<br>You need to regularly visit a GP to receive a prescription.<br>You need to visit a pharmacy to fill a prescription.<br>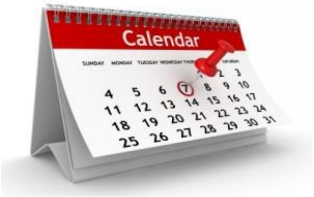<br><br>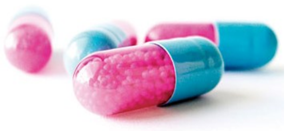

If you take Medicine A, you would live for **6.5 years** (6 years and 6 months) and then die.

If you take Medicine B, you would live for **10 years** and then die.

Would you prefer Medicine A or Medicine B, or are they the same?

Indicate your choice here. You can only choose one option:

○ **Medicine A** Live for 6.5 years

○ **Medicine B** Live for 10 years

○ **Medicine A and Medicine B are the same.**

For peer review only

Repeated section

Remember Medicine A and Medicine B are equally effective and have no risks of a side effect.

The only difference between Medicine A and Medicine B is how you take them.

**Medicine A**	**Medicine B**
You take a tablet only once in your life.<br>You will be given the tablet on the day you come in to see your GP.<br>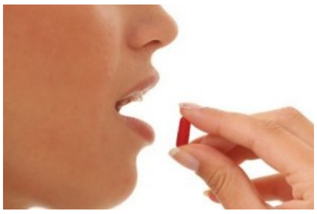	One tablet should be taken once every day, at night with cold water.<br>You need to take this medicine for 10 years.<br>You need to regularly visit a GP to receive a prescription.<br>You need to visit a pharmacy to fill a prescription.<br>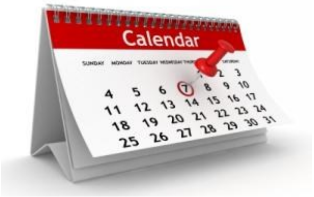<br>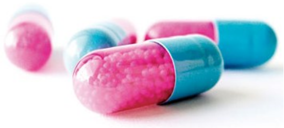

If you take Medicine A, you would live for **7 years** and then die.

If you take Medicine B, you would live for **10 years** and then die.

Would you prefer Medicine A or Medicine B, or are they the same?

Indicate your choice here. You can only choose one option:

◯ **Medicine A** Live for 7 years

◯ **Medicine B** Live for 10 years

◯ **Medicine A and Medicine B are the same.**

For peer review only

Repeated section

Remember Medicine A and Medicine B are equally effective and have no risks of a side effect.

The only difference between Medicine A and Medicine B is how you take them.

**Medicine A**	**Medicine B**
You take a tablet only once in your life.<br>You will be given the tablet on the day you come in to see your GP.<br>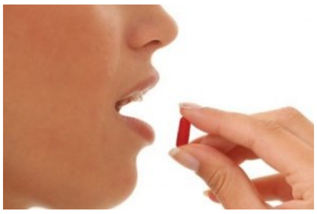	One tablet should be taken once every day, at night with cold water.<br>You need to take this medicine for 10 years.<br>You need to regularly visit a GP to receive a prescription.<br>You need to visit a pharmacy to fill a prescription.<br>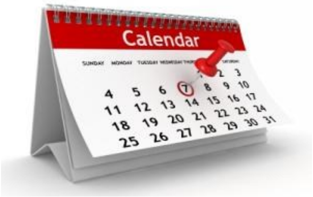<br>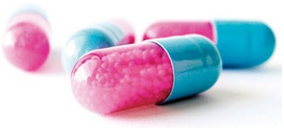

If you take Medicine A, you would live for **7.5 years (7 years and 6 months)** and then die.

If you take Medicine B, you would live for **10 years** and then die.

Would you prefer Medicine A or Medicine B, or are they the same?

Indicate your choice here. You can only choose one option:

◯ **Medicine A** Live for 7.5 years

◯ **Medicine B** Live for 10 years

◯ **Medicine A and Medicine B are the same.**

For peer review only

Repeated section

Remember Medicine A and Medicine B are equally effective and have no risks of a side effect.

The only difference between Medicine A and Medicine B is how you take them.

Medicine A	Medicine B
You take a tablet only once in your life.<br>You will be given the tablet on the day you come in to see your GP.<br>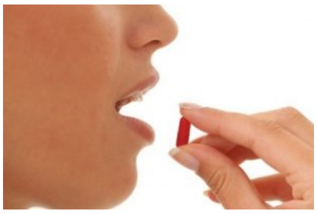	One tablet should be taken once every day, at night with cold water.<br>You need to take this medicine for 10 years.<br>You need to regularly visit a GP to receive a prescription.<br>You need to visit a pharmacy to fill a prescription.<br>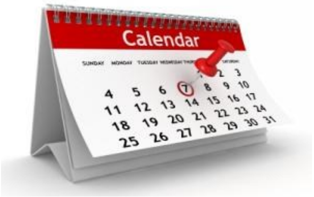<br>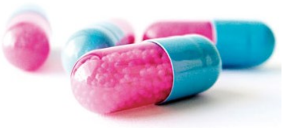

If you take Medicine A, you would live for **7.75 years (7 years and 9 months)** and then die.

If you take Medicine B, you would live for **10 years** and then die.

Would you prefer Medicine A or Medicine B, or are they the same?

Indicate your choice here. You can only choose one option:

◯ **Medicine A** 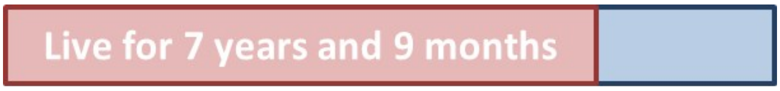

◯ **Medicine B** 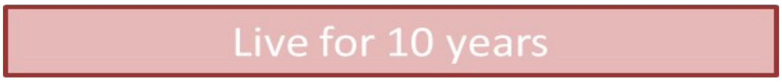

◯ **Medicine A and Medicine B are the same.**

0% 100%

For peer review only

Repeated section

Remember Medicine A and Medicine B are equally effective and have no risks of a side effect.

The only difference between Medicine A and Medicine B is how you take them.

Medicine A	Medicine B
You take a tablet only once in your life.<br>You will be given the tablet on the day you come in to see your GP.<br>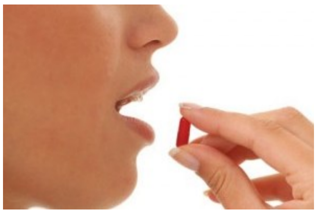	One tablet should be taken once every day, at night with cold water.<br>You need to take this medicine for 10 years.<br>You need to regularly visit a GP to receive a prescription.<br>You need to visit a pharmacy to fill a prescription.<br>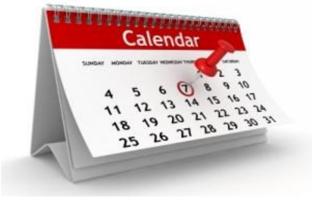 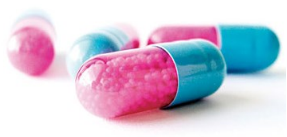

If you take Medicine A, you would live for **8 years** and then die.

If you take Medicine B, you would live for **10 years** and then die.

Would you prefer Medicine A or Medicine B, or are they the same?

Indicate your choice here. You can only choose one option:

○ **Medicine A** Live for 8 years

○ **Medicine B** Live for 10 years

○ **Medicine A and Medicine B are the same.**

0% 100%

For peer review only

Repeated section

Remember Medicine A and Medicine B are equally effective and have no risks of a side effect.

The only difference between Medicine A and Medicine B is how you take them.

Medicine A	Medicine B
You take a tablet only once in your life.<br>You will be given the tablet on the day you come in to see your GP.<br>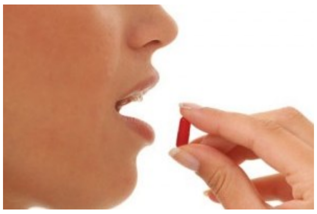	One tablet should be taken once every day, at night with cold water.<br>You need to take this medicine for 10 years.<br>You need to regularly visit a GP to receive a prescription.<br>You need to visit a pharmacy to fill a prescription.<br>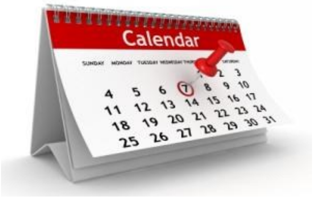<br>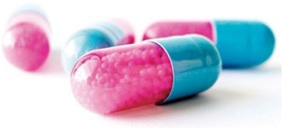

If you take Medicine A, you would live for **8.25 years (8 years and 3 months)** and then die.

If you take Medicine B, you would live for **10 years** and then die.

Would you prefer Medicine A or Medicine B, or are they the same?

Indicate your choice here. You can only choose one option:

○ **Medicine A** Live for 8 years and 3 months

○ **Medicine B** Live for 10 years

○ **Medicine A and Medicine B are the same.**

For peer review only

Repeated section

Remember Medicine A and Medicine B are equally effective and have no risks of a side effect.

The only difference between Medicine A and Medicine B is how you take them.

Medicine A	Medicine B
You take a tablet only once in your life.<br>You will be given the tablet on the day you come in to see your GP.<br>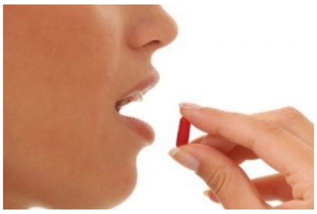	One tablet should be taken once every day, at night with cold water.<br>You need to take this medicine for 10 years.<br>You need to regularly visit a GP to receive a prescription.<br>You need to visit a pharmacy to fill a prescription.<br>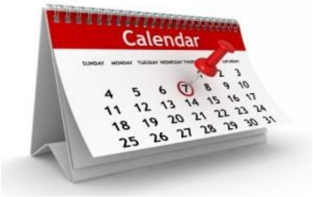<br>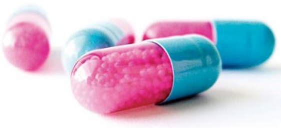

If you take Medicine A, you would live for **8.5 years** (8 years and 6 months) and then die.

If you take Medicine B, you would live for **10 years** and then die.

Would you prefer Medicine A or Medicine B, or are they the same?

Indicate your choice here. You can only choose one option:

◯ **Medicine A** Live for 8.5 years

◯ **Medicine B** Live for 10 years

◯ **Medicine A and Medicine B are the same.**

For peer review only

Repeated section

Remember Medicine A and Medicine B are equally effective and have no risks of a side effect.

The only difference between Medicine A and Medicine B is how you take them.

Medicine A	Medicine B
You take a tablet only once in your life.<br>You will be given the tablet on the day you come in to see your GP.<br>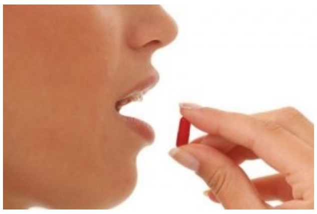	One tablet should be taken once every day, at night with cold water.<br>You need to take this medicine for 10 years.<br>You need to regularly visit a GP to receive a prescription.<br>You need to visit a pharmacy to fill a prescription.<br>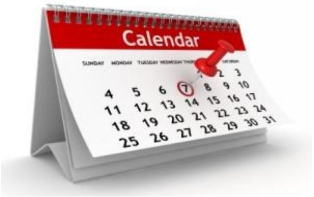<br>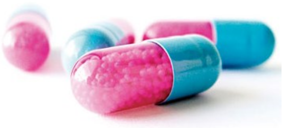

If you take Medicine A, you would live for **8.75 years (8 years and 9 months)** and then die.

If you take Medicine B, you would live for **10 years** and then die.

Would you prefer Medicine A or Medicine B, or are they the same?

Indicate your choice here. You can only choose one option:

◯ **Medicine A** Live for 8 years and 9 months

◯ **Medicine B** Live for 10 years

◯ **Medicine A and Medicine B are the same.**

0% 100%

For peer review only

Repeated section

Remember Medicine A and Medicine B are equally effective and have no risks of a side effect.

The only difference between Medicine A and Medicine B is how you take them.

Medicine A	Medicine B
You take a tablet only once in your life.<br>You will be given the tablet on the day you come in to see your GP.<br>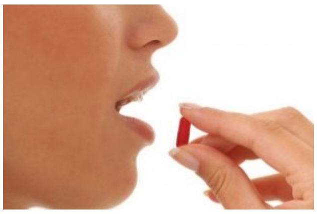	One tablet should be taken once every day, at night with cold water.<br>You need to take this medicine for 10 years.<br>You need to regularly visit a GP to receive a prescription.<br>You need to visit a pharmacy to fill a prescription.<br>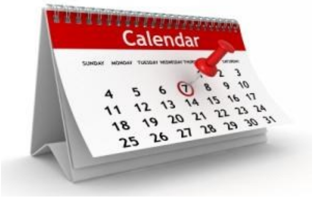<br>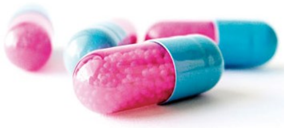

If you take Medicine A, you would live for **9 years** and then die.

If you take Medicine B, you would live for **10 years** and then die.

Would you prefer Medicine A or Medicine B, or are they the same?

Indicate your choice here. You can only choose one option:

◯ **Medicine A**

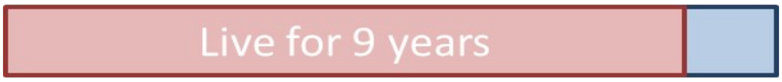

◯ **Medicine B**

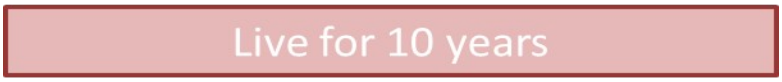

◯ **Medicine A and Medicine B are the same.**

For peer review only

Repeated section

Remember Medicine A and Medicine B are equally effective and have no risks of a side effect.

The only difference between Medicine A and Medicine B is how you take them.

**Medicine A**	**Medicine B**
You take a tablet only once in your life.<br>You will be given the tablet on the day you come in to see your GP.<br>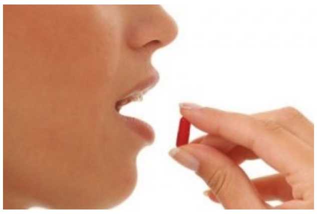	One tablet should be taken once every day, at night with cold water.<br>You need to take this medicine for 10 years.<br>You need to regularly visit a GP to receive a prescription.<br>You need to visit a pharmacy to fill a prescription.<br>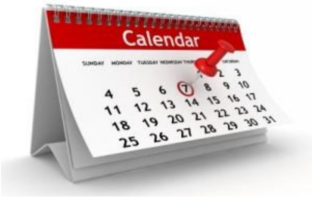<br>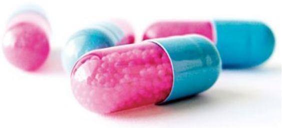

If you take Medicine A, you would live for **9 years and 1 month** and then die.

If you take Medicine B, you would live for **10 years** and then die.

Would you prefer Medicine A or Medicine B, or are they the same?

Indicate your choice here. You can only choose one option:

○ **Medicine A** Live for 9 years 1 month

○ **Medicine B** Live for 10 years

○ **Medicine A and Medicine B are the same.**

0% 100%

For peer review only

Repeated section

Remember Medicine A and Medicine B are equally effective and have no risks of a side effect.

The only difference between Medicine A and Medicine B is how you take them.

Medicine A	Medicine B
You take a tablet only once in your life.<br>You will be given the tablet on the day you come in to see your GP.<br>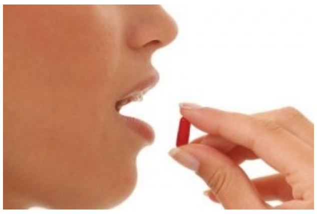	One tablet should be taken once every day, at night with cold water.<br>You need to take this medicine for 10 years.<br>You need to regularly visit a GP to receive a prescription.<br>You need to visit a pharmacy to fill a prescription.<br>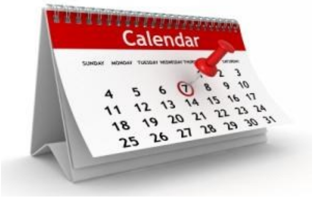<br><br>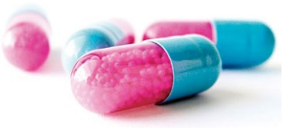

If you take Medicine A, you would live for **9.25 years (9 years and 3 months)** and then die.

If you take Medicine B, you would live for **10 years** and then die.

Would you prefer Medicine A or Medicine B, or are they the same?

Indicate your choice here. You can only choose one option:

◯ **Medicine A** Live for 9 years and 3 months

◯ **Medicine B** Live for 10 years

◯ **Medicine A and Medicine B are the same.**

0% 100%

For peer review only

Repeated section

Remember Medicine A and Medicine B are equally effective and have no risks of a side effect.

The only difference between Medicine A and Medicine B is how you take them.

Medicine A	Medicine B
You take a tablet only once in your life.<br>You will be given the tablet on the day you come in to see your GP.<br>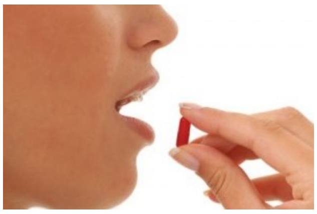	One tablet should be taken once every day, at night with cold water.<br>You need to take this medicine for 10 years.<br>You need to regularly visit a GP to receive a prescription.<br>You need to visit a pharmacy to fill a prescription.<br>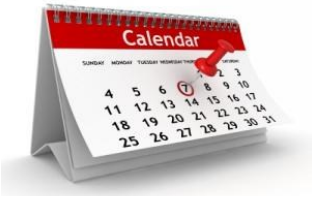<br>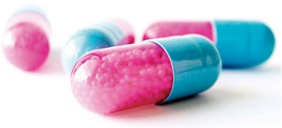

If you take Medicine A, you would live for **9 years and 4 months** and then die.

If you take Medicine B, you would live for **10 years** and then die.

Would you prefer Medicine A or Medicine B, or are they the same?

Indicate your choice here. You can only choose one option:

◯ **Medicine A** Live for 9 years 4 months

◯ **Medicine B** Live for 10 years

◯ **Medicine A and Medicine B are the same.**

For peer review only

Repeated section

Remember Medicine A and Medicine B are equally effective and have no risks of a side effect.

The only difference between Medicine A and Medicine B is how you take them.

**Medicine A**	**Medicine B**
You take a tablet only once in your life.<br>You will be given the tablet on the day you come in to see your GP.<br>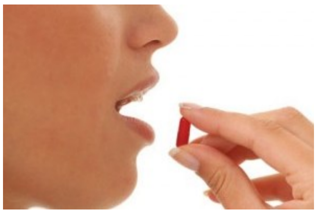	One tablet should be taken once every day, at night with cold water.<br>You need to take this medicine for 10 years.<br>You need to regularly visit a GP to receive a prescription.<br>You need to visit a pharmacy to fill a prescription.<br>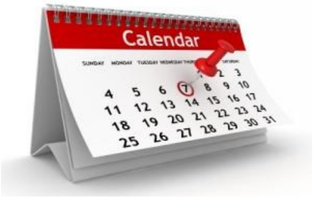<br>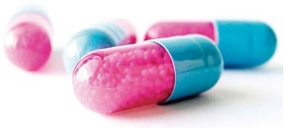

If you take Medicine A, you would live for **9 years and 5 months** and then die.

If you take Medicine B, you would live for **10 years** and then die.

Would you prefer Medicine A or Medicine B, or are they the same?

Indicate your choice here. You can only choose one option:

○ **Medicine A** Live for 9 years 5 months

○ **Medicine B** Live for 10 years

○ **Medicine A and Medicine B are the same.**

For peer review only

Repeated section

Remember Medicine A and Medicine B are equally effective and have no risks of a side effect.

The only difference between Medicine A and Medicine B is how you take them.

Medicine A	Medicine B
You take a tablet only once in your life.<br>You will be given the tablet on the day you come in to see your GP.<br>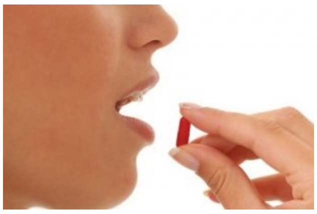	One tablet should be taken once every day, at night with cold water.<br>You need to take this medicine for 10 years.<br>You need to regularly visit a GP to receive a prescription.<br>You need to visit a pharmacy to fill a prescription.<br>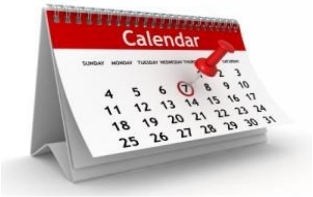<br>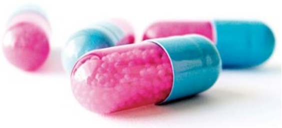

If you take Medicine A, you would live for **9.5 years** (9 years and 6 months) and then die.

If you take Medicine B, you would live for **10 years** and then die.

Would you prefer Medicine A or Medicine B, or are they the same?

Indicate your choice here. You can only choose one option:

◯ **Medicine A** 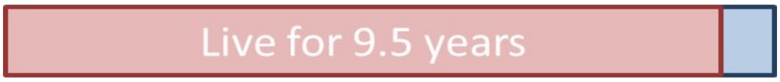

◯ **Medicine B** 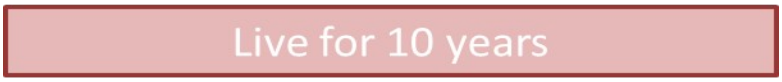

◯ **Medicine A and Medicine B are the same.**

For peer review only

Repeated section

Remember Medicine A and Medicine B are equally effective and have no risks of a side effect.

The only difference between Medicine A and Medicine B is how you take them.

**Medicine A**	**Medicine B**
You take a tablet only once in your life.<br>You will be given the tablet on the day you come in to see your GP.<br>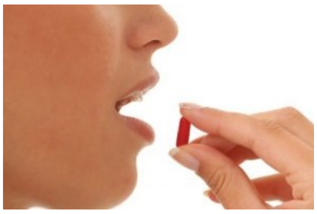	One tablet should be taken once every day, at night with cold water.<br>You need to take this medicine for 10 years.<br>You need to regularly visit a GP to receive a prescription.<br>You need to visit a pharmacy to fill a prescription.<br>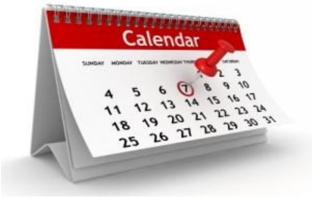<br>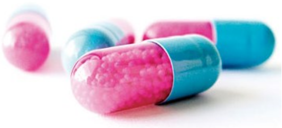

If you take Medicine A, you would live for **5 months less than 10 years** and then die.

If you take Medicine B, you would live for **10 years** and then die.

Would you prefer Medicine A or Medicine B, or are they the same?

Indicate your choice here. You can only choose one option:

○ **Medicine A** Live for (10 years – 5 months)

○ **Medicine B** Live for 10 years

○ **Medicine A and Medicine B are the same.**

For peer review only

Repeated section

Remember Medicine A and Medicine B are equally effective and have no risks of a side effect.

The only difference between Medicine A and Medicine B is how you take them.

**Medicine A**	**Medicine B**
You take a tablet only once in your life.<br>You will be given the tablet on the day you come in to see your GP.<br>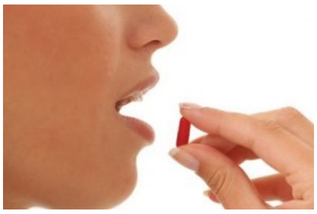	One tablet should be taken once every day, at night with cold water.<br>You need to take this medicine for 10 years.<br>You need to regularly visit a GP to receive a prescription.<br>You need to visit a pharmacy to fill a prescription.<br>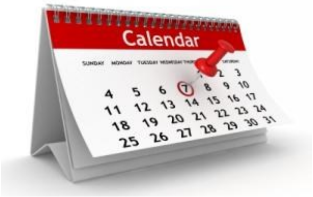<br>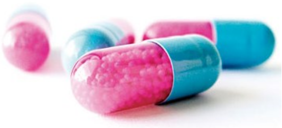

If you take Medicine A, you would live for **4 months less than 10 years** and then die.

If you take Medicine B, you would live for **10 years** and then die.

Would you prefer Medicine A or Medicine B, or are they the same?

Indicate your choice here. You can only choose one option:

◯ **Medicine A** Live for (10 years – 4 months)

◯ **Medicine B** Live for 10 years

◯ **Medicine A and Medicine B are the same.**

For peer review only

Repeated section

Remember Medicine A and Medicine B are equally effective and have no risks of a side effect.

The only difference between Medicine A and Medicine B is how you take them.

Medicine A	Medicine B
You take a tablet only once in your life.<br>You will be given the tablet on the day you come in to see your GP.<br>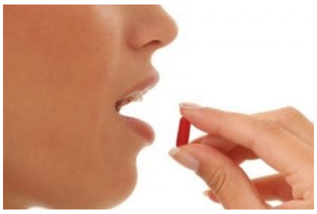	One tablet should be taken once every day, at night with cold water.<br>You need to take this medicine for 10 years.<br>You need to regularly visit a GP to receive a prescription.<br>You need to visit a pharmacy to fill a prescription.<br>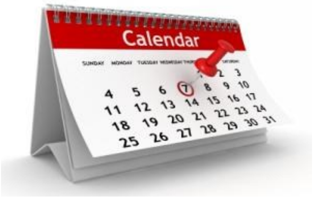<br>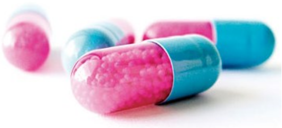

If you take Medicine A, you would live for **3 months less than 10 years** and then die.

If you take Medicine B, you would live for **10 years** and then die.

Would you prefer Medicine A or Medicine B, or are they the same?

Indicate your choice here. You can only choose one option:

◯ **Medicine A** Live for (10 years – 3 months)

◯ **Medicine B** Live for 10 years

◯ **Medicine A and Medicine B are the same.**

For peer review only

Repeated section

Remember Medicine A and Medicine B are equally effective and have no risks of a side effect.

The only difference between Medicine A and Medicine B is how you take them.

Medicine A	Medicine B
You take a tablet only once in your life.<br>You will be given the tablet on the day you come in to see your GP.<br>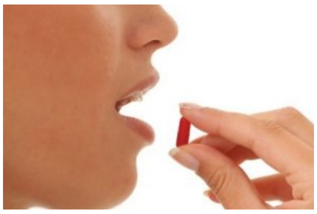	One tablet should be taken once every day, at night with cold water.<br>You need to take this medicine for 10 years.<br>You need to regularly visit a GP to receive a prescription.<br>You need to visit a pharmacy to fill a prescription.<br>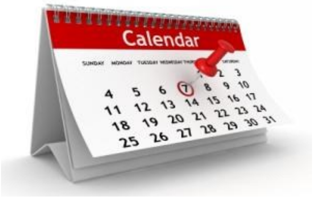<br>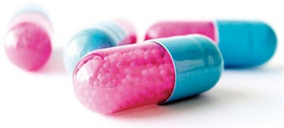

If you take Medicine A, you would live for **2 months less than 10 years** and then die.

If you take Medicine B, you would live for **10 years** and then die.

Would you prefer Medicine A or Medicine B, or are they the same?

Indicate your choice here. You can only choose one option:

◯ **Medicine A** Live for (10 years – 2 months)

◯ **Medicine B** Live for 10 years

◯ **Medicine A and Medicine B are the same.**

For peer review only

Repeated section

Remember Medicine A and Medicine B are equally effective and have no risks of a side effect.

The only difference between Medicine A and Medicine B is how you take them.

Medicine A	Medicine B
You take a tablet only once in your life.<br>You will be given the tablet on the day you come in to see your GP.<br>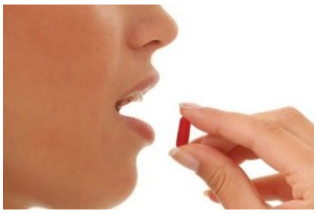	One tablet should be taken once every day, at night with cold water.<br>You need to take this medicine for 10 years.<br>You need to regularly visit a GP to receive a prescription.<br>You need to visit a pharmacy to fill a prescription.<br>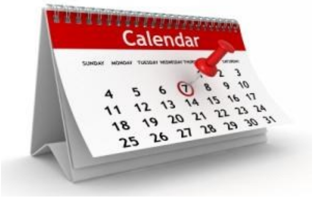 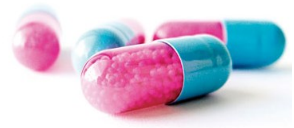

If you take Medicine A, you would live for **1 month less than 10 years** and then die.

If you take Medicine B, you would live for **10 years** and then die.

Would you prefer Medicine A or Medicine B, or are they the same?

Indicate your choice here. You can only choose one option:

◯ **Medicine A** Live for (10 years – 1 month)

◯ **Medicine B** Live for 10 years

◯ **Medicine A and Medicine B are the same.**

For peer review only

Repeated section

Remember Medicine A and Medicine B are equally effective and have no risks of a side effect.

The only difference between Medicine A and Medicine B is how you take them.

Medicine A	Medicine B
You take a tablet only once in your life.<br>You will be given the tablet on the day you come in to see your GP.<br>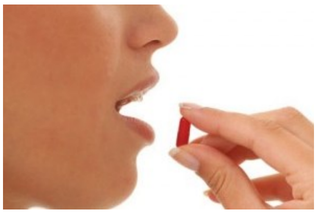	One tablet should be taken once every day, at night with cold water.<br>You need to take this medicine for 10 years.<br>You need to regularly visit a GP to receive a prescription.<br>You need to visit a pharmacy to fill a prescription.<br>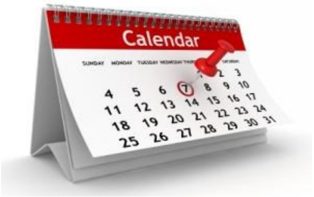<br>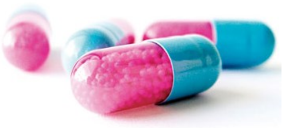

If you take Medicine A, you would live for **3 weeks less than 10 years** and then die.

If you take Medicine B, you would live for **10 years** and then die.

Would you prefer Medicine A or Medicine B, or are they the same?

Indicate your choice here. You can only choose one option:

◯ **Medicine A** Live for (10 years – 3 weeks)

◯ **Medicine B** Live for 10 years

◯ **Medicine A and Medicine B are the same.**

For peer review only

Repeated section

Remember Medicine A and Medicine B are equally effective and have no risks of a side effect.

The only difference between Medicine A and Medicine B is how you take them.

Medicine A	Medicine B
You take a tablet only once in your life.<br>You will be given the tablet on the day you come in to see your GP.<br>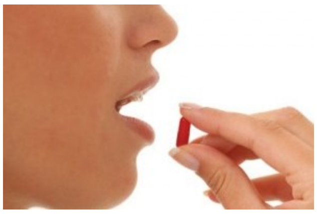	One tablet should be taken once every day, at night with cold water.<br>You need to take this medicine for 10 years.<br>You need to regularly visit a GP to receive a prescription.<br>You need to visit a pharmacy to fill a prescription.<br>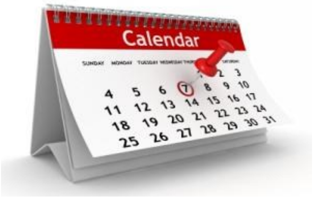<br><br>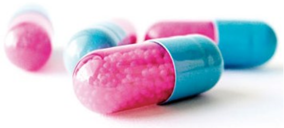

If you take Medicine A, you would live for **2 weeks less than 10 years** and then die.

If you take Medicine B, you would live for **10 years** and then die.

Would you prefer Medicine A or Medicine B, or are they the same?

Indicate your choice here. You can only choose one option:

○ **Medicine A** Live for (10 years – 2 weeks)

○ **Medicine B** Live for 10 years

○ **Medicine A and Medicine B are the same.**

For peer review only

Repeated section

Remember Medicine A and Medicine B are equally effective and have no risks of a side effect.

The only difference between Medicine A and Medicine B is how you take them.

**Medicine A**	**Medicine B**
You take a tablet only once in your life.<br>You will be given the tablet on the day you come in to see your GP.<br>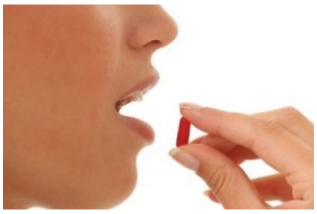	One tablet should be taken once every day, at night with cold water.<br>You need to take this medicine for 10 years.<br>You need to regularly visit a GP to receive a prescription.<br>You need to visit a pharmacy to fill a prescription.<br>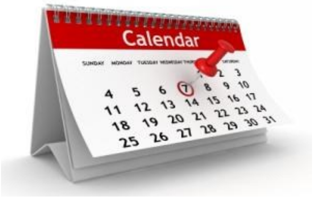<br>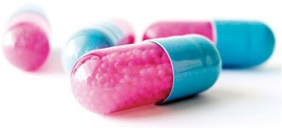

If you take Medicine A, you would live for **10 days less than 10 years** and then die.

If you take Medicine B, you would live for **10 years** and then die.

Would you prefer Medicine A or Medicine B, or are they the same?

Indicate your choice here. You can only choose one option:

○ **Medicine A** Live for (10 years – 10 days)

○ **Medicine B** Live for 10 years

○ **Medicine A and Medicine B are the same.**

0% 100%

For peer review only

Repeated section

Remember Medicine A and Medicine B are equally effective and have no risks of a side effect.

The only difference between Medicine A and Medicine B is how you take them.

Medicine A	Medicine B
You take a tablet only once in your life.<br>You will be given the tablet on the day you come in to see your GP.<br>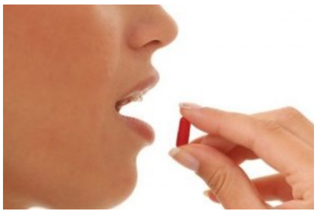	One tablet should be taken once every day, at night with cold water.<br>You need to take this medicine for 10 years.<br>You need to regularly visit a GP to receive a prescription.<br>You need to visit a pharmacy to fill a prescription.<br>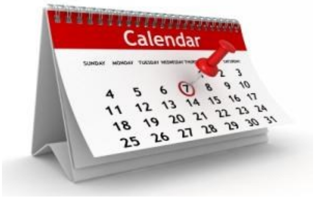<br>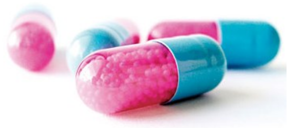

If you take Medicine A, you would live for **1 week less than 10 years** and then die.

If you take Medicine B, you would live for **10 years** and then die.

Would you prefer Medicine A or Medicine B, or are they the same?

Indicate your choice here. You can only choose one option:

○ **Medicine A** Live for (10 years – 1 week)

○ **Medicine B** Live for 10 years

○ **Medicine A and Medicine B are the same.**

0% 100%

For peer review only

Repeated section

Remember Medicine A and Medicine B are equally effective and have no risks of a side effect.

The only difference between Medicine A and Medicine B is how you take them.

Medicine A	Medicine B
You take a tablet only once in your life.<br>You will be given the tablet on the day you come in to see your GP.<br>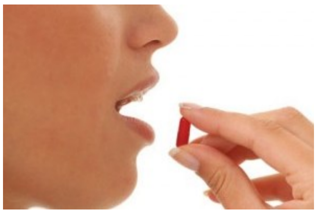	One tablet should be taken once every day, at night with cold water.<br>You need to take this medicine for 10 years.<br>You need to regularly visit a GP to receive a prescription.<br>You need to visit a pharmacy to fill a prescription.<br>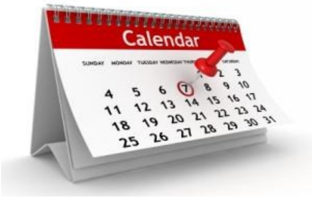<br>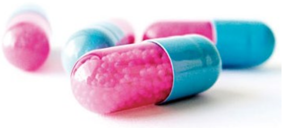

If you take Medicine A, you would live for **5 days less than 10 years** and then die.

If you take Medicine B, you would live for **10 years** and then die.

Would you prefer Medicine A or Medicine B, or are they the same?

Indicate your choice here. You can only choose one option:

○ **Medicine A** — Live for (10 years – 5 days)

○ **Medicine B** — Live for 10 years

○ **Medicine A and Medicine B are the same.**

0% 100%

For peer review only

Repeated section

Remember Medicine A and Medicine B are equally effective and have no risks of a side effect.

The only difference between Medicine A and Medicine B is how you take them.

Medicine A	Medicine B
You take a tablet only once in your life.<br>You will be given the tablet on the day you come in to see your GP.<br>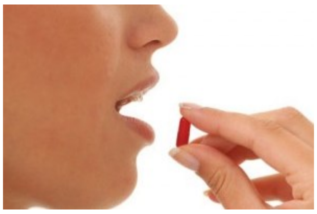	One tablet should be taken once every day, at night with cold water.<br>You need to take this medicine for 10 years.<br>You need to regularly visit a GP to receive a prescription.<br>You need to visit a pharmacy to fill a prescription.<br>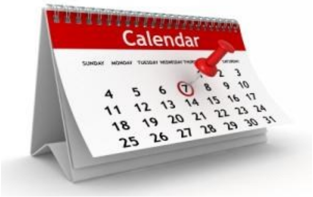 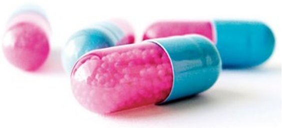

If you take Medicine A, you would live for **3 days less than 10 years** and then die.

If you take Medicine B, you would live for **10 years** and then die.

Would you prefer Medicine A or Medicine B, or are they the same?

Indicate your choice here. You can only choose one option:

◯ **Medicine A** Live for (10 years – 3 days)

◯ **Medicine B** Live for 10 years

◯ **Medicine A and Medicine B are the same.**

For peer review only

Repeated section

Remember Medicine A and Medicine B are equally effective and have no risks of a side effect.

The only difference between Medicine A and Medicine B is how you take them.

Medicine A	Medicine B
You take a tablet only once in your life.<br>You will be given the tablet on the day you come in to see your GP.<br>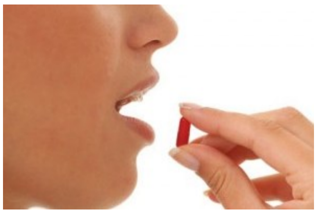	One tablet should be taken once every day, at night with cold water.<br>You need to take this medicine for 10 years.<br>You need to regularly visit a GP to receive a prescription.<br>You need to visit a pharmacy to fill a prescription.<br>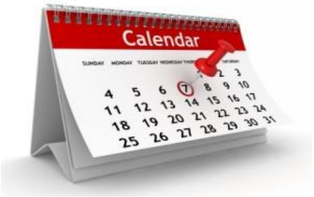<br>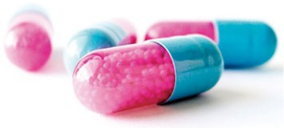

If you take Medicine A, you would live for **1 day less than 10 years** and then die.

If you take Medicine B, you would live for **10 years** and then die.

Would you prefer Medicine A or Medicine B, or are they the same?

Indicate your choice here. You can only choose one option:

○ **Medicine A** — Live for (10 years – 1 day)

○ **Medicine B** — Live for 10 years

○ **Medicine A and Medicine B are the same.**

0% 100%

For peer review only

Question 4a.

You prefer living for 5 years on Medicine A, to living for 10 years on Medicine B.

This means **living fewer than 5 years on Medicine A** can be the same as living for 10 years on Medicine B.

How many years on Medicine A do you think would be the same as living for 10 years on Medicine B?

(Please give a number in years)

0% 100%

peer review only

Was there anything so far which you think is unclear or that you didn't understand?

(Please provide details in the comment box below)

0% 100%

peer review only

Part 2a

Question 5

You have been told that you need to take a medicine to reduce the risk of heart disease.

You can choose between two different medicines. We have called these Medicine A and Medicine B.

Medicine A is a pill that you only need to take once.

Medicine B is a pill that you need to take every day for ten years.

Medicine A and Medicine B are equally effective in reducing your risk of developing heart disease.

Medicine A and Medicine B will reduce your risk of having a heart attack or a stroke within the next 10 years from **10%** to **6%**.

In other words, in a crowd of 1,000 people in the UK, **100** are likely to have a heart attack or a stroke if they do not take the medicine. This is reduced to **60** people out of 1,000 who do take the medicine. This information is also illustrated in this picture.

Risk of heart disease

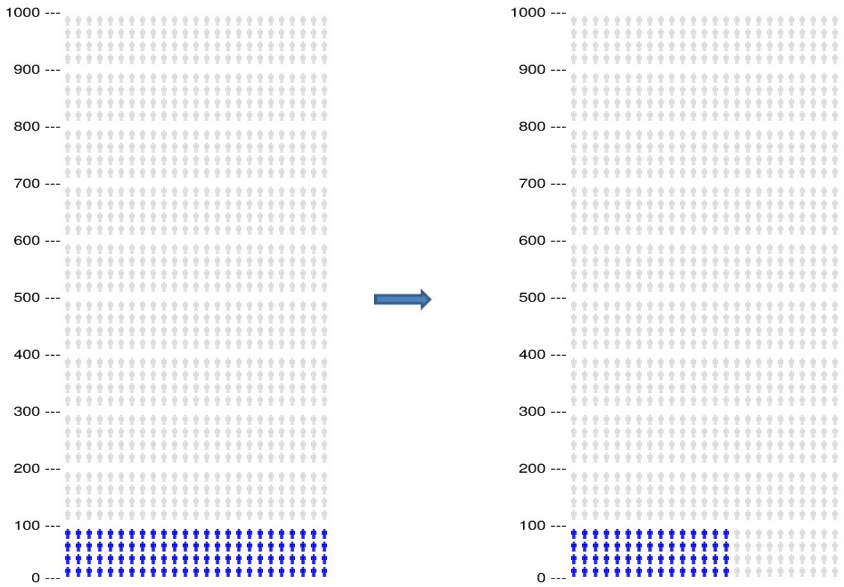

10%
No Treatment

6%
Taking the Medicine

(The figures shaded blue represent people who will have a heart attack or a stroke in the next 10 years)

Please continue to the next page.

For peer review only

Repeated section

Remember Medicine A and Medicine B are equally effective.

However, there are some differences between Medicine A and Medicine B.

Medicine A	Medicine B
**No inconvenience**<br><br>You take a tablet only once in your life.<br>You will be given the tablet on the day you come in to see your GP.	**Inconvenience**<br><br>One tablet should be taken once every day, at night with cold water.<br>You need to take this medicine for 10 years.<br>You need to regularly visit a GP to receive a prescription.<br>You need to visit a pharmacy to fill a prescription.
**Severe side effect - 0%**<br><br>No one taking Medicine A will have a severe side effect.	**Severe side effect - 0.7%**<br><br>Among every 1,000 people taking Medicine B, **7** will experience a severe side effect.
**Minor side effect - 0%**<br><br>No one taking Medicine A will have a minor side effect.	**Minor side effect - 13%**<br><br>Among every 1,000 people taking Medicine B, **130** will experience a minor side effect.

If you take Medicine A, you would live for **5 years** and then die.

If you take Medicine B, you would live for **10 years** and then die.

Would you prefer Medicine A or Medicine B, or are they the same?

Indicate your choice here. You can only choose one option:

○ **Medicine A** Live for 5 years

○ **Medicine B** Live for 10 years

○ **Medicine A and Medicine B are the same.**

0% 100%

Repeated section

Remember Medicine A and Medicine B are equally effective.

However, there are some differences between Medicine A and Medicine B.

Medicine A	Medicine B
**No inconvenience**<br><br>You take a tablet only once in your life.<br>You will be given the tablet on the day you come in to see your GP.	**Inconvenience**<br><br>One tablet should be taken once every day, at night with cold water.<br>You need to take this medicine for 10 years.<br>You need to regularly visit a GP to receive a prescription.<br>You need to visit a pharmacy to fill a prescription.
**Severe side effect - 0%**<br><br>No one taking Medicine A will have a severe side effect.	**Severe side effect - 0.7%**<br><br>Among every 1,000 people taking Medicine B, **7** will experience a severe side effect.
**Minor side effect - 0%**<br><br>No one taking Medicine A will have a minor side effect.	**Minor side effect - 13%**<br><br>Among every 1,000 people taking Medicine B, **130** will experience a minor side effect.

If you take Medicine A, you would live for **5.5 years** (5 years and 6 months) and then die.

If you take Medicine B, you would live for **10 years** and then die.

Would you prefer Medicine A or Medicine B, or are they the same?

Indicate your choice here. You can only choose one option:

○ **Medicine A** Live for 5.5 years

○ **Medicine B** Live for 10 years

○ **Medicine A and Medicine B are the same.**

0% 100%

Repeated section

Remember Medicine A and Medicine B are equally effective.

However, there are some differences between Medicine A and Medicine B.

Medicine A	Medicine B
**No inconvenience**<br><br>You take a tablet only once in your life.<br>You will be given the tablet on the day you come in to see your GP.	**Inconvenience**<br><br>One tablet should be taken once every day, at night with cold water.<br>You need to take this medicine for 10 years.<br>You need to regularly visit a GP to receive a prescription.<br>You need to visit a pharmacy to fill a prescription.
**Severe side effect - 0%**<br><br>No one taking Medicine A will have a severe side effect.	**Severe side effect - 0.7%**<br><br>Among every 1,000 people taking Medicine B, **7** will experience a severe side effect.
**Minor side effect - 0%**<br><br>No one taking Medicine A will have a minor side effect.	**Minor side effect - 13%**<br><br>Among every 1,000 people taking Medicine B, **130** will experience a minor side effect.

If you take Medicine A, you would live for **6 years** and then die.

If you take Medicine B, you would live for **10 years** and then die.

Would you prefer Medicine A or Medicine B, or are they the same?

Indicate your choice here. You can only choose one option:

○ **Medicine A** Live for 6 years

○ **Medicine B** Live for 10 years

○ **Medicine A and Medicine B are the same.**

0% 100%

Repeated section

Remember Medicine A and Medicine B are equally effective.

However, there are some differences between Medicine A and Medicine B.

Medicine A	Medicine B
**No inconvenience**<br><br>You take a tablet only once in your life.<br>You will be given the tablet on the day you come in to see your GP.	**Inconvenience**<br><br>One tablet should be taken once every day, at night with cold water.<br>You need to take this medicine for 10 years.<br>You need to regularly visit a GP to receive a prescription.<br>You need to visit a pharmacy to fill a prescription.
**Severe side effect - 0%**<br><br>No one taking Medicine A will have a severe side effect.	**Severe side effect - 0.7%**<br><br>Among every 1,000 people taking Medicine B, **7** will experience a severe side effect.
**Minor side effect - 0%**<br><br>No one taking Medicine A will have a minor side effect.	**Minor side effect - 13%**<br><br>Among every 1,000 people taking Medicine B, **130** will experience a minor side effect.

If you take Medicine A, you would live for **6.25 years (6 years and 3 months)** and then die.

If you take Medicine B, you would live for **10 years** and then die.

Would you prefer Medicine A or Medicine B, or are they the same?

Indicate your choice here. You can only choose one option:

◯ **Medicine A** Live for 6 years and 3 months

◯ **Medicine B** Live for 10 years

◯ **Medicine A and Medicine B are the same.**

0% 100%

Repeated section

Remember Medicine A and Medicine B are equally effective.

However, there are some differences between Medicine A and Medicine B.

Medicine A	Medicine B
**No inconvenience**<br><br>You take a tablet only once in your life.<br>You will be given the tablet on the day you come in to see your GP.	**Inconvenience**<br><br>One tablet should be taken once every day, at night with cold water.<br>You need to take this medicine for 10 years.<br>You need to regularly visit a GP to receive a prescription.<br>You need to visit a pharmacy to fill a prescription.
**Severe side effect - 0%**<br><br>No one taking Medicine A will have a severe side effect.	**Severe side effect - 0.7%**<br><br>Among every 1,000 people taking Medicine B, **7** will experience a severe side effect.
**Minor side effect - 0%**<br><br>No one taking Medicine A will have a minor side effect.	**Minor side effect - 13%**<br><br>Among every 1,000 people taking Medicine B, **130** will experience a minor side effect.

If you take Medicine A, you would live for **6.5 years** (6 years and 6 months) and then die.

If you take Medicine B, you would live for **10 years** and then die.

Would you prefer Medicine A or Medicine B, or are they the same?

Indicate your choice here. You can only choose one option:

○ **Medicine A**

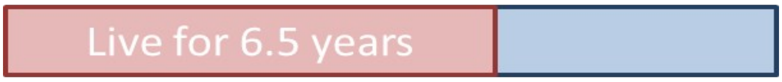

○ **Medicine B**

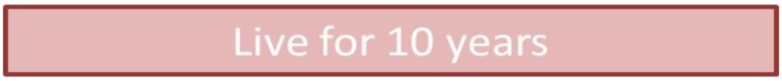

○ **Medicine A and Medicine B are the same.**

0% 100%

Repeated section

Remember Medicine A and Medicine B are equally effective.

However, there are some differences between Medicine A and Medicine B.

Medicine A	Medicine B
**No inconvenience**<br><br>You take a tablet only once in your life.<br>You will be given the tablet on the day you come in to see your GP.	**Inconvenience**<br><br>One tablet should be taken once every day, at night with cold water.<br>You need to take this medicine for 10 years.<br>You need to regularly visit a GP to receive a prescription.<br>You need to visit a pharmacy to fill a prescription.
**Severe side effect - 0%**<br><br>No one taking Medicine A will have a severe side effect.	**Severe side effect - 0.7%**<br><br>Among every 1,000 people taking Medicine B, **7** will experience a severe side effect.
**Minor side effect - 0%**<br><br>No one taking Medicine A will have a minor side effect.	**Minor side effect - 13%**<br><br>Among every 1,000 people taking Medicine B, **130** will experience a minor side effect.

If you take Medicine A, you would live for **7 years** and then die.

If you take Medicine B, you would live for **10 years** and then die.

Would you prefer Medicine A or Medicine B, or are they the same?

Indicate your choice here. You can only choose one option:

◯ **Medicine A** Live for 7 years

◯ **Medicine B** Live for 10 years

◯ **Medicine A and Medicine B are the same.**

0% 100%

Repeated section

Remember Medicine A and Medicine B are equally effective.

However, there are some differences between Medicine A and Medicine B.

Medicine A	Medicine B
**No inconvenience**<br><br>You take a tablet only once in your life.<br>You will be given the tablet on the day you come in to see your GP.	**Inconvenience**<br><br>One tablet should be taken once every day, at night with cold water.<br>You need to take this medicine for 10 years.<br>You need to regularly visit a GP to receive a prescription.<br>You need to visit a pharmacy to fill a prescription.
**Severe side effect - 0%**<br><br>No one taking Medicine A will have a severe side effect.	**Severe side effect - 0.7%**<br><br>Among every 1,000 people taking Medicine B, **7** will experience a severe side effect.
**Minor side effect - 0%**<br><br>No one taking Medicine A will have a minor side effect.	**Minor side effect - 13%**<br><br>Among every 1,000 people taking Medicine B, **130** will experience a minor side effect.

If you take Medicine A, you would live for **7.5 years** (7 years and 6 months) and then die.

If you take Medicine B, you would live for **10 years** and then die.

Would you prefer Medicine A or Medicine B, or are they the same?

Indicate your choice here. You can only choose one option:

◯ **Medicine A**

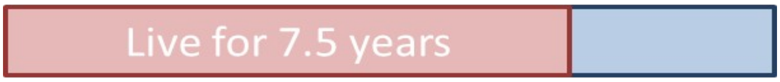

◯ **Medicine B**

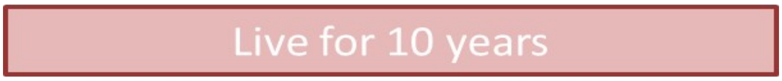

◯ **Medicine A and Medicine B are the same.**

0% 100%

Repeated section

Remember Medicine A and Medicine B are equally effective.

However, there are some differences between Medicine A and Medicine B.

Medicine A	Medicine B
**No inconvenience**<br><br>You take a tablet only once in your life.<br>You will be given the tablet on the day you come in to see your GP.	**Inconvenience**<br><br>One tablet should be taken once every day, at night with cold water.<br>You need to take this medicine for 10 years.<br>You need to regularly visit a GP to receive a prescription.<br>You need to visit a pharmacy to fill a prescription.
**Severe side effect - 0%**<br><br>No one taking Medicine A will have a severe side effect.	**Severe side effect - 0.7%**<br><br>Among every 1,000 people taking Medicine B, **7** will experience a severe side effect.
**Minor side effect - 0%**<br><br>No one taking Medicine A will have a minor side effect.	**Minor side effect - 13%**<br><br>Among every 1,000 people taking Medicine B, **130** will experience a minor side effect.

If you take Medicine A, you would live for **7.75 years (7 years and 9 months)** and then die.

If you take Medicine B, you would live for **10 years** and then die.

Would you prefer Medicine A or Medicine B, or are they the same?

Indicate your choice here. You can only choose one option:

○ **Medicine A** Live for 7 years and 9 months

○ **Medicine B** Live for 10 years

○ **Medicine A and Medicine B are the same.**

0% 100%

Repeated section

Remember Medicine A and Medicine B are equally effective.

However, there are some differences between Medicine A and Medicine B.

Medicine A	Medicine B
**No inconvenience**<br><br>You take a tablet only once in your life.<br>You will be given the tablet on the day you come in to see your GP.	**Inconvenience**<br><br>One tablet should be taken once every day, at night with cold water.<br>You need to take this medicine for 10 years.<br>You need to regularly visit a GP to receive a prescription.<br>You need to visit a pharmacy to fill a prescription.
**Severe side effect - 0%**<br><br>No one taking Medicine A will have a severe side effect.	**Severe side effect - 0.7%**<br><br>Among every 1,000 people taking Medicine B, **7** will experience a severe side effect.
**Minor side effect - 0%**<br><br>No one taking Medicine A will have a minor side effect.	**Minor side effect - 13%**<br><br>Among every 1,000 people taking Medicine B, **130** will experience a minor side effect.

If you take Medicine A, you would live for **8 years** and then die.

If you take Medicine B, you would live for **10 years** and then die.

Would you prefer Medicine A or Medicine B, or are they the same?

Indicate your choice here. You can only choose one option:

◯ **Medicine A** Live for 8 years

◯ **Medicine B** Live for 10 years

◯ **Medicine A and Medicine B are the same.**

0% 100%

Repeated section

Remember Medicine A and Medicine B are equally effective.

However, there are some differences between Medicine A and Medicine B.

Medicine A	Medicine B
**No inconvenience**<br><br>You take a tablet only once in your life.<br>You will be given the tablet on the day you come in to see your GP.	**Inconvenience**<br><br>One tablet should be taken once every day, at night with cold water.<br>You need to take this medicine for 10 years.<br>You need to regularly visit a GP to receive a prescription.<br>You need to visit a pharmacy to fill a prescription.
**Severe side effect - 0%**<br><br>No one taking Medicine A will have a severe side effect.	**Severe side effect - 0.7%**<br><br>Among every 1,000 people taking Medicine B, **7** will experience a severe side effect.
**Minor side effect - 0%**<br><br>No one taking Medicine A will have a minor side effect.	**Minor side effect - 13%**<br><br>Among every 1,000 people taking Medicine B, **130** will experience a minor side effect.

If you take Medicine A, you would live for **8.25 years (8 years and 3 months)** and then die.

If you take Medicine B, you would live for **10 years** and then die.

Would you prefer Medicine A or Medicine B, or are they the same?

Indicate your choice here. You can only choose one option:

- ○ **Medicine A** — Live for 8 years and 3 months
- ○ **Medicine B** — Live for 10 years
- ○ **Medicine A and Medicine B are the same.**

0% 100%

Repeated section

Remember Medicine A and Medicine B are equally effective.

However, there are some differences between Medicine A and Medicine B.

Medicine A	Medicine B
**No inconvenience**<br><br>You take a tablet only once in your life.<br>You will be given the tablet on the day you come in to see your GP.	**Inconvenience**<br><br>One tablet should be taken once every day, at night with cold water.<br>You need to take this medicine for 10 years.<br>You need to regularly visit a GP to receive a prescription.<br>You need to visit a pharmacy to fill a prescription.
**Severe side effect - 0%**<br><br>No one taking Medicine A will have a severe side effect.	**Severe side effect - 0.7%**<br><br>Among every 1,000 people taking Medicine B, **7** will experience a severe side effect.
**Minor side effect - 0%**<br><br>No one taking Medicine A will have a minor side effect.	**Minor side effect - 13%**<br><br>Among every 1,000 people taking Medicine B, **130** will experience a minor side effect.

If you take Medicine A, you would live for **8.5 years** (8 years and 6 months) and then die.

If you take Medicine B, you would live for **10 years** and then die.

Would you prefer Medicine A or Medicine B, or are they the same?

Indicate your choice here. You can only choose one option:

◯ **Medicine A** — Live for 8.5 years

◯ **Medicine B** — Live for 10 years

◯ **Medicine A and Medicine B are the same.**

0% 100%

Repeated section

Remember Medicine A and Medicine B are equally effective.

However, there are some differences between Medicine A and Medicine B.

Medicine A	Medicine B
**No inconvenience**<br><br>You take a tablet only once in your life.<br>You will be given the tablet on the day you come in to see your GP.	**Inconvenience**<br><br>One tablet should be taken once every day, at night with cold water.<br>You need to take this medicine for 10 years.<br>You need to regularly visit a GP to receive a prescription.<br>You need to visit a pharmacy to fill a prescription.
**Severe side effect - 0%**<br><br>No one taking Medicine A will have a severe side effect.	**Severe side effect - 0.7%**<br><br>Among every 1,000 people taking Medicine B, **7** will experience a severe side effect.
**Minor side effect - 0%**<br><br>No one taking Medicine A will have a minor side effect.	**Minor side effect - 13%**<br><br>Among every 1,000 people taking Medicine B, **130** will experience a minor side effect.

If you take Medicine A, you would live for **8.75 years (8 years and 9 months)** and then die.

If you take Medicine B, you would live for **10 years** and then die.

Would you prefer Medicine A or Medicine B, or are they the same?

Indicate your choice here. You can only choose one option:

○ **Medicine A** Live for 8 years and 9 months

○ **Medicine B** Live for 10 years

○ **Medicine A and Medicine B are the same.**

0% 100%

Repeated section

Remember Medicine A and Medicine B are equally effective.

However, there are some differences between Medicine A and Medicine B.

Medicine A	Medicine B
**No inconvenience**<br><br>You take a tablet only once in your life.<br>You will be given the tablet on the day you come in to see your GP.	**Inconvenience**<br><br>One tablet should be taken once every day, at night with cold water.<br>You need to take this medicine for 10 years.<br>You need to regularly visit a GP to receive a prescription.<br>You need to visit a pharmacy to fill a prescription.
**Severe side effect - 0%**<br><br>No one taking Medicine A will have a severe side effect.	**Severe side effect - 0.7%**<br><br>Among every 1,000 people taking Medicine B, **7** will experience a severe side effect.
**Minor side effect - 0%**<br><br>No one taking Medicine A will have a minor side effect.	**Minor side effect - 13%**<br><br>Among every 1,000 people taking Medicine B, **130** will experience a minor side effect.

If you take Medicine A, you would live for **9 years** and then die.

If you take Medicine B, you would live for **10 years** and then die.

Would you prefer Medicine A or Medicine B, or are they the same?

Indicate your choice here. You can only choose one option:

○ **Medicine A** Live for 9 years

○ **Medicine B** Live for 10 years

○ **Medicine A and Medicine B are the same.**

0% 100%

Repeated section

Remember Medicine A and Medicine B are equally effective.

However, there are some differences between Medicine A and Medicine B.

Medicine A	Medicine B
**No inconvenience**<br><br>You take a tablet only once in your life.<br>You will be given the tablet on the day you come in to see your GP.	**Inconvenience**<br><br>One tablet should be taken once every day, at night with cold water.<br>You need to take this medicine for 10 years.<br>You need to regularly visit a GP to receive a prescription.<br>You need to visit a pharmacy to fill a prescription.
**Severe side effect - 0%**<br><br>No one taking Medicine A will have a severe side effect.	**Severe side effect - 0.7%**<br><br>Among every 1,000 people taking Medicine B, **7** will experience a severe side effect.
**Minor side effect - 0%**<br><br>No one taking Medicine A will have a minor side effect.	**Minor side effect - 13%**<br><br>Among every 1,000 people taking Medicine B, **130** will experience a minor side effect.

If you take Medicine A, you would live for **9 years and 1 month** and then die.

If you take Medicine B, you would live for **10 years** and then die.

Would you prefer Medicine A or Medicine B, or are they the same?

Indicate your choice here. You can only choose one option:

◯ **Medicine A** Live for 9 years 1 month

◯ **Medicine B** Live for 10 years

◯ **Medicine A and Medicine B are the same.**

0% 100%

Repeated section

Remember Medicine A and Medicine B are equally effective.

However, there are some differences between Medicine A and Medicine B.

Medicine A	Medicine B
**No inconvenience**<br><br>You take a tablet only once in your life.<br>You will be given the tablet on the day you come in to see your GP.	**Inconvenience**<br><br>One tablet should be taken once every day, at night with cold water.<br>You need to take this medicine for 10 years.<br>You need to regularly visit a GP to receive a prescription.<br>You need to visit a pharmacy to fill a prescription.
**Severe side effect - 0%**<br><br>No one taking Medicine A will have a severe side effect.	**Severe side effect - 0.7%**<br><br>Among every 1,000 people taking Medicine B, **7** will experience a severe side effect.
**Minor side effect - 0%**<br><br>No one taking Medicine A will have a minor side effect.	**Minor side effect - 13%**<br><br>Among every 1,000 people taking Medicine B, **130** will experience a minor side effect.

If you take Medicine A, you would live for **9.25 years (9 years and 3 months)** and then die.

If you take Medicine B, you would live for **10 years** and then die.

Would you prefer Medicine A or Medicine B, or are they the same?

Indicate your choice here. You can only choose one option:

◯ **Medicine A** — Live for 9 years and 3 months

◯ **Medicine B** — Live for 10 years

◯ **Medicine A and Medicine B are the same.**

0% 100%

Repeated section

Remember Medicine A and Medicine B are equally effective.

However, there are some differences between Medicine A and Medicine B.

Medicine A	Medicine B
**No inconvenience**<br><br>You take a tablet only once in your life.<br>You will be given the tablet on the day you come in to see your GP.	**Inconvenience**<br><br>One tablet should be taken once every day, at night with cold water.<br>You need to take this medicine for 10 years.<br>You need to regularly visit a GP to receive a prescription.<br>You need to visit a pharmacy to fill a prescription.
**Severe side effect - 0%**<br><br>No one taking Medicine A will have a severe side effect.	**Severe side effect - 0.7%**<br><br>Among every 1,000 people taking Medicine B, **7** will experience a severe side effect.
**Minor side effect - 0%**<br><br>No one taking Medicine A will have a minor side effect.	**Minor side effect - 13%**<br><br>Among every 1,000 people taking Medicine B, **130** will experience a minor side effect.

If you take Medicine A, you would live for **9 years and 4 months** and then die.

If you take Medicine B, you would live for **10 years** and then die.

Would you prefer Medicine A or Medicine B, or are they the same?

Indicate your choice here. You can only choose one option:

○ **Medicine A** Live for 9 years 4 months

○ **Medicine B** Live for 10 years

○ **Medicine A and Medicine B are the same.**

0% 100%

Repeated section

Remember Medicine A and Medicine B are equally effective.

However, there are some differences between Medicine A and Medicine B.

Medicine A	Medicine B
**No inconvenience**<br><br>You take a tablet only once in your life.<br>You will be given the tablet on the day you come in to see your GP.	**Inconvenience**<br><br>One tablet should be taken once every day, at night with cold water.<br>You need to take this medicine for 10 years.<br>You need to regularly visit a GP to receive a prescription.<br>You need to visit a pharmacy to fill a prescription.
**Severe side effect - 0%**<br><br>No one taking Medicine A will have a severe side effect.	**Severe side effect - 0.7%**<br><br>Among every 1,000 people taking Medicine B, **7** will experience a severe side effect.
**Minor side effect - 0%**<br><br>No one taking Medicine A will have a minor side effect.	**Minor side effect - 13%**<br><br>Among every 1,000 people taking Medicine B, **130** will experience a minor side effect.

If you take Medicine A, you would live for **9 years and 5 months** and then die.

If you take Medicine B, you would live for **10 years** and then die.

Would you prefer Medicine A or Medicine B, or are they the same?

Indicate your choice here. You can only choose one option:

○ **Medicine A** Live for 9 years 5 months

○ **Medicine B** Live for 10 years

○ **Medicine A and Medicine B are the same.**

0% 100%

Repeated section

Remember Medicine A and Medicine B are equally effective.

However, there are some differences between Medicine A and Medicine B.

Medicine A	Medicine B
**No inconvenience**<br><br>You take a tablet only once in your life.<br>You will be given the tablet on the day you come in to see your GP.	**Inconvenience**<br><br>One tablet should be taken once every day, at night with cold water.<br>You need to take this medicine for 10 years.<br>You need to regularly visit a GP to receive a prescription.<br>You need to visit a pharmacy to fill a prescription.
**Severe side effect - 0%**<br><br>No one taking Medicine A will have a severe side effect.	**Severe side effect - 0.7%**<br><br>Among every 1,000 people taking Medicine B, **7** will experience a severe side effect.
**Minor side effect - 0%**<br><br>No one taking Medicine A will have a minor side effect.	**Minor side effect - 13%**<br><br>Among every 1,000 people taking Medicine B, **130** will experience a minor side effect.

If you take Medicine A, you would live for **9.5 years** (9 years and 6 months) and then die.

If you take Medicine B, you would live for **10 years** and then die.

Would you prefer Medicine A or Medicine B, or are they the same?

Indicate your choice here. You can only choose one option:

○ **Medicine A** Live for 9.5 years

○ **Medicine B** Live for 10 years

○ **Medicine A and Medicine B are the same.**

0% 100%

Repeated section

Remember Medicine A and Medicine B are equally effective.

However, there are some differences between Medicine A and Medicine B.

Medicine A	Medicine B
**No inconvenience**<br><br>You take a tablet only once in your life.<br>You will be given the tablet on the day you come in to see your GP.	**Inconvenience**<br><br>One tablet should be taken once every day, at night with cold water.<br>You need to take this medicine for 10 years.<br>You need to regularly visit a GP to receive a prescription.<br>You need to visit a pharmacy to fill a prescription.
**Severe side effect - 0%**<br><br>No one taking Medicine A will have a severe side effect.	**Severe side effect - 0.7%**<br><br>Among every 1,000 people taking Medicine B, **7** will experience a severe side effect.
**Minor side effect - 0%**<br><br>No one taking Medicine A will have a minor side effect.	**Minor side effect - 13%**<br><br>Among every 1,000 people taking Medicine B, **130** will experience a minor side effect.

If you take Medicine A, you would live for **5 months less than 10 years** and then die.

If you take Medicine B, you would live for **10 years** and then die.

Would you prefer Medicine A or Medicine B, or are they the same?

Indicate your choice here. You can only choose one option:

◯ **Medicine A** Live for (10 years – 5 months)

◯ **Medicine B** Live for 10 years

◯ **Medicine A and Medicine B are the same.**

0% 100%

Repeated section

Remember Medicine A and Medicine B are equally effective.

However, there are some differences between Medicine A and Medicine B.

Medicine A	Medicine B
**No inconvenience**<br><br>You take a tablet only once in your life.<br>You will be given the tablet on the day you come in to see your GP.	**Inconvenience**<br><br>One tablet should be taken once every day, at night with cold water.<br>You need to take this medicine for 10 years.<br>You need to regularly visit a GP to receive a prescription.<br>You need to visit a pharmacy to fill a prescription.
**Severe side effect - 0%**<br><br>No one taking Medicine A will have a severe side effect.	**Severe side effect - 0.7%**<br><br>Among every 1,000 people taking Medicine B, **7** will experience a severe side effect.
**Minor side effect - 0%**<br><br>No one taking Medicine A will have a minor side effect.	**Minor side effect - 13%**<br><br>Among every 1,000 people taking Medicine B, **130** will experience a minor side effect.

If you take Medicine A, you would live for **4 months less than 10 years** and then die.

If you take Medicine B, you would live for **10 years** and then die.

Would you prefer Medicine A or Medicine B, or are they the same?

Indicate your choice here. You can only choose one option:

◯ **Medicine A** Live for (10 years – 4 months)

◯ **Medicine B** Live for 10 years

◯ **Medicine A and Medicine B are the same.**

0% 100%

Repeated section

Remember Medicine A and Medicine B are equally effective.

However, there are some differences between Medicine A and Medicine B.

Medicine A	Medicine B
**No inconvenience**<br><br>You take a tablet only once in your life.<br>You will be given the tablet on the day you come in to see your GP.	**Inconvenience**<br><br>One tablet should be taken once every day, at night with cold water.<br>You need to take this medicine for 10 years.<br>You need to regularly visit a GP to receive a prescription.<br>You need to visit a pharmacy to fill a prescription.
**Severe side effect - 0%**<br><br>No one taking Medicine A will have a severe side effect.	**Severe side effect - 0.7%**<br><br>Among every 1,000 people taking Medicine B, **7** will experience a severe side effect.
**Minor side effect - 0%**<br><br>No one taking Medicine A will have a minor side effect.	**Minor side effect - 13%**<br><br>Among every 1,000 people taking Medicine B, **130** will experience a minor side effect.

If you take Medicine A, you would live for **3 months less than 10 years** and then die.

If you take Medicine B, you would live for **10 years** and then die.

Would you prefer Medicine A or Medicine B, or are they the same?

Indicate your choice here. You can only choose one option:

◯ **Medicine A** Live for (10 years – 3 months)

◯ **Medicine B** Live for 10 years

◯ **Medicine A and Medicine B are the same.**

0% 100%

Repeated section

Remember Medicine A and Medicine B are equally effective.

However, there are some differences between Medicine A and Medicine B.

Medicine A	Medicine B
**No inconvenience**<br><br>You take a tablet only once in your life.<br>You will be given the tablet on the day you come in to see your GP.	**Inconvenience**<br><br>One tablet should be taken once every day, at night with cold water.<br>You need to take this medicine for 10 years.<br>You need to regularly visit a GP to receive a prescription.<br>You need to visit a pharmacy to fill a prescription.
**Severe side effect - 0%**<br><br>No one taking Medicine A will have a severe side effect.	**Severe side effect - 0.7%**<br><br>Among every 1,000 people taking Medicine B, **7** will experience a severe side effect.
**Minor side effect - 0%**<br><br>No one taking Medicine A will have a minor side effect.	**Minor side effect - 13%**<br><br>Among every 1,000 people taking Medicine B, **130** will experience a minor side effect.

If you take Medicine A, you would live for **2 months less than 10 years** and then die.

If you take Medicine B, you would live for **10 years** and then die.

Would you prefer Medicine A or Medicine B, or are they the same?

Indicate your choice here. You can only choose one option:

◯ **Medicine A** Live for (10 years – 2 months)

◯ **Medicine B** Live for 10 years

◯ **Medicine A and Medicine B are the same.**

0% 100%

Repeated section

Remember Medicine A and Medicine B are equally effective.

However, there are some differences between Medicine A and Medicine B.

Medicine A	Medicine B
**No inconvenience**<br><br>You take a tablet only once in your life.<br>You will be given the tablet on the day you come in to see your GP.	**Inconvenience**<br><br>One tablet should be taken once every day, at night with cold water.<br>You need to take this medicine for 10 years.<br>You need to regularly visit a GP to receive a prescription.<br>You need to visit a pharmacy to fill a prescription.
**Severe side effect - 0%**<br><br>No one taking Medicine A will have a severe side effect.	**Severe side effect - 0.7%**<br><br>Among every 1,000 people taking Medicine B, **7** will experience a severe side effect.
**Minor side effect - 0%**<br><br>No one taking Medicine A will have a minor side effect.	**Minor side effect - 13%**<br><br>Among every 1,000 people taking Medicine B, **130** will experience a minor side effect.

If you take Medicine A, you would live for **1 month less than 10 years** and then die.

If you take Medicine B, you would live for **10 years** and then die.

Would you prefer Medicine A or Medicine B, or are they the same?

Indicate your choice here. You can only choose one option:

◯ **Medicine A** Live for (10 years – 1 month)

◯ **Medicine B** Live for 10 years

◯ **Medicine A and Medicine B are the same.**

0% 100%

Repeated section

Remember Medicine A and Medicine B are equally effective.

However, there are some differences between Medicine A and Medicine B.

Medicine A	Medicine B
**No inconvenience**<br><br>You take a tablet only once in your life.<br>You will be given the tablet on the day you come in to see your GP.	**Inconvenience**<br><br>One tablet should be taken once every day, at night with cold water.<br>You need to take this medicine for 10 years.<br>You need to regularly visit a GP to receive a prescription.<br>You need to visit a pharmacy to fill a prescription.
**Severe side effect - 0%**<br><br>No one taking Medicine A will have a severe side effect.	**Severe side effect - 0.7%**<br><br>Among every 1,000 people taking Medicine B, **7** will experience a severe side effect.
**Minor side effect - 0%**<br><br>No one taking Medicine A will have a minor side effect.	**Minor side effect - 13%**<br><br>Among every 1,000 people taking Medicine B, **130** will experience a minor side effect.

If you take Medicine A, you would live for **3 weeks less than 10 years** and then die.

If you take Medicine B, you would live for **10 years** and then die.

Would you prefer Medicine A or Medicine B, or are they the same?

Indicate your choice here. You can only choose one option:

○ **Medicine A** Live for (10 years – 3 weeks)

○ **Medicine B** Live for 10 years

○ **Medicine A and Medicine B are the same.**

0% 100%

Repeated section

Remember Medicine A and Medicine B are equally effective.

However, there are some differences between Medicine A and Medicine B.

Medicine A	Medicine B
**No inconvenience**<br><br>You take a tablet only once in your life.<br>You will be given the tablet on the day you come in to see your GP.	**Inconvenience**<br><br>One tablet should be taken once every day, at night with cold water.<br>You need to take this medicine for 10 years.<br>You need to regularly visit a GP to receive a prescription.<br>You need to visit a pharmacy to fill a prescription.
**Severe side effect - 0%**<br><br>No one taking Medicine A will have a severe side effect.	**Severe side effect - 0.7%**<br><br>Among every 1,000 people taking Medicine B, **7** will experience a severe side effect.
**Minor side effect - 0%**<br><br>No one taking Medicine A will have a minor side effect.	**Minor side effect - 13%**<br><br>Among every 1,000 people taking Medicine B, **130** will experience a minor side effect.

If you take Medicine A, you would live for **2 weeks less than 10 years** and then die.

If you take Medicine B, you would live for **10 years** and then die.

Would you prefer Medicine A or Medicine B, or are they the same?

Indicate your choice here. You can only choose one option:

○ **Medicine A** Live for (10 years – 2 weeks)

○ **Medicine B** Live for 10 years

○ **Medicine A and Medicine B are the same.**

0% 100%

Repeated section

Remember Medicine A and Medicine B are equally effective.

However, there are some differences between Medicine A and Medicine B.

Medicine A	Medicine B
**No inconvenience**<br><br>You take a tablet only once in your life.<br>You will be given the tablet on the day you come in to see your GP.	**Inconvenience**<br><br>One tablet should be taken once every day, at night with cold water.<br>You need to take this medicine for 10 years.<br>You need to regularly visit a GP to receive a prescription.<br>You need to visit a pharmacy to fill a prescription.
**Severe side effect - 0%**<br><br>No one taking Medicine A will have a severe side effect.	**Severe side effect - 0.7%**<br><br>Among every 1,000 people taking Medicine B, **7** will experience a severe side effect.
**Minor side effect - 0%**<br><br>No one taking Medicine A will have a minor side effect.	**Minor side effect - 13%**<br><br>Among every 1,000 people taking Medicine B, **130** will experience a minor side effect.

If you take Medicine A, you would live for **10 days less than 10 years** and then die.

If you take Medicine B, you would live for **10 years** and then die.

Would you prefer Medicine A or Medicine B, or are they the same?

Indicate your choice here. You can only choose one option:

◯ **Medicine A** Live for (10 years – 10 days)

◯ **Medicine B** Live for 10 years

◯ **Medicine A and Medicine B are the same.**

0% 100%

Repeated section

Remember Medicine A and Medicine B are equally effective.

However, there are some differences between Medicine A and Medicine B.

Medicine A	Medicine B
**No inconvenience**<br><br>You take a tablet only once in your life.<br>You will be given the tablet on the day you come in to see your GP.	**Inconvenience**<br><br>One tablet should be taken once every day, at night with cold water.<br>You need to take this medicine for 10 years.<br>You need to regularly visit a GP to receive a prescription.<br>You need to visit a pharmacy to fill a prescription.
**Severe side effect - 0%**<br><br>No one taking Medicine A will have a severe side effect.	**Severe side effect - 0.7%**<br><br>Among every 1,000 people taking Medicine B, **7** will experience a severe side effect.
**Minor side effect - 0%**<br><br>No one taking Medicine A will have a minor side effect.	**Minor side effect - 13%**<br><br>Among every 1,000 people taking Medicine B, **130** will experience a minor side effect.

If you take Medicine A, you would live for **1 week less than 10 years** and then die.

If you take Medicine B, you would live for **10 years** and then die.

Would you prefer Medicine A or Medicine B, or are they the same?

Indicate your choice here. You can only choose one option:

○ **Medicine A** Live for (10 years – 1 week)

○ **Medicine B** Live for 10 years

○ **Medicine A and Medicine B are the same.**

0% 100%

Repeated section

Remember Medicine A and Medicine B are equally effective.

However, there are some differences between Medicine A and Medicine B.

Medicine A	Medicine B
**No inconvenience**<br><br>You take a tablet only once in your life.<br>You will be given the tablet on the day you come in to see your GP.	**Inconvenience**<br><br>One tablet should be taken once every day, at night with cold water.<br>You need to take this medicine for 10 years.<br>You need to regularly visit a GP to receive a prescription.<br>You need to visit a pharmacy to fill a prescription.
**Severe side effect - 0%**<br><br>No one taking Medicine A will have a severe side effect.	**Severe side effect - 0.7%**<br><br>Among every 1,000 people taking Medicine B, **7** will experience a severe side effect.
**Minor side effect - 0%**<br><br>No one taking Medicine A will have a minor side effect.	**Minor side effect - 13%**<br><br>Among every 1,000 people taking Medicine B, **130** will experience a minor side effect.

If you take Medicine A, you would live for **5 days less than 10 years** and then die.

If you take Medicine B, you would live for **10 years** and then die.

Would you prefer Medicine A or Medicine B, or are they the same?

Indicate your choice here. You can only choose one option:

○ **Medicine A** Live for (10 years – 5 days)

○ **Medicine B** Live for 10 years

○ **Medicine A and Medicine B are the same.**

0% 100%

Repeated section

Remember Medicine A and Medicine B are equally effective.

However, there are some differences between Medicine A and Medicine B.

Medicine A	Medicine B
**No inconvenience**<br><br>You take a tablet only once in your life.<br>You will be given the tablet on the day you come in to see your GP.	**Inconvenience**<br><br>One tablet should be taken once every day, at night with cold water.<br>You need to take this medicine for 10 years.<br>You need to regularly visit a GP to receive a prescription.<br>You need to visit a pharmacy to fill a prescription.
**Severe side effect - 0%**<br><br>No one taking Medicine A will have a severe side effect.	**Severe side effect - 0.7%**<br><br>Among every 1,000 people taking Medicine B, **7** will experience a severe side effect.
**Minor side effect - 0%**<br><br>No one taking Medicine A will have a minor side effect.	**Minor side effect - 13%**<br><br>Among every 1,000 people taking Medicine B, **130** will experience a minor side effect.

If you take Medicine A, you would live for **3 days less than 10 years** and then die.

If you take Medicine B, you would live for **10 years** and then die.

Would you prefer Medicine A or Medicine B, or are they the same?

Indicate your choice here. You can only choose one option:

◯ **Medicine A** — Live for (10 years – 3 days)

◯ **Medicine B** — Live for 10 years

◯ **Medicine A and Medicine B are the same.**

0% 100%

Repeated section

Remember Medicine A and Medicine B are equally effective.

However, there are some differences between Medicine A and Medicine B.

Medicine A	Medicine B
**No inconvenience**<br><br>You take a tablet only once in your life.<br>You will be given the tablet on the day you come in to see your GP.	**Inconvenience**<br><br>One tablet should be taken once every day, at night with cold water.<br>You need to take this medicine for 10 years.<br>You need to regularly visit a GP to receive a prescription.<br>You need to visit a pharmacy to fill a prescription.
**Severe side effect - 0%**<br><br>No one taking Medicine A will have a severe side effect.	**Severe side effect - 0.7%**<br><br>Among every 1,000 people taking Medicine B, **7** will experience a severe side effect.
**Minor side effect - 0%**<br><br>No one taking Medicine A will have a minor side effect.	**Minor side effect - 13%**<br><br>Among every 1,000 people taking Medicine B, **130** will experience a minor side effect.

If you take Medicine A, you would live for **1 day less than 10 years** and then die.

If you take Medicine B, you would live for **10 years** and then die.

Would you prefer Medicine A or Medicine B, or are they the same?

Indicate your choice here. You can only choose one option:

○ **Medicine A** Live for (10 years – 1 day)

○ **Medicine B** Live for 10 years

○ **Medicine A and Medicine B are the same.**

0% 100%

Question 5a.

You prefer living for 5 years on Medicine A, to living for 10 years on Medicine B.

This means **living fewer than 5 years on Medicine A** can be the same as living for 10 years on Medicine B.

How many years on Medicine A do you think would be the same as living for 10 years on Medicine B?

(Please give a number in years)

0% 100%

peer review only

Thank you for completing this section and indicating your choices.

Was there anything in this section which you think is unclear or that you didn't understand?

(Please provide details in the comment box below)

0% 100%

For peer review only

Part 3. Questions about you

We understand different people feel differently about different treatments.

In this part of the survey, we ask questions about you to understand if people's age, gender or experiences explain the choices made in Part 2.

0% 100%

For peer review only

We would like to learn about your experience.

Are you currently taking, or have you ever taken any of these medicines?

Atorvastatin
Crestor®
Dorisin
Fluvastatin
Lescol®
Lipitor®
Nandovar
Pravastatin
Simvastatin
Simvador®
Zocor®

- ◯ Yes
- ◯ No

0% 100%

view only

What do you think would be the **benefits** from taking this medicine?

What do you think would be the **harms** from taking this medicine?

Have you experienced any side effects from taking this medicine?

- ◯ Yes
- ◯ No

Do you find it inconvenient to take your statin? Please specify if you think it is inconvenient.

What is your opinion about taking this medicine?

- ◯ I don't mind taking them because I have to take them for a reason.
- ◯ I do not like taking them because they remind me that I am not perfectly healthy.
- ◯ I have no opinion.
- ◯ Other (please specify)

0% 100%

Do you take other medicines prescribed by your doctor?

- ◯ Yes
- ◯ No

In total, how many medicines do you take on a regular basis?
(Please do not include pills you take only once in a while)

- ◯ None
- ◯ One
- ◯ Two to five
- ◯ Six to Ten
- ◯ More than ten

How many different times a day do you regularly take pills?

- ◯ None
- ◯ Once per day
- ◯ 2 times a day
- ◯ 3 times a day
- ◯ More than 3 times a day

Do you find it inconvenient to take your medicine(s)? Please specify if you think it is inconvenient.

What is your opinion about taking this medicine?

- ◯ I don't mind taking them because I have to take them for a reason.
- ◯ I do not like taking them because they remind me that I am not perfectly healthy.
- ◯ I have no opinion.
- ◯ Other (please specify)

0% 100%

For peer review only

We would like to ask questions about you to better understand your choices.

What is your gender?

- ◯ Male
- ◯ Female

Which age category do you fall into?

- ◯ 34 years or younger
- ◯ 35-44 years
- ◯ 45-54 years
- ◯ 55-64 years
- ◯ 65-74 years
- ◯ 75-84 years
- ◯ 85 years or over

Which best reflects your ethnic group?

- ◯ White British/Irish
- ◯ White other
- ◯ Mixed/Multiple ethnic origins
- ◯ Black/African/Caribbean/Black British
- ◯ Asian/Asian British
- ◯ Chinese
- ◯ Other (please specify) [ ]

0% 100%

What is your occupational status?

- ◯ Employed full-time
- ◯ Employed part-time
- ◯ Self-employed
- ◯ Unemployed
- ◯ Retired
- ◯ Looking after a home/family
- ◯ Student
- ◯ Freelance or temping
- ◯ Long-term sickness

Could you tell us what your occupation is?
(Previous occupation if retired.)

What is the highest level of education you have obtained?
(for example, GCSEs, Degrees, etc.)

What is your religion?

- ◯ No religion
- ◯ Christian
- ◯ Buddhist
- ◯ Jewish
- ◯ Hindu
- ◯ Muslim
- ◯ Sikh
- ◯ Other ______
- ◯ Prefer not to say

0% 100%

For peer review only

How do you feel about numbers?

This section will ask you some questions to understand how familiar you are with numbers and probabilities.

If you are unsure of the answer, you can leave the response blank.

0% 100%

For peer review only

Question 1

Imagine I flip a coin 1,000 times.

What is your best guess about how many times the coin would fall heads up in 1,000 flips?

- Answer:
- Prefer not to answer
- Don't know.

Question 2

If there was a lottery where the chance of winning £10 is 1%, if 1,000 people bought a ticket, how many would you expect to win?

- Answer:
- Prefer not to answer
- Don't know.

Question 3

In another lottery, the chance of winning a car is 1 in 1,000.

What percentage of tickets in the lottery will win a car?

- Answer: %
- Prefer not to answer
- Don't know

0% 100%

How confident are you that you would answer the questions the same if faced with the situations in real-life?

	Very confident	Confident	Not sure	Not confident	Not confident at all	
Confident	◯	◯	◯	◯	◯	Not confident at all

How easy or difficult did you find making choices between alternatives?

	Very easy	Easy	Average	Somewhat difficult	Very difficult	
Easy	◯	◯	◯	◯	◯	Difficult

How easy or difficult did you find the survey to understand?

	Very easy	Easy	Average	Somewhat difficult	Very difficult	
Easy	◯	◯	◯	◯	◯	Difficult

0% 100%

eview only

Was there anything in the survey that was particularly unclear?

(Please write in the box below)

Is there anything else about the survey we could change to make it easier to understand (for example, phrasing and explanation)?

(Optional) If you have any further comments or suggestions, please write them here.

0% 100%

For peer review only - http://bmjopen.bmj.com/site/about/guidelines.xhtml

Thank you for completing this survey

If you have any questions or concerns please contact ResearchNow Support.

If you would like more information about heart disease please visit the NHS Choices website here.

MANCHESTER 1824
The University of Manchester

0% 100%

peer review only

Note:

When respondents take the survey in regular mode this page will not be displayed. Respondents will be redirected to the link below:

https://dkr1.ssisurveys.com/projects/end?rst=1&psid= [Script] &basic=99423

0% 100%

For peer review only

Note:

When respondents take the survey in regular mode this page will not be displayed. Respondents will be redirected to the link below:

https://dkr1.ssisurveys.com/projects/end?rst=2&psid= [Script]

0% 100%

For peer review only

Note:

When respondents take the survey in regular mode this page will not be displayed. Respondents will be redirected to the link below:

https://dkr1.ssisurveys.com/projects/end?rst=3&psid= [Script]

0% 100%

For peer review only

Quantifying the impact of taking medicines for long-term conditions

A survey to understand your views about taking medicines long term for the prevention of osteoporotic fractures

In this survey, we will be asking you about a drug treatment which may be offered to people at an increased risk of having a fracture from osteoporosis.

There are no right or wrong answers, we are just interested in hearing about your views.

For peer review only

Quantifying the impact of taking medicines for long-term conditions

Taking medicines long term for the prevention of osteoporotic fracture

Participant Consent Form

You are being invited to take part in a research study that aims to understand your views about taking medicines long-term. Before you decide, it is important for you to understand why the research is being done and what it will involve.

Please click on the sections below for information on the research.

What is the aim of the research?

Why have I been chosen?

What happens to the data collected?

How is confidentiality maintained?

How long will it take me?

Who will conduct the research?

What would I be asked to do if I took part?

Will the outcomes of the research be published?

What happens if I do not want to take part or if I change my mind?

Who has reviewed the research project?

I understand that my participation in the study is voluntary and that I am free to withdraw at any time without giving a reason by exiting the survey.

I confirm that I have read the information above.

◯ Yes, I would like to take part in the survey.

◯ No, I do not want to participate (exit survey).

0% 100%

What is your gender?

- ◯ Female
- ◯ Male

0% 100%

For peer review only

[QUOTA: QuotaCheck]

Note:

This page only contains quota information and only appears in preview mode.

0% 100%

For peer review only

By selecting one box in each group below, please indicate which statements best describe your own health state today.

Mobility

- ◯ I have no problems in walking about
- ◯ I have some problems in walking about
- ◯ I am confined to bed

Self-Care

- ◯ I have no problems with self-care
- ◯ I have some problems washing or dressing myself
- ◯ I am unable to wash or dress myself

Usual Activities (e.g. work, study, housework, family or leisure activities)

- ◯ I have no problems with performing my usual activities
- ◯ I have some problems with performing my usual activities
- ◯ I am unable to perform my usual activities

Pain/Discomfort

- ◯ I have no pain or discomfort
- ◯ I have moderate pain or discomfort
- ◯ I have extreme pain or discomfort

Anxiety/Depression

- ◯ I am not anxious or depressed
- ◯ I am moderately anxious or depressed
- ◯ I am extremely anxious or depressed

0% 100%

Part 1. Background

This section of the survey explains: osteoporosis (thinning of the bones), how medicine treatments may be used for people at risk of osteoporotic fracture, and what the treatment involves.

We will follow a story of Alex.

Click next to continue.

0% 100%

For peer review only

Part 1. Background

Alex has been to see a doctor who told her that she has weak bones.

The doctor has said Alex needs to start a medicine to prevent broken bones (fractures) because of her weak bones.

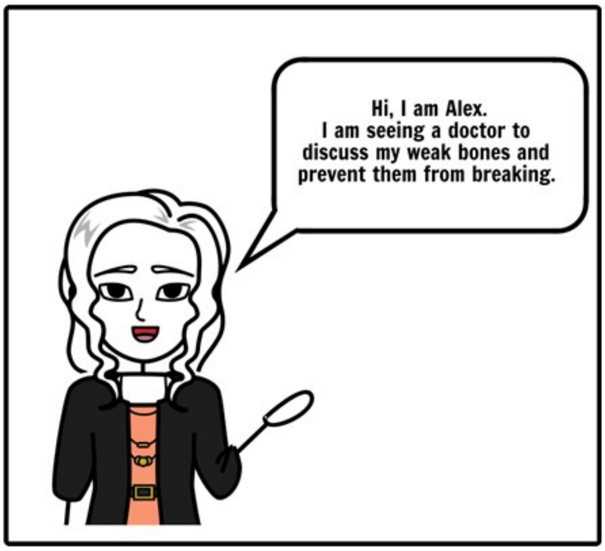

We will take you through Alex's story.

0% 100%

Part 1. Background

What is osteoporosis?

Alex hears about osteoporosis from her GP.

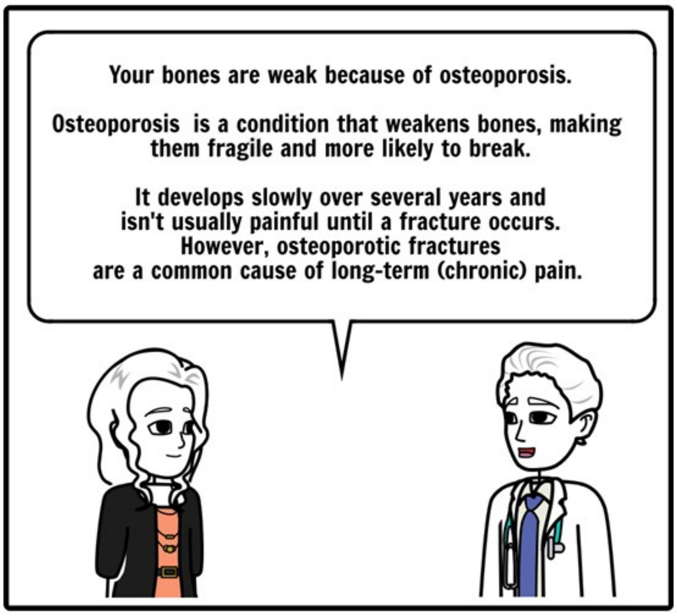

0% 100%

nly

Part 1. Background

Impact of osteoporosis

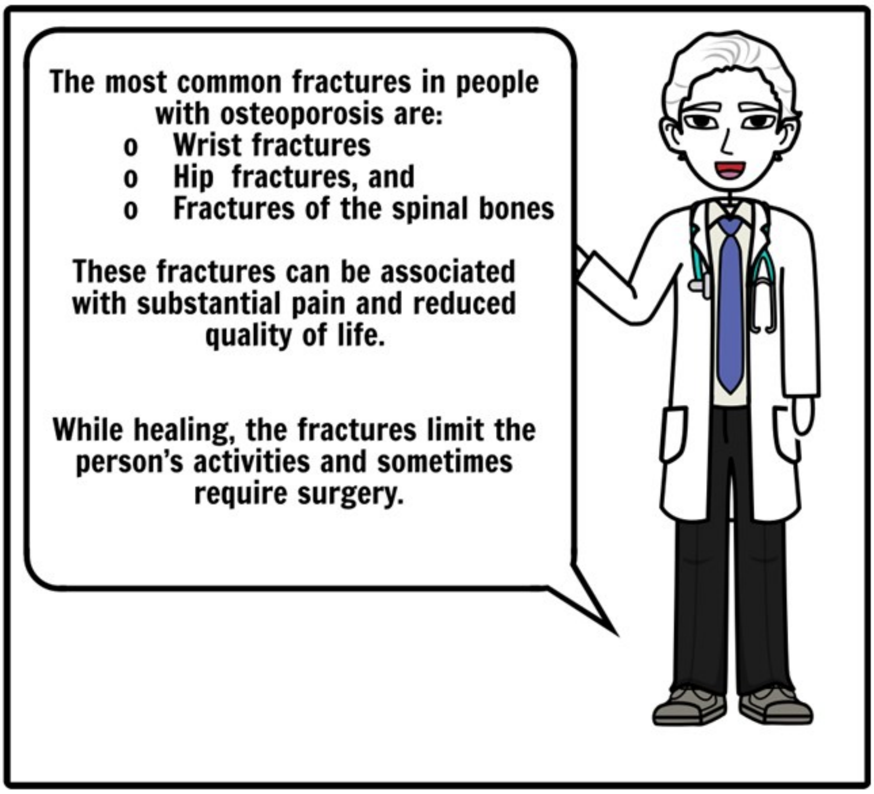

0% 100%

Part 1. Background

Risk of osteoporotic fractures

Her GP explains that Alex has some risk of fracture as her family has a history of hip fracture.

Also, Alex has a low body mass index (BMI). BMI is a measure of body fat.

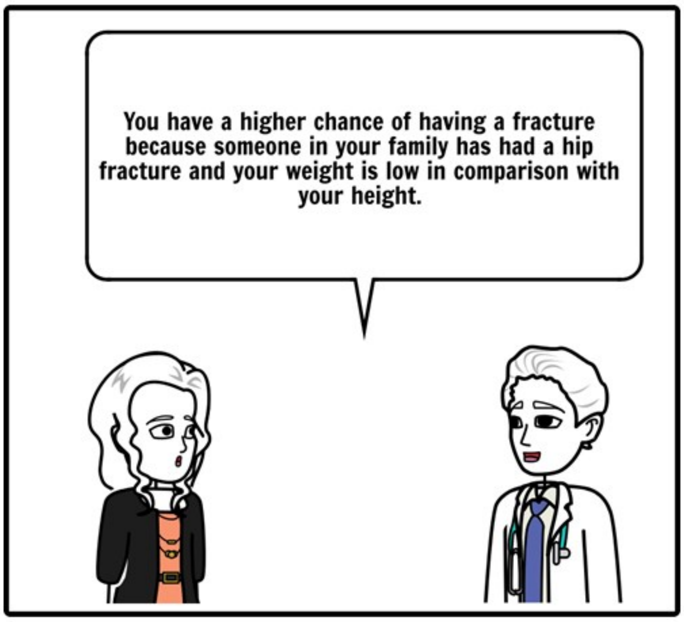

0% 100%

Part 1. Background

What is risk?

- **Risk** is: a term used to explain the chance that something bad might happen.

- **10% risk** means:

  Out of every **1,000** people, with osteoporosis,

  100 people would have a fracture, and **900** people would not.

0% 100%

nly

Part 1. Background

What is a 10% risk?

- This diagram shows a 10% risk of having a fracture because of their osteoporosis.
- The people shaded blue are the ones who have a fracture.

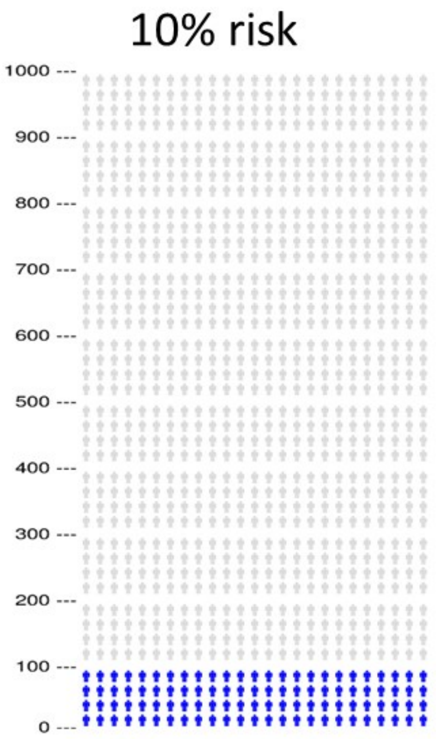

0% 100%

Part 1. Background

Prevention of fracture from osteoporosis

- Alex's doctor can estimate how likely it is that she will experience osteoporotic fracture over the next 5 years.
- The estimate is based on things such as Alex's age and sex, ethnicity, family fracture history, and body mass index.
- The doctor may prescribe certain medicines such as bisphosphonates to help reduce the chance of Alex having a fracture.
- The medicine will prevent some of the people who take it from having a fracture because of osteoporosis.
- Fractures may still happen in some of the people who take the medicine.

0% 100%

Part 1. Background

Prevention of fractures from osteoporosis

The doctor prescribes Alex a medicine to reduce the risk of her having a fracture in the future because of her osteoporosis.

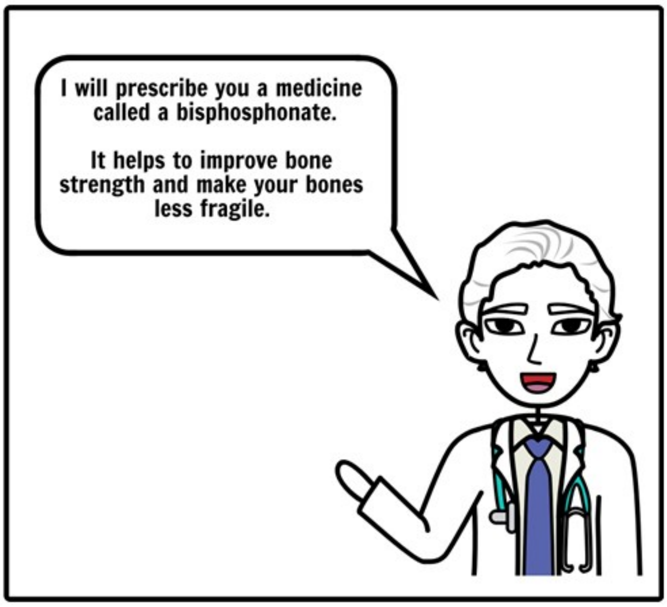

0% 100%

Part 1. Background

Collecting the medicine from the pharmacy

The pharmacist explains to Alex how to take the medicine.

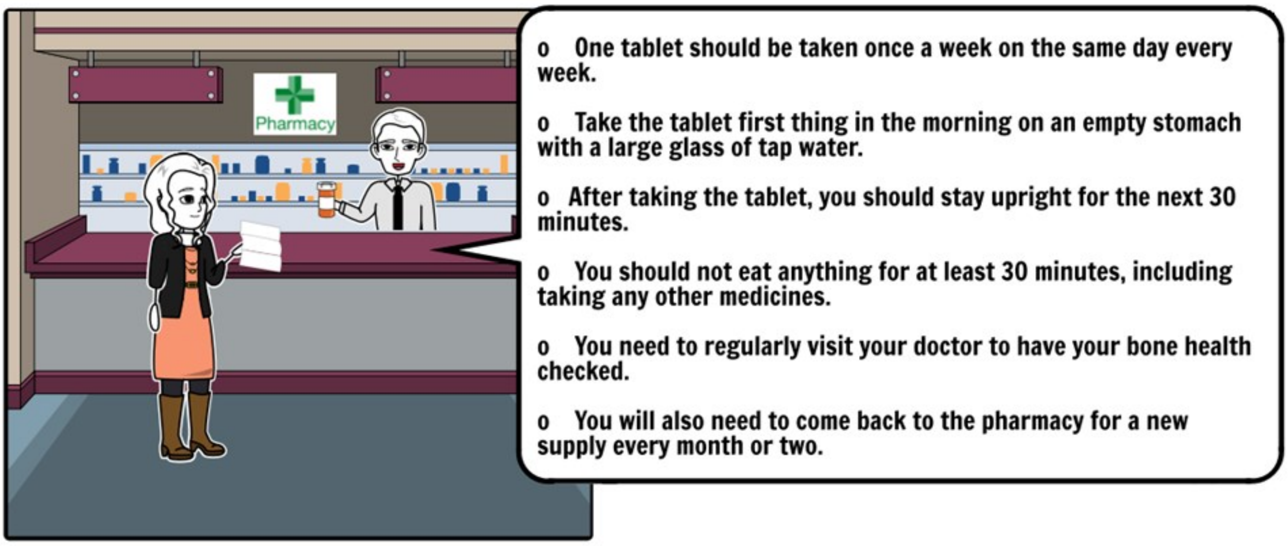

0% 100%

Part 1. Background

Taking the medicine for prevention

Alex goes home with her new tablets.

She then considers whether she wants to take them as the doctor and the pharmacist have advised.

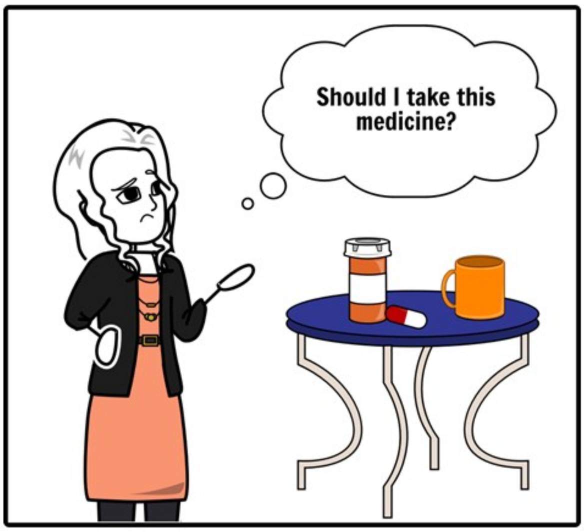

0% 100%

nly

Part 1. Background

Alex may consider *three key things,*
when deciding whether to take the medicine.

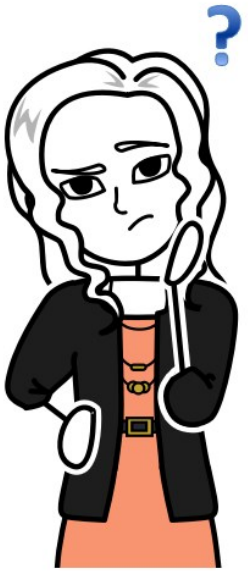

Effectiveness of the medicine

- How good the medicine is in terms of preventing a fracture from osteoporosis

Side effects of the medicine

- Whether there is any potential harm from the medicine

Inconvenience of the medicine

- Whether taking the medicine would fit in her lifestyle or cause her inconvenience

0% 100%

only

Part 1. Background

How effective is the medicine?

- Some people, but not everyone, who take the medicine will avoid a serious event, such as hip fracture, as a result of taking the tablet.
- The effectiveness of the medicine can be described in terms of a reduction in this risk.

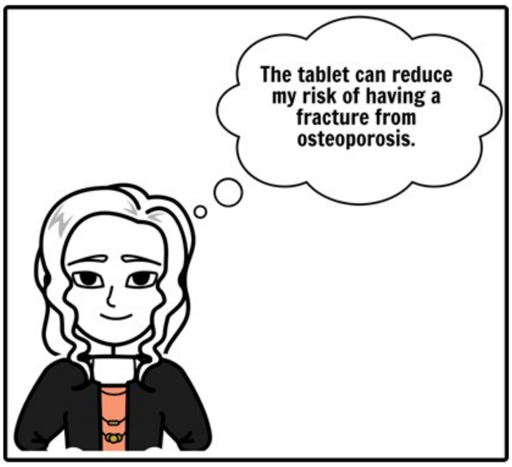

0% 100%

Part 1. Background

What does ‘reduction in risk’ mean?

- A ‘reduction in risk from 10% to 6%’ means:

The medicine will reduce your risk of experiencing a fracture, such as hip fracture, within the next 5 years from 10% to 6%.

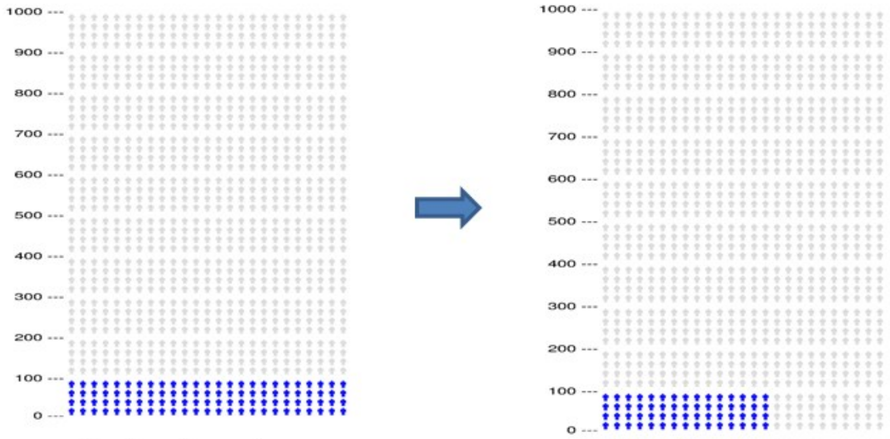

No treatment: 10% risk

Taking the medicine: 6% risk

0% 100%

Part 1. Background

Does the medicine have side effects?

The potential for side effects from the medicine can be described in terms of the **risk of a side effect**.

Alex hears about minor but more common side effects & severe but rarer side effects from her GP.

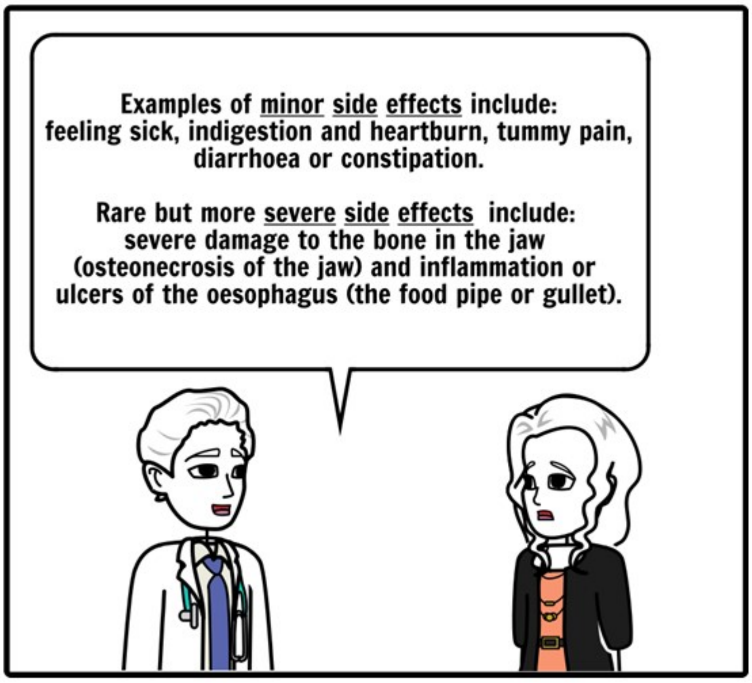

0% 100%

Part 1. Background

Risk of a minor side effect from the medicine

- A 5% risk of a minor side effect means:

Among every **1,000** people taking the medicine,

50 will experience a minor side effect such as either: feeling sick, indigestion, diarrhoea or constipation.

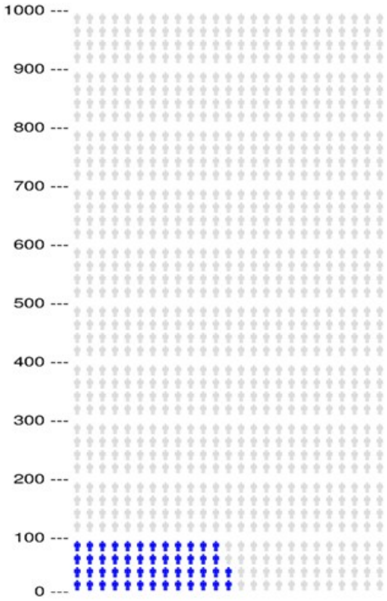

5% risk of a side effect

0% 100%

Part 1. Background

Risk of a severe side effect from the medicine

- A 0.3% risk of severe side effect means:

Among every **1,000** people taking the medicine,

3 will experience a severe side effect such as severe loss or destruction of the bone in the jaw.

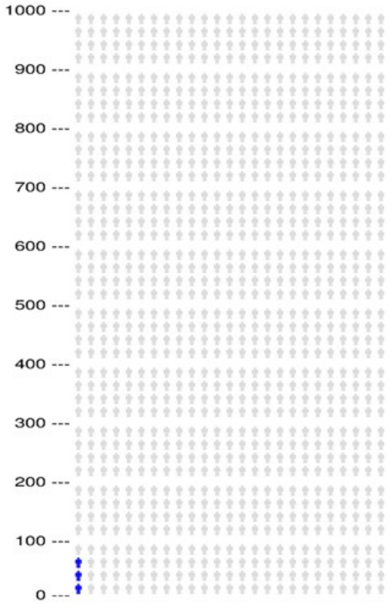

0.3% risk of a side effect

0% 100%

Part 1. Background

How inconvenient is it when taking the medicine?

Alex also thinks about what it means to have to take this medicine for the next 5 years.

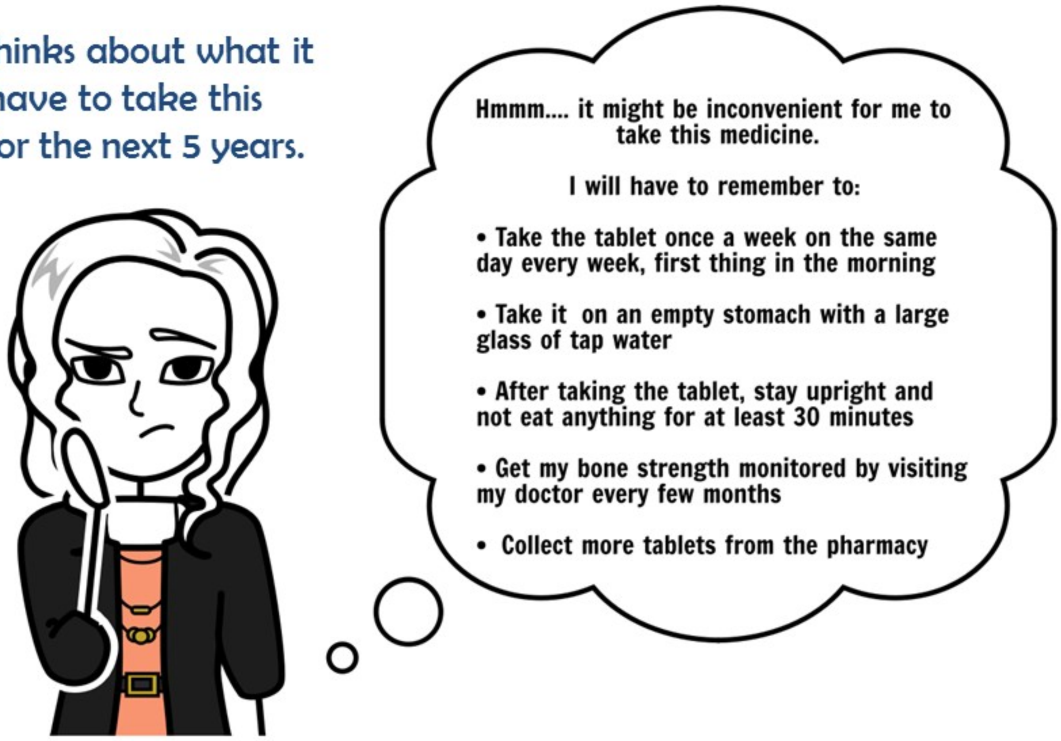

0% 100%

Part 1. Background

In the questions that follow in this survey,
we will ask you about how you think about taking a medicine to prevent osteoporotic fracture.

0% 100%

nly

In general, how would you feel about taking a medicine for the first time?

- ◯ **Happy**
  I trust in my doctor and I am willing to try new things
- ◯ **Unsure**
  I'd like to think about it really carefully
- ◯ **Unhappy**
  I don't like the idea of trying a new medicine even if the doctor says it will help

0% 100%

peer review only

Part 2: Your views on the inconvenience of taking a medicine to prevent fractures

Now, we will ask you about how you think about the inconvenience of taking a medicine to prevent osteoporotic fractures.

There are four questions.

Each question may look similar but differs in terms of the detail for each of the three features.

0% 100%

For peer review only

Warm up question

You have been told that you need to take a medicine to reduce the risk of fracture.

You can choose between two different medicines. We have called these Medicine A and Medicine B.

Medicine A is a pill that you only need to take once.

Medicine B is a pill that you need to take every week for 5 years.

Medicine A and Medicine B are equally effective in reducing your risk of having a fracture.
Medicine A and Medicine B will reduce your risk of having an osteoporotic fracture within the next 5 years from **10%** to **6%**.

In other words, in a crowd of **1,000** people in the UK, **100** are likely to have a fracture if they do not take the medicine. This is reduced to **60** people out of 1,000 who do take the medicine.
This information is also illustrated in this picture.

Risk of fracture

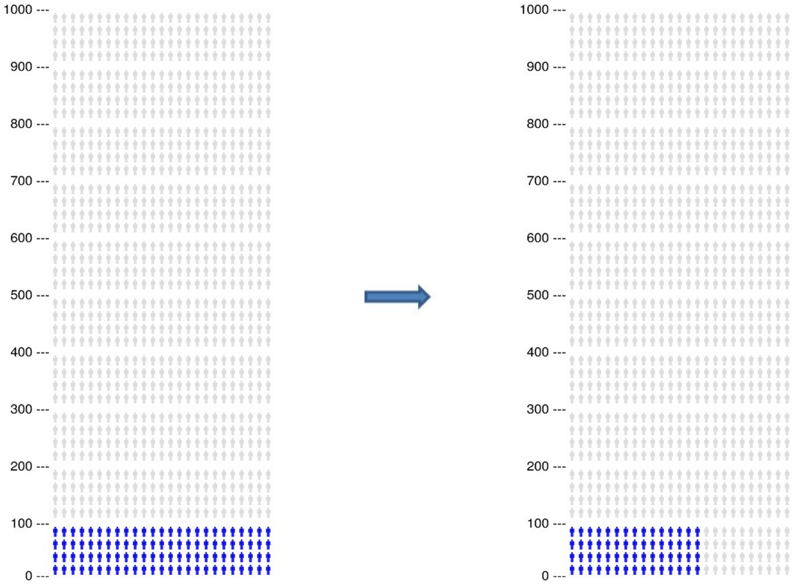

10%
No Treatment

6%
Taking the Medicine

(The figures shaded blue represent people who will have a fracture in the next 5 years)

In this scenario, you will not have any side effects from Medicine A or Medicine B.

Risk of a side effect

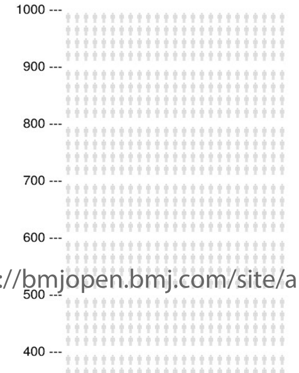

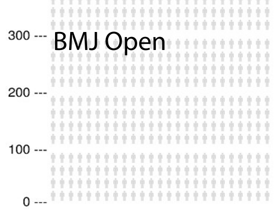

0%
No one will have a side effect.

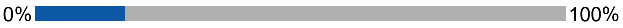

or peer review only

Remember Medicine A and Medicine B are equally effective and have no risks of a side effect.

The only difference between Medicine A and Medicine B is how you take them.

Medicine A	Medicine B
You take a tablet only once in your life.<br>You will be given the tablet on the day you come in to see your GP.<br>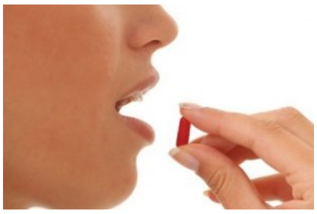	One tablet should be taken once a week on the same day every week.<br>You need to take this medicine for 5 years.<br><br>You need to take the tablet first thing in the morning on an empty stomach with a large glass of tap water.<br>After taking the tablet, you should stay upright for the next 30 minutes.<br>You should not eat anything for at least 30 minutes, including taking any other medicines.<br><br>You need to regularly visit your doctor to have your bone health checked.<br>You need to regularly visit a pharmacy for a new supply.<br>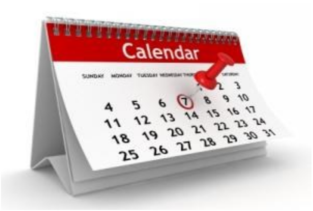 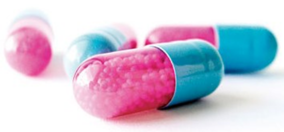

If you take Medicine A, you would live for **1 year** and then die.

If you take Medicine B, you would live for **5 years** and then die.

Would you prefer Medicine A or Medicine B, or are they the same?

Indicate your choice here. You can only choose one option:

○ **Medicine A**

○ **Medicine B**

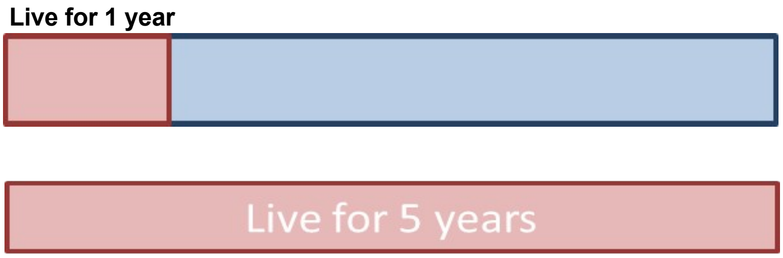

○ **Medicine A and Medicine B are the same.**

0% 100%

Now we are ready to start the survey questions.

0% 100%

For peer review only

Part 2

Question 1

You have been told that you need to take a medicine to reduce the risk of fracture.

You can choose between two different medicines. We have called these Medicine A and Medicine B.

Medicine A is a pill that you only need to take once.

Medicine B is a pill that you need to take every week for 5 years.

Medicine A and Medicine B are equally effective in reducing your risk of having a fracture.
Medicine A and Medicine B will reduce your risk of experiencing a fracture, such as hip fracture, within the next 5 years from **10%** to **6%**.

In other words, in a crowd of **1,000** people in the UK, **100** are likely to have a fracture if they do not take the medicine. This is reduced to **60** people out of 100 who do take the medicine.
This information is also illustrated in this picture.

Risk of fracture

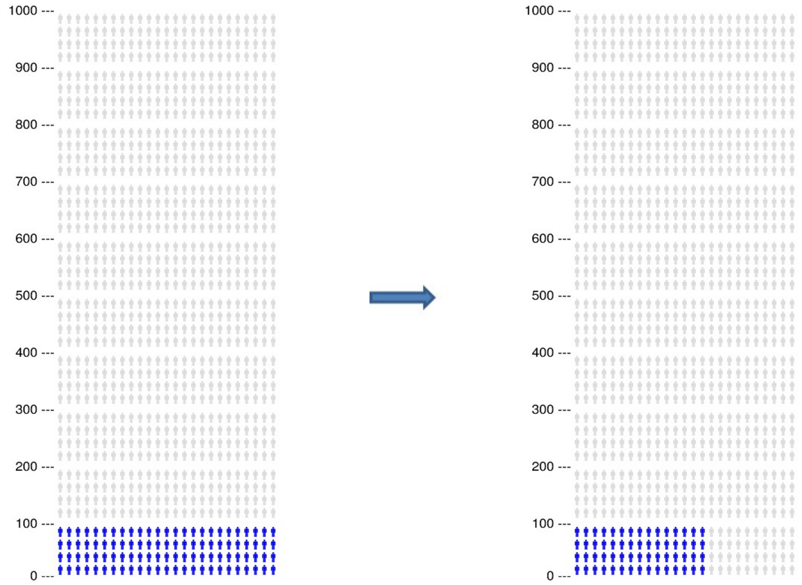

10%
No Treatment

6%
Taking the Medicine

(The figures shaded blue represent people who will have a fracture, such as hip fracture, within the next 5 years)

In this scenario, you will not have any side effects from Medicine A or Medicine B.

Risk of a side effect

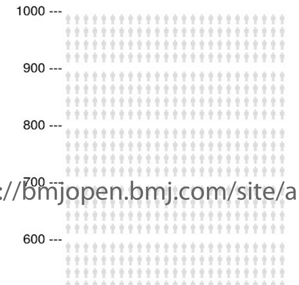

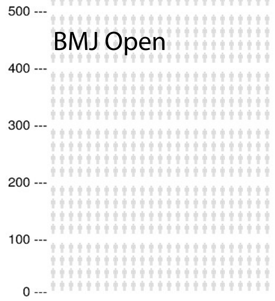

0 %
No one will have a side effect.

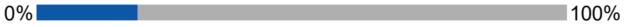

For peer review only

Repeated section

Remember Medicine A and Medicine B are equally effective and have no risks of a side effect.

The only difference between Medicine A and Medicine B is how you take them.

Medicine A	Medicine B
You take a tablet only once in your life. You will be given the tablet on the day you come in to see your GP. 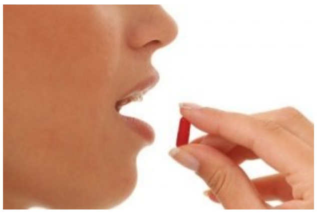	One tablet should be taken once a week on the same day every week. You need to take this medicine for 5 years. You need to take the tablet first thing in the morning on an empty stomach with a large glass of tap water. After taking the tablet, you should stay upright for the next 30 minutes. You should not eat anything for at least 30 minutes, including taking any other medicines. You need to regularly visit your doctor to have your bone health checked. You need to regularly visit a pharmacy for a new supply. 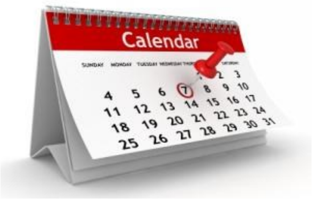 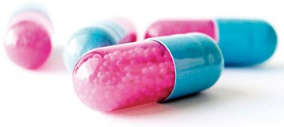

If you take Medicine A, you would live for **2.5 years (2 years and 6 months)** and then die.

If you take Medicine B, you would live for **5 years** and then die.

Would you prefer Medicine A or Medicine B, or are they the same?

Indicate your choice here. You can only choose one option:

○ **Medicine A** 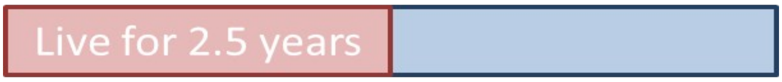

○ **Medicine B** 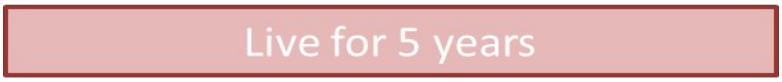

○ **The Same - Medicine A and Medicine B are the same.**

For peer review only

Repeated section

Remember Medicine A and Medicine B are equally effective and have no risks of a side effect.

The only difference between Medicine A and Medicine B is how you take them.

**Medicine A**	**Medicine B**
You take a tablet only once in your life. You will be given the tablet on the day you come in to see your GP. 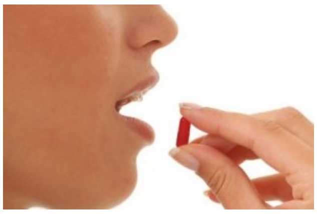	One tablet should be taken once a week on the same day every week.<br>You need to take this medicine for 5 years.<br><br>You need to take the tablet first thing in the morning on an empty stomach with a large glass of tap water.<br>After taking the tablet, you should stay upright for the next 30 minutes.<br>You should not eat anything for at least 30 minutes, including taking any other medicines.<br><br>You need to regularly visit your doctor to have your bone health checked.<br>You need to regularly visit a pharmacy for a new supply.<br>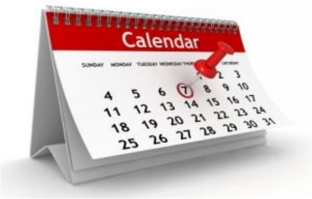 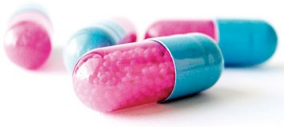

If you take Medicine A, you would live for **3 years** and then die.

If you take Medicine B, you would live for **5 years** and then die.

Would you prefer Medicine A or Medicine B, or are they the same?

Indicate your choice here. You can only choose one option:

◯ **Medicine A** Live for 3 years

◯ **Medicine B** Live for 5 years

◯ **The Same - Medicine A and Medicine B are the same.**

0% 100%

For peer review only

Repeated section

Remember Medicine A and Medicine B are equally effective and have no risks of a side effect.

The only difference between Medicine A and Medicine B is how you take them.

Medicine A	Medicine B
You take a tablet only once in your life. You will be given the tablet on the day you come in to see your GP.<br>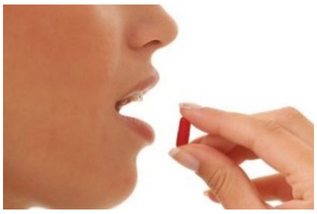	One tablet should be taken once a week on the same day every week.<br>You need to take this medicine for 5 years.<br><br>You need to take the tablet first thing in the morning on an empty stomach with a large glass of tap water.<br>After taking the tablet, you should stay upright for the next 30 minutes.<br>You should not eat anything for at least 30 minutes, including taking any other medicines.<br><br>You need to regularly visit your doctor to have your bone health checked.<br>You need to regularly visit a pharmacy for a new supply.<br>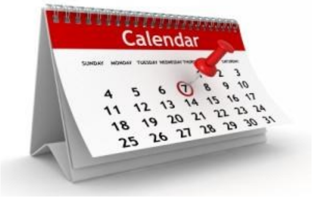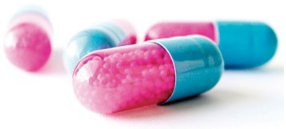

If you take Medicine A, you would live for **3.25 years** (3 years and 3 months) and then die.

If you take Medicine B, you would live for **5 years** and then die.

Would you prefer Medicine A or Medicine B, or are they the same?

Indicate your choice here. You can only choose one option:

◯ **Medicine A** Live for 3 years 3 months

◯ **Medicine B** Live for 5 years

◯ **The Same - Medicine A and Medicine B are the same.**

0% 100%

For peer review only

Repeated section

Remember Medicine A and Medicine B are equally effective and have no risks of a side effect.

The only difference between Medicine A and Medicine B is how you take them.

Medicine A	Medicine B
You take a tablet only once in your life. You will be given the tablet on the day you come in to see your GP. 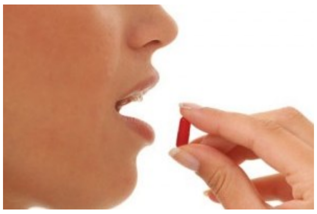	One tablet should be taken once a week on the same day every week. You need to take this medicine for 5 years.<br><br>You need to take the tablet first thing in the morning on an empty stomach with a large glass of tap water. After taking the tablet, you should stay upright for the next 30 minutes. You should not eat anything for at least 30 minutes, including taking any other medicines.<br><br>You need to regularly visit your doctor to have your bone health checked. You need to regularly visit a pharmacy for a new supply. 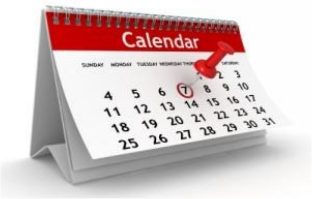 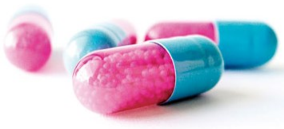

If you take Medicine A, you would live for **3.5 years** (3 years and 6 months) and then die.

If you take Medicine B, you would live for **5 years** and then die.

Would you prefer Medicine A or Medicine B, or are they the same?

Indicate your choice here. You can only choose one option:

◯ **Medicine A** Live for 3 years 6 months

◯ **Medicine B** Live for 5 years

◯ **The Same - Medicine A and Medicine B are the same.**

0% 100%

For peer review only

Repeated section

Remember Medicine A and Medicine B are equally effective and have no risks of a side effect.

The only difference between Medicine A and Medicine B is how you take them.

Medicine A	Medicine B
You take a tablet only once in your life. You will be given the tablet on the day you come in to see your GP.<br>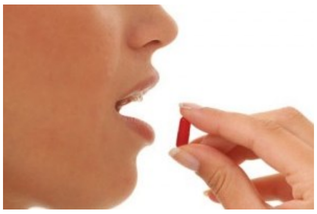	One tablet should be taken once a week on the same day every week.<br>You need to take this medicine for 5 years.<br><br>You need to take the tablet first thing in the morning on an empty stomach with a large glass of tap water.<br>After taking the tablet, you should stay upright for the next 30 minutes.<br>You should not eat anything for at least 30 minutes, including taking any other medicines.<br><br>You need to regularly visit your doctor to have your bone health checked.<br>You need to regularly visit a pharmacy for a new supply.<br>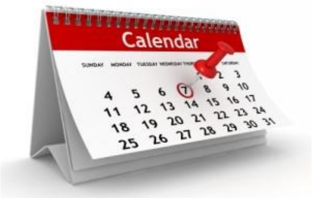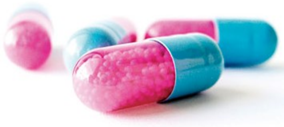

If you take Medicine A, you would live for **3.75 years** (3 years and 9 months) and then die.

If you take Medicine B, you would live for **5 years** and then die.

Would you prefer Medicine A or Medicine B, or are they the same?

Indicate your choice here. You can only choose one option:

◯ **Medicine A** Live for 3 years 9 months

◯ **Medicine B** Live for 5 years

◯ **The Same - Medicine A and Medicine B are the same.**

0% 100%

For peer review only

Repeated section

Remember Medicine A and Medicine B are equally effective and have no risks of a side effect.

The only difference between Medicine A and Medicine B is how you take them.

Medicine A	Medicine B
You take a tablet only once in your life. You will be given the tablet on the day you come in to see your GP. 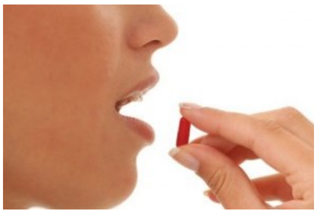	One tablet should be taken once a week on the same day every week. You need to take this medicine for 5 years.<br><br>You need to take the tablet first thing in the morning on an empty stomach with a large glass of tap water. After taking the tablet, you should stay upright for the next 30 minutes. You should not eat anything for at least 30 minutes, including taking any other medicines.<br><br>You need to regularly visit your doctor to have your bone health checked. You need to regularly visit a pharmacy for a new supply. 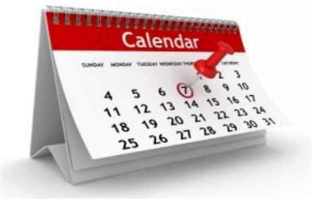 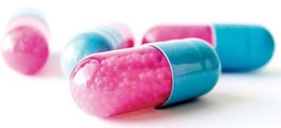

If you take Medicine A, you would live for **3 years and 10 months** and then die.

If you take Medicine B, you would live for **5 years** and then die.

Would you prefer Medicine A or Medicine B, or are they the same?

Indicate your choice here. You can only choose one option:

○ **Medicine A** Live for 3 years 10 months

○ **Medicine B** Live for 5 years

○ **The Same - Medicine A and Medicine B are the same.**

For peer review only

Repeated section

Remember Medicine A and Medicine B are equally effective and have no risks of a side effect.

The only difference between Medicine A and Medicine B is how you take them.

Medicine A	Medicine B
You take a tablet only once in your life.<br>You will be given the tablet on the day you come in to see your GP.<br>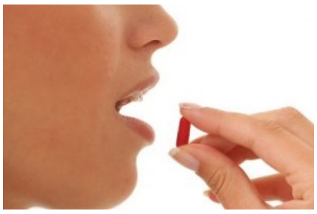	One tablet should be taken once a week on the same day every week.<br>You need to take this medicine for 5 years.<br><br>You need to take the tablet first thing in the morning on an empty stomach with a large glass of tap water.<br>After taking the tablet, you should stay upright for the next 30 minutes.<br>You should not eat anything for at least 30 minutes, including taking any other medicines.<br><br>You need to regularly visit your doctor to have your bone health checked.<br>You need to regularly visit a pharmacy for a new supply.<br>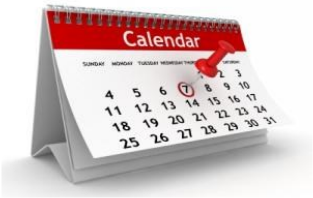 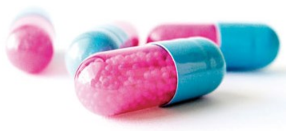

If you take Medicine A, you would live for **4 years** and then die.

If you take Medicine B, you would live for **5 years** and then die.

Would you prefer Medicine A or Medicine B, or are they the same?

Indicate your choice here. You can only choose one option:

◯ **Medicine A** — Live for 4 years

◯ **Medicine B** — Live for 5 years

◯ **The Same - Medicine A and Medicine B are the same.**

0% 100%

For peer review only

Repeated section

Remember Medicine A and Medicine B are equally effective and have no risks of a side effect.

The only difference between Medicine A and Medicine B is how you take them.

Medicine A	Medicine B
You take a tablet only once in your life. You will be given the tablet on the day you come in to see your GP. 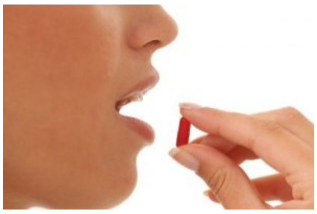	One tablet should be taken once a week on the same day every week. You need to take this medicine for 5 years.<br><br>You need to take the tablet first thing in the morning on an empty stomach with a large glass of tap water. After taking the tablet, you should stay upright for the next 30 minutes. You should not eat anything for at least 30 minutes, including taking any other medicines.<br><br>You need to regularly visit your doctor to have your bone health checked. You need to regularly visit a pharmacy for a new supply.<br>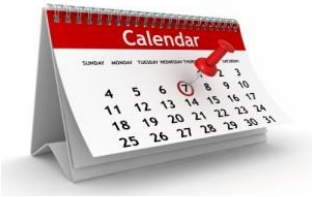 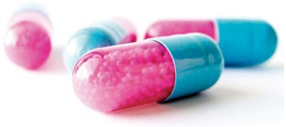

If you take Medicine A, you would live for **4 years and 2 months** and then die.

If you take Medicine B, you would live for **5 years** and then die.

Would you prefer Medicine A or Medicine B, or are they the same?

Indicate your choice here. You can only choose one option:

◯ **Medicine A** Live for 4 years 2 months

◯ **Medicine B** Live for 5 years

◯ **The Same - Medicine A and Medicine B are the same.**

0% 100%

For peer review only

Repeated section

Remember Medicine A and Medicine B are equally effective and have no risks of a side effect.

The only difference between Medicine A and Medicine B is how you take them.

Medicine A	Medicine B
You take a tablet only once in your life. You will be given the tablet on the day you come in to see your GP. 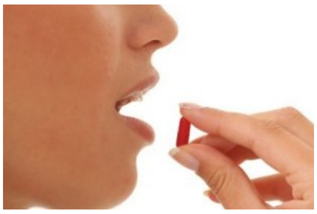	One tablet should be taken once a week on the same day every week. You need to take this medicine for 5 years.<br><br>You need to take the tablet first thing in the morning on an empty stomach with a large glass of tap water. After taking the tablet, you should stay upright for the next 30 minutes. You should not eat anything for at least 30 minutes, including taking any other medicines.<br><br>You need to regularly visit your doctor to have your bone health checked. You need to regularly visit a pharmacy for a new supply. 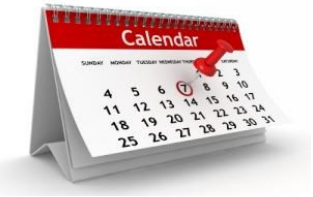 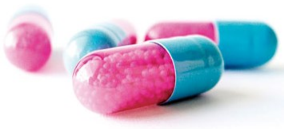

If you take Medicine A, you would live for **4.25 years** (4 years and 3 months) and then die.

If you take Medicine B, you would live for **5 years** and then die.

Would you prefer Medicine A or Medicine B, or are they the same?

Indicate your choice here. You can only choose one option:

◯ **Medicine A** Live for 4 years 3 months

◯ **Medicine B** Live for 5 years

◯ **The Same - Medicine A and Medicine B are the same.**

For peer review only

Repeated section

Remember Medicine A and Medicine B are equally effective and have no risks of a side effect.

The only difference between Medicine A and Medicine B is how you take them.

Medicine A	Medicine B
You take a tablet only once in your life. You will be given the tablet on the day you come in to see your GP. 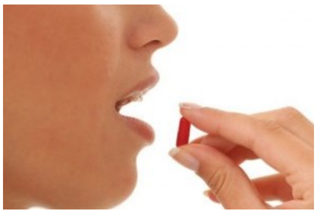	One tablet should be taken once a week on the same day every week. You need to take this medicine for 5 years.<br><br>You need to take the tablet first thing in the morning on an empty stomach with a large glass of tap water. After taking the tablet, you should stay upright for the next 30 minutes. You should not eat anything for at least 30 minutes, including taking any other medicines.<br><br>You need to regularly visit your doctor to have your bone health checked. You need to regularly visit a pharmacy for a new supply. 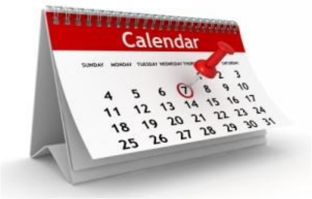 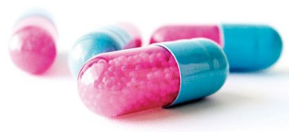

If you take Medicine A, you would live for **4 years 4 months** and then die.

If you take Medicine B, you would live for **5 years** and then die.

Would you prefer Medicine A or Medicine B, or are they the same?

Indicate your choice here. You can only choose one option:

○ **Medicine A** Live for 4 years 4 months

○ **Medicine B** Live for 5 years

○ **The Same - Medicine A and Medicine B are the same.**

0% 100%

For peer review only

Repeated section

Remember Medicine A and Medicine B are equally effective and have no risks of a side effect.

The only difference between Medicine A and Medicine B is how you take them.

Medicine A	Medicine B
You take a tablet only once in your life.<br>You will be given the tablet on the day you come in to see your GP.<br>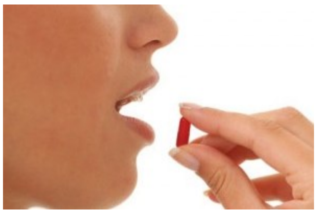	One tablet should be taken once a week on the same day every week.<br>You need to take this medicine for 5 years.<br><br>You need to take the tablet first thing in the morning on an empty stomach with a large glass of tap water.<br>After taking the tablet, you should stay upright for the next 30 minutes.<br>You should not eat anything for at least 30 minutes, including taking any other medicines.<br><br>You need to regularly visit your doctor to have your bone health checked.<br>You need to regularly visit a pharmacy for a new supply.<br>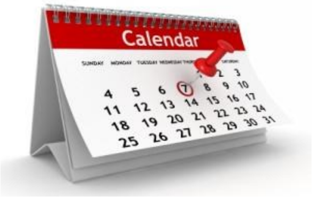 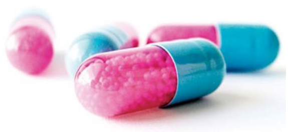

If you take Medicine A, you would live for **4.5 years** (4 years and 6 months) and then die.

If you take Medicine B, you would live for **5 years** and then die.

Would you prefer Medicine A or Medicine B, or are they the same?

Indicate your choice here. You can only choose one option:

◯ **Medicine A**

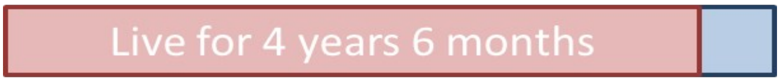

◯ **Medicine B**

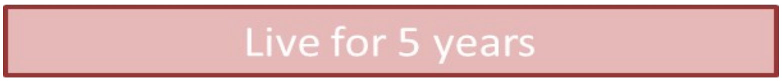

◯ **The Same - Medicine A and Medicine B are the same.**

For peer review only

Repeated section

Remember Medicine A and Medicine B are equally effective and have no risks of a side effect.

The only difference between Medicine A and Medicine B is how you take them.

Medicine A	Medicine B
You take a tablet only once in your life. You will be given the tablet on the day you come in to see your GP.<br>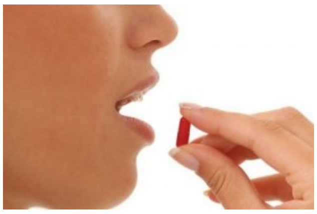	One tablet should be taken once a week on the same day every week.<br>You need to take this medicine for 5 years.<br><br>You need to take the tablet first thing in the morning on an empty stomach with a large glass of tap water.<br>After taking the tablet, you should stay upright for the next 30 minutes.<br>You should not eat anything for at least 30 minutes, including taking any other medicines.<br><br>You need to regularly visit your doctor to have your bone health checked.<br>You need to regularly visit a pharmacy for a new supply.<br>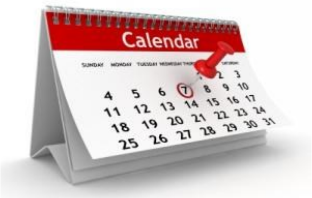 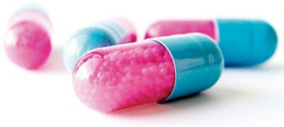

If you take Medicine A, you would live for **4 years and 7 months** and then die.

If you take Medicine B, you would live for **5 years** and then die.

Would you prefer Medicine A or Medicine B, or are they the same?

Indicate your choice here. You can only choose one option:

○ **Medicine A**

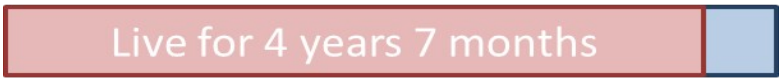

○ **Medicine B**

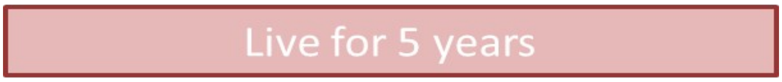

○ **The Same - Medicine A and Medicine B are the same.**

0% 100%

For peer review only

Repeated section

Remember Medicine A and Medicine B are equally effective and have no risks of a side effect.

The only difference between Medicine A and Medicine B is how you take them.

Medicine A	Medicine B
You take a tablet only once in your life.<br>You will be given the tablet on the day you come in to see your GP.<br>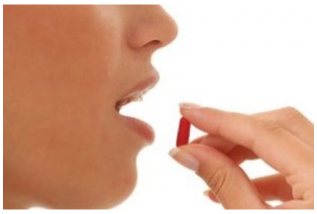	One tablet should be taken once a week on the same day every week.<br>You need to take this medicine for 5 years.<br><br>You need to take the tablet first thing in the morning on an empty stomach with a large glass of tap water.<br>After taking the tablet, you should stay upright for the next 30 minutes.<br>You should not eat anything for at least 30 minutes, including taking any other medicines.<br><br>You need to regularly visit your doctor to have your bone health checked.<br>You need to regularly visit a pharmacy for a new supply.<br>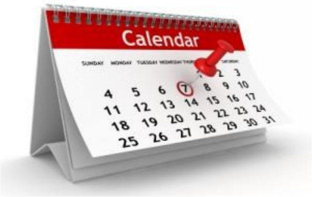 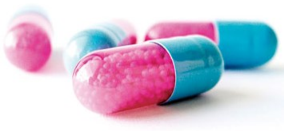

If you take Medicine A, you would live for **4 years and 9 months** and then die.

If you take Medicine B, you would live for **5 years** and then die.

Would you prefer Medicine A or Medicine B, or are they the same?

Indicate your choice here. You can only choose one option:

○ **Medicine A** Live for 4 years 9 months

○ **Medicine B** Live for 5 years

○ **The Same - Medicine A and Medicine B are the same.**

0% 100%

For peer review only

Repeated section

Remember Medicine A and Medicine B are equally effective and have no risks of a side effect.

The only difference between Medicine A and Medicine B is how you take them.

Medicine A	Medicine B
You take a tablet only once in your life. You will be given the tablet on the day you come in to see your GP. 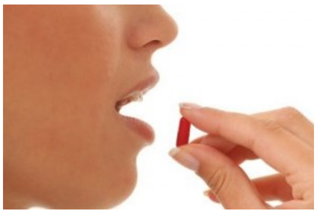	One tablet should be taken once a week on the same day every week. You need to take this medicine for 5 years.<br><br>You need to take the tablet first thing in the morning on an empty stomach with a large glass of tap water. After taking the tablet, you should stay upright for the next 30 minutes. You should not eat anything for at least 30 minutes, including taking any other medicines.<br><br>You need to regularly visit your doctor to have your bone health checked. You need to regularly visit a pharmacy for a new supply. 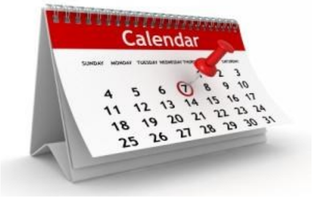 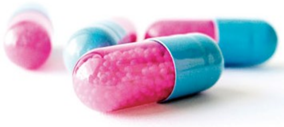

If you take Medicine A, you would live for **2 months less than 5 years** and then die.

If you take Medicine B, you would live for **5 years** and then die.

Would you prefer Medicine A or Medicine B, or are they the same?

Indicate your choice here. You can only choose one option:

○ **Medicine A** — Live for (5 years – 2 months)

○ **Medicine B** — Live for 5 years

○ **The Same - Medicine A and Medicine B are the same.**

For peer review only

Repeated section

Remember Medicine A and Medicine B are equally effective and have no risks of a side effect.

The only difference between Medicine A and Medicine B is how you take them.

Medicine A	Medicine B
You take a tablet only once in your life. You will be given the tablet on the day you come in to see your GP. 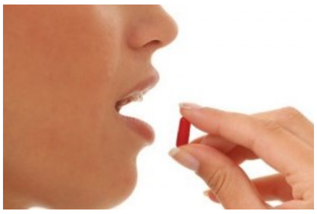	One tablet should be taken once a week on the same day every week. You need to take this medicine for 5 years.<br><br>You need to take the tablet first thing in the morning on an empty stomach with a large glass of tap water. After taking the tablet, you should stay upright for the next 30 minutes. You should not eat anything for at least 30 minutes, including taking any other medicines.<br><br>You need to regularly visit your doctor to have your bone health checked. You need to regularly visit a pharmacy for a new supply. 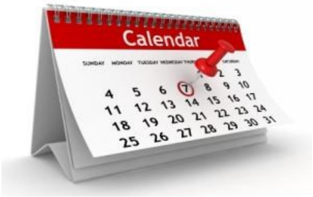 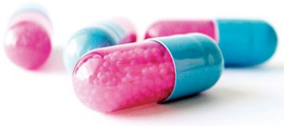

If you take Medicine A, you would live for **1 month less than 5 years** and then die.

If you take Medicine B, you would live for **5 years** and then die.

Would you prefer Medicine A or Medicine B, or are they the same?

Indicate your choice here. You can only choose one option:

◯ **Medicine A** Live for (5 years – 1 month)

◯ **Medicine B** Live for 5 years

◯ **The Same - Medicine A and Medicine B are the same.**

For peer review only

Repeated section

Remember Medicine A and Medicine B are equally effective and have no risks of a side effect.

The only difference between Medicine A and Medicine B is how you take them.

Medicine A	Medicine B
You take a tablet only once in your life. You will be given the tablet on the day you come in to see your GP.<br>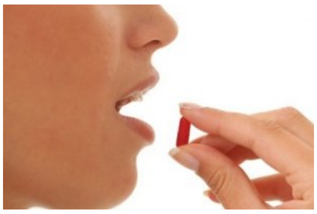	One tablet should be taken once a week on the same day every week.<br>You need to take this medicine for 5 years.<br><br>You need to take the tablet first thing in the morning on an empty stomach with a large glass of tap water.<br>After taking the tablet, you should stay upright for the next 30 minutes.<br>You should not eat anything for at least 30 minutes, including taking any other medicines.<br><br>You need to regularly visit your doctor to have your bone health checked.<br>You need to regularly visit a pharmacy for a new supply.<br>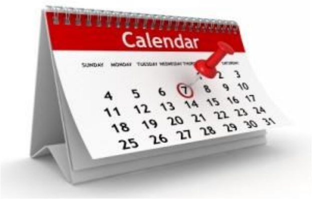 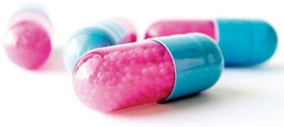

If you take Medicine A, you would live for **2 weeks less than 5 years** and then die.

If you take Medicine B, you would live for **5 years** and then die.

Would you prefer Medicine A or Medicine B, or are they the same?

Indicate your choice here. You can only choose one option:

◯ **Medicine A** Live for (5 years – 2 weeks)

◯ **Medicine B** Live for 5 years

◯ **The Same - Medicine A and Medicine B are the same.**

0% 100%

For peer review only

Repeated section

Remember Medicine A and Medicine B are equally effective and have no risks of a side effect.

The only difference between Medicine A and Medicine B is how you take them.

Medicine A	Medicine B
You take a tablet only once in your life. You will be given the tablet on the day you come in to see your GP. 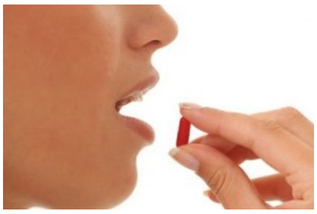	One tablet should be taken once a week on the same day every week. You need to take this medicine for 5 years.<br><br>You need to take the tablet first thing in the morning on an empty stomach with a large glass of tap water. After taking the tablet, you should stay upright for the next 30 minutes. You should not eat anything for at least 30 minutes, including taking any other medicines.<br><br>You need to regularly visit your doctor to have your bone health checked. You need to regularly visit a pharmacy for a new supply.<br><br>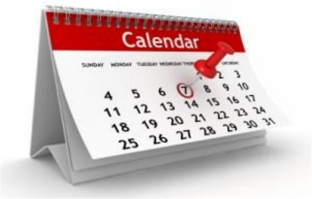 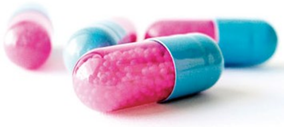

If you take Medicine A, you would live for **1 week less than 5 years** and then die.

If you take Medicine B, you would live for **5 years** and then die.

Would you prefer Medicine A or Medicine B, or are they the same?

Indicate your choice here. You can only choose one option:

◯ **Medicine A** Live for (5 years – 1 week)

◯ **Medicine B** Live for 5 years

◯ **The Same - Medicine A and Medicine B are the same.**

0% 100%

For peer review only

Repeated section

Remember Medicine A and Medicine B are equally effective and have no risks of a side effect.

The only difference between Medicine A and Medicine B is how you take them.

Medicine A	Medicine B
You take a tablet only once in your life. You will be given the tablet on the day you come in to see your GP.<br>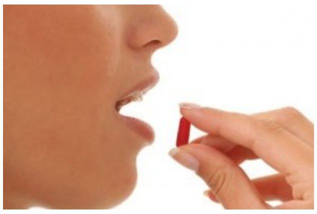	One tablet should be taken once a week on the same day every week.<br>You need to take this medicine for 5 years.<br><br>You need to take the tablet first thing in the morning on an empty stomach with a large glass of tap water.<br>After taking the tablet, you should stay upright for the next 30 minutes.<br>You should not eat anything for at least 30 minutes, including taking any other medicines.<br><br>You need to regularly visit your doctor to have your bone health checked.<br>You need to regularly visit a pharmacy for a new supply.<br>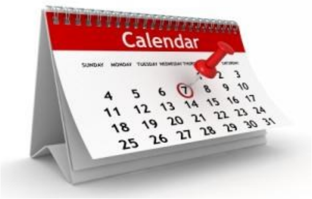 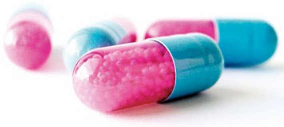

If you take Medicine A, you would live for **5 days less than 5 years** and then die.

If you take Medicine B, you would live for **5 years** and then die.

Would you prefer Medicine A or Medicine B, or are they the same?

Indicate your choice here. You can only choose one option:

○ **Medicine A** Live for (5 years – 5 days)

○ **Medicine B** Live for 5 years

○ **The Same - Medicine A and Medicine B are the same.**

0% 100%

For peer review only

Repeated section

Remember Medicine A and Medicine B are equally effective and have no risks of a side effect.

The only difference between Medicine A and Medicine B is how you take them.

Medicine A	Medicine B
You take a tablet only once in your life. You will be given the tablet on the day you come in to see your GP.<br>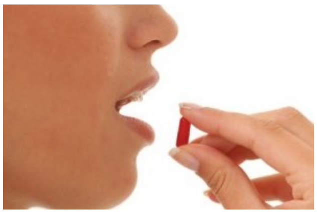	One tablet should be taken once a week on the same day every week.<br>You need to take this medicine for 5 years.<br><br>You need to take the tablet first thing in the morning on an empty stomach with a large glass of tap water.<br>After taking the tablet, you should stay upright for the next 30 minutes.<br>You should not eat anything for at least 30 minutes, including taking any other medicines.<br><br>You need to regularly visit your doctor to have your bone health checked.<br>You need to regularly visit a pharmacy for a new supply.<br>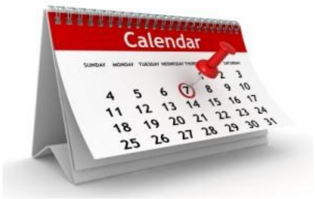 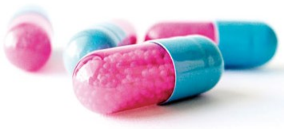

If you take Medicine A, you would live for **3 days less than 5 years** and then die.

If you take Medicine B, you would live for **5 years** and then die.

Would you prefer Medicine A or Medicine B, or are they the same?

Indicate your choice here. You can only choose one option:

◯ **Medicine A** Live for (5 years – 3 days)

◯ **Medicine B** Live for 5 years

◯ **The Same - Medicine A and Medicine B are the same.**

0% 100%

For peer review only

Repeated section

Remember Medicine A and Medicine B are equally effective and have no risks of a side effect.

The only difference between Medicine A and Medicine B is how you take them.

Medicine A	Medicine B
You take a tablet only once in your life. You will be given the tablet on the day you come in to see your GP. 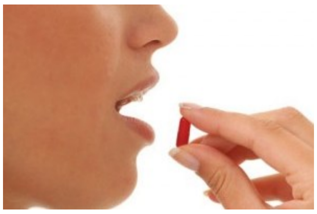	One tablet should be taken once a week on the same day every week. You need to take this medicine for 5 years.<br><br>You need to take the tablet first thing in the morning on an empty stomach with a large glass of tap water. After taking the tablet, you should stay upright for the next 30 minutes. You should not eat anything for at least 30 minutes, including taking any other medicines.<br><br>You need to regularly visit your doctor to have your bone health checked. You need to regularly visit a pharmacy for a new supply. 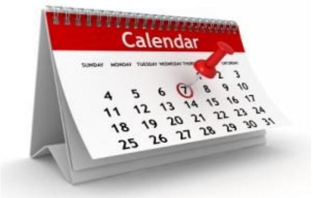 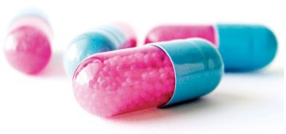

If you take Medicine A, you would live for **1 day less than 5 years** and then die.

If you take Medicine B, you would live for **5 years** and then die.

Would you prefer Medicine A or Medicine B, or are they the same?

Indicate your choice here. You can only choose one option:

◯ **Medicine A** Live for (5 years – 1 day)

◯ **Medicine B** Live for 5 years

◯ **The Same - Medicine A and Medicine B are the same.**

0% 100%

For peer review only

Question 1a.

You prefer living for 2.5 years (2 years and 6 months) on Medicine A, to living for 5 years on Medicine B.

This means **living fewer than 2.5 years on Medicine A** can be the same as living for 5 years on Medicine B.

How many years on Medicine A do you think would be the same as living for 5 years on Medicine B?

(Please give a number in years)

0% 100%

peer review only

Part 2

Question 2

You have been told that you need to take a medicine to reduce the risk of fracture.

You can choose between two different medicines. We have called these Medicine A and Medicine B.

Medicine A is a pill that you only need to take once.

Medicine B is a pill that you need to take every week for 5 years.

Medicine A and Medicine B are equally effective in reducing your risk of having a fracture.
Medicine A and Medicine B will reduce your risk of experiencing a fracture, such as hip fracture, within the next 5 years from **10%** to **6%**.

In other words, in a crowd of **1,000** people in the UK, **100** are likely to have a fracture if they do not take the medicine. This is reduced to **60** people out of 1,000 who do take the medicine.
This information is also illustrated in this picture.

Risk of fracture

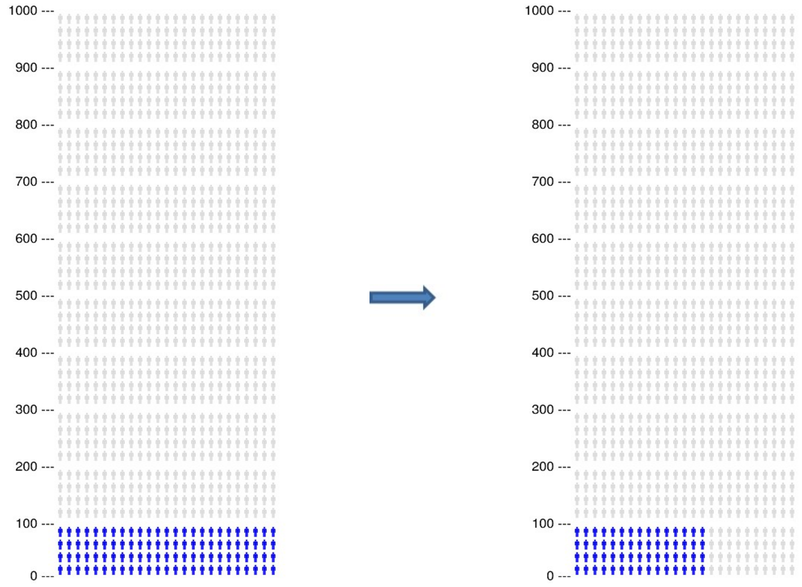

10%
No Treatment

6%
Taking the Medicine

(The figures shaded blue represent people who will have a fracture, such as hip fracture, within the next 5 years)

In this scenario, there is some risk of a minor side effect from Medicine A or Medicine B.

Among every **1,000** people taking Medicine A or Medicine B, **50** people are likely to have a minor side effect such as nausea (feeling sick) and indigestion.

Risk of a minor side effect

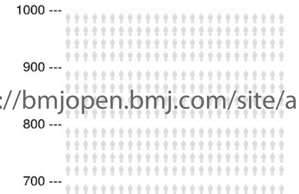

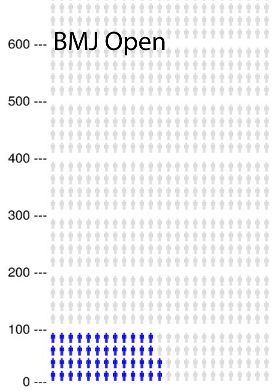

5% of people will have a minor side effect.

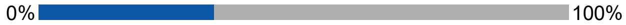

For peer review only

Repeated section

Remember Medicine A and Medicine B are equally effective and have some risk of a minor side effect.

The only difference between Medicine A and Medicine B is how you take them.

Medicine A	Medicine B
You take a tablet only once in your life. You will be given the tablet on the day you come in to see your GP. 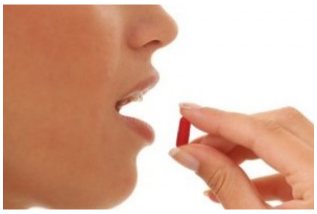	One tablet should be taken once a week on the same day every week. You need to take this medicine for 5 years.<br><br>You need to take the tablet first thing in the morning on an empty stomach with a large glass of tap water. After taking the tablet, you should stay upright for the next 30 minutes. You should not eat anything for at least 30 minutes, including taking any other medicines.<br><br>You need to regularly visit your doctor to have your bone health checked. You need to regularly visit a pharmacy for a new supply.<br>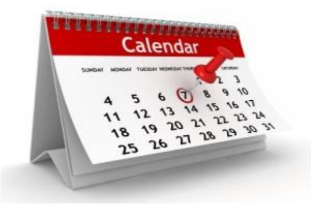 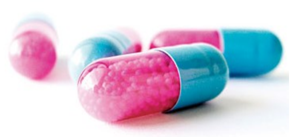

If you take Medicine A, you would live for **2.5 years (2 years and 6 months)** and then die.

If you take Medicine B, you would live for **5 years** and then die.

Would you prefer Medicine A or Medicine B, or are they the same?

Indicate your choice here. You can only choose one option:

○ **Medicine A** Live for 2.5 years

○ **Medicine B** Live for 5 years

○ **The Same - Medicine A and Medicine B are the same.**

0% 100%

For peer review only

Repeated section

Remember Medicine A and Medicine B are equally effective and have some risk of a minor side effect.

The only difference between Medicine A and Medicine B is how you take them.

Medicine A	Medicine B
You take a tablet only once in your life. You will be given the tablet on the day you come in to see your GP. 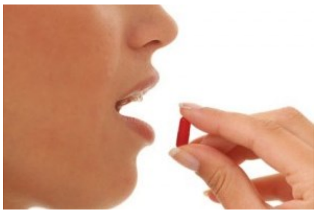	One tablet should be taken once a week on the same day every week.<br>You need to take this medicine for 5 years.<br><br>You need to take the tablet first thing in the morning on an empty stomach with a large glass of tap water.<br>After taking the tablet, you should stay upright for the next 30 minutes.<br>You should not eat anything for at least 30 minutes, including taking any other medicines.<br><br>You need to regularly visit your doctor to have your bone health checked.<br>You need to regularly visit a pharmacy for a new supply.<br>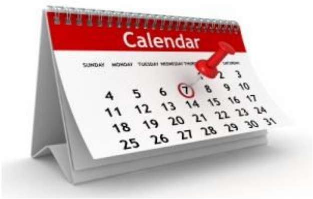 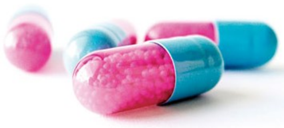

If you take Medicine A, you would live for **3 years** and then die.

If you take Medicine B, you would live for **5 years** and then die.

Would you prefer Medicine A or Medicine B, or are they the same?

Indicate your choice here. You can only choose one option:

◯ **Medicine A** Live for 3 years

◯ **Medicine B** Live for 5 years

◯ **The Same - Medicine A and Medicine B are the same.**

0% 100%

For peer review only

Repeated section

Remember Medicine A and Medicine B are equally effective and have some risk of a minor side effect.

The only difference between Medicine A and Medicine B is how you take them.

Medicine A	Medicine B
You take a tablet only once in your life.<br>You will be given the tablet on the day you come in to see your GP.<br>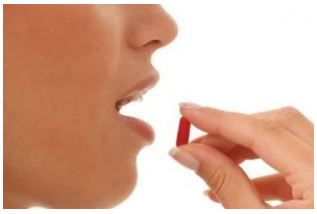	One tablet should be taken once a week on the same day every week.<br>You need to take this medicine for 5 years.<br><br>You need to take the tablet first thing in the morning on an empty stomach with a large glass of tap water.<br>After taking the tablet, you should stay upright for the next 30 minutes.<br>You should not eat anything for at least 30 minutes, including taking any other medicines.<br><br>You need to regularly visit your doctor to have your bone health checked.<br>You need to regularly visit a pharmacy for a new supply.<br>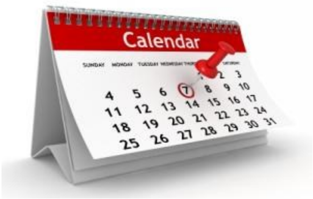 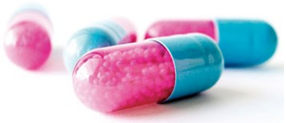

If you take Medicine A, you would live for **3.25 years** (3 years and 3 months) and then die.

If you take Medicine B, you would live for **5 years** and then die.

Would you prefer Medicine A or Medicine B, or are they the same?

Indicate your choice here. You can only choose one option:

◯ **Medicine A** Live for 3 years 3 months

◯ **Medicine B** Live for 5 years

◯ **The Same - Medicine A and Medicine B are the same.**

For peer review only

Repeated section

Remember Medicine A and Medicine B are equally effective and have some risk of a minor side effect.

The only difference between Medicine A and Medicine B is how you take them.

Medicine A	Medicine B
You take a tablet only once in your life. You will be given the tablet on the day you come in to see your GP. 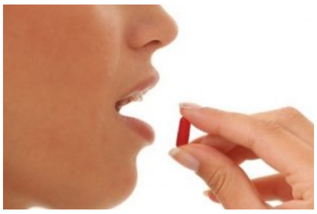	One tablet should be taken once a week on the same day every week. You need to take this medicine for 5 years.<br><br>You need to take the tablet first thing in the morning on an empty stomach with a large glass of tap water. After taking the tablet, you should stay upright for the next 30 minutes. You should not eat anything for at least 30 minutes, including taking any other medicines.<br><br>You need to regularly visit your doctor to have your bone health checked. You need to regularly visit a pharmacy for a new supply.<br>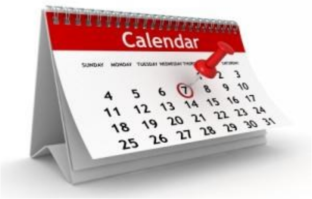 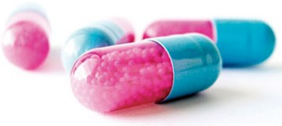

If you take Medicine A, you would live for **3.5 years** (3 years and 6 months) and then die.

If you take Medicine B, you would live for **5 years** and then die.

Would you prefer Medicine A or Medicine B, or are they the same?

Indicate your choice here. You can only choose one option:

◯ **Medicine A** Live for 3 years 6 months

◯ **Medicine B** Live for 5 years

◯ **The Same - Medicine A and Medicine B are the same.**

0% 100%

For peer review only

Repeated section

Remember Medicine A and Medicine B are equally effective and have some risk of a minor side effect.

The only difference between Medicine A and Medicine B is how you take them.

Medicine A	Medicine B
You take a tablet only once in your life. You will be given the tablet on the day you come in to see your GP. 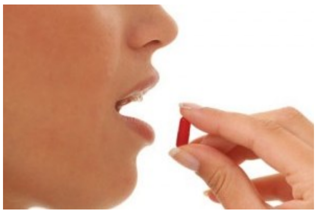	One tablet should be taken once a week on the same day every week. You need to take this medicine for 5 years.<br><br>You need to take the tablet first thing in the morning on an empty stomach with a large glass of tap water. After taking the tablet, you should stay upright for the next 30 minutes. You should not eat anything for at least 30 minutes, including taking any other medicines.<br><br>You need to regularly visit your doctor to have your bone health checked. You need to regularly visit a pharmacy for a new supply. 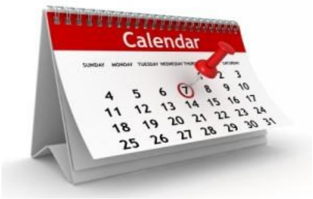 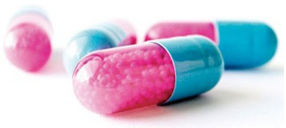

If you take Medicine A, you would live for **3.75 years** (3 years and 9 months) and then die.

If you take Medicine B, you would live for **5 years** and then die.

Would you prefer Medicine A or Medicine B, or are they the same?

Indicate your choice here. You can only choose one option:

◯ **Medicine A** Live for 3 years 9 months

◯ **Medicine B** Live for 5 years

◯ **The Same - Medicine A and Medicine B are the same.**

For peer review only

Repeated section

Remember Medicine A and Medicine B are equally effective and have some risk of a minor side effect.

The only difference between Medicine A and Medicine B is how you take them.

Medicine A	Medicine B
You take a tablet only once in your life.<br>You will be given the tablet on the day you come in to see your GP.<br>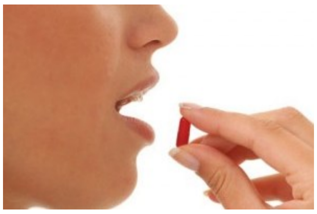	One tablet should be taken once a week on the same day every week.<br>You need to take this medicine for 5 years.<br><br>You need to take the tablet first thing in the morning on an empty stomach with a large glass of tap water.<br>After taking the tablet, you should stay upright for the next 30 minutes.<br>You should not eat anything for at least 30 minutes, including taking any other medicines.<br><br>You need to regularly visit your doctor to have your bone health checked.<br>You need to regularly visit a pharmacy for a new supply.<br>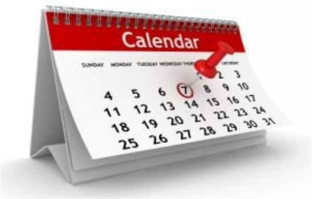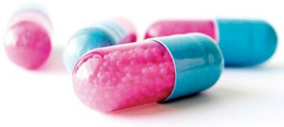

If you take Medicine A, you would live for **3 years and 10 months** and then die.

If you take Medicine B, you would live for **5 years** and then die.

Would you prefer Medicine A or Medicine B, or are they the same?

Indicate your choice here. You can only choose one option:

○ **Medicine A** Live for 3 years 10 months

○ **Medicine B** Live for 5 years

○ **The Same - Medicine A and Medicine B are the same.**

0% 100%

For peer review only

Repeated section

Remember Medicine A and Medicine B are equally effective and have some risk of a minor side effect.

The only difference between Medicine A and Medicine B is how you take them.

Medicine A	Medicine B
You take a tablet only once in your life.<br>You will be given the tablet on the day you come in to see your GP.<br>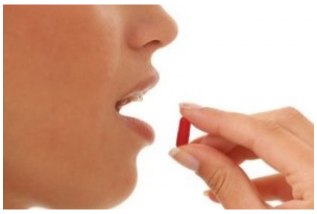	One tablet should be taken once a week on the same day every week.<br>You need to take this medicine for 5 years.<br><br>You need to take the tablet first thing in the morning on an empty stomach with a large glass of tap water.<br>After taking the tablet, you should stay upright for the next 30 minutes.<br>You should not eat anything for at least 30 minutes, including taking any other medicines.<br><br>You need to regularly visit your doctor to have your bone health checked.<br>You need to regularly visit a pharmacy for a new supply.<br>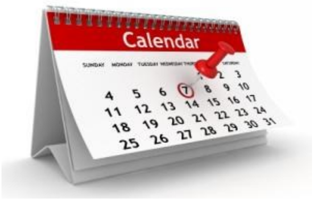 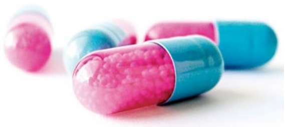

If you take Medicine A, you would live for **4 years** and then die.

If you take Medicine B, you would live for **5 years** and then die.

Would you prefer Medicine A or Medicine B, or are they the same?

Indicate your choice here. You can only choose one option:

◯ **Medicine A** Live for 4 years

◯ **Medicine B** Live for 5 years

◯ **The Same - Medicine A and Medicine B are the same.**

0% 100%

For peer review only

Repeated section

Remember Medicine A and Medicine B are equally effective and have some risk of a minor side effect.

The only difference between Medicine A and Medicine B is how you take them.

Medicine A	Medicine B
You take a tablet only once in your life. You will be given the tablet on the day you come in to see your GP. 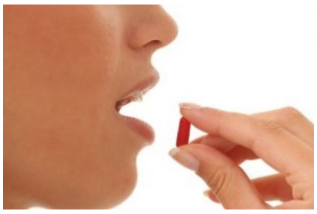	One tablet should be taken once a week on the same day every week. You need to take this medicine for 5 years. You need to take the tablet first thing in the morning on an empty stomach with a large glass of tap water. After taking the tablet, you should stay upright for the next 30 minutes. You should not eat anything for at least 30 minutes, including taking any other medicines. You need to regularly visit your doctor to have your bone health checked. You need to regularly visit a pharmacy for a new supply. 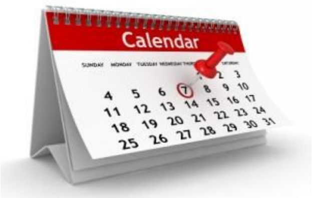 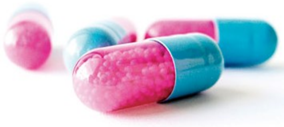

If you take Medicine A, you would live for **4 years and 2 months** and then die.

If you take Medicine B, you would live for **5 years** and then die.

Would you prefer Medicine A or Medicine B, or are they the same?

Indicate your choice here. You can only choose one option:

◯ **Medicine A** Live for 4 years 2 months

◯ **Medicine B** Live for 5 years

◯ **The Same - Medicine A and Medicine B are the same.**

For peer review only

Repeated section

Remember Medicine A and Medicine B are equally effective and have some risk of a minor side effect.

The only difference between Medicine A and Medicine B is how you take them.

Medicine A	Medicine B
You take a tablet only once in your life.<br>You will be given the tablet on the day you come in to see your GP.<br>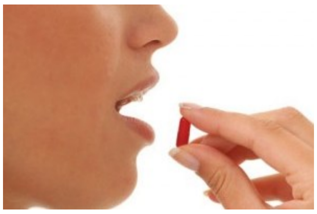	One tablet should be taken once a week on the same day every week.<br>You need to take this medicine for 5 years.<br><br>You need to take the tablet first thing in the morning on an empty stomach with a large glass of tap water.<br>After taking the tablet, you should stay upright for the next 30 minutes.<br>You should not eat anything for at least 30 minutes, including taking any other medicines.<br><br>You need to regularly visit your doctor to have your bone health checked.<br>You need to regularly visit a pharmacy for a new supply.<br>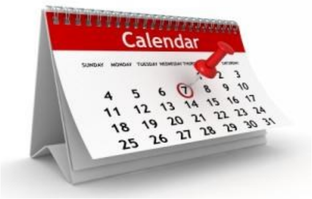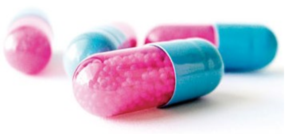

If you take Medicine A, you would live for **4.25 years** (4 years and 3 months) and then die.

If you take Medicine B, you would live for **5 years** and then die.

Would you prefer Medicine A or Medicine B, or are they the same?

Indicate your choice here. You can only choose one option:

◯ **Medicine A** Live for 4 years 3 months

◯ **Medicine B** Live for 5 years

◯ **The Same - Medicine A and Medicine B are the same.**

0% 100%

For peer review only

Repeated section

Remember Medicine A and Medicine B are equally effective and have some risk of a minor side effect.

The only difference between Medicine A and Medicine B is how you take them.

Medicine A	Medicine B
You take a tablet only once in your life.<br>You will be given the tablet on the day you come in to see your GP.<br>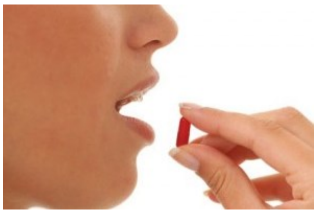	One tablet should be taken once a week on the same day every week.<br>You need to take this medicine for 5 years.<br><br>You need to take the tablet first thing in the morning on an empty stomach with a large glass of tap water.<br>After taking the tablet, you should stay upright for the next 30 minutes.<br>You should not eat anything for at least 30 minutes, including taking any other medicines.<br><br>You need to regularly visit your doctor to have your bone health checked.<br>You need to regularly visit a pharmacy for a new supply.<br>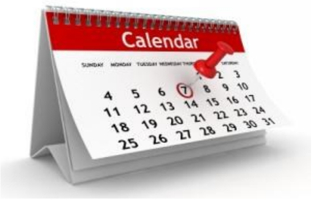 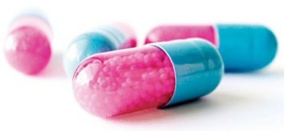

If you take Medicine A, you would live for **4 years 4 months** and then die.

If you take Medicine B, you would live for **5 years** and then die.

Would you prefer Medicine A or Medicine B, or are they the same?

Indicate your choice here. You can only choose one option:

◯ **Medicine A** Live for 4 years 4 months

◯ **Medicine B** Live for 5 years

◯ **The Same - Medicine A and Medicine B are the same.**

0% 100%

For peer review only

Repeated section

Remember Medicine A and Medicine B are equally effective and have no risks of a minor side effect.

The only difference between Medicine A and Medicine B is how you take them.

Medicine A	Medicine B
You take a tablet only once in your life. You will be given the tablet on the day you come in to see your GP.<br>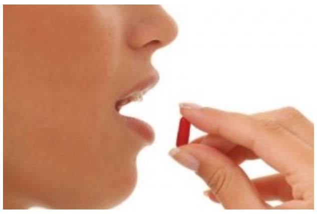	One tablet should be taken once a week on the same day every week.<br>You need to take this medicine for 5 years.<br><br>You need to take the tablet first thing in the morning on an empty stomach with a large glass of tap water.<br>After taking the tablet, you should stay upright for the next 30 minutes.<br>You should not eat anything for at least 30 minutes, including taking any other medicines.<br><br>You need to regularly visit your doctor to have your bone health checked.<br>You need to regularly visit a pharmacy for a new supply.<br>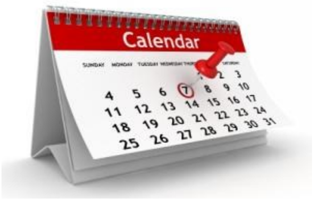 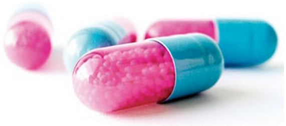

If you take Medicine A, you would live for **4.5 years** (4 years and 6 months) and then die.

If you take Medicine B, you would live for **5 years** and then die.

Would you prefer Medicine A or Medicine B, or are they the same?

Indicate your choice here. You can only choose one option:

○ **Medicine A** Live for 4 years 6 months

○ **Medicine B** Live for 5 years

○ **The Same - Medicine A and Medicine B are the same.**

0% 100%

For peer review only

Repeated section

Remember Medicine A and Medicine B are equally effective and have some risk of a minor side effect.

The only difference between Medicine A and Medicine B is how you take them.

Medicine A	Medicine B
You take a tablet only once in your life. You will be given the tablet on the day you come in to see your GP. 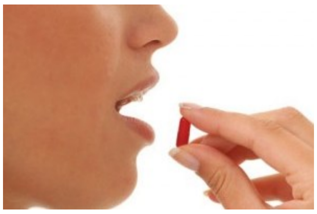	One tablet should be taken once a week on the same day every week.<br>You need to take this medicine for 5 years.<br><br>You need to take the tablet first thing in the morning on an empty stomach with a large glass of tap water.<br>After taking the tablet, you should stay upright for the next 30 minutes.<br>You should not eat anything for at least 30 minutes, including taking any other medicines.<br><br>You need to regularly visit your doctor to have your bone health checked.<br>You need to regularly visit a pharmacy for a new supply.<br>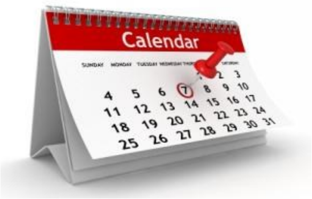 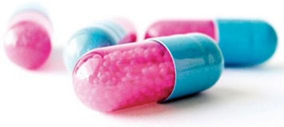

If you take Medicine A, you would live for **4 years and 7 months** and then die.

If you take Medicine B, you would live for **5 years** and then die.

Would you prefer Medicine A or Medicine B, or are they the same?

Indicate your choice here. You can only choose one option:

◯ **Medicine A** Live for 4 years 7 months

◯ **Medicine B** Live for 5 years

◯ **The Same - Medicine A and Medicine B are the same.**

0% 100%

For peer review only

Repeated section

Remember Medicine A and Medicine B are equally effective and have some risk of a minor side effect.

The only difference between Medicine A and Medicine B is how you take them.

Medicine A	Medicine B
You take a tablet only once in your life. You will be given the tablet on the day you come in to see your GP. 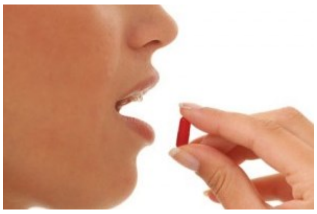	One tablet should be taken once a week on the same day every week.<br>You need to take this medicine for 5 years.<br><br>You need to take the tablet first thing in the morning on an empty stomach with a large glass of tap water.<br>After taking the tablet, you should stay upright for the next 30 minutes.<br>You should not eat anything for at least 30 minutes, including taking any other medicines.<br><br>You need to regularly visit your doctor to have your bone health checked.<br>You need to regularly visit a pharmacy for a new supply.<br>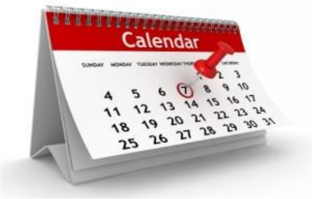 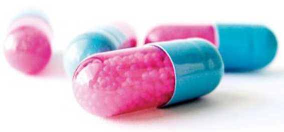

If you take Medicine A, you would live for **4 years and 9 months** and then die.

If you take Medicine B, you would live for **5 years** and then die.

Would you prefer Medicine A or Medicine B, or are they the same?

Indicate your choice here. You can only choose one option:

○ **Medicine A** Live for 4 years 9 months

○ **Medicine B** Live for 5 years

○ **The Same - Medicine A and Medicine B are the same.**

For peer review only

Repeated section

Remember Medicine A and Medicine B are equally effective and have some risk of a minor side effect.

The only difference between Medicine A and Medicine B is how you take them.

Medicine A	Medicine B
You take a tablet only once in your life.<br>You will be given the tablet on the day you come in to see your GP.<br>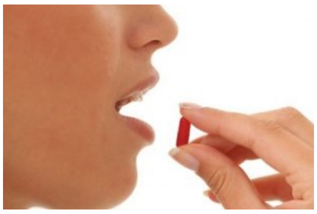	One tablet should be taken once a week on the same day every week.<br>You need to take this medicine for 5 years.<br><br>You need to take the tablet first thing in the morning on an empty stomach with a large glass of tap water.<br>After taking the tablet, you should stay upright for the next 30 minutes.<br>You should not eat anything for at least 30 minutes, including taking any other medicines.<br><br>You need to regularly visit your doctor to have your bone health checked.<br>You need to regularly visit a pharmacy for a new supply.<br>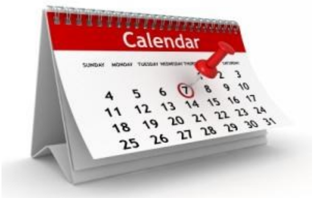 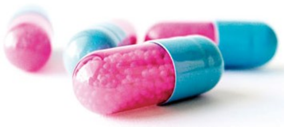

If you take Medicine A, you would live for **2 months less than 5 years** and then die.

If you take Medicine B, you would live for **5 years** and then die.

Would you prefer Medicine A or Medicine B, or are they the same?

Indicate your choice here. You can only choose one option:

○ **Medicine A** Live for (5 years – 2 months)

○ **Medicine B** Live for 5 years

○ **The Same - Medicine A and Medicine B are the same.**

0% 100%

For peer review only

Repeated section

Remember Medicine A and Medicine B are equally effective and have some risk of a minor side effect.

The only difference between Medicine A and Medicine B is how you take them.

**Medicine A**	**Medicine B**
You take a tablet only once in your life. You will be given the tablet on the day you come in to see your GP.<br>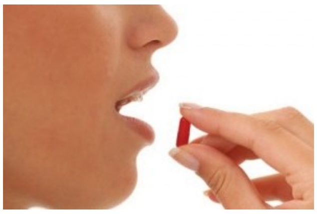	One tablet should be taken once a week on the same day every week.<br>You need to take this medicine for 5 years.<br><br>You need to take the tablet first thing in the morning on an empty stomach with a large glass of tap water.<br>After taking the tablet, you should stay upright for the next 30 minutes.<br>You should not eat anything for at least 30 minutes, including taking any other medicines.<br><br>You need to regularly visit your doctor to have your bone health checked.<br>You need to regularly visit a pharmacy for a new supply.<br>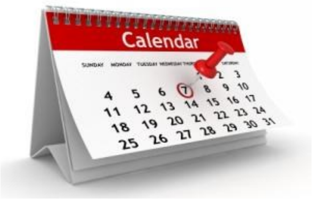 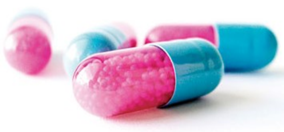

If you take Medicine A, you would live for **1 month less than 5 years** and then die.

If you take Medicine B, you would live for **5 years** and then die.

Would you prefer Medicine A or Medicine B, or are they the same?

Indicate your choice here. You can only choose one option:

◯ **Medicine A** Live for (5 years – 1 month)

◯ **Medicine B** Live for 5 years

◯ **The Same - Medicine A and Medicine B are the same.**

0% 100%

For peer review only

Repeated section

Remember Medicine A and Medicine B are equally effective and have some risk of a minor side effect.

The only difference between Medicine A and Medicine B is how you take them.

Medicine A	Medicine B
You take a tablet only once in your life.<br>You will be given the tablet on the day you come in to see your GP.<br>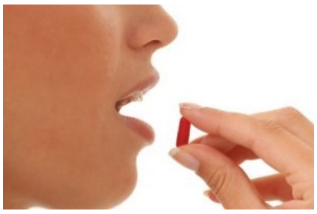	One tablet should be taken once a week on the same day every week.<br>You need to take this medicine for 5 years.<br><br>You need to take the tablet first thing in the morning on an empty stomach with a large glass of tap water.<br>After taking the tablet, you should stay upright for the next 30 minutes.<br>You should not eat anything for at least 30 minutes, including taking any other medicines.<br><br>You need to regularly visit your doctor to have your bone health checked.<br>You need to regularly visit a pharmacy for a new supply.<br>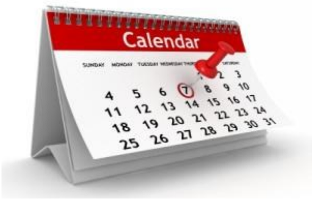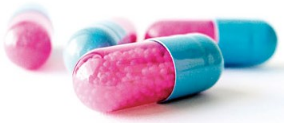

If you take Medicine A, you would live for **2 weeks less than 5 years** and then die.

If you take Medicine B, you would live for **5 years** and then die.

Would you prefer Medicine A or Medicine B, or are they the same?

Indicate your choice here. You can only choose one option:

- ◯ **Medicine A** Live for (5 years – 2 weeks)
- ◯ **Medicine B** Live for 5 years
- ◯ **The Same - Medicine A and Medicine B are the same.**

For peer review only

Repeated section

Remember Medicine A and Medicine B are equally effective and have some risk of a minor side effect.

The only difference between Medicine A and Medicine B is how you take them.

**Medicine A**	**Medicine B**
You take a tablet only once in your life. You will be given the tablet on the day you come in to see your GP. 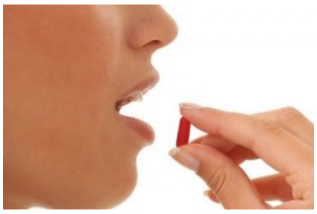	One tablet should be taken once a week on the same day every week.<br>You need to take this medicine for 5 years.<br><br>You need to take the tablet first thing in the morning on an empty stomach with a large glass of tap water.<br>After taking the tablet, you should stay upright for the next 30 minutes.<br>You should not eat anything for at least 30 minutes, including taking any other medicines.<br><br>You need to regularly visit your doctor to have your bone health checked.<br>You need to regularly visit a pharmacy for a new supply. 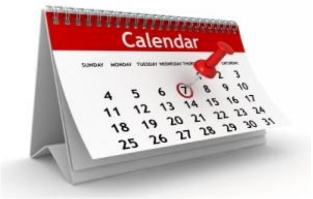 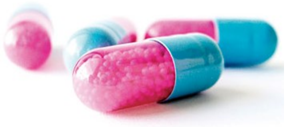

If you take Medicine A, you would live for **1 week less than 5 years** and then die.

If you take Medicine B, you would live for **5 years** and then die.

Would you prefer Medicine A or Medicine B, or are they the same?

Indicate your choice here. You can only choose one option:

◯ **Medicine A** Live for (5 years – 1 week)

◯ **Medicine B** Live for 5 years

◯ **The Same - Medicine A and Medicine B are the same.**

0% 100%

For peer review only

Repeated section

Remember Medicine A and Medicine B are equally effective and have some risk of a minor side effect.

The only difference between Medicine A and Medicine B is how you take them.

Medicine A	Medicine B
You take a tablet only once in your life. You will be given the tablet on the day you come in to see your GP. 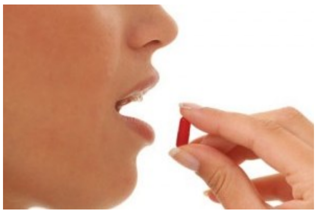	One tablet should be taken once a week on the same day every week. You need to take this medicine for 5 years.<br><br>You need to take the tablet first thing in the morning on an empty stomach with a large glass of tap water. After taking the tablet, you should stay upright for the next 30 minutes. You should not eat anything for at least 30 minutes, including taking any other medicines.<br><br>You need to regularly visit your doctor to have your bone health checked. You need to regularly visit a pharmacy for a new supply. 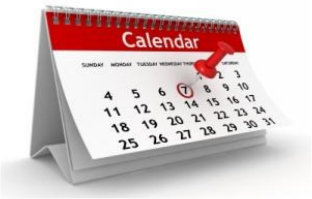 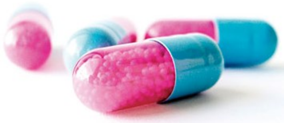

If you take Medicine A, you would live for **5 days less than 5 years** and then die.

If you take Medicine B, you would live for **5 years** and then die.

Would you prefer Medicine A or Medicine B, or are they the same?

Indicate your choice here. You can only choose one option:

○ **Medicine A** Live for (5 years – 5 days)

○ **Medicine B** Live for 5 years

○ **The Same - Medicine A and Medicine B are the same.**

0% 100%

For peer review only

Repeated section

Remember Medicine A and Medicine B are equally effective and have some risk of a minor side effect.

The only difference between Medicine A and Medicine B is how you take them.

**Medicine A**	**Medicine B**
You take a tablet only once in your life.<br>You will be given the tablet on the day you come in to see your GP.<br>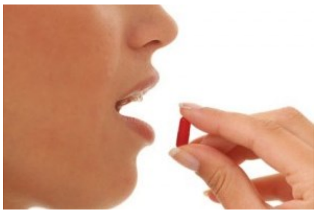	One tablet should be taken once a week on the same day every week.<br>You need to take this medicine for 5 years.<br><br>You need to take the tablet first thing in the morning on an empty stomach with a large glass of tap water.<br>After taking the tablet, you should stay upright for the next 30 minutes.<br>You should not eat anything for at least 30 minutes, including taking any other medicines.<br><br>You need to regularly visit your doctor to have your bone health checked.<br>You need to regularly visit a pharmacy for a new supply.<br>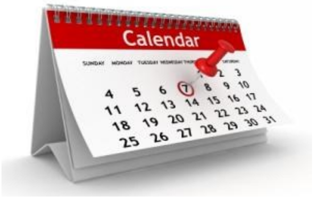 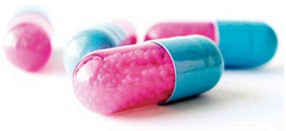

If you take Medicine A, you would live for **3 days less than 5 years** and then die.

If you take Medicine B, you would live for **5 years** and then die.

Would you prefer Medicine A or Medicine B, or are they the same?

Indicate your choice here. You can only choose one option:

◯ **Medicine A** Live for (5 years – 3 days)

◯ **Medicine B** Live for 5 years

◯ **The Same - Medicine A and Medicine B are the same.**

0% 100%

For peer review only

Repeated section

Remember Medicine A and Medicine B are equally effective and have some risk of a minor side effect.

The only difference between Medicine A and Medicine B is how you take them.

Medicine A	Medicine B
You take a tablet only once in your life. You will be given the tablet on the day you come in to see your GP.<br>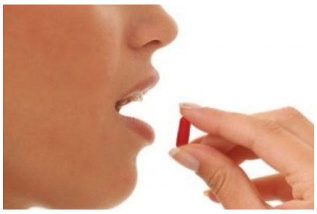	One tablet should be taken once a week on the same day every week.<br>You need to take this medicine for 5 years.<br><br>You need to take the tablet first thing in the morning on an empty stomach with a large glass of tap water.<br>After taking the tablet, you should stay upright for the next 30 minutes.<br>You should not eat anything for at least 30 minutes, including taking any other medicines.<br><br>You need to regularly visit your doctor to have your bone health checked.<br>You need to regularly visit a pharmacy for a new supply.<br>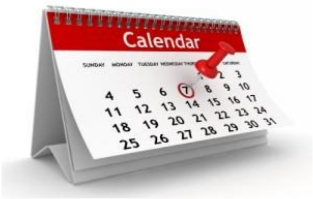 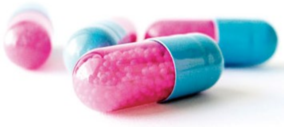

If you take Medicine A, you would live for **1 day less than 5 years** and then die.

If you take Medicine B, you would live for **5 years** and then die.

Would you prefer Medicine A or Medicine B, or are they the same?

Indicate your choice here. You can only choose one option:

○ **Medicine A** Live for (5 years – 1 day)

○ **Medicine B** Live for 5 years

○ **The Same - Medicine A and Medicine B are the same.**

0% 100%

For peer review only

Question 2a.

You prefer living for 2.5 years (2 years and 6 months) on Medicine A, to living for 5 years on Medicine B.

This means **living fewer than 2.5 years on Medicine A** can be the same as living for 5 years on Medicine B.

How many years on Medicine A do you think would be the same as living for 5 years on Medicine B?

(Please give a number in years)

0% 100%

peer review only

Part 2

Question 3

You have been told that you need to take a medicine to reduce the risk of fracture.

You can choose between two different medicines. We have called these Medicine A and Medicine B.

Medicine A is a pill that you only need to take once.

Medicine B is a pill that you need to take every week for 5 years.

Medicine A and Medicine B are equally effective in reducing your risk of having a fracture.
Medicine A and Medicine B will reduce your risk of experiencing a fracture, such as hip fracture, within the next 5 years from **10%** to **6%**.

In other words, in a crowd of **1,000** people in the UK, **100** are likely to have a fracture if they do not take the medicine. This is reduced to **60** people out of 1,000 who do take the medicine.
This information is also illustrated in this picture.

Risk of fracture

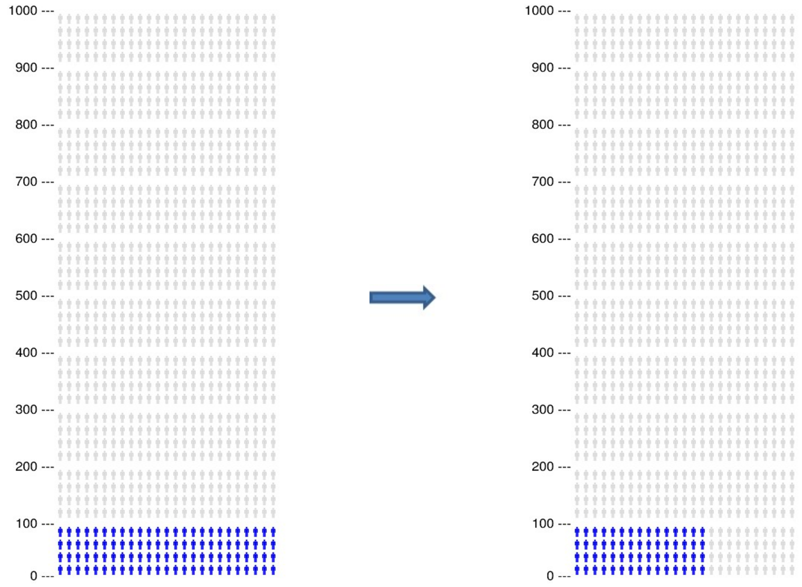

10%
No Treatment

6%
Taking the Medicine

(The figures shaded blue represent people who will have a fracture, such as hip fracture, within the next 5 years)

In this scenario, there is some risk of a severe side effect from Medicine A or Medicine B.

Among every 1,000 people taking Medicine A or Medicine B, **3** people are likely to have a severe side effect such as severe loss or destruction of the bone in the jaw.

Risk of a severe side effect

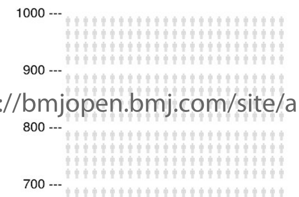

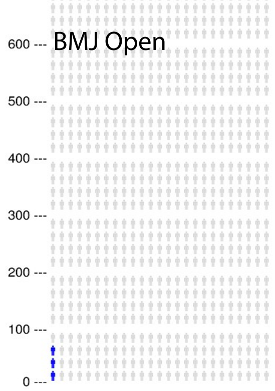

0.3% of people will have a severe side effect.

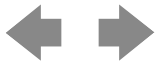

For peer review only

Repeated section

Remember Medicine A and Medicine B are equally effective and have some risk of a severe side effect.

The only difference between Medicine A and Medicine B is how you take them.

Medicine A	Medicine B
You take a tablet only once in your life. You will be given the tablet on the day you come in to see your GP. 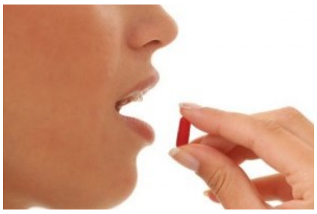	One tablet should be taken once a week on the same day every week.<br>You need to take this medicine for 5 years.<br><br>You need to take the tablet first thing in the morning on an empty stomach with a large glass of tap water.<br>After taking the tablet, you should stay upright for the next 30 minutes.<br>You should not eat anything for at least 30 minutes, including taking any other medicines.<br><br>You need to regularly visit your doctor to have your bone health checked.<br>You need to regularly visit a pharmacy for a new supply. 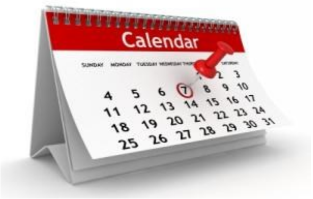 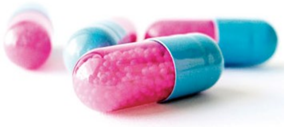

If you take Medicine A, you would live for **2.5 years (2 years and 6 months)** and then die.

If you take Medicine B, you would live for **5 years** and then die.

Would you prefer Medicine A or Medicine B, or are they the same?

Indicate your choice here. You can only choose one option:

◯ **Medicine A** Live for 2.5 years

◯ **Medicine B** Live for 5 years

◯ **The Same - Medicine A and Medicine B are the same.**

0% 100%

For peer review only

Repeated section

Remember Medicine A and Medicine B are equally effective and have some risk of a severe side effect.

The only difference between Medicine A and Medicine B is how you take them.

Medicine A	Medicine B
You take a tablet only once in your life. You will be given the tablet on the day you come in to see your GP.<br>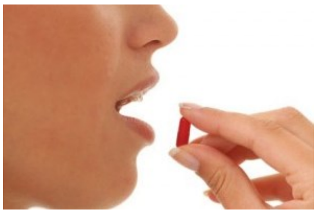	One tablet should be taken once a week on the same day every week.<br>You need to take this medicine for 5 years.<br><br>You need to take the tablet first thing in the morning on an empty stomach with a large glass of tap water.<br>After taking the tablet, you should stay upright for the next 30 minutes.<br>You should not eat anything for at least 30 minutes, including taking any other medicines.<br><br>You need to regularly visit your doctor to have your bone health checked.<br>You need to regularly visit a pharmacy for a new supply.<br>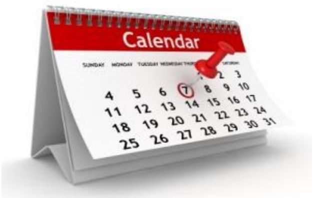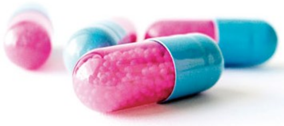

If you take Medicine A, you would live for **3 years** and then die.

If you take Medicine B, you would live for **5 years** and then die.

Would you prefer Medicine A or Medicine B, or are they the same?

Indicate your choice here. You can only choose one option:

○ **Medicine A**

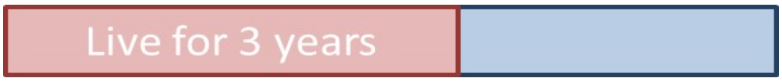

○ **Medicine B**

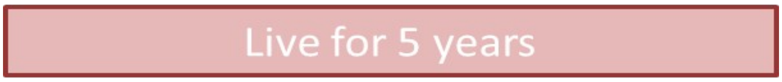

○ **The Same - Medicine A and Medicine B are the same.**

0% 100%

For peer review only

Repeated section

Remember Medicine A and Medicine B are equally effective and have some risk of a severe side effect.

The only difference between Medicine A and Medicine B is how you take them.

Medicine A	Medicine B
You take a tablet only once in your life. You will be given the tablet on the day you come in to see your GP. 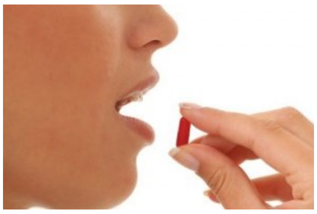	One tablet should be taken once a week on the same day every week.<br>You need to take this medicine for 5 years.<br><br>You need to take the tablet first thing in the morning on an empty stomach with a large glass of tap water.<br>After taking the tablet, you should stay upright for the next 30 minutes.<br>You should not eat anything for at least 30 minutes, including taking any other medicines.<br><br>You need to regularly visit your doctor to have your bone health checked.<br>You need to regularly visit a pharmacy for a new supply.<br>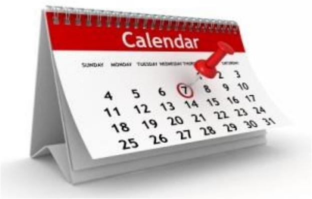 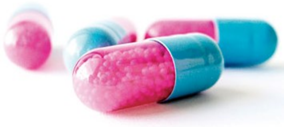

If you take Medicine A, you would live for **3.25 years** (3 years and 3 months) and then die.

If you take Medicine B, you would live for **5 years** and then die.

Would you prefer Medicine A or Medicine B, or are they the same?

Indicate your choice here. You can only choose one option:

◯ **Medicine A** Live for 3 years 3 months

◯ **Medicine B** Live for 5 years

◯ **The Same - Medicine A and Medicine B are the same.**

0% 100%

For peer review only

Repeated section

Remember Medicine A and Medicine B are equally effective and have some risk of a severe side effect.

The only difference between Medicine A and Medicine B is how you take them.

Medicine A	Medicine B
You take a tablet only once in your life. You will be given the tablet on the day you come in to see your GP. 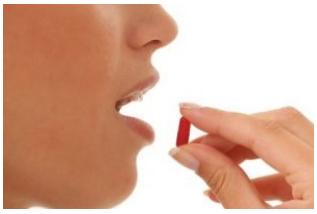	One tablet should be taken once a week on the same day every week.<br>You need to take this medicine for 5 years.<br><br>You need to take the tablet first thing in the morning on an empty stomach with a large glass of tap water.<br>After taking the tablet, you should stay upright for the next 30 minutes.<br>You should not eat anything for at least 30 minutes, including taking any other medicines.<br><br>You need to regularly visit your doctor to have your bone health checked.<br>You need to regularly visit a pharmacy for a new supply.<br>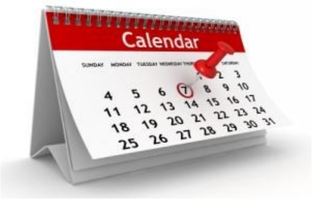 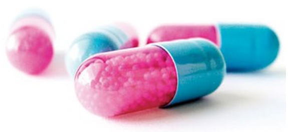

If you take Medicine A, you would live for **3.5 years** (3 years and 6 months) and then die.

If you take Medicine B, you would live for **5 years** and then die.

Would you prefer Medicine A or Medicine B, or are they the same?

Indicate your choice here. You can only choose one option:

◯ **Medicine A** Live for 3 years 6 months

◯ **Medicine B** Live for 5 years

◯ **The Same - Medicine A and Medicine B are the same.**

0% 100%

For peer review only

Repeated section

Remember Medicine A and Medicine B are equally effective and have no risks of a side effect.

The only difference between Medicine A and Medicine B is how you take them.

**Medicine A**	**Medicine B**
You take a tablet only once in your life. You will be given the tablet on the day you come in to see your GP. 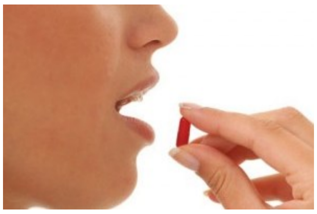	One tablet should be taken once a week on the same day every week.<br>You need to take this medicine for 5 years.<br><br>You need to take the tablet first thing in the morning on an empty stomach with a large glass of tap water.<br>After taking the tablet, you should stay upright for the next 30 minutes.<br>You should not eat anything for at least 30 minutes, including taking any other medicines.<br><br>You need to regularly visit your doctor to have your bone health checked.<br>You need to regularly visit a pharmacy for a new supply.<br>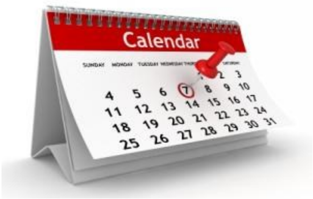 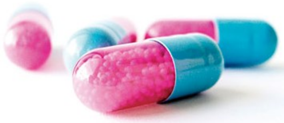

If you take Medicine A, you would live for **3.75 years** (3 years and 9 months) and then die.

If you take Medicine B, you would live for **5 years** and then die.

Would you prefer Medicine A or Medicine B, or are they the same?

Indicate your choice here. You can only choose one option:

○ **Medicine A** Live for 3 years 9 months

○ **Medicine B** Live for 5 years

○ **The Same - Medicine A and Medicine B are the same.**

0% 100%

For peer review only

Repeated section

Remember Medicine A and Medicine B are equally effective and have some risk of a severe side effect.

The only difference between Medicine A and Medicine B is how you take them.

Medicine A	Medicine B
You take a tablet only once in your life. You will be given the tablet on the day you come in to see your GP. 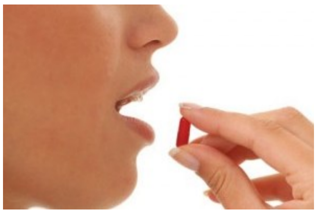	One tablet should be taken once a week on the same day every week. You need to take this medicine for 5 years.<br><br>You need to take the tablet first thing in the morning on an empty stomach with a large glass of tap water. After taking the tablet, you should stay upright for the next 30 minutes. You should not eat anything for at least 30 minutes, including taking any other medicines.<br><br>You need to regularly visit your doctor to have your bone health checked. You need to regularly visit a pharmacy for a new supply. 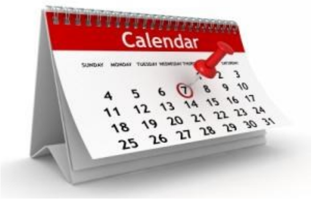 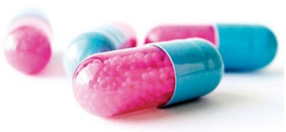

If you take Medicine A, you would live for **3 years and 10 months** and then die.

If you take Medicine B, you would live for **5 years** and then die.

Would you prefer Medicine A or Medicine B, or are they the same?

Indicate your choice here. You can only choose one option:

◯ **Medicine A** Live for 3 years 10 months

◯ **Medicine B** Live for 5 years

◯ **The Same - Medicine A and Medicine B are the same.**

0% 100%

For peer review only

Repeated section

Remember Medicine A and Medicine B are equally effective and have some risk of a severe side effect.

The only difference between Medicine A and Medicine B is how you take them.

Medicine A	Medicine B
You take a tablet only once in your life. You will be given the tablet on the day you come in to see your GP. 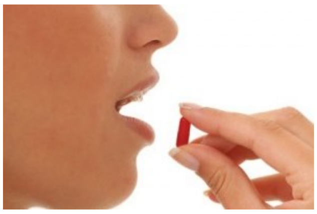	One tablet should be taken once a week on the same day every week. You need to take this medicine for 5 years.<br><br>You need to take the tablet first thing in the morning on an empty stomach with a large glass of tap water. After taking the tablet, you should stay upright for the next 30 minutes. You should not eat anything for at least 30 minutes, including taking any other medicines.<br><br>You need to regularly visit your doctor to have your bone health checked. You need to regularly visit a pharmacy for a new supply. 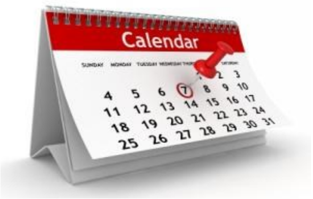 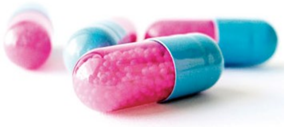

If you take Medicine A, you would live for **4 years** and then die.

If you take Medicine B, you would live for **5 years** and then die.

Would you prefer Medicine A or Medicine B, or are they the same?

Indicate your choice here. You can only choose one option:

◯ **Medicine A** Live for 4 years

◯ **Medicine B** Live for 5 years

◯ **The Same - Medicine A and Medicine B are the same.**

0% 100%

For peer review only

Repeated section

Remember Medicine A and Medicine B are equally effective and have some risk of a severe side effect.

The only difference between Medicine A and Medicine B is how you take them.

Medicine A	Medicine B
You take a tablet only once in your life.<br>You will be given the tablet on the day you come in to see your GP.<br>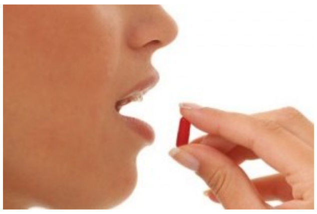	One tablet should be taken once a week on the same day every week.<br>You need to take this medicine for 5 years.<br><br>You need to take the tablet first thing in the morning on an empty stomach with a large glass of tap water.<br>After taking the tablet, you should stay upright for the next 30 minutes.<br>You should not eat anything for at least 30 minutes, including taking any other medicines.<br><br>You need to regularly visit your doctor to have your bone health checked.<br>You need to regularly visit a pharmacy for a new supply.<br>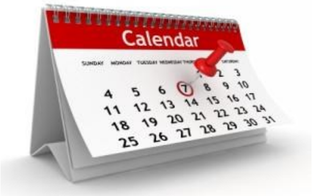 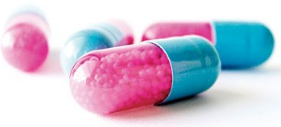

If you take Medicine A, you would live for **4 years and 2 months** and then die.

If you take Medicine B, you would live for **5 years** and then die.

Would you prefer Medicine A or Medicine B, or are they the same?

Indicate your choice here. You can only choose one option:

◯ **Medicine A** Live for 4 years 2 months

◯ **Medicine B** Live for 5 years

◯ **The Same - Medicine A and Medicine B are the same.**

0% 100%

For peer review only

Repeated section

Remember Medicine A and Medicine B are equally effective and have some risk of a severe side effect.

The only difference between Medicine A and Medicine B is how you take them.

**Medicine A**	**Medicine B**
You take a tablet only once in your life.<br>You will be given the tablet on the day you come in to see your GP.<br>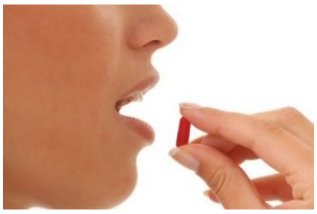	One tablet should be taken once a week on the same day every week.<br>You need to take this medicine for 5 years.<br><br>You need to take the tablet first thing in the morning on an empty stomach with a large glass of tap water.<br>After taking the tablet, you should stay upright for the next 30 minutes.<br>You should not eat anything for at least 30 minutes, including taking any other medicines.<br><br>You need to regularly visit your doctor to have your bone health checked.<br>You need to regularly visit a pharmacy for a new supply.<br>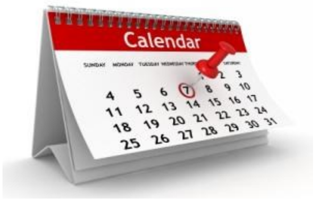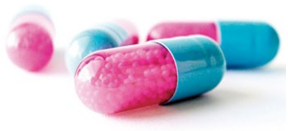

If you take Medicine A, you would live for **4.25 years** (4 years and 3 months) and then die.

If you take Medicine B, you would live for **5 years** and then die.

Would you prefer Medicine A or Medicine B, or are they the same?

Indicate your choice here. You can only choose one option:

◯ **Medicine A** Live for 4 years 3 months

◯ **Medicine B** Live for 5 years

◯ **The Same - Medicine A and Medicine B are the same.**

0% 100%

For peer review only

Repeated section

Remember Medicine A and Medicine B are equally effective and have some risk of a severe side effect.

The only difference between Medicine A and Medicine B is how you take them.

**Medicine A**	**Medicine B**
You take a tablet only once in your life.<br>You will be given the tablet on the day you come in to see your GP.<br>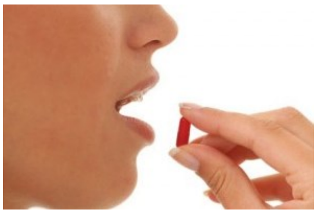	One tablet should be taken once a week on the same day every week.<br>You need to take this medicine for 5 years.<br><br>You need to take the tablet first thing in the morning on an empty stomach with a large glass of tap water.<br>After taking the tablet, you should stay upright for the next 30 minutes.<br>You should not eat anything for at least 30 minutes, including taking any other medicines.<br><br>You need to regularly visit your doctor to have your bone health checked.<br>You need to regularly visit a pharmacy for a new supply.<br>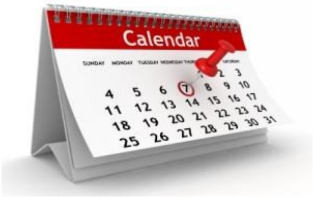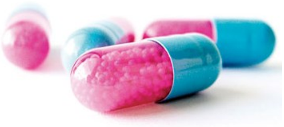

If you take Medicine A, you would live for **4 years 4 months** and then die.

If you take Medicine B, you would live for **5 years** and then die.

Would you prefer Medicine A or Medicine B, or are they the same?

Indicate your choice here. You can only choose one option:

◯ **Medicine A** Live for 4 years 4 months

◯ **Medicine B** Live for 5 years

◯ **The Same - Medicine A and Medicine B are the same.**

0% 100%

For peer review only

Repeated section

Remember Medicine A and Medicine B are equally effective and have some risk of a severe side effect.

The only difference between Medicine A and Medicine B is how you take them.

Medicine A	Medicine B
You take a tablet only once in your life. You will be given the tablet on the day you come in to see your GP. 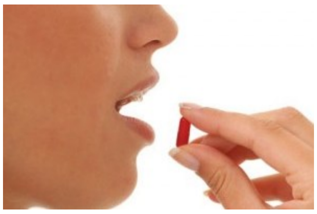	One tablet should be taken once a week on the same day every week.<br>You need to take this medicine for 5 years.<br><br>You need to take the tablet first thing in the morning on an empty stomach with a large glass of tap water.<br>After taking the tablet, you should stay upright for the next 30 minutes.<br>You should not eat anything for at least 30 minutes, including taking any other medicines.<br><br>You need to regularly visit your doctor to have your bone health checked.<br>You need to regularly visit a pharmacy for a new supply. 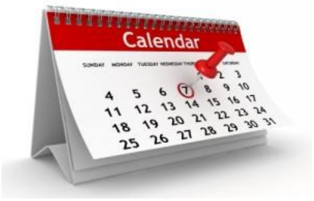 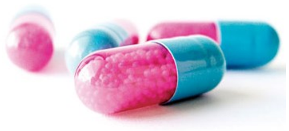

If you take Medicine A, you would live for **4.5 years** (4 years and 6 months) and then die.

If you take Medicine B, you would live for **5 years** and then die.

Would you prefer Medicine A or Medicine B, or are they the same?

Indicate your choice here. You can only choose one option:

◯ **Medicine A** Live for 4 years 6 months

◯ **Medicine B** Live for 5 years

◯ **The Same - Medicine A and Medicine B are the same.**

0% 100%

For peer review only

Repeated section

Remember Medicine A and Medicine B are equally effective and have some risk of a severe side effect.

The only difference between Medicine A and Medicine B is how you take them.

Medicine A	Medicine B
You take a tablet only once in your life. You will be given the tablet on the day you come in to see your GP. 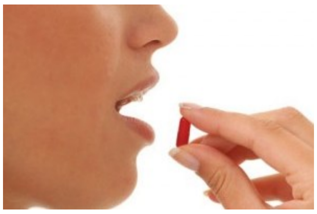	One tablet should be taken once a week on the same day every week. You need to take this medicine for 5 years.<br><br>You need to take the tablet first thing in the morning on an empty stomach with a large glass of tap water. After taking the tablet, you should stay upright for the next 30 minutes. You should not eat anything for at least 30 minutes, including taking any other medicines.<br><br>You need to regularly visit your doctor to have your bone health checked. You need to regularly visit a pharmacy for a new supply. 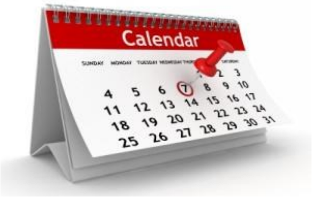 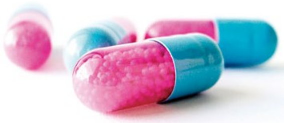

If you take Medicine A, you would live for **4 years and 7 months** and then die.

If you take Medicine B, you would live for **5 years** and then die.

Would you prefer Medicine A or Medicine B, or are they the same?

Indicate your choice here. You can only choose one option:

◯ **Medicine A** Live for 4 years 7 months

◯ **Medicine B** Live for 5 years

◯ **The Same - Medicine A and Medicine B are the same.**

For peer review only

Repeated section

Remember Medicine A and Medicine B are equally effective and have some risk of a severe side effect.

The only difference between Medicine A and Medicine B is how you take them.

Medicine A	Medicine B
You take a tablet only once in your life. You will be given the tablet on the day you come in to see your GP.<br>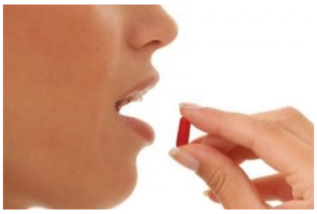	One tablet should be taken once a week on the same day every week.<br>You need to take this medicine for 5 years.<br><br>You need to take the tablet first thing in the morning on an empty stomach with a large glass of tap water.<br>After taking the tablet, you should stay upright for the next 30 minutes.<br>You should not eat anything for at least 30 minutes, including taking any other medicines.<br><br>You need to regularly visit your doctor to have your bone health checked.<br>You need to regularly visit a pharmacy for a new supply.<br>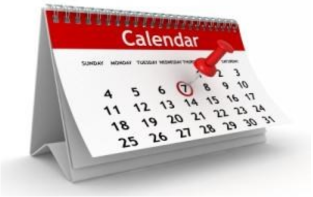 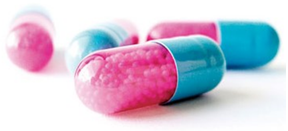

If you take Medicine A, you would live for **4 years and 9 months** and then die.

If you take Medicine B, you would live for **5 years** and then die.

Would you prefer Medicine A or Medicine B, or are they the same?

Indicate your choice here. You can only choose one option:

○ **Medicine A** — Live for 4 years 9 months

○ **Medicine B** — Live for 5 years

○ **The Same - Medicine A and Medicine B are the same.**

0% 100%

For peer review only

Repeated section

Remember Medicine A and Medicine B are equally effective and have some risk of a severe side effect.

The only difference between Medicine A and Medicine B is how you take them.

**Medicine A**	**Medicine B**
You take a tablet only once in your life.<br>You will be given the tablet on the day you come in to see your GP.<br>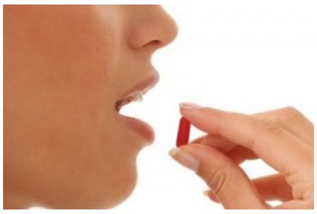	One tablet should be taken once a week on the same day every week.<br>You need to take this medicine for 5 years.<br><br>You need to take the tablet first thing in the morning on an empty stomach with a large glass of tap water.<br>After taking the tablet, you should stay upright for the next 30 minutes.<br>You should not eat anything for at least 30 minutes, including taking any other medicines.<br><br>You need to regularly visit your doctor to have your bone health checked.<br>You need to regularly visit a pharmacy for a new supply.<br>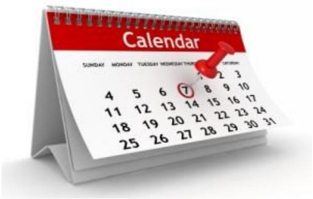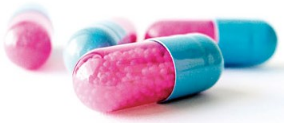

If you take Medicine A, you would live for **2 months less than 5 years** and then die.

If you take Medicine B, you would live for **5 years** and then die.

Would you prefer Medicine A or Medicine B, or are they the same?

Indicate your choice here. You can only choose one option:

◯ **Medicine A** Live for (5 years – 2 months)

◯ **Medicine B** Live for 5 years

◯ **The Same - Medicine A and Medicine B are the same.**

0% 100%

For peer review only

Repeated section

Remember Medicine A and Medicine B are equally effective and have some risk of a severe side effect.

The only difference between Medicine A and Medicine B is how you take them.

Medicine A	Medicine B
You take a tablet only once in your life.<br>You will be given the tablet on the day you come in to see your GP.<br>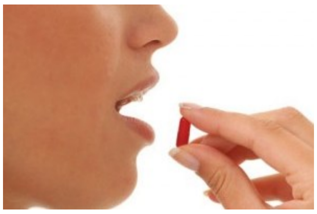	One tablet should be taken once a week on the same day every week.<br>You need to take this medicine for 5 years.<br><br>You need to take the tablet first thing in the morning on an empty stomach with a large glass of tap water.<br>After taking the tablet, you should stay upright for the next 30 minutes.<br>You should not eat anything for at least 30 minutes, including taking any other medicines.<br><br>You need to regularly visit your doctor to have your bone health checked.<br>You need to regularly visit a pharmacy for a new supply.<br>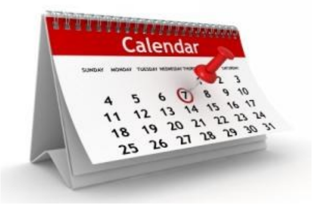 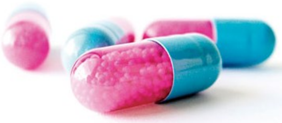

If you take Medicine A, you would live for **1 month less than 5 years** and then die.

If you take Medicine B, you would live for **5 years** and then die.

Would you prefer Medicine A or Medicine B, or are they the same?

Indicate your choice here. You can only choose one option:

◯ **Medicine A** Live for (5 years – 1 month)

◯ **Medicine B** Live for 5 years

◯ **The Same - Medicine A and Medicine B are the same.**

0% 100%

For peer review only

Repeated section

Remember Medicine A and Medicine B are equally effective and have some risk of a severe side effect.

The only difference between Medicine A and Medicine B is how you take them.

**Medicine A**	**Medicine B**
You take a tablet only once in your life. You will be given the tablet on the day you come in to see your GP. 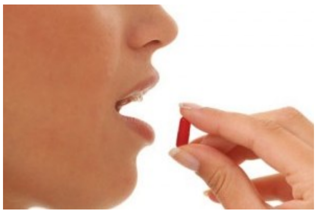	One tablet should be taken once a week on the same day every week. You need to take this medicine for 5 years.<br><br>You need to take the tablet first thing in the morning on an empty stomach with a large glass of tap water. After taking the tablet, you should stay upright for the next 30 minutes. You should not eat anything for at least 30 minutes, including taking any other medicines.<br><br>You need to regularly visit your doctor to have your bone health checked. You need to regularly visit a pharmacy for a new supply. 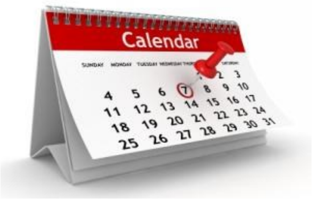 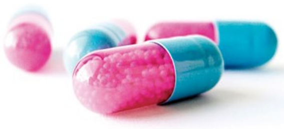

If you take Medicine A, you would live for **2 weeks less than 5 years** and then die.

If you take Medicine B, you would live for **5 years** and then die.

Would you prefer Medicine A or Medicine B, or are they the same?

Indicate your choice here. You can only choose one option:

○ **Medicine A** Live for (5 years – 2 weeks)

○ **Medicine B** Live for 5 years

○ **The Same - Medicine A and Medicine B are the same.**

0% 100%

For peer review only

Repeated section

Remember Medicine A and Medicine B are equally effective and have some risk of a severe side effect.

The only difference between Medicine A and Medicine B is how you take them.

Medicine A	Medicine B
You take a tablet only once in your life. You will be given the tablet on the day you come in to see your GP. 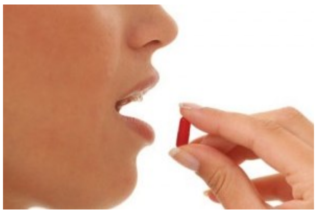	One tablet should be taken once a week on the same day every week. You need to take this medicine for 5 years. You need to take the tablet first thing in the morning on an empty stomach with a large glass of tap water. After taking the tablet, you should stay upright for the next 30 minutes. You should not eat anything for at least 30 minutes, including taking any other medicines. You need to regularly visit your doctor to have your bone health checked. You need to regularly visit a pharmacy for a new supply. 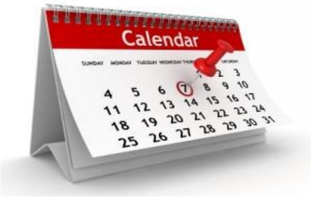 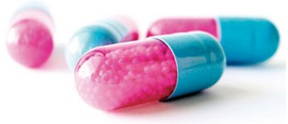

If you take Medicine A, you would live for **1 week less than 5 years** and then die.

If you take Medicine B, you would live for **5 years** and then die.

Would you prefer Medicine A or Medicine B, or are they the same?

Indicate your choice here. You can only choose one option:

◯ **Medicine A** Live for (5 years – 1 week)

◯ **Medicine B** Live for 5 years

◯ **The Same - Medicine A and Medicine B are the same.**

0% 100%

For peer review only

Repeated section

Remember Medicine A and Medicine B are equally effective and have some risk of a severe side effect.

The only difference between Medicine A and Medicine B is how you take them.

**Medicine A**	**Medicine B**
You take a tablet only once in your life. You will be given the tablet on the day you come in to see your GP. 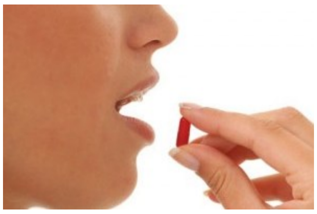	One tablet should be taken once a week on the same day every week. You need to take this medicine for 5 years.<br><br>You need to take the tablet first thing in the morning on an empty stomach with a large glass of tap water. After taking the tablet, you should stay upright for the next 30 minutes. You should not eat anything for at least 30 minutes, including taking any other medicines.<br><br>You need to regularly visit your doctor to have your bone health checked. You need to regularly visit a pharmacy for a new supply. 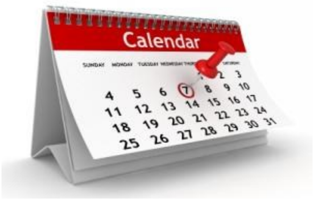 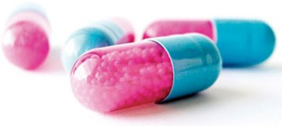

If you take Medicine A, you would live for **5 days less than 5 years** and then die.

If you take Medicine B, you would live for **5 years** and then die.

Would you prefer Medicine A or Medicine B, or are they the same?

Indicate your choice here. You can only choose one option:

◯ **Medicine A** Live for (5 years – 5 days)

◯ **Medicine B** Live for 5 years

◯ **The Same - Medicine A and Medicine B are the same.**

0% 100%

For peer review only

Repeated section

Remember Medicine A and Medicine B are equally effective and have some risk of a severe side effect.

The only difference between Medicine A and Medicine B is how you take them.

Medicine A	Medicine B
You take a tablet only once in your life. You will be given the tablet on the day you come in to see your GP. 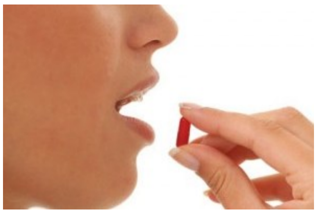	One tablet should be taken once a week on the same day every week.<br>You need to take this medicine for 5 years.<br><br>You need to take the tablet first thing in the morning on an empty stomach with a large glass of tap water.<br>After taking the tablet, you should stay upright for the next 30 minutes.<br>You should not eat anything for at least 30 minutes, including taking any other medicines.<br><br>You need to regularly visit your doctor to have your bone health checked.<br>You need to regularly visit a pharmacy for a new supply.<br>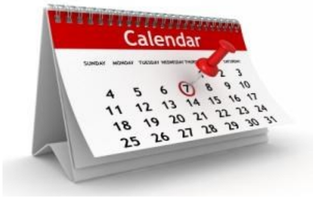 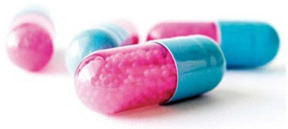

If you take Medicine A, you would live for **3 days less than 5 years** and then die.

If you take Medicine B, you would live for **5 years** and then die.

Would you prefer Medicine A or Medicine B, or are they the same?

Indicate your choice here. You can only choose one option:

◯ **Medicine A** Live for (5 years – 3 days)

◯ **Medicine B** Live for 5 years

◯ **The Same - Medicine A and Medicine B are the same.**

For peer review only

Repeated section

Remember Medicine A and Medicine B are equally effective and have some risk of a severe side effect.

The only difference between Medicine A and Medicine B is how you take them.

Medicine A	Medicine B
You take a tablet only once in your life.<br>You will be given the tablet on the day you come in to see your GP.<br>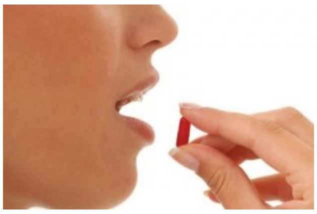	One tablet should be taken once a week on the same day every week.<br>You need to take this medicine for 5 years.<br><br>You need to take the tablet first thing in the morning on an empty stomach with a large glass of tap water.<br>After taking the tablet, you should stay upright for the next 30 minutes.<br>You should not eat anything for at least 30 minutes, including taking any other medicines.<br><br>You need to regularly visit your doctor to have your bone health checked.<br>You need to regularly visit a pharmacy for a new supply.<br>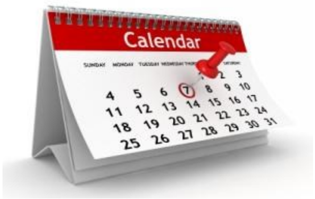 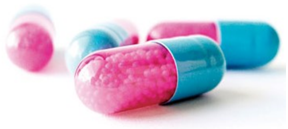

If you take Medicine A, you would live for **1 day less than 5 years** and then die.

If you take Medicine B, you would live for **5 years** and then die.

Would you prefer Medicine A or Medicine B, or are they the same?

Indicate your choice here. You can only choose one option:

◯ **Medicine A** Live for (5 years – 1 day)

◯ **Medicine B** Live for 5 years

◯ **The Same - Medicine A and Medicine B are the same.**

0% 100%

For peer review only

Question 3a.

You prefer living for 2.5 years (2 years and 6 months) on Medicine A, to living for 5 years on Medicine B.

This means **living fewer than 2.5 years on Medicine A** can be the same as living for 5 years on Medicine B.

How many years on Medicine A do you think would be the same as living for 5 years on Medicine B?

(Please give a number in years)

0% 100%

peer review only

Part 2

Question 4

Now, Medicine A and Medicine B will give you less benefit than before because you belong to a group of people who may not respond to the treatment as well as other groups of people.

As before,

Medicine A is a pill that you only need to take once.

Medicine B is a pill that you need to take every week for 5 years.

Medicine A and Medicine B are equally effective in reducing your risk of having a fracture.

Medicine A and Medicine B will reduce your risk of experiencing a fracture, such as hip fracture, within the next 5 years from **10%** to **9%**.

In other words, in a crowd of **1,000** people in the UK, **100** are likely to have a fracture if they do not take the medicine. This is reduced to **90** people out of 1,000 who do take the medicine. This information is also illustrated in this picture.

Risk of fracture

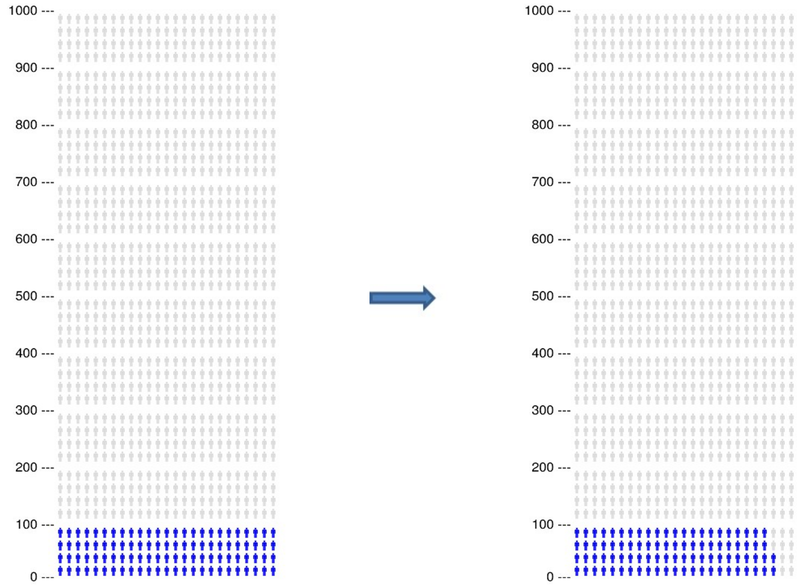

10%
No Treatment

9%
Taking the Medicine

(The figures shaded blue represent people who will have a fracture, such as hip fracture, within the next 5 years)

In this scenario, you will not have any side effects from Medicine A or Medicine B.

Risk of a side effect

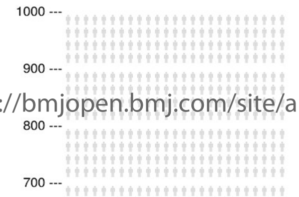

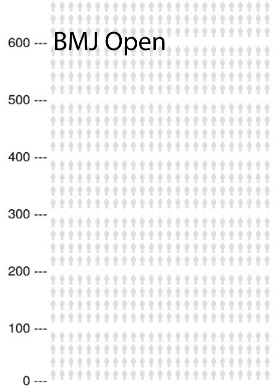

0 %

No one will have a side effect.

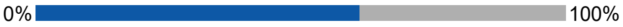

eer review only

Repeated section

Remember Medicine A and Medicine B are equally effective and have no risks of a side effect.

The only difference between Medicine A and Medicine B is how you take them.

Medicine A	Medicine B
You take a tablet only once in your life. You will be given the tablet on the day you come in to see your GP.<br>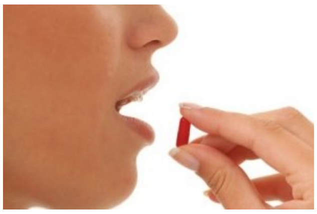	One tablet should be taken once a week on the same day every week.<br>You need to take this medicine for 5 years.<br><br>You need to take the tablet first thing in the morning on an empty stomach with a large glass of tap water.<br>After taking the tablet, you should stay upright for the next 30 minutes.<br>You should not eat anything for at least 30 minutes, including taking any other medicines.<br><br>You need to regularly visit your doctor to have your bone health checked.<br>You need to regularly visit a pharmacy for a new supply.<br>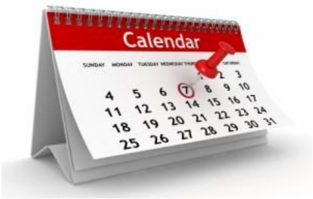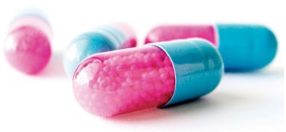

If you take Medicine A, you would live for **2.5 years (2 years and 6 months)** and then die.

If you take Medicine B, you would live for **5 years** and then die.

Would you prefer Medicine A or Medicine B, or are they the same?

Indicate your choice here. You can only choose one option:

○ **Medicine A** Live for 2.5 years

○ **Medicine B** Live for 5 years

○ **The Same - Medicine A and Medicine B are the same.**

0% 100%

For peer review only

Repeated section

Remember Medicine A and Medicine B are equally effective and have no risks of a side effect.

The only difference between Medicine A and Medicine B is how you take them.

Medicine A	Medicine B
You take a tablet only once in your life. You will be given the tablet on the day you come in to see your GP.<br>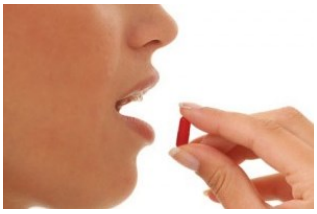	One tablet should be taken once a week on the same day every week.<br>You need to take this medicine for 5 years.<br><br>You need to take the tablet first thing in the morning on an empty stomach with a large glass of tap water.<br>After taking the tablet, you should stay upright for the next 30 minutes.<br>You should not eat anything for at least 30 minutes, including taking any other medicines.<br><br>You need to regularly visit your doctor to have your bone health checked.<br>You need to regularly visit a pharmacy for a new supply.<br>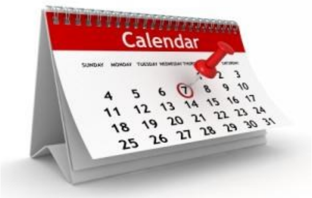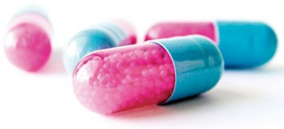

If you take Medicine A, you would live for **3 years** and then die.

If you take Medicine B, you would live for **5 years** and then die.

Would you prefer Medicine A or Medicine B, or are they the same?

Indicate your choice here. You can only choose one option:

◯ **Medicine A** Live for 3 years

◯ **Medicine B** Live for 5 years

◯ **The Same - Medicine A and Medicine B are the same.**

0% 100%

For peer review only

Repeated section

Remember Medicine A and Medicine B are equally effective and have no risks of a side effect.

The only difference between Medicine A and Medicine B is how you take them.

**Medicine A**	**Medicine B**
You take a tablet only once in your life.<br>You will be given the tablet on the day you come in to see your GP.<br>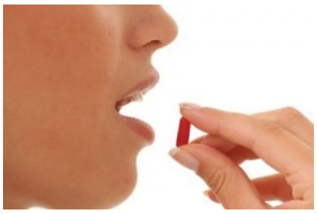	One tablet should be taken once a week on the same day every week.<br>You need to take this medicine for 5 years.<br><br>You need to take the tablet first thing in the morning on an empty stomach with a large glass of tap water.<br>After taking the tablet, you should stay upright for the next 30 minutes.<br>You should not eat anything for at least 30 minutes, including taking any other medicines.<br><br>You need to regularly visit your doctor to have your bone health checked.<br>You need to regularly visit a pharmacy for a new supply.<br>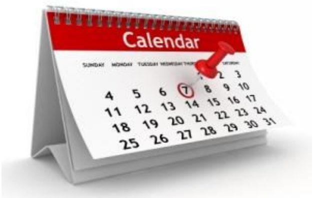 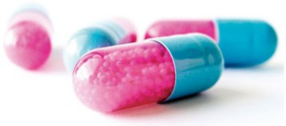

If you take Medicine A, you would live for **3.25 years** (3 years and 3 months) and then die.

If you take Medicine B, you would live for **5 years** and then die.

Would you prefer Medicine A or Medicine B, or are they the same?

Indicate your choice here. You can only choose one option:

◯ **Medicine A**

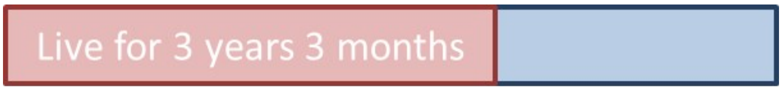

◯ **Medicine B**

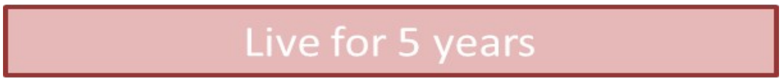

◯ **The Same - Medicine A and Medicine B are the same.**

For peer review only

Repeated section

Remember Medicine A and Medicine B are equally effective and have no risks of a side effect.

The only difference between Medicine A and Medicine B is how you take them.

Medicine A	Medicine B
You take a tablet only once in your life. You will be given the tablet on the day you come in to see your GP. 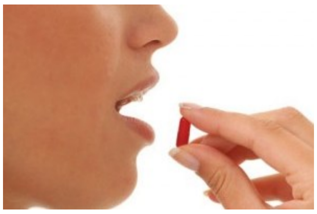	One tablet should be taken once a week on the same day every week. You need to take this medicine for 5 years.<br><br>You need to take the tablet first thing in the morning on an empty stomach with a large glass of tap water. After taking the tablet, you should stay upright for the next 30 minutes. You should not eat anything for at least 30 minutes, including taking any other medicines.<br><br>You need to regularly visit your doctor to have your bone health checked. You need to regularly visit a pharmacy for a new supply. 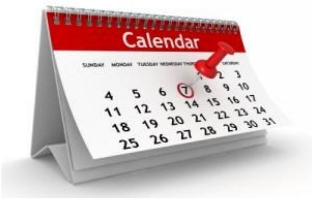 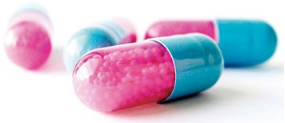

If you take Medicine A, you would live for **3.5 years** (3 years and 6 months) and then die.

If you take Medicine B, you would live for **5 years** and then die.

Would you prefer Medicine A or Medicine B, or are they the same?

Indicate your choice here. You can only choose one option:

◯ **Medicine A** Live for 3 years 6 months

◯ **Medicine B** Live for 5 years

◯ **The Same - Medicine A and Medicine B are the same.**

0% 100%

For peer review only

Repeated section

Remember Medicine A and Medicine B are equally effective and have no risks of a side effect.

The only difference between Medicine A and Medicine B is how you take them.

Medicine A	Medicine B
You take a tablet only once in your life.<br>You will be given the tablet on the day you come in to see your GP.<br>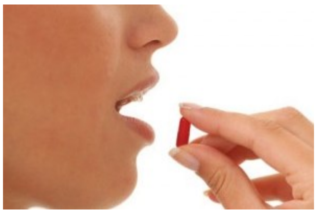	One tablet should be taken once a week on the same day every week.<br>You need to take this medicine for 5 years.<br><br>You need to take the tablet first thing in the morning on an empty stomach with a large glass of tap water.<br>After taking the tablet, you should stay upright for the next 30 minutes.<br>You should not eat anything for at least 30 minutes, including taking any other medicines.<br><br>You need to regularly visit your doctor to have your bone health checked.<br>You need to regularly visit a pharmacy for a new supply.<br>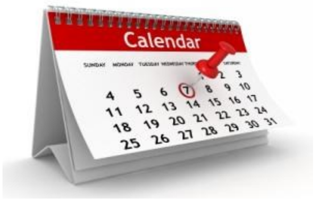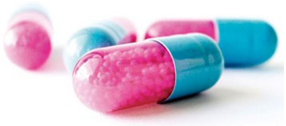

If you take Medicine A, you would live for **3.75 years** (3 years and 9 months) and then die.

If you take Medicine B, you would live for **5 years** and then die.

Would you prefer Medicine A or Medicine B, or are they the same?

Indicate your choice here. You can only choose one option:

◯ **Medicine A** Live for 3 years 9 months

◯ **Medicine B** Live for 5 years

◯ **The Same - Medicine A and Medicine B are the same.**

For peer review only

Repeated section

Remember Medicine A and Medicine B are equally effective and have no risks of a side effect.

The only difference between Medicine A and Medicine B is how you take them.

Medicine A	Medicine B
You take a tablet only once in your life.<br>You will be given the tablet on the day you come in to see your GP.<br>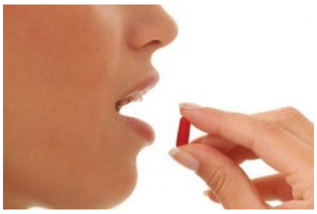	One tablet should be taken once a week on the same day every week.<br>You need to take this medicine for 5 years.<br><br>You need to take the tablet first thing in the morning on an empty stomach with a large glass of tap water.<br>After taking the tablet, you should stay upright for the next 30 minutes.<br>You should not eat anything for at least 30 minutes, including taking any other medicines.<br><br>You need to regularly visit your doctor to have your bone health checked.<br>You need to regularly visit a pharmacy for a new supply.<br>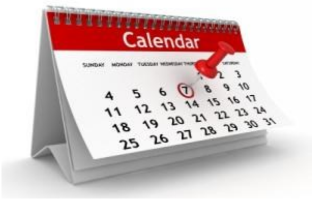 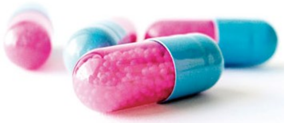

If you take Medicine A, you would live for **3 years and 10 months** and then die.

If you take Medicine B, you would live for **5 years** and then die.

Would you prefer Medicine A or Medicine B, or are they the same?

Indicate your choice here. You can only choose one option:

○ **Medicine A** Live for 3 years 10 months

○ **Medicine B** Live for 5 years

○ **The Same - Medicine A and Medicine B are the same.**

0% 100%

For peer review only

Repeated section

Remember Medicine A and Medicine B are equally effective and have no risks of a side effect.

The only difference between Medicine A and Medicine B is how you take them.

Medicine A	Medicine B
You take a tablet only once in your life. You will be given the tablet on the day you come in to see your GP. 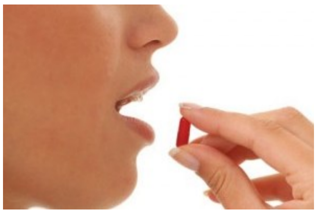	One tablet should be taken once a week on the same day every week. You need to take this medicine for 5 years. <br>You need to take the tablet first thing in the morning on an empty stomach with a large glass of tap water. After taking the tablet, you should stay upright for the next 30 minutes. You should not eat anything for at least 30 minutes, including taking any other medicines. <br>You need to regularly visit your doctor to have your bone health checked. You need to regularly visit a pharmacy for a new supply. 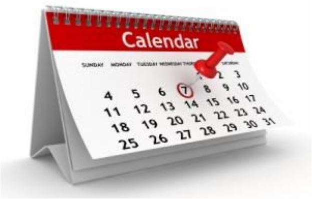 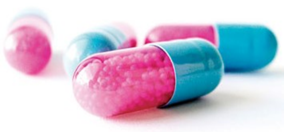

If you take Medicine A, you would live for **4 years** and then die.

If you take Medicine B, you would live for **5 years** and then die.

Would you prefer Medicine A or Medicine B, or are they the same?

Indicate your choice here. You can only choose one option:

◯ **Medicine A** Live for 4 years

◯ **Medicine B** Live for 5 years

◯ **The Same - Medicine A and Medicine B are the same.**

0% 100%

For peer review only

Repeated section

Remember Medicine A and Medicine B are equally effective and have no risks of a side effect.

The only difference between Medicine A and Medicine B is how you take them.

Medicine A	Medicine B
You take a tablet only once in your life. You will be given the tablet on the day you come in to see your GP. 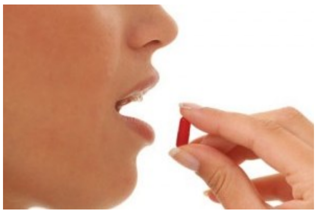	One tablet should be taken once a week on the same day every week.<br>You need to take this medicine for 5 years.<br><br>You need to take the tablet first thing in the morning on an empty stomach with a large glass of tap water.<br>After taking the tablet, you should stay upright for the next 30 minutes.<br>You should not eat anything for at least 30 minutes, including taking any other medicines.<br><br>You need to regularly visit your doctor to have your bone health checked.<br>You need to regularly visit a pharmacy for a new supply.<br>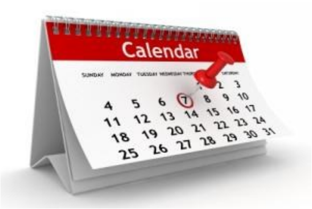 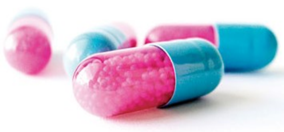

If you take Medicine A, you would live for **4 years and 2 months** and then die.

If you take Medicine B, you would live for **5 years** and then die.

Would you prefer Medicine A or Medicine B, or are they the same?

Indicate your choice here. You can only choose one option:

◯ **Medicine A** Live for 4 years 2 months

◯ **Medicine B** Live for 5 years

◯ **The Same - Medicine A and Medicine B are the same.**

0% 100%

For peer review only

Repeated section

Remember Medicine A and Medicine B are equally effective and have no risks of a side effect.

The only difference between Medicine A and Medicine B is how you take them.

**Medicine A**	**Medicine B**
You take a tablet only once in your life.<br>You will be given the tablet on the day you come in to see your GP.<br>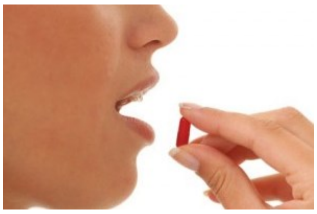	One tablet should be taken once a week on the same day every week.<br>You need to take this medicine for 5 years.<br><br>You need to take the tablet first thing in the morning on an empty stomach with a large glass of tap water.<br>After taking the tablet, you should stay upright for the next 30 minutes.<br>You should not eat anything for at least 30 minutes, including taking any other medicines.<br><br>You need to regularly visit your doctor to have your bone health checked.<br>You need to regularly visit a pharmacy for a new supply.<br>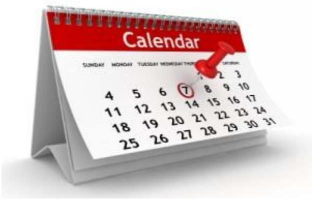 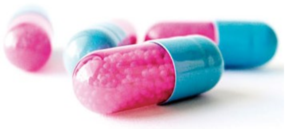

If you take Medicine A, you would live for **4.25 years** (4 years and 3 months) and then die.

If you take Medicine B, you would live for **5 years** and then die.

Would you prefer Medicine A or Medicine B, or are they the same?

Indicate your choice here. You can only choose one option:

◯ **Medicine A** Live for 4 years 3 months

◯ **Medicine B** Live for 5 years

◯ **The Same - Medicine A and Medicine B are the same.**

0% 100%

For peer review only

Repeated section

Remember Medicine A and Medicine B are equally effective and have no risks of a side effect.

The only difference between Medicine A and Medicine B is how you take them.

Medicine A	Medicine B
You take a tablet only once in your life.<br>You will be given the tablet on the day you come in to see your GP.<br>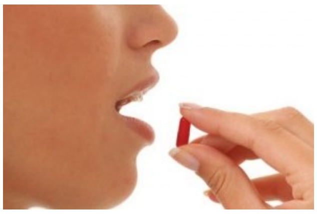	One tablet should be taken once a week on the same day every week.<br>You need to take this medicine for 5 years.<br><br>You need to take the tablet first thing in the morning on an empty stomach with a large glass of tap water.<br>After taking the tablet, you should stay upright for the next 30 minutes.<br>You should not eat anything for at least 30 minutes, including taking any other medicines.<br><br>You need to regularly visit your doctor to have your bone health checked.<br>You need to regularly visit a pharmacy for a new supply.<br>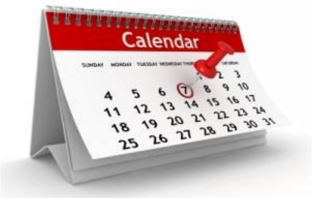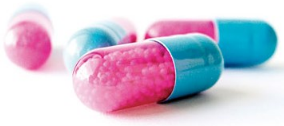

If you take Medicine A, you would live for **4 years 4 months** and then die.

If you take Medicine B, you would live for **5 years** and then die.

Would you prefer Medicine A or Medicine B, or are they the same?

Indicate your choice here. You can only choose one option:

◯ **Medicine A** Live for 4 years 4 months

◯ **Medicine B** Live for 5 years

◯ **The Same - Medicine A and Medicine B are the same.**

0% 100%

For peer review only

Repeated section

Remember Medicine A and Medicine B are equally effective and have no risks of a side effect.

The only difference between Medicine A and Medicine B is how you take them.

Medicine A	Medicine B
You take a tablet only once in your life. You will be given the tablet on the day you come in to see your GP.<br>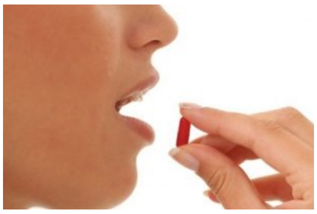	One tablet should be taken once a week on the same day every week.<br>You need to take this medicine for 5 years.<br><br>You need to take the tablet first thing in the morning on an empty stomach with a large glass of tap water.<br>After taking the tablet, you should stay upright for the next 30 minutes.<br>You should not eat anything for at least 30 minutes, including taking any other medicines.<br><br>You need to regularly visit your doctor to have your bone health checked.<br>You need to regularly visit a pharmacy for a new supply.<br>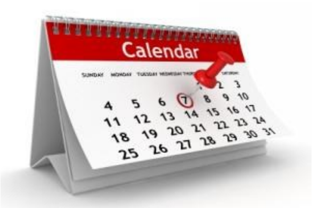 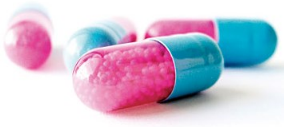

If you take Medicine A, you would live for **4.5 years** (4 years and 6 months) and then die.

If you take Medicine B, you would live for **5 years** and then die.

Would you prefer Medicine A or Medicine B, or are they the same?

Indicate your choice here. You can only choose one option:

◯ **Medicine A** Live for 4 years 6 months

◯ **Medicine B** Live for 5 years

◯ **The Same - Medicine A and Medicine B are the same.**

0% 100%

For peer review only

Repeated section

Remember Medicine A and Medicine B are equally effective and have no risks of a side effect.

The only difference between Medicine A and Medicine B is how you take them.

Medicine A	Medicine B
You take a tablet only once in your life. You will be given the tablet on the day you come in to see your GP.<br>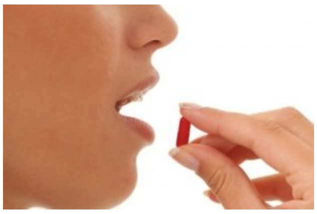	One tablet should be taken once a week on the same day every week.<br>You need to take this medicine for 5 years.<br><br>You need to take the tablet first thing in the morning on an empty stomach with a large glass of tap water.<br>After taking the tablet, you should stay upright for the next 30 minutes.<br>You should not eat anything for at least 30 minutes, including taking any other medicines.<br><br>You need to regularly visit your doctor to have your bone health checked.<br>You need to regularly visit a pharmacy for a new supply.<br>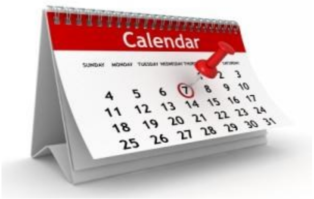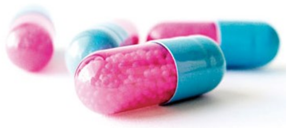

If you take Medicine A, you would live for **4 years and 7 months** and then die.

If you take Medicine B, you would live for **5 years** and then die.

Would you prefer Medicine A or Medicine B, or are they the same?

Indicate your choice here. You can only choose one option:

◯ **Medicine A** Live for 4 years 7 months

◯ **Medicine B** Live for 5 years

◯ **The Same - Medicine A and Medicine B are the same.**

0% 100%

For peer review only

Repeated section

Remember Medicine A and Medicine B are equally effective and have no risks of a side effect.

The only difference between Medicine A and Medicine B is how you take them.

Medicine A	Medicine B
You take a tablet only once in your life. You will be given the tablet on the day you come in to see your GP. 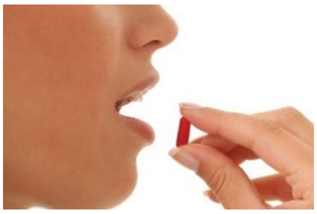	One tablet should be taken once a week on the same day every week. You need to take this medicine for 5 years. You need to take the tablet first thing in the morning on an empty stomach with a large glass of tap water. After taking the tablet, you should stay upright for the next 30 minutes. You should not eat anything for at least 30 minutes, including taking any other medicines. You need to regularly visit your doctor to have your bone health checked. You need to regularly visit a pharmacy for a new supply. 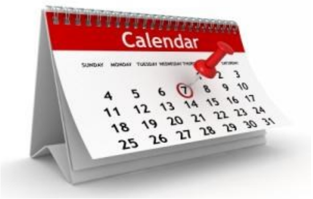 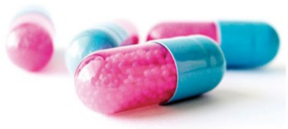

If you take Medicine A, you would live for **4 years and 9 months** and then die.

If you take Medicine B, you would live for **5 years** and then die.

Would you prefer Medicine A or Medicine B, or are they the same?

Indicate your choice here. You can only choose one option:

○ **Medicine A** Live for 4 years 9 months

○ **Medicine B** Live for 5 years

○ **The Same - Medicine A and Medicine B are the same.**

0% 100%

For peer review only

Repeated section

Remember Medicine A and Medicine B are equally effective and have no risks of a side effect.

The only difference between Medicine A and Medicine B is how you take them.

Medicine A	Medicine B
You take a tablet only once in your life. You will be given the tablet on the day you come in to see your GP.<br>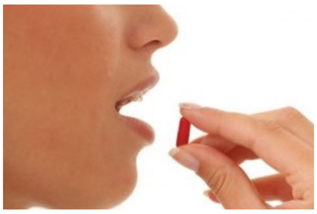	One tablet should be taken once a week on the same day every week.<br>You need to take this medicine for 5 years.<br><br>You need to take the tablet first thing in the morning on an empty stomach with a large glass of tap water.<br>After taking the tablet, you should stay upright for the next 30 minutes.<br>You should not eat anything for at least 30 minutes, including taking any other medicines.<br><br>You need to regularly visit your doctor to have your bone health checked.<br>You need to regularly visit a pharmacy for a new supply.<br>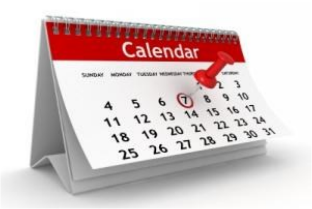 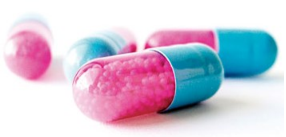

If you take Medicine A, you would live for **2 months less than 5 years** and then die.

If you take Medicine B, you would live for **5 years** and then die.

Would you prefer Medicine A or Medicine B, or are they the same?

Indicate your choice here. You can only choose one option:

◯ **Medicine A** Live for (5 years – 2 months)

◯ **Medicine B** Live for 5 years

◯ **The Same - Medicine A and Medicine B are the same.**

0% 100%

For peer review only

Repeated section

Remember Medicine A and Medicine B are equally effective and have no risks of a side effect.

The only difference between Medicine A and Medicine B is how you take them.

**Medicine A**	**Medicine B**
You take a tablet only once in your life.<br>You will be given the tablet on the day you come in to see your GP.<br>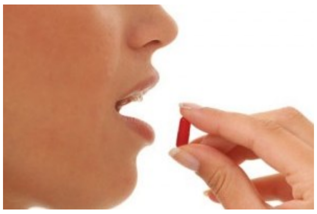	One tablet should be taken once a week on the same day every week.<br>You need to take this medicine for 5 years.<br><br>You need to take the tablet first thing in the morning on an empty stomach with a large glass of tap water.<br>After taking the tablet, you should stay upright for the next 30 minutes.<br>You should not eat anything for at least 30 minutes, including taking any other medicines.<br><br>You need to regularly visit your doctor to have your bone health checked.<br>You need to regularly visit a pharmacy for a new supply.<br>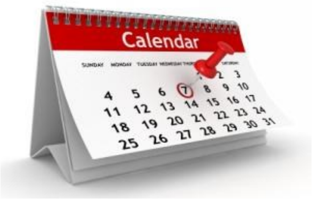 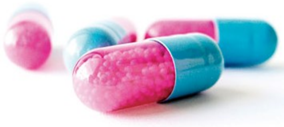

If you take Medicine A, you would live for **1 month less than 5 years** and then die.

If you take Medicine B, you would live for **5 years** and then die.

Would you prefer Medicine A or Medicine B, or are they the same?

Indicate your choice here. You can only choose one option:

◯ **Medicine A** Live for (5 years – 1 month)

◯ **Medicine B** Live for 5 years

◯ **The Same - Medicine A and Medicine B are the same.**

0% 100%

For peer review only

Repeated section

Remember Medicine A and Medicine B are equally effective and have no risks of a side effect.

The only difference between Medicine A and Medicine B is how you take them.

Medicine A	Medicine B
You take a tablet only once in your life. You will be given the tablet on the day you come in to see your GP. 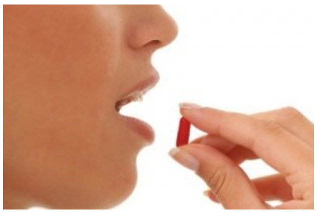	One tablet should be taken once a week on the same day every week. You need to take this medicine for 5 years.<br><br>You need to take the tablet first thing in the morning on an empty stomach with a large glass of tap water. After taking the tablet, you should stay upright for the next 30 minutes. You should not eat anything for at least 30 minutes, including taking any other medicines.<br><br>You need to regularly visit your doctor to have your bone health checked. You need to regularly visit a pharmacy for a new supply.<br>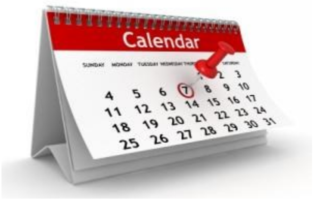 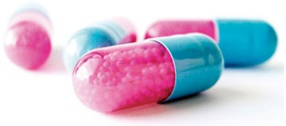

If you take Medicine A, you would live for **2 weeks less than 5 years** and then die.

If you take Medicine B, you would live for **5 years** and then die.

Would you prefer Medicine A or Medicine B, or are they the same?

Indicate your choice here. You can only choose one option:

○ **Medicine A** Live for (5 years – 2 weeks)

○ **Medicine B** Live for 5 years

○ **The Same - Medicine A and Medicine B are the same.**

For peer review only

Repeated section

Remember Medicine A and Medicine B are equally effective and have no risks of a side effect.

The only difference between Medicine A and Medicine B is how you take them.

Medicine A	Medicine B
You take a tablet only once in your life.<br>You will be given the tablet on the day you come in to see your GP.<br>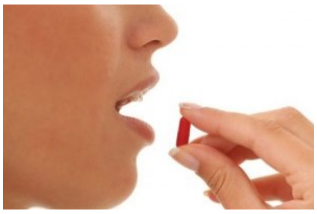	One tablet should be taken once a week on the same day every week.<br>You need to take this medicine for 5 years.<br><br>You need to take the tablet first thing in the morning on an empty stomach with a large glass of tap water.<br>After taking the tablet, you should stay upright for the next 30 minutes.<br>You should not eat anything for at least 30 minutes, including taking any other medicines.<br><br>You need to regularly visit your doctor to have your bone health checked.<br>You need to regularly visit a pharmacy for a new supply.<br>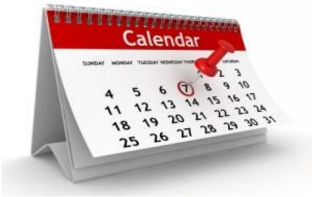 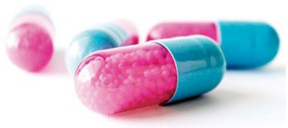

If you take Medicine A, you would live for **1 week less than 5 years** and then die.

If you take Medicine B, you would live for **5 years** and then die.

Would you prefer Medicine A or Medicine B, or are they the same?

Indicate your choice here. You can only choose one option:

○ **Medicine A** Live for (5 years – 1 week)

○ **Medicine B** Live for 5 years

○ **The Same - Medicine A and Medicine B are the same.**

0% 100%

For peer review only

Repeated section

Remember Medicine A and Medicine B are equally effective and have no risks of a side effect.

The only difference between Medicine A and Medicine B is how you take them.

Medicine A	Medicine B
You take a tablet only once in your life. You will be given the tablet on the day you come in to see your GP. 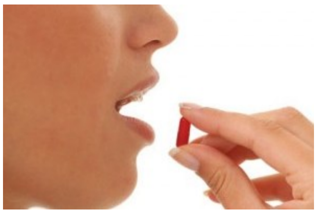	One tablet should be taken once a week on the same day every week. You need to take this medicine for 5 years. You need to take the tablet first thing in the morning on an empty stomach with a large glass of tap water. After taking the tablet, you should stay upright for the next 30 minutes. You should not eat anything for at least 30 minutes, including taking any other medicines. You need to regularly visit your doctor to have your bone health checked. You need to regularly visit a pharmacy for a new supply. 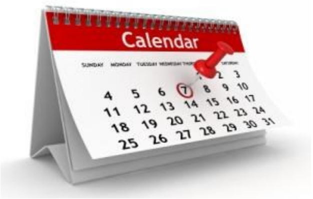 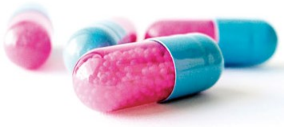

If you take Medicine A, you would live for **5 days less than 5 years** and then die.

If you take Medicine B, you would live for **5 years** and then die.

Would you prefer Medicine A or Medicine B, or are they the same?

Indicate your choice here. You can only choose one option:

◯ **Medicine A** Live for (5 years – 5 days)

◯ **Medicine B** Live for 5 years

◯ **The Same - Medicine A and Medicine B are the same.**

0% 100%

For peer review only

Repeated section

Remember Medicine A and Medicine B are equally effective and have no risks of a side effect.

The only difference between Medicine A and Medicine B is how you take them.

Medicine A	Medicine B
You take a tablet only once in your life. You will be given the tablet on the day you come in to see your GP. 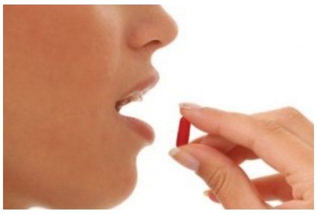	One tablet should be taken once a week on the same day every week. You need to take this medicine for 5 years. You need to take the tablet first thing in the morning on an empty stomach with a large glass of tap water. After taking the tablet, you should stay upright for the next 30 minutes. You should not eat anything for at least 30 minutes, including taking any other medicines. You need to regularly visit your doctor to have your bone health checked. You need to regularly visit a pharmacy for a new supply. 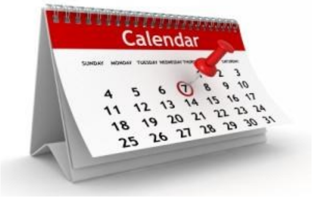 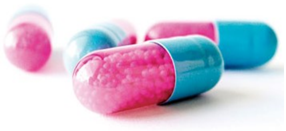

If you take Medicine A, you would live for **3 days less than 5 years** and then die.

If you take Medicine B, you would live for **5 years** and then die.

Would you prefer Medicine A or Medicine B, or are they the same?

Indicate your choice here. You can only choose one option:

○ **Medicine A** Live for (5 years – 3 days)

○ **Medicine B** Live for 5 years

○ **The Same - Medicine A and Medicine B are the same.**

0% 100%

For peer review only

Repeated section

Remember Medicine A and Medicine B are equally effective and have no risks of a side effect.

The only difference between Medicine A and Medicine B is how you take them.

Medicine A	Medicine B
You take a tablet only once in your life. You will be given the tablet on the day you come in to see your GP.<br>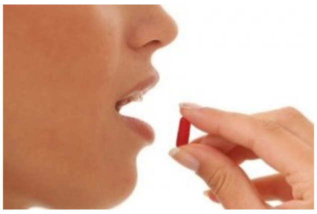	One tablet should be taken once a week on the same day every week.<br>You need to take this medicine for 5 years.<br><br>You need to take the tablet first thing in the morning on an empty stomach with a large glass of tap water.<br>After taking the tablet, you should stay upright for the next 30 minutes.<br>You should not eat anything for at least 30 minutes, including taking any other medicines.<br><br>You need to regularly visit your doctor to have your bone health checked.<br>You need to regularly visit a pharmacy for a new supply.<br>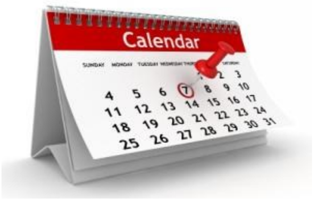 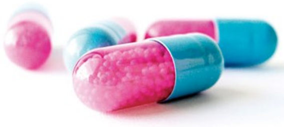

If you take Medicine A, you would live for **1 day less than 5 years** and then die.

If you take Medicine B, you would live for **5 years** and then die.

Would you prefer Medicine A or Medicine B, or are they the same?

Indicate your choice here. You can only choose one option:

◯ **Medicine A** Live for (5 years – 1 day)

◯ **Medicine B** Live for 5 years

◯ **The Same - Medicine A and Medicine B are the same.**

0% 100%

For peer review only

Question 4a.

You prefer living for 2.5 years (2 years and 6 months) on Medicine A, to living for 5 years on Medicine B.

This means **living fewer than 2.5 years on Medicine A** can be the same as living for 5 years on Medicine B.

How many years on Medicine A do you think would be the same as living for 5 years on Medicine B?

(Please give a number in years)

0% 100%

peer review only

Was there anything so far which you think is unclear or that you didn't understand?

(Please provide details in the comment box below)

0% 100%

peer review only

Part 2a

Question 5

You have been told that you need to take a medicine to reduce the risk of fracture.

You can choose between two different medicines. We have called these Medicine A and Medicine B.

Medicine A is a pill that you only need to take once.

Medicine B is a pill that you need to take every week for 5 years.

Medicine A and Medicine B are equally effective in reducing your risk of having a fracture.
Medicine A and Medicine B will reduce your risk of experiencing a fracture, such as hip fracture, within the next 5 years from **10%** to **6%**.

In other words, in a crowd of **1,000** people in the UK, **100** are likely to have a fracture if they do not take the medicine. This is reduced to **60** people out of 1,000 who do take the medicine.
This information is also illustrated in this picture.

Risk of fracture

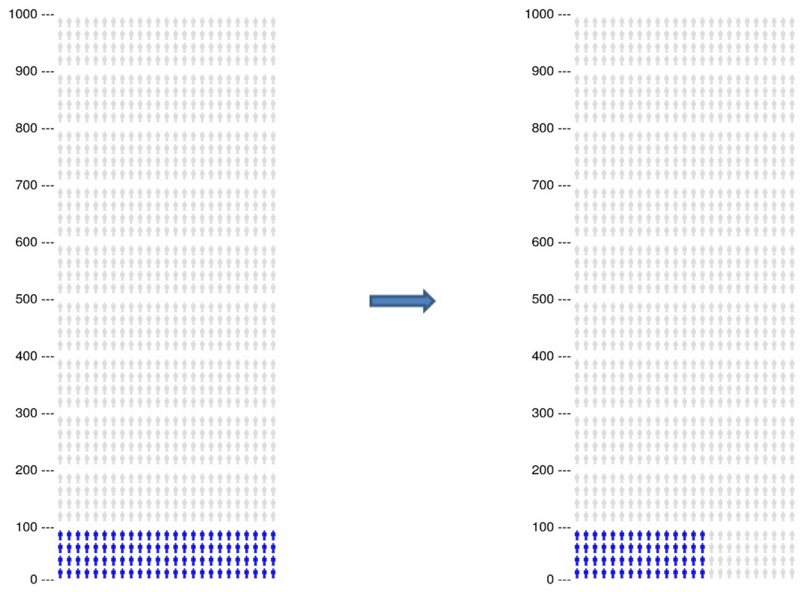

10%
No Treatment

6%
Taking the Medicine

(The figures shaded blue represent people who will have a fracture, such as hip fracture, within the next 5 years)

Please continue to the next page.

0% 100%

Repeated section

Remember Medicine A and Medicine B are equally effective.

However, there are some differences between Medicine A and Medicine B.

Medicine A	Medicine B
**No inconvenience**<br><br>You take a tablet only once in your life.<br>You will be given the tablet on the day you come in to see your GP.	**Inconvenience**<br><br>One tablet should be taken once a week on the same day every week.<br>You need to take this medicine for 5 years.<br><br>You need to take the tablet first thing in the morning on an empty stomach with a large glass of tap water.<br>After taking the tablet, you should stay upright for the next 30 minutes.<br><br>You should not eat anything for at least 30 minutes, including taking any other medicines.<br><br>You need to regularly visit your doctor to have your bone health checked.<br>You need to regularly visit a pharmacy for a new supply.
**Severe side effect - 0%**<br><br>No one taking Medicine A will have a severe side effect.	**Severe side effect - 0.7%**<br><br>Among every 1,000 people taking Medicine B, **7** will experience a severe side effect.
**Minor side effect - 0%**<br><br>No one taking Medicine A will have a minor side effect.	**Minor side effect - 13%**<br><br>Among every 1,000 people taking Medicine B, **130** will experience a minor side effect.

If you take Medicine A, you would live for **2.5 years (2 years and 6 months)** and then die.

If you take Medicine B, you would live for **5 years** and then die.

Would you prefer Medicine A or Medicine B, or are they the same?

Indicate your choice here. You can only choose one option:

○ **Medicine A**

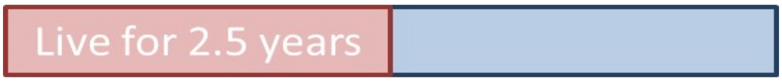

○ **Medicine B**

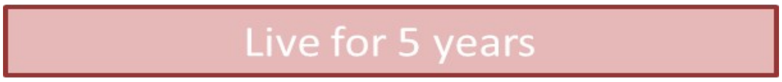

○ **The Same - Medicine A and Medicine B are the same.**

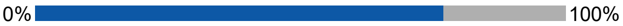
0%
100%

Repeated section

Remember Medicine A and Medicine B are equally effective.

However, there are some differences between Medicine A and Medicine B.

Medicine A	Medicine B
**No inconvenience**<br><br>You take a tablet only once in your life.<br>You will be given the tablet on the day you come in to see your GP.	**Inconvenience**<br><br>One tablet should be taken once a week on the same day every week.<br>You need to take this medicine for 5 years.<br><br>You need to take the tablet first thing in the morning on an empty stomach with a large glass of tap water.<br>After taking the tablet, you should stay upright for the next 30 minutes.<br><br>You should not eat anything for at least 30 minutes, including taking any other medicines.<br><br>You need to regularly visit your doctor to have your bone health checked.<br>You need to regularly visit a pharmacy for a new supply.
**Severe side effect - 0%**<br><br>No one taking Medicine A will have a severe side effect.	**Severe side effect - 0.7%**<br><br>Among every 1,000 people taking Medicine B, **7** will experience a severe side effect.
**Minor side effect - 0%**<br><br>No one taking Medicine A will have a minor side effect.	**Minor side effect - 13%**<br><br>Among every 1,000 people taking Medicine B, **130** will experience a minor side effect.

If you take Medicine A, you would live for **3 years** and then die.

If you take Medicine B, you would live for **5 years** and then die.

Would you prefer Medicine A or Medicine B, or are they the same?

Indicate your choice here. You can only choose one option:

○ **Medicine A**

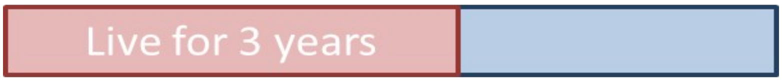

○ **Medicine B**

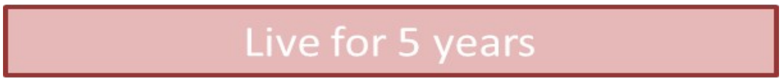

○ **The Same - Medicine A and Medicine B are the same.**

0% 100%

For peer review only

Repeated section

Remember Medicine A and Medicine B are equally effective.

However, there are some differences between Medicine A and Medicine B.

Medicine A	Medicine B
**No inconvenience**<br><br>You take a tablet only once in your life.<br>You will be given the tablet on the day you come in to see your GP.	**Inconvenience**<br><br>One tablet should be taken once a week on the same day every week.<br>You need to take this medicine for 5 years.<br><br>You need to take the tablet first thing in the morning on an empty stomach with a large glass of tap water.<br>After taking the tablet, you should stay upright for the next 30 minutes.<br><br>You should not eat anything for at least 30 minutes, including taking any other medicines.<br><br>You need to regularly visit your doctor to have your bone health checked.<br>You need to regularly visit a pharmacy for a new supply.
**Severe side effect - 0%**<br><br>No one taking Medicine A will have a severe side effect.	**Severe side effect - 0.7%**<br><br>Among every 1,000 people taking Medicine B, **7** will experience a severe side effect.
**Minor side effect - 0%**<br><br>No one taking Medicine A will have a minor side effect.	**Minor side effect - 13%**<br><br>Among every 1,000 people taking Medicine B, **130** will experience a minor side effect.

If you take Medicine A, you would live for **3.25 years** (3 years and 3 months) and then die.

If you take Medicine B, you would live for **5 years** and then die.

Would you prefer Medicine A or Medicine B, or are they the same?

Indicate your choice here. You can only choose one option:

◯ **Medicine A** 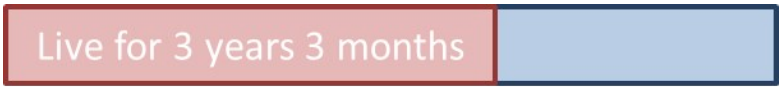

◯ **Medicine B** 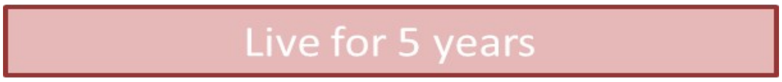

◯ **The Same - Medicine A and Medicine B are the same.**

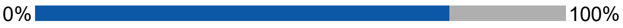

For peer review only

Repeated section

Remember Medicine A and Medicine B are equally effective.

However, there are some differences between Medicine A and Medicine B.

Medicine A	Medicine B
**No inconvenience**<br><br>You take a tablet only once in your life.<br>You will be given the tablet on the day you come in to see your GP.	**Inconvenience**<br><br>One tablet should be taken once a week on the same day every week.<br>You need to take this medicine for 5 years.<br><br>You need to take the tablet first thing in the morning on an empty stomach with a large glass of tap water.<br>After taking the tablet, you should stay upright for the next 30 minutes.<br><br>You should not eat anything for at least 30 minutes, including taking any other medicines.<br><br>You need to regularly visit your doctor to have your bone health checked.<br>You need to regularly visit a pharmacy for a new supply.
**Severe side effect - 0%**<br><br>No one taking Medicine A will have a severe side effect.	**Severe side effect - 0.7%**<br><br>Among every 1,000 people taking Medicine B, **7** will experience a severe side effect.
**Minor side effect - 0%**<br><br>No one taking Medicine A will have a minor side effect.	**Minor side effect - 13%**<br><br>Among every 1,000 people taking Medicine B, **130** will experience a minor side effect.

If you take Medicine A, you would live for **3.5 years** (3 years and 6 months) and then die.

If you take Medicine B, you would live for **5 years** and then die.

Would you prefer Medicine A or Medicine B, or are they the same?

Indicate your choice here. You can only choose one option:

○ **Medicine A** — Live for 3 years 6 months

○ **Medicine B** — Live for 5 years

○ **The Same - Medicine A and Medicine B are the same.**

0% 100%

For peer review only

Repeated section

Remember Medicine A and Medicine B are equally effective.

However, there are some differences between Medicine A and Medicine B.

Medicine A	Medicine B
**No inconvenience**<br><br>You take a tablet only once in your life.<br>You will be given the tablet on the day you come in to see your GP.	**Inconvenience**<br><br>One tablet should be taken once a week on the same day every week.<br>You need to take this medicine for 5 years.<br><br>You need to take the tablet first thing in the morning on an empty stomach with a large glass of tap water.<br>After taking the tablet, you should stay upright for the next 30 minutes.<br><br>You should not eat anything for at least 30 minutes, including taking any other medicines.<br><br>You need to regularly visit your doctor to have your bone health checked.<br>You need to regularly visit a pharmacy for a new supply.
**Severe side effect - 0%**<br><br>No one taking Medicine A will have a severe side effect.	**Severe side effect - 0.7%**<br><br>Among every 1,000 people taking Medicine B, **7** will experience a severe side effect.
**Minor side effect - 0%**<br><br>No one taking Medicine A will have a minor side effect.	**Minor side effect - 13%**<br><br>Among every 1,000 people taking Medicine B, **130** will experience a minor side effect.

If you take Medicine A, you would live for **3.75 years** (3 years and 9 months) and then die.

If you take Medicine B, you would live for **5 years** and then die.

Would you prefer Medicine A or Medicine B, or are they the same?

Indicate your choice here. You can only choose one option:

○ **Medicine A** Live for 3 years 9 months

○ **Medicine B** Live for 5 years

○ **The Same - Medicine A and Medicine B are the same.**

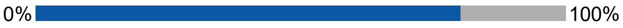

For peer review only

Repeated section

Remember Medicine A and Medicine B are equally effective.

However, there are some differences between Medicine A and Medicine B.

Medicine A	Medicine B
**No inconvenience**<br><br>You take a tablet only once in your life.<br>You will be given the tablet on the day you come in to see your GP.	**Inconvenience**<br><br>One tablet should be taken once a week on the same day every week.<br>You need to take this medicine for 5 years.<br><br>You need to take the tablet first thing in the morning on an empty stomach with a large glass of tap water.<br>After taking the tablet, you should stay upright for the next 30 minutes.<br><br>You should not eat anything for at least 30 minutes, including taking any other medicines.<br><br>You need to regularly visit your doctor to have your bone health checked.<br>You need to regularly visit a pharmacy for a new supply.
**Severe side effect - 0%**<br><br>No one taking Medicine A will have a severe side effect.	**Severe side effect - 0.7%**<br><br>Among every 1,000 people taking Medicine B, **7** will experience a severe side effect.
**Minor side effect - 0%**<br><br>No one taking Medicine A will have a minor side effect.	**Minor side effect - 13%**<br><br>Among every 1,000 people taking Medicine B, **130** will experience a minor side effect.

If you take Medicine A, you would live for **3 years and 10 months** and then die.

If you take Medicine B, you would live for **5 years** and then die.

Would you prefer Medicine A or Medicine B, or are they the same?

Indicate your choice here. You can only choose one option:

○ **Medicine A** Live for 3 years 10 months

○ **Medicine B** Live for 5 years

○ **The Same - Medicine A and Medicine B are the same.**

0% 100%

For peer review only

Repeated section

Remember Medicine A and Medicine B are equally effective.

However, there are some differences between Medicine A and Medicine B.

Medicine A	Medicine B
**No inconvenience**<br><br>You take a tablet only once in your life.<br>You will be given the tablet on the day you come in to see your GP.	**Inconvenience**<br><br>One tablet should be taken once a week on the same day every week.<br>You need to take this medicine for 5 years.<br><br>You need to take the tablet first thing in the morning on an empty stomach with a large glass of tap water.<br>After taking the tablet, you should stay upright for the next 30 minutes.<br><br>You should not eat anything for at least 30 minutes, including taking any other medicines.<br><br>You need to regularly visit your doctor to have your bone health checked.<br>You need to regularly visit a pharmacy for a new supply.
**Severe side effect - 0%**<br><br>No one taking Medicine A will have a severe side effect.	**Severe side effect - 0.7%**<br><br>Among every 1,000 people taking Medicine B, **7** will experience a severe side effect.
**Minor side effect - 0%**<br><br>No one taking Medicine A will have a minor side effect.	**Minor side effect - 13%**<br><br>Among every 1,000 people taking Medicine B, **130** will experience a minor side effect.

If you take Medicine A, you would live for **4 years** and then die.

If you take Medicine B, you would live for **5 years** and then die.

Would you prefer Medicine A or Medicine B, or are they the same?

Indicate your choice here. You can only choose one option:

◯ **Medicine A**

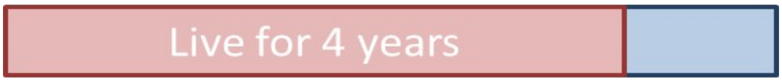

◯ **Medicine B**

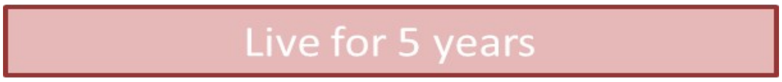

◯ **The Same - Medicine A and Medicine B are the same.**

0% 100%

For peer review only

Repeated section

Remember Medicine A and Medicine B are equally effective.

However, there are some differences between Medicine A and Medicine B.

Medicine A	Medicine B
**No inconvenience**<br><br>You take a tablet only once in your life.<br>You will be given the tablet on the day you come in to see your GP.	**Inconvenience**<br><br>One tablet should be taken once a week on the same day every week.<br>You need to take this medicine for 5 years.<br><br>You need to take the tablet first thing in the morning on an empty stomach with a large glass of tap water.<br>After taking the tablet, you should stay upright for the next 30 minutes.<br><br>You should not eat anything for at least 30 minutes, including taking any other medicines.<br><br>You need to regularly visit your doctor to have your bone health checked.<br>You need to regularly visit a pharmacy for a new supply.
**Severe side effect - 0%**<br><br>No one taking Medicine A will have a severe side effect.	**Severe side effect - 0.7%**<br><br>Among every 1,000 people taking Medicine B, **7** will experience a severe side effect.
**Minor side effect - 0%**<br><br>No one taking Medicine A will have a minor side effect.	**Minor side effect - 13%**<br><br>Among every 1,000 people taking Medicine B, **130** will experience a minor side effect.

If you take Medicine A, you would live for **4 years and 2 months** and then die.

If you take Medicine B, you would live for **5 years** and then die.

Would you prefer Medicine A or Medicine B, or are they the same?

Indicate your choice here. You can only choose one option:

◯ **Medicine A** Live for 4 years 2 months

◯ **Medicine B** Live for 5 years

◯ **The Same - Medicine A and Medicine B are the same.**

0% 100%

For peer review only

Repeated section

Remember Medicine A and Medicine B are equally effective.

However, there are some differences between Medicine A and Medicine B.

Medicine A	Medicine B
**No inconvenience**<br><br>You take a tablet only once in your life.<br>You will be given the tablet on the day you come in to see your GP.	**Inconvenience**<br><br>One tablet should be taken once a week on the same day every week.<br>You need to take this medicine for 5 years.<br><br>You need to take the tablet first thing in the morning on an empty stomach with a large glass of tap water.<br>After taking the tablet, you should stay upright for the next 30 minutes.<br><br>You should not eat anything for at least 30 minutes, including taking any other medicines.<br><br>You need to regularly visit your doctor to have your bone health checked.<br>You need to regularly visit a pharmacy for a new supply.
**Severe side effect - 0%**<br><br>No one taking Medicine A will have a severe side effect.	**Severe side effect - 0.7%**<br><br>Among every 1,000 people taking Medicine B, **7** will experience a severe side effect.
**Minor side effect - 0%**<br><br>No one taking Medicine A will have a minor side effect.	**Minor side effect - 13%**<br><br>Among every 1,000 people taking Medicine B, **130** will experience a minor side effect.

If you take Medicine A, you would live for **4.25 years** (4 years and 3 months) and then die.

If you take Medicine B, you would live for **5 years** and then die.

Would you prefer Medicine A or Medicine B, or are they the same?

Indicate your choice here. You can only choose one option:

○ **Medicine A**

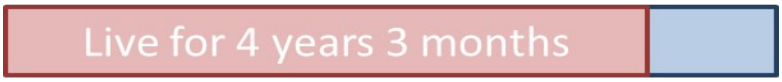

○ **Medicine B**

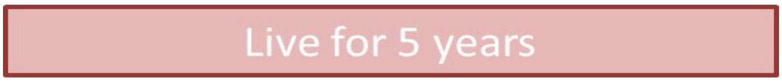

○ **The Same - Medicine A and Medicine B are the same.**

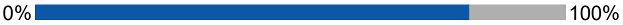

For peer review only

Repeated section

Remember Medicine A and Medicine B are equally effective.

However, there are some differences between Medicine A and Medicine B.

Medicine A	Medicine B
**No inconvenience**<br><br>You take a tablet only once in your life.<br>You will be given the tablet on the day you come in to see your GP.	**Inconvenience**<br><br>One tablet should be taken once a week on the same day every week.<br>You need to take this medicine for 5 years.<br><br>You need to take the tablet first thing in the morning on an empty stomach with a large glass of tap water.<br>After taking the tablet, you should stay upright for the next 30 minutes.<br><br>You should not eat anything for at least 30 minutes, including taking any other medicines.<br><br>You need to regularly visit your doctor to have your bone health checked.<br>You need to regularly visit a pharmacy for a new supply.
**Severe side effect - 0%**<br><br>No one taking Medicine A will have a severe side effect.	**Severe side effect - 0.7%**<br><br>Among every 1,000 people taking Medicine B, **7** will experience a severe side effect.
**Minor side effect - 0%**<br><br>No one taking Medicine A will have a minor side effect.	**Minor side effect - 13%**<br><br>Among every 1,000 people taking Medicine B, **130** will experience a minor side effect.

If you take Medicine A, you would live for **4 years 4 months** and then die.

If you take Medicine B, you would live for **5 years** and then die.

Would you prefer Medicine A or Medicine B, or are they the same?

Indicate your choice here. You can only choose one option:

◯ **Medicine A** 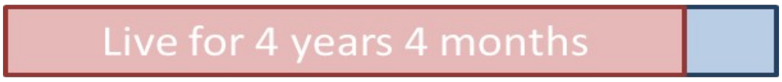

◯ **Medicine B** 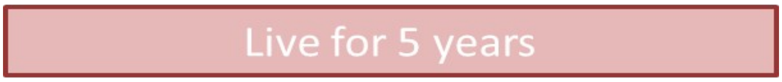

◯ **The Same - Medicine A and Medicine B are the same.**

0% 100%

For peer review only

Repeated section

Remember Medicine A and Medicine B are equally effective.

However, there are some differences between Medicine A and Medicine B.

Medicine A	Medicine B
**No inconvenience**<br><br>You take a tablet only once in your life.<br>You will be given the tablet on the day you come in to see your GP.	**Inconvenience**<br><br>One tablet should be taken once a week on the same day every week.<br>You need to take this medicine for 5 years.<br><br>You need to take the tablet first thing in the morning on an empty stomach with a large glass of tap water.<br>After taking the tablet, you should stay upright for the next 30 minutes.<br><br>You should not eat anything for at least 30 minutes, including taking any other medicines.<br><br>You need to regularly visit your doctor to have your bone health checked.<br>You need to regularly visit a pharmacy for a new supply.
**Severe side effect - 0%**<br><br>No one taking Medicine A will have a severe side effect.	**Severe side effect - 0.7%**<br><br>Among every 1,000 people taking Medicine B, **7** will experience a severe side effect.
**Minor side effect - 0%**<br><br>No one taking Medicine A will have a minor side effect.	**Minor side effect - 13%**<br><br>Among every 1,000 people taking Medicine B, **130** will experience a minor side effect.

If you take Medicine A, you would live for **4.5 years** (4 years and 6 months) and then die.

If you take Medicine B, you would live for **5 years** and then die.

Would you prefer Medicine A or Medicine B, or are they the same?

Indicate your choice here. You can only choose one option:

◯ **Medicine A** Live for 4 years 6 months

◯ **Medicine B** Live for 5 years

◯ **The Same - Medicine A and Medicine B are the same.**

0% 100%

For peer review only

Repeated section

Remember Medicine A and Medicine B are equally effective.

However, there are some differences between Medicine A and Medicine B.

Medicine A	Medicine B
**No inconvenience**<br><br>You take a tablet only once in your life.<br>You will be given the tablet on the day you come in to see your GP.	**Inconvenience**<br><br>One tablet should be taken once a week on the same day every week.<br>You need to take this medicine for 5 years.<br><br>You need to take the tablet first thing in the morning on an empty stomach with a large glass of tap water.<br>After taking the tablet, you should stay upright for the next 30 minutes.<br><br>You should not eat anything for at least 30 minutes, including taking any other medicines.<br><br>You need to regularly visit your doctor to have your bone health checked.<br>You need to regularly visit a pharmacy for a new supply.
**Severe side effect - 0%**<br><br>No one taking Medicine A will have a severe side effect.	**Severe side effect - 0.7%**<br><br>Among every 1,000 people taking Medicine B, **7** will experience a severe side effect.
**Minor side effect - 0%**<br><br>No one taking Medicine A will have a minor side effect.	**Minor side effect - 13%**<br><br>Among every 1,000 people taking Medicine B, **130** will experience a minor side effect.

If you take Medicine A, you would live for **4 years and 7 months** and then die.

If you take Medicine B, you would live for **5 years** and then die.

Would you prefer Medicine A or Medicine B, or are they the same?

Indicate your choice here. You can only choose one option:

◯ **Medicine A** 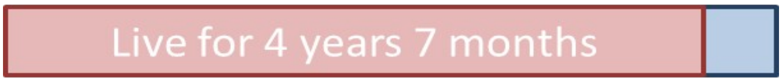

◯ **Medicine B** 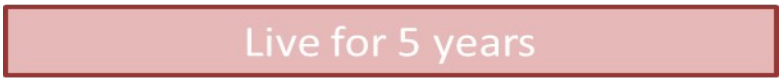

◯ **The Same - Medicine A and Medicine B are the same.**

0% 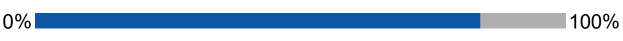 100%

For peer review only

Repeated section

Remember Medicine A and Medicine B are equally effective.

However, there are some differences between Medicine A and Medicine B.

Medicine A	Medicine B
**No inconvenience**<br><br>You take a tablet only once in your life.<br>You will be given the tablet on the day you come in to see your GP.	**Inconvenience**<br><br>One tablet should be taken once a week on the same day every week.<br>You need to take this medicine for 5 years.<br><br>You need to take the tablet first thing in the morning on an empty stomach with a large glass of tap water.<br>After taking the tablet, you should stay upright for the next 30 minutes.<br><br>You should not eat anything for at least 30 minutes, including taking any other medicines.<br><br>You need to regularly visit your doctor to have your bone health checked.<br>You need to regularly visit a pharmacy for a new supply.
**Severe side effect - 0%**<br><br>No one taking Medicine A will have a severe side effect.	**Severe side effect - 0.7%**<br><br>Among every 1,000 people taking Medicine B, **7** will experience a severe side effect.
**Minor side effect - 0%**<br><br>No one taking Medicine A will have a minor side effect.	**Minor side effect - 13%**<br><br>Among every 1,000 people taking Medicine B, **130** will experience a minor side effect.

If you take Medicine A, you would live for **4 years and 9 months** and then die.

If you take Medicine B, you would live for **5 years** and then die.

Would you prefer Medicine A or Medicine B, or are they the same?

Indicate your choice here. You can only choose one option:

◯ **Medicine A**

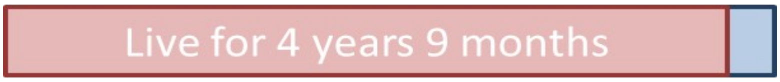

◯ **Medicine B**

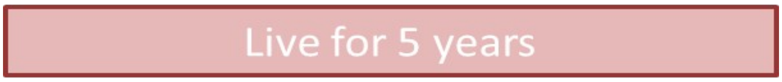

◯ **The Same - Medicine A and Medicine B are the same.**

0% 100%

For peer review only

Repeated section

Remember Medicine A and Medicine B are equally effective.

However, there are some differences between Medicine A and Medicine B.

Medicine A	Medicine B
**No inconvenience**<br><br>You take a tablet only once in your life.<br>You will be given the tablet on the day you come in to see your GP.	**Inconvenience**<br><br>One tablet should be taken once a week on the same day every week.<br>You need to take this medicine for 5 years.<br><br>You need to take the tablet first thing in the morning on an empty stomach with a large glass of tap water.<br>After taking the tablet, you should stay upright for the next 30 minutes.<br><br>You should not eat anything for at least 30 minutes, including taking any other medicines.<br><br>You need to regularly visit your doctor to have your bone health checked.<br>You need to regularly visit a pharmacy for a new supply.
**Severe side effect - 0%**<br><br>No one taking Medicine A will have a severe side effect.	**Severe side effect - 0.7%**<br><br>Among every 1,000 people taking Medicine B, **7** will experience a severe side effect.
**Minor side effect - 0%**<br><br>No one taking Medicine A will have a minor side effect.	**Minor side effect - 13%**<br><br>Among every 1,000 people taking Medicine B, **130** will experience a minor side effect.

If you take Medicine A, you would live for **2 months less than 5 years** and then die.

If you take Medicine B, you would live for **5 years** and then die.

Would you prefer Medicine A or Medicine B, or are they the same?

Indicate your choice here. You can only choose one option:

○ **Medicine A**

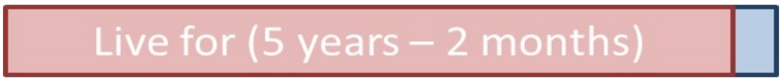

○ **Medicine B**

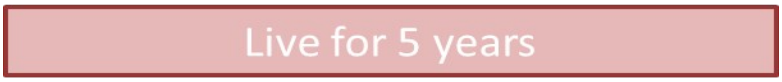

○ **The Same - Medicine A and Medicine B are the same.**

0% 100%

For peer review only

Repeated section

Remember Medicine A and Medicine B are equally effective.

However, there are some differences between Medicine A and Medicine B.

Medicine A	Medicine B
**No inconvenience**<br><br>You take a tablet only once in your life.<br>You will be given the tablet on the day you come in to see your GP.	**Inconvenience**<br><br>One tablet should be taken once a week on the same day every week.<br>You need to take this medicine for 5 years.<br><br>You need to take the tablet first thing in the morning on an empty stomach with a large glass of tap water.<br>After taking the tablet, you should stay upright for the next 30 minutes.<br><br>You should not eat anything for at least 30 minutes, including taking any other medicines.<br><br>You need to regularly visit your doctor to have your bone health checked.<br>You need to regularly visit a pharmacy for a new supply.
**Severe side effect - 0%**<br><br>No one taking Medicine A will have a severe side effect.	**Severe side effect - 0.7%**<br><br>Among every 1,000 people taking Medicine B, **7** will experience a severe side effect.
**Minor side effect - 0%**<br><br>No one taking Medicine A will have a minor side effect.	**Minor side effect - 13%**<br><br>Among every 1,000 people taking Medicine B, **130** will experience a minor side effect.

If you take Medicine A, you would live for **1 month less than 5 years** and then die.

If you take Medicine B, you would live for **5 years** and then die.

Would you prefer Medicine A or Medicine B, or are they the same?

Indicate your choice here. You can only choose one option:

◯ **Medicine A** Live for (5 years – 1 month)

◯ **Medicine B** Live for 5 years

◯ **The Same - Medicine A and Medicine B are the same.**

0% 100%

For peer review only

Repeated section

Remember Medicine A and Medicine B are equally effective.

However, there are some differences between Medicine A and Medicine B.

Medicine A	Medicine B
**No inconvenience**<br><br>You take a tablet only once in your life.<br>You will be given the tablet on the day you come in to see your GP.	**Inconvenience**<br><br>One tablet should be taken once a week on the same day every week.<br>You need to take this medicine for 5 years.<br><br>You need to take the tablet first thing in the morning on an empty stomach with a large glass of tap water.<br>After taking the tablet, you should stay upright for the next 30 minutes.<br><br>You should not eat anything for at least 30 minutes, including taking any other medicines.<br><br>You need to regularly visit your doctor to have your bone health checked.<br>You need to regularly visit a pharmacy for a new supply.
**Severe side effect - 0%**<br><br>No one taking Medicine A will have a severe side effect.	**Severe side effect - 0.7%**<br><br>Among every 1,000 people taking Medicine B, **7** will experience a severe side effect.
**Minor side effect - 0%**<br><br>No one taking Medicine A will have a minor side effect.	**Minor side effect - 13%**<br><br>Among every 1,000 people taking Medicine B, **130** will experience a minor side effect.

If you take Medicine A, you would live for **2 weeks less than 5 years** and then die.

If you take Medicine B, you would live for **5 years** and then die.

Would you prefer Medicine A or Medicine B, or are they the same?

Indicate your choice here. You can only choose one option:

◯ **Medicine A** Live for (5 years – 2 weeks)

◯ **Medicine B** Live for 5 years

◯ **The Same - Medicine A and Medicine B are the same.**

0% 100%

For peer review only

Repeated section

Remember Medicine A and Medicine B are equally effective.

However, there are some differences between Medicine A and Medicine B.

Medicine A	Medicine B
**No inconvenience**<br><br>You take a tablet only once in your life.<br>You will be given the tablet on the day you come in to see your GP.	**Inconvenience**<br><br>One tablet should be taken once a week on the same day every week.<br>You need to take this medicine for 5 years.<br><br>You need to take the tablet first thing in the morning on an empty stomach with a large glass of tap water.<br>After taking the tablet, you should stay upright for the next 30 minutes.<br><br>You should not eat anything for at least 30 minutes, including taking any other medicines.<br><br>You need to regularly visit your doctor to have your bone health checked.<br>You need to regularly visit a pharmacy for a new supply.
**Severe side effect - 0%**<br><br>No one taking Medicine A will have a severe side effect.	**Severe side effect - 0.7%**<br><br>Among every 1,000 people taking Medicine B, **7** will experience a severe side effect.
**Minor side effect - 0%**<br><br>No one taking Medicine A will have a minor side effect.	**Minor side effect - 13%**<br><br>Among every 1,000 people taking Medicine B, **130** will experience a minor side effect.

If you take Medicine A, you would live for **1 week less than 5 years** and then die.

If you take Medicine B, you would live for **5 years** and then die.

Would you prefer Medicine A or Medicine B, or are they the same?

Indicate your choice here. You can only choose one option:

○ **Medicine A** Live for (5 years – 1 week)

○ **Medicine B** Live for 5 years

○ **The Same - Medicine A and Medicine B are the same.**

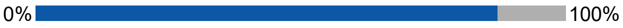

For peer review only

Repeated section

Remember Medicine A and Medicine B are equally effective.

However, there are some differences between Medicine A and Medicine B.

Medicine A	Medicine B
**No inconvenience**<br><br>You take a tablet only once in your life.<br>You will be given the tablet on the day you come in to see your GP.	**Inconvenience**<br><br>One tablet should be taken once a week on the same day every week.<br>You need to take this medicine for 5 years.<br><br>You need to take the tablet first thing in the morning on an empty stomach with a large glass of tap water.<br>After taking the tablet, you should stay upright for the next 30 minutes.<br><br>You should not eat anything for at least 30 minutes, including taking any other medicines.<br><br>You need to regularly visit your doctor to have your bone health checked.<br>You need to regularly visit a pharmacy for a new supply.
**Severe side effect - 0%**<br><br>No one taking Medicine A will have a severe side effect.	**Severe side effect - 0.7%**<br><br>Among every 1,000 people taking Medicine B, **7** will experience a severe side effect.
**Minor side effect - 0%**<br><br>No one taking Medicine A will have a minor side effect.	**Minor side effect - 13%**<br><br>Among every 1,000 people taking Medicine B, **130** will experience a minor side effect.

If you take Medicine A, you would live for **5 days less than 5 years** and then die.

If you take Medicine B, you would live for **5 years** and then die.

Would you prefer Medicine A or Medicine B, or are they the same?

Indicate your choice here. You can only choose one option:

○ **Medicine A** Live for (5 years – 5 days)

○ **Medicine B** Live for 5 years

○ **The Same - Medicine A and Medicine B are the same.**

0% 100%

For peer review only

Repeated section

Remember Medicine A and Medicine B are equally effective.

However, there are some differences between Medicine A and Medicine B.

Medicine A	Medicine B
**No inconvenience**<br><br>You take a tablet only once in your life.<br>You will be given the tablet on the day you come in to see your GP.	**Inconvenience**<br><br>One tablet should be taken once a week on the same day every week.<br>You need to take this medicine for 5 years.<br><br>You need to take the tablet first thing in the morning on an empty stomach with a large glass of tap water.<br>After taking the tablet, you should stay upright for the next 30 minutes.<br><br>You should not eat anything for at least 30 minutes, including taking any other medicines.<br><br>You need to regularly visit your doctor to have your bone health checked.<br>You need to regularly visit a pharmacy for a new supply.
**Severe side effect - 0%**<br><br>No one taking Medicine A will have a severe side effect.	**Severe side effect - 0.7%**<br><br>Among every 1,000 people taking Medicine B, **7** will experience a severe side effect.
**Minor side effect - 0%**<br><br>No one taking Medicine A will have a minor side effect.	**Minor side effect - 13%**<br><br>Among every 1,000 people taking Medicine B, **130** will experience a minor side effect.

If you take Medicine A, you would live for **3 days less than 5 years** and then die.

If you take Medicine B, you would live for **5 years** and then die.

Would you prefer Medicine A or Medicine B, or are they the same?

Indicate your choice here. You can only choose one option:

○ **Medicine A** Live for (5 years – 3 days)

○ **Medicine B** Live for 5 years

○ **The Same - Medicine A and Medicine B are the same.**

0% 100%

For peer review only

Repeated section

Remember Medicine A and Medicine B are equally effective.

However, there are some differences between Medicine A and Medicine B.

Medicine A	Medicine B
**No inconvenience**<br><br>You take a tablet only once in your life.<br>You will be given the tablet on the day you come in to see your GP.	**Inconvenience**<br><br>One tablet should be taken once a week on the same day every week.<br>You need to take this medicine for 5 years.<br><br>You need to take the tablet first thing in the morning on an empty stomach with a large glass of tap water.<br>After taking the tablet, you should stay upright for the next 30 minutes.<br><br>You should not eat anything for at least 30 minutes, including taking any other medicines.<br><br>You need to regularly visit your doctor to have your bone health checked.<br>You need to regularly visit a pharmacy for a new supply.
**Severe side effect - 0%**<br><br>No one taking Medicine A will have a severe side effect.	**Severe side effect - 0.7%**<br><br>Among every 1,000 people taking Medicine B, **7** will experience a severe side effect.
**Minor side effect - 0%**<br><br>No one taking Medicine A will have a minor side effect.	**Minor side effect - 13%**<br><br>Among every 1,000 people taking Medicine B, **130** will experience a minor side effect.

If you take Medicine A, you would live for **1 day less than 5 years** and then die.

If you take Medicine B, you would live for **5 years** and then die.

Would you prefer Medicine A or Medicine B, or are they the same?

Indicate your choice here. You can only choose one option:

◯ **Medicine A** Live for (5 years – 1 day)

◯ **Medicine B** Live for 5 years

◯ **The Same - Medicine A and Medicine B are the same.**

0% 100%

For peer review only

Question 5a.

You prefer living for 2.5 years (2 years and 6 months) on Medicine A, to living for 5 years on Medicine B.

This means **living fewer than 2.5 years on Medicine A** can be the same as living for 5 years on Medicine B.

How many years on Medicine A do you think would be the same as living for 5 years on Medicine B?

(Please give a number in years)

0% 100%

peer review only

Thank you for completing this section and indicating your choices.

Was there anything in this section which you think is unclear or that you didn't understand?

(Please provide details in the comment box below)

0% 100%

For peer review only

Part 3. Questions about you

We understand different people feel differently about different treatments.

In this part of the survey, we ask questions about you to understand if people's age, gender or experiences explain the choices made in Part 2.

0% 100%

For peer review only

We would like to learn about your experience.

Are you currently taking, or have you ever taken any of these medicines (bisphosphonates)?

Aclasta®
Actonel®
Alendronic acid
Binosto®
Bondronat®
Bonefos®
Bonviva®
Clasteon®
Fosamax®
Iasibon®
Ibandronic acid
Loron®
Pamidronate disodium
Quodixor®
Risedronate sodium
Sodium clodronate
Zerlinda®
Zoledronic acid
Zometa®

◯ Yes
◯ No

0% 100%

What do you think would be the **benefits** from taking this medicine (a bisphosphonate)?

What do you think would be the **harms** from taking this medicine (a bisphosphonate)?

Have you experienced any side effects from taking this medicine (a bisphosphonate)?

- ◯ Yes
- ◯ No

Do you find it inconvenient to take this medicine (a bisphosphonate)?
Please specify if you think it is inconvenient.

What is your opinion about taking this medicine (a bisphosphonate)?

- ◯ I don't mind taking them because I have to take them for a reason.
- ◯ I do not like taking them because they remind me that I am not perfectly healthy.
- ◯ I have no opinion.
- ◯ Other (please specify)

0% 100%

Do you take other medicines prescribed by your doctor?

- ◯ Yes
- ◯ No

In total, how many pills do you take on a regular basis?
(Please do not include pills you take only once in a while)

- ◯ None
- ◯ One
- ◯ Two to five
- ◯ Six to ten
- ◯ More than ten

How many different times a day do you regularly take pills?

- ◯ None
- ◯ Once per day
- ◯ 2 times a day
- ◯ 3 times a day
- ◯ More than 3 times a day

Do you find it inconvenient to take this medicine?
Please specify if you think it is inconvenient.

What is your opinion about taking this medicine?

- ◯ I don't mind taking them because I have to take them for a reason.
- ◯ I do not like taking them because they remind me that I am not perfectly healthy.
- ◯ I have no opinion.
- ◯ Other (please specify)

For peer review only

We would like to ask questions about you to better understand your choices.

What is your gender?

- ◯ Male
- ◯ Female
- ◯ Other (please specify) [ ]

Which age category do you fall into?

- ◯ 34 years or younger
- ◯ 35-44 years
- ◯ 45-54 years
- ◯ 55-64 years
- ◯ 65-74 years
- ◯ 75-84 years
- ◯ 85 years or over

Which best reflects your ethnic group?

- ◯ White British/Irish
- ◯ White other
- ◯ Mixed/Multiple ethnic origins
- ◯ Black/African/Caribbean/Black British
- ◯ Asian/Asian British
- ◯ Other (please specify) [ ]

0% 100%

What is your occupational status?

- ◯ Employed full-time
- ◯ Employed part-time
- ◯ Self-employed
- ◯ Unemployed
- ◯ Retired
- ◯ Looking after a home/family
- ◯ Student
- ◯ Freelance or temping
- ◯ Long-term sickness

Could you tell us what your occupation is?
(Previous occupation if retired.)

What is the highest level of education you have obtained?
(for example, GCSEs, Degrees, etc.)

What is your religion?

- ◯ No religion
- ◯ Christian
- ◯ Buddhist
- ◯ Jewish
- ◯ Hindu
- ◯ Muslim
- ◯ Sikh
- ◯ Other

0% 100%

How do you feel about numbers?

This section will ask you some questions to understand how familiar you are with numbers and probabilities.

If you are unsure of the answer, you can leave the response blank.

0% 100%

For peer review only

Question 1

Imagine I flip a coin 1,000 times.

What is your best guess about how many times the coin would fall heads up in 1,000 flips?

- Answer: ________
- Prefer not to answer
- Don't know.

Question 2

If there was a lottery where the chance of winning £10 is 1%, if 1,000 people bought a ticket, how many would you expect to win?

- Answer: ________
- Prefer not to answer
- Don't know.

Question 3

In another lottery, the chance of winning a car is 1 in 1,000.

What percentage of tickets in the lottery will win a car?

- Answer: ________ **%**
- Prefer not to answer
- Don't know

0% 100%

How confident are you that you would answer the questions the same if faced with the situations in real-life?

	Very confident	Confident	Not sure	Not confident	Not confident at all	
Confident	◯	◯	◯	◯	◯	Not confident at all

How easy or difficult did you find making choices between alternatives?

	Very easy	Easy	Average	Somewhat difficult	Very difficult	
Easy	◯	◯	◯	◯	◯	Difficult

How easy or difficult did you find the survey to understand?

	Very easy	Easy	Average	Somewhat difficult	Very difficult	
Easy	◯	◯	◯	◯	◯	Difficult

0% 100%

eview only

Was there anything in the survey that was particularly unclear?

(Please write in the box below)

Is there anything else about the survey we could change to make it easier to uncerstand (for example, phrasing and explanation)?

(Optional) If you have any further comments or suggestions, please write them here.

0% 100%

y only

Thank you for completing this survey

If you would like more information about osteoporosis please visit the NHS Choices website here.

MANCHESTER
1824
The University of Manchester

0% 100%

For peer review only

Note:

When respondents take the survey in regular mode this page will not be displayed. Respondents will be redirected to the link below:

https://dkr1.ssisurveys.com/projects/end?rst=1&psid= [Script] &basic=99423

0% 100%

For peer review only

Note:

When respondents take the survey in regular mode this page will not be displayed. Respondents will be redirected to the link below:

https://dkr1.ssisurveys.com/projects/end?rst=2&psid= [Script]

0% 100%

For peer review only

Note:

When respondents take the survey in regular mode this page will not be displayed. Respondents will be redirected to the link below:

https://dkr1.ssisurveys.com/projects/end?rst=3&psid= [Script]

0% 100%

For peer review only

Supplementary Appendix 1: The statin survey

See separate pdf file

For peer review only

Supplementary Appendix 2: The bisphosphonate survey

See separate pdf file

For peer review only

Supplementary Appendix 3: Training materials for statin survey

Statins for the prevention of CVD

Bobbie has been to see a doctor.

The doctor has said Bobbie needs to start a medicine to prevent serious events because of his heart disease.

We will take you through Bobbie's story.

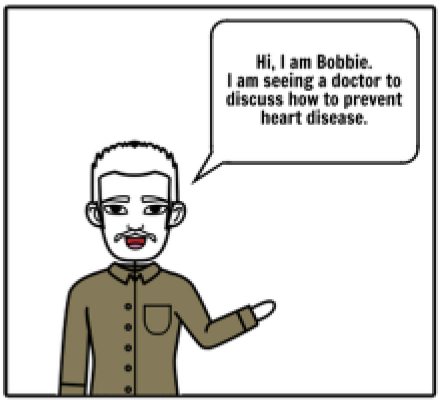

What is heart disease?

Bobbie hears about heart disease from his GP.

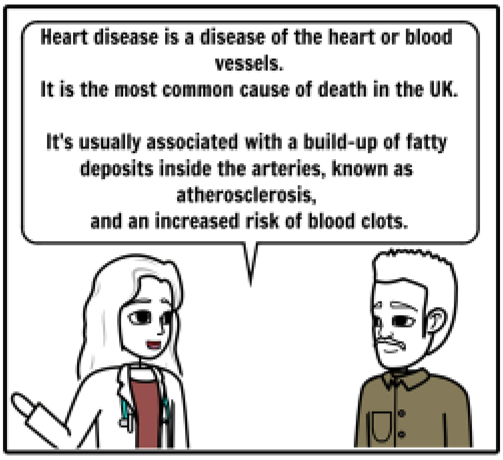

Impact of heart disease

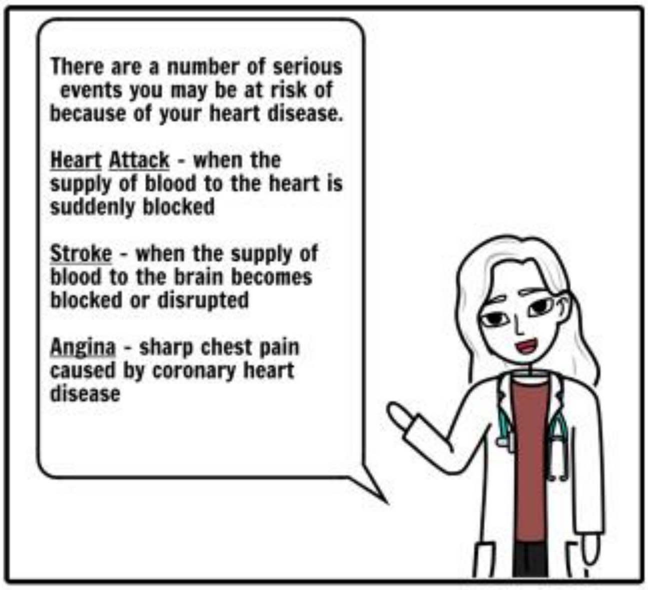

Risk of Heart disease

The GP explains that there is a chance that because of his cholesterol level Bobbie is at risk of developing one of these serious events in the future.

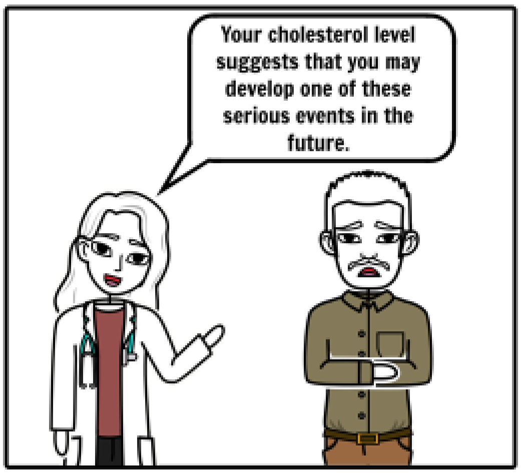

What is risk?

- **Risk** is: a term used to explain the chance that something bad might happen.

- **10% risk** means:

  Out of every **1,000** people, with heart disease,

  100 people would develop a heart attack, and **900** people would not.

What is a 10% risk?

- This diagram shows a 10% risk of having a heart attack because of their heart disease.

- The people shaded blue are the ones who have a heart attack.

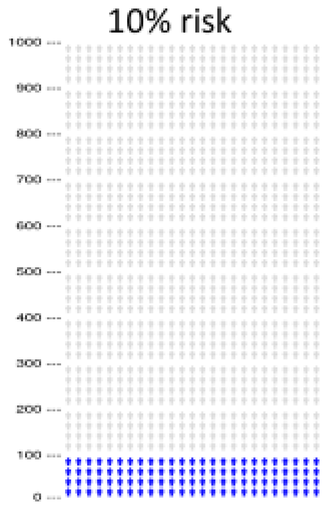

Prevention of serious events from heart disease

- Bobbie's doctor can estimate how likely it is that he will develop heart disease over the next 10 years.
- The estimate is based on things such as Bobbie's age and sex, family medical history, blood pressure and cholesterol level.
- The doctor may prescribe certain medicines such as statins to help reduce the chance of Bobbie having heart disease.
- The medicine will prevent some of the people who take it from having a serious event because of the heart disease.
- A serious event from the heart disease will still happen to some of the people who take the medicine.

Prevention of serious events from heart disease

The doctor prescribes Bobbie a medicine to reduce the chance of his heart disease causing a heart attack or other heart conditions in the future.

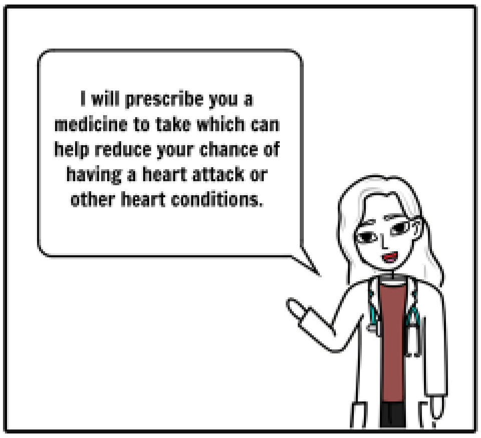

Collecting the medicine from the pharmacy

Bobbie goes to a pharmacy to collect his medicine.

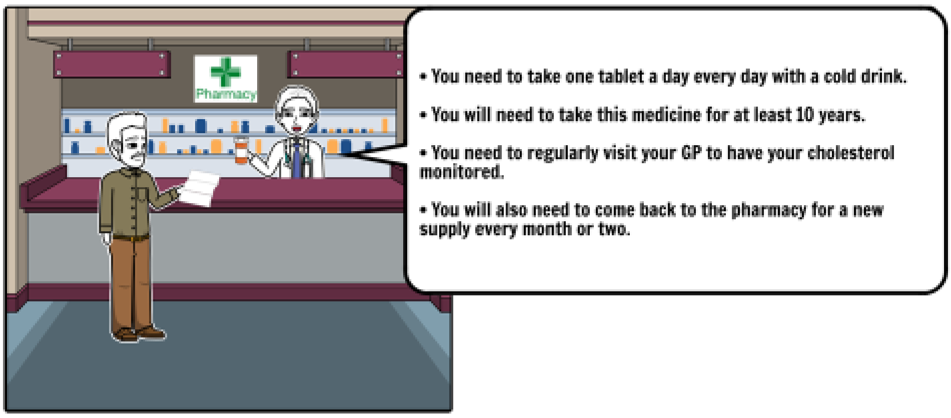

Taking the medicine for prevention

Bobbie goes home with his new tablets.

He then has to decide whether he wants to take them as the doctor and the pharmacist have advised.

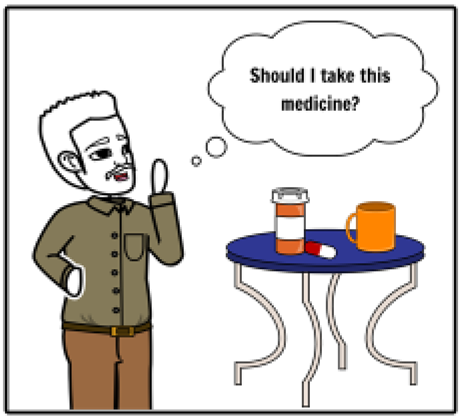

- Bobbie may consider three key things, when deciding whether to take the medicine.

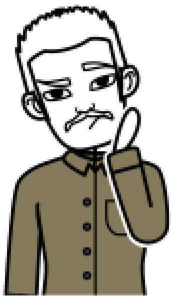

Effectiveness of the medicine

- How good the medicine is in terms of preventing a condition like a heart attack

Side effects of the medicine

- Whether there is any potential harm from the medicine

Inconvenience of the medicine

- Whether taking the medicine would fit in his lifestyle or cause him inconvenience

How effective is the medicine?

- Some people, but not everyone, who take the medicine will avoid a serious event, such as a heart attack or stroke as a result of taking the tablet.

- The effectiveness of the medicine can be described in terms of a reduction in this risk.

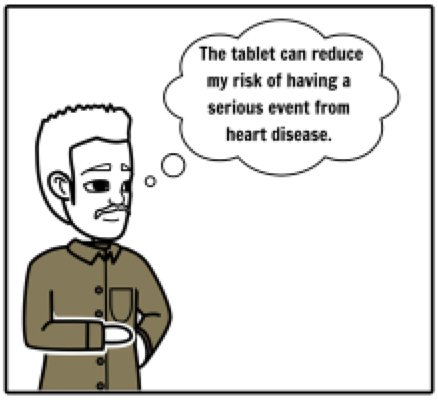

What does ‘reduction in risk’ mean?

- A ‘reduction in risk from 10% to 6%’ means:

The medicine will reduce your risk of experiencing an event, such as a heart attack, within the next 10 years from 10% to 6%.

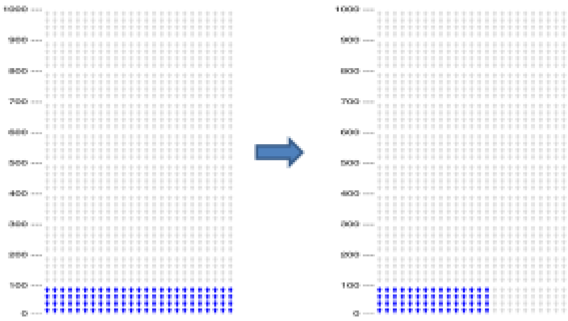

No treatment: 10% risk

Taking the medicine: 6% risk

Does the medicine have side effects?

The potential for side effects from the medicine can be described in terms of the risk of a side effect.

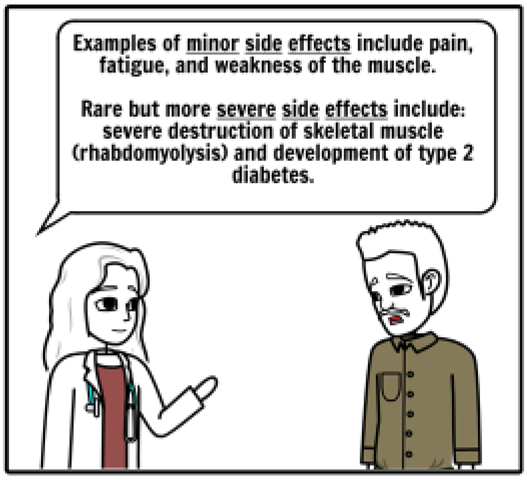

Risk of a minor side effect from the medicine

- A 5% risk of a **minor** side effect means:

Among every 1,000 people taking the medicine,

50 will experience a minor side effect such as either: pain, fatigue, and weakness of the muscle.

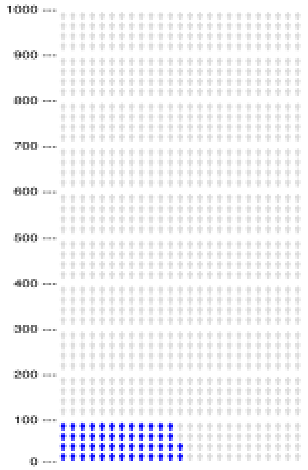

5% risk of a minor side effect

Risk of a severe side effect from the medicine

- A 0.1% risk of a **severe** side effect means:

Among every 1,000 people taking the medicine,

1 will experience a severe side effect such as either: severe destruction of skeletal muscle or development of type 2 diabetes.

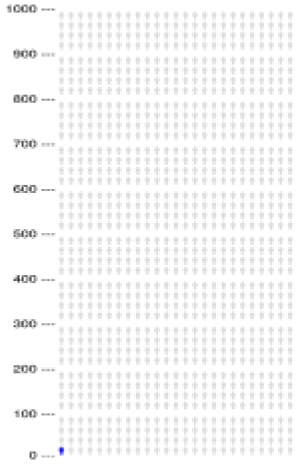

0.1% risk of a severe side effect

How inconvenient is it when taking the medicine?

Bobbie also thinks about what it means to have to take this medicine for the next ten years.

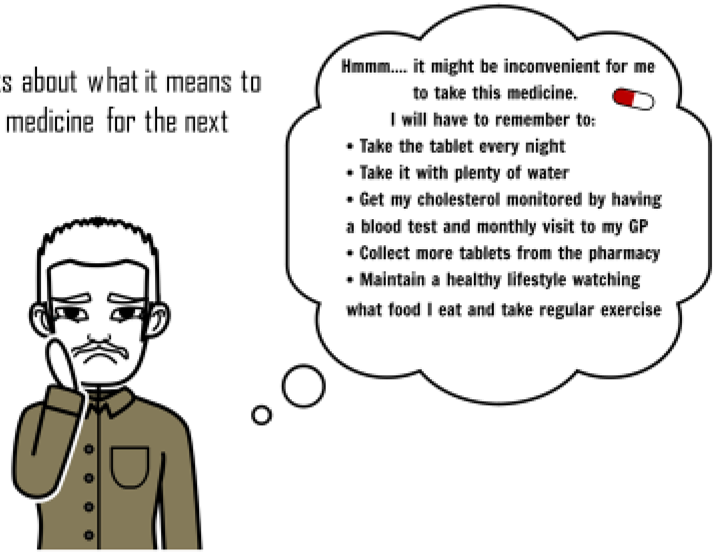

In the questions that follow in this survey, we will ask you about how you think about taking a medicine to prevent heart disease.

Appendix 4: Training materials for bisphosphonates survey

Bisphosphonates for the prevention of osteoporotic fractures

Alex has been to see a doctor who told her that she has weak bones.

The doctor has said Alex needs to start a medicine to prevent broken bones (fractures) because of her weak bones.

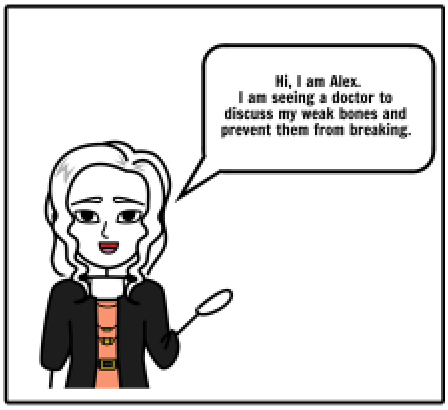

We will take you through Alex's story.

What is osteoporosis?

Alex hears about osteoporosis from her GP.

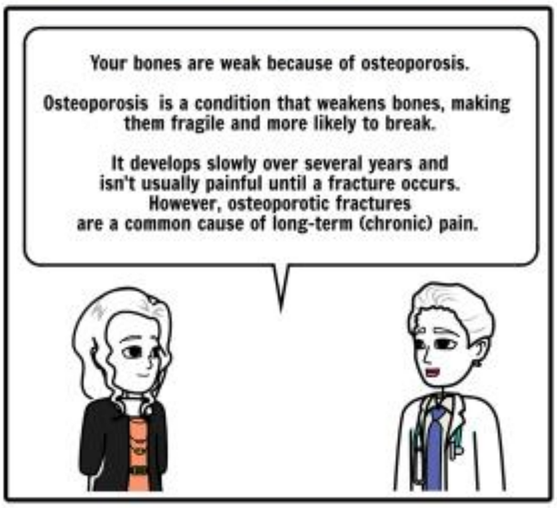

Impact of osteoporosis

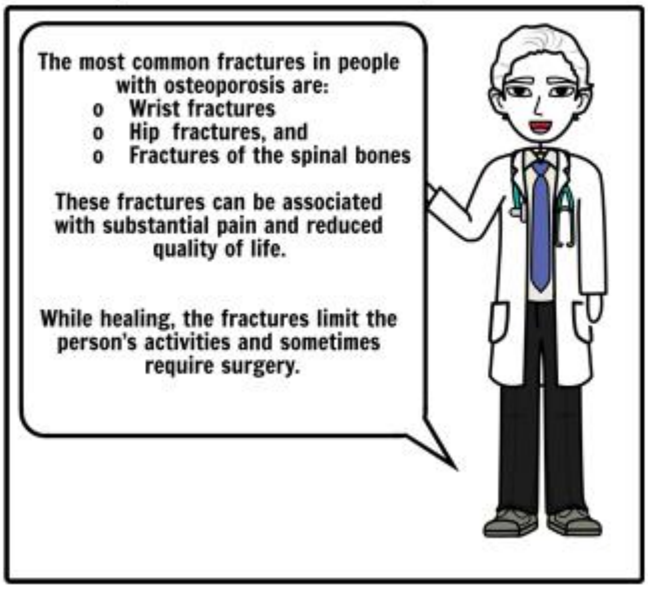

Risk of osteoporotic fractures

Her GP explains that Alex has some risk of fracture as her family has a history of hip fracture.

Also, Alex has a low body mass index (BMI). BMI is a measure of body fat.

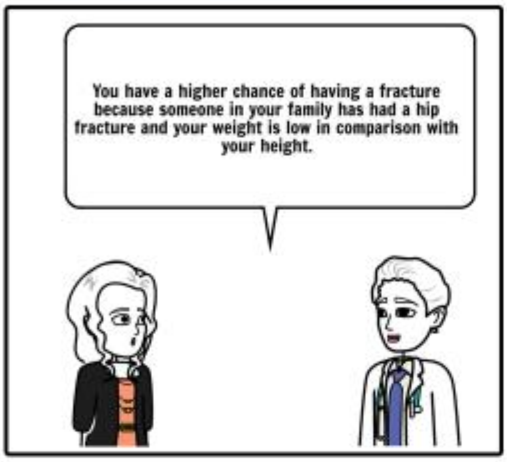

What is risk?

- **Risk** is: a term used to explain the chance that something bad might happen.

- **10% risk** means:

  Out of every **1,000** people, with osteoporosis,

  100 people would have a fracture, and **900** people would not.

What is a 10% risk?

- This diagram shows a 10% risk of having a fracture because of their osteoporosis.

- The people shaded blue are the ones who have a fracture.

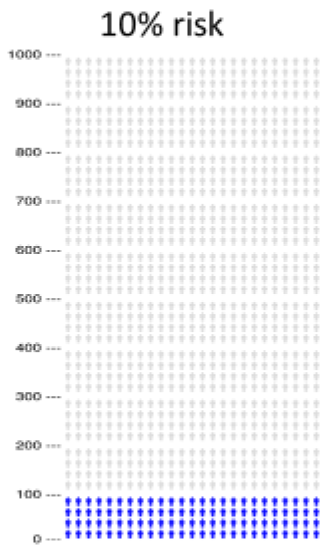

Prevention of fracture from osteoporosis

- Alex's doctor can estimate how likely it is that she will experience osteoporotic fracture over the next 5 years.
- The estimate is based on things such as Alex's age and sex, ethnicity, family fracture history, and body mass index.
- The doctor may prescribe certain medicines such as bisphosphonates to help reduce the chance of Alex having a fracture.
- The medicine will prevent some of the people who take it from having a fracture because of osteoporosis.
- Fractures may still happen in some of the people who take the medicine.

Prevention of fractures from osteoporosis

The doctor prescribes Alex a medicine to reduce the risk of her having a fracture in the future because of her osteoporosis.

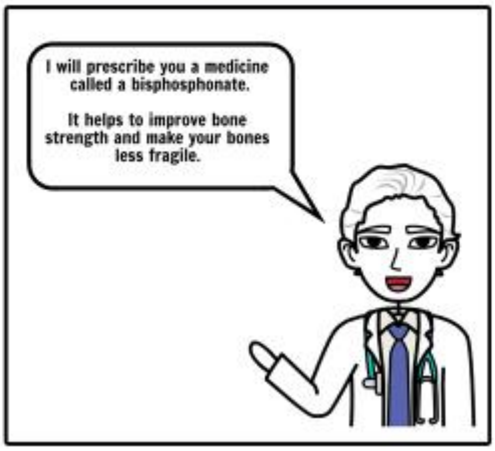

Collecting the medicine from the pharmacy

The pharmacist explains to Alex how to take the medicine.

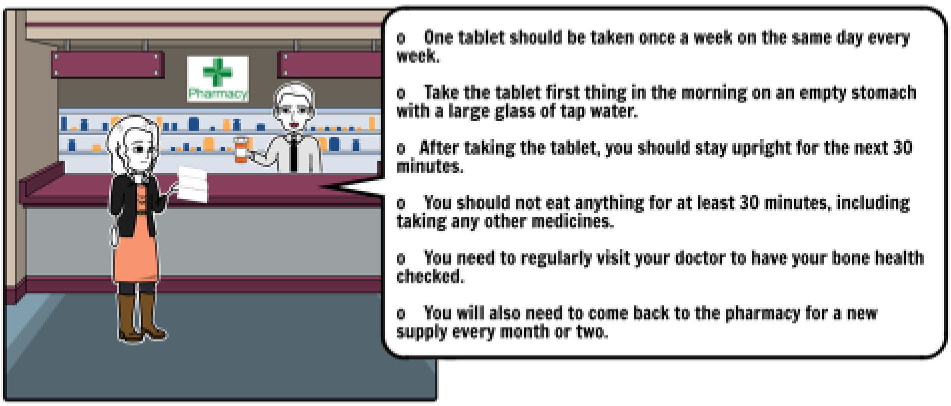

Taking the medicine for prevention

Alex goes home with her new tablets.

She then considers whether she wants to take them as the doctor and the pharmacist have advised.

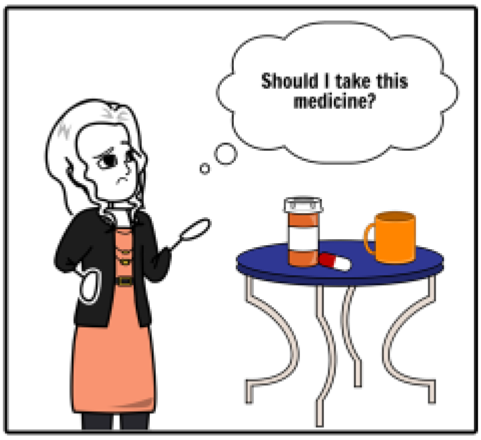

Alex may consider *three key things*, when deciding whether to take the medicine.

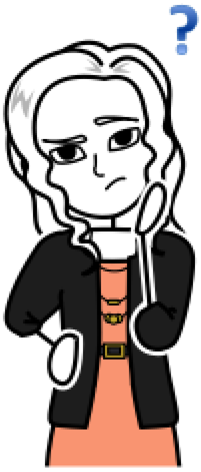

Effectiveness of the medicine

- How good the medicine is in terms of preventing a fracture from osteoporosis

Side effects of the medicine

- Whether there is any potential harm from the medicine

Inconvenience of the medicine

- Whether taking the medicine would fit in her lifestyle or cause her inconvenience

How effective is the medicine?

- Some people, but not everyone, who take the medicine will avoid a serious event, such as hip fracture, as a result of taking the tablet.
- The effectiveness of the medicine can be described in terms of a reduction in this risk.

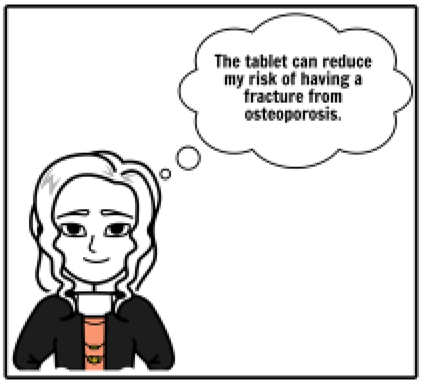

What does 'reduction in risk' mean?

- A 'reduction in risk from 10% to 6%' means:

The medicine will reduce your risk of experiencing a fracture, such as hip fracture, within the next 5 years from 10% to 6%.

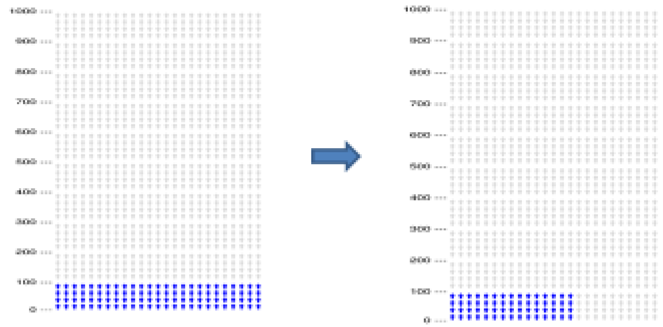

No treatment: 10% risk

Taking the medicine: 6% risk

Does the medicine have side effects?

The potential for side effects from the medicine can be described in terms of the **risk of a side effect**.

Alex hears about minor but more common side effects & severe but rarer side effects from her GP.

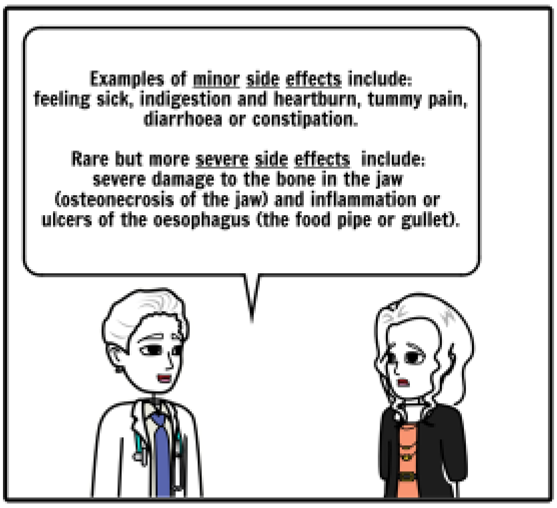

Risk of a minor side effect from the medicine

- A 5% risk of a minor side effect means:

Among every **1,000** people taking the medicine, **50** will experience a minor side effect such as either: feeling sick, indigestion, diarrhoea or constipation.

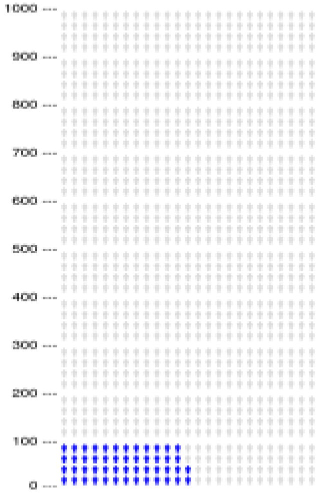

5% risk of a side effect

Risk of a severe side effect from the medicine

- A 0.3% risk of severe side effect means:

Among every **1,000** people taking the medicine, **3** will experience a severe side effect such as severe loss or destruction of the bone in the jaw.

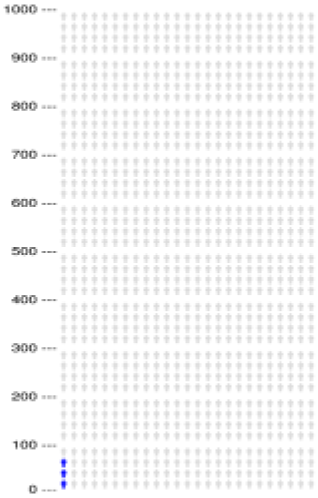

0.3% risk of a side effect

How inconvenient is it when taking the medicine?

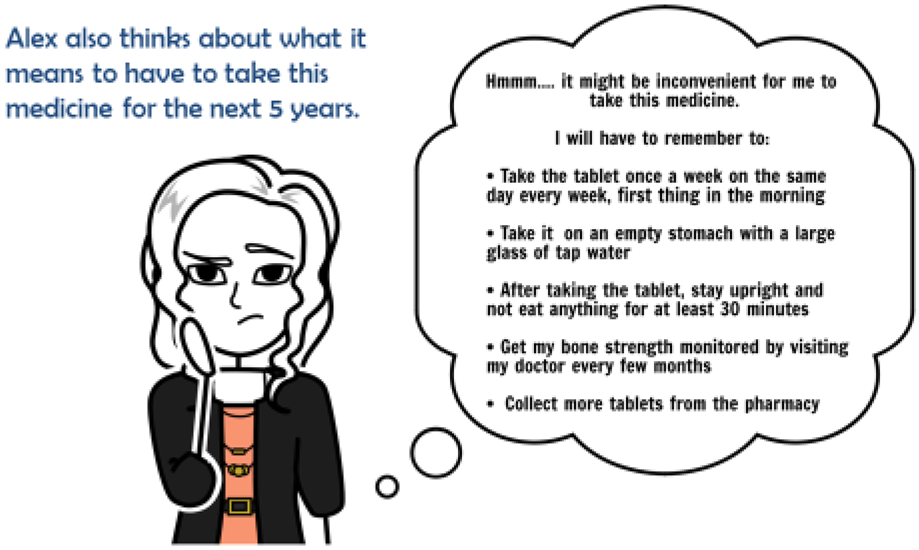

In the questions that follow in this survey, we will ask you about how you think about taking a medicine to prevent osteoporotic fracture.

Supplementary Appendix 5: Description of the whole sample characteristics

	**Statin survey**			**Bisphosphonate survey**			
	Patient[a]	Public[b]	Total	Patient[a]	Public[b]	Total	Total
	N=260	N=376	N=636	N=110	N=359	N=469	N= 1105
**Age**							
Less than 35	2 (1.2%)	25 (7.9%)	27 (5.5%)	0 (0.0%)	26 (8.8%)	26 (7.5%)	53 (6%)
35-44	5 (2.9%)	74 (23.3%)	79 (16.2%)	1 (1.9%)	51 (17.3%)	52 (14.9%)	131 (16%)
45-54	10 (5.9%)	57 (17.9%)	67 (13.7%)	3 (5.7%)	41 (13.9%)	44 (12.6%)	111 (13%)
55-64	49 (28.8%)	84 (26.4%)	133 (27.3%)	14 (26.4%)	57 (19.3%)	71 (20.4%)	204 (24%)
65-74	77 (45.3%)	70 (22.0%)	147 (30.1%)	20 (37.7%)	107 (36.3%)	127 (36.5%)	274 (33%)
75+	27 (15.9%)	8 (2.5%)	35 (7.2%)	15 (28.3%)	13 (4.4%)	28 (8.0%)	63 (8%)
Missing	90	58	148	57	64	121	269
**Sex**							
Female	57 (33.5%)	159 (50.0%)	216 (44.3%)	46 (86.8%)	185 (62.9%)	231 (66.6%)	447 (54%)
Male	113 (66.5%)	159 (50.0%)	272 (55.7%)	7 (13.2%)	109 (37.1%)	116 (33.4%)	388 (46%)
Missing	90	58	148	57	65	122	270
**Ethnicity**							
White British/Irish	160 (94.1%)	288 (90.6%)	448 (91.8%)	51 (96.2%)	263 (89.2%)	314 (90.2%)	762 (91%)
White other	4 (2.4%)	16 (5.0%)	20 (4.1%)	1 (1.9%)	12 (4.1%)	13 (3.7%)	33 (4%)
Mixed/Multiple ethnic origins	0 (0.0%)	2 (0.6%)	2 (0.4%)	0 (0.0%)	5 (1.7%)	5 (1.4%)	7 (1%)
Black/African/Caribbean/Black British	0 (0.0%)	3 (0.9%)	3 (0.6%)	0 (0.0%)	4 (1.4%)	4 (1.1%)	7 (1%)
Asian/Asian British	0 (0.0%)	7 (2.2%)	7 (1.4%)	0 (0.0%)	9 (3.1%)	9 (2.6%)	16 (2%)
Chinese	0 (0.0%)	1 (0.3%)	1 (0.2%)	1 (1.9%)	2 (0.7%)	3 (0.9%)	4 (%)
Other ethnicity	6 (3.5%)	1 (0.3%)	7 (1.4%)	0 (0%)	0 (0%)	0 (0%)	(%)
Missing	90	58	148	57	64	121	269
**Number of pills taken daily**							
None	0 (0.0%)	133 (41.8%)	133 (27.3%)	0 (0.0%)	99 (33.6%)	99 (28.4%)	232 (28%)
One	4 (2.4%)	73 (23.0%)	77 (15.8%)	6 (11.3%)	51 (17.3%)	57 (16.4%)	134 (16%)
Two to five	123 (72.4%)	87 (27.4%)	210 (43.0%)	32 (60.4%)	109 (36.9%)	141 (40.5%)	351 (42%)
Six to ten	36 (21.2%)	18 (5.7%)	54 (11.1%)	9 (17.0%)	23 (7.8%)	32 (9.2%)	86 (10%)
More than ten	7 (4.1%)	7 (2.2%)	14 (2.9%)	6 (11.3%)	13 (4.4%)	19 (5.5%)	33 (4%)
Missing	90	58	148	57	64	121	269
**Number of different times pill taken per day**							
None	3 (1.8%)	134 (42.1%)	137 (28.1%)	0 (0.0%)	97 (32.9%)	97 (27.9%)	234 (28%)
Once per day	40 (23.5%)	104 (32.7%)	144 (29.5%)	22 (41.5%)	100 (33.9%)	122 (35.1%)	266 (32%)
2 times a day	100 (58.8%)	61 (19.2%)	161 (33.0%)	20 (37.7%)	68 (23.1%)	88 (25.3%)	249 (30%)
3 times a day	23 (13.5%)	13 (4.1%)	36 (7.4%)	7 (13.2%)	24 (8.1%)	31 (8.9%)	67 (8%)
More than 3 times a day	4 (2.4%)	6 (1.9%)	10 (2.0%)	4 (7.5%)	6 (2.0%)	10 (2.9%)	20 (2%)
Missing	90	58	148	57	64	121	269
**EQ-5D-3L utility**	0.820 (0.2)	0.803 (0.3)	0.810 (0.3)	0.771 (0.2)	0.790 (0.2)	0.786 (0.2)	0.800 (0.3)
Missing	41	38	79	35	41	76	155

For peer review only

Supplementary Appendix 6: Propensity for dominant preference

Male	0.988
	[0.716, 1.364]
White other	1.263
	[0.594,2.684]
Mixed/Multiple ethnic	0.370
	[0.043,3.171]
Black/African/Caribbean/Bla	0.903
	[0.156,5.244]
Asian/Asian British	1.230
	[0.329, 4.604]
Chinese	0.748
	[0.077, 7.290]
Other ethnicity	0.568
	[0.066, 4.902]
Less than 35	0
	[.,.]
35-44	0.709
	[0.377, 1.333]
45-54	0.521
	[0.265, 1.023]
55-64	0.507
	[0.262,0.978]
65-74	0.480
	[0.2540, 0.907]
75+	0.962
	[0.399, 2.318]
Public	1.544*
	[1.047, 2.278]
None	0
	[.,.]
Once per day	1.092
	[0.600,1.986]
2 times a day	0.860
	[0.417,1.775]
3 times a day	1.077
	[0.427,2.715]
More than 3 times a day	2.797
	[0.819,9.554]
None	0
	[.,.]
One	1.0211
	[0.507,2.055]
Two to five	0.875
	[0.446,1.716]
Six to ten	1.012
	[0.386,2.654]
More than ten	1.263
	[0.451,3.536]
Statin sample	0.927
	[0.678,1.268]
Constant	0.367*
	[0.135,1.00]
Observations	1105

For peer review only

Supplementary Appendix 7: Root mean squared error (RMSE) for the competing models to fit TTO data

	Statins			Bisphosphonates		
Imputation number	OLS[1]	ZOIB[2]	MBR[3]	OLS[1]	ZOIB[2]	MBR[3]
Imputation 1	0.0692	0.0694	0.0699	0.0706	0.0719	0.0716
Imputation 2	0.0697	0.0703	0.0695	0.0717	0.0712	0.0718
Imputation 3	0.0693	0.0709	0.0715	0.0718	0.0721	0.0717
Imputation 4	0.0697	0.0705	0.0721	0.0706	0.0722	0.0733
Imputation 5	0.0698	0.0688	0.0717	0.0717	0.0693	0.0734
Mean	0.0695	0.0700	0.0709	0.0713	0.0713	0.0723

[1] Ordinary least squares; [2] Zero inflated beta regression; [3] Mixed beta regression

For peer review only

BMJ Open

Quantifying the impact of taking medicines for primary prevention: a time-trade off study to elicit direct treatment disutility in the UK

Journal:	*BMJ Open*
Manuscript ID	bmjopen-2022-063800.R1
Article Type:	Original research
Date Submitted by the Author:	30-Mar-2023
Complete List of Authors:	Thompson, Alexander; The University of Manchester<br>Youn, Ji-Hee; The University of Manchester<br>Guthrie, Bruce; University of Edinburgh College of Medicine and Veterinary Medicine,<br>Hainsworth, Robert; The University of Manchester<br>Donnan, Peter; University of Dundee, Dundee Epidemiology and Biostatistics Unit<br>Rogers, Gabriel; The University of Manchester<br>Morales, Daniel; University of Dundee, Division of Population Health Sciences<br>Payne, Katherine; The University of Manchester, Health Economics at Manchester
**Primary Subject Heading**:	Health economics
Secondary Subject Heading:	Patient-centred medicine, Research methods, Health economics, Communication, Cardiovascular medicine
Keywords:	Health economics < HEALTH SERVICES ADMINISTRATION & MANAGEMENT, Coronary heart disease < CARDIOLOGY, PREVENTIVE MEDICINE

SCHOLARONE™
Manuscripts

I, the Submitting Author has the right to grant and does grant on behalf of all authors of the Work (as defined in the below author licence), an exclusive licence and/or a non-exclusive licence for contributions from authors who are: i) UK Crown employees; ii) where BMJ has agreed a CC-BY licence shall apply, and/or iii) in accordance with the terms applicable for US Federal Government officers or employees acting as part of their official duties; on a worldwide, perpetual, irrevocable, royalty-free basis to BMJ Publishing Group Ltd ("BMJ") its licensees and where the relevant Journal is co-owned by BMJ to the co-owners of the Journal, to publish the Work in this journal and any other BMJ products and to exploit all rights, as set out in our licence.

The Submitting Author accepts and understands that any supply made under these terms is made by BMJ to the Submitting Author unless you are acting as an employee on behalf of your employer or a postgraduate student of an affiliated institution which is paying any applicable article publishing charge ("APC") for Open Access articles. Where the Submitting Author wishes to make the Work available on an Open Access basis (and intends to pay the relevant APC), the terms of reuse of such Open Access shall be governed by a Creative Commons licence – details of these licences and which Creative Commons licence will apply to this Work are set out in our licence referred to above.

Other than as permitted in any relevant BMJ Author's Self Archiving Policies, I confirm this Work has not been accepted for publication elsewhere, is not being considered for publication elsewhere and does not duplicate material already published. I confirm all authors consent to publication of this Work and authorise the granting of this licence.

For peer review only

Title: Quantifying the impact of taking medicines for primary prevention: a time-trade off study to elicit direct treatment disutility in the UK

Running title: Estimating values for direct treatment disutility

Authors:

Alexander J Thompson, PhD[1a*] (alexander.thompson@manchester.ac.uk)

Ji-Hee Youn, PhD[1a]

Bruce Guthrie, MB BChir PhD[3]

Robert Hainsworth, MSc[1]

Peter Donnan, PhD[2]

Gabriel Rogers[1]

Daniel Morales, PhD[2]

Katherine Payne, PhD[1]

[a] joint first authors

[1] *Manchester Centre for Health Economics, Division of Population Health, Health Services Research and Primary Care, University of Manchester*

[2] *Division of Population Health and Genomics, University of Dundee*

[3] *Usher Institute, College of Medicine and Veterinary Medicine, University of Edinburgh*

**Corresponding author: Alexander Thompson, Floor 4, Jean Mcfarlane Building, Oxford Road, The University of Manchester, Manchester, M13 9PL, UK*

e-mail: alexander.thompson@manchester.ac.uk

ORCiD: 0000-0003-4930-5107

Keywords: direct treatment disutility, cardiovascular disease, statins, osteoporosis, bisphosphonates

Word Count: 3637

For peer review only

Abstract

Direct treatment disutility (DTD) represents an individual's disutility associated with the inconvenience of taking a medicine over a long period of time.

Objectives: The main aim of this study was to elicit DTD values for taking a statin or a bisphosphonate for primary prevention. A secondary aim was to understand factors which influence DTD values.

Methods

Design: We used a cross-sectional study consisting of time-trade off (TTO) exercises embedded within online surveys. Respondents were asked to compare a one-off pill ('Medicine A') assumed to have no inconvenience and a daily pill ('Medicine B') over ten-years (statins) or five-years (bisphosphonates).

Setting: Individuals from NHS primary care and the general population were surveyed using an online panel company.

Participants: Two types of participants were recruited. First, a purposive sample of patients with experience of taking a statin (n=260) or bisphosphonate (n=100) were recruited from an NHS sampling frame. Patients needed to be aged over 30, have experience of taking the medicine of interest and have no diagnosis of dementia or of using dementia drugs. Second, a demographically balanced sample of members of the public were recruited for statins (n=376) and bisphosphonates (n=359).

Primary and secondary outcome measures: Primary outcome was mean direct treatment disutility. Regression analysis explored factors which could influence DTD values.

Results: A total of 879 respondents were included for analysis (514 for statins and 365 for bisphosphonates). The majority of respondents reported a disutility associated with medicine use. Mean DTD for statins was 0.034 and for bisphosphonates 0.067 respectively. Respondent characteristics including age and sex did not influence DTD. Experience of bisphosphonate-use reduced reported disutilities.

Conclusions: Statins and bisphosphonates have a quantifiable direct treatment disutility. The size of estimated disutilities suggest they are likely to be important for cost-effectiveness, particularly in individuals at low-risk when treated for primary prevention.

For peer review only - http://bmjopen.bmj.com/site/about/guidelines.xhtml

Strength and limitations

- We use a large representative sample of both public and patients for our estimated values.
- We used patients to inform and trial our survey and designed storyboards and visual arrays to contextualize the exercises and communicate absolute risk.
- The time trade-off methods employed were consistent with those first used to derive the EQ-5D-3L, a widely used measure of health-related quality of life.
- Survey respondents self-selected and this could be related to their ability to complete the survey or their self-reported health.

For peer review only

1 Introduction

Medicines for the primary prevention of disease are typically taken long-term, often for life, following identification that an individual is at risk of a harmful event, such as a stroke due to cardiovascular disease (1) or a hip fracture as a result of osteoporosis (2). Taking a medicine is considered, in general, to have a minimal day-to-day treatment burden because, in the traditional clinical sense, the action of medicine-taking is perceived to be non-invasive. Yet there is qualitative evidence to suggest that taking a medicine long-term is associated with treatment burden (3). Moreover, supporting this qualitative evidence is a quantitative literature seeking to estimate the utility of pill-taking, or alternatively, the negative impact of undergoing long-term treatment with medicines, called 'direct treatment disutility'.

Direct treatment disutility (DTD) represents an individual's strength of preference not to take a medicine. DTD could occur for a number of reasons including the inconvenience of obtaining prescriptions and medicines, needing to modify lifestyles to take medicines and attending healthcare visits for monitoring treatment (4). DTD therefore can be considered in addition to the potential impact adverse drug reactions could have on an individual's utility and/or the out-of-pocket costs (financial burden) medicines may incur. To date, the quantification of DTD in the literature has focused primarily on long-term medicines for cardiovascular disease with values estimated using hypothetical thought experiments such as 'time trade-off' (TTO) exercises where respondents are often asked whether they would forego some length of life not to undergo treatment (5–7). Estimates from this literature suggest that a substantial proportion of people would be willing to trade some length of life or risk of death not to take a long-term preventative treatment (5–7).

A previous literature review in 2015 (4) highlighted the small number of published cost-effectiveness analyses (CEA) that have included DTD values. Where DTD was incorporated in analysis, it was an important factor impacting upon cost-effectiveness results making primary preventative treatments, such as statins, much less likely to be cost-effective (8–14). In these analyses, DTD values were often assumed (10,13,14) rather than based on empirical estimates. Meanwhile, the small number of previous empirical DTD studies, likely for reasons of practicality, have either adopted small study sizes (5) or sampling

For peer review only

frames which are either not representative of patients (7) or the general population (6). More recently, further cost-effectiveness analyses (15–18) and other decision-making approaches (19,20) have incorporated DTD values, and found them to be highly influential on results, yet have had to rely on DTD values sourced from the same limited set of empirical studies.

The aim of this study was to build upon the previous empirical literature and elicit values of DTD for long-term primary preventative medicines from a mixed sample of the general population and patients. We focus on two populations who might require long-term treatment with medicines: those taking statins for the primary prevention of cardiovascular disease (CVD) and bisphosphonates for the primary prevention of bone fractures. A secondary aim of this study was to explore if survey participant characteristics were associated with the elicited DTD values.

2 Methods

Ethical approval for this study was granted by an NHS Health Research Authority Research Ethics Committee (REC reference: 17/NW/0124; project number: 220492). Respondents gave informed consent after reading a participant information sheet online and indicating they agreed to participate in the survey online.

There is no agreement on the appropriate method to elicit DTD (5–7). This study used cross sectional surveys, based on a single time point in each survey, to conduct time trade off valuation exercises (21,22). This valuation method is a widely recognised and has been the standard approach for eliciting utility values to generate tariffs used by national decision-making bodies such as the National Institute for Health and Care Excellence (NICE) (23) who have used the EQ-5D-3L to quantify health-related quality of life, valued using a time trade-off exercise (24).

For peer review only - http://bmjopen.bmj.com/site/about/guidelines.xhtml

2.1 Selection of medicine examples

Statins for primary prevention of CVD was selected because it is an example of an orally administered medicine that is perceived to be benign, but which some people perceive as harmful. Bisphosphonates for the primary prevention of bone fractures was selected because it is an example of a medicine that has an obvious potential impact on day-to-day life. Patients undergoing treatment with bisphosphonates must take the medication on an empty stomach, drink a full glass of water, stand for 30 minutes after taking the medication and avoid food and drink for a further 2 hours.

2.2 Patient and public involvement

The genesis for the concept of direct treatment disutility (DTD) was informed by previous research conducted with patients exploring the impact of medications in those with multimorbidity (25). To quantify DTD, two patient experts contributed experience of taking medicines long-term, alongside clinical input from the research team, to develop the description of the health (medicine-taking) states used in this study. Separate online surveys were designed for the two selected medicine examples (see Supplementary Appendix 1 for the statin survey and Supplementary Appendix 2 for the bisphosphonate survey). Pilot studies were conducted for each survey. First, a qualitative pilot study, using the think-aloud method (26,27) from a sample of patients from a GP practice in Greater Manchester was used to understand whether the surveys were sufficiently clear for respondents to complete as intended. Second, a quantitative pilot study with 30 respondents (for statin survey and bisphosphonate survey) identified by panel company Dynata was used to allow a preliminary analysis of data collected from the valuation exercise. No changes were made following the quantitative pilot study. The final surveys were formatted and administered online using Sawtooth software (28). Respondents were sent a secure link to complete a survey.

2.3 Study sample

Two purposive samples of patients taking a statin or bisphosphonate were recruited from two sampling frames: from general practices via the NHS Research Scotland Primary Care Network (29) and the Scottish Health Research Register (SHARE – a register of people living in Scotland allowing recruitment after a search of their medical records)(30). For both, the inclusion criteria for recruiting patients with

experience of taking a statin (bisphosphonate) were: prescription of a statin (or a bisphosphonate) in the last year; aged 30 years and over; not been diagnosed with dementia (ICD10 code: F00, F01, F02, F03, G10, G20, G30, G31.0, F05.1, R54 and all child codes); not taking a dementia drug (all drugs in British National Formulary chapter 0411); not also taking a bisphosphonate (or statin as appropriate). Patients deemed unsuitable for any reason by their general practitioner were also excluded.

A purposive sample of members of the public were recruited using an online panel company (Dynata) (31). This online panel company provided a sample of respondents with pre-defined (age 30 years and over; equal gender-balance) characteristics and a demographically-balanced sample from England and Scotland. There were no exclusion criteria for members of the general public who self-select if they were capable of completing an online survey. Respondents from the public or patients could only take part in either a statins or bisphosphonate survey but not both.

Sample-size calculations for valuation exercises are not well established (32). This study aimed for a pragmatic sample size of a minimum of 500 respondents (250 patients; 250 on-line panel) for each medicine example. This resulted in a target total sample size of 1000 respondents (500 for the statin survey and 500 for the bisphosphonate survey).

2.4 Valuation exercise

The time trade-off method employed was similar in approach to that taken by Hutchins et al (6,7) when valuing the utility of pill taking. See Supplementary Appendix 1 for the whole survey format. The duration within the exercise for daily pill taking was 10-years for statins and 5-years for bisphosphonates. Respondents were asked to imagine taking a pill every day for 10-years (5-years) and then dying. Respondents were then asked whether they would be willing to trade that health state for an alternative where only a single pill was taken at the start of the time-period. In this second health state, however, the respondent would live for a shorter duration and then die. A process of 'iteration' was then used to see whether respondents would trade between the health states of differing lengths until a point of indifference occurs between the two health states. The trade-off being quantified here was whether the participant would be willing to choose to live a shorter life but without daily pill taking or alternatively,

For peer review only - http://bmjopen.bmj.com/site/about/guidelines.xhtml

to have a one-off pill but forego some length of life. The process of iteration to find the potential indifference point followed a standardised process called the Measurement and Valuation of Health protocol whereby a combination of 'bisection' (in which the length of life is always the midpoint of the remaining scale section (bisected)) and 'titration' (in which the length of life is sequentially altered by fixed increments/ decrements). The Measurement and Valuation of Health protocol has been recommended previously to encourage comparability between utility values elicited for the purposes of health technology assessment (32).

The exercises previously described were contextualised with four different background scenarios in order to understand whether DTD values differed depending on the framing of benefits and harms of the medications being used. We asked respondents to consider the exercise with the context that there was: no side-effects (scenario 1) associated with any of the pills; mild side-effects (scenario 2); severe side-effects (scenario 3); and reduced effectiveness (scenario 4). We developed training materials using a story-board approach (32) to enable the communication of the background concepts for respondents completing each survey (see Supplementary Appendix 3 & 4).

2.5 Data analysis

Summary statistics were calculated for a master sample combining data from Scottish Primary Care Research Network (SPCRN) and SHARE ('patient respondents') and Dynata ('public respondents') for both statin and bisphosphonate questionnaires. For each of the four questions per respondent, the estimated utility (or the value attached to a health state of daily medicine use) was calculated as the ratio x/t. In this calculation, x is the final time period, measured in years, whereby participants were indifferent between living in the health states of Medicine A (one pill taken once) and Medicine B. t represents the health state of a pill taken every day for either statins or bisphosphonates measured as 10 years or 5 years respectively (22). The final DTD was calculated by subtracting the estimated utility from 1 or full health. Respondents who indicated they would be unwilling to initiate preventative therapy, by selecting five years for Medicine A (or the lowest time trade-off score attainable of 0.5) in any one of the questions were removed from the dataset. This was because we inferred such respondents to have dominant preferences to not undergo preventative treatment (33,34).

For peer review only - http://bmjopen.bmj.com/site/about/guidelines.xhtml

Missing data for background characteristics as well as for the time trade-off scores were multiply imputed (m=5) using chained equations with predictive mean matching (35). Differences in respondent time trade-off scores associated with medicine type (statins versus bisphosphonates), framing of the survey question (question 1, question 2, question 3, question 4), background characteristics (age, ethnicity, sex) and experience of taking medication (pills taken per day, number of times medication taken per day) were explored using ordinary least squares regression accounting for the multiply imputed datasets. Model specification was informed by summary statistics and kernel density plots of the DTD values. Sensitivity to alternative regression models, were explored with competing models compared using root mean squared error (RMSE). Propensity to trade was explored through a logistic regression for the whole sample with a dummy variable coded 1 for those willing to trade (0.5 <TTO < 1) and 0 for those unwilling to trade (TTO = 1).

For the regression models, a $p < 0.05$ was considered statistically significant. Analysis was carried out in Microsoft Excel and Stata version 16.0 with code available in Supplementary appendix 5.

3 Results

Characteristics for statin respondents (n=514) and bisphosphonate respondents (n=365) who were included in the analysis set are reported in Table 1. Characteristics for the whole sample (n=1105), including those excluded for having dominant preferences (20.1%) are presented in Supplementary appendix 6. Demographic characteristics, and experience of taking medicines was not associated with having dominant preferences to avoid preventative treatment (n=226). However, respondents from the public were more likely to have dominant preferences than those from the public (Supplementary appendix 7).

In the analysis set, patients in the statin survey tended to be older versus those in the public cohort with a higher proportion of male respondents (66.5% versus 50.0%). Compared with their public counterparts, patient respondents also tended to take more pills, at more times of the day, yet surprisingly, they also

For peer review only - http://bmjopen.bmj.com/site/about/guidelines.xhtml

reported a slight improvement in health (EQ-5D-3L utility: 0.827 versus 0.818). Patient respondents in the bisphosphonate sample also tended to be older than those from the public sample but with a higher proportion of females than in the public cohort (89.2% versus 62.4%). Bisphosphonate patient respondents tended to take more pills, at more times of the day versus those in the public. In contrast to the statin survey, bisphosphonate patient respondents reported comparatively lower health than public respondents (EQ-5D-3L utility: 0.779 versus 0.786 respectively).

Figure 1 shows the pre-imputation distributional properties for time trade-off values reported by patients and public, stratified by the question-context, for both the statins and bisphosphonates surveys. As can clearly be observed, the spread of responses from participants was highly variable suggesting individual respondents differed greatly in how negatively they valued taking a pill every day. Due to this high variability, Table 2 presents a wider range of summary statistics than might be typical. Overall, 78% of respondents reported disutility from a taking a pill with a higher proportion indicating some disutility for bisphosphonates (84%) than for statins (73%). Bisphosphonate patients reported much higher mean time trade-off scores than public respondents (difference: 0.024 (se: 0.006)) with this difference being statistically significant. For both statins and bisphosphonates, changing the question context did not alter the mean time trade-off scores by more than 0.01. Irrespective of the type of question or respondent, there was a clear difference between statins and bisphosphonates survey results. Consequently, regression models were run separately on these two cohorts with ordinary least squares regression favoured as it typically produced the least amount of error (Supplementary appendix 8).

For peer review only

Table 1: Description of the sample characteristics

	Statin survey			Bisphosphonate survey			
	Patient[a]	Public[b]	Total	Patient[a]	Public[b]	Total	Total
	N=227	N=287	N=514	N=86	N=279	N=365	N= 879
**Age**							
Less than 35	1 (0.7%)	14 (6.1%)	15 (4.0%)	0 (0.0%)	18 (7.9%)	18 (6.8%)	33 (5.2%)
35-44	4 (2.8%)	44 (19.1%)	48 (12.9%)	1 (2.7%)	42 (18.5%)	43 (16.3%)	91 (14.3%)
45-54	6 (4.2%)	46 (20.0%)	52 (14.0%)	2 (5.4%)	32 (14.1%)	34 (12.9%)	86 (13.5%)
55-64	43 (30.3%)	63 (27.4%)	106 (28.5%)	10 (27.0%)	45 (19.8%)	55 (20.8%)	161 (25.3%)
65-74	66 (46.5%)	60 (26.1%)	126 (33.9%)	15 (40.5%)	80 (35.2%)	95 (36.0%)	221 (34.7%)
75+	22 (15.5%)	3 (1.3%)	25 (6.7%)	9 (24.3%)	10 (4.4%)	19 (7.2%)	44 (6.9%)
Missing	85	57	142	49	52	101	243
**Sex**							
Female	49 (34.5%)	115 (50.0%)	164 (44.1%)	33 (89.2%)	141 (62.4%)	174 (66.2%)	338 (53.2%)
Male	93 (65.5%)	115 (50.0%)	208 (55.9%)	4 (10.8%)	85 (37.6%)	89 (33.8%)	297 (46.8%)
Missing	85	57	142	49	53	102	244
**Ethnicity**							
White British/Irish	133 (93.7%)	211 (91.7%)	344 (92.5%)	36 (97.3%)	203 (89.4%)	239 (90.5%)	583 (91.7%)
White other	3 (2.1%)	10 (4.3%)	13 (3.5%)	1 (2.7%)	8 (3.5%)	9 (3.4%)	22 (3.5%)
Mixed/Multiple ethnic origins	0 (0.0%)	1 (0.4%)	1 (0.3%)	0 (0.0%)	5 (2.2%)	5 (1.9%)	6 (0.9%)
Black/African/Caribbean/Black [illegible]	0 (0.0%)	3 (1.3%)	3 (0.8%)	0 (0.0%)	2 (0.9%)	2 (0.8%)	5 (0.8%)
Asian/Asian British	0 (0.0%)	4 (1.7%)	4 (1.1%)	0 (0.0%)	7 (3.1%)	7 (2.7%)	11 (1.7%)
Chinese	0 (0.0%)	1 (0.4%)	1 (0.3%)	0 (0.0%)	2 (0.9%)	2 (0.8%)	3 (0.5%)
Other ethnicity	6 (4.2%)	0 (0.0%)	6 (1.6%)	0 (0%)	0 (0%)	0 (0%)	6 (0.9%)
Missing	85	57	142	49	52	101	243
**Number of pills taken daily**							
None	0 (0.0%)	91 (39.6%)	91 (24.5%)	0 (0.0%)	77 (33.9%)	77 (29.2%)	168 (26.4%)
One	4 (2.8%)	52 (22.6%)	56 (15.1%)	4 (10.8%)	38 (16.7%)	42 (15.9%)	98 (15.4%)
Two to five	104 (73.2%)	69 (30.0%)	173 (46.5%)	23 (62.2%)	86 (37.9%)	109 (41.3%)	282 (44.3%)
Six to ten	31 (21.8%)	14 (6.1%)	45 (12.1%)	5 (13.5%)	16 (7.0%)	21 (8.0%)	66 (10.4%)
More than ten	3 (2.1%)	4 (1.7%)	7 (1.9%)	5 (13.5%)	10 (4.4%)	15 (5.7%)	22 (3.5%)
Missing	85	57	142	49	52	101	243
**Number of different times pill taken per day**							
None	3 (2.1%)	94 (40.9%)	97 (26.1%)	0 (0.0%)	75 (33.0%)	75 (28.4%)	172 (27.0%)
Once per day	33 (23.2%)	74 (32.2%)	107 (28.8%)	18 (48.6%)	75 (33.0%)	93 (35.2%)	200 (31.4%)
2 times a day	87 (61.3%)	48 (20.9%)	135 (36.3%)	12 (32.4%)	54 (23.8%)	66 (25.0%)	201 (31.6%)
3 times a day	17 (12.0%)	11 (4.8%)	28 (7.5%)	5 (13.5%)	19 (8.4%)	24 (9.1%)	52 (8.2%)
More than 3 times a day	2 (1.4%)	3 (1.3%)	5 (1.3%)	2 (5.4%)	4 (1.8%)	6 (2.3%)	11 (1.7%)
Missing	85	57	142	49	52	101	243
**EQ-5D-3L utility**	0.827 (0.2)	0.818 (0.2)	0.822 (0.2)	0.770 (0.2)	0.786 (0.2)	0.783 (0.2)	0.806 (0.2)
Missing	56	41	97	35	33	68	165

Footnote: [a] patient sample was recruited from general practitioners in the NHS Research Scotland Primary Care Network or the Scottish Health Research Register (SHARE); [b]public sample was

For peer review only - http://bmjopen.bmj.com/site/about/guidelines.xhtml

Table 2: Summary statistics of time trade-off values							
**Medicine**	**Respondent**	**Question context**	**Mean**	**Standard deviation**	**Count**	**p50***	**Proportion reporting disutility**
Statins	Public	No side effects	0.965	0.057	237	0.983	0.746
Statins	Public	Some minor side effects	0.964	0.063	233	0.995	0.721
Statins	Public	Some severe side effects	0.964	0.060	232	0.988	0.725
Statins	Public	Reduced effectiveness	0.964	0.063	232	0.983	0.735
**Statins in the public**			0.964	0.061	934	0.992	0.731
Statins	Patients	No side effects	0.974	0.054	161	0.996	0.718
Statins	Patients	Some minor side effects	0.972	0.056	156	0.996	0.718
Statins	Patients	Some severe side effects	0.968	0.060	150	0.997	0.718
Statins	Patients	Reduced effectiveness	0.970	0.055	148	0.997	0.714
**Statins in patients**			0.971	0.056	615	0.997	0.717
**Statins all respondents**			0.967	0.059	1549	0.995	0.725
Bisphosphonates	Public	No side effects	0.925	0.081	219	0.967	0.860
Bisphosphonates	Public	Some minor side effects	0.930	0.076	216	0.967	0.846
Bisphosphonates	Public	Some severe side effects	0.932	0.076	214	0.967	0.846
Bisphosphonates	Public	Reduced effectiveness	0.933	0.078	216	0.967	0.842
**Bisphosphonates in the public**			0.930	0.078	865	0.967	0.849
Bisphosphonates	Patients	No side effects	0.949	0.069	42	0.983	0.814
Bisphosphonates	Patients	Some minor side effects	0.955	0.055	40	0.967	0.826
Bisphosphonates	Patients	Some severe side effects	0.958	0.060	40	0.975	0.802
Bisphosphonates	Patients	Reduced effectiveness	0.956	0.065	39	0.967	0.802
**Bisphosphonates in patients**			0.954	0.062	161	0.967	0.811
**Bisphosphonates all respondents**			0.934	0.076	1026	0.967	.840
**All statins and bisphosphonates**			0.954	0.068	2575	0.975	0.773

For peer review only - http://bmjopen.bmj.com/site/about/guidelines.xhtml

The mean conditional time trade-off value associated with statin use was 0.966 which generates a DTD of 0.034 (calculated as 1, representing full health, minus the estimated utility score). The mean conditional time trade-off value associated with bisphosphonate use was 0.933 consequently giving a DTD of 0.067. In the statin cohort, none of the explanatory variables were significantly associated with time trade-off results, with the exception that respondents from the Asian/Asian British group reported higher DTD associated with statin-use (0.044 greater than white British / Irish respondents) although this finding is only based on a very small sample (n=7) (Table 3). In the bisphosphonate cohort, the public provided a DTD level that was 0.011 larger than patients undergoing treatment with bisphosphonates. Furthermore, those who had experience of taking medications more than three times a day provided a much lower DTD value than those who had no experience of daily medicine use. None of the other explanatory variables had a statistically significant impact on DTD size, including the number of pills taken per day. Results from the logistic regression model also reported in Table 3 suggest that respondents in the statin survey were less likely to trade than those in the bisphosphonate survey; this is consistent with the finding of higher mean time trade-off (lower mean DTD) values in the statin scenarios. No other variables in the logistic regression were statistically significant.

For peer review only - http://bmjopen.bmj.com/site/about/guidelines.xhtml

Table 3: Regression results

	Statins	Bisphosphonates	Statins & Bisphosphonates
	OLS	OLS	Logistic
	Utility score [95% CI]	Utility score [95% CI]	Traders [a] [95% CI]
Sex			
Female (Reference)	0	0	1
Male	-0.00282	0.00141	0.859
	[-0.0104,0.00478]	[-0.00984,0.0127]	[0.726,1.016]
Ethnicity			
White British/Irish (Reference)	0	0	1
White other	-0.00042	0.0223	1.629
	[-0.0157,0.0149]	[-0.00800,0.0525]	[0.873,3.04]
Mixed/Multiple ethnic origins	-0.0105	-0.00997	1.511
	[-0.0707,0.0497]	[-0.0458,0.0259]	[0.482,4.734]
Black/African/Caribbean/Black British	-0.0349	-0.0128	1.866
	[-0.0852,0.0154]	[-0.0650,0.0395]	[0.578,6.027]
Asian/Asian British	-0.0467**	-0.0127	2.516
	[-0.0790,-0.0144]	[-0.0483,0.0228]	[0.866,7.309]
Chinese	0.0198	-0.0364	3.813
	[-0.0359,0.0755]	[-0.0895,0.0168]	[0.448,32.477]
Other ethnicity	0.0145	0.00717	0.936
	[-0.0106,0.0395]	[-0.144,0.158]	[0.418,2.097]
Age			
Less than 35 (Reference)	0	0	1
35-44	-0.00906	-0.0198	1.028
	[-0.0299,0.0118]	[-0.0496,0.0100]	[0.658,1.607]
45-54	-0.00428	-0.000518	1.516
	[-0.0322,0.0237]	[-0.0349,0.0339]	[0.921,2.496]
55-64	0.00732	0.00321	0.943
	[-0.0145,0.0292]	[-0.0240,0.0305]	[0.615,1.446]
65-74	0.00472	-0.00585	1.129
	[-0.0176,0.0271]	[-0.0352,0.0235]	[0.711,1.792]
75+	0.00398	-0.0184	0.77
	[-0.0237,0.0317]	[-0.0545,0.0177]	[0.424,1.397]
Number of pills taken per day			
None (Reference)	0	0	1
One	0.00379	-0.00288	0.903
	[-0.0120,0.0196]	[-0.0216,0.0158]	[0.658,1.239]
Two to five	-0.00776	-0.00873	1.085
	[-0.0286,0.0131]	[-0.0272,0.00977]	[0.811,1.452]
Six to ten	-0.00794	-0.00263	0.867
	[-0.0346,0.0188]	[-0.0253,0.0201]	[0.583,1.289]
More than ten	0.00295	-0.0127	1.242
	[-0.0308,0.0367]	[-0.0411,0.0157]	[0.63,2.446]
Number of different times pill are taken per day			
None (Reference)	0	0	1
Once per day	0.00475	0.00892	0.788
	[-0.0149,0.0244]	[-0.00798,0.0258]	[0.596,1.042]
2 times a day	0.0176	0.00988	0.757

For peer review only - http://bmjopen.bmj.com/site/about/guidelines.xhtml

	[-0.00781,0.0430]	[-0.0111,0.0309]	[0.568,1.009]
3 times a day	0.0197	0.00321	0.856
	[-0.00677,0.0462]	[-0.0254,0.0318]	[0.562,1.303]
More than 3 times a day	0.0257	0.0426*	1.327
	[-0.0166,0.0680]	[0.00658,0.0785]	[0.549,3.205]
Respondent			
Patient (Reference)	0	0	1
Public	-0.00176	-0.0106*	1.004
	[-0.0118,0.00830]	[-0.0202,-0.000937]	[0.831,1.214]
Question context			
Question 1 (Reference)	0	0	1
Question 2	-0.00118	0.00795	0.938
	[-0.00978,0.00741]	[-0.00695,0.0229]	[0.746,1.178]
Question 3	-0.00266	0.0065	0.932
	[-0.0143,0.00893]	[-0.00796,0.0210]	[0.742,1.171]
Question 4	-0.00216	0.00709	0.941
	[-0.0117,0.00737]	[-0.00909,0.0233]	[0.749,1.182]
Sample			
Statins			0.531***
			[0.444,0.635]
Constant	0.969***	0.941***	6.11***
	[0.947,0.991]	[0.907,0.975]	[3.615,10.335]
Observations	2056	1460	3516
Individuals	514	365	879

* $p < 0.05$, ** $p < 0.01$, *** $p < 0.001$

[a] Those with TTO utility scores <0 are coded. Results represent unstandardised coefficients and 95% CI for the OLS models and adjusted odds ratio and 95% CI for the logistic model Coefficients reflect odds ratios. Above 1 implies more likely to provide a DTD value or willing to trade, less than 1 implies less likely to provide a DTD.

4 Discussion

In this study we find that long-term statin use is associated with a direct treatment disutility (DTD) of 0.034 among people willing to take statins. We find that bisphosphonate use is associated with a DTD of 0.067 among people willing to take bisphosphonates. These values imply that, even if medicines have no adverse effects, the act of taking them has a nontrivial impact on people's health-related quality of life. For statins, our study suggests that respondents on average would trade approximately 17 weeks of full health over 10 years whilst for bisphosphonates it would be more than half a year of life over 10 years. The findings for statins are particularly striking given these treatments are often thought by medical professionals to have minimal impact on users' daily routines.

For peer review only - http://bmjopen.bmj.com/site/about/guidelines.xhtml

Existing empirical studies have estimated a range of values of DTD but the general order of the size of the disutility is around 0.01 on average, which is equivalent to a loss of 5 weeks of perfect health over 10 years. In line with previous empirical studies, we find evidence for three different groups or types of respondent: (1) some never trading, suggesting zero disutility associated with treatments; (2) some suggesting they would be unlikely to initiate treatment and (3) some willing to trade length of life for no ongoing treatment, suggesting a DTD. In our survey, the groups willing to trade and generate a DTD made up the majority of those surveyed with approximately 73% and 84% for statins and bisphosphonates respondents, respectively.

We find that estimated mean DTD do not differ depending on whether the treatments were framed as more or less effective or having more or fewer side-effects or based upon demographic characteristics such as age or sex. Similar to Hutchins et al (6), we do find evidence suggesting that those from a non-white background, in our case an Asian/Asian British ethnic minority background, might perceive a higher level of disutility associated with long-term statin use although this is based upon a small sample of participants. This may also be an important part of ethnic health disparities to medication adherence and intensification of treatment. For example, British South Asians have been shown to more slowly intensify diabetes treatment than white groups (36). We found no difference between patient and public disutilities for statins but we did find that bisphosphonate patients generated smaller disutility values than the values coming from the general public. This finding could support theories rooted in experience utility (37,38) whereby those possessing the “lived" knowledge of disease, or treatment of disease, do not perceive the negative effects to be as severe as those in the general public, trying to imagine it. Our findings suggest there could be “hedonistic adaption” (39), with the additional disutility of bisphosphonates being less severe for those who are already taking medicines more than three times a day anyway.

The implications of our findings for future cost–utility analyses evaluating treatment pathways featuring statins or bisphosphonates (and potentially other oral medicines) are not straightforward. On the one hand, cost-effectiveness analyses should ideally capture the impact of all relevant costs and consequences associated with alternative forms of treatment (40), so it must be relevant that we have demonstrated

For peer review only - http://bmjopen.bmj.com/site/about/guidelines.xhtml

that the average person anticipates the act of taking statins or bisphosphonates will have a nontrivial impact on their health-related quality of life. Accounting for this disutility is likely to reduce the desirability of treatments that are currently considered very cost-effective: estimates of cost-effectiveness for long-term preventative interventions have been shown to be particularly sensitive to the inclusion of DTD (9,13,10,12,11,14,41). Indeed, we have previously shown that, for some people for whom guidelines currently recommend statins (e.g. those at a 10% risk of a cardiovascular event over 10 years), a DTD that appears moderate in light of the current study (0.015) would result in treatment doing more harm than good (25,42). Another reason routinely to account for DTD is that, without it, it is not possible to value innovations with positive process characteristics.

On the other hand, the apparent existence of distinct preference groups among our respondents requires careful consideration. A substantial minority of participants repeatedly indicated that they would be unwilling to trade any life expectancy to avoid taking these medicines, suggesting they consider any inconvenience with which they are associated negligible. It would be difficult to deny access to a treatment on the grounds that the average person would be bothered by its process characteristics, which is a danger if population-level cost-effectiveness estimates routinely incorporate average DTD. In view of these conflicting considerations, we recommend that decision-makers review scenarios with and without DTD. If evidence suggests that including DTD would materially alter the balance of benefits, harms and costs associated with treatment, this should be highlighted in population-level guidance, enabling prescribers at an individual level to engage in shared decision-making that gives appropriate weight to the person's preferences for avoiding the treatment's process characteristics. Such an approach fits well with the guideline development methods for NICE(43), which encourage the explicit identification of 'preference-sensitive decision-points', taking the practicalities of possible treatments into account. Future research could seek to develop tools which could quickly determine the level of DTD which could inform shared decision-making for preference-sensitive decisions.

Our study has several strengths. Firstly, the time trade-off methods we employ are consistent with the Measurement and Valuation of Health protocol first used to derive the EQ-5D-3L. Moreover, unlike previous studies that have attempted to elicit these values, we use a large representative sample of both

For peer review only - http://bmjopen.bmj.com/site/about/guidelines.xhtml

public and patients for our estimated values. Finally, due to the challenges associated with understanding and communicating risk, particularly in vulnerable older age-groups, we extensively trialed and developed the use of innovative approaches. For example, we used story-boards and visual arrays to contextualize the time trade-off exercise and communicate absolute risk. There are some limitations to our study which need to be considered. First, whilst we made best endeavours to communicate the exercise, the underlying absolute risks and the context for the research question, this had a set of clear trade-offs for participants. Second, the length of the survey, the cognitive burden and the time required were noted as challenges. Third, some of our respondents reported that they had difficulty understanding the time trade-off questions whilst others reported inconsistent values across the survey questions or had missing values, although where there was missingness we did multiply impute, assuming missing at random. Finally, those who took part in our survey ultimately self-selected and this could be related to their ability to complete the survey as well as their self-reported health. Applicability for a different patient or general population should be made based upon a careful judgement of the self-reported characteristics summarised for those reporting values in this study cohort.

5 Conclusion

Long-term preventative interventions, such as statins for cardiovascular disease or bisphosphonates for bone fractures, have a quantifiable direct treatment disutility associated with their use. The majority of respondents in our surveys, including public and patient-users, indicate at least some DTD. Bisphosphonates had larger disutilities than statins. Disutility was largely unaffected by respondents' self-reported characteristics but patient users of bisphosphonates did provide smaller DTDs than the public. Future model-based studies assessing the cost-effectiveness of long-term preventative interventions should incorporate DTD values within scenario analyses.

For peer review only

Declarations

Funding: This study/project is funded by the National Institute for Health Research (NIHR) Health Services and Delivery Research Programme (project reference 15/12/22). The views expressed are those of the authors and not necessarily those of the NIHR or the Department of Health and Social Care. The authors had full and sole access the data, and the funder had no role in the conduct of the research or the decision to publish.

Conflicts of interest. DM reports that he is supported by a Wellcome Trust Clinical Research Fellowship (214588/Z/18/Z) and is a member of the European Medicines Agency Pharmacovigilance Risk Assessment Committee. No other authors report any conflicts of interest directly relevant to the content of this article.

Consent to participate: Respondents gave informed consent after reading the participant information sheet online and indicating they agreed to participate in the survey online.

Consent for publication: The participant information sheet indicated that the results of the survey would be published and so we obtained informed consent.

Data availability: It is not possible to share the original data as this was a criterion for consent of participants.

Code availability: Survey design can be made available on request. Data analysis code included in Supplementary Appendix 5.

Contribution: All authors meet International Committee of Medical Journal Editors (ICMJE) criteria for authorship.

AJT obtained funding for this study, was involved in formulating the research question, provided advice on the design for the overall study, analysed data and produced a first draft of the manuscript. AJT acts as guarantor for this work.

JY was involved in formulating the research question, completed the process for ethical approval, produced the online version of the survey and contributed to writing the manuscript.

BG obtained funding for this study, was involved in formulating the research question, provided advice on the design for the overall study, contributed to the design of the online survey and writing the manuscript.

RH helped clean and analyse the data and contributed to writing the manuscript.

PD obtained funding for this study, was involved in formulating the research question, provided advice on the design for the overall study, contributed to the design of the online survey and writing the manuscript.

GR contributed to data analysis and contributed to writing the manuscript.

DM obtained funding for this study, was involved in formulating the research question, provided advice on the design for the overall study and contributed to writing the manuscript.

KP obtained funding for this study, formulated the research question, provided advice on the design for the overall study, contributed to the design of the online survey and oversaw data collection and analysis and contributed to writing the manuscript.

For peer review only - http://bmjopen.bmj.com/site/about/guidelines.xhtml

This manuscript has been read and approved by all the authors.

For peer review only

Ethics approval

Ethical approval for this study was granted by an NHS Health Research Authority Research Ethics Committee (REC reference: 17/NW/0124; project number: 220492).

Acknowledgements: We dedicate the article to the memory of Graham Bell who helped us greatly when formulating the research question and study design. We also wish to thank our other patient and public representatives that were integral to the design of this study. Our sincere thanks goes to all the respondents who piloted the initial survey and completed the final surveys for this study. We acknowledge the insightful contributions of Dr Shona Livingstone, Research Fellow in Statistics, to the survey design.

For peer review only

References

1. Taylor F, Huffman MD, Macedo AF, Moore THM, Burke M, Davey Smith G, et al. Statins for the primary prevention of cardiovascular disease. Cochrane Database Syst Rev. 2013 Jan 31;2013(1):CD004816.

2. Papapoulos SE. The role of bisphosphonates in the prevention and treatment of osteoporosis. Am J Med. 1993 Nov 30;95(5 SUPPL. 1):S48–52.

3. Sav A, Kendall E, McMillan SS, Kelly F, Whitty JA, King MA, et al. "You say treatment, I say hard work": treatment burden among people with chronic illness and their carers in Australia. Health Soc Care Community. 2013 Nov;21(6):665–74.

4. Thompson A, Guthrie B, Payne K. Do Pills Have No Ills? Capturing the Impact of Direct Treatment Disutility. PharmacoEconomics. 2016 Apr;34(4):333–6.

5. Fontana M, Asaria P, Moraldo M, Finegold J, Hassanally K, Manisty CH, et al. Patient-Accessible Tool for Shared Decision Making in Cardiovascular Primary Prevention: Balancing Longevity Benefits Against Medication Disutility. Circulation. 2014 Jun 17;129(24):2539–46.

6. Hutchins R, Viera AJ, Sheridan SL, Pignone MP. Quantifying the Utility of Taking Pills for Cardiovascular Prevention. Circ Cardiovasc Qual Outcomes. 2015 Mar;8(2):155–63.

7. Hutchins R, Pignone MP, Sheridan SL, Viera AJ. Quantifying the utility of taking pills for preventing adverse health outcomes: a cross-sectional survey. BMJ Open. 2015 May 11;5(5):e006505–e006505.

8. Augustovski FA, Cantor SB, Thach CT, Spann SJ. Aspirin for primary prevention of cardiovascular events. J Gen Intern Med. 1998 Dec;13(12):824–35.

9. Pignone M, Earnshaw S, Tice JA, Pletcher MJ. Aspirin, Statins, or Both Drugs for the Primary Prevention of Coronary Heart Disease Events in Men: A Cost–Utility Analysis. Ann Intern Med. 2006 Mar 7;144(5):326–36.

10. Timbie JW. Variation in the Net Benefit of Aggressive Cardiovascular Risk Factor Control Across the US Population of Patients With Diabetes Mellitus. Arch Intern Med. 2010 Jun 28;170(12):1037.

11. Lazar LD, Pletcher MJ, Coxson PG, Bibbins-Domingo K, Goldman L. Cost-Effectiveness of Statin Therapy for Primary Prevention in a Low-Cost Statin Era. Circulation. 2011 Jul 12;124(2):146–53.

12. Greving JP, Visseren FLJ, Wit G de, Algra A. Statin treatment for primary prevention of vascular disease: whom to treat? Cost-effectiveness analysis. BMJ. 2011 Mar 30;342:d1672.

13. Pletcher MJ. Comparing Impact and Cost-Effectiveness of Primary Prevention Strategies for Lipid-Lowering. Ann Intern Med. 2009 Feb 17;150(4):243.

14. Pletcher MJ, Pignone M, Earnshaw S, McDade C, Phillips KA, Auer R, et al. Using the Coronary Artery Calcium Score to Guide Statin Therapy: A Cost-Effectiveness Analysis. Circ Cardiovasc Qual Outcomes. 2014 Mar;7(2):276–84.

15. Pandya A, Sy S, Cho S, Weinstein MC, Gaziano TA. Cost-effectiveness of 10-Year Risk Thresholds for Initiation of Statin Therapy for Primary Prevention of Cardiovascular Disease. JAMA. 2015 Jul 14;314(2):142.

16. Heller DJ, Coxson PG, Penko J, Pletcher MJ, Goldman L, Odden MC, et al. Evaluating the Impact and Cost-Effectiveness of Statin Use Guidelines for Primary Prevention of Coronary Heart Disease and Stroke. Circulation. 2017 Sep 19;136(12):1087–98.

For peer review only - http://bmjopen.bmj.com/site/about/guidelines.xhtml

17. Spahillari A, Zhu J, Ferket BS, Hunink MGM, Carr JJ, Terry JG, et al. Cost-effectiveness of Contemporary Statin Use Guidelines With or Without Coronary Artery Calcium Assessment in African American Individuals. JAMA Cardiol. 2020 Aug 1;5(8):871.

18. Kohli-Lynch CN, Lewsey J, Boyd KA, French DD, Jordan N, Moran AE, et al. Beyond 10-Year Risk: A Cost-Effectiveness Analysis of Statins for the Primary Prevention of Cardiovascular Disease. Circulation. 2022 Apr 26;145(17):1312–23.

19. Fitzgerald SP, Bean NG, Ruberu RP. A Method of Decision Analysis Quantifying the Effects of Age and Comorbidities on the Probability of Deriving Significant Benefit from Medical Treatments. J Comorbidity. 2017 Jan;7(1):50–63.

20. Sussman JB, Schell GJ, Lavieri MS, Hayward RA. Implications of True and Perceived Treatment Burden on Cardiovascular Medication Use. MDM Policy Pract. 2017 Jul;2(2):238146831773530.

21. Torrance G. Utility approach to measuring health-related quality of life. J Chronic Dis. 1987;40:593–600.

22. Sackett DL, Torrance GW. The utility of different health states as perceived by the general public. J Chronic Dis. 1978;31(11):697–704.

23. NICE. 4 Economic evaluation | NICE health technology evaluations: the manual | Guidance | NICE [Internet]. NICE; 2022 [cited 2023 Mar 12]. Available from: https://www.nice.org.uk/process/pmg36/chapter/economic-evaluation#measuring-and-valuing-health-effects-in-cost-utility-analyses

24. Dolan P, Gudex C, Kind P, Williams A. The time trade-off method: results from a general population study. Health Econ. 1996;5(2):141–54.

25. Guthrie B, Thompson A, Dumbreck S, Flynn A, Alderson P, Nairn M, et al. Better guidelines for better care: accounting for multimorbidity in clinical guidelines – structured examination of exemplar guidelines and health economic modelling. Health Serv Deliv Res. 2017 Apr;5(16):1–150.

26. Cheraghi-Sohi S, Bower P, Mead N, McDonald R, Whalley D, Roland M. Making sense of patient priorities: applying discrete choice methods in primary care using 'think aloud' technique. Fam Pract. 2007 Jun 1;24(3):276–82.

27. Ryan M, Watson V, Entwistle V. Rationalising the 'irrational': a think aloud study of discrete choice experiment responses. Health Econ. 2009;18(3):321–36.

28. Sawtooth Software SSI Web 8.3.8. Sawtooth;

29. NHS Research Scotland. NRS Primary Care Network [Internet]. Primary Care | NHS Research Scotland. 2023 [cited 2023 Mar 12]. Available from: https://www.registerforshare.org

30. SHARE | RegisterforSHARE [Internet]. Register for SHARE. [cited 2023 Mar 12]. Available from: https://www.registerforshare.org

31. Dynata LLC. Survey Data Platform [Internet]. [cited 2023 Mar 12]. Available from: https://www.dynata.com/

32. Brazier J, Ratcliffe J, Saloman J, Tscuchiya A. Measuring and Valuing Health Benefits for Economic Evaluation. 2nd ed. Oxford University Press; 2017. 372 p.

33. Ryan M, Farrar S. Using conjoint analysis to elicit preferences for health care. BMJ. 2000 Jun 3;320(7248):1530–3.

For peer review only - http://bmjopen.bmj.com/site/about/guidelines.xhtml

For peer review only

34. Scott A. Identifying and analysing dominant preferences in discrete choice experiments: An application in health care. J Econ Psychol. 2002 Jun 1;23(3):383–98.

35. White IR, Royston P, Wood AM. Multiple imputation using chained equations: Issues and guidance for practice. Stat Med. 2011 Feb 20;30(4):377–99.

36. Mathur R, Farmer RE, Eastwood SV, Chaturvedi N, Douglas I, Smeeth L. Ethnic disparities in initiation and intensification of diabetes treatment in adults with type 2 diabetes in the UK, 1990-2017: A cohort study. PLoS Med. 2020 May;17(5):e1003106.

37. Kahneman D, Wakker PP, Sarin R. Back to Bentham? Explorations of Experienced Utility*. Q J Econ. 1997 May 1;112(2):375–406.

38. Dolan P, Kahneman D. Interpretations Of Utility And Their Implications For The Valuation Of Health*. Econ J. 2008;118(525):215–34.

39. Havi Carel. Is Well-being Possible in Illness? In: Phenomenology of Illnes. 1st ed. Oxford University Press; 2016.

40. Drummond MF, Sculpher MJ, Claxton K, Stoddard GL, Torrance GW. Methods for the economic evaluation of healthcare programmes. 4th ed. Oxford University Press; 2015. 464 p.

41. Roberts ET, Horne A, Martin SS, Blaha MJ, Blankstein R, Budoff MJ, et al. Cost-Effectiveness of Coronary Artery Calcium Testing for Coronary Heart and Cardiovascular Disease Risk Prediction to Guide Statin Allocation: The Multi-Ethnic Study of Atherosclerosis (MESA). Steyerberg EW, editor. PLOS ONE. 2015 Mar 18;10(3):e0116377.

42. Thompson A, Guthrie B, Payne K. Using the Payoff Time in Decision-Analytic Models: A Case Study for Using Statins in Primary Prevention. Med Decis Mak Int J Soc Med Decis Mak. 2017 Oct;37(7):759–69.

43. NICE. Shared decision making | NICE guidelines | NICE guidance | Our programmes | What we do | About [Internet]. NICE. NICE; [cited 2023 Mar 12]. Available from: https://www.nice.org.uk/about/what-we-do/our-programmes/nice-guidance/nice-guidelines/shared-decision-making

For peer review only

Figure 1: Kernel density plots showing distribution of TTO responses stratified by medicine, question context and respondent type

For peer review only

For peer review only - http://bmjopen.bmj.com/site/about/guidelines.xhtml

Supplementary Appendices for:

Thompson AJ, Youn J, Payne K, Hainsworth R, Donnan P, Allen A, Bell G, Rogers G, Guthrie B. Quantifying the impact of taking medicines for primary prevention: a time-trade off study to elicit direct treatment disutility

Supplementary Appendix 1: The statin survey

Supplementary Appendix 2: The bisphosphonate survey

Supplementary Appendix 3: Training materials for statin survey

Supplementary Appendix 4: Training materials for bisphosphonates survey

Supplementary Appendix 5: Stata 'do' file

Supplementary Appendix 6: Patient characteristics in those excluded

Supplementary Appendix 7: Propensity for dominant preferences

Supplementary Appendix 8: Performance of different models

For peer review only

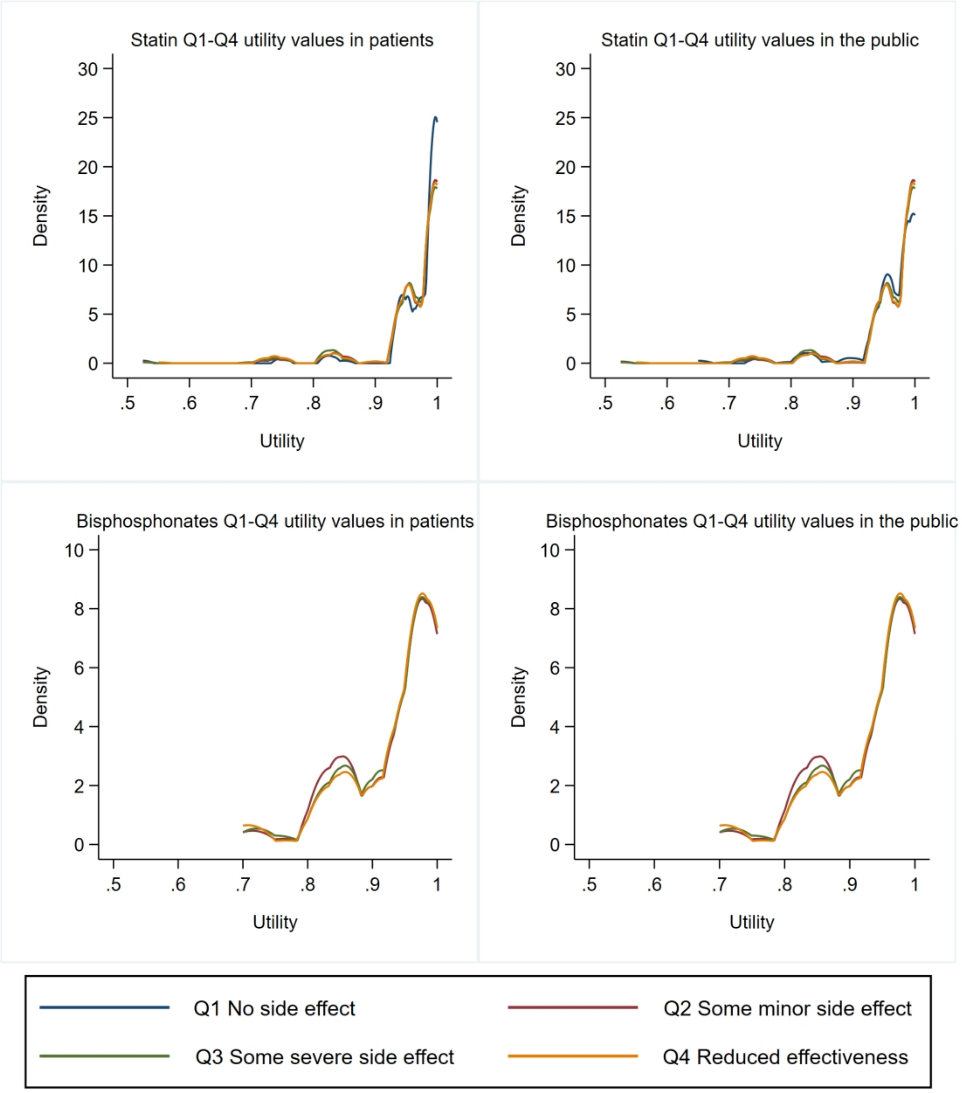

Kernel density plots showing distribution of TTO responses stratified by medicine, question context and respondent type

705x806mm (72 x 72 DPI)

Quantifying the impact of taking medicines for long-term conditions

A survey to understand your views about taking medicines long term for the prevention of heart disease

In this survey, we will be asking you about a drug treatment which may be offered to people at an increased risk of developing heart disease.

There are no right or wrong answers, we are just interested in hearing about your views.

For peer review only

Quantifying the impact of taking medicines for long-term conditions

Taking medicines long term for the prevention of heart disease

Participant Consent Form

You are being invited to take part in a research study that aims to understand your views about taking medicines long-term. We want to understand people's views on about what it is like to take a medicine every day for long periods of time. Before you decide, it is important for you to understand why the research is being done and what it will involve.

Please click on the sections below for information on the research.

What is the aim of the research?

Why have I been chosen?

What happens to the data collected?

How is confidentiality maintained?

How long will it take me?

Who will conduct the research?

What would I be asked to do if I took part?

Will the outcomes of the research be published?

What happens if I do not want to take part or if I change my mind?

Who has reviewed the research project?

I understand that my participation in the study is voluntary and that I am free to withdraw at any time without giving a reason by exiting the survey.

I confirm that I have read the information above.

- ◯ Yes, I would like to take part in the survey.
- ◯ No, I do not want to participate (exit survey).

0% 100%

What is your gender?

- ◯ Female
- ◯ Male

0% 100%

For peer review only

[QUOTA: QuotaCheck]

Note:

This page only contains quota information and only appears in preview mode.

0% 100%

For peer review only

By selecting one box in each group below, please indicate which statements best describe your own health state today.

Mobility

- ◯ I have no problems in walking about
- ◯ I have some problems in walking about
- ◯ I am confined to bed

Self-Care

- ◯ I have no problems with self-care
- ◯ I have some problems washing or dressing myself
- ◯ I am unable to wash or dress myself

Usual Activities (e.g. work, study, housework, family or leisure activities)

- ◯ I have no problems with performaing my usual activities
- ◯ I have some problems with performing my usual activities
- ◯ I am unable to perform my usual activities

Pain/Discomfort

- ◯ I have no pain or discomfort
- ◯ I have moderate pain or discomfort
- ◯ I have extreme pain or discomfort

Anxiety/Depression

- ◯ I am not anxious or depressed
- ◯ I am moderately anxious or depressed
- ◯ I am extremely anxious or depressed

0% 100%

Part 1. Study Background

This section of the survey explains: heart disease, how medicine treatments may be used for people at risk of heart disease, and what the treatment involves.

We will follow a story of Bobbie.

Click next to continue.

0% 100%

For peer review only

Part 1. Background

Bobbie has been to see a doctor.

The doctor has said Bobbie needs to start a medicine to prevent serious events because of his heart disease.

We will take you through Bobbie's story.

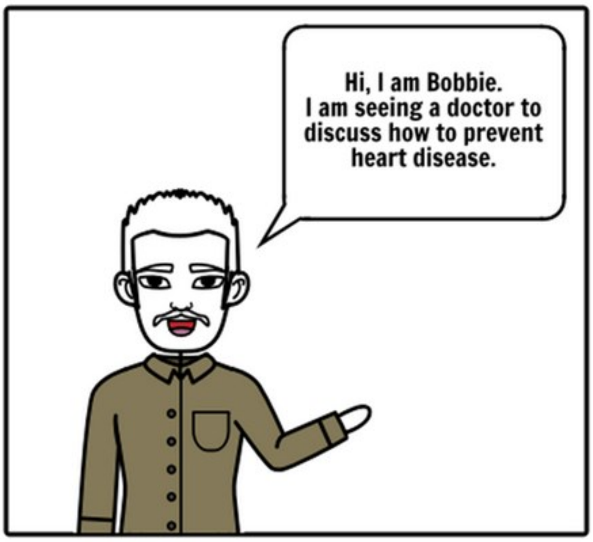

0% 100%

Part 1. Background

What is heart disease?

Bobbie hears about heart disease from his GP.

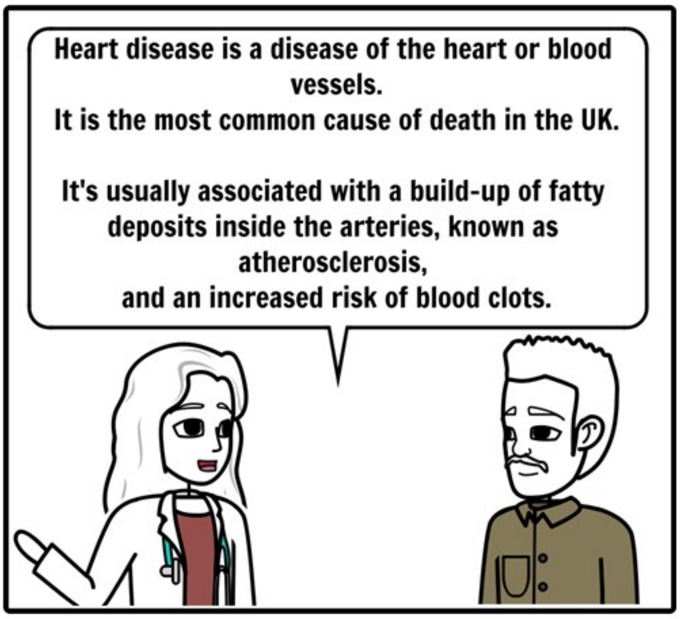

0% 100%

Part 1. Background

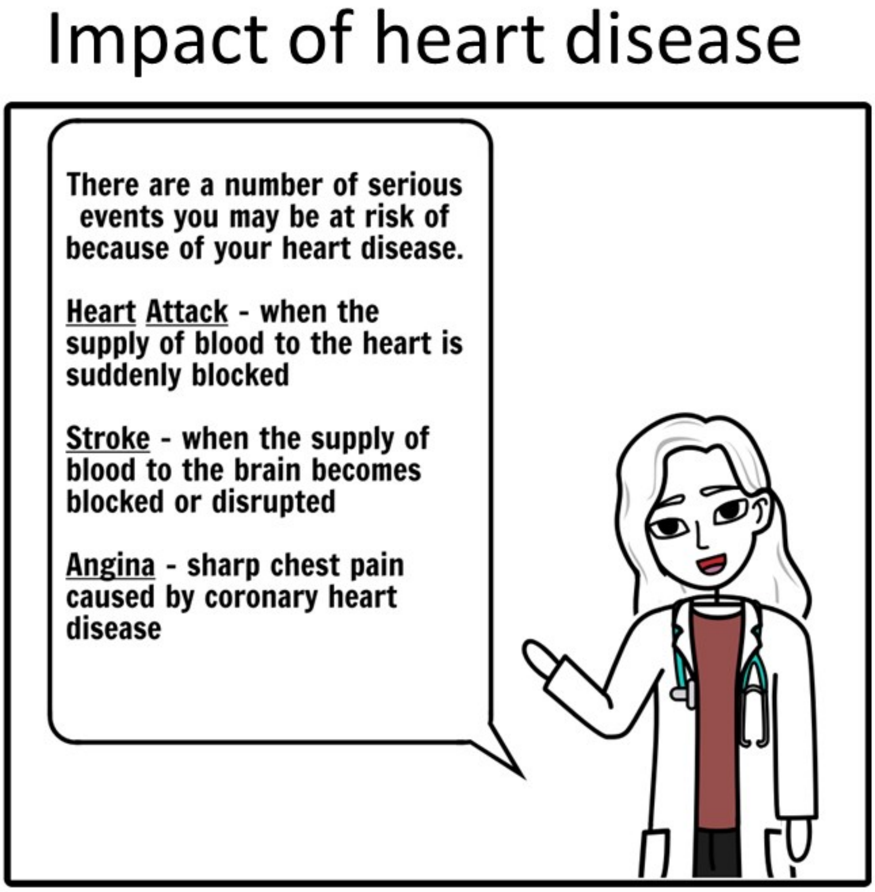

0% 100%

Part 1. Background

Risk of Heart disease

The GP explains that there is a chance that because of his cholesterol level Bobbie is at risk of developing one of these serious events in the future.

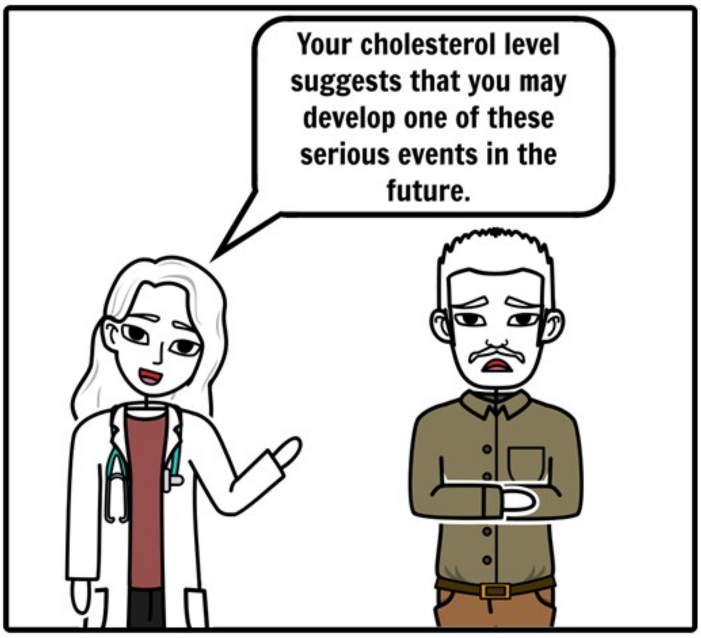

0% 100%

Part 1. Background

What is risk?

- **Risk** is: a term used to explain the chance that something bad might happen.

- **10% risk** means:

  Out of every **1,000** people, with heart disease,

  100 people would develop a heart attack, and **900** people would not.

0% 100%

Part 1. Background

What is a 10% risk?

- This diagram shows a 10% risk of having a heart attack because of their heart disease.
- The people shaded blue are the ones who have a heart attack.

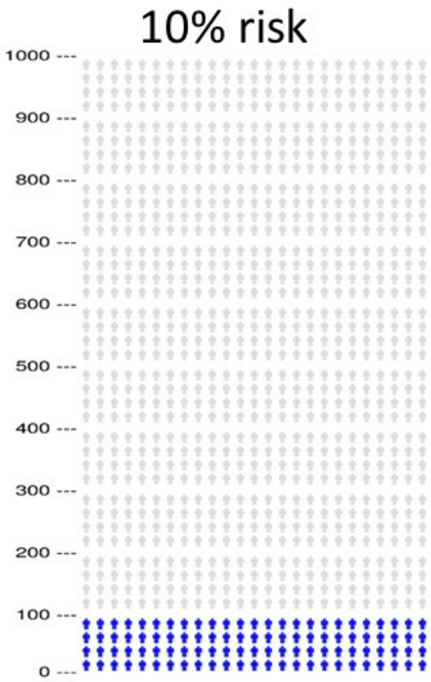

0% 100%

Part 1. Background

Prevention of serious events from heart disease

- Bobbie's doctor can estimate how likely it is that he will develop heart disease over the next 10 years.
- The estimate is based on things such as Bobbie's age and sex, family medical history, blood pressure and cholesterol level.
- The doctor may prescribe certain medicines such as statins to help reduce the chance of Bobbie having heart disease.
- The medicine will prevent some of the people who take it from having a serious event because of the heart disease.
- A serious event from the heart disease will still happen to some of the people who take the medicine.

0% 100%

Part 1. Background

Prevention of serious events from heart disease

The doctor prescribes Bobbie a medicine to reduce the chance of his heart disease causing a heart attack or other heart conditions in the future.

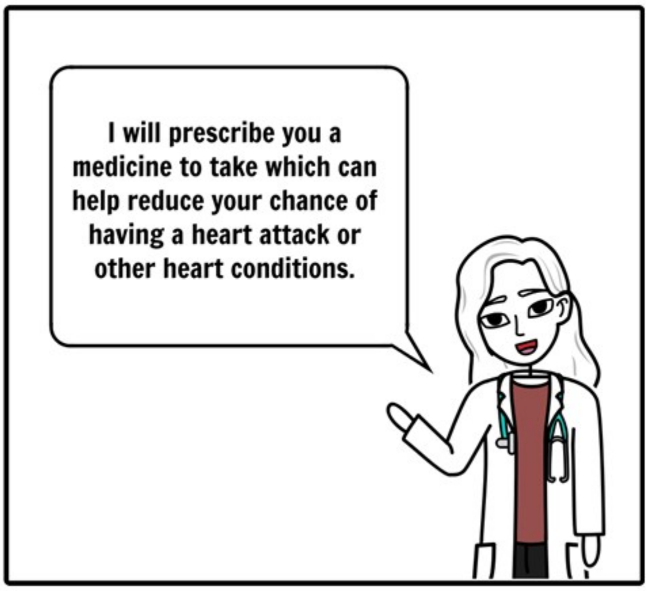

0% 100%

Part 1. Background

Collecting the medicine from the pharmacy

Bobbie goes to a pharmacy to collect his medicine.

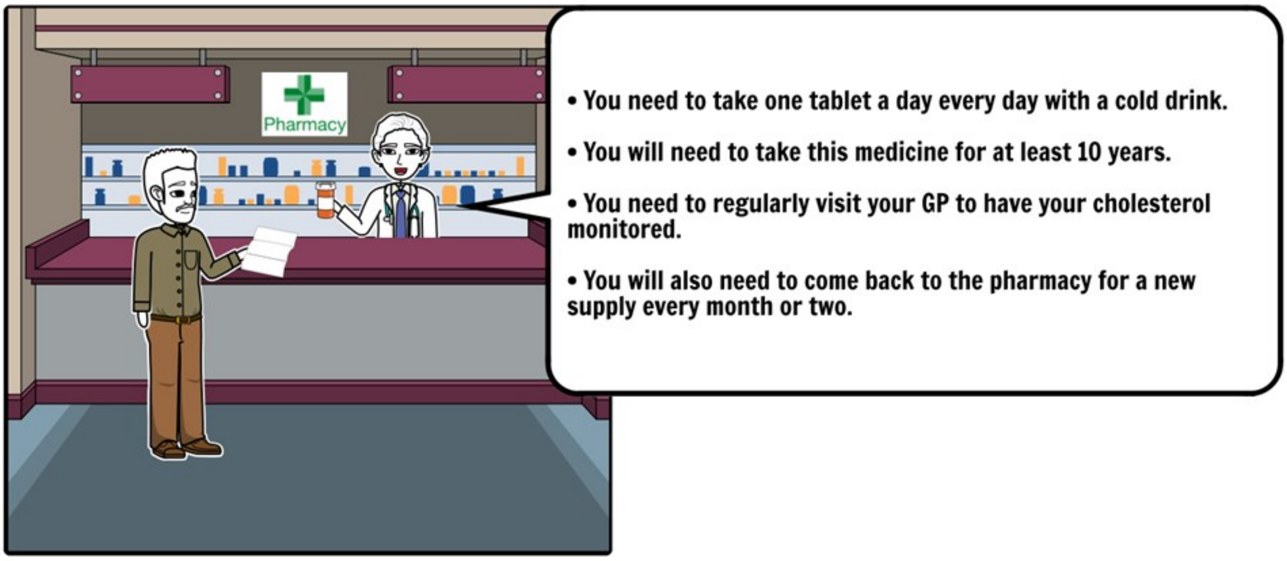

0% 100%

Part 1. Background

Taking the medicine for prevention

Bobbie goes home with his new tablets.

He then has to decide whether he wants to take them as the doctor and the pharmacist have advised.

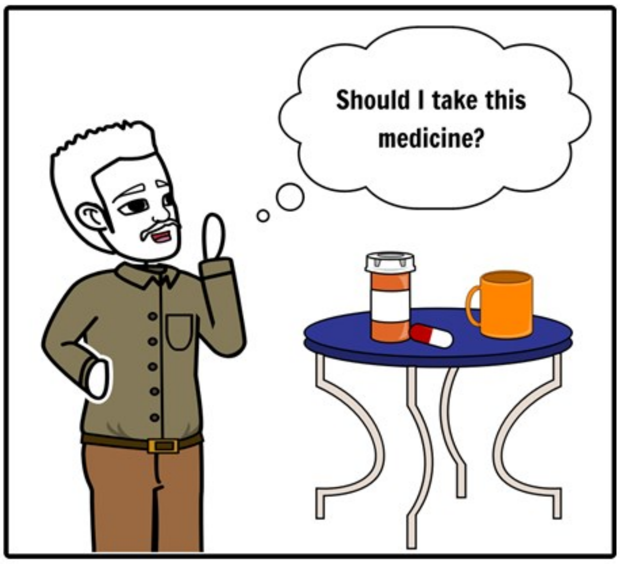

0% 100%

Part 1. Background

- Bobbie may consider three key things, when deciding whether to take the medicine.

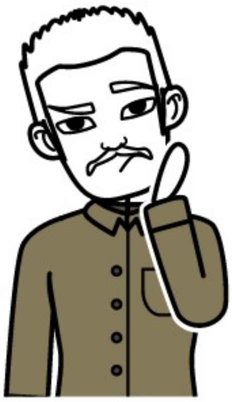

Effectiveness of the medicine

- How good the medicine is in terms of preventing a condition like a heart attack

Side effects of the medicine

- Whether there is any potential harm from the medicine

Inconvenience of the medicine

- Whether taking the medicine would fit in his lifestyle or cause him inconvenience

0% 100%

Part 1. Background

How effective is the medicine?

- Some people, but not everyone, who take the medicine will avoid a serious event, such as a heart attack or stroke as a result of taking the tablet.

- The effectiveness of the medicine can be described in terms of a reduction in this risk.

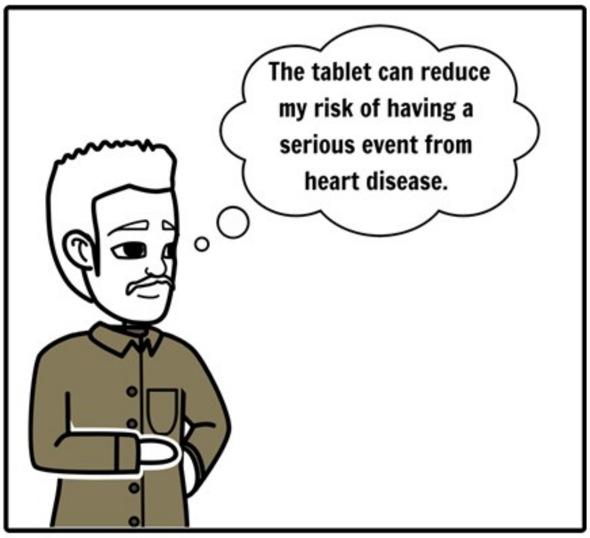

0% 100%

Part 1. Background

What does ‘reduction in risk’ mean?

- A ‘reduction in risk from 10% to 6%’ means:

The medicine will reduce your risk of experiencing an event, such as a heart attack, within the next 10 years from 10% to 6%.

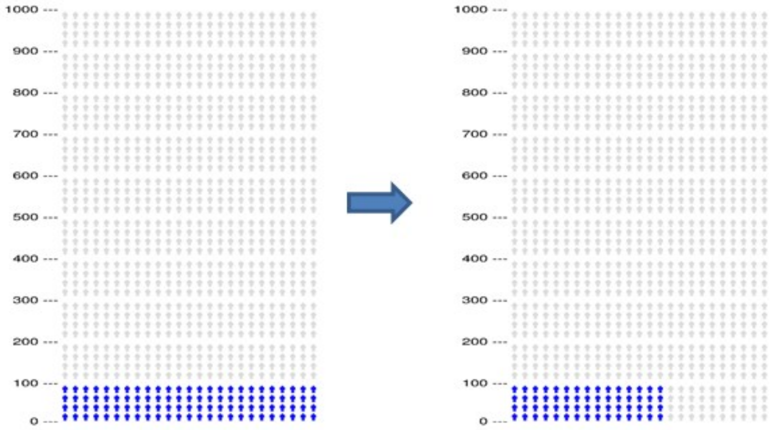

No treatment: 10% risk

Taking the medicine: 6% risk

0% 100%

Part 1. Background

Does the medicine have side effects?

The potential for side effects from the medicine can be described in terms of the risk of a side effect.

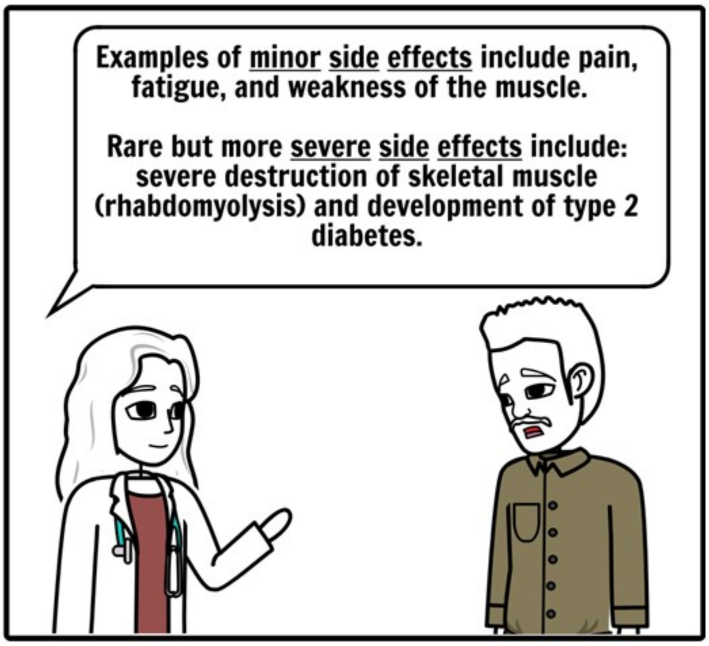

0% 100%

Part 1. Background

Risk of a minor side effect from the medicine

- A 5% risk of a **minor** side effect means:

Among every 1,000 people taking the medicine,

50 will experience a minor side effect such as either: pain, fatigue, and weakness of the muscle.

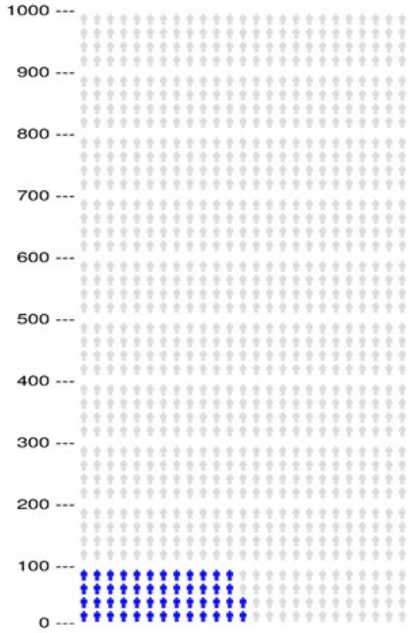

5% risk of a minor side effect

0% 100%

Part 1. Background

Risk of a severe side effect from the medicine

- A 0.1% risk of a **severe** side effect means:

Among every 1,000 people taking the medicine,

1 will experience a severe side effect such as either: severe destruction of skeletal muscle or development of type 2 diabetes.

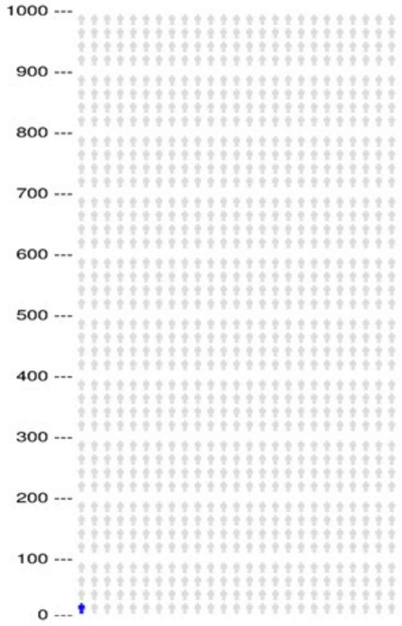

0.1% risk of a severe side effect

0% 100%

Part 1. Background

How inconvenient is it when taking the medicine?

Bobbie also thinks about what it means to have to take this medicine for the next ten years.

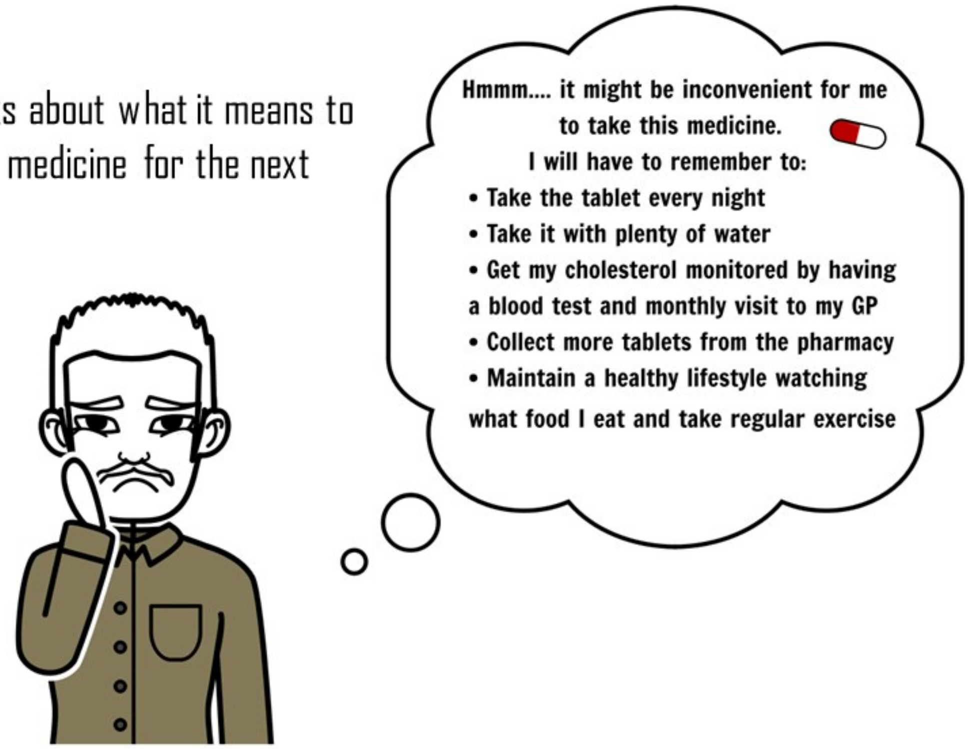

0% 100%

Part 1. Background

In the questions that follow in this survey, we will ask you about how you think about taking a medicine to prevent heart disease.

0% 100%

In general, how would you feel about taking a medicine for the first time?

- ◯ **Happy**
  I trust in my doctor and I am willing to try new things
- ◯ **Unsure**
  I'd like to think about it really carefully
- ◯ **Unhappy**
  I don't like the idea of trying a new medicine even if the doctor says it will help

0% 100%

For peer review only

Part 2: Your views on the inconvenience of taking a medicine to prevent heart disease

Now, we will ask you about how you think about the inconvenience of taking a medicine to prevent heart disease.

There are four questions.

Each question may look similar but differs in terms of the detail for each of the three features.

We will start with a practice question to show you an example of the type of question we will ask you.

0% 100%

For peer review only

Warm up question

You have been told that you need to take a medicine to reduce the risk of heart disease.

You can choose between two different medicines. We have called these Medicine A and Medicine B.

Medicine A is a pill that you only need to take once.

Medicine B is a pill that you need to take every day for ten years.

Medicine A and Medicine B are equally effective in reducing your risk of developing heart disease.
Medicine A and Medicine B will reduce your risk of having a heart attack or a stroke within the next 10 years from **10%** to **6%**.

In other words, in a crowd of **1,000** people in the UK, **100** are likely to have a heart attack or a stroke if they do not take the medicine. This is reduced to **60** people out of 1,000 who do take the medicine. This information is also illustrated in this picture.

Risk of heart disease

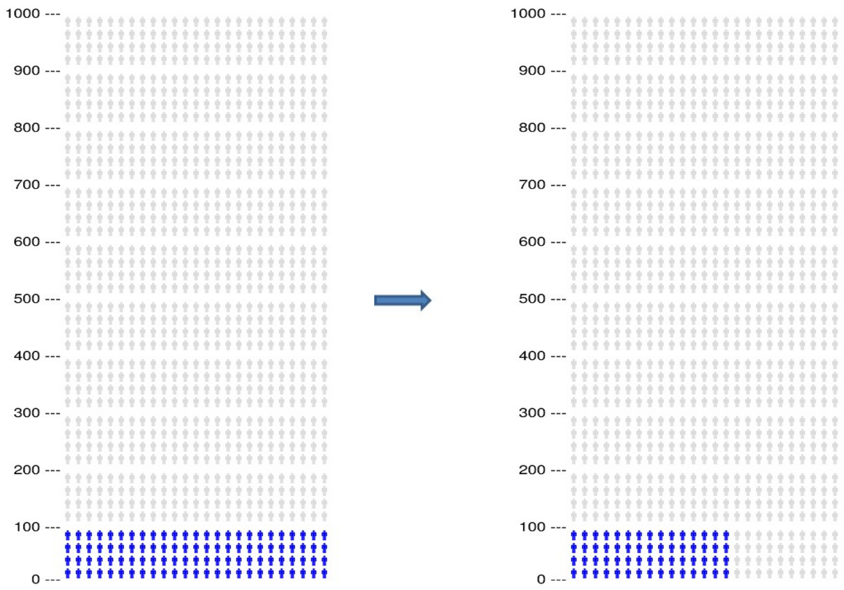

10%
No Treatment

6%
Taking the Medicine

(The figures shaded blue represent people who will have a heart attack or a stroke in the next 10 years)

In this scenario, you will not have any side effects from Medicine A or Medicine B.

Risk of a side effect

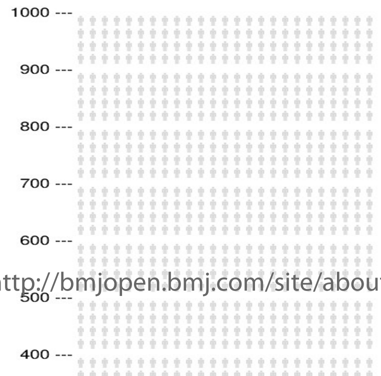

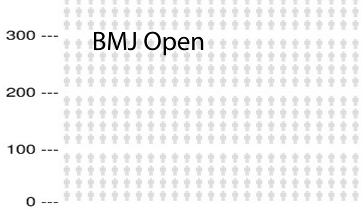

0%
No one will have a side effect.

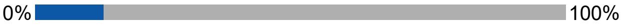

For peer review only

Remember Medicine A and Medicine B are equally effective and have no risks of a side effect.

The only difference between Medicine A and Medicine B is how you take them.

Medicine A	Medicine B
You take a tablet only once in your life.<br>You will be given the tablet on the day you come in to see your GP.<br>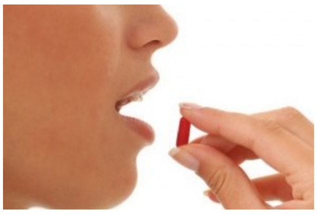	One tablet should be taken once every day, at night with cold water.<br>You need to take this medicine for 10 years.<br>You need to regularly visit a GP to receive a prescription.<br>You need to visit a pharmacy to fill a prescription.<br>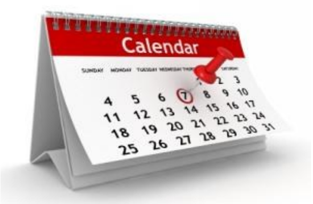 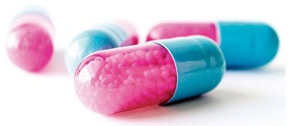

If you take Medicine A, you would live for **1 year** and then die.

If you take Medicine B, you would live for **10 years** and then die.

Would you prefer Medicine A or Medicine B, or are they the same?

Indicate your choice here. You can only choose one option:

Live for 1 year

○ **Medicine A** 1

○ **Medicine B** Live for 10 years

○ **The Same - Medicine A and Medicine B are the same.**

0% 100%

Now we are ready to start the survey questions.

0% 100%

For peer review only

Part 2

Question 1

You have been told that you need to take a medicine to reduce the risk of heart disease.

You can choose between two different medicines. We have called these Medicine A and Medicine B.

Medicine A is a pill that you only need to take once.

Medicine B is a pill that you need to take every day for ten years.

Medicine A and Medicine B are equally effective in reducing your risk of developing heart disease.
Medicine A and Medicine B will reduce your risk of having a heart attack or a stroke within the next 10 years from **10%** to **6%**.

In other words, in a crowd of **1,000** people in the UK, **100** are likely to have a heart attack or a stroke if they do not take the medicine. This is reduced to **60** people out of 1,000 who do take the medicine. This information is also illustrated in this picture.

Risk of heart disease

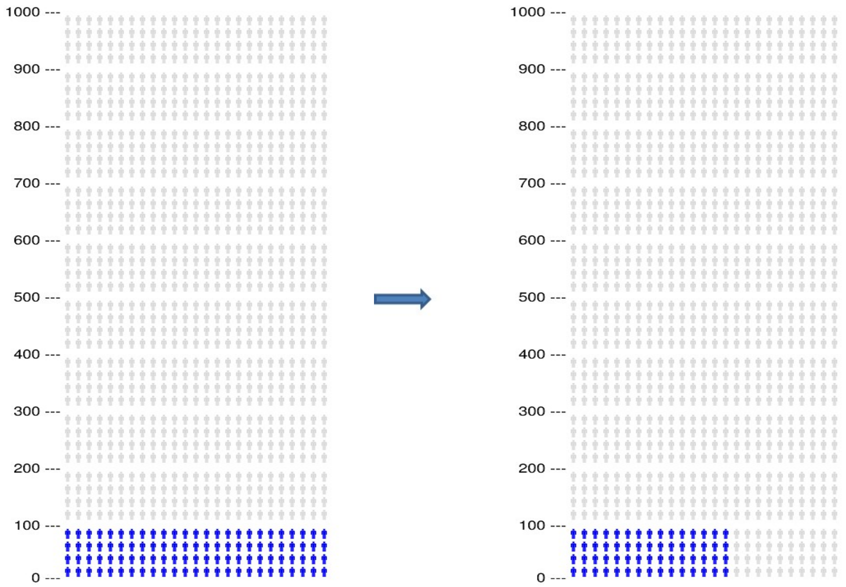

10%
No Treatment

6%
Taking the Medicine

(The figures shaded blue represent people who will have a heart attack or a stroke in the next 10 years)

In this scenario, you will not have any side effects from Medicine A or Medicine B.

Risk of a side effect

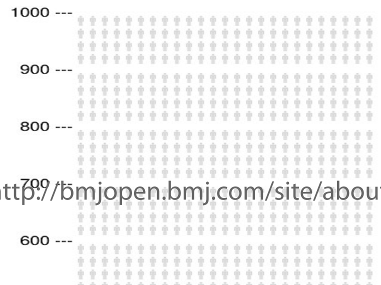

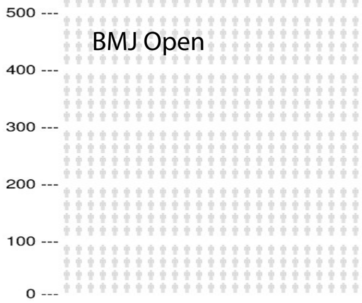

0 %
No one will have a side effect.

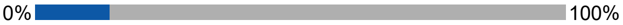

peer review only

Repeated section

Remember Medicine A and Medicine B are equally effective and have no risks of a side effect.

The only difference between Medicine A and Medicine B is how you take them.

Medicine A	Medicine B
You take a tablet only once in your life.<br>You will be given the tablet on the day you come in to see your GP.<br>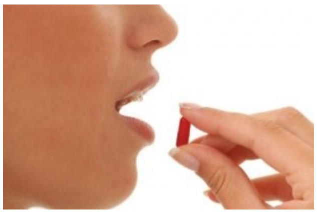	One tablet should be taken once every day, at night with cold water.<br>You need to take this medicine for 10 years.<br>You need to regularly visit a GP to receive a prescription.<br>You need to visit a pharmacy to fill a prescription.<br>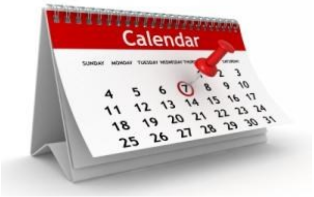<br>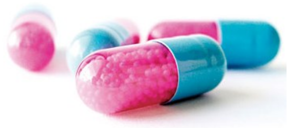

If you take Medicine A, you would live for **5 years** and then die.

If you take Medicine B, you would live for **10 years** and then die.

Would you prefer Medicine A or Medicine B, or are they the same?

Indicate your choice here. You can only choose one option:

○ **Medicine A** Live for 5 years

○ **Medicine B** Live for 10 years

○ **Medicine A and Medicine B are the same.**

0% 100%

For peer review only

Repeated section

Remember Medicine A and Medicine B are equally effective and have no risks of a side effect.

The only difference between Medicine A and Medicine B is how you take them.

**Medicine A**	**Medicine B**
You take a tablet only once in your life.<br>You will be given the tablet on the day you come in to see your GP.<br>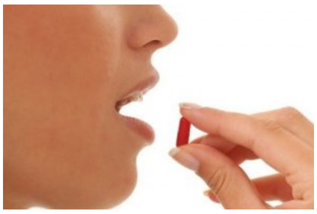	One tablet should be taken once every day, at night with cold water.<br>You need to take this medicine for 10 years.<br>You need to regularly visit a GP to receive a prescription.<br>You need to visit a pharmacy to fill a prescription.<br>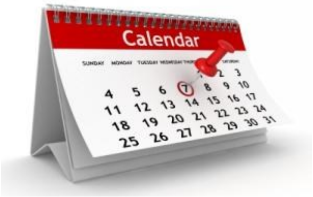<br>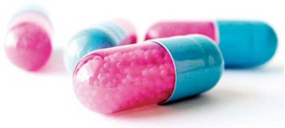

If you take Medicine A, you would live for **5.5 years** (5 years and 6 months) and then die.

If you take Medicine B, you would live for **10 years** and then die.

Would you prefer Medicine A or Medicine B, or are they the same?

Indicate your choice here. You can only choose one option:

◯ **Medicine A** Live for 5.5 years

◯ **Medicine B** Live for 10 years

◯ **Medicine A and Medicine B are the same.**

0% 100%

For peer review only

Repeated section

Remember Medicine A and Medicine B are equally effective and have no risks of a side effect.

The only difference between Medicine A and Medicine B is how you take them.

Medicine A	Medicine B
You take a tablet only once in your life.<br>You will be given the tablet on the day you come in to see your GP.<br>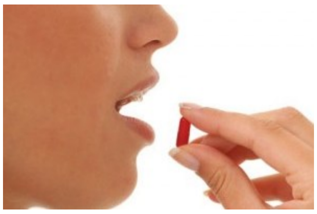	One tablet should be taken once every day, at night with cold water.<br>You need to take this medicine for 10 years.<br>You need to regularly visit a GP to receive a prescription.<br>You need to visit a pharmacy to fill a prescription.<br>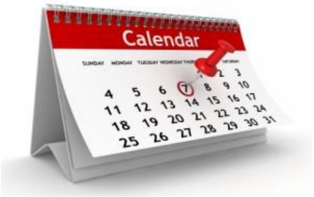<br>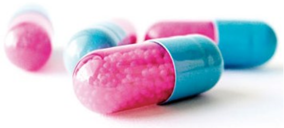

If you take Medicine A, you would live for **6 years** and then die.

If you take Medicine B, you would live for **10 years** and then die.

Would you prefer Medicine A or Medicine B, or are they the same?

Indicate your choice here. You can only choose one option:

◯ **Medicine A** Live for 6 years

◯ **Medicine B** Live for 10 years

◯ **Medicine A and Medicine B are the same.**

For peer review only

Repeated section

Remember Medicine A and Medicine B are equally effective and have no risks of a side effect.

The only difference between Medicine A and Medicine B is how you take them.

Medicine A	Medicine B
You take a tablet only once in your life.<br>You will be given the tablet on the day you come in to see your GP.<br>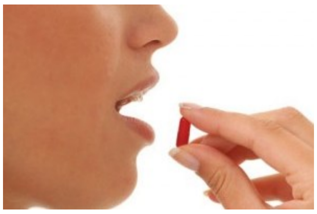	One tablet should be taken once every day, at night with cold water.<br>You need to take this medicine for 10 years.<br>You need to regularly visit a GP to receive a prescription.<br>You need to visit a pharmacy to fill a prescription.<br>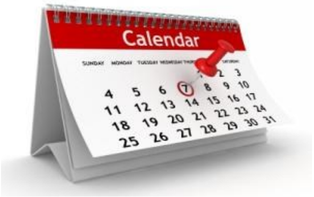<br>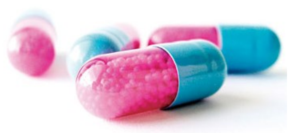

If you take Medicine A, you would live for **6.25 years (6 years and 3 months)** and then die.

If you take Medicine B, you would live for **10 years** and then die.

Would you prefer Medicine A or Medicine B, or are they the same?

Indicate your choice here. You can only choose one option:

◯ **Medicine A** Live for 6 years and 3 months

◯ **Medicine B** Live for 10 years

◯ **Medicine A and Medicine B are the same.**

0% 100%

For peer review only

Repeated section

Remember Medicine A and Medicine B are equally effective and have no risks of a side effect.

The only difference between Medicine A and Medicine B is how you take them.

Medicine A	Medicine B
You take a tablet only once in your life.<br>You will be given the tablet on the day you come in to see your GP.<br>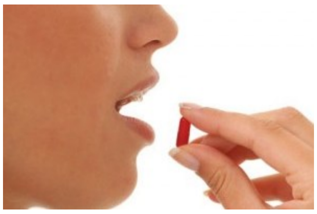	One tablet should be taken once every day, at night with cold water.<br>You need to take this medicine for 10 years.<br>You need to regularly visit a GP to receive a prescription.<br>You need to visit a pharmacy to fill a prescription.<br>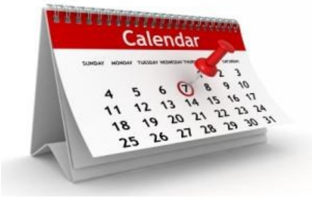<br>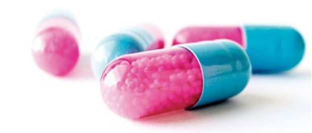

If you take Medicine A, you would live for **6.5 years** (6 years and 6 months) and then die.

If you take Medicine B, you would live for **10 years** and then die.

Would you prefer Medicine A or Medicine B, or are they the same?

Indicate your choice here. You can only choose one option:

◯ **Medicine A**

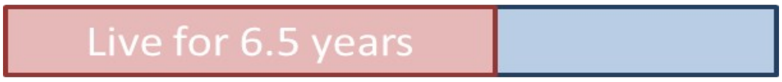

◯ **Medicine B**

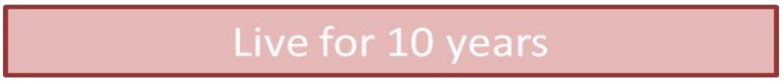

◯ **Medicine A and Medicine B are the same.**

For peer review only

Repeated section

Remember Medicine A and Medicine B are equally effective and have no risks of a side effect.

The only difference between Medicine A and Medicine B is how you take them.

Medicine A	Medicine B
You take a tablet only once in your life.<br>You will be given the tablet on the day you come in to see your GP.<br>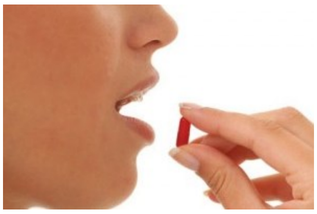	One tablet should be taken once every day, at night with cold water.<br>You need to take this medicine for 10 years.<br>You need to regularly visit a GP to receive a prescription.<br>You need to visit a pharmacy to fill a prescription.<br>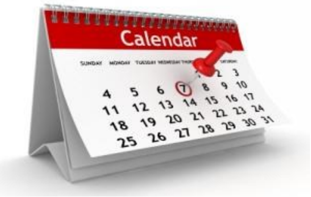<br><br>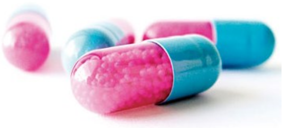

If you take Medicine A, you would live for **7 years** and then die.

If you take Medicine B, you would live for **10 years** and then die.

Would you prefer Medicine A or Medicine B, or are they the same?

Indicate your choice here. You can only choose one option:

○ **Medicine A** Live for 7 years

○ **Medicine B** Live for 10 years

○ **Medicine A and Medicine B are the same.**

0% 100%

For peer review only

Repeated section

Remember Medicine A and Medicine B are equally effective and have no risks of a side effect.

The only difference between Medicine A and Medicine B is how you take them.

Medicine A	Medicine B
You take a tablet only once in your life.<br>You will be given the tablet on the day you come in to see your GP.<br>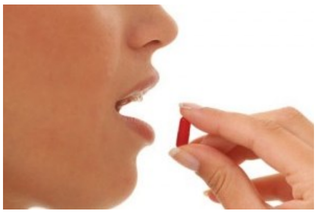	One tablet should be taken once every day, at night with cold water.<br>You need to take this medicine for 10 years.<br>You need to regularly visit a GP to receive a prescription.<br>You need to visit a pharmacy to fill a prescription.<br>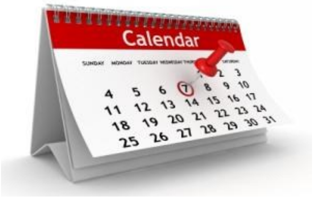<br>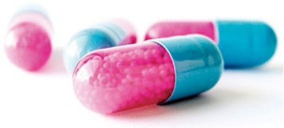

If you take Medicine A, you would live for **7.5 years** (7 years and 6 months) and then die.

If you take Medicine B, you would live for **10 years** and then die.

Would you prefer Medicine A or Medicine B, or are they the same?

Indicate your choice here. You can only choose one option:

◯ **Medicine A**

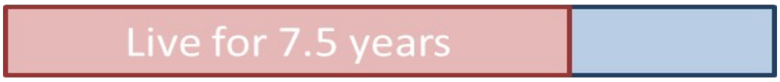

◯ **Medicine B**

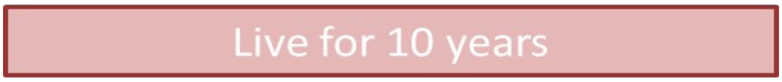

◯ **Medicine A and Medicine B are the same.**

For peer review only

Repeated section

Remember Medicine A and Medicine B are equally effective and have no risks of a side effect.

The only difference between Medicine A and Medicine B is how you take them.

Medicine A	Medicine B
You take a tablet only once in your life.<br>You will be given the tablet on the day you come in to see your GP.<br>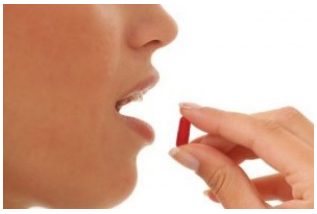	One tablet should be taken once every day, at night with cold water.<br>You need to take this medicine for 10 years.<br>You need to regularly visit a GP to receive a prescription.<br>You need to visit a pharmacy to fill a prescription.<br>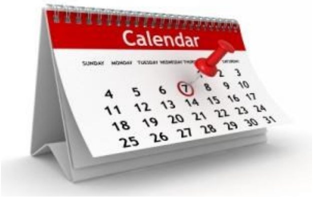<br>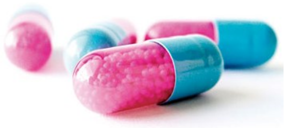

If you take Medicine A, you would live for **7.75 years (7 years and 9 months)** and then die.

If you take Medicine B, you would live for **10 years** and then die.

Would you prefer Medicine A or Medicine B, or are they the same?

Indicate your choice here. You can only choose one option:

◯ **Medicine A** Live for 7 years and 9 months

◯ **Medicine B** Live for 10 years

◯ **Medicine A and Medicine B are the same.**

For peer review only

Repeated section

Remember Medicine A and Medicine B are equally effective and have no risks of a side effect.

The only difference between Medicine A and Medicine B is how you take them.

Medicine A	Medicine B
You take a tablet only once in your life.<br>You will be given the tablet on the day you come in to see your GP.<br>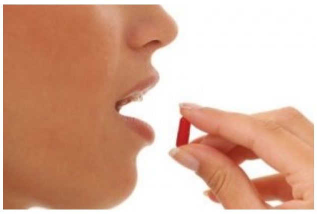	One tablet should be taken once every day, at night with cold water.<br>You need to take this medicine for 10 years.<br>You need to regularly visit a GP to receive a prescription.<br>You need to visit a pharmacy to fill a prescription.<br>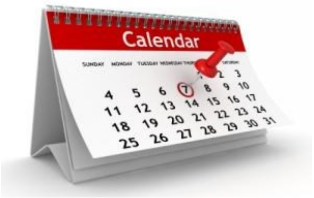<br>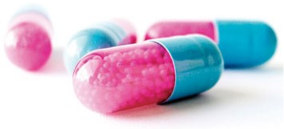

If you take Medicine A, you would live for **8 years** and then die.

If you take Medicine B, you would live for **10 years** and then die.

Would you prefer Medicine A or Medicine B, or are they the same?

Indicate your choice here. You can only choose one option:

◯ **Medicine A** Live for 8 years

◯ **Medicine B** Live for 10 years

◯ **Medicine A and Medicine B are the same.**

For peer review only

Repeated section

Remember Medicine A and Medicine B are equally effective and have no risks of a side effect.

The only difference between Medicine A and Medicine B is how you take them.

**Medicine A**	**Medicine B**
You take a tablet only once in your life.<br>You will be given the tablet on the day you come in to see your GP.<br>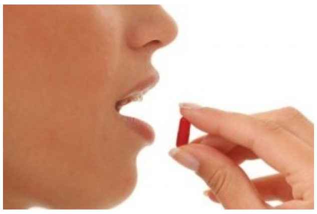	One tablet should be taken once every day, at night with cold water.<br>You need to take this medicine for 10 years.<br>You need to regularly visit a GP to receive a prescription.<br>You need to visit a pharmacy to fill a prescription.<br>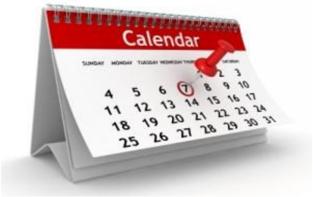<br>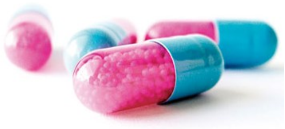

If you take Medicine A, you would live for **8.25 years (8 years and 3 months)** and then die.

If you take Medicine B, you would live for **10 years** and then die.

Would you prefer Medicine A or Medicine B, or are they the same?

Indicate your choice here. You can only choose one option:

○ **Medicine A** Live for 8 years and 3 months

○ **Medicine B** Live for 10 years

○ **Medicine A and Medicine B are the same.**

0% 100%

For peer review only

Repeated section

Remember Medicine A and Medicine B are equally effective and have no risks of a side effect.

The only difference between Medicine A and Medicine B is how you take them.

Medicine A	Medicine B
You take a tablet only once in your life.<br>You will be given the tablet on the day you come in to see your GP.<br>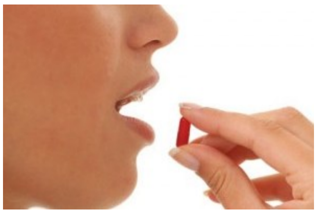	One tablet should be taken once every day, at night with cold water.<br>You need to take this medicine for 10 years.<br>You need to regularly visit a GP to receive a prescription.<br>You need to visit a pharmacy to fill a prescription.<br>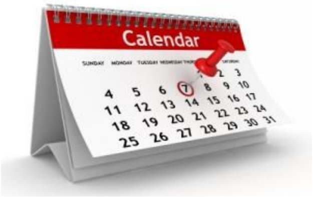<br>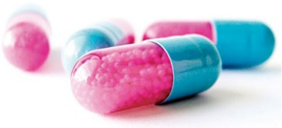

If you take Medicine A, you would live for **8.5 years** (8 years and 6 months) and then die.

If you take Medicine B, you would live for **10 years** and then die.

Would you prefer Medicine A or Medicine B, or are they the same?

Indicate your choice here. You can only choose one option:

◯ **Medicine A** Live for 8.5 years

◯ **Medicine B** Live for 10 years

◯ **Medicine A and Medicine B are the same.**

For peer review only

Repeated section

Remember Medicine A and Medicine B are equally effective and have no risks of a side effect.

The only difference between Medicine A and Medicine B is how you take them.

Medicine A	Medicine B
You take a tablet only once in your life.<br>You will be given the tablet on the day you come in to see your GP.<br>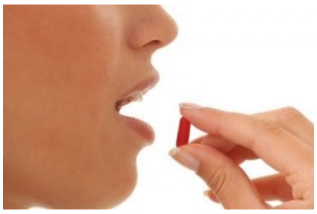	One tablet should be taken once every day, at night with cold water.<br>You need to take this medicine for 10 years.<br>You need to regularly visit a GP to receive a prescription.<br>You need to visit a pharmacy to fill a prescription.<br>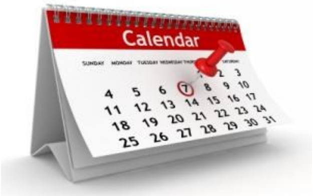<br>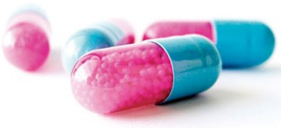

If you take Medicine A, you would live for **8.75 years (8 years and 9 months)** and then die.

If you take Medicine B, you would live for **10 years** and then die.

Would you prefer Medicine A or Medicine B, or are they the same?

Indicate your choice here. You can only choose one option:

◯ **Medicine A** Live for 8 years and 9 months

◯ **Medicine B** Live for 10 years

◯ **Medicine A and Medicine B are the same.**

For peer review only

Repeated section

Remember Medicine A and Medicine B are equally effective and have no risks of a side effect.

The only difference between Medicine A and Medicine B is how you take them.

Medicine A	Medicine B
You take a tablet only once in your life.<br>You will be given the tablet on the day you come in to see your GP.<br>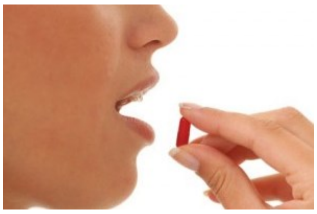	One tablet should be taken once every day, at night with cold water.<br>You need to take this medicine for 10 years.<br>You need to regularly visit a GP to receive a prescription.<br>You need to visit a pharmacy to fill a prescription.<br>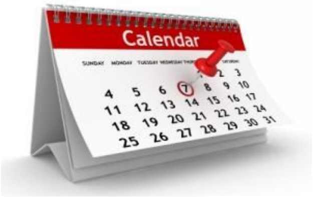<br>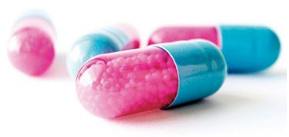

If you take Medicine A, you would live for **9 years** and then die.

If you take Medicine B, you would live for **10 years** and then die.

Would you prefer Medicine A or Medicine B, or are they the same?

Indicate your choice here. You can only choose one option:

◯ **Medicine A** Live for 9 years

◯ **Medicine B** Live for 10 years

◯ **Medicine A and Medicine B are the same.**

0% 100%

For peer review only

Repeated section

Remember Medicine A and Medicine B are equally effective and have no risks of a side effect.

The only difference between Medicine A and Medicine B is how you take them.

**Medicine A**	**Medicine B**
You take a tablet only once in your life.<br>You will be given the tablet on the day you come in to see your GP.<br>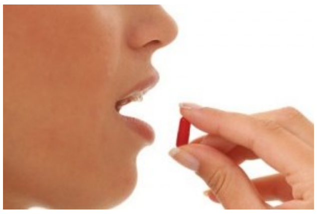	One tablet should be taken once every day, at night with cold water.<br>You need to take this medicine for 10 years.<br>You need to regularly visit a GP to receive a prescription.<br>You need to visit a pharmacy to fill a prescription.<br>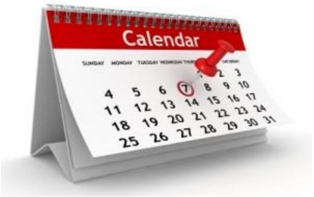<br>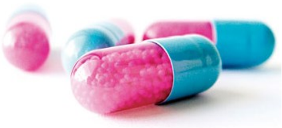

If you take Medicine A, you would live for **9 years and 1 month** and then die.

If you take Medicine B, you would live for **10 years** and then die.

Would you prefer Medicine A or Medicine B, or are they the same?

Indicate your choice here. You can only choose one option:

○ **Medicine A** Live for 9 years 1 month

○ **Medicine B** Live for 10 years

○ **Medicine A and Medicine B are the same.**

For peer review only

Repeated section

Remember Medicine A and Medicine B are equally effective and have no risks of a side effect.

The only difference between Medicine A and Medicine B is how you take them.

Medicine A	Medicine B
You take a tablet only once in your life.<br>You will be given the tablet on the day you come in to see your GP.<br>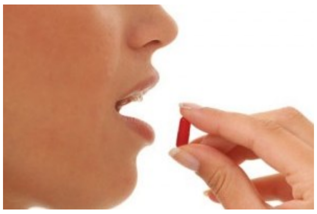	One tablet should be taken once every day, at night with cold water.<br>You need to take this medicine for 10 years.<br>You need to regularly visit a GP to receive a prescription.<br>You need to visit a pharmacy to fill a prescription.<br>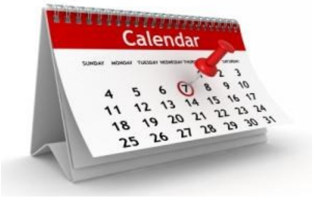<br>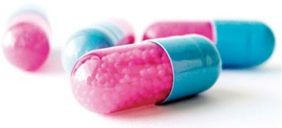

If you take Medicine A, you would live for **9.25 years (9 years and 3 months)** and then die.

If you take Medicine B, you would live for **10 years** and then die.

Would you prefer Medicine A or Medicine B, or are they the same?

Indicate your choice here. You can only choose one option:

◯ **Medicine A** Live for 9 years and 3 months

◯ **Medicine B** Live for 10 years

◯ **Medicine A and Medicine B are the same.**

0% 100%

For peer review only

Repeated section

Remember Medicine A and Medicine B are equally effective and have no risks of a side effect.

The only difference between Medicine A and Medicine B is how you take them.

**Medicine A**	**Medicine B**
You take a tablet only once in your life.<br>You will be given the tablet on the day you come in to see your GP.<br>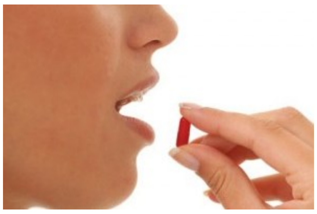	One tablet should be taken once every day, at night with cold water.<br>You need to take this medicine for 10 years.<br>You need to regularly visit a GP to receive a prescription.<br>You need to visit a pharmacy to fill a prescription.<br>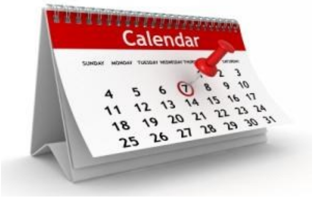<br>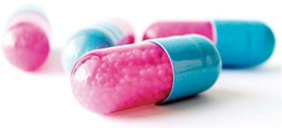

If you take Medicine A, you would live for **9 years and 4 months** and then die.

If you take Medicine B, you would live for **10 years** and then die.

Would you prefer Medicine A or Medicine B, or are they the same?

Indicate your choice here. You can only choose one option:

◯ **Medicine A** Live for 9 years 4 months

◯ **Medicine B** Live for 10 years

◯ **Medicine A and Medicine B are the same.**

0% 100%

For peer review only

Repeated section

Remember Medicine A and Medicine B are equally effective and have no risks of a side effect.

The only difference between Medicine A and Medicine B is how you take them.

**Medicine A**	**Medicine B**
You take a tablet only once in your life.<br>You will be given the tablet on the day you come in to see your GP.<br>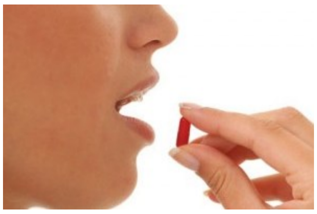	One tablet should be taken once every day, at night with cold water.<br>You need to take this medicine for 10 years.<br>You need to regularly visit a GP to receive a prescription.<br>You need to visit a pharmacy to fill a prescription.<br>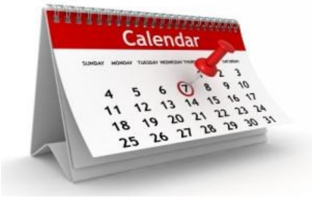<br>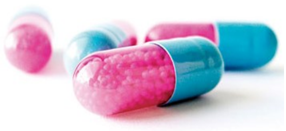

If you take Medicine A, you would live for **9 years and 5 months** and then die.

If you take Medicine B, you would live for **10 years** and then die.

Would you prefer Medicine A or Medicine B, or are they the same?

Indicate your choice here. You can only choose one option:

○ **Medicine A** — Live for 9 years 5 months

○ **Medicine B** — Live for 10 years

○ **Medicine A and Medicine B are the same.**

0% 100%

For peer review only

Repeated section

Remember Medicine A and Medicine B are equally effective and have no risks of a side effect.

The only difference between Medicine A and Medicine B is how you take them.

**Medicine A**	**Medicine B**
You take a tablet only once in your life.<br>You will be given the tablet on the day you come in to see your GP.<br>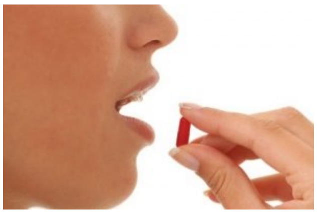	One tablet should be taken once every day, at night with cold water.<br>You need to take this medicine for 10 years.<br>You need to regularly visit a GP to receive a prescription.<br>You need to visit a pharmacy to fill a prescription.<br>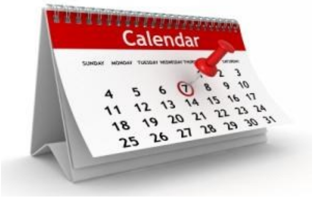<br>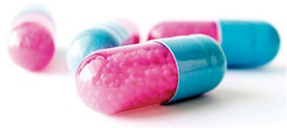

If you take Medicine A, you would live for **9.5 years** (9 years and 6 months) and then die.

If you take Medicine B, you would live for **10 years** and then die.

Would you prefer Medicine A or Medicine B, or are they the same?

Indicate your choice here. You can only choose one option:

○ **Medicine A** 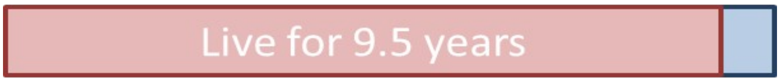

○ **Medicine B** 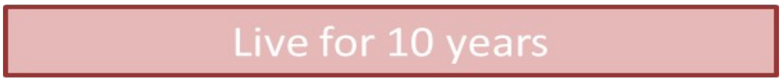

○ **Medicine A and Medicine B are the same.**

For peer review only

Repeated section

Remember Medicine A and Medicine B are equally effective and have no risks of a side effect.

The only difference between Medicine A and Medicine B is how you take them.

**Medicine A**	**Medicine B**
You take a tablet only once in your life. You will be given the tablet on the day you come in to see your GP. 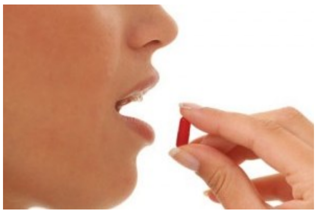	One tablet should be taken once every day, at night with cold water. You need to take this medicine for 10 years. You need to regularly visit a GP to receive a prescription. You need to visit a pharmacy to fill a prescription. 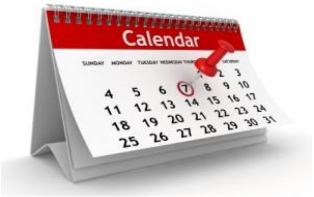 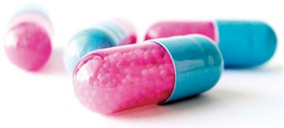

If you take Medicine A, you would live for **5 months less than 10 years** and then die.

If you take Medicine B, you would live for **10 years** and then die.

Would you prefer Medicine A or Medicine B, or are they the same?

Indicate your choice here. You can only choose one option:

◯ **Medicine A** Live for (10 years – 5 months)

◯ **Medicine B** Live for 10 years

◯ **Medicine A and Medicine B are the same.**

0% 100%

For peer review only

Repeated section

Remember Medicine A and Medicine B are equally effective and have no risks of a side effect.

The only difference between Medicine A and Medicine B is how you take them.

Medicine A	Medicine B
You take a tablet only once in your life.<br>You will be given the tablet on the day you come in to see your GP.<br>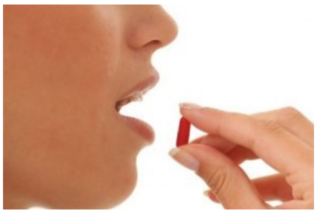	One tablet should be taken once every day, at night with cold water.<br>You need to take this medicine for 10 years.<br>You need to regularly visit a GP to receive a prescription.<br>You need to visit a pharmacy to fill a prescription.<br>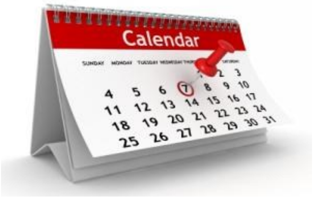<br><br>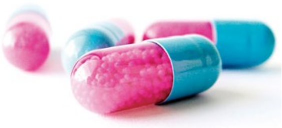

If you take Medicine A, you would live for **4 months less than 10 years** and then die.

If you take Medicine B, you would live for **10 years** and then die.

Would you prefer Medicine A or Medicine B, or are they the same?

Indicate your choice here. You can only choose one option:

◯ **Medicine A** Live for (10 years – 4 months)

◯ **Medicine B** Live for 10 years

◯ **Medicine A and Medicine B are the same.**

0% 100%

For peer review only

Repeated section

Remember Medicine A and Medicine B are equally effective and have no risks of a side effect.

The only difference between Medicine A and Medicine B is how you take them.

Medicine A	Medicine B
You take a tablet only once in your life.<br>You will be given the tablet on the day you come in to see your GP.<br>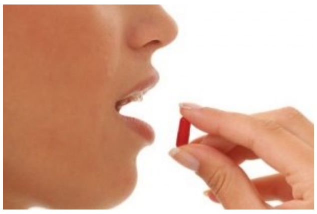	One tablet should be taken once every day, at night with cold water.<br>You need to take this medicine for 10 years.<br>You need to regularly visit a GP to receive a prescription.<br>You need to visit a pharmacy to fill a prescription.<br>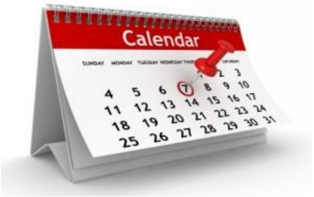<br><br>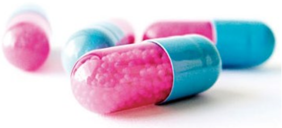

If you take Medicine A, you would live for **3 months less than 10 years** and then die.

If you take Medicine B, you would live for **10 years** and then die.

Would you prefer Medicine A or Medicine B, or are they the same?

Indicate your choice here. You can only choose one option:

◯ **Medicine A** Live for (10 years – 3 months)

◯ **Medicine B** Live for 10 years

◯ **Medicine A and Medicine B are the same.**

For peer review only

Repeated section

Remember Medicine A and Medicine B are equally effective and have no risks of a side effect.

The only difference between Medicine A and Medicine B is how you take them.

**Medicine A**	**Medicine B**
You take a tablet only once in your life.<br>You will be given the tablet on the day you come in to see your GP.<br>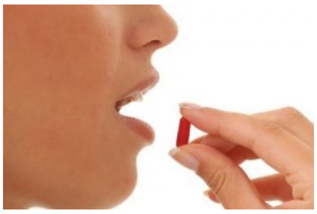	One tablet should be taken once every day, at night with cold water.<br>You need to take this medicine for 10 years.<br>You need to regularly visit a GP to receive a prescription.<br>You need to visit a pharmacy to fill a prescription.<br>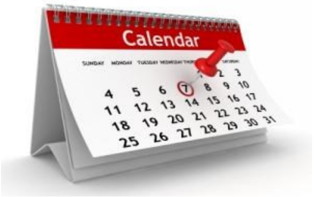<br>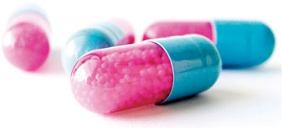

If you take Medicine A, you would live for **2 months less than 10 years** and then die.

If you take Medicine B, you would live for **10 years** and then die.

Would you prefer Medicine A or Medicine B, or are they the same?

Indicate your choice here. You can only choose one option:

◯ **Medicine A** Live for (10 years – 2 months)

◯ **Medicine B** Live for 10 years

◯ **Medicine A and Medicine B are the same.**

For peer review only

Repeated section

Remember Medicine A and Medicine B are equally effective and have no risks of a side effect.

The only difference between Medicine A and Medicine B is how you take them.

**Medicine A**	**Medicine B**
You take a tablet only once in your life.<br>You will be given the tablet on the day you come in to see your GP.<br>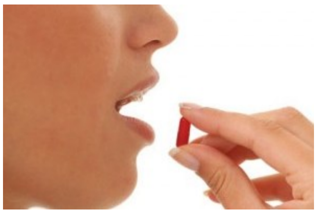	One tablet should be taken once every day, at night with cold water.<br>You need to take this medicine for 10 years.<br>You need to regularly visit a GP to receive a prescription.<br>You need to visit a pharmacy to fill a prescription.<br>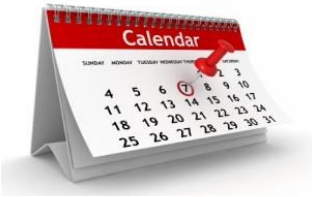<br>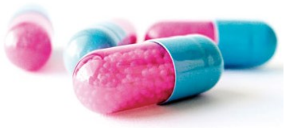

If you take Medicine A, you would live for **1 month less than 10 years** and then die.

If you take Medicine B, you would live for **10 years** and then die.

Would you prefer Medicine A or Medicine B, or are they the same?

Indicate your choice here. You can only choose one option:

○ **Medicine A** Live for (10 years – 1 month)

○ **Medicine B** Live for 10 years

○ **Medicine A and Medicine B are the same.**

For peer review only

Repeated section

Remember Medicine A and Medicine B are equally effective and have no risks of a side effect.

The only difference between Medicine A and Medicine B is how you take them.

Medicine A	Medicine B
You take a tablet only once in your life.<br>You will be given the tablet on the day you come in to see your GP.<br>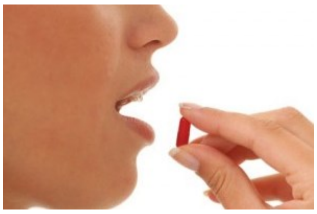	One tablet should be taken once every day, at night with cold water.<br>You need to take this medicine for 10 years.<br>You need to regularly visit a GP to receive a prescription.<br>You need to visit a pharmacy to fill a prescription.<br>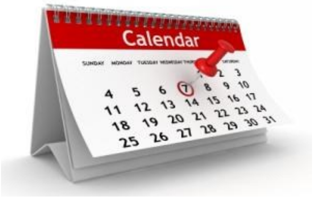<br><br>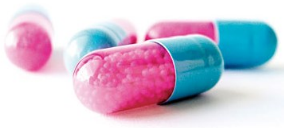

If you take Medicine A, you would live for **3 weeks less than 10 years** and then die.

If you take Medicine B, you would live for **10 years** and then die.

Would you prefer Medicine A or Medicine B, or are they the same?

Indicate your choice here. You can only choose one option:

◯ **Medicine A** Live for (10 years – 3 weeks)

◯ **Medicine B** Live for 10 years

◯ **Medicine A and Medicine B are the same.**

For peer review only

Repeated section

Remember Medicine A and Medicine B are equally effective and have no risks of a side effect.

The only difference between Medicine A and Medicine B is how you take them.

Medicine A	Medicine B
You take a tablet only once in your life.<br>You will be given the tablet on the day you come in to see your GP.<br>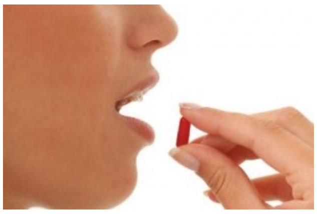	One tablet should be taken once every day, at night with cold water.<br>You need to take this medicine for 10 years.<br>You need to regularly visit a GP to receive a prescription.<br>You need to visit a pharmacy to fill a prescription.<br>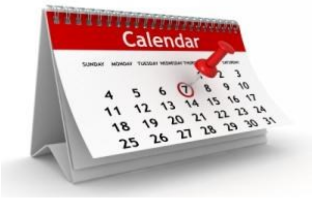<br>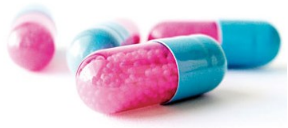

If you take Medicine A, you would live for **2 weeks less than 10 years** and then die.

If you take Medicine B, you would live for **10 years** and then die.

Would you prefer Medicine A or Medicine B, or are they the same?

Indicate your choice here. You can only choose one option:

○ **Medicine A** Live for (10 years – 2 weeks)

○ **Medicine B** Live for 10 years

○ **Medicine A and Medicine B are the same.**

For peer review only

Repeated section

Remember Medicine A and Medicine B are equally effective and have no risks of a side effect.

The only difference between Medicine A and Medicine B is how you take them.

Medicine A	Medicine B
You take a tablet only once in your life.<br>You will be given the tablet on the day you come in to see your GP.<br>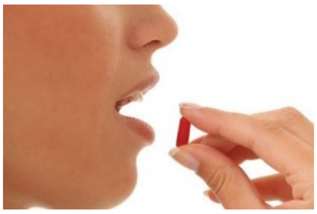	One tablet should be taken once every day, at night with cold water.<br>You need to take this medicine for 10 years.<br>You need to regularly visit a GP to receive a prescription.<br>You need to visit a pharmacy to fill a prescription.<br>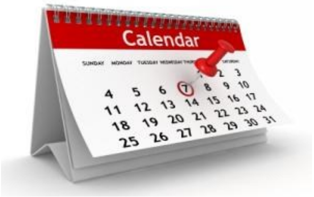<br>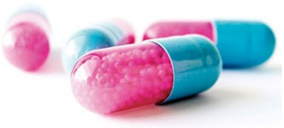

If you take Medicine A, you would live for **10 days less than 10 years** and then die.

If you take Medicine B, you would live for **10 years** and then die.

Would you prefer Medicine A or Medicine B, or are they the same?

Indicate your choice here. You can only choose one option:

○ **Medicine A** Live for (10 years – 10 days)

○ **Medicine B** Live for 10 years

○ **Medicine A and Medicine B are the same.**

For peer review only

Repeated section

Remember Medicine A and Medicine B are equally effective and have no risks of a side effect.

The only difference between Medicine A and Medicine B is how you take them.

**Medicine A**	**Medicine B**
You take a tablet only once in your life.<br>You will be given the tablet on the day you come in to see your GP.<br>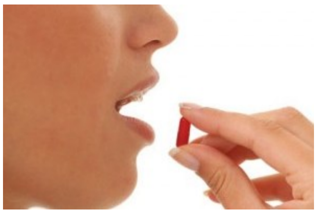	One tablet should be taken once every day, at night with cold water.<br>You need to take this medicine for 10 years.<br>You need to regularly visit a GP to receive a prescription.<br>You need to visit a pharmacy to fill a prescription.<br>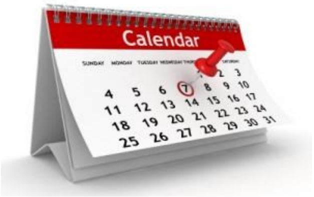<br>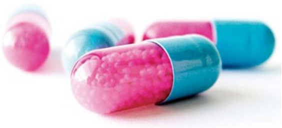

If you take Medicine A, you would live for **1 week less than 10 years** and then die.

If you take Medicine B, you would live for **10 years** and then die.

Would you prefer Medicine A or Medicine B, or are they the same?

Indicate your choice here. You can only choose one option:

○ **Medicine A** Live for (10 years – 1 week)

○ **Medicine B** Live for 10 years

○ **Medicine A and Medicine B are the same.**

For peer review only

Repeated section

Remember Medicine A and Medicine B are equally effective and have no risks of a side effect.

The only difference between Medicine A and Medicine B is how you take them.

Medicine A	Medicine B
You take a tablet only once in your life.<br>You will be given the tablet on the day you come in to see your GP.<br>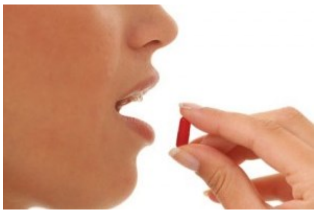	One tablet should be taken once every day, at night with cold water.<br>You need to take this medicine for 10 years.<br>You need to regularly visit a GP to receive a prescription.<br>You need to visit a pharmacy to fill a prescription.<br>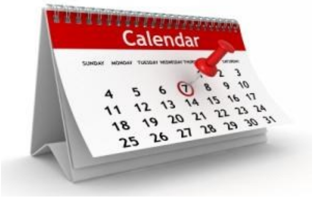<br>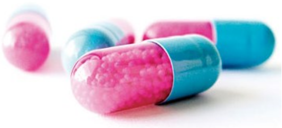

If you take Medicine A, you would live for **5 days less than 10 years** and then die.

If you take Medicine B, you would live for **10 years** and then die.

Would you prefer Medicine A or Medicine B, or are they the same?

Indicate your choice here. You can only choose one option:

○ **Medicine A** — Live for (10 years – 5 days)

○ **Medicine B** — Live for 10 years

○ **Medicine A and Medicine B are the same.**

For peer review only

Repeated section

Remember Medicine A and Medicine B are equally effective and have no risks of a side effect.

The only difference between Medicine A and Medicine B is how you take them.

**Medicine A**	**Medicine B**
You take a tablet only once in your life.<br>You will be given the tablet on the day you come in to see your GP.<br>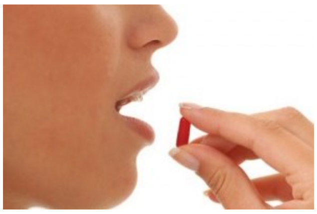	One tablet should be taken once every day, at night with cold water.<br>You need to take this medicine for 10 years.<br>You need to regularly visit a GP to receive a prescription.<br>You need to visit a pharmacy to fill a prescription.<br>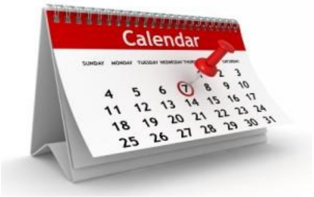 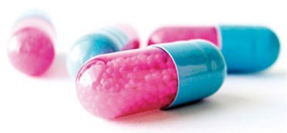

If you take Medicine A, you would live for **3 days less than 10 years** and then die.

If you take Medicine B, you would live for **10 years** and then die.

Would you prefer Medicine A or Medicine B, or are they the same?

Indicate your choice here. You can only choose one option:

◯ **Medicine A** Live for (10 years – 3 days)

◯ **Medicine B** Live for 10 years

◯ **Medicine A and Medicine B are the same.**

0% 100%

For peer review only

Repeated section

Remember Medicine A and Medicine B are equally effective and have no risks of a side effect.

The only difference between Medicine A and Medicine B is how you take them.

Medicine A	Medicine B
You take a tablet only once in your life.<br>You will be given the tablet on the day you come in to see your GP.<br>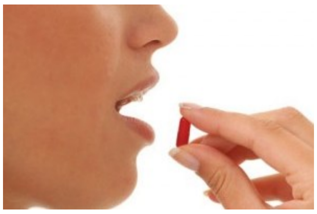	One tablet should be taken once every day, at night with cold water.<br>You need to take this medicine for 10 years.<br>You need to regularly visit a GP to receive a prescription.<br>You need to visit a pharmacy to fill a prescription.<br>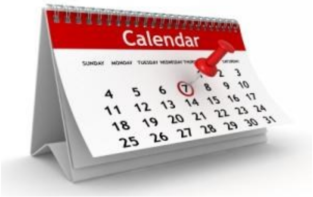<br><br>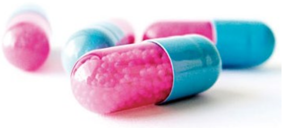

If you take Medicine A, you would live for **1 day less than 10 years** and then die.

If you take Medicine B, you would live for **10 years** and then die.

Would you prefer Medicine A or Medicine B, or are they the same?

Indicate your choice here. You can only choose one option:

○ **Medicine A** Live for (10 years – 1 day)

○ **Medicine B** Live for 10 years

○ **Medicine A and Medicine B are the same.**

0% 100%

For peer review only

Question 1a.

You prefer living for 5 years on Medicine A, to living for 10 years on Medicine B.

This means **living fewer than 5 years on Medicine A** can be the same as living for 10 years on Medicine B.

How many years on Medicine A do you think would be the same as living for 10 years on Medicine B?

(Please give a number in years)

0% 100%

peer review only

Part 2

Question 2

Now, we will assume that there is some risk of a minor side effect from either medicine.

As before,

Medicine A is a pill that you only need to take once.

Medicine B is a pill that you need to take every day for ten years.

Medicine A and Medicine B are equally effective in reducing your risk of developing heart disease.

Medicine A and Medicine B will reduce your risk of having a heart attack or a stroke within the next 10 years from **10%** to **6%**.

In other words, in a crowd of 1,000 people in the UK, **100** are likely to have a heart attack or a stroke if they do not take the medicine. This is reduced to **60** people out of 1,000 who do take the medicine. This information is also illustrated in this picture.

Risk of heart disease

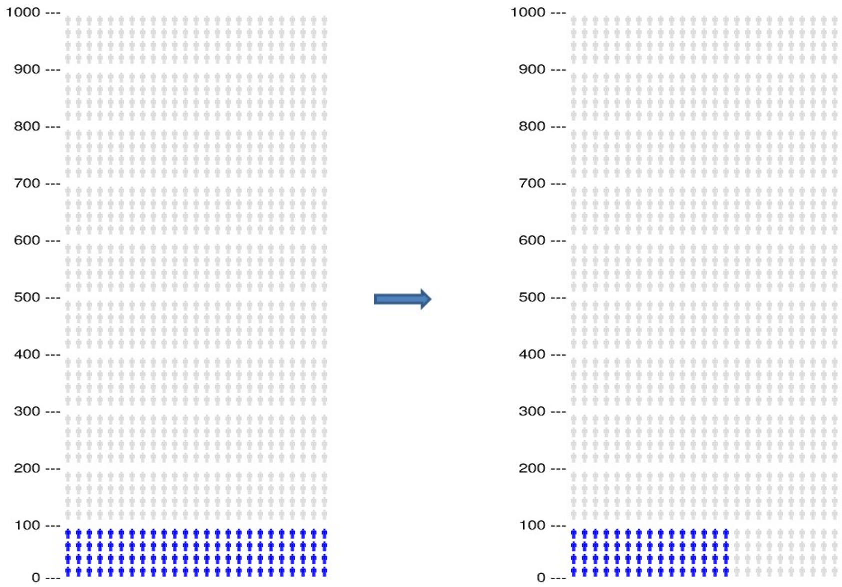

10%
No Treatment

6%
Taking the Medicine

(The figures shaded blue represent people who will have a heart attack or a stroke in the next 10 years)

In this scenario, there is some risk of a minor side effect from Medicine A or Medicine B.

Among every 1,000 people taking Medicine A or Medicine B, **30** people are likely to have a minor side effect such as muscle pain and fatigue.

Risk of a minor side effect

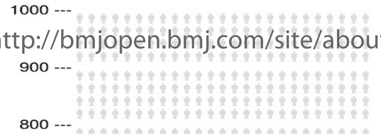

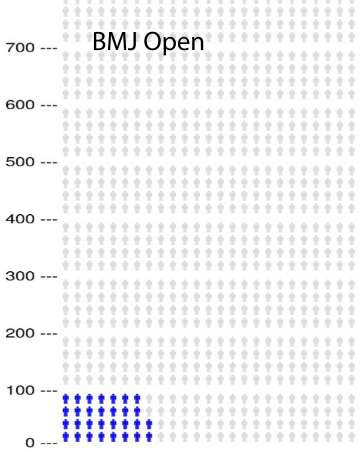

3% of people will have a minor side effect.

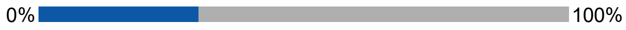

For peer review only

Repeated section

Remember Medicine A and Medicine B are equally effective and have **some risk of a side effect**.

The only difference between Medicine A and Medicine B is how you take them.

Medicine A	Medicine B
You take a tablet only once in your life.<br>You will be given the tablet on the day you come in to see your GP.<br>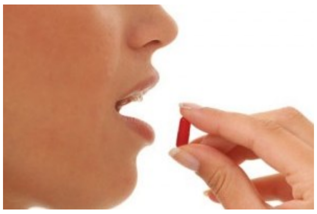	One tablet should be taken once every day, at night with cold water.<br>You need to take this medicine for 10 years.<br>You need to regularly visit a GP to receive a prescription.<br>You need to visit a pharmacy to fill a prescription.<br>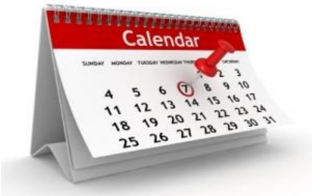<br>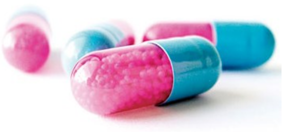

If you take Medicine A, you would live for **5 years** and then die.

If you take Medicine B, you would live for **10 years** and then die.

Would you prefer Medicine A or Medicine B, or are they the same?

Indicate your choice here. You can only choose one option:

○ **Medicine A** 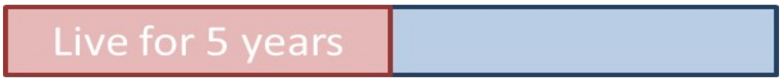

○ **Medicine B** 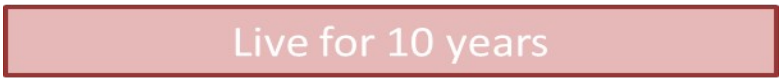

○ **Medicine A and Medicine B are the same.**

For peer review only

Repeated section

Remember Medicine A and Medicine B are equally effective and have **some risk of a side effect**.

The only difference between Medicine A and Medicine B is how you take them.

Medicine A	Medicine B
You take a tablet only once in your life. You will be given the tablet on the day you come in to see your GP. 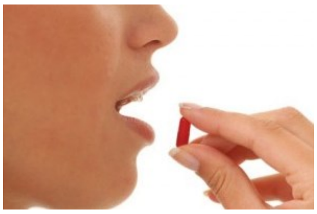	One tablet should be taken once every day, at night with cold water. You need to take this medicine for 10 years. You need to regularly visit a GP to receive a prescription. You need to visit a pharmacy to fill a prescription. 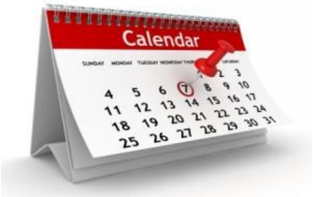 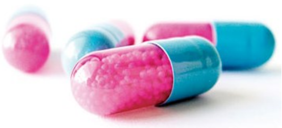

If you take Medicine A, you would live for **5.5 years** (5 years and 6 months) and then die.

If you take Medicine B, you would live for **10 years** and then die.

Would you prefer Medicine A or Medicine B, or are they the same?

Indicate your choice here. You can only choose one option:

◯ **Medicine A** Live for 5.5 years

◯ **Medicine B** Live for 10 years

◯ **Medicine A and Medicine B are the same.**

For peer review only

Repeated section

Remember Medicine A and Medicine B are equally effective and have **some risk of a side effect**.

The only difference between Medicine A and Medicine B is how you take them.

Medicine A	Medicine B
You take a tablet only once in your life.<br>You will be given the tablet on the day you come in to see your GP.<br>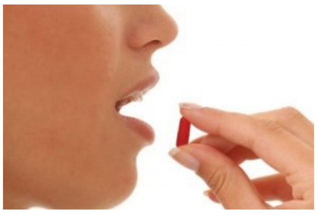	One tablet should be taken once every day, at night with cold water.<br>You need to take this medicine for 10 years.<br>You need to regularly visit a GP to receive a prescription.<br>You need to visit a pharmacy to fill a prescription.<br>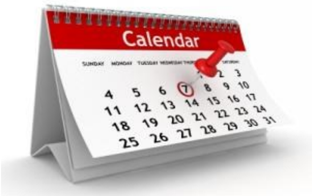<br>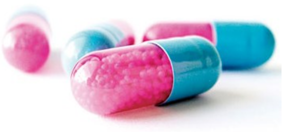

If you take Medicine A, you would live for **6 years** and then die.

If you take Medicine B, you would live for **10 years** and then die.

Would you prefer Medicine A or Medicine B, or are they the same?

Indicate your choice here. You can only choose one option:

○ **Medicine A** Live for 6 years

○ **Medicine B** Live for 10 years

○ **Medicine A and Medicine B are the same.**

For peer review only

Repeated section

Remember Medicine A and Medicine B are equally effective and have **some risk of a side effect**.

The only difference between Medicine A and Medicine B is how you take them.

Medicine A	Medicine B
You take a tablet only once in your life.<br>You will be given the tablet on the day you come in to see your GP.<br>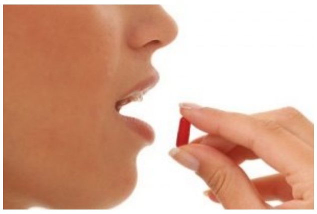	One tablet should be taken once every day, at night with cold water.<br>You need to take this medicine for 10 years.<br>You need to regularly visit a GP to receive a prescription.<br>You need to visit a pharmacy to fill a prescription.<br>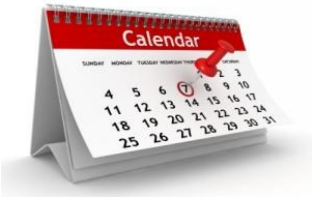 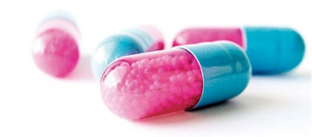

If you take Medicine A, you would live for **6.25 years (6 years and 3 months)** and then die.

If you take Medicine B, you would live for **10 years** and then die.

Would you prefer Medicine A or Medicine B, or are they the same?

Indicate your choice here. You can only choose one option:

◯ **Medicine A** Live for 6 years and 3 months

◯ **Medicine B** Live for 10 years

◯ **Medicine A and Medicine B are the same.**

For peer review only

Repeated section

Remember Medicine A and Medicine B are equally effective and have **some risk of a side effect**.

The only difference between Medicine A and Medicine B is how you take them.

**Medicine A**	**Medicine B**
You take a tablet only once in your life.<br>You will be given the tablet on the day you come in to see your GP.<br>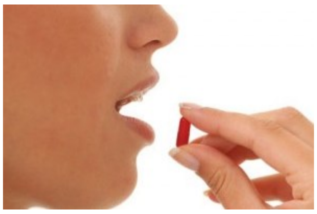	One tablet should be taken once every day, at night with cold water.<br>You need to take this medicine for 10 years.<br>You need to regularly visit a GP to receive a prescription.<br>You need to visit a pharmacy to fill a prescription.<br>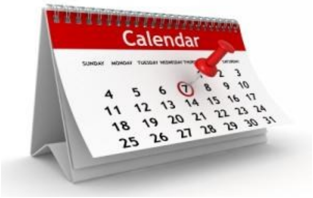<br>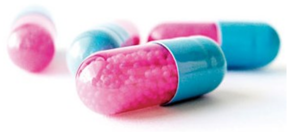

If you take Medicine A, you would live for **6.5 years** (6 years and 6 months) and then die.

If you take Medicine B, you would live for **10 years** and then die.

Would you prefer Medicine A or Medicine B, or are they the same?

Indicate your choice here. You can only choose one option:

◯ **Medicine A** Live for 6.5 years

◯ **Medicine B** Live for 10 years

◯ **Medicine A and Medicine B are the same.**

0% 100%

For peer review only

Repeated section

Remember Medicine A and Medicine B are equally effective and have **some risk of a side effect**.

The only difference between Medicine A and Medicine B is how you take them.

**Medicine A**	**Medicine B**
You take a tablet only once in your life.<br>You will be given the tablet on the day you come in to see your GP.<br>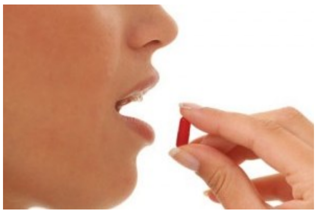	One tablet should be taken once every day, at night with cold water.<br>You need to take this medicine for 10 years.<br>You need to regularly visit a GP to receive a prescription.<br>You need to visit a pharmacy to fill a prescription.<br>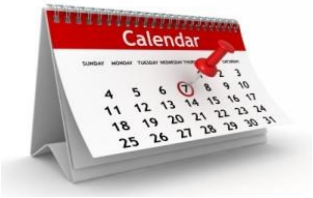<br>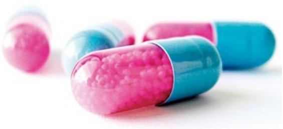

If you take Medicine A, you would live for **7 years** and then die.

If you take Medicine B, you would live for **10 years** and then die.

Would you prefer Medicine A or Medicine B, or are they the same?

Indicate your choice here. You can only choose one option:

○ **Medicine A** Live for 7 years

○ **Medicine B** Live for 10 years

○ **Medicine A and Medicine B are the same.**

0% 100%

For peer review only

Repeated section

Remember Medicine A and Medicine B are equally effective and have **some risk of a side effect**.

The only difference between Medicine A and Medicine B is how you take them.

Medicine A	Medicine B
You take a tablet only once in your life.<br>You will be given the tablet on the day you come in to see your GP.<br>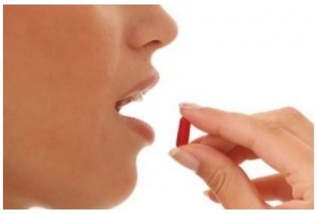	One tablet should be taken once every day, at night with cold water.<br>You need to take this medicine for 10 years.<br>You need to regularly visit a GP to receive a prescription.<br>You need to visit a pharmacy to fill a prescription.<br>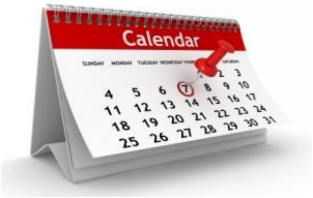<br>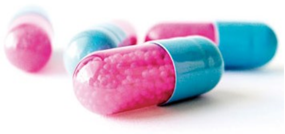

If you take Medicine A, you would live for **7.5 years** (7 years and 6 months) and then die.

If you take Medicine B, you would live for **10 years** and then die.

Would you prefer Medicine A or Medicine B, or are they the same?

Indicate your choice here. You can only choose one option:

○ **Medicine A** Live for 7.5 years

○ **Medicine B** Live for 10 years

○ **Medicine A and Medicine B are the same.**

For peer review only

Repeated section

Remember Medicine A and Medicine B are equally effective and have **some risk of a side effect**.

The only difference between Medicine A and Medicine B is how you take them.

Medicine A	Medicine B
You take a tablet only once in your life.<br>You will be given the tablet on the day you come in to see your GP.<br>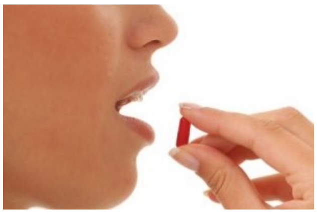	One tablet should be taken once every day, at night with cold water.<br>You need to take this medicine for 10 years.<br>You need to regularly visit a GP to receive a prescription.<br>You need to visit a pharmacy to fill a prescription.<br>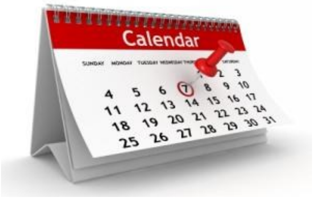<br>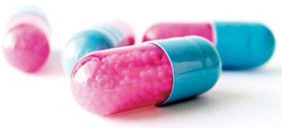

If you take Medicine A, you would live for **7.75 years (7 years and 9 months)** and then die.

If you take Medicine B, you would live for **10 years** and then die.

Would you prefer Medicine A or Medicine B, or are they the same?

Indicate your choice here. You can only choose one option:

○ **Medicine A** 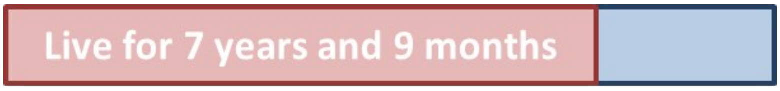

○ **Medicine B** 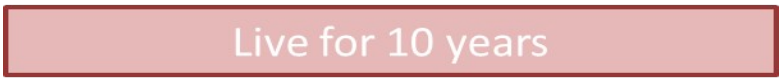

○ **Medicine A and Medicine B are the same.**

For peer review only

Repeated section

Remember Medicine A and Medicine B are equally effective and have **some risk of a side effect**.

The only difference between Medicine A and Medicine B is how you take them.

Medicine A	Medicine B
You take a tablet only once in your life.<br>You will be given the tablet on the day you come in to see your GP.<br>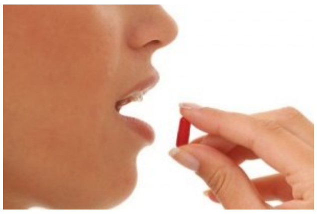	One tablet should be taken once every day, at night with cold water.<br>You need to take this medicine for 10 years.<br>You need to regularly visit a GP to receive a prescription.<br>You need to visit a pharmacy to fill a prescription.<br>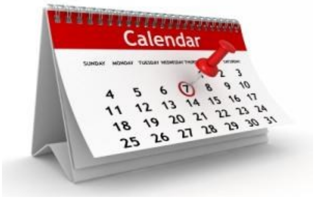<br>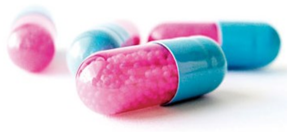

If you take Medicine A, you would live for **8 years** and then die.

If you take Medicine B, you would live for **10 years** and then die.

Would you prefer Medicine A or Medicine B, or are they the same?

Indicate your choice here. You can only choose one option:

○ **Medicine A** Live for 8 years

○ **Medicine B** Live for 10 years

○ **Medicine A and Medicine B are the same.**

For peer review only

Repeated section

Remember Medicine A and Medicine B are equally effective and have **some risk of a side effect**.

The only difference between Medicine A and Medicine B is how you take them.

**Medicine A**	**Medicine B**
You take a tablet only once in your life.<br>You will be given the tablet on the day you come in to see your GP.<br>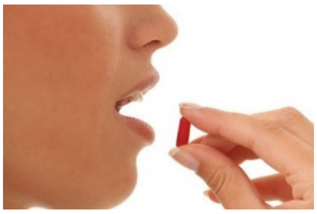	One tablet should be taken once every day, at night with cold water.<br>You need to take this medicine for 10 years.<br>You need to regularly visit a GP to receive a prescription.<br>You need to visit a pharmacy to fill a prescription.<br>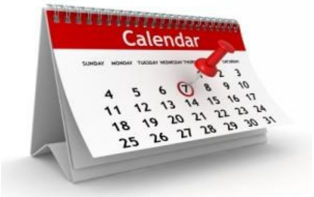<br>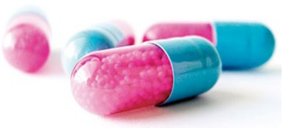

If you take Medicine A, you would live for **8.25 years (8 years and 3 months)** and then die.

If you take Medicine B, you would live for **10 years** and then die.

Would you prefer Medicine A or Medicine B, or are they the same?

Indicate your choice here. You can only choose one option:

○ **Medicine A** Live for 8 years and 3 months

○ **Medicine B** Live for 10 years

○ **Medicine A and Medicine B are the same.**

0% 100%

For peer review only

Repeated section

Remember Medicine A and Medicine B are equally effective and have **some risk of a side effect**.

The only difference between Medicine A and Medicine B is how you take them.

Medicine A	Medicine B
You take a tablet only once in your life.<br>You will be given the tablet on the day you come in to see your GP.<br>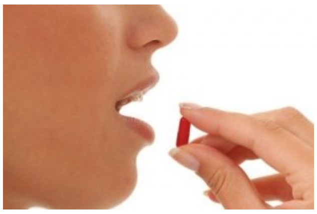	One tablet should be taken once every day, at night with cold water.<br>You need to take this medicine for 10 years.<br>You need to regularly visit a GP to receive a prescription.<br>You need to visit a pharmacy to fill a prescription.<br>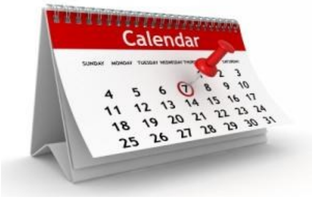<br>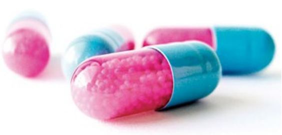

If you take Medicine A, you would live for **8.5 years** (8 years and 6 months) and then die.

If you take Medicine B, you would live for **10 years** and then die.

Would you prefer Medicine A or Medicine B, or are they the same?

Indicate your choice here. You can only choose one option:

○ **Medicine A** Live for 8.5 years

○ **Medicine B** Live for 10 years

○ **Medicine A and Medicine B are the same.**

For peer review only

Repeated section

Remember Medicine A and Medicine B are equally effective and have **some risk of a side effect**.

The only difference between Medicine A and Medicine B is how you take them.

**Medicine A**	**Medicine B**
You take a tablet only once in your life.<br>You will be given the tablet on the day you come in to see your GP.<br>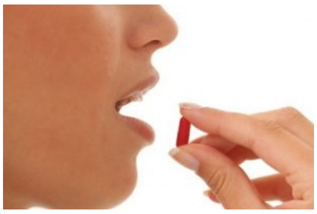	One tablet should be taken once every day, at night with cold water.<br>You need to take this medicine for 10 years.<br>You need to regularly visit a GP to receive a prescription.<br>You need to visit a pharmacy to fill a prescription.<br>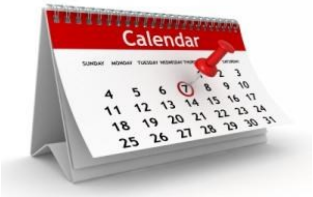<br><br>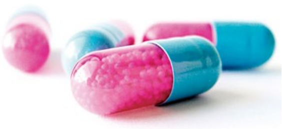

If you take Medicine A, you would live for **8.75 years (8 years and 9 months)** and then die.

If you take Medicine B, you would live for **10 years** and then die.

Would you prefer Medicine A or Medicine B, or are they the same?

Indicate your choice here. You can only choose one option:

◯ **Medicine A** Live for 8 years and 9 months

◯ **Medicine B** Live for 10 years

◯ **Medicine A and Medicine B are the same.**

For peer review only

Repeated section

Remember Medicine A and Medicine B are equally effective and have **some risk of a side effect**.

The only difference between Medicine A and Medicine B is how you take them.

**Medicine A**	**Medicine B**
You take a tablet only once in your life.<br>You will be given the tablet on the day you come in to see your GP.<br>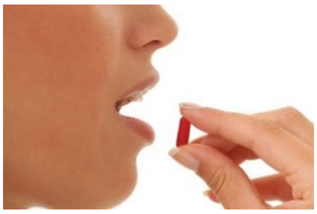	One tablet should be taken once every day, at night with cold water.<br>You need to take this medicine for 10 years.<br>You need to regularly visit a GP to receive a prescription.<br>You need to visit a pharmacy to fill a prescription.<br>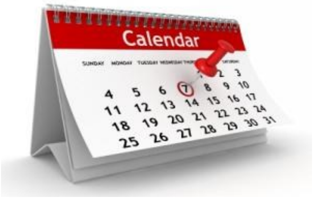<br>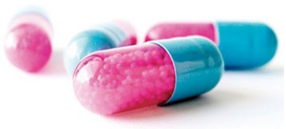

If you take Medicine A, you would live for **9 years** and then die.

If you take Medicine B, you would live for **10 years** and then die.

Would you prefer Medicine A or Medicine B, or are they the same?

Indicate your choice here. You can only choose one option:

◯ **Medicine A** Live for 9 years

◯ **Medicine B** Live for 10 years

◯ **Medicine A and Medicine B are the same.**

0% 100%

For peer review only

Repeated section

Remember Medicine A and Medicine B are equally effective and have **some risk of a side effect**.

The only difference between Medicine A and Medicine B is how you take them.

Medicine A	Medicine B
You take a tablet only once in your life.<br>You will be given the tablet on the day you come in to see your GP.<br>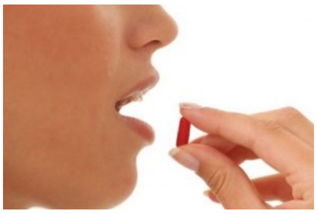	One tablet should be taken once every day, at night with cold water.<br>You need to take this medicine for 10 years.<br>You need to regularly visit a GP to receive a prescription.<br>You need to visit a pharmacy to fill a prescription.<br>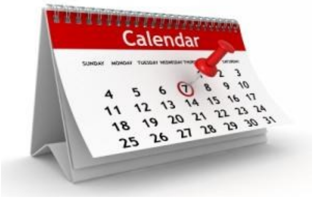<br><br>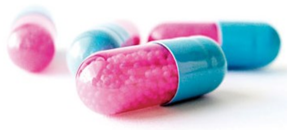

If you take Medicine A, you would live for **9 years and 1 month** and then die.

If you take Medicine B, you would live for **10 years** and then die.

Would you prefer Medicine A or Medicine B, or are they the same?

Indicate your choice here. You can only choose one option:

○ **Medicine A** Live for 9 years 1 month

○ **Medicine B** Live for 10 years

○ **Medicine A and Medicine B are the same.**

For peer review only

Repeated section

Remember Medicine A and Medicine B are equally effective and have **some risk of a side effect**.

The only difference between Medicine A and Medicine B is how you take them.

Medicine A	Medicine B
You take a tablet only once in your life.<br>You will be given the tablet on the day you come in to see your GP.<br>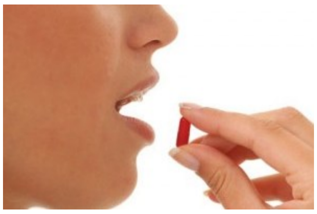	One tablet should be taken once every day, at night with cold water.<br>You need to take this medicine for 10 years.<br>You need to regularly visit a GP to receive a prescription.<br>You need to visit a pharmacy to fill a prescription.<br>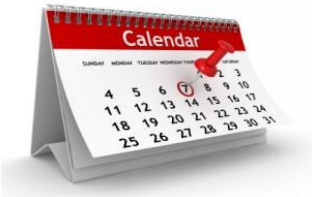<br>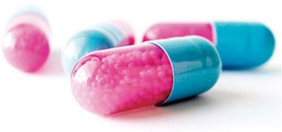

If you take Medicine A, you would live for **9.25 years (9 years and 3 months)** and then die.

If you take Medicine B, you would live for **10 years** and then die.

Would you prefer Medicine A or Medicine B, or are they the same?

Indicate your choice here. You can only choose one option:

◯ **Medicine A** Live for 9 years and 3 months

◯ **Medicine B** Live for 10 years

◯ **Medicine A and Medicine B are the same.**

0% 100%

For peer review only

Repeated section

Remember Medicine A and Medicine B are equally effective and have **some risk of a side effect**.

The only difference between Medicine A and Medicine B is how you take them.

**Medicine A**	**Medicine B**
You take a tablet only once in your life.<br>You will be given the tablet on the day you come in to see your GP.<br>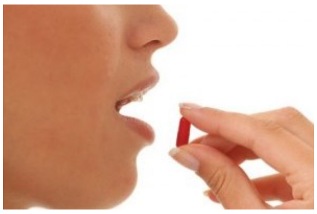	One tablet should be taken once every day, at night with cold water.<br>You need to take this medicine for 10 years.<br>You need to regularly visit a GP to receive a prescription.<br>You need to visit a pharmacy to fill a prescription.<br>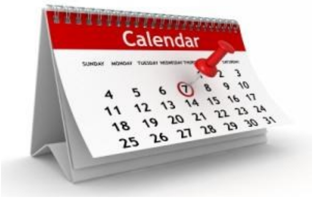<br><br>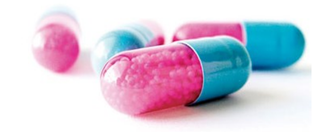

If you take Medicine A, you would live for **9 years and 4 months** and then die.

If you take Medicine B, you would live for **10 years** and then die.

Would you prefer Medicine A or Medicine B, or are they the same?

Indicate your choice here. You can only choose one option:

◯ **Medicine A** Live for 9 years 4 months

◯ **Medicine B** Live for 10 years

◯ **Medicine A and Medicine B are the same.**

For peer review only

Repeated section

Remember Medicine A and Medicine B are equally effective and have **some risk of a side effect**.

The only difference between Medicine A and Medicine B is how you take them.

Medicine A	Medicine B
You take a tablet only once in your life.<br>You will be given the tablet on the day you come in to see your GP.<br>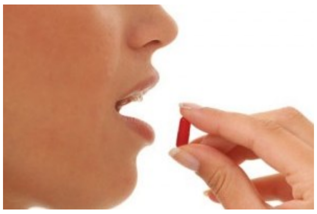	One tablet should be taken once every day, at night with cold water.<br>You need to take this medicine for 10 years.<br>You need to regularly visit a GP to receive a prescription.<br>You need to visit a pharmacy to fill a prescription.<br>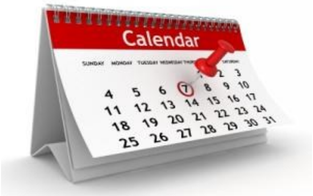<br>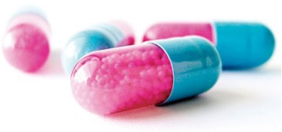

If you take Medicine A, you would live for **9 years and 5 months** and then die.

If you take Medicine B, you would live for **10 years** and then die.

Would you prefer Medicine A or Medicine B, or are they the same?

Indicate your choice here. You can only choose one option:

◯ **Medicine A** Live for 9 years 5 months

◯ **Medicine B** Live for 10 years

◯ **Medicine A and Medicine B are the same.**

0% 100%

For peer review only

Repeated section

Remember Medicine A and Medicine B are equally effective and have **some risk of a side effect**.

The only difference between Medicine A and Medicine B is how you take them.

**Medicine A**	**Medicine B**
You take a tablet only once in your life.<br>You will be given the tablet on the day you come in to see your GP.<br>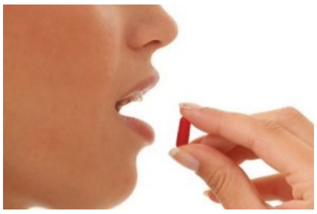	One tablet should be taken once every day, at night with cold water.<br>You need to take this medicine for 10 years.<br>You need to regularly visit a GP to receive a prescription.<br>You need to visit a pharmacy to fill a prescription.<br>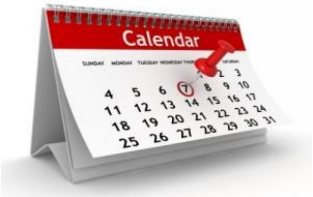<br>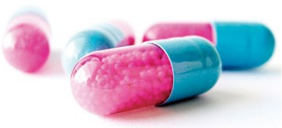

If you take Medicine A, you would live for **9.5 years** (9 years and 6 months) and then die.

If you take Medicine B, you would live for **10 years** and then die.

Would you prefer Medicine A or Medicine B, or are they the same?

Indicate your choice here. You can only choose one option:

○ **Medicine A** Live for 9.5 years

○ **Medicine B** Live for 10 years

○ **Medicine A and Medicine B are the same.**

0% 100%

For peer review only

Repeated section

Remember Medicine A and Medicine B are equally effective and have **some risk of a side effect**.

The only difference between Medicine A and Medicine B is how you take them.

Medicine A	Medicine B
You take a tablet only once in your life. You will be given the tablet on the day you come in to see your GP. 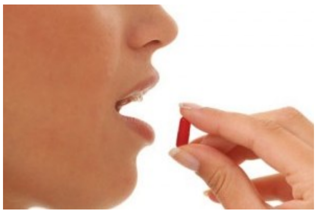	One tablet should be taken once every day, at night with cold water. You need to take this medicine for 10 years. You need to regularly visit a GP to receive a prescription. You need to visit a pharmacy to fill a prescription. 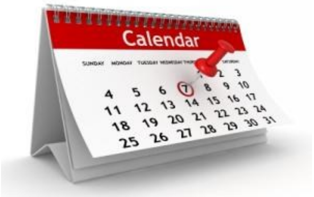 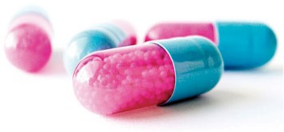

If you take Medicine A, you would live for **5 months less than 10 years** and then die.

If you take Medicine B, you would live for **10 years** and then die.

Would you prefer Medicine A or Medicine B, or are they the same?

Indicate your choice here. You can only choose one option:

○ **Medicine A** Live for (10 years – 5 months)

○ **Medicine B** Live for 10 years

○ **Medicine A and Medicine B are the same.**

For peer review only

Repeated section

Remember Medicine A and Medicine B are equally effective and have **some risk of a side effect**.

The only difference between Medicine A and Medicine B is how you take them.

**Medicine A**	**Medicine B**
You take a tablet only once in your life.<br>You will be given the tablet on the day you come in to see your GP.<br>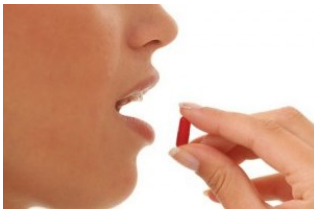	One tablet should be taken once every day, at night with cold water.<br>You need to take this medicine for 10 years.<br>You need to regularly visit a GP to receive a prescription.<br>You need to visit a pharmacy to fill a prescription.<br>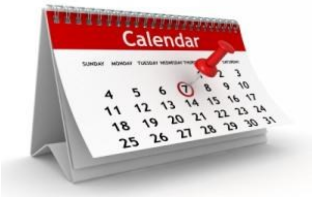<br>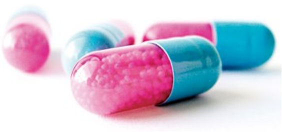

If you take Medicine A, you would live for **4 months less than 10 years** and then die.

If you take Medicine B, you would live for **10 years** and then die.

Would you prefer Medicine A or Medicine B, or are they the same?

Indicate your choice here. You can only choose one option:

○ **Medicine A** Live for (10 years – 4 months)

○ **Medicine B** Live for 10 years

○ **Medicine A and Medicine B are the same.**

0% 100%

For peer review only

Repeated section

Remember Medicine A and Medicine B are equally effective and have **some risk of a side effect**.

The only difference between Medicine A and Medicine B is how you take them.

Medicine A	Medicine B
You take a tablet only once in your life.<br>You will be given the tablet on the day you come in to see your GP.<br>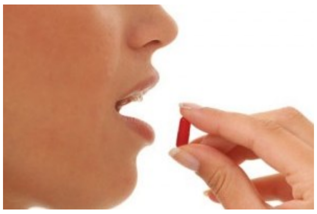	One tablet should be taken once every day, at night with cold water.<br>You need to take this medicine for 10 years.<br>You need to regularly visit a GP to receive a prescription.<br>You need to visit a pharmacy to fill a prescription.<br>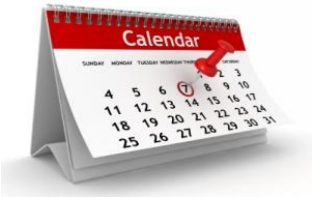 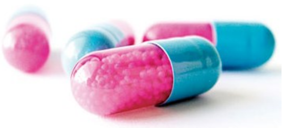

If you take Medicine A, you would live for **3 months less than 10 years** and then die.

If you take Medicine B, you would live for **10 years** and then die.

Would you prefer Medicine A or Medicine B, or are they the same?

Indicate your choice here. You can only choose one option:

○ **Medicine A** — Live for (10 years – 3 months)

○ **Medicine B** — Live for 10 years

○ **Medicine A and Medicine B are the same.**

For peer review only

Repeated section

Remember Medicine A and Medicine B are equally effective and have **some risk of a side effect**.

The only difference between Medicine A and Medicine B is how you take them.

**Medicine A**	**Medicine B**
You take a tablet only once in your life.<br>You will be given the tablet on the day you come in to see your GP.<br>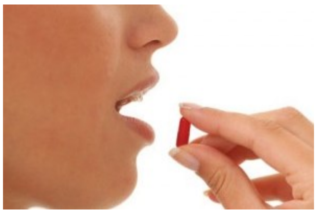	One tablet should be taken once every day, at night with cold water.<br>You need to take this medicine for 10 years.<br>You need to regularly visit a GP to receive a prescription.<br>You need to visit a pharmacy to fill a prescription.<br>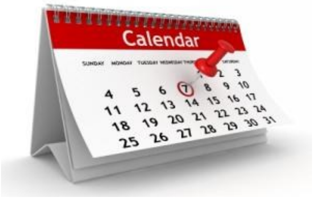<br>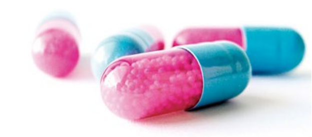

If you take Medicine A, you would live for **2 months less than 10 years** and then die.

If you take Medicine B, you would live for **10 years** and then die.

Would you prefer Medicine A or Medicine B, or are they the same?

Indicate your choice here. You can only choose one option:

○ **Medicine A** Live for (10 years – 2 months)

○ **Medicine B** Live for 10 years

○ **Medicine A and Medicine B are the same.**

For peer review only

Repeated section

Remember Medicine A and Medicine B are equally effective and have **some risk of a side effect**.

The only difference between Medicine A and Medicine B is how you take them.

Medicine A	Medicine B
You take a tablet only once in your life.<br>You will be given the tablet on the day you come in to see your GP.<br>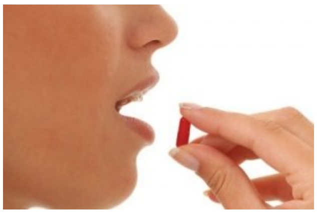	One tablet should be taken once every day, at night with cold water.<br>You need to take this medicine for 10 years.<br>You need to regularly visit a GP to receive a prescription.<br>You need to visit a pharmacy to fill a prescription.<br>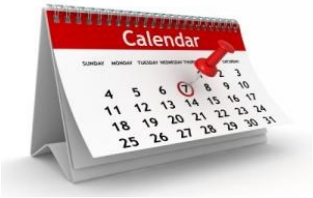<br><br>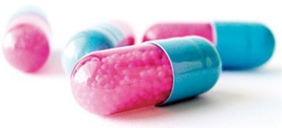

If you take Medicine A, you would live for **1 month less than 10 years** and then die.

If you take Medicine B, you would live for **10 years** and then die.

Would you prefer Medicine A or Medicine B, or are they the same?

Indicate your choice here. You can only choose one option:

○ **Medicine A** Live for (10 years – 1 month)

○ **Medicine B** Live for 10 years

○ **Medicine A and Medicine B are the same.**

0% 100%

For peer review only

Repeated section

Remember Medicine A and Medicine B are equally effective and have **some risk of a side effect**.

The only difference between Medicine A and Medicine B is how you take them.

Medicine A	Medicine B
You take a tablet only once in your life.<br>You will be given the tablet on the day you come in to see your GP.<br>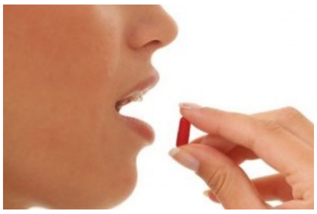	One tablet should be taken once every day, at night with cold water.<br>You need to take this medicine for 10 years.<br>You need to regularly visit a GP to receive a prescription.<br>You need to visit a pharmacy to fill a prescription.<br>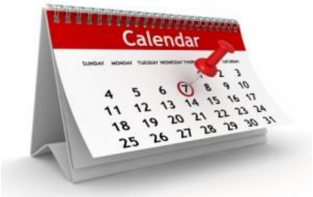<br>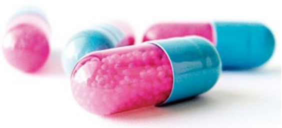

If you take Medicine A, you would live for **3 weeks less than 10 years** and then die.

If you take Medicine B, you would live for **10 years** and then die.

Would you prefer Medicine A or Medicine B, or are they the same?

Indicate your choice here. You can only choose one option:

○ **Medicine A** Live for (10 years – 3 weeks)

○ **Medicine B** Live for 10 years

○ **Medicine A and Medicine B are the same.**

For peer review only

Repeated section

Remember Medicine A and Medicine B are equally effective and have **some risk of a side effect**.

The only difference between Medicine A and Medicine B is how you take them.

**Medicine A**	**Medicine B**
You take a tablet only once in your life.<br>You will be given the tablet on the day you come in to see your GP.<br>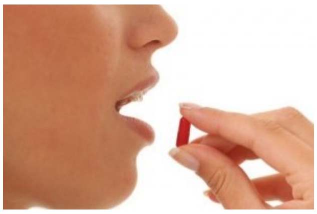	One tablet should be taken once every day, at night with cold water.<br>You need to take this medicine for 10 years.<br>You need to regularly visit a GP to receive a prescription.<br>You need to visit a pharmacy to fill a prescription.<br>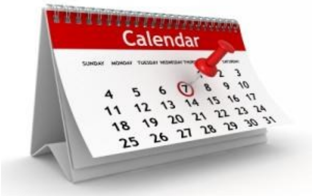<br>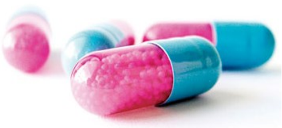

If you take Medicine A, you would live for **2 weeks less than 10 years** and then die.

If you take Medicine B, you would live for **10 years** and then die.

Would you prefer Medicine A or Medicine B, or are they the same?

Indicate your choice here. You can only choose one option:

◯ **Medicine A** Live for (10 years – 2 weeks)

◯ **Medicine B** Live for 10 years

◯ **Medicine A and Medicine B are the same.**

0% 100%

For peer review only

Repeated section

Remember Medicine A and Medicine B are equally effective and have **some risk of a side effect**.

The only difference between Medicine A and Medicine B is how you take them.

Medicine A	Medicine B
You take a tablet only once in your life.<br>You will be given the tablet on the day you come in to see your GP.<br>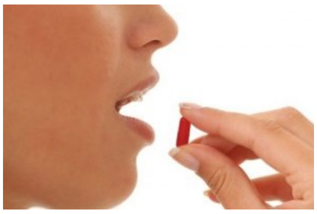	One tablet should be taken once every day, at night with cold water.<br>You need to take this medicine for 10 years.<br>You need to regularly visit a GP to receive a prescription.<br>You need to visit a pharmacy to fill a prescription.<br>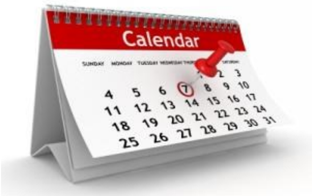<br>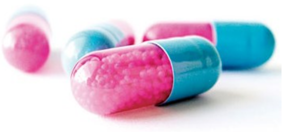

If you take Medicine A, you would live for **10 days less than 10 years** and then die.

If you take Medicine B, you would live for **10 years** and then die.

Would you prefer Medicine A or Medicine B, or are they the same?

Indicate your choice here. You can only choose one option:

○ **Medicine A** Live for (10 years – 10 days)

○ **Medicine B** Live for 10 years

○ **Medicine A and Medicine B are the same.**

0% 100%

For peer review only

Repeated section

Remember Medicine A and Medicine B are equally effective and have **some risk of a side effect**.

The only difference between Medicine A and Medicine B is how you take them.

Medicine A	Medicine B
You take a tablet only once in your life.<br>You will be given the tablet on the day you come in to see your GP.<br>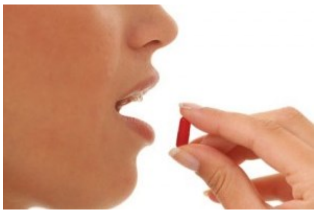	One tablet should be taken once every day, at night with cold water.<br>You need to take this medicine for 10 years.<br>You need to regularly visit a GP to receive a prescription.<br>You need to visit a pharmacy to fill a prescription.<br>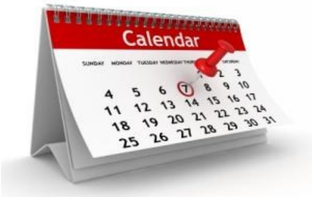 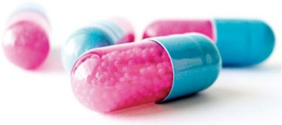

If you take Medicine A, you would live for **1 week less than 10 years** and then die.

If you take Medicine B, you would live for **10 years** and then die.

Would you prefer Medicine A or Medicine B, or are they the same?

Indicate your choice here. You can only choose one option:

○ **Medicine A** Live for (10 years – 1 week)

○ **Medicine B** Live for 10 years

○ **Medicine A and Medicine B are the same.**

For peer review only

Repeated section

Remember Medicine A and Medicine B are equally effective and have **some risk of a side effect**.

The only difference between Medicine A and Medicine B is how you take them.

Medicine A	Medicine B
You take a tablet only once in your life.<br>You will be given the tablet on the day you come in to see your GP.<br>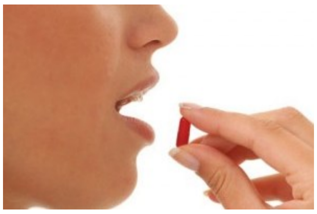	One tablet should be taken once every day, at night with cold water.<br>You need to take this medicine for 10 years.<br>You need to regularly visit a GP to receive a prescription.<br>You need to visit a pharmacy to fill a prescription.<br>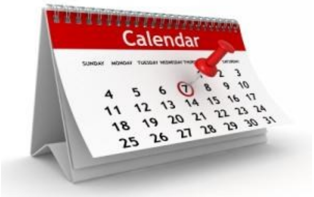 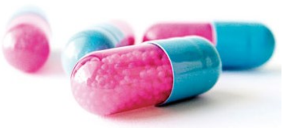

If you take Medicine A, you would live for **5 days less than 10 years** and then die.

If you take Medicine B, you would live for **10 years** and then die.

Would you prefer Medicine A or Medicine B, or are they the same?

Indicate your choice here. You can only choose one option:

○ **Medicine A** Live for (10 years – 5 days)

○ **Medicine B** Live for 10 years

○ **Medicine A and Medicine B are the same.**

0% 100%

For peer review only

Repeated section

Remember Medicine A and Medicine B are equally effective and have **some risk of a side effect**.

The only difference between Medicine A and Medicine B is how you take them.

Medicine A	Medicine B
You take a tablet only once in your life.<br>You will be given the tablet on the day you come in to see your GP.<br>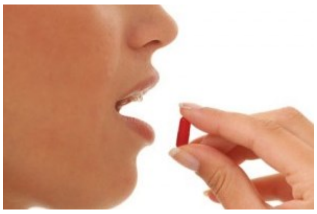	One tablet should be taken once every day, at night with cold water.<br>You need to take this medicine for 10 years.<br>You need to regularly visit a GP to receive a prescription.<br>You need to visit a pharmacy to fill a prescription.<br>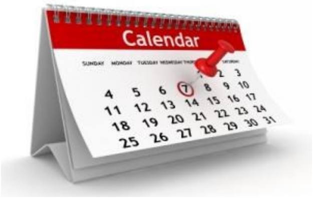<br>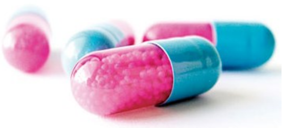

If you take Medicine A, you would live for **3 days less than 10 years** and then die.

If you take Medicine B, you would live for **10 years** and then die.

Would you prefer Medicine A or Medicine B, or are they the same?

Indicate your choice here. You can only choose one option:

◯ **Medicine A** Live for (10 years – 3 days)

◯ **Medicine B** Live for 10 years

◯ **Medicine A and Medicine B are the same.**

For peer review only

Repeated section

Remember Medicine A and Medicine B are equally effective and have **some risk of a side effect**.

The only difference between Medicine A and Medicine B is how you take them.

Medicine A	Medicine B
You take a tablet only once in your life.<br>You will be given the tablet on the day you come in to see your GP.<br>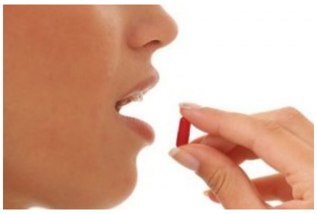	One tablet should be taken once every day, at night with cold water.<br>You need to take this medicine for 10 years.<br>You need to regularly visit a GP to receive a prescription.<br>You need to visit a pharmacy to fill a prescription.<br>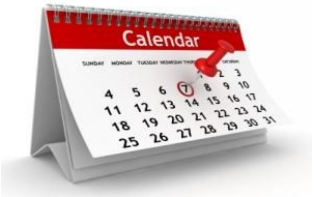<br>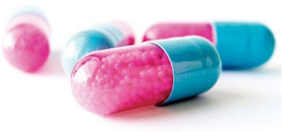

If you take Medicine A, you would live for **1 day less than 10 years** and then die.

If you take Medicine B, you would live for **10 years** and then die.

Would you prefer Medicine A or Medicine B, or are they the same?

Indicate your choice here. You can only choose one option:

○ **Medicine A** Live for (10 years – 1 day)

○ **Medicine B** Live for 10 years

○ **Medicine A and Medicine B are the same.**

0% 100%

For peer review only

Question 2a.

You prefer living for 5 years on Medicine A, to living for 10 years on Medicine B.

This means **living fewer than 5 years on Medicine A** can be the same as living for 10 years on Medicine B.

How many years on Medicine A do you think would be the same as living for 10 years on Medicine B?

(Please give a number in years)

0% 100%

peer review only

Part 2

Question 3

Now, we will assume that there is some risk of a severe side effect from either medicine.

As before,

Medicine A is a pill that you only need to take once.

Medicine B is a pill that you need to take every day for ten years.

Medicine A and Medicine B are equally effective in reducing your risk of developing heart disease.

Medicine A and Medicine B will reduce your risk of having a heart attack or a stroke within the next 10 years from **10%** to **6%**.

In other words, in a crowd of 1,000 people in the UK, **100** are likely to have a heart attack or a stroke if they do not take the medicine. This is reduced to **60** people out of 1,000 who do take the medicine. This information is also illustrated in this picture.

Risk of heart disease

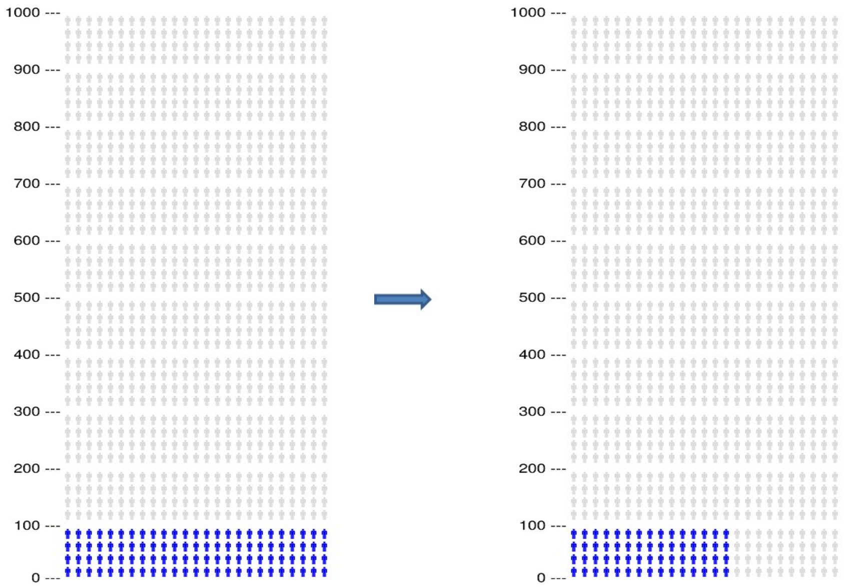

10%
No Treatment

6%
Taking the Medicine

(The figures shaded blue represent people who will have a heart attack or a stroke in the next 10 years)

In this scenario, there is some risk of a severe side effect from Medicine A or Medicine B.

Among every 1,000 people taking Medicine A or Medicine B, **3** people are likely to have a severe side effect such as severe destruction of skeletal muscle (rhabdomyolysis).

Risk of a severe side effect

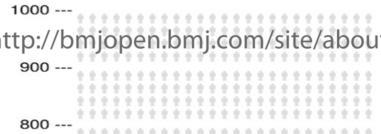

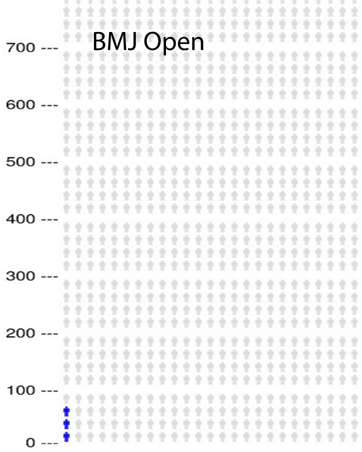

0.3% of people will have a severe side effect.

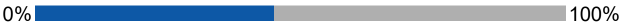

For peer review only

Repeated section

Remember Medicine A and Medicine B are equally effective and have **some risk of a side effect**.

The only difference between Medicine A and Medicine B is how you take them.

Medicine A	Medicine B
You take a tablet only once in your life.<br>You will be given the tablet on the day you come in to see your GP.<br>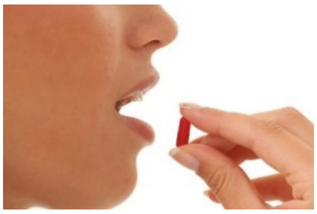	One tablet should be taken once every day, at night with cold water.<br>You need to take this medicine for 10 years.<br>You need to regularly visit a GP to receive a prescription.<br>You need to visit a pharmacy to fill a prescription.<br>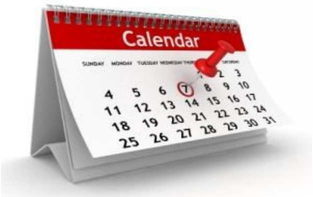<br>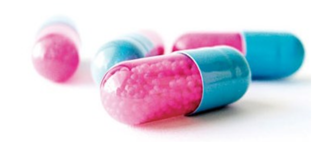

If you take Medicine A, you would live for **5 years** and then die.

If you take Medicine B, you would live for **10 years** and then die.

Would you prefer Medicine A or Medicine B, or are they the same?

Indicate your choice here. You can only choose one option:

◯ **Medicine A** Live for 5 years

◯ **Medicine B** Live for 10 years

◯ **Medicine A and Medicine B are the same.**

0% 100%

For peer review only

Repeated section

Remember Medicine A and Medicine B are equally effective and have **some risk of a side effect**.

The only difference between Medicine A and Medicine B is how you take them.

**Medicine A**	**Medicine B**
You take a tablet only once in your life.<br>You will be given the tablet on the day you come in to see your GP.<br>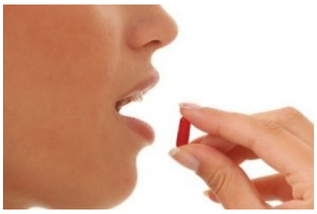	One tablet should be taken once every day, at night with cold water.<br>You need to take this medicine for 10 years.<br>You need to regularly visit a GP to receive a prescription.<br>You need to visit a pharmacy to fill a prescription.<br>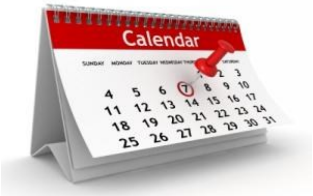<br><br>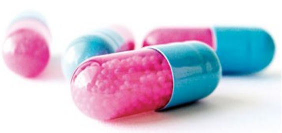

If you take Medicine A, you would live for **5.5 years** (5 years and 6 months) and then die.

If you take Medicine B, you would live for **10 years** and then die.

Would you prefer Medicine A or Medicine B, or are they the same?

Indicate your choice here. You can only choose one option:

○ **Medicine A** Live for 5.5 years

○ **Medicine B** Live for 10 years

○ **Medicine A and Medicine B are the same.**

For peer review only

Repeated section

Remember Medicine A and Medicine B are equally effective and have **some risk of a side effect**.

The only difference between Medicine A and Medicine B is how you take them.

Medicine A	Medicine B
You take a tablet only once in your life.<br>You will be given the tablet on the day you come in to see your GP.<br>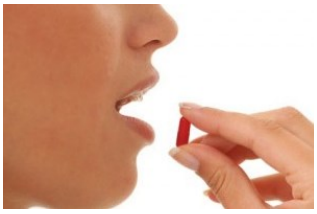	One tablet should be taken once every day, at night with cold water.<br>You need to take this medicine for 10 years.<br>You need to regularly visit a GP to receive a prescription.<br>You need to visit a pharmacy to fill a prescription.<br>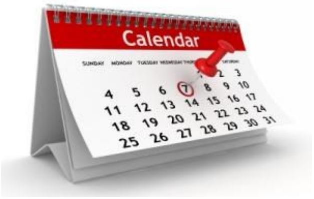<br><br>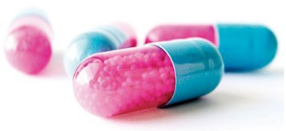

If you take Medicine A, you would live for **6 years** and then die.

If you take Medicine B, you would live for **10 years** and then die.

Would you prefer Medicine A or Medicine B, or are they the same?

Indicate your choice here. You can only choose one option:

◯ **Medicine A** Live for 6 years

◯ **Medicine B** Live for 10 years

◯ **Medicine A and Medicine B are the same.**

For peer review only

Repeated section

Remember Medicine A and Medicine B are equally effective and have **some risk of a side effect**.

The only difference between Medicine A and Medicine B is how you take them.

Medicine A	Medicine B
You take a tablet only once in your life.<br>You will be given the tablet on the day you come in to see your GP.<br>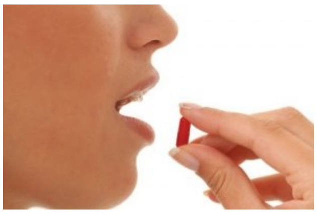	One tablet should be taken once every day, at night with cold water.<br>You need to take this medicine for 10 years.<br>You need to regularly visit a GP to receive a prescription.<br>You need to visit a pharmacy to fill a prescription.<br>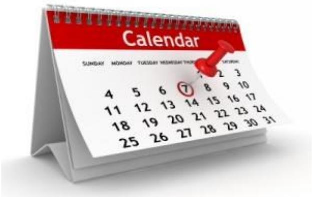<br>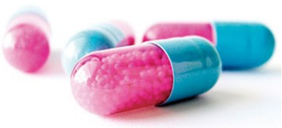

If you take Medicine A, you would live for **6.25 years (6 years and 3 months)** and then die.

If you take Medicine B, you would live for **10 years** and then die.

Would you prefer Medicine A or Medicine B, or are they the same?

Indicate your choice here. You can only choose one option:

◯ **Medicine A** Live for 6 years and 3 months

◯ **Medicine B** Live for 10 years

◯ **Medicine A and Medicine B are the same.**

For peer review only

Repeated section

Remember Medicine A and Medicine B are equally effective and have **some risk of a side effect**.

The only difference between Medicine A and Medicine B is how you take them.

Medicine A	Medicine B
You take a tablet only once in your life.<br>You will be given the tablet on the day you come in to see your GP.<br>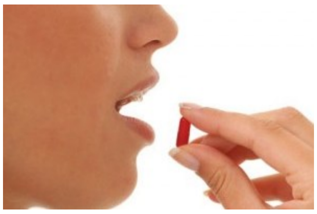	One tablet should be taken once every day, at night with cold water.<br>You need to take this medicine for 10 years.<br>You need to regularly visit a GP to receive a prescription.<br>You need to visit a pharmacy to fill a prescription.<br>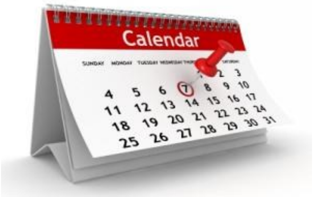<br>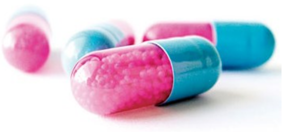

If you take Medicine A, you would live for **6.5 years** (6 years and 6 months) and then die.

If you take Medicine B, you would live for **10 years** and then die.

Would you prefer Medicine A or Medicine B, or are they the same?

Indicate your choice here. You can only choose one option:

○ **Medicine A** Live for 6.5 years

○ **Medicine B** Live for 10 years

○ **Medicine A and Medicine B are the same.**

For peer review only

Repeated section

Remember Medicine A and Medicine B are equally effective and have **some risk of a side effect**.

The only difference between Medicine A and Medicine B is how you take them.

**Medicine A**	**Medicine B**
You take a tablet only once in your life.<br>You will be given the tablet on the day you come in to see your GP.<br>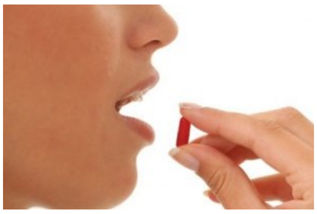	One tablet should be taken once every day, at night with cold water.<br>You need to take this medicine for 10 years.<br>You need to regularly visit a GP to receive a prescription.<br>You need to visit a pharmacy to fill a prescription.<br>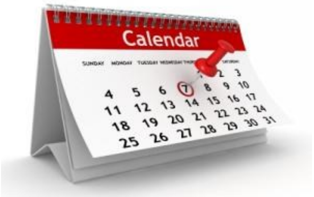<br>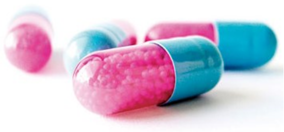

If you take Medicine A, you would live for **7 years** and then die.

If you take Medicine B, you would live for **10 years** and then die.

Would you prefer Medicine A or Medicine B, or are they the same?

Indicate your choice here. You can only choose one option:

◯ **Medicine A**
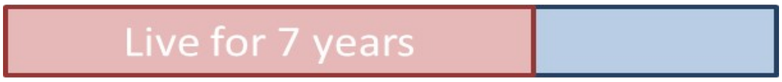

◯ **Medicine B**
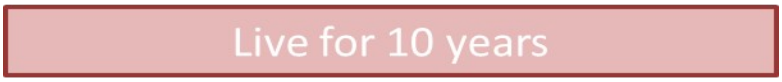

◯ **Medicine A and Medicine B are the same.**

0% 100%

For peer review only

Repeated section

Remember Medicine A and Medicine B are equally effective and have **some risk of a side effect**.

The only difference between Medicine A and Medicine B is how you take them.

Medicine A	Medicine B
You take a tablet only once in your life.<br>You will be given the tablet on the day you come in to see your GP.<br>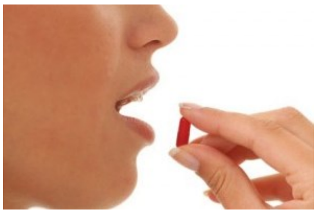	One tablet should be taken once every day, at night with cold water.<br>You need to take this medicine for 10 years.<br>You need to regularly visit a GP to receive a prescription.<br>You need to visit a pharmacy to fill a prescription.<br>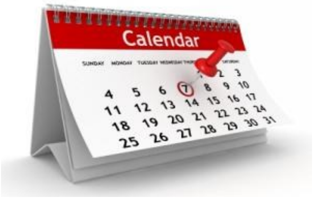<br>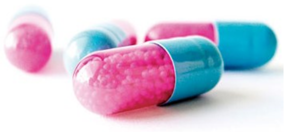

If you take Medicine A, you would live for **7.5 years** (7 years and 6 months) and then die.

If you take Medicine B, you would live for **10 years** and then die.

Would you prefer Medicine A or Medicine B, or are they the same?

Indicate your choice here. You can only choose one option:

◯ **Medicine A** Live for 7.5 years

◯ **Medicine B** Live for 10 years

◯ **Medicine A and Medicine B are the same.**

0% 100%

For peer review only

Repeated section

Remember Medicine A and Medicine B are equally effective and have **some risk of a side effect**.

The only difference between Medicine A and Medicine B is how you take them.

Medicine A	Medicine B
You take a tablet only once in your life.<br>You will be given the tablet on the day you come in to see your GP.<br>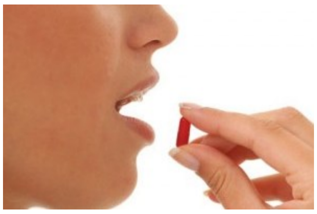	One tablet should be taken once every day, at night with cold water.<br>You need to take this medicine for 10 years.<br>You need to regularly visit a GP to receive a prescription.<br>You need to visit a pharmacy to fill a prescription.<br>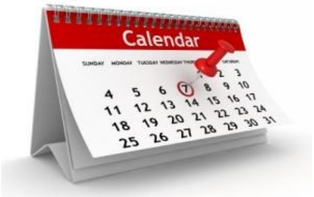<br>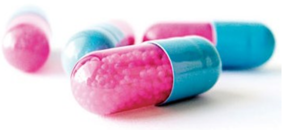

If you take Medicine A, you would live for **7.75 years (7 years and 9 months)** and then die.

If you take Medicine B, you would live for **10 years** and then die.

Would you prefer Medicine A or Medicine B, or are they the same?

Indicate your choice here. You can only choose one option:

◯ **Medicine A** Live for 7 years and 9 months

◯ **Medicine B** Live for 10 years

◯ **Medicine A and Medicine B are the same.**

For peer review only

Repeated section

Remember Medicine A and Medicine B are equally effective and have **some risk of a side effect**.

The only difference between Medicine A and Medicine B is how you take them.

**Medicine A**	**Medicine B**
You take a tablet only once in your life. You will be given the tablet on the day you come in to see your GP. 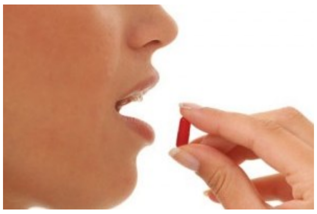	One tablet should be taken once every day, at night with cold water. You need to take this medicine for 10 years. You need to regularly visit a GP to receive a prescription. You need to visit a pharmacy to fill a prescription. 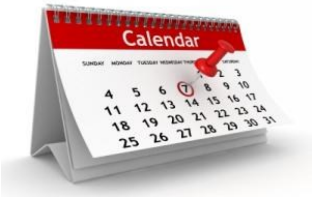 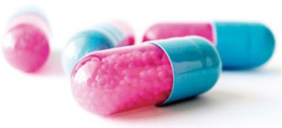

If you take Medicine A, you would live for **8 years** and then die.

If you take Medicine B, you would live for **10 years** and then die.

Would you prefer Medicine A or Medicine B, or are they the same?

Indicate your choice here. You can only choose one option:

◯ **Medicine A** Live for 8 years

◯ **Medicine B** Live for 10 years

◯ **Medicine A and Medicine B are the same.**

For peer review only

Repeated section

Remember Medicine A and Medicine B are equally effective and have **some risk of a side effect**.

The only difference between Medicine A and Medicine B is how you take them.

Medicine A	Medicine B
You take a tablet only once in your life.<br>You will be given the tablet on the day you come in to see your GP.<br>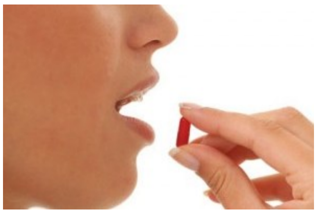	One tablet should be taken once every day, at night with cold water.<br>You need to take this medicine for 10 years.<br>You need to regularly visit a GP to receive a prescription.<br>You need to visit a pharmacy to fill a prescription.<br>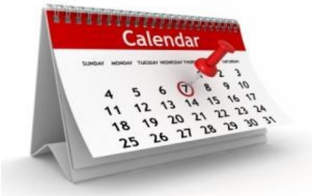<br>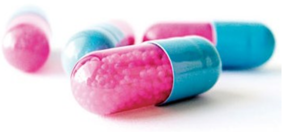

If you take Medicine A, you would live for **8.25 years (8 years and 3 months)** and then die.

If you take Medicine B, you would live for **10 years** and then die.

Would you prefer Medicine A or Medicine B, or are they the same?

Indicate your choice here. You can only choose one option:

◯ **Medicine A** Live for 8 years and 3 months

◯ **Medicine B** Live for 10 years

◯ **Medicine A and Medicine B are the same.**

0% 100%

For peer review only

Repeated section

Remember Medicine A and Medicine B are equally effective and have **some risk of a side effect**.

The only difference between Medicine A and Medicine B is how you take them.

**Medicine A**	**Medicine B**
You take a tablet only once in your life. You will be given the tablet on the day you come in to see your GP. 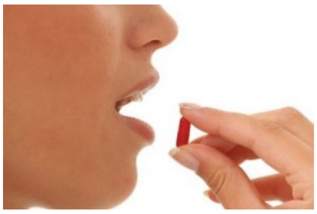	One tablet should be taken once every day, at night with cold water. You need to take this medicine for 10 years. You need to regularly visit a GP to receive a prescription. You need to visit a pharmacy to fill a prescription. 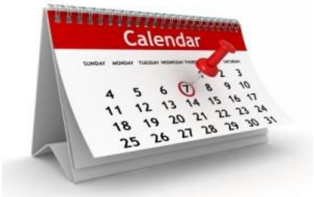 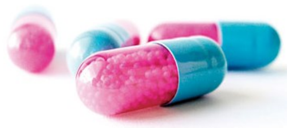

If you take Medicine A, you would live for **8.5 years** (8 years and 6 months) and then die.

If you take Medicine B, you would live for **10 years** and then die.

Would you prefer Medicine A or Medicine B, or are they the same?

Indicate your choice here. You can only choose one option:

○ **Medicine A** 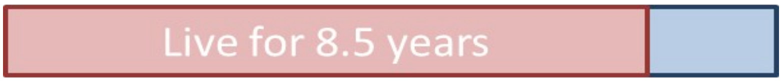

○ **Medicine B** 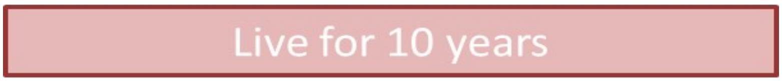

○ **Medicine A and Medicine B are the same.**

For peer review only

Repeated section

Remember Medicine A and Medicine B are equally effective and have **some risk of a side effect**.

The only difference between Medicine A and Medicine B is how you take them.

**Medicine A**	**Medicine B**
You take a tablet only once in your life.<br>You will be given the tablet on the day you come in to see your GP.<br>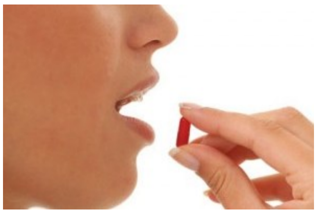	One tablet should be taken once every day, at night with cold water.<br>You need to take this medicine for 10 years.<br>You need to regularly visit a GP to receive a prescription.<br>You need to visit a pharmacy to fill a prescription.<br>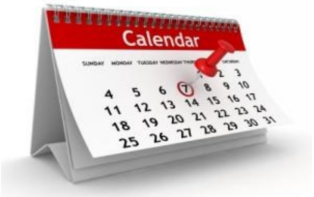<br>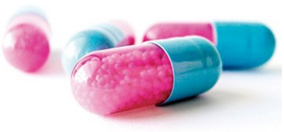

If you take Medicine A, you would live for **8.75 years (8 years and 9 months)** and then die.

If you take Medicine B, you would live for **10 years** and then die.

Would you prefer Medicine A or Medicine B, or are they the same?

Indicate your choice here. You can only choose one option:

○ **Medicine A** Live for 8 years and 9 months

○ **Medicine B** Live for 10 years

○ **Medicine A and Medicine B are the same.**

0% 100%

For peer review only

Repeated section

Remember Medicine A and Medicine B are equally effective and have **some risk of a side effect**.

The only difference between Medicine A and Medicine B is how you take them.

Medicine A	Medicine B
You take a tablet only once in your life.<br>You will be given the tablet on the day you come in to see your GP.<br>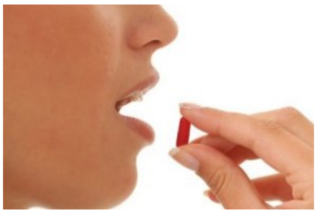	One tablet should be taken once every day, at night with cold water.<br>You need to take this medicine for 10 years.<br>You need to regularly visit a GP to receive a prescription.<br>You need to visit a pharmacy to fill a prescription.<br>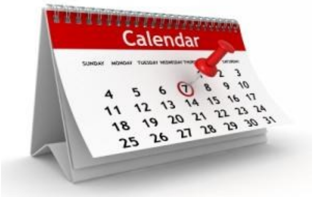<br>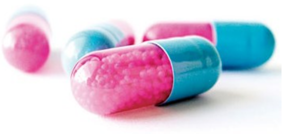

If you take Medicine A, you would live for **9 years** and then die.

If you take Medicine B, you would live for **10 years** and then die.

Would you prefer Medicine A or Medicine B, or are they the same?

Indicate your choice here. You can only choose one option:

○ **Medicine A** Live for 9 years

○ **Medicine B** Live for 10 years

○ **Medicine A and Medicine B are the same.**

For peer review only

Repeated section

Remember Medicine A and Medicine B are equally effective and have **some risk of a side effect**.

The only difference between Medicine A and Medicine B is how you take them.

Medicine A	Medicine B
You take a tablet only once in your life.<br>You will be given the tablet on the day you come in to see your GP.<br>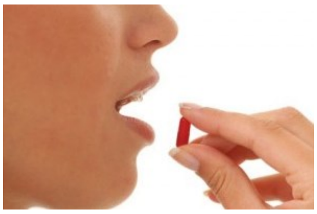	One tablet should be taken once every day, at night with cold water.<br>You need to take this medicine for 10 years.<br>You need to regularly visit a GP to receive a prescription.<br>You need to visit a pharmacy to fill a prescription.<br>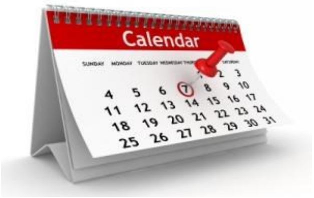<br><br>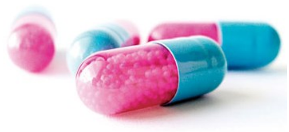

If you take Medicine A, you would live for **9 years and 1 month** and then die.

If you take Medicine B, you would live for **10 years** and then die.

Would you prefer Medicine A or Medicine B, or are they the same?

Indicate your choice here. You can only choose one option:

○ **Medicine A** Live for 9 years 1 month

○ **Medicine B** Live for 10 years

○ **Medicine A and Medicine B are the same.**

For peer review only

Repeated section

Remember Medicine A and Medicine B are equally effective and have **some risk of a side effect**.

The only difference between Medicine A and Medicine B is how you take them.

Medicine A	Medicine B
You take a tablet only once in your life.<br>You will be given the tablet on the day you come in to see your GP.<br>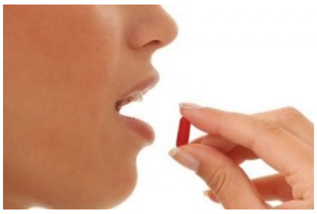	One tablet should be taken once every day, at night with cold water.<br>You need to take this medicine for 10 years.<br>You need to regularly visit a GP to receive a prescription.<br>You need to visit a pharmacy to fill a prescription.<br>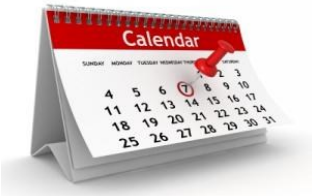<br>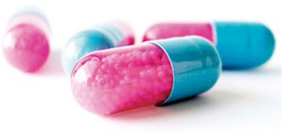

If you take Medicine A, you would live for **9.25 years (9 years and 3 months)** and then die.

If you take Medicine B, you would live for **10 years** and then die.

Would you prefer Medicine A or Medicine B, or are they the same?

Indicate your choice here. You can only choose one option:

◯ **Medicine A** Live for 9 years and 3 months

◯ **Medicine B** Live for 10 years

◯ **Medicine A and Medicine B are the same.**

0% 100%

For peer review only

Repeated section

Remember Medicine A and Medicine B are equally effective and have **some risk of a side effect**.

The only difference between Medicine A and Medicine B is how you take them.

Medicine A	Medicine B
You take a tablet only once in your life.<br>You will be given the tablet on the day you come in to see your GP.<br>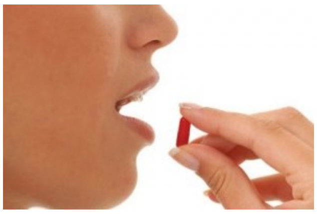	One tablet should be taken once every day, at night with cold water.<br>You need to take this medicine for 10 years.<br>You need to regularly visit a GP to receive a prescription.<br>You need to visit a pharmacy to fill a prescription.<br>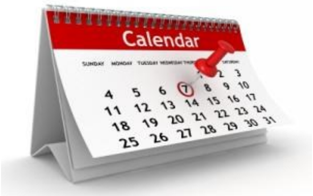<br>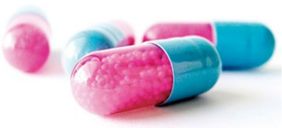

If you take Medicine A, you would live for **9 years and 4 months** and then die.

If you take Medicine B, you would live for **10 years** and then die.

Would you prefer Medicine A or Medicine B, or are they the same?

Indicate your choice here. You can only choose one option:

◯ **Medicine A** Live for 9 years 4 months

◯ **Medicine B** Live for 10 years

◯ **Medicine A and Medicine B are the same.**

0% 100%

For peer review only

Repeated section

Remember Medicine A and Medicine B are equally effective and have **some risk of a side effect**.

The only difference between Medicine A and Medicine B is how you take them.

Medicine A	Medicine B
You take a tablet only once in your life.<br>You will be given the tablet on the day you come in to see your GP.<br>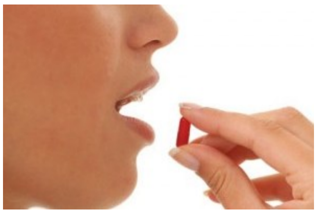	One tablet should be taken once every day, at night with cold water.<br>You need to take this medicine for 10 years.<br>You need to regularly visit a GP to receive a prescription.<br>You need to visit a pharmacy to fill a prescription.<br>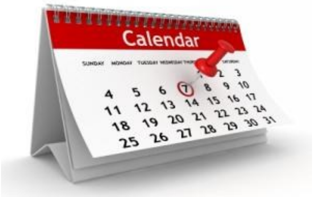 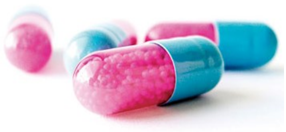

If you take Medicine A, you would live for **9 years and 5 months** and then die.

If you take Medicine B, you would live for **10 years** and then die.

Would you prefer Medicine A or Medicine B, or are they the same?

Indicate your choice here. You can only choose one option:

◯ **Medicine A** Live for 9 years 5 months

◯ **Medicine B** Live for 10 years

◯ **Medicine A and Medicine B are the same.**

For peer review only

Repeated section

Remember Medicine A and Medicine B are equally effective and have **some risk of a side effect**.

The only difference between Medicine A and Medicine B is how you take them.

<table>
<tr><th>Medicine A</th><th>Medicine B</th></tr>
<tr><td>You take a tablet only once in your life.
You will be given the tablet on the day you come in to see your GP.
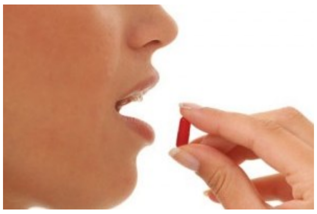</td><td>One tablet should be taken once every day, at night with cold water.
You need to take this medicine for 10 years.
You need to regularly visit a GP to receive a prescription.
You need to visit a pharmacy to fill a prescription.
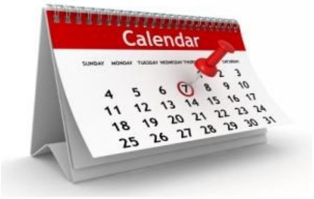

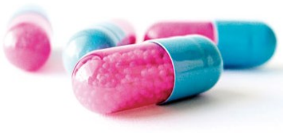</td></tr>
</table>

If you take Medicine A, you would live for **9.5 years** (9 years and 6 months) and then die.

If you take Medicine B, you would live for **10 years** and then die.

Would you prefer Medicine A or Medicine B, or are they the same?

Indicate your choice here. You can only choose one option:

◯ **Medicine A** 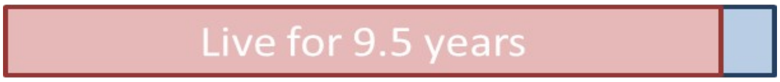

◯ **Medicine B** 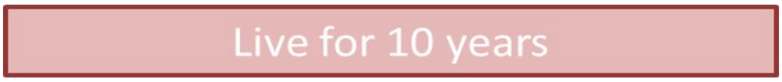

◯ **Medicine A and Medicine B are the same.**

0% 100%

For peer review only

Repeated section

Remember Medicine A and Medicine B are equally effective and have **some risk of a side effect**.

The only difference between Medicine A and Medicine B is how you take them.

Medicine A	Medicine B
You take a tablet only once in your life.<br>You will be given the tablet on the day you come in to see your GP.<br>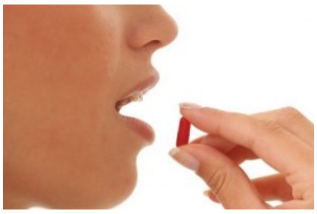	One tablet should be taken once every day, at night with cold water.<br>You need to take this medicine for 10 years.<br>You need to regularly visit a GP to receive a prescription.<br>You need to visit a pharmacy to fill a prescription.<br>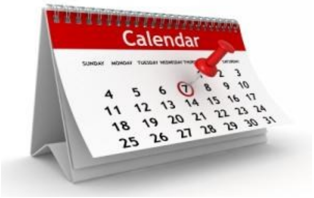<br>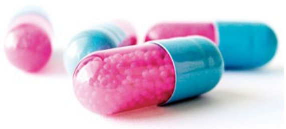

If you take Medicine A, you would live for **5 months less than 10 years** and then die.

If you take Medicine B, you would live for **10 years** and then die.

Would you prefer Medicine A or Medicine B, or are they the same?

Indicate your choice here. You can only choose one option:

○ **Medicine A** Live for (10 years – 5 months)

○ **Medicine B** Live for 10 years

○ **Medicine A and Medicine B are the same.**

For peer review only

Repeated section

Remember Medicine A and Medicine B are equally effective and have **some risk of a side effect**.

The only difference between Medicine A and Medicine B is how you take them.

**Medicine A**	**Medicine B**
You take a tablet only once in your life.<br>You will be given the tablet on the day you come in to see your GP.<br>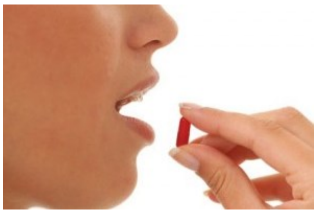	One tablet should be taken once every day, at night with cold water.<br>You need to take this medicine for 10 years.<br>You need to regularly visit a GP to receive a prescription.<br>You need to visit a pharmacy to fill a prescription.<br>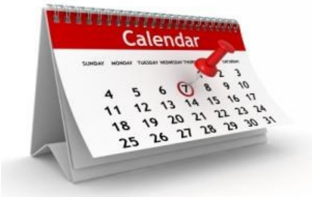<br>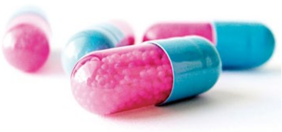

If you take Medicine A, you would live for **4 months less than 10 years** and then die.

If you take Medicine B, you would live for **10 years** and then die.

Would you prefer Medicine A or Medicine B, or are they the same?

Indicate your choice here. You can only choose one option:

○ **Medicine A** Live for (10 years – 4 months)

○ **Medicine B** Live for 10 years

○ **Medicine A and Medicine B are the same.**

0% 100%

For peer review only

Repeated section

Remember Medicine A and Medicine B are equally effective and have **some risk of a side effect**.

The only difference between Medicine A and Medicine B is how you take them.

Medicine A	Medicine B
You take a tablet only once in your life.<br>You will be given the tablet on the day you come in to see your GP.<br>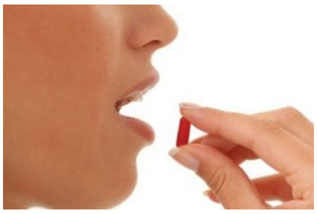	One tablet should be taken once every day, at night with cold water.<br>You need to take this medicine for 10 years.<br>You need to regularly visit a GP to receive a prescription.<br>You need to visit a pharmacy to fill a prescription.<br>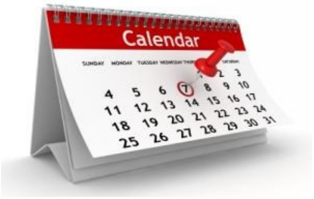<br>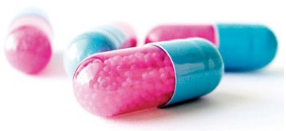

If you take Medicine A, you would live for **3 months less than 10 years** and then die.

If you take Medicine B, you would live for **10 years** and then die.

Would you prefer Medicine A or Medicine B, or are they the same?

Indicate your choice here. You can only choose one option:

○ **Medicine A** Live for (10 years – 3 months)

○ **Medicine B** Live for 10 years

○ **Medicine A and Medicine B are the same.**

For peer review only

Repeated section

Remember Medicine A and Medicine B are equally effective and have **some risk of a side effect**.

The only difference between Medicine A and Medicine B is how you take them.

**Medicine A**	**Medicine B**
You take a tablet only once in your life.<br>You will be given the tablet on the day you come in to see your GP.<br>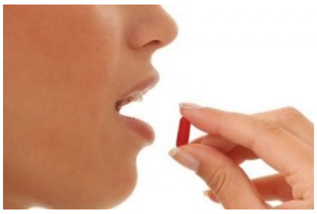	One tablet should be taken once every day, at night with cold water.<br>You need to take this medicine for 10 years.<br>You need to regularly visit a GP to receive a prescription.<br>You need to visit a pharmacy to fill a prescription.<br>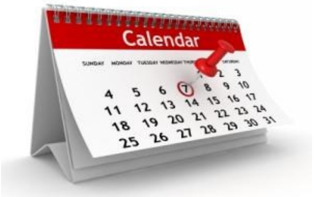<br>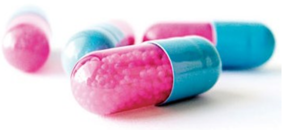

If you take Medicine A, you would live for **2 months less than 10 years** and then die.

If you take Medicine B, you would live for **10 years** and then die.

Would you prefer Medicine A or Medicine B, or are they the same?

Indicate your choice here. You can only choose one option:

◯ **Medicine A** Live for (10 years – 2 months)

◯ **Medicine B** Live for 10 years

◯ **Medicine A and Medicine B are the same.**

For peer review only

Repeated section

Remember Medicine A and Medicine B are equally effective and have **some risk of a side effect**.

The only difference between Medicine A and Medicine B is how you take them.

**Medicine A**	**Medicine B**
You take a tablet only once in your life.<br>You will be given the tablet on the day you come in to see your GP.<br>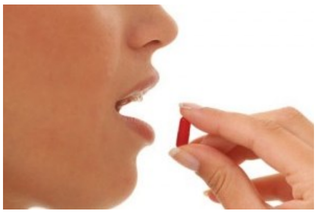	One tablet should be taken once every day, at night with cold water.<br>You need to take this medicine for 10 years.<br>You need to regularly visit a GP to receive a prescription.<br>You need to visit a pharmacy to fill a prescription.<br>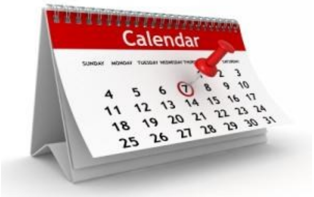<br>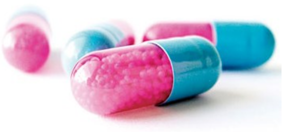

If you take Medicine A, you would live for **1 month less than 10 years** and then die.

If you take Medicine B, you would live for **10 years** and then die.

Would you prefer Medicine A or Medicine B, or are they the same?

Indicate your choice here. You can only choose one option:

○ **Medicine A** Live for (10 years – 1 month)

○ **Medicine B** Live for 10 years

○ **Medicine A and Medicine B are the same.**

For peer review only

Repeated section

Remember Medicine A and Medicine B are equally effective and have **some risk of a side effect**.

The only difference between Medicine A and Medicine B is how you take them.

**Medicine A**	**Medicine B**
You take a tablet only once in your life.<br>You will be given the tablet on the day you come in to see your GP.<br>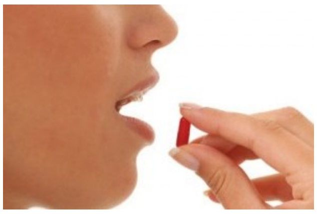	One tablet should be taken once every day, at night with cold water.<br>You need to take this medicine for 10 years.<br>You need to regularly visit a GP to receive a prescription.<br>You need to visit a pharmacy to fill a prescription.<br>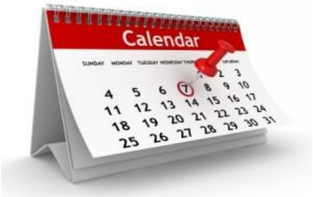<br>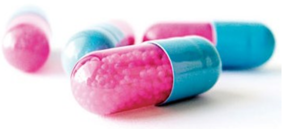

If you take Medicine A, you would live for **3 weeks less than 10 years** and then die.

If you take Medicine B, you would live for **10 years** and then die.

Would you prefer Medicine A or Medicine B, or are they the same?

Indicate your choice here. You can only choose one option:

○ **Medicine A** Live for (10 years – 3 weeks)

○ **Medicine B** Live for 10 years

○ **Medicine A and Medicine B are the same.**

For peer review only

Repeated section

Remember Medicine A and Medicine B are equally effective and have **some risk of a side effect**.

The only difference between Medicine A and Medicine B is how you take them.

**Medicine A**	**Medicine B**
You take a tablet only once in your life.<br>You will be given the tablet on the day you come in to see your GP.<br>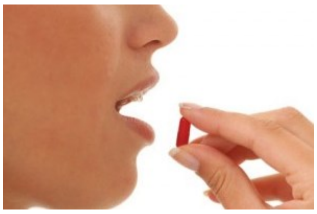	One tablet should be taken once every day, at night with cold water.<br>You need to take this medicine for 10 years.<br>You need to regularly visit a GP to receive a prescription.<br>You need to visit a pharmacy to fill a prescription.<br>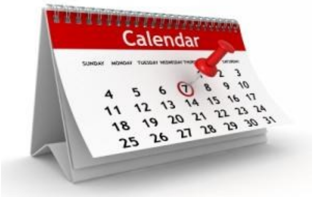<br>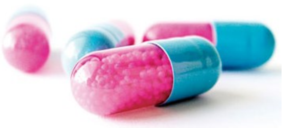

If you take Medicine A, you would live for **2 weeks less than 10 years** and then die.

If you take Medicine B, you would live for **10 years** and then die.

Would you prefer Medicine A or Medicine B, or are they the same?

Indicate your choice here. You can only choose one option:

○ **Medicine A** Live for (10 years – 2 weeks)

○ **Medicine B** Live for 10 years

○ **Medicine A and Medicine B are the same.**

For peer review only

Repeated section

Remember Medicine A and Medicine B are equally effective and have **some risk of a side effect**.

The only difference between Medicine A and Medicine B is how you take them.

Medicine A	Medicine B
You take a tablet only once in your life.<br>You will be given the tablet on the day you come in to see your GP.<br>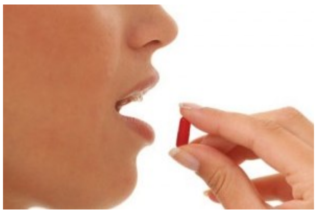	One tablet should be taken once every day, at night with cold water.<br>You need to take this medicine for 10 years.<br>You need to regularly visit a GP to receive a prescription.<br>You need to visit a pharmacy to fill a prescription.<br>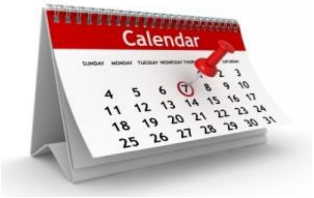<br>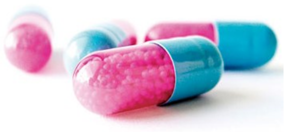

If you take Medicine A, you would live for **10 days less than 10 years** and then die.

If you take Medicine B, you would live for **10 years** and then die.

Would you prefer Medicine A or Medicine B, or are they the same?

Indicate your choice here. You can only choose one option:

○ **Medicine A** Live for (10 years – 10 days)

○ **Medicine B** Live for 10 years

○ **Medicine A and Medicine B are the same.**

0% 100%

For peer review only

Repeated section

Remember Medicine A and Medicine B are equally effective and have **some risk of a side effect**.

The only difference between Medicine A and Medicine B is how you take them.

Medicine A	Medicine B
You take a tablet only once in your life.<br>You will be given the tablet on the day you come in to see your GP.<br>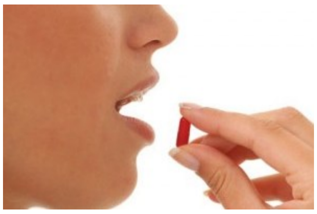	One tablet should be taken once every day, at night with cold water.<br>You need to take this medicine for 10 years.<br>You need to regularly visit a GP to receive a prescription.<br>You need to visit a pharmacy to fill a prescription.<br>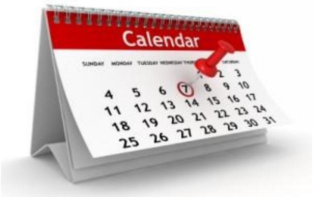<br>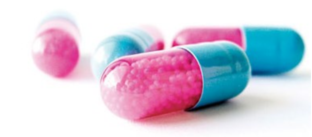

If you take Medicine A, you would live for **1 week less than 10 years** and then die.

If you take Medicine B, you would live for **10 years** and then die.

Would you prefer Medicine A or Medicine B, or are they the same?

Indicate your choice here. You can only choose one option:

○ **Medicine A** Live for (10 years – 1 week)

○ **Medicine B** Live for 10 years

○ **Medicine A and Medicine B are the same.**

For peer review only

Repeated section

Remember Medicine A and Medicine B are equally effective and have **some risk of a side effect**.

The only difference between Medicine A and Medicine B is how you take them.

**Medicine A**	**Medicine B**
You take a tablet only once in your life.<br>You will be given the tablet on the day you come in to see your GP.<br>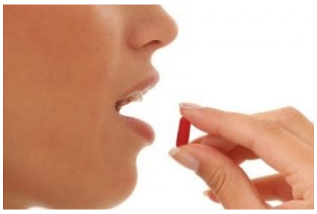	One tablet should be taken once every day, at night with cold water.<br>You need to take this medicine for 10 years.<br>You need to regularly visit a GP to receive a prescription.<br>You need to visit a pharmacy to fill a prescription.<br>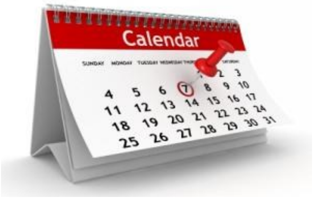<br>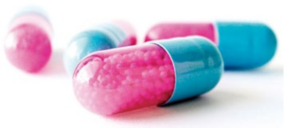

If you take Medicine A, you would live for **5 days less than 10 years** and then die.

If you take Medicine B, you would live for **10 years** and then die.

Would you prefer Medicine A or Medicine B, or are they the same?

Indicate your choice here. You can only choose one option:

○ **Medicine A** Live for (10 years – 5 days)

○ **Medicine B** Live for 10 years

○ **Medicine A and Medicine B are the same.**

For peer review only

Repeated section

Remember Medicine A and Medicine B are equally effective and have **some risk of a side effect**.

The only difference between Medicine A and Medicine B is how you take them.

**Medicine A**	**Medicine B**
You take a tablet only once in your life.<br>You will be given the tablet on the day you come in to see your GP.<br>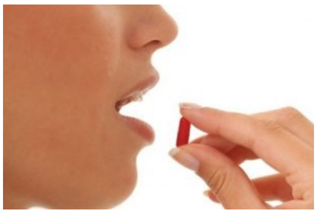	One tablet should be taken once every day, at night with cold water.<br>You need to take this medicine for 10 years.<br>You need to regularly visit a GP to receive a prescription.<br>You need to visit a pharmacy to fill a prescription.<br>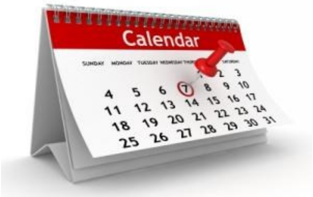<br>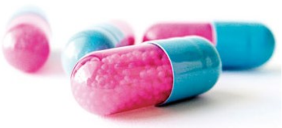

If you take Medicine A, you would live for **3 days less than 10 years** and then die.

If you take Medicine B, you would live for **10 years** and then die.

Would you prefer Medicine A or Medicine B, or are they the same?

Indicate your choice here. You can only choose one option:

○ **Medicine A** Live for (10 years – 3 days)

○ **Medicine B** Live for 10 years

○ **Medicine A and Medicine B are the same.**

For peer review only

Repeated section

Remember Medicine A and Medicine B are equally effective and have **some risk of a side effect**.

The only difference between Medicine A and Medicine B is how you take them.

Medicine A	Medicine B
You take a tablet only once in your life.<br>You will be given the tablet on the day you come in to see your GP.<br>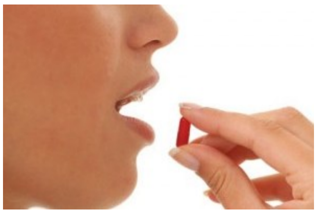	One tablet should be taken once every day, at night with cold water.<br>You need to take this medicine for 10 years.<br>You need to regularly visit a GP to receive a prescription.<br>You need to visit a pharmacy to fill a prescription.<br>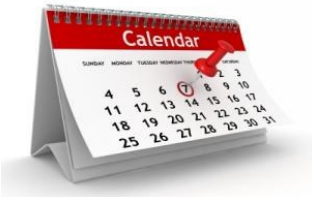<br>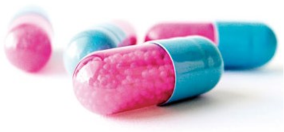

If you take Medicine A, you would live for **1 day less than 10 years** and then die.

If you take Medicine B, you would live for **10 years** and then die.

Would you prefer Medicine A or Medicine B, or are they the same?

Indicate your choice here. You can only choose one option:

○ **Medicine A** Live for (10 years – 1 day)

○ **Medicine B** Live for 10 years

○ **Medicine A and Medicine B are the same.**

For peer review only

Question 3a.

You prefer living for 5 years on Medicine A, to living for 10 years on Medicine B.

This means **living fewer than 5 years on Medicine A** can be the same as living for 10 years on Medicine B.

How many years on Medicine A do you think would be the same as living for 10 years on Medicine B?

(Please give a number in years)

0% 100%

peer review only

Part 2

Question 4

Now, Medicine A and Medicine B will give you less benefit than before because you belong to a group of people who may not respond to the treatment as well as other groups of people.

As before,

Medicine A is a pill that you only need to take once.

Medicine B is a pill that you need to take every day for ten years.

Medicine A and Medicine B are equally effective in reducing your risk of developing heart disease.

Medicine A and Medicine B will reduce your risk of having a heart attack or a stroke within the next 10 years from **10%** to **9%**.

In other words, in a crowd of **1,000** people in the UK, **100** are likely to have a heart attack or a stroke if they do not take the medicine. This is reduced to **90** people out of 1,000 who do take the medicine. This information is also illustrated in this picture.

Risk of heart disease

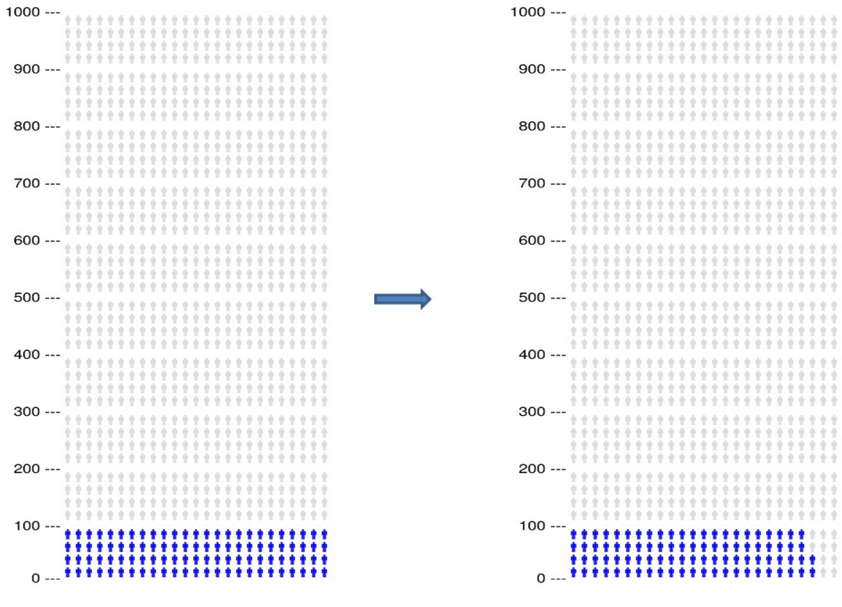

10%
No Treatment

9%
Taking the Medicine

(The figures shaded blue represent people who will have a heart attack or a stroke in the next 10 years)

In this scenario, you will not have any side effects from Medicine A or Medicine B.

Risk of a side effect

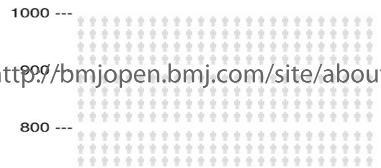

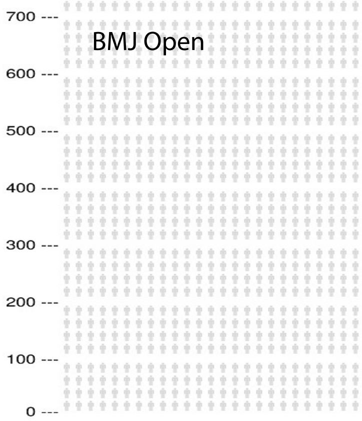

0 %

No one will have a side effect.

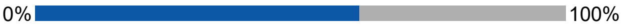

For peer review only

Repeated section

Remember Medicine A and Medicine B are equally effective and have no risks of a side effect.

The only difference between Medicine A and Medicine B is how you take them.

**Medicine A**	**Medicine B**
You take a tablet only once in your life.<br>You will be given the tablet on the day you come in to see your GP.<br>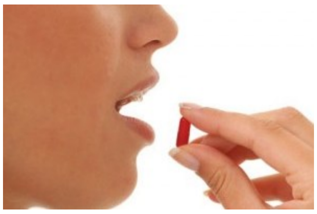	One tablet should be taken once every day, at night with cold water.<br>You need to take this medicine for 10 years.<br>You need to regularly visit a GP to receive a prescription.<br>You need to visit a pharmacy to fill a prescription.<br>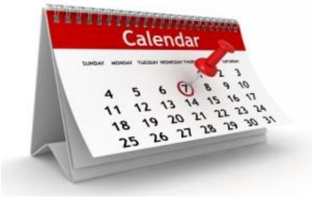<br>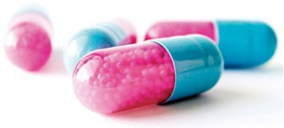

If you take Medicine A, you would live for **5 years** and then die.

If you take Medicine B, you would live for **10 years** and then die.

Would you prefer Medicine A or Medicine B, or are they the same?

Indicate your choice here. You can only choose one option:

○ **Medicine A** Live for 5 years

○ **Medicine B** Live for 10 years

○ **Medicine A and Medicine B are the same.**

For peer review only

Repeated section

Remember Medicine A and Medicine B are equally effective and have no risks of a side effect.

The only difference between Medicine A and Medicine B is how you take them.

Medicine A	Medicine B
You take a tablet only once in your life.<br>You will be given the tablet on the day you come in to see your GP.<br>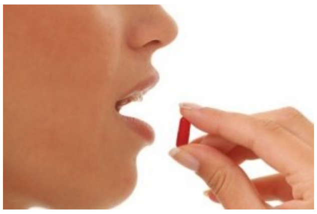	One tablet should be taken once every day, at night with cold water.<br>You need to take this medicine for 10 years.<br>You need to regularly visit a GP to receive a prescription.<br>You need to visit a pharmacy to fill a prescription.<br>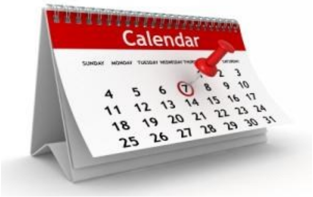<br><br>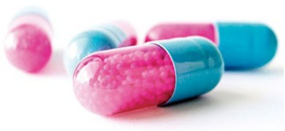

If you take Medicine A, you would live for **5.5 years (5 years and 6 months)** and then die.

If you take Medicine B, you would live for **10 years** and then die.

Would you prefer Medicine A or Medicine B, or are they the same?

Indicate your choice here. You can only choose one option:

◯ **Medicine A** Live for 5.5 years

◯ **Medicine B** Live for 10 years

◯ **Medicine A and Medicine B are the same.**

For peer review only

Repeated section

Remember Medicine A and Medicine B are equally effective and have no risks of a side effect.

The only difference between Medicine A and Medicine B is how you take them.

Medicine A	Medicine B
You take a tablet only once in your life.<br>You will be given the tablet on the day you come in to see your GP.<br>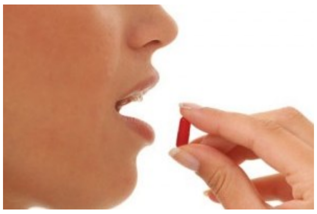	One tablet should be taken once every day, at night with cold water.<br>You need to take this medicine for 10 years.<br>You need to regularly visit a GP to receive a prescription.<br>You need to visit a pharmacy to fill a prescription.<br>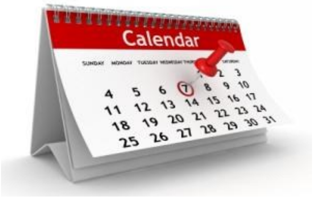<br><br>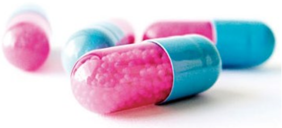

If you take Medicine A, you would live for **6 years** and then die.

If you take Medicine B, you would live for **10 years** and then die.

Would you prefer Medicine A or Medicine B, or are they the same?

Indicate your choice here. You can only choose one option:

○ **Medicine A** Live for 6 years

○ **Medicine B** Live for 10 years

○ **Medicine A and Medicine B are the same.**

For peer review only

Repeated section

Remember Medicine A and Medicine B are equally effective and have no risks of a side effect.

The only difference between Medicine A and Medicine B is how you take them.

Medicine A	Medicine B
You take a tablet only once in your life.<br>You will be given the tablet on the day you come in to see your GP.<br>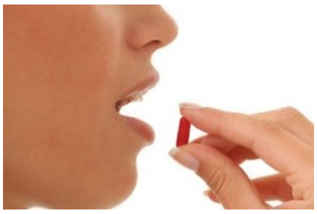	One tablet should be taken once every day, at night with cold water.<br>You need to take this medicine for 10 years.<br>You need to regularly visit a GP to receive a prescription.<br>You need to visit a pharmacy to fill a prescription.<br>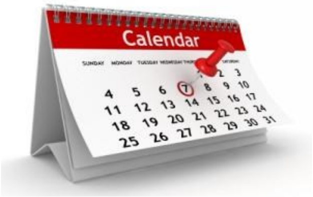<br>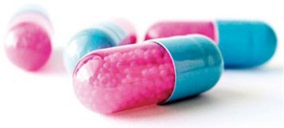

If you take Medicine A, you would live for **6.25 years (6 years and 3 months)** and then die.

If you take Medicine B, you would live for **10 years** and then die.

Would you prefer Medicine A or Medicine B, or are they the same?

Indicate your choice here. You can only choose one option:

○ **Medicine A**

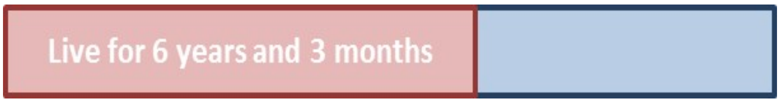

○ **Medicine B**

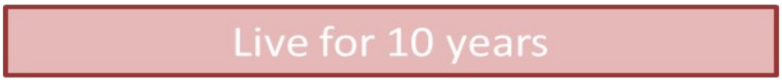

○ **Medicine A and Medicine B are the same.**

For peer review only

Repeated section

Remember Medicine A and Medicine B are equally effective and have no risks of a side effect.

The only difference between Medicine A and Medicine B is how you take them.

Medicine A	Medicine B
You take a tablet only once in your life.<br>You will be given the tablet on the day you come in to see your GP.<br>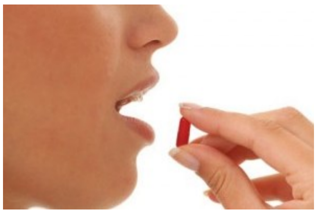	One tablet should be taken once every day, at night with cold water.<br>You need to take this medicine for 10 years.<br>You need to regularly visit a GP to receive a prescription.<br>You need to visit a pharmacy to fill a prescription.<br>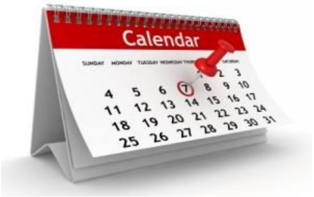<br>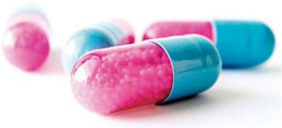

If you take Medicine A, you would live for **6.5 years** (6 years and 6 months) and then die.

If you take Medicine B, you would live for **10 years** and then die.

Would you prefer Medicine A or Medicine B, or are they the same?

Indicate your choice here. You can only choose one option:

◯ **Medicine A** Live for 6.5 years

◯ **Medicine B** Live for 10 years

◯ **Medicine A and Medicine B are the same.**

For peer review only

Repeated section

Remember Medicine A and Medicine B are equally effective and have no risks of a side effect.

The only difference between Medicine A and Medicine B is how you take them.

Medicine A	Medicine B
You take a tablet only once in your life.<br>You will be given the tablet on the day you come in to see your GP.<br>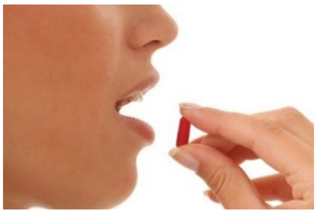	One tablet should be taken once every day, at night with cold water.<br>You need to take this medicine for 10 years.<br>You need to regularly visit a GP to receive a prescription.<br>You need to visit a pharmacy to fill a prescription.<br>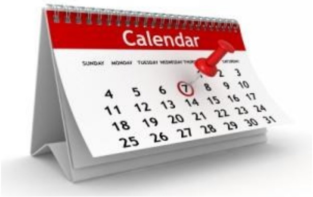<br>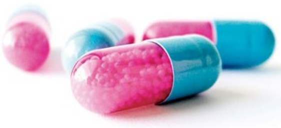

If you take Medicine A, you would live for **7 years** and then die.

If you take Medicine B, you would live for **10 years** and then die.

Would you prefer Medicine A or Medicine B, or are they the same?

Indicate your choice here. You can only choose one option:

◯ **Medicine A** Live for 7 years

◯ **Medicine B** Live for 10 years

◯ **Medicine A and Medicine B are the same.**

For peer review only

Repeated section

Remember Medicine A and Medicine B are equally effective and have no risks of a side effect.

The only difference between Medicine A and Medicine B is how you take them.

Medicine A	Medicine B
You take a tablet only once in your life.<br>You will be given the tablet on the day you come in to see your GP.<br>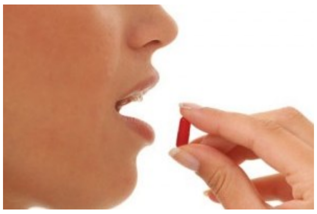	One tablet should be taken once every day, at night with cold water.<br>You need to take this medicine for 10 years.<br>You need to regularly visit a GP to receive a prescription.<br>You need to visit a pharmacy to fill a prescription.<br>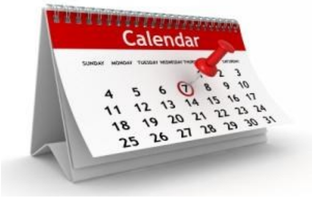<br>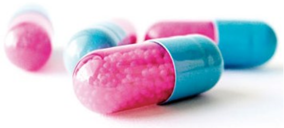

If you take Medicine A, you would live for **7.5 years (7 years and 6 months)** and then die.

If you take Medicine B, you would live for **10 years** and then die.

Would you prefer Medicine A or Medicine B, or are they the same?

Indicate your choice here. You can only choose one option:

◯ **Medicine A** 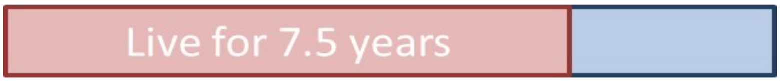

◯ **Medicine B** 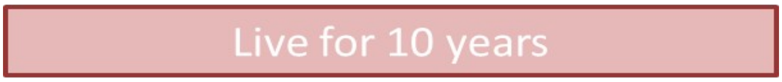

◯ **Medicine A and Medicine B are the same.**

0% 100%

For peer review only

Repeated section

Remember Medicine A and Medicine B are equally effective and have no risks of a side effect.

The only difference between Medicine A and Medicine B is how you take them.

Medicine A	Medicine B
You take a tablet only once in your life.<br>You will be given the tablet on the day you come in to see your GP.<br>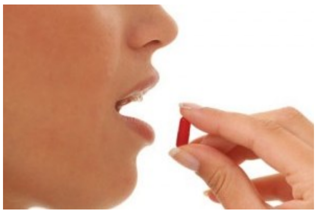	One tablet should be taken once every day, at night with cold water.<br>You need to take this medicine for 10 years.<br>You need to regularly visit a GP to receive a prescription.<br>You need to visit a pharmacy to fill a prescription.<br>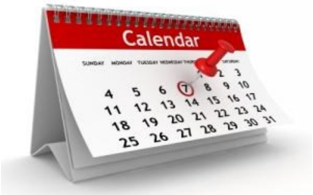<br>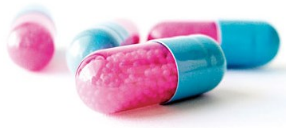

If you take Medicine A, you would live for **7.75 years (7 years and 9 months)** and then die.

If you take Medicine B, you would live for **10 years** and then die.

Would you prefer Medicine A or Medicine B, or are they the same?

Indicate your choice here. You can only choose one option:

◯ **Medicine A** Live for 7 years and 9 months

◯ **Medicine B** Live for 10 years

◯ **Medicine A and Medicine B are the same.**

0% 100%

For peer review only

Repeated section

Remember Medicine A and Medicine B are equally effective and have no risks of a side effect.

The only difference between Medicine A and Medicine B is how you take them.

**Medicine A**	**Medicine B**
You take a tablet only once in your life.<br>You will be given the tablet on the day you come in to see your GP.<br>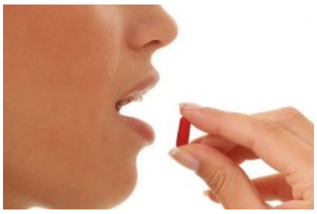	One tablet should be taken once every day, at night with cold water.<br>You need to take this medicine for 10 years.<br>You need to regularly visit a GP to receive a prescription.<br>You need to visit a pharmacy to fill a prescription.<br>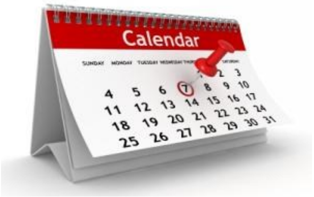<br>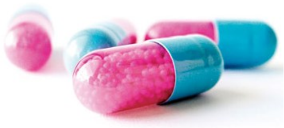

If you take Medicine A, you would live for **8 years** and then die.

If you take Medicine B, you would live for **10 years** and then die.

Would you prefer Medicine A or Medicine B, or are they the same?

Indicate your choice here. You can only choose one option:

◯ **Medicine A** Live for 8 years

◯ **Medicine B** Live for 10 years

◯ **Medicine A and Medicine B are the same.**

For peer review only

Repeated section

Remember Medicine A and Medicine B are equally effective and have no risks of a side effect.

The only difference between Medicine A and Medicine B is how you take them.

**Medicine A**	**Medicine B**
You take a tablet only once in your life.<br>You will be given the tablet on the day you come in to see your GP.<br>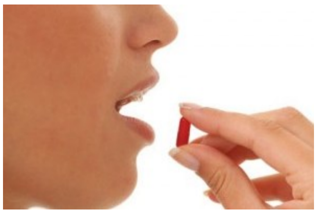	One tablet should be taken once every day, at night with cold water.<br>You need to take this medicine for 10 years.<br>You need to regularly visit a GP to receive a prescription.<br>You need to visit a pharmacy to fill a prescription.<br>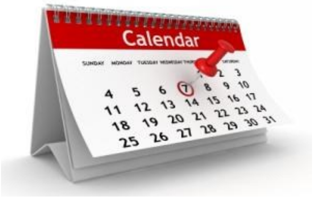<br>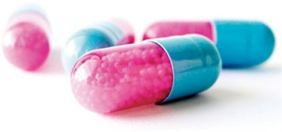

If you take Medicine A, you would live for **8.25 years (8 years and 3 months)** and then die.

If you take Medicine B, you would live for **10 years** and then die.

Would you prefer Medicine A or Medicine B, or are they the same?

Indicate your choice here. You can only choose one option:

◯ **Medicine A** Live for 8 years and 3 months

◯ **Medicine B** Live for 10 years

◯ **Medicine A and Medicine B are the same.**

0% 100%

For peer review only

Repeated section

Remember Medicine A and Medicine B are equally effective and have no risks of a side effect.

The only difference between Medicine A and Medicine B is how you take them.

Medicine A	Medicine B
You take a tablet only once in your life.<br>You will be given the tablet on the day you come in to see your GP.<br>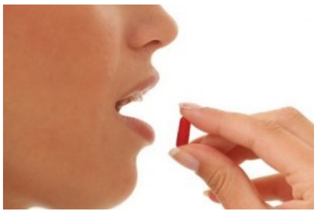	One tablet should be taken once every day, at night with cold water.<br>You need to take this medicine for 10 years.<br>You need to regularly visit a GP to receive a prescription.<br>You need to visit a pharmacy to fill a prescription.<br>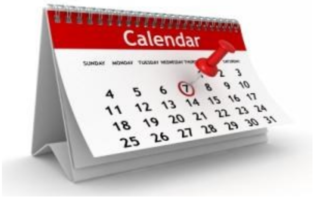<br>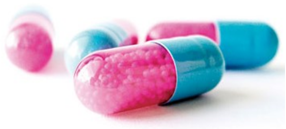

If you take Medicine A, you would live for **8.5 years** (8 years and 6 months) and then die.

If you take Medicine B, you would live for **10 years** and then die.

Would you prefer Medicine A or Medicine B, or are they the same?

Indicate your choice here. You can only choose one option:

◯ **Medicine A** 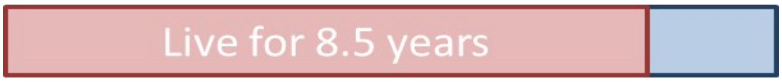

◯ **Medicine B** 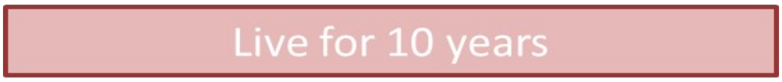

◯ **Medicine A and Medicine B are the same.**

For peer review only

Repeated section

Remember Medicine A and Medicine B are equally effective and have no risks of a side effect.

The only difference between Medicine A and Medicine B is how you take them.

Medicine A	Medicine B
You take a tablet only once in your life.<br>You will be given the tablet on the day you come in to see your GP.<br>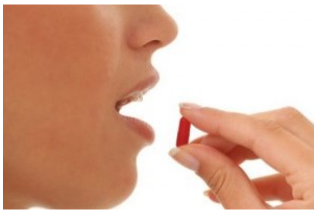	One tablet should be taken once every day, at night with cold water.<br>You need to take this medicine for 10 years.<br>You need to regularly visit a GP to receive a prescription.<br>You need to visit a pharmacy to fill a prescription.<br>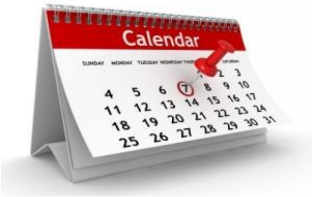<br>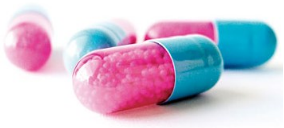

If you take Medicine A, you would live for **8.75 years (8 years and 9 months)** and then die.

If you take Medicine B, you would live for **10 years** and then die.

Would you prefer Medicine A or Medicine B, or are they the same?

Indicate your choice here. You can only choose one option:

○ **Medicine A** Live for 8 years and 9 months

○ **Medicine B** Live for 10 years

○ **Medicine A and Medicine B are the same.**

For peer review only

Repeated section

Remember Medicine A and Medicine B are equally effective and have no risks of a side effect.

The only difference between Medicine A and Medicine B is how you take them.

Medicine A	Medicine B
You take a tablet only once in your life.<br>You will be given the tablet on the day you come in to see your GP.<br>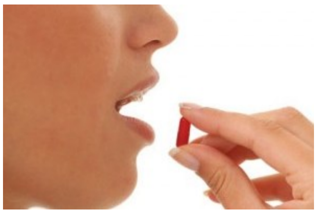	One tablet should be taken once every day, at night with cold water.<br>You need to take this medicine for 10 years.<br>You need to regularly visit a GP to receive a prescription.<br>You need to visit a pharmacy to fill a prescription.<br>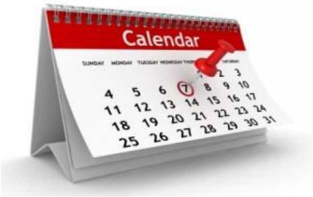 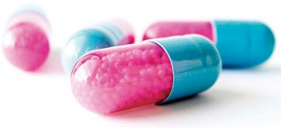

If you take Medicine A, you would live for **9 years** and then die.

If you take Medicine B, you would live for **10 years** and then die.

Would you prefer Medicine A or Medicine B, or are they the same?

Indicate your choice here. You can only choose one option:

◯ **Medicine A** Live for 9 years

◯ **Medicine B** Live for 10 years

◯ **Medicine A and Medicine B are the same.**

0% 100%

For peer review only

Repeated section

Remember Medicine A and Medicine B are equally effective and have no risks of a side effect.

The only difference between Medicine A and Medicine B is how you take them.

Medicine A	Medicine B
You take a tablet only once in your life.<br>You will be given the tablet on the day you come in to see your GP.<br>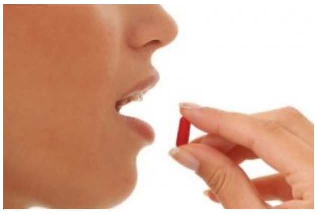	One tablet should be taken once every day, at night with cold water.<br>You need to take this medicine for 10 years.<br>You need to regularly visit a GP to receive a prescription.<br>You need to visit a pharmacy to fill a prescription.<br>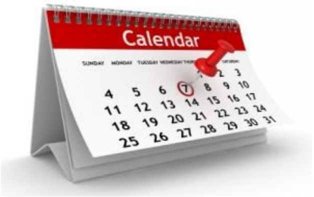<br>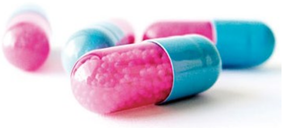

If you take Medicine A, you would live for **9 years and 1 month** and then die.

If you take Medicine B, you would live for **10 years** and then die.

Would you prefer Medicine A or Medicine B, or are they the same?

Indicate your choice here. You can only choose one option:

○ **Medicine A** Live for 9 years 1 month

○ **Medicine B** Live for 10 years

○ **Medicine A and Medicine B are the same.**

0% 100%

For peer review only

Repeated section

Remember Medicine A and Medicine B are equally effective and have no risks of a side effect.

The only difference between Medicine A and Medicine B is how you take them.

Medicine A	Medicine B
You take a tablet only once in your life.<br>You will be given the tablet on the day you come in to see your GP.<br>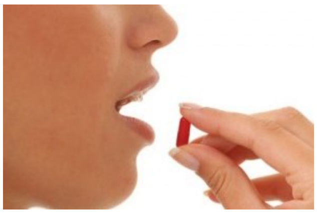	One tablet should be taken once every day, at night with cold water.<br>You need to take this medicine for 10 years.<br>You need to regularly visit a GP to receive a prescription.<br>You need to visit a pharmacy to fill a prescription.<br>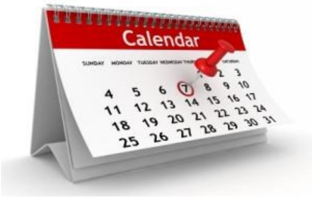<br>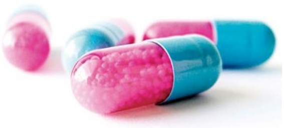

If you take Medicine A, you would live for **9.25 years (9 years and 3 months)** and then die.

If you take Medicine B, you would live for **10 years** and then die.

Would you prefer Medicine A or Medicine B, or are they the same?

Indicate your choice here. You can only choose one option:

○ **Medicine A** Live for 9 years and 3 months

○ **Medicine B** Live for 10 years

○ **Medicine A and Medicine B are the same.**

For peer review only

Repeated section

Remember Medicine A and Medicine B are equally effective and have no risks of a side effect.

The only difference between Medicine A and Medicine B is how you take them.

Medicine A	Medicine B
You take a tablet only once in your life.<br>You will be given the tablet on the day you come in to see your GP.<br>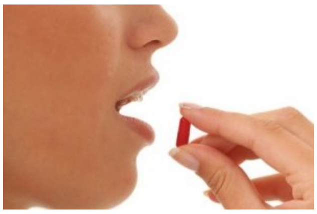	One tablet should be taken once every day, at night with cold water.<br>You need to take this medicine for 10 years.<br>You need to regularly visit a GP to receive a prescription.<br>You need to visit a pharmacy to fill a prescription.<br>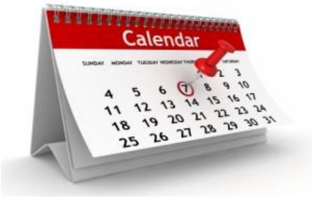<br>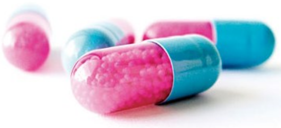

If you take Medicine A, you would live for **9 years and 4 months** and then die.

If you take Medicine B, you would live for **10 years** and then die.

Would you prefer Medicine A or Medicine B, or are they the same?

Indicate your choice here. You can only choose one option:

◯ **Medicine A** Live for 9 years 4 months

◯ **Medicine B** Live for 10 years

◯ **Medicine A and Medicine B are the same.**

0% 100%

For peer review only

Repeated section

Remember Medicine A and Medicine B are equally effective and have no risks of a side effect.

The only difference between Medicine A and Medicine B is how you take them.

Medicine A	Medicine B
You take a tablet only once in your life.<br>You will be given the tablet on the day you come in to see your GP.<br>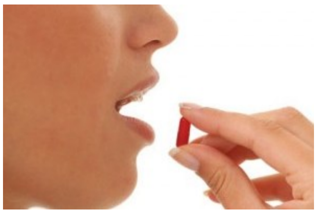	One tablet should be taken once every day, at night with cold water.<br>You need to take this medicine for 10 years.<br>You need to regularly visit a GP to receive a prescription.<br>You need to visit a pharmacy to fill a prescription.<br>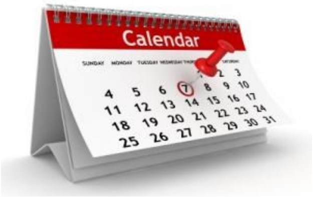<br>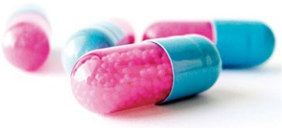

If you take Medicine A, you would live for **9 years and 5 months** and then die.

If you take Medicine B, you would live for **10 years** and then die.

Would you prefer Medicine A or Medicine B, or are they the same?

Indicate your choice here. You can only choose one option:

○ **Medicine A** Live for 9 years 5 months

○ **Medicine B** Live for 10 years

○ **Medicine A and Medicine B are the same.**

For peer review only

Repeated section

Remember Medicine A and Medicine B are equally effective and have no risks of a side effect.

The only difference between Medicine A and Medicine B is how you take them.

**Medicine A**	**Medicine B**
You take a tablet only once in your life.<br>You will be given the tablet on the day you come in to see your GP.<br>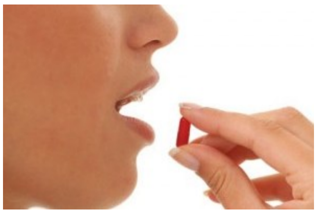	One tablet should be taken once every day, at night with cold water.<br>You need to take this medicine for 10 years.<br>You need to regularly visit a GP to receive a prescription.<br>You need to visit a pharmacy to fill a prescription.<br>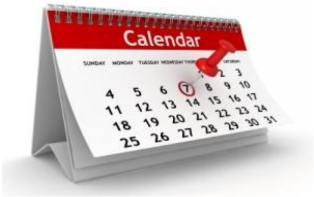<br><br>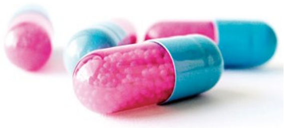

If you take Medicine A, you would live for **9.5 years** (9 years and 6 months) and then die.

If you take Medicine B, you would live for **10 years** and then die.

Would you prefer Medicine A or Medicine B, or are they the same?

Indicate your choice here. You can only choose one option:

◯ **Medicine A** Live for 9.5 years

◯ **Medicine B** Live for 10 years

◯ **Medicine A and Medicine B are the same.**

0% 100%

For peer review only

Repeated section

Remember Medicine A and Medicine B are equally effective and have no risks of a side effect.

The only difference between Medicine A and Medicine B is how you take them.

**Medicine A**	**Medicine B**
You take a tablet only once in your life.<br>You will be given the tablet on the day you come in to see your GP.<br>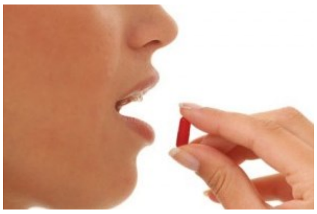	One tablet should be taken once every day, at night with cold water.<br>You need to take this medicine for 10 years.<br>You need to regularly visit a GP to receive a prescription.<br>You need to visit a pharmacy to fill a prescription.<br>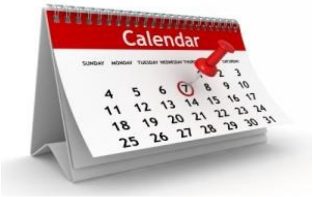<br>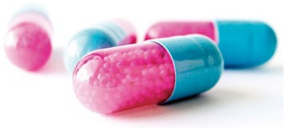

If you take Medicine A, you would live for **5 months less than 10 years** and then die.

If you take Medicine B, you would live for **10 years** and then die.

Would you prefer Medicine A or Medicine B, or are they the same?

Indicate your choice here. You can only choose one option:

○ **Medicine A** — Live for (10 years – 5 months)

○ **Medicine B** — Live for 10 years

○ **Medicine A and Medicine B are the same.**

For peer review only

Repeated section

Remember Medicine A and Medicine B are equally effective and have no risks of a side effect.

The only difference between Medicine A and Medicine B is how you take them.

Medicine A	Medicine B
You take a tablet only once in your life.<br>You will be given the tablet on the day you come in to see your GP.<br>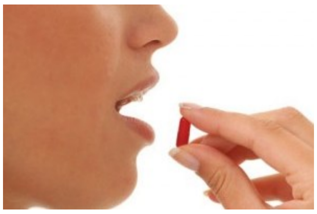	One tablet should be taken once every day, at night with cold water.<br>You need to take this medicine for 10 years.<br>You need to regularly visit a GP to receive a prescription.<br>You need to visit a pharmacy to fill a prescription.<br>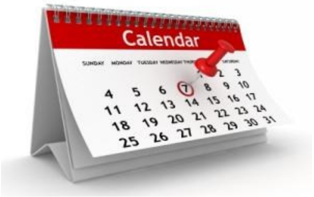<br><br>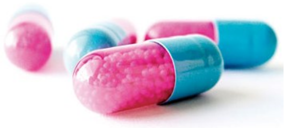

If you take Medicine A, you would live for **4 months less than 10 years** and then die.

If you take Medicine B, you would live for **10 years** and then die.

Would you prefer Medicine A or Medicine B, or are they the same?

Indicate your choice here. You can only choose one option:

○ **Medicine A** Live for (10 years – 4 months)

○ **Medicine B** Live for 10 years

○ **Medicine A and Medicine B are the same.**

0% 100%

For peer review only

Repeated section

Remember Medicine A and Medicine B are equally effective and have no risks of a side effect.

The only difference between Medicine A and Medicine B is how you take them.

Medicine A	Medicine B
You take a tablet only once in your life.<br>You will be given the tablet on the day you come in to see your GP.<br>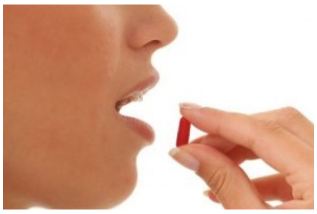	One tablet should be taken once every day, at night with cold water.<br>You need to take this medicine for 10 years.<br>You need to regularly visit a GP to receive a prescription.<br>You need to visit a pharmacy to fill a prescription.<br>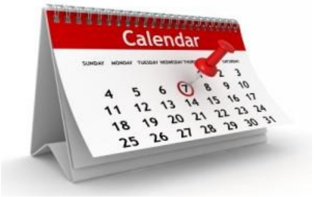<br><br>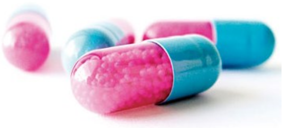

If you take Medicine A, you would live for **3 months less than 10 years** and then die.

If you take Medicine B, you would live for **10 years** and then die.

Would you prefer Medicine A or Medicine B, or are they the same?

Indicate your choice here. You can only choose one option:

○ **Medicine A** Live for (10 years – 3 months)

○ **Medicine B** Live for 10 years

○ **Medicine A and Medicine B are the same.**

For peer review only

Repeated section

Remember Medicine A and Medicine B are equally effective and have no risks of a side effect.

The only difference between Medicine A and Medicine B is how you take them.

Medicine A	Medicine B
You take a tablet only once in your life.<br>You will be given the tablet on the day you come in to see your GP.<br>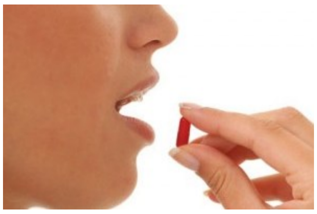	One tablet should be taken once every day, at night with cold water.<br>You need to take this medicine for 10 years.<br>You need to regularly visit a GP to receive a prescription.<br>You need to visit a pharmacy to fill a prescription.<br>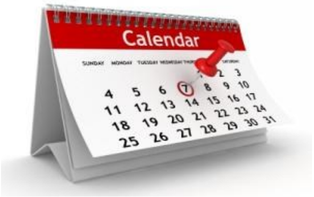<br>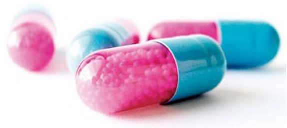

If you take Medicine A, you would live for **2 months less than 10 years** and then die.

If you take Medicine B, you would live for **10 years** and then die.

Would you prefer Medicine A or Medicine B, or are they the same?

Indicate your choice here. You can only choose one option:

◯ **Medicine A** Live for (10 years – 2 months)

◯ **Medicine B** Live for 10 years

◯ **Medicine A and Medicine B are the same.**

For peer review only

Repeated section

Remember Medicine A and Medicine B are equally effective and have no risks of a side effect.

The only difference between Medicine A and Medicine B is how you take them.

Medicine A	Medicine B
You take a tablet only once in your life.<br>You will be given the tablet on the day you come in to see your GP.<br>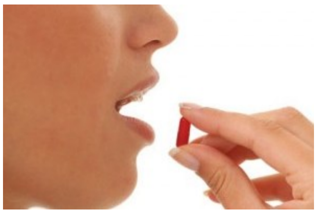	One tablet should be taken once every day, at night with cold water.<br>You need to take this medicine for 10 years.<br>You need to regularly visit a GP to receive a prescription.<br>You need to visit a pharmacy to fill a prescription.<br>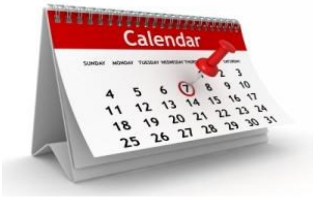<br>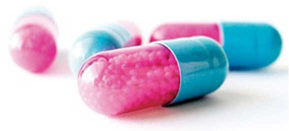

If you take Medicine A, you would live for **1 month less than 10 years** and then die.

If you take Medicine B, you would live for **10 years** and then die.

Would you prefer Medicine A or Medicine B, or are they the same?

Indicate your choice here. You can only choose one option:

○ **Medicine A** Live for (10 years – 1 month)

○ **Medicine B** Live for 10 years

○ **Medicine A and Medicine B are the same.**

For peer review only

Repeated section

Remember Medicine A and Medicine B are equally effective and have no risks of a side effect.

The only difference between Medicine A and Medicine B is how you take them.

**Medicine A**	**Medicine B**
You take a tablet only once in your life.<br>You will be given the tablet on the day you come in to see your GP.<br>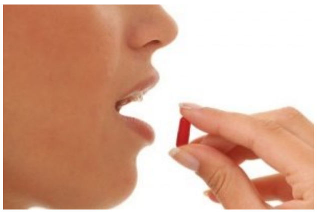	One tablet should be taken once every day, at night with cold water.<br>You need to take this medicine for 10 years.<br>You need to regularly visit a GP to receive a prescription.<br>You need to visit a pharmacy to fill a prescription.<br>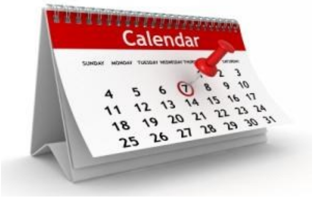<br>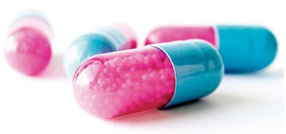

If you take Medicine A, you would live for **3 weeks less than 10 years** and then die.

If you take Medicine B, you would live for **10 years** and then die.

Would you prefer Medicine A or Medicine B, or are they the same?

Indicate your choice here. You can only choose one option:

◯ **Medicine A** Live for (10 years – 3 weeks)

◯ **Medicine B** Live for 10 years

◯ **Medicine A and Medicine B are the same.**

For peer review only

Repeated section

Remember Medicine A and Medicine B are equally effective and have no risks of a side effect.

The only difference between Medicine A and Medicine B is how you take them.

**Medicine A**	**Medicine B**
You take a tablet only once in your life.<br>You will be given the tablet on the day you come in to see your GP.<br>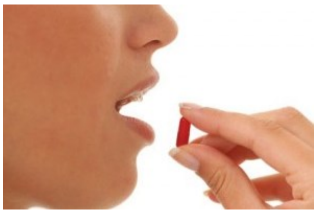	One tablet should be taken once every day, at night with cold water.<br>You need to take this medicine for 10 years.<br>You need to regularly visit a GP to receive a prescription.<br>You need to visit a pharmacy to fill a prescription.<br>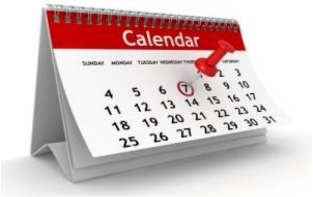<br>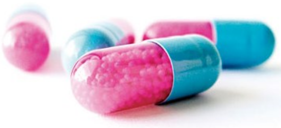

If you take Medicine A, you would live for **2 weeks less than 10 years** and then die.

If you take Medicine B, you would live for **10 years** and then die.

Would you prefer Medicine A or Medicine B, or are they the same?

Indicate your choice here. You can only choose one option:

○ **Medicine A** Live for (10 years – 2 weeks)

○ **Medicine B** Live for 10 years

○ **Medicine A and Medicine B are the same.**

0% 100%

For peer review only

Repeated section

Remember Medicine A and Medicine B are equally effective and have no risks of a side effect.

The only difference between Medicine A and Medicine B is how you take them.

**Medicine A**	**Medicine B**
You take a tablet only once in your life.<br>You will be given the tablet on the day you come in to see your GP.<br>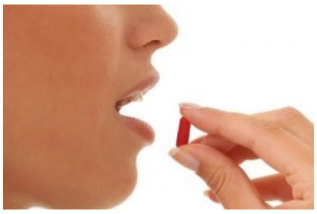	One tablet should be taken once every day, at night with cold water.<br>You need to take this medicine for 10 years.<br>You need to regularly visit a GP to receive a prescription.<br>You need to visit a pharmacy to fill a prescription.<br>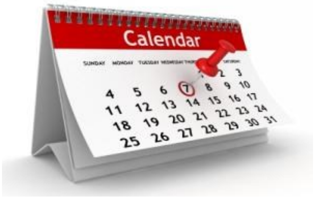<br>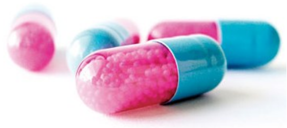

If you take Medicine A, you would live for **10 days less than 10 years** and then die.

If you take Medicine B, you would live for **10 years** and then die.

Would you prefer Medicine A or Medicine B, or are they the same?

Indicate your choice here. You can only choose one option:

○ **Medicine A** Live for (10 years – 10 days)

○ **Medicine B** Live for 10 years

○ **Medicine A and Medicine B are the same.**

For peer review only

Repeated section

Remember Medicine A and Medicine B are equally effective and have no risks of a side effect.

The only difference between Medicine A and Medicine B is how you take them.

**Medicine A**	**Medicine B**
You take a tablet only once in your life.<br>You will be given the tablet on the day you come in to see your GP.<br>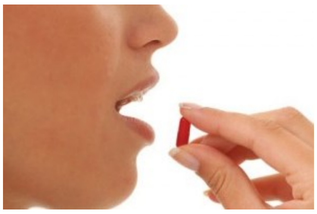	One tablet should be taken once every day, at night with cold water.<br>You need to take this medicine for 10 years.<br>You need to regularly visit a GP to receive a prescription.<br>You need to visit a pharmacy to fill a prescription.<br>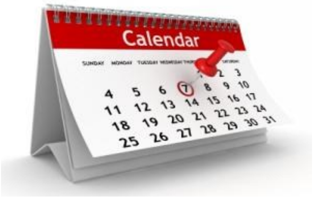<br><br>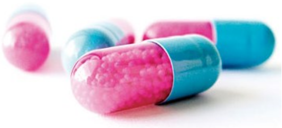

If you take Medicine A, you would live for **1 week less than 10 years** and then die.

If you take Medicine B, you would live for **10 years** and then die.

Would you prefer Medicine A or Medicine B, or are they the same?

Indicate your choice here. You can only choose one option:

○ **Medicine A** Live for (10 years – 1 week)

○ **Medicine B** Live for 10 years

○ **Medicine A and Medicine B are the same.**

For peer review only

Repeated section

Remember Medicine A and Medicine B are equally effective and have no risks of a side effect.

The only difference between Medicine A and Medicine B is how you take them.

Medicine A	Medicine B
You take a tablet only once in your life.<br>You will be given the tablet on the day you come in to see your GP.<br>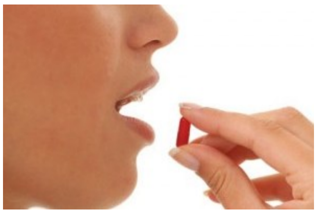	One tablet should be taken once every day, at night with cold water.<br>You need to take this medicine for 10 years.<br>You need to regularly visit a GP to receive a prescription.<br>You need to visit a pharmacy to fill a prescription.<br>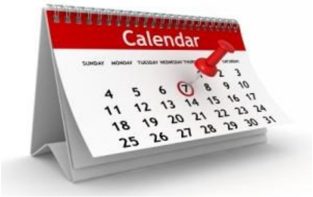<br>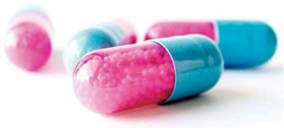

If you take Medicine A, you would live for **5 days less than 10 years** and then die.

If you take Medicine B, you would live for **10 years** and then die.

Would you prefer Medicine A or Medicine B, or are they the same?

Indicate your choice here. You can only choose one option:

◯ **Medicine A** Live for (10 years – 5 days)

◯ **Medicine B** Live for 10 years

◯ **Medicine A and Medicine B are the same.**

For peer review only

Repeated section

Remember Medicine A and Medicine B are equally effective and have no risks of a side effect.

The only difference between Medicine A and Medicine B is how you take them.

**Medicine A**	**Medicine B**
You take a tablet only once in your life.<br>You will be given the tablet on the day you come in to see your GP.<br>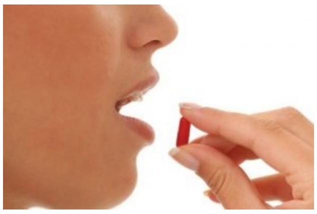	One tablet should be taken once every day, at night with cold water.<br>You need to take this medicine for 10 years.<br>You need to regularly visit a GP to receive a prescription.<br>You need to visit a pharmacy to fill a prescription.<br>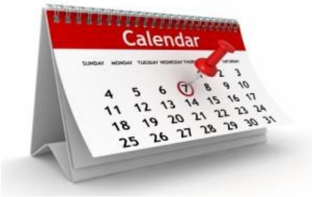<br>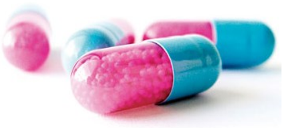

If you take Medicine A, you would live for **3 days less than 10 years** and then die.

If you take Medicine B, you would live for **10 years** and then die.

Would you prefer Medicine A or Medicine B, or are they the same?

Indicate your choice here. You can only choose one option:

○ **Medicine A** Live for (10 years – 3 days)

○ **Medicine B** Live for 10 years

○ **Medicine A and Medicine B are the same.**

0% 100%

For peer review only

Repeated section

Remember Medicine A and Medicine B are equally effective and have no risks of a side effect.

The only difference between Medicine A and Medicine B is how you take them.

**Medicine A**	**Medicine B**
You take a tablet only once in your life.<br>You will be given the tablet on the day you come in to see your GP.<br>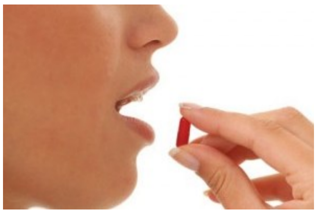	One tablet should be taken once every day, at night with cold water.<br>You need to take this medicine for 10 years.<br>You need to regularly visit a GP to receive a prescription.<br>You need to visit a pharmacy to fill a prescription.<br>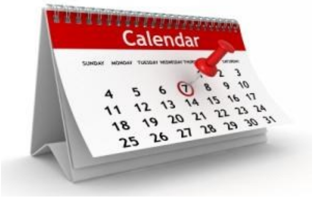 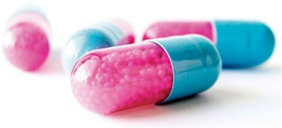

If you take Medicine A, you would live for **1 day less than 10 years** and then die.

If you take Medicine B, you would live for **10 years** and then die.

Would you prefer Medicine A or Medicine B, or are they the same?

Indicate your choice here. You can only choose one option:

- ◯ **Medicine A** — Live for (10 years – 1 day)
- ◯ **Medicine B** — Live for 10 years
- ◯ **Medicine A and Medicine B are the same.**

For peer review only

Question 4a.

You prefer living for 5 years on Medicine A, to living for 10 years on Medicine B.

This means **living fewer than 5 years on Medicine A** can be the same as living for 10 years on Medicine B.

How many years on Medicine A do you think would be the same as living for 10 years on Medicine B?

(Please give a number in years)

0% 100%

peer review only

Was there anything so far which you think is unclear or that you didn't understand?

(Please provide details in the comment box below)

0% 100%

peer review only

Part 2a

Question 5

You have been told that you need to take a medicine to reduce the risk of heart disease.

You can choose between two different medicines. We have called these Medicine A and Medicine B.

Medicine A is a pill that you only need to take once.

Medicine B is a pill that you need to take every day for ten years.

Medicine A and Medicine B are equally effective in reducing your risk of developing heart disease.

Medicine A and Medicine B will reduce your risk of having a heart attack or a stroke within the next 10 years from **10%** to **6%**.

In other words, in a crowd of 1,000 people in the UK, **100** are likely to have a heart attack or a stroke if they do not take the medicine. This is reduced to **60** people out of 1,000 who do take the medicine. This information is also illustrated in this picture.

Risk of heart disease

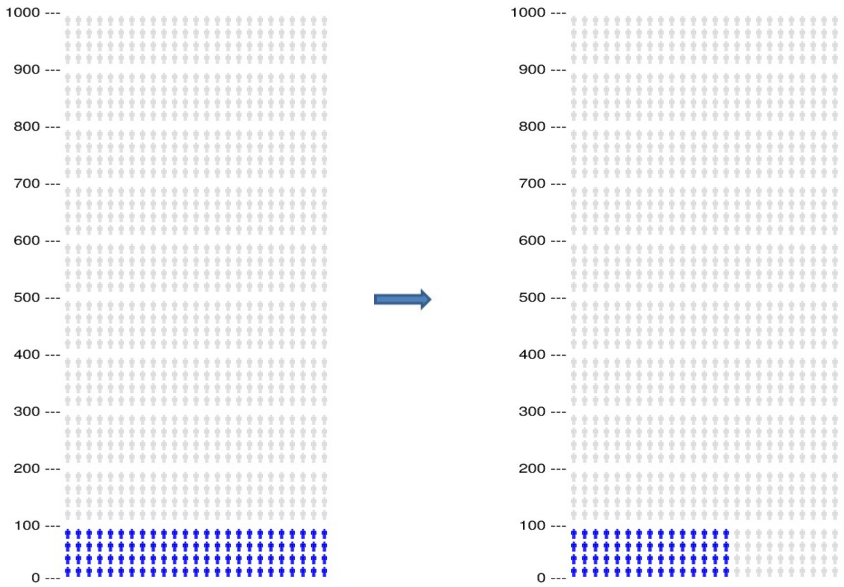

10%
No Treatment

6%
Taking the Medicine

(The figures shaded blue represent people who will have a heart attack or a stroke in the next 10 years)

Please continue to the next page.

For peer review only

Repeated section

Remember Medicine A and Medicine B are equally effective.

However, there are some differences between Medicine A and Medicine B.

Medicine A	Medicine B
**No inconvenience**<br><br>You take a tablet only once in your life.<br>You will be given the tablet on the day you come in to see your GP.	**Inconvenience**<br><br>One tablet should be taken once every day, at night with cold water.<br>You need to take this medicine for 10 years.<br>You need to regularly visit a GP to receive a prescription.<br>You need to visit a pharmacy to fill a prescription.
**Severe side effect - 0%**<br><br>No one taking Medicine A will have a severe side effect.	**Severe side effect - 0.7%**<br><br>Among every 1,000 people taking Medicine B, **7** will experience a severe side effect.
**Minor side effect - 0%**<br><br>No one taking Medicine A will have a minor side effect.	**Minor side effect - 13%**<br><br>Among every 1,000 people taking Medicine B, **130** will experience a minor side effect.

If you take Medicine A, you would live for **5 years** and then die.

If you take Medicine B, you would live for **10 years** and then die.

Would you prefer Medicine A or Medicine B, or are they the same?

Indicate your choice here. You can only choose one option:

◯ **Medicine A** Live for 5 years

◯ **Medicine B** Live for 10 years

◯ **Medicine A and Medicine B are the same.**

0% 100%

Repeated section

Remember Medicine A and Medicine B are equally effective.

However, there are some differences between Medicine A and Medicine B.

Medicine A	Medicine B
**No inconvenience**<br><br>You take a tablet only once in your life.<br>You will be given the tablet on the day you come in to see your GP.	**Inconvenience**<br><br>One tablet should be taken once every day, at night with cold water.<br>You need to take this medicine for 10 years.<br>You need to regularly visit a GP to receive a prescription.<br>You need to visit a pharmacy to fill a prescription.
**Severe side effect - 0%**<br><br>No one taking Medicine A will have a severe side effect.	**Severe side effect - 0.7%**<br><br>Among every 1,000 people taking Medicine B, **7** will experience a severe side effect.
**Minor side effect - 0%**<br><br>No one taking Medicine A will have a minor side effect.	**Minor side effect - 13%**<br><br>Among every 1,000 people taking Medicine B, **130** will experience a minor side effect.

If you take Medicine A, you would live for **5.5 years** (5 years and 6 months) and then die.

If you take Medicine B, you would live for **10 years** and then die.

Would you prefer Medicine A or Medicine B, or are they the same?

Indicate your choice here. You can only choose one option:

◯ **Medicine A**

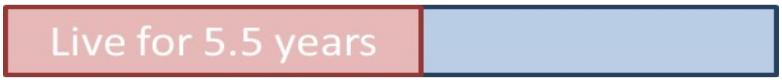

◯ **Medicine B**

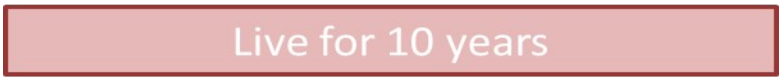

◯ **Medicine A and Medicine B are the same.**

0% 100%

Repeated section

Remember Medicine A and Medicine B are equally effective.

However, there are some differences between Medicine A and Medicine B.

Medicine A	Medicine B
**No inconvenience**<br><br>You take a tablet only once in your life.<br>You will be given the tablet on the day you come in to see your GP.	**Inconvenience**<br><br>One tablet should be taken once every day, at night with cold water.<br>You need to take this medicine for 10 years.<br>You need to regularly visit a GP to receive a prescription.<br>You need to visit a pharmacy to fill a prescription.
**Severe side effect - 0%**<br><br>No one taking Medicine A will have a severe side effect.	**Severe side effect - 0.7%**<br><br>Among every 1,000 people taking Medicine B, **7** will experience a severe side effect.
**Minor side effect - 0%**<br><br>No one taking Medicine A will have a minor side effect.	**Minor side effect - 13%**<br><br>Among every 1,000 people taking Medicine B, **130** will experience a minor side effect.

If you take Medicine A, you would live for **6 years** and then die.

If you take Medicine B, you would live for **10 years** and then die.

Would you prefer Medicine A or Medicine B, or are they the same?

Indicate your choice here. You can only choose one option:

◯ **Medicine A**

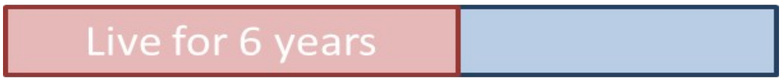

◯ **Medicine B**

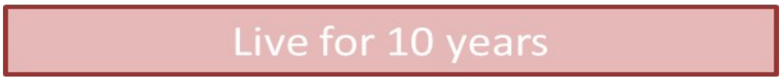

◯ **Medicine A and Medicine B are the same.**

0% 100%

Repeated section

Remember Medicine A and Medicine B are equally effective.

However, there are some differences between Medicine A and Medicine B.

Medicine A	Medicine B
**No inconvenience**<br><br>You take a tablet only once in your life.<br>You will be given the tablet on the day you come in to see your GP.	**Inconvenience**<br><br>One tablet should be taken once every day, at night with cold water.<br>You need to take this medicine for 10 years.<br>You need to regularly visit a GP to receive a prescription.<br>You need to visit a pharmacy to fill a prescription.
**Severe side effect - 0%**<br><br>No one taking Medicine A will have a severe side effect.	**Severe side effect - 0.7%**<br><br>Among every 1,000 people taking Medicine B, **7** will experience a severe side effect.
**Minor side effect - 0%**<br><br>No one taking Medicine A will have a minor side effect.	**Minor side effect - 13%**<br><br>Among every 1,000 people taking Medicine B, **130** will experience a minor side effect.

If you take Medicine A, you would live for **6.25 years (6 years and 3 months)** and then die.

If you take Medicine B, you would live for **10 years** and then die.

Would you prefer Medicine A or Medicine B, or are they the same?

Indicate your choice here. You can only choose one option:

- ○ **Medicine A** — Live for 6 years and 3 months
- ○ **Medicine B** — Live for 10 years
- ○ **Medicine A and Medicine B are the same.**

0% 100%

Repeated section

Remember Medicine A and Medicine B are equally effective.

However, there are some differences between Medicine A and Medicine B.

Medicine A	Medicine B
**No inconvenience**<br><br>You take a tablet only once in your life.<br>You will be given the tablet on the day you come in to see your GP.	**Inconvenience**<br><br>One tablet should be taken once every day, at night with cold water.<br>You need to take this medicine for 10 years.<br>You need to regularly visit a GP to receive a prescription.<br>You need to visit a pharmacy to fill a prescription.
**Severe side effect - 0%**<br><br>No one taking Medicine A will have a severe side effect.	**Severe side effect - 0.7%**<br><br>Among every 1,000 people taking Medicine B, **7** will experience a severe side effect.
**Minor side effect - 0%**<br><br>No one taking Medicine A will have a minor side effect.	**Minor side effect - 13%**<br><br>Among every 1,000 people taking Medicine B, **130** will experience a minor side effect.

If you take Medicine A, you would live for **6.5 years** (6 years and 6 months) and then die.

If you take Medicine B, you would live for **10 years** and then die.

Would you prefer Medicine A or Medicine B, or are they the same?

Indicate your choice here. You can only choose one option:

○ **Medicine A** Live for 6.5 years

○ **Medicine B** Live for 10 years

○ **Medicine A and Medicine B are the same.**

0% 100%

Repeated section

Remember Medicine A and Medicine B are equally effective.

However, there are some differences between Medicine A and Medicine B.

Medicine A	Medicine B
**No inconvenience**<br><br>You take a tablet only once in your life.<br>You will be given the tablet on the day you come in to see your GP.	**Inconvenience**<br><br>One tablet should be taken once every day, at night with cold water.<br>You need to take this medicine for 10 years.<br>You need to regularly visit a GP to receive a prescription.<br>You need to visit a pharmacy to fill a prescription.
**Severe side effect - 0%**<br><br>No one taking Medicine A will have a severe side effect.	**Severe side effect - 0.7%**<br><br>Among every 1,000 people taking Medicine B, **7** will experience a severe side effect.
**Minor side effect - 0%**<br><br>No one taking Medicine A will have a minor side effect.	**Minor side effect - 13%**<br><br>Among every 1,000 people taking Medicine B, **130** will experience a minor side effect.

If you take Medicine A, you would live for **7 years** and then die.

If you take Medicine B, you would live for **10 years** and then die.

Would you prefer Medicine A or Medicine B, or are they the same?

Indicate your choice here. You can only choose one option:

○ **Medicine A** Live for 7 years

○ **Medicine B** Live for 10 years

○ **Medicine A and Medicine B are the same.**

0% 100%

Repeated section

Remember Medicine A and Medicine B are equally effective.

However, there are some differences between Medicine A and Medicine B.

Medicine A	Medicine B
**No inconvenience**<br><br>You take a tablet only once in your life.<br>You will be given the tablet on the day you come in to see your GP.	**Inconvenience**<br><br>One tablet should be taken once every day, at night with cold water.<br>You need to take this medicine for 10 years.<br>You need to regularly visit a GP to receive a prescription.<br>You need to visit a pharmacy to fill a prescription.
**Severe side effect - 0%**<br><br>No one taking Medicine A will have a severe side effect.	**Severe side effect - 0.7%**<br><br>Among every 1,000 people taking Medicine B, **7** will experience a severe side effect.
**Minor side effect - 0%**<br><br>No one taking Medicine A will have a minor side effect.	**Minor side effect - 13%**<br><br>Among every 1,000 people taking Medicine B, **130** will experience a minor side effect.

If you take Medicine A, you would live for **7.5 years** (7 years and 6 months) and then die.

If you take Medicine B, you would live for **10 years** and then die.

Would you prefer Medicine A or Medicine B, or are they the same?

Indicate your choice here. You can only choose one option:

◯ **Medicine A** Live for 7.5 years

◯ **Medicine B** Live for 10 years

◯ **Medicine A and Medicine B are the same.**

0% 100%

Repeated section

Remember Medicine A and Medicine B are equally effective.

However, there are some differences between Medicine A and Medicine B.

Medicine A	Medicine B
**No inconvenience**<br><br>You take a tablet only once in your life.<br>You will be given the tablet on the day you come in to see your GP.	**Inconvenience**<br><br>One tablet should be taken once every day, at night with cold water.<br>You need to take this medicine for 10 years.<br>You need to regularly visit a GP to receive a prescription.<br>You need to visit a pharmacy to fill a prescription.
**Severe side effect - 0%**<br><br>No one taking Medicine A will have a severe side effect.	**Severe side effect - 0.7%**<br><br>Among every 1,000 people taking Medicine B, **7** will experience a severe side effect.
**Minor side effect - 0%**<br><br>No one taking Medicine A will have a minor side effect.	**Minor side effect - 13%**<br><br>Among every 1,000 people taking Medicine B, **130** will experience a minor side effect.

If you take Medicine A, you would live for **7.75 years (7 years and 9 months)** and then die.

If you take Medicine B, you would live for **10 years** and then die.

Would you prefer Medicine A or Medicine B, or are they the same?

Indicate your choice here. You can only choose one option:

○ **Medicine A** Live for 7 years and 9 months

○ **Medicine B** Live for 10 years

○ **Medicine A and Medicine B are the same.**

0% 100%

Repeated section

Remember Medicine A and Medicine B are equally effective.

However, there are some differences between Medicine A and Medicine B.

Medicine A	Medicine B
**No inconvenience**<br><br>You take a tablet only once in your life.<br>You will be given the tablet on the day you come in to see your GP.	**Inconvenience**<br><br>One tablet should be taken once every day, at night with cold water.<br>You need to take this medicine for 10 years.<br>You need to regularly visit a GP to receive a prescription.<br>You need to visit a pharmacy to fill a prescription.
**Severe side effect - 0%**<br><br>No one taking Medicine A will have a severe side effect.	**Severe side effect - 0.7%**<br><br>Among every 1,000 people taking Medicine B, **7** will experience a severe side effect.
**Minor side effect - 0%**<br><br>No one taking Medicine A will have a minor side effect.	**Minor side effect - 13%**<br><br>Among every 1,000 people taking Medicine B, **130** will experience a minor side effect.

If you take Medicine A, you would live for **8 years** and then die.

If you take Medicine B, you would live for **10 years** and then die.

Would you prefer Medicine A or Medicine B, or are they the same?

Indicate your choice here. You can only choose one option:

◯ **Medicine A** Live for 8 years

◯ **Medicine B** Live for 10 years

◯ **Medicine A and Medicine B are the same.**

0% 100%

Repeated section

Remember Medicine A and Medicine B are equally effective.

However, there are some differences between Medicine A and Medicine B.

Medicine A	Medicine B
**No inconvenience**<br><br>You take a tablet only once in your life.<br>You will be given the tablet on the day you come in to see your GP.	**Inconvenience**<br><br>One tablet should be taken once every day, at night with cold water.<br>You need to take this medicine for 10 years.<br>You need to regularly visit a GP to receive a prescription.<br>You need to visit a pharmacy to fill a prescription.
**Severe side effect - 0%**<br><br>No one taking Medicine A will have a severe side effect.	**Severe side effect - 0.7%**<br><br>Among every 1,000 people taking Medicine B, **7** will experience a severe side effect.
**Minor side effect - 0%**<br><br>No one taking Medicine A will have a minor side effect.	**Minor side effect - 13%**<br><br>Among every 1,000 people taking Medicine B, **130** will experience a minor side effect.

If you take Medicine A, you would live for **8.25 years (8 years and 3 months)** and then die.

If you take Medicine B, you would live for **10 years** and then die.

Would you prefer Medicine A or Medicine B, or are they the same?

Indicate your choice here. You can only choose one option:

○ **Medicine A** Live for 8 years and 3 months

○ **Medicine B** Live for 10 years

○ **Medicine A and Medicine B are the same.**

0% 100%

Repeated section

Remember Medicine A and Medicine B are equally effective.

However, there are some differences between Medicine A and Medicine B.

Medicine A	Medicine B
**No inconvenience**<br><br>You take a tablet only once in your life.<br>You will be given the tablet on the day you come in to see your GP.	**Inconvenience**<br><br>One tablet should be taken once every day, at night with cold water.<br>You need to take this medicine for 10 years.<br>You need to regularly visit a GP to receive a prescription.<br>You need to visit a pharmacy to fill a prescription.
**Severe side effect - 0%**<br><br>No one taking Medicine A will have a severe side effect.	**Severe side effect - 0.7%**<br><br>Among every 1,000 people taking Medicine B, **7** will experience a severe side effect.
**Minor side effect - 0%**<br><br>No one taking Medicine A will have a minor side effect.	**Minor side effect - 13%**<br><br>Among every 1,000 people taking Medicine B, **130** will experience a minor side effect.

If you take Medicine A, you would live for **8.5 years** (8 years and 6 months) and then die.

If you take Medicine B, you would live for **10 years** and then die.

Would you prefer Medicine A or Medicine B, or are they the same?

Indicate your choice here. You can only choose one option:

◯ **Medicine A**

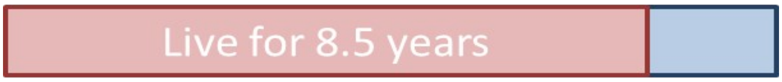

◯ **Medicine B**

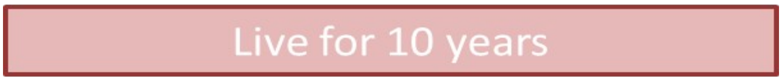

◯ **Medicine A and Medicine B are the same.**

0% 100%

Repeated section

Remember Medicine A and Medicine B are equally effective.

However, there are some differences between Medicine A and Medicine B.

Medicine A	Medicine B
**No inconvenience**<br><br>You take a tablet only once in your life.<br>You will be given the tablet on the day you come in to see your GP.	**Inconvenience**<br><br>One tablet should be taken once every day, at night with cold water.<br>You need to take this medicine for 10 years.<br>You need to regularly visit a GP to receive a prescription.<br>You need to visit a pharmacy to fill a prescription.
**Severe side effect - 0%**<br><br>No one taking Medicine A will have a severe side effect.	**Severe side effect - 0.7%**<br><br>Among every 1,000 people taking Medicine B, **7** will experience a severe side effect.
**Minor side effect - 0%**<br><br>No one taking Medicine A will have a minor side effect.	**Minor side effect - 13%**<br><br>Among every 1,000 people taking Medicine B, **130** will experience a minor side effect.

If you take Medicine A, you would live for **8.75 years (8 years and 9 months)** and then die.

If you take Medicine B, you would live for **10 years** and then die.

Would you prefer Medicine A or Medicine B, or are they the same?

Indicate your choice here. You can only choose one option:

◯ **Medicine A** Live for 8 years and 9 months

◯ **Medicine B** Live for 10 years

◯ **Medicine A and Medicine B are the same.**

0% 100%

Repeated section

Remember Medicine A and Medicine B are equally effective.

However, there are some differences between Medicine A and Medicine B.

Medicine A	Medicine B
**No inconvenience**<br><br>You take a tablet only once in your life.<br>You will be given the tablet on the day you come in to see your GP.	**Inconvenience**<br><br>One tablet should be taken once every day, at night with cold water.<br>You need to take this medicine for 10 years.<br>You need to regularly visit a GP to receive a prescription.<br>You need to visit a pharmacy to fill a prescription.
**Severe side effect - 0%**<br><br>No one taking Medicine A will have a severe side effect.	**Severe side effect - 0.7%**<br><br>Among every 1,000 people taking Medicine B, **7** will experience a severe side effect.
**Minor side effect - 0%**<br><br>No one taking Medicine A will have a minor side effect.	**Minor side effect - 13%**<br><br>Among every 1,000 people taking Medicine B, **130** will experience a minor side effect.

If you take Medicine A, you would live for **9 years** and then die.

If you take Medicine B, you would live for **10 years** and then die.

Would you prefer Medicine A or Medicine B, or are they the same?

Indicate your choice here. You can only choose one option:

◯ **Medicine A**

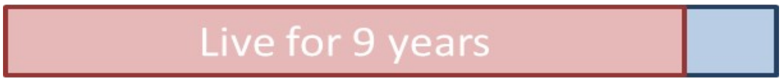

◯ **Medicine B**

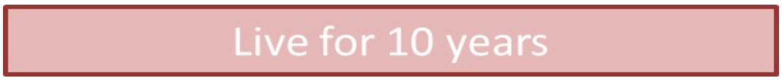

◯ **Medicine A and Medicine B are the same.**

0% 100%

Repeated section

Remember Medicine A and Medicine B are equally effective.

However, there are some differences between Medicine A and Medicine B.

Medicine A	Medicine B
**No inconvenience**<br><br>You take a tablet only once in your life.<br>You will be given the tablet on the day you come in to see your GP.	**Inconvenience**<br><br>One tablet should be taken once every day, at night with cold water.<br>You need to take this medicine for 10 years.<br>You need to regularly visit a GP to receive a prescription.<br>You need to visit a pharmacy to fill a prescription.
**Severe side effect - 0%**<br><br>No one taking Medicine A will have a severe side effect.	**Severe side effect - 0.7%**<br><br>Among every 1,000 people taking Medicine B, **7** will experience a severe side effect.
**Minor side effect - 0%**<br><br>No one taking Medicine A will have a minor side effect.	**Minor side effect - 13%**<br><br>Among every 1,000 people taking Medicine B, **130** will experience a minor side effect.

If you take Medicine A, you would live for **9 years and 1 month** and then die.

If you take Medicine B, you would live for **10 years** and then die.

Would you prefer Medicine A or Medicine B, or are they the same?

Indicate your choice here. You can only choose one option:

◯ **Medicine A** Live for 9 years 1 month

◯ **Medicine B** Live for 10 years

◯ **Medicine A and Medicine B are the same.**

0% 100%

Repeated section

Remember Medicine A and Medicine B are equally effective.

However, there are some differences between Medicine A and Medicine B.

Medicine A	Medicine B
**No inconvenience**<br><br>You take a tablet only once in your life.<br>You will be given the tablet on the day you come in to see your GP.	**Inconvenience**<br><br>One tablet should be taken once every day, at night with cold water.<br>You need to take this medicine for 10 years.<br>You need to regularly visit a GP to receive a prescription.<br>You need to visit a pharmacy to fill a prescription.
**Severe side effect - 0%**<br><br>No one taking Medicine A will have a severe side effect.	**Severe side effect - 0.7%**<br><br>Among every 1,000 people taking Medicine B, **7** will experience a severe side effect.
**Minor side effect - 0%**<br><br>No one taking Medicine A will have a minor side effect.	**Minor side effect - 13%**<br><br>Among every 1,000 people taking Medicine B, **130** will experience a minor side effect.

If you take Medicine A, you would live for **9.25 years (9 years and 3 months)** and then die.

If you take Medicine B, you would live for **10 years** and then die.

Would you prefer Medicine A or Medicine B, or are they the same?

Indicate your choice here. You can only choose one option:

○ **Medicine A** — Live for 9 years and 3 months

○ **Medicine B** — Live for 10 years

○ **Medicine A and Medicine B are the same.**

0% 100%

Repeated section

Remember Medicine A and Medicine B are equally effective.

However, there are some differences between Medicine A and Medicine B.

Medicine A	Medicine B
**No inconvenience**<br><br>You take a tablet only once in your life.<br>You will be given the tablet on the day you come in to see your GP.	**Inconvenience**<br><br>One tablet should be taken once every day, at night with cold water.<br>You need to take this medicine for 10 years.<br>You need to regularly visit a GP to receive a prescription.<br>You need to visit a pharmacy to fill a prescription.
**Severe side effect - 0%**<br><br>No one taking Medicine A will have a severe side effect.	**Severe side effect - 0.7%**<br><br>Among every 1,000 people taking Medicine B, **7** will experience a severe side effect.
**Minor side effect - 0%**<br><br>No one taking Medicine A will have a minor side effect.	**Minor side effect - 13%**<br><br>Among every 1,000 people taking Medicine B, **130** will experience a minor side effect.

If you take Medicine A, you would live for **9 years and 4 months** and then die.

If you take Medicine B, you would live for **10 years** and then die.

Would you prefer Medicine A or Medicine B, or are they the same?

Indicate your choice here. You can only choose one option:

○ **Medicine A** Live for 9 years 4 months

○ **Medicine B** Live for 10 years

○ **Medicine A and Medicine B are the same.**

0% 100%

Repeated section

Remember Medicine A and Medicine B are equally effective.

However, there are some differences between Medicine A and Medicine B.

Medicine A	Medicine B
**No inconvenience**<br><br>You take a tablet only once in your life.<br>You will be given the tablet on the day you come in to see your GP.	**Inconvenience**<br><br>One tablet should be taken once every day, at night with cold water.<br>You need to take this medicine for 10 years.<br>You need to regularly visit a GP to receive a prescription.<br>You need to visit a pharmacy to fill a prescription.
**Severe side effect - 0%**<br><br>No one taking Medicine A will have a severe side effect.	**Severe side effect - 0.7%**<br><br>Among every 1,000 people taking Medicine B, **7** will experience a severe side effect.
**Minor side effect - 0%**<br><br>No one taking Medicine A will have a minor side effect.	**Minor side effect - 13%**<br><br>Among every 1,000 people taking Medicine B, **130** will experience a minor side effect.

If you take Medicine A, you would live for **9 years and 5 months** and then die.

If you take Medicine B, you would live for **10 years** and then die.

Would you prefer Medicine A or Medicine B, or are they the same?

Indicate your choice here. You can only choose one option:

○ **Medicine A** Live for 9 years 5 months

○ **Medicine B** Live for 10 years

○ **Medicine A and Medicine B are the same.**

0% 100%

Repeated section

Remember Medicine A and Medicine B are equally effective.

However, there are some differences between Medicine A and Medicine B.

Medicine A	Medicine B
**No inconvenience**<br><br>You take a tablet only once in your life.<br>You will be given the tablet on the day you come in to see your GP.	**Inconvenience**<br><br>One tablet should be taken once every day, at night with cold water.<br>You need to take this medicine for 10 years.<br>You need to regularly visit a GP to receive a prescription.<br>You need to visit a pharmacy to fill a prescription.
**Severe side effect - 0%**<br><br>No one taking Medicine A will have a severe side effect.	**Severe side effect - 0.7%**<br><br>Among every 1,000 people taking Medicine B, **7** will experience a severe side effect.
**Minor side effect - 0%**<br><br>No one taking Medicine A will have a minor side effect.	**Minor side effect - 13%**<br><br>Among every 1,000 people taking Medicine B, **130** will experience a minor side effect.

If you take Medicine A, you would live for **9.5 years** (9 years and 6 months) and then die.

If you take Medicine B, you would live for **10 years** and then die.

Would you prefer Medicine A or Medicine B, or are they the same?

Indicate your choice here. You can only choose one option:

○ **Medicine A** — Live for 9.5 years

○ **Medicine B** — Live for 10 years

○ **Medicine A and Medicine B are the same.**

0% 100%

Repeated section

Remember Medicine A and Medicine B are equally effective.

However, there are some differences between Medicine A and Medicine B.

Medicine A	Medicine B
**No inconvenience**<br><br>You take a tablet only once in your life.<br>You will be given the tablet on the day you come in to see your GP.	**Inconvenience**<br><br>One tablet should be taken once every day, at night with cold water.<br>You need to take this medicine for 10 years.<br>You need to regularly visit a GP to receive a prescription.<br>You need to visit a pharmacy to fill a prescription.
**Severe side effect - 0%**<br><br>No one taking Medicine A will have a severe side effect.	**Severe side effect - 0.7%**<br><br>Among every 1,000 people taking Medicine B, **7** will experience a severe side effect.
**Minor side effect - 0%**<br><br>No one taking Medicine A will have a minor side effect.	**Minor side effect - 13%**<br><br>Among every 1,000 people taking Medicine B, **130** will experience a minor side effect.

If you take Medicine A, you would live for **5 months less than 10 years** and then die.

If you take Medicine B, you would live for **10 years** and then die.

Would you prefer Medicine A or Medicine B, or are they the same?

Indicate your choice here. You can only choose one option:

◯ **Medicine A**

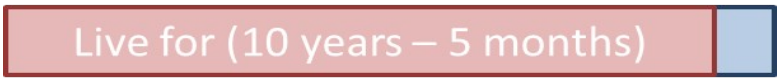

◯ **Medicine B**

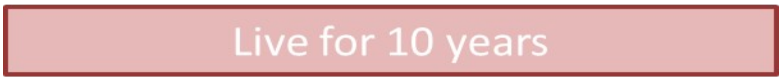

◯ **Medicine A and Medicine B are the same.**

0% 100%

Repeated section

Remember Medicine A and Medicine B are equally effective.

However, there are some differences between Medicine A and Medicine B.

Medicine A	Medicine B
**No inconvenience**<br><br>You take a tablet only once in your life.<br>You will be given the tablet on the day you come in to see your GP.	**Inconvenience**<br><br>One tablet should be taken once every day, at night with cold water.<br>You need to take this medicine for 10 years.<br>You need to regularly visit a GP to receive a prescription.<br>You need to visit a pharmacy to fill a prescription.
**Severe side effect - 0%**<br><br>No one taking Medicine A will have a severe side effect.	**Severe side effect - 0.7%**<br><br>Among every 1,000 people taking Medicine B, **7** will experience a severe side effect.
**Minor side effect - 0%**<br><br>No one taking Medicine A will have a minor side effect.	**Minor side effect - 13%**<br><br>Among every 1,000 people taking Medicine B, **130** will experience a minor side effect.

If you take Medicine A, you would live for **4 months less than 10 years** and then die.

If you take Medicine B, you would live for **10 years** and then die.

Would you prefer Medicine A or Medicine B, or are they the same?

Indicate your choice here. You can only choose one option:

◯ **Medicine A** Live for (10 years – 4 months)

◯ **Medicine B** Live for 10 years

◯ **Medicine A and Medicine B are the same.**

0% 100%

Repeated section

Remember Medicine A and Medicine B are equally effective.

However, there are some differences between Medicine A and Medicine B.

Medicine A	Medicine B
**No inconvenience**<br><br>You take a tablet only once in your life.<br>You will be given the tablet on the day you come in to see your GP.	**Inconvenience**<br><br>One tablet should be taken once every day, at night with cold water.<br>You need to take this medicine for 10 years.<br>You need to regularly visit a GP to receive a prescription.<br>You need to visit a pharmacy to fill a prescription.
**Severe side effect - 0%**<br><br>No one taking Medicine A will have a severe side effect.	**Severe side effect - 0.7%**<br><br>Among every 1,000 people taking Medicine B, **7** will experience a severe side effect.
**Minor side effect - 0%**<br><br>No one taking Medicine A will have a minor side effect.	**Minor side effect - 13%**<br><br>Among every 1,000 people taking Medicine B, **130** will experience a minor side effect.

If you take Medicine A, you would live for **3 months less than 10 years** and then die.

If you take Medicine B, you would live for **10 years** and then die.

Would you prefer Medicine A or Medicine B, or are they the same?

Indicate your choice here. You can only choose one option:

○ **Medicine A** Live for (10 years – 3 months)

○ **Medicine B** Live for 10 years

○ **Medicine A and Medicine B are the same.**

0% 100%

Repeated section

Remember Medicine A and Medicine B are equally effective.

However, there are some differences between Medicine A and Medicine B.

Medicine A	Medicine B
**No inconvenience**<br><br>You take a tablet only once in your life.<br>You will be given the tablet on the day you come in to see your GP.	**Inconvenience**<br><br>One tablet should be taken once every day, at night with cold water.<br>You need to take this medicine for 10 years.<br>You need to regularly visit a GP to receive a prescription.<br>You need to visit a pharmacy to fill a prescription.
**Severe side effect - 0%**<br><br>No one taking Medicine A will have a severe side effect.	**Severe side effect - 0.7%**<br><br>Among every 1,000 people taking Medicine B, **7** will experience a severe side effect.
**Minor side effect - 0%**<br><br>No one taking Medicine A will have a minor side effect.	**Minor side effect - 13%**<br><br>Among every 1,000 people taking Medicine B, **130** will experience a minor side effect.

If you take Medicine A, you would live for **2 months less than 10 years** and then die.

If you take Medicine B, you would live for **10 years** and then die.

Would you prefer Medicine A or Medicine B, or are they the same?

Indicate your choice here. You can only choose one option:

◯ **Medicine A** Live for (10 years – 2 months)

◯ **Medicine B** Live for 10 years

◯ **Medicine A and Medicine B are the same.**

0% 100%

Repeated section

Remember Medicine A and Medicine B are equally effective.

However, there are some differences between Medicine A and Medicine B.

Medicine A	Medicine B
**No inconvenience**<br><br>You take a tablet only once in your life.<br>You will be given the tablet on the day you come in to see your GP.	**Inconvenience**<br><br>One tablet should be taken once every day, at night with cold water.<br>You need to take this medicine for 10 years.<br>You need to regularly visit a GP to receive a prescription.<br>You need to visit a pharmacy to fill a prescription.
**Severe side effect - 0%**<br><br>No one taking Medicine A will have a severe side effect.	**Severe side effect - 0.7%**<br><br>Among every 1,000 people taking Medicine B, **7** will experience a severe side effect.
**Minor side effect - 0%**<br><br>No one taking Medicine A will have a minor side effect.	**Minor side effect - 13%**<br><br>Among every 1,000 people taking Medicine B, **130** will experience a minor side effect.

If you take Medicine A, you would live for **1 month less than 10 years** and then die.

If you take Medicine B, you would live for **10 years** and then die.

Would you prefer Medicine A or Medicine B, or are they the same?

Indicate your choice here. You can only choose one option:

- ○ **Medicine A** — Live for (10 years – 1 month)
- ○ **Medicine B** — Live for 10 years
- ○ **Medicine A and Medicine B are the same.**

0% 100%

Repeated section

Remember Medicine A and Medicine B are equally effective.

However, there are some differences between Medicine A and Medicine B.

Medicine A	Medicine B
**No inconvenience**<br><br>You take a tablet only once in your life.<br>You will be given the tablet on the day you come in to see your GP.	**Inconvenience**<br><br>One tablet should be taken once every day, at night with cold water.<br>You need to take this medicine for 10 years.<br>You need to regularly visit a GP to receive a prescription.<br>You need to visit a pharmacy to fill a prescription.
**Severe side effect - 0%**<br><br>No one taking Medicine A will have a severe side effect.	**Severe side effect - 0.7%**<br><br>Among every 1,000 people taking Medicine B, **7** will experience a severe side effect.
**Minor side effect - 0%**<br><br>No one taking Medicine A will have a minor side effect.	**Minor side effect - 13%**<br><br>Among every 1,000 people taking Medicine B, **130** will experience a minor side effect.

If you take Medicine A, you would live for **3 weeks less than 10 years** and then die.

If you take Medicine B, you would live for **10 years** and then die.

Would you prefer Medicine A or Medicine B, or are they the same?

Indicate your choice here. You can only choose one option:

◯ **Medicine A** Live for (10 years – 3 weeks)

◯ **Medicine B** Live for 10 years

◯ **Medicine A and Medicine B are the same.**

0% 100%

Repeated section

Remember Medicine A and Medicine B are equally effective.

However, there are some differences between Medicine A and Medicine B.

Medicine A	Medicine B
**No inconvenience**<br><br>You take a tablet only once in your life.<br>You will be given the tablet on the day you come in to see your GP.	**Inconvenience**<br><br>One tablet should be taken once every day, at night with cold water.<br>You need to take this medicine for 10 years.<br>You need to regularly visit a GP to receive a prescription.<br>You need to visit a pharmacy to fill a prescription.
**Severe side effect - 0%**<br><br>No one taking Medicine A will have a severe side effect.	**Severe side effect - 0.7%**<br><br>Among every 1,000 people taking Medicine B, **7** will experience a severe side effect.
**Minor side effect - 0%**<br><br>No one taking Medicine A will have a minor side effect.	**Minor side effect - 13%**<br><br>Among every 1,000 people taking Medicine B, **130** will experience a minor side effect.

If you take Medicine A, you would live for **2 weeks less than 10 years** and then die.

If you take Medicine B, you would live for **10 years** and then die.

Would you prefer Medicine A or Medicine B, or are they the same?

Indicate your choice here. You can only choose one option:

◯ **Medicine A**

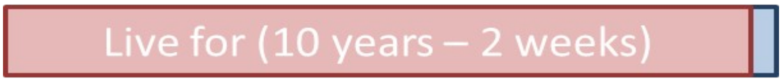

◯ **Medicine B**

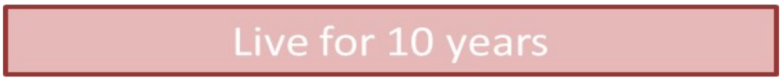

◯ **Medicine A and Medicine B are the same.**

0% 100%

Repeated section

Remember Medicine A and Medicine B are equally effective.

However, there are some differences between Medicine A and Medicine B.

Medicine A	Medicine B
**No inconvenience**<br><br>You take a tablet only once in your life.<br>You will be given the tablet on the day you come in to see your GP.	**Inconvenience**<br><br>One tablet should be taken once every day, at night with cold water.<br>You need to take this medicine for 10 years.<br>You need to regularly visit a GP to receive a prescription.<br>You need to visit a pharmacy to fill a prescription.
**Severe side effect - 0%**<br><br>No one taking Medicine A will have a severe side effect.	**Severe side effect - 0.7%**<br><br>Among every 1,000 people taking Medicine B, **7** will experience a severe side effect.
**Minor side effect - 0%**<br><br>No one taking Medicine A will have a minor side effect.	**Minor side effect - 13%**<br><br>Among every 1,000 people taking Medicine B, **130** will experience a minor side effect.

If you take Medicine A, you would live for **10 days less than 10 years** and then die.

If you take Medicine B, you would live for **10 years** and then die.

Would you prefer Medicine A or Medicine B, or are they the same?

Indicate your choice here. You can only choose one option:

◯ **Medicine A** Live for (10 years – 10 days)

◯ **Medicine B** Live for 10 years

◯ **Medicine A and Medicine B are the same.**

0% 100%

Repeated section

Remember Medicine A and Medicine B are equally effective.

However, there are some differences between Medicine A and Medicine B.

Medicine A	Medicine B
**No inconvenience**<br><br>You take a tablet only once in your life.<br>You will be given the tablet on the day you come in to see your GP.	**Inconvenience**<br><br>One tablet should be taken once every day, at night with cold water.<br>You need to take this medicine for 10 years.<br>You need to regularly visit a GP to receive a prescription.<br>You need to visit a pharmacy to fill a prescription.
**Severe side effect - 0%**<br><br>No one taking Medicine A will have a severe side effect.	**Severe side effect - 0.7%**<br><br>Among every 1,000 people taking Medicine B, **7** will experience a severe side effect.
**Minor side effect - 0%**<br><br>No one taking Medicine A will have a minor side effect.	**Minor side effect - 13%**<br><br>Among every 1,000 people taking Medicine B, **130** will experience a minor side effect.

If you take Medicine A, you would live for **1 week less than 10 years** and then die.

If you take Medicine B, you would live for **10 years** and then die.

Would you prefer Medicine A or Medicine B, or are they the same?

Indicate your choice here. You can only choose one option:

◯ **Medicine A** Live for (10 years – 1 week)

◯ **Medicine B** Live for 10 years

◯ **Medicine A and Medicine B are the same.**

0% 100%

Repeated section

Remember Medicine A and Medicine B are equally effective.

However, there are some differences between Medicine A and Medicine B.

Medicine A	Medicine B
**No inconvenience**<br><br>You take a tablet only once in your life.<br>You will be given the tablet on the day you come in to see your GP.	**Inconvenience**<br><br>One tablet should be taken once every day, at night with cold water.<br>You need to take this medicine for 10 years.<br>You need to regularly visit a GP to receive a prescription.<br>You need to visit a pharmacy to fill a prescription.
**Severe side effect - 0%**<br><br>No one taking Medicine A will have a severe side effect.	**Severe side effect - 0.7%**<br><br>Among every 1,000 people taking Medicine B, **7** will experience a severe side effect.
**Minor side effect - 0%**<br><br>No one taking Medicine A will have a minor side effect.	**Minor side effect - 13%**<br><br>Among every 1,000 people taking Medicine B, **130** will experience a minor side effect.

If you take Medicine A, you would live for **5 days less than 10 years** and then die.

If you take Medicine B, you would live for **10 years** and then die.

Would you prefer Medicine A or Medicine B, or are they the same?

Indicate your choice here. You can only choose one option:

- ◯ **Medicine A** — Live for (10 years – 5 days)
- ◯ **Medicine B** — Live for 10 years
- ◯ **Medicine A and Medicine B are the same.**

0% 100%

Repeated section

Remember Medicine A and Medicine B are equally effective.

However, there are some differences between Medicine A and Medicine B.

Medicine A	Medicine B
**No inconvenience**<br><br>You take a tablet only once in your life.<br>You will be given the tablet on the day you come in to see your GP.	**Inconvenience**<br><br>One tablet should be taken once every day, at night with cold water.<br>You need to take this medicine for 10 years.<br>You need to regularly visit a GP to receive a prescription.<br>You need to visit a pharmacy to fill a prescription.
**Severe side effect - 0%**<br><br>No one taking Medicine A will have a severe side effect.	**Severe side effect - 0.7%**<br><br>Among every 1,000 people taking Medicine B, **7** will experience a severe side effect.
**Minor side effect - 0%**<br><br>No one taking Medicine A will have a minor side effect.	**Minor side effect - 13%**<br><br>Among every 1,000 people taking Medicine B, **130** will experience a minor side effect.

If you take Medicine A, you would live for **3 days less than 10 years** and then die.

If you take Medicine B, you would live for **10 years** and then die.

Would you prefer Medicine A or Medicine B, or are they the same?

Indicate your choice here. You can only choose one option:

◯ **Medicine A** Live for (10 years – 3 days)

◯ **Medicine B** Live for 10 years

◯ **Medicine A and Medicine B are the same.**

0% 100%

Repeated section

Remember Medicine A and Medicine B are equally effective.

However, there are some differences between Medicine A and Medicine B.

Medicine A	Medicine B
**No inconvenience**<br><br>You take a tablet only once in your life.<br>You will be given the tablet on the day you come in to see your GP.	**Inconvenience**<br><br>One tablet should be taken once every day, at night with cold water.<br>You need to take this medicine for 10 years.<br>You need to regularly visit a GP to receive a prescription.<br>You need to visit a pharmacy to fill a prescription.
**Severe side effect - 0%**<br><br>No one taking Medicine A will have a severe side effect.	**Severe side effect - 0.7%**<br><br>Among every 1,000 people taking Medicine B, **7** will experience a severe side effect.
**Minor side effect - 0%**<br><br>No one taking Medicine A will have a minor side effect.	**Minor side effect - 13%**<br><br>Among every 1,000 people taking Medicine B, **130** will experience a minor side effect.

If you take Medicine A, you would live for **1 day less than 10 years** and then die.

If you take Medicine B, you would live for **10 years** and then die.

Would you prefer Medicine A or Medicine B, or are they the same?

Indicate your choice here. You can only choose one option:

◯ **Medicine A** Live for (10 years – 1 day)

◯ **Medicine B** Live for 10 years

◯ **Medicine A and Medicine B are the same.**

0% 100%

Question 5a.

You prefer living for 5 years on Medicine A, to living for 10 years on Medicine B.

This means **living fewer than 5 years on Medicine A** can be the same as living for 10 years on Medicine B.

How many years on Medicine A do you think would be the same as living for 10 years on Medicine B?

(Please give a number in years)

0% 100%

peer review only

Thank you for completing this section and indicating your choices.

Was there anything in this section which you think is unclear or that you didn't understand?

(Please provide details in the comment box below)

0% 100%

For peer review only

Part 3. Questions about you

We understand different people feel differently about different treatments.

In this part of the survey, we ask questions about you to understand if people's age, gender or experiences explain the choices made in Part 2.

0% 100%

For peer review only

We would like to learn about your experience.

Are you currently taking, or have you ever taken any of these medicines?

Atorvastatin
Crestor®
Dorisin
Fluvastatin
Lescol®
Lipitor®
Nandovar
Pravastatin
Simvastatin
Simvador®
Zocor®

- ◯ Yes
- ◯ No

0% 100%

view only

What do you think would be the **benefits** from taking this medicine?

What do you think would be the **harms** from taking this medicine?

Have you experienced any side effects from taking this medicine?

- ◯ Yes
- ◯ No

Do you find it inconvenient to take your statin? Please specify if you think it is inconvenient.

What is your opinion about taking this medicine?

- ◯ I don't mind taking them because I have to take them for a reason.
- ◯ I do not like taking them because they remind me that I am not perfectly healthy.
- ◯ I have no opinion.
- ◯ Other (please specify)

0% 100%

Do you take other medicines prescribed by your doctor?

- ◯ Yes
- ◯ No

In total, how many medicines do you take on a regular basis?
(Please do not include pills you take only once in a while)

- ◯ None
- ◯ One
- ◯ Two to five
- ◯ Six to Ten
- ◯ More than ten

How many different times a day do you regularly take pills?

- ◯ None
- ◯ Once per day
- ◯ 2 times a day
- ◯ 3 times a day
- ◯ More than 3 times a day

Do you find it inconvenient to take your medicine(s)? Please specify if you think it is inconvenient.

What is your opinion about taking this medicine?

- ◯ I don't mind taking them because I have to take them for a reason.
- ◯ I do not like taking them because they remind me that I am not perfectly healthy.
- ◯ I have no opinion.
- ◯ Other (please specify)

0% 100%

For peer review only

We would like to ask questions about you to better understand your choices.

What is your gender?

- ◯ Male
- ◯ Female

Which age category do you fall into?

- ◯ 34 years or younger
- ◯ 35-44 years
- ◯ 45-54 years
- ◯ 55-64 years
- ◯ 65-74 years
- ◯ 75-84 years
- ◯ 85 years or over

Which best reflects your ethnic group?

- ◯ White British/Irish
- ◯ White other
- ◯ Mixed/Multiple ethnic origins
- ◯ Black/African/Caribbean/Black British
- ◯ Asian/Asian British
- ◯ Chinese
- ◯ Other (please specify) ________

0% 100%

What is your occupational status?

- ◯ Employed full-time
- ◯ Employed part-time
- ◯ Self-employed
- ◯ Unemployed
- ◯ Retired
- ◯ Looking after a home/family
- ◯ Student
- ◯ Freelance or temping
- ◯ Long-term sickness

Could you tell us what your occupation is?
(Previous occupation if retired.)

What is the highest level of education you have obtained?
(for example, GCSEs, Degrees, etc.)

What is your religion?

- ◯ No religion
- ◯ Christian
- ◯ Buddhist
- ◯ Jewish
- ◯ Hindu
- ◯ Muslim
- ◯ Sikh
- ◯ Other
- ◯ Prefer not to say

0% 100%

For peer review only

How do you feel about numbers?

This section will ask you some questions to understand how familiar you are with numbers and probabilities.

If you are unsure of the answer, you can leave the response blank.

0% 100%

For peer review only

Question 1

Imagine I flip a coin 1,000 times.

What is your best guess about how many times the coin would fall heads up in 1,000 flips?

◯ Answer: ____

◯ Prefer not to answer

◯ Don't know.

Question 2

If there was a lottery where the chance of winning £10 is 1%, if 1,000 people bought a ticket, how many would you expect to win?

◯ Answer: ____

◯ Prefer not to answer

◯ Don't know.

Question 3

In another lottery, the chance of winning a car is 1 in 1,000.

What percentage of tickets in the lottery will win a car?

◯ Answer: ____ **%**

◯ Prefer not to answer

◯ Don't know

0% 100%

How confident are you that you would answer the questions the same if faced with the situations in real-life?

	Very confident	Confident	Not sure	Not confident	Not confident at all	
Confident	◯	◯	◯	◯	◯	Not confident at all

How easy or difficult did you find making choices between alternatives?

	Very easy	Easy	Average	Somewhat difficult	Very difficult	
Easy	◯	◯	◯	◯	◯	Difficult

How easy or difficult did you find the survey to understand?

	Very easy	Easy	Average	Somewhat difficult	Very difficult	
Easy	◯	◯	◯	◯	◯	Difficult

0% 100%

eview only

Was there anything in the survey that was particularly unclear?

(Please write in the box below)

Is there anything else about the survey we could change to make it easier to understand (for example, phrasing and explanation)?

(Optional) If you have any further comments or suggestions, please write them here.

0% 100%

Thank you for completing this survey

If you have any questions or concerns please contact ResearchNow Support.

If you would like more information about heart disease please visit the NHS Choices website here.

MANCHESTER 1824
The University of Manchester

0% 100%

peer review only

Note:

When respondents take the survey in regular mode this page will not be displayed. Respondents will be redirected to the link below:

https://dkr1.ssisurveys.com/projects/end?rst=1&psid= [Script] &basic=99423

0% 100%

For peer review only

Note:

When respondents take the survey in regular mode this page will not be displayed. Respondents will be redirected to the link below:

https://dkr1.ssisurveys.com/projects/end?rst=2&psid= [Script]

0% 100%

For peer review only

Note:

When respondents take the survey in regular mode this page will not be displayed. Respondents will be redirected to the link below:

https://dkr1.ssisurveys.com/projects/end?rst=3&psid= [Script]

0% 100%

For peer review only

Quantifying the impact of taking medicines for long-term conditions

A survey to understand your views about taking medicines long term for the prevention of osteoporotic fractures

In this survey, we will be asking you about a drug treatment which may be offered to people at an increased risk of having a fracture from osteoporosis.

There are no right or wrong answers, we are just interested in hearing about your views.

For peer review only

Quantifying the impact of taking medicines for long-term conditions

Taking medicines long term for the prevention of osteoporotic fracture

Participant Consent Form

You are being invited to take part in a research study that aims to understand your views about taking medicines long-term. Before you decide, it is important for you to understand why the research is being done and what it will involve.

Please click on the sections below for information on the research.

What is the aim of the research?

Why have I been chosen?

What happens to the data collected?

How is confidentiality maintained?

How long will it take me?

Who will conduct the research?

What would I be asked to do if I took part?

Will the outcomes of the research be published?

What happens if I do not want to take part or if I change my mind?

Who has reviewed the research project?

I understand that my participation in the study is voluntary and that I am free to withdraw at any time without giving a reason by exiting the survey.

I confirm that I have read the information above.

◯ Yes, I would like to take part in the survey.

◯ No, I do not want to participate (exit survey).

0% 100%

What is your gender?

- ◯ Female
- ◯ Male

0% 100%

For peer review only

[QUOTA: QuotaCheck]

Note:

This page only contains quota information and only appears in preview mode.

0% 100%

For peer review only

By selecting one box in each group below, please indicate which statements best describe your own health state today.

Mobility

- ◯ I have no problems in walking about
- ◯ I have some problems in walking about
- ◯ I am confined to bed

Self-Care

- ◯ I have no problems with self-care
- ◯ I have some problems washing or dressing myself
- ◯ I am unable to wash or dress myself

Usual Activities (e.g. work, study, housework, family or leisure activities)

- ◯ I have no problems with performing my usual activities
- ◯ I have some problems with performing my usual activities
- ◯ I am unable to perform my usual activities

Pain/Discomfort

- ◯ I have no pain or discomfort
- ◯ I have moderate pain or discomfort
- ◯ I have extreme pain or discomfort

Anxiety/Depression

- ◯ I am not anxious or depressed
- ◯ I am moderately anxious or depressed
- ◯ I am extremely anxious or depressed

0% 100%

Part 1. Background

This section of the survey explains: osteoporosis (thinning of the bones), how medicine treatments may be used for people at risk of osteoporotic fracture, and what the treatment involves.

We will follow a story of Alex.

Click next to continue.

0% 100%

For peer review only

Part 1. Background

Alex has been to see a doctor who told her that she has weak bones.

The doctor has said Alex needs to start a medicine to prevent broken bones (fractures) because of her weak bones.

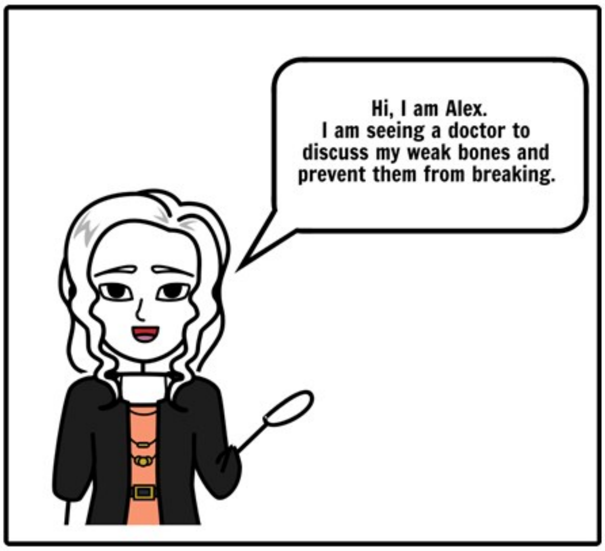

We will take you through Alex's story.

0% 100%

Part 1. Background

What is osteoporosis?

Alex hears about osteoporosis from her GP.

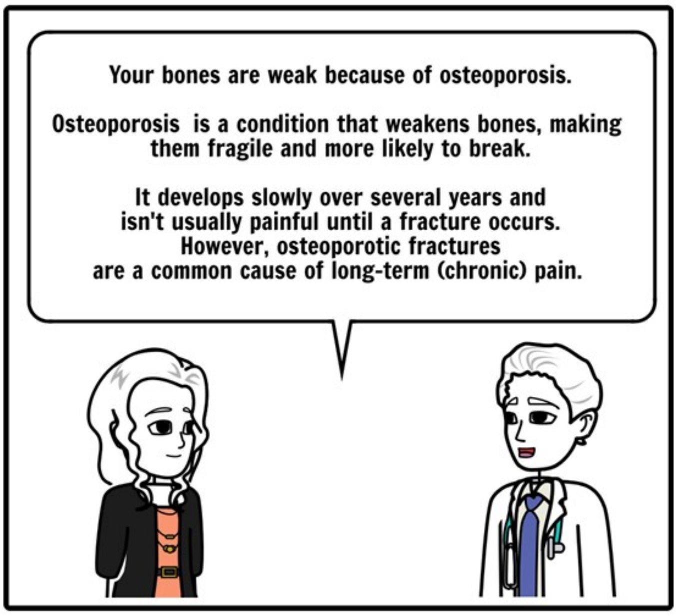

0% 100%

nly

Part 1. Background

Impact of osteoporosis

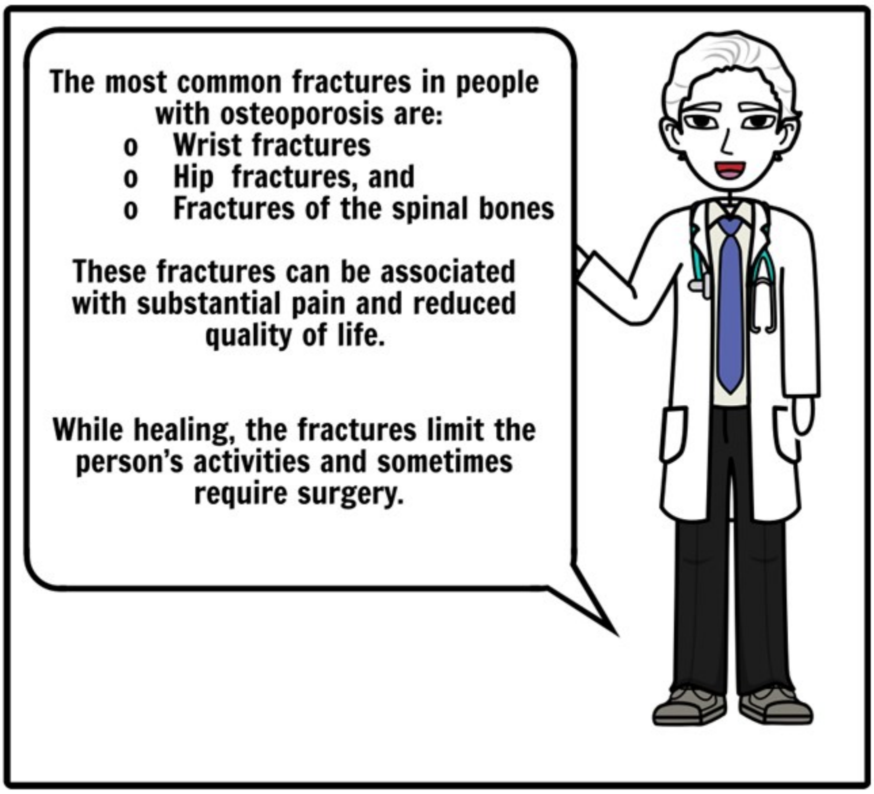

0% 100%

Part 1. Background

Risk of osteoporotic fractures

Her GP explains that Alex has some risk of fracture as her family has a history of hip fracture.

Also, Alex has a low body mass index (BMI). BMI is a measure of body fat.

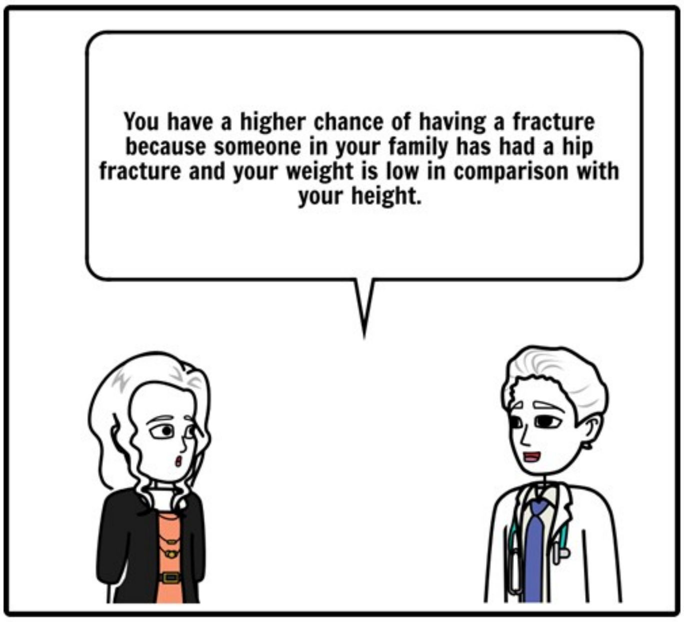

0% 100%

Part 1. Background

What is risk?

- **Risk** is: a term used to explain the chance that something bad might happen.

- **10% risk** means:

  Out of every **1,000** people, with osteoporosis,

  100 people would have a fracture, and **900** people would not.

0% 100%

nly

Part 1. Background

What is a 10% risk?

- This diagram shows a 10% risk of having a fracture because of their osteoporosis.

- The people shaded blue are the ones who have a fracture.

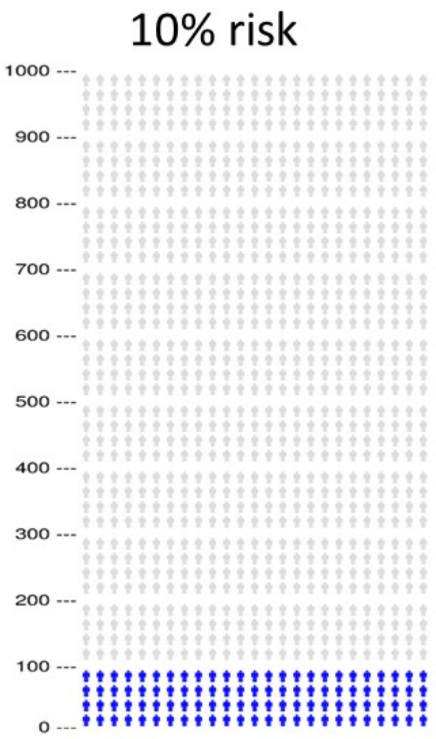

0% 100%

Part 1. Background

Prevention of fracture from osteoporosis

- Alex's doctor can estimate how likely it is that she will experience osteoporotic fracture over the next 5 years.
- The estimate is based on things such as Alex's age and sex, ethnicity, family fracture history, and body mass index.
- The doctor may prescribe certain medicines such as bisphosphonates to help reduce the chance of Alex having a fracture.
- The medicine will prevent some of the people who take it from having a fracture because of osteoporosis.
- Fractures may still happen in some of the people who take the medicine.

0% 100%

Part 1. Background

Prevention of fractures from osteoporosis

The doctor prescribes Alex a medicine to reduce the risk of her having a fracture in the future because of her osteoporosis.

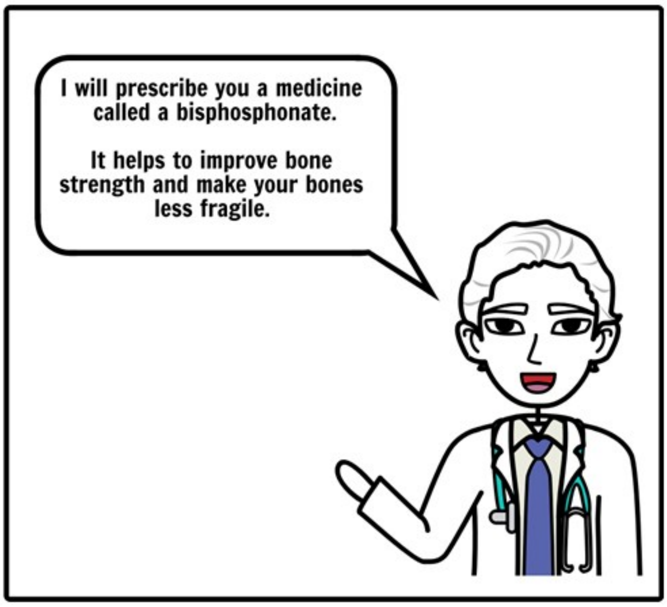

0% 100%

Part 1. Background

Collecting the medicine from the pharmacy

The pharmacist explains to Alex how to take the medicine.

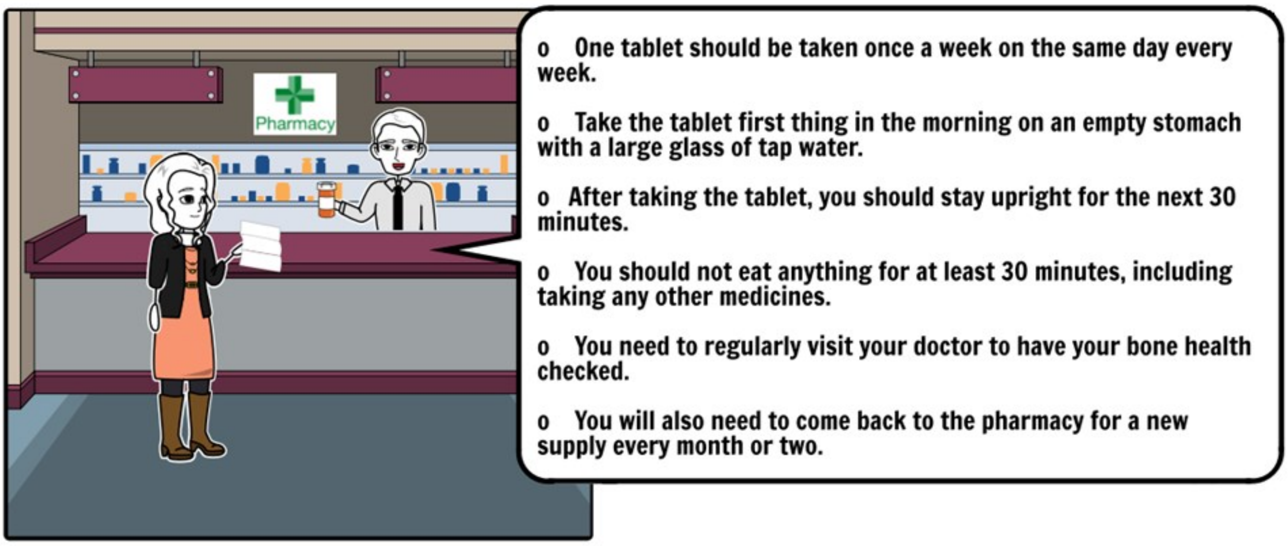

0% 100%

Part 1. Background

Taking the medicine for prevention

Alex goes home with her new tablets.

She then considers whether she wants to take them as the doctor and the pharmacist have advised.

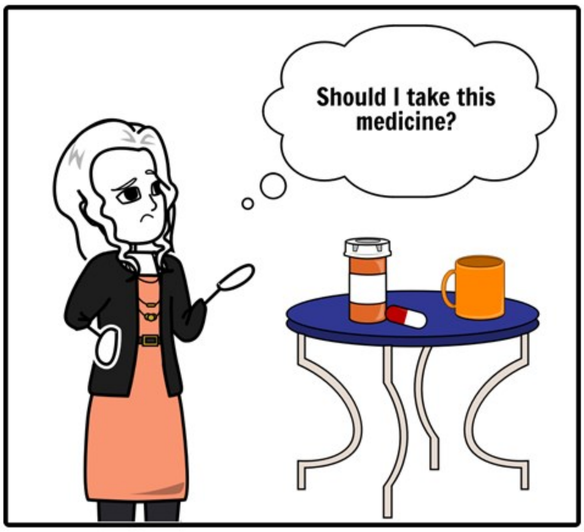

0% 100%

nly

Part 1. Background

Alex may consider *three key things,*
when deciding whether to take the medicine.

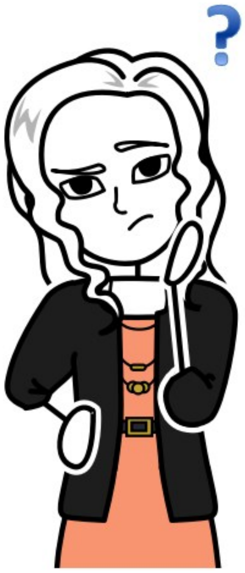

Effectiveness of the medicine

- How good the medicine is in terms of preventing a fracture from osteoporosis

Side effects of the medicine

- Whether there is any potential harm from the medicine

Inconvenience of the medicine

- Whether taking the medicine would fit in her lifestyle or cause her inconvenience

0% 100%

Part 1. Background

How effective is the medicine?

- Some people, but not everyone, who take the medicine will avoid a serious event, such as hip fracture, as a result of taking the tablet.
- The effectiveness of the medicine can be described in terms of a reduction in this risk.

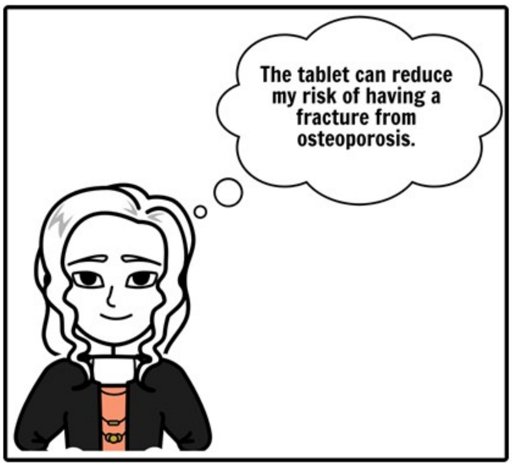

0% 100%

Part 1. Background

What does ‘reduction in risk’ mean?

- A ‘reduction in risk from 10% to 6%’ means:

The medicine will reduce your risk of experiencing a fracture, such as hip fracture, within the next 5 years from 10% to 6%.

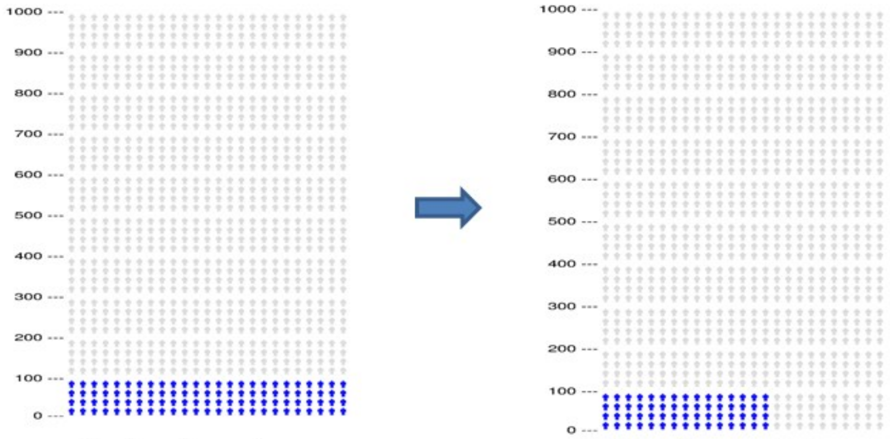

No treatment: 10% risk

Taking the medicine: 6% risk

0% 100%

Part 1. Background

Does the medicine have side effects?

The potential for side effects from the medicine can be described in terms of the **risk of a side effect**.

Alex hears about minor but more common side effects & severe but rarer side effects from her GP.

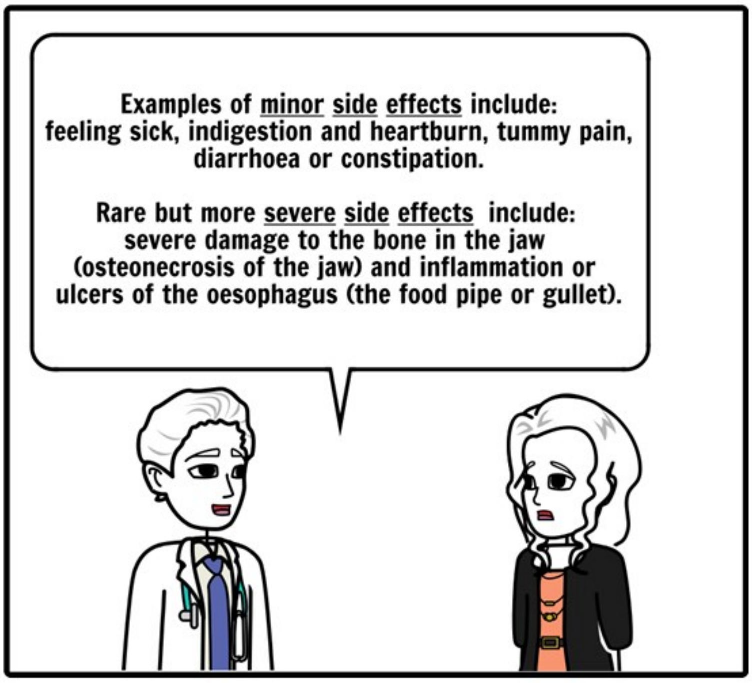

0% 100%

Part 1. Background

Risk of a minor side effect from the medicine

- A 5% risk of a minor side effect means:

Among every **1,000** people taking the medicine,

50 will experience a minor side effect such as either: feeling sick, indigestion, diarrhoea or constipation.

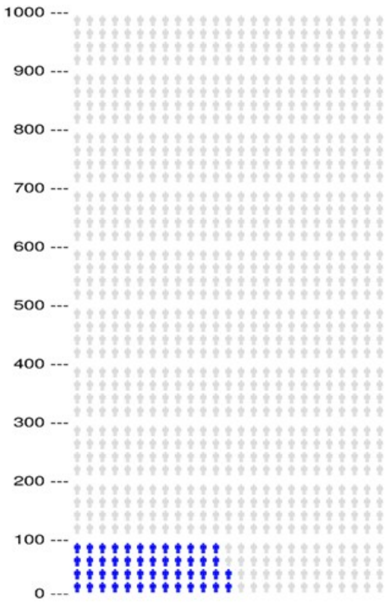

5% risk of a side effect

0% 100%

Part 1. Background

Risk of a severe side effect from the medicine

- A 0.3% risk of severe side effect means:

Among every **1,000** people taking the medicine,

3 will experience a severe side effect such as severe loss or destruction of the bone in the jaw.

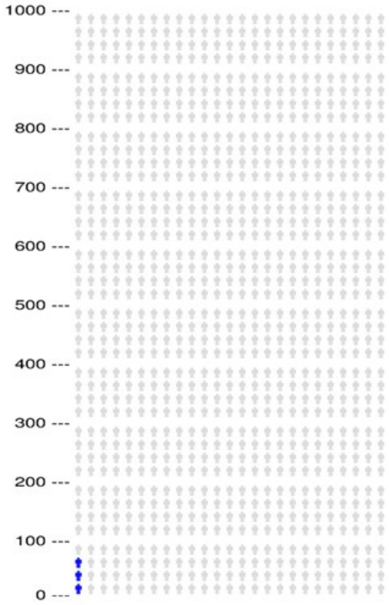

0.3% risk of a side effect

0% 100%

nly

Part 1. Background

How inconvenient is it when taking the medicine?

Alex also thinks about what it means to have to take this medicine for the next 5 years.

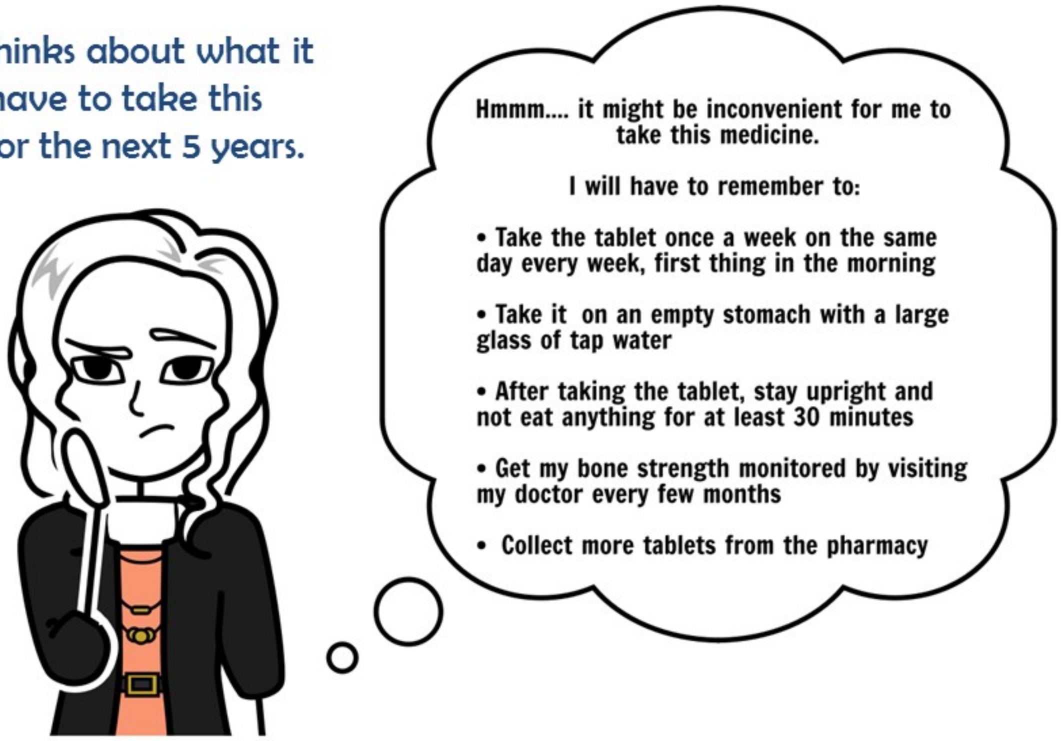

0% 100%

Part 1. Background

In the questions that follow in this survey,
we will ask you about how you think about taking
a medicine to prevent osteoporotic fracture.

0% 100%

In general, how would you feel about taking a medicine for the first time?

- **Happy**
  I trust in my doctor and I am willing to try new things
- **Unsure**
  I'd like to think about it really carefully
- **Unhappy**
  I don't like the idea of trying a new medicine even if the doctor says it will help

0% 100%

peer review only

Part 2: Your views on the inconvenience of taking a medicine to prevent fractures

Now, we will ask you about how you think about the inconvenience of taking a medicine to prevent osteoporotic fractures.

There are four questions.

Each question may look similar but differs in terms of the detail for each of the three features.

0% 100%

For peer review only

Warm up question

You have been told that you need to take a medicine to reduce the risk of fracture.

You can choose between two different medicines. We have called these Medicine A and Medicine B.

Medicine A is a pill that you only need to take once.

Medicine B is a pill that you need to take every week for 5 years.

Medicine A and Medicine B are equally effective in reducing your risk of having a fracture.
Medicine A and Medicine B will reduce your risk of having an osteoporotic fracture within the next 5 years from **10%** to **6%**.

In other words, in a crowd of **1,000** people in the UK, **100** are likely to have a fracture if they do not take the medicine. This is reduced to **60** people out of 1,000 who do take the medicine.
This information is also illustrated in this picture.

Risk of fracture

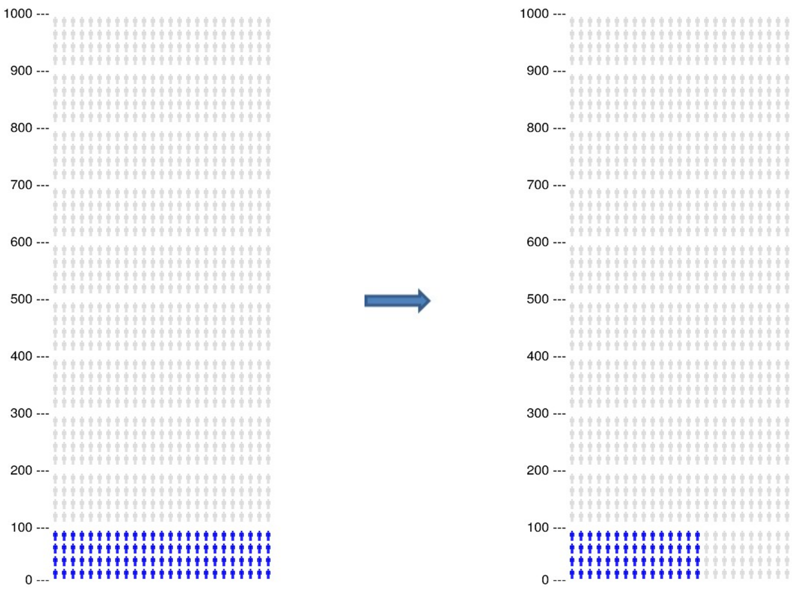

10%
No Treatment

6%
Taking the Medicine

(The figures shaded blue represent people who will have a fracture in the next 5 years)

In this scenario, you will not have any side effects from Medicine A or Medicine B.

Risk of a side effect

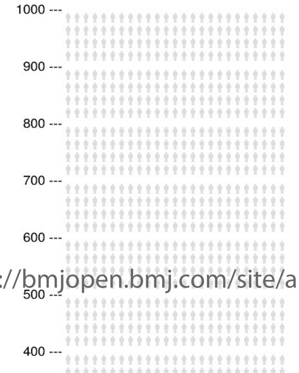

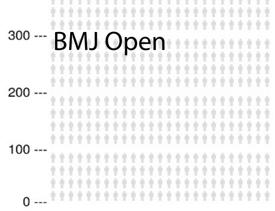

0%
No one will have a side effect.

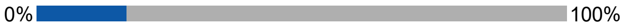

or peer review only

Remember Medicine A and Medicine B are equally effective and have no risks of a side effect.

The only difference between Medicine A and Medicine B is how you take them.

Medicine A	Medicine B
You take a tablet only once in your life.<br>You will be given the tablet on the day you come in to see your GP.<br>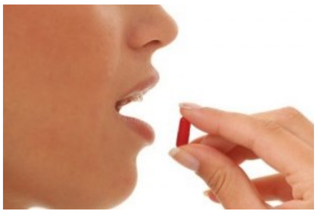	One tablet should be taken once a week on the same day every week.<br>You need to take this medicine for 5 years.<br><br>You need to take the tablet first thing in the morning on an empty stomach with a large glass of tap water.<br>After taking the tablet, you should stay upright for the next 30 minutes.<br>You should not eat anything for at least 30 minutes, including taking any other medicines.<br><br>You need to regularly visit your doctor to have your bone health checked.<br>You need to regularly visit a pharmacy for a new supply.<br>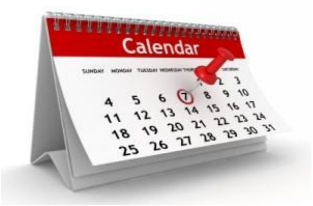 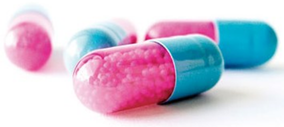

If you take Medicine A, you would live for **1 year** and then die.

If you take Medicine B, you would live for **5 years** and then die.

Would you prefer Medicine A or Medicine B, or are they the same?

Indicate your choice here. You can only choose one option:

Live for 1 year

◯ **Medicine A**

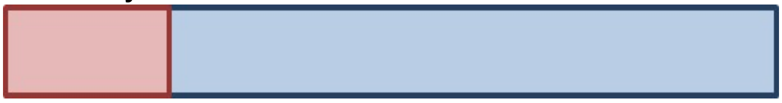

◯ **Medicine B**

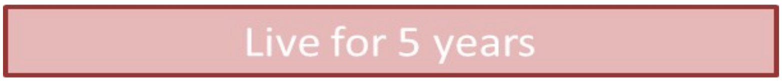

◯ **Medicine A and Medicine B are the same.**

0% 100%

Now we are ready to start the survey questions.

0% 100%

For peer review only

Part 2

Question 1

You have been told that you need to take a medicine to reduce the risk of fracture.

You can choose between two different medicines. We have called these Medicine A and Medicine B.

Medicine A is a pill that you only need to take once.

Medicine B is a pill that you need to take every week for 5 years.

Medicine A and Medicine B are equally effective in reducing your risk of having a fracture.
Medicine A and Medicine B will reduce your risk of experiencing a fracture, such as hip fracture, within the next 5 years from **10%** to **6%**.

In other words, in a crowd of **1,000** people in the UK, **100** are likely to have a fracture if they do not take the medicine. This is reduced to **60** people out of 100 who do take the medicine.
This information is also illustrated in this picture.

Risk of fracture

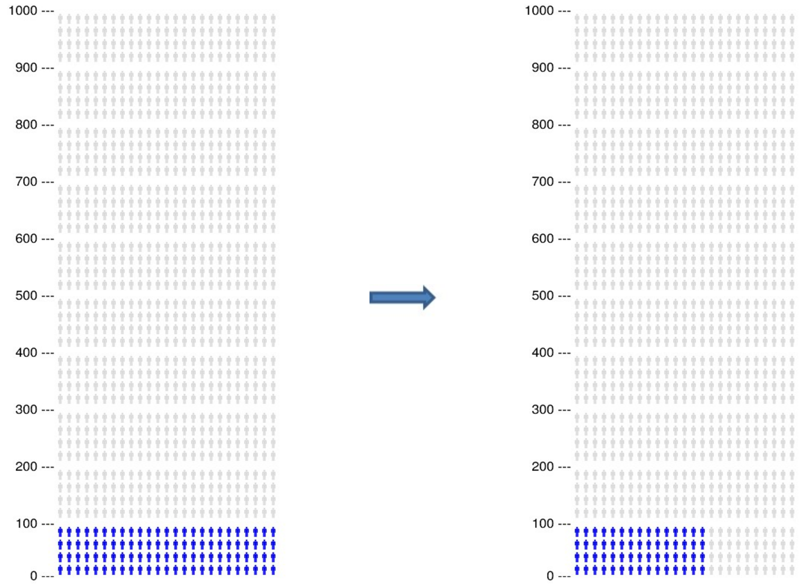

10%
No Treatment

6%
Taking the Medicine

(The figures shaded blue represent people who will have a fracture, such as hip fracture, within the next 5 years)

In this scenario, you will not have any side effects from Medicine A or Medicine B.

Risk of a side effect

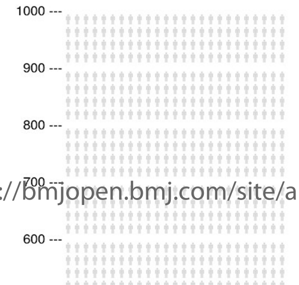

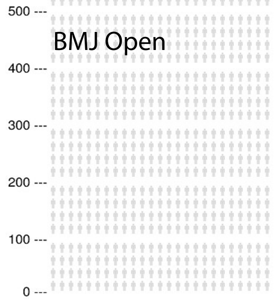

0 %
No one will have a side effect.

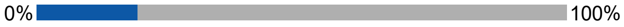

peer review only

Repeated section

Remember Medicine A and Medicine B are equally effective and have no risks of a side effect.

The only difference between Medicine A and Medicine B is how you take them.

Medicine A	Medicine B
You take a tablet only once in your life. You will be given the tablet on the day you come in to see your GP. 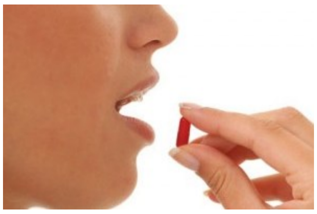	One tablet should be taken once a week on the same day every week. You need to take this medicine for 5 years.<br><br>You need to take the tablet first thing in the morning on an empty stomach with a large glass of tap water. After taking the tablet, you should stay upright for the next 30 minutes. You should not eat anything for at least 30 minutes, including taking any other medicines.<br><br>You need to regularly visit your doctor to have your bone health checked. You need to regularly visit a pharmacy for a new supply. 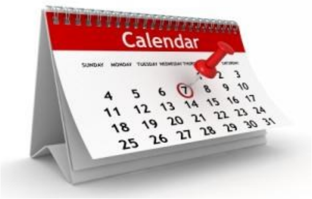 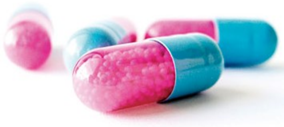

If you take Medicine A, you would live for **2.5 years (2 years and 6 months)** and then die.

If you take Medicine B, you would live for **5 years** and then die.

Would you prefer Medicine A or Medicine B, or are they the same?

Indicate your choice here. You can only choose one option:

○ **Medicine A** Live for 2.5 years

○ **Medicine B** Live for 5 years

○ **The Same - Medicine A and Medicine B are the same.**

0% 100%

For peer review only

Repeated section

Remember Medicine A and Medicine B are equally effective and have no risks of a side effect.

The only difference between Medicine A and Medicine B is how you take them.

**Medicine A**	**Medicine B**
You take a tablet only once in your life. You will be given the tablet on the day you come in to see your GP.<br>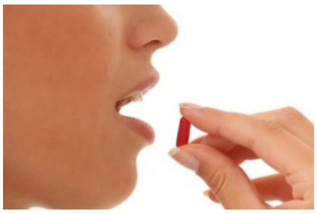	One tablet should be taken once a week on the same day every week.<br>You need to take this medicine for 5 years.<br><br>You need to take the tablet first thing in the morning on an empty stomach with a large glass of tap water.<br>After taking the tablet, you should stay upright for the next 30 minutes.<br>You should not eat anything for at least 30 minutes, including taking any other medicines.<br><br>You need to regularly visit your doctor to have your bone health checked.<br>You need to regularly visit a pharmacy for a new supply.<br>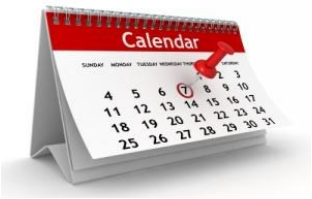 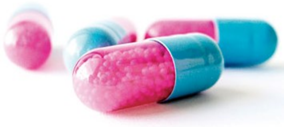

If you take Medicine A, you would live for **3 years** and then die.

If you take Medicine B, you would live for **5 years** and then die.

Would you prefer Medicine A or Medicine B, or are they the same?

Indicate your choice here. You can only choose one option:

◯ **Medicine A** Live for 3 years

◯ **Medicine B** Live for 5 years

◯ **The Same - Medicine A and Medicine B are the same.**

0% 100%

For peer review only

Repeated section

Remember Medicine A and Medicine B are equally effective and have no risks of a side effect.

The only difference between Medicine A and Medicine B is how you take them.

**Medicine A**	**Medicine B**
You take a tablet only once in your life. You will be given the tablet on the day you come in to see your GP. 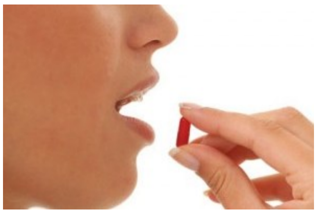	One tablet should be taken once a week on the same day every week. You need to take this medicine for 5 years.<br><br>You need to take the tablet first thing in the morning on an empty stomach with a large glass of tap water. After taking the tablet, you should stay upright for the next 30 minutes. You should not eat anything for at least 30 minutes, including taking any other medicines.<br><br>You need to regularly visit your doctor to have your bone health checked. You need to regularly visit a pharmacy for a new supply. 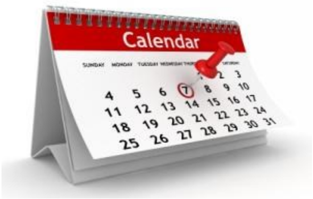 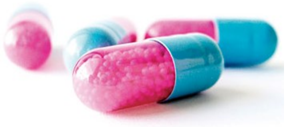

If you take Medicine A, you would live for **3.25 years** (3 years and 3 months) and then die.

If you take Medicine B, you would live for **5 years** and then die.

Would you prefer Medicine A or Medicine B, or are they the same?

Indicate your choice here. You can only choose one option:

◯ **Medicine A** Live for 3 years 3 months

◯ **Medicine B** Live for 5 years

◯ **The Same - Medicine A and Medicine B are the same.**

0% 100%

For peer review only

Repeated section

Remember Medicine A and Medicine B are equally effective and have no risks of a side effect.

The only difference between Medicine A and Medicine B is how you take them.

Medicine A	Medicine B
You take a tablet only once in your life. You will be given the tablet on the day you come in to see your GP. 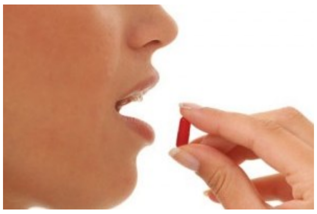	One tablet should be taken once a week on the same day every week. You need to take this medicine for 5 years.<br><br>You need to take the tablet first thing in the morning on an empty stomach with a large glass of tap water. After taking the tablet, you should stay upright for the next 30 minutes. You should not eat anything for at least 30 minutes, including taking any other medicines.<br><br>You need to regularly visit your doctor to have your bone health checked. You need to regularly visit a pharmacy for a new supply. 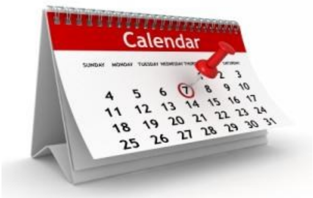 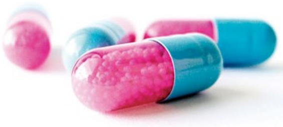

If you take Medicine A, you would live for **3.5 years** (3 years and 6 months) and then die.

If you take Medicine B, you would live for **5 years** and then die.

Would you prefer Medicine A or Medicine B, or are they the same?

Indicate your choice here. You can only choose one option:

○ **Medicine A** Live for 3 years 6 months

○ **Medicine B** Live for 5 years

○ **The Same - Medicine A and Medicine B are the same.**

0% 100%

For peer review only

Repeated section

Remember Medicine A and Medicine B are equally effective and have no risks of a side effect.

The only difference between Medicine A and Medicine B is how you take them.

Medicine A	Medicine B
You take a tablet only once in your life. You will be given the tablet on the day you come in to see your GP. 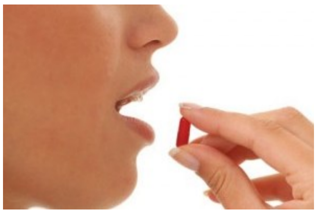	One tablet should be taken once a week on the same day every week. You need to take this medicine for 5 years.<br><br>You need to take the tablet first thing in the morning on an empty stomach with a large glass of tap water. After taking the tablet, you should stay upright for the next 30 minutes. You should not eat anything for at least 30 minutes, including taking any other medicines.<br><br>You need to regularly visit your doctor to have your bone health checked. You need to regularly visit a pharmacy for a new supply. 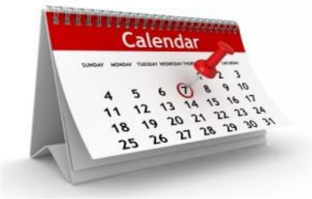 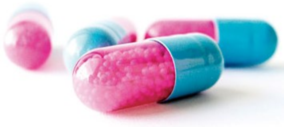

If you take Medicine A, you would live for **3.75 years** (3 years and 9 months) and then die.

If you take Medicine B, you would live for **5 years** and then die.

Would you prefer Medicine A or Medicine B, or are they the same?

Indicate your choice here. You can only choose one option:

○ **Medicine A** Live for 3 years 9 months

○ **Medicine B** Live for 5 years

○ **The Same - Medicine A and Medicine B are the same.**

0% 100%

For peer review only

Repeated section

Remember Medicine A and Medicine B are equally effective and have no risks of a side effect.

The only difference between Medicine A and Medicine B is how you take them.

Medicine A	Medicine B
You take a tablet only once in your life.<br>You will be given the tablet on the day you come in to see your GP.<br>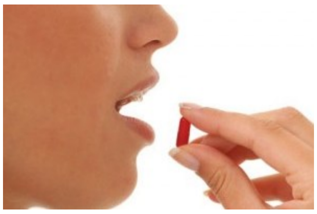	One tablet should be taken once a week on the same day every week.<br>You need to take this medicine for 5 years.<br><br>You need to take the tablet first thing in the morning on an empty stomach with a large glass of tap water.<br>After taking the tablet, you should stay upright for the next 30 minutes.<br>You should not eat anything for at least 30 minutes, including taking any other medicines.<br><br>You need to regularly visit your doctor to have your bone health checked.<br>You need to regularly visit a pharmacy for a new supply.<br>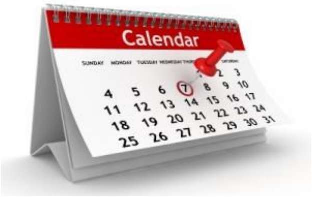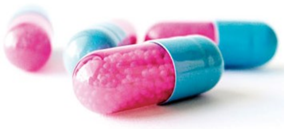

If you take Medicine A, you would live for **3 years and 10 months** and then die.

If you take Medicine B, you would live for **5 years** and then die.

Would you prefer Medicine A or Medicine B, or are they the same?

Indicate your choice here. You can only choose one option:

◯ **Medicine A** Live for 3 years 10 months

◯ **Medicine B** Live for 5 years

◯ **The Same - Medicine A and Medicine B are the same.**

0% 100%

For peer review only

Repeated section

Remember Medicine A and Medicine B are equally effective and have no risks of a side effect.

The only difference between Medicine A and Medicine B is how you take them.

Medicine A	Medicine B
You take a tablet only once in your life. You will be given the tablet on the day you come in to see your GP. 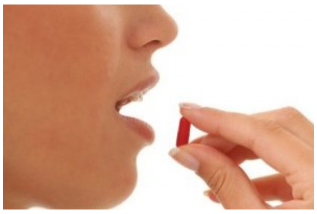	One tablet should be taken once a week on the same day every week. You need to take this medicine for 5 years.<br><br>You need to take the tablet first thing in the morning on an empty stomach with a large glass of tap water. After taking the tablet, you should stay upright for the next 30 minutes. You should not eat anything for at least 30 minutes, including taking any other medicines.<br><br>You need to regularly visit your doctor to have your bone health checked. You need to regularly visit a pharmacy for a new supply. 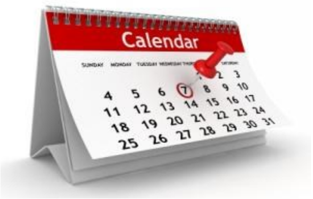 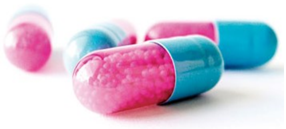

If you take Medicine A, you would live for **4 years** and then die.

If you take Medicine B, you would live for **5 years** and then die.

Would you prefer Medicine A or Medicine B, or are they the same?

Indicate your choice here. You can only choose one option:

◯ **Medicine A**

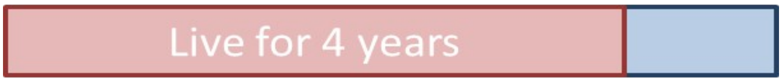

◯ **Medicine B**

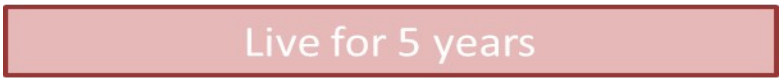

◯ **The Same - Medicine A and Medicine B are the same.**

For peer review only

Repeated section

Remember Medicine A and Medicine B are equally effective and have no risks of a side effect.

The only difference between Medicine A and Medicine B is how you take them.

Medicine A	Medicine B
You take a tablet only once in your life. You will be given the tablet on the day you come in to see your GP. 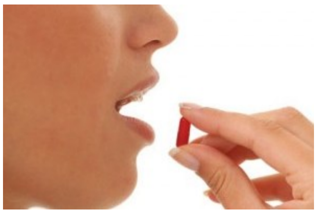	One tablet should be taken once a week on the same day every week. You need to take this medicine for 5 years. You need to take the tablet first thing in the morning on an empty stomach with a large glass of tap water. After taking the tablet, you should stay upright for the next 30 minutes. You should not eat anything for at least 30 minutes, including taking any other medicines. You need to regularly visit your doctor to have your bone health checked. You need to regularly visit a pharmacy for a new supply. 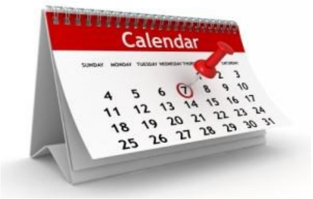 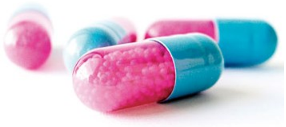

If you take Medicine A, you would live for **4 years and 2 months** and then die.

If you take Medicine B, you would live for **5 years** and then die.

Would you prefer Medicine A or Medicine B, or are they the same?

Indicate your choice here. You can only choose one option:

○ **Medicine A** Live for 4 years 2 months

○ **Medicine B** Live for 5 years

○ **The Same - Medicine A and Medicine B are the same.**

For peer review only

Repeated section

Remember Medicine A and Medicine B are equally effective and have no risks of a side effect.

The only difference between Medicine A and Medicine B is how you take them.

Medicine A	Medicine B
You take a tablet only once in your life. You will be given the tablet on the day you come in to see your GP. 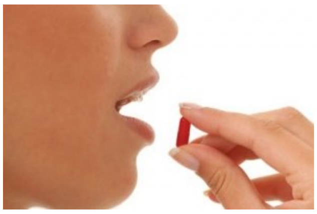	One tablet should be taken once a week on the same day every week. You need to take this medicine for 5 years. You need to take the tablet first thing in the morning on an empty stomach with a large glass of tap water. After taking the tablet, you should stay upright for the next 30 minutes. You should not eat anything for at least 30 minutes, including taking any other medicines. You need to regularly visit your doctor to have your bone health checked. You need to regularly visit a pharmacy for a new supply. 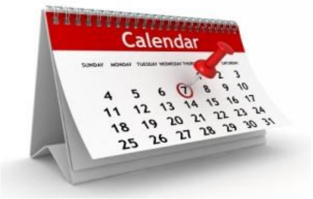 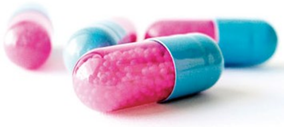

If you take Medicine A, you would live for **4.25 years** (4 years and 3 months) and then die.

If you take Medicine B, you would live for **5 years** and then die.

Would you prefer Medicine A or Medicine B, or are they the same?

Indicate your choice here. You can only choose one option:

◯ **Medicine A** Live for 4 years 3 months

◯ **Medicine B** Live for 5 years

◯ **The Same - Medicine A and Medicine B are the same.**

0% 100%

For peer review only

Repeated section

Remember Medicine A and Medicine B are equally effective and have no risks of a side effect.

The only difference between Medicine A and Medicine B is how you take them.

Medicine A	Medicine B
You take a tablet only once in your life. You will be given the tablet on the day you come in to see your GP. 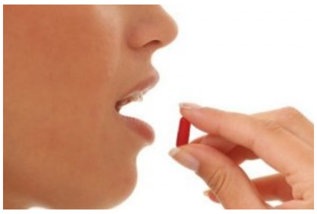	One tablet should be taken once a week on the same day every week. You need to take this medicine for 5 years. You need to take the tablet first thing in the morning on an empty stomach with a large glass of tap water. After taking the tablet, you should stay upright for the next 30 minutes. You should not eat anything for at least 30 minutes, including taking any other medicines. You need to regularly visit your doctor to have your bone health checked. You need to regularly visit a pharmacy for a new supply. 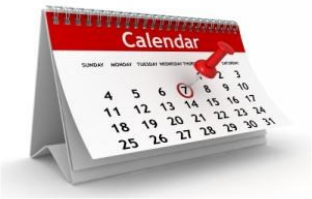 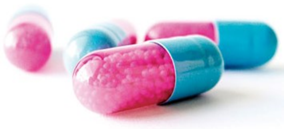

If you take Medicine A, you would live for **4 years 4 months** and then die.

If you take Medicine B, you would live for **5 years** and then die.

Would you prefer Medicine A or Medicine B, or are they the same?

Indicate your choice here. You can only choose one option:

◯ **Medicine A** 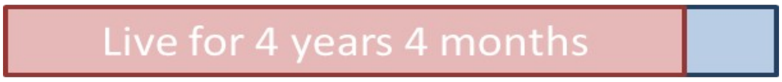

◯ **Medicine B** 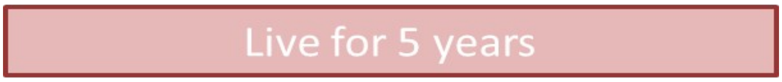

◯ **The Same - Medicine A and Medicine B are the same.**

For peer review only

Repeated section

Remember Medicine A and Medicine B are equally effective and have no risks of a side effect.

The only difference between Medicine A and Medicine B is how you take them.

**Medicine A**	**Medicine B**
You take a tablet only once in your life. You will be given the tablet on the day you come in to see your GP. 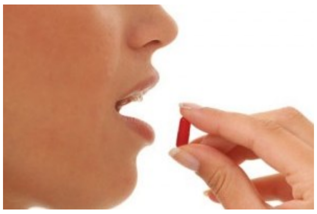	One tablet should be taken once a week on the same day every week. You need to take this medicine for 5 years. You need to take the tablet first thing in the morning on an empty stomach with a large glass of tap water. After taking the tablet, you should stay upright for the next 30 minutes. You should not eat anything for at least 30 minutes, including taking any other medicines. You need to regularly visit your doctor to have your bone health checked. You need to regularly visit a pharmacy for a new supply. 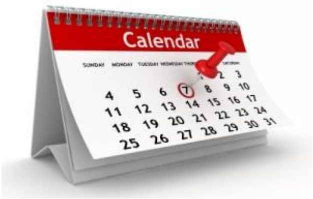 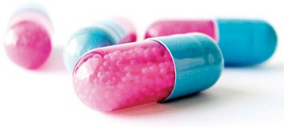

If you take Medicine A, you would live for **4.5 years** (4 years and 6 months) and then die.

If you take Medicine B, you would live for **5 years** and then die.

Would you prefer Medicine A or Medicine B, or are they the same?

Indicate your choice here. You can only choose one option:

◯ **Medicine A** Live for 4 years 6 months

◯ **Medicine B** Live for 5 years

◯ **The Same - Medicine A and Medicine B are the same.**

0% 100%

For peer review only

Repeated section

Remember Medicine A and Medicine B are equally effective and have no risks of a side effect.

The only difference between Medicine A and Medicine B is how you take them.

Medicine A	Medicine B
You take a tablet only once in your life. You will be given the tablet on the day you come in to see your GP. 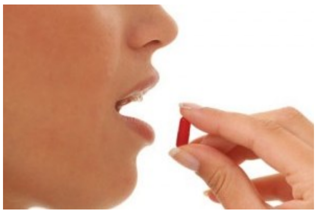	One tablet should be taken once a week on the same day every week.<br>You need to take this medicine for 5 years.<br><br>You need to take the tablet first thing in the morning on an empty stomach with a large glass of tap water.<br>After taking the tablet, you should stay upright for the next 30 minutes.<br>You should not eat anything for at least 30 minutes, including taking any other medicines.<br><br>You need to regularly visit your doctor to have your bone health checked.<br>You need to regularly visit a pharmacy for a new supply.<br>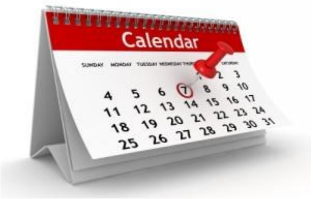 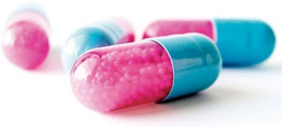

If you take Medicine A, you would live for **4 years and 7 months** and then die.

If you take Medicine B, you would live for **5 years** and then die.

Would you prefer Medicine A or Medicine B, or are they the same?

Indicate your choice here. You can only choose one option:

◯ **Medicine A** Live for 4 years 7 months

◯ **Medicine B** Live for 5 years

◯ **The Same - Medicine A and Medicine B are the same.**

For peer review only

Repeated section

Remember Medicine A and Medicine B are equally effective and have no risks of a side effect.

The only difference between Medicine A and Medicine B is how you take them.

Medicine A	Medicine B
You take a tablet only once in your life.<br>You will be given the tablet on the day you come in to see your GP.<br>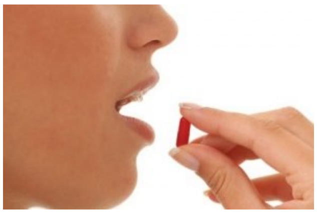	One tablet should be taken once a week on the same day every week.<br>You need to take this medicine for 5 years.<br><br>You need to take the tablet first thing in the morning on an empty stomach with a large glass of tap water.<br>After taking the tablet, you should stay upright for the next 30 minutes.<br>You should not eat anything for at least 30 minutes, including taking any other medicines.<br><br>You need to regularly visit your doctor to have your bone health checked.<br>You need to regularly visit a pharmacy for a new supply.<br>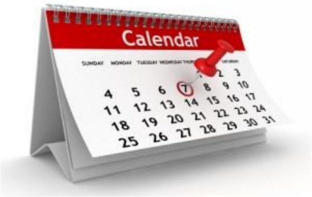 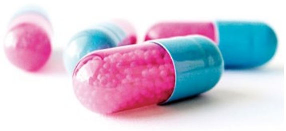

If you take Medicine A, you would live for **4 years and 9 months** and then die.

If you take Medicine B, you would live for **5 years** and then die.

Would you prefer Medicine A or Medicine B, or are they the same?

Indicate your choice here. You can only choose one option:

◯ **Medicine A** Live for 4 years 9 months

◯ **Medicine B** Live for 5 years

◯ **The Same - Medicine A and Medicine B are the same.**

0% 100%

For peer review only

Repeated section

Remember Medicine A and Medicine B are equally effective and have no risks of a side effect.

The only difference between Medicine A and Medicine B is how you take them.

Medicine A	Medicine B
You take a tablet only once in your life. You will be given the tablet on the day you come in to see your GP. 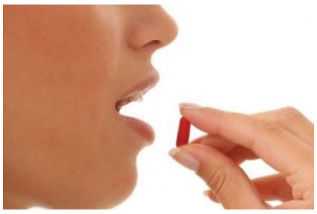	One tablet should be taken once a week on the same day every week.<br>You need to take this medicine for 5 years.<br><br>You need to take the tablet first thing in the morning on an empty stomach with a large glass of tap water.<br>After taking the tablet, you should stay upright for the next 30 minutes.<br>You should not eat anything for at least 30 minutes, including taking any other medicines.<br><br>You need to regularly visit your doctor to have your bone health checked.<br>You need to regularly visit a pharmacy for a new supply.<br>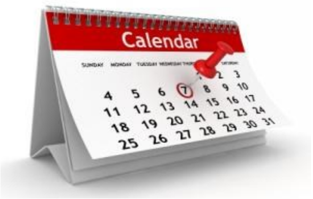 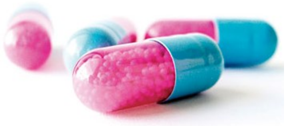

If you take Medicine A, you would live for **2 months less than 5 years** and then die.

If you take Medicine B, you would live for **5 years** and then die.

Would you prefer Medicine A or Medicine B, or are they the same?

Indicate your choice here. You can only choose one option:

◯ **Medicine A** Live for (5 years – 2 months)

◯ **Medicine B** Live for 5 years

◯ **The Same - Medicine A and Medicine B are the same.**

0% 100%

For peer review only

Repeated section

Remember Medicine A and Medicine B are equally effective and have no risks of a side effect.

The only difference between Medicine A and Medicine B is how you take them.

**Medicine A**	**Medicine B**
You take a tablet only once in your life. You will be given the tablet on the day you come in to see your GP. 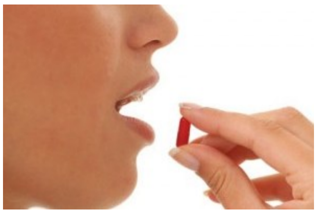	One tablet should be taken once a week on the same day every week.<br>You need to take this medicine for 5 years.<br><br>You need to take the tablet first thing in the morning on an empty stomach with a large glass of tap water.<br>After taking the tablet, you should stay upright for the next 30 minutes.<br>You should not eat anything for at least 30 minutes, including taking any other medicines.<br><br>You need to regularly visit your doctor to have your bone health checked.<br>You need to regularly visit a pharmacy for a new supply.<br>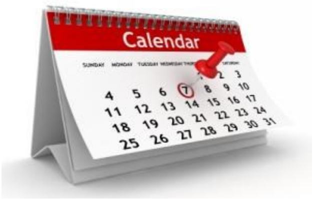 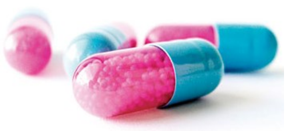

If you take Medicine A, you would live for **1 month less than 5 years** and then die.

If you take Medicine B, you would live for **5 years** and then die.

Would you prefer Medicine A or Medicine B, or are they the same?

Indicate your choice here. You can only choose one option:

○ **Medicine A** Live for (5 years – 1 month)

○ **Medicine B** Live for 5 years

○ **The Same - Medicine A and Medicine B are the same.**

0% 100%

For peer review only

Repeated section

Remember Medicine A and Medicine B are equally effective and have no risks of a side effect.

The only difference between Medicine A and Medicine B is how you take them.

Medicine A	Medicine B
You take a tablet only once in your life. You will be given the tablet on the day you come in to see your GP.<br>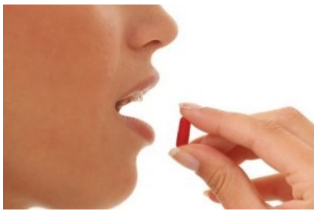	One tablet should be taken once a week on the same day every week.<br>You need to take this medicine for 5 years.<br><br>You need to take the tablet first thing in the morning on an empty stomach with a large glass of tap water.<br>After taking the tablet, you should stay upright for the next 30 minutes.<br>You should not eat anything for at least 30 minutes, including taking any other medicines.<br><br>You need to regularly visit your doctor to have your bone health checked.<br>You need to regularly visit a pharmacy for a new supply.<br>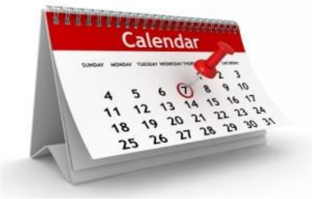 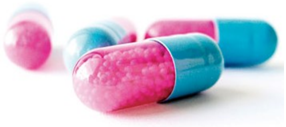

If you take Medicine A, you would live for **2 weeks less than 5 years** and then die.

If you take Medicine B, you would live for **5 years** and then die.

Would you prefer Medicine A or Medicine B, or are they the same?

Indicate your choice here. You can only choose one option:

◯ **Medicine A** Live for (5 years – 2 weeks)

◯ **Medicine B** Live for 5 years

◯ **The Same - Medicine A and Medicine B are the same.**

0% 100%

For peer review only

Repeated section

Remember Medicine A and Medicine B are equally effective and have no risks of a side effect.

The only difference between Medicine A and Medicine B is how you take them.

Medicine A	Medicine B
You take a tablet only once in your life.<br>You will be given the tablet on the day you come in to see your GP.<br>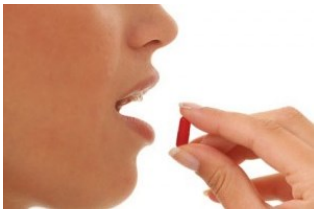	One tablet should be taken once a week on the same day every week.<br>You need to take this medicine for 5 years.<br><br>You need to take the tablet first thing in the morning on an empty stomach with a large glass of tap water.<br>After taking the tablet, you should stay upright for the next 30 minutes.<br>You should not eat anything for at least 30 minutes, including taking any other medicines.<br><br>You need to regularly visit your doctor to have your bone health checked.<br>You need to regularly visit a pharmacy for a new supply.<br>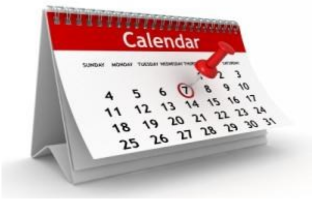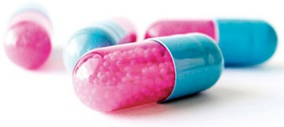

If you take Medicine A, you would live for **1 week less than 5 years** and then die.

If you take Medicine B, you would live for **5 years** and then die.

Would you prefer Medicine A or Medicine B, or are they the same?

Indicate your choice here. You can only choose one option:

○ **Medicine A** Live for (5 years – 1 week)

○ **Medicine B** Live for 5 years

○ **The Same - Medicine A and Medicine B are the same.**

For peer review only

Repeated section

Remember Medicine A and Medicine B are equally effective and have no risks of a side effect.

The only difference between Medicine A and Medicine B is how you take them.

Medicine A	Medicine B
You take a tablet only once in your life. You will be given the tablet on the day you come in to see your GP. 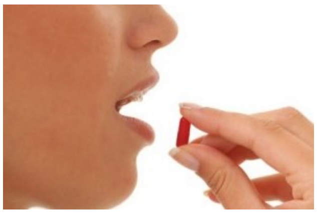	One tablet should be taken once a week on the same day every week. You need to take this medicine for 5 years.<br><br>You need to take the tablet first thing in the morning on an empty stomach with a large glass of tap water. After taking the tablet, you should stay upright for the next 30 minutes. You should not eat anything for at least 30 minutes, including taking any other medicines.<br><br>You need to regularly visit your doctor to have your bone health checked. You need to regularly visit a pharmacy for a new supply.<br>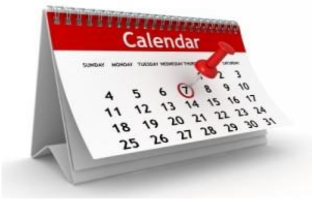 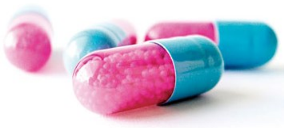

If you take Medicine A, you would live for **5 days less than 5 years** and then die.

If you take Medicine B, you would live for **5 years** and then die.

Would you prefer Medicine A or Medicine B, or are they the same?

Indicate your choice here. You can only choose one option:

- ◯ **Medicine A** — Live for (5 years – 5 days)
- ◯ **Medicine B** — Live for 5 years
- ◯ **The Same - Medicine A and Medicine B are the same.**

0% 100%

For peer review only

Repeated section

Remember Medicine A and Medicine B are equally effective and have no risks of a side effect.

The only difference between Medicine A and Medicine B is how you take them.

Medicine A	Medicine B
You take a tablet only once in your life. You will be given the tablet on the day you come in to see your GP. 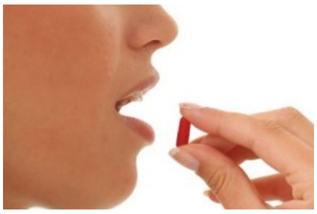	One tablet should be taken once a week on the same day every week. You need to take this medicine for 5 years. You need to take the tablet first thing in the morning on an empty stomach with a large glass of tap water. After taking the tablet, you should stay upright for the next 30 minutes. You should not eat anything for at least 30 minutes, including taking any other medicines. You need to regularly visit your doctor to have your bone health checked. You need to regularly visit a pharmacy for a new supply. 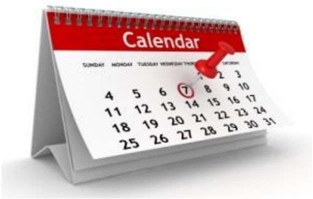 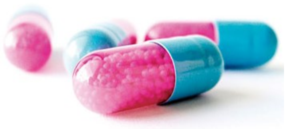

If you take Medicine A, you would live for **3 days less than 5 years** and then die.

If you take Medicine B, you would live for **5 years** and then die.

Would you prefer Medicine A or Medicine B, or are they the same?

Indicate your choice here. You can only choose one option:

◯ **Medicine A** Live for (5 years – 3 days)

◯ **Medicine B** Live for 5 years

◯ **The Same - Medicine A and Medicine B are the same.**

0% 100%

For peer review only

Repeated section

Remember Medicine A and Medicine B are equally effective and have no risks of a side effect.

The only difference between Medicine A and Medicine B is how you take them.

Medicine A	Medicine B
You take a tablet only once in your life. You will be given the tablet on the day you come in to see your GP. 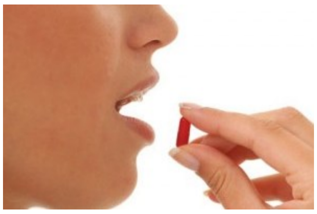	One tablet should be taken once a week on the same day every week. You need to take this medicine for 5 years. You need to take the tablet first thing in the morning on an empty stomach with a large glass of tap water. After taking the tablet, you should stay upright for the next 30 minutes. You should not eat anything for at least 30 minutes, including taking any other medicines. You need to regularly visit your doctor to have your bone health checked. You need to regularly visit a pharmacy for a new supply. 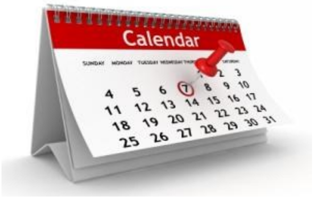 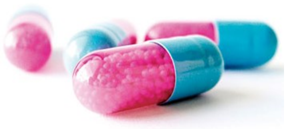

If you take Medicine A, you would live for **1 day less than 5 years** and then die.

If you take Medicine B, you would live for **5 years** and then die.

Would you prefer Medicine A or Medicine B, or are they the same?

Indicate your choice here. You can only choose one option:

◯ **Medicine A** Live for (5 years – 1 day)

◯ **Medicine B** Live for 5 years

◯ **The Same - Medicine A and Medicine B are the same.**

0% 100%

For peer review only

Question 1a.

You prefer living for 2.5 years (2 years and 6 months) on Medicine A, to living for 5 years on Medicine B.

This means **living fewer than 2.5 years on Medicine A** can be the same as living for 5 years on Medicine B.

How many years on Medicine A do you think would be the same as living for 5 years on Medicine B?

(Please give a number in years)

0% 100%

peer review only

Part 2

Question 2

You have been told that you need to take a medicine to reduce the risk of fracture.

You can choose between two different medicines. We have called these Medicine A and Medicine B.

Medicine A is a pill that you only need to take once.

Medicine B is a pill that you need to take every week for 5 years.

Medicine A and Medicine B are equally effective in reducing your risk of having a fracture.
Medicine A and Medicine B will reduce your risk of experiencing a fracture, such as hip fracture, within the next 5 years from **10%** to **6%**.

In other words, in a crowd of **1,000** people in the UK, **100** are likely to have a fracture if they do not take the medicine. This is reduced to **60** people out of 1,000 who do take the medicine.
This information is also illustrated in this picture.

Risk of fracture

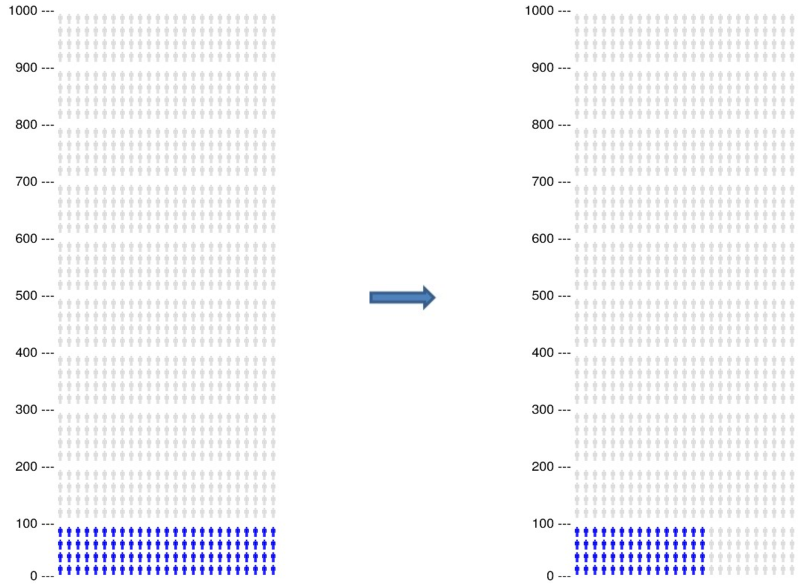

10%
No Treatment

6%
Taking the Medicine

(The figures shaded blue represent people who will have a fracture, such as hip fracture, within the next 5 years)

In this scenario, there is some risk of a minor side effect from Medicine A or Medicine B.

Among every **1,000** people taking Medicine A or Medicine B, **50** people are likely to have a minor side effect such as nausea (feeling sick) and indigestion.

Risk of a minor side effect

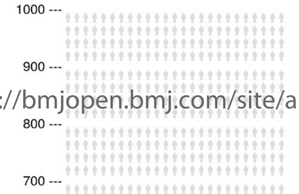

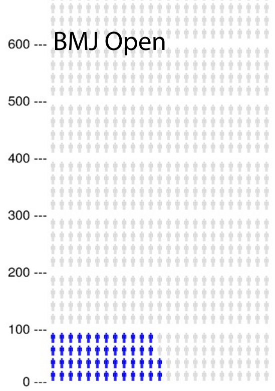

5% of people will have a minor side effect.

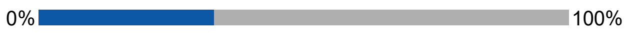

For peer review only

Repeated section

Remember Medicine A and Medicine B are equally effective and have some risk of a minor side effect.

The only difference between Medicine A and Medicine B is how you take them.

Medicine A	Medicine B
You take a tablet only once in your life. You will be given the tablet on the day you come in to see your GP. 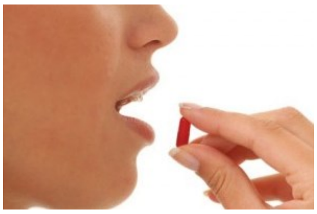	One tablet should be taken once a week on the same day every week. You need to take this medicine for 5 years.<br><br>You need to take the tablet first thing in the morning on an empty stomach with a large glass of tap water. After taking the tablet, you should stay upright for the next 30 minutes. You should not eat anything for at least 30 minutes, including taking any other medicines.<br><br>You need to regularly visit your doctor to have your bone health checked. You need to regularly visit a pharmacy for a new supply. 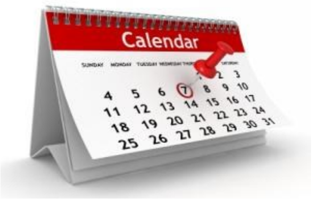 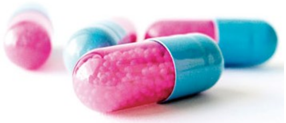

If you take Medicine A, you would live for **2.5 years (2 years and 6 months)** and then die.

If you take Medicine B, you would live for **5 years** and then die.

Would you prefer Medicine A or Medicine B, or are they the same?

Indicate your choice here. You can only choose one option:

◯ **Medicine A** Live for 2.5 years

◯ **Medicine B** Live for 5 years

◯ **The Same - Medicine A and Medicine B are the same.**

0% 100%

For peer review only

Repeated section

Remember Medicine A and Medicine B are equally effective and have some risk of a minor side effect.

The only difference between Medicine A and Medicine B is how you take them.

Medicine A	Medicine B
You take a tablet only once in your life.<br>You will be given the tablet on the day you come in to see your GP.<br>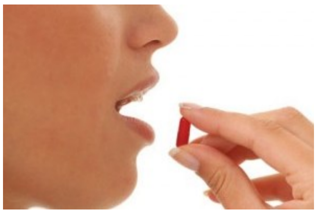	One tablet should be taken once a week on the same day every week.<br>You need to take this medicine for 5 years.<br><br>You need to take the tablet first thing in the morning on an empty stomach with a large glass of tap water.<br>After taking the tablet, you should stay upright for the next 30 minutes.<br>You should not eat anything for at least 30 minutes, including taking any other medicines.<br><br>You need to regularly visit your doctor to have your bone health checked.<br>You need to regularly visit a pharmacy for a new supply.<br>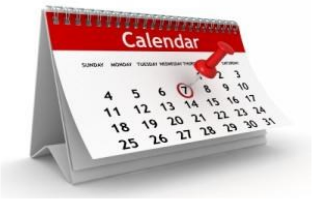 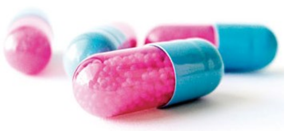

If you take Medicine A, you would live for **3 years** and then die.

If you take Medicine B, you would live for **5 years** and then die.

Would you prefer Medicine A or Medicine B, or are they the same?

Indicate your choice here. You can only choose one option:

◯ **Medicine A** 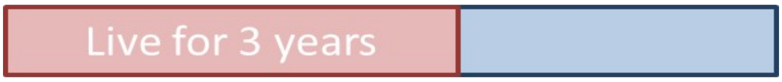

◯ **Medicine B** 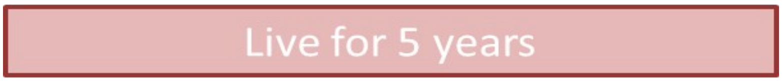

◯ **The Same - Medicine A and Medicine B are the same.**

0% 100%

For peer review only

Repeated section

Remember Medicine A and Medicine B are equally effective and have some risk of a minor side effect.

The only difference between Medicine A and Medicine B is how you take them.

Medicine A	Medicine B
You take a tablet only once in your life.<br>You will be given the tablet on the day you come in to see your GP.<br>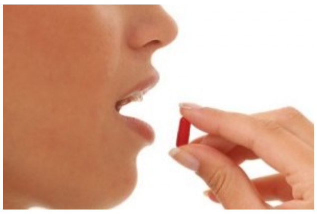	One tablet should be taken once a week on the same day every week.<br>You need to take this medicine for 5 years.<br><br>You need to take the tablet first thing in the morning on an empty stomach with a large glass of tap water.<br>After taking the tablet, you should stay upright for the next 30 minutes.<br>You should not eat anything for at least 30 minutes, including taking any other medicines.<br><br>You need to regularly visit your doctor to have your bone health checked.<br>You need to regularly visit a pharmacy for a new supply.<br>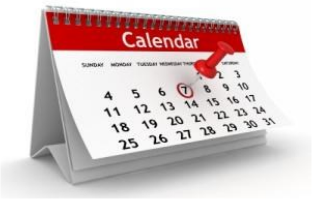 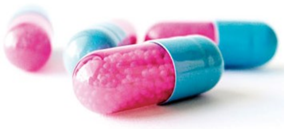

If you take Medicine A, you would live for **3.25 years** (3 years and 3 months) and then die.

If you take Medicine B, you would live for **5 years** and then die.

Would you prefer Medicine A or Medicine B, or are they the same?

Indicate your choice here. You can only choose one option:

◯ **Medicine A** Live for 3 years 3 months

◯ **Medicine B** Live for 5 years

◯ **The Same - Medicine A and Medicine B are the same.**

0% 100%

For peer review only

Repeated section

Remember Medicine A and Medicine B are equally effective and have some risk of a minor side effect.

The only difference between Medicine A and Medicine B is how you take them.

Medicine A	Medicine B
You take a tablet only once in your life. You will be given the tablet on the day you come in to see your GP. 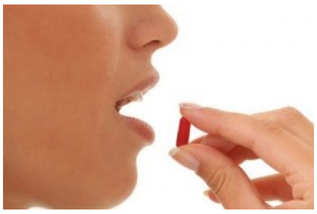	One tablet should be taken once a week on the same day every week.<br>You need to take this medicine for 5 years.<br><br>You need to take the tablet first thing in the morning on an empty stomach with a large glass of tap water.<br>After taking the tablet, you should stay upright for the next 30 minutes.<br>You should not eat anything for at least 30 minutes, including taking any other medicines.<br><br>You need to regularly visit your doctor to have your bone health checked.<br>You need to regularly visit a pharmacy for a new supply. 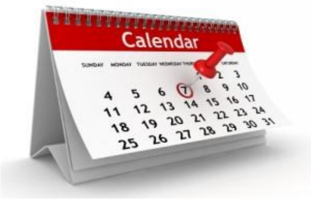 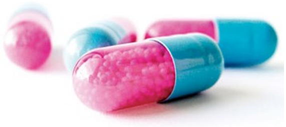

If you take Medicine A, you would live for **3.5 years** (3 years and 6 months) and then die.

If you take Medicine B, you would live for **5 years** and then die.

Would you prefer Medicine A or Medicine B, or are they the same?

Indicate your choice here. You can only choose one option:

○ **Medicine A** Live for 3 years 6 months

○ **Medicine B** Live for 5 years

○ **The Same - Medicine A and Medicine B are the same.**

0% 100%

For peer review only

Repeated section

Remember Medicine A and Medicine B are equally effective and have some risk of a minor side effect.

The only difference between Medicine A and Medicine B is how you take them.

Medicine A	Medicine B
You take a tablet only once in your life. You will be given the tablet on the day you come in to see your GP. 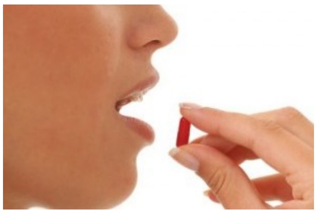	One tablet should be taken once a week on the same day every week.<br>You need to take this medicine for 5 years.<br><br>You need to take the tablet first thing in the morning on an empty stomach with a large glass of tap water.<br>After taking the tablet, you should stay upright for the next 30 minutes.<br>You should not eat anything for at least 30 minutes, including taking any other medicines.<br><br>You need to regularly visit your doctor to have your bone health checked.<br>You need to regularly visit a pharmacy for a new supply.<br>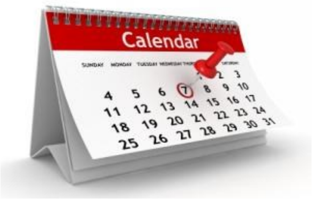 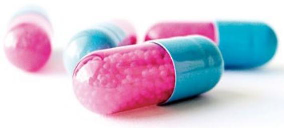

If you take Medicine A, you would live for **3.75 years** (3 years and 9 months) and then die.

If you take Medicine B, you would live for **5 years** and then die.

Would you prefer Medicine A or Medicine B, or are they the same?

Indicate your choice here. You can only choose one option:

○ **Medicine A** Live for 3 years 9 months

○ **Medicine B** Live for 5 years

○ **The Same - Medicine A and Medicine B are the same.**

0% 100%

For peer review only

Repeated section

Remember Medicine A and Medicine B are equally effective and have some risk of a minor side effect.

The only difference between Medicine A and Medicine B is how you take them.

Medicine A	Medicine B
You take a tablet only once in your life. You will be given the tablet on the day you come in to see your GP.<br>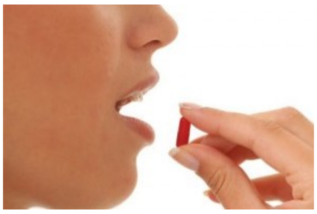	One tablet should be taken once a week on the same day every week.<br>You need to take this medicine for 5 years.<br><br>You need to take the tablet first thing in the morning on an empty stomach with a large glass of tap water.<br>After taking the tablet, you should stay upright for the next 30 minutes.<br>You should not eat anything for at least 30 minutes, including taking any other medicines.<br><br>You need to regularly visit your doctor to have your bone health checked.<br>You need to regularly visit a pharmacy for a new supply.<br>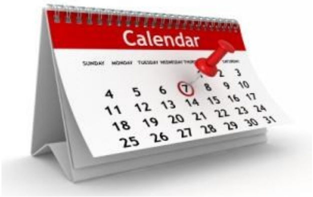 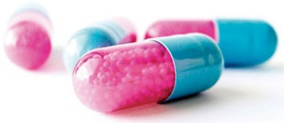

If you take Medicine A, you would live for **3 years and 10 months** and then die.

If you take Medicine B, you would live for **5 years** and then die.

Would you prefer Medicine A or Medicine B, or are they the same?

Indicate your choice here. You can only choose one option:

◯ **Medicine A**

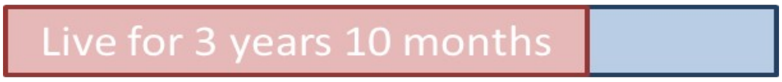

◯ **Medicine B**

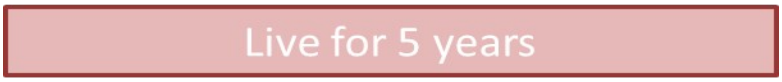

◯ **The Same - Medicine A and Medicine B are the same.**

0% 100%

For peer review only

Repeated section

Remember Medicine A and Medicine B are equally effective and have some risk of a minor side effect.

The only difference between Medicine A and Medicine B is how you take them.

Medicine A	Medicine B
You take a tablet only once in your life.<br>You will be given the tablet on the day you come in to see your GP.<br>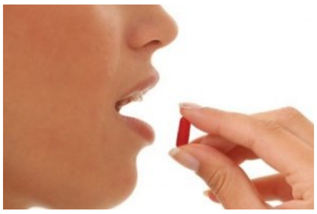	One tablet should be taken once a week on the same day every week.<br>You need to take this medicine for 5 years.<br><br>You need to take the tablet first thing in the morning on an empty stomach with a large glass of tap water.<br>After taking the tablet, you should stay upright for the next 30 minutes.<br>You should not eat anything for at least 30 minutes, including taking any other medicines.<br><br>You need to regularly visit your doctor to have your bone health checked.<br>You need to regularly visit a pharmacy for a new supply.<br>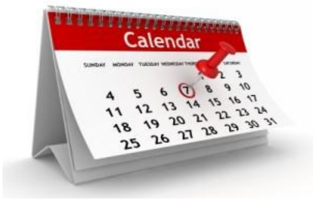 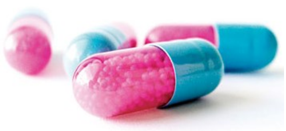

If you take Medicine A, you would live for **4 years** and then die.

If you take Medicine B, you would live for **5 years** and then die.

Would you prefer Medicine A or Medicine B, or are they the same?

Indicate your choice here. You can only choose one option:

○ **Medicine A** — Live for 4 years

○ **Medicine B** — Live for 5 years

○ **The Same - Medicine A and Medicine B are the same.**

0% 100%

For peer review only

Repeated section

Remember Medicine A and Medicine B are equally effective and have some risk of a minor side effect.

The only difference between Medicine A and Medicine B is how you take them.

Medicine A	Medicine B
You take a tablet only once in your life. You will be given the tablet on the day you come in to see your GP. 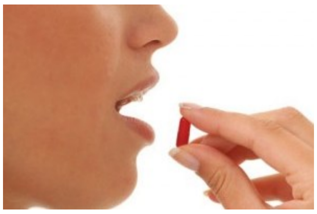	One tablet should be taken once a week on the same day every week. You need to take this medicine for 5 years.<br><br>You need to take the tablet first thing in the morning on an empty stomach with a large glass of tap water. After taking the tablet, you should stay upright for the next 30 minutes. You should not eat anything for at least 30 minutes, including taking any other medicines.<br><br>You need to regularly visit your doctor to have your bone health checked. You need to regularly visit a pharmacy for a new supply. 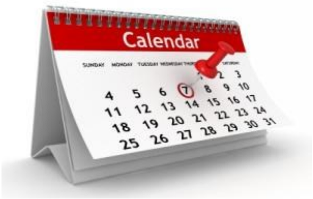 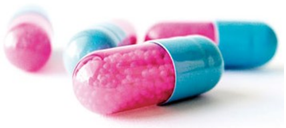

If you take Medicine A, you would live for **4 years and 2 months** and then die.

If you take Medicine B, you would live for **5 years** and then die.

Would you prefer Medicine A or Medicine B, or are they the same?

Indicate your choice here. You can only choose one option:

◯ **Medicine A** Live for 4 years 2 months

◯ **Medicine B** Live for 5 years

◯ **The Same - Medicine A and Medicine B are the same.**

For peer review only

Repeated section

Remember Medicine A and Medicine B are equally effective and have some risk of a minor side effect.

The only difference between Medicine A and Medicine B is how you take them.

Medicine A	Medicine B
You take a tablet only once in your life. You will be given the tablet on the day you come in to see your GP. 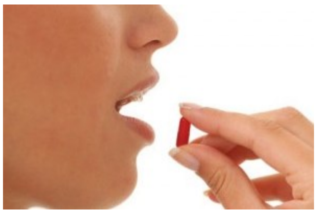	One tablet should be taken once a week on the same day every week.<br>You need to take this medicine for 5 years.<br><br>You need to take the tablet first thing in the morning on an empty stomach with a large glass of tap water.<br>After taking the tablet, you should stay upright for the next 30 minutes.<br>You should not eat anything for at least 30 minutes, including taking any other medicines.<br><br>You need to regularly visit your doctor to have your bone health checked.<br>You need to regularly visit a pharmacy for a new supply.<br>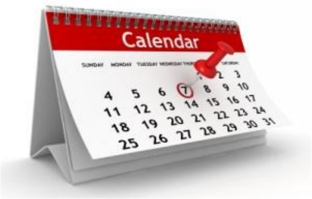 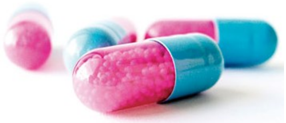

If you take Medicine A, you would live for **4.25 years** (4 years and 3 months) and then die.

If you take Medicine B, you would live for **5 years** and then die.

Would you prefer Medicine A or Medicine B, or are they the same?

Indicate your choice here. You can only choose one option:

◯ **Medicine A**
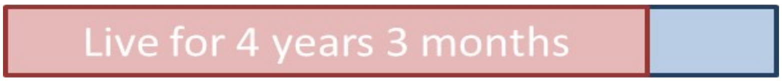

◯ **Medicine B**
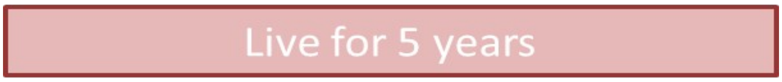

◯ **The Same - Medicine A and Medicine B are the same.**

For peer review only

Repeated section

Remember Medicine A and Medicine B are equally effective and have some risk of a minor side effect.

The only difference between Medicine A and Medicine B is how you take them.

**Medicine A**	**Medicine B**
You take a tablet only once in your life.<br>You will be given the tablet on the day you come in to see your GP.<br>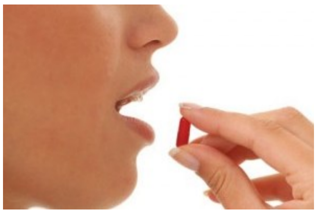	One tablet should be taken once a week on the same day every week.<br>You need to take this medicine for 5 years.<br><br>You need to take the tablet first thing in the morning on an empty stomach with a large glass of tap water.<br>After taking the tablet, you should stay upright for the next 30 minutes.<br>You should not eat anything for at least 30 minutes, including taking any other medicines.<br><br>You need to regularly visit your doctor to have your bone health checked.<br>You need to regularly visit a pharmacy for a new supply.<br>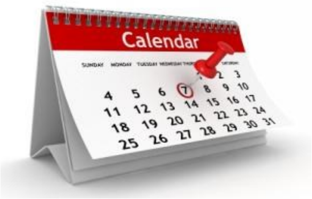 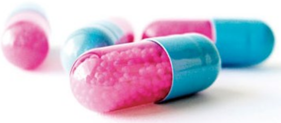

If you take Medicine A, you would live for **4 years 4 months** and then die.

If you take Medicine B, you would live for **5 years** and then die.

Would you prefer Medicine A or Medicine B, or are they the same?

Indicate your choice here. You can only choose one option:

- ◯ **Medicine A** — Live for 4 years 4 months
- ◯ **Medicine B** — Live for 5 years
- ◯ **The Same - Medicine A and Medicine B are the same.**

For peer review only

Repeated section

Remember Medicine A and Medicine B are equally effective and have no risks of a minor side effect.

The only difference between Medicine A and Medicine B is how you take them.

Medicine A	Medicine B
You take a tablet only once in your life. You will be given the tablet on the day you come in to see your GP. 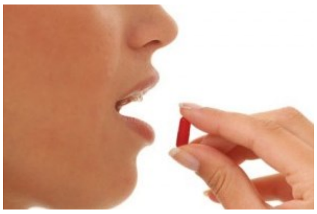	One tablet should be taken once a week on the same day every week.<br>You need to take this medicine for 5 years.<br><br>You need to take the tablet first thing in the morning on an empty stomach with a large glass of tap water.<br>After taking the tablet, you should stay upright for the next 30 minutes.<br>You should not eat anything for at least 30 minutes, including taking any other medicines.<br><br>You need to regularly visit your doctor to have your bone health checked.<br>You need to regularly visit a pharmacy for a new supply.<br>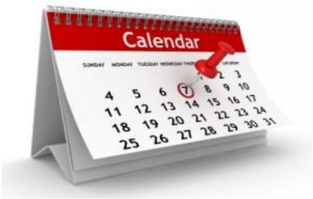 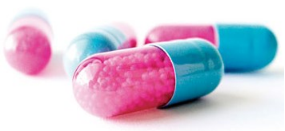

If you take Medicine A, you would live for **4.5 years** (4 years and 6 months) and then die.

If you take Medicine B, you would live for **5 years** and then die.

Would you prefer Medicine A or Medicine B, or are they the same?

Indicate your choice here. You can only choose one option:

◯ **Medicine A** Live for 4 years 6 months

◯ **Medicine B** Live for 5 years

◯ **The Same - Medicine A and Medicine B are the same.**

0% 100%

For peer review only

Repeated section

Remember Medicine A and Medicine B are equally effective and have some risk of a minor side effect.

The only difference between Medicine A and Medicine B is how you take them.

Medicine A	Medicine B
You take a tablet only once in your life. You will be given the tablet on the day you come in to see your GP. 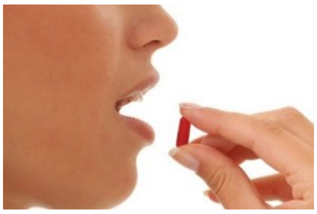	One tablet should be taken once a week on the same day every week. You need to take this medicine for 5 years.<br><br>You need to take the tablet first thing in the morning on an empty stomach with a large glass of tap water. After taking the tablet, you should stay upright for the next 30 minutes. You should not eat anything for at least 30 minutes, including taking any other medicines.<br><br>You need to regularly visit your doctor to have your bone health checked. You need to regularly visit a pharmacy for a new supply. 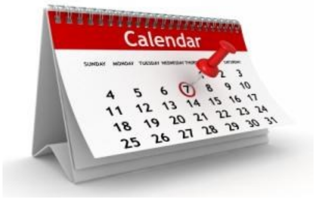 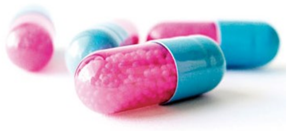

If you take Medicine A, you would live for **4 years and 7 months** and then die.

If you take Medicine B, you would live for **5 years** and then die.

Would you prefer Medicine A or Medicine B, or are they the same?

Indicate your choice here. You can only choose one option:

◯ **Medicine A** Live for 4 years 7 months

◯ **Medicine B** Live for 5 years

◯ **The Same - Medicine A and Medicine B are the same.**

0% 100%

For peer review only

Repeated section

Remember Medicine A and Medicine B are equally effective and have some risk of a minor side effect.

The only difference between Medicine A and Medicine B is how you take them.

Medicine A	Medicine B
You take a tablet only once in your life.<br>You will be given the tablet on the day you come in to see your GP.<br>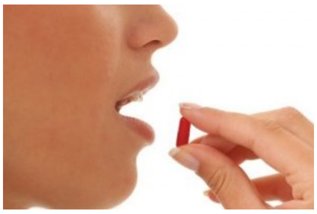	One tablet should be taken once a week on the same day every week.<br>You need to take this medicine for 5 years.<br><br>You need to take the tablet first thing in the morning on an empty stomach with a large glass of tap water.<br>After taking the tablet, you should stay upright for the next 30 minutes.<br>You should not eat anything for at least 30 minutes, including taking any other medicines.<br><br>You need to regularly visit your doctor to have your bone health checked.<br>You need to regularly visit a pharmacy for a new supply.<br>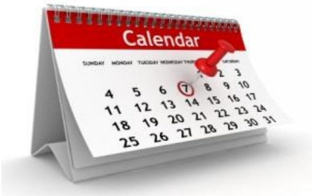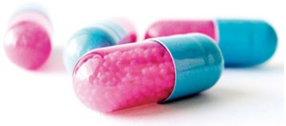

If you take Medicine A, you would live for **4 years and 9 months** and then die.

If you take Medicine B, you would live for **5 years** and then die.

Would you prefer Medicine A or Medicine B, or are they the same?

Indicate your choice here. You can only choose one option:

○ **Medicine A** Live for 4 years 9 months

○ **Medicine B** Live for 5 years

○ **The Same - Medicine A and Medicine B are the same.**

For peer review only

Repeated section

Remember Medicine A and Medicine B are equally effective and have some risk of a minor side effect.

The only difference between Medicine A and Medicine B is how you take them.

Medicine A	Medicine B
You take a tablet only once in your life. You will be given the tablet on the day you come in to see your GP. 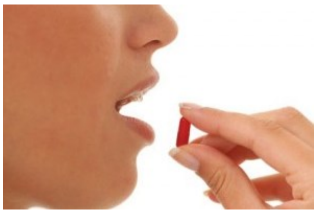	One tablet should be taken once a week on the same day every week. You need to take this medicine for 5 years. You need to take the tablet first thing in the morning on an empty stomach with a large glass of tap water. After taking the tablet, you should stay upright for the next 30 minutes. You should not eat anything for at least 30 minutes, including taking any other medicines. You need to regularly visit your doctor to have your bone health checked. You need to regularly visit a pharmacy for a new supply. 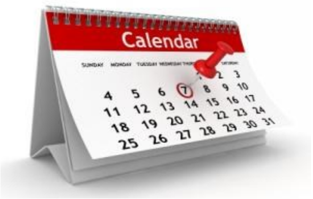 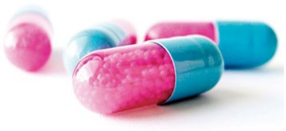

If you take Medicine A, you would live for **2 months less than 5 years** and then die.

If you take Medicine B, you would live for **5 years** and then die.

Would you prefer Medicine A or Medicine B, or are they the same?

Indicate your choice here. You can only choose one option:

◯ **Medicine A** Live for (5 years – 2 months)

◯ **Medicine B** Live for 5 years

◯ **The Same - Medicine A and Medicine B are the same.**

0% 100%

For peer review only

Repeated section

Remember Medicine A and Medicine B are equally effective and have some risk of a minor side effect.

The only difference between Medicine A and Medicine B is how you take them.

Medicine A	Medicine B
You take a tablet only once in your life. You will be given the tablet on the day you come in to see your GP. 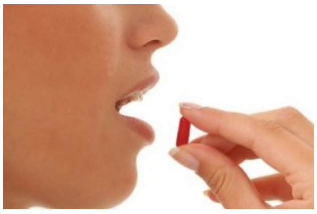	One tablet should be taken once a week on the same day every week. You need to take this medicine for 5 years.<br><br>You need to take the tablet first thing in the morning on an empty stomach with a large glass of tap water. After taking the tablet, you should stay upright for the next 30 minutes. You should not eat anything for at least 30 minutes, including taking any other medicines.<br><br>You need to regularly visit your doctor to have your bone health checked. You need to regularly visit a pharmacy for a new supply. 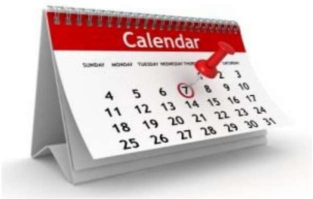 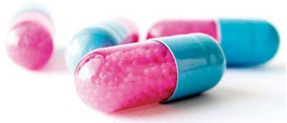

If you take Medicine A, you would live for **1 month less than 5 years** and then die.

If you take Medicine B, you would live for **5 years** and then die.

Would you prefer Medicine A or Medicine B, or are they the same?

Indicate your choice here. You can only choose one option:

◯ **Medicine A** Live for (5 years – 1 month)

◯ **Medicine B** Live for 5 years

◯ **The Same - Medicine A and Medicine B are the same.**

0% 100%

For peer review only

Repeated section

Remember Medicine A and Medicine B are equally effective and have some risk of a minor side effect.

The only difference between Medicine A and Medicine B is how you take them.

Medicine A	Medicine B
You take a tablet only once in your life. You will be given the tablet on the day you come in to see your GP. 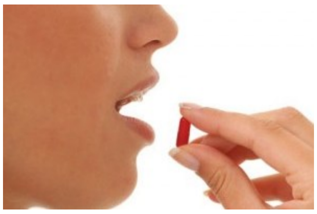	One tablet should be taken once a week on the same day every week. You need to take this medicine for 5 years.<br><br>You need to take the tablet first thing in the morning on an empty stomach with a large glass of tap water. After taking the tablet, you should stay upright for the next 30 minutes. You should not eat anything for at least 30 minutes, including taking any other medicines.<br><br>You need to regularly visit your doctor to have your bone health checked. You need to regularly visit a pharmacy for a new supply. 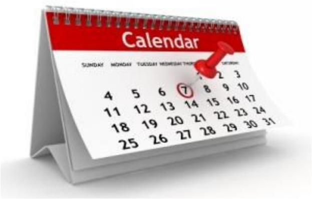 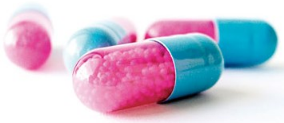

If you take Medicine A, you would live for **2 weeks less than 5 years** and then die.

If you take Medicine B, you would live for **5 years** and then die.

Would you prefer Medicine A or Medicine B, or are they the same?

Indicate your choice here. You can only choose one option:

◯ **Medicine A** Live for (5 years – 2 weeks)

◯ **Medicine B** Live for 5 years

◯ **The Same - Medicine A and Medicine B are the same.**

0% 100%

For peer review only

Repeated section

Remember Medicine A and Medicine B are equally effective and have some risk of a minor side effect.

The only difference between Medicine A and Medicine B is how you take them.

Medicine A	Medicine B
You take a tablet only once in your life. You will be given the tablet on the day you come in to see your GP.<br>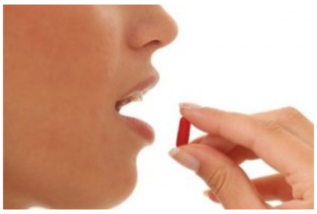	One tablet should be taken once a week on the same day every week.<br>You need to take this medicine for 5 years.<br><br>You need to take the tablet first thing in the morning on an empty stomach with a large glass of tap water.<br>After taking the tablet, you should stay upright for the next 30 minutes.<br>You should not eat anything for at least 30 minutes, including taking any other medicines.<br><br>You need to regularly visit your doctor to have your bone health checked.<br>You need to regularly visit a pharmacy for a new supply.<br>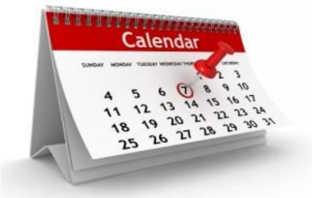 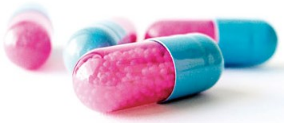

If you take Medicine A, you would live for **1 week less than 5 years** and then die.

If you take Medicine B, you would live for **5 years** and then die.

Would you prefer Medicine A or Medicine B, or are they the same?

Indicate your choice here. You can only choose one option:

◯ **Medicine A** Live for (5 years – 1 week)

◯ **Medicine B** Live for 5 years

◯ **The Same - Medicine A and Medicine B are the same.**

0% 100%

For peer review only

Repeated section

Remember Medicine A and Medicine B are equally effective and have some risk of a minor side effect.

The only difference between Medicine A and Medicine B is how you take them.

Medicine A	Medicine B
You take a tablet only once in your life. You will be given the tablet on the day you come in to see your GP. 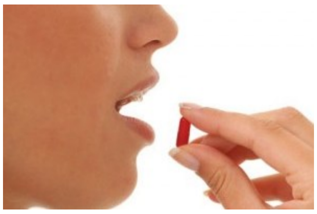	One tablet should be taken once a week on the same day every week. You need to take this medicine for 5 years. You need to take the tablet first thing in the morning on an empty stomach with a large glass of tap water. After taking the tablet, you should stay upright for the next 30 minutes. You should not eat anything for at least 30 minutes, including taking any other medicines. You need to regularly visit your doctor to have your bone health checked. You need to regularly visit a pharmacy for a new supply. 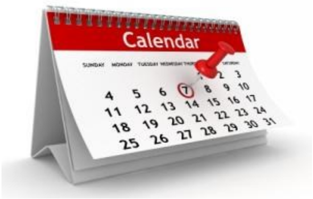 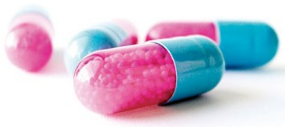

If you take Medicine A, you would live for **5 days less than 5 years** and then die.

If you take Medicine B, you would live for **5 years** and then die.

Would you prefer Medicine A or Medicine B, or are they the same?

Indicate your choice here. You can only choose one option:

◯ **Medicine A** Live for (5 years – 5 days)

◯ **Medicine B** Live for 5 years

◯ **The Same - Medicine A and Medicine B are the same.**

0% 100%

For peer review only

Repeated section

Remember Medicine A and Medicine B are equally effective and have some risk of a minor side effect.

The only difference between Medicine A and Medicine B is how you take them.

Medicine A	Medicine B
You take a tablet only once in your life. You will be given the tablet on the day you come in to see your GP. 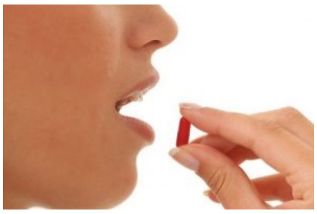	One tablet should be taken once a week on the same day every week. You need to take this medicine for 5 years.<br><br>You need to take the tablet first thing in the morning on an empty stomach with a large glass of tap water. After taking the tablet, you should stay upright for the next 30 minutes. You should not eat anything for at least 30 minutes, including taking any other medicines.<br><br>You need to regularly visit your doctor to have your bone health checked. You need to regularly visit a pharmacy for a new supply. 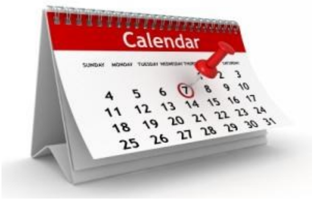 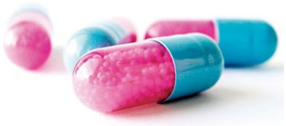

If you take Medicine A, you would live for **3 days less than 5 years** and then die.

If you take Medicine B, you would live for **5 years** and then die.

Would you prefer Medicine A or Medicine B, or are they the same?

Indicate your choice here. You can only choose one option:

○ **Medicine A** Live for (5 years – 3 days)

○ **Medicine B** Live for 5 years

○ **The Same - Medicine A and Medicine B are the same.**

For peer review only

Repeated section

Remember Medicine A and Medicine B are equally effective and have some risk of a minor side effect.

The only difference between Medicine A and Medicine B is how you take them.

Medicine A	Medicine B
You take a tablet only once in your life. You will be given the tablet on the day you come in to see your GP.<br>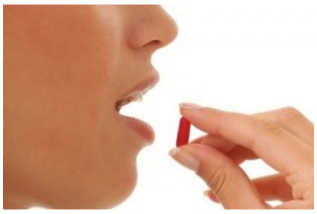	One tablet should be taken once a week on the same day every week.<br>You need to take this medicine for 5 years.<br><br>You need to take the tablet first thing in the morning on an empty stomach with a large glass of tap water.<br>After taking the tablet, you should stay upright for the next 30 minutes.<br>You should not eat anything for at least 30 minutes, including taking any other medicines.<br><br>You need to regularly visit your doctor to have your bone health checked.<br>You need to regularly visit a pharmacy for a new supply.<br>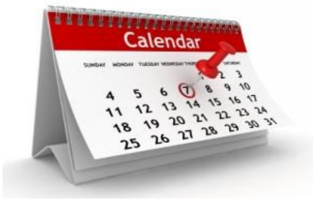 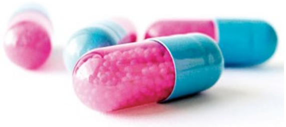

If you take Medicine A, you would live for **1 day less than 5 years** and then die.

If you take Medicine B, you would live for **5 years** and then die.

Would you prefer Medicine A or Medicine B, or are they the same?

Indicate your choice here. You can only choose one option:

◯ **Medicine A** Live for (5 years – 1 day)

◯ **Medicine B** Live for 5 years

◯ **The Same - Medicine A and Medicine B are the same.**

0% 100%

For peer review only

Question 2a.

You prefer living for 2.5 years (2 years and 6 months) on Medicine A, to living for 5 years on Medicine B.

This means **living fewer than 2.5 years on Medicine A** can be the same as living for 5 years on Medicine B.

How many years on Medicine A do you think would be the same as living for 5 years on Medicine B?

(Please give a number in years)

0% 100%

peer review only

Part 2

Question 3

You have been told that you need to take a medicine to reduce the risk of fracture.

You can choose between two different medicines. We have called these Medicine A and Medicine B.

Medicine A is a pill that you only need to take once.

Medicine B is a pill that you need to take every week for 5 years.

Medicine A and Medicine B are equally effective in reducing your risk of having a fracture.
Medicine A and Medicine B will reduce your risk of experiencing a fracture, such as hip fracture, within the next 5 years from **10%** to **6%**.

In other words, in a crowd of **1,000** people in the UK, **100** are likely to have a fracture if they do not take the medicine. This is reduced to **60** people out of 1,000 who do take the medicine.
This information is also illustrated in this picture.

Risk of fracture

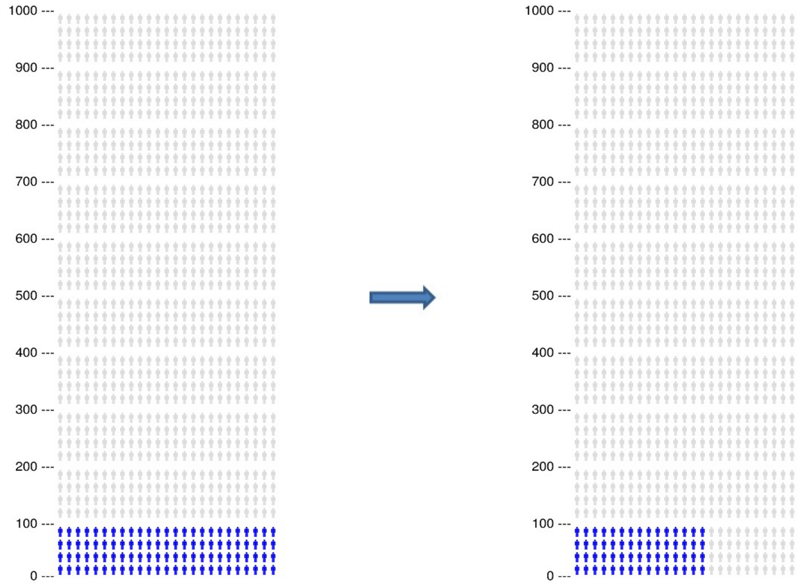

10%
No Treatment

6%
Taking the Medicine

(The figures shaded blue represent people who will have a fracture, such as hip fracture, within the next 5 years)

In this scenario, there is some risk of a severe side effect from Medicine A or Medicine B.

Among every 1,000 people taking Medicine A or Medicine B, **3** people are likely to have a severe side effect such as severe loss or destruction of the bone in the jaw.

Risk of a severe side effect

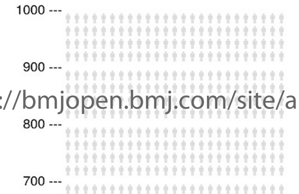

For peer review only - http://bmjopen.bmj.com/site/about/guidelines.xhtml

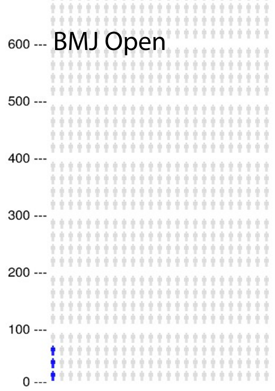

0.3% of people will have a severe side effect.

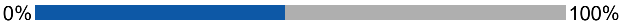

For peer review only

Repeated section

Remember Medicine A and Medicine B are equally effective and have some risk of a severe side effect.

The only difference between Medicine A and Medicine B is how you take them.

Medicine A	Medicine B
You take a tablet only once in your life. You will be given the tablet on the day you come in to see your GP. 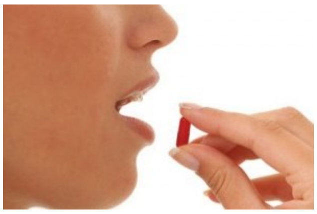	One tablet should be taken once a week on the same day every week.<br>You need to take this medicine for 5 years.<br><br>You need to take the tablet first thing in the morning on an empty stomach with a large glass of tap water.<br>After taking the tablet, you should stay upright for the next 30 minutes.<br>You should not eat anything for at least 30 minutes, including taking any other medicines.<br><br>You need to regularly visit your doctor to have your bone health checked.<br>You need to regularly visit a pharmacy for a new supply. 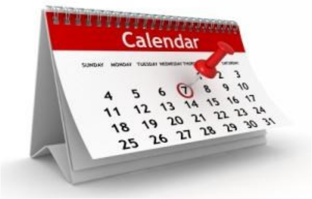 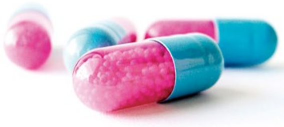

If you take Medicine A, you would live for **2.5 years (2 years and 6 months)** and then die.

If you take Medicine B, you would live for **5 years** and then die.

Would you prefer Medicine A or Medicine B, or are they the same?

Indicate your choice here. You can only choose one option:

◯ **Medicine A**

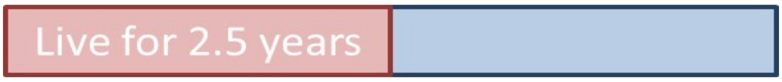

◯ **Medicine B**

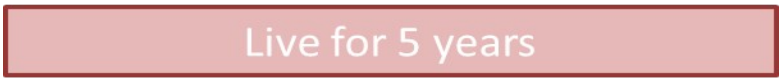

◯ **The Same - Medicine A and Medicine B are the same.**

For peer review only

Repeated section

Remember Medicine A and Medicine B are equally effective and have some risk of a severe side effect.

The only difference between Medicine A and Medicine B is how you take them.

Medicine A	Medicine B
You take a tablet only once in your life. You will be given the tablet on the day you come in to see your GP.<br>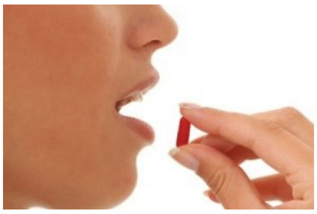	One tablet should be taken once a week on the same day every week.<br>You need to take this medicine for 5 years.<br><br>You need to take the tablet first thing in the morning on an empty stomach with a large glass of tap water.<br>After taking the tablet, you should stay upright for the next 30 minutes.<br>You should not eat anything for at least 30 minutes, including taking any other medicines.<br><br>You need to regularly visit your doctor to have your bone health checked.<br>You need to regularly visit a pharmacy for a new supply.<br>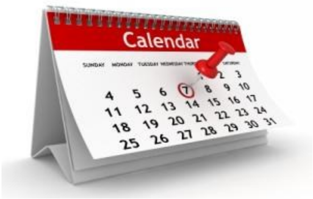 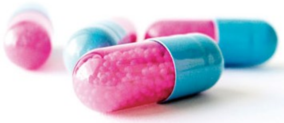

If you take Medicine A, you would live for **3 years** and then die.

If you take Medicine B, you would live for **5 years** and then die.

Would you prefer Medicine A or Medicine B, or are they the same?

Indicate your choice here. You can only choose one option:

◯ **Medicine A**

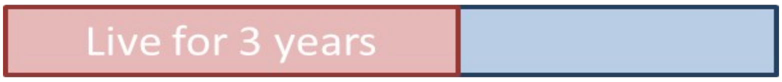

◯ **Medicine B**

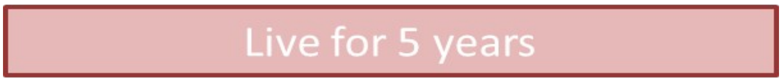

◯ **The Same - Medicine A and Medicine B are the same.**

For peer review only

Repeated section

Remember Medicine A and Medicine B are equally effective and have some risk of a severe side effect.

The only difference between Medicine A and Medicine B is how you take them.

Medicine A	Medicine B
You take a tablet only once in your life.<br>You will be given the tablet on the day you come in to see your GP.<br>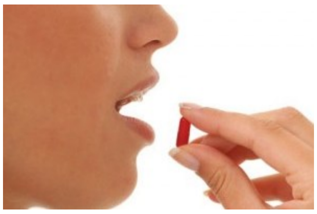	One tablet should be taken once a week on the same day every week.<br>You need to take this medicine for 5 years.<br><br>You need to take the tablet first thing in the morning on an empty stomach with a large glass of tap water.<br>After taking the tablet, you should stay upright for the next 30 minutes.<br>You should not eat anything for at least 30 minutes, including taking any other medicines.<br><br>You need to regularly visit your doctor to have your bone health checked.<br>You need to regularly visit a pharmacy for a new supply.<br>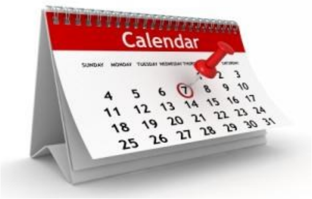 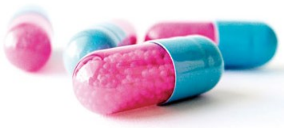

If you take Medicine A, you would live for **3.25 years** (3 years and 3 months) and then die.

If you take Medicine B, you would live for **5 years** and then die.

Would you prefer Medicine A or Medicine B, or are they the same?

Indicate your choice here. You can only choose one option:

◯ **Medicine A** Live for 3 years 3 months

◯ **Medicine B** Live for 5 years

◯ **The Same - Medicine A and Medicine B are the same.**

0% 100%

For peer review only

Repeated section

Remember Medicine A and Medicine B are equally effective and have some risk of a severe side effect.

The only difference between Medicine A and Medicine B is how you take them.

Medicine A	Medicine B
You take a tablet only once in your life.<br>You will be given the tablet on the day you come in to see your GP.<br>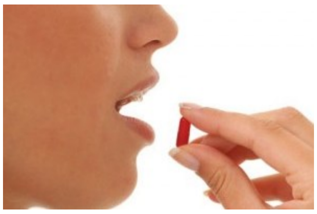	One tablet should be taken once a week on the same day every week.<br>You need to take this medicine for 5 years.<br><br>You need to take the tablet first thing in the morning on an empty stomach with a large glass of tap water.<br>After taking the tablet, you should stay upright for the next 30 minutes.<br>You should not eat anything for at least 30 minutes, including taking any other medicines.<br><br>You need to regularly visit your doctor to have your bone health checked.<br>You need to regularly visit a pharmacy for a new supply.<br>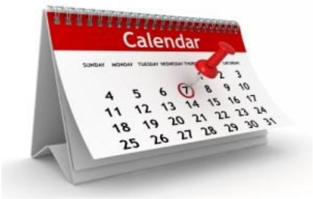 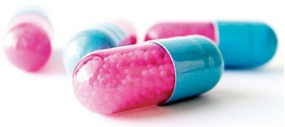

If you take Medicine A, you would live for **3.5 years** (3 years and 6 months) and then die.

If you take Medicine B, you would live for **5 years** and then die.

Would you prefer Medicine A or Medicine B, or are they the same?

Indicate your choice here. You can only choose one option:

○ **Medicine A** Live for 3 years 6 months

○ **Medicine B** Live for 5 years

○ **The Same - Medicine A and Medicine B are the same.**

For peer review only

Repeated section

Remember Medicine A and Medicine B are equally effective and have no risks of a side effect.

The only difference between Medicine A and Medicine B is how you take them.

**Medicine A**	**Medicine B**
You take a tablet only once in your life.<br>You will be given the tablet on the day you come in to see your GP.<br>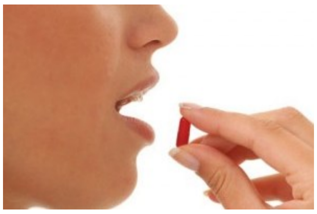	One tablet should be taken once a week on the same day every week.<br>You need to take this medicine for 5 years.<br><br>You need to take the tablet first thing in the morning on an empty stomach with a large glass of tap water.<br>After taking the tablet, you should stay upright for the next 30 minutes.<br>You should not eat anything for at least 30 minutes, including taking any other medicines.<br><br>You need to regularly visit your doctor to have your bone health checked.<br>You need to regularly visit a pharmacy for a new supply.<br>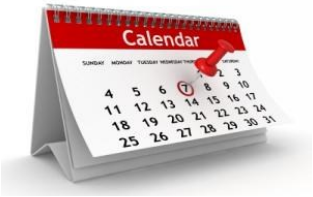 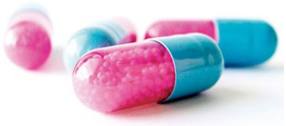

If you take Medicine A, you would live for **3.75 years** (3 years and 9 months) and then die.

If you take Medicine B, you would live for **5 years** and then die.

Would you prefer Medicine A or Medicine B, or are they the same?

Indicate your choice here. You can only choose one option:

○ **Medicine A** Live for 3 years 9 months

○ **Medicine B** Live for 5 years

○ **The Same - Medicine A and Medicine B are the same.**

For peer review only

Repeated section

Remember Medicine A and Medicine B are equally effective and have some risk of a severe side effect.

The only difference between Medicine A and Medicine B is how you take them.

Medicine A	Medicine B
You take a tablet only once in your life.<br>You will be given the tablet on the day you come in to see your GP.<br>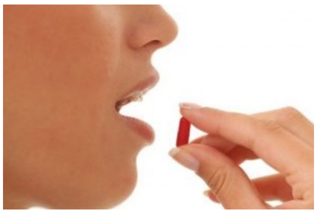	One tablet should be taken once a week on the same day every week.<br>You need to take this medicine for 5 years.<br><br>You need to take the tablet first thing in the morning on an empty stomach with a large glass of tap water.<br>After taking the tablet, you should stay upright for the next 30 minutes.<br>You should not eat anything for at least 30 minutes, including taking any other medicines.<br><br>You need to regularly visit your doctor to have your bone health checked.<br>You need to regularly visit a pharmacy for a new supply.<br>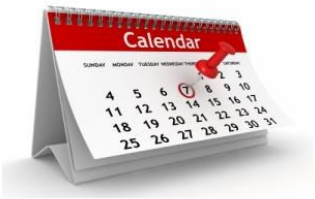 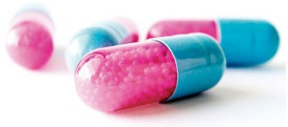

If you take Medicine A, you would live for **3 years and 10 months** and then die.

If you take Medicine B, you would live for **5 years** and then die.

Would you prefer Medicine A or Medicine B, or are they the same?

Indicate your choice here. You can only choose one option:

◯ **Medicine A**

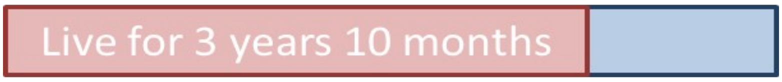

◯ **Medicine B**

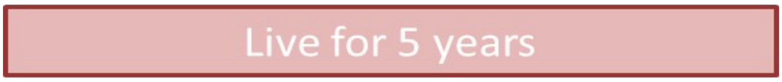

◯ **The Same - Medicine A and Medicine B are the same.**

0% 100%

For peer review only

Repeated section

Remember Medicine A and Medicine B are equally effective and have some risk of a severe side effect.

The only difference between Medicine A and Medicine B is how you take them.

Medicine A	Medicine B
You take a tablet only once in your life.<br>You will be given the tablet on the day you come in to see your GP.<br>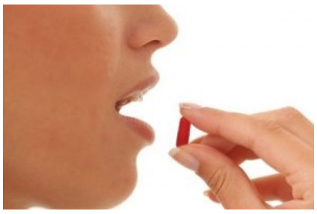	One tablet should be taken once a week on the same day every week.<br>You need to take this medicine for 5 years.<br><br>You need to take the tablet first thing in the morning on an empty stomach with a large glass of tap water.<br>After taking the tablet, you should stay upright for the next 30 minutes.<br>You should not eat anything for at least 30 minutes, including taking any other medicines.<br><br>You need to regularly visit your doctor to have your bone health checked.<br>You need to regularly visit a pharmacy for a new supply.<br>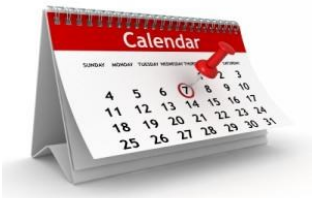 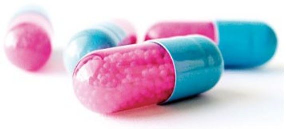

If you take Medicine A, you would live for **4 years** and then die.

If you take Medicine B, you would live for **5 years** and then die.

Would you prefer Medicine A or Medicine B, or are they the same?

Indicate your choice here. You can only choose one option:

◯ **Medicine A** Live for 4 years

◯ **Medicine B** Live for 5 years

◯ **The Same - Medicine A and Medicine B are the same.**

0% 100%

For peer review only

Repeated section

Remember Medicine A and Medicine B are equally effective and have some risk of a severe side effect.

The only difference between Medicine A and Medicine B is how you take them.

Medicine A	Medicine B
You take a tablet only once in your life.<br>You will be given the tablet on the day you come in to see your GP.<br>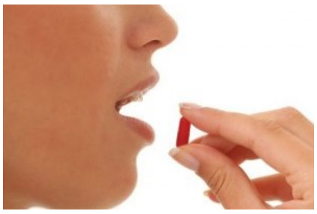	One tablet should be taken once a week on the same day every week.<br>You need to take this medicine for 5 years.<br><br>You need to take the tablet first thing in the morning on an empty stomach with a large glass of tap water.<br>After taking the tablet, you should stay upright for the next 30 minutes.<br>You should not eat anything for at least 30 minutes, including taking any other medicines.<br><br>You need to regularly visit your doctor to have your bone health checked.<br>You need to regularly visit a pharmacy for a new supply.<br>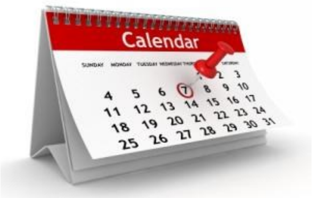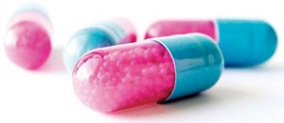

If you take Medicine A, you would live for **4 years and 2 months** and then die.

If you take Medicine B, you would live for **5 years** and then die.

Would you prefer Medicine A or Medicine B, or are they the same?

Indicate your choice here. You can only choose one option:

◯ **Medicine A** Live for 4 years 2 months

◯ **Medicine B** Live for 5 years

◯ **The Same - Medicine A and Medicine B are the same.**

0% 100%

For peer review only

Repeated section

Remember Medicine A and Medicine B are equally effective and have some risk of a severe side effect.

The only difference between Medicine A and Medicine B is how you take them.

**Medicine A**	**Medicine B**
You take a tablet only once in your life.<br>You will be given the tablet on the day you come in to see your GP.<br>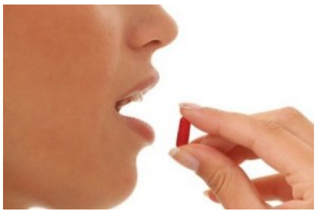	One tablet should be taken once a week on the same day every week.<br>You need to take this medicine for 5 years.<br><br>You need to take the tablet first thing in the morning on an empty stomach with a large glass of tap water.<br>After taking the tablet, you should stay upright for the next 30 minutes.<br>You should not eat anything for at least 30 minutes, including taking any other medicines.<br><br>You need to regularly visit your doctor to have your bone health checked.<br>You need to regularly visit a pharmacy for a new supply.<br>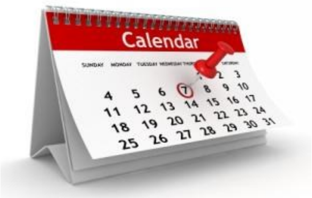 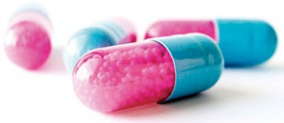

If you take Medicine A, you would live for **4.25 years** (4 years and 3 months) and then die.

If you take Medicine B, you would live for **5 years** and then die.

Would you prefer Medicine A or Medicine B, or are they the same?

Indicate your choice here. You can only choose one option:

○ **Medicine A** Live for 4 years 3 months

○ **Medicine B** Live for 5 years

○ **The Same - Medicine A and Medicine B are the same.**

0% 100%

For peer review only

Repeated section

Remember Medicine A and Medicine B are equally effective and have some risk of a severe side effect.

The only difference between Medicine A and Medicine B is how you take them.

Medicine A	Medicine B
You take a tablet only once in your life. You will be given the tablet on the day you come in to see your GP. 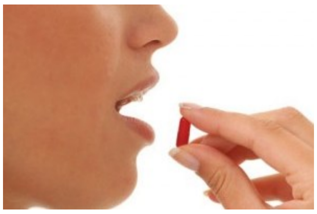	One tablet should be taken once a week on the same day every week. You need to take this medicine for 5 years.<br><br>You need to take the tablet first thing in the morning on an empty stomach with a large glass of tap water. After taking the tablet, you should stay upright for the next 30 minutes. You should not eat anything for at least 30 minutes, including taking any other medicines.<br><br>You need to regularly visit your doctor to have your bone health checked. You need to regularly visit a pharmacy for a new supply. 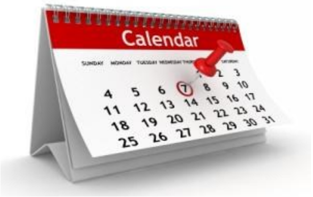 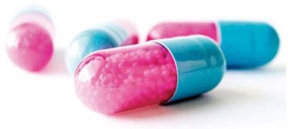

If you take Medicine A, you would live for **4 years 4 months** and then die.

If you take Medicine B, you would live for **5 years** and then die.

Would you prefer Medicine A or Medicine B, or are they the same?

Indicate your choice here. You can only choose one option:

◯ **Medicine A** Live for 4 years 4 months

◯ **Medicine B** Live for 5 years

◯ **The Same - Medicine A and Medicine B are the same.**

0% 100%

For peer review only

Repeated section

Remember Medicine A and Medicine B are equally effective and have some risk of a severe side effect.

The only difference between Medicine A and Medicine B is how you take them.

**Medicine A**	**Medicine B**
You take a tablet only once in your life.<br>You will be given the tablet on the day you come in to see your GP.<br>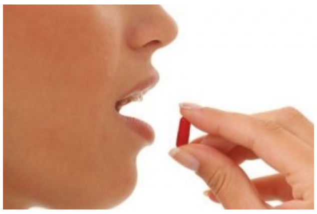	One tablet should be taken once a week on the same day every week.<br>You need to take this medicine for 5 years.<br><br>You need to take the tablet first thing in the morning on an empty stomach with a large glass of tap water.<br>After taking the tablet, you should stay upright for the next 30 minutes.<br>You should not eat anything for at least 30 minutes, including taking any other medicines.<br><br>You need to regularly visit your doctor to have your bone health checked.<br>You need to regularly visit a pharmacy for a new supply.<br>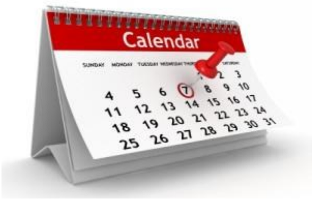 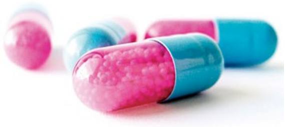

If you take Medicine A, you would live for **4.5 years** (4 years and 6 months) and then die.

If you take Medicine B, you would live for **5 years** and then die.

Would you prefer Medicine A or Medicine B, or are they the same?

Indicate your choice here. You can only choose one option:

◯ **Medicine A** Live for 4 years 6 months

◯ **Medicine B** Live for 5 years

◯ **The Same - Medicine A and Medicine B are the same.**

0% 100%

For peer review only

Repeated section

Remember Medicine A and Medicine B are equally effective and have some risk of a severe side effect.

The only difference between Medicine A and Medicine B is how you take them.

Medicine A	Medicine B
You take a tablet only once in your life. You will be given the tablet on the day you come in to see your GP. 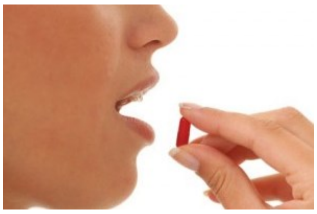	One tablet should be taken once a week on the same day every week. You need to take this medicine for 5 years.<br><br>You need to take the tablet first thing in the morning on an empty stomach with a large glass of tap water. After taking the tablet, you should stay upright for the next 30 minutes. You should not eat anything for at least 30 minutes, including taking any other medicines.<br><br>You need to regularly visit your doctor to have your bone health checked. You need to regularly visit a pharmacy for a new supply. 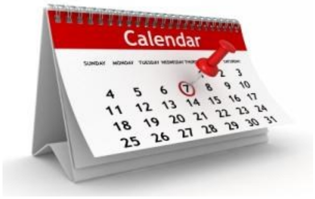 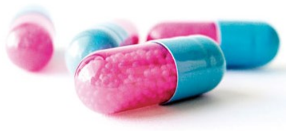

If you take Medicine A, you would live for **4 years and 7 months** and then die.

If you take Medicine B, you would live for **5 years** and then die.

Would you prefer Medicine A or Medicine B, or are they the same?

Indicate your choice here. You can only choose one option:

◯ **Medicine A** Live for 4 years 7 months

◯ **Medicine B** Live for 5 years

◯ **The Same - Medicine A and Medicine B are the same.**

For peer review only

Repeated section

Remember Medicine A and Medicine B are equally effective and have some risk of a severe side effect.

The only difference between Medicine A and Medicine B is how you take them.

Medicine A	Medicine B
You take a tablet only once in your life.<br>You will be given the tablet on the day you come in to see your GP.<br>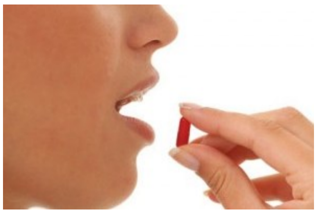	One tablet should be taken once a week on the same day every week.<br>You need to take this medicine for 5 years.<br><br>You need to take the tablet first thing in the morning on an empty stomach with a large glass of tap water.<br>After taking the tablet, you should stay upright for the next 30 minutes.<br>You should not eat anything for at least 30 minutes, including taking any other medicines.<br><br>You need to regularly visit your doctor to have your bone health checked.<br>You need to regularly visit a pharmacy for a new supply.<br>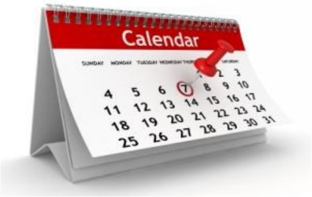 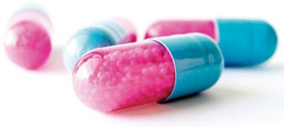

If you take Medicine A, you would live for **4 years and 9 months** and then die.

If you take Medicine B, you would live for **5 years** and then die.

Would you prefer Medicine A or Medicine B, or are they the same?

Indicate your choice here. You can only choose one option:

◯ **Medicine A** — Live for 4 years 9 months

◯ **Medicine B** — Live for 5 years

◯ **The Same - Medicine A and Medicine B are the same.**

0% 100%

For peer review only

Repeated section

Remember Medicine A and Medicine B are equally effective and have some risk of a severe side effect.

The only difference between Medicine A and Medicine B is how you take them.

Medicine A	Medicine B
You take a tablet only once in your life. You will be given the tablet on the day you come in to see your GP. 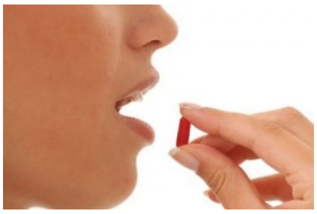	One tablet should be taken once a week on the same day every week.<br>You need to take this medicine for 5 years.<br><br>You need to take the tablet first thing in the morning on an empty stomach with a large glass of tap water.<br>After taking the tablet, you should stay upright for the next 30 minutes.<br>You should not eat anything for at least 30 minutes, including taking any other medicines.<br><br>You need to regularly visit your doctor to have your bone health checked.<br>You need to regularly visit a pharmacy for a new supply.<br>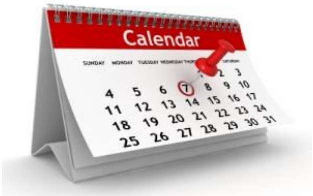 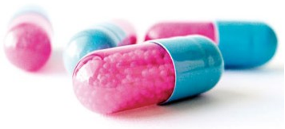

If you take Medicine A, you would live for **2 months less than 5 years** and then die.

If you take Medicine B, you would live for **5 years** and then die.

Would you prefer Medicine A or Medicine B, or are they the same?

Indicate your choice here. You can only choose one option:

◯ **Medicine A** Live for (5 years – 2 months)

◯ **Medicine B** Live for 5 years

◯ **The Same - Medicine A and Medicine B are the same.**

0% 100%

For peer review only

Repeated section

Remember Medicine A and Medicine B are equally effective and have some risk of a severe side effect.

The only difference between Medicine A and Medicine B is how you take them.

**Medicine A**	**Medicine B**
You take a tablet only once in your life. You will be given the tablet on the day you come in to see your GP. 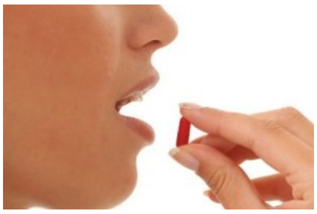	One tablet should be taken once a week on the same day every week. You need to take this medicine for 5 years.<br><br>You need to take the tablet first thing in the morning on an empty stomach with a large glass of tap water. After taking the tablet, you should stay upright for the next 30 minutes. You should not eat anything for at least 30 minutes, including taking any other medicines.<br><br>You need to regularly visit your doctor to have your bone health checked. You need to regularly visit a pharmacy for a new supply. 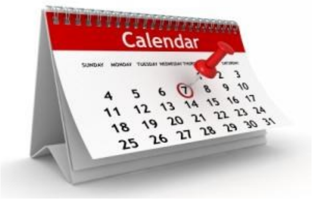 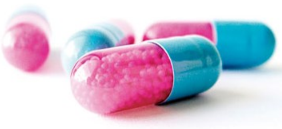

If you take Medicine A, you would live for **1 month less than 5 years** and then die.

If you take Medicine B, you would live for **5 years** and then die.

Would you prefer Medicine A or Medicine B, or are they the same?

Indicate your choice here. You can only choose one option:

◯ **Medicine A** 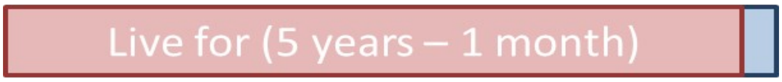

◯ **Medicine B** 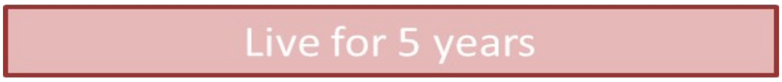

◯ **The Same - Medicine A and Medicine B are the same.**

0% 100%

For peer review only

Repeated section

Remember Medicine A and Medicine B are equally effective and have some risk of a severe side effect.

The only difference between Medicine A and Medicine B is how you take them.

**Medicine A**	**Medicine B**
You take a tablet only once in your life. You will be given the tablet on the day you come in to see your GP. 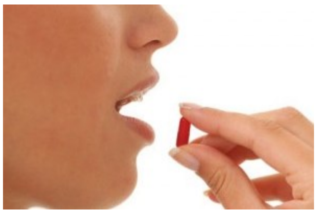	One tablet should be taken once a week on the same day every week. You need to take this medicine for 5 years.<br><br>You need to take the tablet first thing in the morning on an empty stomach with a large glass of tap water. After taking the tablet, you should stay upright for the next 30 minutes. You should not eat anything for at least 30 minutes, including taking any other medicines.<br><br>You need to regularly visit your doctor to have your bone health checked. You need to regularly visit a pharmacy for a new supply. 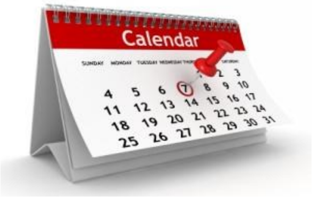 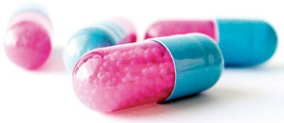

If you take Medicine A, you would live for **2 weeks less than 5 years** and then die.

If you take Medicine B, you would live for **5 years** and then die.

Would you prefer Medicine A or Medicine B, or are they the same?

Indicate your choice here. You can only choose one option:

○ **Medicine A** Live for (5 years – 2 weeks)

○ **Medicine B** Live for 5 years

○ **The Same - Medicine A and Medicine B are the same.**

For peer review only

Repeated section

Remember Medicine A and Medicine B are equally effective and have some risk of a severe side effect.

The only difference between Medicine A and Medicine B is how you take them.

Medicine A	Medicine B
You take a tablet only once in your life.<br>You will be given the tablet on the day you come in to see your GP.<br>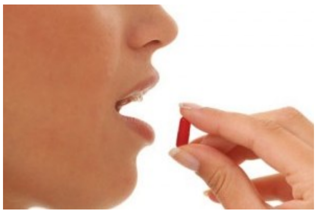	One tablet should be taken once a week on the same day every week.<br>You need to take this medicine for 5 years.<br><br>You need to take the tablet first thing in the morning on an empty stomach with a large glass of tap water.<br>After taking the tablet, you should stay upright for the next 30 minutes.<br>You should not eat anything for at least 30 minutes, including taking any other medicines.<br><br>You need to regularly visit your doctor to have your bone health checked.<br>You need to regularly visit a pharmacy for a new supply.<br>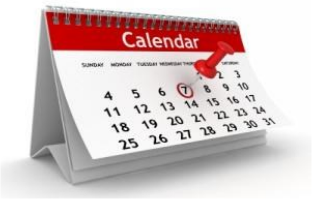 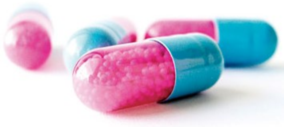

If you take Medicine A, you would live for **1 week less than 5 years** and then die.

If you take Medicine B, you would live for **5 years** and then die.

Would you prefer Medicine A or Medicine B, or are they the same?

Indicate your choice here. You can only choose one option:

○ **Medicine A** Live for (5 years – 1 week)

○ **Medicine B** Live for 5 years

○ **The Same - Medicine A and Medicine B are the same.**

0% 100%

For peer review only

Repeated section

Remember Medicine A and Medicine B are equally effective and have some risk of a severe side effect.

The only difference between Medicine A and Medicine B is how you take them.

Medicine A	Medicine B
You take a tablet only once in your life. You will be given the tablet on the day you come in to see your GP.<br>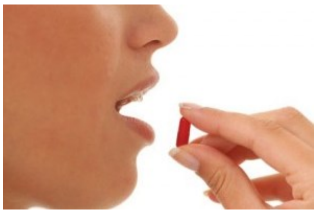	One tablet should be taken once a week on the same day every week.<br>You need to take this medicine for 5 years.<br><br>You need to take the tablet first thing in the morning on an empty stomach with a large glass of tap water.<br>After taking the tablet, you should stay upright for the next 30 minutes.<br>You should not eat anything for at least 30 minutes, including taking any other medicines.<br><br>You need to regularly visit your doctor to have your bone health checked.<br>You need to regularly visit a pharmacy for a new supply.<br>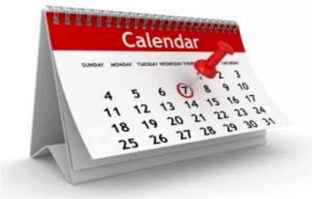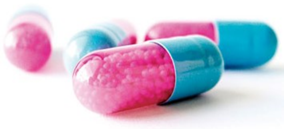

If you take Medicine A, you would live for **5 days less than 5 years** and then die.

If you take Medicine B, you would live for **5 years** and then die.

Would you prefer Medicine A or Medicine B, or are they the same?

Indicate your choice here. You can only choose one option:

◯ **Medicine A** Live for (5 years – 5 days)

◯ **Medicine B** Live for 5 years

◯ **The Same - Medicine A and Medicine B are the same.**

For peer review only

Repeated section

Remember Medicine A and Medicine B are equally effective and have some risk of a severe side effect.

The only difference between Medicine A and Medicine B is how you take them.

Medicine A	Medicine B
You take a tablet only once in your life. You will be given the tablet on the day you come in to see your GP. 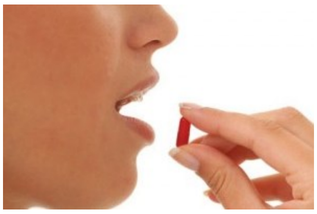	One tablet should be taken once a week on the same day every week.<br>You need to take this medicine for 5 years.<br><br>You need to take the tablet first thing in the morning on an empty stomach with a large glass of tap water.<br>After taking the tablet, you should stay upright for the next 30 minutes.<br>You should not eat anything for at least 30 minutes, including taking any other medicines.<br><br>You need to regularly visit your doctor to have your bone health checked.<br>You need to regularly visit a pharmacy for a new supply. 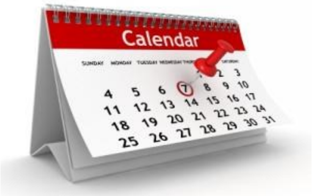 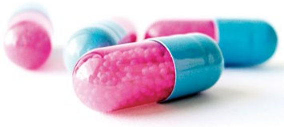

If you take Medicine A, you would live for **3 days less than 5 years** and then die.

If you take Medicine B, you would live for **5 years** and then die.

Would you prefer Medicine A or Medicine B, or are they the same?

Indicate your choice here. You can only choose one option:

◯ **Medicine A** Live for (5 years – 3 days)

◯ **Medicine B** Live for 5 years

◯ **The Same - Medicine A and Medicine B are the same.**

0% 100%

For peer review only

Repeated section

Remember Medicine A and Medicine B are equally effective and have some risk of a severe side effect.

The only difference between Medicine A and Medicine B is how you take them.

**Medicine A**	**Medicine B**
You take a tablet only once in your life. You will be given the tablet on the day you come in to see your GP. 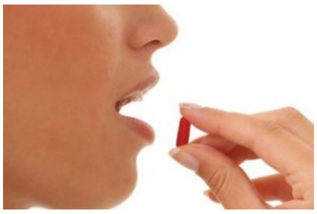	One tablet should be taken once a week on the same day every week. You need to take this medicine for 5 years.<br><br>You need to take the tablet first thing in the morning on an empty stomach with a large glass of tap water. After taking the tablet, you should stay upright for the next 30 minutes. You should not eat anything for at least 30 minutes, including taking any other medicines.<br><br>You need to regularly visit your doctor to have your bone health checked. You need to regularly visit a pharmacy for a new supply.<br><br>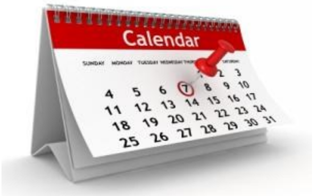 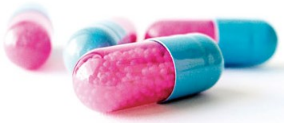

If you take Medicine A, you would live for **1 day less than 5 years** and then die.

If you take Medicine B, you would live for **5 years** and then die.

Would you prefer Medicine A or Medicine B, or are they the same?

Indicate your choice here. You can only choose one option:

○ **Medicine A** Live for (5 years – 1 day)

○ **Medicine B** Live for 5 years

○ **The Same - Medicine A and Medicine B are the same.**

0% 100%

For peer review only

Question 3a.

You prefer living for 2.5 years (2 years and 6 months) on Medicine A, to living for 5 years on Medicine B.

This means **living fewer than 2.5 years on Medicine A** can be the same as living for 5 years on Medicine B.

How many years on Medicine A do you think would be the same as living for 5 years on Medicine B?

(Please give a number in years)

0% 100%

peer review only

Part 2

Question 4

Now, Medicine A and Medicine B will give you less benefit than before because you belong to a group of people who may not respond to the treatment as well as other groups of people.

As before,

Medicine A is a pill that you only need to take once.

Medicine B is a pill that you need to take every week for 5 years.

Medicine A and Medicine B are equally effective in reducing your risk of having a fracture.

Medicine A and Medicine B will reduce your risk of experiencing a fracture, such as hip fracture, within the next 5 years from **10%** to **9%**.

In other words, in a crowd of **1,000** people in the UK, **100** are likely to have a fracture if they do not take the medicine. This is reduced to **90** people out of 1,000 who do take the medicine. This information is also illustrated in this picture.

Risk of fracture

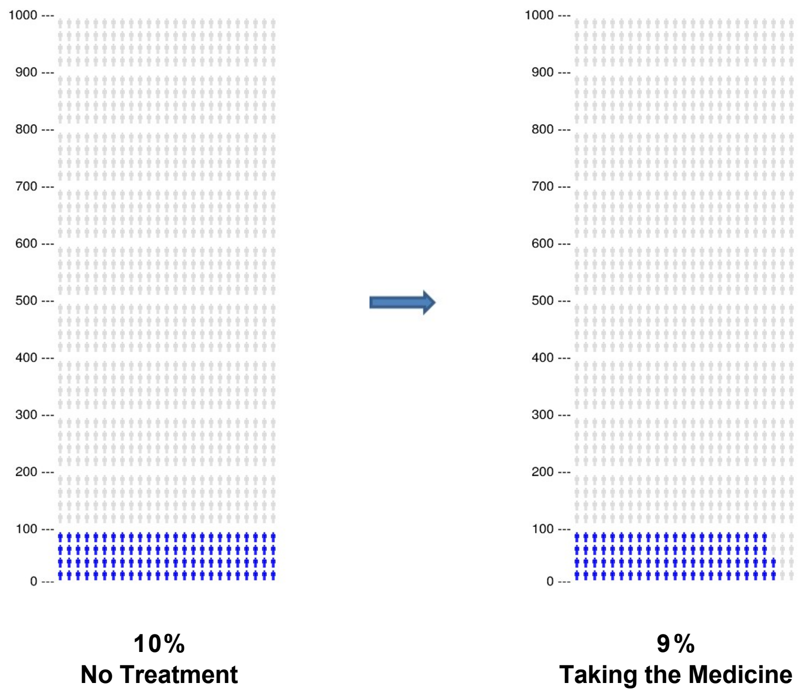

(The figures shaded blue represent people who will have a fracture, such as hip fracture, within the next 5 years)

In this scenario, you will not have any side effects from Medicine A or Medicine B.

Risk of a side effect

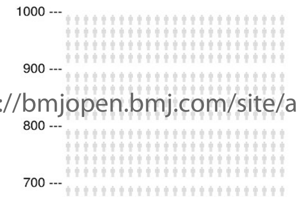

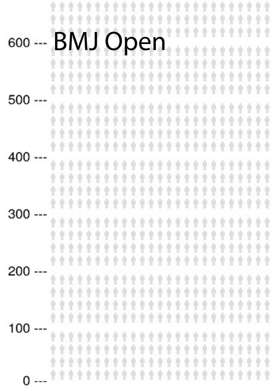

0%
No one will have a side effect.

0% 100%

eer review only

Repeated section

Remember Medicine A and Medicine B are equally effective and have no risks of a side effect.

The only difference between Medicine A and Medicine B is how you take them.

**Medicine A**	**Medicine B**
You take a tablet only once in your life. You will be given the tablet on the day you come in to see your GP. 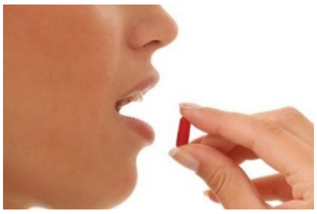	One tablet should be taken once a week on the same day every week. You need to take this medicine for 5 years. You need to take the tablet first thing in the morning on an empty stomach with a large glass of tap water. After taking the tablet, you should stay upright for the next 30 minutes. You should not eat anything for at least 30 minutes, including taking any other medicines. You need to regularly visit your doctor to have your bone health checked. You need to regularly visit a pharmacy for a new supply. 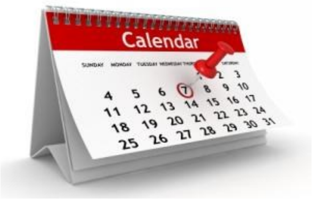 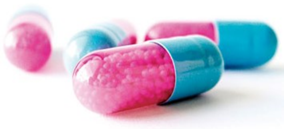

If you take Medicine A, you would live for **2.5 years (2 years and 6 months)** and then die.

If you take Medicine B, you would live for **5 years** and then die.

Would you prefer Medicine A or Medicine B, or are they the same?

Indicate your choice here. You can only choose one option:

◯ **Medicine A** Live for 2.5 years

◯ **Medicine B** Live for 5 years

◯ **The Same - Medicine A and Medicine B are the same.**

For peer review only

Repeated section

Remember Medicine A and Medicine B are equally effective and have no risks of a side effect.

The only difference between Medicine A and Medicine B is how you take them.

Medicine A	Medicine B
You take a tablet only once in your life.<br>You will be given the tablet on the day you come in to see your GP.<br>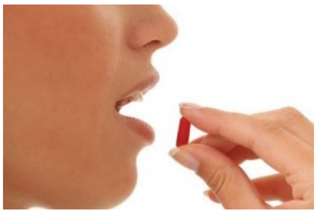	One tablet should be taken once a week on the same day every week.<br>You need to take this medicine for 5 years.<br><br>You need to take the tablet first thing in the morning on an empty stomach with a large glass of tap water.<br>After taking the tablet, you should stay upright for the next 30 minutes.<br>You should not eat anything for at least 30 minutes, including taking any other medicines.<br><br>You need to regularly visit your doctor to have your bone health checked.<br>You need to regularly visit a pharmacy for a new supply.<br>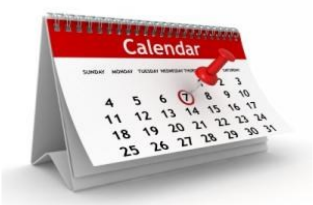 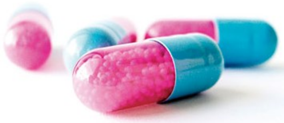

If you take Medicine A, you would live for **3 years** and then die.

If you take Medicine B, you would live for **5 years** and then die.

Would you prefer Medicine A or Medicine B, or are they the same?

Indicate your choice here. You can only choose one option:

◯ **Medicine A** 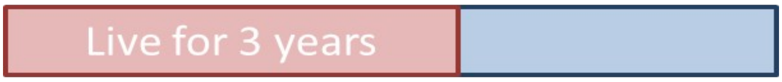

◯ **Medicine B** 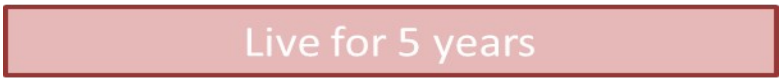

◯ **The Same - Medicine A and Medicine B are the same.**

0% 100%

For peer review only

Repeated section

Remember Medicine A and Medicine B are equally effective and have no risks of a side effect.

The only difference between Medicine A and Medicine B is how you take them.

Medicine A	Medicine B
You take a tablet only once in your life.<br>You will be given the tablet on the day you come in to see your GP.<br>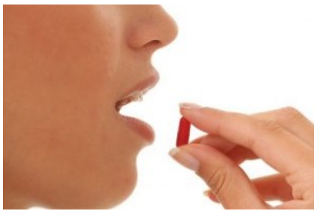	One tablet should be taken once a week on the same day every week.<br>You need to take this medicine for 5 years.<br><br>You need to take the tablet first thing in the morning on an empty stomach with a large glass of tap water.<br>After taking the tablet, you should stay upright for the next 30 minutes.<br>You should not eat anything for at least 30 minutes, including taking any other medicines.<br><br>You need to regularly visit your doctor to have your bone health checked.<br>You need to regularly visit a pharmacy for a new supply.<br>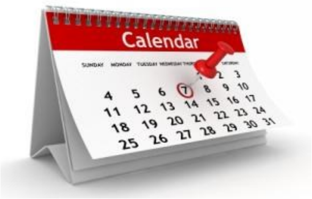 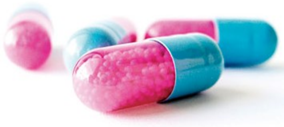

If you take Medicine A, you would live for **3.25 years** (3 years and 3 months) and then die.

If you take Medicine B, you would live for **5 years** and then die.

Would you prefer Medicine A or Medicine B, or are they the same?

Indicate your choice here. You can only choose one option:

◯ **Medicine A**

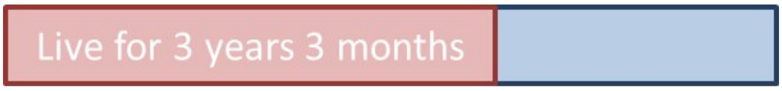

◯ **Medicine B**

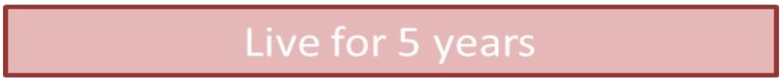

◯ **The Same - Medicine A and Medicine B are the same.**

0% 100%

For peer review only

Repeated section

Remember Medicine A and Medicine B are equally effective and have no risks of a side effect.

The only difference between Medicine A and Medicine B is how you take them.

Medicine A	Medicine B
You take a tablet only once in your life. You will be given the tablet on the day you come in to see your GP. 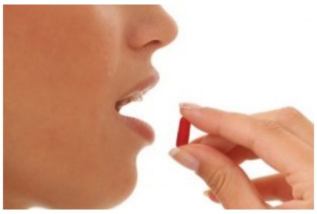	One tablet should be taken once a week on the same day every week. You need to take this medicine for 5 years. <br>You need to take the tablet first thing in the morning on an empty stomach with a large glass of tap water. After taking the tablet, you should stay upright for the next 30 minutes. You should not eat anything for at least 30 minutes, including taking any other medicines. <br>You need to regularly visit your doctor to have your bone health checked. You need to regularly visit a pharmacy for a new supply. 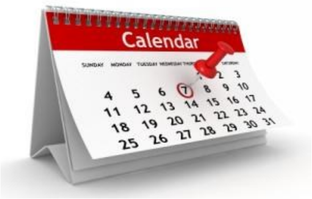 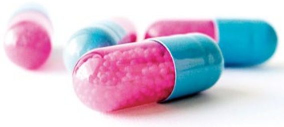

If you take Medicine A, you would live for **3.5 years** (3 years and 6 months) and then die.

If you take Medicine B, you would live for **5 years** and then die.

Would you prefer Medicine A or Medicine B, or are they the same?

Indicate your choice here. You can only choose one option:

◯ **Medicine A** Live for 3 years 6 months

◯ **Medicine B** Live for 5 years

◯ **The Same - Medicine A and Medicine B are the same.**

0% 100%

For peer review only

Repeated section

Remember Medicine A and Medicine B are equally effective and have no risks of a side effect.

The only difference between Medicine A and Medicine B is how you take them.

Medicine A	Medicine B
You take a tablet only once in your life. You will be given the tablet on the day you come in to see your GP.<br>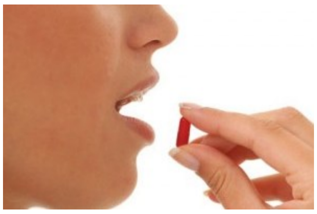	One tablet should be taken once a week on the same day every week.<br>You need to take this medicine for 5 years.<br><br>You need to take the tablet first thing in the morning on an empty stomach with a large glass of tap water.<br>After taking the tablet, you should stay upright for the next 30 minutes.<br>You should not eat anything for at least 30 minutes, including taking any other medicines.<br><br>You need to regularly visit your doctor to have your bone health checked.<br>You need to regularly visit a pharmacy for a new supply.<br>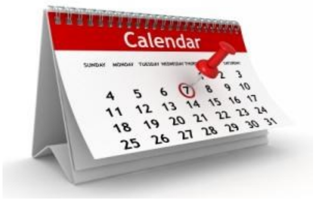 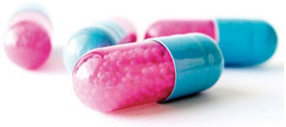

If you take Medicine A, you would live for **3.75 years** (3 years and 9 months) and then die.

If you take Medicine B, you would live for **5 years** and then die.

Would you prefer Medicine A or Medicine B, or are they the same?

Indicate your choice here. You can only choose one option:

◯ **Medicine A** Live for 3 years 9 months

◯ **Medicine B** Live for 5 years

◯ **The Same - Medicine A and Medicine B are the same.**

0% 100%

For peer review only

Repeated section

Remember Medicine A and Medicine B are equally effective and have no risks of a side effect.

The only difference between Medicine A and Medicine B is how you take them.

Medicine A	Medicine B
You take a tablet only once in your life. You will be given the tablet on the day you come in to see your GP.<br>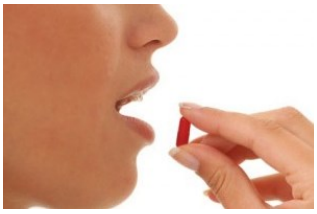	One tablet should be taken once a week on the same day every week.<br>You need to take this medicine for 5 years.<br><br>You need to take the tablet first thing in the morning on an empty stomach with a large glass of tap water.<br>After taking the tablet, you should stay upright for the next 30 minutes.<br>You should not eat anything for at least 30 minutes, including taking any other medicines.<br><br>You need to regularly visit your doctor to have your bone health checked.<br>You need to regularly visit a pharmacy for a new supply.<br>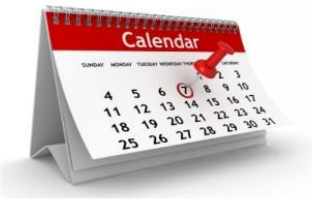 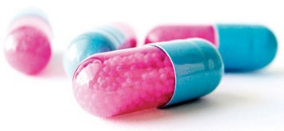

If you take Medicine A, you would live for **3 years and 10 months** and then die.

If you take Medicine B, you would live for **5 years** and then die.

Would you prefer Medicine A or Medicine B, or are they the same?

Indicate your choice here. You can only choose one option:

◯ **Medicine A** Live for 3 years 10 months

◯ **Medicine B** Live for 5 years

◯ **The Same - Medicine A and Medicine B are the same.**

0% 100%

For peer review only

Repeated section

Remember Medicine A and Medicine B are equally effective and have no risks of a side effect.

The only difference between Medicine A and Medicine B is how you take them.

**Medicine A**	**Medicine B**
You take a tablet only once in your life. You will be given the tablet on the day you come in to see your GP. 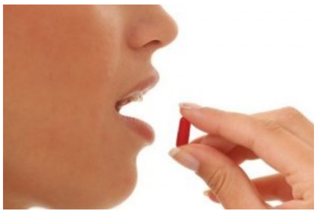	One tablet should be taken once a week on the same day every week. You need to take this medicine for 5 years.<br><br>You need to take the tablet first thing in the morning on an empty stomach with a large glass of tap water. After taking the tablet, you should stay upright for the next 30 minutes. You should not eat anything for at least 30 minutes, including taking any other medicines.<br><br>You need to regularly visit your doctor to have your bone health checked. You need to regularly visit a pharmacy for a new supply.<br>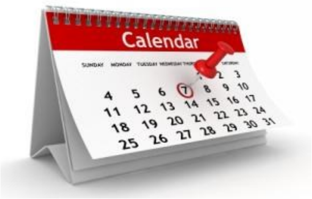 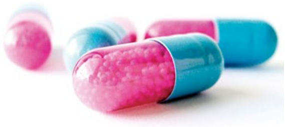

If you take Medicine A, you would live for **4 years** and then die.

If you take Medicine B, you would live for **5 years** and then die.

Would you prefer Medicine A or Medicine B, or are they the same?

Indicate your choice here. You can only choose one option:

◯ **Medicine A** Live for 4 years

◯ **Medicine B** Live for 5 years

◯ **The Same - Medicine A and Medicine B are the same.**

0% 100%

For peer review only

Repeated section

Remember Medicine A and Medicine B are equally effective and have no risks of a side effect.

The only difference between Medicine A and Medicine B is how you take them.

Medicine A	Medicine B
You take a tablet only once in your life. You will be given the tablet on the day you come in to see your GP.<br>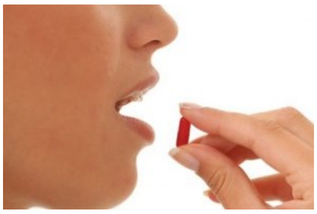	One tablet should be taken once a week on the same day every week.<br>You need to take this medicine for 5 years.<br><br>You need to take the tablet first thing in the morning on an empty stomach with a large glass of tap water.<br>After taking the tablet, you should stay upright for the next 30 minutes.<br>You should not eat anything for at least 30 minutes, including taking any other medicines.<br><br>You need to regularly visit your doctor to have your bone health checked.<br>You need to regularly visit a pharmacy for a new supply.<br>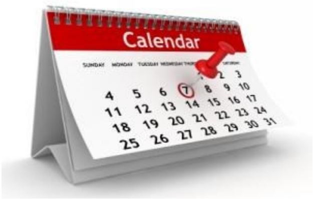 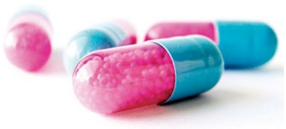

If you take Medicine A, you would live for **4 years and 2 months** and then die.

If you take Medicine B, you would live for **5 years** and then die.

Would you prefer Medicine A or Medicine B, or are they the same?

Indicate your choice here. You can only choose one option:

◯ **Medicine A**

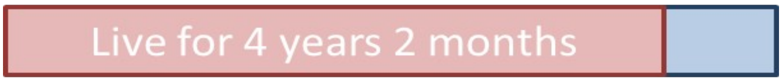

◯ **Medicine B**

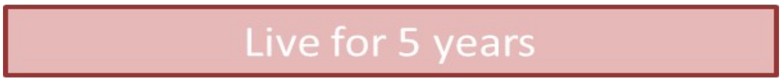

◯ **The Same - Medicine A and Medicine B are the same.**

0% 100%

For peer review only

Repeated section

Remember Medicine A and Medicine B are equally effective and have no risks of a side effect.

The only difference between Medicine A and Medicine B is how you take them.

Medicine A	Medicine B
You take a tablet only once in your life. You will be given the tablet on the day you come in to see your GP.<br>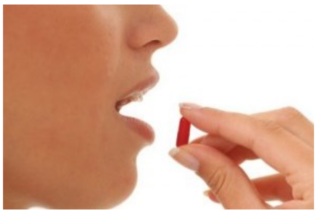	One tablet should be taken once a week on the same day every week.<br>You need to take this medicine for 5 years.<br><br>You need to take the tablet first thing in the morning on an empty stomach with a large glass of tap water.<br>After taking the tablet, you should stay upright for the next 30 minutes.<br>You should not eat anything for at least 30 minutes, including taking any other medicines.<br><br>You need to regularly visit your doctor to have your bone health checked.<br>You need to regularly visit a pharmacy for a new supply.<br>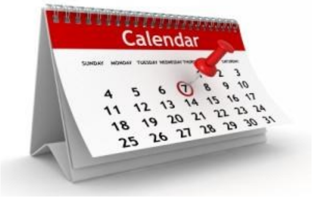 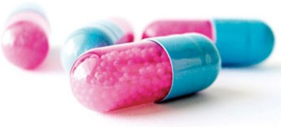

If you take Medicine A, you would live for **4.25 years** (4 years and 3 months) and then die.

If you take Medicine B, you would live for **5 years** and then die.

Would you prefer Medicine A or Medicine B, or are they the same?

Indicate your choice here. You can only choose one option:

◯ **Medicine A** Live for 4 years 3 months

◯ **Medicine B** Live for 5 years

◯ **The Same - Medicine A and Medicine B are the same.**

0% 100%

For peer review only

Repeated section

Remember Medicine A and Medicine B are equally effective and have no risks of a side effect.

The only difference between Medicine A and Medicine B is how you take them.

**Medicine A**	**Medicine B**
You take a tablet only once in your life.<br>You will be given the tablet on the day you come in to see your GP.<br>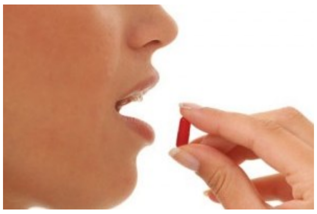	One tablet should be taken once a week on the same day every week.<br>You need to take this medicine for 5 years.<br><br>You need to take the tablet first thing in the morning on an empty stomach with a large glass of tap water.<br>After taking the tablet, you should stay upright for the next 30 minutes.<br>You should not eat anything for at least 30 minutes, including taking any other medicines.<br><br>You need to regularly visit your doctor to have your bone health checked.<br>You need to regularly visit a pharmacy for a new supply.<br>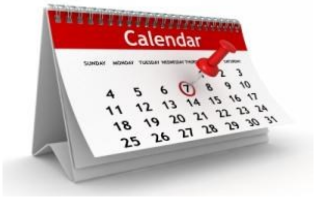 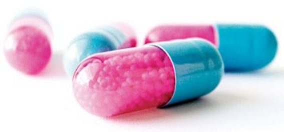

If you take Medicine A, you would live for **4 years 4 months** and then die.

If you take Medicine B, you would live for **5 years** and then die.

Would you prefer Medicine A or Medicine B, or are they the same?

Indicate your choice here. You can only choose one option:

◯ **Medicine A** Live for 4 years 4 months

◯ **Medicine B** Live for 5 years

◯ **The Same - Medicine A and Medicine B are the same.**

0% 100%

For peer review only

Repeated section

Remember Medicine A and Medicine B are equally effective and have no risks of a side effect.

The only difference between Medicine A and Medicine B is how you take them.

Medicine A	Medicine B
You take a tablet only once in your life.<br>You will be given the tablet on the day you come in to see your GP.<br>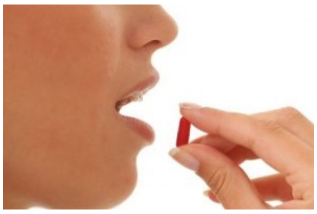	One tablet should be taken once a week on the same day every week.<br>You need to take this medicine for 5 years.<br><br>You need to take the tablet first thing in the morning on an empty stomach with a large glass of tap water.<br>After taking the tablet, you should stay upright for the next 30 minutes.<br>You should not eat anything for at least 30 minutes, including taking any other medicines.<br><br>You need to regularly visit your doctor to have your bone health checked.<br>You need to regularly visit a pharmacy for a new supply.<br>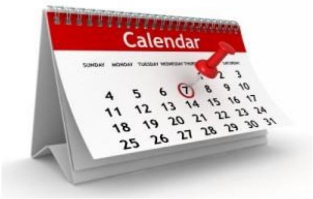 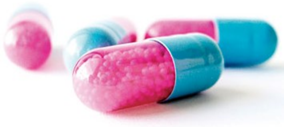

If you take Medicine A, you would live for **4.5 years** (4 years and 6 months) and then die.

If you take Medicine B, you would live for **5 years** and then die.

Would you prefer Medicine A or Medicine B, or are they the same?

Indicate your choice here. You can only choose one option:

◯ **Medicine A** Live for 4 years 6 months

◯ **Medicine B** Live for 5 years

◯ **The Same - Medicine A and Medicine B are the same.**

0% 100%

For peer review only

Repeated section

Remember Medicine A and Medicine B are equally effective and have no risks of a side effect.

The only difference between Medicine A and Medicine B is how you take them.

Medicine A	Medicine B
You take a tablet only once in your life.<br>You will be given the tablet on the day you come in to see your GP.<br>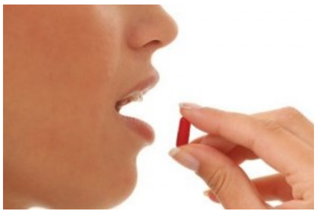	One tablet should be taken once a week on the same day every week.<br>You need to take this medicine for 5 years.<br><br>You need to take the tablet first thing in the morning on an empty stomach with a large glass of tap water.<br>After taking the tablet, you should stay upright for the next 30 minutes.<br>You should not eat anything for at least 30 minutes, including taking any other medicines.<br><br>You need to regularly visit your doctor to have your bone health checked.<br>You need to regularly visit a pharmacy for a new supply.<br>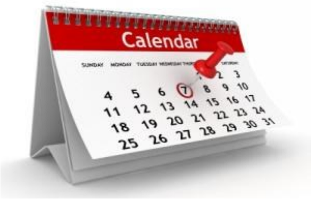 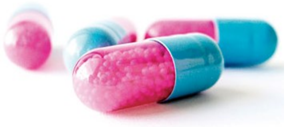

If you take Medicine A, you would live for **4 years and 7 months** and then die.

If you take Medicine B, you would live for **5 years** and then die.

Would you prefer Medicine A or Medicine B, or are they the same?

Indicate your choice here. You can only choose one option:

○ **Medicine A** Live for 4 years 7 months

○ **Medicine B** Live for 5 years

○ **The Same - Medicine A and Medicine B are the same.**

0% 100%

For peer review only

Repeated section

Remember Medicine A and Medicine B are equally effective and have no risks of a side effect.

The only difference between Medicine A and Medicine B is how you take them.

Medicine A	Medicine B
You take a tablet only once in your life. You will be given the tablet on the day you come in to see your GP. 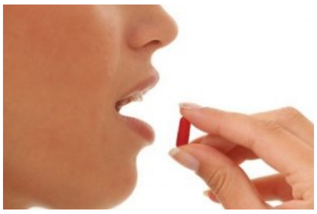	One tablet should be taken once a week on the same day every week. You need to take this medicine for 5 years. You need to take the tablet first thing in the morning on an empty stomach with a large glass of tap water. After taking the tablet, you should stay upright for the next 30 minutes. You should not eat anything for at least 30 minutes, including taking any other medicines. You need to regularly visit your doctor to have your bone health checked. You need to regularly visit a pharmacy for a new supply. 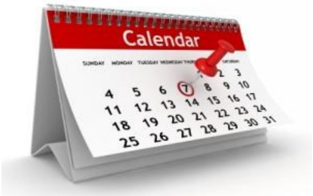 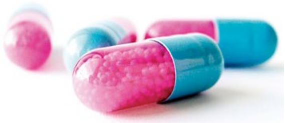

If you take Medicine A, you would live for **4 years and 9 months** and then die.

If you take Medicine B, you would live for **5 years** and then die.

Would you prefer Medicine A or Medicine B, or are they the same?

Indicate your choice here. You can only choose one option:

◯ **Medicine A** Live for 4 years 9 months

◯ **Medicine B** Live for 5 years

◯ **The Same - Medicine A and Medicine B are the same.**

0% 100%

For peer review only

Repeated section

Remember Medicine A and Medicine B are equally effective and have no risks of a side effect.

The only difference between Medicine A and Medicine B is how you take them.

**Medicine A**	**Medicine B**
You take a tablet only once in your life. You will be given the tablet on the day you come in to see your GP.<br>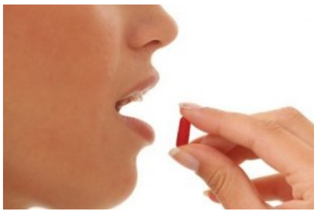	One tablet should be taken once a week on the same day every week.<br>You need to take this medicine for 5 years.<br><br>You need to take the tablet first thing in the morning on an empty stomach with a large glass of tap water.<br>After taking the tablet, you should stay upright for the next 30 minutes.<br>You should not eat anything for at least 30 minutes, including taking any other medicines.<br><br>You need to regularly visit your doctor to have your bone health checked.<br>You need to regularly visit a pharmacy for a new supply.<br>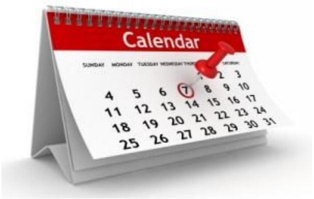 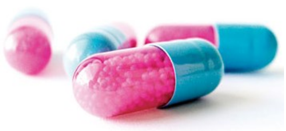

If you take Medicine A, you would live for **2 months less than 5 years** and then die.

If you take Medicine B, you would live for **5 years** and then die.

Would you prefer Medicine A or Medicine B, or are they the same?

Indicate your choice here. You can only choose one option:

◯ **Medicine A** Live for (5 years – 2 months)

◯ **Medicine B** Live for 5 years

◯ **The Same - Medicine A and Medicine B are the same.**

0% 100%

For peer review only

Repeated section

Remember Medicine A and Medicine B are equally effective and have no risks of a side effect.

The only difference between Medicine A and Medicine B is how you take them.

Medicine A	Medicine B
You take a tablet only once in your life.<br>You will be given the tablet on the day you come in to see your GP.<br>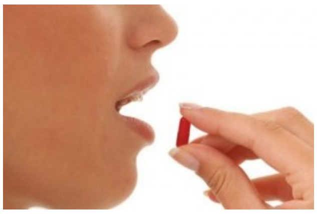	One tablet should be taken once a week on the same day every week.<br>You need to take this medicine for 5 years.<br><br>You need to take the tablet first thing in the morning on an empty stomach with a large glass of tap water.<br>After taking the tablet, you should stay upright for the next 30 minutes.<br>You should not eat anything for at least 30 minutes, including taking any other medicines.<br><br>You need to regularly visit your doctor to have your bone health checked.<br>You need to regularly visit a pharmacy for a new supply.<br>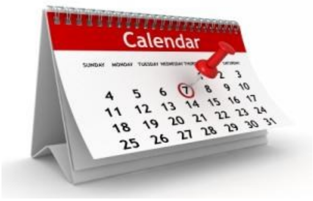 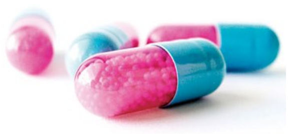

If you take Medicine A, you would live for **1 month less than 5 years** and then die.

If you take Medicine B, you would live for **5 years** and then die.

Would you prefer Medicine A or Medicine B, or are they the same?

Indicate your choice here. You can only choose one option:

◯ **Medicine A** Live for (5 years – 1 month)

◯ **Medicine B** Live for 5 years

◯ **The Same - Medicine A and Medicine B are the same.**

0% 100%

For peer review only

Repeated section

Remember Medicine A and Medicine B are equally effective and have no risks of a side effect.

The only difference between Medicine A and Medicine B is how you take them.

Medicine A	Medicine B
You take a tablet only once in your life.<br>You will be given the tablet on the day you come in to see your GP.<br>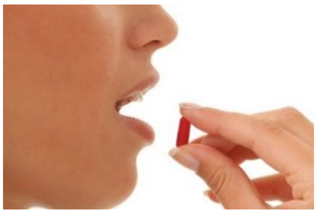	One tablet should be taken once a week on the same day every week.<br>You need to take this medicine for 5 years.<br><br>You need to take the tablet first thing in the morning on an empty stomach with a large glass of tap water.<br>After taking the tablet, you should stay upright for the next 30 minutes.<br>You should not eat anything for at least 30 minutes, including taking any other medicines.<br><br>You need to regularly visit your doctor to have your bone health checked.<br>You need to regularly visit a pharmacy for a new supply.<br>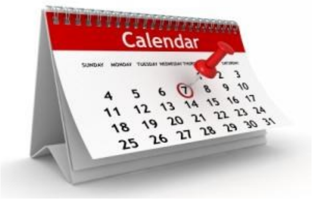 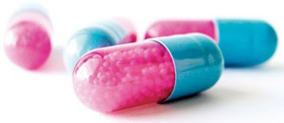

If you take Medicine A, you would live for **2 weeks less than 5 years** and then die.

If you take Medicine B, you would live for **5 years** and then die.

Would you prefer Medicine A or Medicine B, or are they the same?

Indicate your choice here. You can only choose one option:

○ **Medicine A** Live for (5 years – 2 weeks)

○ **Medicine B** Live for 5 years

○ **The Same - Medicine A and Medicine B are the same.**

0% 100%

For peer review only

Repeated section

Remember Medicine A and Medicine B are equally effective and have no risks of a side effect.

The only difference between Medicine A and Medicine B is how you take them.

**Medicine A**	**Medicine B**
You take a tablet only once in your life. You will be given the tablet on the day you come in to see your GP. 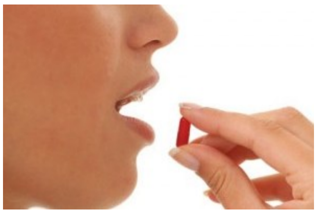	One tablet should be taken once a week on the same day every week. You need to take this medicine for 5 years. You need to take the tablet first thing in the morning on an empty stomach with a large glass of tap water. After taking the tablet, you should stay upright for the next 30 minutes. You should not eat anything for at least 30 minutes, including taking any other medicines. You need to regularly visit your doctor to have your bone health checked. You need to regularly visit a pharmacy for a new supply. 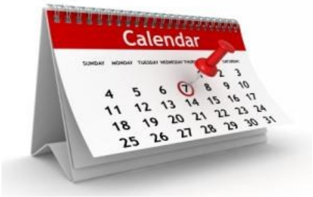 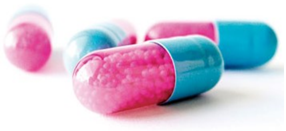

If you take Medicine A, you would live for **1 week less than 5 years** and then die.

If you take Medicine B, you would live for **5 years** and then die.

Would you prefer Medicine A or Medicine B, or are they the same?

Indicate your choice here. You can only choose one option:

◯ **Medicine A** Live for (5 years – 1 week)

◯ **Medicine B** Live for 5 years

◯ **The Same - Medicine A and Medicine B are the same.**

0% 100%

For peer review only

Repeated section

Remember Medicine A and Medicine B are equally effective and have no risks of a side effect.

The only difference between Medicine A and Medicine B is how you take them.

**Medicine A**	**Medicine B**
You take a tablet only once in your life.<br>You will be given the tablet on the day you come in to see your GP.<br>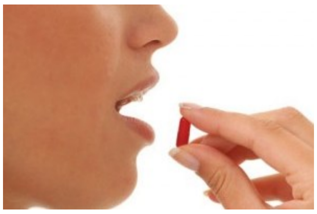	One tablet should be taken once a week on the same day every week.<br>You need to take this medicine for 5 years.<br><br>You need to take the tablet first thing in the morning on an empty stomach with a large glass of tap water.<br>After taking the tablet, you should stay upright for the next 30 minutes.<br>You should not eat anything for at least 30 minutes, including taking any other medicines.<br><br>You need to regularly visit your doctor to have your bone health checked.<br>You need to regularly visit a pharmacy for a new supply.<br>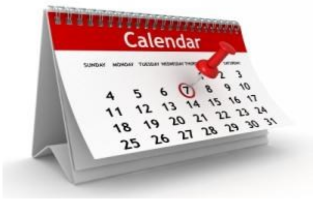 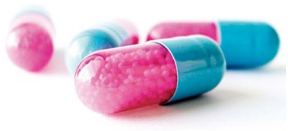

If you take Medicine A, you would live for **5 days less than 5 years** and then die.

If you take Medicine B, you would live for **5 years** and then die.

Would you prefer Medicine A or Medicine B, or are they the same?

Indicate your choice here. You can only choose one option:

◯ **Medicine A** Live for (5 years – 5 days)

◯ **Medicine B** Live for 5 years

◯ **The Same - Medicine A and Medicine B are the same.**

For peer review only

Repeated section

Remember Medicine A and Medicine B are equally effective and have no risks of a side effect.

The only difference between Medicine A and Medicine B is how you take them.

Medicine A	Medicine B
You take a tablet only once in your life. You will be given the tablet on the day you come in to see your GP. 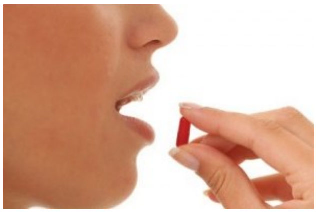	One tablet should be taken once a week on the same day every week.<br>You need to take this medicine for 5 years.<br><br>You need to take the tablet first thing in the morning on an empty stomach with a large glass of tap water.<br>After taking the tablet, you should stay upright for the next 30 minutes.<br>You should not eat anything for at least 30 minutes, including taking any other medicines.<br><br>You need to regularly visit your doctor to have your bone health checked.<br>You need to regularly visit a pharmacy for a new supply.<br>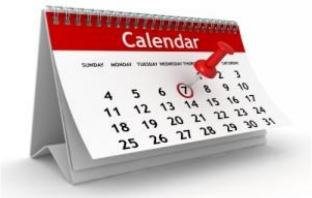 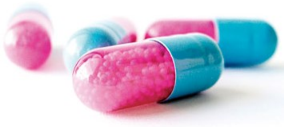

If you take Medicine A, you would live for **3 days less than 5 years** and then die.

If you take Medicine B, you would live for **5 years** and then die.

Would you prefer Medicine A or Medicine B, or are they the same?

Indicate your choice here. You can only choose one option:

◯ **Medicine A** — Live for (5 years – 3 days)

◯ **Medicine B** — Live for 5 years

◯ **The Same - Medicine A and Medicine B are the same.**

0% 100%

For peer review only

Repeated section

Remember Medicine A and Medicine B are equally effective and have no risks of a side effect.

The only difference between Medicine A and Medicine B is how you take them.

**Medicine A**	**Medicine B**
You take a tablet only once in your life.<br>You will be given the tablet on the day you come in to see your GP.<br>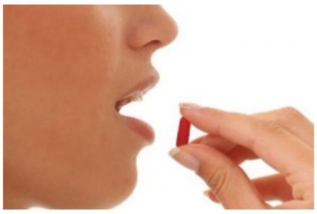	One tablet should be taken once a week on the same day every week.<br>You need to take this medicine for 5 years.<br><br>You need to take the tablet first thing in the morning on an empty stomach with a large glass of tap water.<br>After taking the tablet, you should stay upright for the next 30 minutes.<br>You should not eat anything for at least 30 minutes, including taking any other medicines.<br><br>You need to regularly visit your doctor to have your bone health checked.<br>You need to regularly visit a pharmacy for a new supply.<br>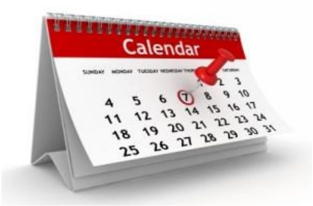 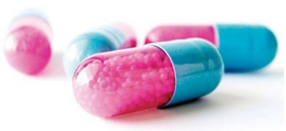

If you take Medicine A, you would live for **1 day less than 5 years** and then die.

If you take Medicine B, you would live for **5 years** and then die.

Would you prefer Medicine A or Medicine B, or are they the same?

Indicate your choice here. You can only choose one option:

- ◯ **Medicine A** — Live for (5 years – 1 day)
- ◯ **Medicine B** — Live for 5 years
- ◯ **The Same - Medicine A and Medicine B are the same.**

0% 100%

For peer review only

Question 4a.

You prefer living for 2.5 years (2 years and 6 months) on Medicine A, to living for 5 years on Medicine B.

This means **living fewer than 2.5 years on Medicine A** can be the same as living for 5 years on Medicine B.

How many years on Medicine A do you think would be the same as living for 5 years on Medicine B?

(Please give a number in years)

0% 100%

peer review only

Was there anything so far which you think is unclear or that you didn't understand?

(Please provide details in the comment box below)

0% 100%

peer review only

Part 2a

Question 5

You have been told that you need to take a medicine to reduce the risk of fracture.

You can choose between two different medicines. We have called these Medicine A and Medicine B.

Medicine A is a pill that you only need to take once.

Medicine B is a pill that you need to take every week for 5 years.

Medicine A and Medicine B are equally effective in reducing your risk of having a fracture.
Medicine A and Medicine B will reduce your risk of experiencing a fracture, such as hip fracture, within the next 5 years from **10%** to **6%**.

In other words, in a crowd of **1,000** people in the UK, **100** are likely to have a fracture if they do not take the medicine. This is reduced to **60** people out of 1,000 who do take the medicine.
This information is also illustrated in this picture.

Risk of fracture

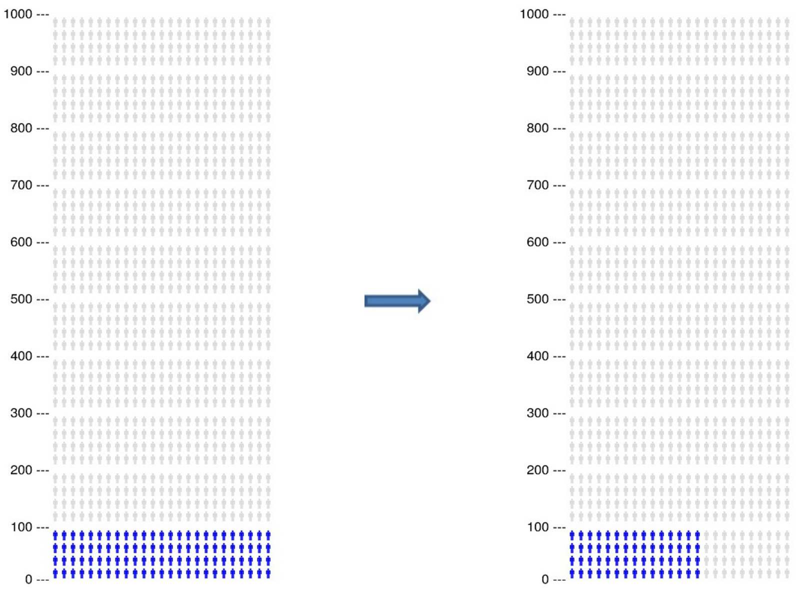

10%
No Treatment

6%
Taking the Medicine

(The figures shaded blue represent people who will have a fracture, such as hip fracture, within the next 5 years)

Please continue to the next page.

0% 100%

Repeated section

Remember Medicine A and Medicine B are equally effective.

However, there are some differences between Medicine A and Medicine B.

**Medicine A**	**Medicine B**
**No inconvenience**<br><br>You take a tablet only once in your life.<br>You will be given the tablet on the day you come in to see your GP.	**Inconvenience**<br><br>One tablet should be taken once a week on the same day every week.<br>You need to take this medicine for 5 years.<br><br>You need to take the tablet first thing in the morning on an empty stomach with a large glass of tap water.<br>After taking the tablet, you should stay upright for the next 30 minutes.<br><br>You should not eat anything for at least 30 minutes, including taking any other medicines.<br><br>You need to regularly visit your doctor to have your bone health checked.<br>You need to regularly visit a pharmacy for a new supply.
**Severe side effect - 0%**<br><br>No one taking Medicine A will have a severe side effect.	**Severe side effect - 0.7%**<br><br>Among every 1,000 people taking Medicine B, **7** will experience a severe side effect.
**Minor side effect - 0%**<br><br>No one taking Medicine A will have a minor side effect.	**Minor side effect - 13%**<br><br>Among every 1,000 people taking Medicine B, **130** will experience a minor side effect.

If you take Medicine A, you would live for **2.5 years (2 years and 6 months)** and then die.

If you take Medicine B, you would live for **5 years** and then die.

Would you prefer Medicine A or Medicine B, or are they the same?

Indicate your choice here. You can only choose one option:

○ **Medicine A**

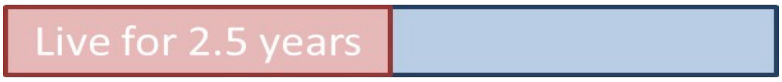

○ **Medicine B**

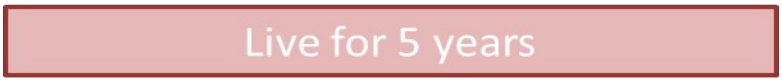

○ **The Same - Medicine A and Medicine B are the same.**

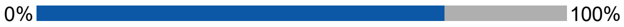

For peer review only

Repeated section

Remember Medicine A and Medicine B are equally effective.

However, there are some differences between Medicine A and Medicine B.

Medicine A	Medicine B
**No inconvenience**<br><br>You take a tablet only once in your life.<br>You will be given the tablet on the day you come in to see your GP.	**Inconvenience**<br><br>One tablet should be taken once a week on the same day every week.<br>You need to take this medicine for 5 years.<br><br>You need to take the tablet first thing in the morning on an empty stomach with a large glass of tap water.<br>After taking the tablet, you should stay upright for the next 30 minutes.<br><br>You should not eat anything for at least 30 minutes, including taking any other medicines.<br><br>You need to regularly visit your doctor to have your bone health checked.<br>You need to regularly visit a pharmacy for a new supply.
**Severe side effect - 0%**<br><br>No one taking Medicine A will have a severe side effect.	**Severe side effect - 0.7%**<br><br>Among every 1,000 people taking Medicine B, **7** will experience a severe side effect.
**Minor side effect - 0%**<br><br>No one taking Medicine A will have a minor side effect.	**Minor side effect - 13%**<br><br>Among every 1,000 people taking Medicine B, **130** will experience a minor side effect.

If you take Medicine A, you would live for **3 years** and then die.

If you take Medicine B, you would live for **5 years** and then die.

Would you prefer Medicine A or Medicine B, or are they the same?

Indicate your choice here. You can only choose one option:

○ **Medicine A** — Live for 3 years

○ **Medicine B** — Live for 5 years

○ **The Same - Medicine A and Medicine B are the same.**

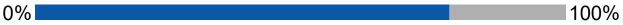

For peer review only

Repeated section

Remember Medicine A and Medicine B are equally effective.

However, there are some differences between Medicine A and Medicine B.

Medicine A	Medicine B
**No inconvenience**<br><br>You take a tablet only once in your life.<br>You will be given the tablet on the day you come in to see your GP.	**Inconvenience**<br><br>One tablet should be taken once a week on the same day every week.<br>You need to take this medicine for 5 years.<br><br>You need to take the tablet first thing in the morning on an empty stomach with a large glass of tap water.<br>After taking the tablet, you should stay upright for the next 30 minutes.<br><br>You should not eat anything for at least 30 minutes, including taking any other medicines.<br><br>You need to regularly visit your doctor to have your bone health checked.<br>You need to regularly visit a pharmacy for a new supply.
**Severe side effect - 0%**<br><br>No one taking Medicine A will have a severe side effect.	**Severe side effect - 0.7%**<br><br>Among every 1,000 people taking Medicine B, **7** will experience a severe side effect.
**Minor side effect - 0%**<br><br>No one taking Medicine A will have a minor side effect.	**Minor side effect - 13%**<br><br>Among every 1,000 people taking Medicine B, **130** will experience a minor side effect.

If you take Medicine A, you would live for **3.25 years** (3 years and 3 months) and then die.

If you take Medicine B, you would live for **5 years** and then die.

Would you prefer Medicine A or Medicine B, or are they the same?

Indicate your choice here. You can only choose one option:

◯ **Medicine A** 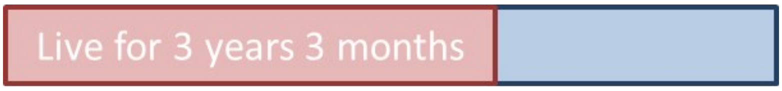

◯ **Medicine B** 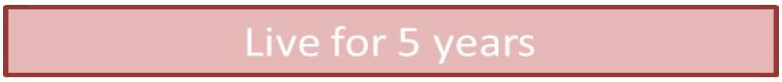

◯ **The Same - Medicine A and Medicine B are the same.**

0% 100%

For peer review only

Repeated section

Remember Medicine A and Medicine B are equally effective.

However, there are some differences between Medicine A and Medicine B.

Medicine A	Medicine B
**No inconvenience**<br><br>You take a tablet only once in your life.<br>You will be given the tablet on the day you come in to see your GP.	**Inconvenience**<br><br>One tablet should be taken once a week on the same day every week.<br>You need to take this medicine for 5 years.<br><br>You need to take the tablet first thing in the morning on an empty stomach with a large glass of tap water.<br>After taking the tablet, you should stay upright for the next 30 minutes.<br><br>You should not eat anything for at least 30 minutes, including taking any other medicines.<br><br>You need to regularly visit your doctor to have your bone health checked.<br>You need to regularly visit a pharmacy for a new supply.
**Severe side effect - 0%**<br><br>No one taking Medicine A will have a severe side effect.	**Severe side effect - 0.7%**<br><br>Among every 1,000 people taking Medicine B, **7** will experience a severe side effect.
**Minor side effect - 0%**<br><br>No one taking Medicine A will have a minor side effect.	**Minor side effect - 13%**<br><br>Among every 1,000 people taking Medicine B, **130** will experience a minor side effect.

If you take Medicine A, you would live for **3.5 years** (3 years and 6 months) and then die.

If you take Medicine B, you would live for **5 years** and then die.

Would you prefer Medicine A or Medicine B, or are they the same?

Indicate your choice here. You can only choose one option:

◯ **Medicine A**

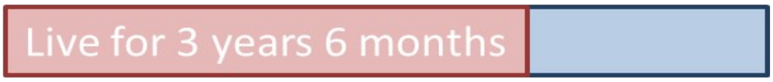

◯ **Medicine B**

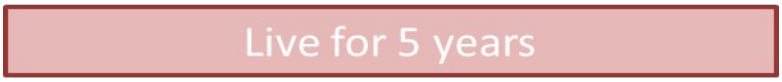

◯ **The Same - Medicine A and Medicine B are the same.**

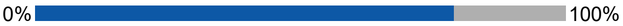

For peer review only

Repeated section

Remember Medicine A and Medicine B are equally effective.

However, there are some differences between Medicine A and Medicine B.

Medicine A	Medicine B
**No inconvenience**<br><br>You take a tablet only once in your life.<br>You will be given the tablet on the day you come in to see your GP.	**Inconvenience**<br><br>One tablet should be taken once a week on the same day every week.<br>You need to take this medicine for 5 years.<br><br>You need to take the tablet first thing in the morning on an empty stomach with a large glass of tap water.<br>After taking the tablet, you should stay upright for the next 30 minutes.<br><br>You should not eat anything for at least 30 minutes, including taking any other medicines.<br><br>You need to regularly visit your doctor to have your bone health checked.<br>You need to regularly visit a pharmacy for a new supply.
**Severe side effect - 0%**<br><br>No one taking Medicine A will have a severe side effect.	**Severe side effect - 0.7%**<br><br>Among every 1,000 people taking Medicine B, **7** will experience a severe side effect.
**Minor side effect - 0%**<br><br>No one taking Medicine A will have a minor side effect.	**Minor side effect - 13%**<br><br>Among every 1,000 people taking Medicine B, **130** will experience a minor side effect.

If you take Medicine A, you would live for **3.75 years** (3 years and 9 months) and then die.

If you take Medicine B, you would live for **5 years** and then die.

Would you prefer Medicine A or Medicine B, or are they the same?

Indicate your choice here. You can only choose one option:

◯ **Medicine A**

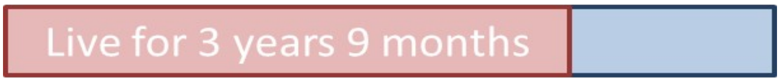

◯ **Medicine B**

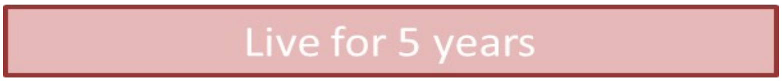

◯ **The Same - Medicine A and Medicine B are the same.**

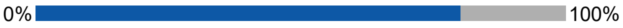

For peer review only

Repeated section

Remember Medicine A and Medicine B are equally effective.

However, there are some differences between Medicine A and Medicine B.

Medicine A	Medicine B
**No inconvenience**<br><br>You take a tablet only once in your life.<br>You will be given the tablet on the day you come in to see your GP.	**Inconvenience**<br><br>One tablet should be taken once a week on the same day every week.<br>You need to take this medicine for 5 years.<br><br>You need to take the tablet first thing in the morning on an empty stomach with a large glass of tap water.<br>After taking the tablet, you should stay upright for the next 30 minutes.<br><br>You should not eat anything for at least 30 minutes, including taking any other medicines.<br><br>You need to regularly visit your doctor to have your bone health checked.<br>You need to regularly visit a pharmacy for a new supply.
**Severe side effect - 0%**<br><br>No one taking Medicine A will have a severe side effect.	**Severe side effect - 0.7%**<br><br>Among every 1,000 people taking Medicine B, **7** will experience a severe side effect.
**Minor side effect - 0%**<br><br>No one taking Medicine A will have a minor side effect.	**Minor side effect - 13%**<br><br>Among every 1,000 people taking Medicine B, **130** will experience a minor side effect.

If you take Medicine A, you would live for **3 years and 10 months** and then die.

If you take Medicine B, you would live for **5 years** and then die.

Would you prefer Medicine A or Medicine B, or are they the same?

Indicate your choice here. You can only choose one option:

○ **Medicine A** 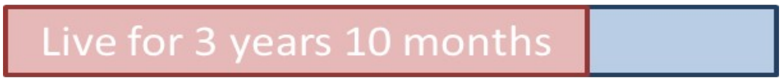

○ **Medicine B** 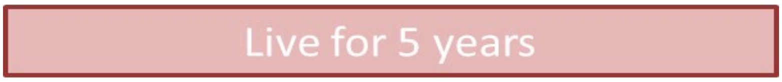

○ **The Same - Medicine A and Medicine B are the same.**

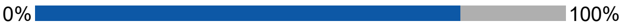

For peer review only

Repeated section

Remember Medicine A and Medicine B are equally effective.

However, there are some differences between Medicine A and Medicine B.

Medicine A	Medicine B
**No inconvenience**<br><br>You take a tablet only once in your life.<br>You will be given the tablet on the day you come in to see your GP.	**Inconvenience**<br><br>One tablet should be taken once a week on the same day every week.<br>You need to take this medicine for 5 years.<br><br>You need to take the tablet first thing in the morning on an empty stomach with a large glass of tap water.<br>After taking the tablet, you should stay upright for the next 30 minutes.<br><br>You should not eat anything for at least 30 minutes, including taking any other medicines.<br><br>You need to regularly visit your doctor to have your bone health checked.<br>You need to regularly visit a pharmacy for a new supply.
**Severe side effect - 0%**<br><br>No one taking Medicine A will have a severe side effect.	**Severe side effect - 0.7%**<br><br>Among every 1,000 people taking Medicine B, **7** will experience a severe side effect.
**Minor side effect - 0%**<br><br>No one taking Medicine A will have a minor side effect.	**Minor side effect - 13%**<br><br>Among every 1,000 people taking Medicine B, **130** will experience a minor side effect.

If you take Medicine A, you would live for **4 years** and then die.

If you take Medicine B, you would live for **5 years** and then die.

Would you prefer Medicine A or Medicine B, or are they the same?

Indicate your choice here. You can only choose one option:

◯ **Medicine A**

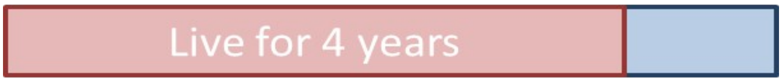

◯ **Medicine B**

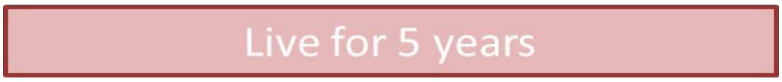

◯ **The Same - Medicine A and Medicine B are the same.**

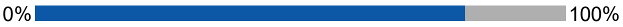

For peer review only

Repeated section

Remember Medicine A and Medicine B are equally effective.

However, there are some differences between Medicine A and Medicine B.

Medicine A	Medicine B
**No inconvenience**<br><br>You take a tablet only once in your life.<br>You will be given the tablet on the day you come in to see your GP.	**Inconvenience**<br><br>One tablet should be taken once a week on the same day every week.<br>You need to take this medicine for 5 years.<br><br>You need to take the tablet first thing in the morning on an empty stomach with a large glass of tap water.<br>After taking the tablet, you should stay upright for the next 30 minutes.<br><br>You should not eat anything for at least 30 minutes, including taking any other medicines.<br><br>You need to regularly visit your doctor to have your bone health checked.<br>You need to regularly visit a pharmacy for a new supply.
**Severe side effect - 0%**<br><br>No one taking Medicine A will have a severe side effect.	**Severe side effect - 0.7%**<br><br>Among every 1,000 people taking Medicine B, **7** will experience a severe side effect.
**Minor side effect - 0%**<br><br>No one taking Medicine A will have a minor side effect.	**Minor side effect - 13%**<br><br>Among every 1,000 people taking Medicine B, **130** will experience a minor side effect.

If you take Medicine A, you would live for **4 years and 2 months** and then die.

If you take Medicine B, you would live for **5 years** and then die.

Would you prefer Medicine A or Medicine B, or are they the same?

Indicate your choice here. You can only choose one option:

○ **Medicine A**

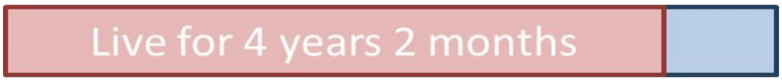

○ **Medicine B**

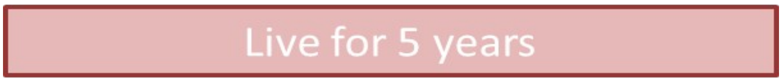

○ **The Same - Medicine A and Medicine B are the same.**

0% 100%

For peer review only

Repeated section

Remember Medicine A and Medicine B are equally effective.

However, there are some differences between Medicine A and Medicine B.

Medicine A	Medicine B
**No inconvenience**<br><br>You take a tablet only once in your life.<br>You will be given the tablet on the day you come in to see your GP.	**Inconvenience**<br><br>One tablet should be taken once a week on the same day every week.<br>You need to take this medicine for 5 years.<br><br>You need to take the tablet first thing in the morning on an empty stomach with a large glass of tap water.<br>After taking the tablet, you should stay upright for the next 30 minutes.<br><br>You should not eat anything for at least 30 minutes, including taking any other medicines.<br><br>You need to regularly visit your doctor to have your bone health checked.<br>You need to regularly visit a pharmacy for a new supply.
**Severe side effect - 0%**<br><br>No one taking Medicine A will have a severe side effect.	**Severe side effect - 0.7%**<br><br>Among every 1,000 people taking Medicine B, **7** will experience a severe side effect.
**Minor side effect - 0%**<br><br>No one taking Medicine A will have a minor side effect.	**Minor side effect - 13%**<br><br>Among every 1,000 people taking Medicine B, **130** will experience a minor side effect.

If you take Medicine A, you would live for **4.25 years** (4 years and 3 months) and then die.

If you take Medicine B, you would live for **5 years** and then die.

Would you prefer Medicine A or Medicine B, or are they the same?

Indicate your choice here. You can only choose one option:

◯ **Medicine A** 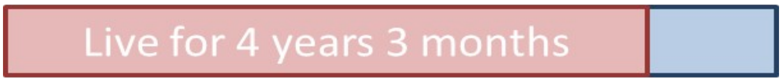

◯ **Medicine B** 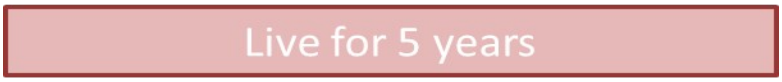

◯ **The Same - Medicine A and Medicine B are the same.**

0% 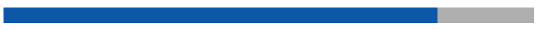 100%

For peer review only

Repeated section

Remember Medicine A and Medicine B are equally effective.

However, there are some differences between Medicine A and Medicine B.

Medicine A	Medicine B
**No inconvenience**<br><br>You take a tablet only once in your life.<br>You will be given the tablet on the day you come in to see your GP.	**Inconvenience**<br><br>One tablet should be taken once a week on the same day every week.<br>You need to take this medicine for 5 years.<br><br>You need to take the tablet first thing in the morning on an empty stomach with a large glass of tap water.<br>After taking the tablet, you should stay upright for the next 30 minutes.<br><br>You should not eat anything for at least 30 minutes, including taking any other medicines.<br><br>You need to regularly visit your doctor to have your bone health checked.<br>You need to regularly visit a pharmacy for a new supply.
**Severe side effect - 0%**<br><br>No one taking Medicine A will have a severe side effect.	**Severe side effect - 0.7%**<br><br>Among every 1,000 people taking Medicine B, **7** will experience a severe side effect.
**Minor side effect - 0%**<br><br>No one taking Medicine A will have a minor side effect.	**Minor side effect - 13%**<br><br>Among every 1,000 people taking Medicine B, **130** will experience a minor side effect.

If you take Medicine A, you would live for **4 years 4 months** and then die.

If you take Medicine B, you would live for **5 years** and then die.

Would you prefer Medicine A or Medicine B, or are they the same?

Indicate your choice here. You can only choose one option:

◯ **Medicine A**

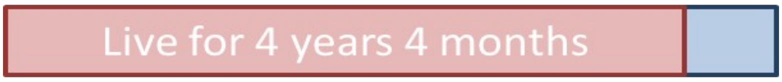

◯ **Medicine B**

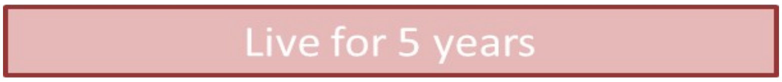

◯ **The Same - Medicine A and Medicine B are the same.**

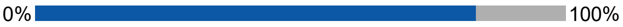

For peer review only

Repeated section

Remember Medicine A and Medicine B are equally effective.

However, there are some differences between Medicine A and Medicine B.

Medicine A	Medicine B
**No inconvenience**<br><br>You take a tablet only once in your life.<br>You will be given the tablet on the day you come in to see your GP.	**Inconvenience**<br><br>One tablet should be taken once a week on the same day every week.<br>You need to take this medicine for 5 years.<br><br>You need to take the tablet first thing in the morning on an empty stomach with a large glass of tap water.<br>After taking the tablet, you should stay upright for the next 30 minutes.<br><br>You should not eat anything for at least 30 minutes, including taking any other medicines.<br><br>You need to regularly visit your doctor to have your bone health checked.<br>You need to regularly visit a pharmacy for a new supply.
**Severe side effect - 0%**<br><br>No one taking Medicine A will have a severe side effect.	**Severe side effect - 0.7%**<br><br>Among every 1,000 people taking Medicine B, **7** will experience a severe side effect.
**Minor side effect - 0%**<br><br>No one taking Medicine A will have a minor side effect.	**Minor side effect - 13%**<br><br>Among every 1,000 people taking Medicine B, **130** will experience a minor side effect.

If you take Medicine A, you would live for **4.5 years** (4 years and 6 months) and then die.

If you take Medicine B, you would live for **5 years** and then die.

Would you prefer Medicine A or Medicine B, or are they the same?

Indicate your choice here. You can only choose one option:

○ **Medicine A** Live for 4 years 6 months

○ **Medicine B** Live for 5 years

○ **The Same - Medicine A and Medicine B are the same.**

0% 100%

For peer review only

Repeated section

Remember Medicine A and Medicine B are equally effective.

However, there are some differences between Medicine A and Medicine B.

Medicine A	Medicine B
**No inconvenience**<br><br>You take a tablet only once in your life.<br>You will be given the tablet on the day you come in to see your GP.	**Inconvenience**<br><br>One tablet should be taken once a week on the same day every week.<br>You need to take this medicine for 5 years.<br><br>You need to take the tablet first thing in the morning on an empty stomach with a large glass of tap water.<br>After taking the tablet, you should stay upright for the next 30 minutes.<br><br>You should not eat anything for at least 30 minutes, including taking any other medicines.<br><br>You need to regularly visit your doctor to have your bone health checked.<br>You need to regularly visit a pharmacy for a new supply.
**Severe side effect - 0%**<br><br>No one taking Medicine A will have a severe side effect.	**Severe side effect - 0.7%**<br><br>Among every 1,000 people taking Medicine B, **7** will experience a severe side effect.
**Minor side effect - 0%**<br><br>No one taking Medicine A will have a minor side effect.	**Minor side effect - 13%**<br><br>Among every 1,000 people taking Medicine B, **130** will experience a minor side effect.

If you take Medicine A, you would live for **4 years and 7 months** and then die.

If you take Medicine B, you would live for **5 years** and then die.

Would you prefer Medicine A or Medicine B, or are they the same?

Indicate your choice here. You can only choose one option:

◯ **Medicine A**

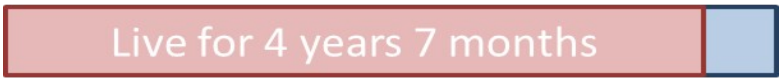

◯ **Medicine B**

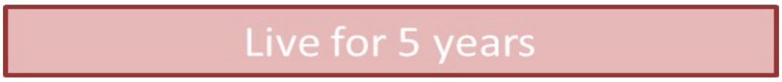

◯ **The Same - Medicine A and Medicine B are the same.**

0% 100%

For peer review only

Repeated section

Remember Medicine A and Medicine B are equally effective.

However, there are some differences between Medicine A and Medicine B.

Medicine A	Medicine B
**No inconvenience**<br><br>You take a tablet only once in your life.<br>You will be given the tablet on the day you come in to see your GP.	**Inconvenience**<br><br>One tablet should be taken once a week on the same day every week.<br>You need to take this medicine for 5 years.<br><br>You need to take the tablet first thing in the morning on an empty stomach with a large glass of tap water.<br>After taking the tablet, you should stay upright for the next 30 minutes.<br><br>You should not eat anything for at least 30 minutes, including taking any other medicines.<br><br>You need to regularly visit your doctor to have your bone health checked.<br>You need to regularly visit a pharmacy for a new supply.
**Severe side effect - 0%**<br><br>No one taking Medicine A will have a severe side effect.	**Severe side effect - 0.7%**<br><br>Among every 1,000 people taking Medicine B, **7** will experience a severe side effect.
**Minor side effect - 0%**<br><br>No one taking Medicine A will have a minor side effect.	**Minor side effect - 13%**<br><br>Among every 1,000 people taking Medicine B, **130** will experience a minor side effect.

If you take Medicine A, you would live for **4 years and 9 months** and then die.

If you take Medicine B, you would live for **5 years** and then die.

Would you prefer Medicine A or Medicine B, or are they the same?

Indicate your choice here. You can only choose one option:

◯ **Medicine A** Live for 4 years 9 months

◯ **Medicine B** Live for 5 years

◯ **The Same - Medicine A and Medicine B are the same.**

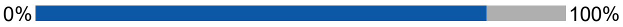

For peer review only

Repeated section

Remember Medicine A and Medicine B are equally effective.

However, there are some differences between Medicine A and Medicine B.

Medicine A	Medicine B
**No inconvenience**<br><br>You take a tablet only once in your life.<br>You will be given the tablet on the day you come in to see your GP.	**Inconvenience**<br><br>One tablet should be taken once a week on the same day every week.<br>You need to take this medicine for 5 years.<br><br>You need to take the tablet first thing in the morning on an empty stomach with a large glass of tap water.<br>After taking the tablet, you should stay upright for the next 30 minutes.<br><br>You should not eat anything for at least 30 minutes, including taking any other medicines.<br><br>You need to regularly visit your doctor to have your bone health checked.<br>You need to regularly visit a pharmacy for a new supply.
**Severe side effect - 0%**<br><br>No one taking Medicine A will have a severe side effect.	**Severe side effect - 0.7%**<br><br>Among every 1,000 people taking Medicine B, **7** will experience a severe side effect.
**Minor side effect - 0%**<br><br>No one taking Medicine A will have a minor side effect.	**Minor side effect - 13%**<br><br>Among every 1,000 people taking Medicine B, **130** will experience a minor side effect.

If you take Medicine A, you would live for **2 months less than 5 years** and then die.

If you take Medicine B, you would live for **5 years** and then die.

Would you prefer Medicine A or Medicine B, or are they the same?

Indicate your choice here. You can only choose one option:

○ **Medicine A** Live for (5 years – 2 months)

○ **Medicine B** Live for 5 years

○ **The Same - Medicine A and Medicine B are the same.**

0% 100%

For peer review only

Repeated section

Remember Medicine A and Medicine B are equally effective.

However, there are some differences between Medicine A and Medicine B.

Medicine A	Medicine B
**No inconvenience**<br><br>You take a tablet only once in your life.<br>You will be given the tablet on the day you come in to see your GP.	**Inconvenience**<br><br>One tablet should be taken once a week on the same day every week.<br>You need to take this medicine for 5 years.<br><br>You need to take the tablet first thing in the morning on an empty stomach with a large glass of tap water.<br>After taking the tablet, you should stay upright for the next 30 minutes.<br><br>You should not eat anything for at least 30 minutes, including taking any other medicines.<br><br>You need to regularly visit your doctor to have your bone health checked.<br>You need to regularly visit a pharmacy for a new supply.
**Severe side effect - 0%**<br><br>No one taking Medicine A will have a severe side effect.	**Severe side effect - 0.7%**<br><br>Among every 1,000 people taking Medicine B, **7** will experience a severe side effect.
**Minor side effect - 0%**<br><br>No one taking Medicine A will have a minor side effect.	**Minor side effect - 13%**<br><br>Among every 1,000 people taking Medicine B, **130** will experience a minor side effect.

If you take Medicine A, you would live for **1 month less than 5 years** and then die.

If you take Medicine B, you would live for **5 years** and then die.

Would you prefer Medicine A or Medicine B, or are they the same?

Indicate your choice here. You can only choose one option:

◯ **Medicine A** 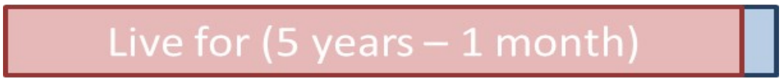

◯ **Medicine B** 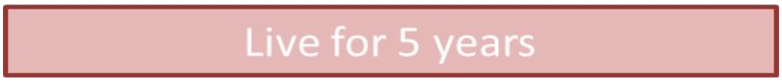

◯ **The Same - Medicine A and Medicine B are the same.**

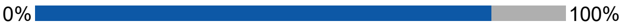

For peer review only

Repeated section

Remember Medicine A and Medicine B are equally effective.

However, there are some differences between Medicine A and Medicine B.

Medicine A	Medicine B
**No inconvenience**<br><br>You take a tablet only once in your life.<br>You will be given the tablet on the day you come in to see your GP.	**Inconvenience**<br><br>One tablet should be taken once a week on the same day every week.<br>You need to take this medicine for 5 years.<br><br>You need to take the tablet first thing in the morning on an empty stomach with a large glass of tap water.<br>After taking the tablet, you should stay upright for the next 30 minutes.<br><br>You should not eat anything for at least 30 minutes, including taking any other medicines.<br><br>You need to regularly visit your doctor to have your bone health checked.<br>You need to regularly visit a pharmacy for a new supply.
**Severe side effect - 0%**<br><br>No one taking Medicine A will have a severe side effect.	**Severe side effect - 0.7%**<br><br>Among every 1,000 people taking Medicine B, **7** will experience a severe side effect.
**Minor side effect - 0%**<br><br>No one taking Medicine A will have a minor side effect.	**Minor side effect - 13%**<br><br>Among every 1,000 people taking Medicine B, **130** will experience a minor side effect.

If you take Medicine A, you would live for **2 weeks less than 5 years** and then die.

If you take Medicine B, you would live for **5 years** and then die.

Would you prefer Medicine A or Medicine B, or are they the same?

Indicate your choice here. You can only choose one option:

◯ **Medicine A** Live for (5 years – 2 weeks)

◯ **Medicine B** Live for 5 years

◯ **The Same - Medicine A and Medicine B are the same.**

0% 100%

For peer review only

Repeated section

Remember Medicine A and Medicine B are equally effective.

However, there are some differences between Medicine A and Medicine B.

Medicine A	Medicine B
**No inconvenience**<br><br>You take a tablet only once in your life.<br>You will be given the tablet on the day you come in to see your GP.	**Inconvenience**<br><br>One tablet should be taken once a week on the same day every week.<br>You need to take this medicine for 5 years.<br><br>You need to take the tablet first thing in the morning on an empty stomach with a large glass of tap water.<br>After taking the tablet, you should stay upright for the next 30 minutes.<br><br>You should not eat anything for at least 30 minutes, including taking any other medicines.<br><br>You need to regularly visit your doctor to have your bone health checked.<br>You need to regularly visit a pharmacy for a new supply.
**Severe side effect - 0%**<br><br>No one taking Medicine A will have a severe side effect.	**Severe side effect - 0.7%**<br><br>Among every 1,000 people taking Medicine B, **7** will experience a severe side effect.
**Minor side effect - 0%**<br><br>No one taking Medicine A will have a minor side effect.	**Minor side effect - 13%**<br><br>Among every 1,000 people taking Medicine B, **130** will experience a minor side effect.

If you take Medicine A, you would live for **1 week less than 5 years** and then die.

If you take Medicine B, you would live for **5 years** and then die.

Would you prefer Medicine A or Medicine B, or are they the same?

Indicate your choice here. You can only choose one option:

○ **Medicine A** Live for (5 years – 1 week)

○ **Medicine B** Live for 5 years

○ **The Same - Medicine A and Medicine B are the same.**

0% 100%

For peer review only

Repeated section

Remember Medicine A and Medicine B are equally effective.

However, there are some differences between Medicine A and Medicine B.

Medicine A	Medicine B
**No inconvenience**<br><br>You take a tablet only once in your life.<br>You will be given the tablet on the day you come in to see your GP.	**Inconvenience**<br><br>One tablet should be taken once a week on the same day every week.<br>You need to take this medicine for 5 years.<br><br>You need to take the tablet first thing in the morning on an empty stomach with a large glass of tap water.<br>After taking the tablet, you should stay upright for the next 30 minutes.<br><br>You should not eat anything for at least 30 minutes, including taking any other medicines.<br><br>You need to regularly visit your doctor to have your bone health checked.<br>You need to regularly visit a pharmacy for a new supply.
**Severe side effect - 0%**<br><br>No one taking Medicine A will have a severe side effect.	**Severe side effect - 0.7%**<br><br>Among every 1,000 people taking Medicine B, **7** will experience a severe side effect.
**Minor side effect - 0%**<br><br>No one taking Medicine A will have a minor side effect.	**Minor side effect - 13%**<br><br>Among every 1,000 people taking Medicine B, **130** will experience a minor side effect.

If you take Medicine A, you would live for **5 days less than 5 years** and then die.

If you take Medicine B, you would live for **5 years** and then die.

Would you prefer Medicine A or Medicine B, or are they the same?

Indicate your choice here. You can only choose one option:

○ **Medicine A** Live for (5 years – 5 days)

○ **Medicine B** Live for 5 years

○ **The Same - Medicine A and Medicine B are the same.**

0% 100%

For peer review only

Repeated section

Remember Medicine A and Medicine B are equally effective.

However, there are some differences between Medicine A and Medicine B.

Medicine A	Medicine B
**No inconvenience**<br><br>You take a tablet only once in your life.<br>You will be given the tablet on the day you come in to see your GP.	**Inconvenience**<br><br>One tablet should be taken once a week on the same day every week.<br>You need to take this medicine for 5 years.<br><br>You need to take the tablet first thing in the morning on an empty stomach with a large glass of tap water.<br>After taking the tablet, you should stay upright for the next 30 minutes.<br><br>You should not eat anything for at least 30 minutes, including taking any other medicines.<br><br>You need to regularly visit your doctor to have your bone health checked.<br>You need to regularly visit a pharmacy for a new supply.
**Severe side effect - 0%**<br><br>No one taking Medicine A will have a severe side effect.	**Severe side effect - 0.7%**<br><br>Among every 1,000 people taking Medicine B, **7** will experience a severe side effect.
**Minor side effect - 0%**<br><br>No one taking Medicine A will have a minor side effect.	**Minor side effect - 13%**<br><br>Among every 1,000 people taking Medicine B, **130** will experience a minor side effect.

If you take Medicine A, you would live for **3 days less than 5 years** and then die.

If you take Medicine B, you would live for **5 years** and then die.

Would you prefer Medicine A or Medicine B, or are they the same?

Indicate your choice here. You can only choose one option:

○ **Medicine A** Live for (5 years – 3 days)

○ **Medicine B** Live for 5 years

○ **The Same - Medicine A and Medicine B are the same.**

0% 100%

For peer review only

Repeated section

Remember Medicine A and Medicine B are equally effective.

However, there are some differences between Medicine A and Medicine B.

Medicine A	Medicine B
**No inconvenience**<br><br>You take a tablet only once in your life.<br>You will be given the tablet on the day you come in to see your GP.	**Inconvenience**<br><br>One tablet should be taken once a week on the same day every week.<br>You need to take this medicine for 5 years.<br><br>You need to take the tablet first thing in the morning on an empty stomach with a large glass of tap water.<br>After taking the tablet, you should stay upright for the next 30 minutes.<br><br>You should not eat anything for at least 30 minutes, including taking any other medicines.<br><br>You need to regularly visit your doctor to have your bone health checked.<br>You need to regularly visit a pharmacy for a new supply.
**Severe side effect - 0%**<br><br>No one taking Medicine A will have a severe side effect.	**Severe side effect - 0.7%**<br><br>Among every 1,000 people taking Medicine B, **7** will experience a severe side effect.
**Minor side effect - 0%**<br><br>No one taking Medicine A will have a minor side effect.	**Minor side effect - 13%**<br><br>Among every 1,000 people taking Medicine B, **130** will experience a minor side effect.

If you take Medicine A, you would live for **1 day less than 5 years** and then die.

If you take Medicine B, you would live for **5 years** and then die.

Would you prefer Medicine A or Medicine B, or are they the same?

Indicate your choice here. You can only choose one option:

◯ **Medicine A** Live for (5 years – 1 day)

◯ **Medicine B** Live for 5 years

◯ **The Same - Medicine A and Medicine B are the same.**

0% 100%

For peer review only

Question 5a.

You prefer living for 2.5 years (2 years and 6 months) on Medicine A, to living for 5 years on Medicine B.

This means **living fewer than 2.5 years on Medicine A** can be the same as living for 5 years on Medicine B.

How many years on Medicine A do you think would be the same as living for 5 years on Medicine B?

(Please give a number in years)

0% 100%

peer review only

Thank you for completing this section and indicating your choices.

Was there anything in this section which you think is unclear or that you didn't understand?

(Please provide details in the comment box below)

0% 100%

For peer review only

Part 3. Questions about you

We understand different people feel differently about different treatments.

In this part of the survey, we ask questions about you to understand if people's age, gender or experiences explain the choices made in Part 2.

0% 100%

For peer review only

We would like to learn about your experience.

Are you currently taking, or have you ever taken any of these medicines (bisphosphonates)?

Aclasta®
Actonel®
Alendronic acid
Binosto®
Bondronat®
Bonefos®
Bonviva®
Clasteon®
Fosamax®
Iasibon®
Ibandronic acid
Loron®
Pamidronate disodium
Quodixor®
Risedronate sodium
Sodium clodronate
Zerlinda®
Zoledronic acid
Zometa®

◯ Yes
◯ No

0% 100%

For peer review only - http://bmjopen.bmj.com/site/about/guidelines.xhtml

What do you think would be the **benefits** from taking this medicine (a bisphosphonate)?

What do you think would be the **harms** from taking this medicine (a bisphosphonate)?

Have you experienced any side effects from taking this medicine (a bisphosphonate)?

- Yes
- No

Do you find it inconvenient to take this medicine (a bisphosphonate)?
Please specify if you think it is inconvenient.

What is your opinion about taking this medicine (a bisphosphonate)?

- I don't mind taking them because I have to take them for a reason.
- I do not like taking them because they remind me that I am not perfectly healthy.
- I have no opinion.
- Other (please specify)

0% 100%

Do you take other medicines prescribed by your doctor?

- ◯ Yes
- ◯ No

In total, how many pills do you take on a regular basis?
(Please do not include pills you take only once in a while)

- ◯ None
- ◯ One
- ◯ Two to five
- ◯ Six to ten
- ◯ More than ten

How many different times a day do you regularly take pills?

- ◯ None
- ◯ Once per day
- ◯ 2 times a day
- ◯ 3 times a day
- ◯ More than 3 times a day

Do you find it inconvenient to take this medicine?
Please specify if you think it is inconvenient.

What is your opinion about taking this medicine?

- ◯ I don't mind taking them because I have to take them for a reason.
- ◯ I do not like taking them because they remind me that I am not perfectly healthy.
- ◯ I have no opinion.
- ◯ Other (please specify)

For peer review only

We would like to ask questions about you to better understand your choices.

What is your gender?

- ◯ Male
- ◯ Female
- ◯ Other (please specify) [ ]

Which age category do you fall into?

- ◯ 34 years or younger
- ◯ 35-44 years
- ◯ 45-54 years
- ◯ 55-64 years
- ◯ 65-74 years
- ◯ 75-84 years
- ◯ 85 years or over

Which best reflects your ethnic group?

- ◯ White British/Irish
- ◯ White other
- ◯ Mixed/Multiple ethnic origins
- ◯ Black/African/Caribbean/Black British
- ◯ Asian/Asian British
- ◯ Other (please specify) [ ]

0% 100%

What is your occupational status?

- ◯ Employed full-time
- ◯ Employed part-time
- ◯ Self-employed
- ◯ Unemployed
- ◯ Retired
- ◯ Looking after a home/family
- ◯ Student
- ◯ Freelance or temping
- ◯ Long-term sickness

Could you tell us what your occupation is?
(Previous occupation if retired.)

What is the highest level of education you have obtained?
(for example, GCSEs, Degrees, etc.)

What is your religion?

- ◯ No religion
- ◯ Christian
- ◯ Buddhist
- ◯ Jewish
- ◯ Hindu
- ◯ Muslim
- ◯ Sikh
- ◯ Other

0% 100%

How do you feel about numbers?

This section will ask you some questions to understand how familiar you are with numbers and probabilities.

If you are unsure of the answer, you can leave the response blank.

0% 100%

For peer review only

Question 1

Imagine I flip a coin 1,000 times.

What is your best guess about how many times the coin would fall heads up in 1,000 flips?

- ◯ Answer: ____
- ◯ Prefer not to answer
- ◯ Don't know.

Question 2

If there was a lottery where the chance of winning £10 is 1%, if 1,000 people bought a ticket, how many would you expect to win?

- ◯ Answer: ____
- ◯ Prefer not to answer
- ◯ Don't know.

Question 3

In another lottery, the chance of winning a car is 1 in 1,000.

What percentage of tickets in the lottery will win a car?

- ◯ Answer: ____ **%**
- ◯ Prefer not to answer
- ◯ Don't know

0% 100%

How confident are you that you would answer the questions the same if faced with the situations in real-life?

	Very confident	Confident	Not sure	Not confident	Not confident at all	
Confident	◯	◯	◯	◯	◯	Not confident at all

How easy or difficult did you find making choices between alternatives?

	Very easy	Easy	Average	Somewhat difficult	Very difficult	
Easy	◯	◯	◯	◯	◯	Difficult

How easy or difficult did you find the survey to understand?

	Very easy	Easy	Average	Somewhat difficult	Very difficult	
Easy	◯	◯	◯	◯	◯	Difficult

0% 100%

eview only

Was there anything in the survey that was particularly unclear?

(Please write in the box below)

Is there anything else about the survey we could change to make it easier to uncerstand (for example, phrasing and explanation)?

(Optional) If you have any further comments or suggestions, please write them here.

0% 100%

only

Thank you for completing this survey

If you would like more information about osteoporosis please visit the NHS Choices website here.

MANCHESTER 1824
The University of Manchester

0% 100%

For peer review only

Note:

When respondents take the survey in regular mode this page will not be displayed. Respondents will be redirected to the link below:

https://dkr1.ssisurveys.com/projects/end?rst=1&psid=
[Script] &basic=99423

0% 100%

For peer review only

Note:

When respondents take the survey in regular mode this page will not be displayed. Respondents will be redirected to the link below:

https://dkr1.ssisurveys.com/projects/end?rst=2&psid= [Script]

0% 100%

For peer review only

Note:

When respondents take the survey in regular mode this page will not be displayed. Respondents will be redirected to the link below:

https://dkr1.ssisurveys.com/projects/end?rst=3&psid= [Script]

0% 100%

For peer review only

Supplementary Appendix 1: The statin survey

See separate pdf file

For peer review only

Supplementary Appendix 2: The bisphosphonate survey

See separate pdf file

For peer review only

Supplementary Appendix 3: Training materials for statin survey

Statins for the prevention of CVD

Bobbie has been to see a doctor.

The doctor has said Bobbie needs to start a medicine to prevent serious events because of his heart disease.

We will take you through Bobbie's story.

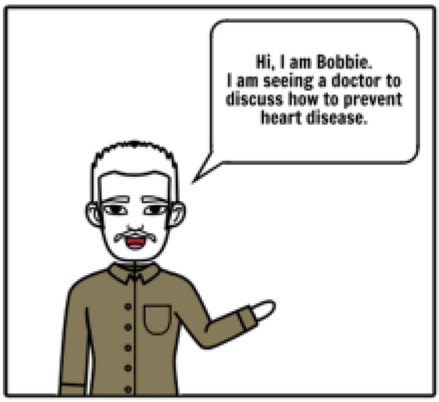

What is heart disease?

Bobbie hears about heart disease from his GP.

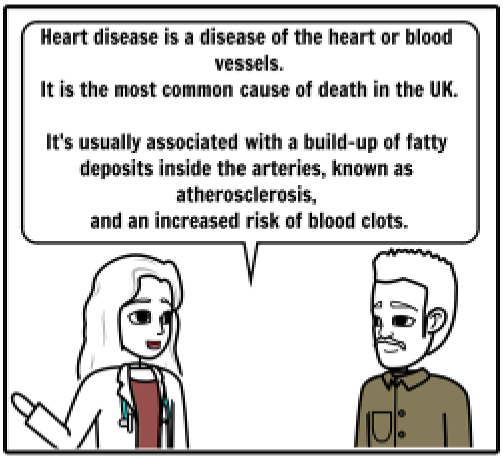

Impact of heart disease

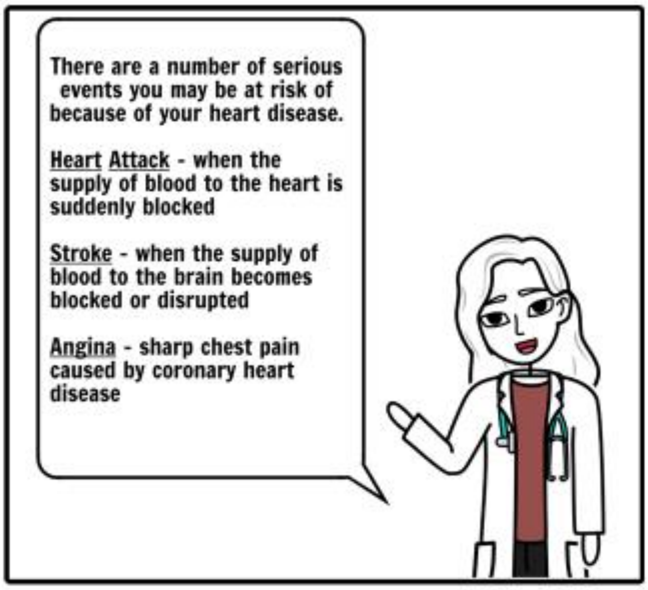

Risk of Heart disease

The GP explains that there is a chance that because of his cholesterol level Bobbie is at risk of developing one of these serious events in the future.

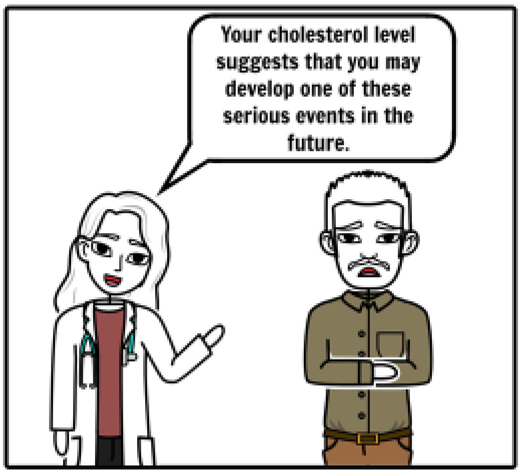

What is risk?

- **Risk** is: a term used to explain the chance that something bad might happen.

- **10% risk** means:

  Out of every **1,000** people, with heart disease,

  100 people would develop a heart attack, and **900** people would not.

What is a 10% risk?

- This diagram shows a 10% risk of having a heart attack because of their heart disease.

- The people shaded blue are the ones who have a heart attack.

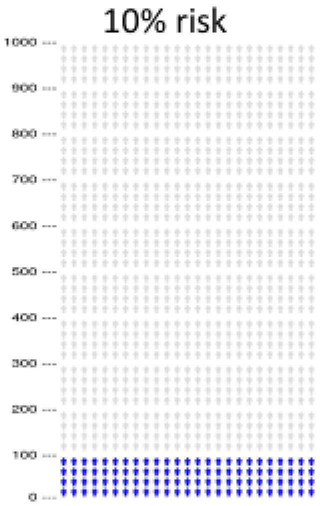

Prevention of serious events from heart disease

- Bobbie's doctor can estimate how likely it is that he will develop heart disease over the next 10 years.
- The estimate is based on things such as Bobbie's age and sex, family medical history, blood pressure and cholesterol level.
- The doctor may prescribe certain medicines such as statins to help reduce the chance of Bobbie having heart disease.
- The medicine will prevent some of the people who take it from having a serious event because of the heart disease.
- A serious event from the heart disease will still happen to some of the people who take the medicine.

Prevention of serious events from heart disease

The doctor prescribes Bobbie a medicine to reduce the chance of his heart disease causing a heart attack or other heart conditions in the future.

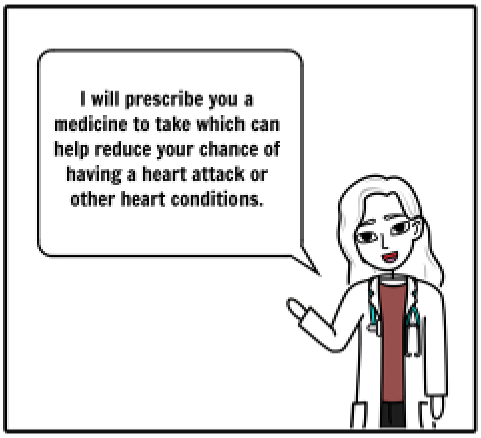

Collecting the medicine from the pharmacy

Bobbie goes to a pharmacy to collect his medicine.

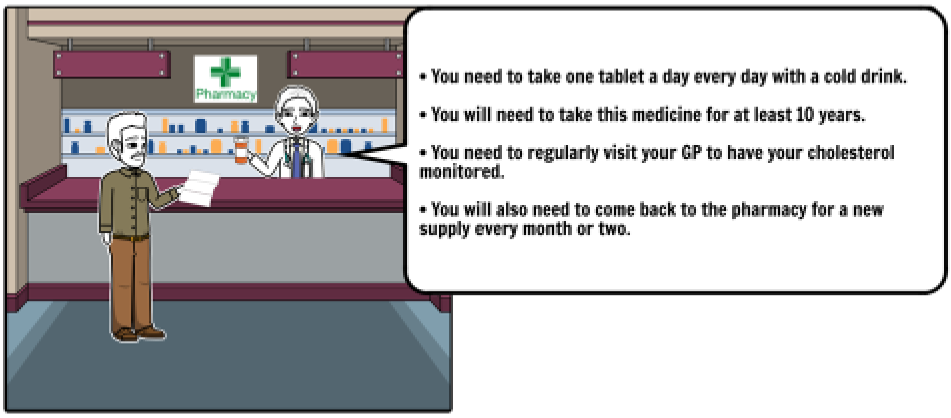

Taking the medicine for prevention

Bobbie goes home with his new tablets.

He then has to decide whether he wants to take them as the doctor and the pharmacist have advised.

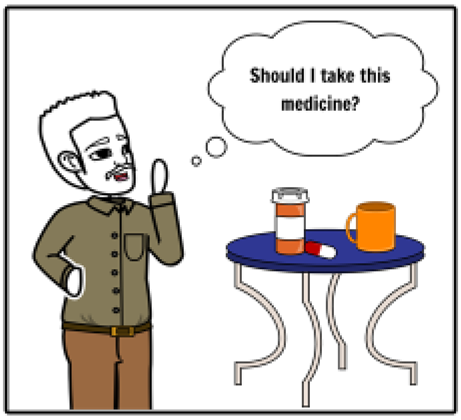

- Bobbie may consider three key things, when deciding whether to take the medicine.

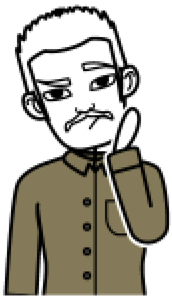

Effectiveness of the medicine

- How good the medicine is in terms of preventing a condition like a heart attack

Side effects of the medicine

- Whether there is any potential harm from the medicine

Inconvenience of the medicine

- Whether taking the medicine would fit in his lifestyle or cause him inconvenience

How effective is the medicine?

- Some people, but not everyone, who take the medicine will avoid a serious event, such as a heart attack or stroke as a result of taking the tablet.

- The effectiveness of the medicine can be described in terms of a reduction in this risk.

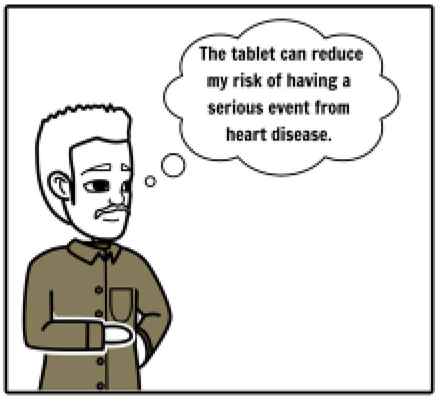

What does 'reduction in risk' mean?

- A 'reduction in risk from 10% to 6%' means:

The medicine will reduce your risk of experiencing an event, such as a heart attack, within the next 10 years from 10% to 6%.

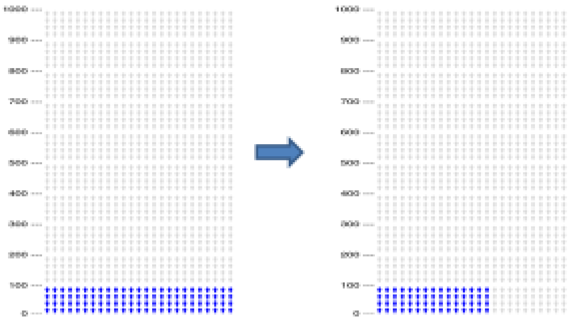

No treatment: 10% risk

Taking the medicine: 6% risk

Does the medicine have side effects?

The potential for side effects from the medicine can be described in terms of the risk of a side effect.

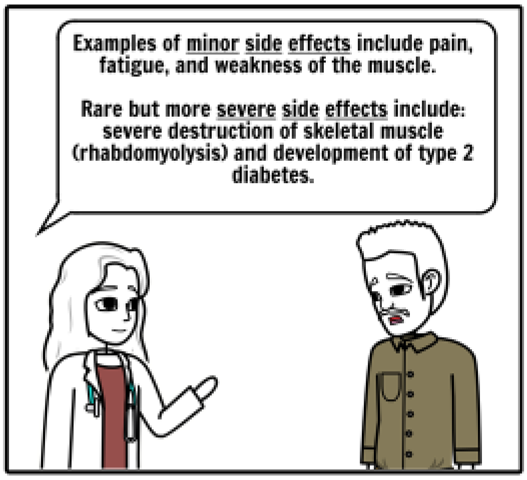

Risk of a minor side effect from the medicine

- A 5% risk of a **minor** side effect means:

Among every 1,000 people taking the medicine,

50 will experience a minor side effect such as either: pain, fatigue, and weakness of the muscle.

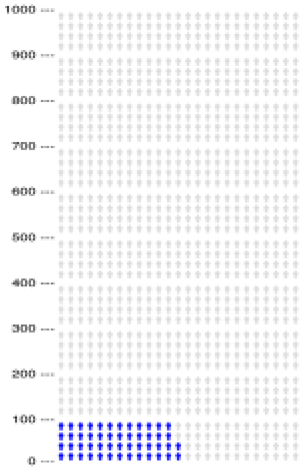

5% risk of a minor side effect

Risk of a severe side effect from the medicine

- A 0.1% risk of a **severe** side effect means:

Among every 1,000 people taking the medicine,

1 will experience a severe side effect such as either: severe destruction of skeletal muscle or development of type 2 diabetes.

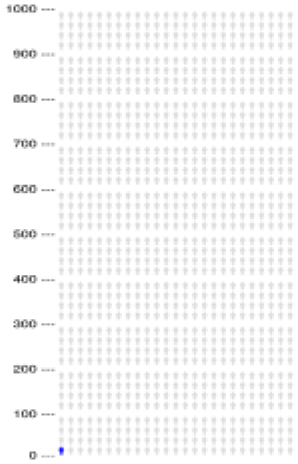

0.1% risk of a severe side effect

How inconvenient is it when taking the medicine?

Bobbie also thinks about what it means to have to take this medicine for the next ten years.

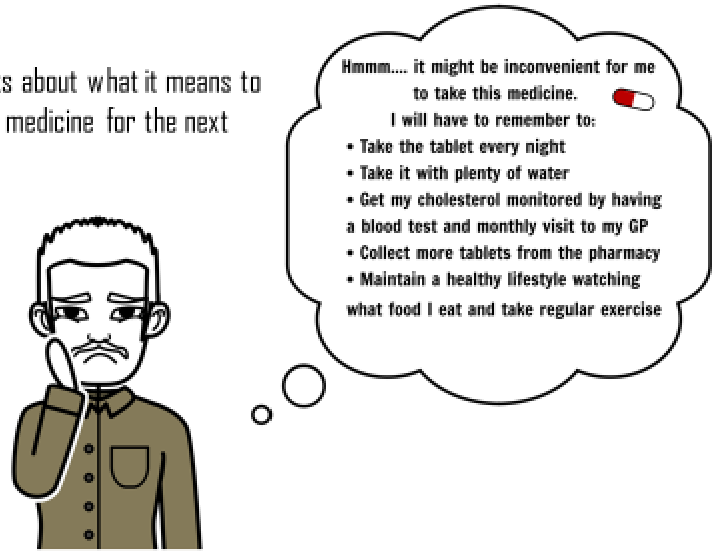

In the questions that follow in this survey, we will ask you about how you think about taking a medicine to prevent heart disease.

Supplementary Appendix 4: Training materials for bisphosphonates survey

Bisphosphonates for the prevention of osteoporotic fractures

Alex has been to see a doctor who told her that she has weak bones.

The doctor has said Alex needs to start a medicine to prevent broken bones (fractures) because of her weak bones.

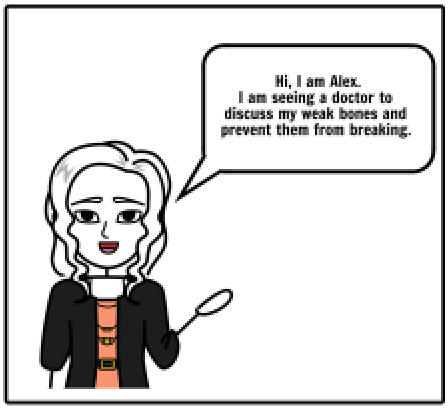

We will take you through Alex's story.

What is osteoporosis?

Alex hears about osteoporosis from her GP.

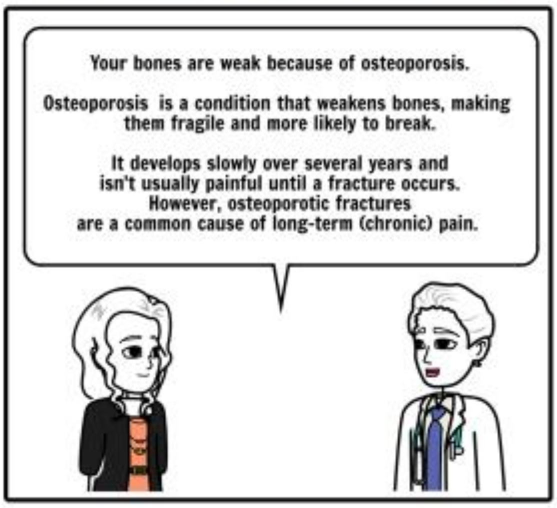

Impact of osteoporosis

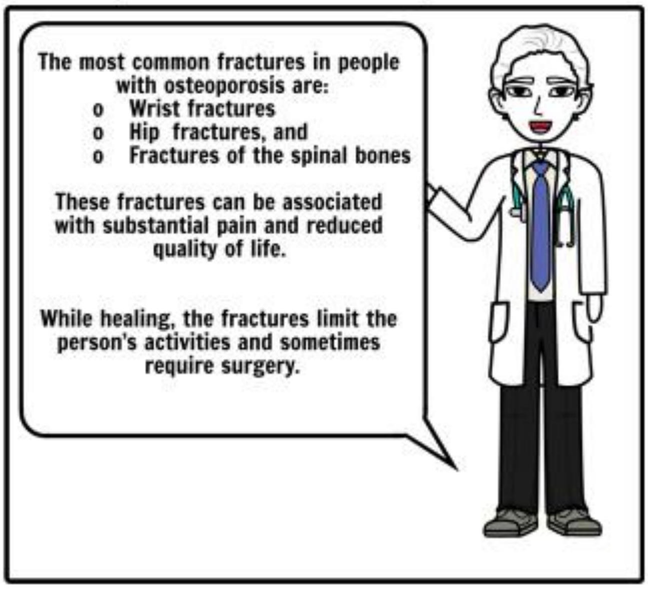

Risk of osteoporotic fractures

Her GP explains that Alex has some risk of fracture as her family has a history of hip fracture.

Also, Alex has a low body mass index (BMI). BMI is a measure of body fat.

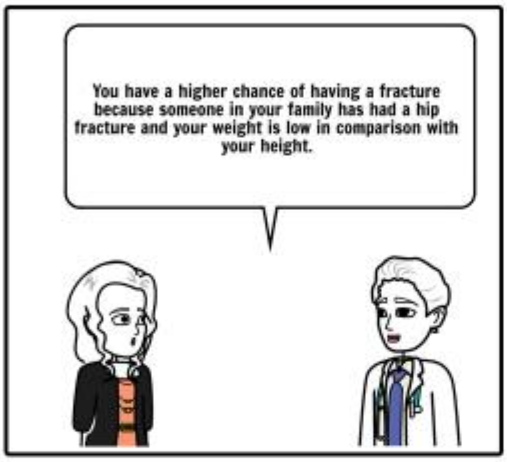

What is risk?

- **Risk** is: a term used to explain the chance that something bad might happen.

- **10% risk** means:

  Out of every **1,000** people, with osteoporosis,

  100 people would have a fracture, and **900** people would not.

What is a 10% risk?

- This diagram shows a 10% risk of having a fracture because of their osteoporosis.

- The people shaded blue are the ones who have a fracture.

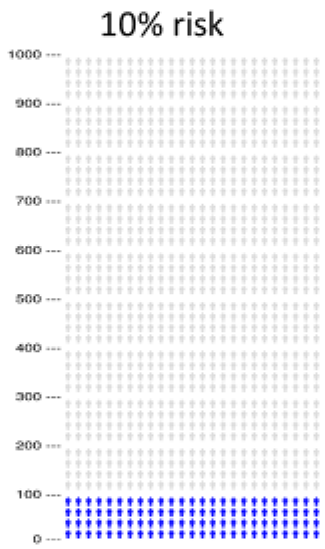

Prevention of fracture from osteoporosis

- Alex's doctor can estimate how likely it is that she will experience osteoporotic fracture over the next 5 years.
- The estimate is based on things such as Alex's age and sex, ethnicity, family fracture history, and body mass index.
- The doctor may prescribe certain medicines such as bisphosphonates to help reduce the chance of Alex having a fracture.
- The medicine will prevent some of the people who take it from having a fracture because of osteoporosis.
- Fractures may still happen in some of the people who take the medicine.

Prevention of fractures from osteoporosis

The doctor prescribes Alex a medicine to reduce the risk of her having a fracture in the future because of her osteoporosis.

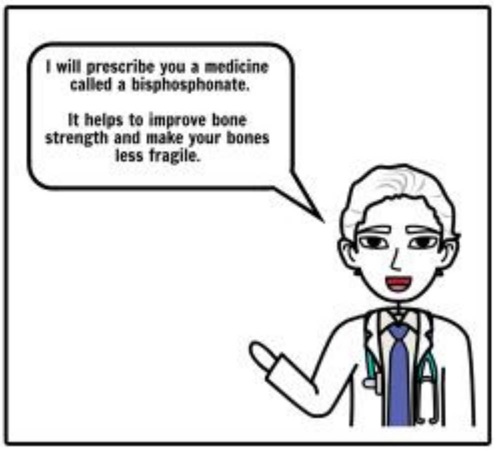

Collecting the medicine from the pharmacy

The pharmacist explains to Alex how to take the medicine.

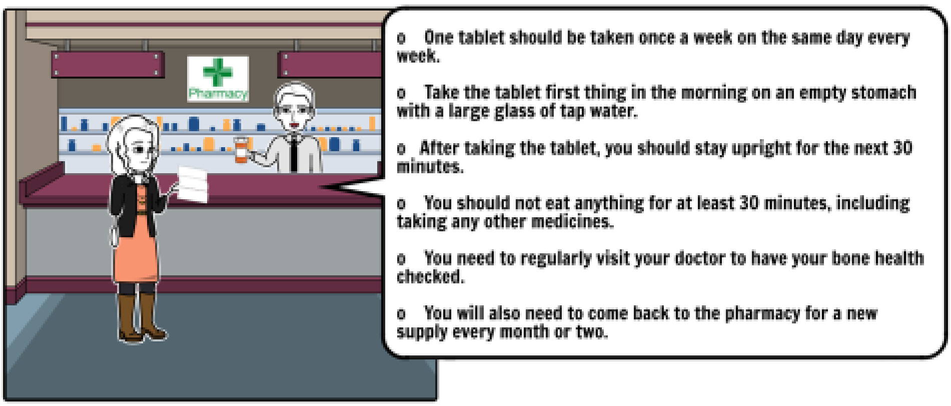

Taking the medicine for prevention

Alex goes home with her new tablets.

She then considers whether she wants to take them as the doctor and the pharmacist have advised.

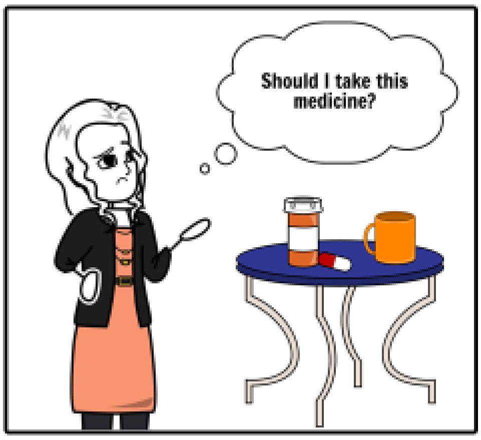

Alex may consider *three key things*, when deciding whether to take the medicine.

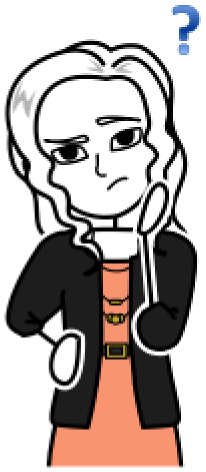

Effectiveness of the medicine

- How good the medicine is in terms of preventing a fracture from osteoporosis

Side effects of the medicine

- Whether there is any potential harm from the medicine

Inconvenience of the medicine

- Whether taking the medicine would fit in her lifestyle or cause her inconvenience

How effective is the medicine?

- Some people, but not everyone, who take the medicine will avoid a serious event, such as hip fracture, as a result of taking the tablet.
- The effectiveness of the medicine can be described in terms of a reduction in this risk.

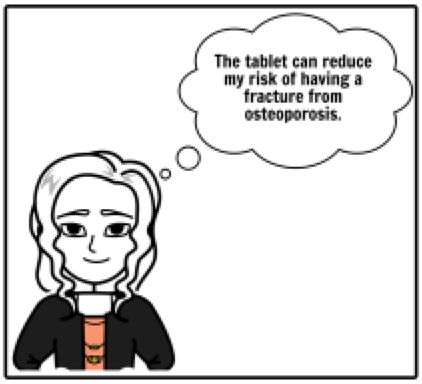

What does 'reduction in risk' mean?

- A 'reduction in risk from 10% to 6%' means:

The medicine will reduce your risk of experiencing a fracture, such as hip fracture, within the next 5 years from 10% to 6%.

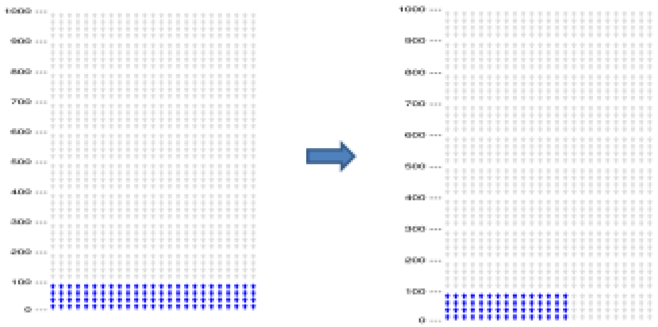

Does the medicine have side effects?

The potential for side effects from the medicine can be described in terms of the **risk of a side effect**.

Alex hears about minor but more common side effects & severe but rarer side effects from her GP.

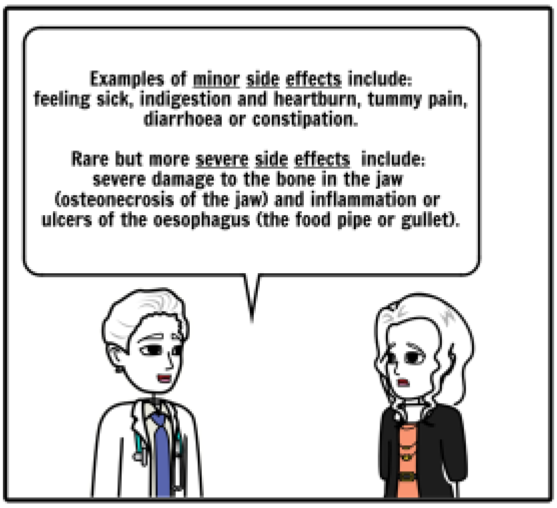

Risk of a minor side effect from the medicine

- A 5% risk of a minor side effect means:

Among every **1,000** people taking the medicine,

50 will experience a minor side effect such as either: feeling sick, indigestion, diarrhoea or constipation.

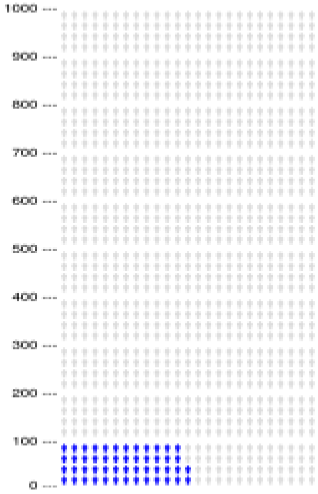

5% risk of a side effect

Risk of a severe side effect from the medicine

- A 0.3% risk of severe side effect means:

Among every **1,000** people taking the medicine,

3 will experience a severe side effect such as severe loss or destruction of the bone in the jaw.

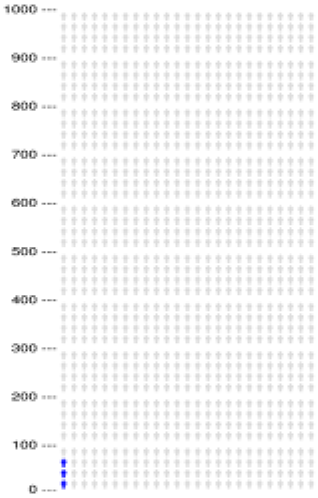

0.3% risk of a side effect

How inconvenient is it when taking the medicine?

Alex also thinks about what it means to have to take this medicine for the next 5 years.

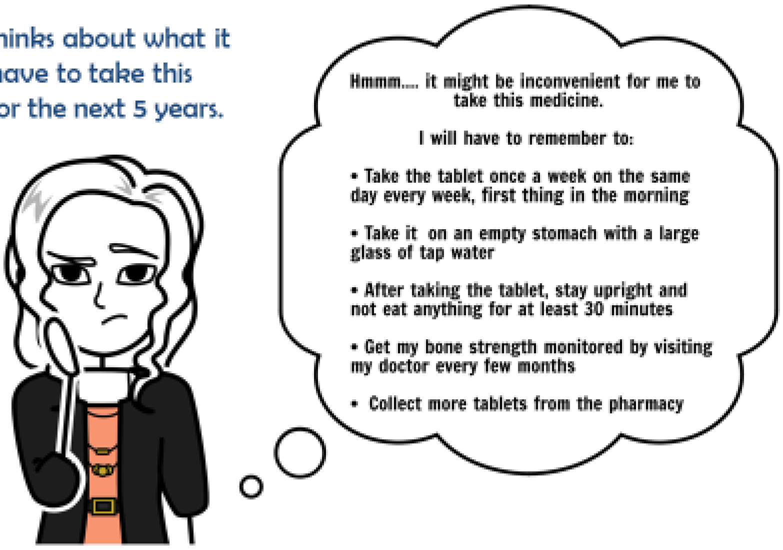

In the questions that follow in this survey, we will ask you about how you think about taking a medicine to prevent osteoporotic fracture.

Supplementary Appendix 5: Stata 'do' file

```
****** ANALYSIS AND TABLES ******
/*
## ---------------------------
##
## Script name: script-01
##
## Purpose of script: Analyse data from DTD study
##
## Author: Dr. Alex Thompson
##
## Date Created: 07/08/2021
##
##
## Email: alexander.thompson@manchester.ac.uk
##
## ---------------------------
##
## Notes:
##
##
## ---------------------------
*/

clear all                               // clears memory of everything
global location 1                       //
set cformat %5.3f                       // controls the output of tables so coefficients are rounded.

if ${location}==1 global cf `"xxxxxxxxxx"'

cd "${cf}"                              // changes the working directory

** Location settings **
if ${location}==1 global sf `"xxxxxxx"'
if ${location}==1 global af `"xxxxxxx"'
if ${location}==1 global gf `"xxxxxxx"'
if ${location}==1 global cf `"xxxxxxx"'

global data             `"${sf}"'
global combined         `"${cf}"'
global save             `"${af}"'
global output           `"${gf}"'

cd "${combined}"

*** Prepare data ***

use statins.dta, clear

append using bisphos.dta, force
gen id = _n

gen patient = 1 if survey ==2 | survey==3
replace patient = 0 if patient==.

gen public = 1 if survey==1
replace public = 0 if public==.

drop if prescribed ==2 & patient==1     // People in 'patient population' not prescribed drugs by doctor

bysort id : gen howmany=_N

save combined.dta, replace
```

For peer review only

```
gen one = 1 if utility_q1==1

replace one = 0 if utility_q1!=1

gen zero = 1 if utility_q1==0.5

replace zero = 0 if utility_q1!=0.5

sum zero

save combined.dta, replace

***********************
*** TABLE 1 OUTPUTS ***
***********************


baselinetable       age sex ethnicity employment_status education1 religion ///
                    prescribed other_medicines ///
                    eq5d3l_u(cts) attitude  inconvenient opinion ///
                     other_pill_number other_pill_times if statins ==1  ///
                    , by(public , totalcolumn) ///
                    pcformat(%5.1f) meanformat(%5.3f) sdformat(%5.1f)  ///
                    exportexcel("${output}/sumstats_statins.xls", replace) reportmissing

baselinetable       age sex ethnicity employment_status education1 religion ///
                    prescribed other_medicines ///
                    eq5d3l_u(cts) attitude  inconvenient opinion ///
                     other_pill_number other_pill_times if statins ==0  ///
                    , by(public , totalcolumn) ///
                    pcformat(%5.1f) meanformat(%5.3f) sdformat(%5.1f)  ///
                    exportexcel("${output}/sumstats_bisphos.xls", replace) reportmissing

baselinetable       age sex ethnicity employment_status education1 religion ///
                    prescribed other_medicines ///
                    eq5d3l_u(cts) attitude  inconvenient opinion ///
                     other_pill_number other_pill_times if statins ==1  ///
                    , by(public , totalcolumn) ///
                    pcformat(%5.1f) meanformat(%5.3f) sdformat(%5.1f)  ///
                    exportexcel("${output}/sumstats_statins_cat.xls", replace) reportmissing catvartab("#")

baselinetable       age sex ethnicity employment_status education1 religion ///
                    prescribed other_medicines ///
                    eq5d3l_u(cts) attitude  inconvenient opinion ///
                     other_pill_number other_pill_times  if statins ==0 ///
                    , by(public , totalcolumn) ///
                    pcformat(%5.1f) meanformat(%5.3f) sdformat(%5.1f)  ///
                    exportexcel("${output}/sumstats_bisphos_cat.xls", replace) reportmissing  catvartab("#")


*****************************************************************************************************


***********************
*** Analysis 1 OUTPUTS ***
***********************


use combined.dta,clear

reshape long utility_q, i(id) j(question)

gen nontraders_half = 1 if utility_q==.5

drop  if utility_q==.5
```

For peer review only

```
ice  ${ice_set_cluster}  , m(5) ///
		saving("${combined}/imputed_cluster.dta",replace) ///
		genmiss(M)  ///
cmd( age: ologit, opinion: ologit, ethnicity: ologit, employment_status: ologit, education1: mlogit, attitude: ologit, ) match persist


qui use "${combined}/imputed_cluster.dta", clear

qui mi import ice, imputed(${ice_set_cluster}) clear

replace utility_q = 1-utility_q

***********************
*** Figure 1  ***
***********************
twoway (kdensity utility_q if statins==1 & _mi_m==0 & question==1 & patient==1 ) (kdensity utility_q if statins==1 & _mi_m==0 & question==2) (kdensity utility_q if statins==1 & _mi_m==0 & question==3) (kdensity utility_q if statins==1 & _mi_m==0 & question==4),   ///
legend(label(1 "Q1 No side effect assumed") label(2 "Q2 Some minor side effect assumed") label(3 "Q3 Some severe side effect assumed") label(4 "Q4 Reduced effectiveness assumed")  size(vsmall) just(center) ) ///
ytitle("Density" " ", size(small)) ///
xtitle(" " "Utility", size(small)) ///
name(kdensity_statins1,replace) ///
xlabel(0(0.1)0.5,  labsize(small)) ///
ylabel(0(5)30,  labsize(small) angle(horizontal) nogrid) ///
graphregion(fcolor(white) lwidth(large)) bgcolor(white) title("Statin Q1-Q4 utility values in patients",size(small) color(black)) ///
ysize(1) xsize(1) saving(kdensity_statins1,replace) scale(.9)

twoway (kdensity utility_q if statins==1 & _mi_m==0 & question==1 & patient==0 ) (kdensity utility_q if statins==1 & _mi_m==0 & question==2) (kdensity utility_q if statins==1 & _mi_m==0 & question==3) (kdensity utility_q if statins==1 & _mi_m==0 & question==4),   ///
legend(label(1 "Q1 No side effect assumed") label(2 "Q2 Some minor side effect assumed") label(3 "Q3 Some severe side effect assumed") label(4 "Q4 Reduced effectiveness assumed")  size(vsmall) just(center) ) ///
ytitle("Density" " ", size(small)) ///
xtitle(" " "Utility", size(small)) ///
name(kdensity_statins2,replace) ///
xlabel(0(0.1)0.5,  labsize(small)) ///
ylabel(0(5)30,  labsize(small) angle(horizontal) nogrid) ///
graphregion(fcolor(white) lwidth(large)) bgcolor(white) title("Statin Q1-Q4 utility values in the public",size(small) color(black)) ///
ysize(1) xsize(1) saving(kdensity_statins2,replace) scale(.9)

twoway (kdensity utility_q if statins==0 & _mi_m==0 & question==0 & patient==1 ) (kdensity utility_q if statins==0 & _mi_m==0 & question==2) (kdensity utility_q if statins==0 & _mi_m==0 & question==3) (kdensity utility_q if statins==0 & _mi_m==0 & question==4),   ///
legend(legend(label(1 "Q1 No side effect assumed") label(2 "Q2 Some minor side effect assumed") label(3 "Q3 Some severe side effect assumed") label(4 "Q4 Reduced effectiveness assumed")  size(vsmall) just(center) ) ///
label(1 "No side effect ") label(2 "Some minor side effect assumed") label(3 "Some severe side effect assumed") label(4 "Reduced effectiveness assumed")  size(vsmall) just(center) ) ///
ytitle("Density" " ", size(small)) ///
xtitle(" " "Utility", size(small)) ///
name(kdensity_bisphos1,replace) ///
xlabel(0(0.1)0.5,  labsize(small)) ///
ylabel(0(2)10,  labsize(small) angle(horizontal) nogrid) ///
graphregion(fcolor(white) lwidth(large)) bgcolor(white) title("Bisphosphonates Q1-Q4 utility values in patients",size(small) color(black)) ///
ysize(1) xsize(1) saving(kdensity_bisphos,replace) scale(.9)

twoway (kdensity utility_q if statins==0 & _mi_m==0 & question==0 & patient==0 ) (kdensity utility_q if statins==0 & _mi_m==0 & question==2) (kdensity utility_q if statins==0 & _mi_m==0 & question==3) (kdensity utility_q if statins==0 & _mi_m==0 & question==4),   ///
legend(label(1 "Q1 No side effect assumed") label(2 "Q2 Some minor side effect assumed") label(3 "Q3 Some severe side effect assumed") label(4 "Q4 Reduced effectiveness assumed")  size(vsmall) just(center) ) ///
ytitle("Density" " ", size(small)) ///
xtitle(" " "Utility", size(small)) ///
```

For peer review only

```
name(kdensity_bisphos2,replace) ///
xlabel(0(0.1)0.5,  labsize(small)) ///
ylabel(0(2)10,  labsize(small) angle(horizontal) nogrid) ///
graphregion(fcolor(white)  lwidth(large))  bgcolor(white)  title("Bisphosphonates  Q1-Q4  utility  values  in  the  public",size(small) color(black)) ///
ysize(1) xsize(1) saving(kdensity_bisphos,replace) scale(.9)

grc1leg kdensity_statins1 kdensity_statins2 kdensity_bisphos1  kdensity_bisphos2                ,       scheme(s1manual)       name(g1, replace) rows(2)
graph display g1, xsize(7) ysize(8)
graph export "${output}/kdensity1.tif",  replace width(2000)

***********************
*** Figure 1 alternative ***
***********************
replace utility_q = 1-utility_q

twoway (kdensity utility_q if statins==1 & _mi_m==0 & question==1 & patient==1 ) (kdensity utility_q if statins==1 & _mi_m==0 & question==2) (kdensity utility_q if statins==1 & _mi_m==0 & question==3) (kdensity utility_q if statins==1 & _mi_m==0 & question==4),   ///
legend(label(1 "Q1 No side effect") label(2 "Q2 Some minor side effect") label(3 "Q3 Some severe side effect") label(4 "Q4 Reduced effectiveness")  size(vsmall) just(center) ) ///
ytitle("Density" " ", size(small)) ///
xtitle(" " "Utility", size(small)) ///
name(kdensity_statins1a,replace) ///
xlabel(0.5(0.1)1,  labsize(small)) ///
ylabel(0(5)30,  labsize(small) angle(horizontal) nogrid) ///
graphregion(fcolor(white) lwidth(large)) bgcolor(white) title("Statin Q1-Q4 utility values in patients",size(small) color(black)) ///
ysize(1) xsize(1) saving(kdensity_statins1,replace) scale(.9)

twoway (kdensity utility_q if statins==1 & _mi_m==0 & question==1 & patient==0 ) (kdensity utility_q if statins==1 & _mi_m==0 & question==2) (kdensity utility_q if statins==1 & _mi_m==0 & question==3) (kdensity utility_q if statins==1 & _mi_m==0 & question==4),   ///
legend(label(1 "Q1 No side effect") label(2 "Q2 Some minor side effect") label(3 "Q3 Some severe side effect") label(4 "Q4 Reduced effectiveness")  size(vsmall) just(center) ) ///
ytitle("Density" " ", size(small)) ///
xtitle(" " "Utility", size(small)) ///
name(kdensity_statins2a,replace) ///
xlabel(0.5(0.1)1,  labsize(small)) ///
ylabel(0(5)30,  labsize(small) angle(horizontal) nogrid) ///
graphregion(fcolor(white) lwidth(large)) bgcolor(white) title("Statin Q1-Q4 utility values in the public",size(small) color(black)) ///
ysize(1) xsize(1) saving(kdensity_statins2,replace) scale(.9)

twoway (kdensity utility_q if statins==0 & _mi_m==0 & question==0 & patient==1 ) (kdensity utility_q if statins==0 & _mi_m==0 & question==2) (kdensity utility_q if statins==0 & _mi_m==0 & question==3) (kdensity utility_q if statins==0 & _mi_m==0 & question==4),   ///
legend(label(1 "Q1 No side effect") label(2 "Q2 Some minor side effect") label(3 "Q3 Some severe side effect") label(4 "Q4 Reduced effectiveness")  size(vsmall) just(center) ) ///
ytitle("Density" " ", size(small)) ///
xtitle(" " "Utility", size(small)) ///
name(kdensity_bisphos1a,replace) ///
xlabel(0.5(0.1)1,  labsize(small)) ///
ylabel(0(2)10,  labsize(small) angle(horizontal) nogrid) ///
graphregion(fcolor(white)  lwidth(large))  bgcolor(white)  title("Bisphosphonates  Q1-Q4  utility  values  in  patients",size(small) color(black)) ///
ysize(1) xsize(1) saving(kdensity_bisphos,replace) scale(.9)

twoway (kdensity utility_q if statins==0 & _mi_m==0 & question==0 & patient==0 ) (kdensity utility_q if statins==0 & _mi_m==0 & question==2) (kdensity utility_q if statins==0 & _mi_m==0 & question==3) (kdensity utility_q if statins==0 & _mi_m==0 & question==4),   ///
legend(label(1 "Q1 No side effect") label(2 "Q2 Some minor side effect") label(3 "Q3 Some severe side effect") label(4 "Q4 Reduced effectiveness")  size(vsmall) just(center) ) ///
ytitle("Density" " ", size(small)) ///
xtitle(" " "Utility", size(small)) ///
name(kdensity_bisphos2a,replace) ///
xlabel(0.5(0.1)1,  labsize(small)) ///
```

For peer review only

```
ylabel(0(2)10,  labsize(small) angle(horizontal) nogrid) ///
graphregion(fcolor(white)  lwidth(large))  bgcolor(white)  title("Bisphosphonates  Q1-Q4  utility  values  in  the  public",size(small)
color(black)) ///
ysize(1) xsize(1) saving(kdensity_bisphos,replace) scale(.9)

grc1leg kdensity_statins1a kdensity_statins2a kdensity_bisphos1a  kdensity_bisphos2a                          ,            scheme(s1manual)
name(g2, replace) rows(2)
graph display g2, xsize(7) ysize(8)
graph export "${output}/kdensity2.tif",  replace width(2000)


***********************
*** TABLE 2 OUTPUTS ***
***********************

gen str coding = ""
replace coding = "s1_p1_q1" if statins==1 & _mi_m==0 & question==1 & patient==1,
replace coding = "s1_p1_q2" if statins==1 & _mi_m==0 & question==2 & patient==1,
replace coding = "s1_p1_q3" if statins==1 & _mi_m==0 & question==3 & patient==1,
replace coding = "s1_p1_q4" if statins==1 & _mi_m==0 & question==4 & patient==1,
replace coding = "s1_p0_q1" if statins==1 & _mi_m==0 & question==1 & patient==0,
replace coding = "s1_p0_q2" if statins==1 & _mi_m==0 & question==2 & patient==0,
replace coding = "s1_p0_q3" if statins==1 & _mi_m==0 & question==3 & patient==0,
replace coding = "s1_p0_q4" if statins==1 & _mi_m==0 & question==4 & patient==0,
replace coding = "s0_p1_q1" if statins==0 & _mi_m==0 & question==1 & patient==1,
replace coding = "s0_p1_q2" if statins==0 & _mi_m==0 & question==2 & patient==1,
replace coding = "s0_p1_q3" if statins==0 & _mi_m==0 & question==3 & patient==1,
replace coding = "s0_p1_q4" if statins==0 & _mi_m==0 & question==4 & patient==1,
replace coding = "s0_p0_q1" if statins==0 & _mi_m==0 & question==1 & patient==0,
replace coding = "s0_p0_q2" if statins==0 & _mi_m==0 & question==2 & patient==0,
replace coding = "s0_p0_q3" if statins==0 & _mi_m==0 & question==3 & patient==0,
replace coding = "s0_p0_q4" if statins==0 & _mi_m==0 & question==4 & patient==0,

estpost tabstat utility_q , statistics(mean sd count skewness kurtosis p10 p25 p50 p75 p90) casewise by(coding)
esttab . using "${output}/table_tabstat.rtf", cells("mean sd count skewness kurtosis p10 p25 p50 p75 p90") noobs

gen str coding2 = ""

replace coding2 = "s1_p1" if statins==1 & _mi_m==0 & patient==1,
replace coding2 = "s1_p0" if statins==1 & _mi_m==0 & patient==0,
replace coding2 = "s0_p1" if statins==0 & _mi_m==0 & patient==1,
replace coding2 = "s0_p0" if statins==0 & _mi_m==0 & patient==0,

estpost tabstat utility_q , statistics(mean sd count skewness kurtosis p10 p25 p50 p75 p90) casewise by(coding2)

esttab . using "${output}/table_tabstat2.rtf", cells("mean sd count skewness kurtosis p10 p25 p50 p75 p90") noobs

gen str coding3 = ""
replace coding3 = "s1" if statins==1 & _mi_m==0
replace coding3 = "s0" if statins==0 & _mi_m==0

estpost tabstat utility_q , statistics(mean sd count skewness kurtosis p10 p25 p50 p75 p90) casewise by(coding3)

esttab . using "${output}/table_tabstat3.rtf", replace cells("mean sd count skewness kurtosis p10 p25 p50 p75 p90") noobs

gen trader = 1 if utility_q!=1 & utility_q!=0.5
replace trader = 0 if trader==.

estpost tabstat trader , statistics(mean sd count skewness kurtosis p10 p25 p50 p75 p90) casewise by(coding)
estpost tabstat trader , statistics(mean sd count skewness kurtosis p10 p25 p50 p75 p90) casewise by(coding2)
estpost tabstat trader , statistics(mean sd count skewness kurtosis p10 p25 p50 p75 p90) casewise by(coding3)

estpost tabstat trader if _mi_m==0 , statistics(mean sd count skewness kurtosis p10 p25 p50 p75 p90) casewise by(statins)

***********************
*** TABLE 3 OUTPUTS ***
```

For peer review only

```
***********************

mi estimate, post:                              reg  utility_q    i.sex  i.ethnicity  i.age  i.other_pill_times  i.other_pill_number    eq5d_index
question_2 question_3 question_4 statins public if statins == 1
estimates store m1

mi estimate, post:                              reg  utility_q    i.sex  i.ethnicity  i.age  i.other_pill_times  i.other_pill_number    eq5d_index
question_2 question_3 question_4 statins public if statins == 0
estimates store m2

gen yes = 1 if utility_q==1
replace yes = 0 if utility_q<1

mi estimate, saving(combined_logs,replace): logistic yes     i.sex     i.ethnicity     i.age     i.other_pill_times     i.other_pill_number
eq5d_index question_2 question_3 question_4 statins public
estimates store m3


esttab m1 m2          using "${output}/table_all_new.rtf" , ar2 label not   replace ci
esttab m3             using "${output}/table_all_logist_new.rtf" , ar2 label not  replace   ci

mi test question_2 question_3 question_4
/*
. mi test question_2 question_3 question_4
note: assuming equal fractions of missing information

 ( 1)  question_2 = 0
 ( 2)  question_3 = 0
 ( 3)  question_4 = 0

       F(  3, 149.7) =    0.15
           Prob > F =    0.9310

.
*/


*****************************************************
*** Appendix analysis 6 ***
*****************************************************
**Propensity for dominant preferences**

gen yes = 1 if utility_q==1
replace yes = 0 if utility_q<1

gen no = 1 if utility_q==0.5
replace no = 0 if no==.

la var yes "Selected 1 for DTD"

mi estimate, post:                                                                                 logistic yes            sex    i.ethnicity
i.education1 i.age   public statins
estimates store m5


mi estimate, post:                                                                                 logistic no             sex    i.ethnicity
i.education1 i.age   public statins
estimates store m6

esttab m5 m6


qui use "${combined}/imputed_cluster.dta", clear

*****************************************************
*** Appendix analysis 7 ***
```

For peer review only

```
******************************************************

************STATINS*****************

qui mi import ice, imputed(${ice_set_cluster}) clear
forvalues i =1/5 {
*ssc install zoib //install zero-one-inflated beta regression package
zoib utility_q   sex i.ethnicity i.education1 i.age i.question public i.other_pill_times i.other_pill_number  if _mi_m==1 & statins==1
predict beta_`i'

betamix utility_q      if _mi_m==1 & statins==1, muvar(sex i.ethnicity i.education1 i.age i.question public i.other_pill_times i.other_pill_number ) lbound(0.5) ubound(1) pmass(1 0 1)  //zero and one inflated, including all values
predict mixedbeta_`i'

reg utility_q                    sex i.ethnicity i.education1 i.age i.question   public i.other_pill_times i.other_pill_number   if _mi_m==`i' & statins==1
predict yhat_`i'

}

mi estimate, saving(statin_ols,replace):                      reg utility_q   i.sex i.ethnicity i.education1 i.age i.question   public i.other_pill_times i.other_pill_number if statins==1

forvalues i =1/5 {
rmse utility_q yhat_`i'                    if _mi_m==`i'
rmse utility_q beta_`i'                    if _mi_m==`i'
rmse utility_q mixedbeta_`i'      if _mi_m==`i'

}

************BISPHOSPHONATES*****************
qui use "${combined}/imputed_cluster.dta", clear

qui mi import ice, imputed(${ice_set_cluster}) clear
forvalues i =1/5 {
*ssc install zoib //install zero-one-inflated beta regression package
zoib utility_q   sex i.ethnicity i.education1 i.age i.question public i.other_pill_times i.other_pill_number  if _mi_m==1 & statins==0
predict beta_`i'

betamix utility_q      if _mi_m==1 & statins==0, muvar(sex i.ethnicity i.education1 i.age i.question public i.other_pill_times i.other_pill_number ) lbound(0.5) ubound(1) pmass(1 0 1)  //zero and one inflated, including all values
predict mixedbeta_`i'

reg utility_q                    sex i.ethnicity i.education1 i.age i.question   public i.other_pill_times i.other_pill_number  if _mi_m==`i' & statins==0
predict yhat_`i'

}

mi estimate, saving(statin_ols,replace):                      reg utility_q   i.sex i.ethnicity i.education1 i.age i.question   public i.other_pill_times i.other_pill_number if statins==0

forvalues i =1/5 {
rmse utility_q yhat_`i'                    if _mi_m==`i'
*gen ols_rmse_`i'  = r(yhat_`i')  if _mi_m==`i'
rmse utility_q beta_`i'                    if _mi_m==`i'
rmse utility_q mixedbeta_`i'      if _mi_m==`i'

}

forvalues i =1/5 {
mean yhat_`i' if _mi_m==`i'                 & statins==0
mean beta_`i' if _mi_m==`i'                 & statins==0
mean mixedbeta_`i' if _mi_m==`i'            & statins==0

}
```

For peer review only

*** Model fit check ***

```
twoway ( scatteri 0.5 0.5 1 1, recast(line) lcolor(gray) lwidth(medthin) lpattern(solid) ) ///
( scatter yhat_1 utility_q  if _mi_m==1 , mcolor(red)  msize(small) msymbol(circle) msymbol(circle_hollow)  ) ( scatter beta_1 utility_q if _mi_m==1 , mcolor(black)  msize(small) msymbol(circle) msymbol(circle_hollow)) ( scatter mixedbeta_1 utility_q  if _mi_m==1 , mcolor(green)  msize(small) msymbol(circle) msymbol(circle_hollow)), ///
 xlabel(0.5(0.25)1,nogrid  labsize(small))  ylabel(0.5(0.25)1,nogrid  labsize(small))  graphregion(  color(white)  )  legend(off)  yscale( nofextend ) xscale(nofextend) ///
ytitle("EQ-5D-3L Predicted JHC", size(small)) xtitle("EQ-5D-3L Observed JHC", size(small)) title(`"`vtext'"', size(small) color(black)) ///
xmtick( 0.5(0.25)1 , grid glcolor(gray) glpattern(dash) glwidth(vthin) nogextend ) ///
ymtick( 0.5(0.25)1 , grid glcolor(gray) glpattern(dash) glwidth(vthin) nogextend ) xsize(1) ysize(1)


twoway (kdensity x if statins==1) (kdensity x if statins==0) (kdensity y if statins==1) (kdensity y if statins==0)  (kdensity yhat if statins==1) (kdensity yhat if statins==0)
```

Supplementary Appendix 6: Description of the whole sample characteristics

	Statin survey			Bisphosphonate survey			
	Patient[a]	Public[b]	Total	Patient[a]	Public[b]	Total	Total
	N=260	N=376	N=636	N=110	N=359	N=469	N= 1105
**Age**							
Less than 35	2 (1.2%)	25 (7.9%)	27 (5.5%)	0 (0.0%)	26 (8.8%)	26 (7.5%)	53 (6%)
35-44	5 (2.9%)	74 (23.3%)	79 (16.2%)	1 (1.9%)	51 (17.3%)	52 (14.9%)	131 (16%)
45-54	10 (5.9%)	57 (17.9%)	67 (13.7%)	3 (5.7%)	41 (13.9%)	44 (12.6%)	111 (13%)
55-64	49 (28.8%)	84 (26.4%)	133 (27.3%)	14 (26.4%)	57 (19.3%)	71 (20.4%)	204 (24%)
65-74	77 (45.3%)	70 (22.0%)	147 (30.1%)	20 (37.7%)	107 (36.3%)	127 (36.5%)	274 (33%)
75+	27 (15.9%)	8 (2.5%)	35 (7.2%)	15 (28.3%)	13 (4.4%)	28 (8.0%)	63 (8%)
Missing	90	58	148	57	64	121	269
**Sex**							
Female	57 (33.5%)	159 (50.0%)	216 (44.3%)	46 (86.8%)	185 (62.9%)	231 (66.6%)	447 (54%)
Male	113 (66.5%)	159 (50.0%)	272 (55.7%)	7 (13.2%)	109 (37.1%)	116 (33.4%)	388 (46%)
Missing	90	58	148	57	65	122	270
**Ethnicity**							
White British/Irish	160 (94.1%)	288 (90.6%)	448 (91.8%)	51 (96.2%)	263 (89.2%)	314 (90.2%)	762 (91%)
White other	4 (2.4%)	16 (5.0%)	20 (4.1%)	1 (1.9%)	12 (4.1%)	13 (3.7%)	33 (4%)
Mixed/Multiple ethnic origins	0 (0.0%)	2 (0.6%)	2 (0.4%)	0 (0.0%)	5 (1.7%)	5 (1.4%)	7 (1%)
Black/African/Caribbean/Black British	0 (0.0%)	3 (0.9%)	3 (0.6%)	0 (0.0%)	4 (1.4%)	4 (1.1%)	7 (1%)
Asian/Asian British	0 (0.0%)	7 (2.2%)	7 (1.4%)	0 (0.0%)	9 (3.1%)	9 (2.6%)	16 (2%)
Chinese	0 (0.0%)	1 (0.3%)	1 (0.2%)	1 (1.9%)	2 (0.7%)	3 (0.9%)	4 (%)
Other ethnicity	6 (3.5%)	1 (0.3%)	7 (1.4%)	0 (0%)	0 (0%)	0 (0%)	(%)
Missing	90	58	148	57	64	121	269
**Number of pills taken daily**							
None	0 (0.0%)	133 (41.8%)	133 (27.3%)	0 (0.0%)	99 (33.6%)	99 (28.4%)	232 (28%)
One	4 (2.4%)	73 (23.0%)	77 (15.8%)	6 (11.3%)	51 (17.3%)	57 (16.4%)	134 (16%)
Two to five	123 (72.4%)	87 (27.4%)	210 (43.0%)	32 (60.4%)	109 (36.9%)	141 (40.5%)	351 (42%)
Six to ten	36 (21.2%)	18 (5.7%)	54 (11.1%)	9 (17.0%)	23 (7.8%)	32 (9.2%)	86 (10%)
More than ten	7 (4.1%)	7 (2.2%)	14 (2.9%)	6 (11.3%)	13 (4.4%)	19 (5.5%)	33 (4%)
Missing	90	58	148	57	64	121	269
**Number of different times pill taken per day**							
None	3 (1.8%)	134 (42.1%)	137 (28.1%)	0 (0.0%)	97 (32.9%)	97 (27.9%)	234 (28%)
Once per day	40 (23.5%)	104 (32.7%)	144 (29.5%)	22 (41.5%)	100 (33.9%)	122 (35.1%)	266 (32%)
2 times a day	100 (58.8%)	61 (19.2%)	161 (33.0%)	20 (37.7%)	68 (23.1%)	88 (25.3%)	249 (30%)
3 times a day	23 (13.5%)	13 (4.1%)	36 (7.4%)	7 (13.2%)	24 (8.1%)	31 (8.9%)	67 (8%)
More than 3 times a day	4 (2.4%)	6 (1.9%)	10 (2.0%)	4 (7.5%)	6 (2.0%)	10 (2.9%)	20 (2%)
Missing	90	58	148	57	64	121	269
**EQ-5D-3L utility[c]**	0.820 (0.2)	0.803 (0.3)	0.810 (0.3)	0.771 (0.2)	0.790 (0.2)	0.786 (0.2)	0.800 (0.3)
Missing	41	38	79	35	41	76	155

Footnote: [a] patient sample was recruited from general practitioners in the NHS Research Scotland Primary Care Network or the Scottish Health Research Register (SHARE); [b]public sample was recruited from Dynata; [c]Health status measured using the EQ-5D-3 level and transformed into a utility score

For peer review only

Supplementary Appendix 7: Propensity for dominant preference

Male	0.988
	[0.716, 1.364]
White other	1.263
	[0.594,2.684]
Mixed/Multiple ethnic	0.370
	[0.043,3.171]
Black/African/Caribbean/Bla	0.903
	[0.156,5.244]
Asian/Asian British	1.230
	[0.329, 4.604]
Chinese	0.748
	[0.077, 7.290]
Other ethnicity	0.568
	[0.066, 4.902]
Less than 35	0
	[.,.]
35-44	0.709
	[0.377, 1.333]
45-54	0.521
	[0.265, 1.023]
55-64	0.507
	[0.262,0.978]
65-74	0.480
	[0.2540, 0.907]
75+	0.962
	[0.399, 2.318]
Public	1.544*
	[1.047, 2.278]
None	0
	[.,.]
Once per day	1.092
	[0.600,1.986]
2 times a day	0.860
	[0.417,1.775]
3 times a day	1.077
	[0.427,2.715]
More than 3 times a day	2.797
	[0.819,9.554]
None	0
	[.,.]
One	1.0211
	[0.507,2.055]
Two to five	0.875
	[0.446,1.716]
Six to ten	1.012
	[0.386,2.654]
More than ten	1.263
	[0.451,3.536]
Statin sample	0.927
	[0.678,1.268]
Constant	0.367*
	[0.135,1.00]
Observations	1105

For peer review only

Supplementary Appendix 8: Root mean squared error (RMSE) for the competing models to fit TTO data

	Statins			Bisphosphonates		
Imputation number	OLS[1]	ZOIB[2]	MBR[3]	OLS[1]	ZOIB[2]	MBR[3]
Imputation 1	0.0692	0.0694	0.0699	0.0706	0.0719	0.0716
Imputation 2	0.0697	0.0703	0.0695	0.0717	0.0712	0.0718
Imputation 3	0.0693	0.0709	0.0715	0.0718	0.0721	0.0717
Imputation 4	0.0697	0.0705	0.0721	0.0706	0.0722	0.0733
Imputation 5	0.0698	0.0688	0.0717	0.0717	0.0693	0.0734
Mean	0.0695	0.0700	0.0709	0.0713	0.0713	0.0723

[1] Ordinary least squares; [2] Zero inflated beta regression; [3] Mixed beta regression

For peer review only

BMJ Open

Quantifying the impact of taking medicines for primary prevention: a time-trade off study to elicit direct treatment disutility in the UK

Journal:	*BMJ Open*
Manuscript ID	bmjopen-2022-063800.R2
Article Type:	Original research
Date Submitted by the Author:	15-Jun-2023
Complete List of Authors:	Thompson, Alexander; The University of Manchester<br>Youn, Ji-Hee; The University of Manchester<br>Guthrie, Bruce; University of Edinburgh College of Medicine and Veterinary Medicine,<br>Hainsworth, Robert; The University of Manchester<br>Donnan, Peter; University of Dundee, Dundee Epidemiology and Biostatistics Unit<br>Rogers, Gabriel; The University of Manchester<br>Morales, Daniel; University of Dundee, Division of Population Health Sciences<br>Payne, Katherine; The University of Manchester, Health Economics at Manchester
**Primary Subject Heading**:	Health economics
Secondary Subject Heading:	Patient-centred medicine, Research methods, Health economics, Communication, Cardiovascular medicine
Keywords:	Health economics < HEALTH SERVICES ADMINISTRATION & MANAGEMENT, Coronary heart disease < CARDIOLOGY, PREVENTIVE MEDICINE

SCHOLARONE™
Manuscripts

I, the Submitting Author has the right to grant and does grant on behalf of all authors of the Work (as defined in the below author licence), an exclusive licence and/or a non-exclusive licence for contributions from authors who are: i) UK Crown employees; ii) where BMJ has agreed a CC-BY licence shall apply, and/or iii) in accordance with the terms applicable for US Federal Government officers or employees acting as part of their official duties; on a worldwide, perpetual, irrevocable, royalty-free basis to BMJ Publishing Group Ltd ("BMJ") its licensees and where the relevant Journal is co-owned by BMJ to the co-owners of the Journal, to publish the Work in this journal and any other BMJ products and to exploit all rights, as set out in our licence.

The Submitting Author accepts and understands that any supply made under these terms is made by BMJ to the Submitting Author unless you are acting as an employee on behalf of your employer or a postgraduate student of an affiliated institution which is paying any applicable article publishing charge ("APC") for Open Access articles. Where the Submitting Author wishes to make the Work available on an Open Access basis (and intends to pay the relevant APC), the terms of reuse of such Open Access shall be governed by a Creative Commons licence – details of these licences and which Creative Commons licence will apply to this Work are set out in our licence referred to above.

Other than as permitted in any relevant BMJ Author's Self Archiving Policies, I confirm this Work has not been accepted for publication elsewhere, is not being considered for publication elsewhere and does not duplicate material already published. I confirm all authors consent to publication of this Work and authorise the granting of this licence.

For peer review only

Title: Quantifying the impact of taking medicines for primary prevention: a time-trade off study to elicit direct treatment disutility in the UK

Running title: Estimating values for direct treatment disutility

Authors:

Alexander J Thompson, PhD[1a*] (alexander.thompson@manchester.ac.uk)

Ji-Hee Youn, PhD[1a]

Bruce Guthrie, MB BChir PhD[3]

Robert Hainsworth, MSc[1]

Peter Donnan, PhD[2]

Gabriel Rogers[1]

Daniel Morales, PhD[2]

Katherine Payne, PhD[1]

[a] joint first authors

[1] *Manchester Centre for Health Economics, Division of Population Health, Health Services Research and Primary Care, University of Manchester*

[2] *Division of Population Health and Genomics, University of Dundee*

[3] *Usher Institute, College of Medicine and Veterinary Medicine, University of Edinburgh*

**Corresponding author: Alexander Thompson, Floor 4, Jean Mcfarlane Building, Oxford Road, The University of Manchester, Manchester, M13 9PL, UK*

e-mail: alexander.thompson@manchester.ac.uk

ORCiD: 0000-0003-4930-5107

Keywords: direct treatment disutility, cardiovascular disease, statins, osteoporosis, bisphosphonates

Word Count: 3637

For peer review only

Abstract

Direct treatment disutility (DTD) represents an individual's disutility associated with the inconvenience of taking a medicine over a long period of time.

Objectives: The main aim of this study was to elicit DTD values for taking a statin or a bisphosphonate for primary prevention. A secondary aim was to understand factors which influence DTD values.

Methods

Design: We used a cross-sectional study consisting of time-trade off (TTO) exercises embedded within online surveys. Respondents were asked to compare a one-off pill ('Medicine A') assumed to have no inconvenience and a daily pill ('Medicine B') over ten-years (statins) or five-years (bisphosphonates).

Setting: Individuals from NHS primary care and the general population were surveyed using an online panel company.

Participants: Two types of participants were recruited. First, a purposive sample of patients with experience of taking a statin (n=260) or bisphosphonate (n=100) were recruited from an NHS sampling frame. Patients needed to be aged over 30, have experience of taking the medicine of interest and have no diagnosis of dementia or of using dementia drugs. Second, a demographically balanced sample of members of the public were recruited for statins (n=376) and bisphosphonates (n=359).

Primary and secondary outcome measures: Primary outcome was mean direct treatment disutility. Regression analysis explored factors which could influence DTD values.

Results: A total of 879 respondents were included for analysis (514 for statins and 365 for bisphosphonates). The majority of respondents reported a disutility associated with medicine use. Mean DTD for statins was 0.034 and for bisphosphonates 0.067 respectively. Respondent characteristics including age and sex did not influence DTD. Experience of bisphosphonate-use reduced reported disutilities.

Conclusions: Statins and bisphosphonates have a quantifiable direct treatment disutility. The size of estimated disutilities suggest they are likely to be important for cost-effectiveness, particularly in individuals at low-risk when treated for primary prevention.

For peer review only - http://bmjopen.bmj.com/site/about/guidelines.xhtml

Strength and limitations

- We use a large representative sample of both public and patients for our estimated values.
- We used patients to inform and trial our survey and designed storyboards and visual arrays to contextualize the exercises and communicate absolute risk.
- The time trade-off methods employed were consistent with those first used to derive the EQ-5D-3L, a widely used measure of health-related quality of life.
- Survey respondents self-selected and this could be related to their ability to complete the survey or their self-reported health.

For peer review only

1 Introduction

Medicines for the primary prevention of disease are typically taken long-term, often for life, following identification that an individual is at risk of a harmful event, such as a stroke due to cardiovascular disease (1) or a hip fracture as a result of osteoporosis (2). Taking a medicine is considered, in general, to have a minimal day-to-day treatment burden because, in the traditional clinical sense, the action of medicine-taking is perceived to be non-invasive. Yet there is qualitative evidence to suggest that taking a medicine long-term is associated with treatment burden (3). Moreover, supporting this qualitative evidence is a quantitative literature seeking to estimate the utility of pill-taking, or alternatively, the negative impact of undergoing long-term treatment with medicines, called 'direct treatment disutility'.

Direct treatment disutility (DTD) represents an individual's strength of preference not to take a medicine. DTD could occur for a number of reasons including the inconvenience of obtaining prescriptions and medicines, needing to modify lifestyles to take medicines and attending healthcare visits for monitoring treatment (4). DTD therefore can be considered in addition to the potential impact adverse drug reactions could have on an individual's utility and/or the out-of-pocket costs (financial burden) medicines may incur. To date, the quantification of DTD in the literature has focused primarily on long-term medicines for cardiovascular disease with values estimated using hypothetical thought experiments such as 'time trade-off' (TTO) exercises where respondents are often asked whether they would forego some length of life not to undergo treatment (5–7). Estimates from this literature suggest that a substantial proportion of people would be willing to trade some length of life or risk of death not to take a long-term preventative treatment (5–7).

A previous literature review in 2015 (4) highlighted the small number of published cost-effectiveness analyses (CEA) that have included DTD values. Where DTD was incorporated in analysis, it was an important factor impacting upon cost-effectiveness results making primary preventative treatments, such as statins, much less likely to be cost-effective (8–14). In these analyses, DTD values were often assumed (10,13,14) rather than based on empirical estimates. Meanwhile, the small number of previous empirical DTD studies, likely for reasons of practicality, have either adopted small study sizes (5) or sampling

For peer review only - http://bmjopen.bmj.com/site/about/guidelines.xhtml

frames which are either not representative of patients (7) or the general population (6). More recently, further cost-effectiveness analyses (15–18) and other decision-making approaches (19,20) have incorporated DTD values, and found them to be highly influential on results, yet have had to rely on DTD values sourced from the same limited set of empirical studies.

The aim of this study was to build upon the previous empirical literature and elicit values of DTD for long-term primary preventative medicines from a mixed sample of the general population and patients. We focus on two populations who might require long-term treatment with medicines: those taking statins for the primary prevention of cardiovascular disease (CVD) and bisphosphonates for the primary prevention of bone fractures. A secondary aim of this study was to explore if survey participant characteristics were associated with the elicited DTD values.

2 Methods

Ethical approval for this study was granted by an NHS Health Research Authority Research Ethics Committee (REC reference: 17/NW/0124; project number: 220492). Respondents gave informed consent after reading a participant information sheet online and indicating they agreed to participate in the survey online.

There is no agreement on the appropriate method to elicit DTD (5–7). This study used cross sectional surveys, based on a single time point in each survey, to conduct time trade off valuation exercises (21,22). This valuation method is a widely recognised and has been the standard approach for eliciting utility values to generate tariffs used by national decision-making bodies such as the National Institute for Health and Care Excellence (NICE) (23) who have used the EQ-5D-3L to quantify health-related quality of life, valued using a time trade-off exercise (24).

For peer review only - http://bmjopen.bmj.com/site/about/guidelines.xhtml

2.1 Selection of medicine examples

Statins for primary prevention of CVD was selected because it is an example of an orally administered medicine that is perceived to be benign, but which some people perceive as harmful. Bisphosphonates for the primary prevention of bone fractures was selected because it is an example of a medicine that has an obvious potential impact on day-to-day life. Patients undergoing treatment with bisphosphonates must take the medication on an empty stomach, drink a full glass of water, stand for 30 minutes after taking the medication and avoid food and drink for a further 2 hours.

2.2 Patient and public involvement

The genesis for the concept of direct treatment disutility (DTD) was informed by previous research conducted with patients exploring the impact of medications in those with multimorbidity (25). To quantify DTD, two patient experts contributed experience of taking medicines long-term, alongside clinical input from the research team, to develop the description of the health (medicine-taking) states used in this study. Separate online surveys were designed for the two selected medicine examples (see Supplementary Appendix 1 for the statin survey and Supplementary Appendix 2 for the bisphosphonate survey). Pilot studies were conducted for each survey. First, a qualitative pilot study, using the think-aloud method (26,27) from a sample of patients from a GP practice in Greater Manchester was used to understand whether the surveys were sufficiently clear for respondents to complete as intended. Second, a quantitative pilot study with 30 respondents (for statin survey and bisphosphonate survey) identified by panel company Dynata was used to allow a preliminary analysis of data collected from the valuation exercise. No changes were made following the quantitative pilot study. The final surveys were formatted and administered online using Sawtooth software (28). Respondents were sent a secure link to complete a survey.

2.3 Study sample

Two purposive samples of patients taking a statin or bisphosphonate were recruited from two sampling frames: from general practices via the NHS Research Scotland Primary Care Network (29) and the Scottish Health Research Register (SHARE – a register of people living in Scotland allowing recruitment after a search of their medical records)(30). For both, the inclusion criteria for recruiting patients with

For peer review only

experience of taking a statin (bisphosphonate) were: prescription of a statin (or a bisphosphonate) in the last year; aged 30 years and over; not been diagnosed with dementia (ICD10 code: F00, F01, F02, F03, G10, G20, G30, G31.0, F05.1, R54 and all child codes); not taking a dementia drug (all drugs in British National Formulary chapter 0411); not also taking a bisphosphonate (or statin as appropriate). Patients deemed unsuitable for any reason by their general practitioner were also excluded.

A purposive sample of members of the public were recruited using an online panel company (Dynata) (31). This online panel company provided a sample of respondents with pre-defined (age 30 years and over; equal gender-balance) characteristics and a demographically-balanced sample from England and Scotland. There were no exclusion criteria for members of the general public who self-select if they were capable of completing an online survey. Respondents from the public or patients could only take part in either a statins or bisphosphonate survey but not both.

Sample-size calculations for valuation exercises are not well established (32). This study aimed for a pragmatic sample size of a minimum of 500 respondents (250 patients; 250 on-line panel) for each medicine example. This resulted in a target total sample size of 1000 respondents (500 for the statin survey and 500 for the bisphosphonate survey).

2.4 Valuation exercise

The time trade-off method employed was similar in approach to that taken by Hutchins et al (6,7) when valuing the utility of pill taking. See Supplementary Appendix 1 for the whole survey format. The duration within the exercise for daily pill taking was 10-years for statins and 5-years for bisphosphonates. Respondents were asked to imagine taking a pill every day for 10-years (5-years) and then dying. Respondents were then asked whether they would be willing to trade that health state for an alternative where only a single pill was taken at the start of the time-period. In this second health state, however, the respondent would live for a shorter duration and then die. A process of 'iteration' was then used to see whether respondents would trade between the health states of differing lengths until a point of indifference occurs between the two health states. The trade-off being quantified here was whether the participant would be willing to choose to live a shorter life but without daily pill taking or alternatively,

For peer review only - http://bmjopen.bmj.com/site/about/guidelines.xhtml

to have a one-off pill but forego some length of life. The process of iteration to find the potential indifference point followed a standardised process called the Measurement and Valuation of Health protocol whereby a combination of 'bisection' (in which the length of life is always the midpoint of the remaining scale section (bisected)) and 'titration' (in which the length of life is sequentially altered by fixed increments/ decrements). The Measurement and Valuation of Health protocol has been recommended previously to encourage comparability between utility values elicited for the purposes of health technology assessment (32).

The exercises previously described were contextualized with four different background scenarios in order to understand whether DTD values differed depending on the framing of benefits and harms of the medications being used. We asked respondents to consider the exercise with the context that there was: no side-effects (scenario 1) associated with any of the pills; mild side-effects (scenario 2); severe side-effects (scenario 3); and reduced effectiveness (scenario 4). We developed training materials using a story-board approach (32) to enable the communication of the background concepts for respondents completing each survey (see Supplementary Appendix 3 & 4).

2.5 Data analysis

Summary statistics were calculated for a master sample combining data from Scottish Primary Care Research Network (SPCRN) and SHARE ('patient respondents') and Dynata ('public respondents') for both statin and bisphosphonate questionnaires. For each of the four questions per respondent, the estimated utility (or the value attached to a health state of daily medicine use) was calculated as the ratio x/t. In this calculation, x is the final time period, measured in years, whereby participants were indifferent between living in the health states of Medicine A (one pill taken once) and Medicine B. t represents the health state of a pill taken every day for either statins or bisphosphonates measured as 10 years or 5 years respectively (22). The final DTD was calculated by subtracting the estimated utility from 1 or full health. Respondents who indicated they would be unwilling to initiate preventative therapy, by selecting five years for Medicine A (or the lowest time trade-off score attainable of 0.5) in any one of the questions were removed from the dataset. This was because we inferred such respondents to have dominant preferences to not undergo preventative treatment (33,34).

For peer review only - http://bmjopen.bmj.com/site/about/guidelines.xhtml

Missing data for background characteristics as well as for the time trade-off scores were multiply imputed (m=5) using chained equations with predictive mean matching (35). Differences in respondent time trade-off scores associated with medicine type (statins versus bisphosphonates), framing of the survey question (question 1, question 2, question 3, question 4), background characteristics (age, ethnicity, sex) and experience of taking medication (pills taken per day, number of times medication taken per day) were explored using ordinary least squares regression accounting for the multiply imputed datasets. Model specification was informed by summary statistics and kernel density plots of the DTD values. Sensitivity to alternative regression models, were explored with competing models compared using root mean squared error (RMSE). Propensity to trade was explored through a logistic regression for the whole sample with a dummy variable coded 1 for those willing to trade (0.5 <TTO < 1) and 0 for those unwilling to trade (TTO = 1).

For the regression models, a $p < 0.05$ was considered statistically significant. Analysis was carried out in Microsoft Excel and Stata version 16.0 with code available in Supplementary appendix 5.

3 Results

Characteristics for statin respondents (n=514) and bisphosphonate respondents (n=365) who were included in the analysis set are reported in Table 1. Characteristics for the whole sample (n=1105), including those excluded for having dominant preferences (20.1%) are presented in Supplementary appendix 6. Demographic characteristics, and experience of taking medicines was not associated with having dominant preferences to avoid preventative treatment (n=226). However, respondents from the public were more likely to have dominant preferences than those with experience of taking medicines (Supplementary appendix 7).

In the analysis set, patients in the statin survey tended to be older versus those in the public cohort with a higher proportion of male respondents (66.5% versus 50.0%). Compared with their public counterparts, patient respondents also tended to take more pills, at more times of the day, yet surprisingly, they also

For peer review only - http://bmjopen.bmj.com/site/about/guidelines.xhtml

reported a slight improvement in health (EQ-5D-3L utility: 0.827 versus 0.818). Patient respondents in the bisphosphonate sample also tended to be older than those from the public sample but with a higher proportion of females than in the public cohort (89.2% versus 62.4%). Bisphosphonate patient respondents tended to take more pills, at more times of the day versus those in the public. In contrast to the statin survey, bisphosphonate patient respondents reported comparatively lower health than public respondents (EQ-5D-3L utility: 0.779 versus 0.786 respectively).

Figure 1 shows the pre-imputation distributional properties for time trade-off values reported by patients and public, stratified by the question-context, for both the statins and bisphosphonates surveys. As can clearly be observed, the spread of responses from participants was highly variable suggesting individual respondents differed greatly in how negatively they valued taking a pill every day. Due to this high variability, Table 2 presents a wider range of summary statistics than might be typical. Overall, 78% of respondents reported disutility from a taking a pill with a higher proportion indicating some disutility for bisphosphonates (84%) than for statins (73%). Bisphosphonate patients reported much higher mean time trade-off scores than public respondents (difference: 0.024 (se: 0.006)) with this difference being statistically significant. For both statins and bisphosphonates, changing the question context did not alter the mean time trade-off scores by more than 0.01. Irrespective of the type of question or respondent, there was a clear difference between statins and bisphosphonates survey results. Consequently, regression models were run separately on these two cohorts with ordinary least squares regression favoured as it typically produced the least amount of error (Supplementary appendix 8).

For peer review only

Table 1: Description of the sample characteristics

	Statin survey			Bisphosphonate survey			
	Patient[a]	Public[b]	Total	Patient[a]	Public[b]	Total	Total
	N=227	N=287	N=514	N=86	N=279	N=365	N= 879
**Age**							
Less than 35	1 (0.7%)	14 (6.1%)	15 (4.0%)	0 (0.0%)	18 (7.9%)	18 (6.8%)	33 (5.2%)
35-44	4 (2.8%)	44 (19.1%)	48 (12.9%)	1 (2.7%)	42 (18.5%)	43 (16.3%)	91 (14.3%)
45-54	6 (4.2%)	46 (20.0%)	52 (14.0%)	2 (5.4%)	32 (14.1%)	34 (12.9%)	86 (13.5%)
55-64	43 (30.3%)	63 (27.4%)	106 (28.5%)	10 (27.0%)	45 (19.8%)	55 (20.8%)	161 (25.3%)
65-74	66 (46.5%)	60 (26.1%)	126 (33.9%)	15 (40.5%)	80 (35.2%)	95 (36.0%)	221 (34.7%)
75+	22 (15.5%)	3 (1.3%)	25 (6.7%)	9 (24.3%)	10 (4.4%)	19 (7.2%)	44 (6.9%)
Missing	85	57	142	49	52	101	243
**Sex**							
Female	49 (34.5%)	115 (50.0%)	164 (44.1%)	33 (89.2%)	141 (62.4%)	174 (66.2%)	338 (53.2%)
Male	93 (65.5%)	115 (50.0%)	208 (55.9%)	4 (10.8%)	85 (37.6%)	89 (33.8%)	297 (46.8%)
Missing	85	57	142	49	53	102	244
**Ethnicity**							
White British/Irish	133 (93.7%)	211 (91.7%)	344 (92.5%)	36 (97.3%)	203 (89.4%)	239 (90.5%)	583 (91.7%)
White other	3 (2.1%)	10 (4.3%)	13 (3.5%)	1 (2.7%)	8 (3.5%)	9 (3.4%)	22 (3.5%)
Mixed/Multiple ethnic origins	0 (0.0%)	1 (0.4%)	1 (0.3%)	0 (0.0%)	5 (2.2%)	5 (1.9%)	6 (0.9%)
Black/African/Caribbean/Black [illegible]	0 (0.0%)	3 (1.3%)	3 (0.8%)	0 (0.0%)	2 (0.9%)	2 (0.8%)	5 (0.8%)
Asian/Asian British	0 (0.0%)	4 (1.7%)	4 (1.1%)	0 (0.0%)	7 (3.1%)	7 (2.7%)	11 (1.7%)
Chinese	0 (0.0%)	1 (0.4%)	1 (0.3%)	0 (0.0%)	2 (0.9%)	2 (0.8%)	3 (0.5%)
Other ethnicity	6 (4.2%)	0 (0.0%)	6 (1.6%)	0 (0%)	0 (0%)	0 (0%)	6 (0.9%)
Missing	85	57	142	49	52	101	243
**Number of pills taken daily**							
None	0 (0.0%)	91 (39.6%)	91 (24.5%)	0 (0.0%)	77 (33.9%)	77 (29.2%)	168 (26.4%)
One	4 (2.8%)	52 (22.6%)	56 (15.1%)	4 (10.8%)	38 (16.7%)	42 (15.9%)	98 (15.4%)
Two to five	104 (73.2%)	69 (30.0%)	173 (46.5%)	23 (62.2%)	86 (37.9%)	109 (41.3%)	282 (44.3%)
Six to ten	31 (21.8%)	14 (6.1%)	45 (12.1%)	5 (13.5%)	16 (7.0%)	21 (8.0%)	66 (10.4%)
More than ten	3 (2.1%)	4 (1.7%)	7 (1.9%)	5 (13.5%)	10 (4.4%)	15 (5.7%)	22 (3.5%)
Missing	85	57	142	49	52	101	243
**Number of different times pill taken per day**							
None	3 (2.1%)	94 (40.9%)	97 (26.1%)	0 (0.0%)	75 (33.0%)	75 (28.4%)	172 (27.0%)
Once per day	33 (23.2%)	74 (32.2%)	107 (28.8%)	18 (48.6%)	75 (33.0%)	93 (35.2%)	200 (31.4%)
2 times a day	87 (61.3%)	48 (20.9%)	135 (36.3%)	12 (32.4%)	54 (23.8%)	66 (25.0%)	201 (31.6%)
3 times a day	17 (12.0%)	11 (4.8%)	28 (7.5%)	5 (13.5%)	19 (8.4%)	24 (9.1%)	52 (8.2%)
More than 3 times a day	2 (1.4%)	3 (1.3%)	5 (1.3%)	2 (5.4%)	4 (1.8%)	6 (2.3%)	11 (1.7%)
Missing	85	57	142	49	52	101	243
**EQ-5D-3L utility**	0.827 (0.2)	0.818 (0.2)	0.822 (0.2)	0.770 (0.2)	0.786 (0.2)	0.783 (0.2)	0.806 (0.2)
Missing	56	41	97	35	33	68	165

Footnote: [a] patient sample was recruited from general practitioners in the NHS Research Scotland Primary Care Network or the Scottish Health Research Register (SHARE); [b] public sample was recruited from Dynata; [c]Health status measured using the EQ-5D-3 level and transformed into a utility score using Dolan et al (39)

For peer review only

Table 2: Summary statistics of time trade-off values

Medicine	Respondent	Question context	Mean	Standard deviation	Count	p50*	Proportion reporting disutility
Statins	Public	No side effects	0.965	0.057	237	0.983	0.746
Statins	Public	Some minor side effects	0.964	0.063	233	0.995	0.721
Statins	Public	Some severe side effects	0.964	0.060	232	0.988	0.725
Statins	Public	Reduced effectiveness	0.964	0.063	232	0.983	0.735
**Statins in the public**			0.964	0.061	934	0.992	0.731
Statins	Patients	No side effects	0.974	0.054	161	0.996	0.718
Statins	Patients	Some minor side effects	0.972	0.056	156	0.996	0.718
Statins	Patients	Some severe side effects	0.968	0.060	150	0.997	0.718
Statins	Patients	Reduced effectiveness	0.970	0.055	148	0.997	0.714
**Statins in patients**			0.971	0.056	615	0.997	0.717
**Statins all respondents**			0.967	0.059	1549	0.995	0.725
Bisphosphonates	Public	No side effects	0.925	0.081	219	0.967	0.860
Bisphosphonates	Public	Some minor side effects	0.930	0.076	216	0.967	0.846
Bisphosphonates	Public	Some severe side effects	0.932	0.076	214	0.967	0.846
Bisphosphonates	Public	Reduced effectiveness	0.933	0.078	216	0.967	0.842
**Bisphosphonates in the public**			0.930	0.078	865	0.967	0.849
Bisphosphonates	Patients	No side effects	0.949	0.069	42	0.983	0.814
Bisphosphonates	Patients	Some minor side effects	0.955	0.055	40	0.967	0.826
Bisphosphonates	Patients	Some severe side effects	0.958	0.060	40	0.975	0.802
Bisphosphonates	Patients	Reduced effectiveness	0.956	0.065	39	0.967	0.802
**Bisphosphonates in patients**			0.954	0.062	161	0.967	0.811
**Bisphosphonates all respondents**			0.934	0.076	1026	0.967	.840
**All statins and bisphosphonates**			0.954	0.068	2575	0.975	0.773

For peer review only - http://bmjopen.bmj.com/site/about/guidelines.xhtml

The mean conditional time trade-off value associated with statin use was 0.966 which generates a DTD of 0.034 (calculated as 1, representing full health, minus the estimated utility score). The mean conditional time trade-off value associated with bisphosphonate use was 0.933 consequently giving a DTD of 0.067. In the statin cohort, none of the explanatory variables were significantly associated with time trade-off results, with the exception that respondents from the Asian/Asian British group reported higher DTD associated with statin-use (0.044 greater than white British / Irish respondents) although this finding is only based on a very small sample (n=7) (Table 3). In the bisphosphonate cohort, the public provided a DTD level that was 0.011 larger than patients undergoing treatment with bisphosphonates. Furthermore, those who had experience of taking medications more than three times a day provided a much lower DTD value than those who had no experience of daily medicine use. None of the other explanatory variables had a statistically significant impact on DTD size, including the number of pills taken per day. Results from the logistic regression model also reported in Table 3 suggest that respondents in the statin survey were less likely to trade than those in the bisphosphonate survey; this is consistent with the finding of higher mean time trade-off (lower mean DTD) values in the statin scenarios. No other variables in the logistic regression were statistically significant.

For peer review only

Table 3: Regression results

	Statins	Bisphosphonates	Statins & Bisphosphonates
	OLS	OLS	Logistic
	Utility score [95% CI]	Utility score [95% CI]	Traders [a] [95% CI]
Sex			
Female (Reference)	0	0	1
Male	-0.00282	0.00141	0.859
	[-0.0104,0.00478]	[-0.00984,0.0127]	[0.726,1.016]
Ethnicity			
White British/Irish (Reference)	0	0	1
White other	-0.00042	0.0223	1.629
	[-0.0157,0.0149]	[-0.00800,0.0525]	[0.873,3.04]
Mixed/Multiple ethnic origins	-0.0105	-0.00997	1.511
	[-0.0707,0.0497]	[-0.0458,0.0259]	[0.482,4.734]
Black/African/Caribbean/Black British	-0.0349	-0.0128	1.866
	[-0.0852,0.0154]	[-0.0650,0.0395]	[0.578,6.027]
Asian/Asian British	-0.0467**	-0.0127	2.516
	[-0.0790,-0.0144]	[-0.0483,0.0228]	[0.866,7.309]
Chinese	0.0198	-0.0364	3.813
	[-0.0359,0.0755]	[-0.0895,0.0168]	[0.448,32.477]
Other ethnicity	0.0145	0.00717	0.936
	[-0.0106,0.0395]	[-0.144,0.158]	[0.418,2.097]
Age			
Less than 35 (Reference)	0	0	1
35-44	-0.00906	-0.0198	1.028
	[-0.0299,0.0118]	[-0.0496,0.0100]	[0.658,1.607]
45-54	-0.00428	-0.000518	1.516
	[-0.0322,0.0237]	[-0.0349,0.0339]	[0.921,2.496]
55-64	0.00732	0.00321	0.943
	[-0.0145,0.0292]	[-0.0240,0.0305]	[0.615,1.446]
65-74	0.00472	-0.00585	1.129
	[-0.0176,0.0271]	[-0.0352,0.0235]	[0.711,1.792]
75+	0.00398	-0.0184	0.77
	[-0.0237,0.0317]	[-0.0545,0.0177]	[0.424,1.397]
Number of pills taken per day			
None (Reference)	0	0	1
One	0.00379	-0.00288	0.903
	[-0.0120,0.0196]	[-0.0216,0.0158]	[0.658,1.239]
Two to five	-0.00776	-0.00873	1.085
	[-0.0286,0.0131]	[-0.0272,0.00977]	[0.811,1.452]
Six to ten	-0.00794	-0.00263	0.867
	[-0.0346,0.0188]	[-0.0253,0.0201]	[0.583,1.289]
More than ten	0.00295	-0.0127	1.242
	[-0.0308,0.0367]	[-0.0411,0.0157]	[0.63,2.446]
Number of different times pill are taken per day			
None (Reference)	0	0	1
Once per day	0.00475	0.00892	0.788
	[-0.0149,0.0244]	[-0.00798,0.0258]	[0.596,1.042]
2 times a day	0.0176	0.00988	0.757

For peer review only - http://bmjopen.bmj.com/site/about/guidelines.xhtml

	[-0.00781,0.0430]	[-0.0111,0.0309]	[0.568,1.009]
3 times a day	0.0197	0.00321	0.856
	[-0.00677,0.0462]	[-0.0254,0.0318]	[0.562,1.303]
More than 3 times a day	0.0257	0.0426*	1.327
	[-0.0166,0.0680]	[0.00658,0.0785]	[0.549,3.205]
Respondent			
Patient (Reference)	0	0	1
Public	-0.00176	-0.0106*	1.004
	[-0.0118,0.00830]	[-0.0202,-0.000937]	[0.831,1.214]
Question context			
Question 1 (Reference)	0	0	1
Question 2	-0.00118	0.00795	0.938
	[-0.00978,0.00741]	[-0.00695,0.0229]	[0.746,1.178]
Question 3	-0.00266	0.0065	0.932
	[-0.0143,0.00893]	[-0.00796,0.0210]	[0.742,1.171]
Question 4	-0.00216	0.00709	0.941
	[-0.0117,0.00737]	[-0.00909,0.0233]	[0.749,1.182]
Sample			
Statins			0.531***
			[0.444,0.635]
Constant	0.969***	0.941***	6.11***
	[0.947,0.991]	[0.907,0.975]	[3.615,10.335]
Observations	2056	1460	3516
Individuals	514	365	879

* $p < 0.05$, ** $p < 0.01$, *** $p < 0.001$

[a] Those with TTO utility scores <0 are coded. Results represent unstandardised coefficients and 95% CI for the OLS models and adjusted odds ratio and 95% CI for the logistic model Coefficients reflect odds ratios. Above 1 implies more likely to provide a DTD value or willing to trade, less than 1 implies less likely to provide a DTD.

4 Discussion

In this study we find that long-term statin use is associated with a direct treatment disutility (DTD) of 0.034 among people willing to take statins. We find that bisphosphonate use is associated with a DTD of 0.067 among people willing to take bisphosphonates. These values imply that, even if medicines have no adverse effects, the act of taking them has a nontrivial impact on people's health-related quality of life. For statins, our study suggests that respondents on average would trade approximately 17 weeks of full health over 10 years whilst for bisphosphonates it would be more than half a year of life over 10 years. The findings for statins are particularly striking given these treatments are often thought by medical professionals to have minimal impact on users' daily routines.

For peer review only - http://bmjopen.bmj.com/site/about/guidelines.xhtml

Existing empirical studies have estimated a range of values of DTD but the general order of the size of the disutility is around 0.01 on average, which is equivalent to a loss of 5 weeks of perfect health over 10 years. In line with previous empirical studies, we find evidence for three different groups or types of respondent: (1) some never trading, suggesting zero disutility associated with treatments; (2) some suggesting they would be unlikely to initiate treatment and (3) some willing to trade length of life for no ongoing treatment, suggesting a DTD. In our survey, the groups willing to trade and generate a DTD made up the majority of those surveyed with approximately 73% and 84% for statins and bisphosphonates respondents, respectively.

We find that estimated mean DTD do not differ depending on whether the treatments were framed as more or less effective or having more or fewer side-effects or based upon demographic characteristics such as age or sex. Similar to Hutchins et al (6), we do find evidence suggesting that those from a non-white background, in our case an Asian/Asian British ethnic minority background, might perceive a higher level of disutility associated with long-term statin use although this is based upon a small sample of participants. This may also be an important part of ethnic health disparities to medication adherence and intensification of treatment. For example, British South Asians have been shown to more slowly intensify diabetes treatment than white groups (36). We found no difference between patient and public disutilities for statins but we did find that bisphosphonate patients generated smaller disutility values than the values coming from the general public. This finding could support theories rooted in experience utility (37,38) whereby those possessing the “lived" knowledge of disease, or treatment of disease, do not perceive the negative effects to be as severe as those in the general public, trying to imagine it. Our findings suggest there could be “hedonistic adaption” (39), with the additional disutility of bisphosphonates being less severe for those who are already taking medicines more than three times a day anyway.

The implications of our findings for future cost–utility analyses evaluating treatment pathways featuring statins or bisphosphonates (and potentially other oral medicines) are not straightforward. On the one hand, cost-effectiveness analyses should ideally capture the impact of all relevant costs and consequences associated with alternative forms of treatment (40), so it must be relevant that we have demonstrated

For peer review only - http://bmjopen.bmj.com/site/about/guidelines.xhtml

that the average person anticipates the act of taking statins or bisphosphonates will have a nontrivial impact on their health-related quality of life. Accounting for this disutility is likely to reduce the desirability of treatments that are currently considered very cost-effective: estimates of cost-effectiveness for long-term preventative interventions have been shown to be particularly sensitive to the inclusion of DTD (9,13,10,12,11,14,41). Indeed, we have previously shown that, for some people for whom guidelines currently recommend statins (e.g. those at a 10% risk of a cardiovascular event over 10 years), a DTD that appears moderate in light of the current study (0.015) would result in treatment doing more harm than good (25,42). Another reason routinely to account for DTD is that, without it, it is not possible to value innovations with positive process characteristics.

On the other hand, the apparent existence of distinct preference groups among our respondents requires careful consideration. A substantial minority of participants repeatedly indicated that they would be unwilling to trade any life expectancy to avoid taking these medicines, suggesting they consider any inconvenience with which they are associated negligible. It would be difficult to deny access to a treatment on the grounds that the average person would be bothered by its process characteristics, which is a danger if population-level cost-effectiveness estimates routinely incorporate average DTD. In view of these conflicting considerations, we recommend that decision-makers review scenarios with and without DTD. If evidence suggests that including DTD would materially alter the balance of benefits, harms and costs associated with treatment, this should be highlighted in population-level guidance, enabling prescribers at an individual level to engage in shared decision-making that gives appropriate weight to the person's preferences for avoiding the treatment's process characteristics. Such an approach fits well with the guideline development methods for NICE(43), which encourage the explicit identification of 'preference-sensitive decision-points', taking the practicalities of possible treatments into account. Future research could seek to develop tools which could quickly determine the level of DTD which could inform shared decision-making for preference-sensitive decisions.

Our study has several strengths. Firstly, the time trade-off methods we employ are consistent with the Measurement and Valuation of Health protocol first used to derive the EQ-5D-3L. Moreover, unlike previous studies that have attempted to elicit these values, we use a large representative sample of both

For peer review only - http://bmjopen.bmj.com/site/about/guidelines.xhtml

public and patients for our estimated values. Finally, due to the challenges associated with understanding and communicating risk, particularly in vulnerable older age-groups, we extensively trialed and developed the use of innovative approaches. For example, we used story-boards and visual arrays to contextualize the time trade-off exercise and communicate absolute risk. There are some limitations to our study which need to be considered. First, whilst we made best endeavours to communicate the exercise, the underlying absolute risks and the context for the research question, this had a set of clear trade-offs for participants. Second, the length of the survey, the cognitive burden and the time required were noted as challenges. Third, some of our respondents reported that they had difficulty understanding the time trade-off questions whilst others reported inconsistent values across the survey questions or had missing values, although where there was missingness we did multiply impute, assuming missing at random. Finally, those who took part in our survey ultimately self-selected and this could be related to their ability to complete the survey as well as their self-reported health. Applicability for a different patient or general population should be made based upon a careful judgement of the self-reported characteristics summarised for those reporting values in this study cohort.

5 Conclusion

Long-term preventative interventions, such as statins for cardiovascular disease or bisphosphonates for bone fractures, have a quantifiable direct treatment disutility associated with their use. The majority of respondents in our surveys, including public and patient-users, indicate at least some DTD. Bisphosphonates had larger disutilities than statins. Disutility was largely unaffected by respondents' self-reported characteristics but patient users of bisphosphonates did provide smaller DTDs than the public. Future model-based studies assessing the cost-effectiveness of long-term preventative interventions should incorporate DTD values within scenario analyses.

For peer review only - http://bmjopen.bmj.com/site/about/guidelines.xhtml

Declarations

Funding: This study/project is funded by the National Institute for Health Research (NIHR) Health Services and Delivery Research Programme (project reference 15/12/22). The views expressed are those of the authors and not necessarily those of the NIHR or the Department of Health and Social Care. The authors had full and sole access the data, and the funder had no role in the conduct of the research or the decision to publish.

Conflicts of interest. DM reports that he is supported by a Wellcome Trust Clinical Research Fellowship (214588/Z/18/Z) and is a member of the European Medicines Agency Pharmacovigilance Risk Assessment Committee. No other authors report any conflicts of interest directly relevant to the content of this article.

Consent to participate: Respondents gave informed consent after reading the participant information sheet online and indicating they agreed to participate in the survey online.

Consent for publication: The participant information sheet indicated that the results of the survey would be published and so we obtained informed consent.

Data availability: It is not possible to share the original data as this was a criterion for consent of participants.

Code availability: Survey design can be made available on request. Data analysis code included in Supplementary Appendix 5.

Contribution: All authors meet International Committee of Medical Journal Editors (ICMJE) criteria for authorship.

AJT obtained funding for this study, was involved in formulating the research question, provided advice on the design for the overall study, analysed data and produced a first draft of the manuscript. AJT acts as guarantor for this work.

JY was involved in formulating the research question, completed the process for ethical approval, produced the online version of the survey and contributed to writing the manuscript.

BG obtained funding for this study, was involved in formulating the research question, provided advice on the design for the overall study, contributed to the design of the online survey and writing the manuscript.

RH helped clean and analyse the data and contributed to writing the manuscript.

PD obtained funding for this study, was involved in formulating the research question, provided advice on the design for the overall study, contributed to the design of the online survey and writing the manuscript.

GR contributed to data analysis and contributed to writing the manuscript.

DM obtained funding for this study, was involved in formulating the research question, provided advice on the design for the overall study and contributed to writing the manuscript.

KP obtained funding for this study, formulated the research question, provided advice on the design for the overall study, contributed to the design of the online survey and oversaw data collection and analysis and contributed to writing the manuscript.

For peer review only - http://bmjopen.bmj.com/site/about/guidelines.xhtml

This manuscript has been read and approved by all the authors.

For peer review only

For peer review only - http://bmjopen.bmj.com/site/about/guidelines.xhtml

Ethics approval

Ethical approval for this study was granted by an NHS Health Research Authority Research Ethics Committee (REC reference: 17/NW/0124; project number: 220492).

Acknowledgements: We dedicate the article to the memory of Graham Bell who helped us greatly when formulating the research question and study design. We also wish to thank our other patient and public representatives that were integral to the design of this study. Our sincere thanks goes to all the respondents who piloted the initial survey and completed the final surveys for this study. We acknowledge the insightful contributions of Dr Shona Livingstone, Research Fellow in Statistics, to the survey design.

For peer review only

References

1. Taylor F, Huffman MD, Macedo AF, Moore THM, Burke M, Davey Smith G, et al. Statins for the primary prevention of cardiovascular disease. Cochrane Database Syst Rev. 2013 Jan 31;2013(1):CD004816.

2. Papapoulos SE. The role of bisphosphonates in the prevention and treatment of osteoporosis. Am J Med. 1993 Nov 30;95(5 SUPPL. 1):S48–52.

3. Sav A, Kendall E, McMillan SS, Kelly F, Whitty JA, King MA, et al. "You say treatment, I say hard work": treatment burden among people with chronic illness and their carers in Australia. Health Soc Care Community. 2013 Nov;21(6):665–74.

4. Thompson A, Guthrie B, Payne K. Do Pills Have No Ills? Capturing the Impact of Direct Treatment Disutility. PharmacoEconomics. 2016 Apr;34(4):333–6.

5. Fontana M, Asaria P, Moraldo M, Finegold J, Hassanally K, Manisty CH, et al. Patient-Accessible Tool for Shared Decision Making in Cardiovascular Primary Prevention: Balancing Longevity Benefits Against Medication Disutility. Circulation. 2014 Jun 17;129(24):2539–46.

6. Hutchins R, Viera AJ, Sheridan SL, Pignone MP. Quantifying the Utility of Taking Pills for Cardiovascular Prevention. Circ Cardiovasc Qual Outcomes. 2015 Mar;8(2):155–63.

7. Hutchins R, Pignone MP, Sheridan SL, Viera AJ. Quantifying the utility of taking pills for preventing adverse health outcomes: a cross-sectional survey. BMJ Open. 2015 May 11;5(5):e006505–e006505.

8. Augustovski FA, Cantor SB, Thach CT, Spann SJ. Aspirin for primary prevention of cardiovascular events. J Gen Intern Med. 1998 Dec;13(12):824–35.

9. Pignone M, Earnshaw S, Tice JA, Pletcher MJ. Aspirin, Statins, or Both Drugs for the Primary Prevention of Coronary Heart Disease Events in Men: A Cost–Utility Analysis. Ann Intern Med. 2006 Mar 7;144(5):326–36.

10. Timbie JW. Variation in the Net Benefit of Aggressive Cardiovascular Risk Factor Control Across the US Population of Patients With Diabetes Mellitus. Arch Intern Med. 2010 Jun 28;170(12):1037.

11. Lazar LD, Pletcher MJ, Coxson PG, Bibbins-Domingo K, Goldman L. Cost-Effectiveness of Statin Therapy for Primary Prevention in a Low-Cost Statin Era. Circulation. 2011 Jul 12;124(2):146–53.

12. Greving JP, Visseren FLJ, Wit G de, Algra A. Statin treatment for primary prevention of vascular disease: whom to treat? Cost-effectiveness analysis. BMJ. 2011 Mar 30;342:d1672.

13. Pletcher MJ. Comparing Impact and Cost-Effectiveness of Primary Prevention Strategies for Lipid-Lowering. Ann Intern Med. 2009 Feb 17;150(4):243.

14. Pletcher MJ, Pignone M, Earnshaw S, McDade C, Phillips KA, Auer R, et al. Using the Coronary Artery Calcium Score to Guide Statin Therapy: A Cost-Effectiveness Analysis. Circ Cardiovasc Qual Outcomes. 2014 Mar;7(2):276–84.

15. Pandya A, Sy S, Cho S, Weinstein MC, Gaziano TA. Cost-effectiveness of 10-Year Risk Thresholds for Initiation of Statin Therapy for Primary Prevention of Cardiovascular Disease. JAMA. 2015 Jul 14;314(2):142.

16. Heller DJ, Coxson PG, Penko J, Pletcher MJ, Goldman L, Odden MC, et al. Evaluating the Impact and Cost-Effectiveness of Statin Use Guidelines for Primary Prevention of Coronary Heart Disease and Stroke. Circulation. 2017 Sep 19;136(12):1087–98.

For peer review only - http://bmjopen.bmj.com/site/about/guidelines.xhtml

17. Spahillari A, Zhu J, Ferket BS, Hunink MGM, Carr JJ, Terry JG, et al. Cost-effectiveness of Contemporary Statin Use Guidelines With or Without Coronary Artery Calcium Assessment in African American Individuals. JAMA Cardiol. 2020 Aug 1;5(8):871.

18. Kohli-Lynch CN, Lewsey J, Boyd KA, French DD, Jordan N, Moran AE, et al. Beyond 10-Year Risk: A Cost-Effectiveness Analysis of Statins for the Primary Prevention of Cardiovascular Disease. Circulation. 2022 Apr 26;145(17):1312–23.

19. Fitzgerald SP, Bean NG, Ruberu RP. A Method of Decision Analysis Quantifying the Effects of Age and Comorbidities on the Probability of Deriving Significant Benefit from Medical Treatments. J Comorbidity. 2017 Jan;7(1):50–63.

20. Sussman JB, Schell GJ, Lavieri MS, Hayward RA. Implications of True and Perceived Treatment Burden on Cardiovascular Medication Use. MDM Policy Pract. 2017 Jul;2(2):238146831773530.

21. Torrance G. Utility approach to measuring health-related quality of life. J Chronic Dis. 1987;40:593–600.

22. Sackett DL, Torrance GW. The utility of different health states as perceived by the general public. J Chronic Dis. 1978;31(11):697–704.

23. NICE. 4 Economic evaluation | NICE health technology evaluations: the manual | Guidance | NICE [Internet]. NICE; 2022 [cited 2023 Mar 12]. Available from: https://www.nice.org.uk/process/pmg36/chapter/economic-evaluation#measuring-and-valuing-health-effects-in-cost-utility-analyses

24. Dolan P, Gudex C, Kind P, Williams A. The time trade-off method: results from a general population study. Health Econ. 1996;5(2):141–54.

25. Guthrie B, Thompson A, Dumbreck S, Flynn A, Alderson P, Nairn M, et al. Better guidelines for better care: accounting for multimorbidity in clinical guidelines – structured examination of exemplar guidelines and health economic modelling. Health Serv Deliv Res. 2017 Apr;5(16):1–150.

26. Cheraghi-Sohi S, Bower P, Mead N, McDonald R, Whalley D, Roland M. Making sense of patient priorities: applying discrete choice methods in primary care using 'think aloud' technique. Fam Pract. 2007 Jun 1;24(3):276–82.

27. Ryan M, Watson V, Entwistle V. Rationalising the 'irrational': a think aloud study of discrete choice experiment responses. Health Econ. 2009;18(3):321–36.

28. Sawtooth Software SSI Web 8.3.8. Sawtooth;

29. NHS Research Scotland. NRS Primary Care Network [Internet]. Primary Care | NHS Research Scotland. 2023 [cited 2023 Mar 12]. Available from: https://www.registerforshare.org

30. SHARE | RegisterforSHARE [Internet]. Register for SHARE. [cited 2023 Mar 12]. Available from: https://www.registerforshare.org

31. Dynata LLC. Survey Data Platform [Internet]. [cited 2023 Mar 12]. Available from: https://www.dynata.com/

32. Brazier J, Ratcliffe J, Saloman J, Tscuchiya A. Measuring and Valuing Health Benefits for Economic Evaluation. 2nd ed. Oxford University Press; 2017. 372 p.

33. Ryan M, Farrar S. Using conjoint analysis to elicit preferences for health care. BMJ. 2000 Jun 3;320(7248):1530–3.

For peer review only - http://bmjopen.bmj.com/site/about/guidelines.xhtml

34. Scott A. Identifying and analysing dominant preferences in discrete choice experiments: An application in health care. J Econ Psychol. 2002 Jun 1;23(3):383–98.

35. White IR, Royston P, Wood AM. Multiple imputation using chained equations: Issues and guidance for practice. Stat Med. 2011 Feb 20;30(4):377–99.

36. Mathur R, Farmer RE, Eastwood SV, Chaturvedi N, Douglas I, Smeeth L. Ethnic disparities in initiation and intensification of diabetes treatment in adults with type 2 diabetes in the UK, 1990-2017: A cohort study. PLoS Med. 2020 May;17(5):e1003106.

37. Kahneman D, Wakker PP, Sarin R. Back to Bentham? Explorations of Experienced Utility*. Q J Econ. 1997 May 1;112(2):375–406.

38. Dolan P, Kahneman D. Interpretations Of Utility And Their Implications For The Valuation Of Health*. Econ J. 2008;118(525):215–34.

39. Havi Carel. Is Well-being Possible in Illness? In: Phenomenology of Illnes. 1st ed. Oxford University Press; 2016.

40. Drummond MF, Sculpher MJ, Claxton K, Stoddard GL, Torrance GW. Methods for the economic evaluation of healthcare programmes. 4th ed. Oxford University Press; 2015. 464 p.

41. Roberts ET, Horne A, Martin SS, Blaha MJ, Blankstein R, Budoff MJ, et al. Cost-Effectiveness of Coronary Artery Calcium Testing for Coronary Heart and Cardiovascular Disease Risk Prediction to Guide Statin Allocation: The Multi-Ethnic Study of Atherosclerosis (MESA). Steyerberg EW, editor. PLOS ONE. 2015 Mar 18;10(3):e0116377.

42. Thompson A, Guthrie B, Payne K. Using the Payoff Time in Decision-Analytic Models: A Case Study for Using Statins in Primary Prevention. Med Decis Mak Int J Soc Med Decis Mak. 2017 Oct;37(7):759–69.

43. NICE. Shared decision making | NICE guidelines | NICE guidance | Our programmes | What we do | About [Internet]. NICE. NICE; [cited 2023 Mar 12]. Available from: https://www.nice.org.uk/about/what-we-do/our-programmes/nice-guidance/nice-guidelines/shared-decision-making

For peer review only

Figure 1: Kernel density plots showing distribution of TTO responses stratified by medicine, question context and respondent type

For peer review only

For peer review only - http://bmjopen.bmj.com/site/about/guidelines.xhtml

Supplementary Appendices for:

Thompson AJ, Youn J, Payne K, Hainsworth R, Donnan P, Allen A, Bell G, Rogers G, Guthrie B. Quantifying the impact of taking medicines for primary prevention: a time-trade off study to elicit direct treatment disutility

Supplementary Appendix 1: The statin survey

Supplementary Appendix 2: The bisphosphonate survey

Supplementary Appendix 3: Training materials for statin survey

Supplementary Appendix 4: Training materials for bisphosphonates survey

Supplementary Appendix 5: Stata 'do' file

Supplementary Appendix 6: Patient characteristics in those excluded

Supplementary Appendix 7: Propensity for dominant preferences

Supplementary Appendix 8: Performance of different models

For peer review only

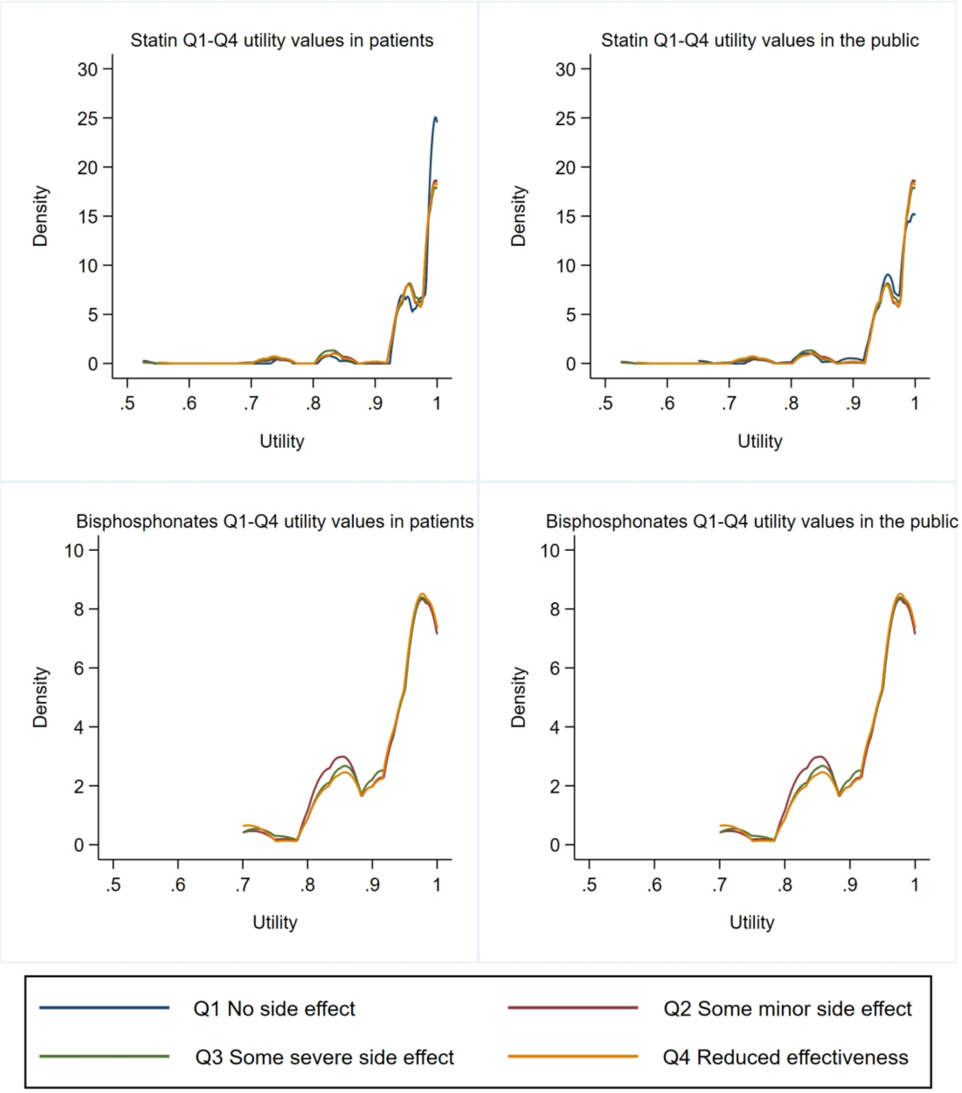

Kernel density plots showing distribution of TTO responses stratified by medicine, question context and respondent type

705x806mm (72 x 72 DPI)

Quantifying the impact of taking medicines for long-term conditions

A survey to understand your views about taking medicines long term for the prevention of heart disease

In this survey, we will be asking you about a drug treatment which may be offered to people at an increased risk of developing heart disease.

There are no right or wrong answers, we are just interested in hearing about your views.

For peer review only

Quantifying the impact of taking medicines for long-term conditions

Taking medicines long term for the prevention of heart disease

Participant Consent Form

You are being invited to take part in a research study that aims to understand your views about taking medicines long-term. We want to understand people's views on about what it is like to take a medicine every day for long periods of time. Before you decide, it is important for you to understand why the research is being done and what it will involve.

Please click on the sections below for information on the research.

What is the aim of the research?

Why have I been chosen?

What happens to the data collected?

How is confidentiality maintained?

How long will it take me?

Who will conduct the research?

What would I be asked to do if I took part?

Will the outcomes of the research be published?

What happens if I do not want to take part or if I change my mind?

Who has reviewed the research project?

I understand that my participation in the study is voluntary and that I am free to withdraw at any time without giving a reason by exiting the survey.

I confirm that I have read the information above.

- ◯ Yes, I would like to take part in the survey.
- ◯ No, I do not want to participate (exit survey).

0% 100%

What is your gender?

◯ Female
◯ Male

0% 100%

For peer review only

[QUOTA: QuotaCheck]

Note:

This page only contains quota information and only appears in preview mode.

0% 100%

For peer review only

By selecting one box in each group below, please indicate which statements best describe your own health state today.

Mobility

- ◯ I have no problems in walking about
- ◯ I have some problems in walking about
- ◯ I am confined to bed

Self-Care

- ◯ I have no problems with self-care
- ◯ I have some problems washing or dressing myself
- ◯ I am unable to wash or dress myself

Usual Activities (e.g. work, study, housework, family or leisure activities)

- ◯ I have no problems with performaing my usual activities
- ◯ I have some problems with performing my usual activities
- ◯ I am unable to perform my usual activities

Pain/Discomfort

- ◯ I have no pain or discomfort
- ◯ I have moderate pain or discomfort
- ◯ I have extreme pain or discomfort

Anxiety/Depression

- ◯ I am not anxious or depressed
- ◯ I am moderately anxious or depressed
- ◯ I am extremely anxious or depressed

0% 100%

Part 1. Study Background

This section of the survey explains: heart disease, how medicine treatments may be used for people at risk of heart disease, and what the treatment involves.

We will follow a story of Bobbie.

Click next to continue.

0% 100%

For peer review only

Part 1. Background

Bobbie has been to see a doctor.

The doctor has said Bobbie needs to start a medicine to prevent serious events because of his heart disease.

We will take you through Bobbie's story.

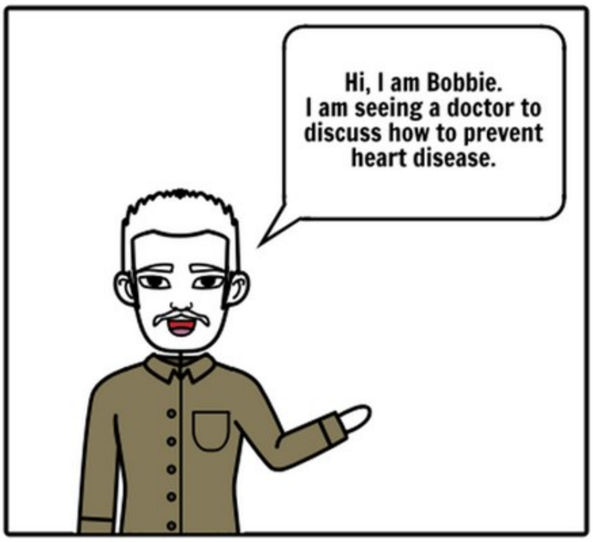

0% 100%

Part 1. Background

What is heart disease?

Bobbie hears about heart disease from his GP.

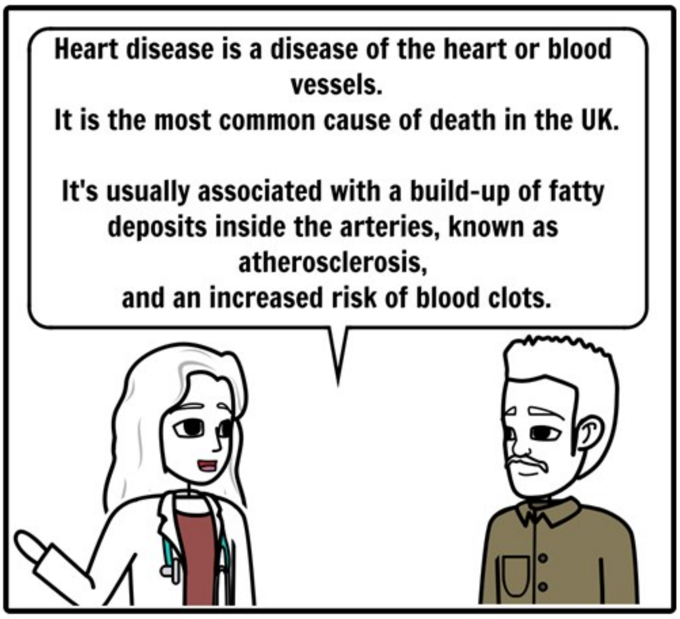

0% 100%

Part 1. Background

Impact of heart disease

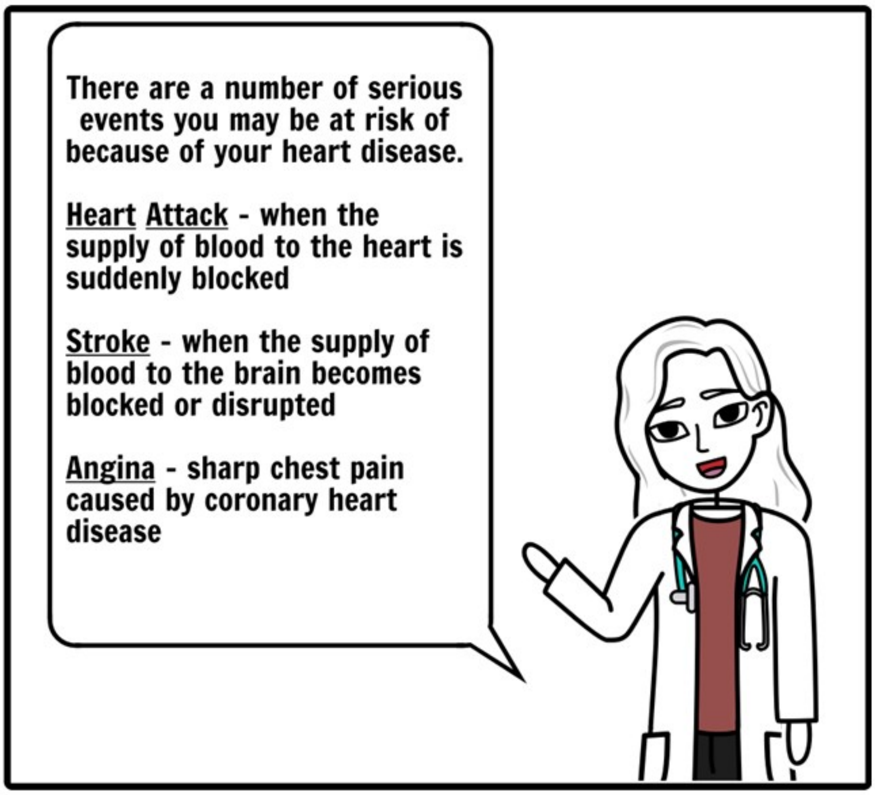

0% 100%

Part 1. Background

Risk of Heart disease

The GP explains that there is a chance that because of his cholesterol level Bobbie is at risk of developing one of these serious events in the future.

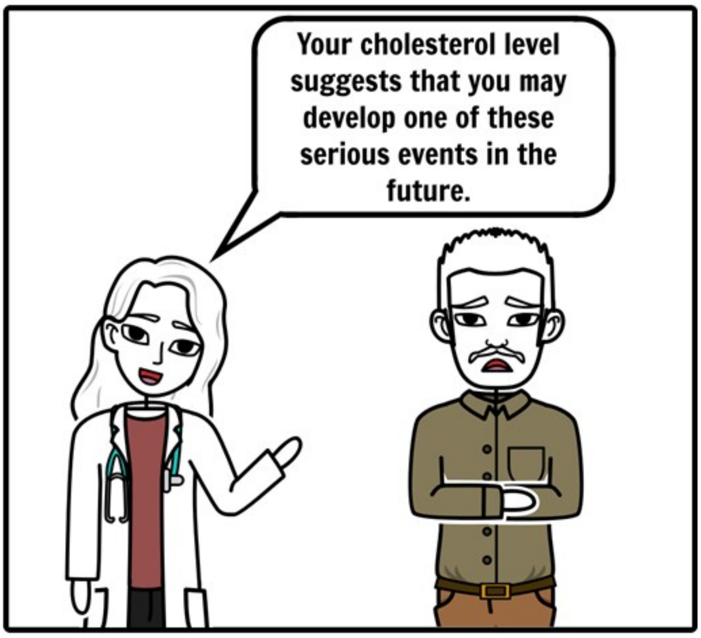

0% 100%

Part 1. Background

What is risk?

- **Risk** is: a term used to explain the chance that something bad might happen.

- **10% risk** means:

  Out of every **1,000** people, with heart disease,

  100 people would develop a heart attack, and **900** people would not.

0% 100%

Part 1. Background

What is a 10% risk?

- This diagram shows a 10% risk of having a heart attack because of their heart disease.
- The people shaded blue are the ones who have a heart attack.

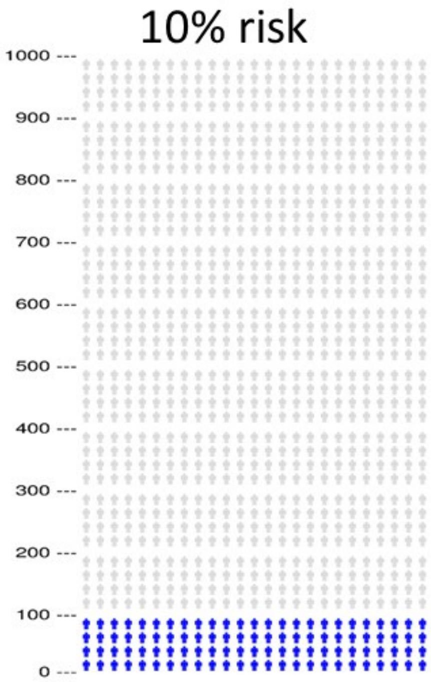

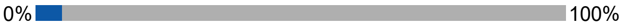

Part 1. Background

Prevention of serious events from heart disease

- Bobbie's doctor can estimate how likely it is that he will develop heart disease over the next 10 years.
- The estimate is based on things such as Bobbie's age and sex, family medical history, blood pressure and cholesterol level.
- The doctor may prescribe certain medicines such as statins to help reduce the chance of Bobbie having heart disease.
- The medicine will prevent some of the people who take it from having a serious event because of the heart disease.
- A serious event from the heart disease will still happen to some of the people who take the medicine.

0% 100%

Part 1. Background

Prevention of serious events from heart disease

The doctor prescribes Bobbie a medicine to reduce the chance of his heart disease causing a heart attack or other heart conditions in the future.

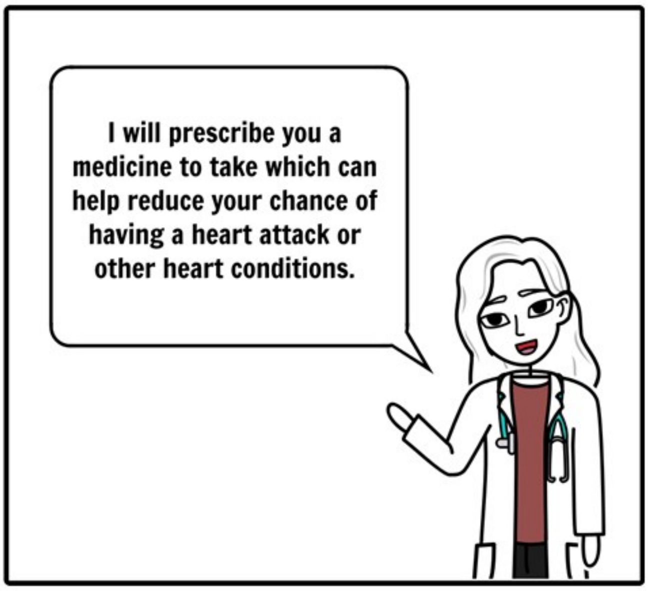

0% 100%

Part 1. Background

Collecting the medicine from the pharmacy

Bobbie goes to a pharmacy to collect his medicine.

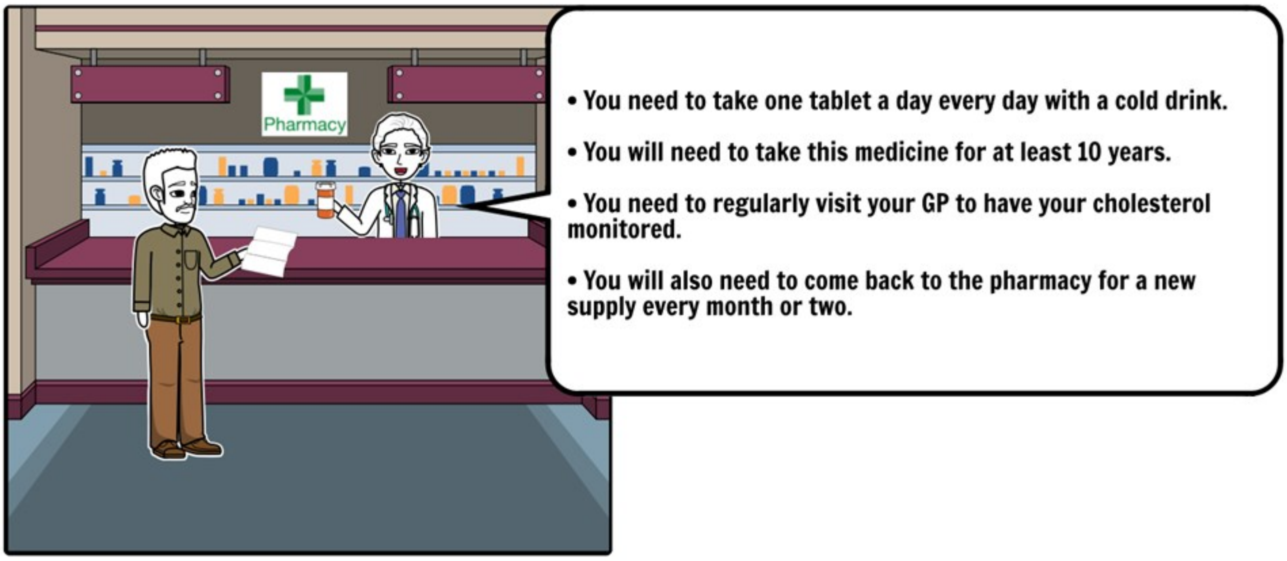

0% 100%

Part 1. Background

Taking the medicine for prevention

Bobbie goes home with his new tablets.

He then has to decide whether he wants to take them as the doctor and the pharmacist have advised.

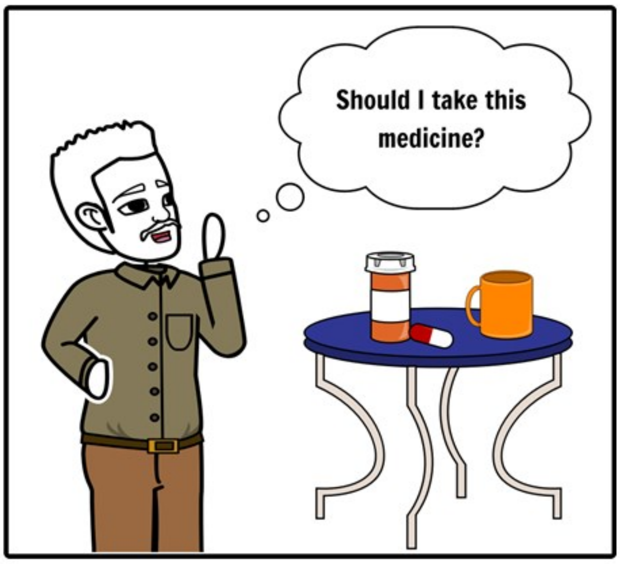

0% 100%

Part 1. Background

- Bobbie may consider three key things, when deciding whether to take the medicine.

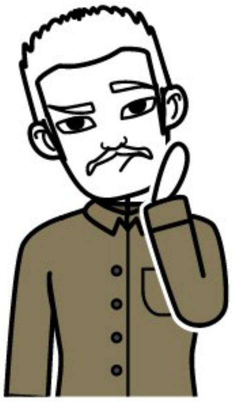

Effectiveness of the medicine

- How good the medicine is in terms of preventing a condition like a heart attack

Side effects of the medicine

- Whether there is any potential harm from the medicine

Inconvenience of the medicine

- Whether taking the medicine would fit in his lifestyle or cause him inconvenience

0% 100%

Part 1. Background

How effective is the medicine?

- Some people, but not everyone, who take the medicine will avoid a serious event, such as a heart attack or stroke as a result of taking the tablet.
- The effectiveness of the medicine can be described in terms of a reduction in this risk.

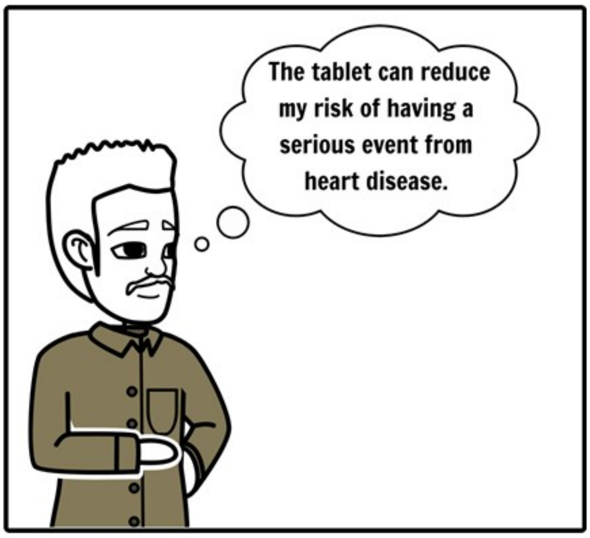

0% 100%

Part 1. Background

What does ‘reduction in risk’ mean?

- A ‘reduction in risk from 10% to 6%’ means:

The medicine will reduce your risk of experiencing an event, such as a heart attack, within the next 10 years from 10% to 6%.

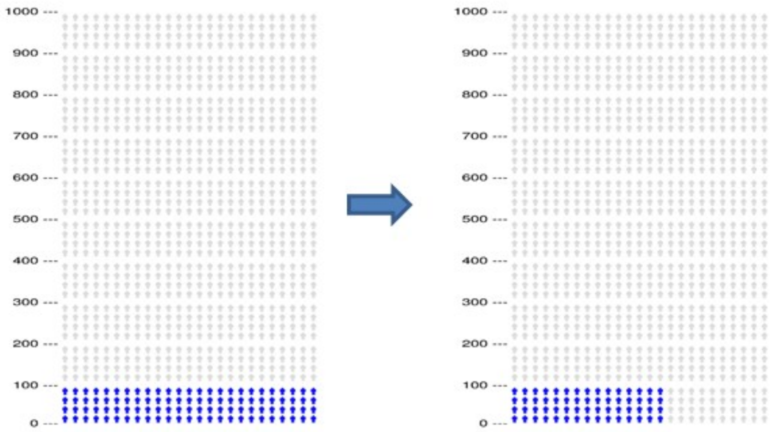

No treatment: 10% risk

Taking the medicine: 6% risk

0% 100%

Part 1. Background

Does the medicine have side effects?

The potential for side effects from the medicine can be described in terms of the risk of a side effect.

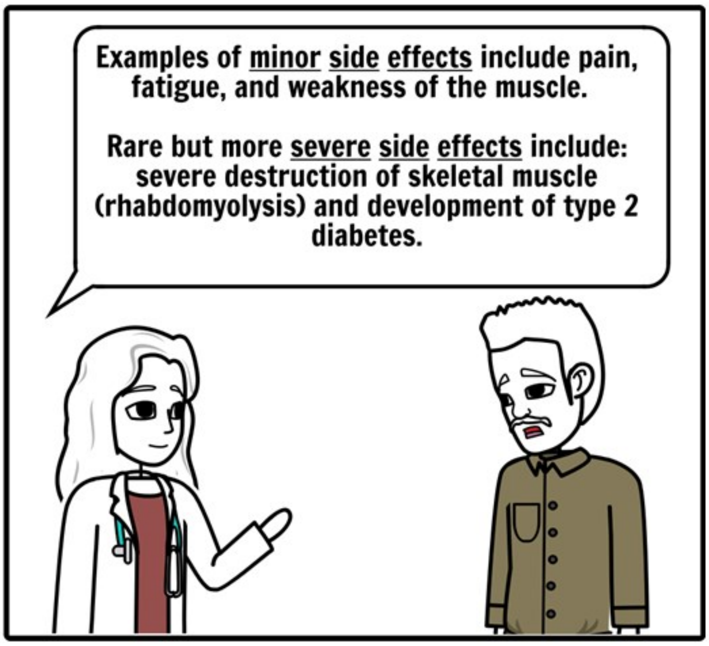

0% 100%

Part 1. Background

Risk of a minor side effect from the medicine

- A 5% risk of a **minor** side effect means:

Among every 1,000 people taking the medicine,

50 will experience a minor side effect such as either: pain, fatigue, and weakness of the muscle.

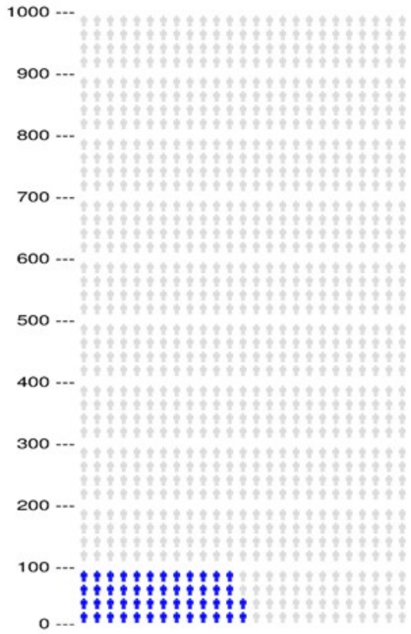

5% risk of a minor side effect

0% 100%

Part 1. Background

Risk of a severe side effect from the medicine

- A 0.1% risk of a **severe** side effect means:

Among every 1,000 people taking the medicine,

1 will experience a severe side effect such as either: severe destruction of skeletal muscle or development of type 2 diabetes.

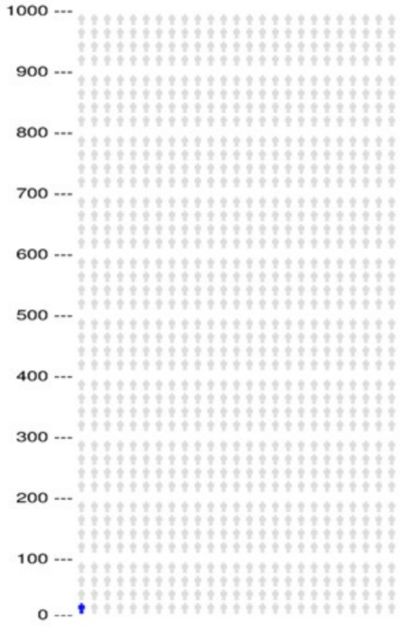

0.1% risk of a severe side effect

0% 100%

Part 1. Background

How inconvenient is it when taking the medicine?

Bobbie also thinks about what it means to have to take this medicine for the next ten years.

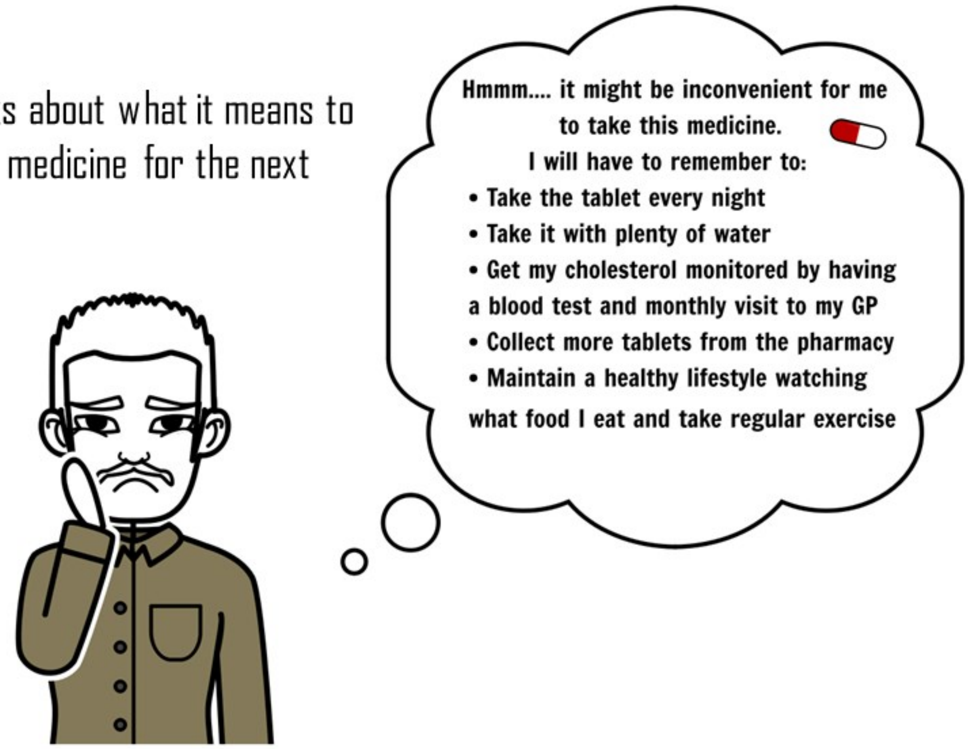

0% 100%

Part 1. Background

In the questions that follow in this survey, we will ask you about how you think about taking a medicine to prevent heart disease.

0% 100%

In general, how would you feel about taking a medicine for the first time?

◯ **Happy**
I trust in my doctor and I am willing to try new things

◯ **Unsure**
I'd like to think about it really carefully

◯ **Unhappy**
I don't like the idea of trying a new medicine even if the doctor says it will help

0% 100%

For peer review only

Part 2: Your views on the inconvenience of taking a medicine to prevent heart disease

Now, we will ask you about how you think about the inconvenience of taking a medicine to prevent heart disease.

There are four questions.

Each question may look similar but differs in terms of the detail for each of the three features.

We will start with a practice question to show you an example of the type of question we will ask you.

0% 100%

eer review only

Warm up question

You have been told that you need to take a medicine to reduce the risk of heart disease.

You can choose between two different medicines. We have called these Medicine A and Medicine B.

Medicine A is a pill that you only need to take once.

Medicine B is a pill that you need to take every day for ten years.

Medicine A and Medicine B are equally effective in reducing your risk of developing heart disease.
Medicine A and Medicine B will reduce your risk of having a heart attack or a stroke within the next 10 years from **10%** to **6%**.

In other words, in a crowd of **1,000** people in the UK, **100** are likely to have a heart attack or a stroke if they do not take the medicine. This is reduced to **60** people out of 1,000 who do take the medicine. This information is also illustrated in this picture.

Risk of heart disease

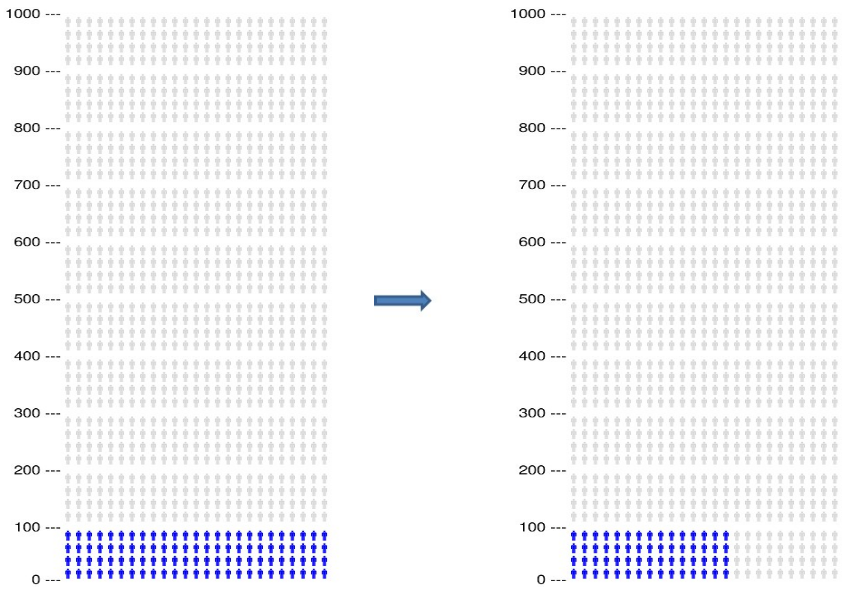

10%
No Treatment

6%
Taking the Medicine

(The figures shaded blue represent people who will have a heart attack or a stroke in the next 10 years)

In this scenario, you will not have any side effects from Medicine A or Medicine B.

Risk of a side effect

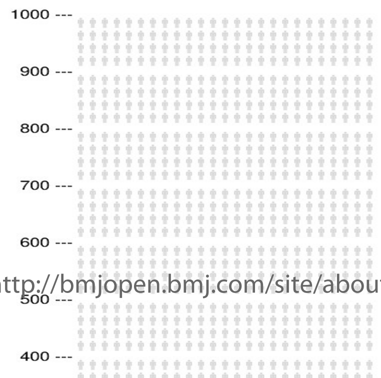

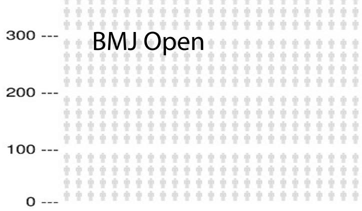

0%
No one will have a side effect.

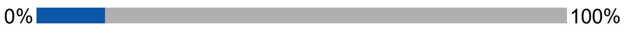

or peer review only

Remember Medicine A and Medicine B are equally effective and have no risks of a side effect.

The only difference between Medicine A and Medicine B is how you take them.

Medicine A	Medicine B
You take a tablet only once in your life.<br>You will be given the tablet on the day you come in to see your GP.<br>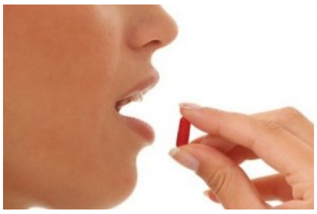	One tablet should be taken once every day, at night with cold water.<br>You need to take this medicine for 10 years.<br>You need to regularly visit a GP to receive a prescription.<br>You need to visit a pharmacy to fill a prescription.<br>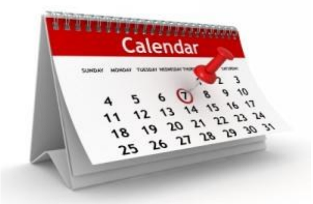 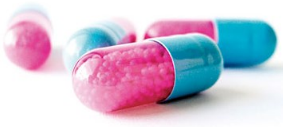

If you take Medicine A, you would live for **1 year** and then die.

If you take Medicine B, you would live for **10 years** and then die.

Would you prefer Medicine A or Medicine B, or are they the same?

Indicate your choice here. You can only choose one option:

Live for 1 year

○ **Medicine A** 1

○ **Medicine B** Live for 10 years

○ **The Same - Medicine A and Medicine B are the same.**

0% 100%

Now we are ready to start the survey questions.

0% 100%

For peer review only

Part 2

Question 1

You have been told that you need to take a medicine to reduce the risk of heart disease.

You can choose between two different medicines. We have called these Medicine A and Medicine B.

Medicine A is a pill that you only need to take once.

Medicine B is a pill that you need to take every day for ten years.

Medicine A and Medicine B are equally effective in reducing your risk of developing heart disease.
Medicine A and Medicine B will reduce your risk of having a heart attack or a stroke within the next 10 years from **10%** to **6%**.

In other words, in a crowd of **1,000** people in the UK, **100** are likely to have a heart attack or a stroke if they do not take the medicine. This is reduced to **60** people out of 1,000 who do take the medicine. This information is also illustrated in this picture.

Risk of heart disease

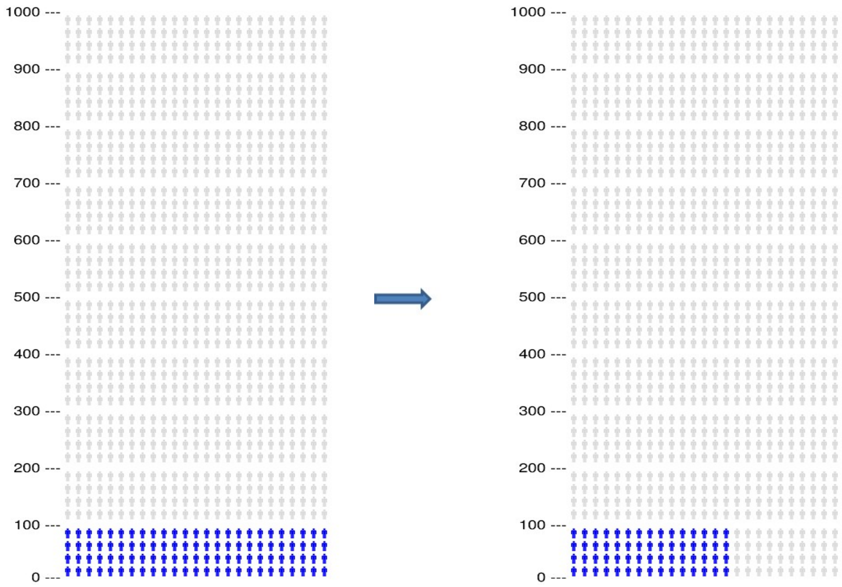

10%
No Treatment

6%
Taking the Medicine

(The figures shaded blue represent people who will have a heart attack or a stroke in the next 10 years)

In this scenario, you will not have any side effects from Medicine A or Medicine B.

Risk of a side effect

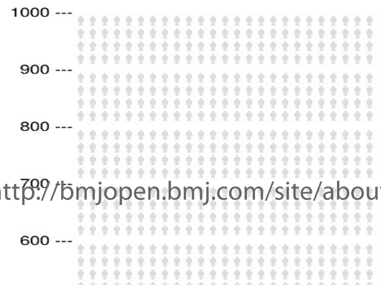

For peer review only - http://bmjopen.bmj.com/site/about/guidelines.xhtml

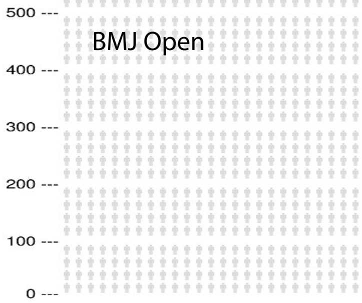

0 %
No one will have a side effect.

0% 100%

peer review only

Repeated section

Remember Medicine A and Medicine B are equally effective and have no risks of a side effect.

The only difference between Medicine A and Medicine B is how you take them.

Medicine A	Medicine B
You take a tablet only once in your life.<br>You will be given the tablet on the day you come in to see your GP.<br>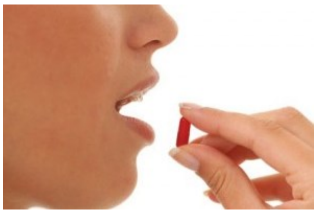	One tablet should be taken once every day, at night with cold water.<br>You need to take this medicine for 10 years.<br>You need to regularly visit a GP to receive a prescription.<br>You need to visit a pharmacy to fill a prescription.<br>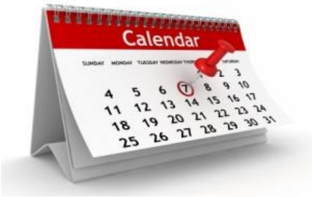<br><br>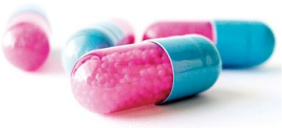

If you take Medicine A, you would live for **5 years** and then die.

If you take Medicine B, you would live for **10 years** and then die.

Would you prefer Medicine A or Medicine B, or are they the same?

Indicate your choice here. You can only choose one option:

○ **Medicine A** Live for 5 years

○ **Medicine B** Live for 10 years

○ **Medicine A and Medicine B are the same.**

0% 100%

For peer review only

Repeated section

Remember Medicine A and Medicine B are equally effective and have no risks of a side effect.

The only difference between Medicine A and Medicine B is how you take them.

**Medicine A**	**Medicine B**
You take a tablet only once in your life.<br>You will be given the tablet on the day you come in to see your GP.<br>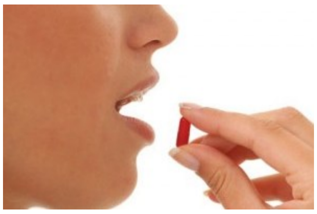	One tablet should be taken once every day, at night with cold water.<br>You need to take this medicine for 10 years.<br>You need to regularly visit a GP to receive a prescription.<br>You need to visit a pharmacy to fill a prescription.<br>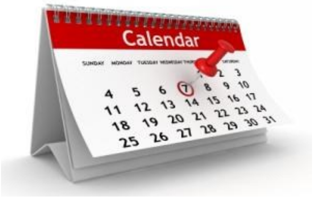<br>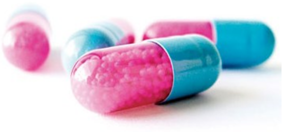

If you take Medicine A, you would live for **5.5 years** (5 years and 6 months) and then die.

If you take Medicine B, you would live for **10 years** and then die.

Would you prefer Medicine A or Medicine B, or are they the same?

Indicate your choice here. You can only choose one option:

◯ **Medicine A** 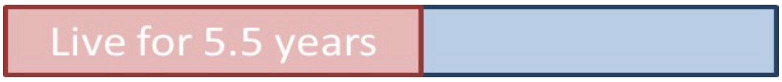

◯ **Medicine B** 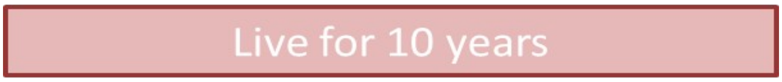

◯ **Medicine A and Medicine B are the same.**

0% 100%

For peer review only

Repeated section

Remember Medicine A and Medicine B are equally effective and have no risks of a side effect.

The only difference between Medicine A and Medicine B is how you take them.

Medicine A	Medicine B
You take a tablet only once in your life.<br>You will be given the tablet on the day you come in to see your GP.<br>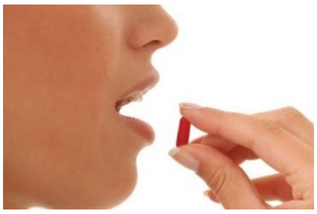	One tablet should be taken once every day, at night with cold water.<br>You need to take this medicine for 10 years.<br>You need to regularly visit a GP to receive a prescription.<br>You need to visit a pharmacy to fill a prescription.<br>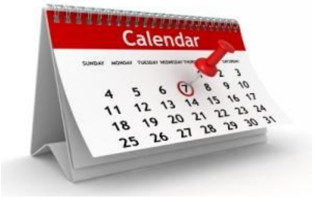<br>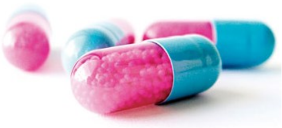

If you take Medicine A, you would live for **6 years** and then die.

If you take Medicine B, you would live for **10 years** and then die.

Would you prefer Medicine A or Medicine B, or are they the same?

Indicate your choice here. You can only choose one option:

◯ **Medicine A** 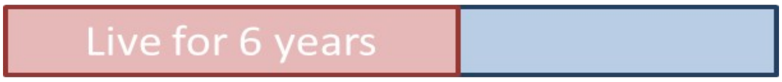

◯ **Medicine B** 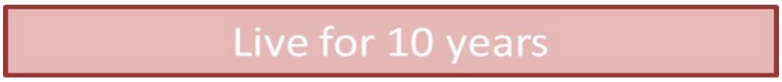

◯ **Medicine A and Medicine B are the same.**

For peer review only

Repeated section

Remember Medicine A and Medicine B are equally effective and have no risks of a side effect.

The only difference between Medicine A and Medicine B is how you take them.

Medicine A	Medicine B
You take a tablet only once in your life.<br>You will be given the tablet on the day you come in to see your GP.<br>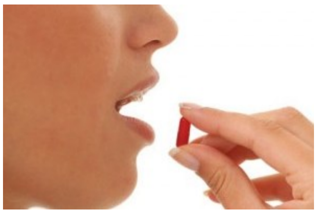	One tablet should be taken once every day, at night with cold water.<br>You need to take this medicine for 10 years.<br>You need to regularly visit a GP to receive a prescription.<br>You need to visit a pharmacy to fill a prescription.<br>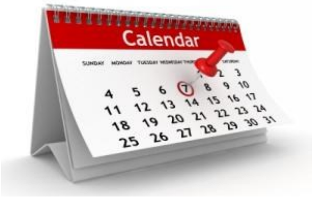<br><br>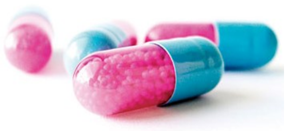

If you take Medicine A, you would live for **6.25 years (6 years and 3 months)** and then die.

If you take Medicine B, you would live for **10 years** and then die.

Would you prefer Medicine A or Medicine B, or are they the same?

Indicate your choice here. You can only choose one option:

◯ **Medicine A** Live for 6 years and 3 months

◯ **Medicine B** Live for 10 years

◯ **Medicine A and Medicine B are the same.**

0% 100%

For peer review only

Repeated section

Remember Medicine A and Medicine B are equally effective and have no risks of a side effect.

The only difference between Medicine A and Medicine B is how you take them.

Medicine A	Medicine B
You take a tablet only once in your life.<br>You will be given the tablet on the day you come in to see your GP.<br>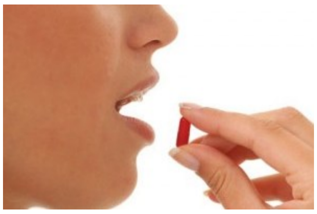	One tablet should be taken once every day, at night with cold water.<br>You need to take this medicine for 10 years.<br>You need to regularly visit a GP to receive a prescription.<br>You need to visit a pharmacy to fill a prescription.<br>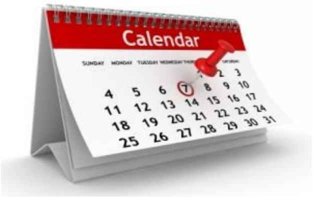<br>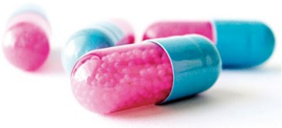

If you take Medicine A, you would live for **6.5 years** (6 years and 6 months) and then die.

If you take Medicine B, you would live for **10 years** and then die.

Would you prefer Medicine A or Medicine B, or are they the same?

Indicate your choice here. You can only choose one option:

◯ **Medicine A** Live for 6.5 years

◯ **Medicine B** Live for 10 years

◯ **Medicine A and Medicine B are the same.**

For peer review only

Repeated section

Remember Medicine A and Medicine B are equally effective and have no risks of a side effect.

The only difference between Medicine A and Medicine B is how you take them.

Medicine A	Medicine B
You take a tablet only once in your life.<br>You will be given the tablet on the day you come in to see your GP.<br>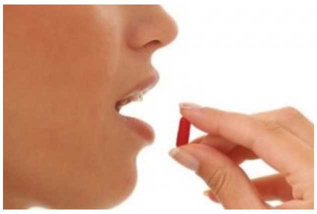	One tablet should be taken once every day, at night with cold water.<br>You need to take this medicine for 10 years.<br>You need to regularly visit a GP to receive a prescription.<br>You need to visit a pharmacy to fill a prescription.<br>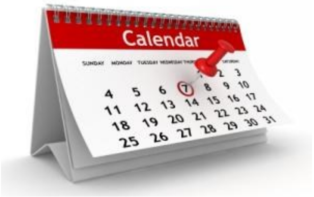<br>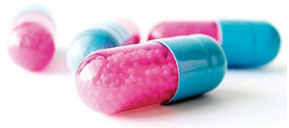

If you take Medicine A, you would live for **7 years** and then die.

If you take Medicine B, you would live for **10 years** and then die.

Would you prefer Medicine A or Medicine B, or are they the same?

Indicate your choice here. You can only choose one option:

◯ **Medicine A** Live for 7 years

◯ **Medicine B** Live for 10 years

◯ **Medicine A and Medicine B are the same.**

For peer review only

Repeated section

Remember Medicine A and Medicine B are equally effective and have no risks of a side effect.

The only difference between Medicine A and Medicine B is how you take them.

Medicine A	Medicine B
You take a tablet only once in your life.<br>You will be given the tablet on the day you come in to see your GP.<br>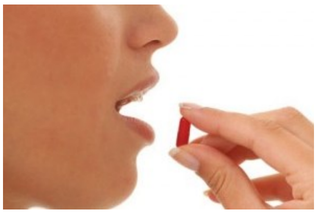	One tablet should be taken once every day, at night with cold water.<br>You need to take this medicine for 10 years.<br>You need to regularly visit a GP to receive a prescription.<br>You need to visit a pharmacy to fill a prescription.<br>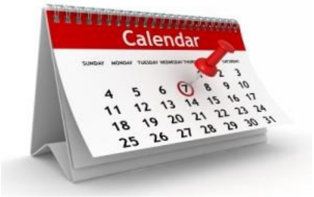<br>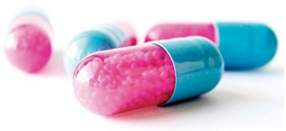

If you take Medicine A, you would live for **7.5 years** (7 years and 6 months) and then die.

If you take Medicine B, you would live for **10 years** and then die.

Would you prefer Medicine A or Medicine B, or are they the same?

Indicate your choice here. You can only choose one option:

◯ **Medicine A** Live for 7.5 years

◯ **Medicine B** Live for 10 years

◯ **Medicine A and Medicine B are the same.**

0% 100%

For peer review only

Repeated section

Remember Medicine A and Medicine B are equally effective and have no risks of a side effect.

The only difference between Medicine A and Medicine B is how you take them.

**Medicine A**	**Medicine B**
You take a tablet only once in your life.<br>You will be given the tablet on the day you come in to see your GP.<br>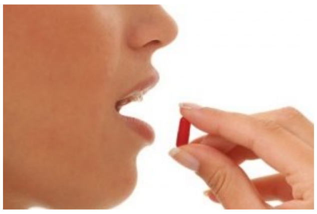	One tablet should be taken once every day, at night with cold water.<br>You need to take this medicine for 10 years.<br>You need to regularly visit a GP to receive a prescription.<br>You need to visit a pharmacy to fill a prescription.<br>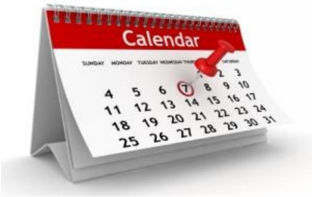<br>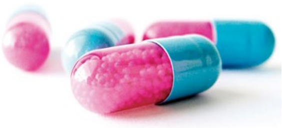

If you take Medicine A, you would live for **7.75 years (7 years and 9 months)** and then die.

If you take Medicine B, you would live for **10 years** and then die.

Would you prefer Medicine A or Medicine B, or are they the same?

Indicate your choice here. You can only choose one option:

◯ **Medicine A** Live for 7 years and 9 months

◯ **Medicine B** Live for 10 years

◯ **Medicine A and Medicine B are the same.**

For peer review only

Repeated section

Remember Medicine A and Medicine B are equally effective and have no risks of a side effect.

The only difference between Medicine A and Medicine B is how you take them.

Medicine A	Medicine B
You take a tablet only once in your life.<br>You will be given the tablet on the day you come in to see your GP.<br>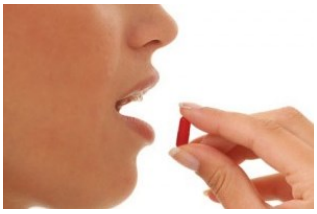	One tablet should be taken once every day, at night with cold water.<br>You need to take this medicine for 10 years.<br>You need to regularly visit a GP to receive a prescription.<br>You need to visit a pharmacy to fill a prescription.<br>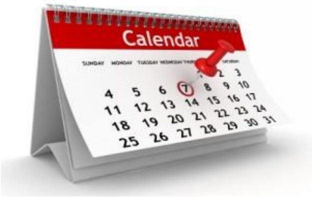 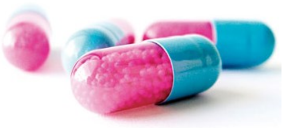

If you take Medicine A, you would live for **8 years** and then die.

If you take Medicine B, you would live for **10 years** and then die.

Would you prefer Medicine A or Medicine B, or are they the same?

Indicate your choice here. You can only choose one option:

◯ **Medicine A** — Live for 8 years

◯ **Medicine B** — Live for 10 years

◯ **Medicine A and Medicine B are the same.**

0% 100%

For peer review only

Repeated section

Remember Medicine A and Medicine B are equally effective and have no risks of a side effect.

The only difference between Medicine A and Medicine B is how you take them.

Medicine A	Medicine B
You take a tablet only once in your life.<br>You will be given the tablet on the day you come in to see your GP.<br>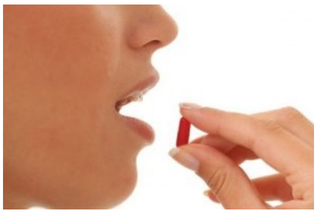	One tablet should be taken once every day, at night with cold water.<br>You need to take this medicine for 10 years.<br>You need to regularly visit a GP to receive a prescription.<br>You need to visit a pharmacy to fill a prescription.<br>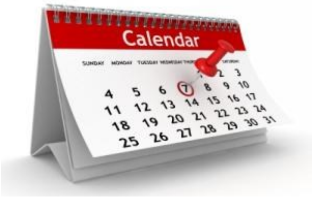<br>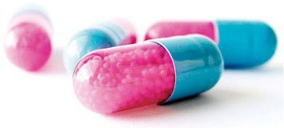

If you take Medicine A, you would live for **8.25 years (8 years and 3 months)** and then die.

If you take Medicine B, you would live for **10 years** and then die.

Would you prefer Medicine A or Medicine B, or are they the same?

Indicate your choice here. You can only choose one option:

○ **Medicine A** Live for 8 years and 3 months

○ **Medicine B** Live for 10 years

○ **Medicine A and Medicine B are the same.**

For peer review only

Repeated section

Remember Medicine A and Medicine B are equally effective and have no risks of a side effect.

The only difference between Medicine A and Medicine B is how you take them.

Medicine A	Medicine B
You take a tablet only once in your life.<br>You will be given the tablet on the day you come in to see your GP.<br>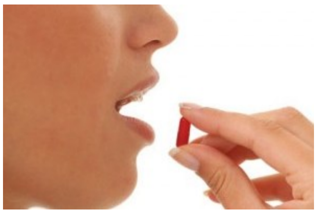	One tablet should be taken once every day, at night with cold water.<br>You need to take this medicine for 10 years.<br>You need to regularly visit a GP to receive a prescription.<br>You need to visit a pharmacy to fill a prescription.<br>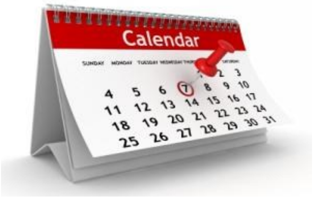<br>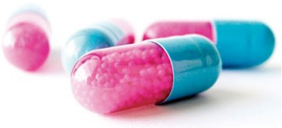

If you take Medicine A, you would live for **8.5 years** (8 years and 6 months) and then die.

If you take Medicine B, you would live for **10 years** and then die.

Would you prefer Medicine A or Medicine B, or are they the same?

Indicate your choice here. You can only choose one option:

○ **Medicine A** Live for 8.5 years

○ **Medicine B** Live for 10 years

○ **Medicine A and Medicine B are the same.**

0% 100%

For peer review only

Repeated section

Remember Medicine A and Medicine B are equally effective and have no risks of a side effect.

The only difference between Medicine A and Medicine B is how you take them.

Medicine A	Medicine B
You take a tablet only once in your life.<br>You will be given the tablet on the day you come in to see your GP.<br>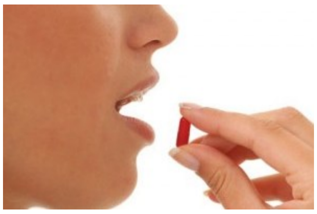	One tablet should be taken once every day, at night with cold water.<br>You need to take this medicine for 10 years.<br>You need to regularly visit a GP to receive a prescription.<br>You need to visit a pharmacy to fill a prescription.<br>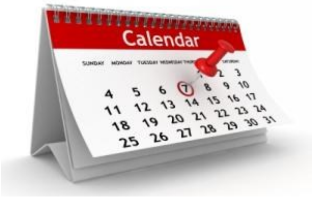 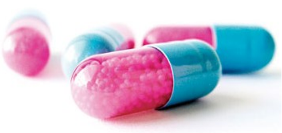

If you take Medicine A, you would live for **8.75 years (8 years and 9 months)** and then die.

If you take Medicine B, you would live for **10 years** and then die.

Would you prefer Medicine A or Medicine B, or are they the same?

Indicate your choice here. You can only choose one option:

◯ **Medicine A** Live for 8 years and 9 months

◯ **Medicine B** Live for 10 years

◯ **Medicine A and Medicine B are the same.**

For peer review only

Repeated section

Remember Medicine A and Medicine B are equally effective and have no risks of a side effect.

The only difference between Medicine A and Medicine B is how you take them.

**Medicine A**	**Medicine B**
You take a tablet only once in your life.<br>You will be given the tablet on the day you come in to see your GP.<br>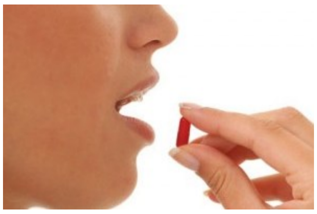	One tablet should be taken once every day, at night with cold water.<br>You need to take this medicine for 10 years.<br>You need to regularly visit a GP to receive a prescription.<br>You need to visit a pharmacy to fill a prescription.<br>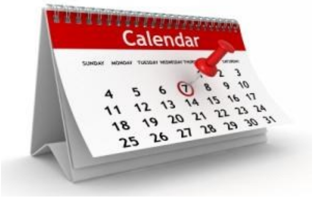<br>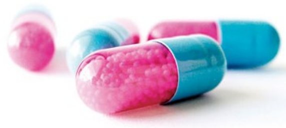

If you take Medicine A, you would live for **9 years** and then die.

If you take Medicine B, you would live for **10 years** and then die.

Would you prefer Medicine A or Medicine B, or are they the same?

Indicate your choice here. You can only choose one option:

◯ **Medicine A** Live for 9 years

◯ **Medicine B** Live for 10 years

◯ **Medicine A and Medicine B are the same.**

0% 100%

For peer review only

Repeated section

Remember Medicine A and Medicine B are equally effective and have no risks of a side effect.

The only difference between Medicine A and Medicine B is how you take them.

**Medicine A**	**Medicine B**
You take a tablet only once in your life.<br>You will be given the tablet on the day you come in to see your GP.<br>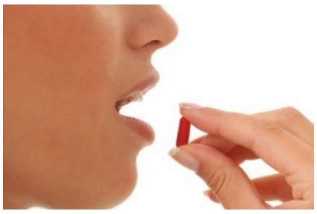	One tablet should be taken once every day, at night with cold water.<br>You need to take this medicine for 10 years.<br>You need to regularly visit a GP to receive a prescription.<br>You need to visit a pharmacy to fill a prescription.<br>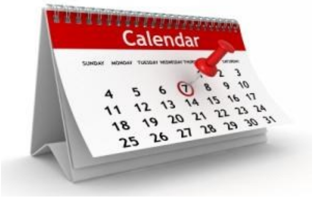<br>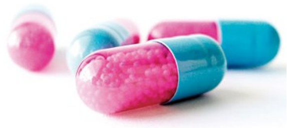

If you take Medicine A, you would live for **9 years and 1 month** and then die.

If you take Medicine B, you would live for **10 years** and then die.

Would you prefer Medicine A or Medicine B, or are they the same?

Indicate your choice here. You can only choose one option:

◯ **Medicine A** Live for 9 years 1 month

◯ **Medicine B** Live for 10 years

◯ **Medicine A and Medicine B are the same.**

0% 100%

For peer review only

Repeated section

Remember Medicine A and Medicine B are equally effective and have no risks of a side effect.

The only difference between Medicine A and Medicine B is how you take them.

Medicine A	Medicine B
You take a tablet only once in your life.<br>You will be given the tablet on the day you come in to see your GP.<br>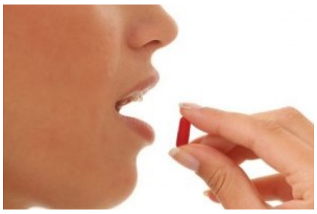	One tablet should be taken once every day, at night with cold water.<br>You need to take this medicine for 10 years.<br>You need to regularly visit a GP to receive a prescription.<br>You need to visit a pharmacy to fill a prescription.<br>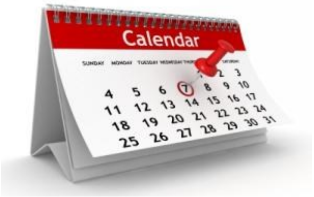<br>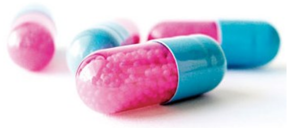

If you take Medicine A, you would live for **9.25 years (9 years and 3 months)** and then die.

If you take Medicine B, you would live for **10 years** and then die.

Would you prefer Medicine A or Medicine B, or are they the same?

Indicate your choice here. You can only choose one option:

◯ **Medicine A** Live for 9 years and 3 months

◯ **Medicine B** Live for 10 years

◯ **Medicine A and Medicine B are the same.**

0% 100%

For peer review only

Repeated section

Remember Medicine A and Medicine B are equally effective and have no risks of a side effect.

The only difference between Medicine A and Medicine B is how you take them.

**Medicine A**	**Medicine B**
You take a tablet only once in your life.<br>You will be given the tablet on the day you come in to see your GP.<br>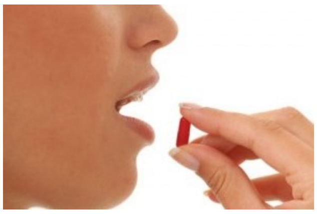	One tablet should be taken once every day, at night with cold water.<br>You need to take this medicine for 10 years.<br>You need to regularly visit a GP to receive a prescription.<br>You need to visit a pharmacy to fill a prescription.<br>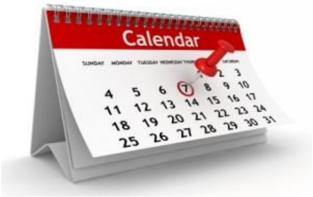<br>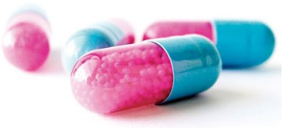

If you take Medicine A, you would live for **9 years and 4 months** and then die.

If you take Medicine B, you would live for **10 years** and then die.

Would you prefer Medicine A or Medicine B, or are they the same?

Indicate your choice here. You can only choose one option:

◯ **Medicine A** Live for 9 years 4 months

◯ **Medicine B** Live for 10 years

◯ **Medicine A and Medicine B are the same.**

0% 100%

For peer review only

Repeated section

Remember Medicine A and Medicine B are equally effective and have no risks of a side effect.

The only difference between Medicine A and Medicine B is how you take them.

Medicine A	Medicine B
You take a tablet only once in your life. You will be given the tablet on the day you come in to see your GP. 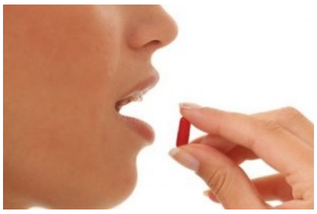	One tablet should be taken once every day, at night with cold water. You need to take this medicine for 10 years. You need to regularly visit a GP to receive a prescription. You need to visit a pharmacy to fill a prescription. 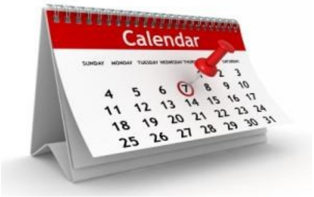 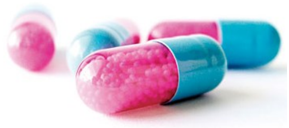

If you take Medicine A, you would live for **9 years and 5 months** and then die.

If you take Medicine B, you would live for **10 years** and then die.

Would you prefer Medicine A or Medicine B, or are they the same?

Indicate your choice here. You can only choose one option:

◯ **Medicine A** Live for 9 years 5 months

◯ **Medicine B** Live for 10 years

◯ **Medicine A and Medicine B are the same.**

For peer review only

Repeated section

Remember Medicine A and Medicine B are equally effective and have no risks of a side effect.

The only difference between Medicine A and Medicine B is how you take them.

Medicine A	Medicine B
You take a tablet only once in your life.<br>You will be given the tablet on the day you come in to see your GP.<br>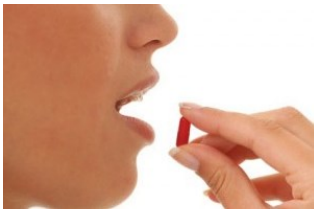	One tablet should be taken once every day, at night with cold water.<br>You need to take this medicine for 10 years.<br>You need to regularly visit a GP to receive a prescription.<br>You need to visit a pharmacy to fill a prescription.<br>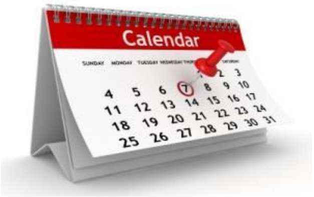<br>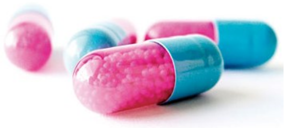

If you take Medicine A, you would live for **9.5 years** (9 years and 6 months) and then die.

If you take Medicine B, you would live for **10 years** and then die.

Would you prefer Medicine A or Medicine B, or are they the same?

Indicate your choice here. You can only choose one option:

○ **Medicine A** Live for 9.5 years

○ **Medicine B** Live for 10 years

○ **Medicine A and Medicine B are the same.**

0% 100%

For peer review only

Repeated section

Remember Medicine A and Medicine B are equally effective and have no risks of a side effect.

The only difference between Medicine A and Medicine B is how you take them.

Medicine A	Medicine B
You take a tablet only once in your life.<br>You will be given the tablet on the day you come in to see your GP.<br>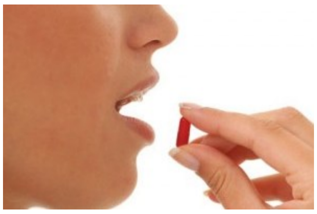	One tablet should be taken once every day, at night with cold water.<br>You need to take this medicine for 10 years.<br>You need to regularly visit a GP to receive a prescription.<br>You need to visit a pharmacy to fill a prescription.<br>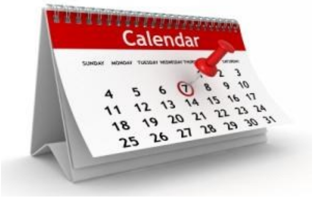<br><br>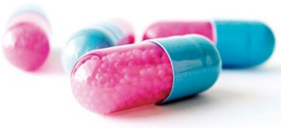

If you take Medicine A, you would live for **5 months less than 10 years** and then die.

If you take Medicine B, you would live for **10 years** and then die.

Would you prefer Medicine A or Medicine B, or are they the same?

Indicate your choice here. You can only choose one option:

◯ **Medicine A** Live for (10 years – 5 months)

◯ **Medicine B** Live for 10 years

◯ **Medicine A and Medicine B are the same.**

For peer review only

Repeated section

Remember Medicine A and Medicine B are equally effective and have no risks of a side effect.

The only difference between Medicine A and Medicine B is how you take them.

Medicine A	Medicine B
You take a tablet only once in your life.<br>You will be given the tablet on the day you come in to see your GP.<br>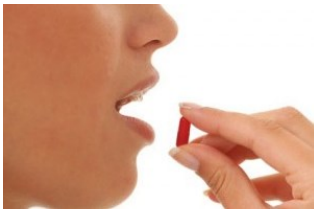	One tablet should be taken once every day, at night with cold water.<br>You need to take this medicine for 10 years.<br>You need to regularly visit a GP to receive a prescription.<br>You need to visit a pharmacy to fill a prescription.<br>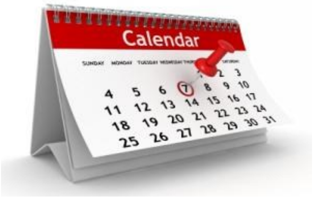<br>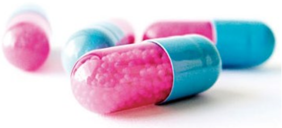

If you take Medicine A, you would live for **4 months less than 10 years** and then die.

If you take Medicine B, you would live for **10 years** and then die.

Would you prefer Medicine A or Medicine B, or are they the same?

Indicate your choice here. You can only choose one option:

◯ **Medicine A** Live for (10 years – 4 months)

◯ **Medicine B** Live for 10 years

◯ **Medicine A and Medicine B are the same.**

0% 100%

For peer review only

Repeated section

Remember Medicine A and Medicine B are equally effective and have no risks of a side effect.

The only difference between Medicine A and Medicine B is how you take them.

Medicine A	Medicine B
You take a tablet only once in your life.<br>You will be given the tablet on the day you come in to see your GP.<br>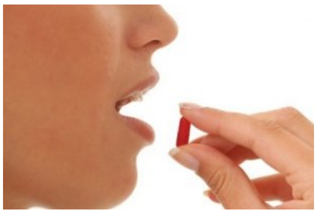	One tablet should be taken once every day, at night with cold water.<br>You need to take this medicine for 10 years.<br>You need to regularly visit a GP to receive a prescription.<br>You need to visit a pharmacy to fill a prescription.<br>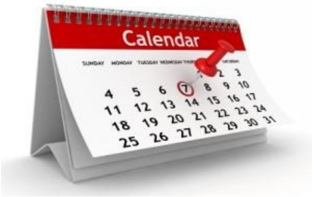<br>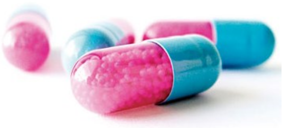

If you take Medicine A, you would live for **3 months less than 10 years** and then die.

If you take Medicine B, you would live for **10 years** and then die.

Would you prefer Medicine A or Medicine B, or are they the same?

Indicate your choice here. You can only choose one option:

○ **Medicine A** Live for (10 years – 3 months)

○ **Medicine B** Live for 10 years

○ **Medicine A and Medicine B are the same.**

0% 100%

For peer review only

Repeated section

Remember Medicine A and Medicine B are equally effective and have no risks of a side effect.

The only difference between Medicine A and Medicine B is how you take them.

Medicine A	Medicine B
You take a tablet only once in your life.<br>You will be given the tablet on the day you come in to see your GP.<br>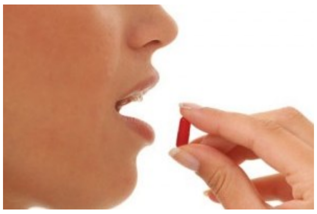	One tablet should be taken once every day, at night with cold water.<br>You need to take this medicine for 10 years.<br>You need to regularly visit a GP to receive a prescription.<br>You need to visit a pharmacy to fill a prescription.<br>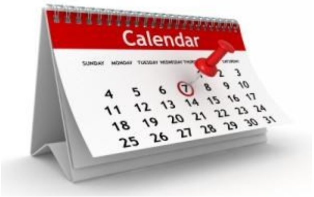<br>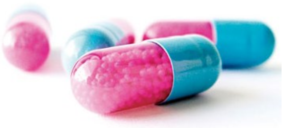

If you take Medicine A, you would live for **2 months less than 10 years** and then die.

If you take Medicine B, you would live for **10 years** and then die.

Would you prefer Medicine A or Medicine B, or are they the same?

Indicate your choice here. You can only choose one option:

○ **Medicine A** Live for (10 years – 2 months)

○ **Medicine B** Live for 10 years

○ **Medicine A and Medicine B are the same.**

For peer review only

Repeated section

Remember Medicine A and Medicine B are equally effective and have no risks of a side effect.

The only difference between Medicine A and Medicine B is how you take them.

Medicine A	Medicine B
You take a tablet only once in your life.<br>You will be given the tablet on the day you come in to see your GP.<br>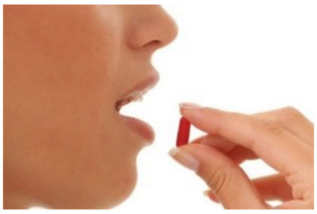	One tablet should be taken once every day, at night with cold water.<br>You need to take this medicine for 10 years.<br>You need to regularly visit a GP to receive a prescription.<br>You need to visit a pharmacy to fill a prescription.<br>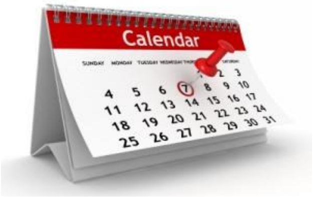<br>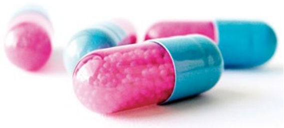

If you take Medicine A, you would live for **1 month less than 10 years** and then die.

If you take Medicine B, you would live for **10 years** and then die.

Would you prefer Medicine A or Medicine B, or are they the same?

Indicate your choice here. You can only choose one option:

◯ **Medicine A** Live for (10 years – 1 month)

◯ **Medicine B** Live for 10 years

◯ **Medicine A and Medicine B are the same.**

0% 100%

For peer review only

Repeated section

Remember Medicine A and Medicine B are equally effective and have no risks of a side effect.

The only difference between Medicine A and Medicine B is how you take them.

Medicine A	Medicine B
You take a tablet only once in your life.<br>You will be given the tablet on the day you come in to see your GP.<br>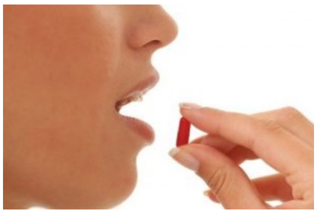	One tablet should be taken once every day, at night with cold water.<br>You need to take this medicine for 10 years.<br>You need to regularly visit a GP to receive a prescription.<br>You need to visit a pharmacy to fill a prescription.<br>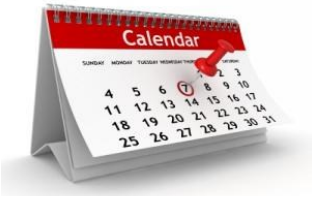<br><br>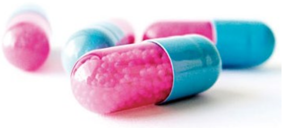

If you take Medicine A, you would live for **3 weeks less than 10 years** and then die.

If you take Medicine B, you would live for **10 years** and then die.

Would you prefer Medicine A or Medicine B, or are they the same?

Indicate your choice here. You can only choose one option:

◯ **Medicine A** Live for (10 years – 3 weeks)

◯ **Medicine B** Live for 10 years

◯ **Medicine A and Medicine B are the same.**

0% 100%

For peer review only

Repeated section

Remember Medicine A and Medicine B are equally effective and have no risks of a side effect.

The only difference between Medicine A and Medicine B is how you take them.

Medicine A	Medicine B
You take a tablet only once in your life.<br>You will be given the tablet on the day you come in to see your GP.<br>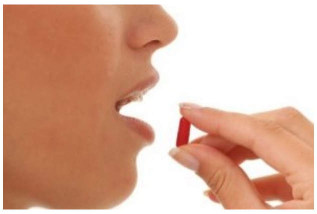	One tablet should be taken once every day, at night with cold water.<br>You need to take this medicine for 10 years.<br>You need to regularly visit a GP to receive a prescription.<br>You need to visit a pharmacy to fill a prescription.<br>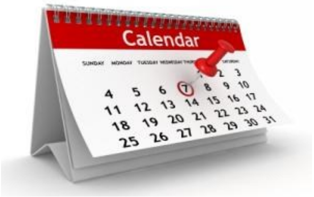<br>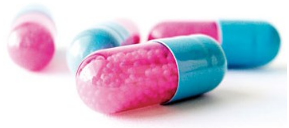

If you take Medicine A, you would live for **2 weeks less than 10 years** and then die.

If you take Medicine B, you would live for **10 years** and then die.

Would you prefer Medicine A or Medicine B, or are they the same?

Indicate your choice here. You can only choose one option:

◯ **Medicine A** Live for (10 years – 2 weeks)

◯ **Medicine B** Live for 10 years

◯ **Medicine A and Medicine B are the same.**

0% 100%

For peer review only

Repeated section

Remember Medicine A and Medicine B are equally effective and have no risks of a side effect.

The only difference between Medicine A and Medicine B is how you take them.

Medicine A	Medicine B
You take a tablet only once in your life.<br>You will be given the tablet on the day you come in to see your GP.<br>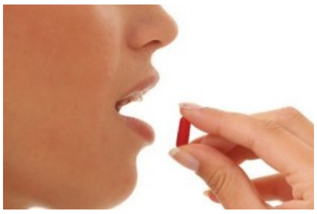	One tablet should be taken once every day, at night with cold water.<br>You need to take this medicine for 10 years.<br>You need to regularly visit a GP to receive a prescription.<br>You need to visit a pharmacy to fill a prescription.<br>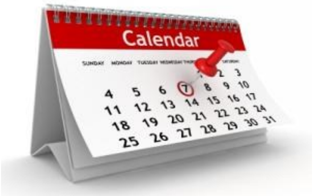<br><br>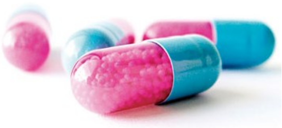

If you take Medicine A, you would live for **10 days less than 10 years** and then die.

If you take Medicine B, you would live for **10 years** and then die.

Would you prefer Medicine A or Medicine B, or are they the same?

Indicate your choice here. You can only choose one option:

◯ **Medicine A** Live for (10 years – 10 days)

◯ **Medicine B** Live for 10 years

◯ **Medicine A and Medicine B are the same.**

For peer review only

Repeated section

Remember Medicine A and Medicine B are equally effective and have no risks of a side effect.

The only difference between Medicine A and Medicine B is how you take them.

Medicine A	Medicine B
You take a tablet only once in your life.<br>You will be given the tablet on the day you come in to see your GP.<br>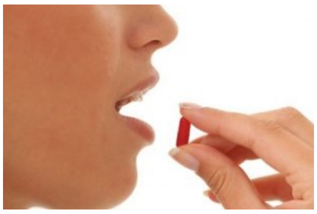	One tablet should be taken once every day, at night with cold water.<br>You need to take this medicine for 10 years.<br>You need to regularly visit a GP to receive a prescription.<br>You need to visit a pharmacy to fill a prescription.<br>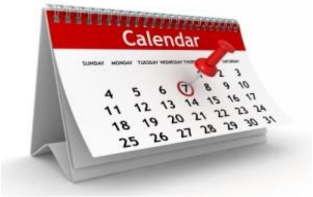 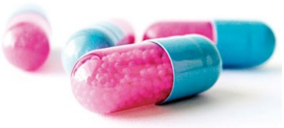

If you take Medicine A, you would live for **1 week less than 10 years** and then die.

If you take Medicine B, you would live for **10 years** and then die.

Would you prefer Medicine A or Medicine B, or are they the same?

Indicate your choice here. You can only choose one option:

◯ **Medicine A** Live for (10 years – 1 week)

◯ **Medicine B** Live for 10 years

◯ **Medicine A and Medicine B are the same.**

For peer review only

Repeated section

Remember Medicine A and Medicine B are equally effective and have no risks of a side effect.

The only difference between Medicine A and Medicine B is how you take them.

**Medicine A**	**Medicine B**
You take a tablet only once in your life.<br>You will be given the tablet on the day you come in to see your GP.<br>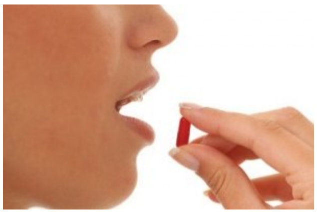	One tablet should be taken once every day, at night with cold water.<br>You need to take this medicine for 10 years.<br>You need to regularly visit a GP to receive a prescription.<br>You need to visit a pharmacy to fill a prescription.<br>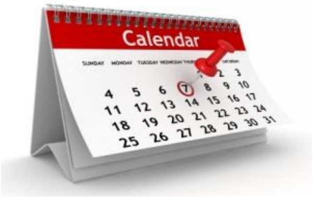<br>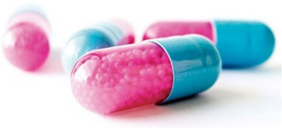

If you take Medicine A, you would live for **5 days less than 10 years** and then die.

If you take Medicine B, you would live for **10 years** and then die.

Would you prefer Medicine A or Medicine B, or are they the same?

Indicate your choice here. You can only choose one option:

- ◯ **Medicine A** — Live for (10 years – 5 days)
- ◯ **Medicine B** — Live for 10 years
- ◯ **Medicine A and Medicine B are the same.**

For peer review only

Repeated section

Remember Medicine A and Medicine B are equally effective and have no risks of a side effect.

The only difference between Medicine A and Medicine B is how you take them.

Medicine A	Medicine B
You take a tablet only once in your life.<br>You will be given the tablet on the day you come in to see your GP.<br>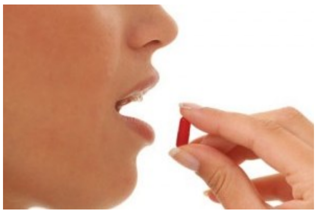	One tablet should be taken once every day, at night with cold water.<br>You need to take this medicine for 10 years.<br>You need to regularly visit a GP to receive a prescription.<br>You need to visit a pharmacy to fill a prescription.<br>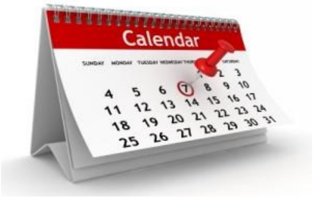<br>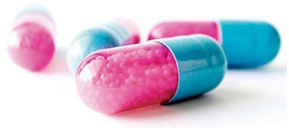

If you take Medicine A, you would live for **3 days less than 10 years** and then die.

If you take Medicine B, you would live for **10 years** and then die.

Would you prefer Medicine A or Medicine B, or are they the same?

Indicate your choice here. You can only choose one option:

○ **Medicine A** Live for (10 years – 3 days)

○ **Medicine B** Live for 10 years

○ **Medicine A and Medicine B are the same.**

0% 100%

For peer review only

Repeated section

Remember Medicine A and Medicine B are equally effective and have no risks of a side effect.

The only difference between Medicine A and Medicine B is how you take them.

Medicine A	Medicine B
You take a tablet only once in your life.<br>You will be given the tablet on the day you come in to see your GP.<br>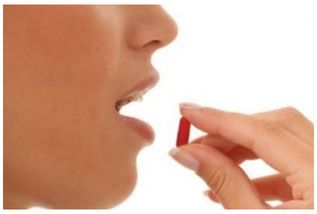	One tablet should be taken once every day, at night with cold water.<br>You need to take this medicine for 10 years.<br>You need to regularly visit a GP to receive a prescription.<br>You need to visit a pharmacy to fill a prescription.<br>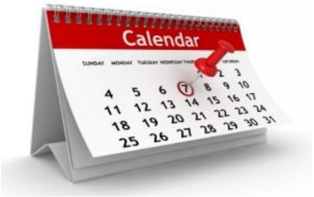<br>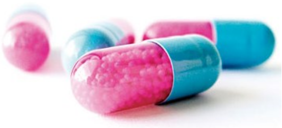

If you take Medicine A, you would live for **1 day less than 10 years** and then die.

If you take Medicine B, you would live for **10 years** and then die.

Would you prefer Medicine A or Medicine B, or are they the same?

Indicate your choice here. You can only choose one option:

○ **Medicine A** — Live for (10 years – 1 day)

○ **Medicine B** — Live for 10 years

○ **Medicine A and Medicine B are the same.**

For peer review only - http://bmjopen.bmj.com/site/about/guidelines.xhtml

0% 100%

For peer review only

Question 1a.

You prefer living for 5 years on Medicine A, to living for 10 years on Medicine B.

This means **living fewer than 5 years on Medicine A** can be the same as living for 10 years on Medicine B.

How many years on Medicine A do you think would be the same as living for 10 years on Medicine B?

(Please give a number in years)

0% 100%

peer review only

Part 2

Question 2

Now, we will assume that there is some risk of a minor side effect from either medicine.

As before,

Medicine A is a pill that you only need to take once.

Medicine B is a pill that you need to take every day for ten years.

Medicine A and Medicine B are equally effective in reducing your risk of developing heart disease.

Medicine A and Medicine B will reduce your risk of having a heart attack or a stroke within the next 10 years from **10%** to **6%**.

In other words, in a crowd of 1,000 people in the UK, **100** are likely to have a heart attack or a stroke if they do not take the medicine. This is reduced to **60** people out of 1,000 who do take the medicine. This information is also illustrated in this picture.

Risk of heart disease

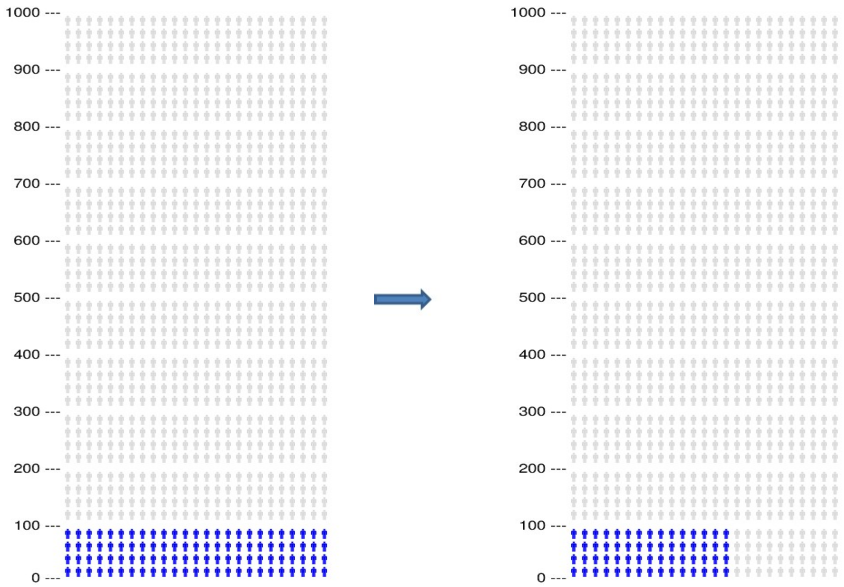

10%
No Treatment

6%
Taking the Medicine

(The figures shaded blue represent people who will have a heart attack or a stroke in the next 10 years)

In this scenario, there is some risk of a minor side effect from Medicine A or Medicine B.

Among every 1,000 people taking Medicine A or Medicine B, **30** people are likely to have a minor side effect such as muscle pain and fatigue.

Risk of a minor side effect

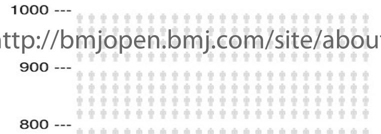

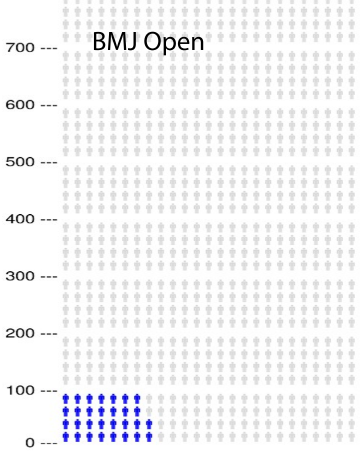

3% of people will have a minor side effect.

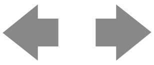

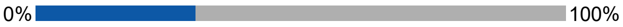

For peer review only

Repeated section

Remember Medicine A and Medicine B are equally effective and have **some risk of a side effect**.

The only difference between Medicine A and Medicine B is how you take them.

Medicine A	Medicine B
You take a tablet only once in your life.<br>You will be given the tablet on the day you come in to see your GP.<br>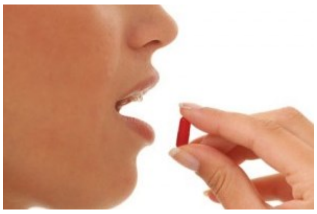	One tablet should be taken once every day, at night with cold water.<br>You need to take this medicine for 10 years.<br>You need to regularly visit a GP to receive a prescription.<br>You need to visit a pharmacy to fill a prescription.<br>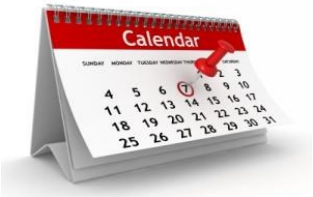<br>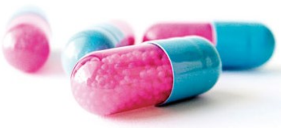

If you take Medicine A, you would live for **5 years** and then die.

If you take Medicine B, you would live for **10 years** and then die.

Would you prefer Medicine A or Medicine B, or are they the same?

Indicate your choice here. You can only choose one option:

◯ **Medicine A** 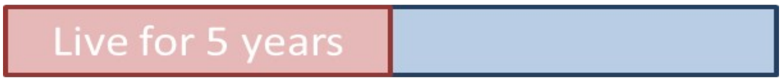

◯ **Medicine B** 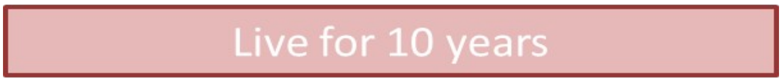

◯ **Medicine A and Medicine B are the same.**

0% 100%

For peer review only

Repeated section

Remember Medicine A and Medicine B are equally effective and have **some risk of a side effect**.

The only difference between Medicine A and Medicine B is how you take them.

Medicine A	Medicine B
You take a tablet only once in your life.<br>You will be given the tablet on the day you come in to see your GP.<br>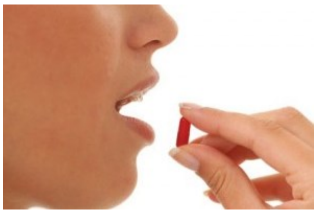	One tablet should be taken once every day, at night with cold water.<br>You need to take this medicine for 10 years.<br>You need to regularly visit a GP to receive a prescription.<br>You need to visit a pharmacy to fill a prescription.<br>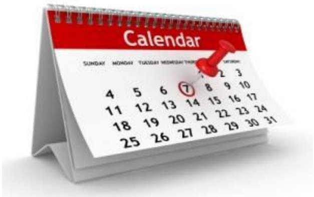<br>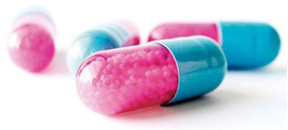

If you take Medicine A, you would live for **5.5 years** (5 years and 6 months) and then die.

If you take Medicine B, you would live for **10 years** and then die.

Would you prefer Medicine A or Medicine B, or are they the same?

Indicate your choice here. You can only choose one option:

◯ **Medicine A** 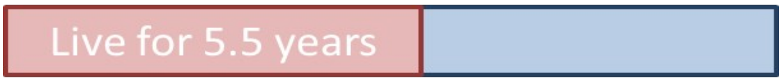

◯ **Medicine B** 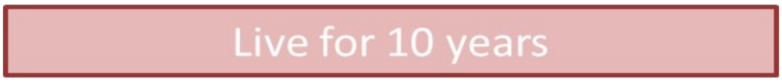

◯ **Medicine A and Medicine B are the same.**

For peer review only

Repeated section

Remember Medicine A and Medicine B are equally effective and have **some risk of a side effect**.

The only difference between Medicine A and Medicine B is how you take them.

Medicine A	Medicine B
You take a tablet only once in your life.<br>You will be given the tablet on the day you come in to see your GP.<br>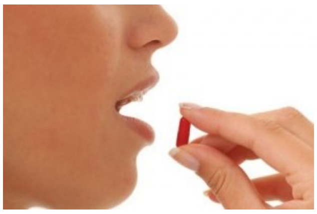	One tablet should be taken once every day, at night with cold water.<br>You need to take this medicine for 10 years.<br>You need to regularly visit a GP to receive a prescription.<br>You need to visit a pharmacy to fill a prescription.<br>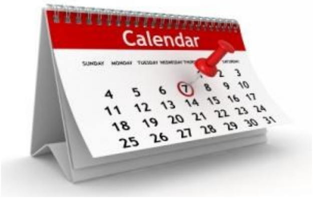<br>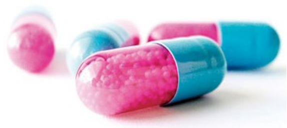

If you take Medicine A, you would live for **6 years** and then die.

If you take Medicine B, you would live for **10 years** and then die.

Would you prefer Medicine A or Medicine B, or are they the same?

Indicate your choice here. You can only choose one option:

◯ **Medicine A** Live for 6 years

◯ **Medicine B** Live for 10 years

◯ **Medicine A and Medicine B are the same.**

For peer review only

Repeated section

Remember Medicine A and Medicine B are equally effective and have **some risk of a side effect**.

The only difference between Medicine A and Medicine B is how you take them.

**Medicine A**	**Medicine B**
You take a tablet only once in your life.<br>You will be given the tablet on the day you come in to see your GP.<br>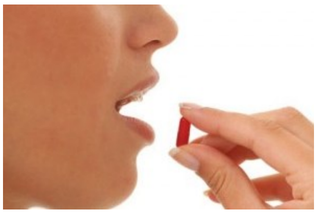	One tablet should be taken once every day, at night with cold water.<br>You need to take this medicine for 10 years.<br>You need to regularly visit a GP to receive a prescription.<br>You need to visit a pharmacy to fill a prescription.<br>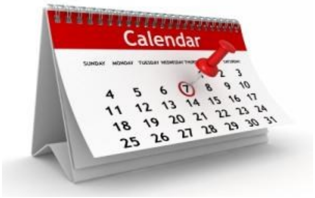<br>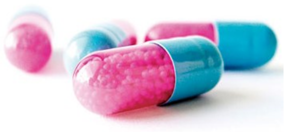

If you take Medicine A, you would live for **6.25 years (6 years and 3 months)** and then die.

If you take Medicine B, you would live for **10 years** and then die.

Would you prefer Medicine A or Medicine B, or are they the same?

Indicate your choice here. You can only choose one option:

○ **Medicine A** Live for 6 years and 3 months

○ **Medicine B** Live for 10 years

○ **Medicine A and Medicine B are the same.**

For peer review only

Repeated section

Remember Medicine A and Medicine B are equally effective and have **some risk of a side effect**.

The only difference between Medicine A and Medicine B is how you take them.

**Medicine A**	**Medicine B**
You take a tablet only once in your life.<br>You will be given the tablet on the day you come in to see your GP.<br>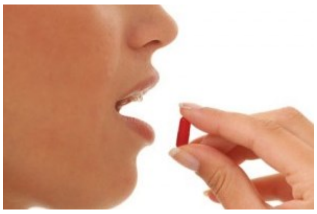	One tablet should be taken once every day, at night with cold water.<br>You need to take this medicine for 10 years.<br>You need to regularly visit a GP to receive a prescription.<br>You need to visit a pharmacy to fill a prescription.<br>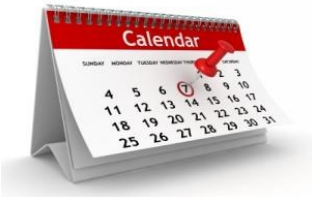<br>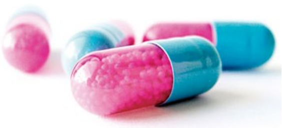

If you take Medicine A, you would live for **6.5 years** (6 years and 6 months) and then die.

If you take Medicine B, you would live for **10 years** and then die.

Would you prefer Medicine A or Medicine B, or are they the same?

Indicate your choice here. You can only choose one option:

○ **Medicine A** Live for 6.5 years

○ **Medicine B** Live for 10 years

○ **Medicine A and Medicine B are the same.**

0% 100%

For peer review only

Repeated section

Remember Medicine A and Medicine B are equally effective and have **some risk of a side effect**.

The only difference between Medicine A and Medicine B is how you take them.

**Medicine A**	**Medicine B**
You take a tablet only once in your life.<br>You will be given the tablet on the day you come in to see your GP.<br>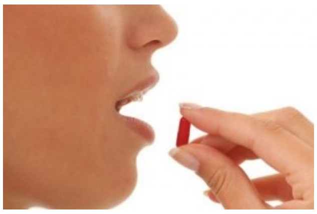	One tablet should be taken once every day, at night with cold water.<br>You need to take this medicine for 10 years.<br>You need to regularly visit a GP to receive a prescription.<br>You need to visit a pharmacy to fill a prescription.<br>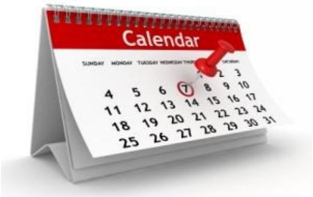<br>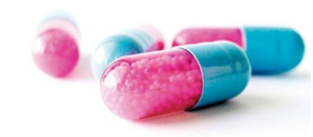

If you take Medicine A, you would live for **7 years** and then die.

If you take Medicine B, you would live for **10 years** and then die.

Would you prefer Medicine A or Medicine B, or are they the same?

Indicate your choice here. You can only choose one option:

◯ **Medicine A** 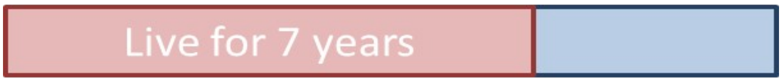

◯ **Medicine B** 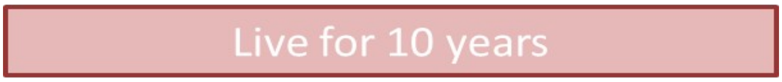

◯ **Medicine A and Medicine B are the same.**

0% 100%

For peer review only

Repeated section

Remember Medicine A and Medicine B are equally effective and have **some risk of a side effect**.

The only difference between Medicine A and Medicine B is how you take them.

Medicine A	Medicine B
You take a tablet only once in your life.<br>You will be given the tablet on the day you come in to see your GP.<br>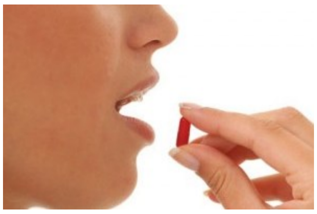	One tablet should be taken once every day, at night with cold water.<br>You need to take this medicine for 10 years.<br>You need to regularly visit a GP to receive a prescription.<br>You need to visit a pharmacy to fill a prescription.<br>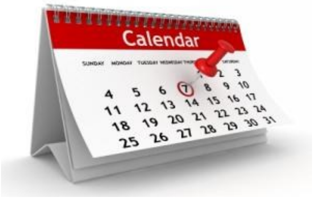<br><br>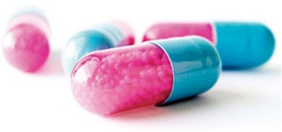

If you take Medicine A, you would live for **7.5 years** (7 years and 6 months) and then die.

If you take Medicine B, you would live for **10 years** and then die.

Would you prefer Medicine A or Medicine B, or are they the same?

Indicate your choice here. You can only choose one option:

○ **Medicine A**

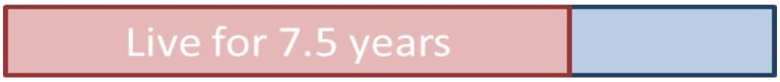

○ **Medicine B**

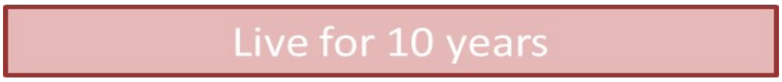

○ **Medicine A and Medicine B are the same.**

For peer review only

Repeated section

Remember Medicine A and Medicine B are equally effective and have **some risk of a side effect**.

The only difference between Medicine A and Medicine B is how you take them.

**Medicine A**	**Medicine B**
You take a tablet only once in your life.<br>You will be given the tablet on the day you come in to see your GP.<br>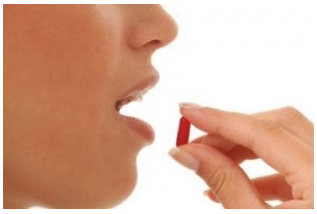	One tablet should be taken once every day, at night with cold water.<br>You need to take this medicine for 10 years.<br>You need to regularly visit a GP to receive a prescription.<br>You need to visit a pharmacy to fill a prescription.<br>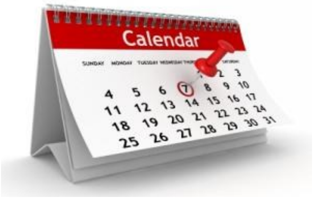<br>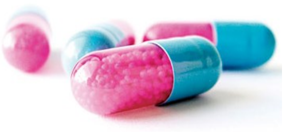

If you take Medicine A, you would live for **7.75 years (7 years and 9 months)** and then die.

If you take Medicine B, you would live for **10 years** and then die.

Would you prefer Medicine A or Medicine B, or are they the same?

Indicate your choice here. You can only choose one option:

○ **Medicine A** Live for 7 years and 9 months

○ **Medicine B** Live for 10 years

○ **Medicine A and Medicine B are the same.**

0% 100%

For peer review only

Repeated section

Remember Medicine A and Medicine B are equally effective and have **some risk of a side effect**.

The only difference between Medicine A and Medicine B is how you take them.

**Medicine A**	**Medicine B**
You take a tablet only once in your life.<br>You will be given the tablet on the day you come in to see your GP.<br>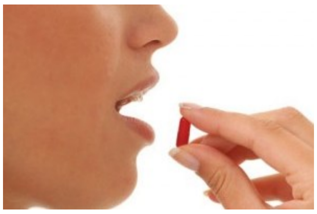	One tablet should be taken once every day, at night with cold water.<br>You need to take this medicine for 10 years.<br>You need to regularly visit a GP to receive a prescription.<br>You need to visit a pharmacy to fill a prescription.<br>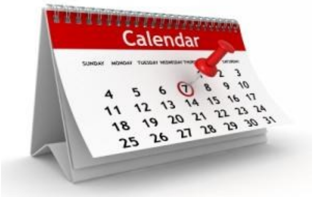<br>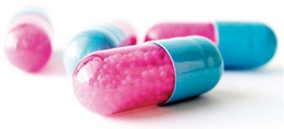

If you take Medicine A, you would live for **8 years** and then die.

If you take Medicine B, you would live for **10 years** and then die.

Would you prefer Medicine A or Medicine B, or are they the same?

Indicate your choice here. You can only choose one option:

◯ **Medicine A** Live for 8 years

◯ **Medicine B** Live for 10 years

◯ **Medicine A and Medicine B are the same.**

For peer review only

Repeated section

Remember Medicine A and Medicine B are equally effective and have **some risk of a side effect**.

The only difference between Medicine A and Medicine B is how you take them.

Medicine A	Medicine B
You take a tablet only once in your life.<br>You will be given the tablet on the day you come in to see your GP.<br>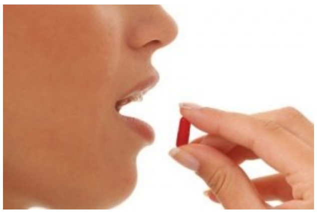	One tablet should be taken once every day, at night with cold water.<br>You need to take this medicine for 10 years.<br>You need to regularly visit a GP to receive a prescription.<br>You need to visit a pharmacy to fill a prescription.<br>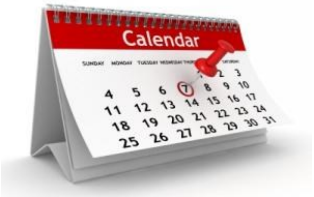<br>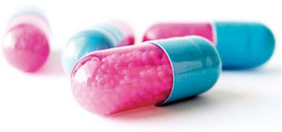

If you take Medicine A, you would live for **8.25 years (8 years and 3 months)** and then die.

If you take Medicine B, you would live for **10 years** and then die.

Would you prefer Medicine A or Medicine B, or are they the same?

Indicate your choice here. You can only choose one option:

○ **Medicine A** Live for 8 years and 3 months

○ **Medicine B** Live for 10 years

○ **Medicine A and Medicine B are the same.**

For peer review only

Repeated section

Remember Medicine A and Medicine B are equally effective and have **some risk of a side effect**.

The only difference between Medicine A and Medicine B is how you take them.

**Medicine A**	**Medicine B**
You take a tablet only once in your life.<br>You will be given the tablet on the day you come in to see your GP.<br>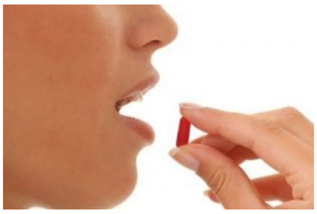	One tablet should be taken once every day, at night with cold water.<br>You need to take this medicine for 10 years.<br>You need to regularly visit a GP to receive a prescription.<br>You need to visit a pharmacy to fill a prescription.<br>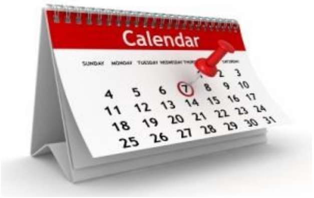<br>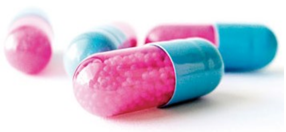

If you take Medicine A, you would live for **8.5 years** (8 years and 6 months) and then die.

If you take Medicine B, you would live for **10 years** and then die.

Would you prefer Medicine A or Medicine B, or are they the same?

Indicate your choice here. You can only choose one option:

◯ **Medicine A** 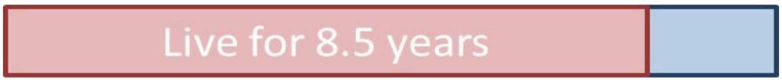

◯ **Medicine B** 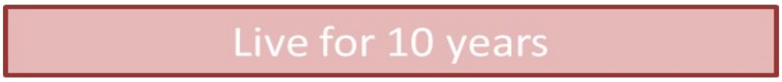

◯ **Medicine A and Medicine B are the same.**

0% 100%

For peer review only

Repeated section

Remember Medicine A and Medicine B are equally effective and have **some risk of a side effect**.

The only difference between Medicine A and Medicine B is how you take them.

**Medicine A**	**Medicine B**
You take a tablet only once in your life.<br>You will be given the tablet on the day you come in to see your GP.<br>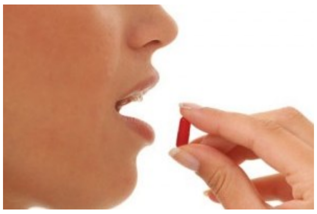	One tablet should be taken once every day, at night with cold water.<br>You need to take this medicine for 10 years.<br>You need to regularly visit a GP to receive a prescription.<br>You need to visit a pharmacy to fill a prescription.<br>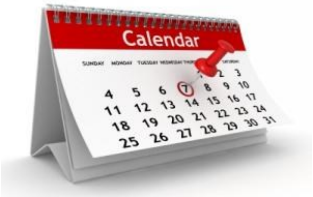<br><br>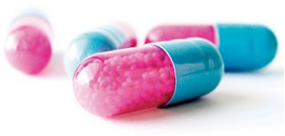

If you take Medicine A, you would live for **8.75 years (8 years and 9 months)** and then die.

If you take Medicine B, you would live for **10 years** and then die.

Would you prefer Medicine A or Medicine B, or are they the same?

Indicate your choice here. You can only choose one option:

- ◯ **Medicine A** — Live for 8 years and 9 months
- ◯ **Medicine B** — Live for 10 years
- ◯ **Medicine A and Medicine B are the same.**

0% 100%

For peer review only

Repeated section

Remember Medicine A and Medicine B are equally effective and have **some risk of a side effect**.

The only difference between Medicine A and Medicine B is how you take them.

**Medicine A**	**Medicine B**
You take a tablet only once in your life.<br>You will be given the tablet on the day you come in to see your GP.<br>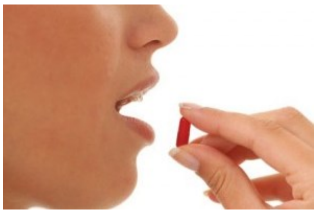	One tablet should be taken once every day, at night with cold water.<br>You need to take this medicine for 10 years.<br>You need to regularly visit a GP to receive a prescription.<br>You need to visit a pharmacy to fill a prescription.<br>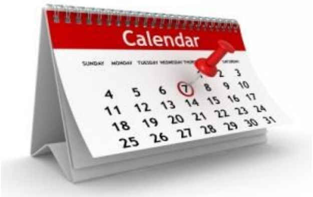<br>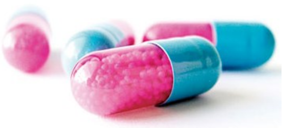

If you take Medicine A, you would live for **9 years** and then die.

If you take Medicine B, you would live for **10 years** and then die.

Would you prefer Medicine A or Medicine B, or are they the same?

Indicate your choice here. You can only choose one option:

◯ **Medicine A**

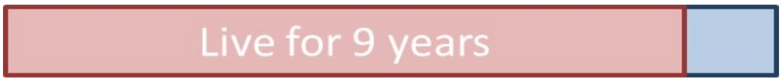

◯ **Medicine B**

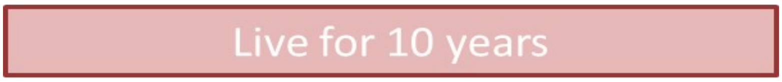

◯ **Medicine A and Medicine B are the same.**

For peer review only

Repeated section

Remember Medicine A and Medicine B are equally effective and have **some risk of a side effect**.

The only difference between Medicine A and Medicine B is how you take them.

**Medicine A**	**Medicine B**
You take a tablet only once in your life.<br>You will be given the tablet on the day you come in to see your GP.<br>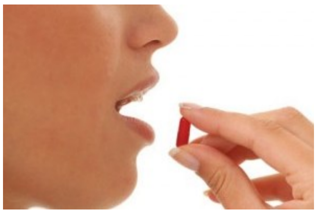	One tablet should be taken once every day, at night with cold water.<br>You need to take this medicine for 10 years.<br>You need to regularly visit a GP to receive a prescription.<br>You need to visit a pharmacy to fill a prescription.<br>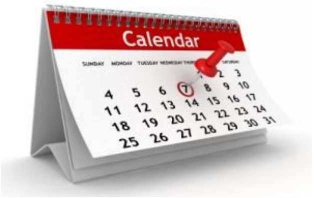<br>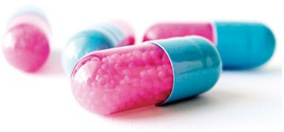

If you take Medicine A, you would live for **9 years and 1 month** and then die.

If you take Medicine B, you would live for **10 years** and then die.

Would you prefer Medicine A or Medicine B, or are they the same?

Indicate your choice here. You can only choose one option:

○ **Medicine A** Live for 9 years 1 month

○ **Medicine B** Live for 10 years

○ **Medicine A and Medicine B are the same.**

For peer review only

Repeated section

Remember Medicine A and Medicine B are equally effective and have **some risk of a side effect**.

The only difference between Medicine A and Medicine B is how you take them.

Medicine A	Medicine B
You take a tablet only once in your life.<br>You will be given the tablet on the day you come in to see your GP.<br>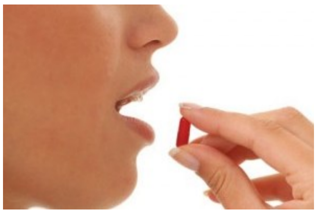	One tablet should be taken once every day, at night with cold water.<br>You need to take this medicine for 10 years.<br>You need to regularly visit a GP to receive a prescription.<br>You need to visit a pharmacy to fill a prescription.<br>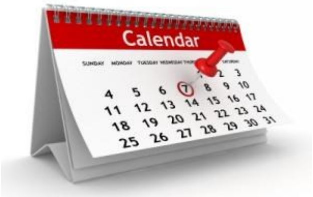<br><br>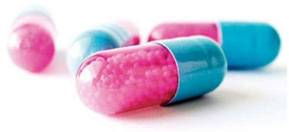

If you take Medicine A, you would live for **9.25 years (9 years and 3 months)** and then die.

If you take Medicine B, you would live for **10 years** and then die.

Would you prefer Medicine A or Medicine B, or are they the same?

Indicate your choice here. You can only choose one option:

◯ **Medicine A** Live for 9 years and 3 months

◯ **Medicine B** Live for 10 years

◯ **Medicine A and Medicine B are the same.**

For peer review only

Repeated section

Remember Medicine A and Medicine B are equally effective and have **some risk of a side effect**.

The only difference between Medicine A and Medicine B is how you take them.

Medicine A	Medicine B
You take a tablet only once in your life.<br>You will be given the tablet on the day you come in to see your GP.<br>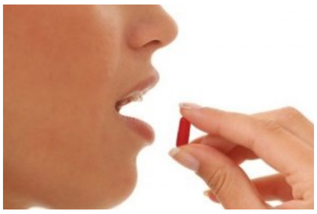	One tablet should be taken once every day, at night with cold water.<br>You need to take this medicine for 10 years.<br>You need to regularly visit a GP to receive a prescription.<br>You need to visit a pharmacy to fill a prescription.<br>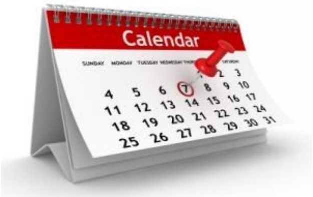<br><br>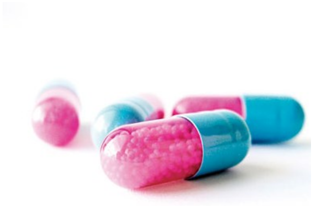

If you take Medicine A, you would live for **9 years and 4 months** and then die.

If you take Medicine B, you would live for **10 years** and then die.

Would you prefer Medicine A or Medicine B, or are they the same?

Indicate your choice here. You can only choose one option:

○ **Medicine A** Live for 9 years 4 months

○ **Medicine B** Live for 10 years

○ **Medicine A and Medicine B are the same.**

0% 100%

For peer review only

Repeated section

Remember Medicine A and Medicine B are equally effective and have **some risk of a side effect**.

The only difference between Medicine A and Medicine B is how you take them.

Medicine A	Medicine B
You take a tablet only once in your life.<br>You will be given the tablet on the day you come in to see your GP.<br>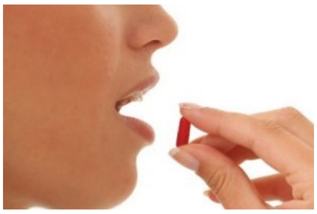	One tablet should be taken once every day, at night with cold water.<br>You need to take this medicine for 10 years.<br>You need to regularly visit a GP to receive a prescription.<br>You need to visit a pharmacy to fill a prescription.<br>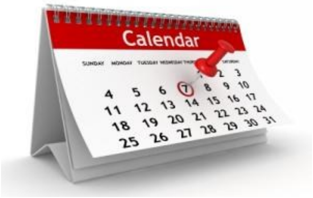<br>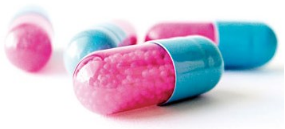

If you take Medicine A, you would live for **9 years and 5 months** and then die.

If you take Medicine B, you would live for **10 years** and then die.

Would you prefer Medicine A or Medicine B, or are they the same?

Indicate your choice here. You can only choose one option:

◯ **Medicine A** Live for 9 years 5 months

◯ **Medicine B** Live for 10 years

◯ **Medicine A and Medicine B are the same.**

0% 100%

For peer review only

Repeated section

Remember Medicine A and Medicine B are equally effective and have **some risk of a side effect**.

The only difference between Medicine A and Medicine B is how you take them.

**Medicine A**	**Medicine B**
You take a tablet only once in your life.<br>You will be given the tablet on the day you come in to see your GP.<br>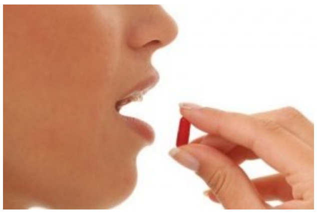	One tablet should be taken once every day, at night with cold water.<br>You need to take this medicine for 10 years.<br>You need to regularly visit a GP to receive a prescription.<br>You need to visit a pharmacy to fill a prescription.<br>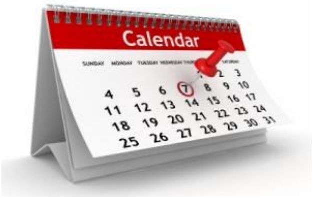<br><br>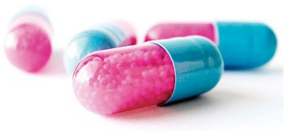

If you take Medicine A, you would live for **9.5 years** (9 years and 6 months) and then die.

If you take Medicine B, you would live for **10 years** and then die.

Would you prefer Medicine A or Medicine B, or are they the same?

Indicate your choice here. You can only choose one option:

◯ **Medicine A** Live for 9.5 years

◯ **Medicine B** Live for 10 years

◯ **Medicine A and Medicine B are the same.**

0% 100%

For peer review only

Repeated section

Remember Medicine A and Medicine B are equally effective and have **some risk of a side effect**.

The only difference between Medicine A and Medicine B is how you take them.

Medicine A	Medicine B
You take a tablet only once in your life.<br>You will be given the tablet on the day you come in to see your GP.<br>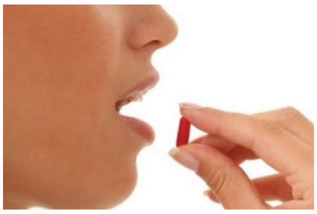	One tablet should be taken once every day, at night with cold water.<br>You need to take this medicine for 10 years.<br>You need to regularly visit a GP to receive a prescription.<br>You need to visit a pharmacy to fill a prescription.<br>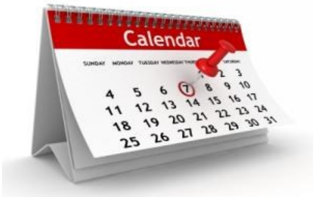<br><br>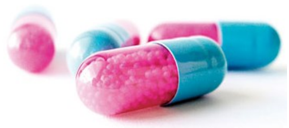

If you take Medicine A, you would live for **5 months less than 10 years** and then die.

If you take Medicine B, you would live for **10 years** and then die.

Would you prefer Medicine A or Medicine B, or are they the same?

Indicate your choice here. You can only choose one option:

◯ **Medicine A** Live for (10 years – 5 months)

◯ **Medicine B** Live for 10 years

◯ **Medicine A and Medicine B are the same.**

For peer review only

Repeated section

Remember Medicine A and Medicine B are equally effective and have **some risk of a side effect**.

The only difference between Medicine A and Medicine B is how you take them.

Medicine A	Medicine B
You take a tablet only once in your life.<br>You will be given the tablet on the day you come in to see your GP.<br>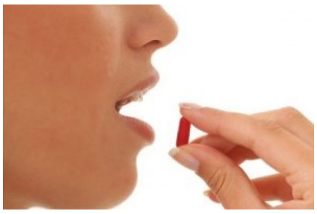	One tablet should be taken once every day, at night with cold water.<br>You need to take this medicine for 10 years.<br>You need to regularly visit a GP to receive a prescription.<br>You need to visit a pharmacy to fill a prescription.<br>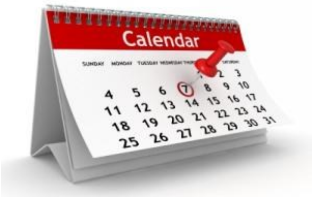<br>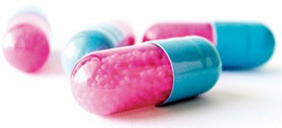

If you take Medicine A, you would live for **4 months less than 10 years** and then die.

If you take Medicine B, you would live for **10 years** and then die.

Would you prefer Medicine A or Medicine B, or are they the same?

Indicate your choice here. You can only choose one option:

○ **Medicine A** Live for (10 years – 4 months)

○ **Medicine B** Live for 10 years

○ **Medicine A and Medicine B are the same.**

0% 100%

For peer review only

Repeated section

Remember Medicine A and Medicine B are equally effective and have **some risk of a side effect**.

The only difference between Medicine A and Medicine B is how you take them.

Medicine A	Medicine B
You take a tablet only once in your life.<br>You will be given the tablet on the day you come in to see your GP.<br>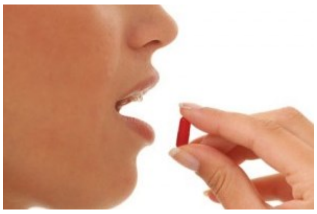	One tablet should be taken once every day, at night with cold water.<br>You need to take this medicine for 10 years.<br>You need to regularly visit a GP to receive a prescription.<br>You need to visit a pharmacy to fill a prescription.<br>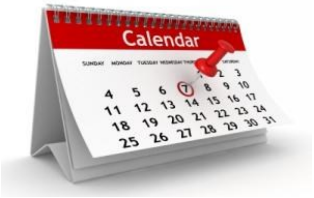<br><br>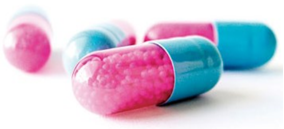

If you take Medicine A, you would live for **3 months less than 10 years** and then die.

If you take Medicine B, you would live for **10 years** and then die.

Would you prefer Medicine A or Medicine B, or are they the same?

Indicate your choice here. You can only choose one option:

○ **Medicine A** — Live for (10 years – 3 months)

○ **Medicine B** — Live for 10 years

○ **Medicine A and Medicine B are the same.**

For peer review only

Repeated section

Remember Medicine A and Medicine B are equally effective and have **some risk of a side effect**.

The only difference between Medicine A and Medicine B is how you take them.

**Medicine A**	**Medicine B**
You take a tablet only once in your life.<br>You will be given the tablet on the day you come in to see your GP.<br>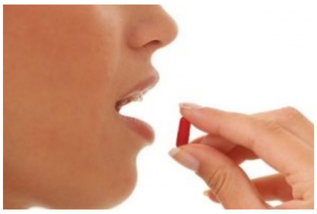	One tablet should be taken once every day, at night with cold water.<br>You need to take this medicine for 10 years.<br>You need to regularly visit a GP to receive a prescription.<br>You need to visit a pharmacy to fill a prescription.<br>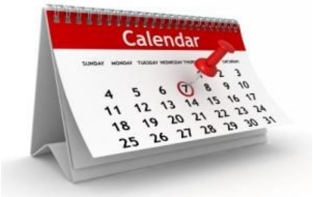<br>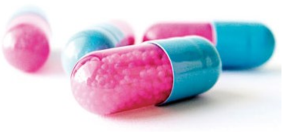

If you take Medicine A, you would live for **2 months less than 10 years** and then die.

If you take Medicine B, you would live for **10 years** and then die.

Would you prefer Medicine A or Medicine B, or are they the same?

Indicate your choice here. You can only choose one option:

○ **Medicine A** Live for (10 years – 2 months)

○ **Medicine B** Live for 10 years

○ **Medicine A and Medicine B are the same.**

For peer review only

Repeated section

Remember Medicine A and Medicine B are equally effective and have **some risk of a side effect**.

The only difference between Medicine A and Medicine B is how you take them.

Medicine A	Medicine B
You take a tablet only once in your life.<br>You will be given the tablet on the day you come in to see your GP.<br>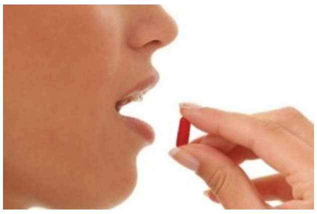	One tablet should be taken once every day, at night with cold water.<br>You need to take this medicine for 10 years.<br>You need to regularly visit a GP to receive a prescription.<br>You need to visit a pharmacy to fill a prescription.<br>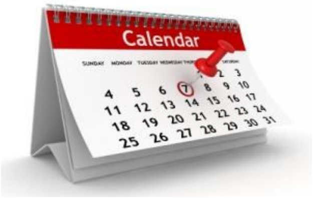<br>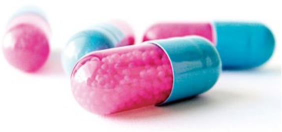

If you take Medicine A, you would live for **1 month less than 10 years** and then die.

If you take Medicine B, you would live for **10 years** and then die.

Would you prefer Medicine A or Medicine B, or are they the same?

Indicate your choice here. You can only choose one option:

○ **Medicine A** Live for (10 years – 1 month)

○ **Medicine B** Live for 10 years

○ **Medicine A and Medicine B are the same.**

For peer review only

Repeated section

Remember Medicine A and Medicine B are equally effective and have **some risk of a side effect**.

The only difference between Medicine A and Medicine B is how you take them.

Medicine A	Medicine B
You take a tablet only once in your life.<br>You will be given the tablet on the day you come in to see your GP.<br>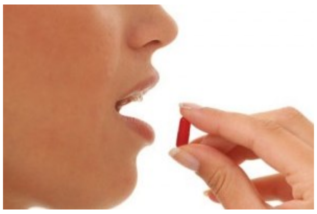	One tablet should be taken once every day, at night with cold water.<br>You need to take this medicine for 10 years.<br>You need to regularly visit a GP to receive a prescription.<br>You need to visit a pharmacy to fill a prescription.<br>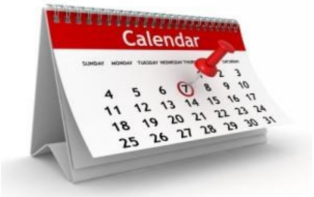<br>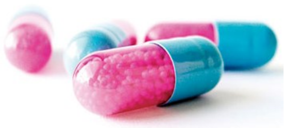

If you take Medicine A, you would live for **3 weeks less than 10 years** and then die.

If you take Medicine B, you would live for **10 years** and then die.

Would you prefer Medicine A or Medicine B, or are they the same?

Indicate your choice here. You can only choose one option:

○ **Medicine A** Live for (10 years – 3 weeks)

○ **Medicine B** Live for 10 years

○ **Medicine A and Medicine B are the same.**

0% 100%

For peer review only

Repeated section

Remember Medicine A and Medicine B are equally effective and have **some risk of a side effect**.

The only difference between Medicine A and Medicine B is how you take them.

**Medicine A**	**Medicine B**
You take a tablet only once in your life.<br>You will be given the tablet on the day you come in to see your GP.<br>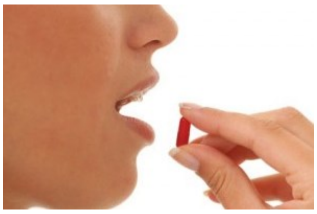	One tablet should be taken once every day, at night with cold water.<br>You need to take this medicine for 10 years.<br>You need to regularly visit a GP to receive a prescription.<br>You need to visit a pharmacy to fill a prescription.<br>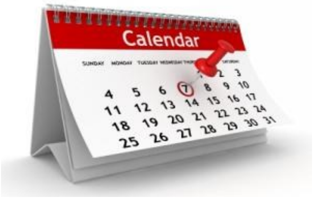<br>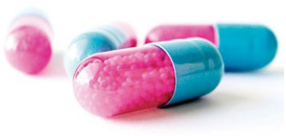

If you take Medicine A, you would live for **2 weeks less than 10 years** and then die.

If you take Medicine B, you would live for **10 years** and then die.

Would you prefer Medicine A or Medicine B, or are they the same?

Indicate your choice here. You can only choose one option:

◯ **Medicine A** Live for (10 years – 2 weeks)

◯ **Medicine B** Live for 10 years

◯ **Medicine A and Medicine B are the same.**

For peer review only

Repeated section

Remember Medicine A and Medicine B are equally effective and have **some risk of a side effect**.

The only difference between Medicine A and Medicine B is how you take them.

**Medicine A**	**Medicine B**
You take a tablet only once in your life.<br>You will be given the tablet on the day you come in to see your GP.<br>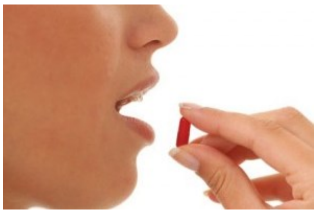	One tablet should be taken once every day, at night with cold water.<br>You need to take this medicine for 10 years.<br>You need to regularly visit a GP to receive a prescription.<br>You need to visit a pharmacy to fill a prescription.<br>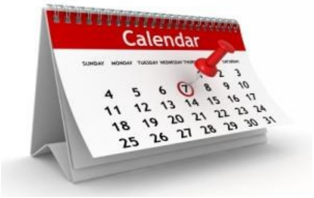<br>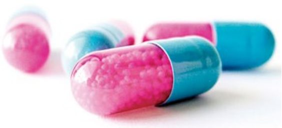

If you take Medicine A, you would live for **10 days less than 10 years** and then die.

If you take Medicine B, you would live for **10 years** and then die.

Would you prefer Medicine A or Medicine B, or are they the same?

Indicate your choice here. You can only choose one option:

○ **Medicine A** Live for (10 years – 10 days)

○ **Medicine B** Live for 10 years

○ **Medicine A and Medicine B are the same.**

0% 100%

For peer review only

Repeated section

Remember Medicine A and Medicine B are equally effective and have **some risk of a side effect**.

The only difference between Medicine A and Medicine B is how you take them.

Medicine A	Medicine B
You take a tablet only once in your life.<br>You will be given the tablet on the day you come in to see your GP.<br>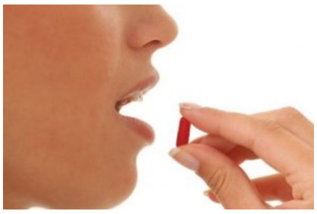	One tablet should be taken once every day, at night with cold water.<br>You need to take this medicine for 10 years.<br>You need to regularly visit a GP to receive a prescription.<br>You need to visit a pharmacy to fill a prescription.<br>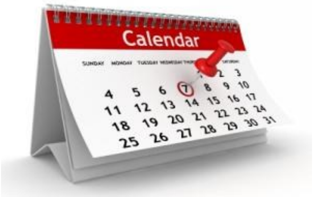<br>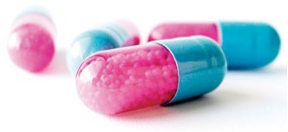

If you take Medicine A, you would live for **1 week less than 10 years** and then die.

If you take Medicine B, you would live for **10 years** and then die.

Would you prefer Medicine A or Medicine B, or are they the same?

Indicate your choice here. You can only choose one option:

- ○ **Medicine A** — Live for (10 years – 1 week)
- ○ **Medicine B** — Live for 10 years
- ○ **Medicine A and Medicine B are the same.**

For peer review only

Repeated section

Remember Medicine A and Medicine B are equally effective and have **some risk of a side effect**.

The only difference between Medicine A and Medicine B is how you take them.

Medicine A	Medicine B
You take a tablet only once in your life.<br>You will be given the tablet on the day you come in to see your GP.<br>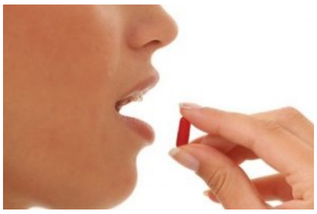	One tablet should be taken once every day, at night with cold water.<br>You need to take this medicine for 10 years.<br>You need to regularly visit a GP to receive a prescription.<br>You need to visit a pharmacy to fill a prescription.<br>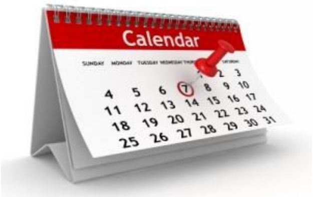<br><br>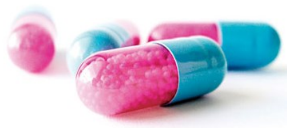

If you take Medicine A, you would live for **5 days less than 10 years** and then die.

If you take Medicine B, you would live for **10 years** and then die.

Would you prefer Medicine A or Medicine B, or are they the same?

Indicate your choice here. You can only choose one option:

○ **Medicine A** Live for (10 years – 5 days)

○ **Medicine B** Live for 10 years

○ **Medicine A and Medicine B are the same.**

0% 100%

For peer review only

Repeated section

Remember Medicine A and Medicine B are equally effective and have **some risk of a side effect**.

The only difference between Medicine A and Medicine B is how you take them.

Medicine A	Medicine B
You take a tablet only once in your life.<br>You will be given the tablet on the day you come in to see your GP.<br>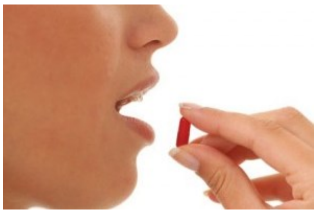	One tablet should be taken once every day, at night with cold water.<br>You need to take this medicine for 10 years.<br>You need to regularly visit a GP to receive a prescription.<br>You need to visit a pharmacy to fill a prescription.<br>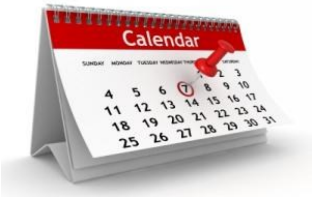<br>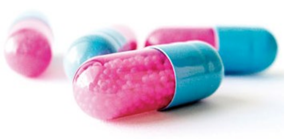

If you take Medicine A, you would live for **3 days less than 10 years** and then die.

If you take Medicine B, you would live for **10 years** and then die.

Would you prefer Medicine A or Medicine B, or are they the same?

Indicate your choice here. You can only choose one option:

○ **Medicine A** Live for (10 years – 3 days)

○ **Medicine B** Live for 10 years

○ **Medicine A and Medicine B are the same.**

0% 100%

For peer review only

For peer review only - http://bmjopen.bmj.com/site/about/guidelines.xhtml

Repeated section

Remember Medicine A and Medicine B are equally effective and have **some risk of a side effect**.

The only difference between Medicine A and Medicine B is how you take them.

Medicine A	Medicine B
You take a tablet only once in your life.<br>You will be given the tablet on the day you come in to see your GP.<br>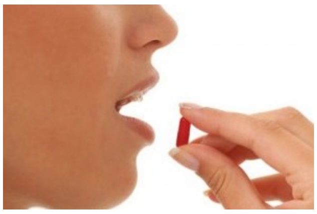	One tablet should be taken once every day, at night with cold water.<br>You need to take this medicine for 10 years.<br>You need to regularly visit a GP to receive a prescription.<br>You need to visit a pharmacy to fill a prescription.<br>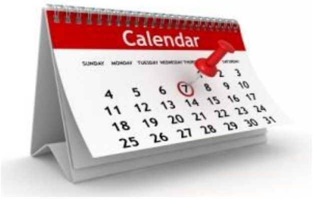 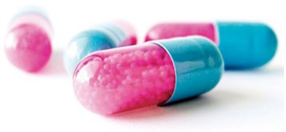

If you take Medicine A, you would live for **1 day less than 10 years** and then die.

If you take Medicine B, you would live for **10 years** and then die.

Would you prefer Medicine A or Medicine B, or are they the same?

Indicate your choice here. You can only choose one option:

- ◯ **Medicine A** — Live for (10 years – 1 day)
- ◯ **Medicine B** — Live for 10 years
- ◯ **Medicine A and Medicine B are the same.**

For peer review only

Question 2a.

You prefer living for 5 years on Medicine A, to living for 10 years on Medicine B.

This means **living fewer than 5 years on Medicine A** can be the same as living for 10 years on Medicine B.

How many years on Medicine A do you think would be the same as living for 10 years on Medicine B?

(Please give a number in years)

0% 100%

peer review only

Part 2

Question 3

Now, we will assume that there is some risk of a severe side effect from either medicine.

As before,

Medicine A is a pill that you only need to take once.

Medicine B is a pill that you need to take every day for ten years.

Medicine A and Medicine B are equally effective in reducing your risk of developing heart disease.

Medicine A and Medicine B will reduce your risk of having a heart attack or a stroke within the next 10 years from **10%** to **6%**.

In other words, in a crowd of 1,000 people in the UK, **100** are likely to have a heart attack or a stroke if they do not take the medicine. This is reduced to **60** people out of 1,000 who do take the medicine. This information is also illustrated in this picture.

Risk of heart disease

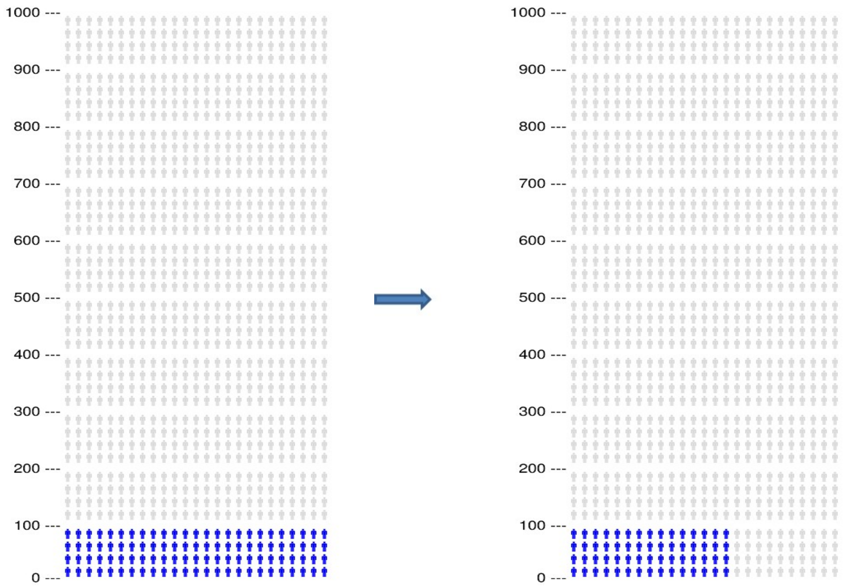

10%
No Treatment

6%
Taking the Medicine

(The figures shaded blue represent people who will have a heart attack or a stroke in the next 10 years)

In this scenario, there is some risk of a severe side effect from Medicine A or Medicine B.

Among every 1,000 people taking Medicine A or Medicine B, **3** people are likely to have a severe side effect such as severe destruction of skeletal muscle (rhabdomyolysis).

Risk of a severe side effect

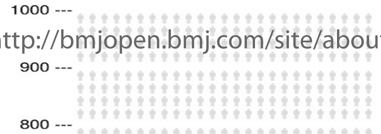

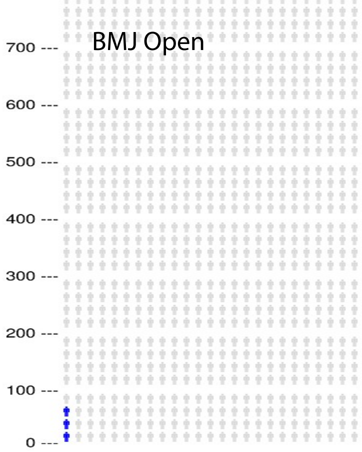

0.3% of people will have a severe side effect.

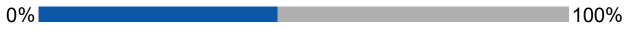

For peer review only

Repeated section

Remember Medicine A and Medicine B are equally effective and have **some risk of a side effect**.

The only difference between Medicine A and Medicine B is how you take them.

Medicine A	Medicine B
You take a tablet only once in your life.<br>You will be given the tablet on the day you come in to see your GP.<br>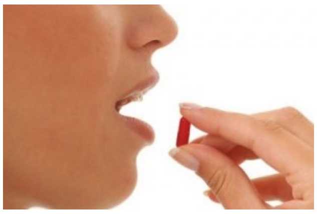	One tablet should be taken once every day, at night with cold water.<br>You need to take this medicine for 10 years.<br>You need to regularly visit a GP to receive a prescription.<br>You need to visit a pharmacy to fill a prescription.<br>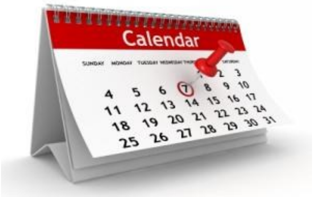<br>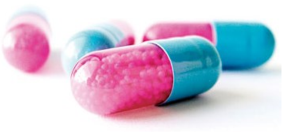

If you take Medicine A, you would live for **5 years** and then die.

If you take Medicine B, you would live for **10 years** and then die.

Would you prefer Medicine A or Medicine B, or are they the same?

Indicate your choice here. You can only choose one option:

◯ **Medicine A** Live for 5 years

◯ **Medicine B** Live for 10 years

◯ **Medicine A and Medicine B are the same.**

For peer review only

Repeated section

Remember Medicine A and Medicine B are equally effective and have **some risk of a side effect**.

The only difference between Medicine A and Medicine B is how you take them.

Medicine A	Medicine B
You take a tablet only once in your life.<br>You will be given the tablet on the day you come in to see your GP.<br>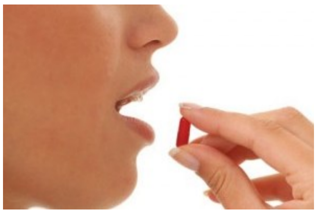	One tablet should be taken once every day, at night with cold water.<br>You need to take this medicine for 10 years.<br>You need to regularly visit a GP to receive a prescription.<br>You need to visit a pharmacy to fill a prescription.<br>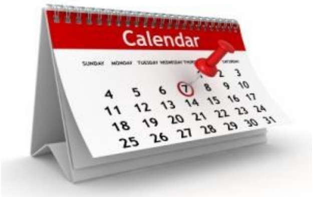<br>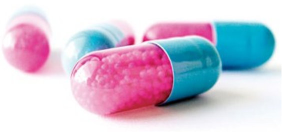

If you take Medicine A, you would live for **5.5 years** (5 years and 6 months) and then die.

If you take Medicine B, you would live for **10 years** and then die.

Would you prefer Medicine A or Medicine B, or are they the same?

Indicate your choice here. You can only choose one option:

○ **Medicine A** Live for 5.5 years

○ **Medicine B** Live for 10 years

○ **Medicine A and Medicine B are the same.**

0% 100%

For peer review only

Repeated section

Remember Medicine A and Medicine B are equally effective and have **some risk of a side effect**.

The only difference between Medicine A and Medicine B is how you take them.

**Medicine A**	**Medicine B**
You take a tablet only once in your life.<br>You will be given the tablet on the day you come in to see your GP.<br>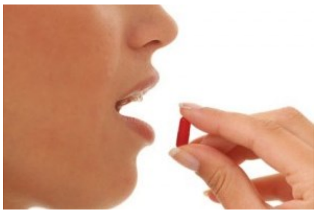	One tablet should be taken once every day, at night with cold water.<br>You need to take this medicine for 10 years.<br>You need to regularly visit a GP to receive a prescription.<br>You need to visit a pharmacy to fill a prescription.<br>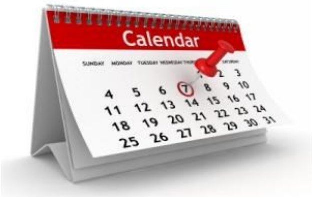<br>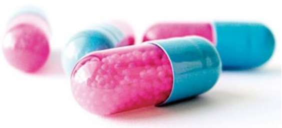

If you take Medicine A, you would live for **6 years** and then die.

If you take Medicine B, you would live for **10 years** and then die.

Would you prefer Medicine A or Medicine B, or are they the same?

Indicate your choice here. You can only choose one option:

◯ **Medicine A** Live for 6 years

◯ **Medicine B** Live for 10 years

◯ **Medicine A and Medicine B are the same.**

0% 100%

For peer review only

Repeated section

Remember Medicine A and Medicine B are equally effective and have **some risk of a side effect**.

The only difference between Medicine A and Medicine B is how you take them.

**Medicine A**	**Medicine B**
You take a tablet only once in your life.<br>You will be given the tablet on the day you come in to see your GP.<br>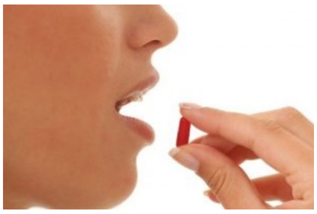	One tablet should be taken once every day, at night with cold water.<br>You need to take this medicine for 10 years.<br>You need to regularly visit a GP to receive a prescription.<br>You need to visit a pharmacy to fill a prescription.<br>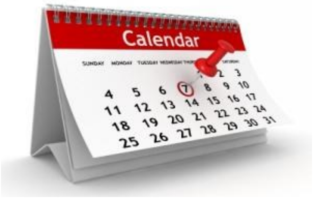<br>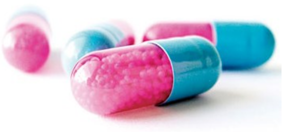

If you take Medicine A, you would live for **6.25 years (6 years and 3 months)** and then die.

If you take Medicine B, you would live for **10 years** and then die.

Would you prefer Medicine A or Medicine B, or are they the same?

Indicate your choice here. You can only choose one option:

◯ **Medicine A** Live for 6 years and 3 months

◯ **Medicine B** Live for 10 years

◯ **Medicine A and Medicine B are the same.**

0% 100%

For peer review only

Repeated section

Remember Medicine A and Medicine B are equally effective and have **some risk of a side effect**.

The only difference between Medicine A and Medicine B is how you take them.

**Medicine A**	**Medicine B**
You take a tablet only once in your life.<br>You will be given the tablet on the day you come in to see your GP.<br>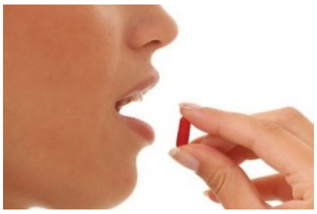	One tablet should be taken once every day, at night with cold water.<br>You need to take this medicine for 10 years.<br>You need to regularly visit a GP to receive a prescription.<br>You need to visit a pharmacy to fill a prescription.<br>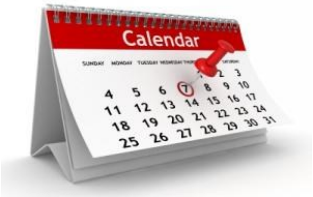<br>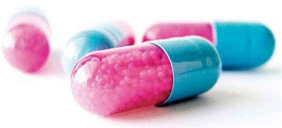

If you take Medicine A, you would live for **6.5 years** (6 years and 6 months) and then die.

If you take Medicine B, you would live for **10 years** and then die.

Would you prefer Medicine A or Medicine B, or are they the same?

Indicate your choice here. You can only choose one option:

◯ **Medicine A**

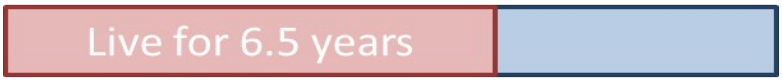

◯ **Medicine B**

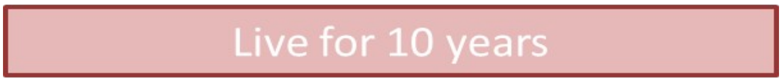

◯ **Medicine A and Medicine B are the same.**

0% 100%

For peer review only

Repeated section

Remember Medicine A and Medicine B are equally effective and have **some risk of a side effect**.

The only difference between Medicine A and Medicine B is how you take them.

Medicine A	Medicine B
You take a tablet only once in your life.<br>You will be given the tablet on the day you come in to see your GP.<br>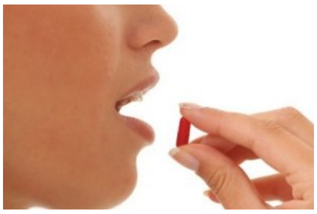	One tablet should be taken once every day, at night with cold water.<br>You need to take this medicine for 10 years.<br>You need to regularly visit a GP to receive a prescription.<br>You need to visit a pharmacy to fill a prescription.<br>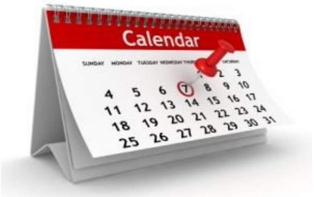 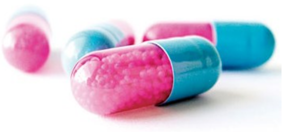

If you take Medicine A, you would live for **7 years** and then die.

If you take Medicine B, you would live for **10 years** and then die.

Would you prefer Medicine A or Medicine B, or are they the same?

Indicate your choice here. You can only choose one option:

◯ **Medicine A** Live for 7 years

◯ **Medicine B** Live for 10 years

◯ **Medicine A and Medicine B are the same.**

0% 100%

For peer review only

Repeated section

Remember Medicine A and Medicine B are equally effective and have **some risk of a side effect**.

The only difference between Medicine A and Medicine B is how you take them.

Medicine A	Medicine B
You take a tablet only once in your life.<br>You will be given the tablet on the day you come in to see your GP.<br>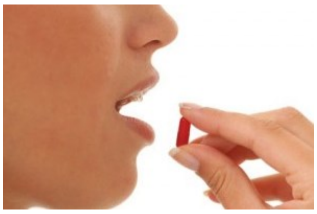	One tablet should be taken once every day, at night with cold water.<br>You need to take this medicine for 10 years.<br>You need to regularly visit a GP to receive a prescription.<br>You need to visit a pharmacy to fill a prescription.<br>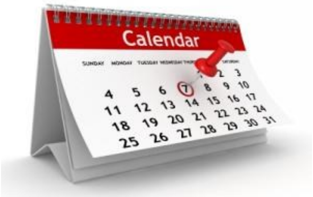<br><br>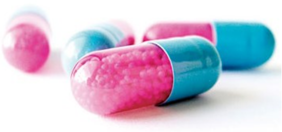

If you take Medicine A, you would live for **7.5 years** (7 years and 6 months) and then die.

If you take Medicine B, you would live for **10 years** and then die.

Would you prefer Medicine A or Medicine B, or are they the same?

Indicate your choice here. You can only choose one option:

◯ **Medicine A** Live for 7.5 years

◯ **Medicine B** Live for 10 years

◯ **Medicine A and Medicine B are the same.**

0% 100%

For peer review only

Repeated section

Remember Medicine A and Medicine B are equally effective and have **some risk of a side effect**.

The only difference between Medicine A and Medicine B is how you take them.

**Medicine A**	**Medicine B**
You take a tablet only once in your life.<br>You will be given the tablet on the day you come in to see your GP.<br>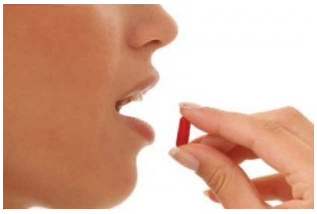	One tablet should be taken once every day, at night with cold water.<br>You need to take this medicine for 10 years.<br>You need to regularly visit a GP to receive a prescription.<br>You need to visit a pharmacy to fill a prescription.<br>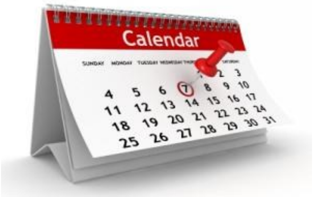 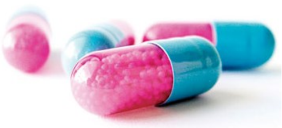

If you take Medicine A, you would live for **7.75 years (7 years and 9 months)** and then die.

If you take Medicine B, you would live for **10 years** and then die.

Would you prefer Medicine A or Medicine B, or are they the same?

Indicate your choice here. You can only choose one option:

◯ **Medicine A** Live for 7 years and 9 months

◯ **Medicine B** Live for 10 years

◯ **Medicine A and Medicine B are the same.**

0% 100%

For peer review only

Repeated section

Remember Medicine A and Medicine B are equally effective and have **some risk of a side effect**.

The only difference between Medicine A and Medicine B is how you take them.

Medicine A	Medicine B
You take a tablet only once in your life.<br>You will be given the tablet on the day you come in to see your GP.<br>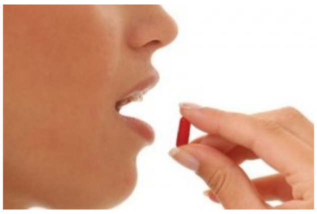	One tablet should be taken once every day, at night with cold water.<br>You need to take this medicine for 10 years.<br>You need to regularly visit a GP to receive a prescription.<br>You need to visit a pharmacy to fill a prescription.<br>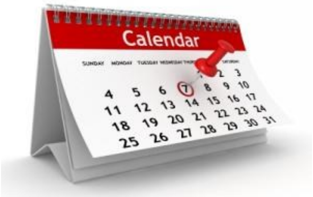<br><br>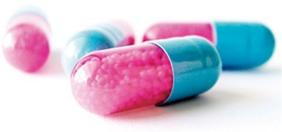

If you take Medicine A, you would live for **8 years** and then die.

If you take Medicine B, you would live for **10 years** and then die.

Would you prefer Medicine A or Medicine B, or are they the same?

Indicate your choice here. You can only choose one option:

◯ **Medicine A** Live for 8 years

◯ **Medicine B** Live for 10 years

◯ **Medicine A and Medicine B are the same.**

For peer review only

Repeated section

Remember Medicine A and Medicine B are equally effective and have **some risk of a side effect**.

The only difference between Medicine A and Medicine B is how you take them.

Medicine A	Medicine B
You take a tablet only once in your life.<br>You will be given the tablet on the day you come in to see your GP.<br>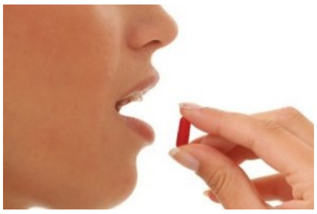	One tablet should be taken once every day, at night with cold water.<br>You need to take this medicine for 10 years.<br>You need to regularly visit a GP to receive a prescription.<br>You need to visit a pharmacy to fill a prescription.<br>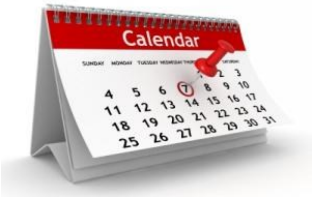<br>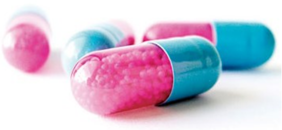

If you take Medicine A, you would live for **8.25 years (8 years and 3 months)** and then die.

If you take Medicine B, you would live for **10 years** and then die.

Would you prefer Medicine A or Medicine B, or are they the same?

Indicate your choice here. You can only choose one option:

○ **Medicine A** Live for 8 years and 3 months

○ **Medicine B** Live for 10 years

○ **Medicine A and Medicine B are the same.**

0% 100%

For peer review only

Repeated section

Remember Medicine A and Medicine B are equally effective and have **some risk of a side effect**.

The only difference between Medicine A and Medicine B is how you take them.

Medicine A	Medicine B
You take a tablet only once in your life.<br>You will be given the tablet on the day you come in to see your GP.<br>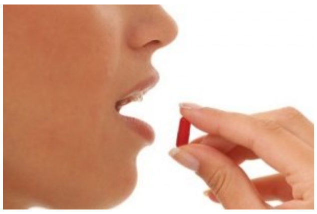	One tablet should be taken once every day, at night with cold water.<br>You need to take this medicine for 10 years.<br>You need to regularly visit a GP to receive a prescription.<br>You need to visit a pharmacy to fill a prescription.<br>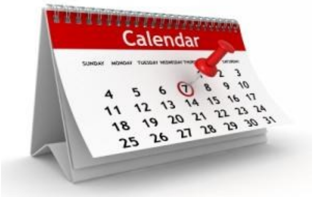<br>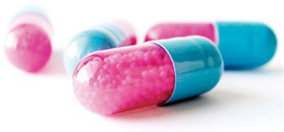

If you take Medicine A, you would live for **8.5 years** (8 years and 6 months) and then die.

If you take Medicine B, you would live for **10 years** and then die.

Would you prefer Medicine A or Medicine B, or are they the same?

Indicate your choice here. You can only choose one option:

○ **Medicine A** Live for 8.5 years

○ **Medicine B** Live for 10 years

○ **Medicine A and Medicine B are the same.**

0% 100%

For peer review only

Repeated section

Remember Medicine A and Medicine B are equally effective and have **some risk of a side effect**.

The only difference between Medicine A and Medicine B is how you take them.

**Medicine A**	**Medicine B**
You take a tablet only once in your life.<br>You will be given the tablet on the day you come in to see your GP.<br>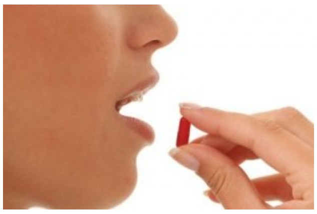	One tablet should be taken once every day, at night with cold water.<br>You need to take this medicine for 10 years.<br>You need to regularly visit a GP to receive a prescription.<br>You need to visit a pharmacy to fill a prescription.<br>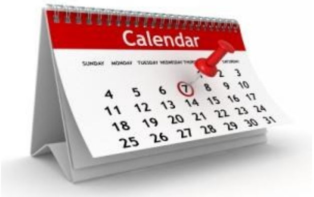<br>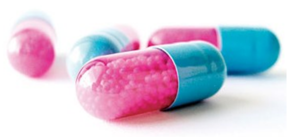

If you take Medicine A, you would live for **8.75 years (8 years and 9 months)** and then die.

If you take Medicine B, you would live for **10 years** and then die.

Would you prefer Medicine A or Medicine B, or are they the same?

Indicate your choice here. You can only choose one option:

◯ **Medicine A** Live for 8 years and 9 months

◯ **Medicine B** Live for 10 years

◯ **Medicine A and Medicine B are the same.**

0% 100%

For peer review only

Repeated section

Remember Medicine A and Medicine B are equally effective and have **some risk of a side effect**.

The only difference between Medicine A and Medicine B is how you take them.

**Medicine A**	**Medicine B**
You take a tablet only once in your life.<br>You will be given the tablet on the day you come in to see your GP.<br>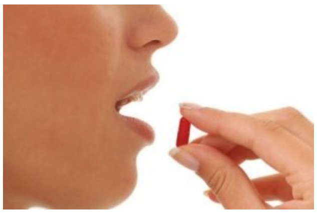	One tablet should be taken once every day, at night with cold water.<br>You need to take this medicine for 10 years.<br>You need to regularly visit a GP to receive a prescription.<br>You need to visit a pharmacy to fill a prescription.<br>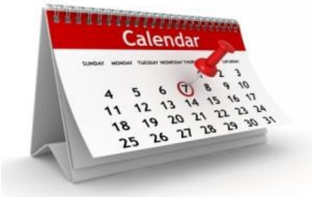<br>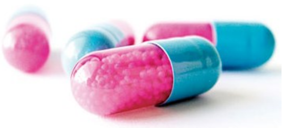

If you take Medicine A, you would live for **9 years** and then die.

If you take Medicine B, you would live for **10 years** and then die.

Would you prefer Medicine A or Medicine B, or are they the same?

Indicate your choice here. You can only choose one option:

○ **Medicine A** Live for 9 years

○ **Medicine B** Live for 10 years

○ **Medicine A and Medicine B are the same.**

For peer review only

Repeated section

Remember Medicine A and Medicine B are equally effective and have **some risk of a side effect**.

The only difference between Medicine A and Medicine B is how you take them.

Medicine A	Medicine B
You take a tablet only once in your life.<br>You will be given the tablet on the day you come in to see your GP.<br>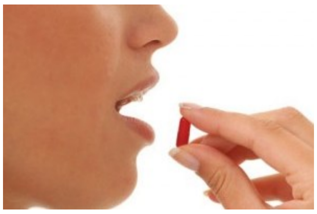	One tablet should be taken once every day, at night with cold water.<br>You need to take this medicine for 10 years.<br>You need to regularly visit a GP to receive a prescription.<br>You need to visit a pharmacy to fill a prescription.<br>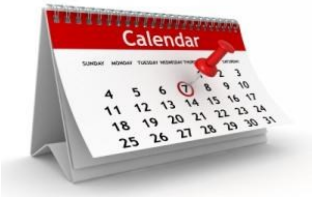<br>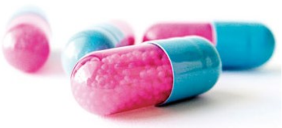

If you take Medicine A, you would live for **9 years and 1 month** and then die.

If you take Medicine B, you would live for **10 years** and then die.

Would you prefer Medicine A or Medicine B, or are they the same?

Indicate your choice here. You can only choose one option:

◯ **Medicine A** Live for 9 years 1 month

◯ **Medicine B** Live for 10 years

◯ **Medicine A and Medicine B are the same.**

For peer review only

Repeated section

Remember Medicine A and Medicine B are equally effective and have **some risk of a side effect**.

The only difference between Medicine A and Medicine B is how you take them.

Medicine A	Medicine B
You take a tablet only once in your life.<br>You will be given the tablet on the day you come in to see your GP.<br>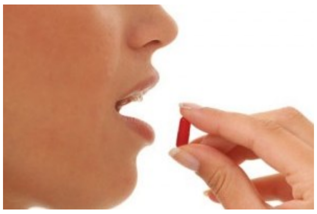	One tablet should be taken once every day, at night with cold water.<br>You need to take this medicine for 10 years.<br>You need to regularly visit a GP to receive a prescription.<br>You need to visit a pharmacy to fill a prescription.<br>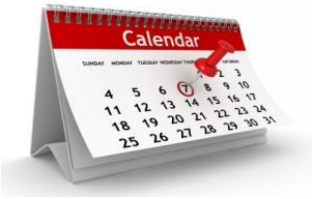<br><br>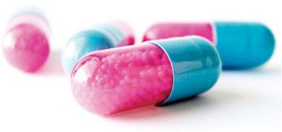

If you take Medicine A, you would live for **9.25 years (9 years and 3 months)** and then die.

If you take Medicine B, you would live for **10 years** and then die.

Would you prefer Medicine A or Medicine B, or are they the same?

Indicate your choice here. You can only choose one option:

○ **Medicine A** Live for 9 years and 3 months

○ **Medicine B** Live for 10 years

○ **Medicine A and Medicine B are the same.**

For peer review only

Repeated section

Remember Medicine A and Medicine B are equally effective and have **some risk of a side effect**.

The only difference between Medicine A and Medicine B is how you take them.

Medicine A	Medicine B
You take a tablet only once in your life.<br>You will be given the tablet on the day you come in to see your GP.<br>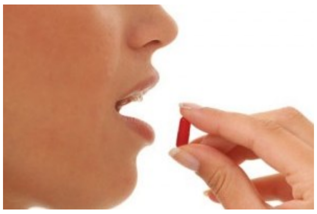	One tablet should be taken once every day, at night with cold water.<br>You need to take this medicine for 10 years.<br>You need to regularly visit a GP to receive a prescription.<br>You need to visit a pharmacy to fill a prescription.<br>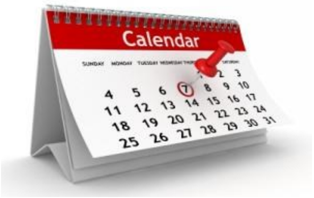<br>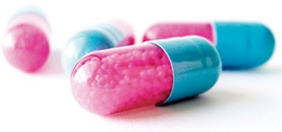

If you take Medicine A, you would live for **9 years and 4 months** and then die.

If you take Medicine B, you would live for **10 years** and then die.

Would you prefer Medicine A or Medicine B, or are they the same?

Indicate your choice here. You can only choose one option:

○ **Medicine A** Live for 9 years 4 months

○ **Medicine B** Live for 10 years

○ **Medicine A and Medicine B are the same.**

0% 100%

For peer review only

Repeated section

Remember Medicine A and Medicine B are equally effective and have **some risk of a side effect**.

The only difference between Medicine A and Medicine B is how you take them.

Medicine A	Medicine B
You take a tablet only once in your life.<br>You will be given the tablet on the day you come in to see your GP.<br>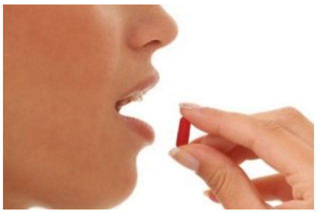	One tablet should be taken once every day, at night with cold water.<br>You need to take this medicine for 10 years.<br>You need to regularly visit a GP to receive a prescription.<br>You need to visit a pharmacy to fill a prescription.<br>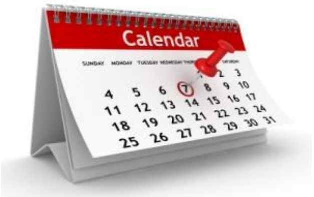<br>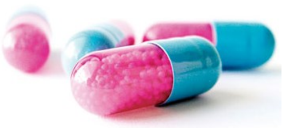

If you take Medicine A, you would live for **9 years and 5 months** and then die.

If you take Medicine B, you would live for **10 years** and then die.

Would you prefer Medicine A or Medicine B, or are they the same?

Indicate your choice here. You can only choose one option:

◯ **Medicine A** 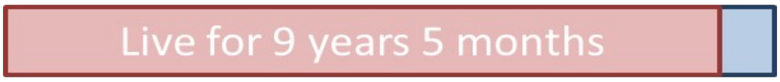

◯ **Medicine B** 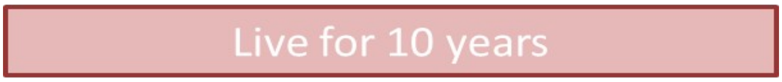

◯ **Medicine A and Medicine B are the same.**

For peer review only

Repeated section

Remember Medicine A and Medicine B are equally effective and have **some risk of a side effect**.

The only difference between Medicine A and Medicine B is how you take them.

**Medicine A**	**Medicine B**
You take a tablet only once in your life.<br>You will be given the tablet on the day you come in to see your GP.<br>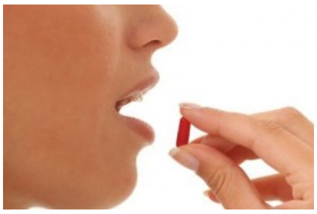	One tablet should be taken once every day, at night with cold water.<br>You need to take this medicine for 10 years.<br>You need to regularly visit a GP to receive a prescription.<br>You need to visit a pharmacy to fill a prescription.<br>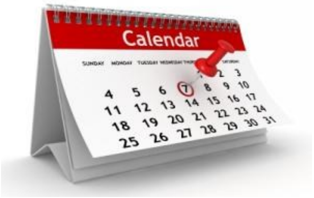<br>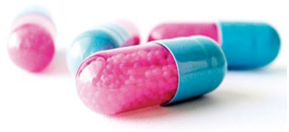

If you take Medicine A, you would live for **9.5 years** (9 years and 6 months) and then die.

If you take Medicine B, you would live for **10 years** and then die.

Would you prefer Medicine A or Medicine B, or are they the same?

Indicate your choice here. You can only choose one option:

○ **Medicine A** Live for 9.5 years

○ **Medicine B** Live for 10 years

○ **Medicine A and Medicine B are the same.**

0% 100%

For peer review only

Repeated section

Remember Medicine A and Medicine B are equally effective and have **some risk of a side effect**.

The only difference between Medicine A and Medicine B is how you take them.

Medicine A	Medicine B
You take a tablet only once in your life.<br>You will be given the tablet on the day you come in to see your GP.<br>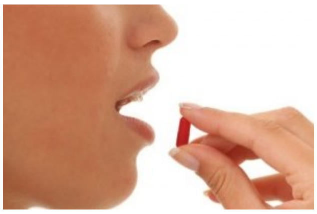	One tablet should be taken once every day, at night with cold water.<br>You need to take this medicine for 10 years.<br>You need to regularly visit a GP to receive a prescription.<br>You need to visit a pharmacy to fill a prescription.<br>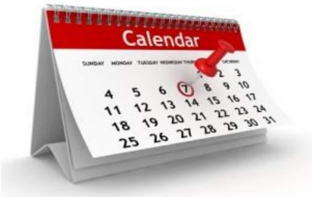<br>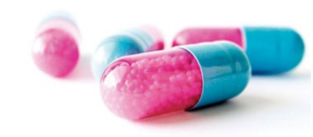

If you take Medicine A, you would live for **5 months less than 10 years** and then die.

If you take Medicine B, you would live for **10 years** and then die.

Would you prefer Medicine A or Medicine B, or are they the same?

Indicate your choice here. You can only choose one option:

○ **Medicine A** Live for (10 years – 5 months)

○ **Medicine B** Live for 10 years

○ **Medicine A and Medicine B are the same.**

0% 100%

For peer review only

Repeated section

Remember Medicine A and Medicine B are equally effective and have **some risk of a side effect**.

The only difference between Medicine A and Medicine B is how you take them.

**Medicine A**	**Medicine B**
You take a tablet only once in your life.<br>You will be given the tablet on the day you come in to see your GP.<br>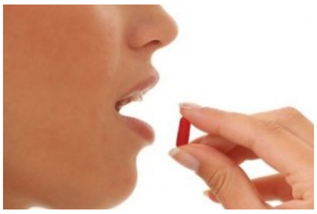	One tablet should be taken once every day, at night with cold water.<br>You need to take this medicine for 10 years.<br>You need to regularly visit a GP to receive a prescription.<br>You need to visit a pharmacy to fill a prescription.<br>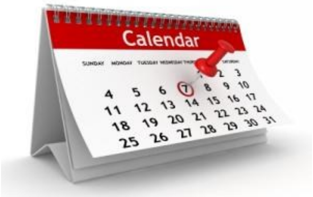<br>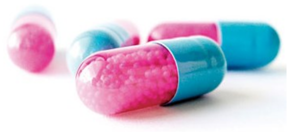

If you take Medicine A, you would live for **4 months less than 10 years** and then die.

If you take Medicine B, you would live for **10 years** and then die.

Would you prefer Medicine A or Medicine B, or are they the same?

Indicate your choice here. You can only choose one option:

◯ **Medicine A** Live for (10 years – 4 months)

◯ **Medicine B** Live for 10 years

◯ **Medicine A and Medicine B are the same.**

For peer review only

Repeated section

Remember Medicine A and Medicine B are equally effective and have **some risk of a side effect**.

The only difference between Medicine A and Medicine B is how you take them.

Medicine A	Medicine B
You take a tablet only once in your life.<br>You will be given the tablet on the day you come in to see your GP.<br>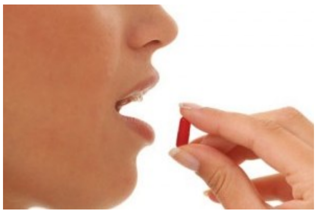	One tablet should be taken once every day, at night with cold water.<br>You need to take this medicine for 10 years.<br>You need to regularly visit a GP to receive a prescription.<br>You need to visit a pharmacy to fill a prescription.<br>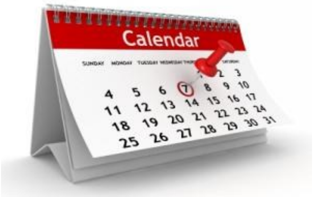<br><br>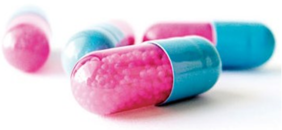

If you take Medicine A, you would live for **3 months less than 10 years** and then die.

If you take Medicine B, you would live for **10 years** and then die.

Would you prefer Medicine A or Medicine B, or are they the same?

Indicate your choice here. You can only choose one option:

◯ **Medicine A** Live for (10 years – 3 months)

◯ **Medicine B** Live for 10 years

◯ **Medicine A and Medicine B are the same.**

0% 100%

For peer review only

Repeated section

Remember Medicine A and Medicine B are equally effective and have **some risk of a side effect**.

The only difference between Medicine A and Medicine B is how you take them.

**Medicine A**	**Medicine B**
You take a tablet only once in your life.<br>You will be given the tablet on the day you come in to see your GP.<br>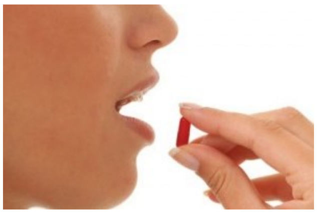	One tablet should be taken once every day, at night with cold water.<br>You need to take this medicine for 10 years.<br>You need to regularly visit a GP to receive a prescription.<br>You need to visit a pharmacy to fill a prescription.<br>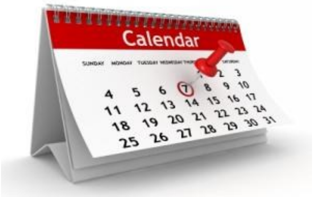<br>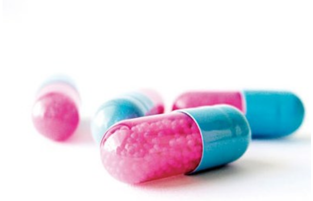

If you take Medicine A, you would live for **2 months less than 10 years** and then die.

If you take Medicine B, you would live for **10 years** and then die.

Would you prefer Medicine A or Medicine B, or are they the same?

Indicate your choice here. You can only choose one option:

◯ **Medicine A** Live for (10 years – 2 months)

◯ **Medicine B** Live for 10 years

◯ **Medicine A and Medicine B are the same.**

For peer review only

Repeated section

Remember Medicine A and Medicine B are equally effective and have **some risk of a side effect**.

The only difference between Medicine A and Medicine B is how you take them.

**Medicine A**	**Medicine B**
You take a tablet only once in your life.<br>You will be given the tablet on the day you come in to see your GP.<br>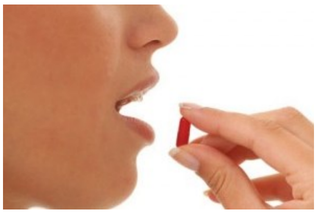	One tablet should be taken once every day, at night with cold water.<br>You need to take this medicine for 10 years.<br>You need to regularly visit a GP to receive a prescription.<br>You need to visit a pharmacy to fill a prescription.<br>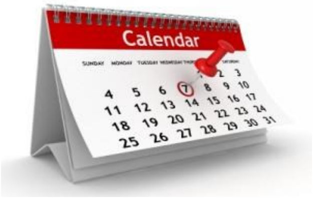 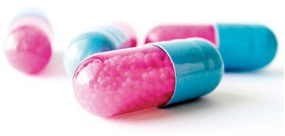

If you take Medicine A, you would live for **1 month less than 10 years** and then die.

If you take Medicine B, you would live for **10 years** and then die.

Would you prefer Medicine A or Medicine B, or are they the same?

Indicate your choice here. You can only choose one option:

◯ **Medicine A** Live for (10 years – 1 month)

◯ **Medicine B** Live for 10 years

◯ **Medicine A and Medicine B are the same.**

For peer review only

Repeated section

Remember Medicine A and Medicine B are equally effective and have **some risk of a side effect**.

The only difference between Medicine A and Medicine B is how you take them.

**Medicine A**	**Medicine B**
You take a tablet only once in your life.<br>You will be given the tablet on the day you come in to see your GP.<br>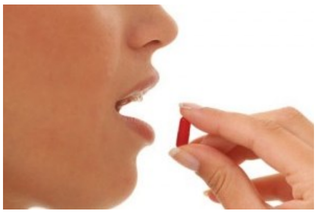	One tablet should be taken once every day, at night with cold water.<br>You need to take this medicine for 10 years.<br>You need to regularly visit a GP to receive a prescription.<br>You need to visit a pharmacy to fill a prescription.<br>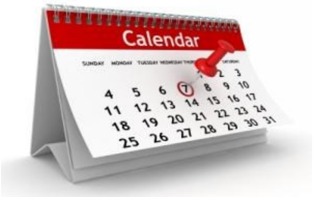<br>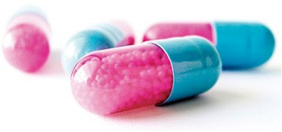

If you take Medicine A, you would live for **3 weeks less than 10 years** and then die.

If you take Medicine B, you would live for **10 years** and then die.

Would you prefer Medicine A or Medicine B, or are they the same?

Indicate your choice here. You can only choose one option:

◯ **Medicine A** Live for (10 years – 3 weeks)

◯ **Medicine B** Live for 10 years

◯ **Medicine A and Medicine B are the same.**

For peer review only

Repeated section

Remember Medicine A and Medicine B are equally effective and have **some risk of a side effect**.

The only difference between Medicine A and Medicine B is how you take them.

Medicine A	Medicine B
You take a tablet only once in your life.<br>You will be given the tablet on the day you come in to see your GP.<br>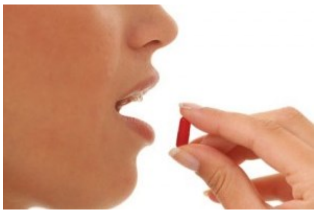	One tablet should be taken once every day, at night with cold water.<br>You need to take this medicine for 10 years.<br>You need to regularly visit a GP to receive a prescription.<br>You need to visit a pharmacy to fill a prescription.<br>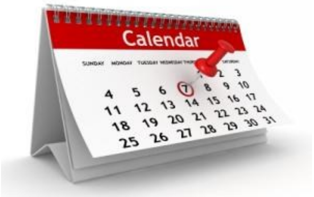<br>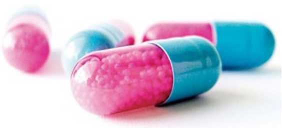

If you take Medicine A, you would live for **2 weeks less than 10 years** and then die.

If you take Medicine B, you would live for **10 years** and then die.

Would you prefer Medicine A or Medicine B, or are they the same?

Indicate your choice here. You can only choose one option:

○ **Medicine A** Live for (10 years – 2 weeks)

○ **Medicine B** Live for 10 years

○ **Medicine A and Medicine B are the same.**

For peer review only

Repeated section

Remember Medicine A and Medicine B are equally effective and have **some risk of a side effect**.

The only difference between Medicine A and Medicine B is how you take them.

Medicine A	Medicine B
You take a tablet only once in your life.<br>You will be given the tablet on the day you come in to see your GP.<br>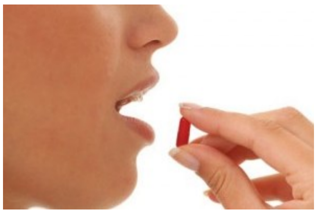	One tablet should be taken once every day, at night with cold water.<br>You need to take this medicine for 10 years.<br>You need to regularly visit a GP to receive a prescription.<br>You need to visit a pharmacy to fill a prescription.<br>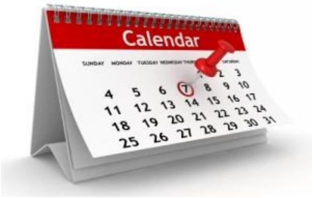<br>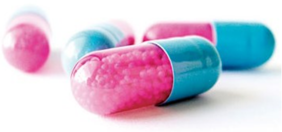

If you take Medicine A, you would live for **10 days less than 10 years** and then die.

If you take Medicine B, you would live for **10 years** and then die.

Would you prefer Medicine A or Medicine B, or are they the same?

Indicate your choice here. You can only choose one option:

○ **Medicine A** Live for (10 years – 10 days)

○ **Medicine B** Live for 10 years

○ **Medicine A and Medicine B are the same.**

For peer review only

Repeated section

Remember Medicine A and Medicine B are equally effective and have **some risk of a side effect**.

The only difference between Medicine A and Medicine B is how you take them.

Medicine A	Medicine B
You take a tablet only once in your life.<br>You will be given the tablet on the day you come in to see your GP.<br>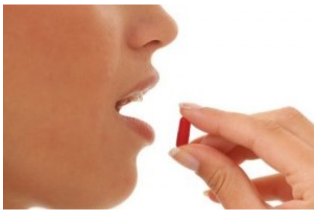	One tablet should be taken once every day, at night with cold water.<br>You need to take this medicine for 10 years.<br>You need to regularly visit a GP to receive a prescription.<br>You need to visit a pharmacy to fill a prescription.<br>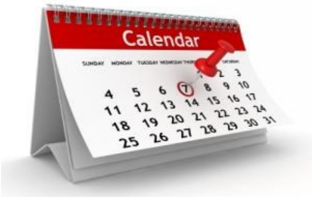<br>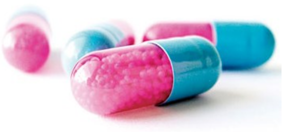

If you take Medicine A, you would live for **1 week less than 10 years** and then die.

If you take Medicine B, you would live for **10 years** and then die.

Would you prefer Medicine A or Medicine B, or are they the same?

Indicate your choice here. You can only choose one option:

○ **Medicine A** Live for (10 years – 1 week)

○ **Medicine B** Live for 10 years

○ **Medicine A and Medicine B are the same.**

For peer review only

Repeated section

Remember Medicine A and Medicine B are equally effective and have **some risk of a side effect**.

The only difference between Medicine A and Medicine B is how you take them.

Medicine A	Medicine B
You take a tablet only once in your life.<br>You will be given the tablet on the day you come in to see your GP.<br>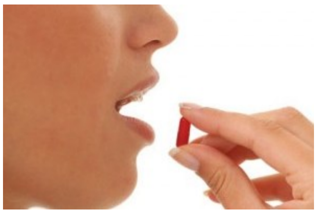	One tablet should be taken once every day, at night with cold water.<br>You need to take this medicine for 10 years.<br>You need to regularly visit a GP to receive a prescription.<br>You need to visit a pharmacy to fill a prescription.<br>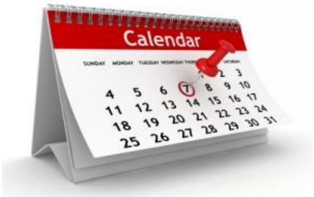<br>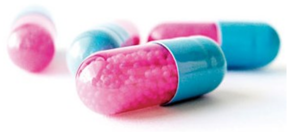

If you take Medicine A, you would live for **5 days less than 10 years** and then die.

If you take Medicine B, you would live for **10 years** and then die.

Would you prefer Medicine A or Medicine B, or are they the same?

Indicate your choice here. You can only choose one option:

◯ **Medicine A** Live for (10 years – 5 days)

◯ **Medicine B** Live for 10 years

◯ **Medicine A and Medicine B are the same.**

For peer review only

Repeated section

Remember Medicine A and Medicine B are equally effective and have **some risk of a side effect**.

The only difference between Medicine A and Medicine B is how you take them.

**Medicine A**	**Medicine B**
You take a tablet only once in your life.<br>You will be given the tablet on the day you come in to see your GP.<br>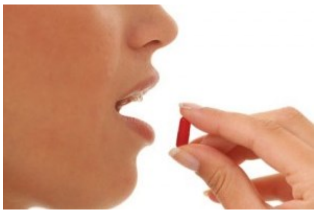	One tablet should be taken once every day, at night with cold water.<br>You need to take this medicine for 10 years.<br>You need to regularly visit a GP to receive a prescription.<br>You need to visit a pharmacy to fill a prescription.<br>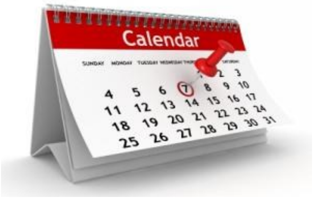<br>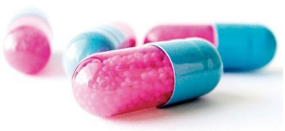

If you take Medicine A, you would live for **3 days less than 10 years** and then die.

If you take Medicine B, you would live for **10 years** and then die.

Would you prefer Medicine A or Medicine B, or are they the same?

Indicate your choice here. You can only choose one option:

○ **Medicine A** Live for (10 years – 3 days)

○ **Medicine B** Live for 10 years

○ **Medicine A and Medicine B are the same.**

For peer review only

Repeated section

Remember Medicine A and Medicine B are equally effective and have **some risk of a side effect**.

The only difference between Medicine A and Medicine B is how you take them.

Medicine A	Medicine B
You take a tablet only once in your life.<br>You will be given the tablet on the day you come in to see your GP.<br>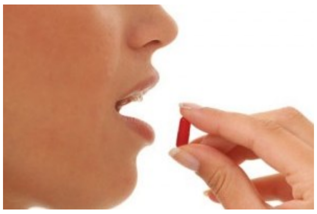	One tablet should be taken once every day, at night with cold water.<br>You need to take this medicine for 10 years.<br>You need to regularly visit a GP to receive a prescription.<br>You need to visit a pharmacy to fill a prescription.<br>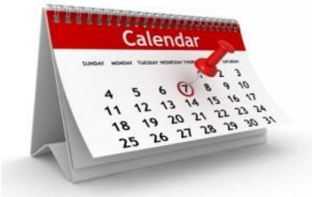<br>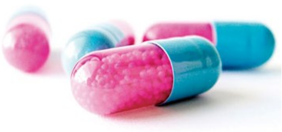

If you take Medicine A, you would live for **1 day less than 10 years** and then die.

If you take Medicine B, you would live for **10 years** and then die.

Would you prefer Medicine A or Medicine B, or are they the same?

Indicate your choice here. You can only choose one option:

○ **Medicine A** Live for (10 years – 1 day)

○ **Medicine B** Live for 10 years

○ **Medicine A and Medicine B are the same.**

For peer review only

Question 3a.

You prefer living for 5 years on Medicine A, to living for 10 years on Medicine B.

This means **living fewer than 5 years on Medicine A** can be the same as living for 10 years on Medicine B.

How many years on Medicine A do you think would be the same as living for 10 years on Medicine B?

(Please give a number in years)

0% 100%

peer review only

Part 2

Question 4

Now, Medicine A and Medicine B will give you less benefit than before because you belong to a group of people who may not respond to the treatment as well as other groups of people.

As before,

Medicine A is a pill that you only need to take once.

Medicine B is a pill that you need to take every day for ten years.

Medicine A and Medicine B are equally effective in reducing your risk of developing heart disease.

Medicine A and Medicine B will reduce your risk of having a heart attack or a stroke within the next 10 years from **10%** to **9%**.

In other words, in a crowd of **1,000** people in the UK, **100** are likely to have a heart attack or a stroke if they do not take the medicine. This is reduced to **90** people out of 1,000 who do take the medicine. This information is also illustrated in this picture.

Risk of heart disease

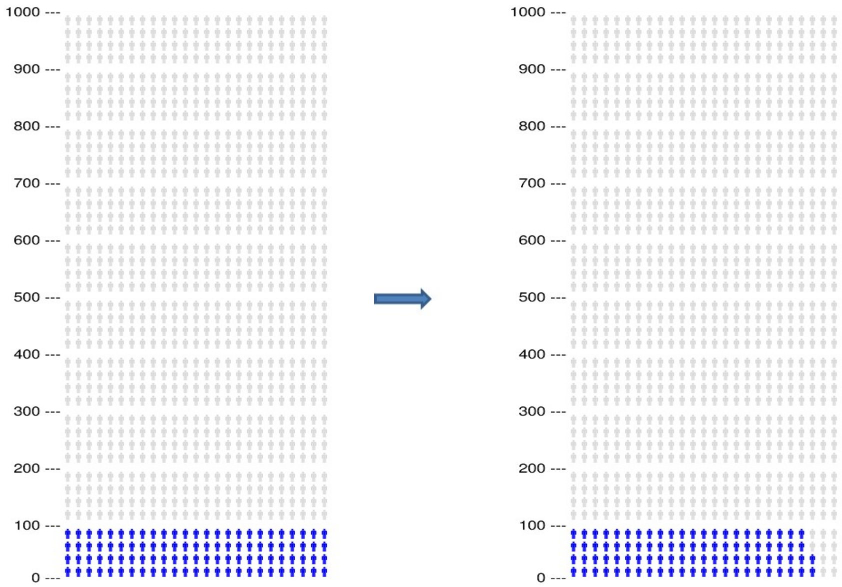

10%
No Treatment

9%
Taking the Medicine

(The figures shaded blue represent people who will have a heart attack or a stroke in the next 10 years)

In this scenario, you will not have any side effects from Medicine A or Medicine B.

Risk of a side effect

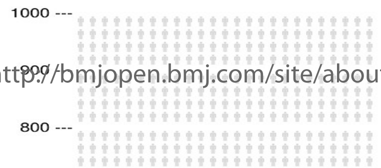

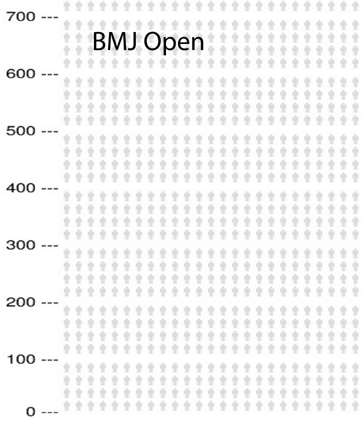

0%
No one will have a side effect.

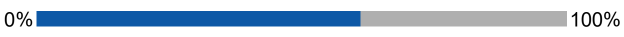

For peer review only

Repeated section

Remember Medicine A and Medicine B are equally effective and have no risks of a side effect.

The only difference between Medicine A and Medicine B is how you take them.

Medicine A	Medicine B
You take a tablet only once in your life.<br>You will be given the tablet on the day you come in to see your GP.<br>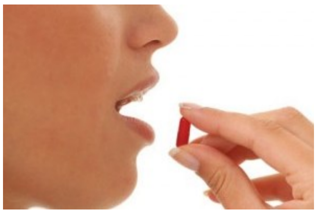	One tablet should be taken once every day, at night with cold water.<br>You need to take this medicine for 10 years.<br>You need to regularly visit a GP to receive a prescription.<br>You need to visit a pharmacy to fill a prescription.<br>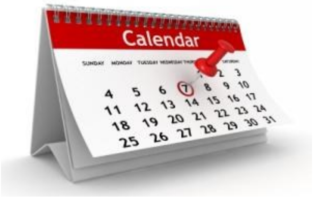<br>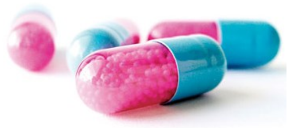

If you take Medicine A, you would live for **5 years** and then die.

If you take Medicine B, you would live for **10 years** and then die.

Would you prefer Medicine A or Medicine B, or are they the same?

Indicate your choice here. You can only choose one option:

○ **Medicine A** Live for 5 years

○ **Medicine B** Live for 10 years

○ **Medicine A and Medicine B are the same.**

For peer review only

Repeated section

Remember Medicine A and Medicine B are equally effective and have no risks of a side effect.

The only difference between Medicine A and Medicine B is how you take them.

**Medicine A**	**Medicine B**
You take a tablet only once in your life.<br>You will be given the tablet on the day you come in to see your GP.<br>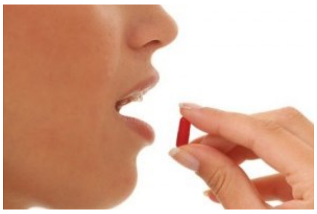	One tablet should be taken once every day, at night with cold water.<br>You need to take this medicine for 10 years.<br>You need to regularly visit a GP to receive a prescription.<br>You need to visit a pharmacy to fill a prescription.<br>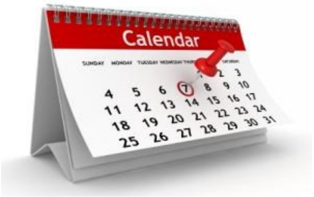<br>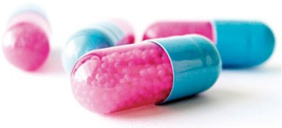

If you take Medicine A, you would live for **5.5 years (5 years and 6 months)** and then die.

If you take Medicine B, you would live for **10 years** and then die.

Would you prefer Medicine A or Medicine B, or are they the same?

Indicate your choice here. You can only choose one option:

○ **Medicine A** 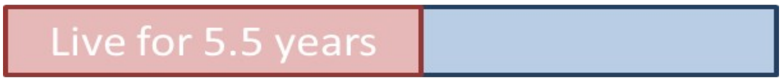

○ **Medicine B** 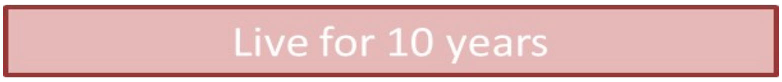

○ **Medicine A and Medicine B are the same.**

For peer review only

Repeated section

Remember Medicine A and Medicine B are equally effective and have no risks of a side effect.

The only difference between Medicine A and Medicine B is how you take them.

Medicine A	Medicine B
You take a tablet only once in your life.<br>You will be given the tablet on the day you come in to see your GP.<br>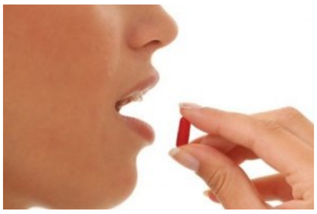	One tablet should be taken once every day, at night with cold water.<br>You need to take this medicine for 10 years.<br>You need to regularly visit a GP to receive a prescription.<br>You need to visit a pharmacy to fill a prescription.<br>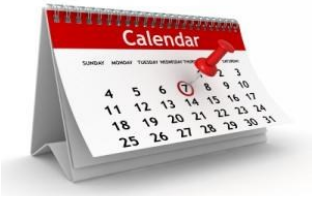<br>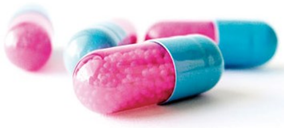

If you take Medicine A, you would live for **6 years** and then die.

If you take Medicine B, you would live for **10 years** and then die.

Would you prefer Medicine A or Medicine B, or are they the same?

Indicate your choice here. You can only choose one option:

◯ **Medicine A** Live for 6 years

◯ **Medicine B** Live for 10 years

◯ **Medicine A and Medicine B are the same.**

For peer review only

Repeated section

Remember Medicine A and Medicine B are equally effective and have no risks of a side effect.

The only difference between Medicine A and Medicine B is how you take them.

Medicine A	Medicine B
You take a tablet only once in your life.<br>You will be given the tablet on the day you come in to see your GP.<br>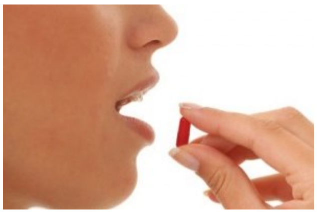	One tablet should be taken once every day, at night with cold water.<br>You need to take this medicine for 10 years.<br>You need to regularly visit a GP to receive a prescription.<br>You need to visit a pharmacy to fill a prescription.<br>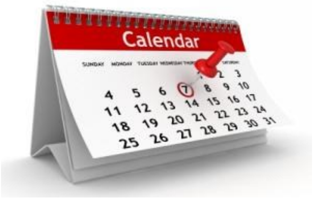<br>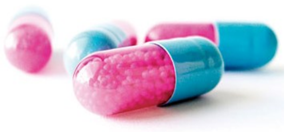

If you take Medicine A, you would live for **6.25 years (6 years and 3 months)** and then die.

If you take Medicine B, you would live for **10 years** and then die.

Would you prefer Medicine A or Medicine B, or are they the same?

Indicate your choice here. You can only choose one option:

◯ **Medicine A** 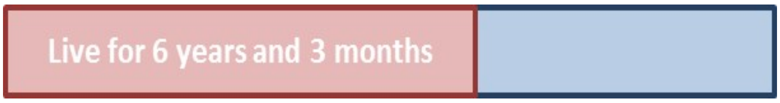

◯ **Medicine B** 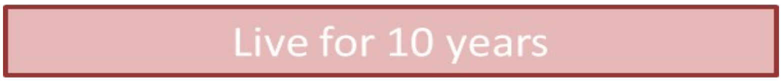

◯ **Medicine A and Medicine B are the same.**

For peer review only

Repeated section

Remember Medicine A and Medicine B are equally effective and have no risks of a side effect.

The only difference between Medicine A and Medicine B is how you take them.

Medicine A	Medicine B
You take a tablet only once in your life.<br>You will be given the tablet on the day you come in to see your GP.<br>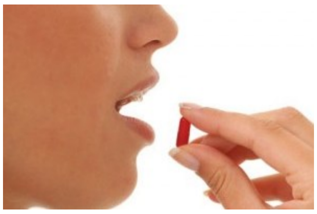	One tablet should be taken once every day, at night with cold water.<br>You need to take this medicine for 10 years.<br>You need to regularly visit a GP to receive a prescription.<br>You need to visit a pharmacy to fill a prescription.<br>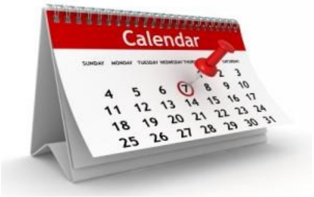 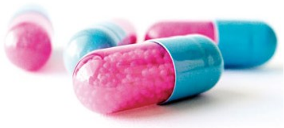

If you take Medicine A, you would live for **6.5 years** (6 years and 6 months) and then die.

If you take Medicine B, you would live for **10 years** and then die.

Would you prefer Medicine A or Medicine B, or are they the same?

Indicate your choice here. You can only choose one option:

◯ **Medicine A** Live for 6.5 years

◯ **Medicine B** Live for 10 years

◯ **Medicine A and Medicine B are the same.**

For peer review only

Repeated section

Remember Medicine A and Medicine B are equally effective and have no risks of a side effect.

The only difference between Medicine A and Medicine B is how you take them.

Medicine A	Medicine B
You take a tablet only once in your life.<br>You will be given the tablet on the day you come in to see your GP.<br>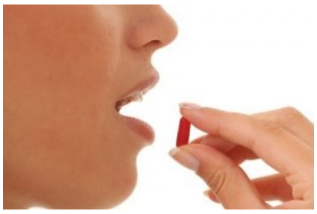	One tablet should be taken once every day, at night with cold water.<br>You need to take this medicine for 10 years.<br>You need to regularly visit a GP to receive a prescription.<br>You need to visit a pharmacy to fill a prescription.<br>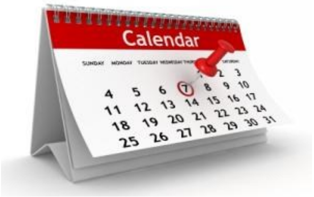<br>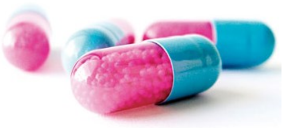

If you take Medicine A, you would live for **7 years** and then die.

If you take Medicine B, you would live for **10 years** and then die.

Would you prefer Medicine A or Medicine B, or are they the same?

Indicate your choice here. You can only choose one option:

○ **Medicine A** Live for 7 years

○ **Medicine B** Live for 10 years

○ **Medicine A and Medicine B are the same.**

0% 100%

For peer review only

Repeated section

Remember Medicine A and Medicine B are equally effective and have no risks of a side effect.

The only difference between Medicine A and Medicine B is how you take them.

Medicine A	Medicine B
You take a tablet only once in your life.<br>You will be given the tablet on the day you come in to see your GP.<br>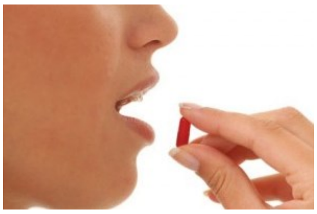	One tablet should be taken once every day, at night with cold water.<br>You need to take this medicine for 10 years.<br>You need to regularly visit a GP to receive a prescription.<br>You need to visit a pharmacy to fill a prescription.<br>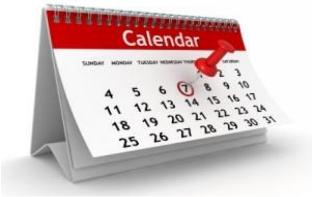<br>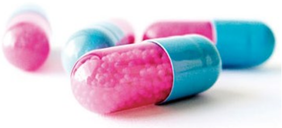

If you take Medicine A, you would live for **7.5 years (7 years and 6 months)** and then die.

If you take Medicine B, you would live for **10 years** and then die.

Would you prefer Medicine A or Medicine B, or are they the same?

Indicate your choice here. You can only choose one option:

◯ **Medicine A** Live for 7.5 years

◯ **Medicine B** Live for 10 years

◯ **Medicine A and Medicine B are the same.**

For peer review only

Repeated section

Remember Medicine A and Medicine B are equally effective and have no risks of a side effect.

The only difference between Medicine A and Medicine B is how you take them.

**Medicine A**	**Medicine B**
You take a tablet only once in your life.<br>You will be given the tablet on the day you come in to see your GP.<br>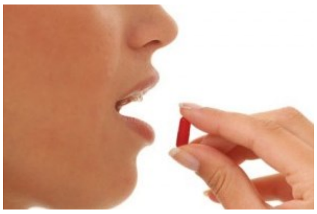	One tablet should be taken once every day, at night with cold water.<br>You need to take this medicine for 10 years.<br>You need to regularly visit a GP to receive a prescription.<br>You need to visit a pharmacy to fill a prescription.<br>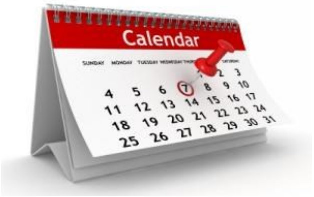<br>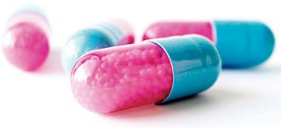

If you take Medicine A, you would live for **7.75 years (7 years and 9 months)** and then die.

If you take Medicine B, you would live for **10 years** and then die.

Would you prefer Medicine A or Medicine B, or are they the same?

Indicate your choice here. You can only choose one option:

◯ **Medicine A** 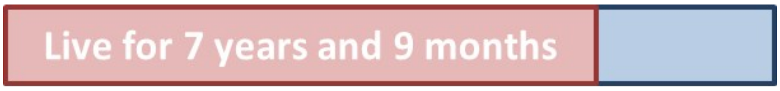

◯ **Medicine B** 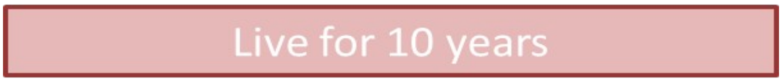

◯ **Medicine A and Medicine B are the same.**

0% 100%

For peer review only

Repeated section

Remember Medicine A and Medicine B are equally effective and have no risks of a side effect.

The only difference between Medicine A and Medicine B is how you take them.

Medicine A	Medicine B
You take a tablet only once in your life.<br>You will be given the tablet on the day you come in to see your GP.<br>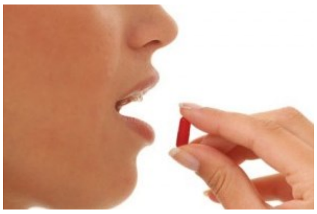	One tablet should be taken once every day, at night with cold water.<br>You need to take this medicine for 10 years.<br>You need to regularly visit a GP to receive a prescription.<br>You need to visit a pharmacy to fill a prescription.<br>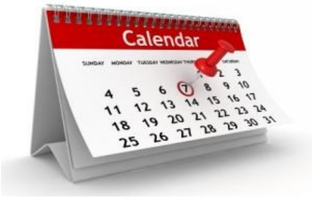<br>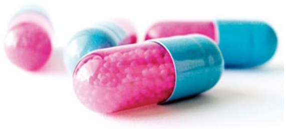

If you take Medicine A, you would live for **8 years** and then die.

If you take Medicine B, you would live for **10 years** and then die.

Would you prefer Medicine A or Medicine B, or are they the same?

Indicate your choice here. You can only choose one option:

○ **Medicine A** Live for 8 years

○ **Medicine B** Live for 10 years

○ **Medicine A and Medicine B are the same.**

For peer review only

Repeated section

Remember Medicine A and Medicine B are equally effective and have no risks of a side effect.

The only difference between Medicine A and Medicine B is how you take them.

Medicine A	Medicine B
You take a tablet only once in your life.<br>You will be given the tablet on the day you come in to see your GP.<br>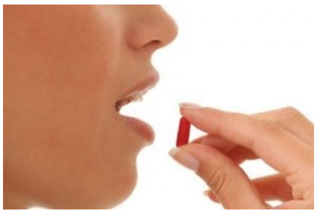	One tablet should be taken once every day, at night with cold water.<br>You need to take this medicine for 10 years.<br>You need to regularly visit a GP to receive a prescription.<br>You need to visit a pharmacy to fill a prescription.<br>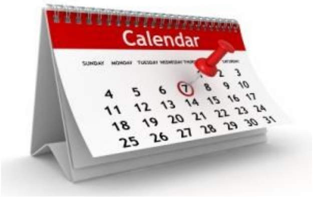<br><br>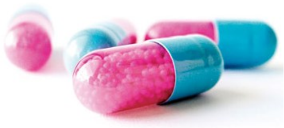

If you take Medicine A, you would live for **8.25 years (8 years and 3 months)** and then die.

If you take Medicine B, you would live for **10 years** and then die.

Would you prefer Medicine A or Medicine B, or are they the same?

Indicate your choice here. You can only choose one option:

○ **Medicine A** Live for 8 years and 3 months

○ **Medicine B** Live for 10 years

○ **Medicine A and Medicine B are the same.**

0% 100%

For peer review only

Repeated section

Remember Medicine A and Medicine B are equally effective and have no risks of a side effect.

The only difference between Medicine A and Medicine B is how you take them.

Medicine A	Medicine B
You take a tablet only once in your life.<br>You will be given the tablet on the day you come in to see your GP.<br>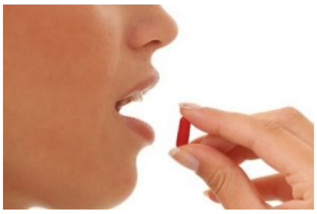	One tablet should be taken once every day, at night with cold water.<br>You need to take this medicine for 10 years.<br>You need to regularly visit a GP to receive a prescription.<br>You need to visit a pharmacy to fill a prescription.<br>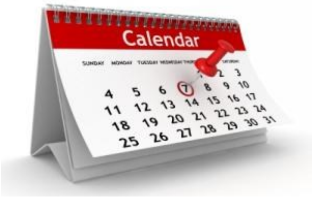<br>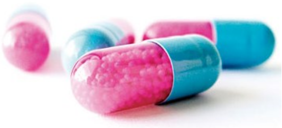

If you take Medicine A, you would live for **8.5 years** (8 years and 6 months) and then die.

If you take Medicine B, you would live for **10 years** and then die.

Would you prefer Medicine A or Medicine B, or are they the same?

Indicate your choice here. You can only choose one option:

◯ **Medicine A** Live for 8.5 years

◯ **Medicine B** Live for 10 years

◯ **Medicine A and Medicine B are the same.**

For peer review only

Repeated section

Remember Medicine A and Medicine B are equally effective and have no risks of a side effect.

The only difference between Medicine A and Medicine B is how you take them.

Medicine A	Medicine B
You take a tablet only once in your life.<br>You will be given the tablet on the day you come in to see your GP.<br>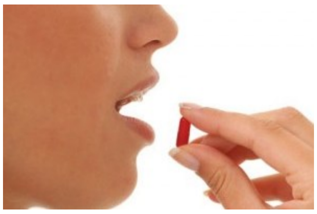	One tablet should be taken once every day, at night with cold water.<br>You need to take this medicine for 10 years.<br>You need to regularly visit a GP to receive a prescription.<br>You need to visit a pharmacy to fill a prescription.<br>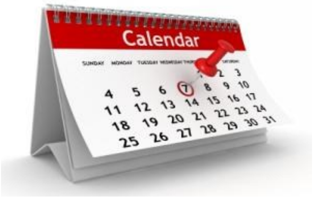 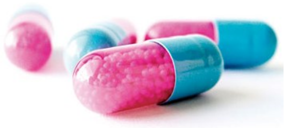

If you take Medicine A, you would live for **8.75 years (8 years and 9 months)** and then die.

If you take Medicine B, you would live for **10 years** and then die.

Would you prefer Medicine A or Medicine B, or are they the same?

Indicate your choice here. You can only choose one option:

○ **Medicine A** Live for 8 years and 9 months

○ **Medicine B** Live for 10 years

○ **Medicine A and Medicine B are the same.**

For peer review only

Repeated section

Remember Medicine A and Medicine B are equally effective and have no risks of a side effect.

The only difference between Medicine A and Medicine B is how you take them.

Medicine A	Medicine B
You take a tablet only once in your life.<br>You will be given the tablet on the day you come in to see your GP.<br>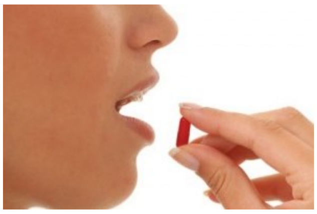	One tablet should be taken once every day, at night with cold water.<br>You need to take this medicine for 10 years.<br>You need to regularly visit a GP to receive a prescription.<br>You need to visit a pharmacy to fill a prescription.<br>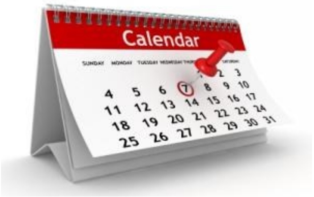<br>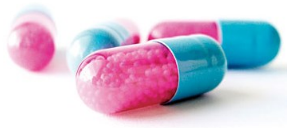

If you take Medicine A, you would live for **9 years** and then die.

If you take Medicine B, you would live for **10 years** and then die.

Would you prefer Medicine A or Medicine B, or are they the same?

Indicate your choice here. You can only choose one option:

○ **Medicine A** Live for 9 years

○ **Medicine B** Live for 10 years

○ **Medicine A and Medicine B are the same.**

For peer review only

Repeated section

Remember Medicine A and Medicine B are equally effective and have no risks of a side effect.

The only difference between Medicine A and Medicine B is how you take them.

Medicine A	Medicine B
You take a tablet only once in your life.<br>You will be given the tablet on the day you come in to see your GP.<br>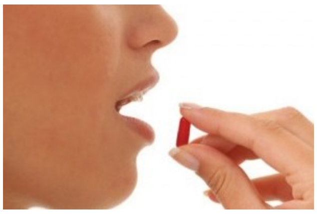	One tablet should be taken once every day, at night with cold water.<br>You need to take this medicine for 10 years.<br>You need to regularly visit a GP to receive a prescription.<br>You need to visit a pharmacy to fill a prescription.<br>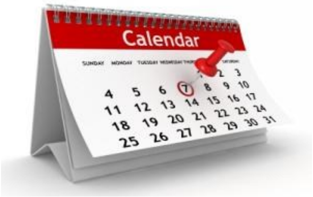<br>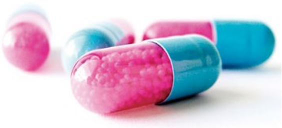

If you take Medicine A, you would live for **9 years and 1 month** and then die.

If you take Medicine B, you would live for **10 years** and then die.

Would you prefer Medicine A or Medicine B, or are they the same?

Indicate your choice here. You can only choose one option:

○ **Medicine A** Live for 9 years 1 month

○ **Medicine B** Live for 10 years

○ **Medicine A and Medicine B are the same.**

0% 100%

For peer review only

Repeated section

Remember Medicine A and Medicine B are equally effective and have no risks of a side effect.

The only difference between Medicine A and Medicine B is how you take them.

**Medicine A**	**Medicine B**
You take a tablet only once in your life.<br>You will be given the tablet on the day you come in to see your GP.<br>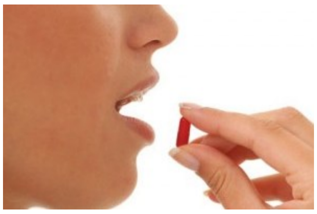	One tablet should be taken once every day, at night with cold water.<br>You need to take this medicine for 10 years.<br>You need to regularly visit a GP to receive a prescription.<br>You need to visit a pharmacy to fill a prescription.<br>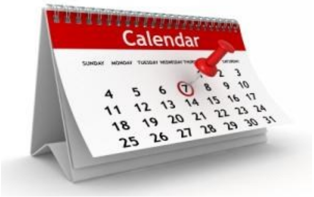<br>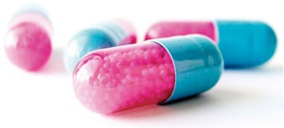

If you take Medicine A, you would live for **9.25 years (9 years and 3 months)** and then die.

If you take Medicine B, you would live for **10 years** and then die.

Would you prefer Medicine A or Medicine B, or are they the same?

Indicate your choice here. You can only choose one option:

○ **Medicine A** Live for 9 years and 3 months

○ **Medicine B** Live for 10 years

○ **Medicine A and Medicine B are the same.**

For peer review only

Repeated section

Remember Medicine A and Medicine B are equally effective and have no risks of a side effect.

The only difference between Medicine A and Medicine B is how you take them.

Medicine A	Medicine B
You take a tablet only once in your life.<br>You will be given the tablet on the day you come in to see your GP.<br>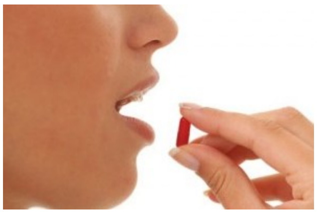	One tablet should be taken once every day, at night with cold water.<br>You need to take this medicine for 10 years.<br>You need to regularly visit a GP to receive a prescription.<br>You need to visit a pharmacy to fill a prescription.<br>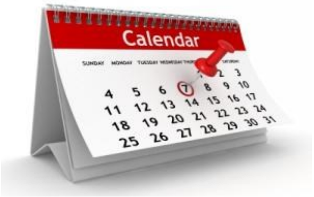<br><br>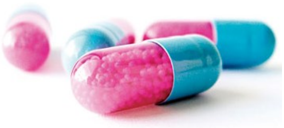

If you take Medicine A, you would live for **9 years and 4 months** and then die.

If you take Medicine B, you would live for **10 years** and then die.

Would you prefer Medicine A or Medicine B, or are they the same?

Indicate your choice here. You can only choose one option:

◯ **Medicine A** Live for 9 years 4 months

◯ **Medicine B** Live for 10 years

◯ **Medicine A and Medicine B are the same.**

0% 100%

For peer review only

Repeated section

Remember Medicine A and Medicine B are equally effective and have no risks of a side effect.

The only difference between Medicine A and Medicine B is how you take them.

Medicine A	Medicine B
You take a tablet only once in your life.<br>You will be given the tablet on the day you come in to see your GP.<br>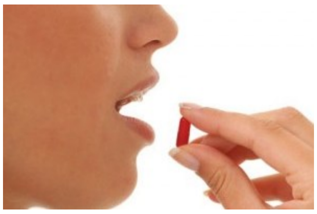	One tablet should be taken once every day, at night with cold water.<br>You need to take this medicine for 10 years.<br>You need to regularly visit a GP to receive a prescription.<br>You need to visit a pharmacy to fill a prescription.<br>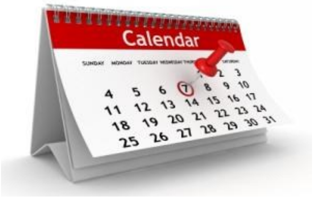<br>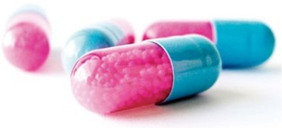

If you take Medicine A, you would live for **9 years and 5 months** and then die.

If you take Medicine B, you would live for **10 years** and then die.

Would you prefer Medicine A or Medicine B, or are they the same?

Indicate your choice here. You can only choose one option:

◯ **Medicine A** Live for 9 years 5 months

◯ **Medicine B** Live for 10 years

◯ **Medicine A and Medicine B are the same.**

For peer review only

Repeated section

Remember Medicine A and Medicine B are equally effective and have no risks of a side effect.

The only difference between Medicine A and Medicine B is how you take them.

**Medicine A**	**Medicine B**
You take a tablet only once in your life.<br>You will be given the tablet on the day you come in to see your GP.<br>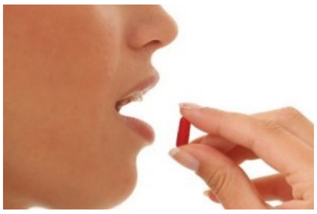	One tablet should be taken once every day, at night with cold water.<br>You need to take this medicine for 10 years.<br>You need to regularly visit a GP to receive a prescription.<br>You need to visit a pharmacy to fill a prescription.<br>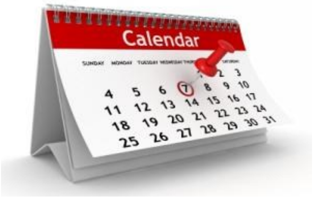<br>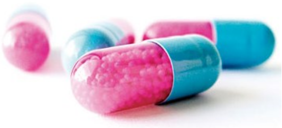

If you take Medicine A, you would live for **9.5 years** (9 years and 6 months) and then die.

If you take Medicine B, you would live for **10 years** and then die.

Would you prefer Medicine A or Medicine B, or are they the same?

Indicate your choice here. You can only choose one option:

◯ **Medicine A** Live for 9.5 years

◯ **Medicine B** Live for 10 years

◯ **Medicine A and Medicine B are the same.**

For peer review only

Repeated section

Remember Medicine A and Medicine B are equally effective and have no risks of a side effect.

The only difference between Medicine A and Medicine B is how you take them.

Medicine A	Medicine B
You take a tablet only once in your life.<br>You will be given the tablet on the day you come in to see your GP.<br>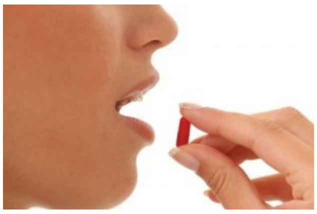	One tablet should be taken once every day, at night with cold water.<br>You need to take this medicine for 10 years.<br>You need to regularly visit a GP to receive a prescription.<br>You need to visit a pharmacy to fill a prescription.<br>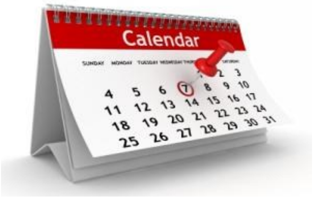<br>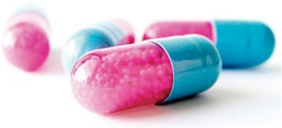

If you take Medicine A, you would live for **5 months less than 10 years** and then die.

If you take Medicine B, you would live for **10 years** and then die.

Would you prefer Medicine A or Medicine B, or are they the same?

Indicate your choice here. You can only choose one option:

○ **Medicine A** Live for (10 years – 5 months)

○ **Medicine B** Live for 10 years

○ **Medicine A and Medicine B are the same.**

0% 100%

For peer review only

Repeated section

Remember Medicine A and Medicine B are equally effective and have no risks of a side effect.

The only difference between Medicine A and Medicine B is how you take them.

Medicine A	Medicine B
You take a tablet only once in your life.<br>You will be given the tablet on the day you come in to see your GP.<br>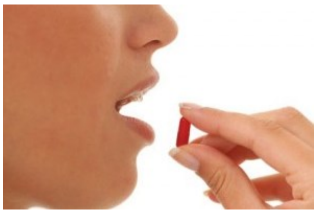	One tablet should be taken once every day, at night with cold water.<br>You need to take this medicine for 10 years.<br>You need to regularly visit a GP to receive a prescription.<br>You need to visit a pharmacy to fill a prescription.<br>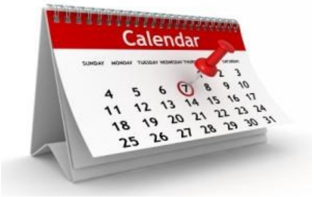<br><br>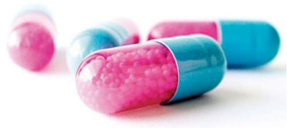

If you take Medicine A, you would live for **4 months less than 10 years** and then die.

If you take Medicine B, you would live for **10 years** and then die.

Would you prefer Medicine A or Medicine B, or are they the same?

Indicate your choice here. You can only choose one option:

◯ **Medicine A** Live for (10 years – 4 months)

◯ **Medicine B** Live for 10 years

◯ **Medicine A and Medicine B are the same.**

0% 100%

For peer review only

Repeated section

Remember Medicine A and Medicine B are equally effective and have no risks of a side effect.

The only difference between Medicine A and Medicine B is how you take them.

Medicine A	Medicine B
You take a tablet only once in your life.<br>You will be given the tablet on the day you come in to see your GP.<br>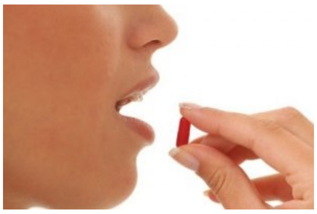	One tablet should be taken once every day, at night with cold water.<br>You need to take this medicine for 10 years.<br>You need to regularly visit a GP to receive a prescription.<br>You need to visit a pharmacy to fill a prescription.<br>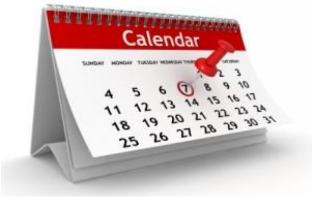 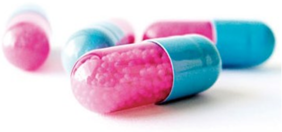

If you take Medicine A, you would live for **3 months less than 10 years** and then die.

If you take Medicine B, you would live for **10 years** and then die.

Would you prefer Medicine A or Medicine B, or are they the same?

Indicate your choice here. You can only choose one option:

○ **Medicine A** Live for (10 years – 3 months)

○ **Medicine B** Live for 10 years

○ **Medicine A and Medicine B are the same.**

For peer review only

Repeated section

Remember Medicine A and Medicine B are equally effective and have no risks of a side effect.

The only difference between Medicine A and Medicine B is how you take them.

Medicine A	Medicine B
You take a tablet only once in your life.<br>You will be given the tablet on the day you come in to see your GP.<br>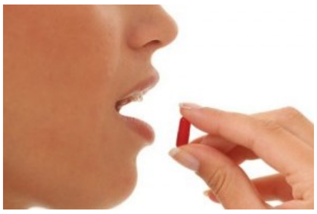	One tablet should be taken once every day, at night with cold water.<br>You need to take this medicine for 10 years.<br>You need to regularly visit a GP to receive a prescription.<br>You need to visit a pharmacy to fill a prescription.<br>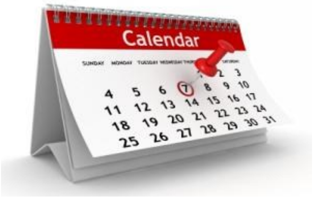<br>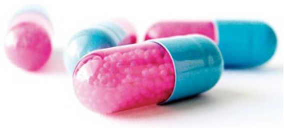

If you take Medicine A, you would live for **2 months less than 10 years** and then die.

If you take Medicine B, you would live for **10 years** and then die.

Would you prefer Medicine A or Medicine B, or are they the same?

Indicate your choice here. You can only choose one option:

○ **Medicine A** Live for (10 years – 2 months)

○ **Medicine B** Live for 10 years

○ **Medicine A and Medicine B are the same.**

For peer review only

Repeated section

Remember Medicine A and Medicine B are equally effective and have no risks of a side effect.

The only difference between Medicine A and Medicine B is how you take them.

**Medicine A**	**Medicine B**
You take a tablet only once in your life.<br>You will be given the tablet on the day you come in to see your GP.<br>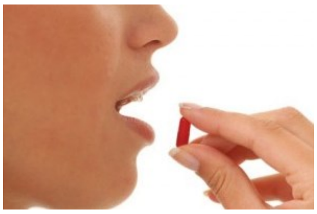	One tablet should be taken once every day, at night with cold water.<br>You need to take this medicine for 10 years.<br>You need to regularly visit a GP to receive a prescription.<br>You need to visit a pharmacy to fill a prescription.<br>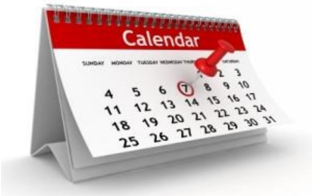<br>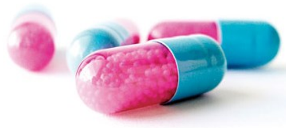

If you take Medicine A, you would live for **1 month less than 10 years** and then die.

If you take Medicine B, you would live for **10 years** and then die.

Would you prefer Medicine A or Medicine B, or are they the same?

Indicate your choice here. You can only choose one option:

◯ **Medicine A** 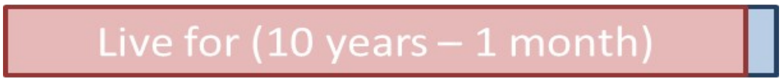

◯ **Medicine B** 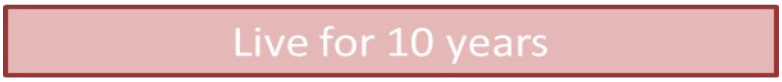

◯ **Medicine A and Medicine B are the same.**

For peer review only

Repeated section

Remember Medicine A and Medicine B are equally effective and have no risks of a side effect.

The only difference between Medicine A and Medicine B is how you take them.

**Medicine A**	**Medicine B**
You take a tablet only once in your life.<br>You will be given the tablet on the day you come in to see your GP.<br>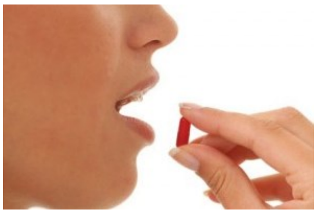	One tablet should be taken once every day, at night with cold water.<br>You need to take this medicine for 10 years.<br>You need to regularly visit a GP to receive a prescription.<br>You need to visit a pharmacy to fill a prescription.<br>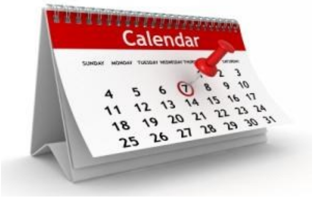<br><br>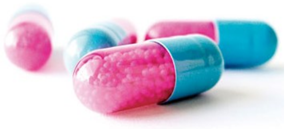

If you take Medicine A, you would live for **3 weeks less than 10 years** and then die.

If you take Medicine B, you would live for **10 years** and then die.

Would you prefer Medicine A or Medicine B, or are they the same?

Indicate your choice here. You can only choose one option:

○ **Medicine A** Live for (10 years – 3 weeks)

○ **Medicine B** Live for 10 years

○ **Medicine A and Medicine B are the same.**

0% 100%

For peer review only

Repeated section

Remember Medicine A and Medicine B are equally effective and have no risks of a side effect.

The only difference between Medicine A and Medicine B is how you take them.

Medicine A	Medicine B
You take a tablet only once in your life. You will be given the tablet on the day you come in to see your GP. 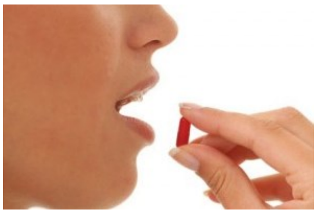	One tablet should be taken once every day, at night with cold water. You need to take this medicine for 10 years. You need to regularly visit a GP to receive a prescription. You need to visit a pharmacy to fill a prescription. 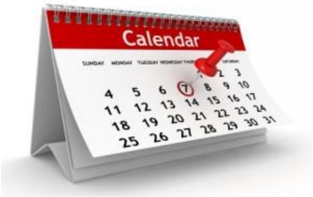 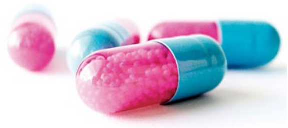

If you take Medicine A, you would live for **2 weeks less than 10 years** and then die.

If you take Medicine B, you would live for **10 years** and then die.

Would you prefer Medicine A or Medicine B, or are they the same?

Indicate your choice here. You can only choose one option:

◯ **Medicine A** Live for (10 years – 2 weeks)

◯ **Medicine B** Live for 10 years

◯ **Medicine A and Medicine B are the same.**

For peer review only

Repeated section

Remember Medicine A and Medicine B are equally effective and have no risks of a side effect.

The only difference between Medicine A and Medicine B is how you take them.

Medicine A	Medicine B
You take a tablet only once in your life.<br>You will be given the tablet on the day you come in to see your GP.<br>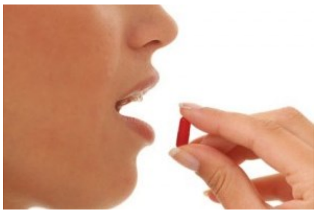	One tablet should be taken once every day, at night with cold water.<br>You need to take this medicine for 10 years.<br>You need to regularly visit a GP to receive a prescription.<br>You need to visit a pharmacy to fill a prescription.<br>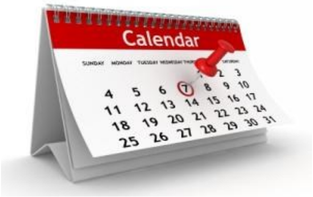<br>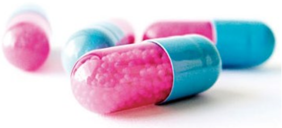

If you take Medicine A, you would live for **10 days less than 10 years** and then die.

If you take Medicine B, you would live for **10 years** and then die.

Would you prefer Medicine A or Medicine B, or are they the same?

Indicate your choice here. You can only choose one option:

○ **Medicine A** Live for (10 years – 10 days)

○ **Medicine B** Live for 10 years

○ **Medicine A and Medicine B are the same.**

For peer review only

Repeated section

Remember Medicine A and Medicine B are equally effective and have no risks of a side effect.

The only difference between Medicine A and Medicine B is how you take them.

Medicine A	Medicine B
You take a tablet only once in your life.<br>You will be given the tablet on the day you come in to see your GP.<br>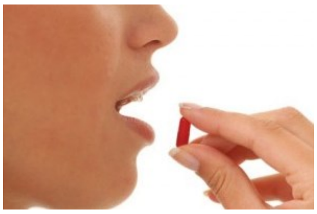	One tablet should be taken once every day, at night with cold water.<br>You need to take this medicine for 10 years.<br>You need to regularly visit a GP to receive a prescription.<br>You need to visit a pharmacy to fill a prescription.<br>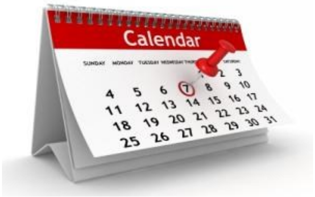<br>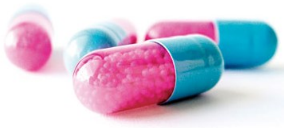

If you take Medicine A, you would live for **1 week less than 10 years** and then die.

If you take Medicine B, you would live for **10 years** and then die.

Would you prefer Medicine A or Medicine B, or are they the same?

Indicate your choice here. You can only choose one option:

◯ **Medicine A** Live for (10 years – 1 week)

◯ **Medicine B** Live for 10 years

◯ **Medicine A and Medicine B are the same.**

For peer review only

Repeated section

Remember Medicine A and Medicine B are equally effective and have no risks of a side effect.

The only difference between Medicine A and Medicine B is how you take them.

Medicine A	Medicine B
You take a tablet only once in your life.<br>You will be given the tablet on the day you come in to see your GP.<br>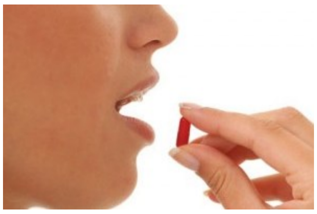	One tablet should be taken once every day, at night with cold water.<br>You need to take this medicine for 10 years.<br>You need to regularly visit a GP to receive a prescription.<br>You need to visit a pharmacy to fill a prescription.<br>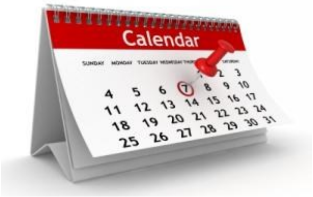 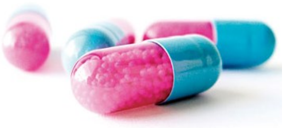

If you take Medicine A, you would live for **5 days less than 10 years** and then die.

If you take Medicine B, you would live for **10 years** and then die.

Would you prefer Medicine A or Medicine B, or are they the same?

Indicate your choice here. You can only choose one option:

○ **Medicine A** Live for (10 years – 5 days)

○ **Medicine B** Live for 10 years

○ **Medicine A and Medicine B are the same.**

For peer review only

Repeated section

Remember Medicine A and Medicine B are equally effective and have no risks of a side effect.

The only difference between Medicine A and Medicine B is how you take them.

Medicine A	Medicine B
You take a tablet only once in your life.<br>You will be given the tablet on the day you come in to see your GP.<br>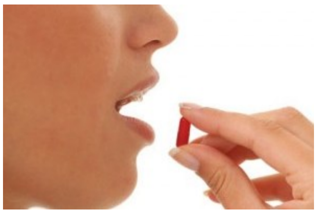	One tablet should be taken once every day, at night with cold water.<br>You need to take this medicine for 10 years.<br>You need to regularly visit a GP to receive a prescription.<br>You need to visit a pharmacy to fill a prescription.<br>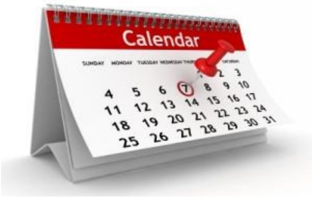<br><br>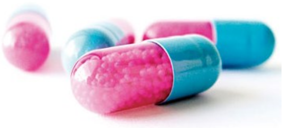

If you take Medicine A, you would live for **3 days less than 10 years** and then die.

If you take Medicine B, you would live for **10 years** and then die.

Would you prefer Medicine A or Medicine B, or are they the same?

Indicate your choice here. You can only choose one option:

○ **Medicine A** Live for (10 years – 3 days)

○ **Medicine B** Live for 10 years

○ **Medicine A and Medicine B are the same.**

For peer review only

Repeated section

Remember Medicine A and Medicine B are equally effective and have no risks of a side effect.

The only difference between Medicine A and Medicine B is how you take them.

Medicine A	Medicine B
You take a tablet only once in your life.<br>You will be given the tablet on the day you come in to see your GP.<br>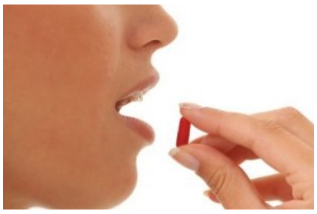	One tablet should be taken once every day, at night with cold water.<br>You need to take this medicine for 10 years.<br>You need to regularly visit a GP to receive a prescription.<br>You need to visit a pharmacy to fill a prescription.<br>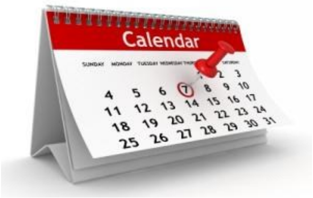 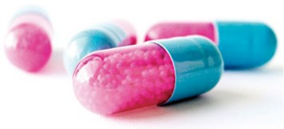

If you take Medicine A, you would live for **1 day less than 10 years** and then die.

If you take Medicine B, you would live for **10 years** and then die.

Would you prefer Medicine A or Medicine B, or are they the same?

Indicate your choice here. You can only choose one option:

○ **Medicine A** Live for (10 years – 1 day)

○ **Medicine B** Live for 10 years

○ **Medicine A and Medicine B are the same.**

0% 100%

For peer review only

Question 4a.

You prefer living for 5 years on Medicine A, to living for 10 years on Medicine B.

This means **living fewer than 5 years on Medicine A** can be the same as living for 10 years on Medicine B.

How many years on Medicine A do you think would be the same as living for 10 years on Medicine B?

(Please give a number in years)

0% 100%

peer review only

Was there anything so far which you think is unclear or that you didn't understand?

(Please provide details in the comment box below)

0% 100%

peer review only

Part 2a

Question 5

You have been told that you need to take a medicine to reduce the risk of heart disease.

You can choose between two different medicines. We have called these Medicine A and Medicine B.

Medicine A is a pill that you only need to take once.

Medicine B is a pill that you need to take every day for ten years.

Medicine A and Medicine B are equally effective in reducing your risk of developing heart disease.

Medicine A and Medicine B will reduce your risk of having a heart attack or a stroke within the next 10 years from **10%** to **6%**.

In other words, in a crowd of 1,000 people in the UK, **100** are likely to have a heart attack or a stroke if they do not take the medicine. This is reduced to **60** people out of 1,000 who do take the medicine. This information is also illustrated in this picture.

Risk of heart disease

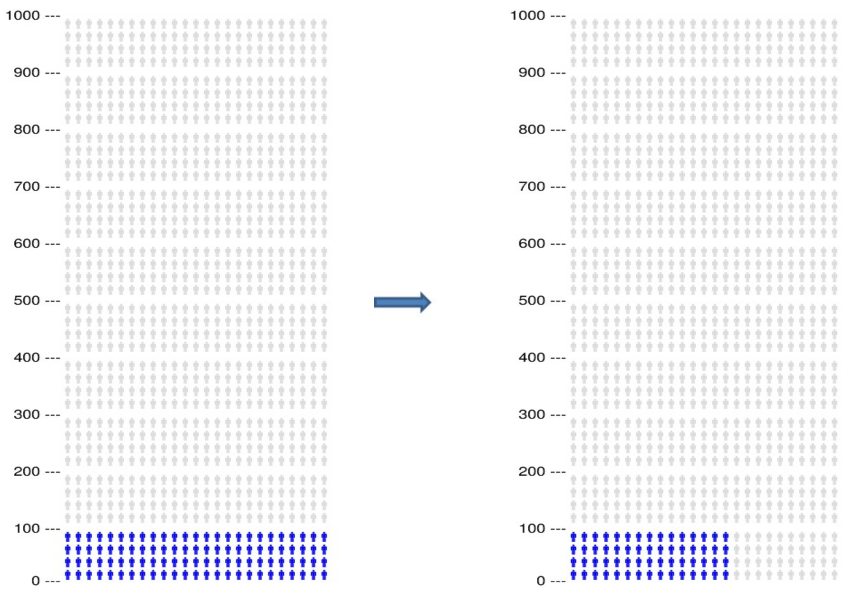

10%
No Treatment

6%
Taking the Medicine

(The figures shaded blue represent people who will have a heart attack or a stroke in the next 10 years)

Please continue to the next page.

For peer review only

Repeated section

Remember Medicine A and Medicine B are equally effective.

However, there are some differences between Medicine A and Medicine B.

Medicine A	Medicine B
**No inconvenience**<br><br>You take a tablet only once in your life.<br>You will be given the tablet on the day you come in to see your GP.	**Inconvenience**<br><br>One tablet should be taken once every day, at night with cold water.<br>You need to take this medicine for 10 years.<br>You need to regularly visit a GP to receive a prescription.<br>You need to visit a pharmacy to fill a prescription.
**Severe side effect - 0%**<br><br>No one taking Medicine A will have a severe side effect.	**Severe side effect - 0.7%**<br><br>Among every 1,000 people taking Medicine B, **7** will experience a severe side effect.
**Minor side effect - 0%**<br><br>No one taking Medicine A will have a minor side effect.	**Minor side effect - 13%**<br><br>Among every 1,000 people taking Medicine B, **130** will experience a minor side effect.

If you take Medicine A, you would live for **5 years** and then die.

If you take Medicine B, you would live for **10 years** and then die.

Would you prefer Medicine A or Medicine B, or are they the same?

Indicate your choice here. You can only choose one option:

◯ **Medicine A**

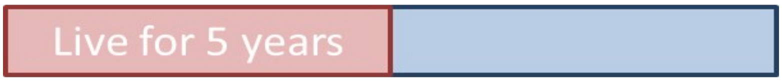

◯ **Medicine B**

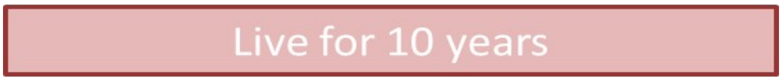

◯ **Medicine A and Medicine B are the same.**

0% 100%

Repeated section

Remember Medicine A and Medicine B are equally effective.

However, there are some differences between Medicine A and Medicine B.

Medicine A	Medicine B
**No inconvenience**<br><br>You take a tablet only once in your life.<br>You will be given the tablet on the day you come in to see your GP.	**Inconvenience**<br><br>One tablet should be taken once every day, at night with cold water.<br>You need to take this medicine for 10 years.<br>You need to regularly visit a GP to receive a prescription.<br>You need to visit a pharmacy to fill a prescription.
**Severe side effect - 0%**<br><br>No one taking Medicine A will have a severe side effect.	**Severe side effect - 0.7%**<br><br>Among every 1,000 people taking Medicine B, **7** will experience a severe side effect.
**Minor side effect - 0%**<br><br>No one taking Medicine A will have a minor side effect.	**Minor side effect - 13%**<br><br>Among every 1,000 people taking Medicine B, **130** will experience a minor side effect.

If you take Medicine A, you would live for **5.5 years** (5 years and 6 months) and then die.

If you take Medicine B, you would live for **10 years** and then die.

Would you prefer Medicine A or Medicine B, or are they the same?

Indicate your choice here. You can only choose one option:

◯ **Medicine A** Live for 5.5 years

◯ **Medicine B** Live for 10 years

◯ **Medicine A and Medicine B are the same.**

0% 100%

Repeated section

Remember Medicine A and Medicine B are equally effective.

However, there are some differences between Medicine A and Medicine B.

Medicine A	Medicine B
**No inconvenience**<br><br>You take a tablet only once in your life.<br>You will be given the tablet on the day you come in to see your GP.	**Inconvenience**<br><br>One tablet should be taken once every day, at night with cold water.<br>You need to take this medicine for 10 years.<br>You need to regularly visit a GP to receive a prescription.<br>You need to visit a pharmacy to fill a prescription.
**Severe side effect - 0%**<br><br>No one taking Medicine A will have a severe side effect.	**Severe side effect - 0.7%**<br><br>Among every 1,000 people taking Medicine B, **7** will experience a severe side effect.
**Minor side effect - 0%**<br><br>No one taking Medicine A will have a minor side effect.	**Minor side effect - 13%**<br><br>Among every 1,000 people taking Medicine B, **130** will experience a minor side effect.

If you take Medicine A, you would live for **6 years** and then die.

If you take Medicine B, you would live for **10 years** and then die.

Would you prefer Medicine A or Medicine B, or are they the same?

Indicate your choice here. You can only choose one option:

○ **Medicine A**

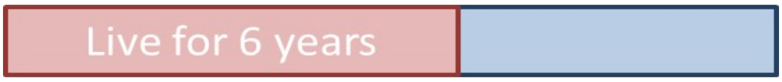

○ **Medicine B**

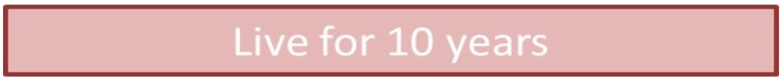

○ **Medicine A and Medicine B are the same.**

0% 100%

Repeated section

Remember Medicine A and Medicine B are equally effective.

However, there are some differences between Medicine A and Medicine B.

Medicine A	Medicine B
**No inconvenience**<br><br>You take a tablet only once in your life.<br>You will be given the tablet on the day you come in to see your GP.	**Inconvenience**<br><br>One tablet should be taken once every day, at night with cold water.<br>You need to take this medicine for 10 years.<br>You need to regularly visit a GP to receive a prescription.<br>You need to visit a pharmacy to fill a prescription.
**Severe side effect - 0%**<br><br>No one taking Medicine A will have a severe side effect.	**Severe side effect - 0.7%**<br><br>Among every 1,000 people taking Medicine B, **7** will experience a severe side effect.
**Minor side effect - 0%**<br><br>No one taking Medicine A will have a minor side effect.	**Minor side effect - 13%**<br><br>Among every 1,000 people taking Medicine B, **130** will experience a minor side effect.

If you take Medicine A, you would live for **6.25 years (6 years and 3 months)** and then die.

If you take Medicine B, you would live for **10 years** and then die.

Would you prefer Medicine A or Medicine B, or are they the same?

Indicate your choice here. You can only choose one option:

◯ **Medicine A**

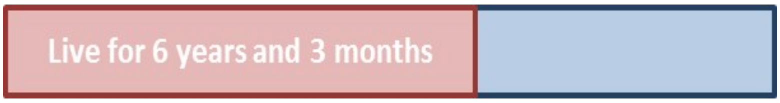

◯ **Medicine B**

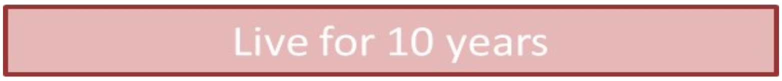

◯ **Medicine A and Medicine B are the same.**

0% 100%

Repeated section

Remember Medicine A and Medicine B are equally effective.

However, there are some differences between Medicine A and Medicine B.

Medicine A	Medicine B
**No inconvenience**<br><br>You take a tablet only once in your life.<br>You will be given the tablet on the day you come in to see your GP.	**Inconvenience**<br><br>One tablet should be taken once every day, at night with cold water.<br>You need to take this medicine for 10 years.<br>You need to regularly visit a GP to receive a prescription.<br>You need to visit a pharmacy to fill a prescription.
**Severe side effect - 0%**<br><br>No one taking Medicine A will have a severe side effect.	**Severe side effect - 0.7%**<br><br>Among every 1,000 people taking Medicine B, **7** will experience a severe side effect.
**Minor side effect - 0%**<br><br>No one taking Medicine A will have a minor side effect.	**Minor side effect - 13%**<br><br>Among every 1,000 people taking Medicine B, **130** will experience a minor side effect.

If you take Medicine A, you would live for **6.5 years** (6 years and 6 months) and then die.

If you take Medicine B, you would live for **10 years** and then die.

Would you prefer Medicine A or Medicine B, or are they the same?

Indicate your choice here. You can only choose one option:

◯ **Medicine A**

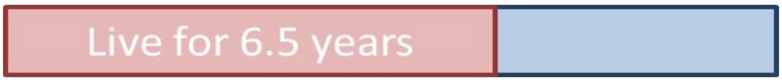

◯ **Medicine B**

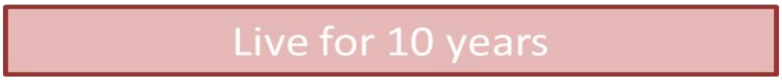

◯ **Medicine A and Medicine B are the same.**

0% 100%

Repeated section

Remember Medicine A and Medicine B are equally effective.

However, there are some differences between Medicine A and Medicine B.

Medicine A	Medicine B
**No inconvenience**<br><br>You take a tablet only once in your life.<br>You will be given the tablet on the day you come in to see your GP.	**Inconvenience**<br><br>One tablet should be taken once every day, at night with cold water.<br>You need to take this medicine for 10 years.<br>You need to regularly visit a GP to receive a prescription.<br>You need to visit a pharmacy to fill a prescription.
**Severe side effect - 0%**<br><br>No one taking Medicine A will have a severe side effect.	**Severe side effect - 0.7%**<br><br>Among every 1,000 people taking Medicine B, **7** will experience a severe side effect.
**Minor side effect - 0%**<br><br>No one taking Medicine A will have a minor side effect.	**Minor side effect - 13%**<br><br>Among every 1,000 people taking Medicine B, **130** will experience a minor side effect.

If you take Medicine A, you would live for **7 years** and then die.

If you take Medicine B, you would live for **10 years** and then die.

Would you prefer Medicine A or Medicine B, or are they the same?

Indicate your choice here. You can only choose one option:

○ **Medicine A** Live for 7 years

○ **Medicine B** Live for 10 years

○ **Medicine A and Medicine B are the same.**

0% 100%

Repeated section

Remember Medicine A and Medicine B are equally effective.

However, there are some differences between Medicine A and Medicine B.

Medicine A	Medicine B
**No inconvenience**<br><br>You take a tablet only once in your life.<br>You will be given the tablet on the day you come in to see your GP.	**Inconvenience**<br><br>One tablet should be taken once every day, at night with cold water.<br>You need to take this medicine for 10 years.<br>You need to regularly visit a GP to receive a prescription.<br>You need to visit a pharmacy to fill a prescription.
**Severe side effect - 0%**<br><br>No one taking Medicine A will have a severe side effect.	**Severe side effect - 0.7%**<br><br>Among every 1,000 people taking Medicine B, **7** will experience a severe side effect.
**Minor side effect - 0%**<br><br>No one taking Medicine A will have a minor side effect.	**Minor side effect - 13%**<br><br>Among every 1,000 people taking Medicine B, **130** will experience a minor side effect.

If you take Medicine A, you would live for **7.5 years** (7 years and 6 months) and then die.

If you take Medicine B, you would live for **10 years** and then die.

Would you prefer Medicine A or Medicine B, or are they the same?

Indicate your choice here. You can only choose one option:

◯ **Medicine A** 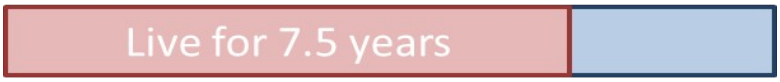

◯ **Medicine B** 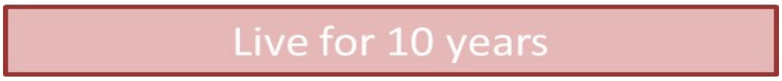

◯ **Medicine A and Medicine B are the same.**

0% 100%

Repeated section

Remember Medicine A and Medicine B are equally effective.

However, there are some differences between Medicine A and Medicine B.

Medicine A	Medicine B
**No inconvenience**<br><br>You take a tablet only once in your life.<br>You will be given the tablet on the day you come in to see your GP.	**Inconvenience**<br><br>One tablet should be taken once every day, at night with cold water.<br>You need to take this medicine for 10 years.<br>You need to regularly visit a GP to receive a prescription.<br>You need to visit a pharmacy to fill a prescription.
**Severe side effect - 0%**<br><br>No one taking Medicine A will have a severe side effect.	**Severe side effect - 0.7%**<br><br>Among every 1,000 people taking Medicine B, **7** will experience a severe side effect.
**Minor side effect - 0%**<br><br>No one taking Medicine A will have a minor side effect.	**Minor side effect - 13%**<br><br>Among every 1,000 people taking Medicine B, **130** will experience a minor side effect.

If you take Medicine A, you would live for **7.75 years (7 years and 9 months)** and then die.

If you take Medicine B, you would live for **10 years** and then die.

Would you prefer Medicine A or Medicine B, or are they the same?

Indicate your choice here. You can only choose one option:

◯ **Medicine A**

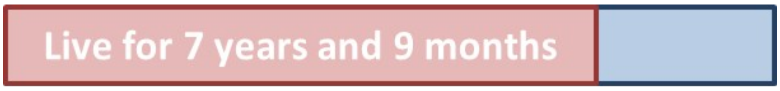

◯ **Medicine B**

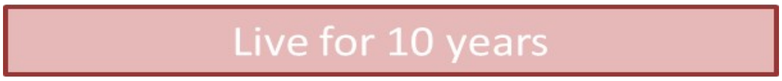

◯ **Medicine A and Medicine B are the same.**

0% 100%

Repeated section

Remember Medicine A and Medicine B are equally effective.

However, there are some differences between Medicine A and Medicine B.

Medicine A	Medicine B
**No inconvenience**<br><br>You take a tablet only once in your life.<br>You will be given the tablet on the day you come in to see your GP.	**Inconvenience**<br><br>One tablet should be taken once every day, at night with cold water.<br>You need to take this medicine for 10 years.<br>You need to regularly visit a GP to receive a prescription.<br>You need to visit a pharmacy to fill a prescription.
**Severe side effect - 0%**<br><br>No one taking Medicine A will have a severe side effect.	**Severe side effect - 0.7%**<br><br>Among every 1,000 people taking Medicine B, **7** will experience a severe side effect.
**Minor side effect - 0%**<br><br>No one taking Medicine A will have a minor side effect.	**Minor side effect - 13%**<br><br>Among every 1,000 people taking Medicine B, **130** will experience a minor side effect.

If you take Medicine A, you would live for **8 years** and then die.

If you take Medicine B, you would live for **10 years** and then die.

Would you prefer Medicine A or Medicine B, or are they the same?

Indicate your choice here. You can only choose one option:

◯ **Medicine A** Live for 8 years

◯ **Medicine B** Live for 10 years

◯ **Medicine A and Medicine B are the same.**

0% 100%

Repeated section

Remember Medicine A and Medicine B are equally effective.

However, there are some differences between Medicine A and Medicine B.

Medicine A	Medicine B
**No inconvenience**<br><br>You take a tablet only once in your life.<br>You will be given the tablet on the day you come in to see your GP.	**Inconvenience**<br><br>One tablet should be taken once every day, at night with cold water.<br>You need to take this medicine for 10 years.<br>You need to regularly visit a GP to receive a prescription.<br>You need to visit a pharmacy to fill a prescription.
**Severe side effect - 0%**<br><br>No one taking Medicine A will have a severe side effect.	**Severe side effect - 0.7%**<br><br>Among every 1,000 people taking Medicine B, **7** will experience a severe side effect.
**Minor side effect - 0%**<br><br>No one taking Medicine A will have a minor side effect.	**Minor side effect - 13%**<br><br>Among every 1,000 people taking Medicine B, **130** will experience a minor side effect.

If you take Medicine A, you would live for **8.25 years (8 years and 3 months)** and then die.

If you take Medicine B, you would live for **10 years** and then die.

Would you prefer Medicine A or Medicine B, or are they the same?

Indicate your choice here. You can only choose one option:

◯ **Medicine A** Live for 8 years and 3 months

◯ **Medicine B** Live for 10 years

◯ **Medicine A and Medicine B are the same.**

0% 100%

Repeated section

Remember Medicine A and Medicine B are equally effective.

However, there are some differences between Medicine A and Medicine B.

Medicine A	Medicine B
**No inconvenience**<br><br>You take a tablet only once in your life.<br>You will be given the tablet on the day you come in to see your GP.	**Inconvenience**<br><br>One tablet should be taken once every day, at night with cold water.<br>You need to take this medicine for 10 years.<br>You need to regularly visit a GP to receive a prescription.<br>You need to visit a pharmacy to fill a prescription.
**Severe side effect - 0%**<br><br>No one taking Medicine A will have a severe side effect.	**Severe side effect - 0.7%**<br><br>Among every 1,000 people taking Medicine B, **7** will experience a severe side effect.
**Minor side effect - 0%**<br><br>No one taking Medicine A will have a minor side effect.	**Minor side effect - 13%**<br><br>Among every 1,000 people taking Medicine B, **130** will experience a minor side effect.

If you take Medicine A, you would live for **8.5 years** (8 years and 6 months) and then die.

If you take Medicine B, you would live for **10 years** and then die.

Would you prefer Medicine A or Medicine B, or are they the same?

Indicate your choice here. You can only choose one option:

◯ **Medicine A** Live for 8.5 years

◯ **Medicine B** Live for 10 years

◯ **Medicine A and Medicine B are the same.**

0% 100%

Repeated section

Remember Medicine A and Medicine B are equally effective.

However, there are some differences between Medicine A and Medicine B.

Medicine A	Medicine B
**No inconvenience**<br><br>You take a tablet only once in your life.<br>You will be given the tablet on the day you come in to see your GP.	**Inconvenience**<br><br>One tablet should be taken once every day, at night with cold water.<br>You need to take this medicine for 10 years.<br>You need to regularly visit a GP to receive a prescription.<br>You need to visit a pharmacy to fill a prescription.
**Severe side effect - 0%**<br><br>No one taking Medicine A will have a severe side effect.	**Severe side effect - 0.7%**<br><br>Among every 1,000 people taking Medicine B, **7** will experience a severe side effect.
**Minor side effect - 0%**<br><br>No one taking Medicine A will have a minor side effect.	**Minor side effect - 13%**<br><br>Among every 1,000 people taking Medicine B, **130** will experience a minor side effect.

If you take Medicine A, you would live for **8.75 years (8 years and 9 months)** and then die.

If you take Medicine B, you would live for **10 years** and then die.

Would you prefer Medicine A or Medicine B, or are they the same?

Indicate your choice here. You can only choose one option:

○ **Medicine A** Live for 8 years and 9 months

○ **Medicine B** Live for 10 years

○ **Medicine A and Medicine B are the same.**

0% 100%

Repeated section

Remember Medicine A and Medicine B are equally effective.

However, there are some differences between Medicine A and Medicine B.

Medicine A	Medicine B
**No inconvenience**<br><br>You take a tablet only once in your life.<br>You will be given the tablet on the day you come in to see your GP.	**Inconvenience**<br><br>One tablet should be taken once every day, at night with cold water.<br>You need to take this medicine for 10 years.<br>You need to regularly visit a GP to receive a prescription.<br>You need to visit a pharmacy to fill a prescription.
**Severe side effect - 0%**<br><br>No one taking Medicine A will have a severe side effect.	**Severe side effect - 0.7%**<br><br>Among every 1,000 people taking Medicine B, **7** will experience a severe side effect.
**Minor side effect - 0%**<br><br>No one taking Medicine A will have a minor side effect.	**Minor side effect - 13%**<br><br>Among every 1,000 people taking Medicine B, **130** will experience a minor side effect.

If you take Medicine A, you would live for **9 years** and then die.

If you take Medicine B, you would live for **10 years** and then die.

Would you prefer Medicine A or Medicine B, or are they the same?

Indicate your choice here. You can only choose one option:

○ **Medicine A**

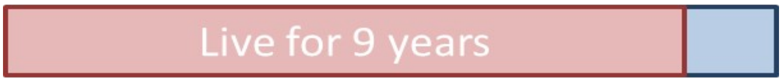

○ **Medicine B**

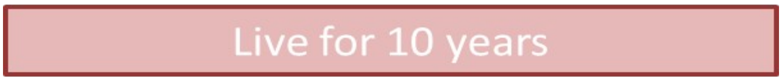

○ **Medicine A and Medicine B are the same.**

0% 100%

Repeated section

Remember Medicine A and Medicine B are equally effective.

However, there are some differences between Medicine A and Medicine B.

Medicine A	Medicine B
**No inconvenience**<br><br>You take a tablet only once in your life.<br>You will be given the tablet on the day you come in to see your GP.	**Inconvenience**<br><br>One tablet should be taken once every day, at night with cold water.<br>You need to take this medicine for 10 years.<br>You need to regularly visit a GP to receive a prescription.<br>You need to visit a pharmacy to fill a prescription.
**Severe side effect - 0%**<br><br>No one taking Medicine A will have a severe side effect.	**Severe side effect - 0.7%**<br><br>Among every 1,000 people taking Medicine B, **7** will experience a severe side effect.
**Minor side effect - 0%**<br><br>No one taking Medicine A will have a minor side effect.	**Minor side effect - 13%**<br><br>Among every 1,000 people taking Medicine B, **130** will experience a minor side effect.

If you take Medicine A, you would live for **9 years and 1 month** and then die.

If you take Medicine B, you would live for **10 years** and then die.

Would you prefer Medicine A or Medicine B, or are they the same?

Indicate your choice here. You can only choose one option:

◯ **Medicine A** Live for 9 years 1 month

◯ **Medicine B** Live for 10 years

◯ **Medicine A and Medicine B are the same.**

0% 100%

Repeated section

Remember Medicine A and Medicine B are equally effective.

However, there are some differences between Medicine A and Medicine B.

Medicine A	Medicine B
**No inconvenience**<br><br>You take a tablet only once in your life.<br>You will be given the tablet on the day you come in to see your GP.	**Inconvenience**<br><br>One tablet should be taken once every day, at night with cold water.<br>You need to take this medicine for 10 years.<br>You need to regularly visit a GP to receive a prescription.<br>You need to visit a pharmacy to fill a prescription.
**Severe side effect - 0%**<br><br>No one taking Medicine A will have a severe side effect.	**Severe side effect - 0.7%**<br><br>Among every 1,000 people taking Medicine B, **7** will experience a severe side effect.
**Minor side effect - 0%**<br><br>No one taking Medicine A will have a minor side effect.	**Minor side effect - 13%**<br><br>Among every 1,000 people taking Medicine B, **130** will experience a minor side effect.

If you take Medicine A, you would live for **9.25 years (9 years and 3 months)** and then die.

If you take Medicine B, you would live for **10 years** and then die.

Would you prefer Medicine A or Medicine B, or are they the same?

Indicate your choice here. You can only choose one option:

◯ **Medicine A** Live for 9 years and 3 months

◯ **Medicine B** Live for 10 years

◯ **Medicine A and Medicine B are the same.**

0% 100%

Repeated section

Remember Medicine A and Medicine B are equally effective.

However, there are some differences between Medicine A and Medicine B.

Medicine A	Medicine B
**No inconvenience**<br><br>You take a tablet only once in your life.<br>You will be given the tablet on the day you come in to see your GP.	**Inconvenience**<br><br>One tablet should be taken once every day, at night with cold water.<br>You need to take this medicine for 10 years.<br>You need to regularly visit a GP to receive a prescription.<br>You need to visit a pharmacy to fill a prescription.
**Severe side effect - 0%**<br><br>No one taking Medicine A will have a severe side effect.	**Severe side effect - 0.7%**<br><br>Among every 1,000 people taking Medicine B, **7** will experience a severe side effect.
**Minor side effect - 0%**<br><br>No one taking Medicine A will have a minor side effect.	**Minor side effect - 13%**<br><br>Among every 1,000 people taking Medicine B, **130** will experience a minor side effect.

If you take Medicine A, you would live for **9 years and 4 months** and then die.

If you take Medicine B, you would live for **10 years** and then die.

Would you prefer Medicine A or Medicine B, or are they the same?

Indicate your choice here. You can only choose one option:

◯ **Medicine A** Live for 9 years 4 months

◯ **Medicine B** Live for 10 years

◯ **Medicine A and Medicine B are the same.**

0% 100%

Repeated section

Remember Medicine A and Medicine B are equally effective.

However, there are some differences between Medicine A and Medicine B.

Medicine A	Medicine B
**No inconvenience**<br><br>You take a tablet only once in your life.<br>You will be given the tablet on the day you come in to see your GP.	**Inconvenience**<br><br>One tablet should be taken once every day, at night with cold water.<br>You need to take this medicine for 10 years.<br>You need to regularly visit a GP to receive a prescription.<br>You need to visit a pharmacy to fill a prescription.
**Severe side effect - 0%**<br><br>No one taking Medicine A will have a severe side effect.	**Severe side effect - 0.7%**<br><br>Among every 1,000 people taking Medicine B, **7** will experience a severe side effect.
**Minor side effect - 0%**<br><br>No one taking Medicine A will have a minor side effect.	**Minor side effect - 13%**<br><br>Among every 1,000 people taking Medicine B, **130** will experience a minor side effect.

If you take Medicine A, you would live for **9 years and 5 months** and then die.

If you take Medicine B, you would live for **10 years** and then die.

Would you prefer Medicine A or Medicine B, or are they the same?

Indicate your choice here. You can only choose one option:

○ **Medicine A** Live for 9 years 5 months

○ **Medicine B** Live for 10 years

○ **Medicine A and Medicine B are the same.**

0% 100%

Repeated section

Remember Medicine A and Medicine B are equally effective.

However, there are some differences between Medicine A and Medicine B.

Medicine A	Medicine B
**No inconvenience**<br><br>You take a tablet only once in your life.<br>You will be given the tablet on the day you come in to see your GP.	**Inconvenience**<br><br>One tablet should be taken once every day, at night with cold water.<br>You need to take this medicine for 10 years.<br>You need to regularly visit a GP to receive a prescription.<br>You need to visit a pharmacy to fill a prescription.
**Severe side effect - 0%**<br><br>No one taking Medicine A will have a severe side effect.	**Severe side effect - 0.7%**<br><br>Among every 1,000 people taking Medicine B, **7** will experience a severe side effect.
**Minor side effect - 0%**<br><br>No one taking Medicine A will have a minor side effect.	**Minor side effect - 13%**<br><br>Among every 1,000 people taking Medicine B, **130** will experience a minor side effect.

If you take Medicine A, you would live for **9.5 years** (9 years and 6 months) and then die.

If you take Medicine B, you would live for **10 years** and then die.

Would you prefer Medicine A or Medicine B, or are they the same?

Indicate your choice here. You can only choose one option:

◯ **Medicine A** Live for 9.5 years

◯ **Medicine B** Live for 10 years

◯ **Medicine A and Medicine B are the same.**

0% 100%

Repeated section

Remember Medicine A and Medicine B are equally effective.

However, there are some differences between Medicine A and Medicine B.

Medicine A	Medicine B
**No inconvenience**<br><br>You take a tablet only once in your life.<br>You will be given the tablet on the day you come in to see your GP.	**Inconvenience**<br><br>One tablet should be taken once every day, at night with cold water.<br>You need to take this medicine for 10 years.<br>You need to regularly visit a GP to receive a prescription.<br>You need to visit a pharmacy to fill a prescription.
**Severe side effect - 0%**<br><br>No one taking Medicine A will have a severe side effect.	**Severe side effect - 0.7%**<br><br>Among every 1,000 people taking Medicine B, **7** will experience a severe side effect.
**Minor side effect - 0%**<br><br>No one taking Medicine A will have a minor side effect.	**Minor side effect - 13%**<br><br>Among every 1,000 people taking Medicine B, **130** will experience a minor side effect.

If you take Medicine A, you would live for **5 months less than 10 years** and then die.

If you take Medicine B, you would live for **10 years** and then die.

Would you prefer Medicine A or Medicine B, or are they the same?

Indicate your choice here. You can only choose one option:

◯ **Medicine A** Live for (10 years – 5 months)

◯ **Medicine B** Live for 10 years

◯ **Medicine A and Medicine B are the same.**

0% 100%

Repeated section

Remember Medicine A and Medicine B are equally effective.

However, there are some differences between Medicine A and Medicine B.

**Medicine A**	**Medicine B**
**No inconvenience**<br><br>You take a tablet only once in your life.<br>You will be given the tablet on the day you come in to see your GP.	**Inconvenience**<br><br>One tablet should be taken once every day, at night with cold water.<br>You need to take this medicine for 10 years.<br>You need to regularly visit a GP to receive a prescription.<br>You need to visit a pharmacy to fill a prescription.
**Severe side effect - 0%**<br><br>No one taking Medicine A will have a severe side effect.	**Severe side effect - 0.7%**<br><br>Among every 1,000 people taking Medicine B, **7** will experience a severe side effect.
**Minor side effect - 0%**<br><br>No one taking Medicine A will have a minor side effect.	**Minor side effect - 13%**<br><br>Among every 1,000 people taking Medicine B, **130** will experience a minor side effect.

If you take Medicine A, you would live for **4 months less than 10 years** and then die.

If you take Medicine B, you would live for **10 years** and then die.

Would you prefer Medicine A or Medicine B, or are they the same?

Indicate your choice here. You can only choose one option:

◯ **Medicine A**

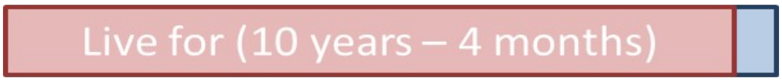

◯ **Medicine B**

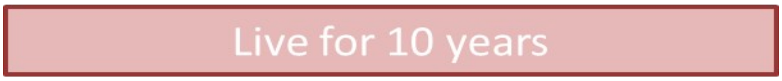

◯ **Medicine A and Medicine B are the same.**

0% 100%

Repeated section

Remember Medicine A and Medicine B are equally effective.

However, there are some differences between Medicine A and Medicine B.

Medicine A	Medicine B
**No inconvenience**<br><br>You take a tablet only once in your life.<br>You will be given the tablet on the day you come in to see your GP.	**Inconvenience**<br><br>One tablet should be taken once every day, at night with cold water.<br>You need to take this medicine for 10 years.<br>You need to regularly visit a GP to receive a prescription.<br>You need to visit a pharmacy to fill a prescription.
**Severe side effect - 0%**<br><br>No one taking Medicine A will have a severe side effect.	**Severe side effect - 0.7%**<br><br>Among every 1,000 people taking Medicine B, **7** will experience a severe side effect.
**Minor side effect - 0%**<br><br>No one taking Medicine A will have a minor side effect.	**Minor side effect - 13%**<br><br>Among every 1,000 people taking Medicine B, **130** will experience a minor side effect.

If you take Medicine A, you would live for **3 months less than 10 years** and then die.

If you take Medicine B, you would live for **10 years** and then die.

Would you prefer Medicine A or Medicine B, or are they the same?

Indicate your choice here. You can only choose one option:

◯ **Medicine A** Live for (10 years – 3 months)

◯ **Medicine B** Live for 10 years

◯ **Medicine A and Medicine B are the same.**

0% 100%

Repeated section

Remember Medicine A and Medicine B are equally effective.

However, there are some differences between Medicine A and Medicine B.

Medicine A	Medicine B
**No inconvenience**<br><br>You take a tablet only once in your life.<br>You will be given the tablet on the day you come in to see your GP.	**Inconvenience**<br><br>One tablet should be taken once every day, at night with cold water.<br>You need to take this medicine for 10 years.<br>You need to regularly visit a GP to receive a prescription.<br>You need to visit a pharmacy to fill a prescription.
**Severe side effect - 0%**<br><br>No one taking Medicine A will have a severe side effect.	**Severe side effect - 0.7%**<br><br>Among every 1,000 people taking Medicine B, **7** will experience a severe side effect.
**Minor side effect - 0%**<br><br>No one taking Medicine A will have a minor side effect.	**Minor side effect - 13%**<br><br>Among every 1,000 people taking Medicine B, **130** will experience a minor side effect.

If you take Medicine A, you would live for **2 months less than 10 years** and then die.

If you take Medicine B, you would live for **10 years** and then die.

Would you prefer Medicine A or Medicine B, or are they the same?

Indicate your choice here. You can only choose one option:

◯ **Medicine A** Live for (10 years – 2 months)

◯ **Medicine B** Live for 10 years

◯ **Medicine A and Medicine B are the same.**

0% 100%

Repeated section

Remember Medicine A and Medicine B are equally effective.

However, there are some differences between Medicine A and Medicine B.

Medicine A	Medicine B
**No inconvenience**<br><br>You take a tablet only once in your life.<br>You will be given the tablet on the day you come in to see your GP.	**Inconvenience**<br><br>One tablet should be taken once every day, at night with cold water.<br>You need to take this medicine for 10 years.<br>You need to regularly visit a GP to receive a prescription.<br>You need to visit a pharmacy to fill a prescription.
**Severe side effect - 0%**<br><br>No one taking Medicine A will have a severe side effect.	**Severe side effect - 0.7%**<br><br>Among every 1,000 people taking Medicine B, **7** will experience a severe side effect.
**Minor side effect - 0%**<br><br>No one taking Medicine A will have a minor side effect.	**Minor side effect - 13%**<br><br>Among every 1,000 people taking Medicine B, **130** will experience a minor side effect.

If you take Medicine A, you would live for **1 month less than 10 years** and then die.

If you take Medicine B, you would live for **10 years** and then die.

Would you prefer Medicine A or Medicine B, or are they the same?

Indicate your choice here. You can only choose one option:

○ **Medicine A** Live for (10 years – 1 month)

○ **Medicine B** Live for 10 years

○ **Medicine A and Medicine B are the same.**

0% 100%

Repeated section

Remember Medicine A and Medicine B are equally effective.

However, there are some differences between Medicine A and Medicine B.

Medicine A	Medicine B
**No inconvenience**<br><br>You take a tablet only once in your life.<br>You will be given the tablet on the day you come in to see your GP.	**Inconvenience**<br><br>One tablet should be taken once every day, at night with cold water.<br>You need to take this medicine for 10 years.<br>You need to regularly visit a GP to receive a prescription.<br>You need to visit a pharmacy to fill a prescription.
**Severe side effect - 0%**<br><br>No one taking Medicine A will have a severe side effect.	**Severe side effect - 0.7%**<br><br>Among every 1,000 people taking Medicine B, **7** will experience a severe side effect.
**Minor side effect - 0%**<br><br>No one taking Medicine A will have a minor side effect.	**Minor side effect - 13%**<br><br>Among every 1,000 people taking Medicine B, **130** will experience a minor side effect.

If you take Medicine A, you would live for **3 weeks less than 10 years** and then die.

If you take Medicine B, you would live for **10 years** and then die.

Would you prefer Medicine A or Medicine B, or are they the same?

Indicate your choice here. You can only choose one option:

○ **Medicine A** — Live for (10 years – 3 weeks)

○ **Medicine B** — Live for 10 years

○ **Medicine A and Medicine B are the same.**

0% 100%

Repeated section

Remember Medicine A and Medicine B are equally effective.

However, there are some differences between Medicine A and Medicine B.

Medicine A	Medicine B
**No inconvenience**<br><br>You take a tablet only once in your life.<br>You will be given the tablet on the day you come in to see your GP.	**Inconvenience**<br><br>One tablet should be taken once every day, at night with cold water.<br>You need to take this medicine for 10 years.<br>You need to regularly visit a GP to receive a prescription.<br>You need to visit a pharmacy to fill a prescription.
**Severe side effect - 0%**<br><br>No one taking Medicine A will have a severe side effect.	**Severe side effect - 0.7%**<br><br>Among every 1,000 people taking Medicine B, **7** will experience a severe side effect.
**Minor side effect - 0%**<br><br>No one taking Medicine A will have a minor side effect.	**Minor side effect - 13%**<br><br>Among every 1,000 people taking Medicine B, **130** will experience a minor side effect.

If you take Medicine A, you would live for **2 weeks less than 10 years** and then die.

If you take Medicine B, you would live for **10 years** and then die.

Would you prefer Medicine A or Medicine B, or are they the same?

Indicate your choice here. You can only choose one option:

○ **Medicine A** Live for (10 years – 2 weeks)

○ **Medicine B** Live for 10 years

○ **Medicine A and Medicine B are the same.**

0% 100%

Repeated section

Remember Medicine A and Medicine B are equally effective.

However, there are some differences between Medicine A and Medicine B.

Medicine A	Medicine B
**No inconvenience**<br><br>You take a tablet only once in your life.<br>You will be given the tablet on the day you come in to see your GP.	**Inconvenience**<br><br>One tablet should be taken once every day, at night with cold water.<br>You need to take this medicine for 10 years.<br>You need to regularly visit a GP to receive a prescription.<br>You need to visit a pharmacy to fill a prescription.
**Severe side effect - 0%**<br><br>No one taking Medicine A will have a severe side effect.	**Severe side effect - 0.7%**<br><br>Among every 1,000 people taking Medicine B, **7** will experience a severe side effect.
**Minor side effect - 0%**<br><br>No one taking Medicine A will have a minor side effect.	**Minor side effect - 13%**<br><br>Among every 1,000 people taking Medicine B, **130** will experience a minor side effect.

If you take Medicine A, you would live for **10 days less than 10 years** and then die.

If you take Medicine B, you would live for **10 years** and then die.

Would you prefer Medicine A or Medicine B, or are they the same?

Indicate your choice here. You can only choose one option:

◯ **Medicine A** — Live for (10 years – 10 days)

◯ **Medicine B** — Live for 10 years

◯ **Medicine A and Medicine B are the same.**

0% 100%

Repeated section

Remember Medicine A and Medicine B are equally effective.

However, there are some differences between Medicine A and Medicine B.

Medicine A	Medicine B
**No inconvenience**<br><br>You take a tablet only once in your life.<br>You will be given the tablet on the day you come in to see your GP.	**Inconvenience**<br><br>One tablet should be taken once every day, at night with cold water.<br>You need to take this medicine for 10 years.<br>You need to regularly visit a GP to receive a prescription.<br>You need to visit a pharmacy to fill a prescription.
**Severe side effect - 0%**<br><br>No one taking Medicine A will have a severe side effect.	**Severe side effect - 0.7%**<br><br>Among every 1,000 people taking Medicine B, **7** will experience a severe side effect.
**Minor side effect - 0%**<br><br>No one taking Medicine A will have a minor side effect.	**Minor side effect - 13%**<br><br>Among every 1,000 people taking Medicine B, **130** will experience a minor side effect.

If you take Medicine A, you would live for **1 week less than 10 years** and then die.

If you take Medicine B, you would live for **10 years** and then die.

Would you prefer Medicine A or Medicine B, or are they the same?

Indicate your choice here. You can only choose one option:

○ **Medicine A** Live for (10 years – 1 week)

○ **Medicine B** Live for 10 years

○ **Medicine A and Medicine B are the same.**

0% 100%

Repeated section

Remember Medicine A and Medicine B are equally effective.

However, there are some differences between Medicine A and Medicine B.

Medicine A	Medicine B
**No inconvenience**<br><br>You take a tablet only once in your life.<br>You will be given the tablet on the day you come in to see your GP.	**Inconvenience**<br><br>One tablet should be taken once every day, at night with cold water.<br>You need to take this medicine for 10 years.<br>You need to regularly visit a GP to receive a prescription.<br>You need to visit a pharmacy to fill a prescription.
**Severe side effect - 0%**<br><br>No one taking Medicine A will have a severe side effect.	**Severe side effect - 0.7%**<br><br>Among every 1,000 people taking Medicine B, **7** will experience a severe side effect.
**Minor side effect - 0%**<br><br>No one taking Medicine A will have a minor side effect.	**Minor side effect - 13%**<br><br>Among every 1,000 people taking Medicine B, **130** will experience a minor side effect.

If you take Medicine A, you would live for **5 days less than 10 years** and then die.

If you take Medicine B, you would live for **10 years** and then die.

Would you prefer Medicine A or Medicine B, or are they the same?

Indicate your choice here. You can only choose one option:

○ **Medicine A** Live for (10 years – 5 days)

○ **Medicine B** Live for 10 years

○ **Medicine A and Medicine B are the same.**

0% 100%

Repeated section

Remember Medicine A and Medicine B are equally effective.

However, there are some differences between Medicine A and Medicine B.

Medicine A	Medicine B
**No inconvenience**<br><br>You take a tablet only once in your life.<br>You will be given the tablet on the day you come in to see your GP.	**Inconvenience**<br><br>One tablet should be taken once every day, at night with cold water.<br>You need to take this medicine for 10 years.<br>You need to regularly visit a GP to receive a prescription.<br>You need to visit a pharmacy to fill a prescription.
**Severe side effect - 0%**<br><br>No one taking Medicine A will have a severe side effect.	**Severe side effect - 0.7%**<br><br>Among every 1,000 people taking Medicine B, **7** will experience a severe side effect.
**Minor side effect - 0%**<br><br>No one taking Medicine A will have a minor side effect.	**Minor side effect - 13%**<br><br>Among every 1,000 people taking Medicine B, **130** will experience a minor side effect.

If you take Medicine A, you would live for **3 days less than 10 years** and then die.

If you take Medicine B, you would live for **10 years** and then die.

Would you prefer Medicine A or Medicine B, or are they the same?

Indicate your choice here. You can only choose one option:

◯ **Medicine A** Live for (10 years – 3 days)

◯ **Medicine B** Live for 10 years

◯ **Medicine A and Medicine B are the same.**

0% 100%

Repeated section

Remember Medicine A and Medicine B are equally effective.

However, there are some differences between Medicine A and Medicine B.

Medicine A	Medicine B
**No inconvenience**<br><br>You take a tablet only once in your life.<br>You will be given the tablet on the day you come in to see your GP.	**Inconvenience**<br><br>One tablet should be taken once every day, at night with cold water.<br>You need to take this medicine for 10 years.<br>You need to regularly visit a GP to receive a prescription.<br>You need to visit a pharmacy to fill a prescription.
**Severe side effect - 0%**<br><br>No one taking Medicine A will have a severe side effect.	**Severe side effect - 0.7%**<br><br>Among every 1,000 people taking Medicine B, **7** will experience a severe side effect.
**Minor side effect - 0%**<br><br>No one taking Medicine A will have a minor side effect.	**Minor side effect - 13%**<br><br>Among every 1,000 people taking Medicine B, **130** will experience a minor side effect.

If you take Medicine A, you would live for **1 day less than 10 years** and then die.

If you take Medicine B, you would live for **10 years** and then die.

Would you prefer Medicine A or Medicine B, or are they the same?

Indicate your choice here. You can only choose one option:

○ **Medicine A** Live for (10 years – 1 day)

○ **Medicine B** Live for 10 years

○ **Medicine A and Medicine B are the same.**

0% 100%

Question 5a.

You prefer living for 5 years on Medicine A, to living for 10 years on Medicine B.

This means **living fewer than 5 years on Medicine A** can be the same as living for 10 years on Medicine B.

How many years on Medicine A do you think would be the same as living for 10 years on Medicine B?

(Please give a number in years)

0% 100%

peer review only

Thank you for completing this section and indicating your choices.

Was there anything in this section which you think is unclear or that you didn't understand?

(Please provide details in the comment box below)

0% 100%

For peer review only

Part 3. Questions about you

We understand different people feel differently about different treatments.

In this part of the survey, we ask questions about you to understand if people's age, gender or experiences explain the choices made in Part 2.

0% 100%

or peer review only

We would like to learn about your experience.

Are you currently taking, or have you ever taken any of these medicines?

Atorvastatin
Crestor®
Dorisin
Fluvastatin
Lescol®
Lipitor®
Nandovar
Pravastatin
Simvastatin
Simvador®
Zocor®

◯ Yes
◯ No

0% 100%

view only

What do you think would be the **benefits** from taking this medicine?

What do you think would be the **harms** from taking this medicine?

Have you experienced any side effects from taking this medicine?

- ◯ Yes
- ◯ No

Do you find it inconvenient to take your statin? Please specify if you think it is inconvenient.

What is your opinion about taking this medicine?

- ◯ I don't mind taking them because I have to take them for a reason.
- ◯ I do not like taking them because they remind me that I am not perfectly healthy.
- ◯ I have no opinion.
- ◯ Other (please specify)

0% 100%

Do you take other medicines prescribed by your doctor?

- ◯ Yes
- ◯ No

In total, how many medicines do you take on a regular basis?
(Please do not include pills you take only once in a while)

- ◯ None
- ◯ One
- ◯ Two to five
- ◯ Six to Ten
- ◯ More than ten

How many different times a day do you regularly take pills?

- ◯ None
- ◯ Once per day
- ◯ 2 times a day
- ◯ 3 times a day
- ◯ More than 3 times a day

Do you find it inconvenient to take your medicine(s)? Please specify if you think it is inconvenient.

What is your opinion about taking this medicine?

- ◯ I don't mind taking them because I have to take them for a reason.
- ◯ I do not like taking them because they remind me that I am not perfectly healthy.
- ◯ I have no opinion.
- ◯ Other (please specify)

0% 100%

For peer review only

We would like to ask questions about you to better understand your choices.

What is your gender?

- ◯ Male
- ◯ Female

Which age category do you fall into?

- ◯ 34 years or younger
- ◯ 35-44 years
- ◯ 45-54 years
- ◯ 55-64 years
- ◯ 65-74 years
- ◯ 75-84 years
- ◯ 85 years or over

Which best reflects your ethnic group?

- ◯ White British/Irish
- ◯ White other
- ◯ Mixed/Multiple ethnic origins
- ◯ Black/African/Caribbean/Black British
- ◯ Asian/Asian British
- ◯ Chinese
- ◯ Other (please specify) ________

0% 100%

What is your occupational status?

- ◯ Employed full-time
- ◯ Employed part-time
- ◯ Self-employed
- ◯ Unemployed
- ◯ Retired
- ◯ Looking after a home/family
- ◯ Student
- ◯ Freelance or temping
- ◯ Long-term sickness

Could you tell us what your occupation is?
(Previous occupation if retired.)

What is the highest level of education you have obtained?
(for example, GCSEs, Degrees, etc.)

What is your religion?

- ◯ No religion
- ◯ Christian
- ◯ Buddhist
- ◯ Jewish
- ◯ Hindu
- ◯ Muslim
- ◯ Sikh
- ◯ Other
- ◯ Prefer not to say

0% 100%

For peer review only

How do you feel about numbers?

This section will ask you some questions to understand how familiar you are with numbers and probabilities.

If you are unsure of the answer, you can leave the response blank.

0% 100%

For peer review only

Question 1

Imagine I flip a coin 1,000 times.

What is your best guess about how many times the coin would fall heads up in 1,000 flips?

- ◯ Answer: ____
- ◯ Prefer not to answer
- ◯ Don't know.

Question 2

If there was a lottery where the chance of winning £10 is 1%, if 1,000 people bought a ticket, how many would you expect to win?

- ◯ Answer: ____
- ◯ Prefer not to answer
- ◯ Don't know.

Question 3

In another lottery, the chance of winning a car is 1 in 1,000.

What percentage of tickets in the lottery will win a car?

- ◯ Answer: ____ %
- ◯ Prefer not to answer
- ◯ Don't know

0% 100%

How confident are you that you would answer the questions the same if faced with the situations in real-life?

	Very confident	Confident	Not sure	Not confident	Not confident at all	
Confident	◯	◯	◯	◯	◯	Not confident at all

How easy or difficult did you find making choices between alternatives?

	Very easy	Easy	Average	Somewhat difficult	Very difficult	
Easy	◯	◯	◯	◯	◯	Difficult

How easy or difficult did you find the survey to understand?

	Very easy	Easy	Average	Somewhat difficult	Very difficult	
Easy	◯	◯	◯	◯	◯	Difficult

0% 100%

eview only

Was there anything in the survey that was particularly unclear?

(Please write in the box below)

Is there anything else about the survey we could change to make it easier to understand (for example, phrasing and explanation)?

(Optional) If you have any further comments or suggestions, please write them here.

0% 100%

Thank you for completing this survey

If you have any questions or concerns please contact ResearchNow Support.

If you would like more information about heart disease please visit the NHS Choices website here.

0% 100%

peer review only

Note:

When respondents take the survey in regular mode this page will not be displayed. Respondents will be redirected to the link below:

https://dkr1.ssisurveys.com/projects/end?rst=1&psid= [Script] &basic=99423

0% 100%

For peer review only

Note:

When respondents take the survey in regular mode this page will not be displayed. Respondents will be redirected to the link below:

https://dkr1.ssisurveys.com/projects/end?rst=2&psid= [Script]

0% 100%

For peer review only

Note:

When respondents take the survey in regular mode this page will not be displayed. Respondents will be redirected to the link below:

https://dkr1.ssisurveys.com/projects/end?rst=3&psid= [Script]

0% 100%

For peer review only

Quantifying the impact of taking medicines for long-term conditions

A survey to understand your views about taking medicines long term for the prevention of osteoporotic fractures

In this survey, we will be asking you about a drug treatment which may be offered to people at an increased risk of having a fracture from osteoporosis.

There are no right or wrong answers, we are just interested in hearing about your views.

For peer review only

Quantifying the impact of taking medicines for long-term conditions

Taking medicines long term for the prevention of osteoporotic fracture

Participant Consent Form

You are being invited to take part in a research study that aims to understand your views about taking medicines long-term. Before you decide, it is important for you to understand why the research is being done and what it will involve.

Please click on the sections below for information on the research.

What is the aim of the research?

Why have I been chosen?

What happens to the data collected?

How is confidentiality maintained?

How long will it take me?

Who will conduct the research?

What would I be asked to do if I took part?

Will the outcomes of the research be published?

What happens if I do not want to take part or if I change my mind?

Who has reviewed the research project?

I understand that my participation in the study is voluntary and that I am free to withdraw at any time without giving a reason by exiting the survey.

I confirm that I have read the information above.

◯ Yes, I would like to take part in the survey.

◯ No, I do not want to participate (exit survey).

0% 100%

What is your gender?

- ◯ Female
- ◯ Male

0% 100%

For peer review only

[QUOTA: QuotaCheck]

Note:

This page only contains quota information and only appears in preview mode.

0% 100%

For peer review only

By selecting one box in each group below, please indicate which statements best describe your own health state today.

Mobility

- ◯ I have no problems in walking about
- ◯ I have some problems in walking about
- ◯ I am confined to bed

Self-Care

- ◯ I have no problems with self-care
- ◯ I have some problems washing or dressing myself
- ◯ I am unable to wash or dress myself

Usual Activities (e.g. work, study, housework, family or leisure activities)

- ◯ I have no problems with performing my usual activities
- ◯ I have some problems with performing my usual activities
- ◯ I am unable to perform my usual activities

Pain/Discomfort

- ◯ I have no pain or discomfort
- ◯ I have moderate pain or discomfort
- ◯ I have extreme pain or discomfort

Anxiety/Depression

- ◯ I am not anxious or depressed
- ◯ I am moderately anxious or depressed
- ◯ I am extremely anxious or depressed

0% 100%

Part 1. Background

This section of the survey explains: osteoporosis (thinning of the bones), how medicine treatments may be used for people at risk of osteoporotic fracture, and what the treatment involves.

We will follow a story of Alex.

Click next to continue.

0% 100%

For peer review only

Part 1. Background

Alex has been to see a doctor who told her that she has weak bones.

The doctor has said Alex needs to start a medicine to prevent broken bones (fractures) because of her weak bones.

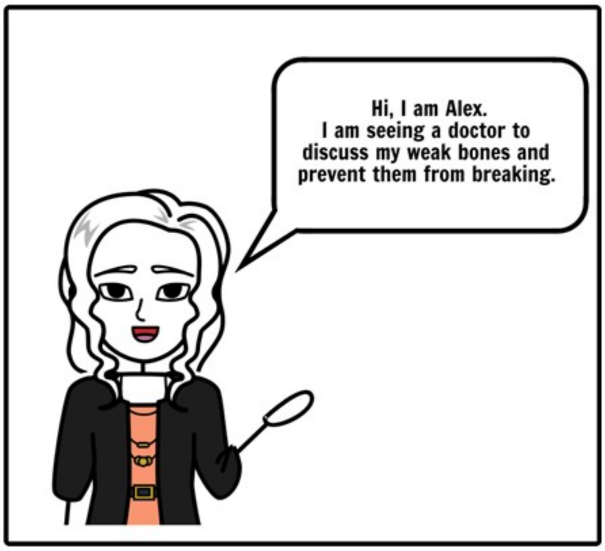

We will take you through Alex's story.

0% 100%

Part 1. Background

What is osteoporosis?

Alex hears about osteoporosis from her GP.

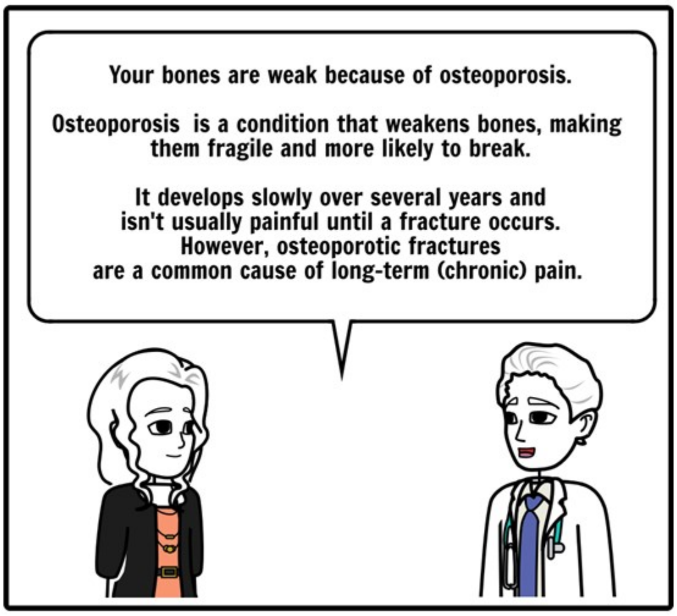

0% 100%

Part 1. Background

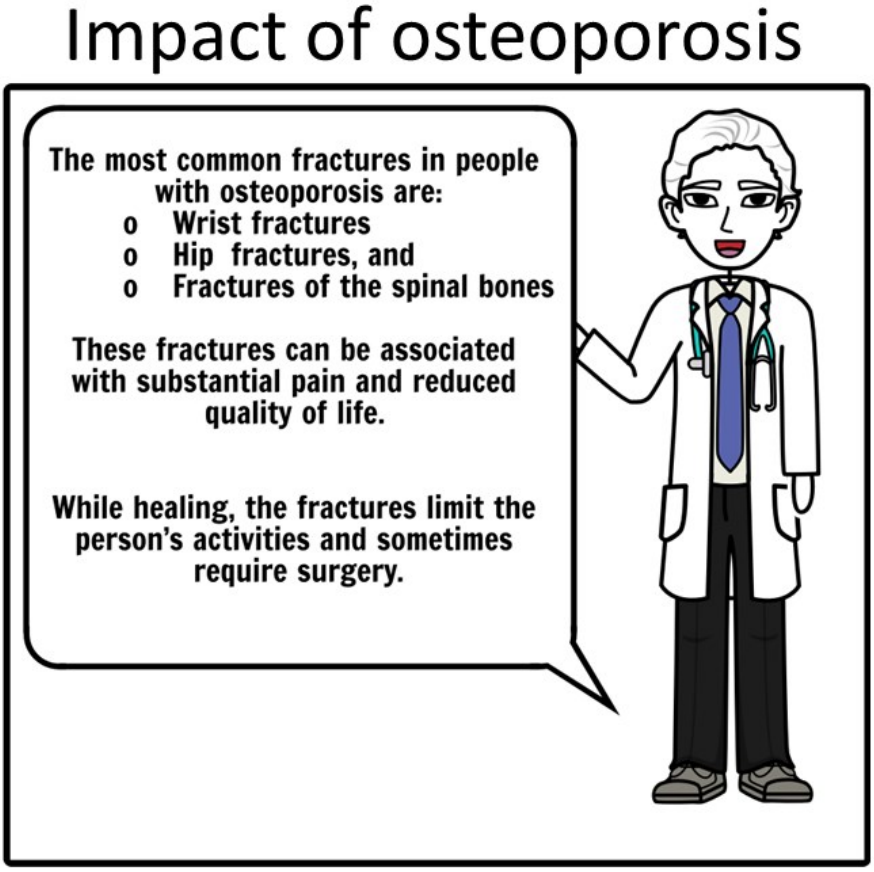

0% 100%

Part 1. Background

Risk of osteoporotic fractures

Her GP explains that Alex has some risk of fracture as her family has a history of hip fracture.

Also, Alex has a low body mass index (BMI). BMI is a measure of body fat.

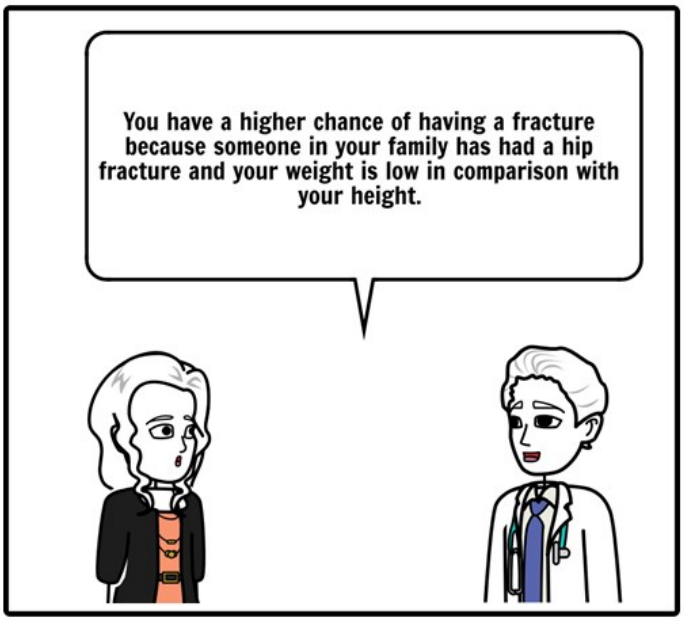

0% 100%

Part 1. Background

What is risk?

- **Risk** is: a term used to explain the chance that something bad might happen.

- **10% risk** means:

  Out of every **1,000** people, with osteoporosis,

  100 people would have a fracture, and **900** people would not.

0% 100%

nly

Part 1. Background

What is a 10% risk?

- This diagram shows a 10% risk of having a fracture because of their osteoporosis.
- The people shaded blue are the ones who have a fracture.

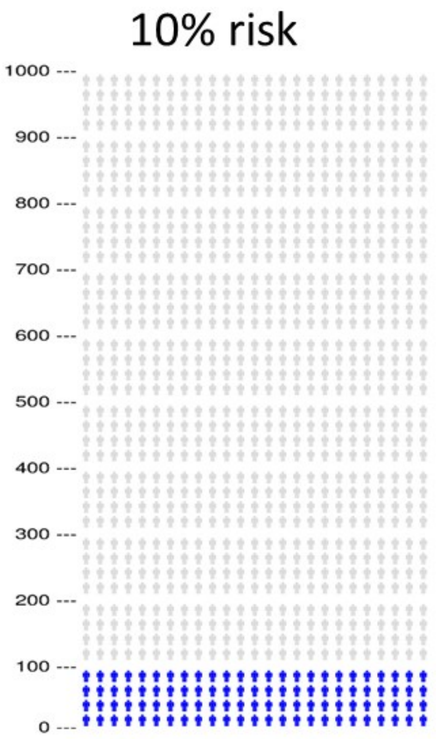

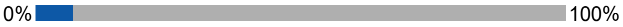

Part 1. Background

Prevention of fracture from osteoporosis

- Alex's doctor can estimate how likely it is that she will experience osteoporotic fracture over the next 5 years.
- The estimate is based on things such as Alex's age and sex, ethnicity, family fracture history, and body mass index.
- The doctor may prescribe certain medicines such as bisphosphonates to help reduce the chance of Alex having a fracture.
- The medicine will prevent some of the people who take it from having a fracture because of osteoporosis.
- Fractures may still happen in some of the people who take the medicine.

0% 100%

Part 1. Background

Prevention of fractures from osteoporosis

The doctor prescribes Alex a medicine to reduce the risk of her having a fracture in the future because of her osteoporosis.

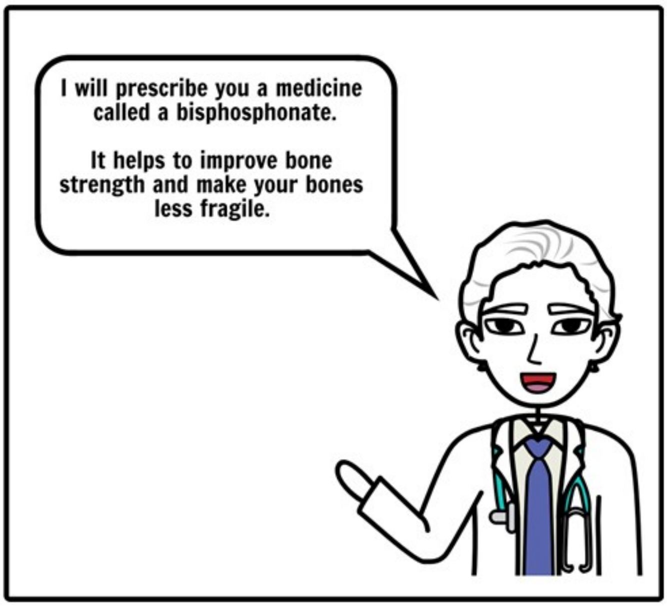

0% 100%

Part 1. Background

Collecting the medicine from the pharmacy

The pharmacist explains to Alex how to take the medicine.

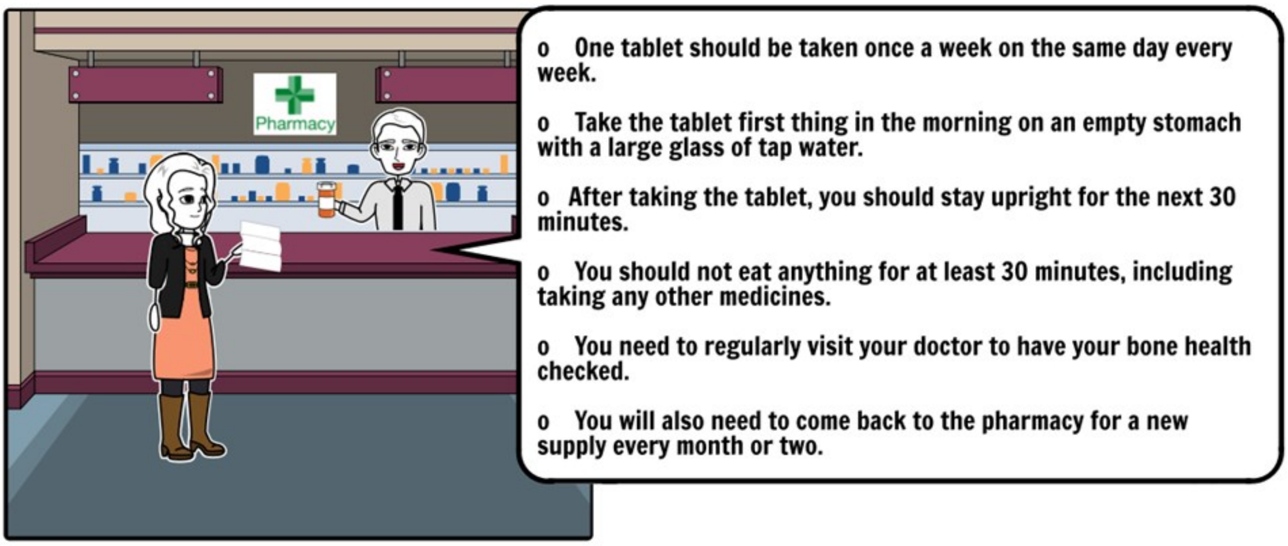

0% 100%

Part 1. Background

Taking the medicine for prevention

Alex goes home with her new tablets.

She then considers whether she wants to take them as the doctor and the pharmacist have advised.

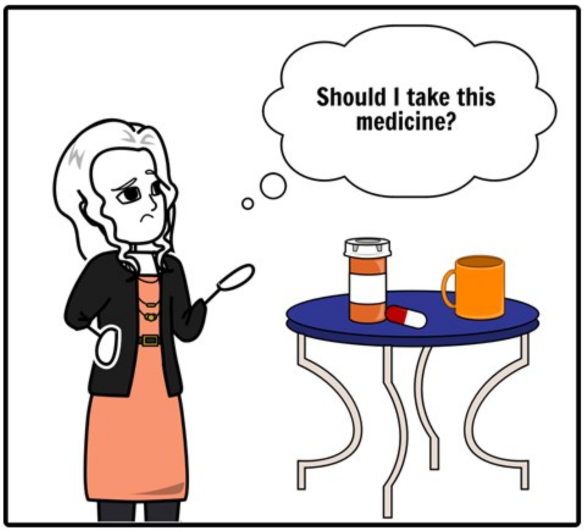

0% 100%

Part 1. Background

Alex may consider *three key things*,
when deciding whether to take the medicine.

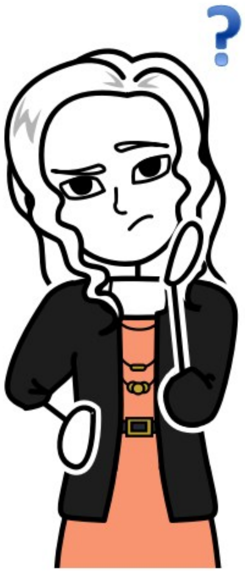

Effectiveness of the medicine

- How good the medicine is in terms of preventing a fracture from osteoporosis

Side effects of the medicine

- Whether there is any potential harm from the medicine

Inconvenience of the medicine

- Whether taking the medicine would fit in her lifestyle or cause her inconvenience

0% 100%

Part 1. Background

How effective is the medicine?

- Some people, but not everyone, who take the medicine will avoid a serious event, such as hip fracture, as a result of taking the tablet.
- The effectiveness of the medicine can be described in terms of a reduction in this risk.

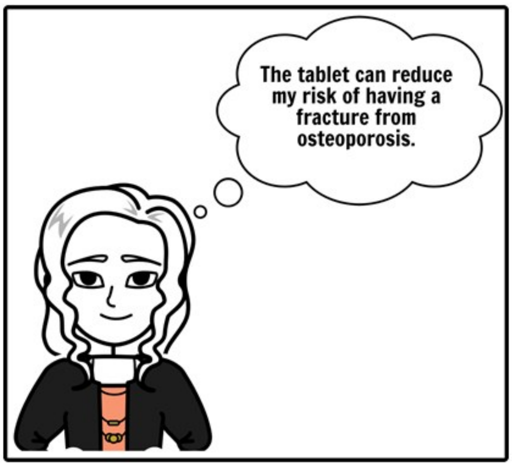

0% 100%

nly

Part 1. Background

What does ‘reduction in risk’ mean?

- A ‘reduction in risk from 10% to 6%’ means:

The medicine will reduce your risk of experiencing a fracture, such as hip fracture, within the next 5 years from 10% to 6%.

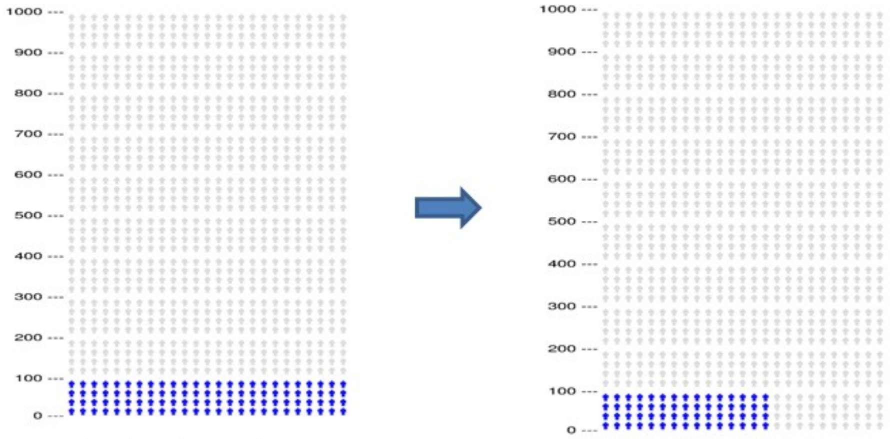

No treatment:
10% risk

Taking the medicine:
6% risk

0% 100%

Part 1. Background

Does the medicine have side effects?

The potential for side effects from the medicine can be described in terms of the **risk of a side effect**.

Alex hears about minor but more common side effects & severe but rarer side effects from her GP.

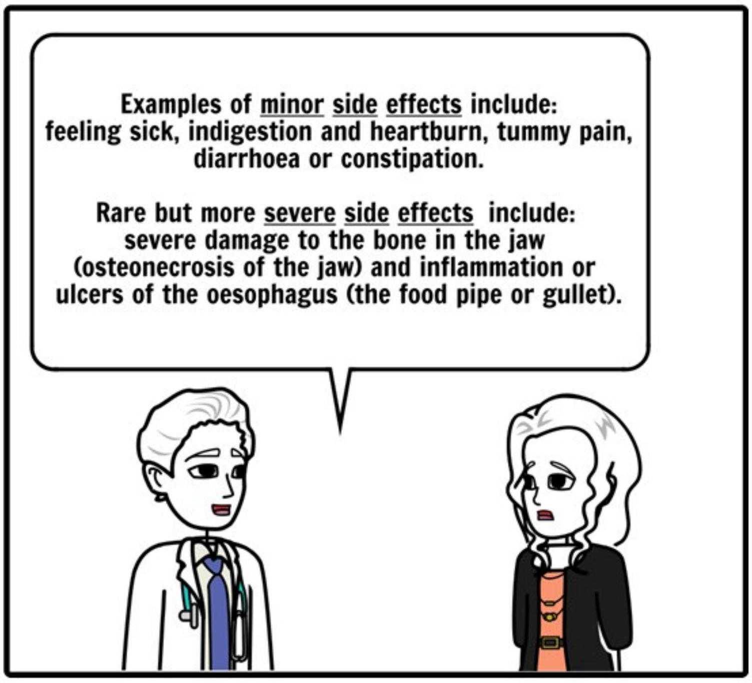

0% 100%

Part 1. Background

Risk of a minor side effect from the medicine

- A 5% risk of a minor side effect means:

Among every **1,000** people taking the medicine,

50 will experience a minor side effect such as either: feeling sick, indigestion, diarrhoea or constipation.

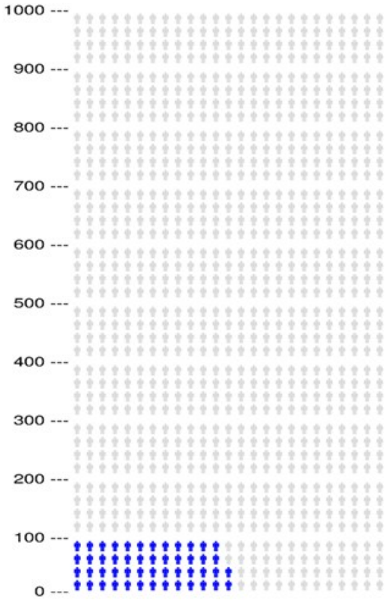

5% risk of a side effect

0% 100%

Part 1. Background

Risk of a severe side effect from the medicine

- A 0.3% risk of severe side effect means:

Among every **1,000** people taking the medicine,

3 will experience a severe side effect such as severe loss or destruction of the bone in the jaw.

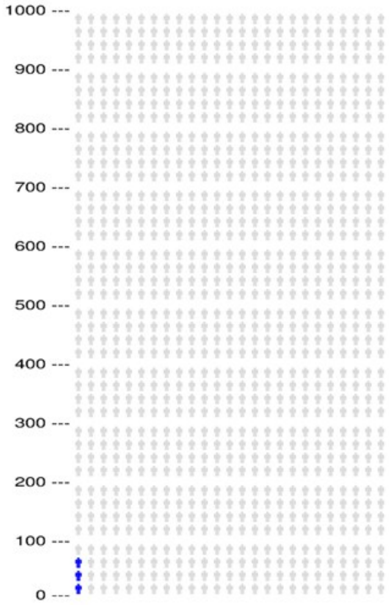

0.3% risk of a side effect

0% 100%

nly

Part 1. Background

How inconvenient is it when taking the medicine?

Alex also thinks about what it means to have to take this medicine for the next 5 years.

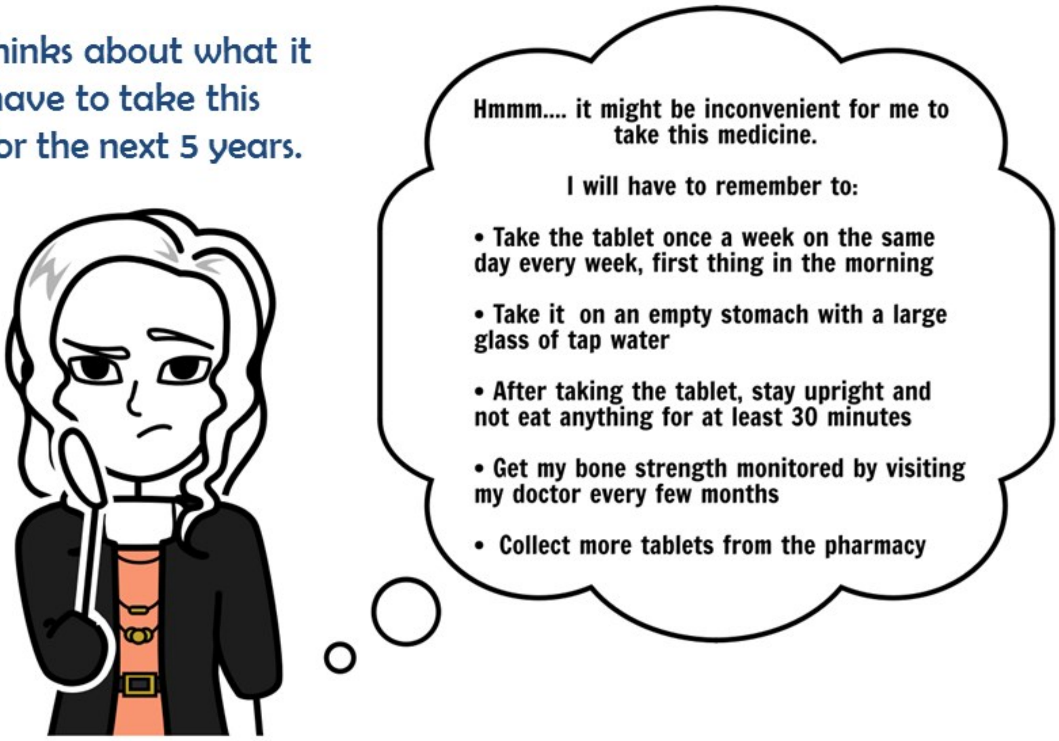

0% 100%

Part 1. Background

In the questions that follow in this survey,
we will ask you about how you think about taking
a medicine to prevent osteoporotic fracture.

0% 100%

nly

In general, how would you feel about taking a medicine for the first time?

- ◯ **Happy**
  I trust in my doctor and I am willing to try new things
- ◯ **Unsure**
  I'd like to think about it really carefully
- ◯ **Unhappy**
  I don't like the idea of trying a new medicine even if the doctor says it will help

0% 100%

For peer review only

Part 2: Your views on the inconvenience of taking a medicine to prevent fractures

Now, we will ask you about how you think about the inconvenience of taking a medicine to prevent osteoporotic fractures.

There are four questions.

Each question may look similar but differs in terms of the detail for each of the three features.

0% 100%

eer review only

Warm up question

You have been told that you need to take a medicine to reduce the risk of fracture.

You can choose between two different medicines. We have called these Medicine A and Medicine B.

Medicine A is a pill that you only need to take once.

Medicine B is a pill that you need to take every week for 5 years.

Medicine A and Medicine B are equally effective in reducing your risk of having a fracture.
Medicine A and Medicine B will reduce your risk of having an osteoporotic fracture within the next 5 years from **10%** to **6%**.

In other words, in a crowd of **1,000** people in the UK, **100** are likely to have a fracture if they do not take the medicine. This is reduced to **60** people out of 1,000 who do take the medicine.
This information is also illustrated in this picture.

Risk of fracture

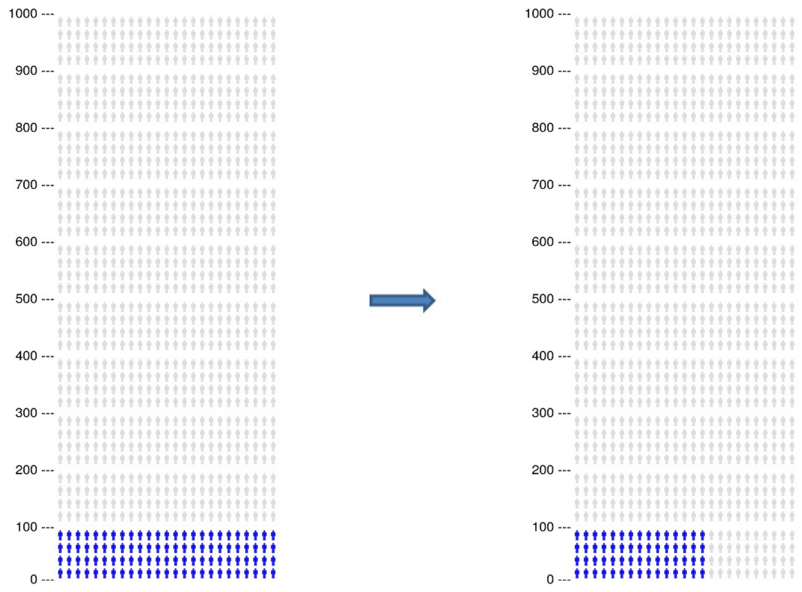

10%
No Treatment

6%
Taking the Medicine

(The figures shaded blue represent people who will have a fracture in the next 5 years)

In this scenario, you will not have any side effects from Medicine A or Medicine B.

Risk of a side effect

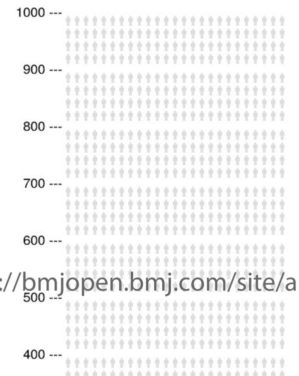

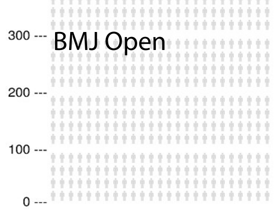

0 %
No one will have a side effect.

0% 100%

or peer review only

Remember Medicine A and Medicine B are equally effective and have no risks of a side effect.

The only difference between Medicine A and Medicine B is how you take them.

Medicine A	Medicine B
You take a tablet only once in your life.<br>You will be given the tablet on the day you come in to see your GP.<br>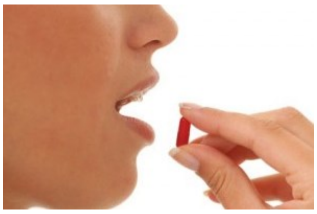	One tablet should be taken once a week on the same day every week.<br>You need to take this medicine for 5 years.<br><br>You need to take the tablet first thing in the morning on an empty stomach with a large glass of tap water.<br>After taking the tablet, you should stay upright for the next 30 minutes.<br>You should not eat anything for at least 30 minutes, including taking any other medicines.<br><br>You need to regularly visit your doctor to have your bone health checked.<br>You need to regularly visit a pharmacy for a new supply.<br>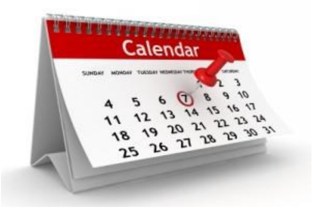 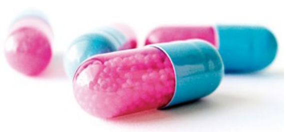

If you take Medicine A, you would live for **1 year** and then die.

If you take Medicine B, you would live for **5 years** and then die.

Would you prefer Medicine A or Medicine B, or are they the same?

Indicate your choice here. You can only choose one option:

Live for 1 year

◯ **Medicine A**

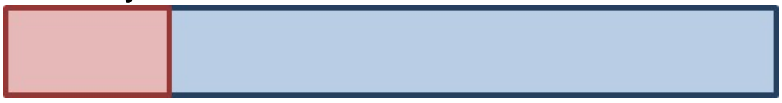

◯ **Medicine B**

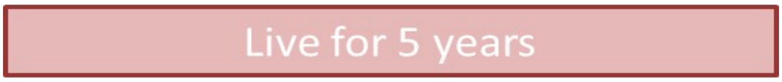

◯ **Medicine A and Medicine B are the same.**

0% 100%

Now we are ready to start the survey questions.

0% 100%

For peer review only

Part 2

Question 1

You have been told that you need to take a medicine to reduce the risk of fracture.

You can choose between two different medicines. We have called these Medicine A and Medicine B.

Medicine A is a pill that you only need to take once.

Medicine B is a pill that you need to take every week for 5 years.

Medicine A and Medicine B are equally effective in reducing your risk of having a fracture.
Medicine A and Medicine B will reduce your risk of experiencing a fracture, such as hip fracture, within the next 5 years from **10%** to **6%**.

In other words, in a crowd of **1,000** people in the UK, **100** are likely to have a fracture if they do not take the medicine. This is reduced to **60** people out of 100 who do take the medicine.
This information is also illustrated in this picture.

Risk of fracture

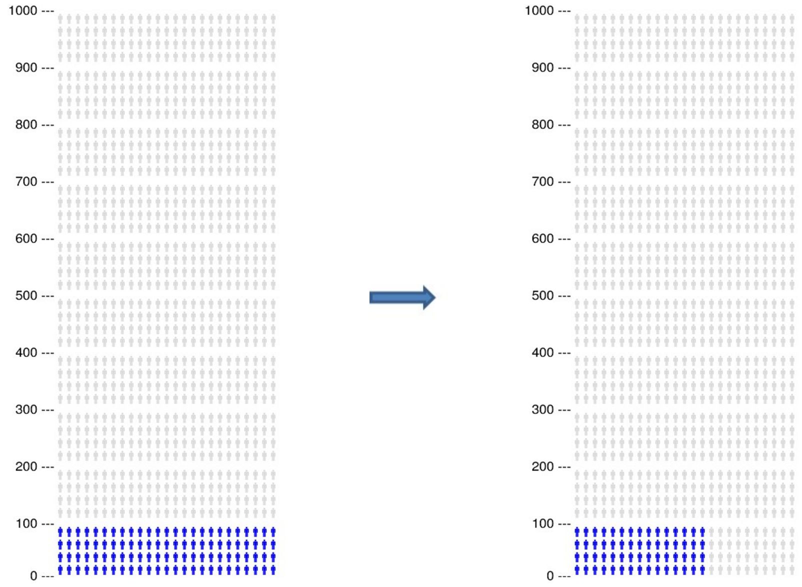

10%
No Treatment

6%
Taking the Medicine

(The figures shaded blue represent people who will have a fracture, such as hip fracture, within the next 5 years)

In this scenario, you will not have any side effects from Medicine A or Medicine B.

Risk of a side effect

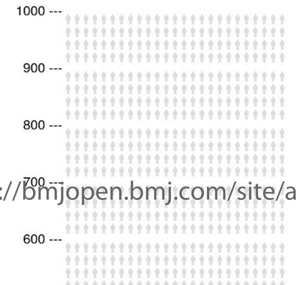

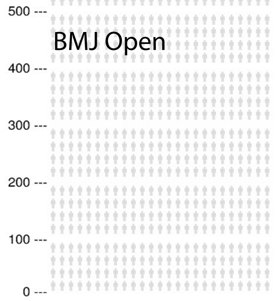

0 %
No one will have a side effect.

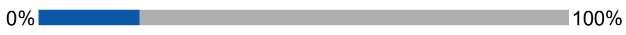

peer review only

Repeated section

Remember Medicine A and Medicine B are equally effective and have no risks of a side effect.

The only difference between Medicine A and Medicine B is how you take them.

Medicine A	Medicine B
You take a tablet only once in your life. You will be given the tablet on the day you come in to see your GP. 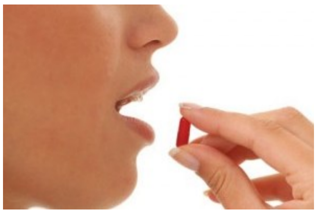	One tablet should be taken once a week on the same day every week. You need to take this medicine for 5 years.<br><br>You need to take the tablet first thing in the morning on an empty stomach with a large glass of tap water. After taking the tablet, you should stay upright for the next 30 minutes. You should not eat anything for at least 30 minutes, including taking any other medicines.<br><br>You need to regularly visit your doctor to have your bone health checked. You need to regularly visit a pharmacy for a new supply. 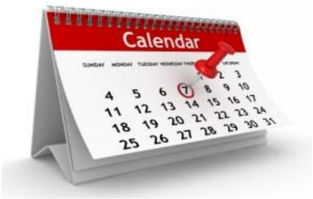 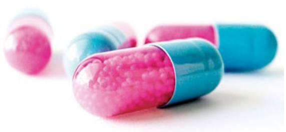

If you take Medicine A, you would live for **2.5 years (2 years and 6 months)** and then die.

If you take Medicine B, you would live for **5 years** and then die.

Would you prefer Medicine A or Medicine B, or are they the same?

Indicate your choice here. You can only choose one option:

◯ **Medicine A** Live for 2.5 years

◯ **Medicine B** Live for 5 years

◯ **The Same - Medicine A and Medicine B are the same.**

0% 100%

For peer review only

Repeated section

Remember Medicine A and Medicine B are equally effective and have no risks of a side effect.

The only difference between Medicine A and Medicine B is how you take them.

**Medicine A**	**Medicine B**
You take a tablet only once in your life. You will be given the tablet on the day you come in to see your GP.<br>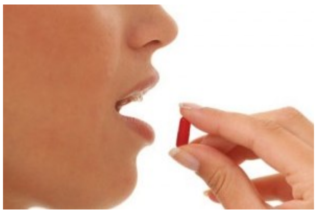	One tablet should be taken once a week on the same day every week.<br>You need to take this medicine for 5 years.<br><br>You need to take the tablet first thing in the morning on an empty stomach with a large glass of tap water.<br>After taking the tablet, you should stay upright for the next 30 minutes.<br>You should not eat anything for at least 30 minutes, including taking any other medicines.<br><br>You need to regularly visit your doctor to have your bone health checked.<br>You need to regularly visit a pharmacy for a new supply.<br>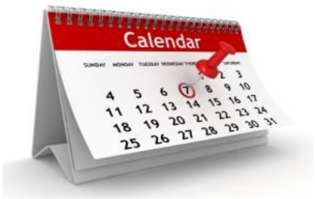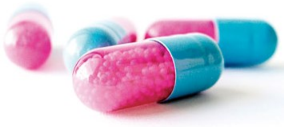

If you take Medicine A, you would live for **3 years** and then die.

If you take Medicine B, you would live for **5 years** and then die.

Would you prefer Medicine A or Medicine B, or are they the same?

Indicate your choice here. You can only choose one option:

◯ **Medicine A** 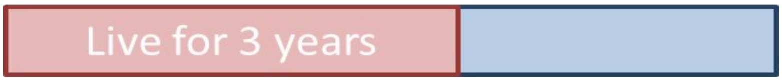

◯ **Medicine B** 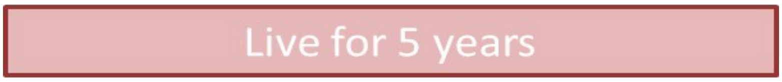

◯ **The Same - Medicine A and Medicine B are the same.**

For peer review only

Repeated section

Remember Medicine A and Medicine B are equally effective and have no risks of a side effect.

The only difference between Medicine A and Medicine B is how you take them.

Medicine A	Medicine B
You take a tablet only once in your life.<br>You will be given the tablet on the day you come in to see your GP.<br>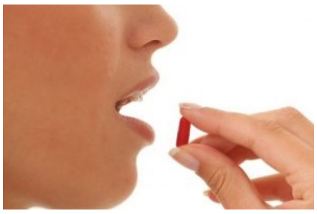	One tablet should be taken once a week on the same day every week.<br>You need to take this medicine for 5 years.<br><br>You need to take the tablet first thing in the morning on an empty stomach with a large glass of tap water.<br>After taking the tablet, you should stay upright for the next 30 minutes.<br>You should not eat anything for at least 30 minutes, including taking any other medicines.<br><br>You need to regularly visit your doctor to have your bone health checked.<br>You need to regularly visit a pharmacy for a new supply.<br>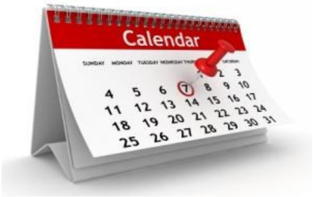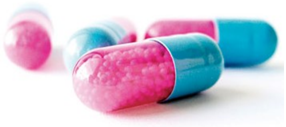

If you take Medicine A, you would live for **3.25 years** (3 years and 3 months) and then die.

If you take Medicine B, you would live for **5 years** and then die.

Would you prefer Medicine A or Medicine B, or are they the same?

Indicate your choice here. You can only choose one option:

○ **Medicine A** Live for 3 years 3 months

○ **Medicine B** Live for 5 years

○ **The Same - Medicine A and Medicine B are the same.**

0% 100%

For peer review only

Repeated section

Remember Medicine A and Medicine B are equally effective and have no risks of a side effect.

The only difference between Medicine A and Medicine B is how you take them.

Medicine A	Medicine B
You take a tablet only once in your life. You will be given the tablet on the day you come in to see your GP.<br>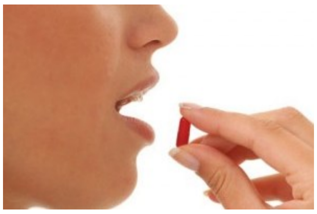	One tablet should be taken once a week on the same day every week.<br>You need to take this medicine for 5 years.<br><br>You need to take the tablet first thing in the morning on an empty stomach with a large glass of tap water.<br>After taking the tablet, you should stay upright for the next 30 minutes.<br>You should not eat anything for at least 30 minutes, including taking any other medicines.<br><br>You need to regularly visit your doctor to have your bone health checked.<br>You need to regularly visit a pharmacy for a new supply.<br>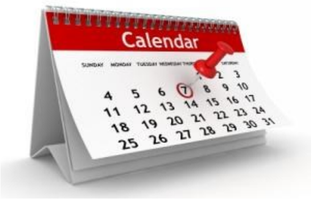 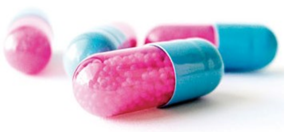

If you take Medicine A, you would live for **3.5 years** (3 years and 6 months) and then die.

If you take Medicine B, you would live for **5 years** and then die.

Would you prefer Medicine A or Medicine B, or are they the same?

Indicate your choice here. You can only choose one option:

◯ **Medicine A** Live for 3 years 6 months

◯ **Medicine B** Live for 5 years

◯ **The Same - Medicine A and Medicine B are the same.**

0% 100%

For peer review only

Repeated section

Remember Medicine A and Medicine B are equally effective and have no risks of a side effect.

The only difference between Medicine A and Medicine B is how you take them.

Medicine A	Medicine B
You take a tablet only once in your life. You will be given the tablet on the day you come in to see your GP. 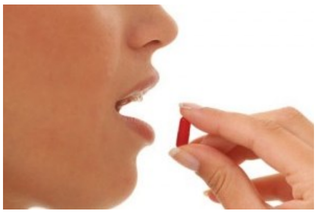	One tablet should be taken once a week on the same day every week. You need to take this medicine for 5 years. You need to take the tablet first thing in the morning on an empty stomach with a large glass of tap water. After taking the tablet, you should stay upright for the next 30 minutes. You should not eat anything for at least 30 minutes, including taking any other medicines. You need to regularly visit your doctor to have your bone health checked. You need to regularly visit a pharmacy for a new supply. 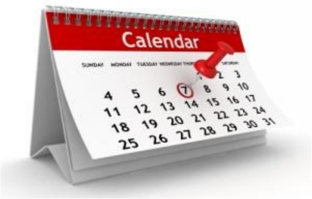 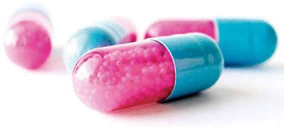

If you take Medicine A, you would live for **3.75 years** (3 years and 9 months) and then die.

If you take Medicine B, you would live for **5 years** and then die.

Would you prefer Medicine A or Medicine B, or are they the same?

Indicate your choice here. You can only choose one option:

○ **Medicine A** Live for 3 years 9 months

○ **Medicine B** Live for 5 years

○ **The Same - Medicine A and Medicine B are the same.**

0% 100%

For peer review only

Repeated section

Remember Medicine A and Medicine B are equally effective and have no risks of a side effect.

The only difference between Medicine A and Medicine B is how you take them.

Medicine A	Medicine B
You take a tablet only once in your life. You will be given the tablet on the day you come in to see your GP. 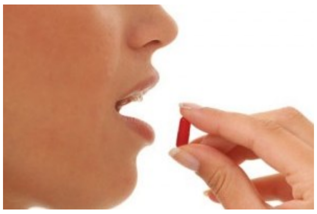	One tablet should be taken once a week on the same day every week. You need to take this medicine for 5 years.<br><br>You need to take the tablet first thing in the morning on an empty stomach with a large glass of tap water. After taking the tablet, you should stay upright for the next 30 minutes. You should not eat anything for at least 30 minutes, including taking any other medicines.<br><br>You need to regularly visit your doctor to have your bone health checked. You need to regularly visit a pharmacy for a new supply.<br>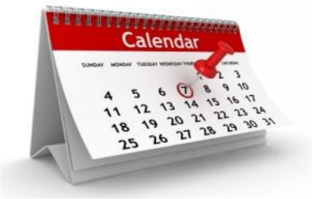 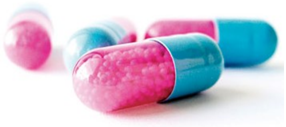

If you take Medicine A, you would live for **3 years and 10 months** and then die.

If you take Medicine B, you would live for **5 years** and then die.

Would you prefer Medicine A or Medicine B, or are they the same?

Indicate your choice here. You can only choose one option:

○ **Medicine A** — Live for 3 years 10 months

○ **Medicine B** — Live for 5 years

○ **The Same - Medicine A and Medicine B are the same.**

0% 100%

For peer review only

Repeated section

Remember Medicine A and Medicine B are equally effective and have no risks of a side effect.

The only difference between Medicine A and Medicine B is how you take them.

Medicine A	Medicine B
You take a tablet only once in your life. You will be given the tablet on the day you come in to see your GP. 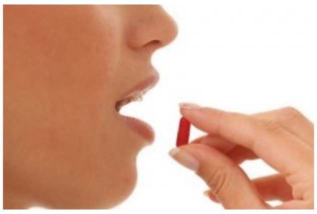	One tablet should be taken once a week on the same day every week. You need to take this medicine for 5 years.<br><br>You need to take the tablet first thing in the morning on an empty stomach with a large glass of tap water. After taking the tablet, you should stay upright for the next 30 minutes. You should not eat anything for at least 30 minutes, including taking any other medicines.<br><br>You need to regularly visit your doctor to have your bone health checked. You need to regularly visit a pharmacy for a new supply. 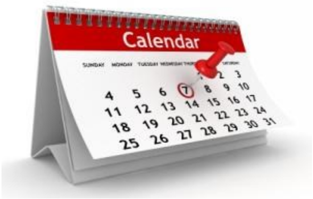 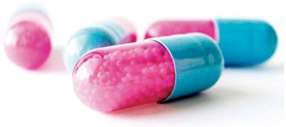

If you take Medicine A, you would live for **4 years** and then die.

If you take Medicine B, you would live for **5 years** and then die.

Would you prefer Medicine A or Medicine B, or are they the same?

Indicate your choice here. You can only choose one option:

◯ **Medicine A**

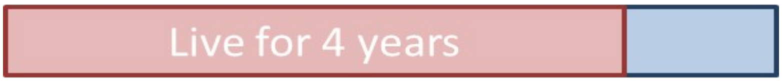

◯ **Medicine B**

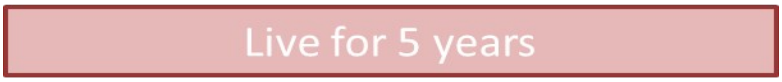

◯ **The Same - Medicine A and Medicine B are the same.**

0% 100%

For peer review only

Repeated section

Remember Medicine A and Medicine B are equally effective and have no risks of a side effect.

The only difference between Medicine A and Medicine B is how you take them.

Medicine A	Medicine B
You take a tablet only once in your life.<br>You will be given the tablet on the day you come in to see your GP.<br>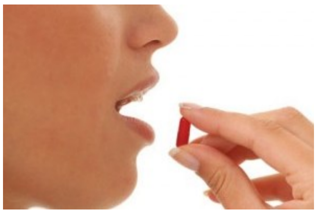	One tablet should be taken once a week on the same day every week.<br>You need to take this medicine for 5 years.<br><br>You need to take the tablet first thing in the morning on an empty stomach with a large glass of tap water.<br>After taking the tablet, you should stay upright for the next 30 minutes.<br>You should not eat anything for at least 30 minutes, including taking any other medicines.<br><br>You need to regularly visit your doctor to have your bone health checked.<br>You need to regularly visit a pharmacy for a new supply.<br>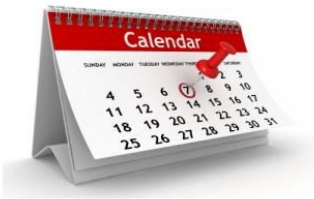 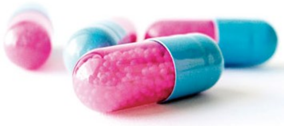

If you take Medicine A, you would live for **4 years and 2 months** and then die.

If you take Medicine B, you would live for **5 years** and then die.

Would you prefer Medicine A or Medicine B, or are they the same?

Indicate your choice here. You can only choose one option:

○ **Medicine A** Live for 4 years 2 months

○ **Medicine B** Live for 5 years

○ **The Same - Medicine A and Medicine B are the same.**

0% 100%

For peer review only

Repeated section

Remember Medicine A and Medicine B are equally effective and have no risks of a side effect.

The only difference between Medicine A and Medicine B is how you take them.

Medicine A	Medicine B
You take a tablet only once in your life. You will be given the tablet on the day you come in to see your GP. 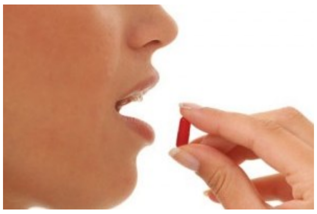	One tablet should be taken once a week on the same day every week. You need to take this medicine for 5 years. <br>You need to take the tablet first thing in the morning on an empty stomach with a large glass of tap water. After taking the tablet, you should stay upright for the next 30 minutes. You should not eat anything for at least 30 minutes, including taking any other medicines. <br>You need to regularly visit your doctor to have your bone health checked. You need to regularly visit a pharmacy for a new supply. 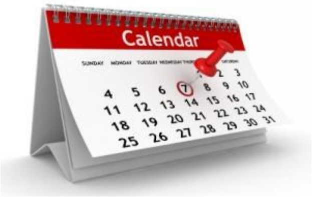 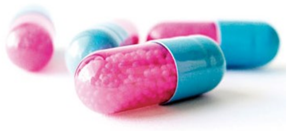

If you take Medicine A, you would live for **4.25 years** (4 years and 3 months) and then die.

If you take Medicine B, you would live for **5 years** and then die.

Would you prefer Medicine A or Medicine B, or are they the same?

Indicate your choice here. You can only choose one option:

◯ **Medicine A** Live for 4 years 3 months

◯ **Medicine B** Live for 5 years

◯ **The Same - Medicine A and Medicine B are the same.**

0% 100%

For peer review only

Repeated section

Remember Medicine A and Medicine B are equally effective and have no risks of a side effect.

The only difference between Medicine A and Medicine B is how you take them.

Medicine A	Medicine B
You take a tablet only once in your life. You will be given the tablet on the day you come in to see your GP. 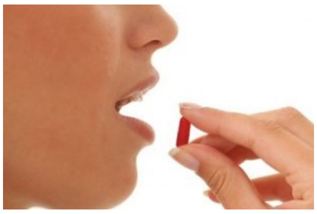	One tablet should be taken once a week on the same day every week. You need to take this medicine for 5 years.<br><br>You need to take the tablet first thing in the morning on an empty stomach with a large glass of tap water. After taking the tablet, you should stay upright for the next 30 minutes. You should not eat anything for at least 30 minutes, including taking any other medicines.<br><br>You need to regularly visit your doctor to have your bone health checked. You need to regularly visit a pharmacy for a new supply. 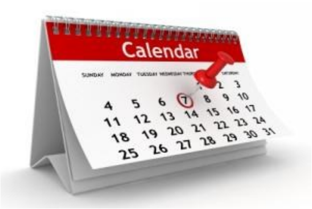 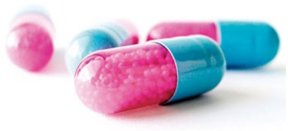

If you take Medicine A, you would live for **4 years 4 months** and then die.

If you take Medicine B, you would live for **5 years** and then die.

Would you prefer Medicine A or Medicine B, or are they the same?

Indicate your choice here. You can only choose one option:

◯ **Medicine A** Live for 4 years 4 months

◯ **Medicine B** Live for 5 years

◯ **The Same - Medicine A and Medicine B are the same.**

0% 100%

For peer review only

Repeated section

Remember Medicine A and Medicine B are equally effective and have no risks of a side effect.

The only difference between Medicine A and Medicine B is how you take them.

**Medicine A**	**Medicine B**
You take a tablet only once in your life.<br>You will be given the tablet on the day you come in to see your GP.<br>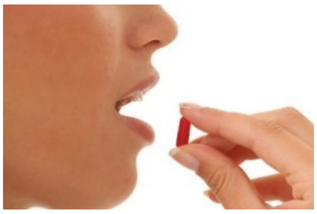	One tablet should be taken once a week on the same day every week.<br>You need to take this medicine for 5 years.<br><br>You need to take the tablet first thing in the morning on an empty stomach with a large glass of tap water.<br>After taking the tablet, you should stay upright for the next 30 minutes.<br>You should not eat anything for at least 30 minutes, including taking any other medicines.<br><br>You need to regularly visit your doctor to have your bone health checked.<br>You need to regularly visit a pharmacy for a new supply.<br>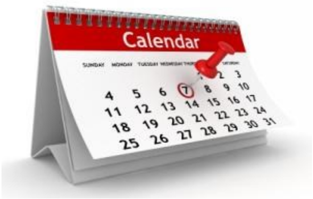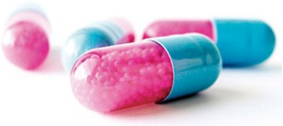

If you take Medicine A, you would live for **4.5 years** (4 years and 6 months) and then die.

If you take Medicine B, you would live for **5 years** and then die.

Would you prefer Medicine A or Medicine B, or are they the same?

Indicate your choice here. You can only choose one option:

◯ **Medicine A** Live for 4 years 6 months

◯ **Medicine B** Live for 5 years

◯ **The Same - Medicine A and Medicine B are the same.**

0% 100%

For peer review only

Repeated section

Remember Medicine A and Medicine B are equally effective and have no risks of a side effect.

The only difference between Medicine A and Medicine B is how you take them.

**Medicine A**	**Medicine B**
You take a tablet only once in your life. You will be given the tablet on the day you come in to see your GP. 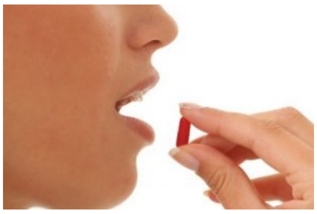	One tablet should be taken once a week on the same day every week. You need to take this medicine for 5 years. <br>You need to take the tablet first thing in the morning on an empty stomach with a large glass of tap water. After taking the tablet, you should stay upright for the next 30 minutes. You should not eat anything for at least 30 minutes, including taking any other medicines. <br>You need to regularly visit your doctor to have your bone health checked. You need to regularly visit a pharmacy for a new supply. 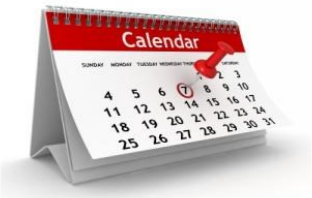 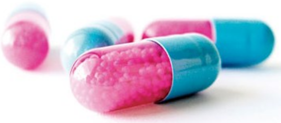

If you take Medicine A, you would live for **4 years and 7 months** and then die.

If you take Medicine B, you would live for **5 years** and then die.

Would you prefer Medicine A or Medicine B, or are they the same?

Indicate your choice here. You can only choose one option:

◯ **Medicine A** Live for 4 years 7 months

◯ **Medicine B** Live for 5 years

◯ **The Same - Medicine A and Medicine B are the same.**

For peer review only

Repeated section

Remember Medicine A and Medicine B are equally effective and have no risks of a side effect.

The only difference between Medicine A and Medicine B is how you take them.

**Medicine A**	**Medicine B**
You take a tablet only once in your life. You will be given the tablet on the day you come in to see your GP. 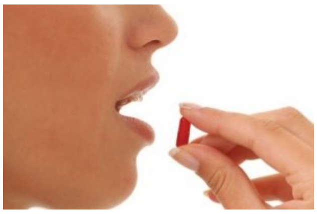	One tablet should be taken once a week on the same day every week. You need to take this medicine for 5 years.<br><br>You need to take the tablet first thing in the morning on an empty stomach with a large glass of tap water. After taking the tablet, you should stay upright for the next 30 minutes. You should not eat anything for at least 30 minutes, including taking any other medicines.<br><br>You need to regularly visit your doctor to have your bone health checked. You need to regularly visit a pharmacy for a new supply. 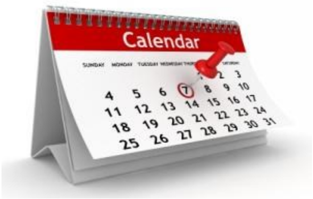 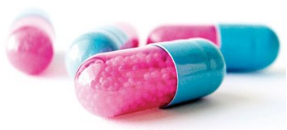

If you take Medicine A, you would live for **4 years and 9 months** and then die.

If you take Medicine B, you would live for **5 years** and then die.

Would you prefer Medicine A or Medicine B, or are they the same?

Indicate your choice here. You can only choose one option:

◯ **Medicine A** Live for 4 years 9 months

◯ **Medicine B** Live for 5 years

◯ **The Same - Medicine A and Medicine B are the same.**

For peer review only

Repeated section

Remember Medicine A and Medicine B are equally effective and have no risks of a side effect.

The only difference between Medicine A and Medicine B is how you take them.

Medicine A	Medicine B
You take a tablet only once in your life. You will be given the tablet on the day you come in to see your GP.<br>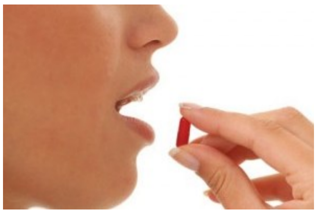	One tablet should be taken once a week on the same day every week. You need to take this medicine for 5 years.<br><br>You need to take the tablet first thing in the morning on an empty stomach with a large glass of tap water. After taking the tablet, you should stay upright for the next 30 minutes. You should not eat anything for at least 30 minutes, including taking any other medicines.<br><br>You need to regularly visit your doctor to have your bone health checked. You need to regularly visit a pharmacy for a new supply.<br>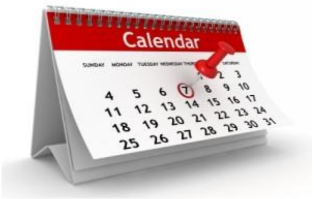 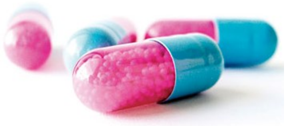

If you take Medicine A, you would live for **2 months less than 5 years** and then die.

If you take Medicine B, you would live for **5 years** and then die.

Would you prefer Medicine A or Medicine B, or are they the same?

Indicate your choice here. You can only choose one option:

○ **Medicine A** Live for (5 years – 2 months)

○ **Medicine B** Live for 5 years

○ **The Same - Medicine A and Medicine B are the same.**

0% 100%

For peer review only

Repeated section

Remember Medicine A and Medicine B are equally effective and have no risks of a side effect.

The only difference between Medicine A and Medicine B is how you take them.

Medicine A	Medicine B
You take a tablet only once in your life. You will be given the tablet on the day you come in to see your GP. 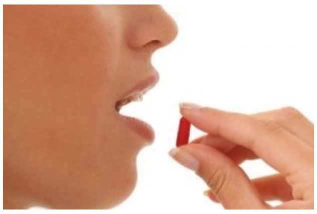	One tablet should be taken once a week on the same day every week. You need to take this medicine for 5 years.<br><br>You need to take the tablet first thing in the morning on an empty stomach with a large glass of tap water. After taking the tablet, you should stay upright for the next 30 minutes. You should not eat anything for at least 30 minutes, including taking any other medicines.<br><br>You need to regularly visit your doctor to have your bone health checked. You need to regularly visit a pharmacy for a new supply.<br>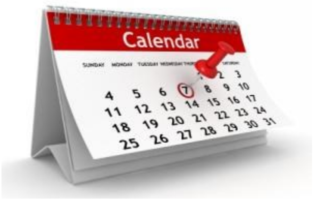 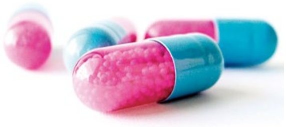

If you take Medicine A, you would live for **1 month less than 5 years** and then die.

If you take Medicine B, you would live for **5 years** and then die.

Would you prefer Medicine A or Medicine B, or are they the same?

Indicate your choice here. You can only choose one option:

◯ **Medicine A** Live for (5 years – 1 month)

◯ **Medicine B** Live for 5 years

◯ **The Same - Medicine A and Medicine B are the same.**

For peer review only

Repeated section

Remember Medicine A and Medicine B are equally effective and have no risks of a side effect.

The only difference between Medicine A and Medicine B is how you take them.

Medicine A	Medicine B
You take a tablet only once in your life. You will be given the tablet on the day you come in to see your GP.<br>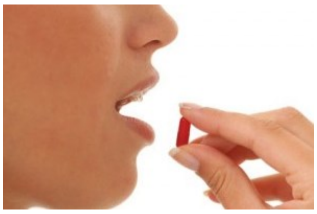	One tablet should be taken once a week on the same day every week.<br>You need to take this medicine for 5 years.<br><br>You need to take the tablet first thing in the morning on an empty stomach with a large glass of tap water.<br>After taking the tablet, you should stay upright for the next 30 minutes.<br>You should not eat anything for at least 30 minutes, including taking any other medicines.<br><br>You need to regularly visit your doctor to have your bone health checked.<br>You need to regularly visit a pharmacy for a new supply.<br>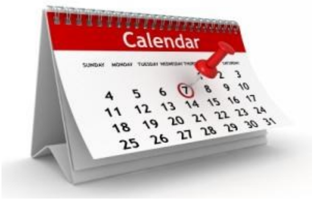 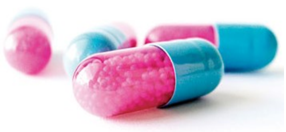

If you take Medicine A, you would live for **2 weeks less than 5 years** and then die.

If you take Medicine B, you would live for **5 years** and then die.

Would you prefer Medicine A or Medicine B, or are they the same?

Indicate your choice here. You can only choose one option:

- ◯ **Medicine A** — Live for (5 years – 2 weeks)
- ◯ **Medicine B** — Live for 5 years
- ◯ **The Same - Medicine A and Medicine B are the same.**

0% 100%

For peer review only

Repeated section

Remember Medicine A and Medicine B are equally effective and have no risks of a side effect.

The only difference between Medicine A and Medicine B is how you take them.

Medicine A	Medicine B
You take a tablet only once in your life. You will be given the tablet on the day you come in to see your GP.<br>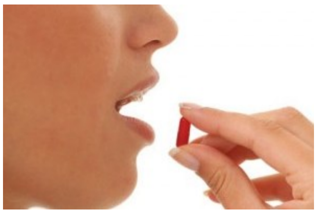	One tablet should be taken once a week on the same day every week.<br>You need to take this medicine for 5 years.<br><br>You need to take the tablet first thing in the morning on an empty stomach with a large glass of tap water.<br>After taking the tablet, you should stay upright for the next 30 minutes.<br>You should not eat anything for at least 30 minutes, including taking any other medicines.<br><br>You need to regularly visit your doctor to have your bone health checked.<br>You need to regularly visit a pharmacy for a new supply.<br>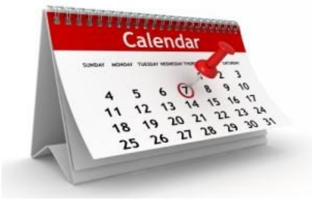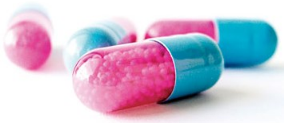

If you take Medicine A, you would live for **1 week less than 5 years** and then die.

If you take Medicine B, you would live for **5 years** and then die.

Would you prefer Medicine A or Medicine B, or are they the same?

Indicate your choice here. You can only choose one option:

○ **Medicine A** Live for (5 years – 1 week)

○ **Medicine B** Live for 5 years

○ **The Same - Medicine A and Medicine B are the same.**

For peer review only

Repeated section

Remember Medicine A and Medicine B are equally effective and have no risks of a side effect.

The only difference between Medicine A and Medicine B is how you take them.

Medicine A	Medicine B
You take a tablet only once in your life. You will be given the tablet on the day you come in to see your GP. 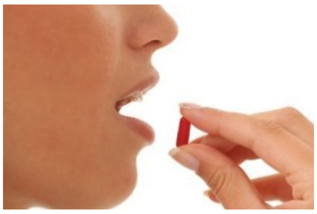	One tablet should be taken once a week on the same day every week. You need to take this medicine for 5 years.<br><br>You need to take the tablet first thing in the morning on an empty stomach with a large glass of tap water. After taking the tablet, you should stay upright for the next 30 minutes. You should not eat anything for at least 30 minutes, including taking any other medicines.<br><br>You need to regularly visit your doctor to have your bone health checked. You need to regularly visit a pharmacy for a new supply. 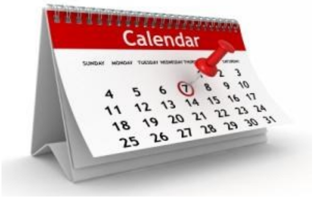 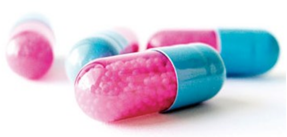

If you take Medicine A, you would live for **5 days less than 5 years** and then die.

If you take Medicine B, you would live for **5 years** and then die.

Would you prefer Medicine A or Medicine B, or are they the same?

Indicate your choice here. You can only choose one option:

◯ **Medicine A** Live for (5 years – 5 days)

◯ **Medicine B** Live for 5 years

◯ **The Same - Medicine A and Medicine B are the same.**

0% 100%

For peer review only

Repeated section

Remember Medicine A and Medicine B are equally effective and have no risks of a side effect.

The only difference between Medicine A and Medicine B is how you take them.

Medicine A	Medicine B
You take a tablet only once in your life. You will be given the tablet on the day you come in to see your GP. 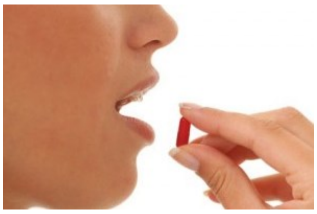	One tablet should be taken once a week on the same day every week.<br>You need to take this medicine for 5 years.<br><br>You need to take the tablet first thing in the morning on an empty stomach with a large glass of tap water.<br>After taking the tablet, you should stay upright for the next 30 minutes.<br>You should not eat anything for at least 30 minutes, including taking any other medicines.<br><br>You need to regularly visit your doctor to have your bone health checked.<br>You need to regularly visit a pharmacy for a new supply.<br>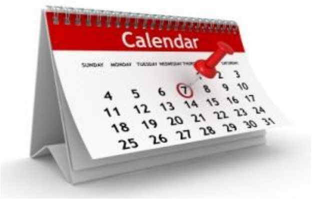 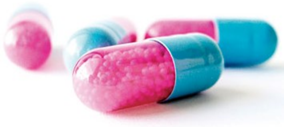

If you take Medicine A, you would live for **3 days less than 5 years** and then die.

If you take Medicine B, you would live for **5 years** and then die.

Would you prefer Medicine A or Medicine B, or are they the same?

Indicate your choice here. You can only choose one option:

◯ **Medicine A** Live for (5 years – 3 days)

◯ **Medicine B** Live for 5 years

◯ **The Same - Medicine A and Medicine B are the same.**

For peer review only

Repeated section

Remember Medicine A and Medicine B are equally effective and have no risks of a side effect.

The only difference between Medicine A and Medicine B is how you take them.

Medicine A	Medicine B
You take a tablet only once in your life. You will be given the tablet on the day you come in to see your GP.<br>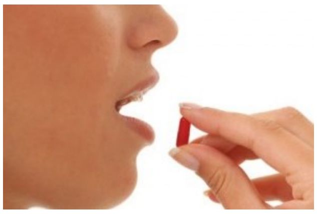	One tablet should be taken once a week on the same day every week.<br>You need to take this medicine for 5 years.<br><br>You need to take the tablet first thing in the morning on an empty stomach with a large glass of tap water.<br>After taking the tablet, you should stay upright for the next 30 minutes.<br>You should not eat anything for at least 30 minutes, including taking any other medicines.<br><br>You need to regularly visit your doctor to have your bone health checked.<br>You need to regularly visit a pharmacy for a new supply.<br>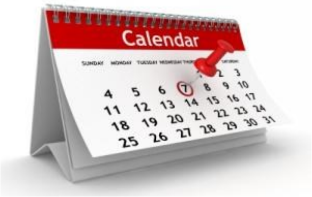 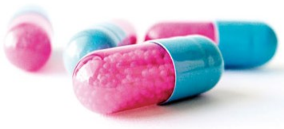

If you take Medicine A, you would live for **1 day less than 5 years** and then die.

If you take Medicine B, you would live for **5 years** and then die.

Would you prefer Medicine A or Medicine B, or are they the same?

Indicate your choice here. You can only choose one option:

◯ **Medicine A** Live for (5 years – 1 day)

◯ **Medicine B** Live for 5 years

◯ **The Same - Medicine A and Medicine B are the same.**

0% 100%

For peer review only

Question 1a.

You prefer living for 2.5 years (2 years and 6 months) on Medicine A, to living for 5 years on Medicine B.

This means **living fewer than 2.5 years on Medicine A** can be the same as living for 5 years on Medicine B.

How many years on Medicine A do you think would be the same as living for 5 years on Medicine B?

(Please give a number in years)

0% 100%

peer review only

Part 2

Question 2

You have been told that you need to take a medicine to reduce the risk of fracture.

You can choose between two different medicines. We have called these Medicine A and Medicine B.

Medicine A is a pill that you only need to take once.

Medicine B is a pill that you need to take every week for 5 years.

Medicine A and Medicine B are equally effective in reducing your risk of having a fracture.
Medicine A and Medicine B will reduce your risk of experiencing a fracture, such as hip fracture, within the next 5 years from **10%** to **6%**.

In other words, in a crowd of **1,000** people in the UK, **100** are likely to have a fracture if they do not take the medicine. This is reduced to **60** people out of 1,000 who do take the medicine.
This information is also illustrated in this picture.

Risk of fracture

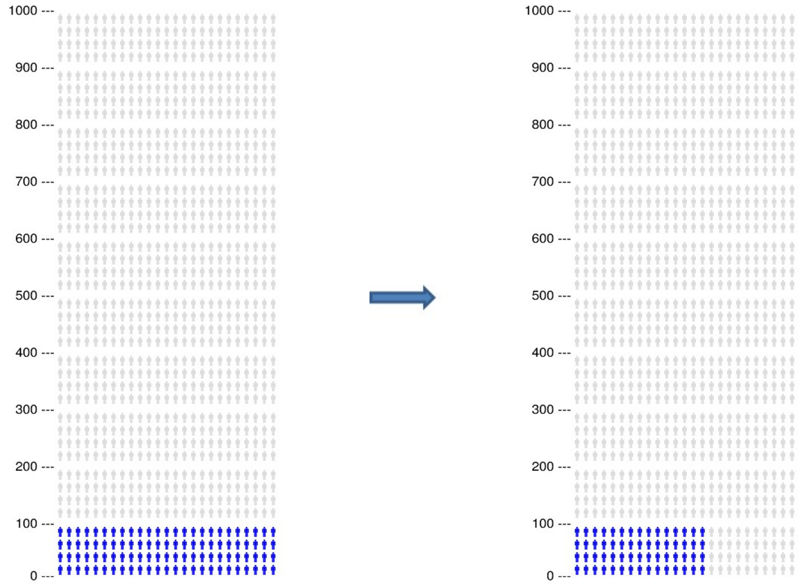

10%
No Treatment

6%
Taking the Medicine

(The figures shaded blue represent people who will have a fracture, such as hip fracture, within the next 5 years)

In this scenario, there is some risk of a minor side effect from Medicine A or Medicine B.

Among every **1,000** people taking Medicine A or Medicine B, **50** people are likely to have a minor side effect such as nausea (feeling sick) and indigestion.

Risk of a minor side effect

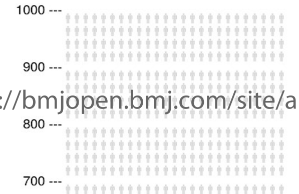

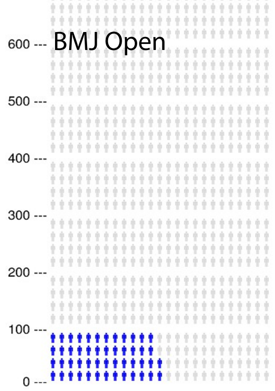

5% of people will have a minor side effect.

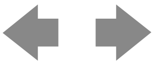

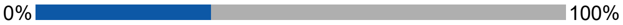

For peer review only

Repeated section

Remember Medicine A and Medicine B are equally effective and have some risk of a minor side effect.

The only difference between Medicine A and Medicine B is how you take them.

Medicine A	Medicine B
You take a tablet only once in your life. You will be given the tablet on the day you come in to see your GP.<br>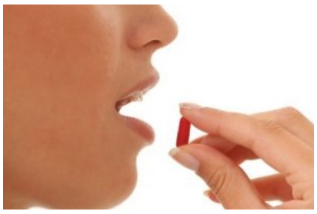	One tablet should be taken once a week on the same day every week.<br>You need to take this medicine for 5 years.<br><br>You need to take the tablet first thing in the morning on an empty stomach with a large glass of tap water.<br>After taking the tablet, you should stay upright for the next 30 minutes.<br>You should not eat anything for at least 30 minutes, including taking any other medicines.<br><br>You need to regularly visit your doctor to have your bone health checked.<br>You need to regularly visit a pharmacy for a new supply.<br>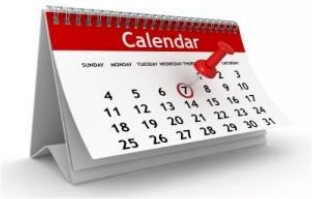 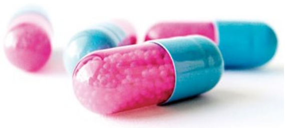

If you take Medicine A, you would live for **2.5 years (2 years and 6 months)** and then die.

If you take Medicine B, you would live for **5 years** and then die.

Would you prefer Medicine A or Medicine B, or are they the same?

Indicate your choice here. You can only choose one option:

○ **Medicine A** Live for 2.5 years

○ **Medicine B** Live for 5 years

○ **The Same - Medicine A and Medicine B are the same.**

0% 100%

For peer review only

Repeated section

Remember Medicine A and Medicine B are equally effective and have some risk of a minor side effect.

The only difference between Medicine A and Medicine B is how you take them.

Medicine A	Medicine B
You take a tablet only once in your life.<br>You will be given the tablet on the day you come in to see your GP.<br>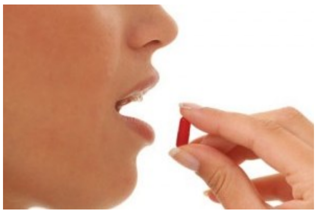	One tablet should be taken once a week on the same day every week.<br>You need to take this medicine for 5 years.<br><br>You need to take the tablet first thing in the morning on an empty stomach with a large glass of tap water.<br>After taking the tablet, you should stay upright for the next 30 minutes.<br>You should not eat anything for at least 30 minutes, including taking any other medicines.<br><br>You need to regularly visit your doctor to have your bone health checked.<br>You need to regularly visit a pharmacy for a new supply.<br>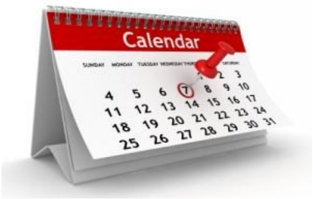 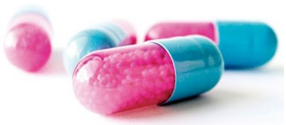

If you take Medicine A, you would live for **3 years** and then die.

If you take Medicine B, you would live for **5 years** and then die.

Would you prefer Medicine A or Medicine B, or are they the same?

Indicate your choice here. You can only choose one option:

◯ **Medicine A**

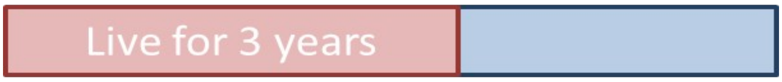

◯ **Medicine B**

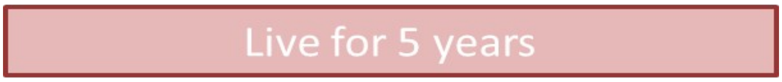

◯ **The Same - Medicine A and Medicine B are the same.**

0% 100%

For peer review only

Repeated section

Remember Medicine A and Medicine B are equally effective and have some risk of a minor side effect.

The only difference between Medicine A and Medicine B is how you take them.

Medicine A	Medicine B
You take a tablet only once in your life. You will be given the tablet on the day you come in to see your GP. 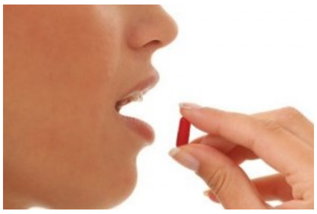	One tablet should be taken once a week on the same day every week. You need to take this medicine for 5 years.<br><br>You need to take the tablet first thing in the morning on an empty stomach with a large glass of tap water. After taking the tablet, you should stay upright for the next 30 minutes. You should not eat anything for at least 30 minutes, including taking any other medicines.<br><br>You need to regularly visit your doctor to have your bone health checked. You need to regularly visit a pharmacy for a new supply.<br>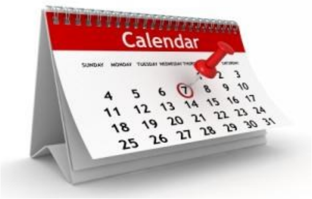 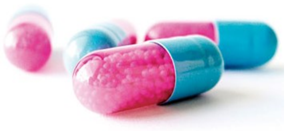

If you take Medicine A, you would live for **3.25 years** (3 years and 3 months) and then die.

If you take Medicine B, you would live for **5 years** and then die.

Would you prefer Medicine A or Medicine B, or are they the same?

Indicate your choice here. You can only choose one option:

◯ **Medicine A** Live for 3 years 3 months

◯ **Medicine B** Live for 5 years

◯ **The Same - Medicine A and Medicine B are the same.**

For peer review only

Repeated section

Remember Medicine A and Medicine B are equally effective and have some risk of a minor side effect.

The only difference between Medicine A and Medicine B is how you take them.

Medicine A	Medicine B
You take a tablet only once in your life. You will be given the tablet on the day you come in to see your GP. 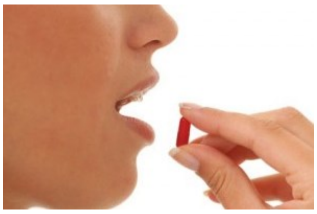	One tablet should be taken once a week on the same day every week. You need to take this medicine for 5 years. You need to take the tablet first thing in the morning on an empty stomach with a large glass of tap water. After taking the tablet, you should stay upright for the next 30 minutes. You should not eat anything for at least 30 minutes, including taking any other medicines. You need to regularly visit your doctor to have your bone health checked. You need to regularly visit a pharmacy for a new supply. 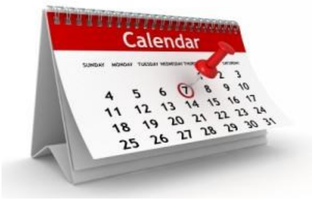 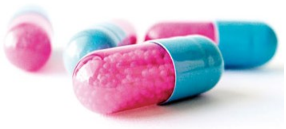

If you take Medicine A, you would live for **3.5 years** (3 years and 6 months) and then die.

If you take Medicine B, you would live for **5 years** and then die.

Would you prefer Medicine A or Medicine B, or are they the same?

Indicate your choice here. You can only choose one option:

◯ **Medicine A** 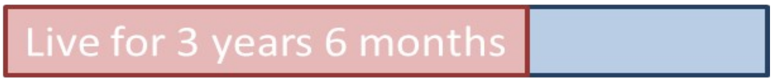

◯ **Medicine B** 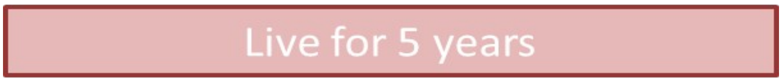

◯ **The Same - Medicine A and Medicine B are the same.**

For peer review only

Repeated section

Remember Medicine A and Medicine B are equally effective and have some risk of a minor side effect.

The only difference between Medicine A and Medicine B is how you take them.

Medicine A	Medicine B
You take a tablet only once in your life. You will be given the tablet on the day you come in to see your GP.<br>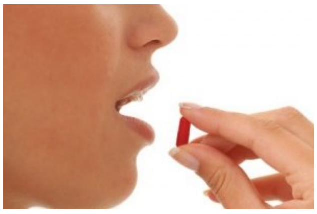	One tablet should be taken once a week on the same day every week.<br>You need to take this medicine for 5 years.<br><br>You need to take the tablet first thing in the morning on an empty stomach with a large glass of tap water.<br>After taking the tablet, you should stay upright for the next 30 minutes.<br>You should not eat anything for at least 30 minutes, including taking any other medicines.<br><br>You need to regularly visit your doctor to have your bone health checked.<br>You need to regularly visit a pharmacy for a new supply.<br>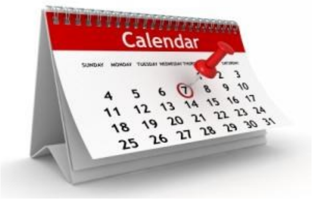 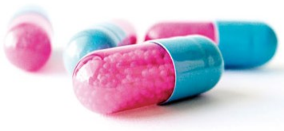

If you take Medicine A, you would live for **3.75 years** (3 years and 9 months) and then die.

If you take Medicine B, you would live for **5 years** and then die.

Would you prefer Medicine A or Medicine B, or are they the same?

Indicate your choice here. You can only choose one option:

◯ **Medicine A** Live for 3 years 9 months

◯ **Medicine B** Live for 5 years

◯ **The Same - Medicine A and Medicine B are the same.**

0% 100%

For peer review only

Repeated section

Remember Medicine A and Medicine B are equally effective and have some risk of a minor side effect.

The only difference between Medicine A and Medicine B is how you take them.

Medicine A	Medicine B
You take a tablet only once in your life.<br>You will be given the tablet on the day you come in to see your GP.<br>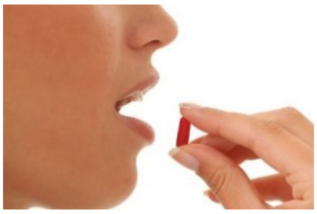	One tablet should be taken once a week on the same day every week.<br>You need to take this medicine for 5 years.<br><br>You need to take the tablet first thing in the morning on an empty stomach with a large glass of tap water.<br>After taking the tablet, you should stay upright for the next 30 minutes.<br>You should not eat anything for at least 30 minutes, including taking any other medicines.<br><br>You need to regularly visit your doctor to have your bone health checked.<br>You need to regularly visit a pharmacy for a new supply.<br>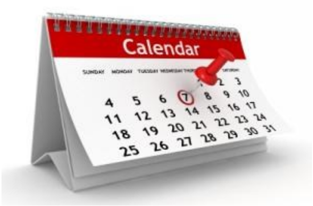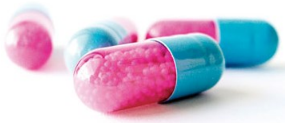

If you take Medicine A, you would live for **3 years and 10 months** and then die.

If you take Medicine B, you would live for **5 years** and then die.

Would you prefer Medicine A or Medicine B, or are they the same?

Indicate your choice here. You can only choose one option:

○ **Medicine A**

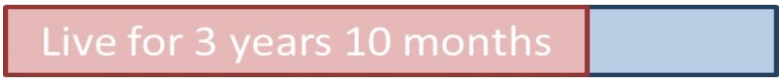

○ **Medicine B**

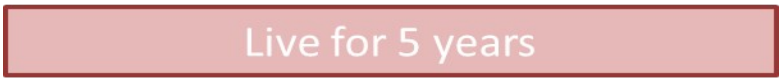

○ **The Same - Medicine A and Medicine B are the same.**

For peer review only

Repeated section

Remember Medicine A and Medicine B are equally effective and have some risk of a minor side effect.

The only difference between Medicine A and Medicine B is how you take them.

Medicine A	Medicine B
You take a tablet only once in your life.<br>You will be given the tablet on the day you come in to see your GP.<br>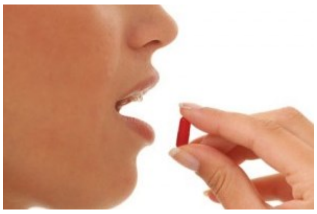	One tablet should be taken once a week on the same day every week.<br>You need to take this medicine for 5 years.<br><br>You need to take the tablet first thing in the morning on an empty stomach with a large glass of tap water.<br>After taking the tablet, you should stay upright for the next 30 minutes.<br>You should not eat anything for at least 30 minutes, including taking any other medicines.<br><br>You need to regularly visit your doctor to have your bone health checked.<br>You need to regularly visit a pharmacy for a new supply.<br>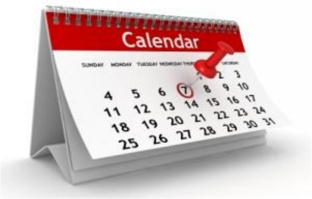 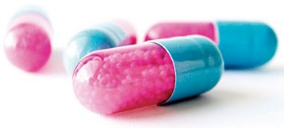

If you take Medicine A, you would live for **4 years** and then die.

If you take Medicine B, you would live for **5 years** and then die.

Would you prefer Medicine A or Medicine B, or are they the same?

Indicate your choice here. You can only choose one option:

◯ **Medicine A**

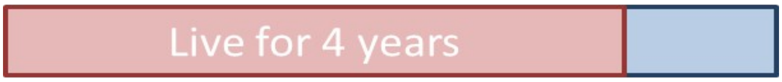

◯ **Medicine B**

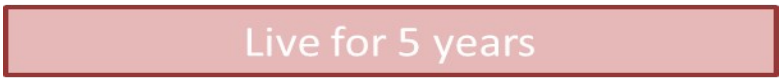

◯ **The Same - Medicine A and Medicine B are the same.**

0% 100%

For peer review only

Repeated section

Remember Medicine A and Medicine B are equally effective and have some risk of a minor side effect.

The only difference between Medicine A and Medicine B is how you take them.

Medicine A	Medicine B
You take a tablet only once in your life.<br>You will be given the tablet on the day you come in to see your GP.<br>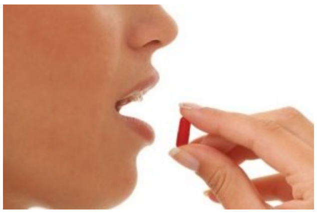	One tablet should be taken once a week on the same day every week.<br>You need to take this medicine for 5 years.<br><br>You need to take the tablet first thing in the morning on an empty stomach with a large glass of tap water.<br>After taking the tablet, you should stay upright for the next 30 minutes.<br>You should not eat anything for at least 30 minutes, including taking any other medicines.<br><br>You need to regularly visit your doctor to have your bone health checked.<br>You need to regularly visit a pharmacy for a new supply.<br>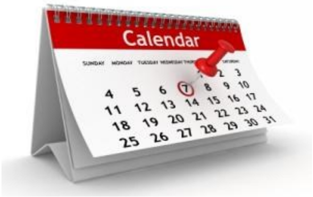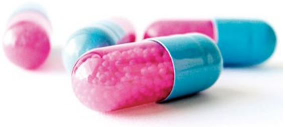

If you take Medicine A, you would live for **4 years and 2 months** and then die.

If you take Medicine B, you would live for **5 years** and then die.

Would you prefer Medicine A or Medicine B, or are they the same?

Indicate your choice here. You can only choose one option:

◯ **Medicine A** Live for 4 years 2 months

◯ **Medicine B** Live for 5 years

◯ **The Same - Medicine A and Medicine B are the same.**

0% 100%

For peer review only

Repeated section

Remember Medicine A and Medicine B are equally effective and have some risk of a minor side effect.

The only difference between Medicine A and Medicine B is how you take them.

Medicine A	Medicine B
You take a tablet only once in your life. You will be given the tablet on the day you come in to see your GP. 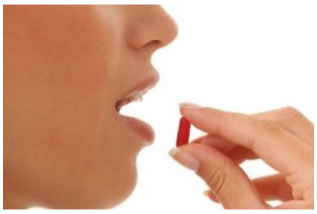	One tablet should be taken once a week on the same day every week. You need to take this medicine for 5 years. You need to take the tablet first thing in the morning on an empty stomach with a large glass of tap water. After taking the tablet, you should stay upright for the next 30 minutes. You should not eat anything for at least 30 minutes, including taking any other medicines. You need to regularly visit your doctor to have your bone health checked. You need to regularly visit a pharmacy for a new supply. 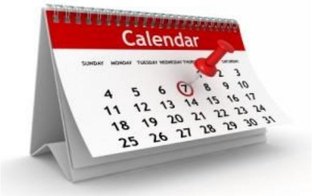 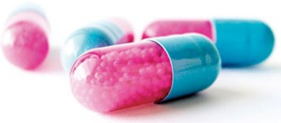

If you take Medicine A, you would live for **4.25 years** (4 years and 3 months) and then die.

If you take Medicine B, you would live for **5 years** and then die.

Would you prefer Medicine A or Medicine B, or are they the same?

Indicate your choice here. You can only choose one option:

◯ **Medicine A** Live for 4 years 3 months

◯ **Medicine B** Live for 5 years

◯ **The Same - Medicine A and Medicine B are the same.**

0% 100%

For peer review only

Repeated section

Remember Medicine A and Medicine B are equally effective and have some risk of a minor side effect.

The only difference between Medicine A and Medicine B is how you take them.

**Medicine A**	**Medicine B**
You take a tablet only once in your life.<br>You will be given the tablet on the day you come in to see your GP.<br>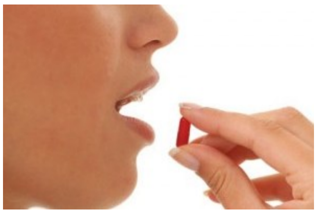	One tablet should be taken once a week on the same day every week.<br>You need to take this medicine for 5 years.<br><br>You need to take the tablet first thing in the morning on an empty stomach with a large glass of tap water.<br>After taking the tablet, you should stay upright for the next 30 minutes.<br>You should not eat anything for at least 30 minutes, including taking any other medicines.<br><br>You need to regularly visit your doctor to have your bone health checked.<br>You need to regularly visit a pharmacy for a new supply.<br>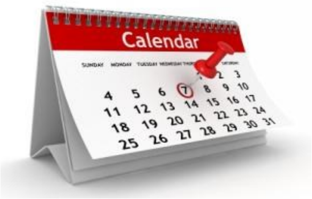 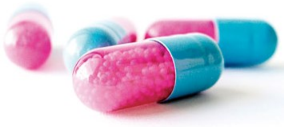

If you take Medicine A, you would live for **4 years 4 months** and then die.

If you take Medicine B, you would live for **5 years** and then die.

Would you prefer Medicine A or Medicine B, or are they the same?

Indicate your choice here. You can only choose one option:

◯ **Medicine A** Live for 4 years 4 months

◯ **Medicine B** Live for 5 years

◯ **The Same - Medicine A and Medicine B are the same.**

0% 100%

For peer review only

Repeated section

Remember Medicine A and Medicine B are equally effective and have no risks of a minor side effect.

The only difference between Medicine A and Medicine B is how you take them.

Medicine A	Medicine B
You take a tablet only once in your life. You will be given the tablet on the day you come in to see your GP. 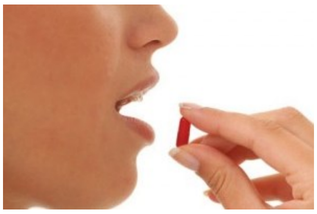	One tablet should be taken once a week on the same day every week. You need to take this medicine for 5 years.<br><br>You need to take the tablet first thing in the morning on an empty stomach with a large glass of tap water. After taking the tablet, you should stay upright for the next 30 minutes. You should not eat anything for at least 30 minutes, including taking any other medicines.<br><br>You need to regularly visit your doctor to have your bone health checked. You need to regularly visit a pharmacy for a new supply. 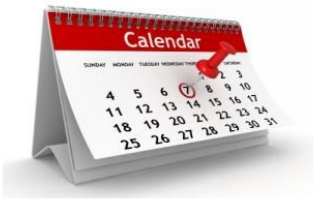 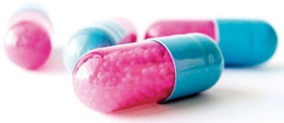

If you take Medicine A, you would live for **4.5 years** (4 years and 6 months) and then die.

If you take Medicine B, you would live for **5 years** and then die.

Would you prefer Medicine A or Medicine B, or are they the same?

Indicate your choice here. You can only choose one option:

○ **Medicine A** Live for 4 years 6 months

○ **Medicine B** Live for 5 years

○ **The Same - Medicine A and Medicine B are the same.**

0% 100%

For peer review only

Repeated section

Remember Medicine A and Medicine B are equally effective and have some risk of a minor side effect.

The only difference between Medicine A and Medicine B is how you take them.

Medicine A	Medicine B
You take a tablet only once in your life. You will be given the tablet on the day you come in to see your GP.<br>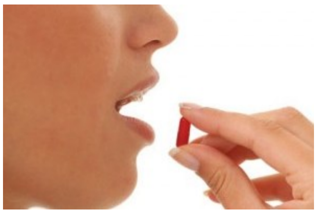	One tablet should be taken once a week on the same day every week.<br>You need to take this medicine for 5 years.<br><br>You need to take the tablet first thing in the morning on an empty stomach with a large glass of tap water.<br>After taking the tablet, you should stay upright for the next 30 minutes.<br>You should not eat anything for at least 30 minutes, including taking any other medicines.<br><br>You need to regularly visit your doctor to have your bone health checked.<br>You need to regularly visit a pharmacy for a new supply.<br>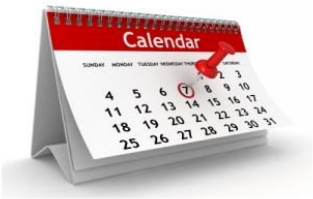 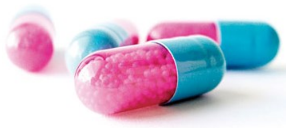

If you take Medicine A, you would live for **4 years and 7 months** and then die.

If you take Medicine B, you would live for **5 years** and then die.

Would you prefer Medicine A or Medicine B, or are they the same?

Indicate your choice here. You can only choose one option:

◯ **Medicine A** Live for 4 years 7 months

◯ **Medicine B** Live for 5 years

◯ **The Same - Medicine A and Medicine B are the same.**

0% 100%

For peer review only

Repeated section

Remember Medicine A and Medicine B are equally effective and have some risk of a minor side effect.

The only difference between Medicine A and Medicine B is how you take them.

**Medicine A**	**Medicine B**
You take a tablet only once in your life. You will be given the tablet on the day you come in to see your GP. 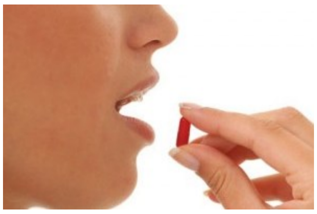	One tablet should be taken once a week on the same day every week. You need to take this medicine for 5 years. You need to take the tablet first thing in the morning on an empty stomach with a large glass of tap water. After taking the tablet, you should stay upright for the next 30 minutes. You should not eat anything for at least 30 minutes, including taking any other medicines. You need to regularly visit your doctor to have your bone health checked. You need to regularly visit a pharmacy for a new supply. 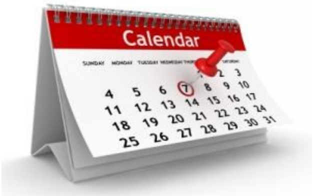 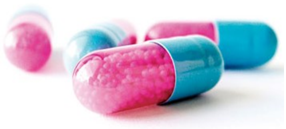

If you take Medicine A, you would live for **4 years and 9 months** and then die.

If you take Medicine B, you would live for **5 years** and then die.

Would you prefer Medicine A or Medicine B, or are they the same?

Indicate your choice here. You can only choose one option:

◯ **Medicine A** 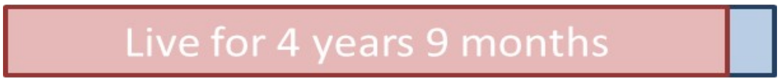

◯ **Medicine B** 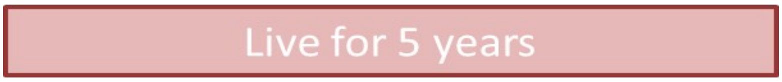

◯ **The Same - Medicine A and Medicine B are the same.**

0% 100%

For peer review only

Repeated section

Remember Medicine A and Medicine B are equally effective and have some risk of a minor side effect.

The only difference between Medicine A and Medicine B is how you take them.

Medicine A	Medicine B
You take a tablet only once in your life.<br>You will be given the tablet on the day you come in to see your GP.<br>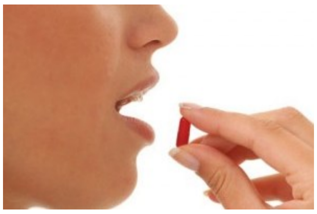	One tablet should be taken once a week on the same day every week.<br>You need to take this medicine for 5 years.<br><br>You need to take the tablet first thing in the morning on an empty stomach with a large glass of tap water.<br>After taking the tablet, you should stay upright for the next 30 minutes.<br>You should not eat anything for at least 30 minutes, including taking any other medicines.<br><br>You need to regularly visit your doctor to have your bone health checked.<br>You need to regularly visit a pharmacy for a new supply.<br>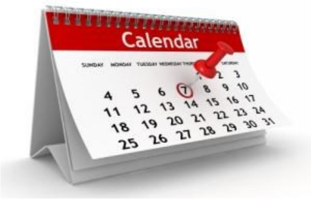 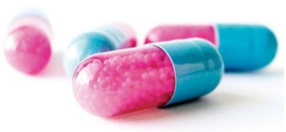

If you take Medicine A, you would live for **2 months less than 5 years** and then die.

If you take Medicine B, you would live for **5 years** and then die.

Would you prefer Medicine A or Medicine B, or are they the same?

Indicate your choice here. You can only choose one option:

◯ **Medicine A** Live for (5 years – 2 months)

◯ **Medicine B** Live for 5 years

◯ **The Same - Medicine A and Medicine B are the same.**

0% 100%

For peer review only

Repeated section

Remember Medicine A and Medicine B are equally effective and have some risk of a minor side effect.

The only difference between Medicine A and Medicine B is how you take them.

Medicine A	Medicine B
You take a tablet only once in your life.<br>You will be given the tablet on the day you come in to see your GP.<br>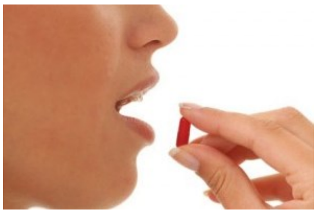	One tablet should be taken once a week on the same day every week.<br>You need to take this medicine for 5 years.<br><br>You need to take the tablet first thing in the morning on an empty stomach with a large glass of tap water.<br>After taking the tablet, you should stay upright for the next 30 minutes.<br>You should not eat anything for at least 30 minutes, including taking any other medicines.<br><br>You need to regularly visit your doctor to have your bone health checked.<br>You need to regularly visit a pharmacy for a new supply.<br>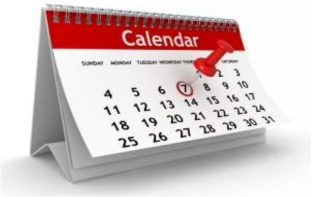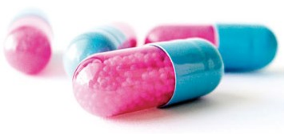

If you take Medicine A, you would live for **1 month less than 5 years** and then die.

If you take Medicine B, you would live for **5 years** and then die.

Would you prefer Medicine A or Medicine B, or are they the same?

Indicate your choice here. You can only choose one option:

◯ **Medicine A** Live for (5 years – 1 month)

◯ **Medicine B** Live for 5 years

◯ **The Same - Medicine A and Medicine B are the same.**

0% 100%

For peer review only

Repeated section

Remember Medicine A and Medicine B are equally effective and have some risk of a minor side effect.

The only difference between Medicine A and Medicine B is how you take them.

Medicine A	Medicine B
You take a tablet only once in your life. You will be given the tablet on the day you come in to see your GP.<br>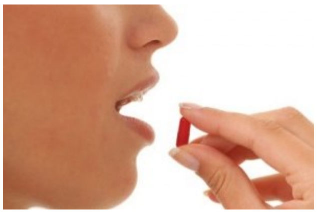	One tablet should be taken once a week on the same day every week.<br>You need to take this medicine for 5 years.<br><br>You need to take the tablet first thing in the morning on an empty stomach with a large glass of tap water.<br>After taking the tablet, you should stay upright for the next 30 minutes.<br>You should not eat anything for at least 30 minutes, including taking any other medicines.<br><br>You need to regularly visit your doctor to have your bone health checked.<br>You need to regularly visit a pharmacy for a new supply.<br>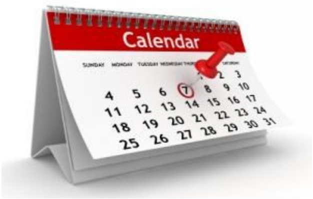 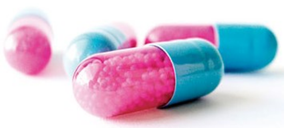

If you take Medicine A, you would live for **2 weeks less than 5 years** and then die.

If you take Medicine B, you would live for **5 years** and then die.

Would you prefer Medicine A or Medicine B, or are they the same?

Indicate your choice here. You can only choose one option:

◯ **Medicine A** Live for (5 years – 2 weeks)

◯ **Medicine B** Live for 5 years

◯ **The Same - Medicine A and Medicine B are the same.**

0% 100%

For peer review only

Repeated section

Remember Medicine A and Medicine B are equally effective and have some risk of a minor side effect.

The only difference between Medicine A and Medicine B is how you take them.

**Medicine A**	**Medicine B**
You take a tablet only once in your life. You will be given the tablet on the day you come in to see your GP. 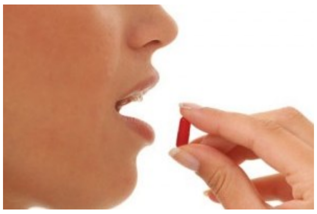	One tablet should be taken once a week on the same day every week. You need to take this medicine for 5 years.<br><br>You need to take the tablet first thing in the morning on an empty stomach with a large glass of tap water. After taking the tablet, you should stay upright for the next 30 minutes. You should not eat anything for at least 30 minutes, including taking any other medicines.<br><br>You need to regularly visit your doctor to have your bone health checked. You need to regularly visit a pharmacy for a new supply. 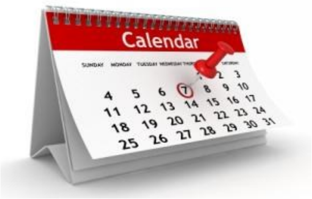 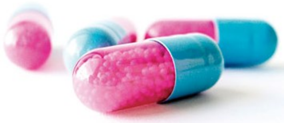

If you take Medicine A, you would live for **1 week less than 5 years** and then die.

If you take Medicine B, you would live for **5 years** and then die.

Would you prefer Medicine A or Medicine B, or are they the same?

Indicate your choice here. You can only choose one option:

○ **Medicine A** Live for (5 years – 1 week)

○ **Medicine B** Live for 5 years

○ **The Same - Medicine A and Medicine B are the same.**

0% 100%

For peer review only

Repeated section

Remember Medicine A and Medicine B are equally effective and have some risk of a minor side effect.

The only difference between Medicine A and Medicine B is how you take them.

Medicine A	Medicine B
You take a tablet only once in your life.<br>You will be given the tablet on the day you come in to see your GP.<br>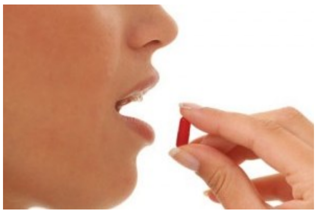	One tablet should be taken once a week on the same day every week.<br>You need to take this medicine for 5 years.<br><br>You need to take the tablet first thing in the morning on an empty stomach with a large glass of tap water.<br>After taking the tablet, you should stay upright for the next 30 minutes.<br>You should not eat anything for at least 30 minutes, including taking any other medicines.<br><br>You need to regularly visit your doctor to have your bone health checked.<br>You need to regularly visit a pharmacy for a new supply.<br>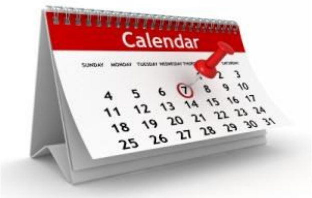 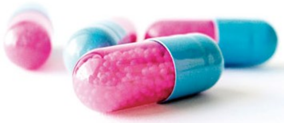

If you take Medicine A, you would live for **5 days less than 5 years** and then die.

If you take Medicine B, you would live for **5 years** and then die.

Would you prefer Medicine A or Medicine B, or are they the same?

Indicate your choice here. You can only choose one option:

○ **Medicine A** Live for (5 years – 5 days)

○ **Medicine B** Live for 5 years

○ **The Same - Medicine A and Medicine B are the same.**

0% 100%

For peer review only

Repeated section

Remember Medicine A and Medicine B are equally effective and have some risk of a minor side effect.

The only difference between Medicine A and Medicine B is how you take them.

Medicine A	Medicine B
You take a tablet only once in your life. You will be given the tablet on the day you come in to see your GP. 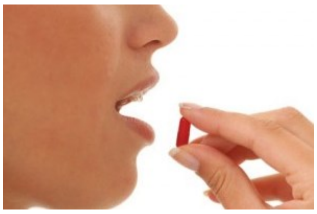	One tablet should be taken once a week on the same day every week.<br>You need to take this medicine for 5 years.<br><br>You need to take the tablet first thing in the morning on an empty stomach with a large glass of tap water.<br>After taking the tablet, you should stay upright for the next 30 minutes.<br>You should not eat anything for at least 30 minutes, including taking any other medicines.<br><br>You need to regularly visit your doctor to have your bone health checked.<br>You need to regularly visit a pharmacy for a new supply.<br>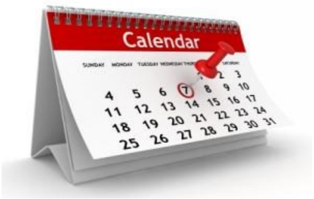 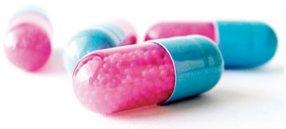

If you take Medicine A, you would live for **3 days less than 5 years** and then die.

If you take Medicine B, you would live for **5 years** and then die.

Would you prefer Medicine A or Medicine B, or are they the same?

Indicate your choice here. You can only choose one option:

◯ **Medicine A** Live for (5 years – 3 days)

◯ **Medicine B** Live for 5 years

◯ **The Same - Medicine A and Medicine B are the same.**

0% 100%

For peer review only

Repeated section

Remember Medicine A and Medicine B are equally effective and have some risk of a minor side effect.

The only difference between Medicine A and Medicine B is how you take them.

Medicine A	Medicine B
You take a tablet only once in your life.<br>You will be given the tablet on the day you come in to see your GP.<br>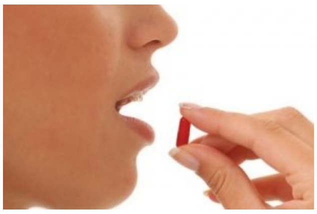	One tablet should be taken once a week on the same day every week.<br>You need to take this medicine for 5 years.<br><br>You need to take the tablet first thing in the morning on an empty stomach with a large glass of tap water.<br>After taking the tablet, you should stay upright for the next 30 minutes.<br>You should not eat anything for at least 30 minutes, including taking any other medicines.<br><br>You need to regularly visit your doctor to have your bone health checked.<br>You need to regularly visit a pharmacy for a new supply.<br>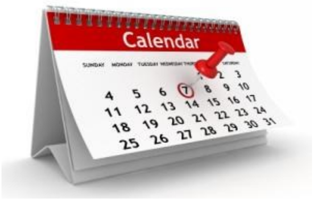 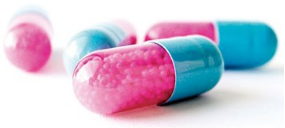

If you take Medicine A, you would live for **1 day less than 5 years** and then die.

If you take Medicine B, you would live for **5 years** and then die.

Would you prefer Medicine A or Medicine B, or are they the same?

Indicate your choice here. You can only choose one option:

◯ **Medicine A** Live for (5 years – 1 day)

◯ **Medicine B** Live for 5 years

◯ **The Same - Medicine A and Medicine B are the same.**

0% 100%

For peer review only

Question 2a.

You prefer living for 2.5 years (2 years and 6 months) on Medicine A, to living for 5 years on Medicine B.

This means **living fewer than 2.5 years on Medicine A** can be the same as living for 5 years on Medicine B.

How many years on Medicine A do you think would be the same as living for 5 years on Medicine B?

(Please give a number in years)

0% 100%

peer review only

Part 2

Question 3

You have been told that you need to take a medicine to reduce the risk of fracture.

You can choose between two different medicines. We have called these Medicine A and Medicine B.

Medicine A is a pill that you only need to take once.

Medicine B is a pill that you need to take every week for 5 years.

Medicine A and Medicine B are equally effective in reducing your risk of having a fracture.
Medicine A and Medicine B will reduce your risk of experiencing a fracture, such as hip fracture, within the next 5 years from **10%** to **6%**.

In other words, in a crowd of **1,000** people in the UK, **100** are likely to have a fracture if they do not take the medicine. This is reduced to **60** people out of 1,000 who do take the medicine.
This information is also illustrated in this picture.

Risk of fracture

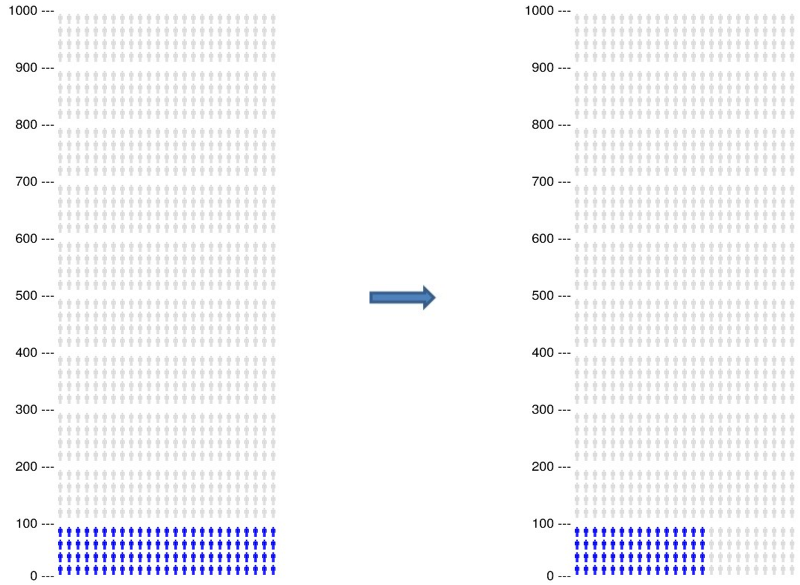

10%
No Treatment

6%
Taking the Medicine

(The figures shaded blue represent people who will have a fracture, such as hip fracture, within the next 5 years)

In this scenario, there is some risk of a severe side effect from Medicine A or Medicine B.

Among every 1,000 people taking Medicine A or Medicine B, **3** people are likely to have a severe side effect such as severe loss or destruction of the bone in the jaw.

Risk of a severe side effect

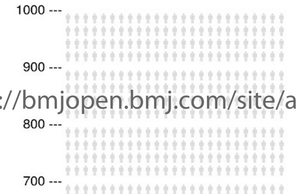

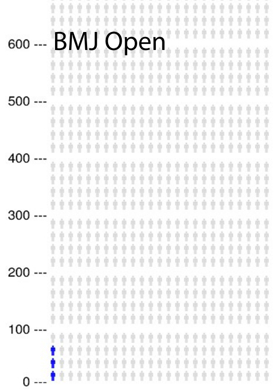

0.3% of people will have a severe side effect.

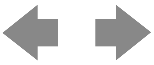

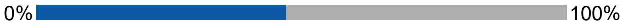

For peer review only

Repeated section

Remember Medicine A and Medicine B are equally effective and have some risk of a severe side effect.

The only difference between Medicine A and Medicine B is how you take them.

Medicine A	Medicine B
You take a tablet only once in your life. You will be given the tablet on the day you come in to see your GP. 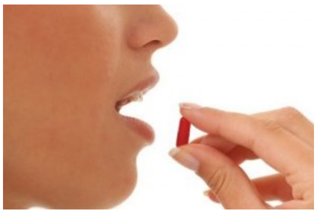	One tablet should be taken once a week on the same day every week.<br>You need to take this medicine for 5 years.<br><br>You need to take the tablet first thing in the morning on an empty stomach with a large glass of tap water.<br>After taking the tablet, you should stay upright for the next 30 minutes.<br>You should not eat anything for at least 30 minutes, including taking any other medicines.<br><br>You need to regularly visit your doctor to have your bone health checked.<br>You need to regularly visit a pharmacy for a new supply.<br>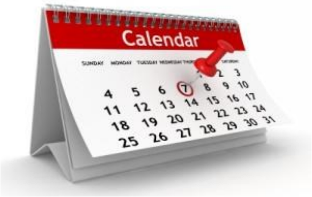 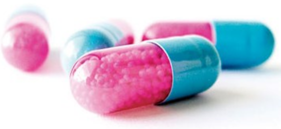

If you take Medicine A, you would live for **2.5 years (2 years and 6 months)** and then die.

If you take Medicine B, you would live for **5 years** and then die.

Would you prefer Medicine A or Medicine B, or are they the same?

Indicate your choice here. You can only choose one option:

◯ **Medicine A** 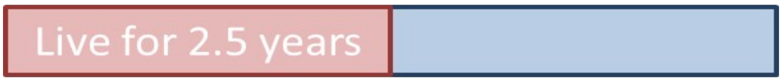

◯ **Medicine B** 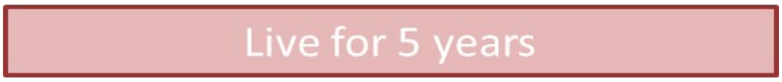

◯ **The Same - Medicine A and Medicine B are the same.**

For peer review only

Repeated section

Remember Medicine A and Medicine B are equally effective and have some risk of a severe side effect.

The only difference between Medicine A and Medicine B is how you take them.

Medicine A	Medicine B
You take a tablet only once in your life.<br>You will be given the tablet on the day you come in to see your GP.<br>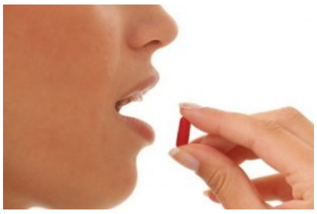	One tablet should be taken once a week on the same day every week.<br>You need to take this medicine for 5 years.<br><br>You need to take the tablet first thing in the morning on an empty stomach with a large glass of tap water.<br>After taking the tablet, you should stay upright for the next 30 minutes.<br>You should not eat anything for at least 30 minutes, including taking any other medicines.<br><br>You need to regularly visit your doctor to have your bone health checked.<br>You need to regularly visit a pharmacy for a new supply.<br>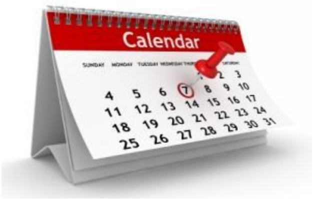 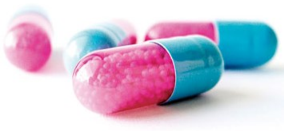

If you take Medicine A, you would live for **3 years** and then die.

If you take Medicine B, you would live for **5 years** and then die.

Would you prefer Medicine A or Medicine B, or are they the same?

Indicate your choice here. You can only choose one option:

○ **Medicine A**

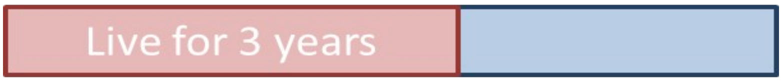

○ **Medicine B**

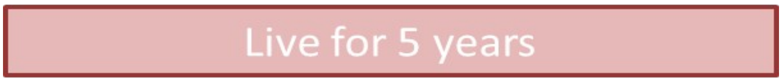

○ **The Same - Medicine A and Medicine B are the same.**

For peer review only

Repeated section

Remember Medicine A and Medicine B are equally effective and have some risk of a severe side effect.

The only difference between Medicine A and Medicine B is how you take them.

Medicine A	Medicine B
You take a tablet only once in your life. You will be given the tablet on the day you come in to see your GP.<br>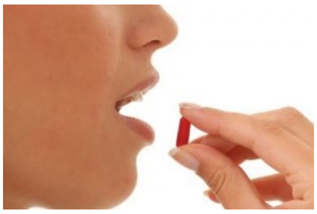	One tablet should be taken once a week on the same day every week.<br>You need to take this medicine for 5 years.<br><br>You need to take the tablet first thing in the morning on an empty stomach with a large glass of tap water.<br>After taking the tablet, you should stay upright for the next 30 minutes.<br>You should not eat anything for at least 30 minutes, including taking any other medicines.<br><br>You need to regularly visit your doctor to have your bone health checked.<br>You need to regularly visit a pharmacy for a new supply.<br>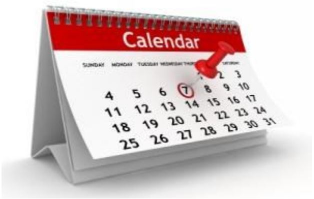 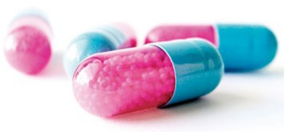

If you take Medicine A, you would live for **3.25 years** (3 years and 3 months) and then die.

If you take Medicine B, you would live for **5 years** and then die.

Would you prefer Medicine A or Medicine B, or are they the same?

Indicate your choice here. You can only choose one option:

○ **Medicine A** — Live for 3 years 3 months

○ **Medicine B** — Live for 5 years

○ **The Same - Medicine A and Medicine B are the same.**

0% 100%

For peer review only

Repeated section

Remember Medicine A and Medicine B are equally effective and have some risk of a severe side effect.

The only difference between Medicine A and Medicine B is how you take them.

Medicine A	Medicine B
You take a tablet only once in your life.<br>You will be given the tablet on the day you come in to see your GP.<br>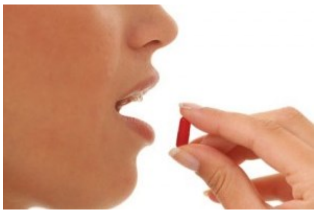	One tablet should be taken once a week on the same day every week.<br>You need to take this medicine for 5 years.<br><br>You need to take the tablet first thing in the morning on an empty stomach with a large glass of tap water.<br>After taking the tablet, you should stay upright for the next 30 minutes.<br>You should not eat anything for at least 30 minutes, including taking any other medicines.<br><br>You need to regularly visit your doctor to have your bone health checked.<br>You need to regularly visit a pharmacy for a new supply.<br>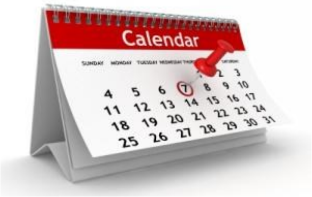 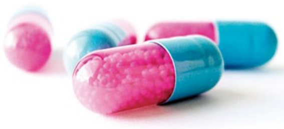

If you take Medicine A, you would live for **3.5 years** (3 years and 6 months) and then die.

If you take Medicine B, you would live for **5 years** and then die.

Would you prefer Medicine A or Medicine B, or are they the same?

Indicate your choice here. You can only choose one option:

◯ **Medicine A** Live for 3 years 6 months

◯ **Medicine B** Live for 5 years

◯ **The Same - Medicine A and Medicine B are the same.**

0% 100%

For peer review only

Repeated section

Remember Medicine A and Medicine B are equally effective and have no risks of a side effect.

The only difference between Medicine A and Medicine B is how you take them.

Medicine A	Medicine B
You take a tablet only once in your life. You will be given the tablet on the day you come in to see your GP.<br>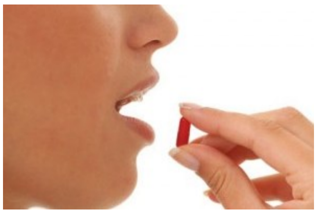	One tablet should be taken once a week on the same day every week.<br>You need to take this medicine for 5 years.<br><br>You need to take the tablet first thing in the morning on an empty stomach with a large glass of tap water.<br>After taking the tablet, you should stay upright for the next 30 minutes.<br>You should not eat anything for at least 30 minutes, including taking any other medicines.<br><br>You need to regularly visit your doctor to have your bone health checked.<br>You need to regularly visit a pharmacy for a new supply.<br>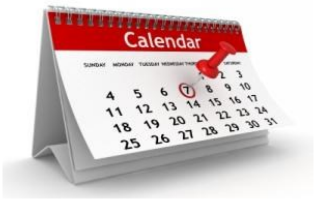 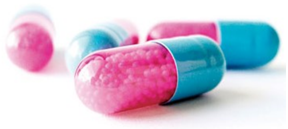

If you take Medicine A, you would live for **3.75 years** (3 years and 9 months) and then die.

If you take Medicine B, you would live for **5 years** and then die.

Would you prefer Medicine A or Medicine B, or are they the same?

Indicate your choice here. You can only choose one option:

◯ **Medicine A** Live for 3 years 9 months

◯ **Medicine B** Live for 5 years

◯ **The Same - Medicine A and Medicine B are the same.**

0% 100%

For peer review only

Repeated section

Remember Medicine A and Medicine B are equally effective and have some risk of a severe side effect.

The only difference between Medicine A and Medicine B is how you take them.

**Medicine A**	**Medicine B**
You take a tablet only once in your life.<br>You will be given the tablet on the day you come in to see your GP.<br>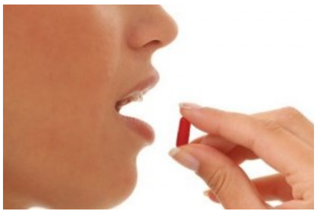	One tablet should be taken once a week on the same day every week.<br>You need to take this medicine for 5 years.<br><br>You need to take the tablet first thing in the morning on an empty stomach with a large glass of tap water.<br>After taking the tablet, you should stay upright for the next 30 minutes.<br>You should not eat anything for at least 30 minutes, including taking any other medicines.<br><br>You need to regularly visit your doctor to have your bone health checked.<br>You need to regularly visit a pharmacy for a new supply.<br>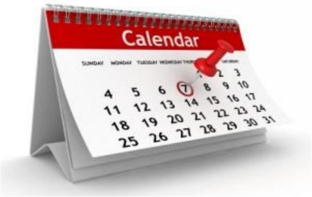 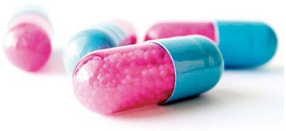

If you take Medicine A, you would live for **3 years and 10 months** and then die.

If you take Medicine B, you would live for **5 years** and then die.

Would you prefer Medicine A or Medicine B, or are they the same?

Indicate your choice here. You can only choose one option:

◯ **Medicine A** Live for 3 years 10 months

◯ **Medicine B** Live for 5 years

◯ **The Same - Medicine A and Medicine B are the same.**

For peer review only

Repeated section

Remember Medicine A and Medicine B are equally effective and have some risk of a severe side effect.

The only difference between Medicine A and Medicine B is how you take them.

Medicine A	Medicine B
You take a tablet only once in your life. You will be given the tablet on the day you come in to see your GP. 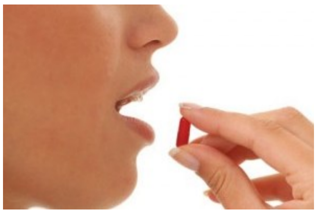	One tablet should be taken once a week on the same day every week. You need to take this medicine for 5 years.<br><br>You need to take the tablet first thing in the morning on an empty stomach with a large glass of tap water. After taking the tablet, you should stay upright for the next 30 minutes. You should not eat anything for at least 30 minutes, including taking any other medicines.<br><br>You need to regularly visit your doctor to have your bone health checked. You need to regularly visit a pharmacy for a new supply. 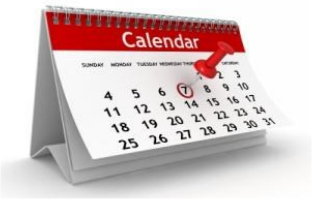 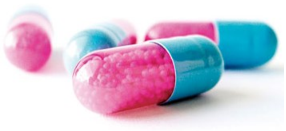

If you take Medicine A, you would live for **4 years** and then die.

If you take Medicine B, you would live for **5 years** and then die.

Would you prefer Medicine A or Medicine B, or are they the same?

Indicate your choice here. You can only choose one option:

◯ **Medicine A** Live for 4 years

◯ **Medicine B** Live for 5 years

◯ **The Same - Medicine A and Medicine B are the same.**

0% 100%

For peer review only

Repeated section

Remember Medicine A and Medicine B are equally effective and have some risk of a severe side effect.

The only difference between Medicine A and Medicine B is how you take them.

Medicine A	Medicine B
You take a tablet only once in your life.<br>You will be given the tablet on the day you come in to see your GP.<br>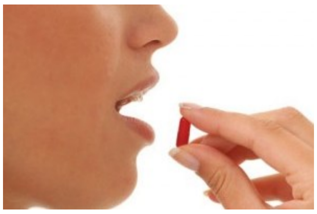	One tablet should be taken once a week on the same day every week.<br>You need to take this medicine for 5 years.<br><br>You need to take the tablet first thing in the morning on an empty stomach with a large glass of tap water.<br>After taking the tablet, you should stay upright for the next 30 minutes.<br>You should not eat anything for at least 30 minutes, including taking any other medicines.<br><br>You need to regularly visit your doctor to have your bone health checked.<br>You need to regularly visit a pharmacy for a new supply.<br>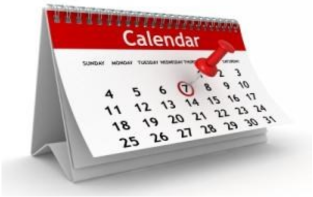 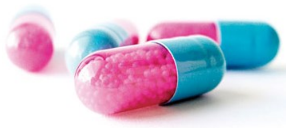

If you take Medicine A, you would live for **4 years and 2 months** and then die.

If you take Medicine B, you would live for **5 years** and then die.

Would you prefer Medicine A or Medicine B, or are they the same?

Indicate your choice here. You can only choose one option:

◯ **Medicine A** Live for 4 years 2 months

◯ **Medicine B** Live for 5 years

◯ **The Same - Medicine A and Medicine B are the same.**

0% 100%

For peer review only

Repeated section

Remember Medicine A and Medicine B are equally effective and have some risk of a severe side effect.

The only difference between Medicine A and Medicine B is how you take them.

Medicine A	Medicine B
You take a tablet only once in your life.<br>You will be given the tablet on the day you come in to see your GP.<br>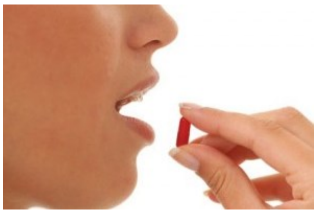	One tablet should be taken once a week on the same day every week.<br>You need to take this medicine for 5 years.<br><br>You need to take the tablet first thing in the morning on an empty stomach with a large glass of tap water.<br>After taking the tablet, you should stay upright for the next 30 minutes.<br>You should not eat anything for at least 30 minutes, including taking any other medicines.<br><br>You need to regularly visit your doctor to have your bone health checked.<br>You need to regularly visit a pharmacy for a new supply.<br>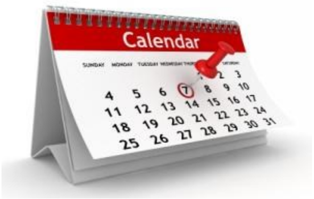 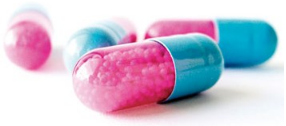

If you take Medicine A, you would live for **4.25 years** (4 years and 3 months) and then die.

If you take Medicine B, you would live for **5 years** and then die.

Would you prefer Medicine A or Medicine B, or are they the same?

Indicate your choice here. You can only choose one option:

◯ **Medicine A** Live for 4 years 3 months

◯ **Medicine B** Live for 5 years

◯ **The Same - Medicine A and Medicine B are the same.**

0% 100%

For peer review only

Repeated section

Remember Medicine A and Medicine B are equally effective and have some risk of a severe side effect.

The only difference between Medicine A and Medicine B is how you take them.

Medicine A	Medicine B
You take a tablet only once in your life. You will be given the tablet on the day you come in to see your GP. 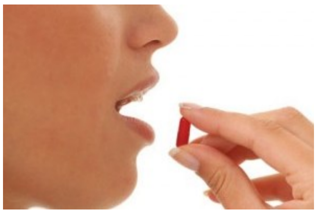	One tablet should be taken once a week on the same day every week. You need to take this medicine for 5 years.<br><br>You need to take the tablet first thing in the morning on an empty stomach with a large glass of tap water. After taking the tablet, you should stay upright for the next 30 minutes. You should not eat anything for at least 30 minutes, including taking any other medicines.<br><br>You need to regularly visit your doctor to have your bone health checked. You need to regularly visit a pharmacy for a new supply.<br>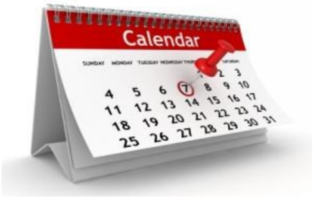 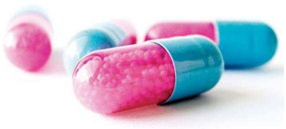

If you take Medicine A, you would live for **4 years 4 months** and then die.

If you take Medicine B, you would live for **5 years** and then die.

Would you prefer Medicine A or Medicine B, or are they the same?

Indicate your choice here. You can only choose one option:

○ **Medicine A** — Live for 4 years 4 months

○ **Medicine B** — Live for 5 years

○ **The Same - Medicine A and Medicine B are the same.**

For peer review only

Repeated section

Remember Medicine A and Medicine B are equally effective and have some risk of a severe side effect.

The only difference between Medicine A and Medicine B is how you take them.

**Medicine A**	**Medicine B**
You take a tablet only once in your life. You will be given the tablet on the day you come in to see your GP. 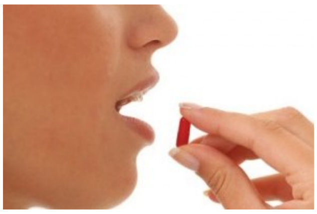	One tablet should be taken once a week on the same day every week.<br>You need to take this medicine for 5 years.<br><br>You need to take the tablet first thing in the morning on an empty stomach with a large glass of tap water.<br>After taking the tablet, you should stay upright for the next 30 minutes.<br>You should not eat anything for at least 30 minutes, including taking any other medicines.<br><br>You need to regularly visit your doctor to have your bone health checked.<br>You need to regularly visit a pharmacy for a new supply.<br>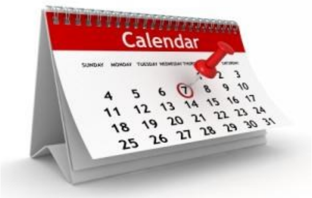 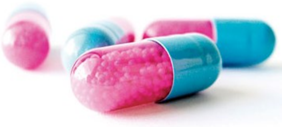

If you take Medicine A, you would live for **4.5 years** (4 years and 6 months) and then die.

If you take Medicine B, you would live for **5 years** and then die.

Would you prefer Medicine A or Medicine B, or are they the same?

Indicate your choice here. You can only choose one option:

◯ **Medicine A** Live for 4 years 6 months

◯ **Medicine B** Live for 5 years

◯ **The Same - Medicine A and Medicine B are the same.**

0% 100%

For peer review only

Repeated section

Remember Medicine A and Medicine B are equally effective and have some risk of a severe side effect.

The only difference between Medicine A and Medicine B is how you take them.

Medicine A	Medicine B
You take a tablet only once in your life. You will be given the tablet on the day you come in to see your GP. 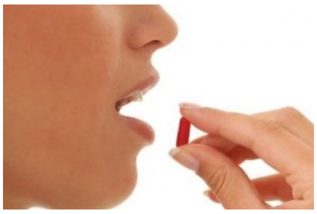	One tablet should be taken once a week on the same day every week. You need to take this medicine for 5 years.<br><br>You need to take the tablet first thing in the morning on an empty stomach with a large glass of tap water. After taking the tablet, you should stay upright for the next 30 minutes. You should not eat anything for at least 30 minutes, including taking any other medicines.<br><br>You need to regularly visit your doctor to have your bone health checked. You need to regularly visit a pharmacy for a new supply. 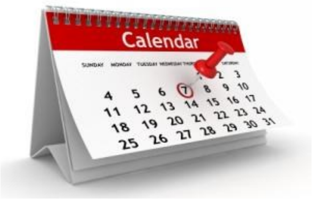 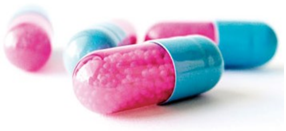

If you take Medicine A, you would live for **4 years and 7 months** and then die.

If you take Medicine B, you would live for **5 years** and then die.

Would you prefer Medicine A or Medicine B, or are they the same?

Indicate your choice here. You can only choose one option:

○ **Medicine A** Live for 4 years 7 months

○ **Medicine B** Live for 5 years

○ **The Same - Medicine A and Medicine B are the same.**

For peer review only

Repeated section

Remember Medicine A and Medicine B are equally effective and have some risk of a severe side effect.

The only difference between Medicine A and Medicine B is how you take them.

Medicine A	Medicine B
You take a tablet only once in your life.<br>You will be given the tablet on the day you come in to see your GP.<br>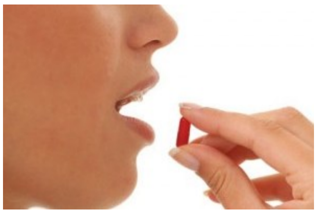	One tablet should be taken once a week on the same day every week.<br>You need to take this medicine for 5 years.<br><br>You need to take the tablet first thing in the morning on an empty stomach with a large glass of tap water.<br>After taking the tablet, you should stay upright for the next 30 minutes.<br>You should not eat anything for at least 30 minutes, including taking any other medicines.<br><br>You need to regularly visit your doctor to have your bone health checked.<br>You need to regularly visit a pharmacy for a new supply.<br>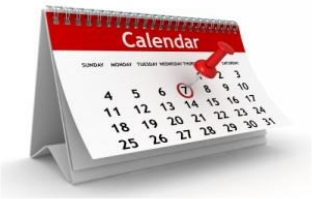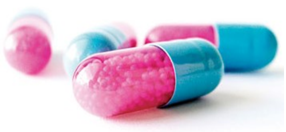

If you take Medicine A, you would live for **4 years and 9 months** and then die.

If you take Medicine B, you would live for **5 years** and then die.

Would you prefer Medicine A or Medicine B, or are they the same?

Indicate your choice here. You can only choose one option:

○ **Medicine A** Live for 4 years 9 months

○ **Medicine B** Live for 5 years

○ **The Same - Medicine A and Medicine B are the same.**

0% 100%

For peer review only

Repeated section

Remember Medicine A and Medicine B are equally effective and have some risk of a severe side effect.

The only difference between Medicine A and Medicine B is how you take them.

Medicine A	Medicine B
You take a tablet only once in your life.<br>You will be given the tablet on the day you come in to see your GP.<br>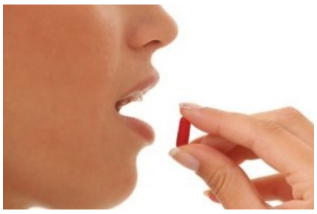	One tablet should be taken once a week on the same day every week.<br>You need to take this medicine for 5 years.<br><br>You need to take the tablet first thing in the morning on an empty stomach with a large glass of tap water.<br>After taking the tablet, you should stay upright for the next 30 minutes.<br>You should not eat anything for at least 30 minutes, including taking any other medicines.<br><br>You need to regularly visit your doctor to have your bone health checked.<br>You need to regularly visit a pharmacy for a new supply.<br>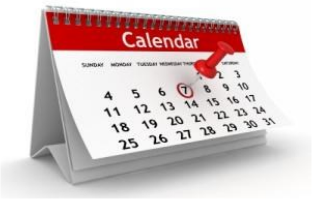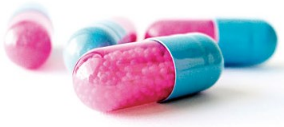

If you take Medicine A, you would live for **2 months less than 5 years** and then die.

If you take Medicine B, you would live for **5 years** and then die.

Would you prefer Medicine A or Medicine B, or are they the same?

Indicate your choice here. You can only choose one option:

○ **Medicine A** Live for (5 years – 2 months)

○ **Medicine B** Live for 5 years

○ **The Same - Medicine A and Medicine B are the same.**

For peer review only

Repeated section

Remember Medicine A and Medicine B are equally effective and have some risk of a severe side effect.

The only difference between Medicine A and Medicine B is how you take them.

Medicine A	Medicine B
You take a tablet only once in your life.<br>You will be given the tablet on the day you come in to see your GP.<br>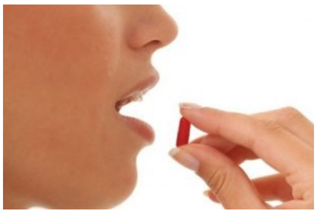	One tablet should be taken once a week on the same day every week.<br>You need to take this medicine for 5 years.<br><br>You need to take the tablet first thing in the morning on an empty stomach with a large glass of tap water.<br>After taking the tablet, you should stay upright for the next 30 minutes.<br>You should not eat anything for at least 30 minutes, including taking any other medicines.<br><br>You need to regularly visit your doctor to have your bone health checked.<br>You need to regularly visit a pharmacy for a new supply.<br>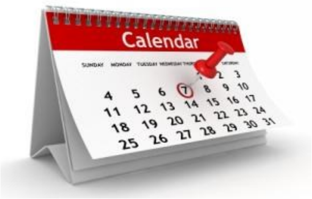 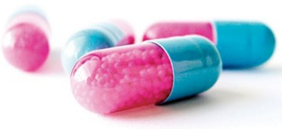

If you take Medicine A, you would live for **1 month less than 5 years** and then die.

If you take Medicine B, you would live for **5 years** and then die.

Would you prefer Medicine A or Medicine B, or are they the same?

Indicate your choice here. You can only choose one option:

◯ **Medicine A** Live for (5 years – 1 month)

◯ **Medicine B** Live for 5 years

◯ **The Same - Medicine A and Medicine B are the same.**

For peer review only

Repeated section

Remember Medicine A and Medicine B are equally effective and have some risk of a severe side effect.

The only difference between Medicine A and Medicine B is how you take them.

Medicine A	Medicine B
You take a tablet only once in your life. You will be given the tablet on the day you come in to see your GP. 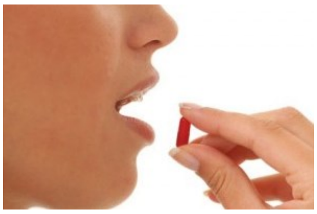	One tablet should be taken once a week on the same day every week. You need to take this medicine for 5 years. <br>You need to take the tablet first thing in the morning on an empty stomach with a large glass of tap water. After taking the tablet, you should stay upright for the next 30 minutes. You should not eat anything for at least 30 minutes, including taking any other medicines. <br>You need to regularly visit your doctor to have your bone health checked. You need to regularly visit a pharmacy for a new supply. 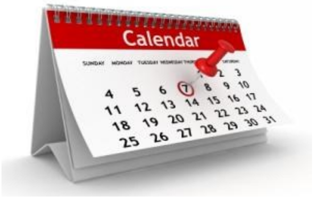 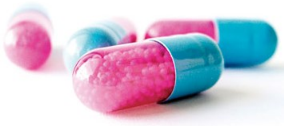

If you take Medicine A, you would live for **2 weeks less than 5 years** and then die.

If you take Medicine B, you would live for **5 years** and then die.

Would you prefer Medicine A or Medicine B, or are they the same?

Indicate your choice here. You can only choose one option:

◯ **Medicine A** Live for (5 years – 2 weeks)

◯ **Medicine B** Live for 5 years

◯ **The Same - Medicine A and Medicine B are the same.**

0% 100%

For peer review only

Repeated section

Remember Medicine A and Medicine B are equally effective and have some risk of a severe side effect.

The only difference between Medicine A and Medicine B is how you take them.

**Medicine A**	**Medicine B**
You take a tablet only once in your life. You will be given the tablet on the day you come in to see your GP.<br>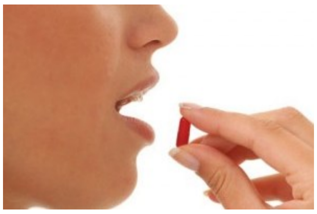	One tablet should be taken once a week on the same day every week.<br>You need to take this medicine for 5 years.<br><br>You need to take the tablet first thing in the morning on an empty stomach with a large glass of tap water.<br>After taking the tablet, you should stay upright for the next 30 minutes.<br>You should not eat anything for at least 30 minutes, including taking any other medicines.<br><br>You need to regularly visit your doctor to have your bone health checked.<br>You need to regularly visit a pharmacy for a new supply.<br>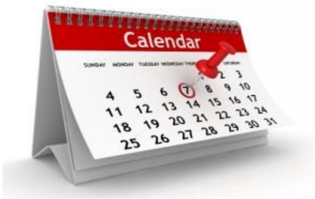 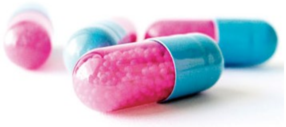

If you take Medicine A, you would live for **1 week less than 5 years** and then die.

If you take Medicine B, you would live for **5 years** and then die.

Would you prefer Medicine A or Medicine B, or are they the same?

Indicate your choice here. You can only choose one option:

◯ **Medicine A** Live for (5 years – 1 week)

◯ **Medicine B** Live for 5 years

◯ **The Same - Medicine A and Medicine B are the same.**

0% 100%

For peer review only

Repeated section

Remember Medicine A and Medicine B are equally effective and have some risk of a severe side effect.

The only difference between Medicine A and Medicine B is how you take them.

Medicine A	Medicine B
You take a tablet only once in your life.<br>You will be given the tablet on the day you come in to see your GP.<br>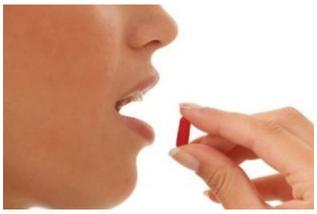	One tablet should be taken once a week on the same day every week.<br>You need to take this medicine for 5 years.<br><br>You need to take the tablet first thing in the morning on an empty stomach with a large glass of tap water.<br>After taking the tablet, you should stay upright for the next 30 minutes.<br>You should not eat anything for at least 30 minutes, including taking any other medicines.<br><br>You need to regularly visit your doctor to have your bone health checked.<br>You need to regularly visit a pharmacy for a new supply.<br>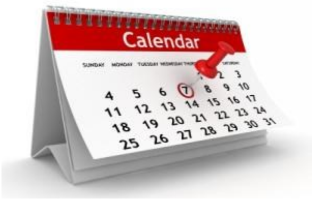 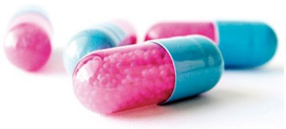

If you take Medicine A, you would live for **5 days less than 5 years** and then die.

If you take Medicine B, you would live for **5 years** and then die.

Would you prefer Medicine A or Medicine B, or are they the same?

Indicate your choice here. You can only choose one option:

◯ **Medicine A** Live for (5 years – 5 days)

◯ **Medicine B** Live for 5 years

◯ **The Same - Medicine A and Medicine B are the same.**

0% 100%

For peer review only

Repeated section

Remember Medicine A and Medicine B are equally effective and have some risk of a severe side effect.

The only difference between Medicine A and Medicine B is how you take them.

Medicine A	Medicine B
You take a tablet only once in your life.<br>You will be given the tablet on the day you come in to see your GP.<br>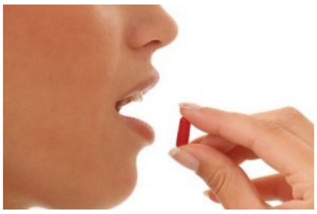	One tablet should be taken once a week on the same day every week.<br>You need to take this medicine for 5 years.<br><br>You need to take the tablet first thing in the morning on an empty stomach with a large glass of tap water.<br>After taking the tablet, you should stay upright for the next 30 minutes.<br>You should not eat anything for at least 30 minutes, including taking any other medicines.<br><br>You need to regularly visit your doctor to have your bone health checked.<br>You need to regularly visit a pharmacy for a new supply.<br>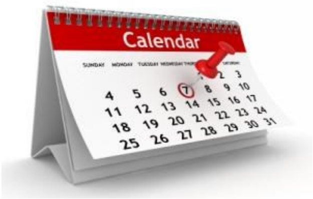 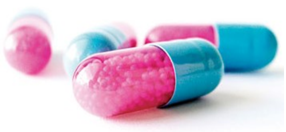

If you take Medicine A, you would live for **3 days less than 5 years** and then die.

If you take Medicine B, you would live for **5 years** and then die.

Would you prefer Medicine A or Medicine B, or are they the same?

Indicate your choice here. You can only choose one option:

◯ **Medicine A** Live for (5 years – 3 days)

◯ **Medicine B** Live for 5 years

◯ **The Same - Medicine A and Medicine B are the same.**

0% 100%

For peer review only

Repeated section

Remember Medicine A and Medicine B are equally effective and have some risk of a severe side effect.

The only difference between Medicine A and Medicine B is how you take them.

Medicine A	Medicine B
You take a tablet only once in your life. You will be given the tablet on the day you come in to see your GP.<br>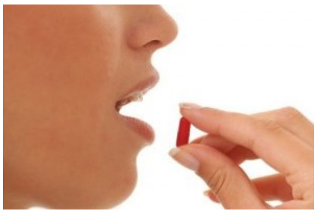	One tablet should be taken once a week on the same day every week.<br>You need to take this medicine for 5 years.<br><br>You need to take the tablet first thing in the morning on an empty stomach with a large glass of tap water.<br>After taking the tablet, you should stay upright for the next 30 minutes.<br>You should not eat anything for at least 30 minutes, including taking any other medicines.<br><br>You need to regularly visit your doctor to have your bone health checked.<br>You need to regularly visit a pharmacy for a new supply.<br>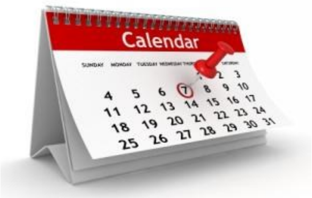 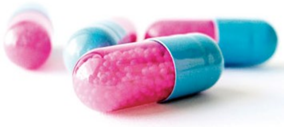

If you take Medicine A, you would live for **1 day less than 5 years** and then die.

If you take Medicine B, you would live for **5 years** and then die.

Would you prefer Medicine A or Medicine B, or are they the same?

Indicate your choice here. You can only choose one option:

◯ **Medicine A** Live for (5 years – 1 day)

◯ **Medicine B** Live for 5 years

◯ **The Same - Medicine A and Medicine B are the same.**

0% 100%

For peer review only

Question 3a.

You prefer living for 2.5 years (2 years and 6 months) on Medicine A, to living for 5 years on Medicine B.

This means **living fewer than 2.5 years on Medicine A** can be the same as living for 5 years on Medicine B.

How many years on Medicine A do you think would be the same as living for 5 years on Medicine B?

(Please give a number in years)

0% 100%

peer review only

Part 2

Question 4

Now, Medicine A and Medicine B will give you less benefit than before because you belong to a group of people who may not respond to the treatment as well as other groups of people.

As before,

Medicine A is a pill that you only need to take once.

Medicine B is a pill that you need to take every week for 5 years.

Medicine A and Medicine B are equally effective in reducing your risk of having a fracture.

Medicine A and Medicine B will reduce your risk of experiencing a fracture, such as hip fracture, within the next 5 years from **10%** to **9%**.

In other words, in a crowd of **1,000** people in the UK, **100** are likely to have a fracture if they do not take the medicine. This is reduced to **90** people out of 1,000 who do take the medicine. This information is also illustrated in this picture.

Risk of fracture

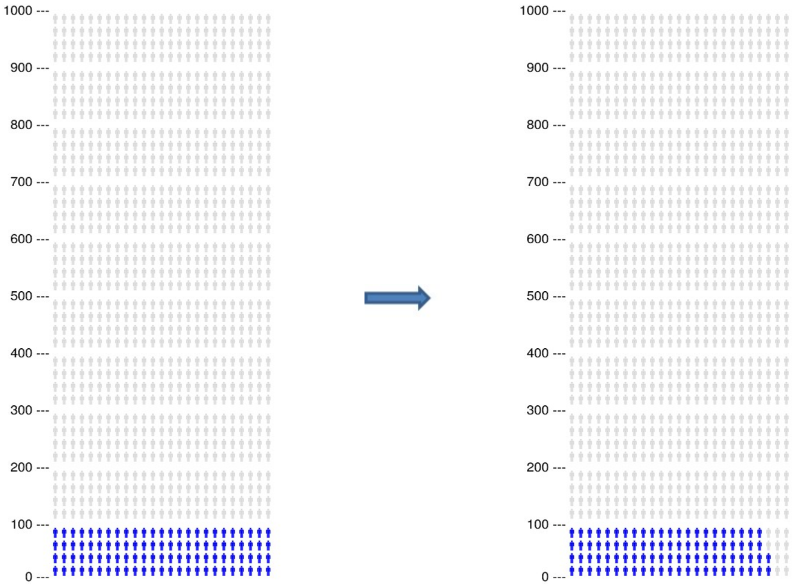

10%
No Treatment

9%
Taking the Medicine

(The figures shaded blue represent people who will have a fracture, such as hip fracture, within the next 5 years)

In this scenario, you will not have any side effects from Medicine A or Medicine B.

Risk of a side effect

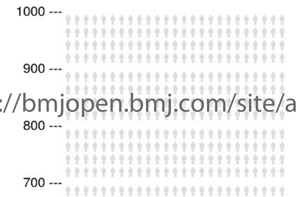

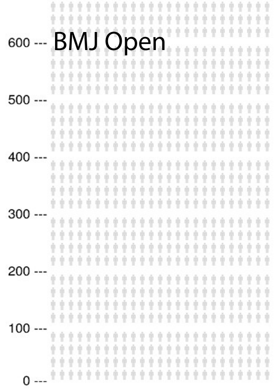

0 %

No one will have a side effect.

0% 100%

eer review only

Repeated section

Remember Medicine A and Medicine B are equally effective and have no risks of a side effect.

The only difference between Medicine A and Medicine B is how you take them.

**Medicine A**	**Medicine B**
You take a tablet only once in your life. You will be given the tablet on the day you come in to see your GP. 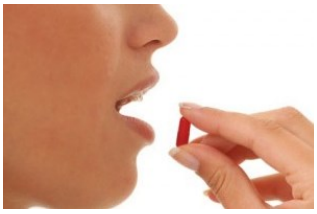	One tablet should be taken once a week on the same day every week. You need to take this medicine for 5 years. You need to take the tablet first thing in the morning on an empty stomach with a large glass of tap water. After taking the tablet, you should stay upright for the next 30 minutes. You should not eat anything for at least 30 minutes, including taking any other medicines. You need to regularly visit your doctor to have your bone health checked. You need to regularly visit a pharmacy for a new supply. 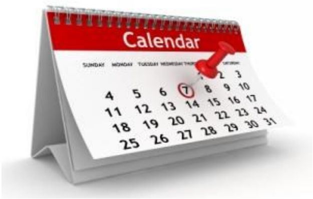 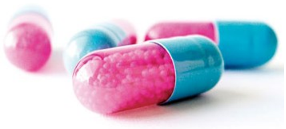

If you take Medicine A, you would live for **2.5 years (2 years and 6 months)** and then die.

If you take Medicine B, you would live for **5 years** and then die.

Would you prefer Medicine A or Medicine B, or are they the same?

Indicate your choice here. You can only choose one option:

○ **Medicine A** — Live for 2.5 years

○ **Medicine B** — Live for 5 years

○ **The Same - Medicine A and Medicine B are the same.**

0% 100%

For peer review only

Repeated section

Remember Medicine A and Medicine B are equally effective and have no risks of a side effect.

The only difference between Medicine A and Medicine B is how you take them.

**Medicine A**	**Medicine B**
You take a tablet only once in your life. You will be given the tablet on the day you come in to see your GP. 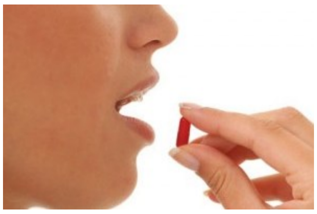	One tablet should be taken once a week on the same day every week. You need to take this medicine for 5 years.<br><br>You need to take the tablet first thing in the morning on an empty stomach with a large glass of tap water. After taking the tablet, you should stay upright for the next 30 minutes. You should not eat anything for at least 30 minutes, including taking any other medicines.<br><br>You need to regularly visit your doctor to have your bone health checked. You need to regularly visit a pharmacy for a new supply. 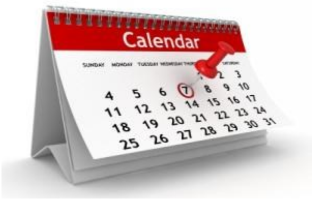 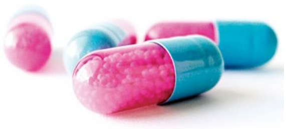

If you take Medicine A, you would live for **3 years** and then die.

If you take Medicine B, you would live for **5 years** and then die.

Would you prefer Medicine A or Medicine B, or are they the same?

Indicate your choice here. You can only choose one option:

○ **Medicine A** 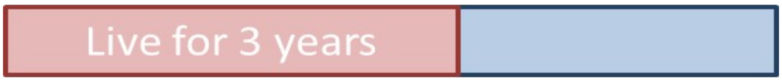

○ **Medicine B** 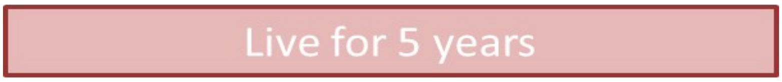

○ **The Same - Medicine A and Medicine B are the same.**

0% 100%

For peer review only

Repeated section

Remember Medicine A and Medicine B are equally effective and have no risks of a side effect.

The only difference between Medicine A and Medicine B is how you take them.

Medicine A	Medicine B
You take a tablet only once in your life.<br>You will be given the tablet on the day you come in to see your GP.<br>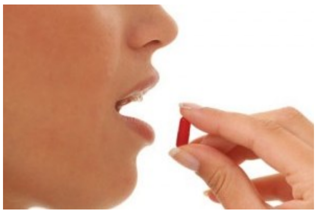	One tablet should be taken once a week on the same day every week.<br>You need to take this medicine for 5 years.<br><br>You need to take the tablet first thing in the morning on an empty stomach with a large glass of tap water.<br>After taking the tablet, you should stay upright for the next 30 minutes.<br>You should not eat anything for at least 30 minutes, including taking any other medicines.<br><br>You need to regularly visit your doctor to have your bone health checked.<br>You need to regularly visit a pharmacy for a new supply.<br>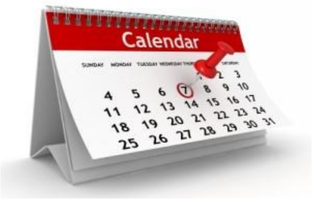 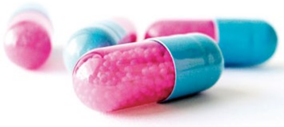

If you take Medicine A, you would live for **3.25 years** (3 years and 3 months) and then die.

If you take Medicine B, you would live for **5 years** and then die.

Would you prefer Medicine A or Medicine B, or are they the same?

Indicate your choice here. You can only choose one option:

◯ **Medicine A** Live for 3 years 3 months

◯ **Medicine B** Live for 5 years

◯ **The Same - Medicine A and Medicine B are the same.**

For peer review only

Repeated section

Remember Medicine A and Medicine B are equally effective and have no risks of a side effect.

The only difference between Medicine A and Medicine B is how you take them.

Medicine A	Medicine B
You take a tablet only once in your life. You will be given the tablet on the day you come in to see your GP.<br>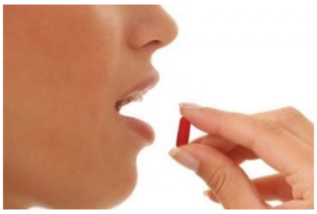	One tablet should be taken once a week on the same day every week.<br>You need to take this medicine for 5 years.<br><br>You need to take the tablet first thing in the morning on an empty stomach with a large glass of tap water.<br>After taking the tablet, you should stay upright for the next 30 minutes.<br>You should not eat anything for at least 30 minutes, including taking any other medicines.<br><br>You need to regularly visit your doctor to have your bone health checked.<br>You need to regularly visit a pharmacy for a new supply.<br>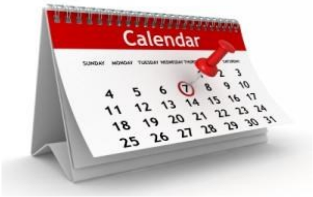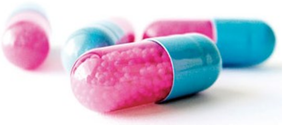

If you take Medicine A, you would live for **3.5 years** (3 years and 6 months) and then die.

If you take Medicine B, you would live for **5 years** and then die.

Would you prefer Medicine A or Medicine B, or are they the same?

Indicate your choice here. You can only choose one option:

◯ **Medicine A** Live for 3 years 6 months

◯ **Medicine B** Live for 5 years

◯ **The Same - Medicine A and Medicine B are the same.**

0% 100%

For peer review only

Repeated section

Remember Medicine A and Medicine B are equally effective and have no risks of a side effect.

The only difference between Medicine A and Medicine B is how you take them.

**Medicine A**	**Medicine B**
You take a tablet only once in your life. You will be given the tablet on the day you come in to see your GP. 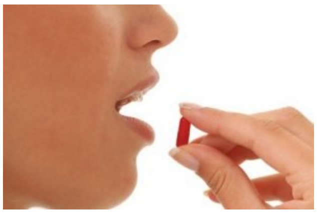	One tablet should be taken once a week on the same day every week. You need to take this medicine for 5 years.<br><br>You need to take the tablet first thing in the morning on an empty stomach with a large glass of tap water. After taking the tablet, you should stay upright for the next 30 minutes. You should not eat anything for at least 30 minutes, including taking any other medicines.<br><br>You need to regularly visit your doctor to have your bone health checked. You need to regularly visit a pharmacy for a new supply.<br>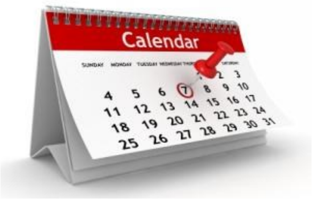 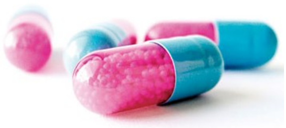

If you take Medicine A, you would live for **3.75 years** (3 years and 9 months) and then die.

If you take Medicine B, you would live for **5 years** and then die.

Would you prefer Medicine A or Medicine B, or are they the same?

Indicate your choice here. You can only choose one option:

◯ **Medicine A** Live for 3 years 9 months

◯ **Medicine B** Live for 5 years

◯ **The Same - Medicine A and Medicine B are the same.**

For peer review only

Repeated section

Remember Medicine A and Medicine B are equally effective and have no risks of a side effect.

The only difference between Medicine A and Medicine B is how you take them.

Medicine A	Medicine B
You take a tablet only once in your life.<br>You will be given the tablet on the day you come in to see your GP.<br>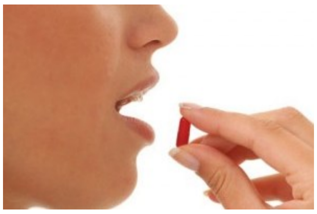	One tablet should be taken once a week on the same day every week.<br>You need to take this medicine for 5 years.<br><br>You need to take the tablet first thing in the morning on an empty stomach with a large glass of tap water.<br>After taking the tablet, you should stay upright for the next 30 minutes.<br>You should not eat anything for at least 30 minutes, including taking any other medicines.<br><br>You need to regularly visit your doctor to have your bone health checked.<br>You need to regularly visit a pharmacy for a new supply.<br>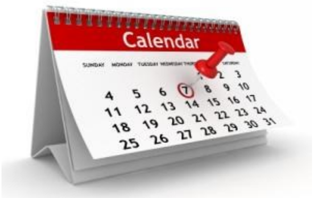 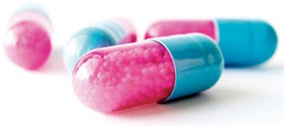

If you take Medicine A, you would live for **3 years and 10 months** and then die.

If you take Medicine B, you would live for **5 years** and then die.

Would you prefer Medicine A or Medicine B, or are they the same?

Indicate your choice here. You can only choose one option:

○ **Medicine A** Live for 3 years 10 months

○ **Medicine B** Live for 5 years

○ **The Same - Medicine A and Medicine B are the same.**

0% 100%

For peer review only

Repeated section

Remember Medicine A and Medicine B are equally effective and have no risks of a side effect.

The only difference between Medicine A and Medicine B is how you take them.

Medicine A	Medicine B
You take a tablet only once in your life. You will be given the tablet on the day you come in to see your GP. 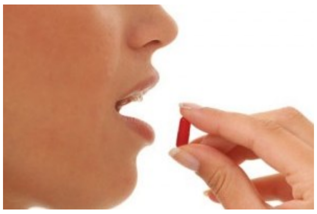	One tablet should be taken once a week on the same day every week. You need to take this medicine for 5 years.<br><br>You need to take the tablet first thing in the morning on an empty stomach with a large glass of tap water. After taking the tablet, you should stay upright for the next 30 minutes. You should not eat anything for at least 30 minutes, including taking any other medicines.<br><br>You need to regularly visit your doctor to have your bone health checked. You need to regularly visit a pharmacy for a new supply.<br>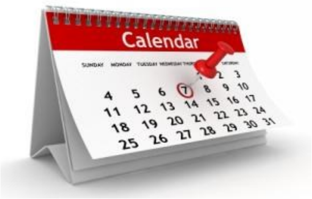 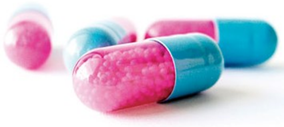

If you take Medicine A, you would live for **4 years** and then die.

If you take Medicine B, you would live for **5 years** and then die.

Would you prefer Medicine A or Medicine B, or are they the same?

Indicate your choice here. You can only choose one option:

◯ **Medicine A**

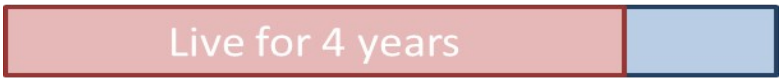

◯ **Medicine B**

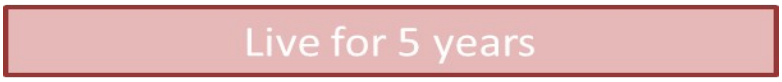

◯ **The Same - Medicine A and Medicine B are the same.**

For peer review only

Repeated section

Remember Medicine A and Medicine B are equally effective and have no risks of a side effect.

The only difference between Medicine A and Medicine B is how you take them.

Medicine A	Medicine B
You take a tablet only once in your life.<br>You will be given the tablet on the day you come in to see your GP.<br>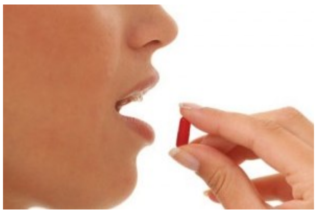	One tablet should be taken once a week on the same day every week.<br>You need to take this medicine for 5 years.<br><br>You need to take the tablet first thing in the morning on an empty stomach with a large glass of tap water.<br>After taking the tablet, you should stay upright for the next 30 minutes.<br>You should not eat anything for at least 30 minutes, including taking any other medicines.<br><br>You need to regularly visit your doctor to have your bone health checked.<br>You need to regularly visit a pharmacy for a new supply.<br>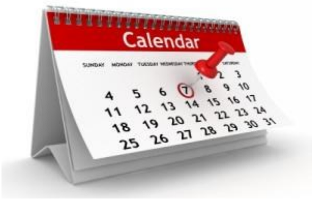 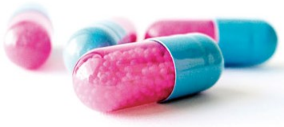

If you take Medicine A, you would live for **4 years and 2 months** and then die.

If you take Medicine B, you would live for **5 years** and then die.

Would you prefer Medicine A or Medicine B, or are they the same?

Indicate your choice here. You can only choose one option:

◯ **Medicine A** Live for 4 years 2 months

◯ **Medicine B** Live for 5 years

◯ **The Same - Medicine A and Medicine B are the same.**

0% 100%

For peer review only

Repeated section

Remember Medicine A and Medicine B are equally effective and have no risks of a side effect.

The only difference between Medicine A and Medicine B is how you take them.

Medicine A	Medicine B
You take a tablet only once in your life. You will be given the tablet on the day you come in to see your GP. 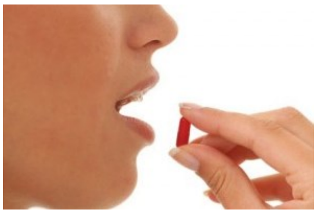	One tablet should be taken once a week on the same day every week. You need to take this medicine for 5 years.<br><br>You need to take the tablet first thing in the morning on an empty stomach with a large glass of tap water. After taking the tablet, you should stay upright for the next 30 minutes. You should not eat anything for at least 30 minutes, including taking any other medicines.<br><br>You need to regularly visit your doctor to have your bone health checked. You need to regularly visit a pharmacy for a new supply. 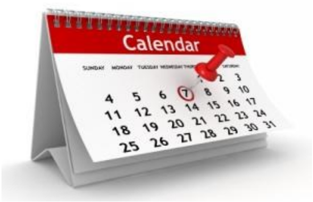 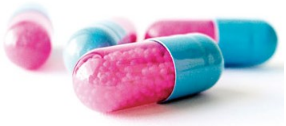

If you take Medicine A, you would live for **4.25 years** (4 years and 3 months) and then die.

If you take Medicine B, you would live for **5 years** and then die.

Would you prefer Medicine A or Medicine B, or are they the same?

Indicate your choice here. You can only choose one option:

◯ **Medicine A** Live for 4 years 3 months

◯ **Medicine B** Live for 5 years

◯ **The Same - Medicine A and Medicine B are the same.**

0% 100%

For peer review only

Repeated section

Remember Medicine A and Medicine B are equally effective and have no risks of a side effect.

The only difference between Medicine A and Medicine B is how you take them.

Medicine A	Medicine B
You take a tablet only once in your life.<br>You will be given the tablet on the day you come in to see your GP.<br>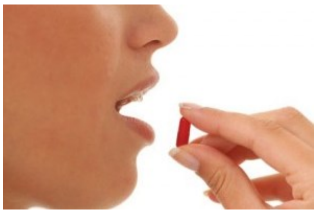	One tablet should be taken once a week on the same day every week.<br>You need to take this medicine for 5 years.<br><br>You need to take the tablet first thing in the morning on an empty stomach with a large glass of tap water.<br>After taking the tablet, you should stay upright for the next 30 minutes.<br>You should not eat anything for at least 30 minutes, including taking any other medicines.<br><br>You need to regularly visit your doctor to have your bone health checked.<br>You need to regularly visit a pharmacy for a new supply.<br>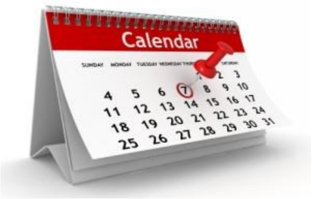 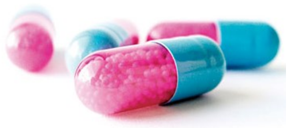

If you take Medicine A, you would live for **4 years 4 months** and then die.

If you take Medicine B, you would live for **5 years** and then die.

Would you prefer Medicine A or Medicine B, or are they the same?

Indicate your choice here. You can only choose one option:

○ **Medicine A** Live for 4 years 4 months

○ **Medicine B** Live for 5 years

○ **The Same - Medicine A and Medicine B are the same.**

0% 100%

For peer review only

Repeated section

Remember Medicine A and Medicine B are equally effective and have no risks of a side effect.

The only difference between Medicine A and Medicine B is how you take them.

Medicine A	Medicine B
You take a tablet only once in your life. You will be given the tablet on the day you come in to see your GP.<br>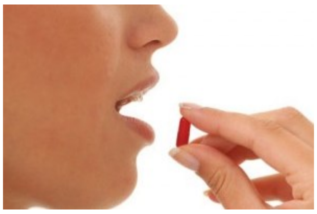	One tablet should be taken once a week on the same day every week.<br>You need to take this medicine for 5 years.<br><br>You need to take the tablet first thing in the morning on an empty stomach with a large glass of tap water.<br>After taking the tablet, you should stay upright for the next 30 minutes.<br>You should not eat anything for at least 30 minutes, including taking any other medicines.<br><br>You need to regularly visit your doctor to have your bone health checked.<br>You need to regularly visit a pharmacy for a new supply.<br>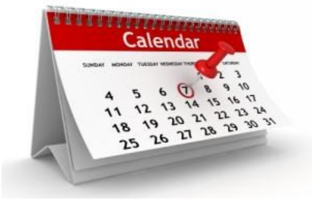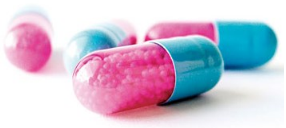

If you take Medicine A, you would live for **4.5 years** (4 years and 6 months) and then die.

If you take Medicine B, you would live for **5 years** and then die.

Would you prefer Medicine A or Medicine B, or are they the same?

Indicate your choice here. You can only choose one option:

○ **Medicine A** Live for 4 years 6 months

○ **Medicine B** Live for 5 years

○ **The Same - Medicine A and Medicine B are the same.**

0% 100%

For peer review only

Repeated section

Remember Medicine A and Medicine B are equally effective and have no risks of a side effect.

The only difference between Medicine A and Medicine B is how you take them.

**Medicine A**	**Medicine B**
You take a tablet only once in your life. You will be given the tablet on the day you come in to see your GP. 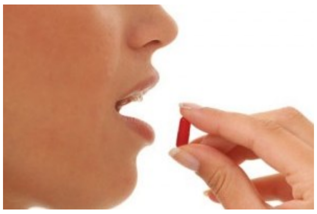	One tablet should be taken once a week on the same day every week.<br>You need to take this medicine for 5 years.<br><br>You need to take the tablet first thing in the morning on an empty stomach with a large glass of tap water.<br>After taking the tablet, you should stay upright for the next 30 minutes.<br>You should not eat anything for at least 30 minutes, including taking any other medicines.<br><br>You need to regularly visit your doctor to have your bone health checked.<br>You need to regularly visit a pharmacy for a new supply.<br>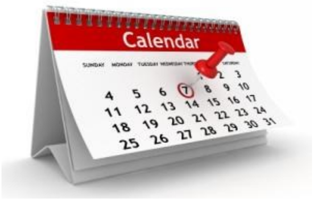 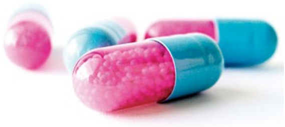

If you take Medicine A, you would live for **4 years and 7 months** and then die.

If you take Medicine B, you would live for **5 years** and then die.

Would you prefer Medicine A or Medicine B, or are they the same?

Indicate your choice here. You can only choose one option:

◯ **Medicine A** Live for 4 years 7 months

◯ **Medicine B** Live for 5 years

◯ **The Same - Medicine A and Medicine B are the same.**

0% 100%

For peer review only

Repeated section

Remember Medicine A and Medicine B are equally effective and have no risks of a side effect.

The only difference between Medicine A and Medicine B is how you take them.

Medicine A	Medicine B
You take a tablet only once in your life.<br>You will be given the tablet on the day you come in to see your GP.<br>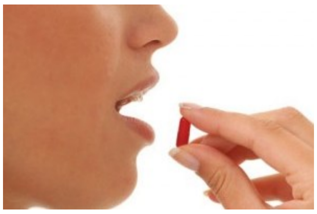	One tablet should be taken once a week on the same day every week.<br>You need to take this medicine for 5 years.<br><br>You need to take the tablet first thing in the morning on an empty stomach with a large glass of tap water.<br>After taking the tablet, you should stay upright for the next 30 minutes.<br>You should not eat anything for at least 30 minutes, including taking any other medicines.<br><br>You need to regularly visit your doctor to have your bone health checked.<br>You need to regularly visit a pharmacy for a new supply.<br>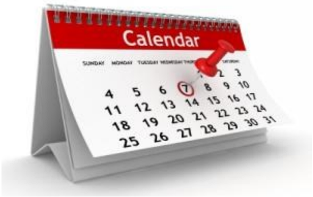 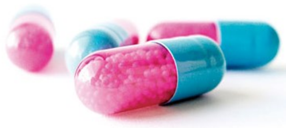

If you take Medicine A, you would live for **4 years and 9 months** and then die.

If you take Medicine B, you would live for **5 years** and then die.

Would you prefer Medicine A or Medicine B, or are they the same?

Indicate your choice here. You can only choose one option:

◯ **Medicine A** Live for 4 years 9 months

◯ **Medicine B** Live for 5 years

◯ **The Same - Medicine A and Medicine B are the same.**

0% 100%

For peer review only

Repeated section

Remember Medicine A and Medicine B are equally effective and have no risks of a side effect.

The only difference between Medicine A and Medicine B is how you take them.

Medicine A	Medicine B
You take a tablet only once in your life. You will be given the tablet on the day you come in to see your GP. 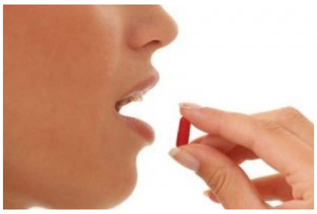	One tablet should be taken once a week on the same day every week. You need to take this medicine for 5 years. You need to take the tablet first thing in the morning on an empty stomach with a large glass of tap water. After taking the tablet, you should stay upright for the next 30 minutes. You should not eat anything for at least 30 minutes, including taking any other medicines. You need to regularly visit your doctor to have your bone health checked. You need to regularly visit a pharmacy for a new supply. 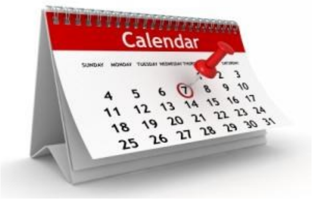 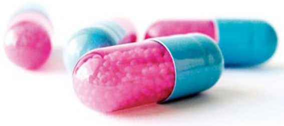

If you take Medicine A, you would live for **2 months less than 5 years** and then die.

If you take Medicine B, you would live for **5 years** and then die.

Would you prefer Medicine A or Medicine B, or are they the same?

Indicate your choice here. You can only choose one option:

◯ **Medicine A** Live for (5 years – 2 months)

◯ **Medicine B** Live for 5 years

◯ **The Same - Medicine A and Medicine B are the same.**

0% 100%

For peer review only

Repeated section

Remember Medicine A and Medicine B are equally effective and have no risks of a side effect.

The only difference between Medicine A and Medicine B is how you take them.

Medicine A	Medicine B
You take a tablet only once in your life.<br>You will be given the tablet on the day you come in to see your GP.<br>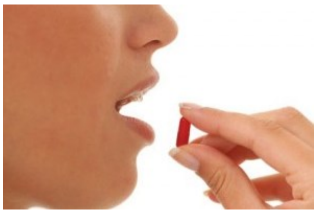	One tablet should be taken once a week on the same day every week.<br>You need to take this medicine for 5 years.<br><br>You need to take the tablet first thing in the morning on an empty stomach with a large glass of tap water.<br>After taking the tablet, you should stay upright for the next 30 minutes.<br>You should not eat anything for at least 30 minutes, including taking any other medicines.<br><br>You need to regularly visit your doctor to have your bone health checked.<br>You need to regularly visit a pharmacy for a new supply.<br>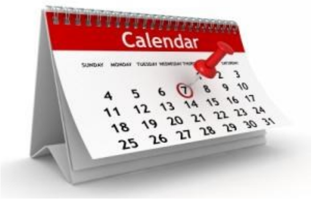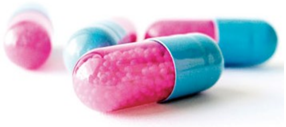

If you take Medicine A, you would live for **1 month less than 5 years** and then die.

If you take Medicine B, you would live for **5 years** and then die.

Would you prefer Medicine A or Medicine B, or are they the same?

Indicate your choice here. You can only choose one option:

○ **Medicine A** Live for (5 years – 1 month)

○ **Medicine B** Live for 5 years

○ **The Same - Medicine A and Medicine B are the same.**

0% 100%

For peer review only

Repeated section

Remember Medicine A and Medicine B are equally effective and have no risks of a side effect.

The only difference between Medicine A and Medicine B is how you take them.

**Medicine A**	**Medicine B**
You take a tablet only once in your life. You will be given the tablet on the day you come in to see your GP.<br>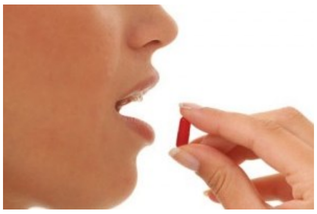	One tablet should be taken once a week on the same day every week.<br>You need to take this medicine for 5 years.<br><br>You need to take the tablet first thing in the morning on an empty stomach with a large glass of tap water.<br>After taking the tablet, you should stay upright for the next 30 minutes.<br>You should not eat anything for at least 30 minutes, including taking any other medicines.<br><br>You need to regularly visit your doctor to have your bone health checked.<br>You need to regularly visit a pharmacy for a new supply.<br>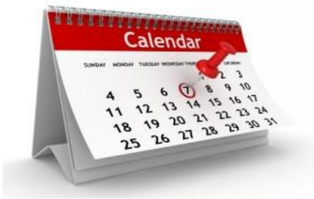 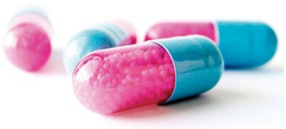

If you take Medicine A, you would live for **2 weeks less than 5 years** and then die.

If you take Medicine B, you would live for **5 years** and then die.

Would you prefer Medicine A or Medicine B, or are they the same?

Indicate your choice here. You can only choose one option:

- ◯ **Medicine A** Live for (5 years – 2 weeks)
- ◯ **Medicine B** Live for 5 years
- ◯ **The Same - Medicine A and Medicine B are the same.**

0% 100%

For peer review only

Repeated section

Remember Medicine A and Medicine B are equally effective and have no risks of a side effect.

The only difference between Medicine A and Medicine B is how you take them.

Medicine A	Medicine B
You take a tablet only once in your life. You will be given the tablet on the day you come in to see your GP. 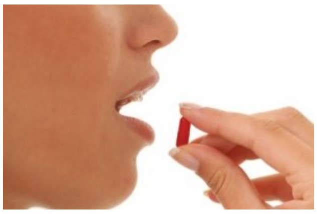	One tablet should be taken once a week on the same day every week. You need to take this medicine for 5 years. You need to take the tablet first thing in the morning on an empty stomach with a large glass of tap water. After taking the tablet, you should stay upright for the next 30 minutes. You should not eat anything for at least 30 minutes, including taking any other medicines. You need to regularly visit your doctor to have your bone health checked. You need to regularly visit a pharmacy for a new supply. 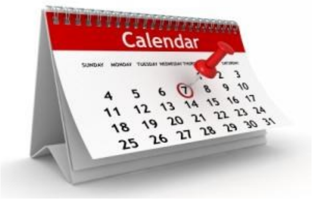 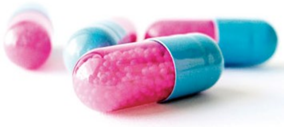

If you take Medicine A, you would live for **1 week less than 5 years** and then die.

If you take Medicine B, you would live for **5 years** and then die.

Would you prefer Medicine A or Medicine B, or are they the same?

Indicate your choice here. You can only choose one option:

◯ **Medicine A** Live for (5 years – 1 week)

◯ **Medicine B** Live for 5 years

◯ **The Same - Medicine A and Medicine B are the same.**

0% 100%

For peer review only

Repeated section

Remember Medicine A and Medicine B are equally effective and have no risks of a side effect.

The only difference between Medicine A and Medicine B is how you take them.

Medicine A	Medicine B
You take a tablet only once in your life. You will be given the tablet on the day you come in to see your GP. 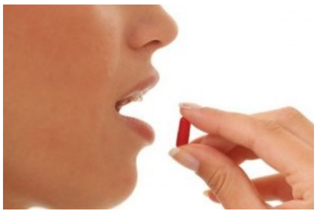	One tablet should be taken once a week on the same day every week. You need to take this medicine for 5 years. You need to take the tablet first thing in the morning on an empty stomach with a large glass of tap water. After taking the tablet, you should stay upright for the next 30 minutes. You should not eat anything for at least 30 minutes, including taking any other medicines. You need to regularly visit your doctor to have your bone health checked. You need to regularly visit a pharmacy for a new supply. 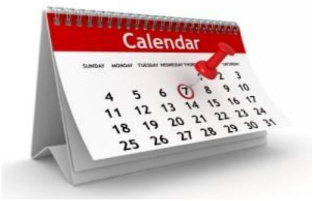 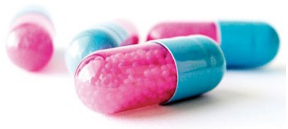

If you take Medicine A, you would live for **5 days less than 5 years** and then die.

If you take Medicine B, you would live for **5 years** and then die.

Would you prefer Medicine A or Medicine B, or are they the same?

Indicate your choice here. You can only choose one option:

○ **Medicine A** Live for (5 years – 5 days)

○ **Medicine B** Live for 5 years

○ **The Same - Medicine A and Medicine B are the same.**

0% 100%

For peer review only

Repeated section

Remember Medicine A and Medicine B are equally effective and have no risks of a side effect.

The only difference between Medicine A and Medicine B is how you take them.

Medicine A	Medicine B
You take a tablet only once in your life. You will be given the tablet on the day you come in to see your GP.<br>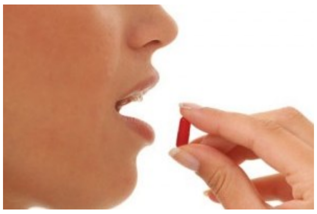	One tablet should be taken once a week on the same day every week.<br>You need to take this medicine for 5 years.<br><br>You need to take the tablet first thing in the morning on an empty stomach with a large glass of tap water.<br>After taking the tablet, you should stay upright for the next 30 minutes.<br>You should not eat anything for at least 30 minutes, including taking any other medicines.<br><br>You need to regularly visit your doctor to have your bone health checked.<br>You need to regularly visit a pharmacy for a new supply.<br>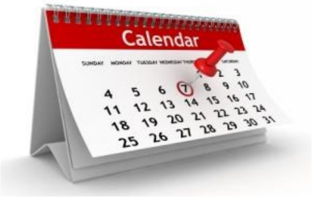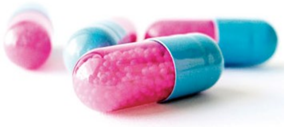

If you take Medicine A, you would live for **3 days less than 5 years** and then die.

If you take Medicine B, you would live for **5 years** and then die.

Would you prefer Medicine A or Medicine B, or are they the same?

Indicate your choice here. You can only choose one option:

◯ **Medicine A** Live for (5 years – 3 days)

◯ **Medicine B** Live for 5 years

◯ **The Same - Medicine A and Medicine B are the same.**

0% 100%

For peer review only

Repeated section

Remember Medicine A and Medicine B are equally effective and have no risks of a side effect.

The only difference between Medicine A and Medicine B is how you take them.

Medicine A	Medicine B
You take a tablet only once in your life. You will be given the tablet on the day you come in to see your GP.<br>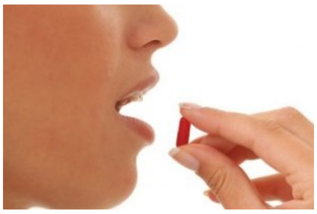	One tablet should be taken once a week on the same day every week.<br>You need to take this medicine for 5 years.<br><br>You need to take the tablet first thing in the morning on an empty stomach with a large glass of tap water.<br>After taking the tablet, you should stay upright for the next 30 minutes.<br>You should not eat anything for at least 30 minutes, including taking any other medicines.<br><br>You need to regularly visit your doctor to have your bone health checked.<br>You need to regularly visit a pharmacy for a new supply.<br>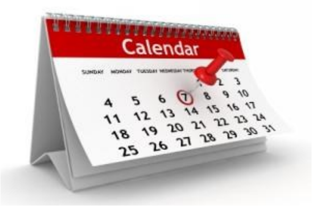 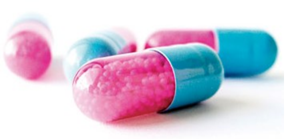

If you take Medicine A, you would live for **1 day less than 5 years** and then die.

If you take Medicine B, you would live for **5 years** and then die.

Would you prefer Medicine A or Medicine B, or are they the same?

Indicate your choice here. You can only choose one option:

◯ **Medicine A** Live for (5 years – 1 day)

◯ **Medicine B** Live for 5 years

◯ **The Same - Medicine A and Medicine B are the same.**

For peer review only

Question 4a.

You prefer living for 2.5 years (2 years and 6 months) on Medicine A, to living for 5 years on Medicine B.

This means **living fewer than 2.5 years on Medicine A** can be the same as living for 5 years on Medicine B.

How many years on Medicine A do you think would be the same as living for 5 years on Medicine B?

(Please give a number in years)

0% 100%

peer review only

Was there anything so far which you think is unclear or that you didn't understand?

(Please provide details in the comment box below)

0% 100%

peer review only

Part 2a

Question 5

You have been told that you need to take a medicine to reduce the risk of fracture.

You can choose between two different medicines. We have called these Medicine A and Medicine B.

Medicine A is a pill that you only need to take once.

Medicine B is a pill that you need to take every week for 5 years.

Medicine A and Medicine B are equally effective in reducing your risk of having a fracture.
Medicine A and Medicine B will reduce your risk of experiencing a fracture, such as hip fracture, within the next 5 years from **10%** to **6%**.

In other words, in a crowd of **1,000** people in the UK, **100** are likely to have a fracture if they do not take the medicine. This is reduced to **60** people out of 1,000 who do take the medicine.
This information is also illustrated in this picture.

Risk of fracture

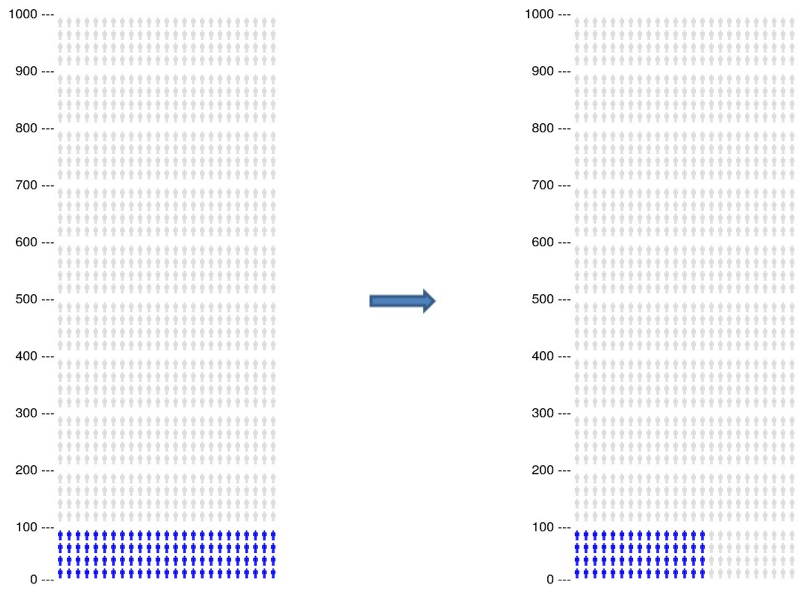

**10%**	**6%**
**No Treatment**	**Taking the Medicine**

(The figures shaded blue represent people who will have a fracture, such as hip fracture, within the next 5 years)

Please continue to the next page.

0% 100%

Repeated section

Remember Medicine A and Medicine B are equally effective.

However, there are some differences between Medicine A and Medicine B.

Medicine A	Medicine B
**No inconvenience**<br><br>You take a tablet only once in your life.<br>You will be given the tablet on the day you come in to see your GP.	**Inconvenience**<br><br>One tablet should be taken once a week on the same day every week.<br>You need to take this medicine for 5 years.<br><br>You need to take the tablet first thing in the morning on an empty stomach with a large glass of tap water.<br>After taking the tablet, you should stay upright for the next 30 minutes.<br><br>You should not eat anything for at least 30 minutes, including taking any other medicines.<br><br>You need to regularly visit your doctor to have your bone health checked.<br>You need to regularly visit a pharmacy for a new supply.
**Severe side effect - 0%**<br><br>No one taking Medicine A will have a severe side effect.	**Severe side effect - 0.7%**<br><br>Among every 1,000 people taking Medicine B, **7** will experience a severe side effect.
**Minor side effect - 0%**<br><br>No one taking Medicine A will have a minor side effect.	**Minor side effect - 13%**<br><br>Among every 1,000 people taking Medicine B, **130** will experience a minor side effect.

If you take Medicine A, you would live for **2.5 years (2 years and 6 months)** and then die.

If you take Medicine B, you would live for **5 years** and then die.

Would you prefer Medicine A or Medicine B, or are they the same?

Indicate your choice here. You can only choose one option:

○ **Medicine A** 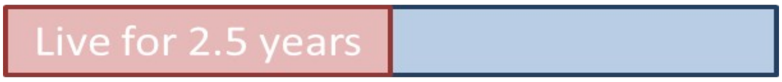

○ **Medicine B** 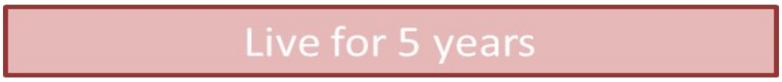

○ **The Same - Medicine A and Medicine B are the same.**

0% 100%

For peer review only

Repeated section

Remember Medicine A and Medicine B are equally effective.

However, there are some differences between Medicine A and Medicine B.

Medicine A	Medicine B
**No inconvenience**<br><br>You take a tablet only once in your life.<br>You will be given the tablet on the day you come in to see your GP.	**Inconvenience**<br><br>One tablet should be taken once a week on the same day every week.<br>You need to take this medicine for 5 years.<br><br>You need to take the tablet first thing in the morning on an empty stomach with a large glass of tap water.<br>After taking the tablet, you should stay upright for the next 30 minutes.<br><br>You should not eat anything for at least 30 minutes, including taking any other medicines.<br><br>You need to regularly visit your doctor to have your bone health checked.<br>You need to regularly visit a pharmacy for a new supply.
**Severe side effect - 0%**<br><br>No one taking Medicine A will have a severe side effect.	**Severe side effect - 0.7%**<br><br>Among every 1,000 people taking Medicine B, **7** will experience a severe side effect.
**Minor side effect - 0%**<br><br>No one taking Medicine A will have a minor side effect.	**Minor side effect - 13%**<br><br>Among every 1,000 people taking Medicine B, **130** will experience a minor side effect.

If you take Medicine A, you would live for **3 years** and then die.

If you take Medicine B, you would live for **5 years** and then die.

Would you prefer Medicine A or Medicine B, or are they the same?

Indicate your choice here. You can only choose one option:

◯ **Medicine A**

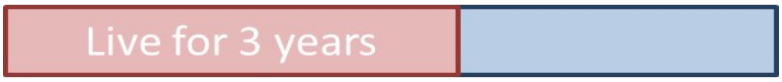

◯ **Medicine B**

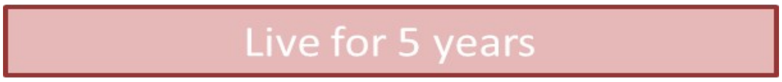

◯ **The Same - Medicine A and Medicine B are the same.**

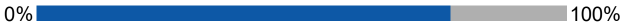

For peer review only

Repeated section

Remember Medicine A and Medicine B are equally effective.

However, there are some differences between Medicine A and Medicine B.

Medicine A	Medicine B
**No inconvenience**<br><br>You take a tablet only once in your life.<br>You will be given the tablet on the day you come in to see your GP.	**Inconvenience**<br><br>One tablet should be taken once a week on the same day every week.<br>You need to take this medicine for 5 years.<br><br>You need to take the tablet first thing in the morning on an empty stomach with a large glass of tap water.<br>After taking the tablet, you should stay upright for the next 30 minutes.<br><br>You should not eat anything for at least 30 minutes, including taking any other medicines.<br><br>You need to regularly visit your doctor to have your bone health checked.<br>You need to regularly visit a pharmacy for a new supply.
**Severe side effect - 0%**<br><br>No one taking Medicine A will have a severe side effect.	**Severe side effect - 0.7%**<br><br>Among every 1,000 people taking Medicine B, **7** will experience a severe side effect.
**Minor side effect - 0%**<br><br>No one taking Medicine A will have a minor side effect.	**Minor side effect - 13%**<br><br>Among every 1,000 people taking Medicine B, **130** will experience a minor side effect.

If you take Medicine A, you would live for **3.25 years** (3 years and 3 months) and then die.

If you take Medicine B, you would live for **5 years** and then die.

Would you prefer Medicine A or Medicine B, or are they the same?

Indicate your choice here. You can only choose one option:

○ **Medicine A**

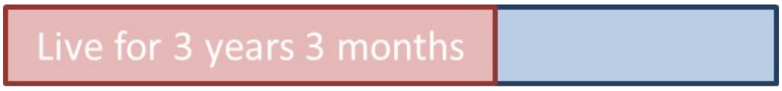

○ **Medicine B**

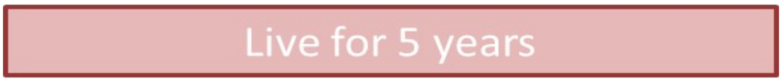

○ **The Same - Medicine A and Medicine B are the same.**

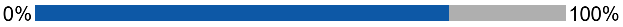

For peer review only

Repeated section

Remember Medicine A and Medicine B are equally effective.

However, there are some differences between Medicine A and Medicine B.

Medicine A	Medicine B
**No inconvenience**<br><br>You take a tablet only once in your life.<br>You will be given the tablet on the day you come in to see your GP.	**Inconvenience**<br><br>One tablet should be taken once a week on the same day every week.<br>You need to take this medicine for 5 years.<br><br>You need to take the tablet first thing in the morning on an empty stomach with a large glass of tap water.<br>After taking the tablet, you should stay upright for the next 30 minutes.<br><br>You should not eat anything for at least 30 minutes, including taking any other medicines.<br><br>You need to regularly visit your doctor to have your bone health checked.<br>You need to regularly visit a pharmacy for a new supply.
**Severe side effect - 0%**<br><br>No one taking Medicine A will have a severe side effect.	**Severe side effect - 0.7%**<br><br>Among every 1,000 people taking Medicine B, **7** will experience a severe side effect.
**Minor side effect - 0%**<br><br>No one taking Medicine A will have a minor side effect.	**Minor side effect - 13%**<br><br>Among every 1,000 people taking Medicine B, **130** will experience a minor side effect.

If you take Medicine A, you would live for **3.5 years** (3 years and 6 months) and then die.

If you take Medicine B, you would live for **5 years** and then die.

Would you prefer Medicine A or Medicine B, or are they the same?

Indicate your choice here. You can only choose one option:

◯ **Medicine A**

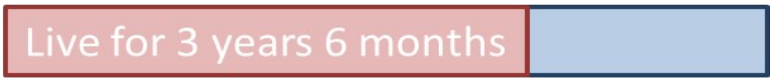

◯ **Medicine B**

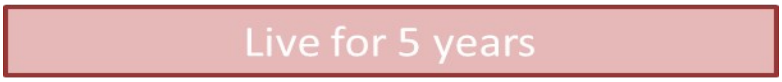

◯ **The Same - Medicine A and Medicine B are the same.**

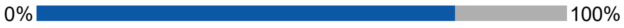

For peer review only

Repeated section

Remember Medicine A and Medicine B are equally effective.

However, there are some differences between Medicine A and Medicine B.

Medicine A	Medicine B
**No inconvenience**<br><br>You take a tablet only once in your life.<br>You will be given the tablet on the day you come in to see your GP.	**Inconvenience**<br><br>One tablet should be taken once a week on the same day every week.<br>You need to take this medicine for 5 years.<br><br>You need to take the tablet first thing in the morning on an empty stomach with a large glass of tap water.<br>After taking the tablet, you should stay upright for the next 30 minutes.<br><br>You should not eat anything for at least 30 minutes, including taking any other medicines.<br><br>You need to regularly visit your doctor to have your bone health checked.<br>You need to regularly visit a pharmacy for a new supply.
**Severe side effect - 0%**<br><br>No one taking Medicine A will have a severe side effect.	**Severe side effect - 0.7%**<br><br>Among every 1,000 people taking Medicine B, **7** will experience a severe side effect.
**Minor side effect - 0%**<br><br>No one taking Medicine A will have a minor side effect.	**Minor side effect - 13%**<br><br>Among every 1,000 people taking Medicine B, **130** will experience a minor side effect.

If you take Medicine A, you would live for **3.75 years** (3 years and 9 months) and then die.

If you take Medicine B, you would live for **5 years** and then die.

Would you prefer Medicine A or Medicine B, or are they the same?

Indicate your choice here. You can only choose one option:

◯ **Medicine A**

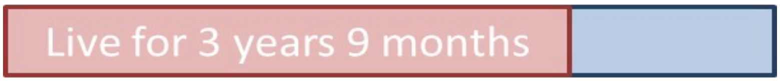

◯ **Medicine B**

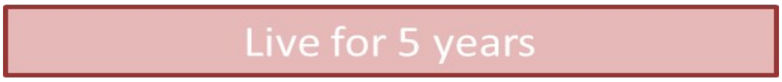

◯ **The Same - Medicine A and Medicine B are the same.**

0% 100%

For peer review only

Repeated section

Remember Medicine A and Medicine B are equally effective.

However, there are some differences between Medicine A and Medicine B.

Medicine A	Medicine B
**No inconvenience**<br><br>You take a tablet only once in your life.<br>You will be given the tablet on the day you come in to see your GP.	**Inconvenience**<br><br>One tablet should be taken once a week on the same day every week.<br>You need to take this medicine for 5 years.<br><br>You need to take the tablet first thing in the morning on an empty stomach with a large glass of tap water.<br>After taking the tablet, you should stay upright for the next 30 minutes.<br><br>You should not eat anything for at least 30 minutes, including taking any other medicines.<br><br>You need to regularly visit your doctor to have your bone health checked.<br>You need to regularly visit a pharmacy for a new supply.
**Severe side effect - 0%**<br><br>No one taking Medicine A will have a severe side effect.	**Severe side effect - 0.7%**<br><br>Among every 1,000 people taking Medicine B, **7** will experience a severe side effect.
**Minor side effect - 0%**<br><br>No one taking Medicine A will have a minor side effect.	**Minor side effect - 13%**<br><br>Among every 1,000 people taking Medicine B, **130** will experience a minor side effect.

If you take Medicine A, you would live for **3 years and 10 months** and then die.

If you take Medicine B, you would live for **5 years** and then die.

Would you prefer Medicine A or Medicine B, or are they the same?

Indicate your choice here. You can only choose one option:

◯ **Medicine A**

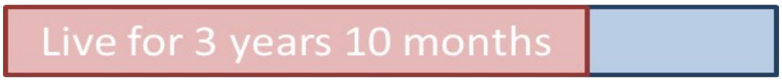

◯ **Medicine B**

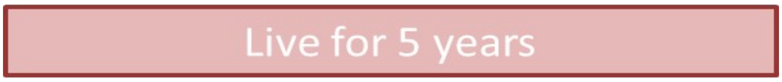

◯ **The Same - Medicine A and Medicine B are the same.**

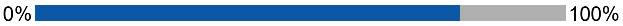

For peer review only

Repeated section

Remember Medicine A and Medicine B are equally effective.

However, there are some differences between Medicine A and Medicine B.

Medicine A	Medicine B
**No inconvenience**<br><br>You take a tablet only once in your life.<br>You will be given the tablet on the day you come in to see your GP.	**Inconvenience**<br><br>One tablet should be taken once a week on the same day every week.<br>You need to take this medicine for 5 years.<br><br>You need to take the tablet first thing in the morning on an empty stomach with a large glass of tap water.<br>After taking the tablet, you should stay upright for the next 30 minutes.<br><br>You should not eat anything for at least 30 minutes, including taking any other medicines.<br><br>You need to regularly visit your doctor to have your bone health checked.<br>You need to regularly visit a pharmacy for a new supply.
**Severe side effect - 0%**<br><br>No one taking Medicine A will have a severe side effect.	**Severe side effect - 0.7%**<br><br>Among every 1,000 people taking Medicine B, **7** will experience a severe side effect.
**Minor side effect - 0%**<br><br>No one taking Medicine A will have a minor side effect.	**Minor side effect - 13%**<br><br>Among every 1,000 people taking Medicine B, **130** will experience a minor side effect.

If you take Medicine A, you would live for **4 years** and then die.

If you take Medicine B, you would live for **5 years** and then die.

Would you prefer Medicine A or Medicine B, or are they the same?

Indicate your choice here. You can only choose one option:

◯ **Medicine A**

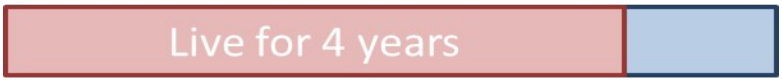

◯ **Medicine B**

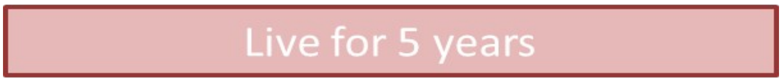

◯ **The Same - Medicine A and Medicine B are the same.**

0% 100%

For peer review only

Repeated section

Remember Medicine A and Medicine B are equally effective.

However, there are some differences between Medicine A and Medicine B.

Medicine A	Medicine B
**No inconvenience**<br><br>You take a tablet only once in your life.<br>You will be given the tablet on the day you come in to see your GP.	**Inconvenience**<br><br>One tablet should be taken once a week on the same day every week.<br>You need to take this medicine for 5 years.<br><br>You need to take the tablet first thing in the morning on an empty stomach with a large glass of tap water.<br>After taking the tablet, you should stay upright for the next 30 minutes.<br><br>You should not eat anything for at least 30 minutes, including taking any other medicines.<br><br>You need to regularly visit your doctor to have your bone health checked.<br>You need to regularly visit a pharmacy for a new supply.
**Severe side effect - 0%**<br><br>No one taking Medicine A will have a severe side effect.	**Severe side effect - 0.7%**<br><br>Among every 1,000 people taking Medicine B, **7** will experience a severe side effect.
**Minor side effect - 0%**<br><br>No one taking Medicine A will have a minor side effect.	**Minor side effect - 13%**<br><br>Among every 1,000 people taking Medicine B, **130** will experience a minor side effect.

If you take Medicine A, you would live for **4 years and 2 months** and then die.

If you take Medicine B, you would live for **5 years** and then die.

Would you prefer Medicine A or Medicine B, or are they the same?

Indicate your choice here. You can only choose one option:

○ **Medicine A** 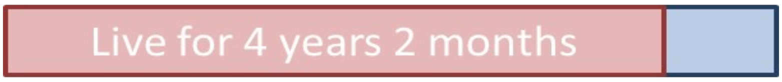

○ **Medicine B** 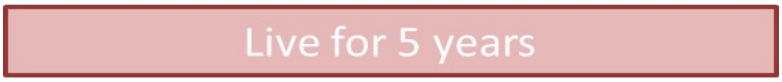

○ **The Same - Medicine A and Medicine B are the same.**

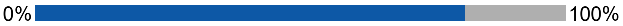

For peer review only

Repeated section

Remember Medicine A and Medicine B are equally effective.

However, there are some differences between Medicine A and Medicine B.

Medicine A	Medicine B
**No inconvenience**<br><br>You take a tablet only once in your life.<br>You will be given the tablet on the day you come in to see your GP.	**Inconvenience**<br><br>One tablet should be taken once a week on the same day every week.<br>You need to take this medicine for 5 years.<br><br>You need to take the tablet first thing in the morning on an empty stomach with a large glass of tap water.<br>After taking the tablet, you should stay upright for the next 30 minutes.<br><br>You should not eat anything for at least 30 minutes, including taking any other medicines.<br><br>You need to regularly visit your doctor to have your bone health checked.<br>You need to regularly visit a pharmacy for a new supply.
**Severe side effect - 0%**<br><br>No one taking Medicine A will have a severe side effect.	**Severe side effect - 0.7%**<br><br>Among every 1,000 people taking Medicine B, **7** will experience a severe side effect.
**Minor side effect - 0%**<br><br>No one taking Medicine A will have a minor side effect.	**Minor side effect - 13%**<br><br>Among every 1,000 people taking Medicine B, **130** will experience a minor side effect.

If you take Medicine A, you would live for **4.25 years** (4 years and 3 months) and then die.

If you take Medicine B, you would live for **5 years** and then die.

Would you prefer Medicine A or Medicine B, or are they the same?

Indicate your choice here. You can only choose one option:

○ **Medicine A** Live for 4 years 3 months

○ **Medicine B** Live for 5 years

○ **The Same - Medicine A and Medicine B are the same.**

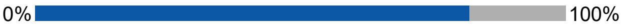

For peer review only

Repeated section

Remember Medicine A and Medicine B are equally effective.

However, there are some differences between Medicine A and Medicine B.

Medicine A	Medicine B
**No inconvenience**<br><br>You take a tablet only once in your life.<br>You will be given the tablet on the day you come in to see your GP.	**Inconvenience**<br><br>One tablet should be taken once a week on the same day every week.<br>You need to take this medicine for 5 years.<br><br>You need to take the tablet first thing in the morning on an empty stomach with a large glass of tap water.<br>After taking the tablet, you should stay upright for the next 30 minutes.<br><br>You should not eat anything for at least 30 minutes, including taking any other medicines.<br><br>You need to regularly visit your doctor to have your bone health checked.<br>You need to regularly visit a pharmacy for a new supply.
**Severe side effect - 0%**<br><br>No one taking Medicine A will have a severe side effect.	**Severe side effect - 0.7%**<br><br>Among every 1,000 people taking Medicine B, **7** will experience a severe side effect.
**Minor side effect - 0%**<br><br>No one taking Medicine A will have a minor side effect.	**Minor side effect - 13%**<br><br>Among every 1,000 people taking Medicine B, **130** will experience a minor side effect.

If you take Medicine A, you would live for **4 years 4 months** and then die.

If you take Medicine B, you would live for **5 years** and then die.

Would you prefer Medicine A or Medicine B, or are they the same?

Indicate your choice here. You can only choose one option:

◯ **Medicine A** Live for 4 years 4 months

◯ **Medicine B** Live for 5 years

◯ **The Same - Medicine A and Medicine B are the same.**

0% 100%

For peer review only

Repeated section

Remember Medicine A and Medicine B are equally effective.

However, there are some differences between Medicine A and Medicine B.

Medicine A	Medicine B
**No inconvenience**<br><br>You take a tablet only once in your life.<br>You will be given the tablet on the day you come in to see your GP.	**Inconvenience**<br><br>One tablet should be taken once a week on the same day every week.<br>You need to take this medicine for 5 years.<br><br>You need to take the tablet first thing in the morning on an empty stomach with a large glass of tap water.<br>After taking the tablet, you should stay upright for the next 30 minutes.<br><br>You should not eat anything for at least 30 minutes, including taking any other medicines.<br><br>You need to regularly visit your doctor to have your bone health checked.<br>You need to regularly visit a pharmacy for a new supply.
**Severe side effect - 0%**<br><br>No one taking Medicine A will have a severe side effect.	**Severe side effect - 0.7%**<br><br>Among every 1,000 people taking Medicine B, **7** will experience a severe side effect.
**Minor side effect - 0%**<br><br>No one taking Medicine A will have a minor side effect.	**Minor side effect - 13%**<br><br>Among every 1,000 people taking Medicine B, **130** will experience a minor side effect.

If you take Medicine A, you would live for **4.5 years** (4 years and 6 months) and then die.

If you take Medicine B, you would live for **5 years** and then die.

Would you prefer Medicine A or Medicine B, or are they the same?

Indicate your choice here. You can only choose one option:

◯ **Medicine A** Live for 4 years 6 months

◯ **Medicine B** Live for 5 years

◯ **The Same - Medicine A and Medicine B are the same.**

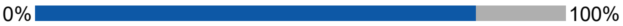

For peer review only

Repeated section

Remember Medicine A and Medicine B are equally effective.

However, there are some differences between Medicine A and Medicine B.

Medicine A	Medicine B
**No inconvenience**<br><br>You take a tablet only once in your life.<br>You will be given the tablet on the day you come in to see your GP.	**Inconvenience**<br><br>One tablet should be taken once a week on the same day every week.<br>You need to take this medicine for 5 years.<br><br>You need to take the tablet first thing in the morning on an empty stomach with a large glass of tap water.<br>After taking the tablet, you should stay upright for the next 30 minutes.<br><br>You should not eat anything for at least 30 minutes, including taking any other medicines.<br><br>You need to regularly visit your doctor to have your bone health checked.<br>You need to regularly visit a pharmacy for a new supply.
**Severe side effect - 0%**<br><br>No one taking Medicine A will have a severe side effect.	**Severe side effect - 0.7%**<br><br>Among every 1,000 people taking Medicine B, **7** will experience a severe side effect.
**Minor side effect - 0%**<br><br>No one taking Medicine A will have a minor side effect.	**Minor side effect - 13%**<br><br>Among every 1,000 people taking Medicine B, **130** will experience a minor side effect.

If you take Medicine A, you would live for **4 years and 7 months** and then die.

If you take Medicine B, you would live for **5 years** and then die.

Would you prefer Medicine A or Medicine B, or are they the same?

Indicate your choice here. You can only choose one option:

◯ **Medicine A** Live for 4 years 7 months

◯ **Medicine B** Live for 5 years

◯ **The Same - Medicine A and Medicine B are the same.**

0% 100%

For peer review only

Repeated section

Remember Medicine A and Medicine B are equally effective.

However, there are some differences between Medicine A and Medicine B.

Medicine A	Medicine B
**No inconvenience**<br><br>You take a tablet only once in your life.<br>You will be given the tablet on the day you come in to see your GP.	**Inconvenience**<br><br>One tablet should be taken once a week on the same day every week.<br>You need to take this medicine for 5 years.<br><br>You need to take the tablet first thing in the morning on an empty stomach with a large glass of tap water.<br>After taking the tablet, you should stay upright for the next 30 minutes.<br><br>You should not eat anything for at least 30 minutes, including taking any other medicines.<br><br>You need to regularly visit your doctor to have your bone health checked.<br>You need to regularly visit a pharmacy for a new supply.
**Severe side effect - 0%**<br><br>No one taking Medicine A will have a severe side effect.	**Severe side effect - 0.7%**<br><br>Among every 1,000 people taking Medicine B, **7** will experience a severe side effect.
**Minor side effect - 0%**<br><br>No one taking Medicine A will have a minor side effect.	**Minor side effect - 13%**<br><br>Among every 1,000 people taking Medicine B, **130** will experience a minor side effect.

If you take Medicine A, you would live for **4 years and 9 months** and then die.

If you take Medicine B, you would live for **5 years** and then die.

Would you prefer Medicine A or Medicine B, or are they the same?

Indicate your choice here. You can only choose one option:

◯ **Medicine A** Live for 4 years 9 months

◯ **Medicine B** Live for 5 years

◯ **The Same - Medicine A and Medicine B are the same.**

0% 100%

For peer review only

Repeated section

Remember Medicine A and Medicine B are equally effective.

However, there are some differences between Medicine A and Medicine B.

Medicine A	Medicine B
**No inconvenience**<br><br>You take a tablet only once in your life.<br>You will be given the tablet on the day you come in to see your GP.	**Inconvenience**<br><br>One tablet should be taken once a week on the same day every week.<br>You need to take this medicine for 5 years.<br><br>You need to take the tablet first thing in the morning on an empty stomach with a large glass of tap water.<br>After taking the tablet, you should stay upright for the next 30 minutes.<br><br>You should not eat anything for at least 30 minutes, including taking any other medicines.<br><br>You need to regularly visit your doctor to have your bone health checked.<br>You need to regularly visit a pharmacy for a new supply.
**Severe side effect - 0%**<br><br>No one taking Medicine A will have a severe side effect.	**Severe side effect - 0.7%**<br><br>Among every 1,000 people taking Medicine B, **7** will experience a severe side effect.
**Minor side effect - 0%**<br><br>No one taking Medicine A will have a minor side effect.	**Minor side effect - 13%**<br><br>Among every 1,000 people taking Medicine B, **130** will experience a minor side effect.

If you take Medicine A, you would live for **2 months less than 5 years** and then die.

If you take Medicine B, you would live for **5 years** and then die.

Would you prefer Medicine A or Medicine B, or are they the same?

Indicate your choice here. You can only choose one option:

○ **Medicine A** Live for (5 years – 2 months)

○ **Medicine B** Live for 5 years

○ **The Same - Medicine A and Medicine B are the same.**

0% 100%

For peer review only

Repeated section

Remember Medicine A and Medicine B are equally effective.

However, there are some differences between Medicine A and Medicine B.

Medicine A	Medicine B
**No inconvenience**<br><br>You take a tablet only once in your life.<br>You will be given the tablet on the day you come in to see your GP.	**Inconvenience**<br><br>One tablet should be taken once a week on the same day every week.<br>You need to take this medicine for 5 years.<br><br>You need to take the tablet first thing in the morning on an empty stomach with a large glass of tap water.<br>After taking the tablet, you should stay upright for the next 30 minutes.<br><br>You should not eat anything for at least 30 minutes, including taking any other medicines.<br><br>You need to regularly visit your doctor to have your bone health checked.<br>You need to regularly visit a pharmacy for a new supply.
**Severe side effect - 0%**<br><br>No one taking Medicine A will have a severe side effect.	**Severe side effect - 0.7%**<br><br>Among every 1,000 people taking Medicine B, **7** will experience a severe side effect.
**Minor side effect - 0%**<br><br>No one taking Medicine A will have a minor side effect.	**Minor side effect - 13%**<br><br>Among every 1,000 people taking Medicine B, **130** will experience a minor side effect.

If you take Medicine A, you would live for **1 month less than 5 years** and then die.

If you take Medicine B, you would live for **5 years** and then die.

Would you prefer Medicine A or Medicine B, or are they the same?

Indicate your choice here. You can only choose one option:

○ **Medicine A** 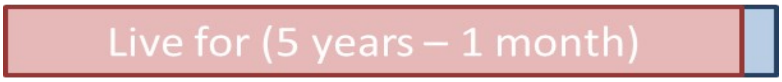

○ **Medicine B** 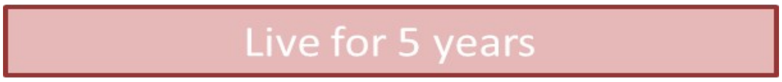

○ **The Same - Medicine A and Medicine B are the same.**

0% 100%

For peer review only

Repeated section

Remember Medicine A and Medicine B are equally effective.

However, there are some differences between Medicine A and Medicine B.

Medicine A	Medicine B
**No inconvenience**<br><br>You take a tablet only once in your life.<br>You will be given the tablet on the day you come in to see your GP.	**Inconvenience**<br><br>One tablet should be taken once a week on the same day every week.<br>You need to take this medicine for 5 years.<br><br>You need to take the tablet first thing in the morning on an empty stomach with a large glass of tap water.<br>After taking the tablet, you should stay upright for the next 30 minutes.<br><br>You should not eat anything for at least 30 minutes, including taking any other medicines.<br><br>You need to regularly visit your doctor to have your bone health checked.<br>You need to regularly visit a pharmacy for a new supply.
**Severe side effect - 0%**<br><br>No one taking Medicine A will have a severe side effect.	**Severe side effect - 0.7%**<br><br>Among every 1,000 people taking Medicine B, **7** will experience a severe side effect.
**Minor side effect - 0%**<br><br>No one taking Medicine A will have a minor side effect.	**Minor side effect - 13%**<br><br>Among every 1,000 people taking Medicine B, **130** will experience a minor side effect.

If you take Medicine A, you would live for **2 weeks less than 5 years** and then die.

If you take Medicine B, you would live for **5 years** and then die.

Would you prefer Medicine A or Medicine B, or are they the same?

Indicate your choice here. You can only choose one option:

◯ **Medicine A** Live for (5 years – 2 weeks)

◯ **Medicine B** Live for 5 years

◯ **The Same - Medicine A and Medicine B are the same.**

0% 100%

For peer review only

Repeated section

Remember Medicine A and Medicine B are equally effective.

However, there are some differences between Medicine A and Medicine B.

Medicine A	Medicine B
**No inconvenience**<br><br>You take a tablet only once in your life.<br>You will be given the tablet on the day you come in to see your GP.	**Inconvenience**<br><br>One tablet should be taken once a week on the same day every week.<br>You need to take this medicine for 5 years.<br><br>You need to take the tablet first thing in the morning on an empty stomach with a large glass of tap water.<br>After taking the tablet, you should stay upright for the next 30 minutes.<br><br>You should not eat anything for at least 30 minutes, including taking any other medicines.<br><br>You need to regularly visit your doctor to have your bone health checked.<br>You need to regularly visit a pharmacy for a new supply.
**Severe side effect - 0%**<br><br>No one taking Medicine A will have a severe side effect.	**Severe side effect - 0.7%**<br><br>Among every 1,000 people taking Medicine B, **7** will experience a severe side effect.
**Minor side effect - 0%**<br><br>No one taking Medicine A will have a minor side effect.	**Minor side effect - 13%**<br><br>Among every 1,000 people taking Medicine B, **130** will experience a minor side effect.

If you take Medicine A, you would live for **1 week less than 5 years** and then die.

If you take Medicine B, you would live for **5 years** and then die.

Would you prefer Medicine A or Medicine B, or are they the same?

Indicate your choice here. You can only choose one option:

○ **Medicine A** Live for (5 years – 1 week)

○ **Medicine B** Live for 5 years

○ **The Same - Medicine A and Medicine B are the same.**

0% 100%

For peer review only

Repeated section

Remember Medicine A and Medicine B are equally effective.

However, there are some differences between Medicine A and Medicine B.

Medicine A	Medicine B
**No inconvenience**<br><br>You take a tablet only once in your life.<br>You will be given the tablet on the day you come in to see your GP.	**Inconvenience**<br><br>One tablet should be taken once a week on the same day every week.<br>You need to take this medicine for 5 years.<br><br>You need to take the tablet first thing in the morning on an empty stomach with a large glass of tap water.<br>After taking the tablet, you should stay upright for the next 30 minutes.<br><br>You should not eat anything for at least 30 minutes, including taking any other medicines.<br><br>You need to regularly visit your doctor to have your bone health checked.<br>You need to regularly visit a pharmacy for a new supply.
**Severe side effect - 0%**<br><br>No one taking Medicine A will have a severe side effect.	**Severe side effect - 0.7%**<br><br>Among every 1,000 people taking Medicine B, **7** will experience a severe side effect.
**Minor side effect - 0%**<br><br>No one taking Medicine A will have a minor side effect.	**Minor side effect - 13%**<br><br>Among every 1,000 people taking Medicine B, **130** will experience a minor side effect.

If you take Medicine A, you would live for **5 days less than 5 years** and then die.

If you take Medicine B, you would live for **5 years** and then die.

Would you prefer Medicine A or Medicine B, or are they the same?

Indicate your choice here. You can only choose one option:

○ **Medicine A** — Live for (5 years – 5 days)

○ **Medicine B** — Live for 5 years

○ **The Same - Medicine A and Medicine B are the same.**

0% 100%

For peer review only

Repeated section

Remember Medicine A and Medicine B are equally effective.

However, there are some differences between Medicine A and Medicine B.

Medicine A	Medicine B
**No inconvenience**<br><br>You take a tablet only once in your life.<br>You will be given the tablet on the day you come in to see your GP.	**Inconvenience**<br><br>One tablet should be taken once a week on the same day every week.<br>You need to take this medicine for 5 years.<br><br>You need to take the tablet first thing in the morning on an empty stomach with a large glass of tap water.<br>After taking the tablet, you should stay upright for the next 30 minutes.<br><br>You should not eat anything for at least 30 minutes, including taking any other medicines.<br><br>You need to regularly visit your doctor to have your bone health checked.<br>You need to regularly visit a pharmacy for a new supply.
**Severe side effect - 0%**<br><br>No one taking Medicine A will have a severe side effect.	**Severe side effect - 0.7%**<br><br>Among every 1,000 people taking Medicine B, **7** will experience a severe side effect.
**Minor side effect - 0%**<br><br>No one taking Medicine A will have a minor side effect.	**Minor side effect - 13%**<br><br>Among every 1,000 people taking Medicine B, **130** will experience a minor side effect.

If you take Medicine A, you would live for **3 days less than 5 years** and then die.

If you take Medicine B, you would live for **5 years** and then die.

Would you prefer Medicine A or Medicine B, or are they the same?

Indicate your choice here. You can only choose one option:

◯ **Medicine A** Live for (5 years – 3 days)

◯ **Medicine B** Live for 5 years

◯ **The Same - Medicine A and Medicine B are the same.**

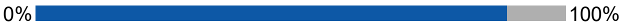

For peer review only

Repeated section

Remember Medicine A and Medicine B are equally effective.

However, there are some differences between Medicine A and Medicine B.

Medicine A	Medicine B
**No inconvenience**<br><br>You take a tablet only once in your life.<br>You will be given the tablet on the day you come in to see your GP.	**Inconvenience**<br><br>One tablet should be taken once a week on the same day every week.<br>You need to take this medicine for 5 years.<br><br>You need to take the tablet first thing in the morning on an empty stomach with a large glass of tap water.<br>After taking the tablet, you should stay upright for the next 30 minutes.<br><br>You should not eat anything for at least 30 minutes, including taking any other medicines.<br><br>You need to regularly visit your doctor to have your bone health checked.<br>You need to regularly visit a pharmacy for a new supply.
**Severe side effect - 0%**<br><br>No one taking Medicine A will have a severe side effect.	**Severe side effect - 0.7%**<br><br>Among every 1,000 people taking Medicine B, **7** will experience a severe side effect.
**Minor side effect - 0%**<br><br>No one taking Medicine A will have a minor side effect.	**Minor side effect - 13%**<br><br>Among every 1,000 people taking Medicine B, **130** will experience a minor side effect.

If you take Medicine A, you would live for **1 day less than 5 years** and then die.

If you take Medicine B, you would live for **5 years** and then die.

Would you prefer Medicine A or Medicine B, or are they the same?

Indicate your choice here. You can only choose one option:

◯ **Medicine A** Live for (5 years – 1 day)

◯ **Medicine B** Live for 5 years

◯ **The Same - Medicine A and Medicine B are the same.**

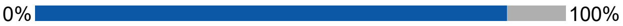

For peer review only

Question 5a.

You prefer living for 2.5 years (2 years and 6 months) on Medicine A, to living for 5 years on Medicine B.

This means **living fewer than 2.5 years on Medicine A** can be the same as living for 5 years on Medicine B.

How many years on Medicine A do you think would be the same as living for 5 years on Medicine B?

(Please give a number in years)

0% 100%

peer review only

Thank you for completing this section and indicating your choices.

Was there anything in this section which you think is unclear or that you didn't understand?

(Please provide details in the comment box below)

0% 100%

For peer review only

Part 3. Questions about you

We understand different people feel differently about different treatments.

In this part of the survey, we ask questions about you to understand if people's age, gender or experiences explain the choices made in Part 2.

0% 100%

For peer review only

We would like to learn about your experience.

Are you currently taking, or have you ever taken any of these medicines (bisphosphonates)?

Aclasta®
Actonel®
Alendronic acid
Binosto®
Bondronat®
Bonefos®
Bonviva®
Clasteon®
Fosamax®
Iasibon®
Ibandronic acid
Loron®
Pamidronate disodium
Quodixor®
Risedronate sodium
Sodium clodronate
Zerlinda®
Zoledronic acid
Zometa®

◯ Yes
◯ No

0% 100%

nly

What do you think would be the **benefits** from taking this medicine (a bisphosphonate)?

What do you think would be the **harms** from taking this medicine (a bisphosphonate)?

Have you experienced any side effects from taking this medicine (a bisphosphonate)?

- ◯ Yes
- ◯ No

Do you find it inconvenient to take this medicine (a bisphosphonate)?
Please specify if you think it is inconvenient.

What is your opinion about taking this medicine (a bisphosphonate)?

- ◯ I don't mind taking them because I have to take them for a reason.
- ◯ I do not like taking them because they remind me that I am not perfectly healthy.
- ◯ I have no opinion.
- ◯ Other (please specify)

0% 100%

Do you take other medicines prescribed by your doctor?

- ◯ Yes
- ◯ No

In total, how many pills do you take on a regular basis?
(Please do not include pills you take only once in a while)

- ◯ None
- ◯ One
- ◯ Two to five
- ◯ Six to ten
- ◯ More than ten

How many different times a day do you regularly take pills?

- ◯ None
- ◯ Once per day
- ◯ 2 times a day
- ◯ 3 times a day
- ◯ More than 3 times a day

Do you find it inconvenient to take this medicine?
Please specify if you think it is inconvenient.

What is your opinion about taking this medicine?

- ◯ I don't mind taking them because I have to take them for a reason.
- ◯ I do not like taking them because they remind me that I am not perfectly healthy.
- ◯ I have no opinion.
- ◯ Other (please specify)

For peer review only

We would like to ask questions about you to better understand your choices.

What is your gender?

- ◯ Male
- ◯ Female
- ◯ Other (please specify) [ ]

Which age category do you fall into?

- ◯ 34 years or younger
- ◯ 35-44 years
- ◯ 45-54 years
- ◯ 55-64 years
- ◯ 65-74 years
- ◯ 75-84 years
- ◯ 85 years or over

Which best reflects your ethnic group?

- ◯ White British/Irish
- ◯ White other
- ◯ Mixed/Multiple ethnic origins
- ◯ Black/African/Caribbean/Black British
- ◯ Asian/Asian British
- ◯ Other (please specify) [ ]

0% 100%

What is your occupational status?

- ◯ Employed full-time
- ◯ Employed part-time
- ◯ Self-employed
- ◯ Unemployed
- ◯ Retired
- ◯ Looking after a home/family
- ◯ Student
- ◯ Freelance or temping
- ◯ Long-term sickness

Could you tell us what your occupation is?
(Previous occupation if retired.)

What is the highest level of education you have obtained?
(for example, GCSEs, Degrees, etc.)

What is your religion?

- ◯ No religion
- ◯ Christian
- ◯ Buddhist
- ◯ Jewish
- ◯ Hindu
- ◯ Muslim
- ◯ Sikh
- ◯ Other

0% 100%

How do you feel about numbers?

This section will ask you some questions to understand how familiar you are with numbers and probabilities.

If you are unsure of the answer, you can leave the response blank.

0% 100%

For peer review only

Question 1

Imagine I flip a coin 1,000 times.

What is your best guess about how many times the coin would fall heads up in 1,000 flips?

- ◯ Answer:
- ◯ Prefer not to answer
- ◯ Don't know.

Question 2

If there was a lottery where the chance of winning £10 is 1%, if 1,000 people bought a ticket, how many would you expect to win?

- ◯ Answer:
- ◯ Prefer not to answer
- ◯ Don't know.

Question 3

In another lottery, the chance of winning a car is 1 in 1,000.

What percentage of tickets in the lottery will win a car?

- ◯ Answer: %
- ◯ Prefer not to answer
- ◯ Don't know

0% 100%

How confident are you that you would answer the questions the same if faced with the situations in real-life?

	Very confident	Confident	Not sure	Not confident	Not confident at all	
Confident	◯	◯	◯	◯	◯	Not confident at all

How easy or difficult did you find making choices between alternatives?

	Very easy	Easy	Average	Somewhat difficult	Very difficult	
Easy	◯	◯	◯	◯	◯	Difficult

How easy or difficult did you find the survey to understand?

	Very easy	Easy	Average	Somewhat difficult	Very difficult	
Easy	◯	◯	◯	◯	◯	Difficult

0% 100%

eview only

Was there anything in the survey that was particularly unclear?

(Please write in the box below)

Is there anything else about the survey we could change to make it easier to uncerstand (for example, phrasing and explanation)?

(Optional) If you have any further comments or suggestions, please write them here.

0% 100%

y only

Thank you for completing this survey

If you would like more information about osteoporosis please visit the NHS Choices website here.

0% 100%

For peer review only

Note:

When respondents take the survey in regular mode this page will not be displayed. Respondents will be redirected to the link below:

https://dkr1.ssisurveys.com/projects/end?rst=1&psid= [Script] &basic=99423

0% 100%

For peer review only

Note:

When respondents take the survey in regular mode this page will not be displayed. Respondents will be redirected to the link below:

https://dkr1.ssisurveys.com/projects/end?rst=2&psid= [Script]

0% 100%

For peer review only

Note:

When respondents take the survey in regular mode this page will not be displayed. Respondents will be redirected to the link below:

https://dkr1.ssisurveys.com/projects/end?rst=3&psid= [Script]

0% 100%

For peer review only

Supplementary Appendix 1: The statin survey

See separate pdf file

For peer review only

Supplementary Appendix 2: The bisphosphonate survey

See separate pdf file

For peer review only

Supplementary Appendix 3: Training materials for statin survey

Statins for the prevention of CVD

Bobbie has been to see a doctor.

The doctor has said Bobbie needs to start a medicine to prevent serious events because of his heart disease.

We will take you through Bobbie's story.

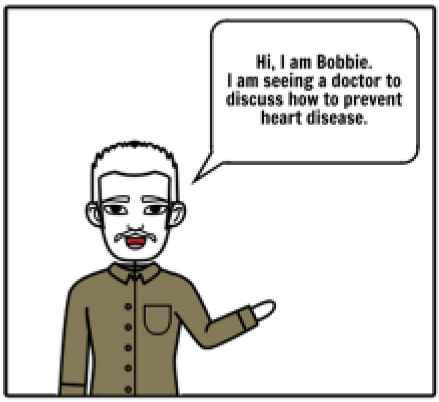

What is heart disease?

Bobbie hears about heart disease from his GP.

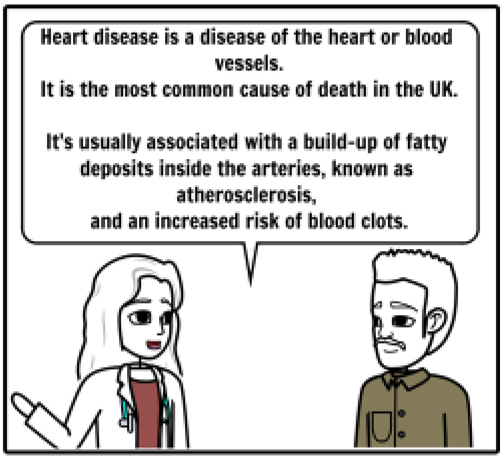

Impact of heart disease

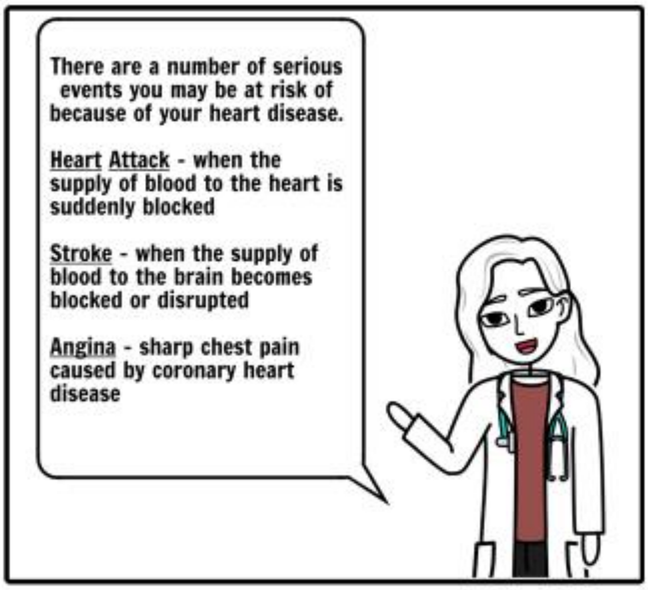

Risk of Heart disease

The GP explains that there is a chance that because of his cholesterol level Bobbie is at risk of developing one of these serious events in the future.

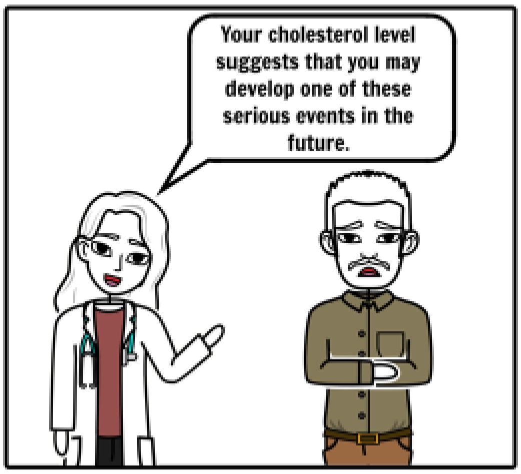

What is risk?

- **Risk** is: a term used to explain the chance that something bad might happen.

- **10% risk** means:

  Out of every **1,000** people, with heart disease, **100** people would develop a heart attack, and **900** people would not.

What is a 10% risk?

- This diagram shows a 10% risk of having a heart attack because of their heart disease.

- The people shaded blue are the ones who have a heart attack.

10% risk

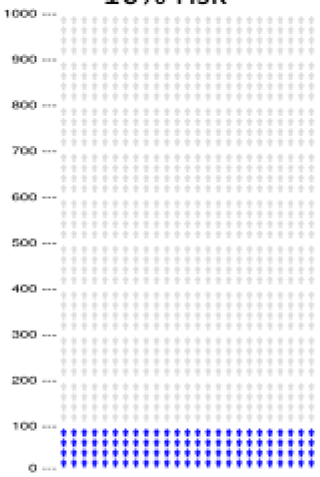

Prevention of serious events from heart disease

- Bobbie's doctor can estimate how likely it is that he will develop heart disease over the next 10 years.
- The estimate is based on things such as Bobbie's age and sex, family medical history, blood pressure and cholesterol level.
- The doctor may prescribe certain medicines such as statins to help reduce the chance of Bobbie having heart disease.
- The medicine will prevent some of the people who take it from having a serious event because of the heart disease.
- A serious event from the heart disease will still happen to some of the people who take the medicine.

Prevention of serious events from heart disease

The doctor prescribes Bobbie a medicine to reduce the chance of his heart disease causing a heart attack or other heart conditions in the future.

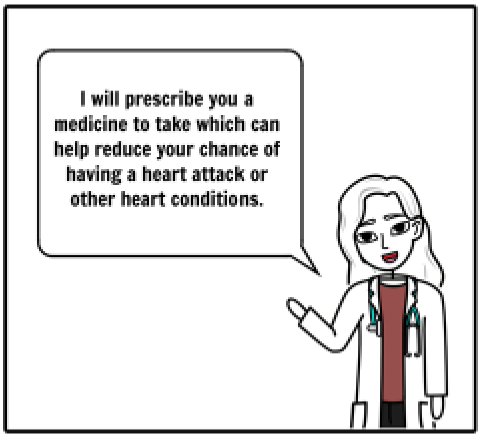

Collecting the medicine from the pharmacy

Bobbie goes to a pharmacy to collect his medicine.

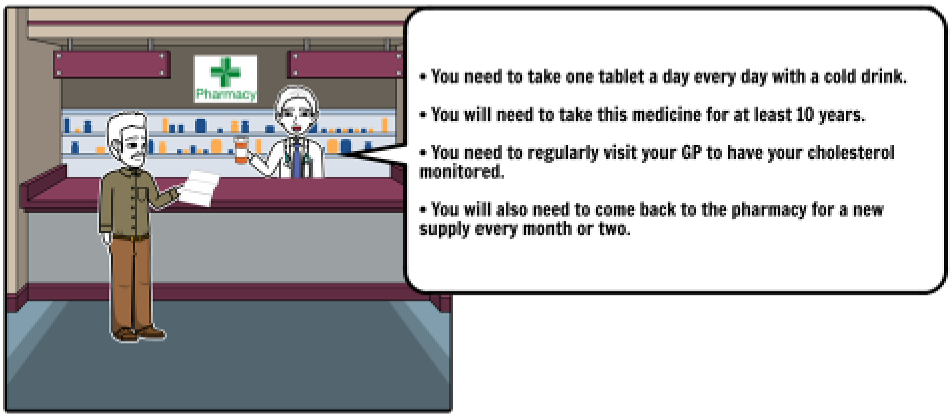

Taking the medicine for prevention

Bobbie goes home with his new tablets.

He then has to decide whether he wants to take them as the doctor and the pharmacist have advised.

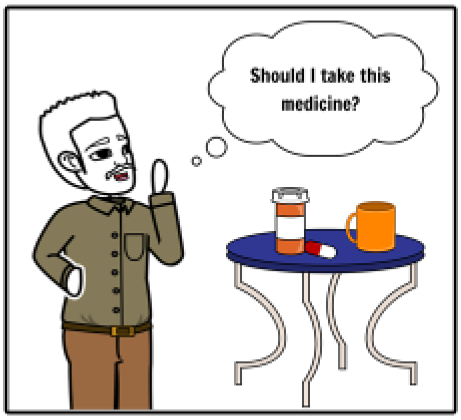

- Bobbie may consider three key things, when deciding whether to take the medicine.

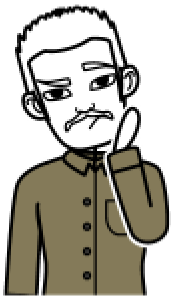

Effectiveness of the medicine

- How good the medicine is in terms of preventing a condition like a heart attack

Side effects of the medicine

- Whether there is any potential harm from the medicine

Inconvenience of the medicine

- Whether taking the medicine would fit in his lifestyle or cause him inconvenience

How effective is the medicine?

- Some people, but not everyone, who take the medicine will avoid a serious event, such as a heart attack or stroke as a result of taking the tablet.
- The effectiveness of the medicine can be described in terms of a reduction in this risk.

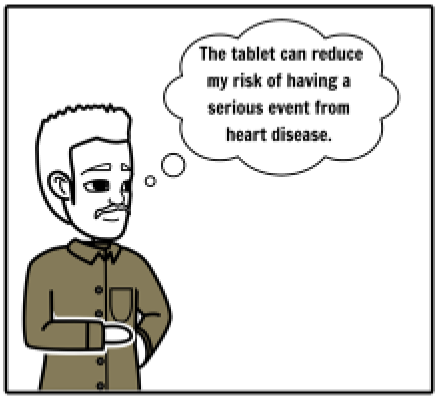

What does ‘reduction in risk’ mean?

- A ‘reduction in risk from 10% to 6%’ means:

The medicine will reduce your risk of experiencing an event, such as a heart attack, within the next 10 years from 10% to 6%.

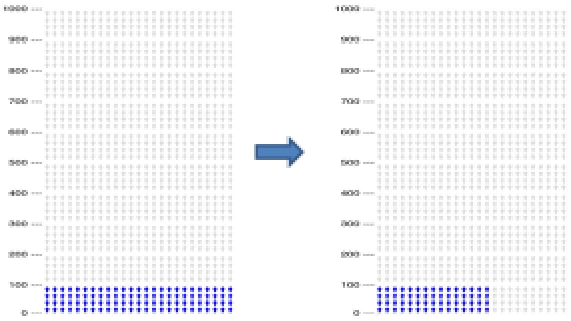

No treatment: 10% risk

Taking the medicine: 6% risk

Does the medicine have side effects?

The potential for side effects from the medicine can be described in terms of the risk of a side effect.

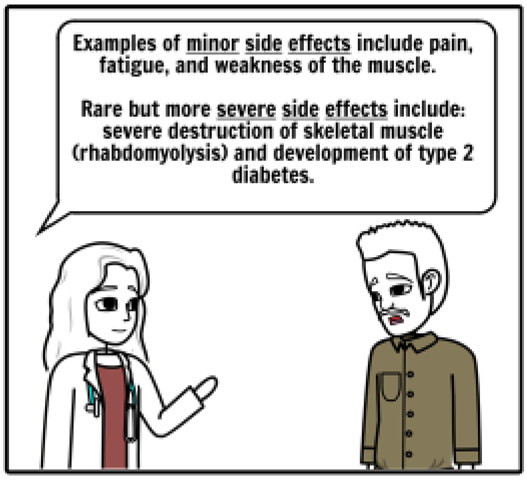

Risk of a minor side effect from the medicine

- A 5% risk of a **minor** side effect means:

  Among every 1,000 people taking the medicine,
  50 will experience a minor side effect such as either: pain, fatigue, and weakness of the muscle.

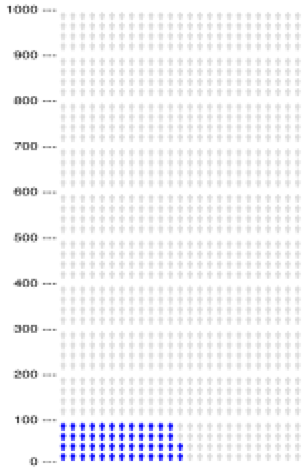

5% risk of a minor side effect

Risk of a severe side effect from the medicine

- A 0.1% risk of a **severe** side effect means:

  Among every 1,000 people taking the medicine,
  1 will experience a severe side effect such as either: severe destruction of skeletal muscle or development of type 2 diabetes.

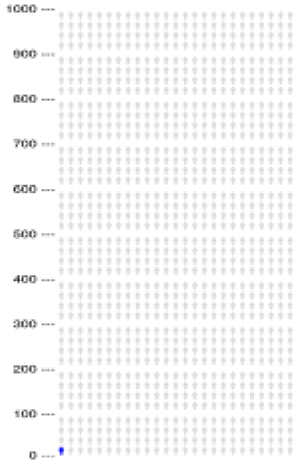

0.1% risk of a severe side effect

How inconvenient is it when taking the medicine?

Bobbie also thinks about what it means to have to take this medicine for the next ten years.

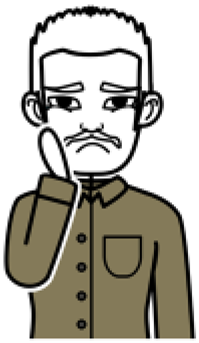

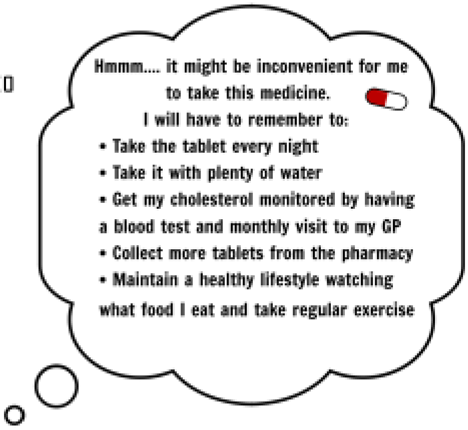

In the questions that follow in this survey, we will ask you about how you think about taking a medicine to prevent heart disease.

Supplementary Appendix 4: Training materials for bisphosphonates survey

Bisphosphonates for the prevention of osteoporotic fractures

Alex has been to see a doctor who told her that she has weak bones.

The doctor has said Alex needs to start a medicine to prevent broken bones (fractures) because of her weak bones.

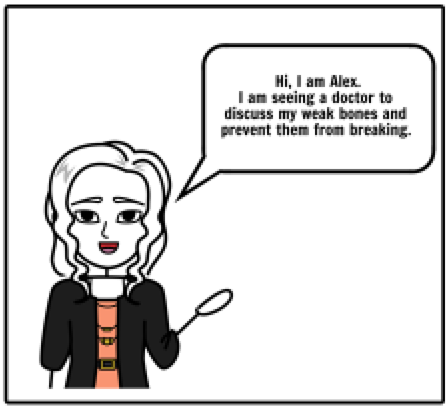

We will take you through Alex's story.

What is osteoporosis?

Alex hears about osteoporosis from her GP.

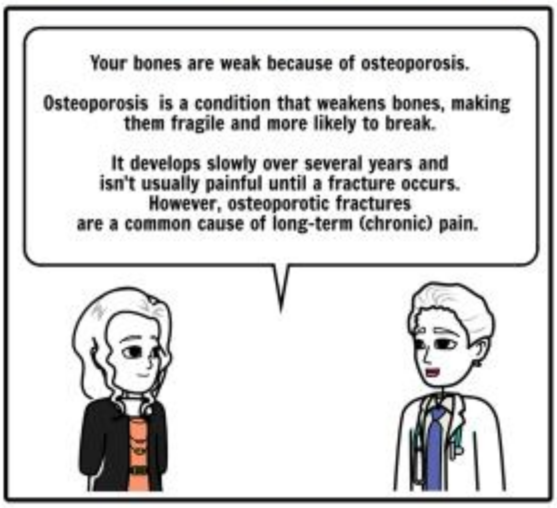

Impact of osteoporosis

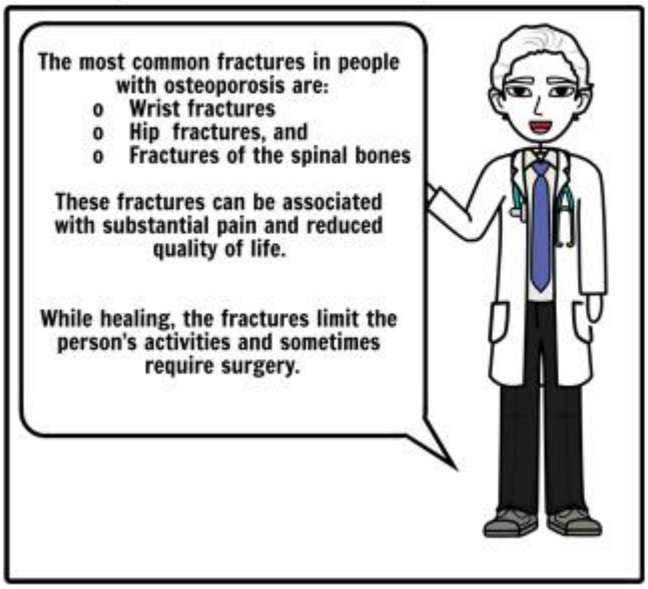

Risk of osteoporotic fractures

Her GP explains that Alex has some risk of fracture as her family has a history of hip fracture.
Also, Alex has a low body mass index (BMI). BMI is a measure of body fat.

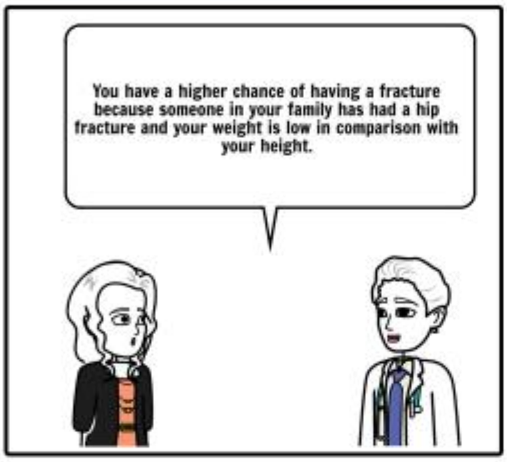

What is risk?

- **Risk** is: a term used to explain the chance that something bad might happen.

- **10% risk** means:

  Out of every **1,000** people, with osteoporosis, **100** people would have a fracture, and **900** people would not.

What is a 10% risk?

- This diagram shows a 10% risk of having a fracture because of their osteoporosis.

- The people shaded blue are the ones who have a fracture.

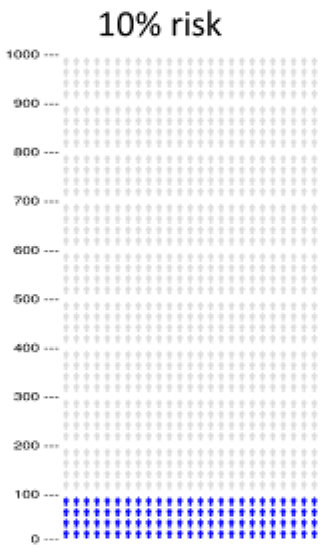

Prevention of fracture from osteoporosis

- Alex's doctor can estimate how likely it is that she will experience osteoporotic fracture over the next 5 years.
- The estimate is based on things such as Alex's age and sex, ethnicity, family fracture history, and body mass index.
- The doctor may prescribe certain medicines such as bisphosphonates to help reduce the chance of Alex having a fracture.
- The medicine will prevent some of the people who take it from having a fracture because of osteoporosis.
- Fractures may still happen in some of the people who take the medicine.

Prevention of fractures from osteoporosis

The doctor prescribes Alex a medicine to reduce the risk of her having a fracture in the future because of her osteoporosis.

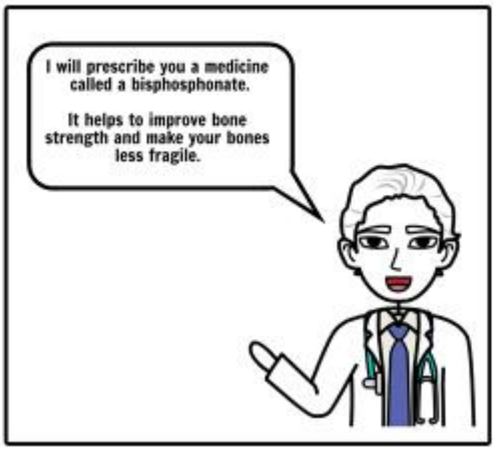

Collecting the medicine from the pharmacy

The pharmacist explains to Alex how to take the medicine.

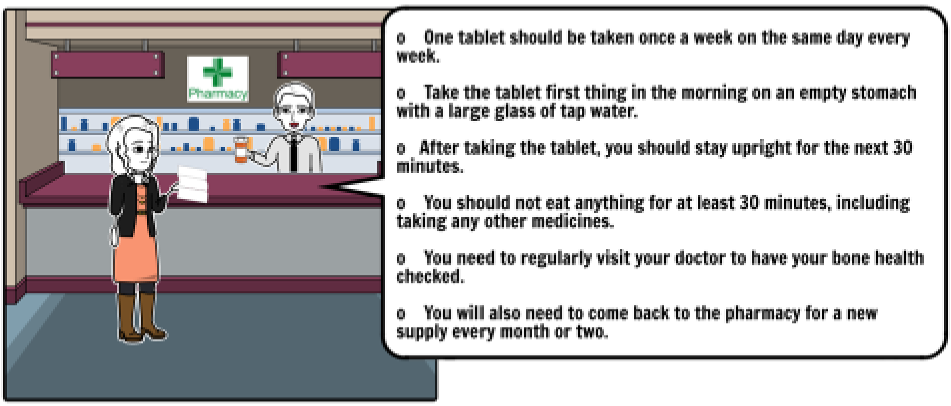

Taking the medicine for prevention

Alex goes home with her new tablets.

She then considers whether she wants to take them as the doctor and the pharmacist have advised.

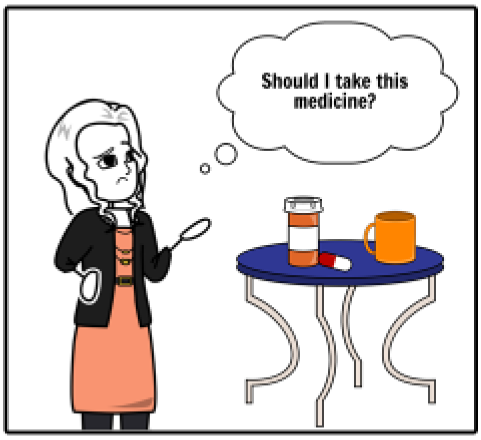

Alex may consider *three key things*,
when deciding whether to take the medicine.

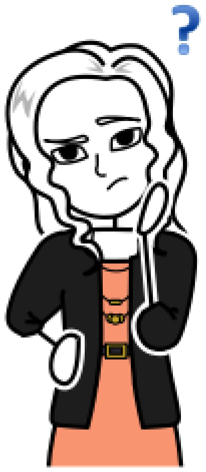

Effectiveness of the medicine

- How good the medicine is in terms of preventing a fracture from osteoporosis

Side effects of the medicine

- Whether there is any potential harm from the medicine

Inconvenience of the medicine

- Whether taking the medicine would fit in her lifestyle or cause her inconvenience

How effective is the medicine?

- Some people, but not everyone, who take the medicine will avoid a serious event, such as hip fracture, as a result of taking the tablet.
- The effectiveness of the medicine can be described in terms of a reduction in this risk.

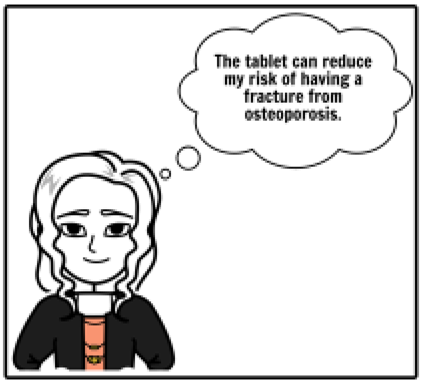

What does 'reduction in risk' mean?

- A 'reduction in risk from 10% to 6%' means:

The medicine will reduce your risk of experiencing a fracture, such as hip fracture, within the next 5 years from 10% to 6%.

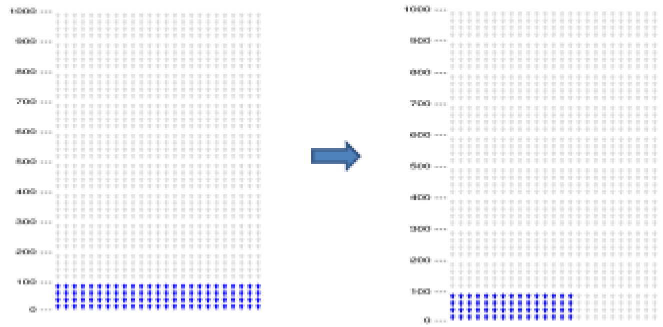

No treatment: 10% risk → Taking the medicine: 6% risk

Does the medicine have side effects?

The potential for side effects from the medicine can be described in terms of the **risk of a side effect**.

Alex hears about minor but more common side effects & severe but rarer side effects from her GP.

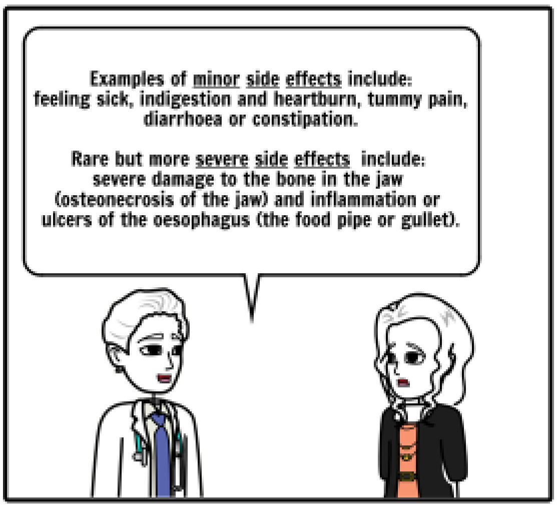

Risk of a minor side effect from the medicine

- A 5% risk of a minor side effect means:

  Among every **1,000** people taking the medicine,
  50 will experience a minor side effect such as either: feeling sick, indigestion, diarrhoea or constipation.

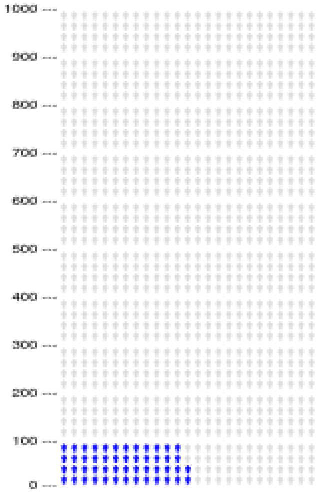

5% risk of a side effect

Risk of a severe side effect from the medicine

- A 0.3% risk of severe side effect means:

  Among every **1,000** people taking the medicine,
  3 will experience a severe side effect such as severe loss or destruction of the bone in the jaw.

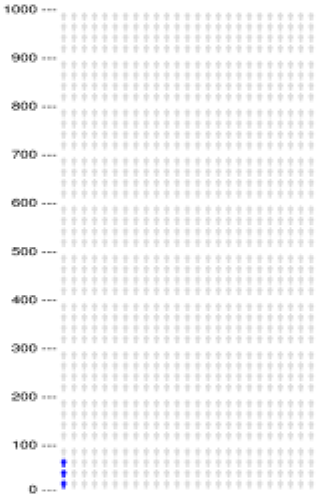

0.3% risk of a side effect

How inconvenient is it when taking the medicine?

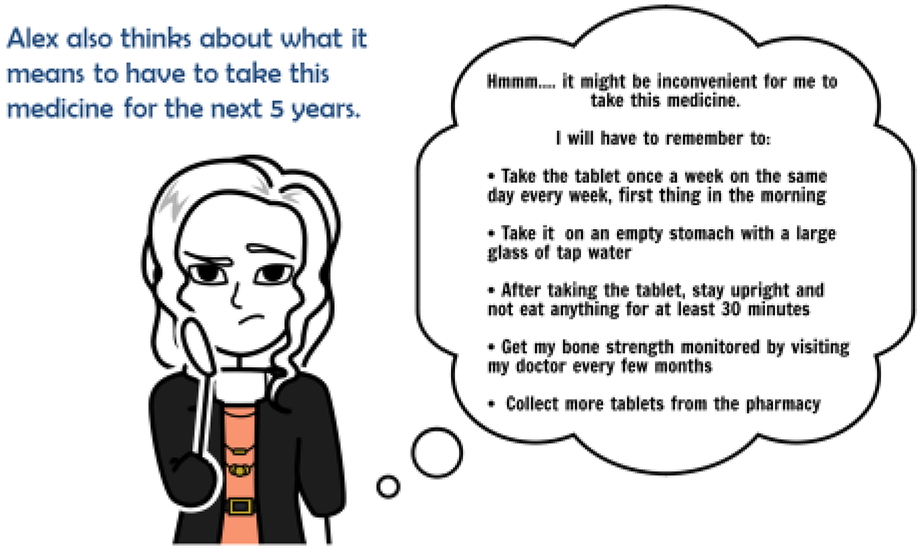

In the questions that follow in this survey, we will ask you about how you think about taking a medicine to prevent osteoporotic fracture.

Supplementary Appendix 5: Stata 'do' file

```
****** ANALYSIS AND TABLES ******
/*
## ---------------------------
##
## Script name: script-01
##
## Purpose of script: Analyse data from DTD study
##
## Author: Dr. Alex Thompson
##
## Date Created: 07/08/2021
##
##
## Email: alexander.thompson@manchester.ac.uk
##
## ---------------------------
##
## Notes:
##
##
## ---------------------------
*/

clear all                                   // clears memory of everything
global location 1                           //
set cformat %5.3f                           // controls the output of tables so coefficients are rounded.

if ${location}==1 global cf `"xxxxxxxxxx"'

cd "${cf}"                                  // changes the working directory

** Location settings **
if ${location}==1 global sf `"xxxxxxx"'
if ${location}==1 global af `"xxxxxxx"'
if ${location}==1 global gf `"xxxxxxx"'
if ${location}==1 global cf `"xxxxxxx"'

global data              `"${sf}"'
global combined          `"${cf}"'
global save              `"${af}"'
global output            `"${gf}"'

cd "${combined}"

*** Prepare data ***

use statins.dta, clear

append using bisphos.dta, force
gen id = _n

gen patient = 1 if survey ==2 | survey==3
replace patient = 0 if patient==.

gen public = 1 if survey==1
replace public = 0 if public==.

drop if prescribed ==2 & patient==1        // People in 'patient population' not prescribed drugs by doctor

bysort id : gen howmany=_N

save combined.dta, replace
```

For peer review only

```
gen one = 1 if utility_q1==1

replace one = 0 if utility_q1!=1

gen zero = 1 if utility_q1==0.5

replace zero = 0 if utility_q1!=0.5

sum zero

save combined.dta, replace

**********************
*** TABLE 1 OUTPUTS ***
**********************


baselinetable       age sex ethnicity employment_status education1 religion ///
                    prescribed other_medicines ///
                    eq5d3l_u(cts) attitude  inconvenient opinion ///
                     other_pill_number other_pill_times if statins ==1  ///
                    , by(public , totalcolumn) ///
                    pcformat(%5.1f) meanformat(%5.3f) sdformat(%5.1f)  ///
                    exportexcel("${output}/sumstats_statins.xls", replace) reportmissing

baselinetable       age sex ethnicity employment_status education1 religion ///
                    prescribed other_medicines ///
                    eq5d3l_u(cts) attitude  inconvenient opinion ///
                     other_pill_number other_pill_times if statins ==0  ///
                    , by(public , totalcolumn) ///
                    pcformat(%5.1f) meanformat(%5.3f) sdformat(%5.1f)  ///
                    exportexcel("${output}/sumstats_bisphos.xls", replace) reportmissing

baselinetable       age sex ethnicity employment_status education1 religion ///
                    prescribed other_medicines ///
                    eq5d3l_u(cts) attitude  inconvenient opinion ///
                     other_pill_number other_pill_times if statins ==1  ///
                    , by(public , totalcolumn) ///
                    pcformat(%5.1f) meanformat(%5.3f) sdformat(%5.1f)  ///
                    exportexcel("${output}/sumstats_statins_cat.xls", replace) reportmissing catvartab("#")

baselinetable       age sex ethnicity employment_status education1 religion ///
                    prescribed other_medicines ///
                    eq5d3l_u(cts) attitude  inconvenient opinion ///
                     other_pill_number other_pill_times  if statins ==0 ///
                    , by(public , totalcolumn) ///
                    pcformat(%5.1f) meanformat(%5.3f) sdformat(%5.1f)  ///
                    exportexcel("${output}/sumstats_bisphos_cat.xls", replace) reportmissing  catvartab("#")



************************************************************************************************

**********************
*** Analysis 1 OUTPUTS ***
**********************


use combined.dta,clear

reshape long utility_q, i(id) j(question)

gen nontraders_half = 1 if utility_q==.5

drop  if utility_q==.5
```

For peer review only

```
ice  ${ice_set_cluster}  , m(5) ///
		saving("${combined}/imputed_cluster.dta",replace) ///
		genmiss(M)  ///
														cmd(  age:  ologit,  opinion:  ologit,  ethnicity:  ologit,
employment_status: ologit, education1: mlogit, attitude: ologit, ) match persist


qui use "${combined}/imputed_cluster.dta", clear

qui mi import ice, imputed(${ice_set_cluster}) clear

replace utility_q = 1-utility_q

***********************
*** Figure 1  ***
***********************
twoway (kdensity utility_q if statins==1 & _mi_m==0 & question==1 & patient==1 ) (kdensity utility_q if statins==1 & _mi_m==0
& question==2) (kdensity utility_q if statins==1 & _mi_m==0 & question==3) (kdensity utility_q if statins==1 & _mi_m==0 &
question==4),   ///
legend(label(1 "Q1 No side effect assumed") label(2 "Q2 Some minor side effect assumed") label(3 "Q3 Some severe side effect
assumed") label(4 "Q4 Reduced effectiveness assumed")  size(vsmall) just(center) ) ///
ytitle("Density" " ", size(small)) ///
xtitle(" " "Utility", size(small)) ///
name(kdensity_statins1,replace) ///
xlabel(0(0.1)0.5,  labsize(small)) ///
ylabel(0(5)30,  labsize(small) angle(horizontal) nogrid) ///
graphregion(fcolor(white) lwidth(large)) bgcolor(white) title("Statin Q1-Q4 utility values in patients",size(small) color(black)) ///
ysize(1) xsize(1) saving(kdensity_statins1,replace) scale(.9)

twoway (kdensity utility_q if statins==1 & _mi_m==0 & question==1 & patient==0 ) (kdensity utility_q if statins==1 & _mi_m==0
& question==2) (kdensity utility_q if statins==1 & _mi_m==0 & question==3) (kdensity utility_q if statins==1 & _mi_m==0 &
question==4),   ///
legend(label(1 "Q1 No side effect assumed") label(2 "Q2 Some minor side effect assumed") label(3 "Q3 Some severe side effect
assumed") label(4 "Q4 Reduced effectiveness assumed")  size(vsmall) just(center) ) ///
ytitle("Density" " ", size(small)) ///
xtitle(" " "Utility", size(small)) ///
name(kdensity_statins2,replace) ///
xlabel(0(0.1)0.5,  labsize(small)) ///
ylabel(0(5)30,  labsize(small) angle(horizontal) nogrid) ///
graphregion(fcolor(white) lwidth(large)) bgcolor(white) title("Statin Q1-Q4 utility values in the public",size(small) color(black)) ///
ysize(1) xsize(1) saving(kdensity_statins2,replace) scale(.9)

twoway (kdensity utility_q if statins==0 & _mi_m==0 & question==0 & patient==1 ) (kdensity utility_q if statins==0 & _mi_m==0
& question==2) (kdensity utility_q if statins==0 & _mi_m==0 & question==3) (kdensity utility_q if statins==0 & _mi_m==0 &
question==4),   ///
legend(legend(label(1 "Q1 No side effect assumed") label(2 "Q2 Some minor side effect assumed") label(3 "Q3 Some severe side
effect assumed") label(4 "Q4 Reduced effectiveness assumed")  size(vsmall) just(center) ) ///
label(1 "No side effect ") label(2 "Some minor side effect assumed") label(3 "Some severe side effect assumed") label(4 "Reduced
effectiveness assumed")  size(vsmall) just(center) ) ///
ytitle("Density" " ", size(small)) ///
xtitle(" " "Utility", size(small)) ///
name(kdensity_bisphos1,replace) ///
xlabel(0(0.1)0.5,  labsize(small)) ///
ylabel(0(2)10,  labsize(small) angle(horizontal) nogrid) ///
graphregion(fcolor(white)  lwidth(large))  bgcolor(white)  title("Bisphosphonates  Q1-Q4  utility  values  in  patients",size(small)
color(black)) ///
ysize(1) xsize(1) saving(kdensity_bisphos,replace) scale(.9)

twoway (kdensity utility_q if statins==0 & _mi_m==0 & question==0 & patient==0 ) (kdensity utility_q if statins==0 & _mi_m==0
& question==2) (kdensity utility_q if statins==0 & _mi_m==0 & question==3) (kdensity utility_q if statins==0 & _mi_m==0 &
question==4),   ///
legend(label(1 "Q1 No side effect assumed") label(2 "Q2 Some minor side effect assumed") label(3 "Q3 Some severe side effect
assumed") label(4 "Q4 Reduced effectiveness assumed")  size(vsmall) just(center) ) ///
ytitle("Density" " ", size(small)) ///
xtitle(" " "Utility", size(small)) ///
```

For peer review only

```
name(kdensity_bisphos2,replace) ///
xlabel(0(0.1)0.5,  labsize(small)) ///
ylabel(0(2)10,  labsize(small) angle(horizontal) nogrid) ///
graphregion(fcolor(white) lwidth(large)) bgcolor(white) title("Bisphosphonates Q1-Q4 utility values in the public",size(small) color(black)) ///
ysize(1) xsize(1) saving(kdensity_bisphos,replace) scale(.9)

grc1leg kdensity_statins1 kdensity_statins2 kdensity_bisphos1  kdensity_bisphos2 , scheme(s1manual) name(g1, replace) rows(2)
graph display g1, xsize(7) ysize(8)
graph export "${output}/kdensity1.tif",  replace width(2000)

***********************
*** Figure 1 alternative ***
***********************
replace utility_q = 1-utility_q

twoway (kdensity utility_q if statins==1 & _mi_m==0 & question==1 & patient==1 ) (kdensity utility_q if statins==1 & _mi_m==0 & question==2) (kdensity utility_q if statins==1 & _mi_m==0 & question==3) (kdensity utility_q if statins==1 & _mi_m==0 & question==4),   ///
legend(label(1 "Q1 No side effect") label(2 "Q2 Some minor side effect") label(3 "Q3 Some severe side effect") label(4 "Q4 Reduced effectiveness")  size(vsmall) just(center) ) ///
ytitle("Density" " ", size(small)) ///
xtitle(" " "Utility", size(small)) ///
name(kdensity_statins1a,replace) ///
xlabel(0.5(0.1)1,  labsize(small)) ///
ylabel(0(5)30,  labsize(small) angle(horizontal) nogrid) ///
graphregion(fcolor(white) lwidth(large)) bgcolor(white) title("Statin Q1-Q4 utility values in patients",size(small) color(black)) ///
ysize(1) xsize(1) saving(kdensity_statins1,replace) scale(.9)

twoway (kdensity utility_q if statins==1 & _mi_m==0 & question==1 & patient==0 ) (kdensity utility_q if statins==1 & _mi_m==0 & question==2) (kdensity utility_q if statins==1 & _mi_m==0 & question==3) (kdensity utility_q if statins==1 & _mi_m==0 & question==4),   ///
legend(label(1 "Q1 No side effect") label(2 "Q2 Some minor side effect") label(3 "Q3 Some severe side effect") label(4 "Q4 Reduced effectiveness")  size(vsmall) just(center) ) ///
ytitle("Density" " ", size(small)) ///
xtitle(" " "Utility", size(small)) ///
name(kdensity_statins2a,replace) ///
xlabel(0.5(0.1)1,  labsize(small)) ///
ylabel(0(5)30,  labsize(small) angle(horizontal) nogrid) ///
graphregion(fcolor(white) lwidth(large)) bgcolor(white) title("Statin Q1-Q4 utility values in the public",size(small) color(black)) ///
ysize(1) xsize(1) saving(kdensity_statins2,replace) scale(.9)

twoway (kdensity utility_q if statins==0 & _mi_m==0 & question==0 & patient==1 ) (kdensity utility_q if statins==0 & _mi_m==0 & question==2) (kdensity utility_q if statins==0 & _mi_m==0 & question==3) (kdensity utility_q if statins==0 & _mi_m==0 & question==4),   ///
legend(label(1 "Q1 No side effect") label(2 "Q2 Some minor side effect") label(3 "Q3 Some severe side effect") label(4 "Q4 Reduced effectiveness")  size(vsmall) just(center) ) ///
ytitle("Density" " ", size(small)) ///
xtitle(" " "Utility", size(small)) ///
name(kdensity_bisphos1a,replace) ///
xlabel(0.5(0.1)1,  labsize(small)) ///
ylabel(0(2)10,  labsize(small) angle(horizontal) nogrid) ///
graphregion(fcolor(white) lwidth(large)) bgcolor(white) title("Bisphosphonates Q1-Q4 utility values in patients",size(small) color(black)) ///
ysize(1) xsize(1) saving(kdensity_bisphos,replace) scale(.9)

twoway (kdensity utility_q if statins==0 & _mi_m==0 & question==0 & patient==0 ) (kdensity utility_q if statins==0 & _mi_m==0 & question==2) (kdensity utility_q if statins==0 & _mi_m==0 & question==3) (kdensity utility_q if statins==0 & _mi_m==0 & question==4),   ///
legend(label(1 "Q1 No side effect") label(2 "Q2 Some minor side effect") label(3 "Q3 Some severe side effect") label(4 "Q4 Reduced effectiveness")  size(vsmall) just(center) ) ///
ytitle("Density" " ", size(small)) ///
xtitle(" " "Utility", size(small)) ///
name(kdensity_bisphos2a,replace) ///
xlabel(0.5(0.1)1,  labsize(small)) ///
```

For peer review only

```
ylabel(0(2)10,  labsize(small) angle(horizontal) nogrid) ///
graphregion(fcolor(white) lwidth(large)) bgcolor(white) title("Bisphosphonates Q1-Q4 utility values in the public",size(small)
color(black)) ///
ysize(1) xsize(1) saving(kdensity_bisphos,replace) scale(.9)

grc1leg kdensity_statins1a kdensity_statins2a kdensity_bisphos1a  kdensity_bisphos2a , scheme(s1manual)
name(g2, replace) rows(2)
graph display g2, xsize(7) ysize(8)
graph export "${output}/kdensity2.tif",  replace width(2000)


***********************
*** TABLE 2 OUTPUTS ***
***********************

gen str coding = ""
replace coding = "s1_p1_q1" if statins==1 & _mi_m==0 & question==1 & patient==1,
replace coding = "s1_p1_q2" if statins==1 & _mi_m==0 & question==2 & patient==1,
replace coding = "s1_p1_q3" if statins==1 & _mi_m==0 & question==3 & patient==1,
replace coding = "s1_p1_q4" if statins==1 & _mi_m==0 & question==4 & patient==1,
replace coding = "s1_p0_q1" if statins==1 & _mi_m==0 & question==1 & patient==0,
replace coding = "s1_p0_q2" if statins==1 & _mi_m==0 & question==2 & patient==0,
replace coding = "s1_p0_q3" if statins==1 & _mi_m==0 & question==3 & patient==0,
replace coding = "s1_p0_q4" if statins==1 & _mi_m==0 & question==4 & patient==0,
replace coding = "s0_p1_q1" if statins==0 & _mi_m==0 & question==1 & patient==1,
replace coding = "s0_p1_q2" if statins==0 & _mi_m==0 & question==2 & patient==1,
replace coding = "s0_p1_q3" if statins==0 & _mi_m==0 & question==3 & patient==1,
replace coding = "s0_p1_q4" if statins==0 & _mi_m==0 & question==4 & patient==1,
replace coding = "s0_p0_q1" if statins==0 & _mi_m==0 & question==1 & patient==0,
replace coding = "s0_p0_q2" if statins==0 & _mi_m==0 & question==2 & patient==0,
replace coding = "s0_p0_q3" if statins==0 & _mi_m==0 & question==3 & patient==0,
replace coding = "s0_p0_q4" if statins==0 & _mi_m==0 & question==4 & patient==0,

estpost tabstat utility_q , statistics(mean sd count skewness kurtosis p10 p25 p50 p75 p90) casewise by(coding)
esttab . using "${output}/table_tabstat.rtf", cells("mean sd count skewness kurtosis p10 p25 p50 p75 p90") noobs

gen str coding2 = ""

replace coding2 = "s1_p1" if statins==1 & _mi_m==0 & patient==1,
replace coding2 = "s1_p0" if statins==1 & _mi_m==0 & patient==0,
replace coding2 = "s0_p1" if statins==0 & _mi_m==0 & patient==1,
replace coding2 = "s0_p0" if statins==0 & _mi_m==0 & patient==0,

estpost tabstat utility_q , statistics(mean sd count skewness kurtosis p10 p25 p50 p75 p90) casewise by(coding2)

esttab . using "${output}/table_tabstat2.rtf", cells("mean sd count skewness kurtosis p10 p25 p50 p75 p90") noobs

gen str coding3 = ""
replace coding3 = "s1" if statins==1 & _mi_m==0
replace coding3 = "s0" if statins==0 & _mi_m==0

estpost tabstat utility_q , statistics(mean sd count skewness kurtosis p10 p25 p50 p75 p90) casewise by(coding3)

esttab . using "${output}/table_tabstat3.rtf", replace cells("mean sd count skewness kurtosis p10 p25 p50 p75 p90") noobs

gen trader = 1 if utility_q!=1 & utility_q!=0.5
replace trader = 0 if trader==.

estpost tabstat trader , statistics(mean sd count skewness kurtosis p10 p25 p50 p75 p90) casewise by(coding)
estpost tabstat trader , statistics(mean sd count skewness kurtosis p10 p25 p50 p75 p90) casewise by(coding2)
estpost tabstat trader , statistics(mean sd count skewness kurtosis p10 p25 p50 p75 p90) casewise by(coding3)

estpost tabstat trader if _mi_m==0 , statistics(mean sd count skewness kurtosis p10 p25 p50 p75 p90) casewise by(statins)

***********************
*** TABLE 3 OUTPUTS ***
```

For peer review only

```
**********************

mi estimate, post:                    reg  utility_q   i.sex  i.ethnicity  i.age  i.other_pill_times  i.other_pill_number   eq5d_index
question_2 question_3 question_4 statins public if statins == 1
estimates store m1

mi estimate, post:                    reg  utility_q   i.sex  i.ethnicity  i.age  i.other_pill_times  i.other_pill_number   eq5d_index
question_2 question_3 question_4 statins public if statins == 0
estimates store m2

gen yes = 1 if utility_q==1
replace yes = 0 if utility_q<1

mi estimate, saving(combined_logs,replace): logistic yes     i.sex    i.ethnicity    i.age    i.other_pill_times    i.other_pill_number
eq5d_index question_2 question_3 question_4 statins public
estimates store m3


esttab m1 m2         using "${output}/table_all_new.rtf" , ar2 label not   replace ci
esttab m3            using "${output}/table_all_logist_new.rtf" , ar2 label not  replace   ci

mi test question_2 question_3 question_4
/*
. mi test question_2 question_3 question_4
note: assuming equal fractions of missing information

 ( 1)  question_2 = 0
 ( 2)  question_3 = 0
 ( 3)  question_4 = 0

       F(  3, 149.7) =    0.15
           Prob > F =    0.9310


.
*/


****************************************************
*** Appendix analysis 6 ***
****************************************************
**Propensity for dominant preferences**

gen yes = 1 if utility_q==1
replace yes = 0 if utility_q<1

gen no = 1 if utility_q==0.5
replace no = 0 if no==.

la var yes "Selected 1 for DTD"

mi estimate, post:                                                          logistic yes          sex    i.ethnicity
i.education1 i.age   public statins
estimates store m5


mi estimate, post:                                                          logistic no           sex    i.ethnicity
i.education1 i.age   public statins
estimates store m6

esttab m5 m6


qui use "${combined}/imputed_cluster.dta", clear

****************************************************
*** Appendix analysis 7 ***
```

For peer review only

```
****************************************************

************STATINS*****************

qui mi import ice, imputed(${ice_set_cluster}) clear
forvalues i =1/5 {
*ssc install zoib //install zero-one-inflated beta regression package
zoib utility_q   sex i.ethnicity i.education1 i.age i.question public i.other_pill_times i.other_pill_number  if _mi_m==1 & statins==1
predict beta_`i'

betamix utility_q     if  _mi_m==1  &  statins==1,  muvar(sex  i.ethnicity  i.education1  i.age  i.question  public  i.other_pill_times
i.other_pill_number ) lbound(0.5) ubound(1) pmass(1 0 1)  //zero and one inflated, including all values
predict mixedbeta_`i'

reg utility_q                     sex  i.ethnicity  i.education1  i.age  i.question    public  i.other_pill_times  i.other_pill_number    if
_mi_m==`i' & statins==1
predict yhat_`i'

}

mi estimate, saving(statin_ols,replace):                         reg  utility_q   i.sex  i.ethnicity  i.education1  i.age  i.question   public
i.other_pill_times i.other_pill_number if statins==1

forvalues i =1/5 {
rmse utility_q yhat_`i'                        if _mi_m==`i'
rmse utility_q beta_`i'                        if _mi_m==`i'
rmse utility_q mixedbeta_`i'     if _mi_m==`i'

}

************BISPHOSPHONATES*****************
qui use "${combined}/imputed_cluster.dta", clear

qui mi import ice, imputed(${ice_set_cluster}) clear
forvalues i =1/5 {
*ssc install zoib //install zero-one-inflated beta regression package
zoib utility_q   sex i.ethnicity i.education1 i.age i.question public i.other_pill_times i.other_pill_number  if _mi_m==1 & statins==0
predict beta_`i'

betamix utility_q     if  _mi_m==1  &  statins==0,  muvar(sex  i.ethnicity  i.education1  i.age  i.question  public  i.other_pill_times
i.other_pill_number ) lbound(0.5) ubound(1) pmass(1 0 1)  //zero and one inflated, including all values
predict mixedbeta_`i'

reg utility_q                     sex   i.ethnicity   i.education1   i.age   i.question     public  i.other_pill_times   i.other_pill_number   if
_mi_m==`i' & statins==0
predict yhat_`i'

}

mi estimate, saving(statin_ols,replace):                         reg  utility_q   i.sex  i.ethnicity  i.education1  i.age  i.question   public
i.other_pill_times i.other_pill_number if statins==0

forvalues i =1/5 {
rmse utility_q yhat_`i'                        if _mi_m==`i'
*gen ols_rmse_`i'  = r(yhat_`i')  if _mi_m==`i'
rmse utility_q beta_`i'                        if _mi_m==`i'
rmse utility_q mixedbeta_`i'     if _mi_m==`i'

}

forvalues i =1/5 {
mean yhat_`i' if _mi_m==`i'                    & statins==0
mean beta_`i' if _mi_m==`i'                    & statins==0
mean mixedbeta_`i' if _mi_m==`i'           & statins==0

}
```

For peer review only

*** Model fit check ***

```
twoway ( scatteri 0.5 0.5 1 1, recast(line) lcolor(gray) lwidth(medthin) lpattern(solid) ) ///
( scatter yhat_1 utility_q  if _mi_m==1 , mcolor(red)  msize(small) msymbol(circle) msymbol(circle_hollow)  ) ( scatter beta_1 utility_q if _mi_m==1 , mcolor(black)  msize(small) msymbol(circle) msymbol(circle_hollow)) ( scatter mixedbeta_1 utility_q  if _mi_m==1 , mcolor(green)  msize(small) msymbol(circle) msymbol(circle_hollow)), ///
 xlabel(0.5(0.25)1,nogrid  labsize(small))  ylabel(0.5(0.25)1,nogrid  labsize(small))  graphregion(  color(white)  )  legend(off)  yscale( nofextend ) xscale(nofextend) ///
ytitle("EQ-5D-3L Predicted JHC", size(small)) xtitle("EQ-5D-3L Observed JHC", size(small)) title(`"`vtext'"', size(small) color(black)) ///
xmtick( 0.5(0.25)1 , grid glcolor(gray) glpattern(dash) glwidth(vthin) nogextend ) ///
ymtick( 0.5(0.25)1 , grid glcolor(gray) glpattern(dash) glwidth(vthin) nogextend ) xsize(1) ysize(1)


twoway (kdensity x if statins==1) (kdensity x if statins==0) (kdensity y if statins==1) (kdensity y if statins==0)  (kdensity yhat if statins==1) (kdensity yhat if statins==0)
```

For peer review only

Supplementary Appendix 6: Description of the whole sample characteristics

	Statin survey			Bisphosphonate survey			
	Patient[a] N=260	Public[b] N=376	Total N=636	Patient[a] N=110	Public[b] N=359	Total N=469	Total N= 1105
**Age**							
Less than 35	2 (1.2%)	25 (7.9%)	27 (5.5%)	0 (0.0%)	26 (8.8%)	26 (7.5%)	53 (6%)
35-44	5 (2.9%)	74 (23.3%)	79 (16.2%)	1 (1.9%)	51 (17.3%)	52 (14.9%)	131 (16%)
45-54	10 (5.9%)	57 (17.9%)	67 (13.7%)	3 (5.7%)	41 (13.9%)	44 (12.6%)	111 (13%)
55-64	49 (28.8%)	84 (26.4%)	133 (27.3%)	14 (26.4%)	57 (19.3%)	71 (20.4%)	204 (24%)
65-74	77 (45.3%)	70 (22.0%)	147 (30.1%)	20 (37.7%)	107 (36.3%)	127 (36.5%)	274 (33%)
75+	27 (15.9%)	8 (2.5%)	35 (7.2%)	15 (28.3%)	13 (4.4%)	28 (8.0%)	63 (8%)
Missing	90	58	148	57	64	121	269
**Sex**							
Female	57 (33.5%)	159 (50.0%)	216 (44.3%)	46 (86.8%)	185 (62.9%)	231 (66.6%)	447 (54%)
Male	113 (66.5%)	159 (50.0%)	272 (55.7%)	7 (13.2%)	109 (37.1%)	116 (33.4%)	388 (46%)
Missing	90	58	148	57	65	122	270
**Ethnicity**							
White British/Irish	160 (94.1%)	288 (90.6%)	448 (91.8%)	51 (96.2%)	263 (89.2%)	314 (90.2%)	762 (91%)
White other	4 (2.4%)	16 (5.0%)	20 (4.1%)	1 (1.9%)	12 (4.1%)	13 (3.7%)	33 (4%)
Mixed/Multiple ethnic origins	0 (0.0%)	2 (0.6%)	2 (0.4%)	0 (0.0%)	5 (1.7%)	5 (1.4%)	7 (1%)
Black/African/Caribbean/Black British	0 (0.0%)	3 (0.9%)	3 (0.6%)	0 (0.0%)	4 (1.4%)	4 (1.1%)	7 (1%)
Asian/Asian British	0 (0.0%)	7 (2.2%)	7 (1.4%)	0 (0.0%)	9 (3.1%)	9 (2.6%)	16 (2%)
Chinese	0 (0.0%)	1 (0.3%)	1 (0.2%)	1 (1.9%)	2 (0.7%)	3 (0.9%)	4 (%)
Other ethnicity	6 (3.5%)	1 (0.3%)	7 (1.4%)	0 (0%)	0 (0%)	0 (0%)	(%)
Missing	90	58	148	57	64	121	269
**Number of pills taken daily**							
None	0 (0.0%)	133 (41.8%)	133 (27.3%)	0 (0.0%)	99 (33.6%)	99 (28.4%)	232 (28%)
One	4 (2.4%)	73 (23.0%)	77 (15.8%)	6 (11.3%)	51 (17.3%)	57 (16.4%)	134 (16%)
Two to five	123 (72.4%)	87 (27.4%)	210 (43.0%)	32 (60.4%)	109 (36.9%)	141 (40.5%)	351 (42%)
Six to ten	36 (21.2%)	18 (5.7%)	54 (11.1%)	9 (17.0%)	23 (7.8%)	32 (9.2%)	86 (10%)
More than ten	7 (4.1%)	7 (2.2%)	14 (2.9%)	6 (11.3%)	13 (4.4%)	19 (5.5%)	33 (4%)
Missing	90	58	148	57	64	121	269
**Number of different times pill taken per day**							
None	3 (1.8%)	134 (42.1%)	137 (28.1%)	0 (0.0%)	97 (32.9%)	97 (27.9%)	234 (28%)
Once per day	40 (23.5%)	104 (32.7%)	144 (29.5%)	22 (41.5%)	100 (33.9%)	122 (35.1%)	266 (32%)
2 times a day	100 (58.8%)	61 (19.2%)	161 (33.0%)	20 (37.7%)	68 (23.1%)	88 (25.3%)	249 (30%)
3 times a day	23 (13.5%)	13 (4.1%)	36 (7.4%)	7 (13.2%)	24 (8.1%)	31 (8.9%)	67 (8%)
More than 3 times a day	4 (2.4%)	6 (1.9%)	10 (2.0%)	4 (7.5%)	6 (2.0%)	10 (2.9%)	20 (2%)
Missing	90	58	148	57	64	121	269
**EQ-5D-3L utility[c]**	0.820 (0.2)	0.803 (0.3)	0.810 (0.3)	0.771 (0.2)	0.790 (0.2)	0.786 (0.2)	0.800 (0.3)
Missing	41	38	79	35	41	76	155

Footnote: [a] patient sample was recruited from general practitioners in the NHS Research Scotland Primary Care Network or the Scottish Health Research Register (SHARE); [b]public sample was recruited from Dynata; [c]Health status measured using the EQ-5D-3 level and transformed into a utility score

Supplementary Appendix 7: Propensity for dominant preference

Male	0.988
	[0.716, 1.364]
White other	1.263
	[0.594,2.684]
Mixed/Multiple ethnic	0.370
	[0.043,3.171]
Black/African/Caribbean/Bla	0.903
	[0.156,5.244]
Asian/Asian British	1.230
	[0.329, 4.604]
Chinese	0.748
	[0.077, 7.290]
Other ethnicity	0.568
	[0.066, 4.902]
Less than 35	0
	[.,.]
35-44	0.709
	[0.377, 1.333]
45-54	0.521
	[0.265, 1.023]
55-64	0.507
	[0.262,0.978]
65-74	0.480
	[0.2540, 0.907]
75+	0.962
	[0.399, 2.318]
Public	1.544*
	[1.047, 2.278]
None	0
	[.,.]
Once per day	1.092
	[0.600,1.986]
2 times a day	0.860
	[0.417,1.775]
3 times a day	1.077
	[0.427,2.715]
More than 3 times a day	2.797
	[0.819,9.554]
None	0
	[.,.]
One	1.0211
	[0.507,2.055]
Two to five	0.875
	[0.446,1.716]
Six to ten	1.012
	[0.386,2.654]
More than ten	1.263
	[0.451,3.536]
Statin sample	0.927
	[0.678,1.268]
Constant	0.367*
	[0.135,1.00]
Observations	1105

For peer review only

Supplementary Appendix 8: Root mean squared error (RMSE) for the competing models to fit TTO data

	Statins			Bisphosphonates		
Imputation number	OLS[1]	ZOIB[2]	MBR[3]	OLS[1]	ZOIB[2]	MBR[3]
Imputation 1	0.0692	0.0694	0.0699	0.0706	0.0719	0.0716
Imputation 2	0.0697	0.0703	0.0695	0.0717	0.0712	0.0718
Imputation 3	0.0693	0.0709	0.0715	0.0718	0.0721	0.0717
Imputation 4	0.0697	0.0705	0.0721	0.0706	0.0722	0.0733
Imputation 5	0.0698	0.0688	0.0717	0.0717	0.0693	0.0734
Mean	0.0695	0.0700	0.0709	0.0713	0.0713	0.0723

[1] Ordinary least squares; [2] Zero inflated beta regression; [3] Mixed beta regression

For peer review only
